# Transcriptomic Profiling of Laser Capture Microdissected FFPE Esophageal Tissues Identifies Candidate Genes and Pathways Associated with Esophageal Adenocarcinoma

**DOI:** 10.3390/ijms27188164

**Published:** 2026-09-14

**Authors:** Md Sazzad Hassan, Matthew Guggenbiller, Annie Ritter, Elizabeth Heffernan, Jun Li, Min Yan, Niranjan Awasthi, Urs von Holzen

**Affiliations:** 1 Department of Surgery, Indiana University School of Medicine, South Bend, IN 46617, USA; nawasthi@iu.edu (N.A.); uvonholz@iu.edu (U.v.H.); 2 Harper Cancer Research Institute, University of Notre Dame, South Bend, IN 46617, USA; 3 College of Science, University of Notre Dame, Notre Dame, IN 46556, USA; mguggenb@alumni.nd.edu (M.G.); aritter3@alumni.nd.edu (A.R.); eheffer2@nd.edu (E.H.); jun.li@nd.edu (J.L.); 4 Goshen Center for Cancer Care, Goshen, IN 46526, USA; myan@goshenhealth.com; 5 School of Medicine, University of Basel, CH-4056 Basel, Switzerland

**Keywords:** esophageal adenocarcinoma, gastro-esophageal reflux disease, Barrett’s esophagus, dysplasia, RNA sequencing, laser capture microdissection

## Abstract

Esophageal adenocarcinoma (AC) incidence has increased markedly in the United States and other Western countries over recent decades. Gastro-esophageal reflux disease (GERD) can cause Barrett’s esophagus, which is strongly associated with the development of AC. However, the mechanisms underlying the pathogenesis of AC remain poorly elucidated. In this exploratory pilot study, we performed transcriptomic profiling by RNA sequencing (RNA-seq) on six normal esophagus (WT), four Barrett’s esophagus with low-grade dysplasia (BL), and six esophageal adenocarcinoma (AC) samples using RNA extracted via laser capture microdissection (LCM) of histologically confirmed formalin-fixed paraffin-embedded (FFPE) esophageal biopsies. Differential gene expression analysis demonstrated partial separation according to histological group, with overlap between BL and AC. BL and AC groups displayed a multitude of differentially expressed genes relative to WT samples; however, fewer differentially expressed genes were found when comparing BL and AC groups. We identified 3062 differentially expressed genes across comparisons (2289 WT vs. AC; 1854 WT vs. BL; 130 BL vs. AC). Using KEGG pathway analysis and functional annotation, candidate genes and pathways were identified. Genes related to the extracellular matrix, focal adhesion, and PI3K-AKT pathways were upregulated in BL and AC versus WT, while genes relating to keratinization, Ras/Rap1, and Hippo signaling were downregulated in BL and AC versus WT. A small set of genes (e.g., ANPEP, REG4, MEP1A) distinguished BL from AC and represent differentially expressed candidates for further investigation. This exploratory transcriptomic profiling provides insights into gene expression changes associated with AC pathogenesis and identifies candidate genes and pathways that warrant independent validation by qRT-PCR, immunohistochemistry, and functional studies.

## 1. Introduction

Esophageal cancer (EC) is among the deadliest and most aggressive cancers, bearing a high mortality rate. Globally, esophageal cancer is the eighth most prevalent cancer and the sixth most common cause of cancer-related mortality [1]. EC is classified into two major histological subtypes: squamous cell carcinoma (SCC), which affects stratified squamous epithelial cells of the predominantly upper esophageal lining, and esophageal adenocarcinoma (AC), which arises most commonly in the distal esophagus from columnar glandular cells lining the epithelium [2]. The incidence of AC has increased approximately 600 percent since the 1970s, with AC now accounting for approximately 60 percent of all EC in Western countries [3,4,5,6]. Gastro-esophageal reflux disease (GERD) as one of the most potent risk factors for the development of AC [7]. It has been suggested that reflux of bile and gastric acid, a common symptom of GERD, may elicit erosive esophagitis, resulting in the development of metaplastic epithelium following an aberrant healing process [8].

In addition to its association with AC, GERD can lead to Barrett’s esophagus (BE), a premalignant condition in which the normal squamous epithelial lining of the distal esophagus is replaced by metaplastic columnar epithelial cells [9,10]. BE has been proposed to develop in response to reflux of gastric acid and bile through the gastro-esophageal junction, causing either transdifferentiation (mature esophageal squamous cells change into columnar cells) or transcommitment (progenitor cells of the esophagus undergo commitment to columnar rather than squamous differentiation) [11]. BE is the only recognized precursor of AC and the strongest risk factor for its development, with patients with BE displaying a 30- to 60-fold increase in risk for AC [12,13]. BE is thought to progress from non-dysplastic through low-grade and high-grade dysplasia before developing into AC [14]. As such, the current risk stratification of developing AC for individuals with BE is dependent on the grade of dysplasia present and histological classification [15].

Currently, endoscopic surveillance biopsies aimed at intervention before the development of invasive AC are recommended for patients with BE, as a histological diagnosis of dysplasia remains the only validated marker for identifying the risk of developing AC [16]. While such methods are associated with a better prognosis, predominantly as AC is identified at an earlier stage, several issues with the current strategy exist. First, the plethora of patients with BE do not develop AC; therefore, endoscopic surveillance biopsies are unnecessary for many and impose significant financial strain on both patients and the healthcare system, and may result in serious, even fatal, complications. Additionally, as BE is largely asymptomatic, a significant portion of patients remain undiagnosed [17,18]. Lastly, surveillance and risk assessment are based on the grade of dysplasia present, which is not only susceptible to interrater variability and unpredictability of progression but also can be difficult to distinguish from regenerative change or active inflammation [19].

Despite the lack of high-quality evidence supporting their benefit, surveillance endoscopic biopsies remain the most common intervention for patients with BE. Thus, improved markers are needed to direct endoscopic surveillance to those patients with high risk of developing AC, diminishing unnecessary and costly intervention for those at low risk. Although the mechanisms underlying the pathogenesis of AC remain poorly elucidated, several genetic association studies have reported a greater preponderance for developing BE and AC within families, suggesting that genetic predisposition may mediate one’s risk for developing AC [20,21]. For example, inherited germline polymorphisms in the E cadherin gene are associated with an increased risk of developing gastro-esophageal cancers due to the crucial role of E-cadherin as a tumor suppressor and mediator of cell–cell adhesion [22]. Consequently, elucidating the molecular mechanisms underlying the development of BE and its progression to AC is crucial to improving the efficacy of detection and intervention strategies.

The current pilot study focuses on understanding the pathogenesis of AC by identifying candidate genes and pathways associated with the histological groups. This could be utilized as inexpensive and effective differentially expressed candidates guiding intervention and potentially treatment. Gene expression profiles created via microarray have proven to be a potent method for elucidating genes and pathways associated with cancer progression [23]. Many alternative markers of increased risk, including telomere length, measures of cell proliferation, and loss of heterozygosity at p53 and p16, among several other loci, have been proposed [24,25,26,27]. However, to date, few studies have conducted analyses comparing the gene expression patterns of individuals with BE displaying varying levels of dysplasia to individuals with AC [28,29].

To identify novel candidate genes which are differentially expressed among normal esophagus, BE displaying low-grade dysplasia, and AC, the current study utilizes RNA sequencing analysis of histologically confirmed cells isolated from esophageal patient biopsies. By precisely capturing cells of interest from formalin-fixed paraffin-embedded (FFPE) samples, laser capture microdissection (LCM) was utilized to isolate a pure population of cells from the heterogeneous esophageal tumor biopsies, yielding a more accurate profile of the biological pathways important in the pathogenesis of BE and AC. Further, high-throughput RNA sequencing was performed as it offers single-base resolution, aligned to a reference genome and annotation, and has miniscule background noise. We hypothesized that unique gene expression profiles will define and contrast normal esophagus, BE with different degrees of dysplasia, and AC. Although expression patterns of housekeeping genes were expected to be similar, we anticipated identifying transcripts that significantly distinguish the sample groups by focusing on genes with greater than a two-fold difference in expression. After KEGG pathway analysis and functional annotation of the differentially expressed genes, we expected to identify potential biomolecular pathways significantly associated with expression patterns in each stage of AC pathogenesis. We further aimed to identify novel effective differentially expressed candidates with clinical utility in predicting and assisting the early diagnosis of AC. Our exploratory analysis of genes differentially expressed among the sample groups contributes to a greater understanding of the pathogenesis of AC and may guide the development of novel therapeutic approaches.

## 2. Results

### 2.1. Quality of RNA Extracted from Selected FFPE Samples

**Figure 1:**
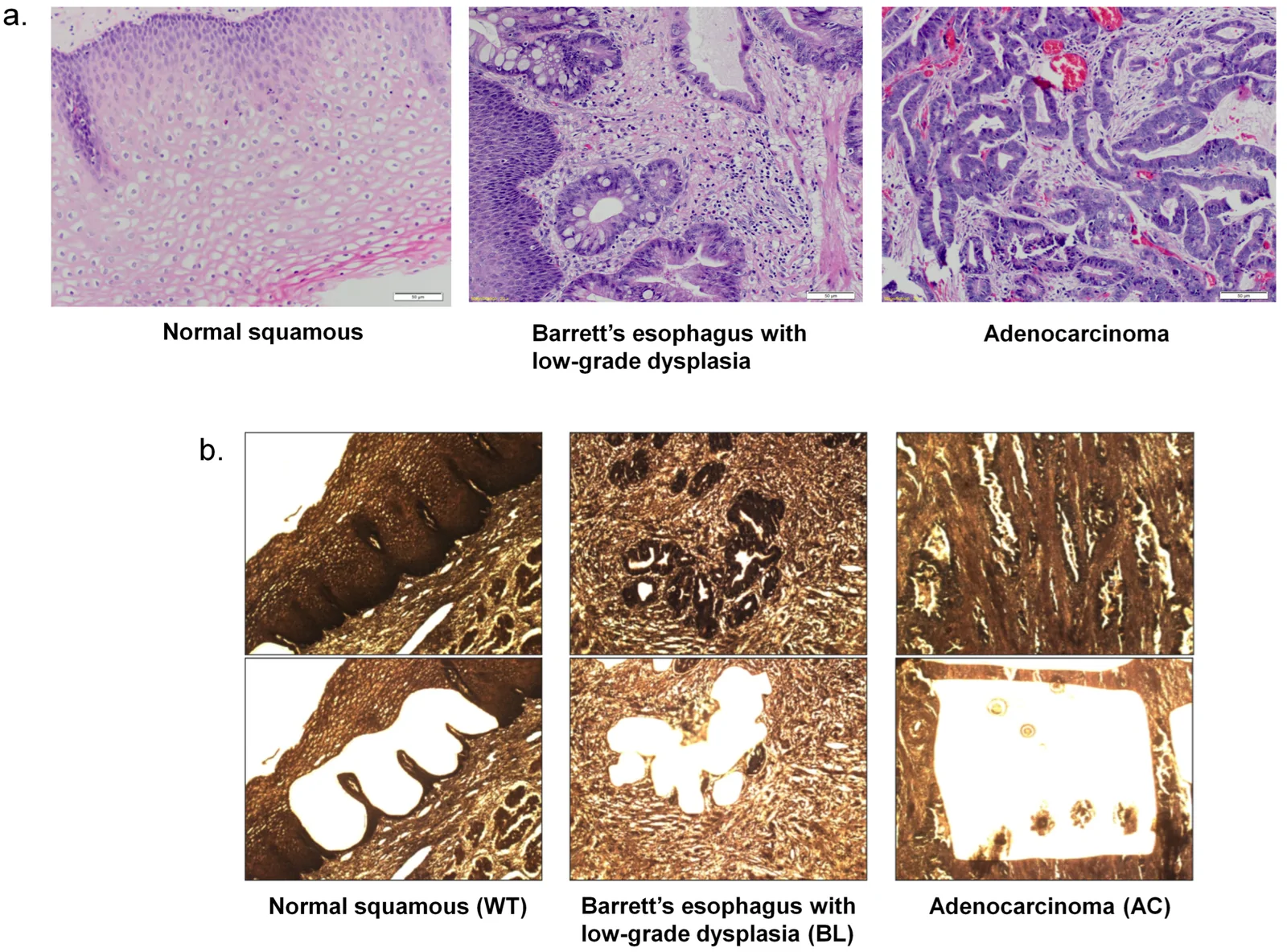
(**a**) **Hematoxylin and eosin (H&E) staining showing histopathology of selected samples for laser capture microdissection.** Transition of Barrett’s esophagus (BE) through dysplasia to esophageal adenocarcinoma (AC). Normal squamous epithelial lining of the esophagus is shown in the first panel. The second panel shows Barrett’s esophagus with low-grade dysplasia (BL). The last panel shows esophageal adenocarcinoma. (**b**) **Representative image of the laser capture microdissection process.** The first row of panels depicts histologically confirmed Arcturus Histogene-stained FFPE tissue sections of the normal squamous epithelial lining, BE sample displaying low-grade dysplasia, and glandular columnar epithelial cells of an AC sample. Second row of panels shows same tissue sections after laser capture microdissection (LCM) using the AutoPix system (Arcturus Bioscience). This means that the chosen tissue areas have been removed from the tissue sections and transferred to collection tubes.

**Figure 2:**
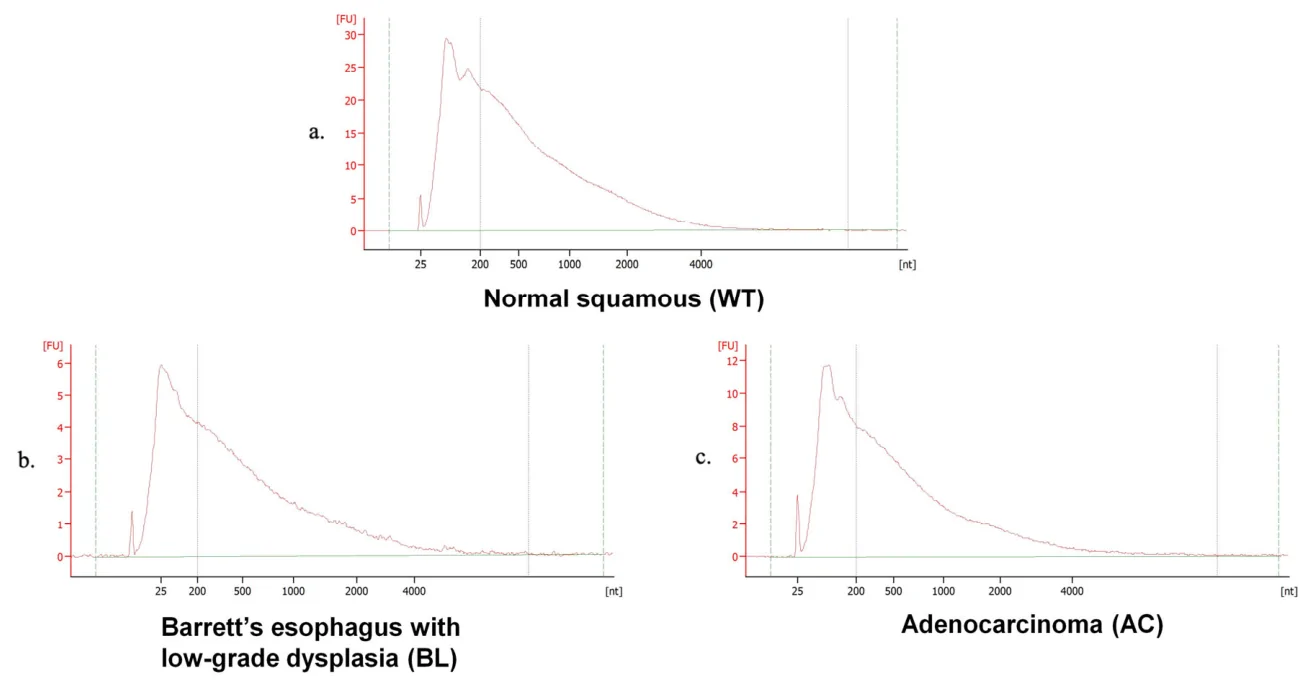
Comparative analysis of total RNA from LCM of FFPE tissues: 1 μL of RNA was fractionated in the Agilent Bioanalyzer 2100 and loaded the Bioanalyzer Pico RNA Chip. (**a**) Representative electropherogram of normal squamous epithelial lining of esophagus (WT) samples. (**b**) Representative electropherogram of Barrett’s esophagus with low-grade dysplasia (BL) samples. (**c**) Representative electropherogram of esophageal adenocarcinoma (AC) samples.

Hematoxylin and eosin (H&E) staining of selected samples showed transition of normal esophagus to BE through dysplasia then to AC (Figure 1a). Laser capture microdissection (LCM) of histologically confirmed Arcturus Histogene-stained FFPE were successful (Figure 1b). The quality of the RNA was assessed by evaluating the shape of the electropherograms produced from RNA quality assessment using the Agilent Bioanalyzer 2100. Figure 2 depicts a comparative analysis of the representative electropherograms of RNA obtained from FFPE tissues using LCM. The full RNA quality assessment reports can be found in Supplementary Figure S1. RNA recovered from FFPE samples by LCM exhibited a heterogeneous profile, and ribosomal RNA peaks were broadened. Both the broadening of ribosomal RNA peaks and the presence of fragmentation products between the 18S ribosomal subunit and the control marker (first peak in the electropherogram) indicate that RNA quality is affected by tissue fixation. RNA was degraded, as expected for FFPE tissue, but was considered suitable for the selected library preparation workflow.

### 2.2. Analysis of Tissue Types

**Figure 3:**
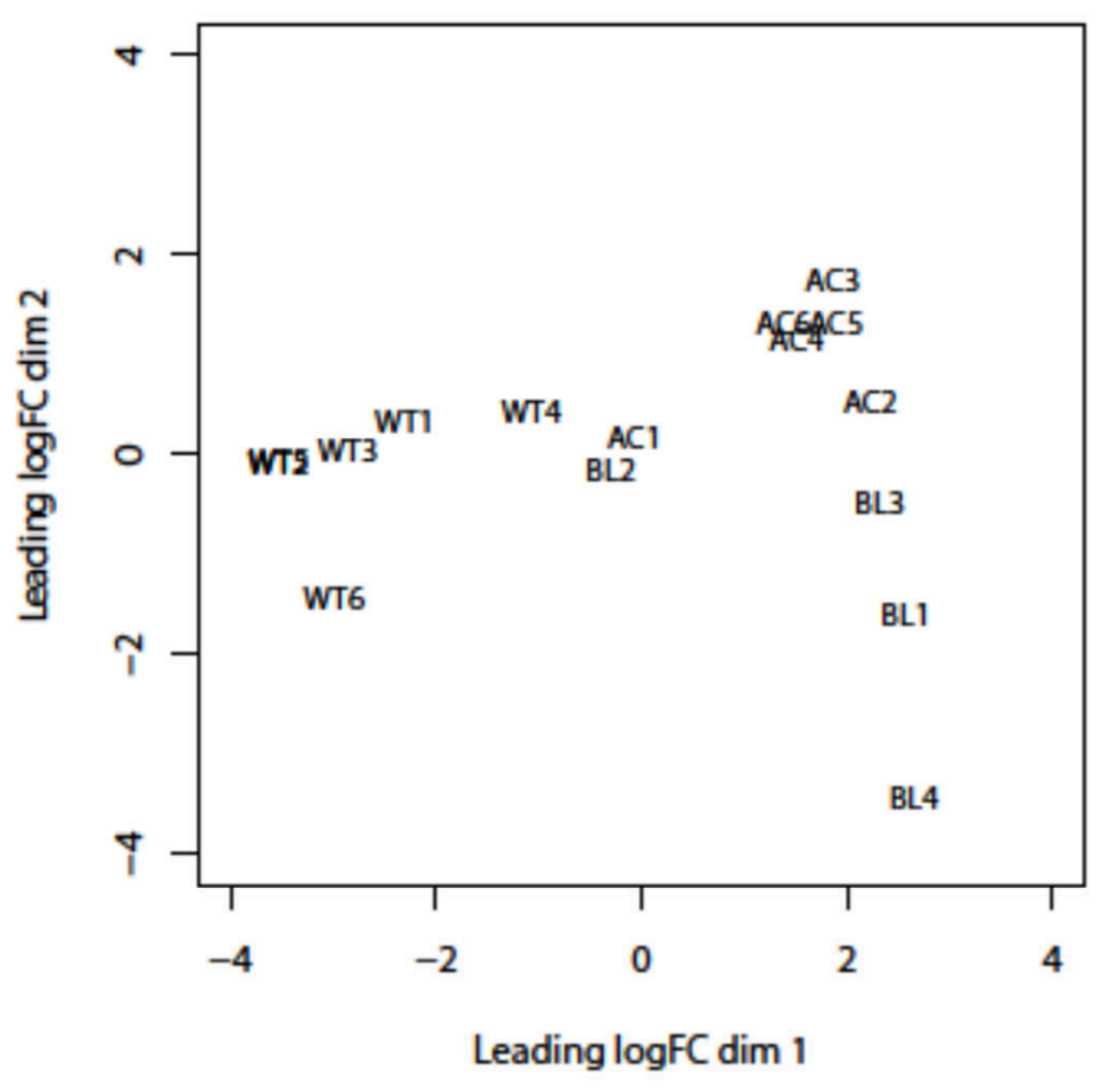
MDS plot over linear discriminant functions logFC 1 and logFC 2 demonstrating distinguishable clusters of normal squamous epithelial lining of esophagus (WT), Barrett’s esophagus with low-grade dysplasia (BL), and esophageal adenocarcinoma (AC) tissues. BL and AC clusters displayed similar separation from the WT cluster; however, some overlap was present between BL and AC clusters. In AC cluster AC3, AC4, AC5, AC6 overlapped, and in WT cluster WT2 and WT5 overlapped. MDS was performed using edgeR’s leading log-fold-change distance, calculated from the 500 genes with the largest absolute log2 fold changes for each pair of samples.

A total of 16 histologically confirmed samples, including 6 WT, 4 BL, and 6 AC, were collected and analyzed. The sequencing core of the University of Notre Dame Genomics & Bioinformatics Core Facility provided raw reads. Raw reads and basic quality were generated from raw FASTQ files. Alignment rates, uniquely mapped, and multimapping rates were generated by the alignment software when mapping reads to the reference genome. Duplicate rate, rRNA fraction, exonic/intronic/intergenic fractions, insert size, and gene body coverage were calculated from the aligned BAM files using standard RNA-seq QC tools. Number of detected genes and processed count matrix were generated at the quantification step. Summary statistics for RNA sequencing mapping were added as Supplementary Table S1. The raw sequencing data and the processed datasets generated during and/or analyzed during the current study are available from the corresponding author on reasonable request. After normalization and background correction, multidimensional scaling (MDS) of the remaining differentially expressed genes separated the samples into three main clusters comprising their known histological class. As shown in Figure 3, WT, BL, and AC clusters were predominantly tight and distinct from one another. Consistent with the columnar phenotype of BL and AC tissues and the squamous phenotype of WT tissues, BL samples clustered more closely with AC samples than with WT samples. Concordant with previous studies, the separation between squamous and columnar tissues was predominantly driven by differential expression of genes associated with the columnar phenotype, increasing their utility as potential differentially expressed candidates [30,31]. It is primarily an unsupervised illustration of separations between samples, using the highly popular plotMDS function in edgeR. No cross validation was used. This was an unsupervised check.

### 2.3. Analysis of Differential Expression

**Figure 4:**
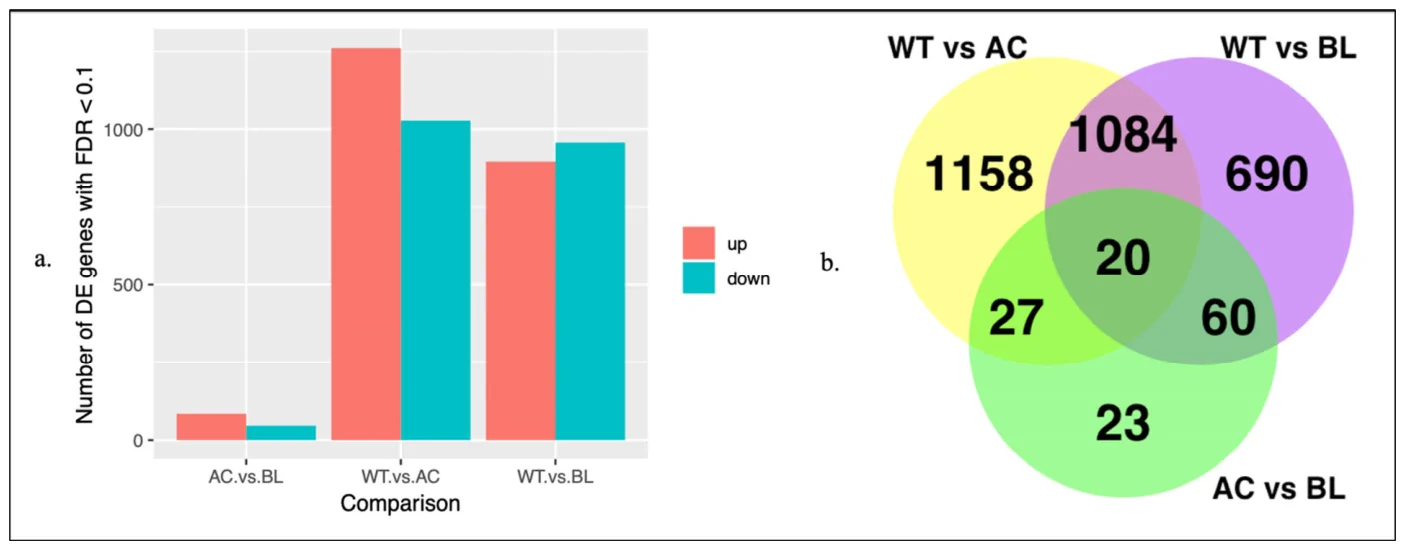
Number and overlap of differentially expressed genes between normal squamous epithelial lining of esophagus (WT), Barrett’s esophagus with low-grade dysplasia (BL), and esophageal adenocarcinoma (AC) groups. (**a**) A bar chart with log2 FC 1 and FDR cutoff of 0.1. The total number of genes differentially expressed between WT, BL, and AC stratified by direction of expression difference relative to WT (for WT vs. BL and WT vs. AC comparisons) or AC (for BL vs. AC comparison). (**b**) The total number of genes differentially expressed presented in Venn-diagram form under FDR cutoff of 0.1 and fold change of 2 to illustrate overlap between the three tissue types. The number of genes differentially expressed in more than one tissue group are depicted in overlapping regions of the diagram.

**Figure 5:**
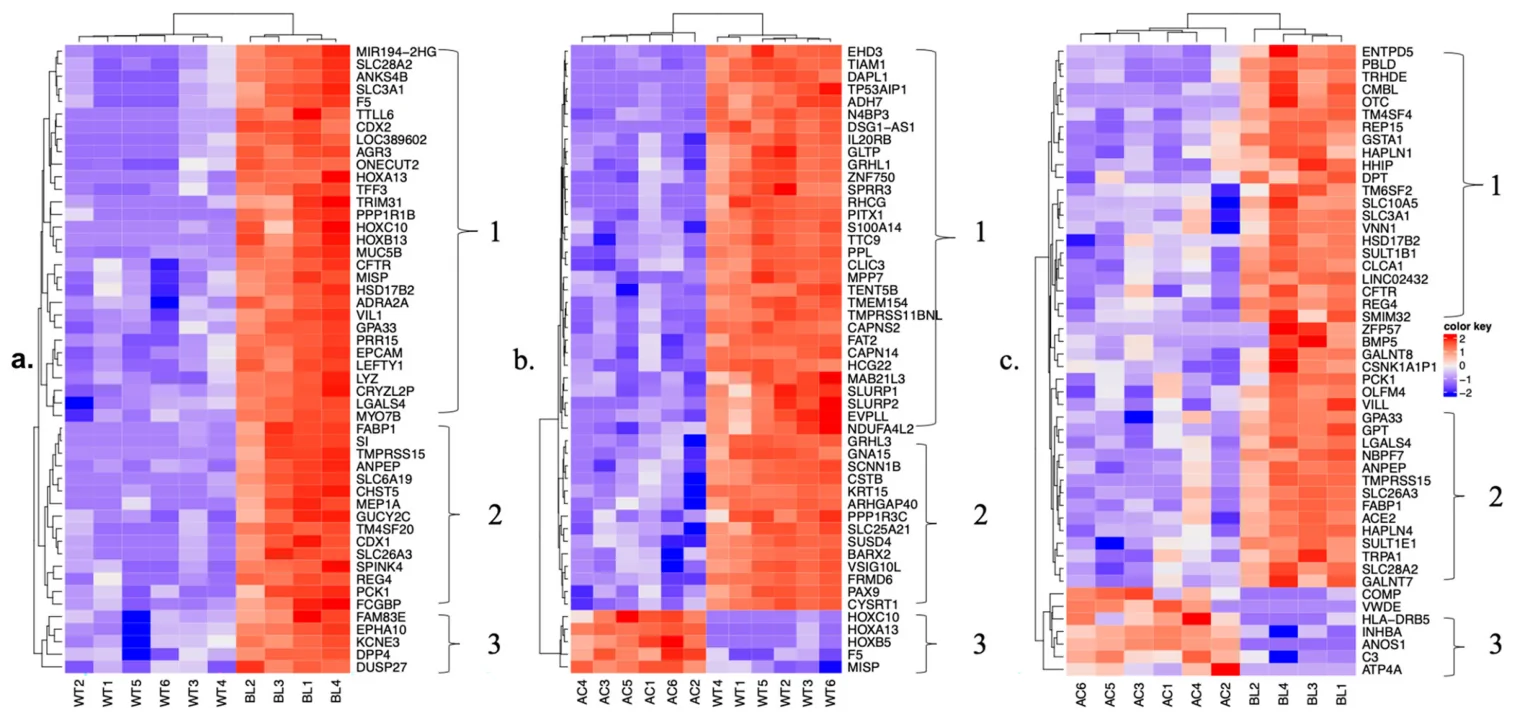
Top 50 differentially expressed genes displayed in hierarchical clustering heatmap (log (1+normalized counts). The values were scaled by gene, and hierarchical clustering was performed using Euclidean distance and complete linkage. (**a**) The dendrogram of the normal squamous epithelial lining of esophagus (WT) vs. Barrett’s esophagus with low-grade dysplasia (BL) comparison demonstrates three main branches, labeled 1, 2, and 3, all of which were upregulated in BL relative to WT and separated further into smaller clusters. (**b**) The dendrogram of the WT vs. esophageal adenocarcinoma (AC) comparison separated into three unique branches, labeled 1, 2, and 3. Branches 1 and 2 were significantly downregulated in AC relative to WT while branch 3 was significantly upregulated in AC relative to WT. (**c**) The dendrogram of the BL vs. AC comparison displayed 2 main branches, one of which encompassed subbranches 1 and 2 and displayed downregulation in AC relative to BL, and branch 3, which was upregulated in AC relative to BL.

A total of 3062 genes were identified as differentially expressed (absolute log2 > 2, FDR < 0.1) between WT, BL, and AC groups: 2289 between WT and AC, 1854 between WT and BL, and 130 between BL and AC (Figure 4a). As shown in Figure 4b, there was substantial overlap of differentially expressed genes among comparisons, especially regarding BL and AC groups when compared to WT. The full data for each differential comparison can be found in Supplementary Tables S2–S4. Concordant with the overlap of BL and AC clusters in Figure 3, 1084 genes were differentially expressed in both AC and BL relative to WT, predominately genes associated with the columnar phenotype. Indeed, several of the top 20 differentially expressed genes, including *TRIM31*, *S100A14*, *LNX1*, *DUOX2*, and *ANPEP*, have been linked to columnar phenotype development [32,33,34]. However, most genes differentially expressed in the BL vs. AC comparison were not associated with the columnar phenotype, thus having potential utility as histological stage specific, rather than phenotype-specific effective differentially expressed candidates. Nonetheless, as an abundance of genes were identified as differentially expressed among WT, BL, and AC groups—especially regarding comparisons of either BL or AC to WT—a heatmap displaying the top 50 differentially expressed genes in each comparison was created. A hierarchical dendrogram was utilized to segregate these genes based on known biological associations and variability among samples, as shown in Figure 5. Despite the similarity in the number of genes which were upregulated and downregulated in each comparison (Figure 4a), most of the top 50 differentially expressed genes in each heatmap displayed the same direction of regulation (i.e., all genes in Figure 5a were upregulated in BL relative to WT, while most genes in Figure 5b,c were downregulated in AC relative to WT and BL, respectively).

### 2.4. KEGG Pathway Analysis and Functional Annotation

To relate differences in gene expression among groups to biological functions, KEGG pathway analysis was used to identify biological pathways, cellular processes, and cellular components which were overrepresented or underrepresented among genes with fold change > 2 and FDR < 0.001 for the WT vs. BL and WT vs. AC comparisons, or FDR < 0.1 for the BL vs. AC comparison. Further, functional annotation through GO analysis was performed to identify common functionality among genes which were identified as differentially expressed using the aforementioned criteria. In practice, the magnitude of transcriptional differences varies substantially among pairs of conditions. Applying the same FDR threshold to all comparisons may therefore result in an excessively large number of differentially expressed genes for some comparisons and too few for others. Either scenario can reduce the informativeness and statistical power of downstream pathway enrichment analyses. For this reason, different FDR thresholds were used across comparisons to obtain gene sets of appropriate size for pathway enrichment analysis. The gene ratio, the raw *p* values, adjusted *p* values, the multiple correction method, and gene contributing to each pathway were given in Supplementary Figure S2.

**Figure 6:**
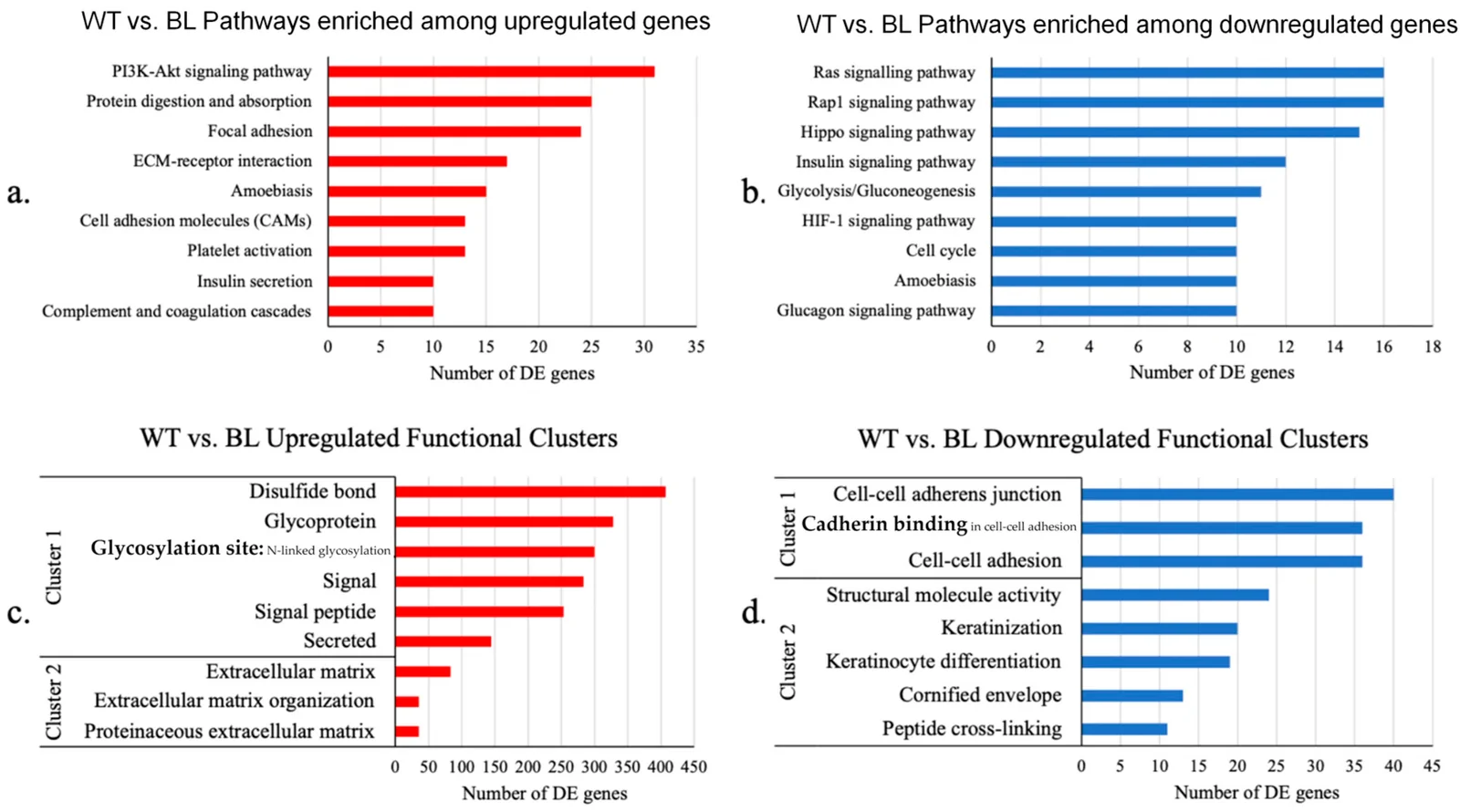
Top differentially regulated biological pathways/processes as determined by KEGG pathway analysis and differentially regulated functional clusters as determined by functional annotation of differentially expressed genes between normal squamous epithelial lining of esophagus (WT) and Barrett’s esophagus with low-grade dysplasia (BL) groups. (**a**) Several of the top pathways, most notably that pertaining to PI3 kinase-Akt signaling, extracellular matrix signaling, and focal adhesion, which were upregulated in BL relative to WT. (**b**) Several of the top pathways, most notably that of Ras signaling, Rap1 signaling, and Hippo signaling, which were downregulated in BL relative to WT. (**c**) The biological functional clusters, extracellular signaling (cluster 1), and extracellular matrix structure (cluster 2) displaying the greatest number of differentially overexpressed genes in BL relative to WT samples. (**d**) The top functional clusters, cell–cell adhesion, and tight junction formation (Cluster 1) and epidermal keratinization (Cluster 2), which were downregulated in BL relative to WT.

All branches of Figure 6a contain a multitude of genes that were upregulated in BL. KEGG pathway analysis of all differentially expressed genes demonstrated several pathways with a significant number of upregulated genes (Figure 6a). Although none of the top 50 differentially expressed genes in Figure 5a were downregulated relative to WT, several pathways were identified in KEGG pathway analysis to consist of several genes which were downregulated in BL (Figure 6b). KEGG pathway analysis revealed that significantly upregulated genes in BL were predominantly involved with the cell proliferation, extracellular matrix, focal adhesion, and cell membrane transporters signaling. Conversely, downregulated genes in BL were predominately associated with signaling in Ras, Rap1, Hippo, and metabolic pathways (Figure 6b). Functional annotation revealed that extracellular signaling (Cluster 1) and extracellular matrix structure (Cluster 2) displayed the greatest number of differentially overexpressed genes in BL relative to WT (Figure 6c). The top functional clusters that were downregulated in BL relative to WT included cell–cell adhesion and tight junction formation (Cluster 1) and epidermal keratinization (Cluster 2) (Figure 6d).

**Figure 7:**
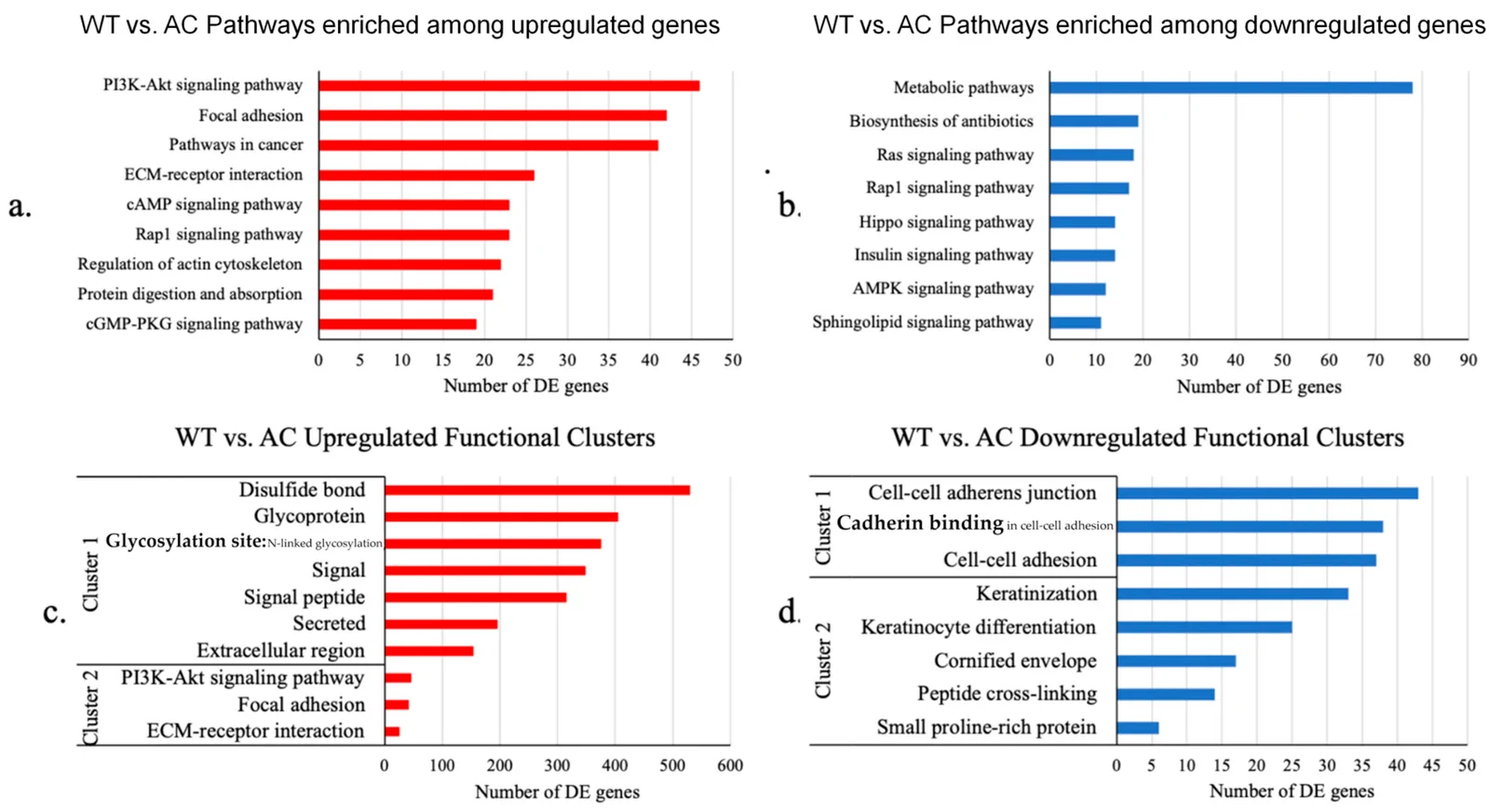
Top differentially regulated biological pathways/processes as determined by KEGG pathway analysis and differentially regulated functional clusters as determined by functional annotation of differentially expressed genes between normal squamous epithelial lining of esophagus (WT) and esophageal adenocarcinoma (AC) groups. (**a**) Several of the top pathways, most notably that of PI3 kinase-Akt signaling and focal adhesion, which were upregulated in AC relative to WT. (**b**) Several of the top pathways, most notably that of metabolic pathways, Ras signaling, Rap1 signaling, and Hippo signaling, which were downregulated in AC relative to WT. (**c**) The biological functional clusters, extracellular signaling (cluster 1), and focal adhesion (cluster 2) displaying the greatest number of differentially overexpressed genes in AC relative to WT samples. (**d**) The top functional clusters, cell–cell adhesion and tight junction formation (Cluster 1) and epidermal keratinization (Cluster 2) which were downregulated in AC relative to WT.

As with BL tissues, KEGG pathway analysis revealed that upregulated genes in AC were largely associated with the extracellular matrix, especially pertaining to collagen binding, signal transduction, and the cell cycle (Figure 7a). Interestingly, although Ras, Rap1, and Hippo signaling were downregulated in AC as in BL tissues, the greatest number of downregulated genes was associated with cellular metabolic pathways (Figure 7b). Nonetheless, functional annotation highlighted those upregulated genes largely clustered into extracellular matrix signaling and focal adhesion pathways (Figure 7c) while downregulated genes clustered into cell–cell adhesion/tight junction formation and keratinization (Figure 7d).

**Figure 8:**
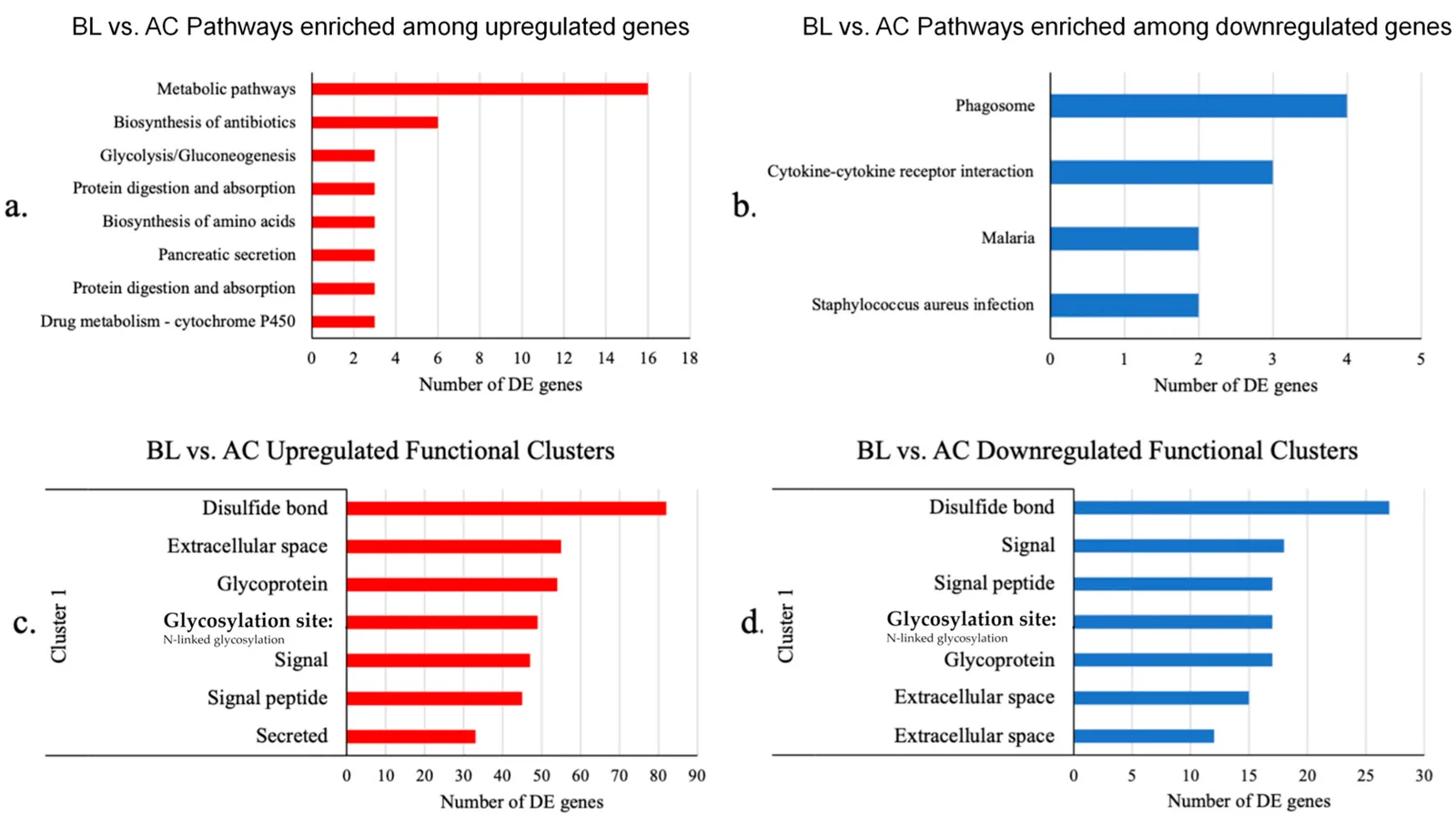
Top differentially regulated biological pathways/processes as determined by KEGG pathway analysis and differentially regulated functional clusters as determined by functional annotation of differentially expressed genes between Barrett’s esophagus with low-grade dysplasia (BL) and esophageal adenocarcinoma (AC) groups. (**a**) Several of the top pathways, most notably metabolic pathways, which were upregulated in BL relative to AC. (**b**) Several of the top pathways, which were downregulated in BL relative to AC. Extracellular signaling (Cluster 1) was the only functional cluster which was (**c**) upregulated and (**d**) downregulated in BL relative to AC.

KEGG pathways analysis identified several pathways with relatively few genes when comparing BL with AC; most upregulated genes in BL were associated with metabolic pathways and signaling as seen in Figure 8a.

## 3. Discussion

Transcriptomic profiling of normal esophagus (WT), Barrett’s esophagus with low-grade dysplasia (BL), and esophageal adenocarcinoma (AC) revealed stage-specific gene expression changes during AC progression. RNA sequencing of LCM-isolated FFPE tissues identified distinct expression clusters corresponding to histological groups, with partial overlap between BL and AC samples. Consistent with these observations, far more genes were differentially expressed when comparing WT with BL or AC than between BL and AC, suggesting that major transcriptional reprogramming occurs early in the metaplasia–carcinoma sequence. Although all our samples went through the laboratory steps and the gene sequencing machine at the exact same time using the exact same methods, clinical variability may contribute to some clustering differences; the overall separation among groups indicates robust molecular distinctions associated with disease progression. However, an increase in sample size with including barrette’s esophagus having no dysplasia or high-grade dysplasia groups would be required to clearly determine the changes associated with columnar lineage conversion leading to dysplasia followed by adenocarcinoma. Similar preponderance of a single direction of differential expression has been reported in prostate and colorectal cancer, associated with the downregulation of pathways inhibiting cellular growth and promoting contact dependence [35,36].

BL and AC samples displayed upregulation in genes associated with the extracellular matrix and its signaling, especially regarding the PI3K-Akt signaling pathway, focal adhesion, and some metabolic pathways. Downregulation of genes associated with Ras, Rap1, and Hippo signaling pathways was common among BL and AC samples. Further, BL and AC samples displayed downregulation of genes associated with the functional clusters of cell–cell adhesion, tight junction formation, and keratinization. Although many differentially expressed genes were common in BL and AC due to their association with the columnar phenotype, several unique genes/pathways were identified with promise for novel differentially expressed candidate development. Several of the genes associated with these pathways have been reported previously. For example, *CDX1* and *CDX2* have been identified as key drivers in the epithelial to mesenchymal transition and the formation of goblet-enriched intestinal mucosa [37,38]. Additionally, *FABP1*, *AGR3*, *EPCAM*, and *HOX13A* have all been linked to tumorigenesis in pathways associated with growth signaling, nutrient uptake, and regulation of transcription [39,40,41,42]. Further, *VIL1* has previously been linked with the severity of nuclear atypia in endometrial adenocarcinoma, warranting further investigation into its role in the epithelial-to-mesenchymal transition in the progression of AC [43].

A significant portion of the clusters within branch 1 of Figure 5a was associated with extracellular signaling and cell proliferation. Branches 1 and 3 of Figure 5a displayed significant enrichment in the focal adhesion and related PI3K-Akt signaling pathways. *ANKS4B* and *MPP7*, highly upregulated across BL samples, have been well documented as critical for adhesion complex formation and induction of actin-supported cell-surface protrusions for nutrient and solute uptake [29,44]. Interestingly, *FAM83H*, a biomarker with significant prognostic value in the proliferation, metastasis, and precision therapy resistance of non-small-cell lung cancer, was among the top differentially expressed genes in BL tissues, warranting further investigation as a potential differentially expressed candidate [45]. Additionally, several genes from branch 2 of Figure 5a, including *MEP1A*, *REG4*, and *ANPEP,* displayed connections with cluster 1 of Figure 6c and have been identified as differentially expressed candidates for several other carcinomas [46,47].

Though oncogenic upregulation of Ras and Rap1 signaling has been implicated in a plethora of cancers, genes identified as downregulated in Figure 5b were predominantly linked to inhibition/regulation of Ras and Rap1 signaling, indirectly eliciting similar outcomes of proliferation and loss of contact inhibition [48,49,50]. Similarly, downregulated genes in the Hippo signaling pathway, including *MST1/2* and *STK3/4*, have been associated with regulation of cell proliferation and promotion of apoptosis. Interestingly, BL tissues displayed a downregulation of several genes associated with metabolic pathways involving oxidative phosphorylation, hypothetically characteristic of the Warburg effect—a well-known alteration associated with increased aerobic glycolysis and decreased reliance on mitochondrial metabolic processes [51]. Furthermore, functional annotation revealed that a multitude of genes were constituents of keratinization pathways. As several serpins and other negative regulators of apoptosis clustered tightly with genes associated with keratinization and are adjacently located on chromosome 19, a common deletion of the aforementioned chromosomal region may have occurred in BL samples, though further analysis of this region is needed to determine the pattern of expression across groups [52].

Despite several pathways and functional clusters being shared between BL and AC samples, several unique pathways were identified in AC samples and contained genes largely associated with a proliferation signature, especially related to cytokinesis and cell cycle checkpoints. For example, the mitotic spindle positioning (*MISP*) protein, identified as crucial for centrosome clustering and mitotic spindle organization, has been investigated as a biomarker for a variety of epithelial tumors [53,54]. Additionally, although few appeared in the top 50 differentially expressed, several upregulated genes, including *FABP1*, *AGR3*, and *MAP3K*, were linked with extracellular matrix/focal adhesion signaling, providing further evidence of the significance of such pathways in the progression of AC [39,55]. Interestingly, *MPP7*, commonly associated with adhesion complex formation, was downregulated in AC samples but upregulated in BL samples relative to WT [56]. We hypothesize that this discrepancy may be associated with *MPP7*’s secondary role as a positive regulator of autophagy and tight junction formation given the weakening of tight junctions for AC cells undergoing epithelial-to-mesenchymal transitions [57]. Such hypothesis is further supported by the downregulation of the functional cluster associated with tight junction formation identified in Figure 7d. Branch 1 of Figure 5b consisted of common tumor suppressors such as *TP53* and *HCG22* which were downregulated in AC tissues [58,59]. Additionally, transcription factors associated with complement system activation such as *SUSD4*, and those involved in growth inhibition, such as *PAX9*, were also identified in branch 1 of Figure 5b [60,61]. Branches 1 and 2 had several genes linked with Ras, Rap1, and Hippo signaling that were similarly downregulated in AC as in BL tissues; however, branch 1 contained several genes associated with metabolic pathways which were significantly more downregulated in AC samples, especially those related to traditional carbohydrate metabolism. For example, the downregulation of *FAT2* and *ADH7* has been linked to several processes in cellular metabolism, reflecting a greater reliance on metabolic pathways without oxidative phosphorylation characteristic of Warburg metabolism [62,63,64]. As shown in Figure 7b, several genes including *GLTP* and *CPTP* which have been linked to the uptake of sphingolipids were downregulated in AC samples, and hypothesized for by the regulatory role several sphingolipids have been suggested to display regarding apoptosis and autophagy [65,66]. As with BL samples, functional annotation highlighted the significant downregulation of clusters associated with keratinization in AC samples, especially related to kallikreins and serpins (Figure 7d), warranting further investigation into the nature and frequency of deletions in the chromosomal region spanning such genes [52].

As only 130 genes were differentially expressed between BL and AC groups, differentially regulated KEGG pathways and functional clusters predominantly consisted of few genes, as seen in Figure 8. For example, *ANPEP*, *REG4*, and *MEP1A*, were among the top 50 genes differentially upregulated in BL relative to both WT and AC. Such pattern may be indicative of a distinct change in cellular metabolic profiles between BL and AC tissues, with AC tissues displaying lower activity in canonical cellular metabolic pathways, especially those involving oxidative phosphorylation [46,47]. Additionally, downregulated genes in BL relative to AC (branch 3 of Figure 5c) were associated with proliferation and cytokine–cytokine interaction and have been investigated as potential biomarkers for gastric and colon cancers [67,68,69]; however, the relatively small number of genes accompanying such pathways warrants further investigation to determine their link with AC pathogenesis.

*MPP7*, upregulated in BL and downregulated in AC relative to WT, has been linked with adhesion complex, tight junction formation, and hypothesized positive regulation of autophagy [57]. The differential expression of *MPP7* in BL and AC groups warrants further investigation into the hypothesis of its utility as a stage-specific biomarker and elucidation of its role in the epithelial–mesenchymal transition associated with AC progression. Although metabolic pathways were upregulated in both BL and AC, several genes associated with canonical metabolic pathways were significantly downregulated in AC relative to BL, including *MEP1A*, *REG4*, and *ANPEP*. These genes were among the top 50 differentially expressed in the WT vs. BL and BL vs. AC comparisons. Given that secreted proteins are able to be measured in blood serum levels, *MEP1A*, *REG4*, and *ANPEP* would have significant practical value as a biomarker, justifying further investigation, especially given their prognostic value as biomarkers in the progression of hepatocellular and esophageal squamous cell carcinomas [46,47]. Further studies are required to determine whether these differentially expressed candidates can be detected at the protein level in serum and whether they have diagnostic or prognostic utility. Lastly, both AC and BL tissues displayed downregulation of genes associated with the keratinization functional cluster. The coordination of kallikreins and serpins in such cluster and their chromosomal proximity hypothesize that a chromosomal deletion may have occurred in some BL and AC tissues, though additional research into the nature and frequency of such deletions is needed before evaluation of their efficacy as differentially expressed candidates.

Despite the intriguing nature of the potential biomarkers identified, there are limitations to the findings. Major limitations of this study include the small sample size, particularly the inclusion of only four BL samples, and the absence of a high-grade dysplasia group. These limitations may reduce statistical power and increase the possibility of false-positive findings. The cross-sectional design of this study also limits the identification of changes associated with disease progression because gene expression was assessed at a single time point. Other key limitations include the lack of longitudinal progression data, lack of independent validation, degraded RNA inherent to FFPE tissues, potential clinical confounding, including sex imbalance, and the inability to fully distinguish epithelial lineage-associated differences from changes associated with disease progression. Patient sex and other clinical characteristics could not be evaluated as potential covariates because these data were unavailable for analysis under the restrictions of our IRB-approved protocol. Therefore, potential confounding by sex or other unmeasured clinical variables cannot be excluded, particularly given the differential expression of XIST observed between BL and AC samples. Although library preparation and sequencing were performed in the same batch, other sources of technical variability cannot be completely excluded.

Given the descriptive nature of the experimental design, conclusions regarding the predictive value of these biomarkers in the progression of BL to AC cannot be made, as the impact of variable spatiotemporal gene expression and sampling region cannot be ruled out as causes for differential expression patterns. Future work to assess the efficacy of potential biomarkers would entail validation of the RNA-Seq results via qRT-PCR and immunohistochemistry analysis. In vitro analysis using cell culture models and in vivo studies using mouse models would help to identify the functional role of such genes in the pathogenesis of AC. Our ongoing studies have provided preliminary evidence supporting a role for TRIM31 in the pathogenesis of BE and AC [70]. In addition, a previously published study demonstrating a role for HOXA13 in the etiology and oncogenic potential of Barrett’s esophagus is consistent with our findings [42]. However, the candidates identified in the present study require independent protein-level and functional validation.

## 4. Materials and Methods

### 4.1. Sample Collection

In total, 16 samples were profiled: 6 normal esophagus (WT), 4 BE displaying low-grade dysplasia (BL), and 6 AC samples. Each sample was obtained from a different patient, and WT samples were histologically normal tissue obtained from individuals without BE/AC. Patient tissue samples were collected from patients being evaluated at the Goshen Center for Cancer Care in Goshen, IN, according to a protocol approved by the Goshen Health Goshen Hospital Institutional Review Board. Our Institutional Review Boards (IRBs) restrict patient characteristics to protect privacy and comply with the regulations. Patient characteristics data are fully anonymized and restricted to prevent re-identification. According to our IRB protocol, patient clinical traits cannot be shared. Tissue samples were submitted to the Laboratory of Pathology at Goshen Health for confirmation that the tissue material represented an adequate sample of the relevant tissue. Tissues were divided, with one of the pieces undergoing histopathological review by a board-certified pathologist after hematoxylin and eosin (H&E) staining of FFPE tissue block sections (Figure 1a), while the other pieces were frozen at −80 °C. Ten-micrometer sections of FFPE tissue blocks were cut with a Leica microtome and mounted on PEN Membrane Glass Slides (ThermoFisher Scientific, Catalog #LCM0522, Waltham, MA, USA).

### 4.2. Laser Capture Microdissection (LCM)

After deparaffinization, FFPE tissue sections (10 µm) were stained using Arcturus Histogene Staining Solution (Arcturus Bioscience, KIT0415, Mountain View, CA, USA), a stain optimized for production of high-quality RNA. To isolate pure cell populations from the heterogeneous tumor tissue specimens (Figure 1b), allowing for accurate molecular profiling of cancerous and noncancerous tissues, LCM was performed on these histologically confirmed Arcturus Histogene-stained FFPE tissue sections using the AutoPix system (Arcturus Bioscience) at the Tissue Biorepository Facility of the Harper Cancer Research Institute. Collected tissue samples were subsequently stored at −80 °C until further processing.

### 4.3. RNA Isolation and Quality Assessment

For laser capture microdissected samples, the caps containing the pure population of cells from the FFPE samples were transferred to a microcentrifuge tube and lysed according to the manufacturer’s protocol. Total RNA was isolated using the Ambion RecoverAll kit (Life Technologies, Grand Island, NY, USA) according to manufacturer’s protocol and eluted in 20 μL of RNAse-free H_2_O. RNA concentration was determined using the Invitrogen Qubit fluorometer (Life Technologies, Grand Island, NY, USA) and RNA quality assessment was performed using the Agilent 2100 Bioanalyzer (Agilent Technologies, Santa Clara, CA, USA). The RNA concentrations, RNA Integrity Number (RIN), and DV200 metric were assessed before RNA sequencing was performed (Table 1).

RNA quality did not differ significantly among WT, AC, and BL groups. Because of the small sample size and no significant differences in RNA quality among the groups we, did not consider whether RNA quality is associated with the statistical analysis.

### 4.4. RNA Sequencing and Analysis

After quality assessment, the extracted RNA was sent to the Genomics & Bioinformatics Core Facility at the University of Notre Dame for RNA sequencing library preparation, Illumina high-throughput sequencing, alignment, and data analysis. RNA sequencing library preparation is conducted in the same batch, and sequencing is run in the same batch to minimize technical noise and systematic variations. RNA sequencing libraries were prepared using the Epicentre ScriptSeq Complete Gold Low Input library preparation kit according to manufacturer’s protocol (Illumina, Inc., San Diego, CA, USA). Multiplexed sequencing of 100 bp paired-end reads was performed using the Illumina HiSeq (Illumina, Inc., San Diego, CA, USA) according to manufacturer’s protocols at the Genomics & Bioinformatics Core Facility. Alignment of paired-end reads (FASTQ) to human genome build 38 (hg38) was performed using Genentech’s Genomic Short-Read Nucleotide Alignment Program (GSNAP version 27 June 2013), allowing for a maximum of 5 mismatches, including penalties for insertions and deletions and local or distant splicing, and configured to recognize novel splicing and detect known splice sites from RefGene through the UCSC Genome Browser [27,71]. Reads were additionally aligned to known human long intergenic non-coding RNA (lincRNA) databases [27]. Primary assembly results were aligned to the reference genome using BLAT (version 3.5) with the highest sequence identity and >90 percent coverage of the contig. Reads that could be mapped to the genome were combined with assembled candidate transcripts to predict new genes using Cufflinks (v1.3.0). The predicted new genes were also required to be at least 10 kb away from either end of known genes in the nonredundant gene set (the union of RefSeq, Ensembl and lncRNA) and other predicted new genes. Data cleaning and processing was performed by the Genomics & Bioinformatics Core Facility to assess the expression patterns of each group for further comparison. Analysis of the RNA sequencing data utilized a factorial model and pairwise comparisons of differential expressions of genes and pathways using edgeR (version 3.28). Following output was ultimately used to generate the gene level count matrix analyzed by edgeR: the exact reference genome version was UCSC hg38, the exact gene annotation release was UCSC hg38 annotation (hg38_ucsc.annotated.gtf), transcript levels were counted using HTSeq.scripts.count from htseq version 0.11.2, predicted transcripts were never included in the final analysis, multimapping reads were discarded, duplicated gene read counts were uniquely assigned to transcripts of the same gene and summed to obtain gene-level counts, the normalization was carried out within edgeR, the method used to estimate dispersion was edgeR, design matrix was a simple two-group unpaired comparison, the covariates were not included except for the groupings, the exact statistical contrasts were two biological groups. Differential expressions were determined using a threshold false discovery rate (FDR) of <0.1 and fold change >2. After normalization by sequencing depth and square root transformations, MDS plot was utilized to display hierarchical clustering. The normalization by sequencing depth and square root transformation was only for visualization. The differential expression analysis was conducted with edgeR using raw count data within a negative binomial framework. We used the plotMDS function from the edgeR package with default parameters. This function will use the 500 genes with the largest absolute log2 fold changes for each pair of samples. All fold changes reported in the manuscript refer to log2 fold changes. By convention in edgeR, the log2 fold change represents the log2 ratio of the second condition relative to the first condition. Comprehensive functional annotation analysis including KEGG pathway analysis was performed with for genes with FDR < 0.1 when comparing AC and BL groups and FDR < 0.001 for comparison of WT with BL and WT with AC. We used The Database for Annotation, Visualization, and Integrated Discovery (DAVID, https://davidbioinformatics.nih.gov) for KEGG pathway analysis. The analysis was carried out in March 2019. The default setting of DAVID was the setting for the background gene universe. The number of input genes was different from analysis to analysis; all genes satisfy all the conditions.

## 5. Conclusions

In summary, gene expression analysis of 16 esophageal tissue samples demonstrated 3062 genes which were differentially expressed among the tissue types. Concordant with their phenotypic similarity, BL and AC clusters displayed some overlap but were clearly defined from the WT cluster. KEGG pathway and functional annotation analyses of differentially expressed genes identified several pathways/processes associated with stage(s) of AC progression: compared to WT, pathways associated with the extracellular matrix and its signaling, focal adhesion, and cellular metabolism were commonly upregulated in BL and AC tissues, while keratinization and Ras, Rap1, and Hippo signaling were commonly downregulated in BL and AC tissues. Although significant similarity between BL and AC samples was seen, metabolic pathways were differentially upregulated in BL samples. The results of this study provide a valuable data source for the identification of candidate genes and pathways that require independent validation. Further studies in larger, independent cohorts and functional validation will be necessary to determine their biological and potential clinical significance in AC.

## Figures and Tables

**Table 1:** Sample characteristics.

Sample IDs	Histology	RNA Concentrations	RIN Values	DV200 Values
WT1	Stratified squamous epithelium	216 pg/µL	2.30	65%
WT2	Stratified squamous epithelium	1444 pg/µL	2	75%
WT3	Stratified squamous epithelium	3116 pg/µL	2.20	99%
WT4	Stratified squamous epithelium	2248 pg/µL	2	58%
WT5	Stratified squamous epithelium	670 pg/µL	2.20	60%
WT6	Stratified squamous epithelium	109 pg/µL	2.20	55%
AC1	Esophageal adenocarcinoma	2646 pg/µL	2.10	58%
AC2	Esophageal adenocarcinoma	130 pg/µL	N/A (not available)	90%
AC3	Esophageal adenocarcinoma	940 pg/µL	2.40	59%
AC4	Esophageal adenocarcinoma	839 pg/µL	1	65%
AC5	Esophageal adenocarcinoma	848 pg/µL	2	56%
AC6	Esophageal adenocarcinoma	335 pg/µL	2	68%
BL1	Barrett’s esophagus with low-grade dysplasia	32 pg/µL	1.10	72%
BL2	Barrett’s esophagus with low-grade dysplasia	611 pg/µL	2.20	63%
BL3	Barrett’s esophagus with low-grade dysplasia	285 pg/µL	2.20	84%
BL4	Barrett’s esophagus with low-grade dysplasia	148 pg/µL	2.3	84%

## Data Availability

The original contributions presented in this study are included in the article/Supplementary Material. Further inquiries can be directed to the corresponding author.

## Supplementary Materials

The following supporting information can be downloaded at https://www.mdpi.com/article/10.3390/ijms27188164/s1.

## Abbreviations

esophageal adenocarcinoma (AC), gastro-esophageal reflux disease (GERD). RNA sequencing (RNA-seq), normal esophagus (WT), Barrett’s esophagus with low-grade dysplasia (BL), laser capture microdissection (LCM), formalin-fixed paraffin-embedded (FFPE), Kyoto Encyclopedia of Genes and Genomes (KEGG), Phosphoinositide 3-kinase (PI3K) and Protein Kinase B (PI3K-AKT), Ras-associated protein (RAP-1), Alanyl Aminopeptidase, Membrane/CD13 (ANPEP), Regenerating Family Member 4 (REG4), Meprin A Subunit Alpha (MEP1A), Reverse Transcription Quantitative Real-Time PCR (qRT-PCR), esophageal cancer (EC), squamous cell carcinoma (SCC), false discovery rate (FDR), Multimodal scaling (MDS).
