# Supplementary material for: Transcriptomic Profiling of Laser Capture Microdissected FFPE Esophageal Tissues Identifies Candidate Genes and Pathways Associated with Esophageal Adenocarcinoma
Source: Int J Mol Sci. 2026 Sep 14;27(18):8164. doi: 10.3390/ijms27188164 (PMC13607975; doi:10.3390/ijms27188164)
Supplement: Supplementary file 1 [file ijms-27-08164-s001.zip › Supplementary Figure S1.pdf]

Electropherogram Summary

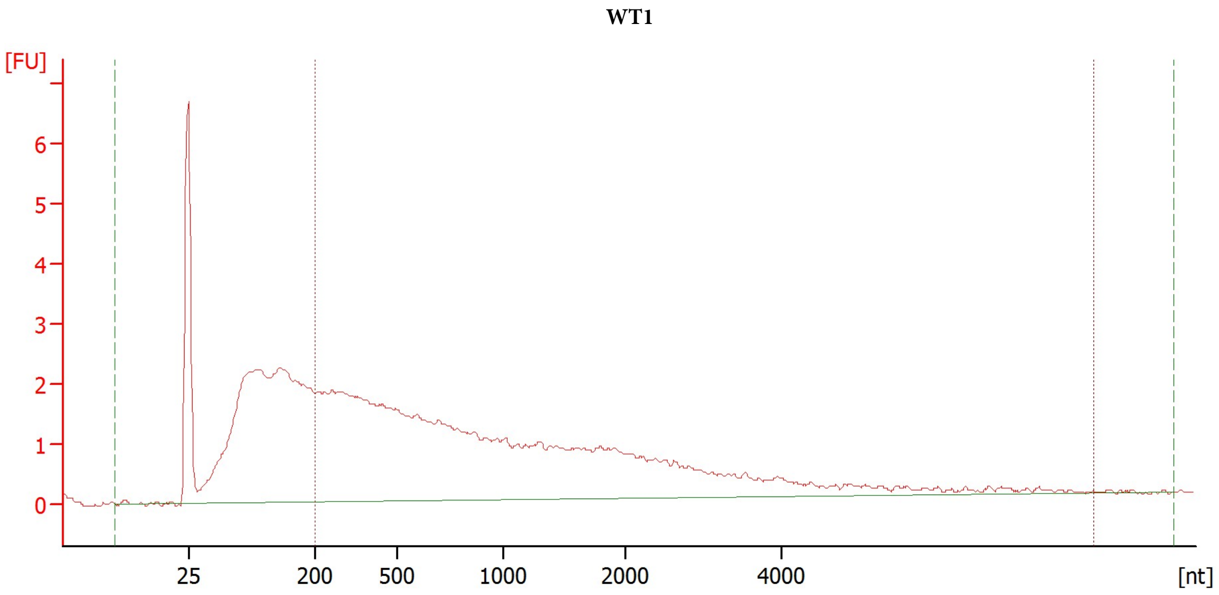

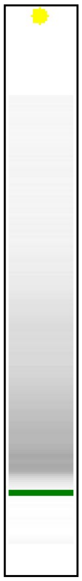

Overall Results for sample:

RNA Area:	93.6	Result Flagging Color:	
RNA Concentration:	216 pg/µl	Result Flagging Label:	RIN: 2.30 ; DV200 50-70%
rRNA Ratio [28s / 18s]:	0.0		
RNA Integrity Number (RIN):	2.3 (B.02.08, Anomaly Threshold(s) manually adapted)	Corr. Area 1:	60.8

Region table for sample:

Name	From [nt]	To [nt]	Corr. Area	% of Total	Colo r
DV200	200	8,000	60.8	65	

Electropherogram Summary

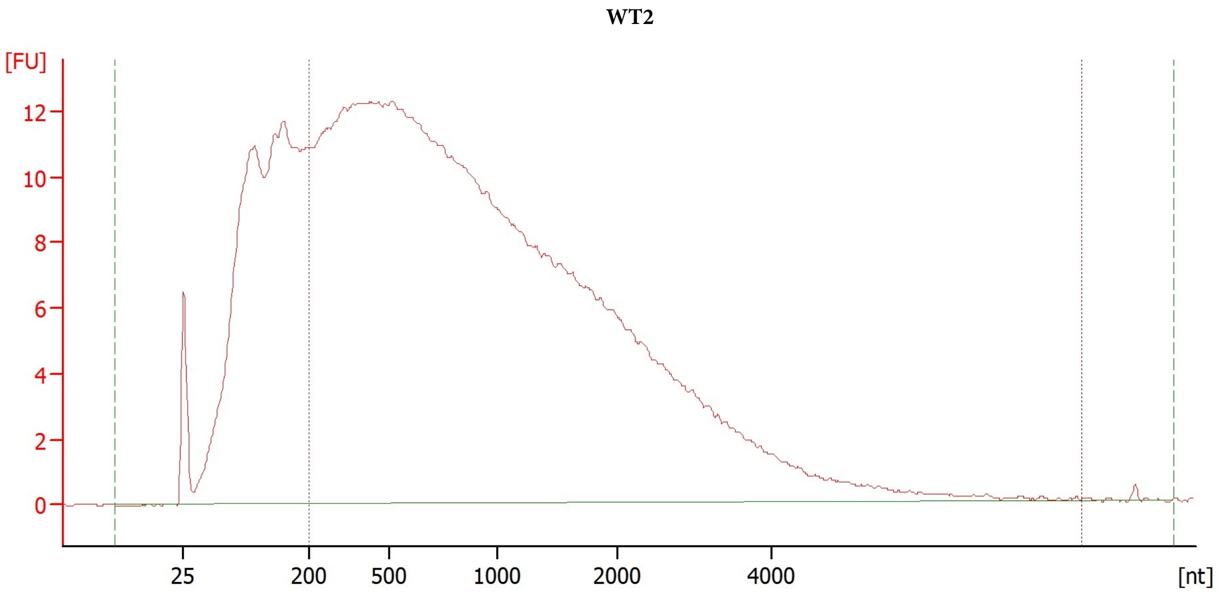

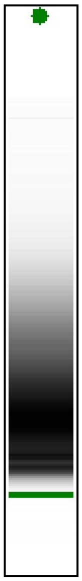

Overall Results for sample:

RNA Area:	625.3	Result Flagging Color:	
RNA Concentration:	1,444 pg/µl	Result Flagging Label:	RIN:2 ; DV200 > 70%
rRNA Ratio [28s / 18s]:	0.0	Corr. Area 1:	469.1
RNA Integrity Number (RIN):	2 (B.02.08, Anomaly Threshold(s) manually adapted)		

Region table for sample:

Name	From [nt]	To [nt]	Corr. Area	% of Total	Colo r
DV200	200	8,000	469.1	75	

Electropherogram Summary

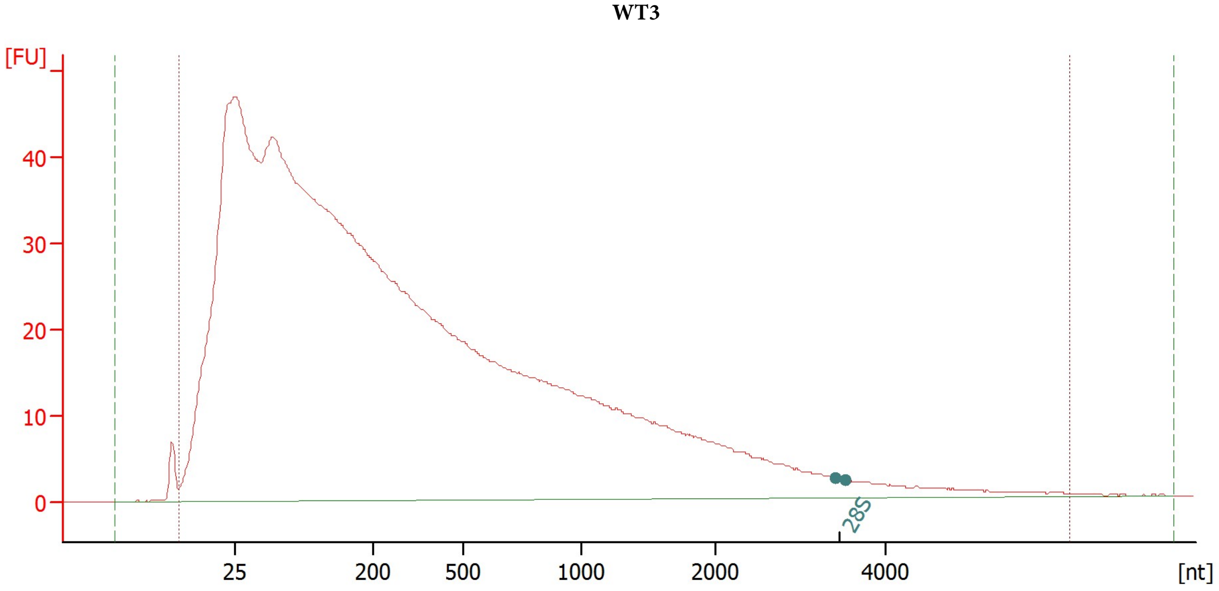

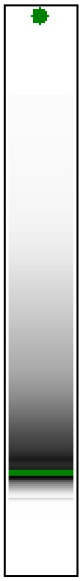

Overall Results for sample:

RNA Area:	1,349.1	Result Flagging Color:	
RNA Concentration:	3,116 pg/µl	Result Flagging Label:	RIN: 2.20 ; DV200 > 70%
rRNA Ratio [28s / 18s]:	0.0		
RNA Integrity Number (RIN):	2.2 (B.02.08, Anomaly Threshold(s) manually adapted)	Corr. Area 1:	1,346.6

Fragment table for sample:

Name	Start Size [nt]	End Size [nt]	Area	% of total Area
28S	3,403	3,528	0.1	0.0

Region table for sample:

Name	From [nt]	To [nt]	Corr. Area	% of Total	Colo r
DV200	0	6,144	1,346.6	99	

Electropherogram Summary

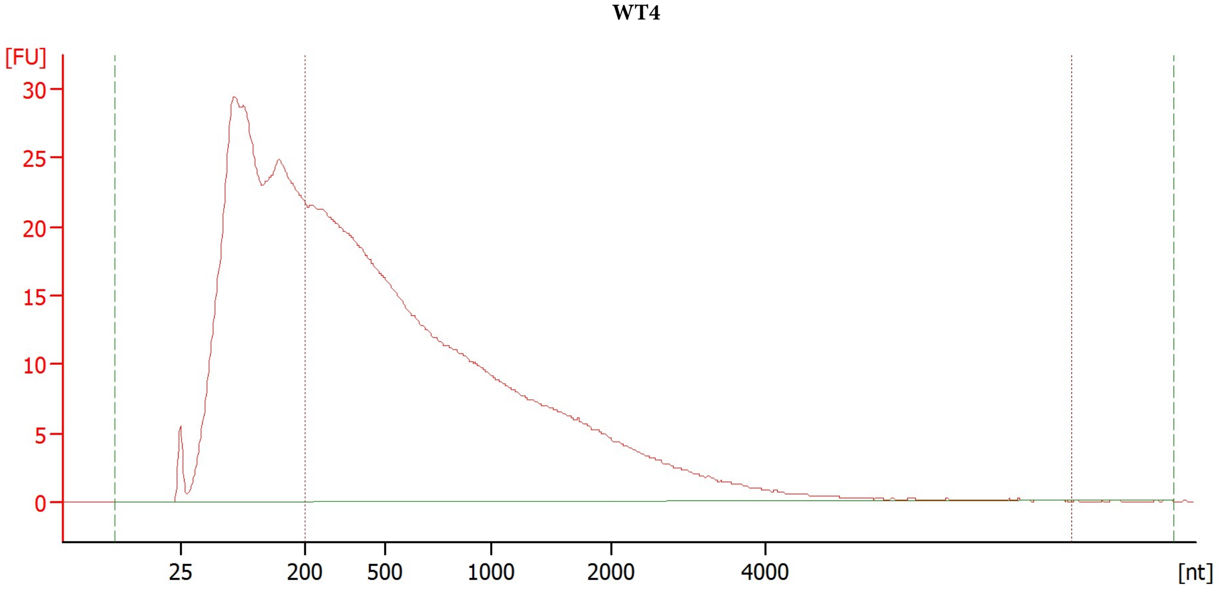

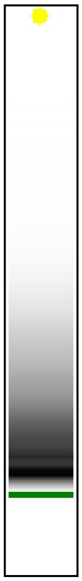

Overall Results for sample:

RNA Area:	973.4	Result Flagging Color:	
RNA Concentration:	2,248 pg/µl	Result Flagging Label:	RIN:2 ; DV200 50-70%
rRNA Ratio [28s / 18s]:	0.0		
RNA Integrity Number (RIN):	2 (B.02.08, Anomaly Threshold(s) manually adapted)	Corr. Area 1:	560.5

Region table for sample:

Name	From [nt]	To [nt]	Corr. Area	% of Total	Color
DV200	200	8,000	560.5	58	

Electropherogram Summary

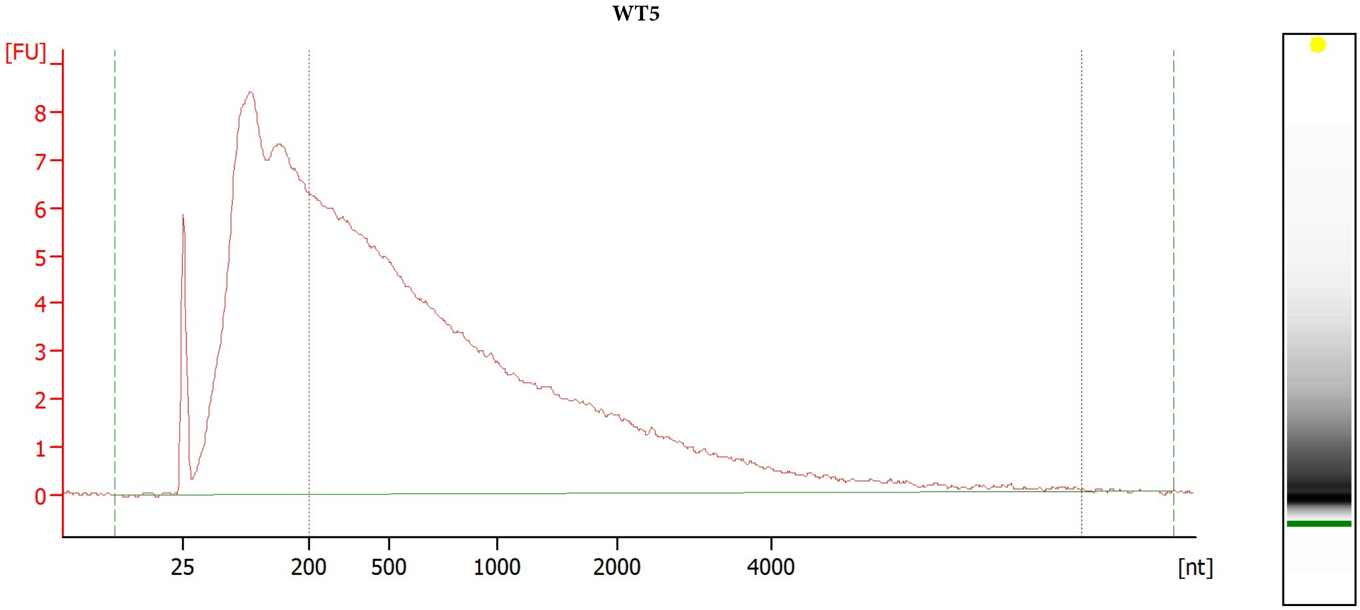

Overall Results for sample:

RNA Area:	290.1	Result Flagging Color:	
RNA Concentration:	670 pg/µl	Result Flagging Label:	RIN: 2.20 ; DV200 50-70%
rRNA Ratio [28s / 18s]:	0.0		
RNA Integrity Number (RIN):	2.2 (B.02.08, Anomaly Threshold(s) manually adapted)	Corr. Area 1:	174.6

Region table for sample:

Name	From [nt]	To [nt]	Corr. Area	% of Total	Color
DV200	200	8,000	174.6	60	

Electropherogram Summary

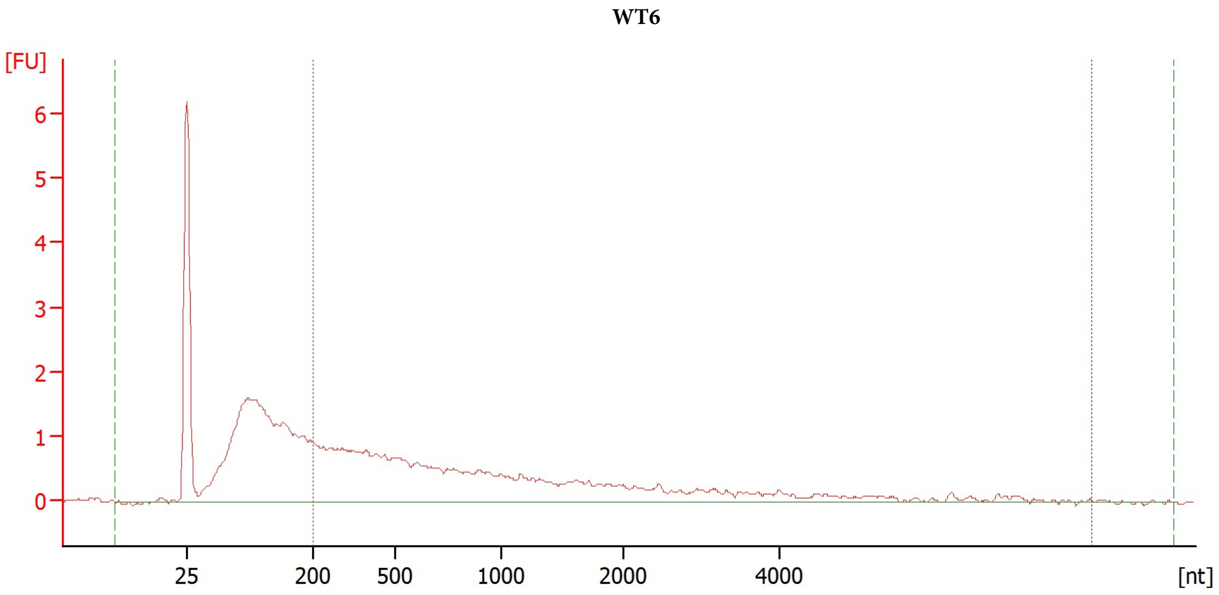

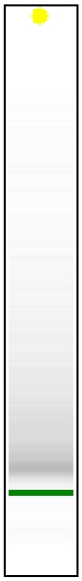

Overall Results for sample:

RNA Area:	47.0	Result Flagging Color:	
RNA Concentration:	109 pg/µl	Result Flagging Label:	RIN: 2.20 ; DV200 50-70%
rRNA Ratio [28s / 18s]:	0.0		
RNA Integrity Number (RIN):	2.2 (B.02.08, Anomaly Threshold(s) manually adapted)	Corr. Area 1:	26.0

Region table for sample:

Name	From [nt]	To [nt]	Corr. Area	% of Total	Colo r
DV200	200	8,000	26.0	55	

Electropherogram Summary

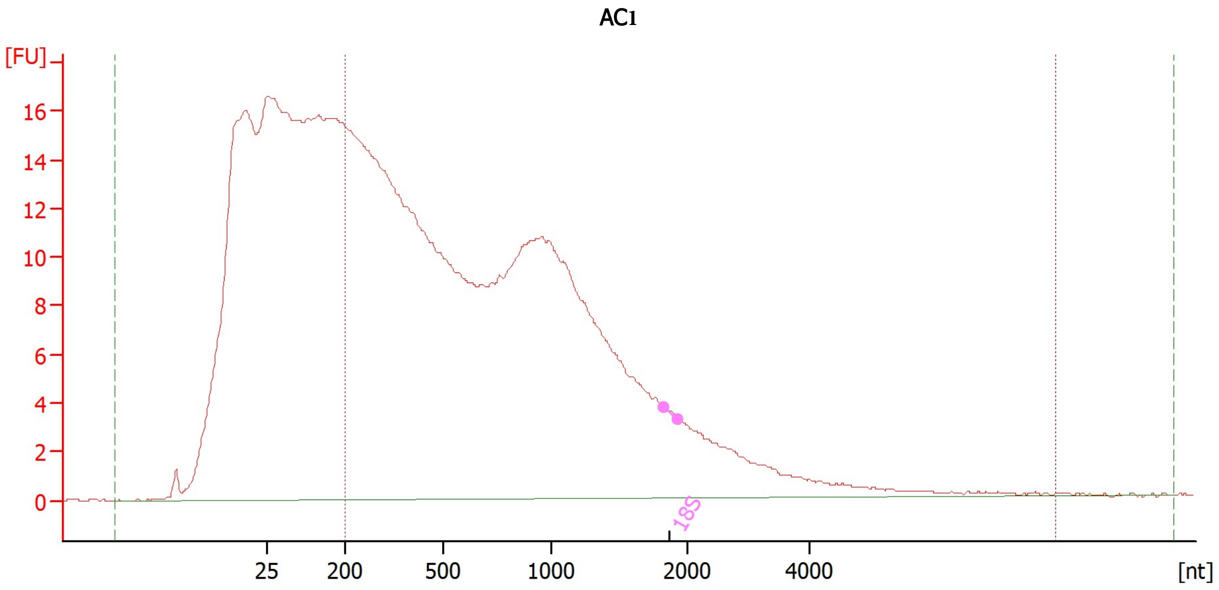

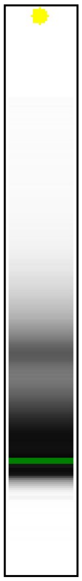

Overall Results for sample:

RNA Area:	739.4	Result Flagging Color:	
RNA Concentration:	2,646 pg/µl	Result Flagging Label:	RIN: 2.10 ; DV200 50-70%
rRNA Ratio [28s / 18s]:	0.0		
RNA Integrity Number (RIN):	2.1 (B.02.08, Anomaly Threshold(s) manually adapted)	Corr. Area 1:	428.6

Fragment table for sample:

Name	Start Size [nt]	End Size [nt]	Area	% of total Area
18S	1,815	1,923	0.1	0.0

Region table for sample:

Name	From [nt]	To [nt]	Corr. Area	% of Total	Colo r
DV200	200	8,000	428.6	58	

Electropherogram Summary

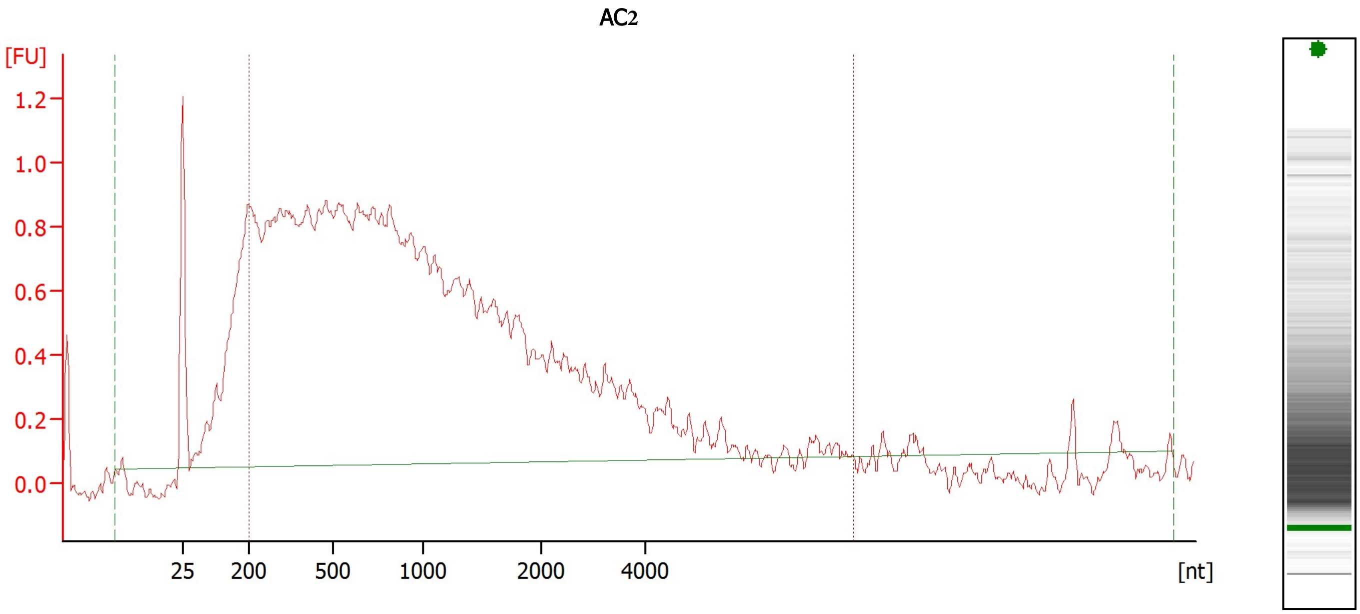

Overall Results for sample:

RNA Area:	36.2	Result Flagging Color:	
RNA Concentration:	130 pg/µl	Result Flagging Label:	RIN N/A ; DV200 > 70%
rRNA Ratio [28s / 18s]:	0.0		
		Corr. Area 1:	32.6

Region table for sample:

Name	From [nt]	To [nt]	Corr. Area	% of Total	Color
DV200	200	8,000	32.6	90	

Electropherogram Summary

AC3

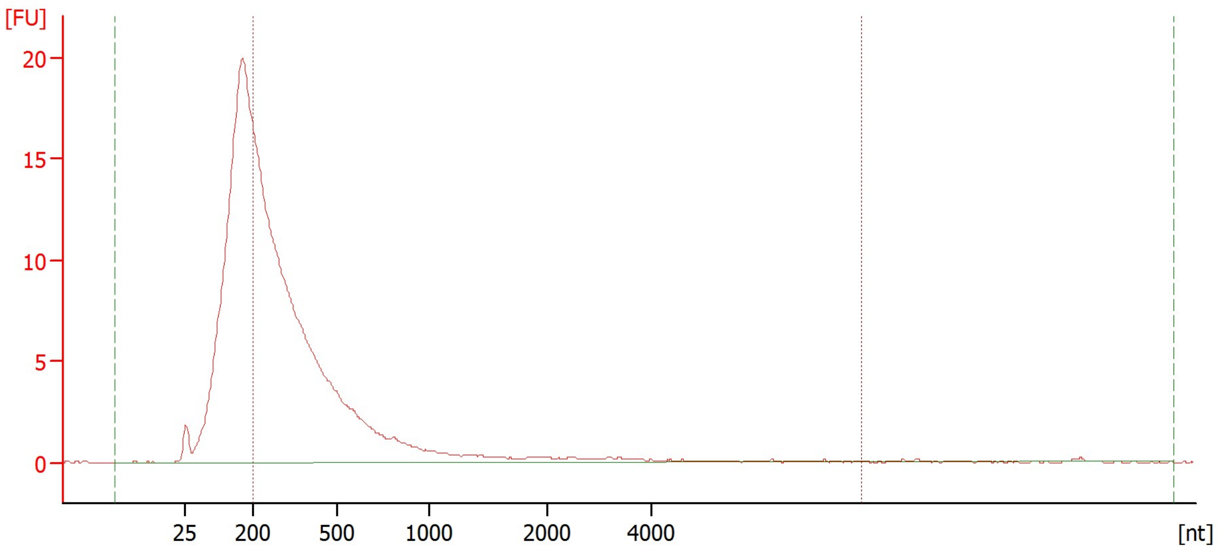

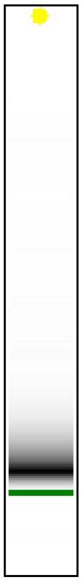

Overall Results for sample:

RNA Area:	262.6	Result Flagging Color:	
RNA Concentration:	940 pg/µl	Result Flagging Label:	RIN: 2.40 ; DV200 50-70%
rRNA Ratio [28s / 18s]:	0.0		
RNA Integrity Number (RIN):	2.4 (B.02.08, Anomaly Threshold(s) manually adapted)	Corr. Area 1:	155.1

Region table for sample:

Name	From [nt]	To [nt]	Corr. Area	% of Total	Colo r
DV200	200	8,000	155.1	59	

Electropherogram Summary

AC4

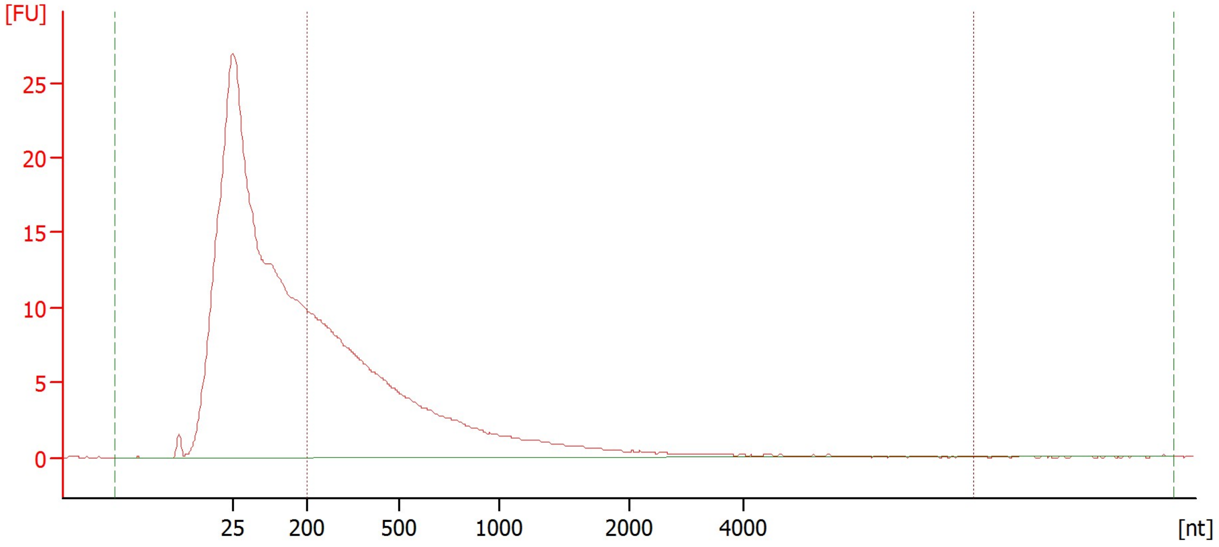

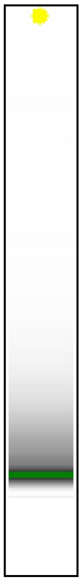

Overall Results for sample:

RNA Area:	234.5	Result Flagging Color:	
RNA Concentration:	839 pg/µl	Result Flagging Label:	RIN:1 ; DV200 50-70%
rRNA Ratio [28s / 18s]:	0.0		
RNA Integrity Number (RIN):	1 (B.02.08, Anomaly Threshold(s) manually adapted)	Corr. Area 1:	153.4

Region table for sample:

Name	From [nt]	To [nt]	Corr. Area	% of Total	Colo r
DV200	200	8,000	153.4	65	

Electropherogram Summary

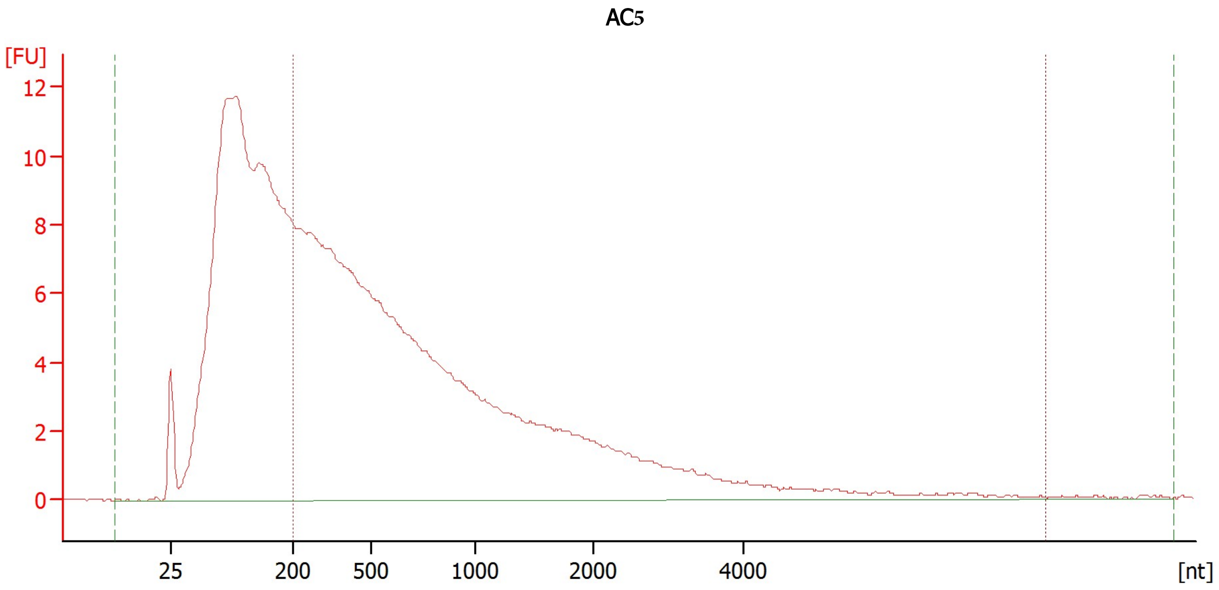

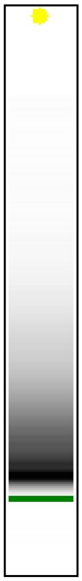

Overall Results for sample:

RNA Area:	367.3	Result Flagging Color:	
RNA Concentration:	848 pg/µl	Result Flagging Label:	RIN:2 ; DV200 50-70%
rRNA Ratio [28s / 18s]:	0.0		
RNA Integrity Number (RIN):	2 (B.02.08, Anomaly Threshold(s) manually adapted)	Corr. Area 1:	206.4

Region table for sample:

Name	From [nt]	To [nt]	Corr. Area	% of Total	Colo r
DV200	200	8,000	206.4	56	

Electropherogram Summary

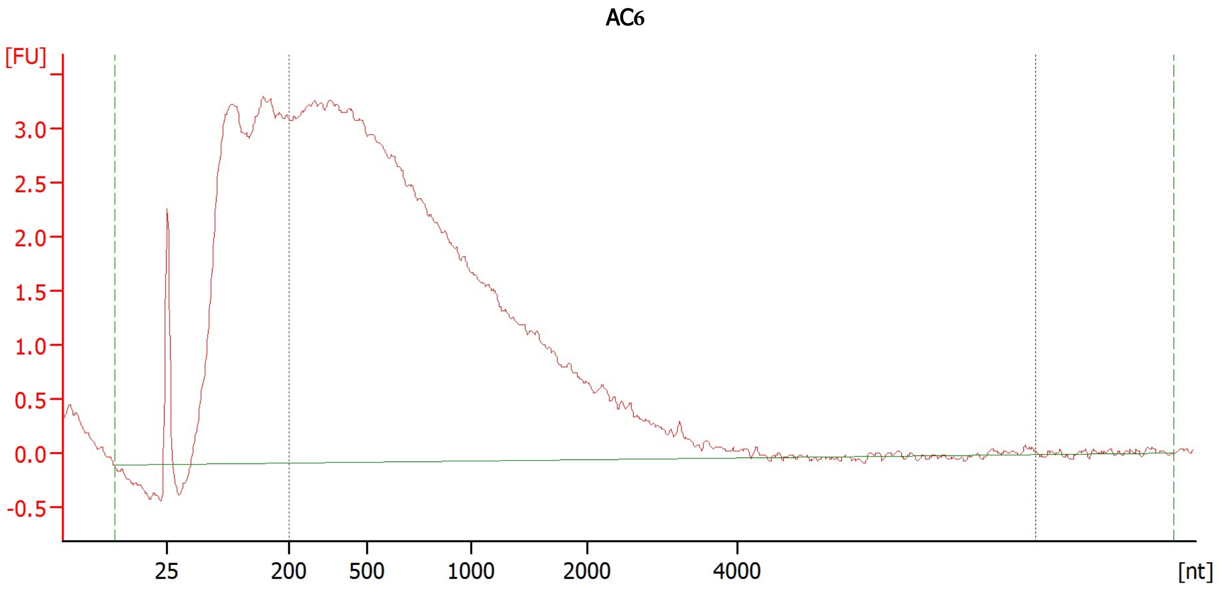

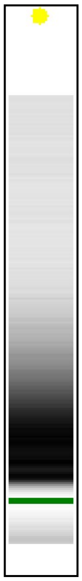

Overall Results for sample:

RNA Area:	145.0	Result Flagging Color:	
RNA Concentration:	335 pg/µl	Result Flagging Label:	RIN:2 ; DV200 50-70%
rRNA Ratio [28s / 18s]:	0.0		
RNA Integrity Number (RIN):	2 (B.02.08, Anomaly Threshold(s) manually adapted)	Corr. Area 1:	99.2

Region table for sample:

Name	From [nt]	To [nt]	Corr. Area	% of Total	Colo r
DV200	200	8,000	99.2	68	

Electropherogram Summary

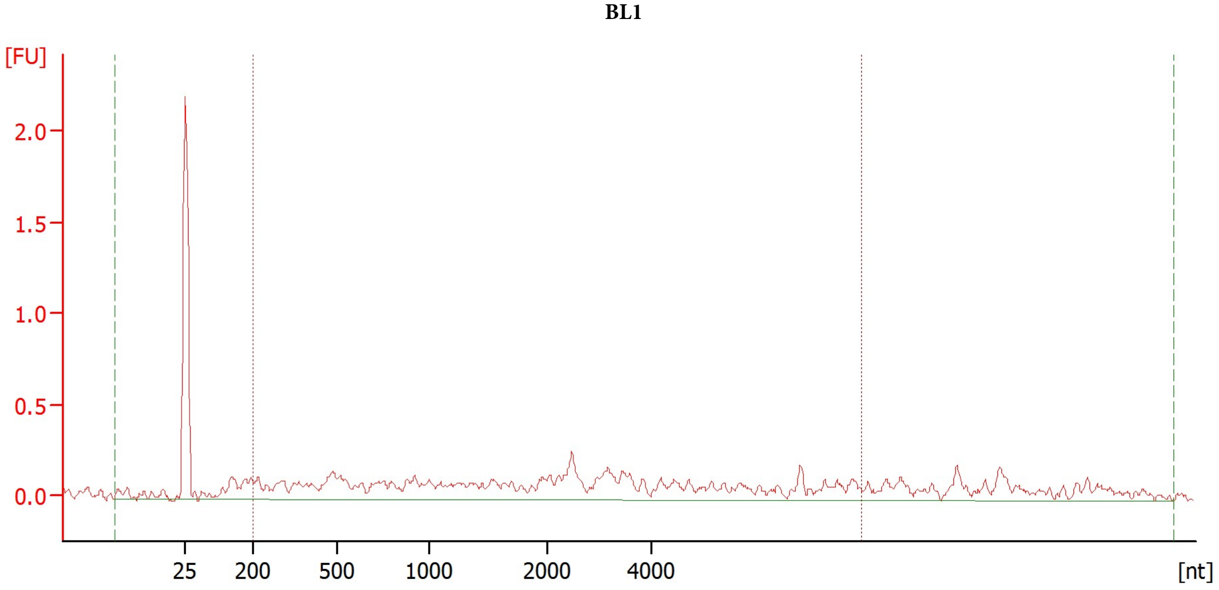

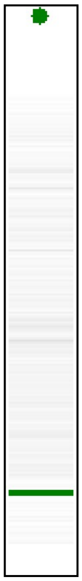

Overall Results for sample:

RNA Area:	8.8	Result Flagging Color:	
RNA Concentration:	32 pg/µl	Result Flagging Label:	RIN: 1.10 ; DV200 > 70%
rRNA Ratio [28s / 18s]:	0.0		
RNA Integrity Number (RIN):	1.1 (B.02.08, Anomaly Threshold(s) manually adapted)	Corr. Area 1:	6.3

Region table for sample:

Name	From [nt]	To [nt]	Corr. Area	% of Total	Color
DV200	200	8,000	6.3	72	

Electropherogram Summary

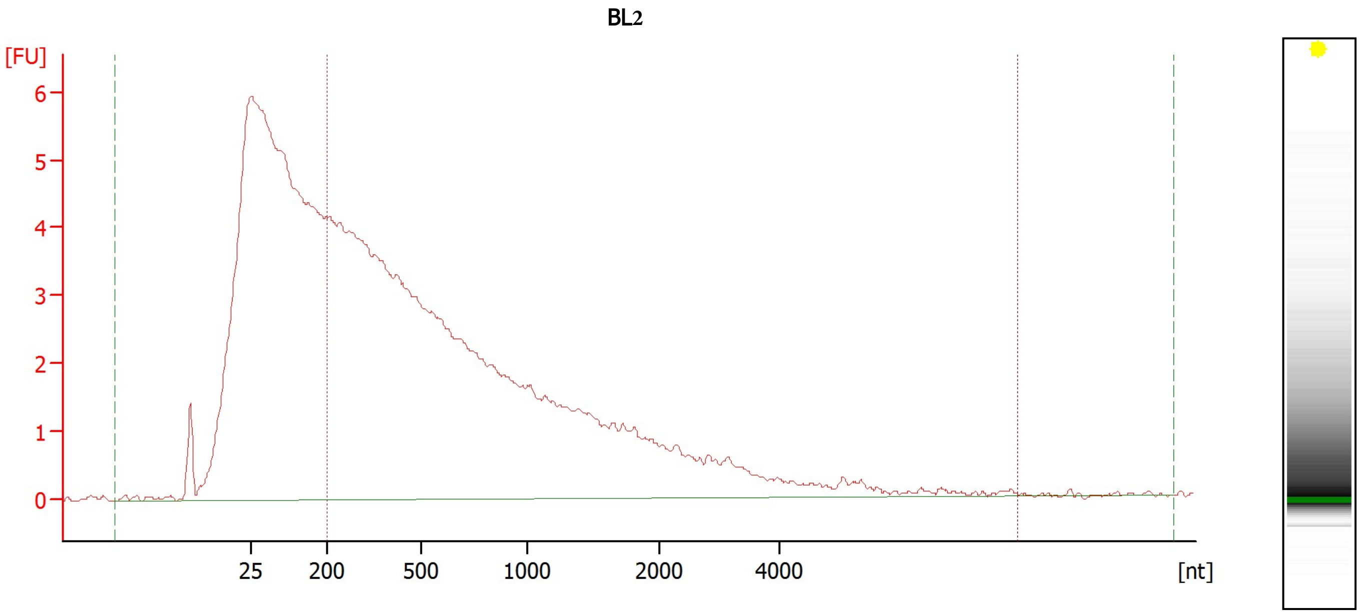

Overall Results for sample:

RNA Area:	170.6	Result Flagging Color:	
RNA Concentration:	611 pg/µl	Result Flagging Label:	RIN: 2.20 ; DV200 50-70%
rRNA Ratio [28s / 18s]:	0.0		
RNA Integrity Number (RIN):	2.2 (B.02.08, Anomaly Threshold(s) manually adapted)	Corr. Area 1:	107.4

Region table for sample:

Name	From [nt]	To [nt]	Corr. Area	% of Total	Colo r
DV200	200	8,000	107.4	63	

Electropherogram Summary

BL3

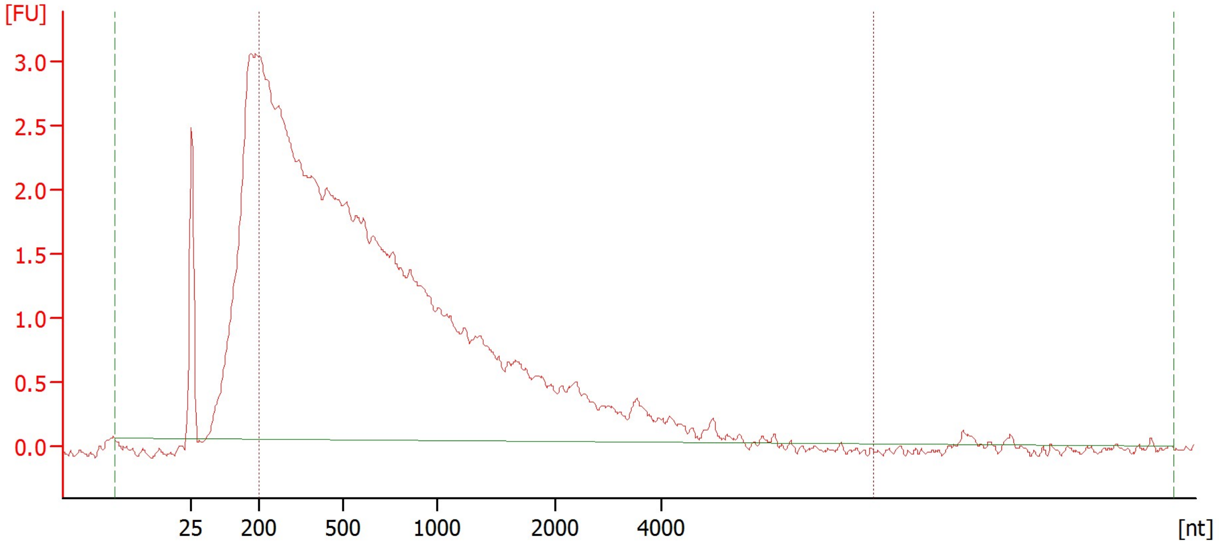

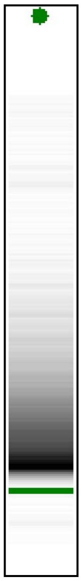

Overall Results for sample:

RNA Area:	79.6	Result Flagging Color:	
RNA Concentration:	285 pg/µl	Result Flagging Label:	RIN: 2.20 ; DV200 > 70%
rRNA Ratio [28s / 18s]:	0.0		
RNA Integrity Number (RIN):	2.2 (B.02.08, Anomaly Threshold(s) manually adapted)	Corr. Area 1:	67.0

Region table for sample:

Name	From [nt]	To [nt]	Corr. Area	% of Total	Color
DV200	200	8,000	67.0	84	

Electropherogram Summary

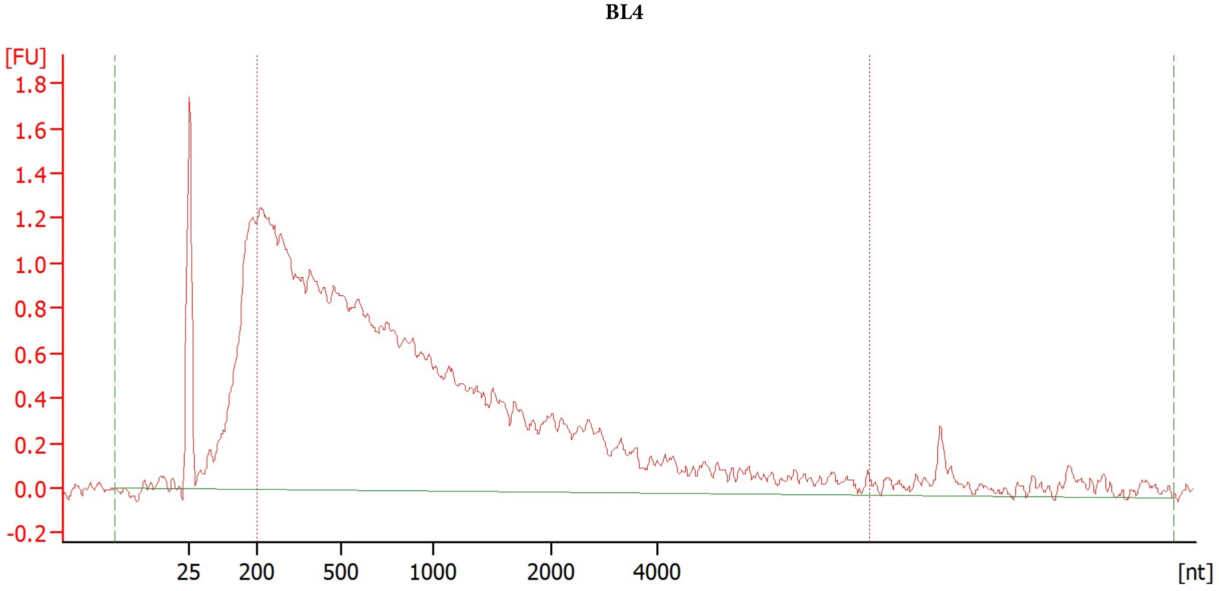

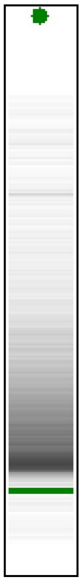

Overall Results for sample:

RNA Area:	41.4	Result Flagging Color:	
RNA Concentration:	148 pg/µl	Result Flagging Label:	RIN: 2.30 ; DV200 > 70%
rRNA Ratio [28s / 18s]:	0.0		
RNA Integrity Number (RIN):	2.3 (B.02.08, Anomaly Threshold(s) manually adapted)	Corr. Area 1:	34.8

Region table for sample:

Name	From [nt]	To [nt]	Corr. Area	% of Total	Colo r
DV200	200	8,000	34.8	84	
