# Supplementary material for: Transcriptomic Profiling of Laser Capture Microdissected FFPE Esophageal Tissues Identifies Candidate Genes and Pathways Associated with Esophageal Adenocarcinoma
Source: Int J Mol Sci. 2026 Sep 14;27(18):8164. doi: 10.3390/ijms27188164 (PMC13607975; doi:10.3390/ijms27188164)
Supplement: Supplementary file 1 [file ijms-27-08164-s001.zip › Supplementary Figure S2.pdf]

For the best experience, open this PDF portfolio in Acrobat X or Adobe Reader X, or later.

Get Adobe Reader Now!
