# Supplementary material for: Transcriptomic Profiling of Laser Capture Microdissected FFPE Esophageal Tissues Identifies Candidate Genes and Pathways Associated with Esophageal Adenocarcinoma
Source: Int J Mol Sci. 2026 Sep 14;27(18):8164. doi: 10.3390/ijms27188164 (PMC13607975; doi:10.3390/ijms27188164)
Supplement: Supplementary file 1 [file ijms-27-08164-s001.zip › Supplementary Table S1_mapstat.csv.pdf]

Supplementary Table S2: Summary statistics for RNA sequencing mapping.

Sample IDs	total	no_feature	ambiguous	too_low_aQual	not_aligned	alignment_not_unique	uniquely_mapped
AC1	41462101	12963319	5588529	4461564	6668620	5858675	5921394
AC2	41976411	19810672	4062680	4078813	5565727	3717606	4740913
AC3	41609198	10537563	4040116	2482619	14563807	5450783	4534310
AC4	35887887	13837697	3317882	2932965	8732359	3766155	3300829
AC5	48582132	16882600	5583404	4054156	10785123	5330478	5946371
AC6	46219278	20592339	5931803	4309770	4024256	5008783	6352327
BL1	35486521	5588372	2733407	2702063	17025970	5107492	2329217
BL2	37114007	14260332	3852892	3328068	7756612	3922026	3994077
BL3	43160313	16717920	4218525	3784900	8579927	5385299	4473742
BL4	34510563	12116503	3504541	3023156	6841415	4895144	4129804
WT1	44660801	16020354	5017334	4634191	7892599	5453332	5642991
WT2	49963600	19198090	7634915	5513612	3542521	5711386	8363076
WT3	57047028	13938685	6342861	4726370	17371713	7992036	6675363
WT4	45026725	10821847	6141271	4028956	8260625	10114824	5659202
WT5	45184505	14351146	6174946	4662919	6644078	6109936	7241480
WT6	45775855	13872187	5724570	3792904	9068739	7057969	6259486
