# Supplementary material for: Transcriptomic Profiling of Laser Capture Microdissected FFPE Esophageal Tissues Identifies Candidate Genes and Pathways Associated with Esophageal Adenocarcinoma
Source: Int J Mol Sci. 2026 Sep 14;27(18):8164. doi: 10.3390/ijms27188164 (PMC13607975; doi:10.3390/ijms27188164)
Supplement: Supplementary file 1 [file ijms-27-08164-s001.zip › Supplementary Table S2_WT vs BL-DE.pdf]

GeneSymb	FDR	pval	log2FC	log2CPM
SI	9.79E-35	7.93E-39	12.86811	7.392578
FABP1	2.23E-27	3.62E-31	11.09642	5.628309
ANPEP	1.10E-26	2.68E-30	8.17457	9.504917
LOC38960[illegible]	3.84E-25	1.45E-28	8.38446	5.550186
TM4SF20	3.84E-25	1.55E-28	8.194249	5.109969
MUC5B	5.94E-22	2.89E-25	4.721855	8.407817
MYO7B	3.62E-21	2.05E-24	6.558733	6.950477
VIL1	1.07E-20	6.94E-24	7.113437	6.047874
CHST5	3.30E-20	2.40E-23	9.546093	4.731768
SLC28A2	8.17E-20	7.26E-23	7.540449	5.456735
LEFTY1	8.17E-20	7.28E-23	7.864942	5.396577
SLC26A3	2.36E-19	2.29E-22	8.590302	4.507325
CDX1	3.48E-19	3.67E-22	8.57034	4.486835
FCGBP	4.23E-19	4.80E-22	6.814422	11.26307
TMPRSS15	3.01E-18	3.66E-21	10.86437	4.249549
F5	6.16E-18	7.99E-21	8.142968	6.523947
LYZ	9.40E-18	1.29E-20	6.104662	9.903605
LGALS4	1.66E-17	2.42E-20	7.57706	8.327479
AGR3	4.16E-17	6.40E-20	7.861366	4.131708
GPA33	4.50E-16	7.28E-19	7.218787	5.599791
MEP1A	7.90E-16	1.34E-18	8.80091	5.473378
TFF3	2.18E-15	3.89E-18	7.727274	5.684583
DUSP27	2.59E-15	4.83E-18	7.069153	5.440598
SLC6A19	2.80E-15	5.44E-18	9.117573	3.686473
SPINK4	3.31E-15	6.70E-18	7.201043	4.160348
PCK1	4.84E-15	1.02E-17	6.814608	5.945009
ANKS4B	1.12E-14	2.46E-17	6.847729	4.792009
HOXA13	2.67E-14	6.06E-17	8.290835	3.939743
ADRA2A	2.68E-14	6.30E-17	5.454553	4.741147
CFTR	4.73E-14	1.15E-16	6.709626	6.760555
SLC3A1	5.22E-14	1.31E-16	7.217646	4.343029
PRR15	8.78E-14	2.28E-16	6.477636	4.292773
TTLL6	2.77E-13	7.40E-16	8.318683	3.533719
PPP1R1B	3.23E-13	8.89E-16	7.683959	4.186208
MISP	1.04E-12	2.94E-15	6.70791	6.560948
EPCAM	2.21E-12	6.43E-15	4.045293	7.917221
REG4	3.50E-12	1.05E-14	7.321924	5.020132
MIR194-2[illegible]	4.07E-12	1.25E-14	7.101416	3.983691
CRYZL2P	1.06E-11	3.34E-14	3.181196	6.413866
HOXC10	6.07E-11	1.97E-13	9.016812	3.587736
CDX2	8.10E-11	2.69E-13	7.783138	3.023987
HOXB13	1.33E-10	4.51E-13	9.514043	2.939989
DPP4	5.03E-10	1.75E-12	4.729302	6.181684
ONECUT2	5.17E-10	1.84E-12	7.22962	6.575202

EPHA10	5.53E-10	2.02E-12	4.485321	4.466542
GUCY2C	6.96E-10	2.59E-12	3.449858	5.458822
HSD17B2	1.18E-09	4.49E-12	5.583906	5.128223
TRIM31	2.21E-09	8.58E-12	7.952804	3.583719
KCNE3	4.09E-09	1.62E-11	5.222203	6.89191
FAM83E	4.66E-09	1.89E-11	3.748839	5.415257
KIAA1211	5.52E-09	2.28E-11	4.592441	5.694129
RNF224	6.46E-09	2.72E-11	-9.26094	3.269079
MUC2	6.46E-09	2.77E-11	8.980037	10.58335
ITLN1	6.58E-09	2.88E-11	6.91196	5.773074
ANXA4	6.91E-09	3.08E-11	2.5571	7.140617
TM6SF2	1.06E-08	4.79E-11	6.997643	3.728718
CELSR3	1.27E-08	5.85E-11	3.834553	4.826644
FAM171A1	1.34E-08	6.29E-11	4.031252	5.707967
OTC	1.51E-08	7.20E-11	7.774416	3.46474
KRT20	1.71E-08	8.31E-11	11.10717	4.492302
PHGR1	2.49E-08	1.23E-10	6.924104	4.00627
PURPL	2.62E-08	1.31E-10	8.041851	3.218717
MYRFL	2.67E-08	1.36E-10	6.027348	3.367509
LINC01814	3.04E-08	1.57E-10	4.729222	3.86838
GCNT3	3.77E-08	1.98E-10	3.269111	5.799448
PADI2	3.89E-08	2.08E-10	4.464909	5.190583
GALM	4.71E-08	2.55E-10	3.37124	4.948966
NMUR2	5.21E-08	2.87E-10	7.150248	2.717474
SCNN1G	5.55E-08	3.10E-10	-5.77848	5.766844
BAIAP2L2	5.65E-08	3.20E-10	3.325419	5.725069
HOXB5	5.68E-08	3.27E-10	7.497134	2.74759
CLCA1	5.70E-08	3.33E-10	5.652167	5.559437
CMBL	5.87E-08	3.47E-10	4.349853	5.866502
VILL	5.90E-08	3.57E-10	3.372286	6.140099
GALNT6	5.90E-08	3.58E-10	3.059013	5.865861
BHLHE40	8.48E-08	5.22E-10	-3.74385	9.745659
MUC3A	9.11E-08	5.68E-10	6.816033	9.582124
SPIRE2	1.41E-07	8.92E-10	3.342979	4.852424
TSPAN1	1.52E-07	9.72E-10	5.305495	6.045905
HCG22	1.68E-07	1.09E-09	-6.11129	5.691869
HAPLN4	1.76E-07	1.16E-09	6.571378	2.783385
AMN	1.76E-07	1.17E-09	3.774671	5.231241
NOS2	1.86E-07	1.25E-09	6.681674	3.592207
FGFR3	2.60E-07	1.77E-09	-6.84624	3.41164
SLC40A1	2.72E-07	1.88E-09	2.981534	6.788965
HKDC1	3.04E-07	2.12E-09	5.18641	5.432188
FUT4	3.28E-07	2.31E-09	3.076276	4.704484
HOXA11-A!	3.34E-07	2.38E-09	9.031037	2.470528
SPRR3	3.56E-07	2.57E-09	-6.34531	4.856718

KCNK5	4.17E-07	3.04E-09	3.361201	6.039218
FER1L6-AS1	4.36E-07	3.22E-09	5.958229	3.336673
GALNT8	4.96E-07	3.70E-09	8.917722	2.378107
ARHGEF38	5.13E-07	3.86E-09	5.573631	4.687226
EREG	5.17E-07	3.94E-09	-3.97375	7.025007
HSD17B11	5.70E-07	4.39E-09	3.700227	4.9812
MIR210HG	7.86E-07	6.11E-09	-3.07449	5.138665
AADACP1	8.60E-07	6.75E-09	7.008649	2.247386
SMPD3	8.87E-07	7.04E-09	3.161298	6.073262
GJB6	9.05E-07	7.26E-09	-6.36211	4.870789
C5orf66-AS	9.82E-07	7.95E-09	-5.1155	4.295322
LOC101928	1.33E-06	1.09E-08	8.974469	2.417101
S100A11	1.35E-06	1.11E-08	-3.36876	9.689715
FEZF1-AS1	1.35E-06	1.13E-08	6.944487	2.641909
TM4SF4	1.46E-06	1.23E-08	6.474425	5.767961
A2M	1.51E-06	1.28E-08	2.910976	9.221827
ZIC2	1.51E-06	1.30E-08	5.984417	2.666007
SLC45A3	1.77E-06	1.54E-08	4.043364	4.074597
IFFO2	1.92E-06	1.68E-08	-3.88563	8.099342
MUC12	1.99E-06	1.75E-08	5.037795	5.716119
DUOX1	2.32E-06	2.07E-08	-6.75191	3.242154
TNFRSF14-	3.39E-06	3.05E-08	2.858712	4.884757
FAM177B	3.54E-06	3.22E-08	4.948918	4.476515
RRAGD	3.74E-06	3.42E-08	-3.40397	6.176945
S100A14	3.75E-06	3.47E-08	-3.18639	10.96295
TACSTD2	3.89E-06	3.62E-08	-3.92481	10.46334
MUC13	4.25E-06	3.99E-08	5.792372	7.321118
ZIC5	4.44E-06	4.25E-08	8.610411	2.103577
BARX2	4.44E-06	4.25E-08	-3.31746	7.793743
KCTD11	4.54E-06	4.38E-08	-3.65201	6.954654
NBPF7	4.73E-06	4.60E-08	8.455686	1.942168
SSTR5-AS1	4.99E-06	4.89E-08	5.481365	2.741083
ESPL1	6.16E-06	6.09E-08	-2.99288	6.230155
CTSZ	6.47E-06	6.45E-08	2.424425	5.918741
ULBP2	6.73E-06	6.76E-08	-6.63578	3.130978
PIGR	6.82E-06	6.91E-08	7.330174	12.60351
RIPK4	7.07E-06	7.21E-08	-3.00664	7.520602
GRHL1	7.41E-06	7.62E-08	-4.35809	9.020353
LOC105373	7.69E-06	7.97E-08	7.686577	2.243437
BTNL3	7.82E-06	8.20E-08	6.724561	4.099489
PRSS12	7.82E-06	8.30E-08	2.288317	5.152909
MOGAT2	7.82E-06	8.31E-08	8.668873	2.146928
KIAA0232	7.82E-06	8.37E-08	-8.63728	2.678596
HCAR2	8.22E-06	8.86E-08	-4.82644	4.778547
PADI3	8.46E-06	9.18E-08	-5.10593	5.133873

PERP	8.71E-06	9.53E-08	-3.6102	11.31236
SULT1C2	9.14E-06	1.01E-07	6.876065	2.624486
AFAP1-AS1	9.14E-06	1.01E-07	3.166674	5.69022
VSNL1	1.02E-05	1.14E-07	-3.56043	5.505633
PARM1	1.21E-05	1.36E-07	2.634147	5.205944
AGT	1.25E-05	1.41E-07	5.705885	3.926042
CREB3L1	1.27E-05	1.45E-07	4.525246	7.653214
NIPAL1	1.28E-05	1.47E-07	-2.34043	6.804587
GNA15	1.33E-05	1.54E-07	-2.99143	5.953841
PAX9	1.39E-05	1.62E-07	-3.07059	7.498641
CELSR2	1.45E-05	1.71E-07	-2.95976	7.408994
SFN	1.45E-05	1.71E-07	-4.03128	9.684595
SPTBN2	1.63E-05	1.94E-07	-3.31305	8.52244
HSPB1	1.64E-05	1.97E-07	-3.48309	10.14123
NECTIN1	1.75E-05	2.11E-07	-3.09475	8.415844
MUC17	1.82E-05	2.21E-07	8.326549	1.823116
ALOX12	1.86E-05	2.28E-07	-4.01018	7.44693
RHPN2	1.96E-05	2.41E-07	3.257372	5.566719
TLN2	2.02E-05	2.51E-07	3.334694	6.523372
NUAK2	2.19E-05	2.73E-07	-2.91685	7.248391
AHDC1	2.27E-05	2.85E-07	-2.32947	7.721545
EVPL	2.28E-05	2.88E-07	-2.97333	10.76343
MICALL1	2.40E-05	3.05E-07	-2.32648	7.278768
FAM102B	2.42E-05	3.09E-07	2.065943	5.440839
P2RY1	2.49E-05	3.20E-07	-4.66729	6.580335
EPHA2	2.55E-05	3.30E-07	-2.9989	8.497769
CLDN3	2.66E-05	3.46E-07	4.944117	3.104283
CRYBG2	2.76E-05	3.62E-07	-3.36876	8.226541
N4BP3	3.08E-05	4.07E-07	-3.15555	5.71838
MAL2	3.13E-05	4.17E-07	-2.74428	9.133115
TMEM154	3.13E-05	4.18E-07	-4.19222	7.669059
SPACA4	3.13E-05	4.21E-07	4.679375	2.931737
CYBA	3.23E-05	4.38E-07	2.430911	6.010621
CTTNBP2	3.23E-05	4.40E-07	-3.30242	6.493967
ZFPM1	3.25E-05	4.45E-07	2.266567	4.783735
CDA	3.49E-05	4.80E-07	-4.28618	6.036464
SIAH2	3.66E-05	5.07E-07	-2.3284	6.266313
TFPI	3.75E-05	5.23E-07	2.544484	4.638669
SERPINB5	3.90E-05	5.46E-07	-2.56003	8.655911
BHLHE41	4.12E-05	5.82E-07	3.196994	5.35248
FAM160A1	4.12E-05	5.84E-07	-2.24068	6.724923
AHNAK2	4.27E-05	6.14E-07	-3.43179	10.21895
FENDRR	4.27E-05	6.16E-07	3.106814	5.125416
SLC2A1	4.27E-05	6.16E-07	-3.87998	8.573693
ZNF750	4.64E-05	6.75E-07	-3.66072	7.710038

TEP1	4.64E-05	6.76E-07	1.836663	7.190261
TSPAN15	5.00E-05	7.32E-07	3.691913	5.198555
NAMPT	5.13E-05	7.57E-07	-3.19584	9.569327
HLA-H	5.38E-05	7.97E-07	3.387076	4.681898
PGAM2	5.59E-05	8.34E-07	-3.34228	4.574444
CELSR1	5.75E-05	8.62E-07	-2.54686	7.842352
LOC28486!	5.78E-05	8.71E-07	8.155161	1.675325
TMC5	5.91E-05	8.98E-07	4.592711	3.283077
TFAP2C	5.91E-05	9.00E-07	-2.59522	5.631603
VTRNA1-2	6.21E-05	9.53E-07	2.779799	8.098133
PHLDB3	6.21E-05	9.56E-07	-2.22901	6.327054
DSP	6.24E-05	9.65E-07	-3.07173	11.87566
C6orf132	6.30E-05	9.79E-07	-2.61127	9.414192
DHCR24	6.34E-05	9.91E-07	-2.24553	8.874601
EMP1	6.48E-05	1.02E-06	-4.20717	12.15455
CCDC9B	6.48E-05	1.02E-06	-2.21917	7.210768
DUSP5	6.48E-05	1.03E-06	-5.33938	7.665938
FGFBP1	6.48E-05	1.03E-06	-5.36734	6.786961
GLTP	6.75E-05	1.08E-06	-3.4859	9.230806
TRIM29	8.33E-05	1.34E-06	-4.39476	10.25956
LINC01395	8.42E-05	1.36E-06	-8.05932	2.146257
BCL2L15	8.46E-05	1.38E-06	4.157852	4.831249
VNN1	8.46E-05	1.39E-06	5.022945	5.001934
C15orf56	8.46E-05	1.39E-06	-8.28192	2.348965
PGM2	8.74E-05	1.44E-06	-2.50002	7.00229
MIR4279	8.77E-05	1.46E-06	-8.15217	2.229266
SERPINB13	9.17E-05	1.53E-06	-8.34903	2.410753
BCAS1	9.28E-05	1.56E-06	4.189784	3.526143
HNF4G	9.38E-05	1.58E-06	3.953387	5.434239
SDC3	9.51E-05	1.61E-06	2.395957	5.485327
ANO7	9.60E-05	1.63E-06	4.060044	4.513998
SEMA3F	9.68E-05	1.66E-06	-2.7284	7.015281
PDE3B	9.74E-05	1.67E-06	2.502164	5.232679
NOTCH3	9.96E-05	1.72E-06	-2.27079	8.376584
SIPA1L3	9.96E-05	1.73E-06	1.990172	7.264128
FER1L6	0.000102	1.78E-06	4.086678	6.982915
TNFRSF1B	0.000102	1.80E-06	2.369042	5.076987
BTBD11	0.000102	1.80E-06	-4.0999	6.063302
MPP7	0.000102	1.81E-06	-3.41104	7.74513
RBM47	0.000102	1.81E-06	-2.07308	5.912244
MALRD1	0.000109	1.94E-06	6.951952	3.955609
FAM162A	0.000109	1.95E-06	-2.33768	6.617562
LOC10537!	0.000111	2.00E-06	6.501008	2.246067
ST6GALNA(	0.000114	2.05E-06	-2.66309	4.486441
PPP2R2C	0.000123	2.23E-06	-5.52802	2.896962

ERO1A	0.000123	2.24E-06	-3.2006	10.15308
IL20RB	0.000125	2.29E-06	-3.6221	6.892451
NEUROG3	0.000127	2.34E-06	7.910975	1.472746
EMP2	0.000127	2.35E-06	-2.11434	8.290292
IGFL1	0.000132	2.45E-06	-5.1363	5.059518
PAG1	0.000138	2.56E-06	2.376659	6.538572
MKNK2	0.000143	2.68E-06	-2.45615	7.823442
KRT16P3	0.000146	2.75E-06	-5.48103	4.133278
GPC1	0.000153	2.89E-06	-2.30802	6.692303
ANKRD20A	0.000154	2.91E-06	-3.84123	4.937153
FABP2	0.000162	3.09E-06	4.882621	3.342159
GPRIN1	0.00017	3.25E-06	4.13469	4.490066
SEPT5-GP1	0.00017	3.26E-06	-4.67403	3.211928
CRYBG1	0.00017	3.28E-06	-2.32352	9.691823
TMEM263	0.000176	3.41E-06	2.051198	5.305912
TSPAN8	0.000177	3.44E-06	6.007233	7.435656
PKDCC	0.000181	3.53E-06	5.728062	5.313014
EHD3	0.000184	3.62E-06	-3.07359	6.811379
ARHGEF4	0.00019	3.75E-06	-2.73629	4.499745
HMGCS2	0.00019	3.76E-06	6.813314	2.151345
HTR1D	0.000191	3.79E-06	7.993565	1.512288
HES2	0.000192	3.84E-06	-3.16826	5.562741
LRRC66	0.000192	3.85E-06	5.548168	3.637789
LINC00698	0.000196	3.94E-06	8.47261	1.982119
DYNLT3	0.000201	4.06E-06	-2.29385	6.903747
GPR157	0.000223	4.52E-06	-3.937	4.411126
ASPG	0.000223	4.54E-06	-3.40638	4.096024
TRIM16	0.000226	4.61E-06	-2.16508	7.624536
STMP1	0.000227	4.65E-06	-2.30924	6.303122
HOXA11	0.00023	4.74E-06	6.971714	1.677883
DHRS1	0.00023	4.76E-06	-5.81466	2.364959
TENT5B	0.000231	4.78E-06	-3.55265	5.786642
SEC16B	0.000238	4.95E-06	2.982842	5.351397
GALNT7	0.000244	5.10E-06	2.043357	7.476354
TNS4	0.000249	5.24E-06	-4.61814	7.841503
SPTBN1	0.000249	5.25E-06	1.805387	7.49857
ADAM21	0.000261	5.51E-06	-4.97204	2.859434
NECTIN4	0.000262	5.56E-06	-3.15247	8.573203
ARL14	0.000275	5.86E-06	4.09073	3.048497
JCHAIN	0.000288	6.17E-06	3.893535	9.569975
DLEU1	0.000288	6.18E-06	6.046656	2.100976
ALDH3B2	0.000302	6.51E-06	-6.04558	2.574958
EMILIN2	0.000304	6.57E-06	2.35018	4.300014
TMEM63A	0.000329	7.14E-06	1.961194	7.085781
FAM177A1	0.00033	7.19E-06	5.612831	3.214786

ITPK1-AS1	0.00034	7.44E-06	3.715047	3.948975
NRTN	0.000341	7.49E-06	-2.20433	5.603798
ELL2	0.000363	7.99E-06	-2.18972	8.739116
PTN	0.000376	8.31E-06	-4.11196	6.114507
ABCC3	0.00039	8.66E-06	2.827865	7.208847
TINAG	0.000395	8.80E-06	7.646737	1.248
SLPI	0.000395	8.84E-06	-2.98516	8.678594
PPP1R14C	0.000396	8.91E-06	-2.54723	5.150048
ANKRD36B	0.000396	8.92E-06	3.768255	5.593352
CERS4	0.000403	9.11E-06	-3.86015	5.313097
MAB21L3	0.000405	9.20E-06	-4.03878	5.447799
MS4A8	0.000418	9.52E-06	5.510125	3.889577
HOTTIP	0.000422	9.64E-06	5.437033	2.840168
ATP13A4	0.00043	9.86E-06	-3.33493	7.115352
ANOS1	0.000433	9.96E-06	-8.36255	2.423448
COL23A1	0.000437	1.01E-05	3.467105	3.309221
CSTB	0.000443	1.03E-05	-4.24137	12.29503
TYRO3	0.000475	1.11E-05	-2.34313	4.981815
FER1L4	0.000484	1.13E-05	2.684223	6.185846
GJB5	0.000485	1.13E-05	-3.58195	4.56242
RNF217	0.000505	1.19E-05	-4.49511	2.739902
FAM83C	0.000506	1.19E-05	-2.94877	6.087335
C2CD4A	0.000532	1.26E-05	4.630583	2.51533
COTL1	0.000533	1.27E-05	2.464112	6.838601
FSIP2	0.000536	1.28E-05	2.851043	5.334766
PITX1	0.000543	1.30E-05	-3.62598	8.863807
RAB10	0.000544	1.31E-05	-2.36194	9.671388
EPHA1	0.00055	1.33E-05	-2.32522	7.068464
PGLYRP3	0.00055	1.33E-05	-3.99363	4.592519
COL6A1	0.000563	1.36E-05	2.301702	8.974038
EPHB1	0.000567	1.38E-05	4.576263	3.238778
EPS8	0.000579	1.41E-05	1.938695	6.114001
NAT2	0.000579	1.42E-05	6.675593	1.41948
ANXA1	0.00059	1.45E-05	-3.87348	12.75246
MPZL2	0.000593	1.46E-05	-2.89856	8.001923
LRRC19	0.000598	1.48E-05	2.956465	3.18681
ZSWIM5	0.000607	1.51E-05	2.665401	3.99078
CYBB	0.000607	1.51E-05	2.484666	4.836574
GJB2	0.000607	1.52E-05	-4.55218	11.10108
SOX7	0.000616	1.54E-05	-2.79758	6.047406
MAP3K20	0.000616	1.55E-05	1.827587	6.384888
ACE2	0.000635	1.60E-05	2.248074	4.480946
MRC1	0.000667	1.69E-05	2.835685	5.866219
GPX3	0.000677	1.72E-05	-2.6068	8.318993
HEPH	0.000691	1.76E-05	7.771211	1.323121

NPEPPS	0.000693	1.77E-05	-2.29315	8.658257
B3GNT8	0.000693	1.78E-05	-2.2792	4.774058
MIR548I2	0.000693	1.78E-05	5.09585	3.143075
GGH	0.000724	1.87E-05	-4.57636	4.36562
DUSP7	0.000724	1.87E-05	-2.96116	6.616638
FAM157A	0.000727	1.88E-05	-3.37845	4.043313
MIR205	0.000734	1.91E-05	-7.75787	1.874552
ARPC1B	0.000747	1.95E-05	1.887302	5.533645
PCSK5	0.000749	1.96E-05	2.057787	5.353953
CEP128	0.000749	1.97E-05	1.63565	5.614058
COL5A2	0.000771	2.03E-05	2.312151	8.052645
ZNF470	0.00078	2.07E-05	-3.26767	3.704472
SASH1	0.00078	2.07E-05	-2.19807	8.95565
GFOD2	0.00078	2.07E-05	-2.16064	5.969352
EFNA3	0.000792	2.12E-05	-2.83497	4.990126
MYO15B	0.000792	2.12E-05	1.997166	7.354565
PGLYRP4	0.000795	2.13E-05	-4.62789	4.11288
HIF1A-AS2	0.000857	2.30E-05	-5.2408	2.627021
PARD6G	0.000867	2.34E-05	-2.23353	5.392868
RCOR1	0.000867	2.35E-05	-2.15734	7.730618
RNF141	0.000907	2.46E-05	-2.01679	7.540737
KRT16P2	0.000952	2.59E-05	-5.28269	3.675968
CYP4V2	0.000952	2.60E-05	2.337456	5.209201
GPR160	0.000999	2.74E-05	2.380505	4.759168
CLRN3	0.001032	2.83E-05	5.011659	3.448405
SCNN1B	0.001055	2.91E-05	-4.32606	6.223749
SCD	0.001055	2.91E-05	-2.94067	9.52138
GID4	0.001069	2.97E-05	-1.87297	5.397892
GLP1R	0.001069	2.97E-05	5.408705	2.57963
SLURP2	0.00107	2.98E-05	-4.92932	3.717423
KLF8	0.001071	2.99E-05	-5.19496	2.584062
CYP4F35P	0.001125	3.15E-05	-4.71216	3.78614
METTL7B	0.001128	3.17E-05	5.447538	2.294979
SPRR4	0.001138	3.21E-05	-8.15966	2.239372
BNIP3	0.001172	3.31E-05	-2.24161	6.418409
CPNE3	0.001174	3.33E-05	-2.02985	7.956373
SCUBE1	0.00118	3.35E-05	4.251908	4.54182
FEM1B	0.001211	3.45E-05	-1.80567	7.278763
TCAF2	0.001216	3.48E-05	-2.53716	4.386853
PHLDA1	0.001218	3.49E-05	-2.41243	8.448733
BMP5	0.00125	3.60E-05	5.546116	3.285257
CYP2S1	0.001264	3.64E-05	2.010898	6.405698
SOX4	0.001265	3.66E-05	1.844491	7.178018
PLS1	0.001265	3.67E-05	3.407713	2.678969
HOOK1	0.001292	3.76E-05	-1.9303	6.98341

TRAM2	0.001318	3.84E-05	1.910641	5.804914
KREMEN1	0.001331	3.89E-05	-1.99773	6.472499
AGR2	0.00134	3.93E-05	4.723909	8.731913
ITPKC	0.001351	3.97E-05	-2.25685	7.480747
CWH43	0.001385	4.08E-05	-7.55883	1.700357
LY6D	0.001399	4.13E-05	-3.75731	8.425192
SLC1A1	0.001436	4.26E-05	2.022258	5.324065
ONECUT3	0.001447	4.30E-05	5.930257	1.772835
HLA-DRB1	0.001457	4.34E-05	3.421262	5.685973
IL17REL	0.001458	4.36E-05	3.413849	3.148852
MRAP2	0.001469	4.40E-05	3.781356	3.30164
LOC10013(	0.001487	4.47E-05	-1.95308	5.90434
ADGRG7	0.001497	4.51E-05	7.406705	1.054277
C2orf72	0.001516	4.58E-05	4.652224	4.83871
ZFP57	0.001523	4.62E-05	8.118988	1.664834
ZNF395	0.001523	4.62E-05	-1.85798	7.516926
WFDC2	0.001523	4.64E-05	5.07129	2.109407
CASTOR1	0.001532	4.68E-05	-3.74206	4.269359
MUC5AC	0.001548	4.75E-05	6.026065	12.80353
HCN2	0.001548	4.75E-05	-7.62905	1.760661
RHOV	0.001559	4.80E-05	-3.00267	7.003276
LOC10012(	0.001598	4.93E-05	1.746426	5.975014
WDFY4	0.001652	5.12E-05	2.140443	4.639391
TMPRSS11(	0.001652	5.12E-05	-7.8175	1.926888
STON2	0.001691	5.26E-05	-1.94595	6.946773
DDIT4	0.00171	5.33E-05	-2.16234	8.956125
NDUFA4L2	0.001729	5.41E-05	-3.54185	7.110965
SERPINB9	0.001754	5.50E-05	-2.6256	6.411921
REP15	0.001754	5.51E-05	3.989275	3.702737
DSG3	0.001754	5.53E-05	-4.43187	11.36929
LOC10013(	0.001778	5.62E-05	-3.85117	3.385679
NUCB2	0.001781	5.64E-05	-2.04595	8.442134
SOWAHC	0.001841	5.85E-05	-2.19638	7.40856
CLIC3	0.001881	5.99E-05	-4.36891	6.157983
KLK11	0.001889	6.03E-05	-3.97636	3.895052
CAMSAP1	0.001899	6.07E-05	-1.83814	7.491481
PIAS3	0.001909	6.12E-05	-1.78128	6.22052
MAST2	0.001922	6.18E-05	1.538528	6.439933
C3orf80	0.001948	6.28E-05	-4.43876	3.436245
PECAM1	0.002025	6.54E-05	2.035805	7.292556
UGCG	0.002047	6.63E-05	-2.05058	7.496782
GALNT10	0.002047	6.65E-05	1.969594	6.052004
TAB3	0.002049	6.67E-05	-1.96248	7.006125
DST	0.002057	6.73E-05	-2.18997	8.200929
CMAS	0.002057	6.73E-05	-2.18446	6.145027

TFF1	0.002082	6.83E-05	5.531566	8.047609
RNF222	0.002112	6.95E-05	-4.63507	4.99
TRANK1	0.002138	7.05E-05	2.171891	6.278203
IMPA2	0.002173	7.18E-05	-1.68894	7.134662
NEBL	0.002178	7.21E-05	-2.15707	5.935854
RAB3B	0.00222	7.37E-05	4.011703	3.629296
LPAR3	0.002224	7.40E-05	-4.24752	3.932838
CAPN8	0.002226	7.43E-05	4.86691	7.275961
CYB5R1	0.002227	7.45E-05	-1.89213	5.648942
HNF4A-AS1	0.002231	7.48E-05	6.449043	2.565927
BCO1	0.002242	7.54E-05	3.471685	2.998517
CYSRT1	0.002248	7.58E-05	-4.29329	6.229443
MOB3B	0.002248	7.59E-05	1.757568	5.70043
IQGAP2	0.002308	7.81E-05	2.822006	4.507689
PAX4	0.002317	7.86E-05	8.040686	1.593755
MAP3K20-A	0.00239	8.14E-05	2.745426	3.817366
VANGL2	0.00239	8.15E-05	-2.98349	5.387785
LRP11	0.002392	8.18E-05	-1.82891	6.702959
AGMAT	0.002418	8.28E-05	2.686017	3.590597
GABRE	0.002441	8.38E-05	-3.00075	5.85623
SYT15	0.002464	8.48E-05	-6.12944	1.912594
TMEM229A	0.002539	8.78E-05	5.537829	2.207546
AEBP1	0.002539	8.78E-05	2.114873	6.650705
FAM83H-A	0.002541	8.81E-05	-1.96283	5.523362
B3GNT5	0.002573	8.94E-05	-1.97284	7.495516
TIAM1	0.002589	9.02E-05	-3.62882	8.147243
FZD10	0.002617	9.14E-05	-4.67427	5.715103
SHH	0.002675	9.36E-05	5.314851	1.994747
OSTF1	0.002676	9.38E-05	-2.08945	6.622821
SULT1B1	0.002727	9.59E-05	3.333638	5.618871
HK2	0.002744	9.68E-05	-2.13838	8.481372
LINC02432	0.002744	9.69E-05	5.597605	1.786749
SEPHS2	0.002763	9.82E-05	1.833453	6.463305
BMP7	0.002763	9.84E-05	-2.6432	4.879226
NID1	0.002763	9.85E-05	2.496275	7.953442
IFI30	0.002763	9.86E-05	2.002401	5.491628
ARHGAP23	0.002763	9.87E-05	-3.51961	5.344599
SULT1E1	0.002782	9.96E-05	2.82169	4.004581
RILP	0.002787	0.0001	5.463349	2.052162
S100A10	0.002787	0.0001	-1.79231	9.844358
PIK3AP1	0.002787	0.000101	3.276872	3.806547
SLC10A6	0.002787	0.000101	-4.01018	4.959624
FAT2	0.002818	0.000102	-4.06254	9.638566
PEAK1	0.002838	0.000103	1.491954	7.519091
ZSWIM4	0.002838	0.000103	-1.78842	6.037346

SMIM32	0.00288	0.000105	4.805526	3.049589
PDZD2	0.002944	0.000108	-1.7674	6.805892
EMB	0.003021	0.000111	1.705726	5.267984
DOCK5	0.003054	0.000112	1.561358	7.083033
SERPINB1	0.003054	0.000112	-2.94008	4.732009
ARL8B	0.003104	0.000114	-1.81408	7.52034
TM4SF1	0.003107	0.000115	-1.99032	8.16102
STAB1	0.003118	0.000115	2.20607	6.954459
BAG3	0.003118	0.000116	-2.06487	6.742399
ACE	0.003129	0.000116	3.198929	3.242691
VAT1L	0.003219	0.00012	5.537248	2.197776
IL1RN	0.003249	0.000121	-4.373	7.047554
PHGDH	0.00326	0.000122	-2.0883	5.108099
FZD5	0.003278	0.000123	1.726542	6.422293
TMEM184/	0.003295	0.000124	-1.73274	7.965688
SULT2B1	0.003326	0.000125	-4.22091	3.657999
TXNDC5	0.003326	0.000126	2.540335	4.036367
HS3ST3A1	0.003326	0.000126	-3.76283	3.963274
CARD17	0.003363	0.000127	-7.64386	1.772362
ME1	0.00338	0.000128	-2.07715	7.141773
ACER2	0.003403	0.00013	2.488207	4.680722
SIGLEC1	0.00344	0.000131	3.16844	3.895096
ZNF431	0.00344	0.000132	-1.96845	7.218344
LINC01133	0.00346	0.000133	1.854103	5.987287
EVPLL	0.003541	0.000136	-3.59307	3.988104
C10orf99	0.003541	0.000136	-5.07893	6.028817
HOXC-AS2	0.003552	0.000137	5.791596	1.423069
LGALSL	0.003575	0.000138	-1.87445	6.041527
MICALL2	0.003575	0.000138	1.589586	6.019366
NES	0.003609	0.00014	1.888877	5.68195
PRRG4	0.003643	0.000142	-2.10831	7.492282
FRMD4B	0.003713	0.000145	-2.04392	7.356206
LGALS7B	0.003713	0.000145	-4.641	4.756464
OTULINL	0.003713	0.000146	2.153429	5.094819
BTNL9	0.003713	0.000146	2.261387	4.49055
FAR1	0.003771	0.000148	-1.55067	8.352694
ALDOB	0.003781	0.000149	4.672801	5.920173
ANKRD20A	0.003781	0.000149	-4.79982	6.27761
ENO1	0.003797	0.00015	-1.65209	6.826728
CCDC69	0.003855	0.000153	1.488763	5.837857
TM4SF19-/	0.003885	0.000154	-5.02492	2.575589
NDRG1	0.003887	0.000155	-2.99909	6.78038
ATP6V1D	0.003902	0.000155	-1.93293	6.337716
LINC01596	0.003957	0.000158	6.298766	1.128397
CLMN	0.003978	0.000159	1.809137	7.439395

SLC16A2	0.003987	0.00016	2.026249	4.379048
WASF2	0.00405	0.000163	-1.89243	5.728002
GRIN2D	0.004124	0.000166	3.012307	4.211446
BICDL1	0.004145	0.000167	-1.87963	5.993655
PDX1	0.004162	0.000168	4.330661	4.289701
TTC38	0.004193	0.00017	2.328598	5.225322
MBOAT2	0.004194	0.00017	-2.10407	7.96776
CAPNS2	0.004215	0.000171	-3.16522	4.693408
STRN	0.004232	0.000172	-1.78733	8.603112
COL4A1	0.004233	0.000173	1.916692	7.97541
CD164L2	0.004296	0.000176	-4.38528	2.971216
TMOD3	0.004341	0.000178	-1.73224	8.940635
PMVK	0.004371	0.00018	-1.7552	5.369166
FIRRE	0.004375	0.00018	4.282119	3.648897
SPINK5	0.004375	0.00018	-4.19879	4.003397
TENT5A	0.004428	0.000183	1.586728	5.744303
MARVELD3	0.004551	0.000188	2.657907	3.308
MAP2K6	0.004558	0.000189	2.471163	3.916864
GAS2L3	0.004629	0.000192	8.170629	1.716003
PIM2	0.00464	0.000193	2.422435	5.825326
PPL	0.00464	0.000194	-3.65305	12.47334
RIOK3	0.004688	0.000196	-1.63315	8.489639
F13A1	0.004692	0.000196	2.040997	5.516296
HIGD1A	0.004706	0.000198	2.515579	3.769001
COL27A1	0.004706	0.000198	1.683723	7.018607
IRF8	0.004706	0.000198	2.797926	4.599903
CYP4F29P	0.00474	0.0002	-3.75569	4.812087
HRASLS	0.004776	0.000202	-6.01333	1.855202
SLFN13	0.004856	0.000206	1.815793	5.431887
CDKN2B	0.004934	0.000209	-5.29328	2.668288
RAB38	0.004942	0.00021	-3.13623	5.018978
COL15A1	0.00496	0.000211	2.059438	6.641345
TTC9	0.004971	0.000212	-3.60178	7.418931
ITGB3	0.005068	0.000217	2.631618	4.176229
TCN1	0.005068	0.000218	-3.95622	5.448629
SCEL-AS1	0.005068	0.000218	-7.63381	1.766854
FCN1	0.005068	0.000219	4.009803	2.602696
COL4A2	0.005068	0.000219	1.958188	8.214655
GNB2	0.005068	0.000219	-1.58986	6.343063
FAM83A	0.005068	0.000219	-4.06345	6.53941
FAM157B	0.005172	0.000224	-4.75828	3.078487
USH1C	0.005175	0.000225	4.448328	4.096494
PTPRG	0.005218	0.000227	1.661758	6.558801
DAPL1	0.005235	0.000229	-3.70688	4.350279
HOXA10	0.005235	0.000229	8.042885	1.521605

ENDOU	0.005235	0.000229	-7.7656	1.88335
MAN1A1	0.005295	0.000232	1.676667	6.457724
SERTAD2	0.005295	0.000232	-2.17288	7.551061
TEC	0.00533	0.000234	-1.66178	5.808955
C9	0.005364	0.000236	-7.99122	2.086679
PRKAR2B	0.005468	0.000241	2.652932	4.470186
DENND2C	0.005532	0.000245	-4.59111	3.157313
PRDM7	0.005532	0.000245	7.190548	0.879096
BAIAP2	0.005584	0.000248	-2.20333	4.325084
POLR2J4	0.005584	0.000248	-1.86575	6.306305
ZNF770	0.005696	0.000254	-1.71903	7.715508
NEAT1	0.005762	0.000257	-1.98412	14.64983
DCHS1	0.005785	0.000259	1.957571	5.122004
GCOM1	0.005796	0.00026	-3.66467	3.9532
MREG	0.005796	0.00026	-2.4175	6.252055
RHCG	0.005809	0.000261	-4.29948	4.388157
RASA3	0.005843	0.000263	2.563353	5.006519
HLA-DRB3	0.005844	0.000264	6.311389	1.523461
FAT1	0.005847	0.000264	1.467545	9.456573
CD9	0.005847	0.000265	-1.84052	9.012103
MEAK7	0.005851	0.000265	-1.80126	6.691025
RXFP4	0.005867	0.000267	7.234876	0.91279
IL2RG	0.005906	0.000269	2.515872	3.980004
TRAK1	0.005914	0.00027	1.438867	6.268737
CDCP1	0.005929	0.000271	-1.64394	7.170374
CD207	0.005947	0.000272	-7.64833	1.777225
RNF138P1	0.005961	0.000273	1.625445	5.33602
ALPI	0.005981	0.000275	6.240641	1.0614
EPS8L2	0.005988	0.000275	-1.632	8.946786
CCNG2	0.005989	0.000276	-1.79968	7.717803
FAM83G	0.005989	0.000277	-1.51661	6.113814
ARHGAP40	0.006027	0.000279	-3.07555	5.836136
AQP3	0.006027	0.000279	-3.65181	10.24369
HGD	0.006101	0.000283	3.798346	4.63994
TTC22	0.006174	0.000287	-1.74153	5.636241
DRAP1	0.006174	0.000288	-1.71983	6.139554
DMRTA1	0.006174	0.000288	-7.44453	1.598267
JAG1	0.006174	0.000289	-1.61076	8.287373
S100A7A	0.006175	0.000289	-8.16297	2.24156
OR13H1	0.006175	0.00029	7.534917	1.128065
MRPS10	0.006178	0.00029	-1.5994	5.879046
AHNAK	0.006184	0.000291	-1.58613	13.60968
GLP2R	0.006197	0.000292	6.082044	2.365734
RANBP9	0.006318	0.000298	-2.05383	8.085244
NAGK	0.006359	0.000301	-1.60173	6.930793

MIR600HG	0.006359	0.000301	-1.61732	5.622336
PRKCH	0.006408	0.000304	-2.05511	6.201298
GVINP1	0.006409	0.000305	2.346591	4.806607
HHEX	0.00657	0.000313	4.195398	2.096697
C5orf66	0.006615	0.000316	-2.51064	7.23229
FBLN5	0.006679	0.000319	2.037575	4.848773
EEF1A2	0.006795	0.000325	-8.42573	2.48092
SLC4A4	0.006821	0.000327	5.845013	1.712157
DNAJC5	0.006866	0.00033	-1.48067	7.657909
CARD14	0.007075	0.00034	-7.22972	1.417494
C1R	0.00714	0.000344	1.91577	6.995178
TGFBI	0.00714	0.000345	1.947818	5.404915
FABP5	0.007152	0.000346	-3.75328	10.14889
HERC2P4	0.007176	0.000348	-4.13875	4.704477
CBR3	0.007231	0.000351	-2.73137	4.375781
FNDC4	0.007235	0.000352	-2.34969	4.125957
POM121L9	0.007241	0.000352	4.780161	2.290279
ZBED2	0.007392	0.00036	-4.06103	4.442325
HSPB8	0.007395	0.000361	-3.73042	6.108238
EXOC6B	0.007508	0.000367	-1.59497	7.33497
MMP15	0.007531	0.000369	2.082866	5.448534
GRPEL2	0.007629	0.000374	-3.16107	7.323848
SDC2	0.007668	0.000377	2.3423	4.926085
RGS12	0.007799	0.000384	-1.73581	4.852203
HHIP	0.007819	0.000386	4.404843	4.762499
EIF4EBP1	0.007887	0.00039	-1.52837	5.221441
ETV5	0.007899	0.000391	2.19546	4.339784
ABCC6P1	0.007965	0.000395	4.109718	2.644104
LBX2-AS1	0.007974	0.000396	3.574679	2.778672
ZNF169	0.00798	0.000397	5.371471	1.574605
MTCL1	0.00807	0.000402	1.752066	5.687496
RCN3	0.008078	0.000403	2.090908	4.08505
LINC01819	0.008137	0.000407	4.573455	3.184545
MT1A	0.008139	0.000407	7.505734	1.127177
PLXND1	0.008266	0.000414	1.70695	6.079633
DSG2	0.008275	0.000416	1.355315	7.355211
CST6	0.008275	0.000416	-4.47055	2.19128
C1QA	0.00829	0.000418	2.143566	4.569263
PEX3	0.008353	0.000422	-1.60393	5.246133
FSCN1	0.008353	0.000422	-1.90842	6.330526
LOC643201	0.008379	0.000425	4.594065	2.846583
CYP2C19	0.008379	0.000425	1.710137	5.466634
TENM4	0.008379	0.000426	-3.04362	4.90548
MICAL3	0.00838	0.000427	1.316538	6.506618
DQX1	0.00838	0.000427	1.891764	4.377381

RALA	0.008451	0.000431	-1.58014	7.744713
EEA1	0.008485	0.000434	-1.60868	8.480074
CFI	0.008673	0.000444	2.309374	5.115505
SLC25A21	0.00872	0.000447	-2.95743	4.7497
FAM83A-A	0.008722	0.000448	-4.07893	5.295376
GCNT1	0.008812	0.000453	3.850666	2.605503
PYGB	0.008812	0.000454	1.555059	8.263562
GSTA4	0.008812	0.000455	-2.45493	5.63849
USP6NL	0.008835	0.000457	-4.03499	4.304463
VIM	0.008835	0.000457	1.836152	7.618384
LOC44043	0.008892	0.000461	-2.03514	6.867957
RCN1	0.008981	0.000466	1.356881	6.321133
CERS3-AS1	0.009019	0.000469	-4.39312	3.66421
RTKN2	0.009056	0.000472	-2.56003	5.380766
ELOVL4	0.00917	0.000479	-3.54153	5.201471
PSMC3IP	0.00917	0.000479	4.076681	1.824742
UBE2Q2P2	0.00923	0.000483	-1.61857	6.162264
CHMP1B2F	0.009244	0.000484	3.142856	2.580807
SH3PXD2A	0.009285	0.000488	-2.52235	5.107009
S100A3	0.009285	0.000488	-4.83282	1.924214
HEBP2	0.009285	0.000489	-1.59327	7.119337
ANGPTL2	0.009291	0.00049	1.778035	5.383996
RAET1L	0.009328	0.000493	-4.15526	4.205849
ATP4A	0.009328	0.000494	-14.674	8.619738
PRSS27	0.009328	0.000494	-4.22813	6.684181
GPR78	0.009328	0.000495	-6.87265	1.120024
SNX24	0.009358	0.000497	-1.87026	5.670788
RFLNB	0.009372	0.000499	2.029638	4.564869
RETREG1	0.009428	0.000502	3.869766	2.355918
CA8	0.009462	0.000505	3.877588	3.659372
RCC2	0.009652	0.000516	-7.09505	1.301341
ALDH9A1	0.009672	0.000518	-1.65889	7.633815
HSD11B2	0.009672	0.000519	1.911475	4.495531
ULK1	0.009679	0.000521	-1.49974	6.26463
CLVS1	0.009679	0.000521	-7.51495	1.666204
ARL4D	0.009726	0.000524	-3.72574	5.429661
STN1	0.009738	0.000525	-1.40726	5.977408
SDR16C5	0.009881	0.000534	-2.33053	5.725577
SPAG17	0.009914	0.000536	-3.47857	5.521132
GSTP1	0.010014	0.000543	-1.52028	8.532779
PELI1	0.010015	0.000543	-1.66859	7.83702
SLC7A1	0.010018	0.000545	-1.55952	8.197202
SRXN1	0.010018	0.000545	-1.68796	5.577864
SESTD1	0.01006	0.000548	1.583018	5.794903
FAM118B	0.010089	0.000551	1.634191	4.672693

SVEP1	0.010163	0.000556	1.947244	5.302098
SPTAN1	0.010222	0.00056	-7.10639	1.311599
DACH1	0.01023	0.000561	6.116922	0.962469
LOC10013(	0.010279	0.000565	2.407621	4.286529
ZNF618	0.01033	0.000569	1.730289	5.405376
UBE2V2	0.01033	0.000569	-1.64394	5.711193
LOC10192(	0.010338	0.00057	-7.88477	1.987388
TRPA1	0.010358	0.000572	3.072625	5.695246
B4GAT1	0.010444	0.000578	2.188122	4.421992
FAM95B1	0.010448	0.000579	-7.10679	1.311582
SMAP2	0.010478	0.000581	1.875914	5.157981
BTNL8	0.010508	0.000584	7.780017	1.378882
YWHAQ	0.01058	0.000589	-1.54954	7.761378
PLAC8	0.010688	0.000596	3.557857	2.487076
C12orf29	0.010688	0.000596	-2.95501	4.57895
PROSER2-A	0.010851	0.000607	-4.1004	3.590997
GMPR2	0.010851	0.000607	-7.37677	1.541237
MAP1B	0.010855	0.000608	2.001878	7.551899
ARF6	0.010917	0.000613	-1.49272	8.96527
VPS4B	0.010925	0.000614	-1.50449	8.143311
TET3	0.010931	0.000616	-1.39296	7.696466
CDR2	0.010931	0.000616	1.759709	4.588794
ACY3	0.010946	0.000618	5.063463	2.559568
CNDP1	0.010973	0.000621	4.932312	2.168572
THBD	0.010973	0.000621	-1.82021	5.971773
GRK5	0.011055	0.000627	1.615655	5.636324
LINC00330	0.011055	0.000629	-5.05196	1.680412
MAST4-AS1	0.011055	0.00063	-5.28255	1.881995
TRABD2A	0.011055	0.000631	4.876241	1.848817
HES1	0.011055	0.000631	-1.54621	7.320564
NUAK1	0.011055	0.000631	1.879095	4.389001
ANXA9	0.011094	0.000635	-1.87045	5.096734
FBN1	0.011094	0.000635	1.78336	7.440773
SLURP1	0.011123	0.000638	-4.97944	6.410798
ABHD5	0.011197	0.000644	-1.63981	6.136162
DNAJB1	0.011197	0.000644	-2.13822	7.037249
ANO1	0.011212	0.000646	2.451633	4.523274
ARAP2	0.011241	0.000649	-1.63381	7.452872
LINC00964	0.011241	0.000649	-5.1634	2.215666
ANKRD20A	0.011241	0.00065	-3.43045	5.618852
SH3GLB1	0.011293	0.000655	-7.56375	1.704014
AXIN2	0.011293	0.000655	1.965987	4.738497
LSM12	0.011362	0.000661	-2.81552	4.710448
TXNDC17	0.011362	0.000661	-1.716	5.339565
TMBIM1	0.011379	0.000663	-1.43853	8.335541

TCIM	0.011466	0.000669	2.55538	5.405075
LAMB1	0.011489	0.000671	1.782743	8.235087
PPP1R3C	0.011495	0.000672	-4.73521	7.851539
S100A2	0.011532	0.000676	-4.01293	9.033733
PCP4L1	0.011532	0.000676	-3.28893	3.823287
JUNB	0.011542	0.000678	-1.82069	7.616985
TOMM20L	0.01156	0.00068	7.29901	0.962636
CPA3	0.011581	0.000683	2.228857	4.810134
LOC101927	0.011581	0.000683	3.573457	2.881371
ADCY4	0.011652	0.000688	7.289168	0.951184
PRPSAP1	0.011729	0.000694	-1.32871	5.959404
CACNA2D3	0.011748	0.000696	-3.29839	3.779848
MEI1	0.011765	0.000698	3.538987	3.331457
PLCE1-AS2	0.011765	0.000699	3.371592	2.979068
LINC00887	0.01179	0.000701	-3.88423	4.531103
ALDH3A2	0.011797	0.000702	-7.513	1.657964
CLDN1	0.011825	0.000705	-1.69058	6.376618
AFDN	0.011894	0.00071	-1.54747	5.495294
LOC388282	0.011914	0.000712	-7.09781	1.303661
FOXRED1	0.01192	0.000713	-6.82287	1.077542
KRT32	0.011933	0.000715	-7.57167	1.709048
PGA5	0.011933	0.000716	-15.8162	9.761158
PROM2	0.011941	0.000718	-1.94726	5.368874
ZBTB5	0.012	0.000722	-1.44208	5.807952
ZNF512B	0.012003	0.000723	-1.3932	5.731146
TSPAN11	0.012097	0.00073	2.743664	4.034801
CPED1	0.012198	0.000737	2.244601	3.731403
SSC5D	0.012201	0.000738	2.454998	4.154066
RTL8C	0.012238	0.000742	-1.51444	6.182488
RELCH	0.012238	0.000743	-1.51451	8.000074
KRT1	0.012238	0.000743	-6.76745	8.448341
CRIP1	0.012257	0.000746	1.746503	4.645834
GNG11	0.012284	0.000748	2.213645	3.581178
GRN	0.012335	0.000752	-1.4722	8.351598
SLC37A1	0.012394	0.000758	1.434692	6.46193
LPL	0.012394	0.000758	3.051859	2.757995
KCNF1	0.012425	0.000761	7.822715	1.411179
CCND2-AS1	0.012435	0.000762	6.637479	1.997675
COL3A1	0.01248	0.000766	2.006346	10.55123
DHRS3	0.012578	0.000773	-1.44536	6.850947
RNF225	0.012631	0.000778	-4.05833	2.901416
TMCO3	0.012814	0.00079	1.453959	6.546868
FSTL1	0.012827	0.000792	1.707001	6.771207
ABI3BP	0.012861	0.000795	1.572555	5.441848
GAN	0.012881	0.000797	-1.51346	6.827217

ASPHD2	0.012969	0.000804	2.150558	3.932664
ANKRD22	0.012969	0.000805	-1.44605	6.072217
GNAI3	0.012986	0.000807	-1.62546	7.823081
SPON1	0.01305	0.000814	1.801767	6.235189
SEMA4G	0.01305	0.000814	3.014857	2.758483
PCOLCE	0.01305	0.000814	2.669475	3.547994
AKR1C3	0.013134	0.00082	5.184521	1.89653
NMRK1	0.01326	0.000829	-5.60167	1.496458
LOC154761	0.013439	0.000841	-1.56369	5.097721
TRIOBP	0.013476	0.000845	-2.29988	5.952703
ZFP36	0.013499	0.000847	-1.94198	9.037704
BMP2	0.013671	0.00086	1.531619	5.022783
LYPD3	0.013671	0.00086	-4.89883	9.15492
KLHL6	0.013781	0.000868	2.830204	5.000038
PLEKHM1P	0.01384	0.000873	-1.49413	6.299073
CYP3A5	0.013921	0.00088	1.522546	6.768882
TMPRSS11	0.013955	0.000883	-4.44823	6.383356
CAPN5	0.014006	0.000887	1.321622	6.984872
FKBP10	0.014006	0.000888	2.418002	4.250138
TPRG1	0.014031	0.000891	-4.17172	6.690791
ATP11B	0.014046	0.000893	-1.44217	8.793565
FAM110C	0.014255	0.000908	-1.87426	6.971669
VWF	0.014355	0.000915	1.504362	8.21807
VAT1	0.014355	0.000916	-1.48414	6.834233
CYP2W1	0.014543	0.000931	3.265585	2.780859
AQP5	0.014543	0.000932	3.177903	3.286378
CCDC162P	0.014543	0.000932	3.71267	3.354252
LOC34009(	0.014543	0.000933	4.03107	1.715694
CARD10	0.014552	0.000935	1.402243	6.022492
IPPK	0.014659	0.000943	-1.8152	6.439224
COL1A2	0.01476	0.00095	1.928794	9.855943
KRT13	0.014802	0.000954	-4.59056	6.745002
ZNF703	0.014881	0.000961	1.66171	5.126189
RASSF4	0.014896	0.000964	3.037426	4.411008
HOXC8	0.014896	0.000965	3.905345	2.20664
SLC9A3R1	0.014896	0.000965	-1.36625	8.335603
PAIP2B	0.014932	0.000969	-2.61533	6.015567
SPRR2F	0.015034	0.000977	-5.50098	4.129457
BCL11A	0.015082	0.000981	-3.14378	4.690319
SLC27A4	0.015112	0.000984	-1.5034	5.655052
APLF	0.015134	0.000987	-2.02249	4.568706
LTB4R	0.015163	0.00099	-2.76119	6.620712
CLIP1-AS1	0.015163	0.000991	-3.13325	3.946928
NPR2	0.015175	0.000993	3.010523	3.541145
CLDN23	0.015371	0.001007	2.402726	4.109264

MDK	0.015371	0.001008	7.377277	1.030846
ADH7	0.015483	0.001017	-3.93151	3.072933
SOWAHB	0.015483	0.001018	-3.3225	3.736606
SRD5A1	0.015491	0.00102	-1.67525	4.903367
C3orf70	0.015502	0.001022	3.012333	3.42265
NGEF	0.015507	0.001024	3.437616	2.885165
ANO8	0.015712	0.001038	-1.61024	5.318234
TMEM187	0.015938	0.001055	-3.53815	2.601032
LOC102724	0.016035	0.001062	4.289742	2.570151
GNA14	0.016155	0.001072	2.318626	2.845232
OLFM4	0.016155	0.001073	5.73926	9.058132
HAPLN1	0.016262	0.001081	2.784353	3.821523
IL13RA1	0.016352	0.001089	-1.40126	7.436639
MMP12	0.016412	0.001094	4.448079	2.642886
LOC101927	0.016467	0.001099	4.86102	1.427768
KIFC3	0.016543	0.001107	-5.03606	2.021118
PTPRH	0.016543	0.001108	4.755918	2.672168
C6orf222	0.016543	0.001108	4.550042	1.942861
FBN2	0.016572	0.001111	2.544628	4.081667
MZB1	0.016573	0.001113	3.324775	3.86243
ULK4	0.016722	0.001125	1.580195	5.107655
CALM2	0.016722	0.001126	1.840673	4.945981
GVQW3	0.016821	0.001134	2.736617	2.990497
KCTD12	0.016827	0.001135	1.439882	6.586287
MAST3	0.016874	0.00114	1.579078	4.90525
RMND5A	0.01692	0.001144	-1.40373	7.717531
WDR91	0.016941	0.001147	-1.42457	5.933114
PLOD3	0.017002	0.001153	2.352029	4.648192
TMTC3	0.017002	0.001154	-1.58317	6.659287
CDH5	0.017043	0.001158	1.653873	5.010834
MXRA5	0.017116	0.001165	1.602969	8.040355
LAD1	0.017267	0.001177	-1.55842	8.891261
SFXN3	0.017267	0.001178	1.722428	4.448765
SQLE	0.017271	0.001179	-1.71804	7.2118
TPSAB1	0.017455	0.001193	2.292386	4.275594
ITGA5	0.017526	0.0012	1.687114	6.249318
TRPM2-AS	0.017536	0.001202	5.269717	1.234173
JARID2	0.017537	0.001206	-1.68792	5.055726
TSPAN13	0.017537	0.001206	1.313264	5.657904
GAL3ST2	0.017537	0.001206	6.578298	1.354154
RNF186	0.017591	0.001211	7.176549	0.848044
UBE2G1	0.017623	0.001215	-1.63461	6.784239
RNF223	0.017636	0.001217	-2.99833	3.382873
CEP290	0.017702	0.001223	1.507711	5.637816
CDHR1	0.01771	0.001225	-2.65026	4.824805

PRR15L	0.017733	0.001228	2.243687	5.863884
SUSD4	0.017764	0.001232	-3.84287	4.977944
B4GALNT4	0.017799	0.001236	-3.01583	4.554367
SLC6A4	0.017799	0.001237	-3.28935	2.693967
PRKCA	0.017816	0.00124	2.452518	6.309159
GPR87	0.017816	0.001241	-4.02091	5.346201
PMM1	0.017913	0.001249	-1.67005	5.808388
MGAM2	0.017979	0.001255	4.589323	2.686965
FOXN3	0.018009	0.00126	2.370005	2.805707
PBLD	0.018009	0.00126	2.763204	4.814911
UBL3	0.018219	0.001276	-1.34102	7.209344
MIR1296	0.018425	0.001292	-6.96415	1.192021
ABCG5	0.018642	0.001309	6.016484	0.91314
KLC3	0.018656	0.001312	-3.90708	4.902813
CD24	0.018656	0.001313	-2.82718	4.167947
SOD3	0.018657	0.001315	2.393509	4.248185
NBPF25P	0.018806	0.001327	-1.56453	5.408438
KAZALD1	0.018901	0.001335	3.194156	2.951514
DRAM1	0.018941	0.001339	1.954686	4.038588
AKR1C4	0.018997	0.001346	6.879536	0.629462
C22orf46	0.018997	0.001346	1.232226	5.854991
RPGR	0.019022	0.00135	1.287927	5.655769
LGI3	0.019077	0.001355	-3.80615	2.674434
HACD3	0.019183	0.001366	1.338998	5.845164
SPNS2	0.019183	0.001366	-1.64631	6.784781
LAMC1	0.019235	0.001371	1.453474	7.963107
PLEKHA6	0.019321	0.00138	1.371229	7.168215
PLP2	0.019321	0.001382	-1.62517	6.704149
ATP1B3	0.019321	0.001382	-1.57341	7.026035
FAM30A	0.019392	0.001389	3.22513	4.473341
FAM25A	0.019412	0.001391	-4.49607	6.578564
SPPL3	0.01948	0.001398	-1.44445	6.478917
SOX21	0.019539	0.001404	-2.84549	4.202341
OGFRL1	0.019573	0.001408	-1.68762	6.395441
STOX2	0.019592	0.001411	-2.42193	4.661867
PHF13	0.019613	0.001414	-1.78094	4.996476
EFNA2	0.019667	0.001419	4.325977	1.923898
ANKRD37	0.01971	0.001424	-3.3609	3.994296
DLEU7	0.019765	0.00143	3.412041	2.569991
SOX21-AS1	0.019765	0.001431	-3.18597	3.558905
CASP1P2	0.019849	0.001439	-4.16281	3.446029
PKP1	0.019909	0.001445	-3.75846	4.082
PSMC6	0.019954	0.00145	-1.41873	6.537216
NNT-AS1	0.019954	0.001451	2.084801	3.972929
RPS27L	0.019967	0.001454	1.481411	5.28811

CNFN	0.02023	0.001475	-4.55813	9.149036
HECTD1	0.02025	0.001478	-1.39729	9.720358
PGK1	0.020373	0.00149	-1.43375	8.128497
EMP3	0.020373	0.00149	3.366333	2.232657
RBX1	0.020518	0.001502	-2.46574	4.48484
MPEG1	0.020521	0.001504	1.818527	4.865584
MANBA	0.020619	0.001513	1.296129	5.331025
STARD9	0.020703	0.001524	1.619905	5.936998
ADAM17	0.020703	0.001524	-1.69314	6.533187
YBX2	0.020703	0.001524	-4.89406	1.544818
FNDC10	0.020744	0.001529	-3.58648	2.156754
CD93	0.020826	0.001537	1.618547	6.035925
MAP1A	0.020841	0.00154	1.994456	5.128132
DOCK3	0.02127	0.001573	-1.66477	4.919098
5-Mar	0.021445	0.001588	-1.69257	5.794374
LOC101928	0.021473	0.001592	-5.14004	2.785317
FOXD1	0.021473	0.001594	2.358558	3.382178
GPT	0.021473	0.001597	1.979645	4.302884
VLDLR	0.021473	0.001598	-7.12805	1.328024
COL1A1	0.021473	0.001598	1.906084	10.20288
PLEKHG3	0.021589	0.001609	-1.33606	6.914309
KCTD9	0.02204	0.001644	-1.49584	7.121518
LINC00032	0.022106	0.001651	3.496138	2.556535
ATP6V1G1	0.022234	0.001662	-1.50048	6.307789
MTF1	0.022305	0.001669	-1.60133	6.595629
DUSP8	0.022318	0.001672	4.440928	2.29973
BNC1	0.022365	0.001677	-6.62181	0.91817
OAF	0.022375	0.00168	1.432366	5.184878
RAB21	0.022375	0.001682	-1.31421	7.702489
P3H4	0.022396	0.001685	2.169116	2.979145
HOXC-AS1	0.022417	0.001689	6.082576	1.483859
SLC6A11	0.022481	0.001695	-2.99647	4.293469
RETNLB	0.022647	0.00171	7.380843	1.03677
ASTN2-AS1	0.022707	0.001717	3.590638	2.372255
KATNBL1	0.022707	0.001718	-1.55801	5.655028
C3orf67	0.022803	0.001727	-2.69432	5.215866
EIF1	0.022833	0.001731	-1.32279	7.672659
SLC25A20	0.023136	0.001757	1.676313	4.090432
PXDN	0.023136	0.001758	1.817363	6.230698
LINC01269	0.023137	0.00176	-5.84828	1.707089
GLCCI1	0.023242	0.001772	1.754951	4.889617
14-Sep	0.023242	0.001773	7.290236	0.978727
HS1BP3	0.023242	0.001773	1.538164	4.396847
ANKRD36B	0.02337	0.001785	-1.7734	5.295028
IGDCC4	0.023462	0.001794	2.4905	3.621405

SLC12A7	0.023546	0.001802	2.236047	4.936091
ERV3-1	0.02363	0.001811	-1.4381	5.982812
RNASET2	0.023632	0.001813	1.463714	5.436209
BMP6	0.023675	0.001821	2.748941	2.902143
LRIG2	0.023675	0.001823	3.748352	2.633385
SNORD101	0.023675	0.001823	-4.1073	1.995085
VASP	0.023675	0.001824	1.206613	6.669682
SYNGR1	0.023675	0.001827	-2.73662	4.852872
SLC10A5	0.023675	0.001828	2.467005	3.31094
PSMD14	0.023701	0.001831	-1.28341	5.701086
ROCK2	0.023731	0.001836	1.165584	7.746077
HCG4	0.023737	0.001838	-7.06318	1.274872
TENT5C	0.023748	0.001841	2.461703	6.872686
MSMO1	0.023826	0.001849	-3.54281	3.08348
IGFBP5	0.023842	0.001852	1.93668	9.967748
GPRIN3	0.023875	0.001857	1.710864	5.275107
LHFPL2	0.023912	0.001861	1.474787	5.517106
B3GNT7	0.02397	0.00187	2.564169	4.871718
CARD11	0.02397	0.001871	2.479834	4.240955
CCL28	0.02397	0.001872	3.437044	1.786825
ZNF275	0.02397	0.001874	2.002791	4.246384
TLE4	0.02397	0.001875	-6.45269	0.788495
CYGB	0.024083	0.001886	2.706032	3.595006
CAST	0.024144	0.001893	-1.99798	4.454407
HACD2	0.024212	0.001902	-1.68065	5.012511
SLC22A10	0.024212	0.001903	8.043052	1.602041
SLCO4A1	0.024212	0.001905	-2.26561	5.180594
RASGRF2	0.024212	0.001906	2.037353	4.35177
PLEKHH1	0.02422	0.001909	1.336598	6.145377
DNM1P41	0.02428	0.001915	-3.34792	2.484682
MIOS	0.024354	0.001923	-1.37894	6.191424
SP100	0.024364	0.001926	1.491659	5.251498
RIPOR2	0.024364	0.001929	4.217518	1.77848
TCAF2P1	0.024364	0.00193	-3.15117	2.729252
SPATA6L	0.024506	0.001943	2.203695	4.104989
LPIN2	0.024581	0.001951	1.326278	7.076285
TINCR	0.024604	0.001955	-3.1391	4.441956
HERC2P3	0.024611	0.001958	1.469679	4.646396
REXO2	0.024622	0.001961	-1.32241	6.095019
YPEL5	0.024622	0.001962	-7.2345	1.418847
ECM1	0.024731	0.001974	-6.54981	0.860794
LINC01356	0.024731	0.001975	5.425667	0.952784
ATP6V1E2	0.024825	0.001985	3.987509	1.116485
ARRDC3	0.024877	0.001992	-1.34077	8.06592
SHROOM2	0.024877	0.001993	-2.4224	4.969822

FAM83B	0.024915	0.001998	-1.62022	7.749377
UNC5C	0.024922	0.002	2.764565	4.022815
TIMP3	0.02495	0.002005	1.577107	7.52304
IRX3	0.025164	0.002024	-3.14612	5.208032
OBSCN-AS1	0.025165	0.002026	-6.70318	0.978987
PNLIPRP3	0.025165	0.002028	-5.35424	1.941062
TCP11L2	0.025202	0.002033	-1.78803	5.85349
TYRP1	0.025362	0.002048	4.130297	2.334442
FBXL18	0.025375	0.002051	-1.47141	4.83209
SULT1C3	0.025397	0.002055	5.514404	2.550869
EPB41L4B	0.025506	0.002066	2.008718	4.603232
SPTB	0.025506	0.002068	-1.52392	4.583951
CTGF	0.025559	0.002074	2.297608	6.97203
MAGI1	0.0256	0.00208	-6.52419	0.838841
GPC4	0.025619	0.002083	2.06798	4.447133
RPE65	0.025738	0.002095	-5.25852	3.118701
PLEKHO1	0.025747	0.002098	3.97854	1.994995
VPS25	0.025766	0.002102	-1.41411	5.03342
EPN2-AS1	0.025766	0.002104	-2.20731	4.339971
H1F0	0.025779	0.002108	-1.42197	7.89822
OVOL1	0.025779	0.002109	-2.2495	6.293065
MIR627	0.025982	0.002128	-6.46048	0.789854
COL10A1	0.0261	0.002139	1.964352	3.429386
GID8	0.026115	0.002143	-1.25413	7.576522
OVOL1-AS1	0.026146	0.002148	-3.88617	3.367081
MIA2	0.026146	0.00215	2.063636	3.966143
CFH	0.026239	0.002159	1.796354	6.498566
PLUT	0.026287	0.002165	5.956864	0.845959
SLC38A10	0.026295	0.002168	1.283062	5.373487
EFCC1	0.026347	0.002175	3.366499	2.957467
EIF5AL1	0.026362	0.002178	-1.69776	4.609086
HSH2D	0.026531	0.002194	5.753202	1.38762
LOC101927	0.026605	0.002202	3.307698	2.541224
ANKMY2	0.026651	0.002208	1.446254	4.898717
ZG16B	0.026719	0.002216	3.927367	2.675281
OSBPL7	0.026749	0.002221	2.762589	5.171178
PADI1	0.026911	0.002236	-4.99572	9.167289
FAM238C	0.027105	0.002255	-3.97492	2.311258
GPX4	0.027327	0.002275	4.391898	1.027497
PECR	0.027341	0.002279	2.408467	3.368279
MIR648	0.027482	0.002293	3.222002	1.672547
LRRC37A4P	0.027506	0.002297	-1.34634	6.947329
JCAD	0.027611	0.00231	1.400889	5.355005
ROMO1	0.027611	0.00231	-1.66015	4.36234
HBEGF	0.027624	0.002314	-2.69079	6.089513

RAP2B	0.027934	0.002342	-1.48933	7.557587
POU2AF1	0.028014	0.002351	2.234176	5.287101
LCP2	0.028014	0.002353	1.698955	4.633964
JAK3	0.028172	0.002369	1.798417	4.02474
KLF2	0.028201	0.002373	1.924942	4.509148
ZNF785	0.028223	0.002377	1.436213	4.454081
FRRS1	0.028563	0.002408	-1.61733	5.212303
RNF128	0.028563	0.002411	2.738727	3.353616
SCPEP1	0.028565	0.002415	-1.51832	5.786049
DIRAS3	0.028565	0.002416	-4.64173	2.538316
CIPC	0.028576	0.002419	-1.46894	5.72972
ARFGEF3	0.028599	0.002423	2.056737	6.932178
TET1	0.028646	0.002429	1.895242	4.235952
TALDO1	0.028646	0.002432	-1.27008	7.461558
DESI1	0.02865	0.002434	-1.32904	6.542966
ANKRD18B	0.029001	0.002466	-3.62515	4.684233
ANAPC7	0.02913	0.00248	-1.69205	4.528439
CNNM4	0.029138	0.002483	1.145192	5.79354
LOC10192[illegible]	0.02934	0.002502	-3.37188	2.499934
GOLGA6L1[illegible]	0.029342	0.002507	-1.66705	5.134609
WDFY2	0.029342	0.002509	-1.90983	6.271929
TRPM4	0.029342	0.00251	3.534976	2.763743
KAT2B	0.029352	0.002513	-1.47609	6.870368
KIAA1614	0.029374	0.002523	2.226064	2.895606
C1QB	0.029374	0.002524	2.016416	5.136429
VDAC2	0.029374	0.002525	-2.39346	3.884614
LOC10192[illegible]	0.029374	0.002528	-3.88904	2.677745
TCF21	0.029374	0.00253	2.719225	3.840965
PREX1	0.029374	0.002532	1.592888	5.956168
NNMT	0.029374	0.002533	2.510748	4.521619
YOD1	0.029374	0.002535	-7.07529	1.284591
LINC00482	0.029374	0.002536	-3.92571	2.272071
MGAT5	0.029429	0.002543	1.168073	7.100977
BICDL2	0.029589	0.00256	-1.42015	7.114339
DNAH6	0.029591	0.002562	2.852047	3.173854
TOX3	0.029606	0.002566	4.890459	2.594327
PGM2L1	0.02971	0.002577	1.961435	4.695695
C1QL1	0.029711	0.00258	5.776491	0.749387
NPAS2	0.029807	0.002591	-1.29797	7.08764
ZDHHC21	0.029811	0.002594	-1.56358	7.495905
CYP2E1	0.029811	0.002596	-2.4054	4.076161
GSDMC	0.029921	0.002608	-4.15174	6.678573
TICAM1	0.029921	0.002614	-1.57907	5.191763
ALDH1B1	0.029921	0.002614	1.963947	4.019015
FBXO45	0.029921	0.002615	-1.8439	5.597325

METTL1	0.029952	0.002622	7.073554	0.798643
LINC00551	0.029952	0.002623	-3.59749	3.63898
EFNA5	0.029952	0.002625	-2.27982	5.697008
LYVE1	0.030189	0.002648	2.509382	2.97561
GPSM2	0.030189	0.002651	-1.41164	6.272833
FAM219B	0.03037	0.002669	-1.41151	6.798417
GIMAP4	0.030556	0.002689	1.707914	4.694704
TTLL12	0.030556	0.002692	-1.28419	7.33197
SYNPR-AS1	0.030556	0.002693	7.050941	0.781098
KRT2	0.030556	0.002695	-5.46183	2.477837
MGC12916	0.030556	0.002698	-4.17343	1.736798
TUBB4B	0.030656	0.002711	-1.23258	7.355732
SYNE3	0.030656	0.002712	2.340028	4.263143
EVADR	0.030656	0.002714	4.40477	1.612007
ANGPTL4	0.030669	0.002719	-6.8494	1.096761
HLF	0.030669	0.00272	-1.92966	5.238657
PPDPF	0.030828	0.002737	-1.34065	8.345586
ATG13	0.031042	0.002758	-6.3737	0.724458
TBX2	0.031227	0.002778	2.45791	4.585563
PLXNA4	0.031227	0.00278	3.090912	3.533987
LGALS3BP	0.031384	0.002796	1.198059	7.67412
MET	0.031391	0.002799	7.86119	1.444946
OSER1	0.031621	0.002822	-1.58618	5.714673
KRT15	0.031663	0.002829	-3.68147	9.85682
MLXIP	0.031667	0.002832	1.222433	7.42234
RNF125	0.031667	0.002834	1.672312	3.909747
ZFHX2	0.031903	0.002858	1.721279	4.147017
RGS20	0.031949	0.002865	-7.28881	1.463511
MCAM	0.031959	0.002868	1.596034	5.770042
SYCN	0.032184	0.002891	-4.11631	2.088114
GOLGA6L5	0.032216	0.002896	-1.6397	4.403441
SOX15	0.032377	0.002914	-3.14342	4.622253
KMT5A	0.032557	0.002934	-1.17044	6.63865
LFNG	0.032557	0.002936	4.610912	1.393538
NEGR1	0.032557	0.002938	2.215815	4.388039
KIF13A	0.032591	0.002943	1.485079	4.822149
CLMP	0.032669	0.002953	1.582033	5.268978
SAP30L-AS	0.03315	0.002999	2.358294	3.559649
SLC9A3-AS	0.033309	0.003016	-1.70914	5.454648
AGAP11	0.033483	0.003035	-3.20957	3.795729
MT1H	0.033684	0.003056	3.489624	3.257231
TMEM159	0.033719	0.003065	-7.0674	1.276766
ELK3	0.033719	0.003068	-1.28457	5.941753
EMILIN1	0.033719	0.003069	2.291146	6.252048
PLA2G4E	0.033719	0.00307	-5.24541	4.291528

SNORA22	0.033883	0.003087	-1.44489	5.115621
CDCP2	0.034041	0.003105	6.869575	0.642484
TMEM176/	0.034118	0.003114	2.795305	3.791921
SLC25A43	0.034135	0.003119	-1.50962	5.356153
DSG1	0.03421	0.003129	-4.67071	8.330647
CSGALNAC	0.03421	0.003131	1.389997	5.150313
ERG28	0.034211	0.003134	-1.3962	5.219557
ZFP28	0.0343	0.003145	-3.15418	2.836606
CAVIN2	0.034609	0.003176	1.669593	5.304195
IGLON5	0.034704	0.003188	-3.45614	4.188304
ENGASE	0.03493	0.003211	1.57347	5.853896
FABP12	0.035419	0.003259	-6.53575	0.847598
ADM	0.035554	0.003274	-2.11481	6.596185
ADGRF4	0.03569	0.00329	-3.58266	5.188468
GBP3	0.035735	0.003297	1.451389	6.273115
RNF208	0.035735	0.0033	-2.72471	3.309295
TMC3	0.035808	0.003309	5.385872	0.936017
UBC	0.035911	0.003322	-1.25884	10.09036
TGM1	0.035952	0.003329	-4.32666	10.11834
MIR573	0.035952	0.003331	-6.38595	0.732445
SNX33	0.03619	0.003356	-1.39071	6.955981
TMEM109	0.0363	0.003369	-1.30074	6.105615
LGI4	0.03642	0.003384	2.207916	3.665876
DLEU1-AS1	0.036944	0.003435	3.810197	1.826368
MGAT4A	0.037046	0.00345	1.781228	4.938532
CYP4F22	0.037046	0.003451	-3.98813	6.000337
CAMK1D	0.037213	0.003473	-1.44814	5.345168
NID2	0.037213	0.003481	1.748729	4.646992
PYGL	0.037213	0.003482	-6.88977	1.130096
SLC7A5	0.037213	0.003483	-2.2239	6.693668
HOXB6	0.037213	0.003485	3.088285	4.342926
AFF4	0.037213	0.003488	-1.25277	8.982593
CUBN	0.037213	0.003489	3.056303	3.531401
FOXC1	0.037213	0.003491	-1.67268	5.254067
KLRG2	0.037213	0.003495	-5.09775	2.145775
CDK5R1	0.037213	0.003496	-2.62494	4.182958
OR7E91P	0.037245	0.003502	-4.30385	1.907159
LOC10099(	0.037267	0.003508	5.441006	0.977461
GBE1	0.037317	0.003516	-1.25927	6.022672
FOXE1	0.037317	0.003518	-3.5798	4.220353
RNF11	0.037531	0.003542	-1.26915	7.285892
SERPINF1	0.037549	0.003547	1.510094	4.912144
CDC42EP5	0.037549	0.003549	4.51114	1.840951
ACSL5	0.037655	0.003562	3.714508	2.170696
RDH12	0.037732	0.003573	-3.20424	3.7399

CPQ	0.037902	0.003592	2.056018	4.419091
C1orf74	0.03794	0.003602	-2.31538	5.214923
VWDE	0.03794	0.003603	-6.71511	0.986599
GLDN	0.03794	0.003605	2.876647	2.468584
RINT1	0.038039	0.00362	-1.50876	5.01503
S100A12	0.038039	0.003621	-3.91155	4.558168
PPP1R14B	0.038039	0.003623	-1.26418	5.648272
ANKRD31	0.03807	0.003629	-3.06878	3.960308
XIST	0.038101	0.003635	-10.5041	7.547878
PAGR1	0.038323	0.00366	1.160737	6.19091
ZMIZ1	0.038323	0.003663	-1.20762	8.254325
CLIP1	0.038529	0.003686	-4.38135	1.8468
SMIM24	0.038602	0.003698	4.298512	6.158674
GIMAP2	0.038602	0.003699	2.584045	2.908452
IRAK1BP1	0.038753	0.003718	1.923488	3.180811
CACNA2D3	0.038753	0.003722	-6.57363	0.877252
GIPC2	0.038753	0.003723	3.874051	1.550904
LOC10050€	0.038905	0.003741	3.218743	3.35791
SNORD57	0.039001	0.003753	1.656258	6.790266
NOS3	0.039133	0.003769	-2.05893	4.621708
PCDHB9	0.039212	0.00378	2.265828	2.905409
CYP51A1-A	0.039272	0.003788	7.238218	0.898527
BIRC8	0.039382	0.003802	6.975923	0.738811
ZDHHC5	0.039461	0.003813	-1.22206	7.989851
TMIE	0.039722	0.003842	-5.04287	1.665994
GREB1	0.039767	0.003849	2.407564	4.07581
ATP2A2	0.039815	0.003857	-1.42029	7.806673
IL1RAP	0.040063	0.003887	-6.41471	0.756737
HOXB3	0.040063	0.003888	1.433925	4.721143
SMG7-AS1	0.040146	0.003899	3.181632	2.353143
KIF3B	0.040365	0.003924	1.21053	6.41376
VSIG10L	0.040365	0.003927	-3.97592	8.366579
C18orf25	0.040365	0.00393	-7.4342	1.592925
ATP10D	0.040407	0.003937	-1.47911	7.035704
PPP1R13L	0.040521	0.003954	-7.90245	2.002849
ADGRL3	0.040521	0.003955	2.242356	4.618566
TM9SF3	0.040661	0.003972	1.10442	9.112826
RNF157	0.040679	0.003977	2.385367	3.245597
CPA2	0.040724	0.003985	-10.0116	3.992763
CHDH	0.040866	0.004002	2.491092	3.850242
GCSH	0.040951	0.004014	6.929896	0.690435
PPP2R3A	0.041062	0.004029	-2.25191	5.15597
PALMD	0.041062	0.004033	-1.53334	6.686878
ACTR10	0.041062	0.004035	-1.39113	5.665947
KLK10	0.041062	0.004038	-3.63054	2.879646

CYTIP	0.04118	0.004053	2.069271	5.401146
DSEL	0.041202	0.004058	2.341141	3.652957
PRSS22	0.041563	0.004097	-1.71176	6.518394
SRRM2-AS1	0.041733	0.004117	-4.01151	2.197509
HNRNPA1F	0.041743	0.004122	-3.60803	2.383467
CAMK2N1	0.041794	0.00413	1.237159	7.034166
IL1RL2	0.041803	0.004135	-3.08302	4.209738
UBA6	0.041803	0.004138	-1.28935	7.18349
DSG1-AS1	0.041862	0.004147	-3.53102	2.620988
ARSJ	0.041876	0.004154	1.472181	4.358228
KCNMB2	0.041876	0.004155	6.277803	1.701146
ABCC13	0.041876	0.004161	5.539456	1.044725
ZDHHC18	0.041876	0.004162	-1.44419	5.660944
NBAS	0.042402	0.00422	3.567398	1.58336
LUM	0.042402	0.004223	1.933164	7.000524
MROH8	0.042402	0.004227	-7.22684	1.410347
UBXN2A	0.042402	0.004228	-1.80476	4.783993
LINC01762	0.042539	0.004245	6.765828	0.578279
HORMAD2	0.042547	0.004249	-6.78576	1.050212
ZNF460	0.042559	0.004255	-1.25541	7.445832
ARHGAP32	0.042559	0.004257	-1.39694	5.670664
PDE11A	0.04259	0.004264	3.698284	2.133491
ARHGAP44	0.042626	0.004271	2.783936	4.229238
C1orf115	0.042849	0.004299	2.038698	5.063682
DDI2	0.042849	0.004305	-1.2561	5.875877
QRFPR	0.042849	0.004306	-7.05143	1.26294
RBBP6	0.042849	0.004307	-7.23876	1.421312
BNIP3L	0.042915	0.004317	-1.2649	7.638746
LRP6	0.043044	0.004334	1.13329	6.958392
N4BP1	0.043044	0.004337	-1.24389	7.294064
UVRAG	0.04312	0.004348	1.171196	5.278696
ZNF222	0.043321	0.004372	6.55008	1.342908
GRM6	0.043371	0.004381	4.02664	2.595992
LINC01005	0.043372	0.004389	7.112029	0.772502
ITGA9	0.043372	0.00439	1.80834	4.423794
SLC16A9	0.043372	0.004391	-1.72438	5.485538
LOC440300	0.043397	0.004397	-1.26996	6.581634
NTF4	0.043411	0.004402	-6.8447	1.092297
CGGBP1	0.043427	0.004409	-7.5962	1.730199
CDH22	0.043427	0.004411	-6.16229	0.564739
WSB1	0.043686	0.004442	-1.29856	5.266967
LYPD2	0.043686	0.004444	-3.52401	4.932207
CCDC6	0.043843	0.004465	-1.27104	7.998386
GRAMD2A	0.043843	0.004467	2.185245	2.613868
SOX2	0.04412	0.004499	-1.52708	5.797812

CATIP-AS2	0.044249	0.00452	4.633409	1.2571
LOC33929ε	0.044249	0.00452	6.571512	1.171517
H3F3B	0.044249	0.004523	-1.23575	9.022043
LILRB5	0.044463	0.004549	5.546591	0.557657
PLET1	0.044519	0.004558	-4.70522	2.247711
VPS9D1	0.0448	0.00459	-1.39873	4.587501
C15orf62	0.04486	0.004601	-1.56758	4.65937
BCYRN1	0.04486	0.004604	-2.60041	4.175087
LINC00668	0.044909	0.004614	3.978671	2.434738
EGLN3	0.044909	0.004616	-2.36048	3.921705
B3GNT2	0.045163	0.004646	-1.22807	5.714811
HSPA4	0.045163	0.00465	-1.26952	8.166629
VAMP8	0.045282	0.004665	-1.16852	6.539531
NR2F1	0.045343	0.004675	1.795245	5.002074
LINC00336	0.045425	0.004687	6.551645	1.156122
UBXN10	0.045451	0.004697	2.554925	2.621016
LRRFIP2	0.045451	0.004698	-1.41122	5.299514
UCP2	0.045451	0.004701	1.936154	4.1045
GNA11	0.045457	0.004707	1.136889	6.77348
PACS2	0.045457	0.004709	-7.24747	1.42737
SLC25A25-/	0.045477	0.004715	1.526482	4.92005
QSER1	0.045527	0.004729	-1.50724	7.421494
SLC5A12	0.045527	0.004731	4.780424	1.379685
GJA8	0.045527	0.004731	6.933788	0.691556
CYP4A11	0.04561	0.004744	-3.72537	2.358745
RIC8B	0.045701	0.004757	1.837003	3.744242
OAS1	0.045837	0.004775	2.86095	2.516881
PTPRZ1	0.045858	0.00478	-1.89711	3.954695
COPZ1	0.04592	0.004791	6.378612	1.091621
DGKD	0.046029	0.004807	4.57313	1.492293
KCMF1	0.046029	0.004809	-1.19837	7.024885
POLR2J	0.046182	0.004829	-1.57615	4.696497
ADH1B	0.046202	0.004835	6.613846	0.463034
ACAP3	0.046285	0.004847	-1.17491	6.699287
ZNF117	0.046409	0.004864	-1.379	6.693991
MBD2	0.046631	0.004891	-1.31994	8.153332
ARHGEF38-	0.04671	0.004903	4.052367	1.788466
HK3	0.046829	0.004923	2.804732	2.459347
TCIRG1	0.046829	0.004923	1.88417	3.760178
ARHGAP27	0.047039	0.004949	-1.27341	7.011798
UGT1A9	0.047075	0.004957	3.215956	2.729428
ARX	0.047192	0.004973	4.209663	2.69564
AGBL4	0.047437	0.005003	7.115459	0.815177
ABHD2	0.047489	0.005012	1.945422	3.523268
TPRXL	0.047578	0.005026	-3.02733	3.644731

COL6A2	0.047578	0.005029	1.874764	4.706294
GIGYF2	0.048083	0.005086	-7.44373	1.598939
ATP6AP1	0.048202	0.005103	-1.23073	6.156034
RNF187	0.048245	0.005111	-1.20687	6.415623
H1FX-AS1	0.048638	0.005157	2.673107	3.042004
ALDOC	0.048672	0.005164	-2.59828	4.612242
PLCL2	0.048806	0.005184	4.556428	1.787204
BCAP31	0.048806	0.005187	-3.68084	1.821731
UNC5B-AS1	0.049064	0.005218	-3.03684	2.618899
TMEM229I	0.049118	0.005228	3.039622	2.879759
METAP1	0.049118	0.005232	-1.28505	6.770802
LOC28314(	0.049164	0.005241	2.384179	3.615803
JUP	0.049166	0.005245	-2.38739	4.470337
SNORA11	0.049166	0.005249	1.623002	6.754212
SDK1	0.049218	0.005258	1.504043	4.49944
LRRN4CL	0.049557	0.005298	3.174032	2.036998
CLOCK	0.049828	0.005335	-2.33993	3.394929
KRT78	0.049828	0.005335	-3.65121	5.824689
DNAJC18	0.049852	0.005342	2.200084	3.334748
UBAP1	0.049856	0.005347	-2.13072	3.411309
SREK1IP1	0.049856	0.005351	-1.27263	6.259849
SIGLEC14	0.049869	0.005356	4.053588	1.250712
ZNF302	0.050194	0.005395	3.228297	2.264619
R3HDML	0.050245	0.005407	4.770998	0.815316
TENM3	0.050245	0.005411	-2.91582	5.325621
RSC1A1	0.050245	0.005413	-1.58403	4.835909
ELOA	0.050379	0.005431	-1.17357	6.839828
NOTCH2NL	0.050395	0.005437	-1.75619	5.449616
MUC21	0.050502	0.005457	-3.74178	2.709771
FGF2	0.050502	0.005459	1.880626	3.790361
SAMD4B	0.050502	0.005461	-1.77632	5.327793
OXSR1	0.050578	0.005473	-1.27175	7.685166
PDILT	0.050753	0.005496	-9.65884	3.649972
LINC02584	0.050824	0.005508	5.025471	0.967099
TNR	0.050925	0.005523	5.647371	1.141257
CRCT1	0.050968	0.005532	-4.18455	7.622149
LGR4	0.05126	0.005568	1.076813	7.028026
POFUT1	0.051267	0.005573	1.083007	5.812366
LINC00955	0.051549	0.005607	6.966629	0.701915
PAK6	0.051869	0.005647	-3.80427	2.160079
PI3	0.05189	0.005653	-2.96955	9.835502
FAM83D	0.051912	0.00566	-1.96445	5.571555
SOSTDC1	0.051979	0.005671	2.877839	2.715974
EGFR	0.052034	0.005681	-1.15918	7.779
PAQR7	0.052072	0.00569	-1.85979	5.303715

FBXL16	0.052298	0.005719	-1.41084	5.310941
KDF1	0.052347	0.005734	-2.0098	3.738526
ARG2	0.052347	0.005743	-2.64002	4.637584
OTOP2	0.052347	0.005743	-3.49713	4.734963
GOLM1	0.052347	0.005746	4.598678	1.994736
IQSEC1	0.052347	0.005751	1.339689	4.692623
LINC00958	0.052347	0.005754	-3.32929	3.989269
MARCKSL1	0.052347	0.005757	3.726878	1.36923
GTPBP3	0.052347	0.005758	-4.53877	1.859357
EVI2B	0.052647	0.005795	1.460442	5.095123
KLK9	0.052647	0.005801	-3.62658	2.791108
MAOB	0.052647	0.005807	1.805801	4.73015
NCR3LG1	0.052647	0.005808	1.349612	4.598906
ADAM23	0.052915	0.005842	-2.06343	5.136861
KRT16P1	0.053031	0.005859	-3.01401	4.038318
SH3TC2	0.053349	0.005898	-1.64106	4.667822
MIDN	0.053447	0.005914	-1.2978	7.731688
LIPE-AS1	0.053643	0.00594	-4.5149	1.95779
ACER1	0.053675	0.005947	-3.38275	2.522409
HPS4	0.053752	0.00596	-5.1774	1.08826
XPOT	0.053758	0.005965	-1.16769	7.188849
GIMAP8	0.053928	0.005989	1.864046	4.517733
GYS2	0.054044	0.006006	-3.19466	3.500753
PLVAP	0.054305	0.006039	1.465428	5.749551
EP300-AS1	0.054314	0.006045	-3.75372	2.026781
SLC35F6	0.054314	0.006051	-1.18328	6.319534
CCL11	0.054314	0.006053	2.971562	3.400127
EPCAM-DT	0.054375	0.006073	4.273478	1.157762
GIF	0.054375	0.006074	-7.83592	7.530868
MYO5C	0.054375	0.006077	1.136783	7.44994
WNK4	0.054375	0.006078	1.467089	5.963463
DTX4	0.05478	0.006128	3.146406	2.085433
CCDC85C	0.055145	0.006173	-1.17703	6.322789
RNF167	0.055267	0.006191	-1.14547	5.810749
ERN2	0.055288	0.006198	4.181261	1.084609
IVL	0.055313	0.006208	-4.69835	9.62887
SH3BP1	0.055313	0.006212	-1.34506	4.896834
DOCK2	0.055313	0.006219	1.949815	5.205949
CALML3	0.055313	0.006221	-4.35513	8.593752
NAALADL2	0.055313	0.006223	1.787909	4.307162
S100A9	0.055397	0.006237	-3.97037	11.65271
ZNF239	0.055429	0.006245	4.42036	1.447455
CNTNAP1	0.05546	0.006253	1.750889	4.484491
FUK	0.055774	0.006293	1.469561	4.42575
LCP1	0.055879	0.006312	1.37594	5.720897

MIR4531	0.055879	0.006314	7.444586	1.096047
KIF12	0.056179	0.006352	3.195764	4.090884
GAS6-DT	0.056202	0.00636	3.454794	1.474542
CHPT1	0.056306	0.006376	1.487074	5.105406
ABCC6P2	0.056617	0.006416	4.176124	2.005514
KCNH8	0.057385	0.006512	3.314728	3.479326
C11orf42	0.057385	0.006512	5.365256	0.922519
PRKX	0.057385	0.006518	-1.16497	6.16304
ACAP2	0.057385	0.006521	-1.26616	7.971792
CLCA2	0.057431	0.006531	-4.10804	9.212139
TNFRSF25	0.057581	0.006553	-2.61427	3.6415
FAM135A	0.057672	0.006568	-1.73305	3.549569
ANKRD20A	0.057705	0.006576	-2.47065	4.173533
PINK1	0.05789	0.006606	-1.42357	4.439183
PGC	0.05789	0.006607	-8.41178	10.78306
CXorf38	0.058365	0.006666	1.247513	4.693313
DDAH1	0.058407	0.006675	2.361032	3.203813
RCSD1	0.058474	0.006688	1.824469	4.625216
SNORD143	0.058516	0.006699	-6.87732	1.119715
PZP	0.058516	0.006702	2.470362	3.594053
TSPAN12	0.058573	0.006717	2.918756	4.032854
PARD6G-AS	0.058573	0.006722	-2.87817	3.784627
TRHDE	0.058573	0.006723	3.719849	3.424066
KLK8	0.058586	0.006734	-6.73418	1.004521
IFNE	0.058586	0.006734	-3.8695	2.608218
CYP27C1	0.058586	0.006738	-3.06454	3.225826
TSPAN5	0.058625	0.006754	-1.40194	5.274902
LINC00540	0.058625	0.006754	4.61607	0.667186
MRM3	0.058625	0.006757	-4.89955	0.871323
NLRP13	0.058662	0.006766	6.815178	0.583558
FOXA3	0.058662	0.006775	3.198537	3.235459
TPSB2	0.058662	0.006776	2.186225	3.647962
PROKR1	0.058691	0.006784	7.391905	1.05311
ITPRIP	0.058756	0.006798	-2.49399	4.454783
PRDM1	0.058756	0.006807	-1.5435	6.430386
CALM1	0.058756	0.006808	-1.13148	9.548347
PGAM1	0.058756	0.006817	-2.54246	3.421307
POLR2I	0.058756	0.006819	-1.47265	4.241989
C11orf86	0.058756	0.00682	5.904631	1.661324
PABPC1	0.059038	0.006857	-1.24002	11.52974
PSTPIP1	0.059108	0.00687	1.591374	4.191113
HECA	0.059173	0.006883	-1.16971	7.297347
CADM3-AS	0.059452	0.00692	5.110263	1.755273
METTL4	0.059462	0.00693	4.448657	1.955861
GOLGA2P7	0.059462	0.006931	-1.88647	3.778334

HKR1	0.059777	0.006973	1.109843	5.809578
RAP2A	0.059777	0.00698	-1.25136	6.805866
CSNK1A1P1	0.059777	0.006982	2.719827	2.968778
MXD4	0.059866	0.006997	1.127493	5.511451
S100P	0.060086	0.007028	1.285133	6.160526
LNX1-AS2	0.060113	0.007036	4.542885	1.175443
KRT18	0.060306	0.007068	7.347187	1.032554
NBL1	0.060306	0.007068	1.958263	2.954589
CPD	0.060385	0.007082	1.765812	4.123694
WNT7B	0.060408	0.007095	-2.81287	4.548597
ARHGAP28	0.060408	0.007095	2.577747	3.511275
LMTK3	0.060448	0.007104	-2.22969	3.896547
WTAPP1	0.060674	0.007138	4.326376	1.616544
RASA4B	0.060674	0.007144	2.339618	2.606928
HID1	0.060674	0.007146	2.104475	6.10562
DNAH5	0.06071	0.007156	2.451561	3.559866
CSK	0.06071	0.00716	-2.71996	2.176643
CHRM1	0.060735	0.007168	4.737002	1.653952
FBXO27	0.060986	0.007202	-3.28802	3.720171
LOC729966	0.061014	0.00721	2.783782	2.629536
ZFHX3	0.061029	0.007217	1.156285	5.10528
IGF2BP3	0.061032	0.007222	-2.02369	4.080052
FGL2	0.061412	0.007272	1.332197	5.966419
CCDC88C	0.061594	0.007299	0.981971	7.57881
FUCA1	0.061787	0.007327	1.163811	4.98224
CBLB	0.061947	0.007353	1.074301	5.838917
GSTA2	0.061947	0.007361	5.33012	0.919664
HEXB	0.061947	0.007361	2.169683	2.744923
SEC22C	0.062054	0.007379	1.358873	4.883045
NVL	0.062269	0.007409	7.315443	1.007151
C1orf61	0.062319	0.00742	6.773953	0.57231
SMARCA4	0.062332	0.007427	-2.87734	2.856806
SUDS3	0.062356	0.007439	-1.12969	7.205889
TRMT10C	0.062356	0.00744	-1.49125	4.211404
NDRG4	0.062473	0.007459	-3.97535	1.751302
AGPAT4-IT	0.062501	0.007467	-2.87993	2.749181
RPS6KB2	0.062855	0.00752	-1.30115	6.459044
FRZB	0.062855	0.007524	2.669447	2.644384
MGC2889	0.062855	0.007525	-4.40889	1.550153
TMPRSS11	0.062861	0.007535	-3.6013	8.177046
SLC4A8	0.062861	0.007541	4.99851	0.672216
LHFPL6	0.062861	0.007544	1.492032	4.881819
HOXC9	0.062861	0.007547	4.22333	1.532036
USP32	0.062861	0.007551	-1.18524	7.099204
LOC72855	0.063072	0.007585	-1.28389	6.351205

SGPP2	0.063072	0.007586	-1.07076	5.777969
FBXO42	0.063136	0.007603	-1.16973	5.851751
LOXL2	0.063136	0.007604	2.218604	4.638355
NAA20	0.063296	0.007629	-2.82008	2.682186
NMRAL2P	0.063396	0.007646	-2.67693	3.999183
KCNJ9	0.063486	0.007662	3.526265	2.379986
ARHGEF37	0.063596	0.00768	-1.39166	6.259595
RNASEK	0.063608	0.007689	-7.18762	1.379419
SRR	0.063608	0.007692	-6.34255	0.702971
RNU5D-1	0.063672	0.007706	-2.01817	4.856966
CEACAM1	0.063672	0.007714	-3.43345	2.991803
CDH6	0.063672	0.007715	1.937683	4.704622
GBP1P1	0.063943	0.007753	-2.92376	3.306476
GLIS3	0.064199	0.00779	2.589394	2.628532
HM13-AS1	0.064217	0.007797	-5.23742	1.19394
GTF2F2	0.064298	0.007812	-1.25259	5.040794
KRT10	0.064467	0.007838	-2.28871	6.828921
DBNL	0.064558	0.007854	7.280396	0.96319
CUL4B	0.064667	0.007878	2.790258	3.001284
HPDL	0.064667	0.007878	2.899046	2.168552
CLEC4E	0.064865	0.007907	3.345517	2.50209
LINC01002	0.065193	0.007952	-2.18193	3.881961
ANKRD44	0.065214	0.00796	1.861749	4.14981
ADGRE4P	0.065366	0.007984	3.024241	1.905336
CNKSR3	0.065664	0.008026	-1.24663	6.473771
CBLL1	0.065664	0.008037	5.9333	1.099402
TMPRSS11	0.065664	0.008039	-3.55172	5.927124
LINC01184	0.065664	0.008046	1.26078	5.90335
GRHL3	0.065664	0.008047	-3.35056	5.411525
PLCE1-AS1	0.065702	0.008057	3.261357	2.361134
DIS3	0.065713	0.008064	-2.63403	2.891958
IFI44L	0.066111	0.008118	2.354158	4.186811
KRT3	0.066111	0.008123	-2.773	3.377423
CHST2	0.066372	0.008161	2.571385	3.672393
FAM83F	0.066581	0.008192	-1.19339	5.23401
NCCRP1	0.066788	0.008223	-4.16019	8.449263
FAM200A	0.066956	0.008251	-2.18839	3.442656
H2AFY2	0.066956	0.008254	-2.61451	3.98016
ABCA6	0.067106	0.008278	1.803811	4.764277
TLR1	0.067284	0.008306	2.344003	2.461344
TEX13B	0.067431	0.008329	-6.05058	0.483716
GIPC3	0.067566	0.008351	2.695943	2.658588
EXOC5	0.067703	0.008376	-1.21877	6.853033
VWA7	0.067703	0.008379	3.497519	1.270928
RALBP1	0.067824	0.0084	-1.10607	8.250002

RARRES2	0.0679	0.008428	2.28751	4.069731
UBLCP1	0.0679	0.00843	-1.28341	5.160781
ETNK1	0.0679	0.008438	1.053758	7.071007
IER3	0.0679	0.008442	-2.67373	4.268048
PIEZO2	0.0679	0.008444	1.682481	5.317946
ADAMTS6	0.0679	0.008445	2.43805	3.467124
CHN2	0.0679	0.008448	3.145537	2.516273
FOXN1	0.068015	0.008468	-2.93413	4.652752
SLC48A1	0.068054	0.008479	-1.2818	4.90796
RPL23A	0.068054	0.008483	-1.04239	7.435134
MIR3174	0.068174	0.008504	-5.96263	0.423965
NUGGC	0.068408	0.008544	2.393939	4.214272
SIK1	0.068408	0.008548	-2.78601	5.793857
SEC22B	0.068408	0.00855	1.006888	6.090652
AACS	0.068683	0.00859	-1.11121	6.331505
SLC6A9	0.068725	0.0086	-6.43052	0.776387
MAMDC4	0.068871	0.008624	1.29329	4.68309
KCNG1	0.068917	0.008636	-3.72216	1.748107
CALML3-AS	0.069043	0.008666	-6.45876	0.789189
DLL4	0.069043	0.008667	2.195832	3.070257
TRIM7	0.069043	0.008668	-3.48035	2.478333
TP53TG5	0.069157	0.008688	-4.85035	0.886611
BIVM	0.069248	0.008705	-6.45826	0.790733
F2RL1	0.06932	0.00872	1.175701	5.265134
LINC01843	0.069394	0.008735	4.060489	0.958412
PHTF1	0.069461	0.008749	1.38359	4.490511
HSBP1	0.069528	0.008763	-1.38887	6.544754
CNNM3	0.069603	0.008778	4.212764	1.384195
MPPED2	0.069727	0.008799	-4.06926	1.832182
FER1L6-AS	0.069893	0.008826	4.002193	1.599875
FABP5P3	0.070073	0.008854	-4.55285	1.253667
MKX-AS1	0.070292	0.00889	6.502428	1.329684
MTSS1L	0.070292	0.008893	-1.08909	6.635429
LINC01348	0.070307	0.008902	-6.73219	1.003738
LOC10050	0.070307	0.008908	-6.53483	0.849385
LOC10050	0.070307	0.008912	2.424142	3.02254
MIR5585	0.070545	0.008954	5.708083	0.690216
SLC39A14	0.070545	0.008959	2.96382	2.583934
LOC10192	0.070545	0.008962	4.115139	0.809824
CDH26	0.070545	0.008965	-2.96423	3.148509
UGT1A10	0.070602	0.008978	3.071601	3.161137
KCNS2	0.07066	0.008991	2.894896	2.468853
NRN1	0.070783	0.009013	6.625872	0.461376
SMILR	0.070992	0.009045	-4.02675	2.494214
LOC10012	0.07105	0.00906	-2.02276	4.626168

SBSN	0.07105	0.009064	-3.98041	9.444019
CECR7	0.071099	0.009076	-2.85388	3.089199
KLRK1	0.071175	0.009091	7.163256	0.886886
CYP4X1	0.071471	0.009135	-1.55205	4.429502
RAB7A	0.07163	0.009161	-1.13823	8.223154
OMP	0.071829	0.009192	2.838376	1.923262
GFPT1	0.071862	0.009202	2.659305	1.803806
ST3GAL2	0.071876	0.00921	1.635881	3.732601
SLAIN1	0.071951	0.009226	3.769108	2.025774
CILP	0.072263	0.009282	1.781455	3.951912
TMPRSS11	0.072263	0.009282	-4.29397	9.106536
COX6A1	0.072263	0.009283	-1.05153	5.625007
KPNA4	0.072392	0.009306	-1.10906	7.888999
ZXDA	0.072415	0.009314	2.203122	3.15454
KLHL21	0.072472	0.009329	-1.40581	6.011641
CBX4	0.072472	0.009337	-1.09002	5.992454
MIR4785	0.072472	0.009339	-6.47165	0.79965
TMPRSS11	0.072493	0.009348	-3.93898	6.773233
TMEM63C	0.072537	0.009359	-2.69215	4.137543
SCCPDH	0.072917	0.009414	1.670207	4.223769
FAM157C	0.072953	0.009426	-1.56704	4.72116
PLAT	0.072953	0.009431	-7.57598	1.713377
EFHD2	0.073098	0.009456	1.175779	7.676078
UNC5CL	0.073174	0.009471	1.802358	4.826836
PI4K2A	0.073297	0.009493	-1.6312	5.678627
MIR4257	0.073332	0.009505	-3.64584	1.457046
SNORD130	0.073332	0.00951	-6.32275	0.690342
COL12A1	0.073332	0.009516	1.844704	6.214539
TBC1D2	0.073332	0.009527	-6.28724	0.657678
ATP1A1	0.073332	0.009537	1.460739	4.951827
CASC4	0.073332	0.009539	1.606379	3.649784
DUOXA1	0.073332	0.009543	-3.22517	2.671791
MIR224	0.073332	0.009545	-6.36602	0.726632
IGFBP2	0.073451	0.009567	-2.64726	3.160337
ZNFX1	0.07352	0.009582	1.014786	7.035232
ARHGEF34	0.073604	0.009598	-1.33999	7.677044
CSF2RB	0.073639	0.00961	1.754256	4.475306
MCRIP2	0.073639	0.009615	1.757813	3.288713
ARMCX4	0.07374	0.009634	1.433728	4.348689
BCAS2	0.073939	0.009666	-1.33733	5.061404
CBX2	0.074133	0.009697	-2.68553	3.125835
WNT11	0.074374	0.009738	-6.72803	1.001193
GALNT5	0.074374	0.009741	0.991597	6.414764
FASN	0.074768	0.009799	-1.14283	8.168737
PCDHB12	0.074789	0.009812	3.525444	1.756671

SLC9A9-AS	0.074789	0.009813	-5.17793	1.1393
SNORA11B	0.074801	0.009821	1.876886	3.613535
SLC38A3	0.075032	0.009858	-6.39569	0.74182
B2M	0.075033	0.009864	1.204407	6.690734
DENND2A	0.07527	0.009905	1.932706	4.185734
NEK4	0.07527	0.009907	-4.67218	0.74521
SNORD113	0.075276	0.009917	1.715781	3.311725
RNF5	0.075276	0.009922	2.391622	2.812507
LOC101928	0.075276	0.009934	-5.03002	1.022423
COL9A2	0.075276	0.009939	2.630288	4.022589
CCDC68	0.075276	0.009946	5.634084	1.508131
HDAC3	0.075276	0.009949	-1.29219	5.787795
POLR2A	0.075276	0.009951	-1.07798	9.210402
GARS-DT	0.075276	0.009957	1.168285	5.209118
PDE7B	0.075412	0.009981	2.010328	3.691218
FRMD6-AS1	0.075629	0.010016	-3.16988	4.067986
ZNF812P	0.075739	0.010036	-2.99125	5.400601
FRMD6	0.075846	0.010057	-3.09208	4.569083
MIR3909	0.07607	0.010094	-6.51781	0.839529
SCARNA14	0.07607	0.010099	-6.31909	0.681273
CLEC14A	0.076221	0.010125	2.038682	3.073353
HERC2P7	0.076245	0.010134	1.537036	3.857468
CTRB1	0.076293	0.010147	5.838873	0.7941
KLK1	0.076351	0.010161	2.517986	3.066629
PGA4	0.076351	0.010167	-10.2067	7.057404
ZNF883	0.076425	0.010183	-3.98352	1.757592
TIFA	0.076475	0.010196	-1.94779	4.96956
FLJ13224	0.076547	0.010212	1.991061	2.654711
PDIA4	0.076724	0.010241	1.007317	7.992612
RLF	0.076755	0.010252	-1.17369	6.85528
PLCD3	0.076776	0.010261	1.276716	6.156692
RAMAC	0.076897	0.010288	-2.47766	3.078905
AREG	0.076897	0.010294	-2.33962	5.683169
L3MBTL4	0.076897	0.010301	-1.99035	3.643691
CALML5	0.076897	0.010302	-3.12166	3.251736
RPL39	0.076961	0.010317	-1.29385	6.384992
RAET1E	0.077035	0.010335	-3.01534	6.671003
SMDT1	0.077035	0.010339	-3.40724	2.882473
ROR2	0.077229	0.010377	1.879688	3.644579
SYNJ2BP	0.077229	0.010378	1.049928	6.692017
NBEAL2	0.077229	0.010396	-1.18874	9.152659
A2ML1	0.077229	0.010396	-4.38829	9.915249
MMRN1	0.077229	0.010396	2.889268	4.428069
TPRKB	0.077524	0.010451	-2.2203	4.210993
PTGDS	0.077524	0.010454	1.465506	4.70807

FMO9P	0.077524	0.010455	-6.25246	0.634639
HIST1H2AC	0.077754	0.010492	-1.1937	5.276391
MAP3K10	0.077754	0.010499	1.443435	4.261632
TMC1	0.077816	0.010514	-3.31103	2.372328
BDKRB2	0.077816	0.01052	-1.29737	6.203385
RNF169	0.078156	0.010572	-1.05785	7.403038
DUSP11	0.078389	0.01061	-1.25835	5.103337
COL16A1	0.078395	0.010617	1.312919	6.457915
YWHAG	0.078666	0.01066	-1.09611	7.983079
LOC100128	0.078666	0.010666	-1.11125	6.588686
EPN3	0.078873	0.010701	-1.39805	6.154882
TGM2	0.079364	0.010781	1.985222	4.56399
ABLIM1	0.079364	0.010785	-1.43427	4.811326
C11orf80	0.079364	0.010788	-3.13508	2.44165
ZNF780B	0.079364	0.010793	1.036927	5.569848
SLC2A13	0.079379	0.010802	1.411375	4.706461
LOC285889	0.0794	0.010811	4.273784	1.145782
GJA1	0.079474	0.010827	-1.40427	7.512887
NXF3	0.079705	0.010871	3.054855	2.208576
PTCHD4	0.079705	0.010876	3.001776	2.252654
RPL9	0.079705	0.010878	-6.12635	0.53841
TUBA1C	0.079792	0.010897	-3.93036	1.892435
RARG	0.079981	0.010929	-2.38671	3.032471
STX12	0.080505	0.011008	-1.04872	6.775012
LY75	0.080505	0.011013	2.215395	3.327975
RTL8B	0.08067	0.011043	-2.37669	3.431351
GNAS-AS1	0.081093	0.011108	-6.23503	0.622537
ERMP1	0.081093	0.011114	-1.02767	7.32194
LOC101928	0.081148	0.011128	-6.05399	0.487185
NOTCH2	0.081148	0.011134	-1.11445	8.141603
CDC42EP2	0.081154	0.011142	2.166362	2.789566
C1orf52	0.081172	0.011151	5.642364	1.060537
SPEF2	0.081317	0.011177	2.770695	2.223679
FAM43A	0.081381	0.011193	-1.52157	4.838936
DTX2P1-UP	0.081453	0.011212	-1.10773	5.845029
PRTG	0.081453	0.011216	2.691497	2.556704
SEMA3A	0.081453	0.011222	1.864144	3.422827
FTLP10	0.081499	0.011241	-3.12263	3.917321
BICC1	0.081499	0.011242	2.235795	3.937983
LURAP1L	0.081561	0.011257	1.985768	3.607772
APOBEC3D	0.081578	0.011268	2.280002	2.474851
ZSCAN18	0.081578	0.011278	-7.14341	1.339586
CTSC	0.081578	0.011281	1.327978	5.437299
SENP6	0.081578	0.011286	-2.46981	2.76532
UBXN7	0.081578	0.011293	-1.07496	6.804806

NDNF	0.081704	0.011317	2.083537	4.05273
MMP2	0.081877	0.011347	2.430108	3.485802
B4GALT5	0.081956	0.011368	-1.13169	7.179516
IHH	0.081956	0.011371	3.360226	2.749861
TNFAIP2	0.082237	0.011417	-1.30156	8.029069
KIAA1217	0.082774	0.011498	-1.09923	7.418064
TARID	0.082944	0.011529	3.871126	2.218948
RNVU1-1	0.082955	0.011537	-3.9588	1.641948
MINDY1	0.083084	0.011562	-2.27943	3.249891
BRI3BP	0.083084	0.011568	1.752709	3.772514
RHOBTB3	0.083141	0.011583	1.660088	5.991105
KANSL1	0.08323	0.011602	-2.56878	2.867488
DNAJC7	0.083353	0.011626	-4.51427	0.632164
CSNK1G1	0.083498	0.011653	-1.06783	6.562837
PRKCA-AS1	0.083584	0.011676	1.83843	2.987633
LURAP1L-A	0.083584	0.011679	5.550447	0.551545
CALD1	0.083645	0.011694	1.793495	7.785448
LOC101927	0.083722	0.011715	-7.10241	1.304884
MIR4720	0.083722	0.011718	-6.00396	0.453781
MIR4480	0.083848	0.011742	-4.39185	1.046194
SERPINB11	0.084118	0.011789	-6.48665	0.815901
HOXB-AS3	0.084118	0.011794	4.485311	1.291421
FUCA2	0.084206	0.011813	1.143284	5.024024
YWHAZ	0.084276	0.01183	-1.18278	5.267954
TCEANC2	0.084643	0.011888	1.100262	5.215527
KCTD21	0.084723	0.011909	-1.76298	3.867866
NBEA	0.084723	0.011913	1.604245	5.196536
DNAJB6	0.084982	0.011968	-1.27275	6.180165
NUP205	0.084982	0.01197	-1.16086	6.605307
GALNT17	0.084982	0.01197	2.616989	2.364505
RABAC1	0.085078	0.011991	1.145104	4.461695
CYP2C9	0.085136	0.012006	1.291427	5.141554
IL2RA	0.085356	0.012044	5.65447	0.655824
ASB16-AS1	0.085408	0.012058	-6.19485	0.591392
EDEM3	0.085467	0.012073	0.996213	7.429164
LOC103908	0.085809	0.012128	-6.25093	0.641359
DGKQ	0.086133	0.012181	0.998521	5.310695
MOGAT1	0.086198	0.012197	-6.3487	0.705383
CSTA	0.086414	0.012235	-4.52166	10.63373
PIK3CG	0.086449	0.012247	6.321452	1.177311
TXLNG	0.087216	0.012366	-2.86933	2.182779
RNFT1-DT	0.087216	0.01237	3.395848	1.819518
LEKR1	0.087399	0.012403	-6.16517	0.568027
HIST1H3C	0.087642	0.012444	-1.39721	4.189866
B4GALT1	0.088093	0.012522	-1.0034	7.541485

TMEM67	0.088093	0.012523	-6.30304	0.679936
TOX	0.088093	0.012537	2.90265	4.297196
LOC28576€	0.088093	0.012537	6.86742	0.660709
BRWD3	0.088111	0.01255	-1.07023	7.437996
DGAT1	0.088111	0.012554	1.029125	5.338268
FGD2	0.088186	0.012571	1.697844	4.406026
NPDC1	0.088388	0.012607	1.707526	5.01669
ZNF888	0.088881	0.012685	1.44724	4.23471
MAPK6	0.088975	0.012706	-1.22953	8.082101
SLC24A3	0.08911	0.012732	-1.27005	5.901091
ZNRF1	0.089193	0.012752	-1.00023	5.793585
MYC	0.089193	0.012758	-1.25172	6.864484
LOC72968:	0.089255	0.012774	3.330529	1.914234
LTN1	0.089269	0.012784	-1.03446	6.531829
IL12A	0.089501	0.012824	-2.66071	2.741847
LYPLAL1-D	0.089505	0.012832	5.528026	0.577258
RBP7	0.089896	0.012899	-2.66285	3.255069
ATP4B	0.089896	0.01291	-8.06264	7.027862
CDKL2	0.089896	0.012913	-3.73083	1.930897
SDR9C7	0.089896	0.012931	-3.11697	3.825081
VSTM4	0.089896	0.012938	1.758827	3.980971
CD200R1L	0.089896	0.012938	6.84535	0.644272
ABCC1	0.089896	0.012939	-1.36199	5.801115
HS3ST4	0.090152	0.012983	-6.3021	0.675023
FRG1DP	0.090325	0.013015	-6.83421	1.09553
NR0B2	0.090569	0.013058	4.027408	1.421718
WWC3	0.09064	0.013075	-1.15374	6.489602
KDELC1	0.090673	0.013088	2.504904	2.011261
LINC00664	0.090857	0.013121	-6.1114	0.530832
KIAA1549L	0.090935	0.01314	-2.4545	3.948184
DCAF13	0.091302	0.013201	-1.19556	5.114672
MAB21L4	0.091302	0.013208	-3.01266	3.818279
ZSCAN16	0.091379	0.013226	1.924066	3.294868
UBE2D4	0.091622	0.013276	-1.34988	4.850917
EMC3	0.091622	0.013277	-1.34444	4.960646
LIFR-AS1	0.091648	0.013288	-7.14926	1.341558
RFTN1	0.092007	0.013347	1.281422	4.878209
NONO	0.092013	0.013355	4.470707	0.822629
ESRRB	0.092136	0.013381	-5.87549	1.717955
ADGRA2	0.092353	0.01342	1.623784	5.26237
PPIL1	0.093048	0.013528	-1.42272	4.401643
CMTM7	0.093195	0.013564	4.945441	0.620055
TGFB1	0.093195	0.013565	-1.59445	7.045314
ANXA3	0.093208	0.013574	-1.12069	6.494392
SMG6	0.093222	0.013586	-1.08617	6.950358

BLACAT1	0.093222	0.013591	-2.45358	3.249657
RAB22A	0.093766	0.013678	-1.066	7.520616
HDGFL3	0.093958	0.013722	-1.56522	4.793279
RAB12	0.093958	0.013723	-1.09408	5.965775
HILPDA	0.093958	0.013729	-6.72761	1.000064
MIR590	0.094109	0.013765	-6.10976	0.533476
TIMP2	0.094109	0.013766	1.139147	6.928571
HAVCR2	0.094122	0.013783	2.585516	3.322284
KPNA3	0.094122	0.013789	-1.0221	6.395843
STK32A	0.094122	0.013791	3.127747	1.301049
MFSD14B	0.094491	0.013853	-1.08087	6.277691
GPC6	0.094725	0.013895	2.438515	4.687083
THSD7B	0.094816	0.013916	-2.55456	3.133738
RNF149	0.09485	0.013929	-1.18659	6.868347
KCNN4	0.095324	0.014006	2.10137	4.548716
KLLN	0.095729	0.014073	2.213535	2.675024
ZRANB1	0.095885	0.014104	-1.12687	6.922858
SMCO4	0.096077	0.01414	1.970862	3.709671
DPYD-AS2	0.096341	0.014187	-6.28154	0.668343
GMDS	0.09656	0.014227	1.696297	3.227317
IRF6	0.096716	0.014264	-1.9652	3.153612
LOC441204	0.096716	0.014265	3.592421	1.302324
FRY	0.096839	0.014291	1.458573	5.494057
PTPRC	0.097017	0.014326	1.72866	4.251044
SNORA58	0.097049	0.014338	1.353031	6.798788
WNT5A	0.097063	0.014356	-2.13289	4.032645
LOC101928	0.097063	0.014361	-1.41266	4.960858
TP53AIP1	0.097063	0.014364	-3.0695	3.013659
PDGFRA	0.097353	0.014421	1.125262	7.081103
POLR3G	0.097353	0.014422	-2.24	3.938029
PTPN14	0.097611	0.014469	-1.00434	7.145436
PPP1R3G	0.097719	0.014492	-2.23009	2.82071
GBP6	0.097823	0.014516	-4.02917	10.60052
COL6A3	0.097848	0.014527	1.863634	2.791411
MT1M	0.097855	0.014536	3.342069	2.633159
TGM3	0.098143	0.014593	-4.50006	10.91612
SLC19A3	0.098143	0.014595	3.432198	1.340341
SP9	0.098244	0.014618	6.220467	1.081167
SLAIN2	0.098291	0.014636	-1.01962	7.245264
CLDN4	0.098291	0.014646	-1.16958	7.100239
FAM155A-I	0.098291	0.014649	2.860992	3.071211
BLVRB	0.098408	0.014676	-1.33389	5.397308
DIP2B	0.098408	0.014682	-1.04372	7.948996
IMP3	0.098429	0.014693	-1.30295	4.977846
DDX60	0.098474	0.014714	2.03688	5.659209

EPHB3	0.098474	0.014719	-1.14606	6.474796
ABCA9	0.098474	0.014724	2.197327	3.801167
LXN	0.098568	0.014746	1.798092	3.262736
LOC14869(	0.098724	0.014777	1.048367	5.880997
LINC01134	0.098845	0.014803	4.322954	1.337236
ASCL2	0.09931	0.014885	2.596153	1.810977
CAMK1	0.09931	0.014889	1.722183	3.55917
CYP3A7-CY	0.099456	0.014919	-6.17627	0.575151
PRKAA2	0.099488	0.014932	-2.76413	3.852713
ZC3H12D	0.099766	0.014982	2.183587	2.840699
GDPD3	0.10005	0.015035	-1.46612	5.379295
WEE1	0.10005	0.015041	-1.95207	3.843234
CRACR2B	0.100484	0.015122	3.355957	2.072283
SNX31	0.100484	0.015122	-2.80761	3.643446
MIR6124	0.100541	0.015139	4.738336	0.80932
DIP2C	0.10068	0.015168	1.056043	5.819133
ZBTB7C	0.100779	0.015191	-1.03638	7.191175
CHRNB2	0.100845	0.015209	-5.01244	1.303309
RRM1	0.101049	0.015256	-1.04793	6.303005
AKAP10	0.101049	0.015256	-0.93721	5.965389
KRT6C	0.101055	0.015265	-3.91554	10.3237
BMP8B	0.101226	0.0153	2.772833	2.079119
ENTPD5	0.101425	0.015344	1.173633	5.929702
DIRC2	0.101425	0.015346	-0.9681	5.447662
THAP9-AS1	0.101456	0.015362	-3.10452	2.38008
PDIA3P1	0.101456	0.015367	0.941003	5.971096
CDR2L	0.101471	0.015378	1.263725	4.709729
LINC00926	0.101654	0.015414	2.334573	2.087965
PODXL2	0.101654	0.015424	1.735317	4.007286
JMJD7	0.101654	0.01543	-1.26626	4.210265
ZNF675	0.101714	0.015457	-1.5727	4.540416
ZXDB	0.101714	0.01546	1.374988	4.191017
MARK2	0.101714	0.015464	-4.30637	1.773154
LINC02487	0.101937	0.015506	-3.78374	7.813126
MTRNR2L3	0.102263	0.015564	-3.66876	2.155673
HPX	0.1023	0.015578	-6.39632	0.737983
ING1	0.102599	0.015638	2.440403	2.412876
YBX3P1	0.102599	0.01564	-1.66372	3.439514
FNBP1L	0.102642	0.015655	1.152365	4.795967
SHB	0.102949	0.01571	-1.06126	6.287502
FAM81A	0.103132	0.015746	3.045189	2.502684
GPR182	0.103167	0.01576	-6.1017	0.520272
HLA-DRA	0.103218	0.015776	1.436922	5.340804
ARL6IP1	0.103243	0.015788	1.662017	4.077363
MIR3137	0.103353	0.015816	-6.1045	0.526388

APOBR	0.103353	0.015822	1.518693	4.483414
ZBTB38	0.103376	0.015841	1.087353	6.861566
BLZF1	0.103376	0.015842	-1.11208	5.805246
KIAA1257	0.103424	0.015858	3.921197	1.086358
IFI35	0.103796	0.015923	1.630809	3.633879
IBSP	0.103806	0.015933	6.630068	0.487091
CRACR2A	0.103913	0.015969	1.88369	3.313656
RAB14	0.103913	0.01597	-0.99534	7.41356
SNORA80B	0.103913	0.015975	2.213157	3.048275
SARM1	0.104601	0.016089	2.022183	2.651952
FMNL1	0.104863	0.016142	1.258067	4.735389
PHLDA2	0.104863	0.016154	-6.43781	0.774411
SLA	0.104863	0.016155	2.866523	1.664779
CTBP1-DT	0.105091	0.016199	1.428244	3.609137
GBA2	0.105118	0.016218	0.960524	6.157998
PCDHB15	0.105118	0.01622	2.589391	2.12969
CLDN10-AS	0.105138	0.016238	4.292806	1.343919
SORBS1	0.105138	0.01624	-5.94372	0.41569
PPM1H	0.105357	0.016282	2.16523	4.32265
MARK1	0.105386	0.016295	-5.43984	1.35645
GPD1L	0.105447	0.016315	-1.23243	6.518262
LMOD1	0.105447	0.016322	2.075518	6.497614
DMKN	0.105633	0.016367	-3.32371	2.018188
NR5A2	0.105633	0.016368	4.793217	0.827202
PTGER4	0.106354	0.016488	1.383767	4.675739
CCDC190	0.1064	0.016504	-3.81743	2.557346
FXYD3	0.106419	0.016515	2.368404	2.928539
WNK1	0.106553	0.016545	-1.33433	4.173349
PARVA	0.106687	0.016587	1.01512	6.458865
MVD	0.106687	0.016589	-1.36125	5.333454
FALEC	0.106687	0.016592	-3.28273	2.204325
SMYD2	0.106744	0.016609	-1.18177	4.963506
HIST2H2BE	0.1068	0.016626	-1.48756	4.85257
HOXB8	0.107111	0.016683	4.86841	1.007593
FOXL1	0.107278	0.016718	3.529486	1.831033
ZNF518B	0.107471	0.016757	-1.66831	4.783549
LY86	0.10751	0.016772	3.080246	2.077363
GLYCAM1	0.107683	0.016808	6.550535	0.430574
SHISAL1	0.10774	0.016825	1.514636	3.351962
MDGA1	0.107924	0.016863	-1.48413	4.464179
EPB41L2	0.108855	0.017017	2.141602	3.312575
ZNF541	0.108957	0.017042	-6.21238	0.605039
SDC4	0.109361	0.017114	-1.02023	8.062568
FAM25BP	0.10942	0.017132	-3.52762	2.185093
SH3RF3	0.109566	0.017164	1.481968	3.963671

MKRN3	0.109653	0.017186	-3.49867	2.119639
TSG101	0.109722	0.017206	-1.00199	5.839081
CASC15	0.109842	0.017233	2.709629	2.950348
CD200	0.109925	0.017255	6.523022	0.411222
NCLN	0.110062	0.017286	1.097758	6.069182
PRDX6	0.110064	0.017295	-0.974	7.227105
PLCB1	0.110265	0.017335	6.06955	0.978419
GIPC1	0.110284	0.017354	-3.38347	1.762756
FRAT2	0.110284	0.017356	1.152765	5.056294
RPL35	0.110302	0.017368	-0.98756	8.476164
SHROOM3	0.11041	0.0174	0.985491	8.288477
MGAT2	0.11041	0.017403	1.199256	5.065586
ATRNL1	0.110668	0.017456	2.460523	2.671218
LZTS3	0.110668	0.017462	1.816514	5.022711
LINC01695	0.110729	0.01748	3.584571	1.490541
MEPCE	0.11091	0.017518	1.563234	3.485065
CYSTM1	0.110938	0.017531	1.947365	6.123064
NTSR1	0.110957	0.017543	4.354687	0.986329
SNORA25	0.111209	0.017592	1.10212	6.046674
C5orf67	0.111479	0.017644	6.49499	0.391603
SLC17A5	0.111561	0.017666	1.200994	4.871097
SEMA4D	0.112001	0.017745	-1.06487	6.285795
USP31	0.112172	0.017781	-1.17538	6.200018
CD74	0.112228	0.017799	1.910923	2.745264
XKR4	0.112802	0.017899	2.749415	2.105928
GOLGA2P6	0.112996	0.017939	-7.10267	1.305615
RALGAPA2	0.11307	0.01796	0.997476	7.784311
MSI2	0.113159	0.017983	1.444382	3.892556
RRAGB	0.113479	0.018043	-6.1154	0.530785
UBA7	0.113491	0.018063	1.290237	5.431124
HIVEP2	0.113491	0.018063	-1.15415	7.345364
EML5	0.114055	0.018164	-1.53272	3.821493
MLEC	0.114055	0.018171	2.453684	2.079999
KSR1	0.114309	0.018221	-1.00726	6.251339
TNFRSF10D	0.114369	0.01824	1.763433	4.189465
TMEM163	0.114457	0.018263	3.826152	1.611735
PLXNA1	0.115258	0.0184	-0.97967	6.610128
HRNR	0.115374	0.018428	-1.66322	4.299235
NCKAP5	0.116019	0.018547	-1.18132	4.913433
RMND1	0.116019	0.01855	-4.28139	1.438815
TMEM119	0.116077	0.018569	2.808698	2.543821
NCK1	0.116525	0.018659	-6.44029	0.779055
EPS8L1	0.116525	0.018668	-3.30717	3.66297
LINC00266	0.116525	0.018672	-3.69728	1.27065
RBM20	0.116525	0.018688	-2.69831	4.985982

NAB2	0.116525	0.018703	-1.68871	5.171065
DBNDD1	0.116525	0.018714	-4.94368	1.248524
VPS37B	0.116525	0.018715	-1.02142	7.247983
RBM8A	0.116525	0.018716	-1.01988	7.343986
S100A8	0.116616	0.01874	-4.05084	11.80356
ARPC5L	0.116827	0.018784	-1.03794	5.956343
SH3GL3	0.117017	0.018824	-6.6033	0.911601
NAA25	0.117028	0.018835	-0.98316	6.785655
SPRR2C	0.117107	0.018857	-3.54321	4.579551
FAM206A	0.117275	0.018894	-1.37659	4.584524
CRLF3	0.11737	0.018922	-4.72483	1.81062
PLA2G3	0.11737	0.018928	-2.88551	3.81066
CSNK2A2	0.117513	0.01896	-0.96906	5.323394
FBLN1	0.117523	0.018972	1.333661	5.54824
DPT	0.117641	0.019002	1.901482	4.494601
LOC10192€	0.117641	0.01901	2.852164	3.385812
HLA-DRB5	0.118071	0.01909	-5.66076	2.796824
ALOX12B	0.118071	0.019104	-2.1756	3.993153
ZNF366	0.118071	0.019112	1.527035	3.448275
TLN1	0.118071	0.019118	0.917627	8.542311
KIAA1107	0.118071	0.019133	1.370045	3.577863
CPS1-IT1	0.118071	0.019138	3.900268	1.310447
SPTLC2	0.118071	0.019146	-1.00885	8.011746
ERICH4	0.118142	0.019167	4.627448	0.755491
C12orf75	0.118195	0.019185	1.492133	4.608217
CDPF1	0.118769	0.019288	1.798858	2.691191
MVB12B	0.118843	0.01931	1.685234	3.700845
RAB3D	0.118892	0.019327	-1.05314	7.846724
LINC01206	0.119116	0.019373	-3.72691	2.686242
ANKRD36	0.119163	0.019391	1.010438	6.951587
TTN-AS1	0.119199	0.019414	-2.77965	2.36466
PGA3	0.119199	0.019429	-11.9239	11.6838
COX7B	0.119199	0.019433	-0.9655	5.566666
GSTA1	0.119199	0.019435	2.137708	5.02065
PATJ	0.11921	0.019447	-0.96947	8.719026
LOC101927	0.119518	0.019512	-2.57048	3.781731
LINC01214	0.119518	0.019516	-6.7051	0.989484
TSHZ2	0.119576	0.019535	-1.94739	3.926723
MAPK15	0.119707	0.019567	-1.91855	4.05233
UNC13D	0.119724	0.019579	-1.14907	5.785889
DIO2	0.11981	0.019612	-2.65208	3.359944
FLG	0.11981	0.019613	-3.28901	9.51085
FOSL2	0.11985	0.019629	-0.97263	9.623455
ACAA2	0.119947	0.019654	0.939087	5.282353
CPT1A	0.120051	0.019685	0.923522	6.077067

LINC02544	0.120051	0.019691	3.764057	1.30173
PLAGL2	0.120074	0.019704	-0.97064	6.077533
SF3B3	0.120188	0.019738	-0.95726	7.881081
FBXW4P1	0.120188	0.019743	-1.33703	4.227835
C6orf141	0.120296	0.01977	1.48521	3.69856
PITPNA	0.120365	0.019791	-0.91834	7.224902
AJUBA	0.120881	0.019886	-3.60397	1.74242
OR7D2	0.121561	0.02001	-6.72433	0.99514
NRIP1	0.121561	0.020017	-1.12359	7.882291
GACAT2	0.121565	0.020028	4.408371	0.595322
SOCS5	0.122092	0.020124	4.646001	0.700002
PLEKHF1	0.1222	0.02017	-1.9242	2.732026
DOCK9-DT	0.1222	0.020172	-3.24291	2.36245
EHBP1	0.1222	0.020177	-6.10781	0.531487
TPM4	0.1222	0.020182	-1.07658	7.056204
SFMBT1	0.122418	0.020236	0.940483	5.869165
PORCN	0.122418	0.020238	-6.29277	0.665236
TMEM65	0.122652	0.020296	-1.14026	5.740845
SLK	0.122652	0.020296	-4.68143	1.356241
HIST1H1A	0.122769	0.020331	-1.63189	3.317759
HIST1H2AK	0.122769	0.020336	-2.46427	2.224733
EXOC3L4	0.122769	0.020345	2.85334	3.011677
E2F2	0.122837	0.020367	-1.93899	4.061807
RNASE7	0.122848	0.020378	-2.48082	3.799139
KRT76	0.12304	0.02042	-6.08763	0.512638
SLC16A3	0.123175	0.020453	-2.49273	3.134416
PROCR	0.123417	0.020505	1.663396	3.091275
NOS1AP	0.123417	0.020513	2.312375	2.493966
PNPLA2	0.123417	0.020523	0.900399	5.86525
HEXA	0.123965	0.020624	1.041447	5.447447
DCN	0.124048	0.020648	2.239886	3.890976
CLEC12A	0.124244	0.020691	5.076405	0.715531
ACRBP	0.124284	0.020707	2.256432	2.233325
KRT24	0.124692	0.020793	-3.50773	6.560841
ANKRD35	0.124692	0.020803	-3.35495	2.038819
CNIH1	0.124692	0.020806	-1.12183	5.400286
RP2	0.124755	0.020831	-1.23922	5.204406
PTGS1	0.124755	0.020836	-4.11916	1.305847
NIPAL2	0.124802	0.020854	-1.0417	6.430931
LINC00857	0.125112	0.020924	2.395963	1.663456
NAP1L3	0.125112	0.020926	3.166586	0.957085
GLUD1	0.125135	0.02094	-0.91939	7.511629
SH3BGRL3	0.12525	0.02097	-1.02763	7.631892
PSMB10	0.125397	0.021005	1.290441	4.21984
PDZK1P1	0.125704	0.021066	1.589261	3.340388

DLX5	0.125796	0.021092	-4.38352	1.526474
SPRR1B	0.125889	0.021126	-4.01362	9.306035
CTSE	0.125889	0.021128	3.908171	0.885995
NANS	0.126233	0.021196	0.880973	5.918234
HEG1	0.126895	0.021317	-1.28725	7.362878
C4orf19	0.127156	0.021371	4.066165	1.421123
KIF1B	0.127526	0.021444	-0.97758	8.061844
AKR1C2	0.127663	0.021477	-4.41271	1.050566
SPRR2A	0.127694	0.021503	-4.0068	9.521905
CPZ	0.127694	0.02151	-6.29476	0.665551
WDR66	0.127694	0.021513	-6.27098	0.649812
RP1	0.127966	0.021569	-7.18113	1.369551
CYP2J2	0.128114	0.021605	-1.44039	4.59555
PIP4K2B	0.128139	0.021619	1.64136	4.779747
KIAA1755	0.128184	0.021642	2.101031	2.5911
GIMAP7	0.128184	0.021655	1.815135	4.08679
MORC3	0.128184	0.021658	-1.13338	7.155267
ZBTB11	0.128411	0.021707	-0.92227	5.930841
CCKAR	0.128741	0.021773	-6.68531	3.135385
SCARNA2	0.128801	0.021794	1.112304	7.940297
UBE2E2	0.128883	0.021818	-1.90246	4.524017
LINC00365	0.129059	0.021858	3.604186	1.592606
LINC00989	0.129312	0.021912	4.995261	1.005267
LDLRAD4-A	0.129322	0.021924	2.838826	1.628636
ZNF445	0.129505	0.021965	1.004733	6.220308
DLEU2	0.129552	0.021986	1.459923	3.917728
UTP14C	0.129552	0.021994	0.87324	5.610377
DEFB1	0.13061	0.02219	-1.85581	3.960212
DLGAP4	0.13061	0.022195	1.179729	4.150127
NUP107	0.130708	0.022238	-1.17475	5.320283
LINC01003	0.130708	0.022242	-3.44899	1.647524
RAB25	0.130708	0.022243	-2.49418	2.559714
DNAJC1	0.130735	0.022259	0.87903	5.724003
LINC00896	0.130992	0.022313	2.648308	2.138668
CCDC82	0.131576	0.022423	0.89084	5.76483
CAT	0.131764	0.022466	0.986869	6.072628
C12orf54	0.132043	0.022524	-6.37019	0.731702
TKT	0.132132	0.02255	-2.61958	1.993513
PSMA7	0.132284	0.022587	-0.94224	6.555471
TEAD1	0.132491	0.022633	-0.96848	7.770008
PIP5K1C	0.132696	0.022682	2.1414	2.874633
EPGN	0.132696	0.0227	-3.72902	2.229386
TMEM223	0.132696	0.0227	1.624496	3.006929
GNG7	0.133389	0.022829	2.11635	3.813258
KLKB1	0.133764	0.022904	3.320641	1.111364

KRT17	0.134032	0.022961	-3.45434	8.915834
DNAJA1	0.134064	0.022977	1.367793	4.091741
LYNX1	0.134097	0.022994	-5.18568	1.144953
CHST7	0.134273	0.023035	2.35486	2.924782
CMTM3	0.134283	0.02305	-4.3721	0.474983
MAFF	0.134283	0.023059	-6.34702	0.703387
MIR3654	0.134554	0.023116	-1.11692	5.057921
SLC16A13	0.134782	0.023166	-1.80326	3.208718
ACP6	0.13481	0.023182	1.347057	4.237691
FOXF1	0.13485	0.023203	1.418532	4.447084
MMADHC	0.13485	0.02321	-0.99046	5.822488
MAFB	0.134955	0.023239	-0.99885	6.216807
RASSF2	0.135632	0.023367	2.86724	1.505605
MYORG	0.135797	0.023407	1.143612	5.893736
PARP6	0.135816	0.023426	-1.04028	5.763452
FAM126A	0.135816	0.023432	-1.02802	6.410941
SCGB1A1	0.136156	0.023501	-3.47836	2.649296
ADRB2	0.136201	0.02352	-2.04055	3.999108
MEGF9	0.136212	0.023533	-1.17475	6.696571
SNORD91A	0.136482	0.023599	-3.7663	1.434719
GALNT14	0.136482	0.02361	-6.31063	0.681034
TOR4A	0.136482	0.023624	1.069047	5.302266
UBE4A	0.136482	0.023633	5.922397	0.723148
CISD3	0.136482	0.023635	2.689108	1.853414
RGS2	0.136689	0.023682	1.566924	5.854874
KLK13	0.136909	0.023731	-4.00184	10.05032
MIR6739	0.136934	0.023749	3.069064	1.81621
MBD1	0.136934	0.023758	-4.0869	1.296815
LAPTM5	0.137023	0.023784	1.111586	5.546672
ABCA12	0.137325	0.023848	-6.10576	0.524706
DIPK2B	0.137428	0.023881	1.580985	4.045195
CCDC171	0.137428	0.023889	1.251737	4.299361
CGN	0.137428	0.023899	1.019953	7.620755
TENM2	0.137428	0.02391	-3.35834	2.009219
HNF1A-AS1	0.137585	0.023959	3.34358	4.737271
NPC1	0.137585	0.02396	-0.97685	6.286957
LRRFIP1	0.13778	0.024011	-1.32919	4.451564
CRYM-AS1	0.13778	0.024016	-2.47956	2.90317
GNA13	0.137819	0.024034	1.261231	3.960586
HSPA7	0.137842	0.024049	1.917097	3.909188
LHFPL3	0.137945	0.024078	4.025334	1.541106
PER2	0.138101	0.024132	-1.24887	7.170644
ARF5	0.138101	0.024133	-0.86099	6.023852
FBXL12	0.138101	0.02414	2.913236	1.278645
ZFHX4	0.138101	0.024157	1.149138	4.555052

BNIPL	0.138101	0.024163	-3.29582	1.988981
AMIGO1	0.138101	0.024175	1.928218	2.536905
GSTM5	0.138101	0.024184	3.700185	1.027097
EGLN1	0.138334	0.024251	-0.97635	6.853129
SPR	0.138334	0.024254	1.585144	3.903589
SNORD99	0.138334	0.024258	1.203509	5.776918
RAD51AP2	0.138647	0.024335	-6.35957	0.709093
GPR108	0.138647	0.024336	-1.01021	5.58394
DUSP1	0.138673	0.024351	-1.44068	8.699128
PGGHG	0.138683	0.024364	1.747935	4.923104
EPB41L4A	0.139249	0.024475	-0.95488	7.265716
DYM	0.139387	0.024511	-0.89453	5.763017
TIMP1	0.139748	0.024596	1.466869	4.822802
BAG2	0.139748	0.024597	1.996944	2.791356
VSIG8	0.139778	0.024613	-1.84067	3.292391
A4GNT	0.140004	0.024664	3.375903	1.650822
ANXA10	0.140412	0.024748	3.545887	5.799451
TTYH3	0.140458	0.024767	1.098315	5.674499
ARHGAP27	0.140537	0.024802	-3.19811	1.886904
IL1RAPL1	0.140537	0.024804	3.252326	1.320693
GAS6	0.140818	0.024865	1.083341	6.281745
LINC02363	0.140854	0.024883	3.879371	1.19758
ANXA5	0.140907	0.024904	0.877599	7.374963
INTS13	0.141123	0.024953	-1.02505	5.829662
ARHGDIB	0.141383	0.025019	1.063309	5.455896
ATP13A4-A	0.141383	0.025044	-4.19778	1.371071
PAPSS1	0.141383	0.025045	-0.84177	5.976024
LINC02516	0.141383	0.025048	-2.52755	2.434845
COX15	0.141383	0.025056	-1.5569	3.605023
RAF1	0.141785	0.025139	-0.88377	7.06413
RPS28	0.142325	0.025246	-1.17082	5.400917
RC3H2	0.142386	0.025269	-1.63247	3.96483
SH3RF2	0.142484	0.025298	-0.99582	6.907164
HOOK3	0.142644	0.025346	-0.96686	8.466339
SHANK2	0.142644	0.025354	1.243633	4.578749
KCND2	0.142644	0.025361	-4.23594	2.047045
ARHGAP26	0.142872	0.025413	2.339439	2.027641
SH3PXD2A	0.142959	0.02544	-0.93568	9.267991
ADIPOR2	0.143118	0.02548	-0.88491	7.106811
DCAF12	0.143252	0.025515	-0.97954	6.591924
SPTSSA	0.143315	0.025538	-1.1171	5.137455
EI24	0.143389	0.025563	3.885452	0.644172
LINC01978	0.143856	0.025658	2.285502	2.072467
ZNF93	0.143996	0.025694	-2.71045	2.336462
SDHAF3	0.144043	0.025714	1.607123	3.917342

P3H3	0.144046	0.025727	1.435986	3.591745
GPT2	0.144135	0.025754	-2.03351	3.645344
SPEG	0.144177	0.025773	1.474684	4.183712
ITGA1	0.144201	0.025789	1.47724	5.474789
GHITM	0.144461	0.025849	-0.8736	7.194582
LOC101928	0.144461	0.025859	2.483704	2.056961
CYP2U1	0.144986	0.025965	1.884469	3.590746
PHACTR4	0.145058	0.026007	-1.35719	3.954194
PAX8-AS1	0.145058	0.026012	-0.97176	7.003268
KDM2A	0.145058	0.026013	-2.05871	2.898978
SORBS3	0.14506	0.026025	1.608651	3.145115
TBKBP1	0.145149	0.026053	-1.19609	4.418823
RNU11	0.145824	0.026186	1.063463	9.224017
LINC01117	0.146386	0.026299	-6.30427	0.67063
SIGLEC15	0.146484	0.026338	3.595607	1.090868
AP4B1-AS1	0.146484	0.02634	2.937207	2.249533
RPL13	0.146634	0.026379	4.068535	1.637265
CADM4	0.146764	0.026415	-1.33125	4.696189
ABCC6	0.146764	0.026432	2.766195	1.955876
BDKRB1	0.146764	0.026438	-2.69162	2.338339
LOC101927	0.14697	0.026492	-2.19791	3.791603
SPRR2E	0.14697	0.026499	-3.8381	7.853072
SFT2D2	0.147112	0.026536	-1.24401	5.178301
GAL3ST1	0.14787	0.026692	3.371659	1.733051
TIMM10	0.14787	0.026697	-1.35054	4.131279
SUPT20H	0.147946	0.026727	1.915857	2.925819
RPTN	0.147946	0.026735	-3.48823	3.863873
CAPN14	0.147981	0.026753	-3.30341	9.437042
MOXD1	0.148092	0.026785	-2.26403	4.544351
GGACT	0.148291	0.026843	3.572514	0.758315
LINC01359	0.148291	0.026845	2.434392	1.974344
ABCA10	0.148497	0.026894	1.260693	4.600789
HS6ST1	0.148832	0.026967	-1.02116	5.514201
NPR1	0.148927	0.026997	1.960916	2.703895
IL36A	0.149717	0.027157	-3.08557	6.419807
PSAP	0.149717	0.027164	-6.03649	0.474813
EBP	0.149758	0.027183	-1.13672	5.632919
NANOS1	0.150221	0.027282	-3.54421	0.866222
NIPSNAP3A	0.150221	0.027292	-1.40185	4.504139
CPE	0.150578	0.027371	1.102259	4.806106
ITPKA	0.150578	0.027381	3.246003	2.424032
SLC9A2	0.150597	0.027397	2.263609	5.056841
BIRC3	0.150763	0.027439	1.985884	4.797422
GABARAPL	0.151129	0.027518	-0.90499	5.535213
TRIM31-AS	0.151169	0.027538	3.912286	1.457646

SMURF2	0.151505	0.027611	0.888822	5.553474
TMEM248	0.152074	0.027727	0.843666	6.877817
KRT16	0.152434	0.027805	-3.9814	10.08698
PDE5A	0.152644	0.027856	2.644343	2.146673
SPON2	0.152822	0.027904	2.468414	2.032596
ERCC6	0.152822	0.027913	-0.93334	5.560573
KRT42P	0.153032	0.027964	-6.19087	0.58776
SMARCA2	0.1532	0.028007	2.619718	1.744123
MSH4	0.153277	0.028033	3.982226	1.518374
PLEKHS1	0.153573	0.0281	3.664828	1.486511
HCG26	0.153594	0.028116	1.987172	3.368361
LOC727896	0.153812	0.028184	1.636772	4.031655
ABHD4	0.153812	0.028193	-1.01933	5.472529
TPM1	0.153812	0.028193	3.002371	2.760753
HOXB7	0.154077	0.028255	2.00872	4.192975
TNKS2	0.154227	0.028295	-0.93369	7.585237
LINC01730	0.154339	0.028328	3.683977	0.699168
BDH2	0.154438	0.028358	1.05731	4.868518
LYPLA2	0.154668	0.028426	-1.05248	5.297139
HMCN1	0.154668	0.028432	1.345645	6.34631
SLC7A4	0.154668	0.028446	-3.22676	1.804055
MIR454	0.154668	0.028451	4.180405	1.784567
UBE2R2	0.154915	0.028509	-0.9647	7.015743
MUC6	0.155286	0.028589	3.237004	9.368423
FKBP14	0.155384	0.02862	-6.31999	0.683144
SERPINB3	0.155659	0.028695	-3.94433	10.53799
HSPA2	0.155659	0.028701	-1.13353	5.77404
MDFIC	0.155659	0.028709	-6.6198	0.917947
HLA-DRB4	0.156085	0.0288	4.529617	1.940538
ANXA8L1	0.156326	0.028857	-4.41771	1.147077
NDUFS8	0.156356	0.028875	-1.18674	4.360297
FAM155A	0.156779	0.028966	2.762959	2.383356
NPY6R	0.156779	0.028979	1.982857	2.793151
LOC283922	0.156779	0.028991	1.030404	5.270893
LINC00707	0.156806	0.029009	-6.26385	0.647849
FAM27E2	0.157025	0.029063	2.664893	1.954936
FAM166A	0.157025	0.029075	-6.18873	0.583272
SORL1	0.157069	0.029096	0.856036	7.490451
KRR1	0.157294	0.029162	-1.03195	5.420834
PLXNC1	0.157294	0.029163	1.716029	3.385104
CERS3	0.157679	0.029247	-4.31617	0.48659
PLEKHA1	0.158062	0.029338	-4.16682	1.388488
SERPINB2	0.158062	0.029344	-2.89273	2.63788
BICD2	0.158094	0.029362	-1.70999	5.27206
TRPV2	0.15838	0.029429	1.68255	2.898681

C3P1	0.158539	0.029474	3.5777	1.909804
CT83	0.158539	0.029484	4.513658	2.376172
MYCBP2	0.159015	0.029585	-0.91398	9.108412
NRADDP	0.159171	0.029627	-4.33366	1.080001
LINC00173	0.159213	0.029648	-3.61239	1.203121
C9orf16	0.159225	0.029663	-1.40556	4.245546
ZBED5-AS1	0.159332	0.0297	-2.58337	1.842287
ACPP	0.159332	0.029709	-2.01184	3.751612
UTP11	0.159544	0.029761	-1.01812	5.099975
NREP	0.159605	0.029785	2.804344	1.120274
WDR45B	0.159716	0.029821	-1.07771	7.310723
NUP188	0.159716	0.029832	-0.87922	6.860296
SLIT1	0.15989	0.029888	2.785006	2.021512
HSD3BP4	0.15989	0.02989	2.102094	2.967449
TSPAN18	0.159928	0.02991	1.41783	3.661296
OR2V2	0.159953	0.029928	5.089572	0.683165
RAI1	0.160007	0.029951	-0.96961	6.604564
KRT5	0.160257	0.030011	-4.04055	12.42845
IL36G	0.160597	0.030088	-6.06026	0.498707
NHP2	0.160723	0.030124	-2.75043	1.812039
MAP3K1	0.160861	0.030163	-0.88528	7.160158
MIR548O	0.161052	0.030212	-5.99587	0.45608
ECSIT	0.161062	0.030227	-5.30685	1.769737
LINC00265	0.16108	0.030244	-1.19831	4.533344
TWIST1	0.16108	0.030256	2.174489	2.780129
ADCK5	0.161987	0.030449	1.28446	3.752203
HCAR3	0.161987	0.030463	-3.03943	3.455708
PDE12	0.161987	0.030471	-0.94124	5.622666
MARS	0.161987	0.030479	-0.98983	6.157554
MUCL3	0.162069	0.030508	2.023763	6.04652
XPO5	0.162322	0.030568	-0.98202	6.276217
GALNT1	0.16235	0.030587	-1.01385	7.97742
TNS3	0.162666	0.03066	1.402459	6.756815
SMA4	0.162667	0.030673	0.972372	5.036375
SPINK7	0.162845	0.03072	-2.58214	5.748959
S1PR1	0.162848	0.030733	1.329386	4.166058
CRNN	0.163043	0.030783	-4.18112	12.22206
ABCG2	0.163194	0.030825	3.575163	1.35678
OSBPL1A	0.163285	0.030856	1.326247	3.729466
SEMA3C	0.163499	0.030909	1.213885	5.450274
CA12	0.163582	0.030944	-6.16948	0.575656
TBL3	0.163582	0.030952	-1.05171	4.501906
TMEM41A	0.163598	0.030968	-1.03306	5.088678
PPAN	0.163622	0.030986	-4.49608	0.612947
SRSF6	0.163631	0.031001	1.720429	3.048295

ERP29	0.163758	0.031038	2.450227	1.830205
SIGLEC8	0.164202	0.031135	4.306551	0.745085
AQR	0.164387	0.031184	-0.87275	6.652527
NARS2	0.164565	0.031231	2.418447	1.968194
PPP4R1L	0.164722	0.031274	1.195779	4.25046
MIR4712	0.164725	0.031298	-6.78184	1.046279
KLHDC8B	0.164725	0.031301	-1.75913	4.109205
RASAL3	0.165114	0.031388	1.592542	3.545296
WDR6	0.165142	0.031407	0.921803	6.571677
PRDX2	0.165427	0.031475	-0.90508	6.117718
MMP25	0.165563	0.031521	2.065607	2.488219
LOC10012[illegible]	0.165563	0.031536	4.725749	1.05363
LINC00476	0.165563	0.031541	1.508343	3.637506
FRMD6-AS[illegible]	0.165602	0.031562	-2.00563	3.526106
LOC25739[illegible]	0.165657	0.031586	4.646473	1.022294
PREX2	0.165713	0.03161	1.810345	3.663342
SH3KBP1	0.165893	0.031657	2.020856	2.29104
BBC3	0.165946	0.031681	3.999864	0.92712
PIWIL4	0.165946	0.031711	2.307207	2.510639
TAF9B	0.165946	0.031721	-1.16268	5.076693
RNF181	0.165946	0.031721	-1.93904	3.703505
FICD	0.16629	0.031801	1.893248	2.708714
ZNF345	0.166333	0.031822	-3.07094	2.119533
SLC25A23	0.166363	0.031842	-0.93579	6.906671
PIP4K2A	0.166543	0.031901	0.901393	5.373721
ADAMTS1[illegible]	0.166543	0.03191	2.482251	2.877067
EIF4A3	0.166543	0.031924	-0.97765	6.128678
LOC28304[illegible]	0.166543	0.03193	-2.93397	3.657255
IGFBP4	0.166543	0.031943	1.19169	7.01348
KPRP	0.166757	0.031998	-2.9403	4.862303
8-Mar	0.166787	0.032017	2.341664	2.529799
LINC02538	0.166874	0.032048	-3.29445	3.722804
FPR3	0.166967	0.03208	2.351428	3.251143
DKK4	0.166967	0.032092	-3.6942	1.895281
HLA-DOA	0.167183	0.032152	4.427851	1.700389
ANXA2	0.167183	0.032171	-1.98897	3.912023
TXNRD1	0.167183	0.032216	3.823429	0.768103
NBAT1	0.167183	0.03222	2.674135	2.603647
TUBA3D	0.167183	0.032222	-3.37477	1.452706
FUT1	0.167183	0.032228	-1.89819	3.283819
USP53	0.167183	0.032229	-0.92167	7.611761
CNPY3	0.167338	0.032276	1.221066	5.268199
CNNM2	0.167338	0.032286	3.648448	1.170954
FGD1	0.167338	0.032299	-1.25366	4.183058
EGFR-AS1	0.167357	0.032317	-1.49906	3.825596

MYADM	0.167427	0.032344	3.552342	3.092568
SLCO1B3	0.167519	0.032375	4.307374	1.613661
PHF14	0.167883	0.032459	0.891253	6.333951
DPP8	0.168506	0.032593	3.380062	0.649672
FGFR2	0.168808	0.032676	4.018342	0.765853
IGFLR1	0.168808	0.032679	2.225904	1.888619
KRT4	0.168808	0.0327	-4.2493	14.75006
KRT6B	0.168808	0.032706	-3.82289	10.26581
ZNF462	0.168815	0.032721	-0.94607	7.365827
GNPNAT1	0.168899	0.032751	0.934014	5.642206
PCDHGC4	0.16949	0.032879	-5.96807	0.43233
HIRIP3	0.16977	0.032947	1.228806	4.552014
LOC14366(	0.169858	0.032978	-2.50577	2.470306
TNFAIP1	0.169927	0.033005	-0.91433	5.882872
PSMD7	0.170231	0.033089	-0.83348	7.104422
CRAT37	0.170231	0.033092	3.965512	1.170551
GLO1	0.170363	0.033156	-1.07679	6.371452
FLJ46906	0.170363	0.033165	-1.85987	3.499793
RTL5	0.170363	0.033173	1.547785	3.563049
CHMP1B	0.170363	0.033177	-0.99683	6.670995
AFDN-DT	0.170363	0.033196	-2.24128	2.528268
LOC10013(	0.170363	0.0332	-1.43147	3.776209
TREX2	0.170659	0.033279	-6.03543	0.482119
HNMT	0.170659	0.033286	2.001453	3.565842
ALDH4A1	0.170891	0.033345	-6.14243	0.55433
GOLGA2	0.170964	0.033373	-0.8799	8.328119
ABCB6	0.171018	0.033397	-1.06738	4.686062
UBE2U	0.171056	0.03344	3.604493	1.055152
IFT140	0.171056	0.033441	0.960713	5.666006
FGF10	0.171056	0.033456	3.952242	1.059218
FAM220A	0.171056	0.03346	-2.14195	3.793893
CD55	0.171349	0.03356	-2.85911	3.760003
IPMK	0.171349	0.033572	-1.43458	5.589258
ATP8B1	0.171349	0.033578	0.845567	7.950347
EIF1AX	0.171349	0.033581	-1.01719	5.716092
PPP1R36	0.171349	0.033591	2.927148	2.391192
IZUMO1	0.171349	0.033617	-3.3895	1.410819
SH3BP4	0.171349	0.033623	1.129815	5.804637
ANXA2P1	0.171349	0.033629	1.56196	3.195922
ANO9	0.171442	0.033661	0.827863	5.389237
GPR84	0.171742	0.033734	-6.14307	0.549421
DCTN1-AS1	0.171861	0.033771	4.632834	0.904401
PPP2CA	0.172572	0.033925	-0.92661	7.327598
LOC28445⁄	0.172742	0.033972	-1.75414	5.872707
HLA-DMB	0.173332	0.034102	3.915171	1.373792

FAM186B	0.173941	0.034236	-4.12626	1.36417
EAF1	0.17403	0.034267	-0.88542	5.646931
RASD2	0.174093	0.034298	2.543677	1.690834
3-Mar	0.174093	0.034308	1.80984	2.943763
ARHGAP29	0.17478	0.034458	1.079739	5.395626
KIFC2	0.17482	0.034488	-0.88702	6.001636
ZNF296	0.17482	0.034494	-2.68074	2.501289
AIDA	0.174831	0.034524	1.142287	4.755909
ZNF536	0.174831	0.034524	2.433263	2.936977
ZNF654	0.174847	0.034547	-1.09921	6.098374
HLA-B	0.174847	0.034556	1.149675	8.043531
PLXNA3	0.174858	0.034572	0.870548	6.055113
ADAMTS7	0.175101	0.034644	1.684216	3.262235
BCL10	0.175101	0.034649	-0.96707	5.568862
FUT6	0.175263	0.034695	-2.58519	2.520315
DDX60L	0.175317	0.03474	1.340605	3.831906
HRH1	0.175317	0.034747	-3.96604	1.480373
NRBP1	0.175317	0.034756	-0.9082	7.59562
C9orf152	0.175317	0.034768	1.830083	4.962454
TBC1D8B	0.175317	0.034777	1.0228	4.164502
ESPN	0.17534	0.034795	-1.09067	5.155735
SKOR1	0.175459	0.034844	3.354653	0.899168
CTSK	0.175459	0.034847	1.443984	4.578817
PRKG1	0.175813	0.034932	2.125477	2.936019
LOC100507	0.176322	0.035057	3.75078	0.988485
CATSPERB	0.176322	0.035062	3.404001	1.563076
CABLES1	0.176547	0.035132	1.989814	2.654705
DLGAP1-AS	0.176547	0.035142	2.138654	2.405605
TSR1	0.176547	0.035149	-0.90728	6.334926
AGAP2	0.176587	0.035171	4.027021	0.728786
EIF3H	0.176988	0.035266	-0.89879	7.557302
MAF	0.177065	0.035295	-1.05738	5.981566
ANKRD20A	0.177133	0.035343	-3.88439	2.055408
BMT2	0.177133	0.035348	-1.30728	5.010361
C1orf159	0.177133	0.035352	-1.87842	3.682426
PACS1	0.177157	0.035383	-0.82997	6.515177
FSIP1	0.177157	0.035385	2.680148	1.539627
CAPN9	0.177181	0.035416	4.215062	0.939133
CKAP4	0.177181	0.035419	-0.84395	7.269098
KLHDC10	0.177192	0.035442	-0.86237	6.284248
UNC93B1	0.177192	0.03545	0.834537	5.435673
LINC02362	0.177195	0.035465	4.642054	0.782856
FMN1	0.17773	0.035586	1.063738	4.523294
CFB	0.177903	0.035641	2.181811	1.953364
OXCT1	0.177903	0.035655	-0.92451	6.320846

NYNRIN	0.177903	0.035664	-0.82144	5.910531
FNDC3A	0.178018	0.035702	1.989026	2.732257
LINC00311	0.178185	0.03575	4.026735	1.082554
LOC10193(	0.17831	0.035789	2.041613	2.301683
TNRC18	0.178343	0.035824	0.824834	8.281928
SOCS7	0.178343	0.035831	-3.11102	3.057033
LTBR	0.178343	0.035839	-1.29892	4.214762
NAA15	0.178709	0.035927	-0.92187	7.053991
SEC24D	0.17881	0.035962	0.822562	5.700771
SH3RF1	0.178955	0.036006	0.922589	6.074422
ARHGAP42	0.179125	0.036054	1.324457	4.347465
RAC1	0.17918	0.03608	-2.95735	2.147631
MYO1F	0.179288	0.036116	1.290615	4.771191
BMP2K	0.179512	0.036176	-1.23089	5.242668
LNX2	0.179711	0.03624	-0.91101	6.296146
WDR27	0.179711	0.036245	-2.01773	3.838322
LINC02595	0.18009	0.036336	-2.13946	3.060294
RNF10	0.18037	0.036418	-0.82278	7.752518
LINC01637	0.18037	0.036422	2.659498	1.793264
CLCA4	0.180421	0.036447	-3.02203	2.33757
PDE4D	0.180852	0.036559	1.341337	4.724322
BTN2A3P	0.180852	0.036563	1.847764	3.026511
METTL2B	0.180897	0.036587	-1.03669	4.414689
LOC65316(	0.181432	0.03671	1.614262	2.69349
TOPORS	0.18158	0.036758	3.275981	1.321835
PBDC1	0.18158	0.036769	-6.27102	0.655554
PLXNB1	0.181585	0.036785	1.620816	3.606659
MIR938	0.181736	0.03683	-6.01183	0.458942
LINC01629	0.181738	0.036856	-6.03191	0.47117
NDUFS4	0.181738	0.036867	-1.00402	5.645596
ADORA2B	0.181738	0.036875	-1.75562	3.435242
TNKS1BP1	0.181799	0.036902	-0.84529	9.246757
KATNBL1P(	0.181996	0.036956	-2.61093	1.939171
TBC1D22A	0.182213	0.037015	1.417919	3.25499
QKI	0.18223	0.03704	-2.91622	2.349799
PP7080	0.18223	0.037048	1.28533	6.10023
SMIM5	0.182408	0.037099	-0.87992	5.510703
LOC40094(	0.182741	0.037182	-6.823	1.076325
TNFRSF17	0.183043	0.037271	2.779214	2.154369
IL22RA1	0.183043	0.037273	-1.27643	5.141137
ADI1	0.183597	0.037407	1.436753	3.819147
HS6ST3	0.183597	0.037415	-6.93182	1.158816
RAC2	0.183666	0.037459	1.356668	3.435586
ZBTB45	0.183666	0.037459	3.614931	0.827573
H1FX	0.18372	0.037485	0.87527	6.12216

NEU3	0.183747	0.037505	1.15626	4.179587
ASAP2	0.183793	0.03753	2.035136	2.192183
ZNF37BP	0.183868	0.03756	0.929328	5.817963
RAB20	0.184055	0.037613	2.184199	3.894475
CCDC159	0.18418	0.037654	1.070594	4.018388
SUCLA2	0.184195	0.037671	-0.8849	5.531051
LINC00536	0.184257	0.037705	-5.99179	0.443865
WFDC21P	0.184257	0.037714	-2.58928	4.661845
SRF	0.184984	0.037878	1.16065	4.813522
DEK	0.185311	0.03796	2.623134	1.710701
LOC101928	0.18536	0.03801	2.777333	2.340613
MROH2B	0.18536	0.038014	3.602445	1.120624
IMPAD1	0.18536	0.038015	-0.84664	7.205311
HIF1A	0.185443	0.038047	-1.97094	4.715451
CCDC200	0.185443	0.038062	2.165902	2.147608
SERPINB8	0.185528	0.038104	-3.52888	1.752972
GDF11	0.185528	0.038109	1.253735	3.753757
BCL3	0.185764	0.038181	-1.10847	5.337368
PYROXD2	0.185764	0.038188	1.384243	3.265201
ARL13A	0.185866	0.03823	3.918494	1.439245
GOLGA4	0.185866	0.038239	-3.30474	1.197984
SULT1C2P1	0.185919	0.038265	3.426968	1.066799
ZNF107	0.186133	0.038324	-6.12813	0.541193
NFKBIA	0.186363	0.038396	-0.94583	7.432613
LOC100506	0.186363	0.038402	3.928781	0.518178
PMAIP1	0.186442	0.038443	-1.95772	4.590316
RGL4	0.186442	0.038448	0.98329	5.08432
EHF	0.186453	0.038466	-2.91986	2.94835
MOCOS	0.186581	0.038524	-1.29921	3.843946
LOC100233	0.186581	0.038527	-4.56263	0.969593
SLC25A34	0.186581	0.038537	1.916869	2.275393
MRPS17	0.186701	0.038577	-1.74382	3.902485
COBL	0.186776	0.038608	1.226916	3.935483
CABP7	0.186784	0.038637	3.989698	0.459904
RPL4	0.186784	0.038647	-0.83684	9.794299
TNS2	0.186784	0.038655	2.348201	1.57181
IFIT2	0.186821	0.038691	2.370158	2.911159
POMP	0.186821	0.038714	-0.8352	5.593611
LOC441081	0.186821	0.038724	1.188163	4.341377
ID4	0.186821	0.038746	-1.95329	4.912574
RSPH3	0.186821	0.038753	-0.83055	5.598641
PSME2	0.186821	0.038753	0.820452	5.616462
LBH	0.186867	0.038778	1.478571	4.187623
HPS5	0.186926	0.038805	-6.84155	1.08962
NDUFV2	0.187119	0.038861	-0.87982	6.31649

ARMC9	0.187426	0.038944	2.08503	2.968713
ESRP2	0.187426	0.038957	-0.92071	7.397999
ID1	0.187426	0.038981	-6.04035	0.479763
ASF1B	0.187426	0.038985	1.730869	2.652232
SLC7A8	0.187909	0.039101	1.301516	5.06493
USP15	0.188111	0.039158	-1.25507	4.294289
CAPN6	0.188625	0.039292	1.50338	3.471951
CRYBG3	0.188625	0.039296	-0.9375	8.288673
PARVB	0.188933	0.039375	2.890023	1.259677
ARL5B	0.189475	0.039503	-1.21906	5.508428
SNORA38B	0.189556	0.03954	1.716924	4.923178
GAS6-AS1	0.189556	0.039551	1.459636	3.041592
MIR34AHG	0.18986	0.03963	0.812655	5.439233
MIEN1	0.189872	0.039659	-1.01455	4.62721
ATP5PO	0.189872	0.039667	-0.9358	6.304815
PLK3	0.189872	0.039678	-1.48068	5.540429
ALS2CL	0.190367	0.039797	-2.1373	3.650184
FAIM2	0.190436	0.039841	2.612902	1.671805
COPS6	0.190436	0.039842	-0.84838	6.222207
DLEU7-AS1	0.190539	0.03988	3.615486	1.372015
LINC02532	0.190671	0.039923	4.531096	0.692095
SMAGP	0.190759	0.039957	-4.13297	0.613313
GATA6	0.190989	0.04002	2.679041	5.414049
FZD4	0.191394	0.040121	1.331245	4.950776
PPP1CB	0.19142	0.040141	-1.7717	2.624348
VMA21	0.191999	0.040278	-0.88872	5.493468
MYO3A	0.191999	0.040294	-3.11244	1.568309
KATNAL2	0.192638	0.040444	1.3562	3.535846
ZNF398	0.193011	0.040538	-2.43359	2.25114
KCNA2	0.193112	0.040574	3.609995	1.239833
LINC01271	0.193131	0.040594	-6.0652	0.495747
EXT1	0.19315	0.040614	0.892636	6.362118
COMMD1	0.193218	0.040644	-1.14612	4.245027
COLEC12	0.193229	0.040662	1.587114	3.408573
CKB	0.193243	0.040698	-2.04406	5.905041
MIR222	0.193243	0.040705	-5.92448	0.402913
MRPL13	0.193243	0.040712	-1.56366	3.945567
NHLRC1	0.193258	0.040756	2.920923	1.716632
KRT6A	0.193258	0.040767	-3.87273	13.21807
TMEM33	0.193258	0.040768	-0.90192	6.807524
RNF220	0.193258	0.040777	0.877061	5.254513
ZNF208	0.194349	0.041032	2.083923	3.112922
WDR18	0.194349	0.041039	-1.1772	4.195791
TELO2	0.194429	0.041075	0.957313	4.829753
TRPS1	0.194429	0.041099	-3.41837	1.347045

NSUN5P2	0.194429	0.041103	2.277873	1.696458
APOBEC4	0.194447	0.041137	2.393564	2.03951
RAB8B	0.194447	0.041148	0.926654	5.456594
SELENOI	0.194447	0.041154	-0.88693	7.136888
DPYSL4	0.194594	0.041201	3.464254	1.297753
FMNL2	0.194789	0.041258	0.876815	6.38891
INAFM2	0.195251	0.041372	-1.45279	4.292187
SMCO2	0.195313	0.041401	-2.62118	2.142595
CXADRP2	0.196394	0.041656	3.694485	1.369085
DCAKD	0.196394	0.041662	2.449161	2.123166
C2orf70	0.196507	0.041702	4.148981	0.877984
YWHAH	0.196544	0.041725	0.773243	6.663737
PAXBP1-AS	0.196708	0.041789	1.122422	3.926186
CCAT1	0.196708	0.041792	-1.48071	5.09077
ALX4	0.196985	0.041868	3.241202	0.881462
TMEM256	0.196985	0.041883	-1.2096	4.38749
SNORD114	0.19699	0.041915	2.21401	1.678762
MSX2	0.19699	0.041916	2.771088	1.396754
COX6C	0.197004	0.041935	-0.92067	6.525131
MIR548AQ	0.197254	0.042004	4.075694	0.918053
ADCY9	0.197478	0.042068	0.851481	5.948824
BEND3	0.197609	0.042112	1.155455	3.933697
FLNB	0.197698	0.04215	-2.68513	2.038612
IL17RD	0.197698	0.042206	-1.78838	3.566393
DICER1	0.197698	0.042207	-1.91819	2.824278
SCARNA17	0.197698	0.04221	0.858558	7.045394
MIR4505	0.197698	0.042222	-3.91748	0.75644
CELF2-AS1	0.197698	0.042227	1.867043	3.680924
CLDN34	0.198077	0.042324	-6.47397	0.796506
MLPH	0.198121	0.042356	3.423333	1.295098
BATF	0.198121	0.042365	2.386826	2.020663
VGLL3	0.198496	0.042488	1.415014	3.713176
S100A7	0.198496	0.042488	-3.99848	7.772407
SHC3	0.198496	0.042503	1.406056	4.242442
KLF9	0.198496	0.042509	1.020345	6.685979
PROB1	0.198745	0.042579	1.928724	2.529906
APOB	0.198803	0.042607	2.54042	3.639639
LOC105372	0.198851	0.042634	-6.08236	0.505789
DNAH12	0.198867	0.042667	3.022582	1.168001
LOC101927	0.198867	0.04267	-5.97309	0.432702
B3GALNT2	0.198908	0.042695	-0.90686	5.353322
TBL2	0.198911	0.042712	0.827912	5.190369
TBC1D1	0.198911	0.042737	2.586612	1.885001
ARHGAP1	0.198911	0.042746	-0.87039	7.21734
CCR10	0.198911	0.04276	3.078705	1.247466

SP2	0.198978	0.042795	-1.0767	5.297203
EIF2S1	0.198978	0.042806	-0.81289	6.522877
LRRC18	0.199119	0.042853	3.994896	0.91417
MIR3188	0.199273	0.042906	4.117521	1.541368
RPS12	0.199273	0.042918	-0.84171	8.42814
RBP1	0.199476	0.04298	-2.12286	3.256645
ZNF793	0.199476	0.042994	-1.90818	3.591838
KIAA2012	0.199512	0.043018	3.844879	0.507915
PCF11-AS1	0.20018	0.043178	-3.92861	1.14971
RRP1B	0.200569	0.043278	-0.77953	6.007577
ALOX12-AS	0.2006	0.043301	-4.39475	1.022838
ME3	0.200706	0.043341	4.576319	0.668171
AMFR	0.201057	0.043433	-0.80989	6.747339
NOP14-AS1	0.201548	0.043561	-1.34778	3.525776
SKIDA1	0.201548	0.043594	-3.03087	1.50396
ELMO1	0.201548	0.043604	2.282794	2.166334
PITHD1	0.201548	0.043604	-1.01751	6.341257
CCNB1IP1	0.201739	0.043677	-4.62788	0.705508
PCBP1	0.201739	0.043678	-0.84421	7.675927
PIK3R1	0.201919	0.043746	0.884677	5.685007
BCDIN3D	0.201919	0.04375	-2.08854	2.248259
ABCD2	0.202275	0.043847	3.461931	1.252345
PPP2CB	0.202275	0.04386	-0.90431	6.585115
TYMSOS	0.202326	0.043887	3.826038	0.991074
SHANK3	0.202562	0.043971	0.887716	5.851638
TONSL-AS1	0.202562	0.043986	1.855002	2.182314
TMEM144	0.202562	0.043987	0.959745	4.601923
LACTB2	0.20283	0.044062	1.182426	3.720724
RNF144B	0.204147	0.044365	-1.80993	6.001402
LHCGR	0.20418	0.044388	4.365371	1.04821
NIN	0.204294	0.04443	-1.056	4.765915
MIA3	0.204826	0.044564	0.761385	6.969043
WDR3	0.204826	0.044579	-0.86014	6.084524
VAMP1	0.204948	0.044622	2.862359	1.630077
GPR149	0.205227	0.044699	5.153467	0.799237
RRP7A	0.20523	0.044728	-0.92686	4.945804
CACNA1C-A	0.20523	0.044748	3.312625	0.860307
SFTA2	0.20523	0.04475	-3.28581	1.378122
NR4A3	0.205543	0.044835	-6.88245	1.125143
SLFN5	0.205583	0.04486	0.913305	6.209026
LOC100132	0.205618	0.044884	2.479519	1.701986
MTA3	0.205654	0.044909	2.155006	2.137849
IKBIP	0.206004	0.045002	2.648315	2.078269
PRKXP1	0.206163	0.045075	-0.96313	4.699686
PANDAR	0.206163	0.045082	2.195099	1.816686

KRT86	0.206163	0.045087	3.396417	0.986852
OSBPL2	0.206222	0.045116	-4.32657	0.781767
PTGDR2	0.206457	0.045205	2.27181	3.088782
STARD10	0.206457	0.045207	1.150452	6.478484
PPP4R1-AS	0.206457	0.045218	-4.47596	0.906626
CH25H	0.20747	0.045456	-3.16288	1.518664
TGS1	0.207552	0.045514	2.329983	1.374476
LOC10013(	0.207552	0.045523	2.476699	2.035486
NEK5	0.207552	0.045525	2.382015	1.49227
KCNQ1OT1	0.207784	0.045593	0.941875	9.234919
RNLS	0.208182	0.045697	2.13996	1.763906
SYNPO2	0.208527	0.045789	1.514677	6.633871
FHOD1	0.208619	0.04584	1.117369	4.418054
TTC39A	0.208619	0.045854	-3.4112	1.623329
GIMAP1	0.208619	0.04587	2.836537	2.037719
FOXI2	0.208619	0.045877	-3.84188	1.28588
DHRS9	0.208728	0.045918	-2.58338	3.337119
ZNF365	0.20873	0.045936	-2.87154	4.946344
TMEM265	0.208839	0.045993	-1.14735	4.513834
PIGB	0.208839	0.045993	1.416148	4.023462
FGD5	0.208839	0.04601	1.278122	4.925086
FHL1	0.209006	0.046064	3.176607	1.629307
SLCO2A1	0.209103	0.046102	1.186144	4.891899
SASH3	0.209362	0.046176	1.315473	3.484452
MT1E	0.209498	0.046223	1.90982	5.361414
HMCN2	0.20962	0.046267	1.772996	4.879952
ZCCHC24	0.209721	0.046306	1.155627	4.584812
MAP3K7CL	0.209844	0.046351	-4.34948	0.99375
RB1	0.209889	0.046378	-0.84837	7.142953
FAM50A	0.209895	0.046396	0.856856	6.311979
MIR635	0.210019	0.04644	3.306617	1.564239
TMEM238l	0.21005	0.046467	1.855875	3.556289
ZDHHC23	0.21005	0.046481	1.406805	4.275232
PRRT1	0.210408	0.046577	2.832619	0.919282
CLUH	0.210461	0.046606	-0.80254	7.173183
KDELR3	0.210505	0.046642	1.590357	3.665936
MIR4697H(	0.210505	0.046666	1.497535	3.536864
NUDCD2	0.210505	0.046673	-1.38505	3.663555
GUSBP9	0.210505	0.046684	2.352955	2.816276
GDF15	0.210584	0.046719	1.90132	3.423238
DEPP1	0.210669	0.046755	1.368435	4.896318
TAF1B	0.210783	0.046797	-1.01157	4.440247
TMC8	0.211186	0.046904	1.378939	4.143906
ASB16	0.211201	0.046924	1.440941	3.388098
ZNF681	0.211222	0.046946	-1.84179	3.764279

CGAS	0.211668	0.047074	1.419357	3.037333
EPS15L1	0.211668	0.047093	-1.60728	3.805481
ZNF440	0.211668	0.047096	-1.1497	5.391102
LDLRAD2	0.211758	0.047146	2.013567	2.361233
PLEKHM2	0.211758	0.047153	-0.91207	6.629367
SYNRG	0.211758	0.047168	4.176171	0.994219
HPCAL1	0.211855	0.047217	2.19852	3.302976
MAPK1	0.211855	0.047231	-0.8107	6.871612
DTHD1	0.211855	0.047241	2.703746	2.190901
TTC32	0.212387	0.047377	-2.93792	1.872484
CLEC3B	0.212502	0.04742	2.472324	1.9201
MARVELD1	0.212832	0.047511	1.133143	4.463022
IGFBP7	0.213329	0.047639	1.865291	2.342173
IL18R1	0.214256	0.047863	3.403932	1.625527
OVOL2	0.214821	0.048007	2.442523	1.882699
ADCY7	0.214958	0.048055	-0.872	6.05861
SNORA80E	0.214977	0.048076	1.028789	7.737137
LARGE-AS1	0.215011	0.04813	1.717756	2.763518
RPL23AP64	0.215011	0.048131	-2.55305	2.535653
TMPRSS13	0.215011	0.048136	-3.01215	2.475605
CEMIP2	0.215388	0.048238	2.42695	1.516022
OSGIN1	0.215902	0.048371	-1.58336	2.850633
ATP6V0A2	0.216022	0.048458	0.957765	5.841317
CINP	0.216022	0.048463	-1.26198	3.430754
ZFP69	0.216022	0.048466	1.428268	3.249721
ARHGEF3	0.216022	0.048468	-1.49134	3.166768
ARMC1	0.216081	0.048498	-2.848	1.730978
KCNK3	0.216155	0.048533	2.12813	2.202784
UGT2B17	0.216654	0.048662	3.823429	1.516199
RELL1	0.216707	0.048691	0.93831	4.760644
BMS1P22	0.216963	0.048781	3.348883	1.188641
AATBC	0.216963	0.048787	-1.8899	3.128365
SLC27A1	0.216963	0.048802	1.048613	4.615784
ITPR2	0.217449	0.048944	-0.82705	8.662243
ITPR1	0.217449	0.048958	4.116124	1.120164
MYH8	0.217449	0.048964	3.405178	0.769067
DENND5B	0.217794	0.049059	3.148257	1.398691
CYB5RL	0.217826	0.049084	1.538182	2.857193
MIR1255B2	0.217835	0.049104	3.894236	0.963781
CETN2	0.21799	0.049156	-1.25548	4.626048
GFER	0.218046	0.049187	1.219487	3.591865
RBBP8NL	0.218132	0.049224	-1.32979	3.389577
BLMH	0.218433	0.049314	-0.89433	5.26211
SNHG22	0.218433	0.049335	-2.32925	2.757708
DDX43	0.218433	0.049345	1.507912	2.948961

PLEKHA2	0.218497	0.049377	-0.92761	7.022515
RFX6	0.21877	0.049473	3.170468	2.516113
LSM3	0.21877	0.049506	-0.82375	5.553985
LOC441242	0.21877	0.049509	1.629739	2.707886
MERTK	0.21877	0.049511	1.047661	5.351336
CCDC13	0.21877	0.049527	2.563614	2.115921
TMEM43	0.218821	0.049556	-0.80244	6.945409
ZNF320	0.218989	0.049612	0.962672	5.29192
LEXM	0.219061	0.049656	-6.23402	0.620654
CNTRL	0.219061	0.049668	0.85552	6.403336
GPR39	0.219061	0.049682	1.935534	2.256136
C22orf34	0.219216	0.049752	2.184115	2.832095
CD72	0.219216	0.049752	2.397228	1.781317
ACACB	0.219354	0.049802	1.576684	6.550863
SRMS	0.219379	0.049825	1.982377	2.319097
EEPD1	0.21939	0.049845	1.057869	4.678335
ITGA11	0.219628	0.049917	2.205957	4.201327
ITM2B	0.219657	0.049941	-0.84337	8.967028
MGAT4B	0.219852	0.050004	1.247303	3.940169
RRN3P2	0.220018	0.050059	1.587217	3.74097
EHMT1	0.220095	0.050094	-0.9261	5.436558
KRT14	0.220147	0.050124	-3.18293	9.911428
CMTM6	0.220168	0.050147	-0.78209	6.870488
ANKRD13A	0.220363	0.050209	-0.88995	6.94662
KIAA1324	0.220647	0.050292	3.212324	2.181949
PRAG1	0.220716	0.050325	0.880361	5.139383
SHE	0.221008	0.050418	1.533938	3.799573
INTS6	0.221008	0.050428	-1.00975	5.133587
MIR142	0.22209	0.050693	3.371376	0.953293
TXNDC8	0.22293	0.050902	-6.49169	0.813891
ODAM	0.223057	0.050949	3.503369	1.141828
CCDC73	0.223355	0.051035	-3.57166	1.837878
LINC00466	0.224203	0.051247	4.422041	0.861061
CPTP	0.224253	0.051277	-1.06257	4.774764
FAM234B	0.22474	0.051407	-1.44059	3.226604
NDUFB1	0.224895	0.05146	-0.83175	5.170577
TMEM253	0.224923	0.051485	1.51985	3.047203
MIR1-1HG-	0.224979	0.051516	3.427348	1.371959
MCUR1	0.225119	0.051577	-0.86446	5.898987
LRRK2	0.225119	0.051584	-1.70971	6.644365
TCTEX1D4	0.22518	0.051617	-2.20401	2.494865
FOXF2	0.225243	0.051649	1.923978	3.374544
MTHFD1	0.225278	0.051687	-0.75475	6.413151
ZNF843	0.225278	0.051694	3.267748	1.103267
APOBEC3C	0.225302	0.051718	-1.14841	4.758633

ITK	0.225302	0.051736	1.303813	3.729643
MTMR1	0.22535	0.051765	-1.59702	4.275256
LOC38983	0.225801	0.051887	-1.20665	6.265805
CTTNBP2N	0.22612	0.05198	-0.90511	6.985583
RIOK1	0.22612	0.051997	-1.22013	4.547208
PNMA8B	0.226271	0.05205	3.130842	1.244442
SERPINB6	0.226489	0.052119	1.060522	5.507488
ZDHHC2	0.226795	0.052218	1.270987	4.284274
FNDC3B	0.226795	0.052237	2.16963	2.37096
LOC10192	0.226795	0.052244	1.040047	4.54038
KCP	0.226807	0.052279	1.212571	3.431629
DPY30	0.226807	0.052301	-1.30086	4.131636
LOC72973	0.226807	0.052313	-1.39262	5.481928
NAPSB	0.226807	0.05232	2.367151	1.688423
IL23R	0.226991	0.052381	2.84694	1.147464
LPIN1	0.227065	0.052425	-1.92903	3.342442
SLC41A2	0.227065	0.052435	1.712261	5.083859
NEK6	0.227176	0.052479	2.906314	2.579243
RAMP3	0.227925	0.05267	2.311519	2.058542
KCTD3	0.228218	0.052757	-0.81537	7.772747
HIST2H2BA	0.22842	0.052822	-3.02162	1.962224
CROCCP2	0.228475	0.052853	0.909983	4.712181
DNAAF1	0.22865	0.052926	2.966441	1.354216
DNAJA4	0.22865	0.052931	2.550939	1.200888
ADGRF1	0.22865	0.052952	-1.55694	4.903107
ATP6V1A	0.22865	0.052968	-0.83173	7.265355
ARL5A	0.228667	0.05299	-3.3115	0.869649
FTH1	0.228889	0.05306	-0.94283	10.14576
ADAMTS18	0.229152	0.05314	2.664788	1.94652
DPY19L1P2	0.229348	0.053204	2.598555	2.082424
HOXD1	0.229651	0.053296	-4.01371	0.823028
NAPA-AS1	0.229651	0.053311	-2.53281	2.500484
YPEL3	0.229937	0.053396	-1.48273	3.290054
FBN3	0.230266	0.053491	3.248432	1.782646
SNORD58C	0.23031	0.05352	-4.63837	0.715853
MIR3142H	0.230384	0.053556	4.160818	1.816643
SNORD50A	0.230427	0.053585	1.663665	2.945206
PDCL3	0.230482	0.053616	-0.95577	5.081276
MPHOSPH	0.230768	0.053708	-0.82553	6.787108
ARHGEF16	0.230768	0.05372	0.803871	6.080876
MIR6512	0.23101	0.053795	-2.88903	1.677657
KCTD5	0.231045	0.053822	-0.89021	5.401897
KCNE4	0.231069	0.053846	2.310688	2.542427
TPMT	0.231083	0.053868	0.940967	5.199041
ITPRIPL2	0.231249	0.053926	1.270141	3.405417

KIAA1522	0.231274	0.05395	1.931396	2.997504
SLC9A9	0.231461	0.054013	-1.18924	5.044373
SNORA57	0.231473	0.05405	1.004165	5.795879
FAM27E3	0.231473	0.054053	1.56215	3.059245
ERAP1	0.232263	0.054256	-1.12989	4.739707
LENG8	0.232301	0.054284	1.005449	5.178432
DAD1	0.232307	0.054304	-0.80266	5.882629
XYLB	0.232749	0.054426	0.920721	4.704257
CD2	0.232916	0.054501	1.80753	3.014338
PDGFRB	0.232916	0.054503	0.96509	6.145553
PAFAH1B1	0.232946	0.054532	-0.75448	7.779587
NACA4P	0.232946	0.054548	-1.09795	4.581353
ADAM20	0.233109	0.054605	-2.11304	3.086175
LINC01605	0.23324	0.054664	-2.19927	2.747474
ARHGAP10	0.23324	0.054681	-0.94495	6.974799
LINC00294	0.23324	0.054692	1.036435	4.165896
RHOJ	0.233364	0.05474	1.403659	3.396271
APP	0.233555	0.054829	2.686055	0.953282
HS3ST3B1	0.233555	0.054831	-2.48255	1.825252
HIST1H4D	0.233555	0.054842	-1.6419	3.475379
HSP90B1	0.233676	0.054889	0.732506	9.618665
ZNF282	0.233828	0.054953	2.959796	0.850261
OXER1	0.233828	0.054965	1.632082	2.898498
RAB6D	0.233828	0.054982	2.40758	2.454109
TOGARAM:	0.234158	0.055083	2.558419	1.730284
SPRR2D	0.234158	0.055103	-2.98807	7.123991
OTUB2	0.234158	0.055116	-6.25093	0.63658
TRMT11	0.234287	0.055171	0.853792	4.875801
PLEK2	0.234287	0.055195	1.179413	4.191904
ZNF736	0.234287	0.055222	-1.23743	4.531313
RGS3	0.234287	0.055222	2.274162	2.501093
GARS	0.234378	0.055264	2.699167	1.870825
AHCYL2	0.234378	0.055283	2.115788	2.408112
STAT5B	0.234378	0.055301	0.726813	6.036358
CNPY4	0.234604	0.055392	1.417194	3.16859
PSMD2	0.234604	0.055396	-0.95528	5.184599
NPLOC4	0.234604	0.055411	-0.7754	6.4152
AASDHPPT	0.23464	0.055439	-1.00379	4.959157
RDH14	0.23513	0.055573	2.111492	1.888454
POLQ	0.235296	0.055632	-0.96731	5.264225
MRPS15	0.235541	0.055727	-0.89817	5.444791
HIST1H2BN	0.235541	0.055728	-1.38869	3.626262
CMTM8	0.23578	0.055803	1.678801	2.62468
TET2	0.236157	0.055912	-0.76671	7.180816
IFT46	0.236715	0.056079	3.3279	1.217821

IL12A-AS1	0.236715	0.056092	-2.59253	3.026671
FAM204A	0.236715	0.056118	-6.18787	0.582641
PLK1	0.236715	0.05612	0.972631	4.239828
LOC103021	0.236775	0.056154	-1.42068	3.648574
TPTEP1	0.236785	0.056176	2.331541	1.821104
NAV2-AS5	0.23716	0.05629	3.684984	1.482454
TIGD1	0.23716	0.056303	1.341782	3.789224
CEP112	0.237166	0.056324	2.786249	1.535868
DENR	0.237469	0.056415	-0.77947	6.824135
FMO2	0.237512	0.056444	-2.15512	2.594869
KIRREL3	0.237853	0.056544	-6.2864	0.655868
FRY-AS1	0.238369	0.056687	2.080381	1.745426
LRRK1	0.238508	0.056739	0.816644	6.117578
LOC101928	0.239268	0.056939	-1.53503	4.137255
CEACAM6	0.239347	0.056977	0.877849	9.593812
FTL	0.239684	0.057077	0.805361	8.890157
CYP4Z1	0.239704	0.057125	-3.67799	1.486951
MCM7	0.239704	0.057131	1.438413	2.686287
EXOSC1	0.239704	0.05714	-1.39564	3.223421
KC6	0.23975	0.05717	-6.01886	0.464606
INSYN2B	0.239891	0.057223	2.225782	2.043857
ZNF813	0.240011	0.057271	-1.98494	3.296747
SSNA1	0.240048	0.05731	-1.04848	4.574911
TMEM35B	0.240048	0.057338	1.653874	2.641808
HIST1H1B	0.240048	0.057339	-0.81176	5.68464
NYAP2	0.240314	0.057422	-4.0795	0.877506
WIPF3	0.240338	0.057447	-2.86756	3.37969
MIF-AS1	0.240983	0.05762	2.167742	2.55258
PPP1R26	0.241536	0.057772	0.81836	5.218664
LINC00898	0.241653	0.057823	-6.98181	1.2045
TIAM2	0.241653	0.057839	1.443451	3.054487
ERMN	0.241793	0.057892	4.917529	0.592765
SLC6A7	0.241893	0.057939	3.846383	1.175567
SMIM4	0.241893	0.057956	-1.61924	2.909266
TNFSF4	0.24224	0.058058	3.370115	0.510697
TPP1	0.242323	0.058098	0.722055	6.557576
PDCD6IP	0.242427	0.058143	1.058241	3.78191
RHOF	0.242441	0.058166	2.794264	1.939974
TEX9	0.242545	0.05821	4.288991	1.519096
LNP1	0.243227	0.058393	2.101255	2.18633
AGAP1-IT1	0.24361	0.058505	1.187487	3.665264
PSMC1	0.243651	0.058554	-1.27007	4.955053
FAS-AS1	0.243651	0.058555	3.543	1.434499
PCSK6	0.24373	0.058624	2.843088	1.483473
CXCR4	0.24373	0.058632	2.251106	2.175146

GALR2	0.24373	0.058633	3.133898	0.892926
LOC10192‹	0.243742	0.058655	3.120783	1.787002
RSL24D1	0.243862	0.058711	-0.82876	5.611492
TDG	0.243862	0.058724	-0.99086	5.473705
CHAF1B	0.244438	0.058882	-1.49081	3.397237
SLC33A1	0.244854	0.059002	1.187146	3.847469
CD248	0.244956	0.059046	1.213178	4.234948
CNTNAP3	0.245043	0.059102	-1.68237	3.92434
MSL3P1	0.245043	0.059107	1.582408	2.69025
BLK	0.245063	0.059137	3.206044	1.210676
USP46-AS1	0.245063	0.059152	-1.96329	2.278971
FUT3	0.245199	0.059204	-2.15654	3.180583
RACK1	0.245436	0.059301	-0.7567	9.239476
ADAMTS2	0.245436	0.059302	1.288364	4.759478
TOX2	0.245645	0.059381	3.896092	1.2613
PRRT2	0.245645	0.059402	2.677015	1.593574
TM4SF5	0.245645	0.059412	2.862575	2.300963
ARHGEF35	0.246013	0.059521	-1.15412	4.19221
TMEM221	0.246124	0.059567	-3.65448	1.717016
ERGIC2	0.246154	0.05961	-0.94247	5.520365
LINC00861	0.246154	0.059624	1.885159	2.586748
TULP3	0.246154	0.059656	-1.02913	5.598085
TLR6	0.246154	0.05966	2.063437	2.77491
CYP7B1	0.246154	0.059683	-1.1371	4.08539
CBFA2T2	0.246154	0.059694	1.871871	1.705652
RGS5	0.246178	0.059736	-2.64074	1.470101
CMTM4	0.246178	0.05974	0.880773	6.103199
LINC01342	0.247005	0.059961	2.845176	2.326403
LOC10192‹	0.247082	0.059999	-5.93799	0.414995
LINC00941	0.247117	0.060028	3.235552	0.997195
GLYCTK-AS	0.247273	0.060086	2.194184	1.874615
CPSF3	0.24733	0.060127	-0.84336	5.796248
FBRS	0.24733	0.060157	-0.77562	7.073849
NCDN	0.24733	0.060177	-6.25461	0.63088
VWA2	0.24733	0.06018	1.097893	5.524446
LOC10192ᵟ	0.247457	0.060231	-1.7673	2.743836
BTAF1	0.247498	0.060264	-0.77886	7.820918
PPM1B	0.247498	0.060281	1.045314	4.375611
ZMAT2	0.247755	0.060366	-0.83373	7.254072
LINC00885	0.247755	0.060384	-3.15808	1.081878
SLC39A8	0.247802	0.060423	3.224407	1.113078
PDLIM2	0.247802	0.060435	-1.24848	4.108476
CDSN	0.247948	0.060503	3.590094	1.069929
LOC10192ᵟ	0.247948	0.060511	-6.28063	0.651621
ACTB	0.248075	0.060567	-0.78006	12.02445

FCGR1B	0.248075	0.060582	-4.63305	0.639186
CHGB	0.248741	0.060765	-6.98387	1.201299
ADGRF3	0.248782	0.060795	-2.48195	1.664969
TNC	0.249059	0.060895	-1.13101	7.639762
KAZN	0.249059	0.060905	-2.36868	2.605794
METTL8	0.249059	0.060923	-0.79547	5.920491
CALM3	0.249438	0.061047	0.71678	7.875993
LINC00310	0.249438	0.06106	-4.15823	1.326904
SMC3	0.249438	0.061088	-0.72374	7.204622
CD1D	0.249438	0.061097	2.424409	1.515451
GLIS2	0.249526	0.061139	2.264599	1.796455
IL1RL1	0.24958	0.061172	1.795443	2.11809
C8orf74	0.249815	0.06125	-5.88933	0.377387
TRABD2B	0.250043	0.061326	2.090372	2.598178
ZNF83	0.250638	0.061492	-2.32977	2.162591
SLC44A5	0.251047	0.061613	-6.86474	1.105786
SIRPG-AS1	0.251586	0.061766	2.289223	1.058946
IKZF2	0.251625	0.061795	-2.53424	2.44491
PDE4DIP	0.251739	0.061844	1.129099	4.753263
ADAMTSL5	0.252009	0.061942	-1.08123	4.4754
LOC10013(	0.252009	0.061971	4.780669	0.54643
SNORD113	0.252009	0.061971	3.697356	1.34077
LOC285762	0.252601	0.062145	3.304993	1.200982
MOV10	0.252601	0.062158	-3.44739	1.06896
LOC72908(	0.2527	0.062203	-6.67694	0.95658
C3orf67-AS	0.252719	0.062256	-3.26848	2.463509
NUP214	0.252719	0.062275	-0.81778	7.44607
SELP	0.252719	0.062294	2.549392	1.390827
SOBP	0.252719	0.062297	1.740702	3.183479
PCBP2-OT1	0.252719	0.062327	-2.64014	1.214643
DLG2	0.252719	0.062347	2.50065	2.111595
ACSM6	0.252719	0.062351	2.09027	2.679584
CASC20	0.252738	0.062376	-6.44705	0.777469
PPCS	0.252762	0.062402	2.608823	1.628789
HTR5A-AS1	0.252804	0.062433	-7.07613	1.283081
AGAP9	0.253836	0.062723	1.091391	4.432299
LINC01363	0.253836	0.062729	4.281737	1.055374
LCN2	0.253908	0.062767	-1.0887	8.126497
PTPN9	0.254314	0.062891	0.699331	5.410008
DNAJC25	0.254314	0.062909	3.03671	1.359693
TMEM44	0.254491	0.062973	3.135409	1.634732
SLC7A6OS	0.254628	0.063028	1.223265	3.425937
E2F3	0.254848	0.063113	2.574572	1.513295
MICU2	0.254848	0.063124	-0.82297	5.471738
MCM5	0.255419	0.063286	-1.01894	4.931062

MFHAS1	0.255595	0.06335	1.325667	5.019894
ENDOD1	0.255735	0.063426	-0.81596	6.92519
RAVER1	0.255735	0.063426	0.813942	5.416978
DHX29	0.255902	0.063489	-0.73916	6.594593
PTRH1	0.256283	0.063604	2.609663	1.042903
SPTSSB	0.256522	0.063693	-1.25501	4.042217
TPRG1L	0.256522	0.063705	-0.89332	6.030408
RAPGEF5	0.256585	0.063741	-0.78929	7.209019
CPOX	0.256676	0.063796	-0.84018	5.426925
IPP	0.256676	0.063805	-1.32372	3.11131
HDDC2	0.256677	0.063826	-1.04616	5.270708
TBX6	0.256964	0.063922	-1.8685	2.652865
PDCD6IPP2	0.256964	0.063949	-1.45025	3.136554
TRMT5	0.256964	0.06396	-1.02486	4.293974
RFNG	0.257046	0.064001	-1.2076	3.505451
WDR12	0.257166	0.064086	-1.00603	4.506091
EID3	0.257166	0.064091	1.457277	3.384317
PTP4A2	0.257166	0.064098	1.248898	4.729642
FIBIN	0.257166	0.064115	2.173455	2.951915
UBXN6	0.25732	0.064174	1.978573	2.193317
DTD1	0.257391	0.064216	-1.04955	4.946049
KBTBD12	0.257391	0.064233	2.328276	1.986831
PCSK7	0.2574	0.064258	0.702474	6.778261
PMS2P1	0.2574	0.064277	1.099279	4.147064
AVPR1A	0.257942	0.064452	1.687529	2.778211
STX11	0.257942	0.064458	-2.21139	3.144586
GJA3	0.257942	0.064475	-2.89933	1.532879
HPRT1	0.258027	0.064517	-0.92467	4.906644
LRRC70	0.258057	0.064546	-3.3463	1.226295
ARFGAP1	0.258059	0.064567	2.243047	1.637952
LDB1	0.258102	0.064599	1.294503	3.378685
ATM	0.258349	0.064681	0.805381	7.203196
BTG2	0.258393	0.064713	0.974591	8.032321
SPRY4-IT1	0.258737	0.064821	2.000126	1.759364
GMPS	0.258892	0.064886	-0.86082	6.058195
POLR3GL	0.258892	0.064901	0.958156	5.44187
AKR1B15	0.2589	0.064924	-2.20593	2.387253
DLG5	0.259007	0.064972	-1.17453	4.344095
MBP	0.25944	0.065102	-0.83873	5.721521
RASGRP2	0.25959	0.06516	-4.57304	0.663689
ZNF800	0.259692	0.065207	-0.71865	5.870562
TUSC8	0.259929	0.065287	-6.02747	0.465548
RAB11FIP1	0.259963	0.065317	-0.97114	5.506565
CASTOR3	0.260154	0.065386	1.074661	3.559277
CEBPA-DT	0.260157	0.065411	1.943853	2.195893

EEF1AKMT	0.260157	0.065429	4.131717	1.398051
PCDHB18P	0.260381	0.065506	2.512952	1.293771
CYMP	0.260464	0.065549	-6.05572	0.486426
ADAMTS9-:	0.260485	0.065575	2.210287	2.362732
ZNF253	0.260669	0.065642	1.042032	3.884083
PCDHB11	0.261013	0.06575	2.695374	1.377865
CEP135	0.261013	0.065771	0.908685	4.384185
SMAD1-AS:	0.26104	0.065799	1.695884	2.602276
PPP1R13B	0.261533	0.065956	-0.72397	6.861495
FAM210A	0.261533	0.065976	-2.66858	1.691544
HPGDS	0.261533	0.065999	2.754562	2.068264
TXN	0.261533	0.066008	-2.56768	1.880144
HIVEP1	0.26187	0.066115	-0.93815	6.86234
SNAPC1	0.26187	0.066136	-1.60938	4.4512
KIAA1958	0.261924	0.066178	-6.11848	0.531193
KLHL29	0.261924	0.066192	-1.47395	3.962019
SSH3	0.262196	0.066282	-0.81828	6.400611
TMEM79	0.262348	0.066341	-2.62371	2.518765
HSPA1A	0.26264	0.066437	-2.17202	6.105292
TMEM220	0.26293	0.066543	0.924937	4.126374
ZBTB2	0.26293	0.066552	0.977039	4.290312
MIR6510	0.263371	0.066685	-2.60228	2.332139
TCF3	0.263415	0.066738	1.811872	2.422677
RORA-AS1	0.263415	0.066739	-1.18743	4.329829
LLGL2	0.263461	0.066772	1.194158	3.401024
LOC100287	0.263864	0.066897	-2.23404	2.175156
LOC644285	0.263864	0.066917	0.768621	5.315685
SDHAP1	0.264166	0.067021	-0.73522	5.971917
ACTG1P20	0.264166	0.067036	-1.23213	3.310755
DOT1L	0.265035	0.067278	-0.74612	6.660866
SRGN	0.26514	0.067337	4.123821	0.830035
OLFML1	0.26514	0.067348	1.353003	3.41147
SOCS1	0.265308	0.067412	1.600899	2.756973
ZFPM2	0.265524	0.067516	1.746766	3.717428
KCNQ1-AS:	0.265524	0.067517	3.664719	0.733481
PA2G4	0.265524	0.067546	-0.77912	7.370271
XDH	0.265524	0.067553	-1.09835	6.747964
HCP5	0.265558	0.067607	1.045803	4.542191
MRPL47	0.265558	0.067616	-3.6485	0.556515
IFT74	0.265558	0.067626	-6.36262	0.713914
SYDE2	0.265956	0.067749	1.475384	3.457184
SYT11	0.266955	0.068025	1.710366	3.339337
SPTY2D1	0.267061	0.068074	-0.88408	6.014594
CD44	0.267858	0.068299	-1.30901	3.803065
LY6G6C	0.267957	0.068346	-2.97307	1.594392

STEAP3-AS	0.267977	0.068387	1.608368	2.919104
LINC00333	0.267977	0.068394	3.603394	0.618787
LOC10192€	0.268251	0.068507	2.454679	1.209866
PGBD2	0.268251	0.068508	2.013942	1.76298
PTMS	0.268275	0.068536	0.892691	8.14466
ZNF608	0.268343	0.068575	0.881643	6.123101
STRAP	0.268345	0.068597	-0.74498	6.41743
EIF2AK4	0.2684	0.068633	0.711078	6.543986
LOC10050ᴇ	0.268788	0.068759	-2.59023	1.686124
NTNG2	0.268788	0.068807	3.096763	1.263584
IGSF21	0.268788	0.06882	2.716553	1.835996
BRCA2	0.268788	0.068846	0.88428	4.757448
SQSTM1	0.268788	0.068862	-1.75132	2.16816
HRCT1	0.268788	0.068864	2.986575	0.998168
RTP1	0.268788	0.068884	-5.96166	0.423218
SNORD105	0.269123	0.069009	1.258827	3.761362
LOC101927	0.269123	0.069034	-2.78252	1.871624
ZNF710	0.269123	0.069035	-0.80275	5.869559
TMEM8B	0.269199	0.069084	1.970066	1.892872
TMEM47	0.269199	0.06911	1.431778	3.873068
ZNF818P	0.269199	0.06912	-1.70839	3.050094
CHST3	0.269374	0.069187	-1.23308	5.318074
NT5DC3	0.270346	0.069459	0.855818	5.373179
MCTS2P	0.270608	0.069555	1.104305	4.237865
FLJ44635	0.270608	0.069579	1.900984	1.861765
ELMO1-AS1	0.270608	0.069592	3.192782	1.216074
PXDC1	0.27068	0.069632	0.946884	4.758804
CHI3L1	0.270919	0.06973	-1.52377	4.001914
ACVR1	0.270919	0.069738	2.650435	1.883359
SCARNA12	0.271499	0.069909	-0.76975	6.780317
GDNF-AS1	0.271681	0.069978	3.14557	0.687819
STXBP6	0.271696	0.070004	3.305087	0.748807
B3GALT2	0.271738	0.070036	-1.15839	4.008279
IKBKG	0.271778	0.070069	-6.12234	0.539867
B3GNT3	0.271851	0.070119	0.852292	6.308005
PTGES	0.271851	0.070132	-2.36846	2.729131
TRMO	0.271896	0.070182	1.058072	3.686099
CRLS1	0.271896	0.070199	1.792755	1.807576
UGT1A7	0.271896	0.070209	-1.45932	4.578399
TMEM177	0.272067	0.070276	2.801534	1.086449
BMS1P1	0.272069	0.070298	0.747099	5.942426
TYW1	0.272236	0.070363	0.756515	5.610459
DKK1	0.272297	0.070401	-2.87395	3.375048
PLA2G6	0.27234	0.070451	-4.1157	0.807939
TBXAS1	0.27234	0.070459	-4.36821	0.455984

NUDT16	0.27234	0.070478	1.755721	2.086072
MAP4K3-D	0.272401	0.070516	1.195245	3.995369
KANK2	0.272477	0.070578	1.62015	4.404168
HIBADH	0.272477	0.07058	0.973321	4.945016
CWC27	0.272687	0.070657	-1.81913	3.332787
TDRD1	0.272713	0.070685	-4.42617	1.41567
EML4	0.272776	0.070739	1.541699	2.805526
PELI2	0.272776	0.070762	1.39578	5.051015
TRIAP1	0.272776	0.070768	-1.1025	3.769425
PBRM1	0.273094	0.070873	-0.7408	7.959073
GABARAPL	0.273127	0.070903	4.256533	0.722655
RPL22L1	0.273316	0.070975	-1.23288	4.631196
HIST1H4H	0.273996	0.071173	-0.97727	4.311807
PRKAR2A	0.274214	0.071256	-0.76823	6.361164
MAPK8IP2	0.274214	0.071274	-2.98514	1.660402
SLC44A3-A	0.274324	0.071325	1.949115	2.405725
FGF11	0.274681	0.071449	-2.1278	4.000772
SMC4	0.274681	0.071508	-4.95163	0.883277
NFE2L1	0.274681	0.071528	0.688599	8.385861
DACT3-AS1	0.274681	0.071547	3.254085	0.564921
RRN3P1	0.274681	0.07155	1.323358	3.566527
SREBF2	0.274681	0.071551	-0.88033	5.024289
SRP68	0.274704	0.07158	1.864629	2.329845
ASB8	0.275255	0.071754	-0.86463	4.631368
DCBLD1	0.275255	0.071782	0.861765	4.833422
LGALS1	0.275255	0.07179	1.057334	4.223608
GRIN2B	0.275434	0.071859	2.936872	0.882613
VTRNA1-1	0.275458	0.071888	0.882864	7.678537
SNORD41	0.275575	0.071954	1.060315	5.317335
MTHFD2L	0.275575	0.071963	-1.41863	2.936141
LOC100506	0.27576	0.072037	1.610392	2.455551
USP37	0.27576	0.072068	-0.7914	5.90744
TK1	0.27576	0.072078	0.898658	4.050166
TMCC1	0.275766	0.072102	-1.28627	3.407051
EMD	0.276546	0.072328	-0.99579	4.753028
RBM12B	0.276655	0.072379	0.703034	5.857965
DVL3	0.276863	0.072456	-0.77475	7.038927
STK17B	0.276939	0.072498	0.84431	6.380308
TRAPPC4	0.277064	0.072554	1.098206	3.375216
IL1A	0.277084	0.072581	-2.04707	3.940469
MYO10	0.277833	0.0728	0.743124	8.315394
RPSAP9	0.277968	0.072865	2.776393	1.172856
RIPK3	0.277968	0.072881	1.062325	4.108025
SPIN3	0.278156	0.072952	0.976632	4.093633
RNA5-8SN5	0.278283	0.073008	-0.90121	12.56283

SLC18A2	0.278333	0.073044	0.828016	5.039521
MDC1	0.278543	0.073121	-1.39125	4.422641
BCHE	0.278643	0.073197	3.286414	1.261085
VASH1	0.278643	0.073208	0.884752	5.28668
FAM230B	0.278643	0.073215	3.833783	1.177272
MTA2	0.279061	0.073396	-0.73683	7.402815
STK35	0.279061	0.073406	-0.69423	6.760892
VAPB	0.279061	0.073406	-1.61678	3.184572
ADAM19	0.279061	0.073416	0.853168	5.811841
TRIM2	0.279643	0.073592	2.884808	1.224249
TARSL2	0.279921	0.073703	0.885073	4.930644
GUSBP2	0.279921	0.073713	1.803078	2.461657
TAB1	0.279921	0.073733	0.935813	4.365156
PCNP	0.280251	0.073842	-0.85555	6.316621
PRKD1	0.280471	0.073923	2.154554	2.848365
HACE1	0.280748	0.074019	-2.59757	2.003598
LOC101928	0.281015	0.074112	-1.55381	3.37328
KRT7	0.281109	0.074159	1.286514	6.337114
LGALS7	0.281181	0.074244	-2.58598	3.019377
CFAP44-AS	0.281181	0.074245	3.651891	0.844035
GSKIP	0.281181	0.074247	1.572945	2.751037
ENDOV	0.281192	0.07429	2.593993	0.802677
KIAA0355	0.281192	0.074295	-0.9405	5.120092
MTMR10	0.281243	0.074332	-1.21687	3.967123
MECOM	0.281424	0.074402	0.842203	5.271077
SSH2	0.281454	0.074433	2.267792	1.582136
DHX32	0.281568	0.074486	-0.76842	5.974862
KCTD7	0.281699	0.074543	1.633066	2.622221
MYDGF	0.281849	0.074606	0.79518	5.107015
C15orf39	0.281999	0.074668	-0.81981	5.070219
LOC100130	0.282099	0.074718	-1.0663	4.686699
SH3BGRL2	0.282266	0.074785	-0.77262	8.366694
GTF2IRD2B	0.282617	0.074907	1.179164	4.443197
COX5A	0.282617	0.074924	-0.74425	5.346182
TOMM70	0.282917	0.075026	-0.72453	6.747775
FOXD2	0.28314	0.075108	2.081725	2.171633
LINC00870	0.283297	0.075173	4.191695	0.897333
LAMB2	0.284097	0.075408	0.770659	6.850848
MIR6081	0.284303	0.075486	-3.25096	1.092446
KRT31	0.284502	0.075584	-3.70672	1.517426
KRT37	0.284502	0.075584	-6.26792	0.643349
TXNIP	0.284684	0.07568	1.118528	6.11622
TAF8	0.284684	0.075694	-0.81673	5.12723
LOC285074	0.284684	0.075702	-0.75148	5.8031
GATAD2B	0.285241	0.075873	-0.94548	4.372496

MCU	0.285407	0.075941	1.261824	3.769742
LOC10192[illegible]	0.285912	0.076098	-2.76439	1.145125
TLR5	0.285983	0.07614	-1.32292	3.745135
PRKAG2	0.286041	0.076179	1.864868	2.388108
LOC15368[illegible]	0.286062	0.076207	1.530725	3.335856
ITPR3	0.286578	0.076368	0.70503	8.526999
TP53INP2	0.286666	0.076415	-0.86064	7.326261
PGM5	0.286781	0.076469	1.717394	4.532261
LOC38919[illegible]	0.286843	0.076509	-5.61885	1.48711
LINC02411	0.286891	0.076545	-7.35858	1.517957
RRP1	0.287604	0.076758	-0.85522	5.609215
EPB41L3	0.288582	0.077047	3.188785	1.235018
DISP1	0.288582	0.077066	1.392021	3.418666
C11orf24	0.288628	0.077102	1.37371	3.436161
PROX1	0.289066	0.077242	2.477878	1.843222
CLDND1	0.289242	0.077343	-2.81842	1.500234
SBK1	0.289242	0.077346	-2.55041	0.941515
THRIL	0.289242	0.077389	1.047045	4.044342
FAM169B	0.289242	0.077396	3.126714	1.285598
GUK1	0.289242	0.077406	-1.61398	3.276114
MRPS7	0.289369	0.077463	-0.82165	5.451944
LSM14B	0.289538	0.077534	-0.6964	5.848446
FDPSP2	0.289538	0.077563	-0.67534	5.980755
MGC3280[illegible]	0.289538	0.077588	2.656095	1.621968
RPIA	0.289538	0.077603	-0.90192	4.571356
FCGRT	0.289704	0.07767	1.556457	3.144631
TOLLIP	0.289746	0.077705	-0.7748	6.335712
RNF114	0.289873	0.077763	0.72054	5.335872
ZNF429	0.289982	0.077815	-1.29023	4.17203
FAM78A	0.290085	0.077867	1.235467	3.224419
MINDY3	0.29022	0.077966	-0.91404	4.893526
NRAV	0.29022	0.077972	-1.31951	3.712928
MAD2L1	0.29022	0.07802	1.289824	4.414245
ATL1	0.29022	0.078025	-7.33977	1.506071
PHC2	0.29022	0.078031	1.638855	2.408315
RPS23	0.29022	0.078044	-0.72422	8.402764
PHETA2	0.290516	0.078147	1.678581	2.330496
COA5	0.290774	0.078243	0.872637	4.902027
NXPH3	0.290774	0.078264	2.254631	1.972331
OTUD3	0.290811	0.078304	0.880899	5.305224
ANXA2P2	0.290811	0.078321	-0.76125	7.9273
DPYSL2	0.290894	0.078366	1.503883	3.167144
RPL39L	0.29101	0.078421	-3.0576	0.424417
DUSP12	0.291884	0.07868	-1.27314	3.229211
CBLN3	0.291915	0.078733	2.422398	2.209059

C5orf51	0.291915	0.078737	-0.68757	5.897777
LGI2	0.291915	0.07876	3.24385	0.586652
LAMP2	0.292057	0.078822	-0.82082	6.692207
TMEM121	0.292936	0.079102	-6.05345	0.486265
STK10	0.292936	0.079106	0.768598	5.978652
PDE3A	0.292995	0.079159	3.087028	0.802848
VRTN	0.292995	0.079187	-6.61371	0.906914
ZMYM5	0.292995	0.079194	-1.31564	3.977642
NR4A1	0.293006	0.07922	1.693841	2.948106
LRP10	0.293245	0.079309	-0.7328	8.010957
ADD3-AS1	0.293669	0.079447	-3.57753	0.50844
LOC100128	0.293788	0.079503	-2.7409	1.206936
ARHGAP18	0.293838	0.07954	1.502649	5.704029
LOC440173	0.293967	0.079614	-3.54388	1.750901
LINC02421	0.293967	0.079623	-3.96224	0.531217
PKN2	0.29424	0.079721	-0.71566	7.930766
ADAMTS19	0.29429	0.079758	2.748934	1.440197
ST3GAL3	0.294329	0.079792	2.50474	2.101169
RAVER2	0.294987	0.079995	0.703761	5.566655
DNAH7	0.29515	0.080063	1.730678	3.061181
GJC2	0.295401	0.080155	2.155404	1.797722
TXLNB	0.295625	0.08025	2.031634	2.437585
MCC	0.295625	0.080264	-1.36368	3.199461
TMEM165	0.295694	0.080306	-2.02844	2.416215
IER5	0.295804	0.08036	-0.74343	6.564877
SLC6A6	0.296083	0.08046	2.955053	0.999913
LOC101927	0.296234	0.080525	2.651889	1.161386
MICU1	0.296572	0.080641	2.002615	1.800153
HIST2H3D	0.296967	0.080772	-3.65458	0.523386
ITGBL1	0.297336	0.080897	2.113814	1.564835
BZW1	0.297352	0.080925	-2.5932	1.499103
ZBTB11-AS	0.297362	0.08098	-1.80197	2.858834
TNFSF12	0.297362	0.081001	2.541629	2.223292
GDPGP1	0.297362	0.081005	1.632723	2.190612
ZNF286B	0.297362	0.081042	1.302412	3.082864
GPS1	0.297362	0.081057	1.800966	2.286975
PLEKHG1	0.297362	0.081073	-0.778	6.749954
MIR4449	0.297455	0.081129	1.007568	4.59661
GALNT4	0.297455	0.081146	2.76005	2.327316
SRSF11	0.297579	0.081204	-6.06747	0.49552
LINC00937	0.297826	0.081296	3.298691	0.772507
VTI1B	0.297848	0.081335	-0.75971	5.736813
SCAMP2	0.297848	0.08135	-0.74202	6.328585
OTUD6B-AS	0.298178	0.081464	1.00088	3.866489
FAM19A2	0.298212	0.081498	2.327798	1.579159

MROH6	0.29837	0.0816	-2.33383	1.399747
CRISPLD2	0.29837	0.08161	1.119215	6.837704
LOC10050€	0.29837	0.081613	1.801526	2.489289
NAA38	0.298411	0.081677	-1.02476	3.865223
SMIM14	0.298411	0.08168	2.171528	1.968067
PUS7	0.298411	0.081697	-0.73971	5.540598
VWA5A	0.298677	0.081796	2.344793	1.462384
SBF2	0.298677	0.081876	-0.78112	7.100006
LINC00673	0.298677	0.081892	-1.53553	5.029461
FXR1	0.298677	0.081892	2.541884	1.096459
CAPZA1	0.298677	0.081909	-0.77385	7.699706
MIR320D1	0.298677	0.081928	2.995923	1.074801
INSIG1	0.298677	0.081939	-3.40905	1.686156
IQGAP1	0.298748	0.082004	-0.7095	10.03499
TGFBR2	0.298748	0.082007	3.135265	0.599617
SMYD3	0.298897	0.082072	2.894344	1.089115
ALDH1L1-A	0.299102	0.082152	-6.18918	0.583212
SCP2	0.299775	0.082362	1.349384	3.268386
LOC100507	0.300152	0.08249	2.495198	1.641674
C10orf25	0.300234	0.082536	3.387488	0.449477
ST8SIA3	0.300398	0.082626	3.076106	1.672116
ECPAS	0.300398	0.08263	-0.67754	8.077717
RPL19P12	0.30043	0.082678	1.432158	2.62019
GSDMA	0.30043	0.082688	-2.964	1.131112
IGSF10	0.301296	0.08295	1.447205	4.117476
NDUFAF2	0.301312	0.082987	-1.23813	4.092288
LOC101928	0.301312	0.083008	1.657225	2.212889
C16orf87	0.301312	0.083028	-1.53834	3.84534
SLF1	0.301442	0.083088	-1.11976	3.976933
THSD4-AS2	0.301542	0.083161	-1.17568	4.411769
RHOXF1-AS	0.301542	0.083165	1.774154	2.378504
SPDYE5	0.302256	0.083386	-0.9439	4.233246
PHLDA3	0.302256	0.083444	-1.52292	2.288822
TPCN2	0.302256	0.083458	0.701208	5.451457
ABO	0.302256	0.083459	-0.90767	6.29758
STAT6	0.302474	0.083544	1.282269	3.116596
OVCH2	0.302511	0.083579	2.830944	1.227143
STXBP5-AS	0.302667	0.083647	-2.45108	2.010972
RPL37	0.302754	0.083702	-0.68572	8.416476
LINC00261	0.302754	0.083742	2.610109	6.165362
CYP20A1	0.302754	0.083744	0.772247	6.108257
SNORA55	0.302979	0.083847	1.124115	7.126208
B4GALT4-A	0.302979	0.083877	2.9257	0.987576
UCKL1-AS1	0.302979	0.08388	-0.98255	4.248396
IDH1	0.303081	0.083933	1.202448	3.23319

SERINC5	0.303142	0.083974	-0.72736	7.787204
NTN4	0.303693	0.084151	1.06074	5.180172
ORMDL2	0.303752	0.084207	-1.03275	4.173866
EMC2	0.303752	0.084225	-0.7482	6.088577
BCL9L	0.303752	0.084242	-0.69329	7.752441
SPRY1	0.30406	0.084368	1.600582	2.48289
MIR181A1I	0.30406	0.084392	3.573442	0.871028
RHBDF1	0.30406	0.084417	-0.93547	5.156655
LOC728613	0.30406	0.084426	1.404304	3.254863
HIST1H2BN	0.30426	0.084506	-1.27906	3.489314
LOC10272:	0.30497	0.084728	3.252828	0.713942
LIPK	0.30525	0.08483	-3.8214	0.680979
NRROS	0.305762	0.084997	2.359753	1.15024
SNORD54	0.305792	0.08506	1.214147	3.414594
UBL4B	0.305792	0.085075	-6.44908	0.778894
ATP13A5	0.305792	0.085109	2.337774	1.774708
HMGB1	0.305792	0.085122	0.834992	4.452289
SLC35A3	0.305792	0.085129	2.110309	1.499981
CHD8	0.306239	0.085294	-1.13346	3.715444
LINC00842	0.306239	0.085303	-2.76407	1.291503
PHF23	0.306527	0.085408	-2.01312	1.630472
RFK	0.306582	0.085449	1.171848	4.328751
FAM174A	0.306833	0.085544	-0.93775	4.775186
SUGP1	0.307097	0.085656	-0.79258	5.196815
PNPO	0.307097	0.085688	0.67452	5.739199
SOGA3	0.307097	0.085692	2.097928	2.133078
TMEM95	0.307122	0.085724	-3.10049	1.688133
ARAP3	0.307446	0.085839	0.894165	4.601584
ANKRD44-I	0.307609	0.085909	1.153741	3.035583
LPCAT1	0.307882	0.08601	1.789252	2.583877
LOC101929	0.308415	0.086184	2.970908	1.177163
TPI1	0.308495	0.086232	-2.08914	3.079801
SMIM12	0.308797	0.086341	2.498376	0.718775
PIK3CA	0.308942	0.086407	-0.77554	5.675407
RPS10P7	0.309169	0.086495	1.574035	2.560214
LINC00426	0.309216	0.086533	2.428202	1.040883
PRO1804	0.309333	0.086605	-1.74167	2.727953
CCDC153	0.309333	0.08665	2.744931	1.155048
EME2	0.309333	0.086661	1.187743	3.46265
DNAH1	0.309333	0.086666	0.757518	5.962873
SLC35C2	0.309798	0.086826	1.229346	3.387894
RPS6KL1	0.309798	0.086847	1.163117	3.448321
LOC100996	0.310479	0.087073	-1.96705	3.434349
NELFCD	0.310479	0.087088	-0.76508	5.622459
MAGI2-AS3	0.310906	0.087233	1.280126	4.206377

ARMC5	0.310913	0.08726	-2.82224	1.690157
GAU1	0.311015	0.087314	2.245299	0.808052
KIF4A	0.31119	0.087388	1.256376	4.133272
YEATS2	0.311296	0.087443	-0.74883	7.048601
HK1	0.312428	0.087808	-1.72105	2.724999
HMGCS1	0.312428	0.087812	-3.95487	0.772069
STAMBPL1	0.312475	0.08785	1.261323	3.826704
R3HDM4	0.312484	0.087878	-0.76089	6.063857
FAM198A	0.312516	0.087912	2.227823	2.264137
DOK4	0.312747	0.088014	0.694941	5.986404
MIR5706	0.312747	0.088035	-3.74439	0.621402
DLX3	0.312747	0.088099	-2.52202	2.377261
STIM1	0.312747	0.0881	-3.37458	0.59094
MKKS	0.312747	0.088104	1.973926	2.308041
F11R	0.312981	0.088227	-0.73806	8.211146
GNG12	0.312981	0.088228	-0.74322	7.790383
SELENOM	0.312981	0.088246	1.764687	3.45977
PLA2G12A	0.313207	0.088335	-0.71289	6.019196
PTPRJ	0.313322	0.088393	1.131972	5.821921
DLAT	0.313502	0.088469	-0.6491	5.880305
ACCS	0.313895	0.088605	2.079391	1.607488
DSCAS	0.314208	0.088719	-2.48393	1.810683
LOC10013(	0.314466	0.088818	2.207965	2.305055
HIST1H2BD	0.314592	0.088879	1.88609	2.377778
ATP11A-AS	0.314641	0.088918	1.373406	3.410532
NLGN1	0.315176	0.089095	2.72875	1.815874
FZD7	0.315435	0.089239	-1.02629	4.374913
B3GNT9	0.315435	0.089239	1.208962	3.13276
BCL9	0.315435	0.089245	0.695955	5.371534
SPRR2B	0.315668	0.089336	-2.80269	1.693915
INTU	0.315903	0.089428	-0.90209	4.525192
JKAMP	0.316009	0.089484	2.942624	1.241486
TMEM54	0.316148	0.089549	0.696625	5.457153
ITGB8	0.316216	0.089606	-0.84486	7.174065
MIR7845	0.316216	0.089619	2.888833	1.110845
INIP	0.316293	0.089667	-1.23858	4.060671
MEOX2	0.316323	0.089719	2.743248	2.062794
ANKDD1B	0.316323	0.089726	1.825381	1.832668
WSCD1	0.316559	0.089819	2.557242	1.487527
ASNA1	0.316659	0.089873	-1.22918	4.226245
NPHS1	0.316813	0.089942	-4.17869	0.388665
DNAJC15	0.316876	0.089986	0.900261	4.956394
RNF115	0.316988	0.090043	0.797746	5.338098
GGNBP2	0.317124	0.090126	1.615583	3.350529
IL23A	0.317124	0.090147	-2.98239	1.826123

ZNF707	0.317124	0.090167	3.057704	0.941302
AARS	0.317124	0.090191	0.722421	6.91395
RBM45	0.317124	0.090211	1.181504	3.37088
LOC10028ɛ	0.317484	0.09035	-1.0722	3.742855
HOXD-AS2	0.317484	0.090364	3.244902	0.419231
TRIM17	0.317554	0.09041	-3.33442	0.601481
MTAP	0.317691	0.090475	-0.6822	6.020006
ZNF702P	0.31774	0.090515	2.302831	2.379361
FLG-AS1	0.31797	0.090606	-4.02623	1.21295
FBXW5	0.318214	0.090701	-0.70089	6.138515
LINC01733	0.318475	0.090801	-6.69512	0.970957
RAE1	0.318601	0.090863	-2.97273	0.455146
LINC02123	0.318875	0.090967	-7.181	1.370687
MIB2	0.318893	0.090998	2.590467	1.350902
TFF2	0.319156	0.091101	2.321216	6.059017
SEL1L	0.319156	0.091125	0.655675	7.072986
MYCT1	0.319637	0.091333	1.587025	2.158962
CBARP	0.319637	0.091338	-2.12933	2.20057
ACOT4	0.319637	0.09134	1.953485	2.175173
ENTPD3-AS	0.31986	0.091429	1.622219	2.639586
ENPP1	0.320499	0.091638	1.792657	2.902754
RPL37A	0.32076	0.091738	-0.67918	8.407047
CXorf21	0.320976	0.091849	2.492416	2.26942
RPA1	0.320976	0.091852	-0.68127	6.016026
ENPP4	0.321014	0.091889	1.575175	3.22284
COQ2	0.321143	0.091952	-1.03142	3.948912
MFN1	0.32185	0.092181	-0.77442	7.147383
ZFP30	0.322009	0.092252	-1.02833	4.140899
ZDHHC13	0.322372	0.092404	-3.73431	0.993953
MIR944	0.322372	0.09242	-3.67033	1.23218
PPIF	0.322372	0.092435	-0.91124	6.228565
DDHD1	0.32268	0.092581	2.508919	1.464109
MEIS2	0.32268	0.092605	1.4782	1.886915
PROC	0.32268	0.092615	2.600677	1.414087
MIR4513	0.32268	0.092627	-3.5894	0.979484
RRP15	0.322921	0.092723	-0.78306	4.873404
RUNDC3B	0.323251	0.092852	4.185814	0.625347
DND1	0.323251	0.092913	1.425541	2.651381
CTR9	0.323251	0.092927	-0.66681	6.879149
ZNF337-AS	0.323251	0.09294	-1.86528	2.549891
TRIM59	0.323251	0.092973	-0.96299	3.976739
DNAJB3	0.323251	0.092995	-2.46103	1.917111
LOC10028ɛ	0.323251	0.093009	1.978276	2.2969
LOC33980ɜ	0.323251	0.093027	-2.31757	1.01795
CEPT1	0.323294	0.093066	1.945729	1.919851

CYP4Z2P	0.323438	0.093133	-2.33773	3.632987
LRRC26	0.323807	0.093307	2.345893	1.945594
WDR36	0.323807	0.09331	-0.71625	6.233437
MT1X	0.323807	0.093371	-1.19068	5.419284
SCARNA5	0.323807	0.09339	0.620865	7.458058
FAM129B	0.323807	0.093393	-1.18389	4.322173
SGCB	0.323807	0.093409	0.917909	5.195497
EXOSC6	0.323807	0.093423	-0.95735	3.980431
TRMT10A	0.324306	0.093618	3.059926	0.557817
ACSS1	0.324306	0.093642	3.064149	0.934181
ZNF586	0.324306	0.093646	-3.91795	0.51264
ENPP6	0.324358	0.093738	2.48959	0.782332
BEAN1-AS1	0.324358	0.093743	-3.77869	1.144832
YIPF6	0.324358	0.093787	3.255872	0.790608
ARVCF	0.324358	0.093831	1.213728	3.318147
TSPAN31	0.324358	0.093831	-0.88264	4.185604
WDR54	0.324358	0.093835	1.655774	2.937629
LOC101928	0.324358	0.093845	1.6899	2.316777
SPATA5	0.324675	0.093971	1.002626	3.996579
BRAF	0.324675	0.093989	-0.70105	5.923341
ARHGEF19	0.324725	0.09403	-1.19126	4.084449
GM2A	0.324834	0.094088	-1.70078	2.762858
AMER1	0.325042	0.094174	-0.86015	4.972759
HHIPL1	0.325086	0.094218	3.109441	0.934656
TAS2R5	0.325086	0.09424	1.883963	2.122794
ARHGDIG	0.325573	0.094407	-6.55817	0.858161
TLX2	0.325619	0.094447	1.912848	1.821802
RN7SL1	0.325723	0.094526	-0.82037	14.84703
CYP4F2	0.325723	0.094535	2.386409	1.625587
TF	0.325723	0.094559	-2.29613	3.626713
GATM	0.325723	0.094583	0.730924	6.020684
TRAPPC10	0.325846	0.094645	-0.7149	6.240916
GTPBP10	0.325901	0.094707	-2.39508	1.474306
CD27	0.325901	0.094713	1.638831	1.785146
EPAS1	0.326968	0.09505	0.690171	7.892645
GFRA3	0.327156	0.095131	2.959925	0.606716
LOC728743	0.327211	0.095174	1.081117	3.191465
IRX6	0.328167	0.095497	-6.33776	0.697863
ATP8B2	0.328167	0.095507	0.940485	4.824701
CHRNA3	0.328167	0.095554	3.006886	0.966277
EDF1	0.328167	0.095582	1.187489	3.331037
ORMDL3	0.328167	0.095585	0.691944	5.668301
CRIP2	0.32824	0.095638	-0.99271	4.778368
DPM2	0.32824	0.095659	-1.09405	3.964851
RPS6KA3	0.328493	0.095759	0.625434	7.254416

LOC10050€	0.328612	0.095821	-1.39512	3.11574
RIMS3	0.32877	0.095893	-1.70665	2.782957
LOC101927	0.328817	0.095934	2.93572	0.844602
MZT2A	0.329087	0.096039	-1.24559	3.656708
TWISTNB	0.329143	0.096082	-1.34612	4.238389
ADCY6	0.329143	0.096109	0.670855	6.594155
PLEKHA7	0.329383	0.096206	-0.69472	7.802132
TPD52	0.329844	0.096367	2.394747	1.388293
ADM2	0.329857	0.096397	1.126299	3.403674
WDR92	0.330313	0.096557	1.669512	2.761742
ZBTB47	0.330404	0.09665	0.859513	5.057142
DNAH11	0.330404	0.096662	-1.66835	4.185016
ZC3H12A	0.330404	0.096672	-1.09644	5.609867
LINC01426	0.330404	0.096691	2.649107	1.157667
MAGEC1	0.330416	0.096722	-7.11496	1.315019
SMCR5	0.331031	0.096928	-1.02243	3.751043
GSPT1	0.331113	0.096979	2.856796	1.28565
PALB2	0.331131	0.097031	-1.01292	4.883907
ZNF780A	0.331131	0.097038	0.986714	4.147002
NOP10	0.331374	0.097162	-0.69116	5.148115
PABPC3	0.331374	0.097163	-1.29387	3.046478
ANTXR1	0.331388	0.097194	0.944456	6.280954
GNMT	0.331738	0.097324	-3.63524	2.692482
MIR663A	0.331923	0.097405	1.315246	3.074519
KCNE2	0.332034	0.097475	-5.14541	4.081919
RHOB	0.332034	0.097491	0.941049	7.021892
EXOSC5	0.332431	0.097634	-1.20311	3.340691
DNAH3	0.332518	0.097687	1.541502	2.598376
ACTG2	0.332627	0.097746	1.898047	2.64335
RPS26P11	0.332959	0.09787	-1.27641	3.969415
RTF1	0.333379	0.098057	-0.63751	7.195188
TMTC2	0.333379	0.098078	-0.76722	6.29897
FKBP4	0.333379	0.098095	-0.70223	7.260853
CHP1	0.333379	0.098102	-0.68629	8.014113
MTURN	0.333379	0.098146	0.767069	6.258761
RPL21	0.333379	0.098156	-0.85108	6.159495
MCF2L	0.333555	0.098235	1.900897	1.810863
COMP	0.333804	0.098335	-4.01702	0.598603
SLC6A1	0.33401	0.098438	-2.07903	2.870738
NXF1	0.33401	0.098453	0.778712	5.067199
SNORA63	0.33401	0.098477	-0.77047	7.072163
CFAP100	0.334022	0.098507	3.531736	1.050051
CLEC11A	0.334329	0.098631	1.617381	2.336918
PKIB	0.334329	0.098688	2.805707	0.577398
PINK1-AS	0.334329	0.098702	0.973797	3.460214

NEK7	0.334329	0.098727	-0.89232	6.326204
CCDC15	0.334329	0.098733	-1.25734	4.035404
ABCC5	0.334363	0.098771	-0.6906	7.377066
SLC38A5	0.334765	0.098943	-1.3994	2.793159
PKD1P1	0.334765	0.098944	0.645995	6.657069
CDRT15P1	0.335195	0.099098	-3.72508	0.614493
TSPAN33	0.335351	0.099186	1.635765	3.110283
XKRX	0.335351	0.099231	-1.59347	3.100451
TCEAL7	0.335351	0.099264	2.364201	1.014669
HECTD3	0.335351	0.099265	0.66336	6.433055
TFAP2A	0.335351	0.099304	-1.32086	2.893987
LINC01106	0.335351	0.099307	1.146172	3.801709
RPS7	0.335542	0.099399	-0.6955	7.495869
ADAM1A	0.335542	0.099418	1.273906	3.487763
LSP1	0.335886	0.099547	1.194868	2.998541
ARL2BP	0.335886	0.099574	-0.87156	5.332229
PTGER2	0.336045	0.099667	2.304594	2.603016
LOC440896	0.336045	0.099702	2.661148	0.746182
SCARNA4	0.336045	0.099703	1.032931	8.148264
SH2B1	0.336412	0.099839	0.791633	4.761318
GPC2	0.336513	0.099896	2.228091	2.022576
ELF2	0.336603	0.099965	-1.02451	3.860381
SVIL-AS1	0.336603	0.099978	-1.49855	2.476082
INO80D	0.336894	0.100091	-0.67792	8.061289
USP32P1	0.337155	0.10021	-1.68788	3.941769
ARL6IP5	0.337155	0.100223	0.702783	5.972698
ANGEL1	0.337444	0.100337	0.702445	4.839617
UBAC1	0.337659	0.100449	-0.79495	5.171144
LMAN2	0.337659	0.100455	-0.70114	7.134193
ASAP1-IT1	0.338166	0.100642	1.261894	2.757216
ACTC1	0.338166	0.100661	3.483862	1.257816
SMIM6	0.338661	0.100846	1.634896	2.039671
ZNF217	0.338661	0.100863	-0.65413	6.991284
PDZK1IP1	0.338726	0.10091	-0.85094	5.591919
PATL2	0.33879	0.100963	1.858405	2.103137
LINC01623	0.33879	0.100984	4.06964	0.545057
UBQLN2	0.338845	0.101028	-0.72592	5.745749
ZEB2	0.339266	0.101182	1.383097	4.183096
MRPL16	0.339266	0.101254	0.829678	4.871229
RNASE6	0.339266	0.101263	2.038513	1.967439
PHF3	0.339266	0.101263	1.2012	3.165514
GLDC	0.33948	0.101354	-1.78371	3.225154
CLNK	0.340204	0.101613	2.547493	1.245405
LINC00907	0.340204	0.101626	3.3047	1.532082
PROSER2	0.340467	0.101732	-1.59215	3.844309

ABCC12	0.340518	0.101783	-6.12877	0.535433
CFAP65	0.340518	0.101802	-2.61577	1.227424
GSN-AS1	0.340673	0.101876	0.774964	5.079564
CRIM1	0.340719	0.101918	0.597631	7.07171
FOXL2NB	0.340755	0.101991	4.191641	0.476318
LOC105378	0.340755	0.102006	-2.91104	1.231442
KCTD15	0.340755	0.102011	-2.34193	1.921735
C9orf170	0.340792	0.10205	3.546165	0.889606
HAAO	0.341221	0.102206	1.553376	2.938478
LOC550113	0.341349	0.102291	-2.62306	2.61888
NIT1	0.341349	0.102344	0.992656	3.75229
SNORA67	0.341349	0.102345	-0.7558	6.28929
LINC02367	0.341349	0.102355	2.45472	1.41185
TLR3	0.341606	0.102479	-1.02956	3.93875
MIR221	0.341606	0.102512	-2.82939	1.812077
CKMT1A	0.341606	0.102515	-1.06911	3.582743
CCR8	0.341641	0.102553	2.970867	0.484075
SSBP1	0.341763	0.102637	1.799875	2.468153
NELFA	0.341763	0.102645	-0.76478	4.970185
RPL5	0.341788	0.102684	-0.69173	9.332403
DNTTIP2	0.341788	0.102708	-0.63719	6.401598
NAPG	0.342279	0.102883	-0.7028	5.828839
MMP9	0.342486	0.102973	2.704431	1.427197
NLRC5	0.342601	0.103036	0.762183	5.902114
FBXO41	0.343036	0.103194	0.923694	3.898501
DDX3X	0.343109	0.103286	-1.01544	4.792674
BCS1L	0.343109	0.103341	2.839682	0.901149
ZNF781	0.343109	0.103351	2.335567	1.341975
PUSL1	0.343109	0.10336	-1.26403	3.491982
ABT1	0.343109	0.103387	-0.95673	4.238442
KCNA1	0.343109	0.103406	3.497216	1.123391
TMEM128	0.343109	0.103411	2.338481	1.633019
IRF1	0.343365	0.103555	1.127434	5.339447
WDFY3	0.343365	0.103592	-0.66524	7.917284
LNPK	0.343365	0.103639	2.443061	0.518122
EYA2	0.343365	0.103644	-3.24952	0.634496
LACTB2-AS	0.343365	0.103648	1.393619	3.391271
FCER1A	0.343365	0.103671	-2.41521	1.736824
CTBP1-AS	0.343365	0.103683	1.180706	2.969377
EPB41L4A-	0.343394	0.103719	-2.44101	1.751444
SCLT1	0.343394	0.103767	0.711618	5.325887
APOL6	0.343394	0.103775	0.631161	7.085603
KLHL11	0.343705	0.103897	-0.76486	5.094299
LOC100049	0.344005	0.104015	1.012513	3.267757
TACC2	0.34457	0.104214	-1.66753	2.641292

ABCA7	0.344589	0.104247	-0.82647	5.534962
SPDYE4	0.345079	0.104444	4.004632	1.369268
PCED1B-AS	0.345079	0.104452	1.900333	2.358068
C16orf72	0.345362	0.104565	-0.6213	6.828833
AMPD2	0.345444	0.104641	1.959427	2.262852
LINC01402	0.345444	0.104646	3.575539	0.513126
SNORD110	0.346167	0.104893	-1.36443	3.477996
AMOTL1	0.346317	0.104987	-3.04144	1.183907
VAMP3	0.346317	0.104995	-0.71935	6.609769
HIST1H2BF	0.346317	0.105023	-1.12639	3.932027
PHF21A	0.346823	0.105231	-1.98949	1.877347
CBLC	0.346823	0.105232	-2.30382	0.967513
CLDN16	0.347072	0.105336	-3.59418	1.039432
RDH10-AS1	0.347621	0.105531	0.915397	4.511668
FOXH1	0.348199	0.105734	2.42726	0.600779
UGDH-AS1	0.348255	0.10578	1.075625	3.396204
MCEE	0.348344	0.105886	-0.91003	3.979565
BARX1	0.348344	0.10589	-4.41418	1.394552
FAM83H	0.348344	0.105919	-1.55535	6.995635
ARHGEF39	0.348344	0.10592	1.304004	3.015919
HOXB9	0.34852	0.106001	2.441595	2.109419
RNF123	0.348529	0.106046	0.687907	5.661404
LOC101927	0.348529	0.106062	2.538416	1.423946
PPP2R5A	0.348529	0.106089	-0.80093	4.985056
HUWE1	0.348569	0.106129	-0.633	9.777167
ADAP2	0.348598	0.106166	1.130439	3.376877
LOC100506	0.348754	0.10626	1.109825	3.66201
SLC35E2A	0.348754	0.10627	0.753724	4.733092
EGFEM1P	0.348994	0.106372	3.126414	0.541304
SYNE2	0.349246	0.106477	-2.8735	1.572998
BBIP1	0.349311	0.106525	1.556293	2.829834
UBE2L6	0.349333	0.10656	2.883507	0.876453
C12orf76	0.349365	0.106598	1.904783	2.143986
PRRC1	0.349835	0.10677	0.767974	4.864279
MMP28	0.35009	0.10688	2.172368	2.015757
RGS14	0.35009	0.106904	-0.98585	3.98647
SKIV2L	0.350128	0.106944	1.229275	3.117719
TESK1	0.35027	0.107016	0.886478	4.697129
POU3F1	0.350714	0.1072	-2.29374	2.24627
DNAJA2	0.350714	0.107208	-0.65823	7.184356
AP5B1	0.350807	0.107265	-0.75593	5.80442
LOC101928	0.351417	0.107487	-1.76726	2.722514
FEZ1	0.351417	0.107526	1.830474	2.149447
PCYOX1	0.351417	0.107537	-0.7283	7.450826
BPIFA2	0.35165	0.10764	3.545184	0.607852

IL21R-AS1	0.35165	0.107665	-6.26577	0.641871
FYN	0.351781	0.107736	1.056874	3.97727
LINC01232	0.351781	0.107786	1.955994	1.913853
LARP4B	0.351781	0.107791	-0.62469	7.753793
DGKA	0.351825	0.107833	-1.52608	3.335802
TMSB4X	0.351932	0.107894	0.655045	8.350797
HSD17B8	0.352168	0.107995	-3.17602	0.553299
POMGNT1	0.35228	0.108058	2.693025	1.156627
CD63	0.352368	0.108123	1.659152	1.895164
ADIRF	0.352368	0.10815	-0.99303	4.928038
RAB9B	0.352368	0.108171	2.239555	2.267841
RALB	0.352515	0.108244	-0.7291	7.543801
LOC101928	0.353164	0.108472	-3.6024	0.519006
SPRY2	0.353351	0.108558	0.960883	4.265197
DCBLD2	0.353478	0.108626	-1.41336	4.902653
NRON	0.35348	0.10867	2.175917	1.647052
LRRC71	0.35348	0.108684	2.76797	0.599994
LINC00674	0.35368	0.108774	0.659503	6.717541
H2AFV	0.353709	0.108821	0.607461	6.010618
SNORD15B	0.353709	0.10884	-0.66879	8.622238
LIPE	0.353801	0.108897	-1.37453	4.083217
COLGALT1	0.35413	0.109027	0.792495	5.765658
GPCPD1	0.354276	0.109101	-0.88812	6.822158
LOC100294	0.354475	0.109216	0.988721	3.696465
ST6GALNA(	0.354475	0.109219	2.030172	2.204323
BBOX1-AS1	0.355244	0.109485	3.357896	1.044778
RFX1	0.3554	0.109562	-0.97519	4.297174
SUZ12	0.355844	0.109728	-0.67647	6.48033
AVIL	0.355897	0.109773	1.294521	3.333173
TCP10L	0.356073	0.109856	-3.96122	1.460146
GLUD2	0.356095	0.109892	-1.51267	3.055132
ARID2	0.35613	0.109931	-0.66171	7.276311
MTLN	0.356193	0.109979	-1.42212	2.795515
THSD7A	0.356334	0.110057	1.146571	3.629417
AKAP13	0.356334	0.110081	1.39686	3.626177
LNX1	0.356426	0.110138	-1.37804	3.927157
PI4KAP1	0.356497	0.110189	-0.97355	4.4391
TTC3	0.356611	0.110258	0.676733	6.247557
ZSWIM8-A!	0.356611	0.110282	-1.76497	3.009763
LOC100996	0.356647	0.110322	1.348524	2.661344
NKAPP1	0.356769	0.110388	1.197972	2.637828
ZXDC	0.356908	0.11046	-0.93191	4.513942
LINC00222	0.35693	0.110496	2.337077	0.989801
ZNF10	0.357052	0.110563	0.993421	3.978128
VPS37C	0.357155	0.110641	0.704016	5.241978

RSPRY1	0.357155	0.110653	1.589152	2.030813
FLJ45513	0.357285	0.110747	1.784651	2.050515
ZDHHC1	0.357285	0.110767	1.198064	3.112417
PPP2R5C	0.357285	0.110816	1.114623	2.948533
CCDC57	0.357285	0.110871	0.75801	4.97766
2-Sep	0.357285	0.110872	1.115922	3.418684
LINC00865	0.357285	0.110889	1.783655	2.291729
ZNF416	0.357285	0.110895	-1.14441	3.116669
WDR24	0.357625	0.111043	-1.26142	4.250685
ZNF589	0.357625	0.111059	-0.82911	6.070956
HECTD2	0.357635	0.111099	-2.57432	1.278962
AGO3	0.357635	0.11112	2.050163	1.615243
H2AFZ	0.358249	0.11134	-0.66865	5.485005
CA3-AS1	0.358306	0.111387	-2.2106	2.592273
NFATC2IP	0.358323	0.111421	0.650525	6.396177
RAB19	0.358439	0.111518	3.087719	0.645021
SLC6A15	0.358439	0.111541	-2.95211	1.116492
TTLL10	0.358439	0.111544	-3.56839	0.864886
IPO13	0.358646	0.111638	-0.72475	5.256263
ADAMTS3	0.358806	0.111716	2.382806	2.005946
TP53BP2	0.359038	0.111818	-2.5599	1.267553
C3orf36	0.359237	0.111909	2.446835	1.28116
FRG1JP	0.359289	0.111954	-1.31138	2.749512
ZFAND3	0.359379	0.112011	-0.64846	6.186747
RPS8	0.359461	0.112066	-0.64552	9.171406
LOC100129	0.359528	0.112116	2.697795	0.563266
BUD31	0.359616	0.112184	-0.68163	4.994143
RIMKLA	0.359616	0.112202	1.510005	2.850249
DNAJC9-AS	0.360527	0.112515	-3.62344	0.535866
NINJ2	0.360669	0.112589	1.96159	1.741499
TRIM47	0.3607	0.112628	0.851721	4.12485
TIGAR	0.360722	0.112687	0.766709	4.925717
ZNF876P	0.360722	0.112693	-2.10699	1.919085
SST	0.360759	0.112733	2.846537	4.739443
ATP11A	0.36121	0.112904	2.656291	0.93538
CASP16P	0.361245	0.112954	1.717447	2.241512
PRXL2C	0.361245	0.112973	1.419102	3.143036
RAD51-AS1	0.361634	0.11313	0.892563	4.577392
ZNF256	0.361634	0.113153	-2.16099	2.426297
APLNR	0.361746	0.113242	1.722051	2.887867
DHX58	0.361746	0.113247	1.06354	3.5274
CACUL1	0.361932	0.113335	-0.60916	7.071059
SLC35D2	0.362078	0.113436	1.983885	1.520007
LOC100506	0.362078	0.113452	1.37843	2.908813
SLC6A10P	0.362078	0.113468	-2.43065	2.124016

HABP4	0.362135	0.113515	1.318742	3.940381
RWDD2A	0.362258	0.113583	1.958464	2.033217
LRRC4	0.36264	0.113732	-0.84651	5.076051
SERINC3	0.362902	0.113913	-0.75255	6.03305
FAM20C	0.362902	0.113914	0.961146	4.374348
CCDC50	0.362902	0.113932	1.29645	4.029273
MIR23A	0.362902	0.113957	-2.92713	1.649581
DOK6	0.362902	0.113962	1.796281	2.294536
HTRA1	0.363216	0.114133	0.940293	4.789863
TBC1D30	0.363216	0.114134	1.663697	4.024813
MANSC1	0.363216	0.114163	-0.7293	5.951603
TNFSF9	0.363216	0.11419	-2.44258	2.280791
FAM156A	0.363216	0.114208	-6.20357	0.592846
SNHG5	0.363723	0.114396	0.741049	5.440979
MRM2	0.363916	0.114486	-0.86268	4.295938
RHEX	0.364187	0.114601	1.890483	2.416324
CCND1	0.364244	0.114649	-0.66609	8.549599
RPP25	0.365013	0.11492	0.979209	3.917923
LOC100272	0.365031	0.114955	1.645945	2.033649
CCDC12	0.365234	0.115073	-1.96843	2.285543
RTN4R	0.365234	0.115078	1.888589	1.868163
CREB3	0.365487	0.115188	0.776461	4.371895
GDPD5	0.365514	0.115226	2.039846	1.847254
PPP3R1	0.365731	0.115324	-0.70736	7.058944
ZNF135	0.365741	0.115357	2.82605	0.744966
MAN2A2	0.365842	0.115418	0.730102	6.090483
MRPS18B	0.365855	0.115467	1.608847	2.954915
CNTNAP3P	0.365855	0.11551	-0.81955	4.971222
PSMB7	0.365855	0.115511	-0.70184	4.894793
LOC728752	0.366211	0.115653	2.242022	1.647931
MRPS5	0.36679	0.115866	-0.65231	6.542569
SMC2-AS1	0.366792	0.115931	-5.97017	0.428676
KRT19	0.366792	0.115935	0.741523	10.1358
CROT	0.366792	0.115956	-1.82666	1.881174
MIR8058	0.366954	0.116072	4.067347	0.868344
CCDC8	0.366954	0.116077	1.839825	2.552903
PURB	0.366954	0.116096	-0.6822	7.262546
PSMB1	0.366981	0.116134	-0.63405	5.82351
S100A16	0.367198	0.116233	-2.64422	1.617123
AGAP7P	0.367382	0.116321	0.706469	4.635765
OAS2	0.367603	0.116438	0.918283	3.869523
OR52I2	0.367603	0.116476	-6.01023	0.458743
INTS12	0.367603	0.116485	1.967058	1.576534
KCNMB1	0.367603	0.11651	2.056403	2.318731
SNORA87	0.368355	0.116801	1.672929	3.128938

IK	0.368355	0.116823	-0.63399	7.903067
ZNF138	0.368355	0.116838	-2.69522	1.848583
ABCD1	0.368368	0.116898	1.219238	3.296208
RTL6	0.368368	0.116901	0.674633	5.181216
CHRM5	0.368679	0.11703	-3.6361	0.462666
PLTP	0.368822	0.117105	2.457087	1.719616
RANBP10	0.368878	0.117153	-0.62609	5.824244
LAPTM4A	0.369351	0.117366	-0.67745	7.3524
SMLR1	0.369351	0.117392	3.118174	1.38682
ADAMTS17	0.369351	0.117393	-1.00709	4.039666
HP09053	0.369728	0.117565	-2.83643	1.195841
GRTP1-AS1	0.369728	0.117573	1.555205	2.447799
MYH13	0.369882	0.117652	1.6222	3.089986
LINC02615	0.370005	0.117722	1.143107	3.796088
ADAMTS7F	0.370005	0.117751	2.738943	1.564022
STAM2	0.370313	0.117879	-0.65089	6.215371
PDE6C	0.370397	0.117935	-2.98688	1.303183
CD4	0.370537	0.11801	2.442968	1.522673
GLIS1	0.370762	0.118112	1.92148	2.147919
PPTC7	0.37083	0.118179	-0.64988	5.683087
RAB41	0.37083	0.118226	2.457317	1.063852
MRPL46	0.37083	0.118229	-1.29318	3.505819
LINC00665	0.37083	0.11826	-2.44643	1.304254
PARD6B	0.37083	0.118312	-0.63688	5.398243
RTP5	0.37083	0.118314	2.955109	1.01076
PEBP1	0.370851	0.11838	-0.63178	7.150676
LINC02035	0.370851	0.118384	-0.89509	4.468849
RPL27	0.370851	0.11841	-0.63467	8.270408
SNN	0.371344	0.118606	-0.849	4.2293
LMO4	0.371344	0.118628	0.614733	6.870896
MYRF	0.371356	0.118662	2.318767	2.28522
HCG11	0.371363	0.118721	0.76907	4.552369
ATOH8	0.371363	0.118751	1.887642	2.896514
CABIN1	0.371363	0.118754	1.708859	1.486039
C1RL	0.371553	0.118853	1.405691	2.522833
SLC25A53	0.371553	0.118875	1.618152	2.384892
HMGN1	0.372142	0.119097	-0.59353	6.793136
COL4A4	0.372142	0.119163	1.772244	3.55324
RAB44	0.372142	0.119175	1.584004	2.090537
CACNB1	0.372142	0.119184	1.631071	1.917878
LINC02067	0.372289	0.119261	-0.64332	8.546416
ZNF503	0.372396	0.119326	-0.96434	4.473511
RIPOR1	0.372396	0.119356	1.781184	1.939484
LOC65365З	0.372762	0.119504	1.397078	1.976111
TMEM19	0.373321	0.119716	-0.64491	5.806236

ZNF232	0.373321	0.119743	0.898279	3.791556
OSR1	0.373627	0.119883	1.661164	2.270963
DENND6A	0.373627	0.119902	-0.57265	5.918223
AKAP1	0.374602	0.120268	1.811599	2.19494
RPS6KA2	0.374602	0.120298	1.761855	2.731565
GPATCH11	0.374602	0.120306	2.199143	1.007976
LOC339874	0.374701	0.120368	1.703041	2.730724
ASXL1	0.374807	0.120454	0.642842	6.558862
TUBGCP6	0.374807	0.120463	0.631701	6.582375
CA1	0.37509	0.120601	3.195784	1.015829
GNS	0.37509	0.120615	0.625871	6.379334
TMEM14A	0.375544	0.120791	-0.9873	4.478895
LOC285847	0.375721	0.120878	1.956685	1.795388
TUSC1	0.375981	0.120992	-1.35609	2.7737
BCL2	0.37624	0.121106	1.289554	3.903055
CLMAT3	0.376527	0.121229	2.273195	1.403655
CD180	0.376965	0.121401	3.058326	1.220706
CDNF	0.377147	0.12149	-2.8981	1.366702
LINC00957	0.377258	0.121584	-2.26173	1.340724
MIR641	0.377258	0.121587	3.681524	1.038137
SLC2A4RG	0.377531	0.121705	-0.74335	5.205536
SNORD79	0.377615	0.121782	-1.39821	2.913917
DVL1	0.377615	0.121799	-0.63281	6.591988
GOSR2	0.377615	0.12187	0.770033	4.938205
ATP10B	0.377615	0.121877	0.6992	7.089349
C11orf54	0.377615	0.121885	2.810305	1.016089
CFAP44	0.37764	0.121924	0.86978	5.101282
ZBTB18	0.377901	0.122045	1.350485	2.890301
RHOG	0.377901	0.122069	-0.87734	4.773601
KIF13B	0.378125	0.122172	0.665401	8.126634
GGT3P	0.378303	0.122261	2.429003	0.815602
TNFRSF10C	0.378334	0.122335	1.969628	1.910898
SNORD56	0.378334	0.122344	0.721195	5.380106
SMTN	0.378334	0.122362	1.560736	1.933894
QPRT	0.378341	0.122426	1.668425	2.380895
LCA5L	0.378341	0.122454	2.02438	2.486949
EMBP1	0.378341	0.122457	1.425634	2.567141
JPH2	0.378636	0.122585	1.738919	2.439165
MRPS25	0.378636	0.122644	0.573553	6.67512
HLA-DPB1	0.378636	0.122648	1.29507	3.904478
CHIC2	0.378636	0.122675	-0.7249	5.181558
SSU72	0.37875	0.122742	-0.63278	6.480313
DBR1	0.379064	0.122875	0.901453	3.690574
RCAN1	0.379186	0.122945	1.179183	2.936196
TNXB	0.379246	0.122995	1.392694	2.969673

RPS27	0.379838	0.123271	-0.67834	6.708238
MGST1	0.379838	0.12328	-2.71308	1.715339
GRIN3B	0.379838	0.12328	-2.00942	2.083859
SPTBN5	0.379906	0.123351	-1.19183	4.634594
ZNF592	0.379906	0.123363	0.578411	6.424791
CCDC96	0.380021	0.123431	-1.8042	2.008221
GOLGA5	0.380734	0.123694	-0.63089	6.182268
MST1	0.38077	0.123736	-1.1555	3.337169
ZNF706	0.381289	0.123936	-2.02158	2.107441
PLEKHN1	0.381332	0.124021	-3.0342	0.488012
CTAGE8	0.381332	0.124025	-1.84721	2.397859
CREG1	0.381332	0.124042	-0.67094	6.988129
RGS1	0.381371	0.12412	1.174948	6.012075
PRDM15	0.381371	0.124129	2.446467	0.912154
GRHPR	0.381371	0.124148	-0.61952	5.212199
CADPS	0.382	0.124383	3.475766	0.613564
EVA1B	0.382035	0.124426	-1.92566	2.834337
POR	0.382104	0.124504	-0.64619	6.049622
ARHGAP27	0.382104	0.12451	-0.68391	5.662719
PREB	0.382378	0.124667	0.618745	5.259308
ZFP3	0.382378	0.124671	-1.25305	4.036389
LCA5	0.382378	0.124711	2.648296	1.10881
PKD1L3	0.382378	0.124733	1.713141	2.165447
UPK1B	0.382378	0.124754	-1.70491	2.870054
HSP90AB4I	0.382727	0.124899	1.28814	3.891931
PPA1	0.38284	0.125042	-0.61318	6.321872
AKAP8	0.38284	0.125055	0.616614	6.135577
ALDH2	0.38284	0.125074	2.604632	0.538235
MID1	0.38284	0.125091	2.367857	1.934906
KCNS1	0.38284	0.125119	-3.35253	1.218786
UBE2N	0.38284	0.125122	-0.6453	5.789891
INSM1	0.383138	0.125288	2.783295	1.966339
CNP	0.383138	0.125296	-0.63273	6.23663
EIF3E	0.383138	0.125337	-0.64859	7.898156
SH3PXD2B	0.383138	0.125358	0.775239	6.63653
SH3TC1	0.383138	0.125419	-0.73526	5.811242
PLIN5	0.383138	0.125466	-2.51017	3.578578
CATSPER3	0.383138	0.125471	-1.52312	2.781024
SNHG20	0.383138	0.125498	1.323795	2.573581
LOC10012S	0.383138	0.125499	0.974424	3.594739
DLG5-AS1	0.383148	0.12555	-2.56308	1.053725
PRPF40A	0.383148	0.125564	-0.6489	8.075513
LEPROT	0.383442	0.125709	-1.8686	2.019204
LOC10013:	0.383442	0.125723	1.437814	2.510495
RPS6KA2-IT	0.383593	0.125803	2.218336	1.24175

NOV	0.383763	0.12589	-2.32	2.224812
ADH1A	0.383784	0.125951	3.001502	1.143251
ZMYND19	0.383784	0.125959	-1.20958	4.110065
LOC22072[illegible]	0.383789	0.125992	-0.62264	5.59855
BRSK1	0.383823	0.126034	1.546497	2.841016
LZTS1	0.384297	0.126221	-1.25297	3.872826
GNAL	0.384346	0.126299	-2.77431	1.66158
RAD52	0.384346	0.1263	-3.03912	0.649992
PTPN18	0.384494	0.126379	1.438504	2.816306
HMG20B	0.385374	0.1267	0.626876	6.325544
KRTCAP2	0.385611	0.126809	-0.74404	4.49844
ARHGAP15	0.38584	0.126915	1.257537	3.088475
TBC1D16	0.386471	0.127154	1.114409	3.083037
FAM86DP	0.386555	0.127213	1.128637	3.506204
KDM4A-AS	0.386595	0.127258	1.641597	1.651537
LOC40070[illegible]	0.386739	0.127336	-3.461	1.304146
LINC00346	0.38703	0.127464	1.275218	2.909801
IER2	0.387184	0.127546	-0.71519	6.386971
RPL10A	0.387255	0.127603	-0.60583	8.144705
CARHSP1	0.387255	0.127632	-2.416	1.909668
ARF3	0.387328	0.127687	-0.59024	7.566256
ACTA2-AS1	0.387466	0.127764	1.900041	2.407077
DNAH2	0.388253	0.128057	1.211329	2.72039
LOC10012[illegible]	0.388253	0.128086	2.08879	2.116505
JARID2-AS[illegible]	0.388409	0.128185	-1.94884	1.676247
ABCA3	0.388409	0.128227	1.092766	3.200671
SLC22A20P	0.388409	0.128246	-2.83625	1.025384
DCUN1D5	0.388409	0.128264	-0.66364	5.565963
KLF10	0.388857	0.128443	-1.99577	1.171842
EPB41	0.389443	0.128668	-3.55352	0.409322
AVL9	0.3895	0.128718	0.595343	6.52451
SLC17A7	0.389988	0.128936	2.011222	1.300742
UACA	0.389988	0.128943	-1.13432	3.401399
BTBD8	0.390397	0.129118	1.655817	2.489646
LOC40131[illegible]	0.390397	0.129171	-6.03429	0.472603
ZBTB20	0.390397	0.129184	0.990784	3.596063
PYCARD-A[illegible]	0.390397	0.129271	2.387798	0.701298
MED13	0.390397	0.129312	-0.63146	8.144876
SEMA3G	0.390397	0.129313	1.937573	2.677355
CDC42BPG	0.390397	0.129324	-0.73566	6.829015
FAM91A1	0.390397	0.129356	2.811712	0.773959
GOLGA2P5	0.390397	0.129363	0.752662	4.238891
RPN1	0.391144	0.129657	0.566867	7.834244
RAD51D	0.391144	0.129674	2.379611	1.693453
UBTD2	0.391158	0.12971	0.593847	5.151342

FOCAD-AS1	0.391218	0.129762	3.017794	0.832923
DUXAP8	0.391322	0.129842	2.270153	1.337935
SHLD2	0.391322	0.12986	-0.68852	5.411615
AP5Z1	0.391354	0.129902	0.701366	5.2413
ANKDD1A	0.391603	0.130016	-1.17041	3.632414
HLA-J	0.391741	0.130118	1.688627	2.109784
CPSF1	0.391741	0.130125	0.599686	6.414136
RHOD	0.392067	0.130266	-1.71024	2.188405
CAND1	0.392298	0.130374	-0.63869	7.196925
NBR2	0.392301	0.130407	0.802097	4.083537
YARS2	0.392312	0.130442	-1.11647	3.565908
FAM126B	0.392561	0.130557	-0.66385	6.927777
SLC9A7P1	0.392561	0.130589	2.367361	0.863974
SIGIRR	0.392677	0.130733	-3.84432	0.486793
LRTOMT	0.392677	0.130737	-2.84168	1.033147
TBC1D9	0.392677	0.130764	-0.66543	5.69741
IRAK2	0.392677	0.130779	-1.4379	4.088571
THSD4-AS1	0.392677	0.13079	2.57227	0.939729
ZFAND6	0.392677	0.130818	2.066554	1.162242
ARHGAP9	0.392692	0.130855	1.917621	1.034041
PLA2G4D	0.392779	0.130916	-1.75678	3.883503
LRRC40	0.393047	0.131048	-0.98364	4.127634
STRBP	0.393047	0.131069	-1.81038	2.355202
PFKFB2	0.393198	0.131151	0.636988	6.982076
C7orf43	0.393434	0.131262	1.818989	1.331187
GNAI1	0.393691	0.131379	-1.45347	3.148482
TMEM87B	0.393833	0.131459	0.66433	6.833629
PLXNA2	0.393912	0.131517	-0.60477	7.966235
ECE1	0.393974	0.131569	2.258338	1.154936
MRI1	0.394167	0.131666	-1.5559	2.534243
RTN4RL2	0.394191	0.131756	2.060494	1.746882
MAPK1IP1L	0.394191	0.131769	-0.63611	7.469785
PDAP1	0.394191	0.131784	-0.59284	7.728276
VPS35	0.394191	0.131801	-0.63341	7.304865
PPM1F	0.394232	0.131847	-0.56568	5.927741
FLJ20021	0.394276	0.131921	2.256013	1.410496
NXT1	0.394276	0.131927	-1.25879	2.783763
FCER1G	0.394276	0.131974	1.461695	2.715089
SGMS1	0.394276	0.132015	0.574062	5.708345
RSPO3	0.394276	0.132051	2.176386	2.828989
DNAH8	0.394276	0.132053	2.384555	1.27362
AMMECR1	0.394459	0.132147	1.666948	1.265161
HDLBP	0.394467	0.132181	1.711338	2.051184
PLCD4	0.395075	0.132417	0.93582	4.11294
MAPKAPK5	0.395529	0.132601	0.916865	3.528472

GALNTL6-A	0.395822	0.132732	-6.71223	0.984593
PFN2	0.396045	0.132864	-3.11556	1.231051
LINC00475	0.396045	0.132871	2.502828	1.32798
C1orf226	0.396121	0.132956	0.981283	4.012792
COL4A3	0.396121	0.13296	1.413333	2.950268
PPP4R1	0.396263	0.13304	-1.57615	2.924505
GAR1	0.396321	0.133091	2.516977	0.719964
CTHRC1	0.39645	0.133167	2.150984	1.34303
TEAD3	0.396453	0.133211	-0.62485	6.448972
OSGEPL1	0.396453	0.133232	1.062755	2.978109
ZNF471	0.397086	0.133513	-1.59601	3.452929
ITFG1	0.397086	0.133517	1.332853	2.67226
SLC25A28	0.397086	0.133541	-0.79316	4.471569
ERCC5	0.397998	0.13388	1.040687	3.445094
LIN7C	0.398071	0.133965	-0.59584	6.682202
TTC28	0.398071	0.133969	0.758351	5.91566
BTD	0.398238	0.134058	-2.80581	0.595495
ADGRF5	0.398473	0.134176	2.839709	0.943242
ZNRF3-AS1	0.398473	0.134217	1.350677	2.204189
CCDC30	0.398473	0.134234	1.597319	2.354686
LINC01116	0.39851	0.134289	-2.36593	1.125192
LOC101927	0.39851	0.134311	-2.22512	2.329481
RPS13	0.398676	0.134399	-0.63831	7.578925
EBLN3P	0.399145	0.134589	0.589849	6.685174
SENCR	0.399279	0.134667	2.314098	0.992646
PDCD5	0.399358	0.134788	-0.83061	5.033174
PAFAH2	0.399358	0.134796	0.619448	5.275733
LIMK2	0.399358	0.134803	0.820931	4.12805
KLF3	0.399358	0.134823	-0.66611	8.411277
KBTBD8	0.399733	0.134982	1.651562	2.549063
MEIS3P1	0.399888	0.135067	1.114023	2.711629
MIR23B	0.399994	0.135135	-3.19736	1.19422
NME9	0.400295	0.135294	0.677242	5.151643
BTN3A1	0.400295	0.135301	2.200383	1.308668
HAUS5	0.40038	0.135363	-0.8212	4.369273
BIRC2	0.400501	0.135462	1.913669	1.625042
YY1AP1	0.400501	0.135512	-2.76255	1.948479
PRKAG2-AS	0.400501	0.135516	2.03335	1.691231
COBLL1	0.400501	0.135533	-2.78139	1.554752
DPP9	0.400562	0.135586	0.588885	6.185546
RUNDC1	0.400787	0.135695	0.617551	5.486112
ZNF235	0.401167	0.135859	0.93435	4.063416
SPAG5-AS1	0.401167	0.135889	1.833332	2.100835
CLDN15	0.401209	0.135953	1.159722	2.763266
HDAC4	0.401209	0.135968	-0.61693	6.727522

HOPX	0.401742	0.136228	-2.61837	1.541716
LOC101928	0.401742	0.136229	2.119937	1.356049
DUSP2	0.401742	0.136256	-2.34457	2.80305
KIF11	0.401742	0.136279	-0.77363	5.297806
ADH5	0.402399	0.136534	0.563889	6.415786
REM1	0.40259	0.136678	2.956928	1.158414
RPL22	0.40259	0.136693	-0.64622	8.851501
NXPH4	0.40259	0.136697	-2.73467	2.23182
MACO1	0.402767	0.136789	-1.16082	4.523048
ZNF70	0.402773	0.136824	0.916807	3.75962
DYNAP	0.402993	0.136932	-3.00964	1.220264
DECR2	0.403545	0.137152	1.585261	1.687245
BOK	0.403633	0.137219	-0.67949	5.276836
IREB2	0.403633	0.137271	-0.6246	6.770235
COPS5	0.403633	0.137289	-0.66061	5.014046
LINC00423	0.403633	0.137313	-6.65761	0.941065
SNORD59A	0.403703	0.137369	0.688751	5.578499
TAPBPL	0.403811	0.137438	0.938	4.12225
APIP	0.404061	0.137556	0.92015	3.572759
SLC2A10	0.404345	0.137685	0.692916	4.81898
ANKH	0.404429	0.137747	0.590678	6.191432
KCTD18	0.404521	0.137821	-0.79697	4.644131
ZNF665	0.404521	0.137874	-3.02845	1.039254
ENTPD3	0.404521	0.137926	3.142637	1.180409
PSMC3	0.404521	0.137928	-0.58481	6.139823
CYR61	0.404521	0.137942	0.970126	5.989118
LINC01100	0.404591	0.138015	-2.7501	1.530646
RALGPS1	0.404591	0.138032	0.721979	4.532114
COL17A1	0.405102	0.138239	-0.61769	8.002682
CEP350	0.405627	0.138451	-0.59669	8.33645
FAM110D	0.405698	0.138508	2.19378	0.622147
DENND4C	0.40591	0.138613	-1.26862	2.823455
PTOV1-AS2	0.406095	0.138709	0.750896	4.058119
SCAPER	0.406307	0.138825	-0.76157	4.208025
CCSER1	0.406307	0.138847	2.06714	1.799779
LINC00680	0.406616	0.138986	2.21009	1.634616
C20orf202	0.406849	0.139116	3.289487	0.742403
GK3P	0.406849	0.139131	-1.51644	2.925833
TTC33	0.4069	0.139182	0.832443	4.612746
EIF2A	0.407455	0.139405	-0.59909	6.455112
ZNF436-AS	0.407616	0.139493	1.147801	2.619185
MIR1914	0.408092	0.139689	-2.0007	1.723042
GADD45B	0.408249	0.139815	-1.41513	5.372835
RPL34-AS1	0.408249	0.139816	2.35271	1.209028
GAD1	0.408249	0.139842	1.613132	2.048505

RARRES3	0.40852	0.140024	1.389602	2.905805
TRAPPC12	0.40852	0.140032	0.652717	5.554163
VPS9D1-AS	0.40852	0.140054	-0.82058	4.526351
LOC440311	0.40852	0.140067	2.038332	0.94805
GRB7	0.408876	0.140222	-1.30438	2.692728
PPIEL	0.408993	0.140295	0.954125	3.819293
CBR4	0.409224	0.140408	0.624027	5.423188
CC2D2A	0.409608	0.140572	0.631268	5.337696
TPT1-AS1	0.410177	0.140801	1.092629	3.56509
FIGNL1	0.410249	0.140859	-1.44981	2.706049
PAK1IP1	0.410426	0.140953	-1.19655	4.803708
POP7	0.410494	0.141016	0.958628	3.710038
TOR3A	0.410494	0.141059	0.766824	4.222326
LOC150776	0.410494	0.141076	0.742502	4.598736
PPFIBP2	0.410646	0.141167	-0.75231	4.607559
LRP5	0.410646	0.141195	1.534087	2.139652
TRIM44	0.411472	0.141535	0.61696	7.858625
TNFRSF1A	0.411472	0.141545	-0.73958	6.437977
LINC01268	0.411555	0.141607	1.735942	1.587753
YAF2	0.411946	0.141775	0.77999	4.652324
BIK	0.412123	0.14187	-0.83627	4.464693
RBM3	0.412299	0.141964	-0.59321	7.72605
ASPHD1	0.413035	0.142274	1.802692	3.43396
AADAC	0.413035	0.142395	1.526517	3.192227
FREM1	0.413035	0.142396	1.551914	1.798058
ATP13A1	0.413035	0.142405	0.541632	5.868213
PLCG2	0.413035	0.14242	0.690713	5.568255
RXRA	0.413035	0.142445	-0.95635	3.54037
ATP5F1B	0.413035	0.142462	-0.58127	8.369853
MTRNR2L8	0.413035	0.14253	-1.33197	5.551094
KLHL6-AS1	0.413035	0.142551	2.896615	1.198987
AKIRIN2	0.413035	0.142565	-0.82711	5.248201
ITSN1	0.413035	0.142585	1.316204	3.281442
YAP1	0.413097	0.14264	-2.75278	0.833929
LOC101928	0.413214	0.142714	0.953417	3.776994
BORCS8	0.413618	0.142894	-1.46606	2.428408
DANT2	0.413618	0.142936	1.153112	3.918569
GATA2-AS1	0.413618	0.142954	1.914333	1.932589
ADNP2	0.413804	0.143052	-0.55782	5.707269
LOC392232	0.413837	0.143096	-2.67068	2.82679
XRCC5	0.414286	0.143301	-0.58061	8.292674
NINJ1	0.414286	0.143319	0.901979	3.466611
PVRIG	0.414619	0.143468	0.953895	3.891439
SF1	0.414835	0.143576	2.00273	0.847779
UGGT2	0.414939	0.143646	0.728746	5.451832

MOGS	0.415636	0.143921	2.342198	1.117743
FAM135B	0.415855	0.14403	2.037856	1.543718
MGC15885	0.416027	0.144123	1.901606	2.485634
MARCKS	0.416072	0.144173	-0.62009	8.461202
CA5B	0.416863	0.144496	-0.98615	3.662026
ROR1	0.416863	0.144519	1.138282	4.240079
COG5	0.416863	0.144586	0.99953	3.615618
ADAMTS9	0.416863	0.14459	0.828496	5.909486
GRPEL1	0.416863	0.144616	-0.69842	5.078006
CCDC80	0.417684	0.144934	1.267318	3.680964
HERC5	0.418006	0.145111	1.243457	3.12721
CFL1	0.418006	0.145114	0.561098	8.826953
RIMKLB	0.418074	0.145235	-1.22453	3.58544
DISP2	0.418074	0.145254	1.697052	2.659013
SGPL1	0.418074	0.145268	-0.60055	7.600287
TMEM62	0.418074	0.145273	0.737442	4.896968
SNORA71C	0.418131	0.145326	0.933169	5.761856
PRKD3	0.418183	0.145378	-0.56492	5.883454
METAP1D	0.418548	0.145541	1.585304	2.671987
PRKCI	0.418548	0.145573	-0.63946	7.001936
TOR1A	0.419556	0.145958	0.657584	4.563475
SFXN1	0.419571	0.145997	-0.65277	5.238435
SDR42E1	0.419707	0.14608	-0.8217	3.924203
C11orf58	0.419707	0.146112	-0.56016	7.385983
RPS9	0.419732	0.146155	0.928636	3.852782
PRADC1	0.419884	0.146301	-1.28981	2.779651
LAMP1	0.419884	0.146352	-0.62777	7.992752
DUSP14	0.419884	0.146377	-2.50736	2.072729
TOB2	0.419884	0.146393	0.567441	6.99051
ADORA2A-/	0.419884	0.146403	1.604264	1.893834
PRKAA1	0.419884	0.14646	-3.29213	0.662508
EIF3I	0.419884	0.146466	-0.60969	6.609776
CCDC114	0.419884	0.14648	2.124834	1.79743
RPS5	0.420086	0.146584	-0.62415	7.483041
ENTPD1	0.420188	0.146654	2.883746	0.889149
RALGAPA1	0.420364	0.14675	-1.64471	2.766771
LSAMP	0.420599	0.146866	1.092738	5.345115
CCL21	0.420992	0.147037	2.359632	1.485471
RNF213	0.421267	0.147167	0.5552	8.98752
SNORD116	0.421417	0.147253	-1.02468	3.798553
LOC101927	0.421516	0.147322	3.568448	1.343325
SNORD31	0.42178	0.147449	-1.62661	1.980197
KIAA1671	0.421856	0.147509	-0.61233	8.445875
TLR2	0.421995	0.147604	1.391685	3.488605
SLC25A17	0.421995	0.147669	-1.10177	3.151217

PAQR8	0.421995	0.147708	0.722141	6.045077
CNDP2	0.421995	0.147722	0.880907	3.693948
SLC12A6	0.421995	0.14776	-0.96672	4.114462
PKM	0.421995	0.147763	-2.59019	1.450688
LOC645752	0.422017	0.147805	2.366122	1.498557
SFXN2	0.422079	0.147861	0.810147	3.810881
AVEN	0.422133	0.147914	0.734559	4.481885
GRAMD4	0.422388	0.148038	0.597737	6.781803
SPATA46	0.422534	0.148123	1.139106	2.647549
COX7C	0.422722	0.148226	-0.65172	7.607279
LOC101927	0.422722	0.148283	1.544997	1.889244
DDRGK1	0.422722	0.148313	0.771235	6.273136
PPP1R15A	0.422722	0.148326	-0.77369	7.677276
CREB3L2	0.422806	0.14839	0.623871	7.992319
MTERF4	0.423497	0.148685	1.367656	2.493376
NOL12	0.423497	0.148701	-0.65529	5.234292
B3GNT6	0.423526	0.148745	1.307109	3.043317
ARHGAP26	0.424023	0.148962	2.130479	2.183097
PCBP1-AS1	0.424023	0.148993	0.961004	3.378626
MSH3	0.424023	0.149023	0.543703	5.863261
MST1R	0.424133	0.149101	1.867087	0.502851
LINC01264	0.424133	0.149139	-6.50094	0.81808
ABCB11	0.424133	0.149176	-6.50094	0.81808
ZBED3	0.424133	0.149212	1.320778	2.268422
HECTD4	0.424133	0.149233	-0.58283	8.490019
PLXDC1	0.424605	0.149435	0.929419	4.606408
LINC00504	0.424605	0.149468	-2.2907	0.943365
AKR7A3	0.424649	0.149548	1.530224	4.944308
SNORD47	0.424649	0.149552	-0.9694	5.174974
RPS4X	0.424715	0.149635	-0.60449	9.681579
ZNF420	0.424715	0.149675	-1.62099	2.662138
NDUFA13	0.424715	0.149679	-0.58966	6.090224
ASPSCR1	0.424796	0.149755	2.001734	1.206385
TAOK2	0.424796	0.149776	0.709576	4.16191
PLEKHJ1	0.425077	0.14991	0.936879	3.746477
CACNA1C-/	0.425087	0.149948	1.854132	1.83167
RFWD3	0.426168	0.150363	-0.66856	5.382068
VIRMA	0.426587	0.15058	-1.13463	4.638608
RPS26	0.426587	0.15058	-1.56174	4.788134
TRHDE-AS1	0.42664	0.150634	2.57506	1.283175
TTC28-AS1	0.426694	0.1507	-1.13693	3.349941
P2RY11	0.426694	0.150722	-2.35219	1.371038
LUC7L3	0.426865	0.150831	0.664145	5.469556
FBXL7	0.426865	0.150852	1.68194	1.821536
NCMAP	0.427246	0.151069	1.880745	2.578404

SNORA3B	0.427246	0.151107	0.631972	8.080722
LINC00601	0.427246	0.15115	3.628938	0.428617
CD47	0.427246	0.151153	-3.29942	0.795138
LINC02042	0.427246	0.151159	2.862515	1.869948
OGFOD1	0.427289	0.151209	-0.57632	5.763743
ZNF711	0.427349	0.151265	0.799296	4.093095
NTN1	0.427446	0.151334	-0.7076	5.160552
NDUFA6	0.427643	0.151438	-1.31804	5.106106
YES1	0.427778	0.151545	-0.6441	7.517843
P2RX1	0.427778	0.151555	1.902055	2.359496
ITGA4	0.427873	0.151656	1.191326	4.362074
GIHCG	0.427873	0.151658	2.563997	0.947909
MIR1206	0.427897	0.151702	-3.07773	0.876413
IGSF22	0.428489	0.151946	1.576884	1.453666
AKR1B1	0.428596	0.152053	0.986631	4.006268
CCNT1	0.428596	0.152054	-2.72559	0.547556
TYK2	0.42931	0.152385	0.627165	6.046901
FZD10-DT	0.42931	0.152486	-1.83419	2.93752
SNAI3	0.42931	0.152506	1.861532	1.907592
ZNF69	0.42931	0.152529	1.876862	2.444353
HSD17B6	0.42931	0.152533	2.441328	1.754932
CCDC18-AS	0.42931	0.152548	0.668877	5.146599
KPNB1	0.42931	0.15255	-1.11351	2.821126
FLVCR1	0.429774	0.15275	0.719953	4.453807
DNTTIP1	0.429801	0.152794	0.727094	4.530132
FRMPD4	0.430011	0.152904	2.465576	1.070861
PGAM4	0.430109	0.153002	-1.12048	3.543872
CHTF8	0.430109	0.153008	1.516649	2.520461
TRIM3	0.430166	0.153063	-3.84893	0.69268
STARD4-AS	0.430212	0.153115	1.103517	3.884231
GORAB-AS:	0.430225	0.153154	1.696627	0.889152
MTSS1	0.430286	0.153211	-3.19802	1.077573
PANO1	0.430399	0.153286	-3.5003	0.69712
ZNF254	0.430434	0.153347	-2.18591	1.493916
DES	0.430434	0.153368	1.377944	7.56809
NR2E3	0.430873	0.153559	-1.83377	2.86618
SLC46A3	0.431129	0.153685	2.454291	1.717932
ATOX1	0.431247	0.153762	-1.03204	3.80721
LOC100507	0.431284	0.153811	0.882292	3.692599
KXD1	0.431296	0.15385	-2.65042	0.494152
LDHA	0.431302	0.153887	-2.49929	2.542783
WDR90	0.431468	0.153981	0.666403	4.986862
IMPA1	0.431598	0.154074	1.743664	0.737024
VAC14-AS1	0.431598	0.154097	1.929001	1.722971
TATDN2	0.432092	0.154312	-0.57587	6.118422

NDUFA11	0.432092	0.154344	-2.84323	1.279861
ENTPD1-AS	0.432186	0.154412	1.324987	2.502635
SGCA	0.432278	0.154533	-6.4375	0.769096
ZCWPW2	0.432278	0.154555	-2.03963	2.464706
SGK2	0.432278	0.154567	2.445464	1.601983
MGC7087C	0.432278	0.154585	-2.21739	1.845202
ST7-OT3	0.432303	0.154629	-2.24354	1.811345
DYNC2LI1	0.432465	0.154747	2.062456	2.407694
ENO2	0.432465	0.154789	-0.80365	4.501654
LOC72960S	0.432465	0.154824	2.698675	0.617322
ARIH1	0.432465	0.154842	-0.58563	7.146511
CBL	0.432465	0.154887	-0.64608	6.679875
NUP58	0.432465	0.154897	-3.23369	0.893925
AQP7P1	0.432523	0.154962	-0.93277	5.365436
SLC25A45	0.432523	0.154988	2.408707	1.140198
DCP1B	0.432773	0.155113	0.978629	3.52264
PLCG1-AS1	0.433145	0.155281	1.391247	2.745485
RUVBL2	0.43322	0.155343	-0.60108	5.463322
GPKOW	0.43369	0.155547	-0.70733	4.41986
TST	0.433859	0.155661	1.830174	1.796897
LINC01531	0.433859	0.1557	2.604246	1.894568
SCN4A	0.433859	0.1558	2.357378	1.123713
PRPH2	0.433859	0.155806	1.806671	2.349313
RPS6KA1	0.433859	0.155854	1.430691	2.043605
MAP2K1	0.433859	0.155891	-0.58554	6.523267
PRRT3	0.433859	0.155897	1.177064	3.079801
PLD3	0.433859	0.155899	-2.43809	1.030219
ST20-AS1	0.433859	0.155923	1.529338	2.108579
CYP27A1	0.433864	0.155961	1.271899	2.703994
DNAJB14	0.434008	0.156049	1.877563	1.226037
EID2B	0.434008	0.156117	2.64492	0.884888
COMTD1	0.434008	0.15614	-1.04169	4.18228
ICMT	0.434008	0.156153	-0.57778	6.277177
LOC10192&	0.434403	0.15633	1.453019	3.508163
RENBP	0.434643	0.156452	1.323156	2.137031
MID1IP1-A	0.434831	0.156555	1.793255	1.873334
ZNF57	0.435396	0.156793	-1.02244	3.919771
LOC10192&	0.43601	0.15705	-2.86141	1.72654
WASHC2A	0.436052	0.1571	-2.60857	0.726696
AQP7P3	0.436268	0.157214	-2.73792	0.525926
C5orf63	0.436348	0.157294	0.940303	4.187336
OXTR	0.436348	0.157313	1.951581	0.972597
SMC5-AS1	0.437118	0.157626	-2.40763	1.563087
B3GNTL1	0.437136	0.157668	1.849865	1.371006
CCNI	0.437217	0.157732	-0.56555	8.150539

PCED1A	0.437321	0.157805	1.111703	3.072653
TK2	0.437598	0.157941	1.747548	1.782338
PLAA	0.438446	0.158315	-0.60698	5.805215
OLMALINC	0.438446	0.158339	1.097814	2.59217
MANF	0.438446	0.158353	-0.60944	5.500546
MZF1-AS1	0.438481	0.158401	0.772995	4.307212
ADPRHL2	0.438666	0.158504	-0.74613	4.453203
ABCA5	0.438872	0.158614	-0.98985	4.018588
HIST1H3B	0.439113	0.158748	-0.70241	5.047004
LOC100128	0.439113	0.158772	2.145576	1.368169
CA11	0.439289	0.158895	-0.90229	4.134344
SAMD10	0.439289	0.158907	-0.97237	4.030474
LOXL1	0.439547	0.159036	1.223094	3.206687
ANKRD10	0.439697	0.159141	1.305388	2.788725
EPHA3	0.439697	0.159161	2.080516	1.172229
CRIPT	0.439854	0.159254	0.637356	5.204842
ODF2L	0.439944	0.159322	2.510732	1.151465
ARHGAP31	0.44	0.159378	0.790697	5.196367
FBXL14	0.440209	0.159489	0.867527	3.957604
TMEM192	0.440687	0.159698	-0.88783	4.106857
TTLL3	0.440901	0.159839	1.091524	2.734249
PUM3	0.440901	0.159857	-0.61928	6.597575
LINC01689	0.440901	0.15992	3.630317	0.737961
PDZD11	0.440901	0.159924	-0.82145	4.07477
IFNA8	0.440901	0.159965	-6.37115	0.718376
SELE	0.440901	0.15999	2.070904	1.985107
HYDIN2	0.441028	0.160072	1.546946	2.932356
CORO6	0.441152	0.160153	1.645527	1.963408
IPO8	0.441215	0.160241	0.794345	4.014272
OXA1L	0.441215	0.160247	0.601945	6.087453
MXI1	0.441246	0.160327	-1.42004	2.7263
ICAM3	0.441246	0.160363	0.917033	3.280416
ADGRL4	0.441246	0.160372	1.217552	3.975586
LINC02297	0.441246	0.160401	-6.35519	0.709257
RPL24	0.441485	0.160534	-0.57065	7.875107
BRK1	0.441485	0.160589	-0.61334	6.0932
DUOXA2	0.441485	0.160615	-1.14193	4.32076
PWWP2A	0.441485	0.160631	0.68632	4.3472
NUPL2	0.441514	0.160677	0.750008	4.50796
COA3	0.441861	0.160839	-0.83098	4.6676
PI4KAP2	0.441949	0.160907	-0.68425	4.628573
SNORA71E	0.442138	0.161037	0.972082	5.147886
FAM184B	0.442138	0.161055	-1.16754	3.456225
ARFGAP3	0.442138	0.161083	2.881837	1.105432
LINS1	0.442329	0.161205	-1.20821	4.037384

SNORD3C	0.442329	0.161225	0.736656	8.466785
ALDH1A3	0.442501	0.161325	-1.81261	2.755666
ZDHHC7	0.442501	0.161359	1.909028	1.134767
TBX15	0.44251	0.161398	2.814715	0.62298
NUCB1-AS1	0.442574	0.161458	-2.30934	0.483304
ABCA8	0.442832	0.161587	2.35403	1.073312
KDELR1	0.443449	0.161848	0.527619	7.194825
SESN1	0.443614	0.161971	1.148506	3.264402
EMX2OS	0.443614	0.162032	2.70464	1.008132
LINC00696	0.443614	0.16207	-2.17725	2.116063
FGF9	0.443614	0.162098	2.737011	0.626368
SLC36A1	0.443614	0.162118	0.692077	4.773125
BBS10	0.443614	0.162128	-0.92785	3.863338
HSPB6	0.443614	0.16219	1.037411	4.037779
EPN2-IT1	0.443614	0.162222	-1.32906	3.248876
ZHX1-C8or	0.443614	0.162262	-2.79434	0.592979
ZFC3H1	0.443614	0.162268	-0.59413	8.383758
ARC	0.443634	0.162342	-2.64642	1.660003
ZC3H12B	0.443634	0.162352	1.374646	3.173386
EIF5A2	0.443634	0.162393	-2.16427	1.83907
WTIP	0.443634	0.162448	1.429694	2.709625
CHRM3-AS	0.443634	0.162455	1.900819	2.432188
ZNF292	0.44378	0.162545	-0.58306	7.844902
NCF1C	0.443784	0.162582	1.59681	1.548908
AMOT	0.443972	0.162687	1.565459	2.006709
CBX7	0.443987	0.162728	0.721918	5.243825
SPHK2	0.444254	0.162862	2.010824	1.065845
SPATA12	0.445184	0.163239	-0.81924	3.835463
MFSD13A	0.445597	0.163427	1.46826	2.457208
MIR4453H	0.445773	0.163533	1.024817	3.673516
LHFPL3-AS	0.445773	0.163585	2.551784	2.231952
SEC14L2	0.445773	0.163599	-1.79569	2.568975
ARHGAP5-/	0.446099	0.163755	-1.78452	2.506907
MRPS9	0.446196	0.163829	-0.66532	5.0209
KLHL24	0.446196	0.163863	0.571194	6.836149
PREP	0.44692	0.164165	0.59042	6.161216
KCTD13	0.447196	0.164327	2.545667	0.978291
EHD4-AS1	0.447196	0.164403	-1.89949	3.071545
COPG2IT1	0.447196	0.164409	0.794575	4.072822
RPL7L1	0.447196	0.164411	-0.7438	5.505241
SAC3D1	0.447201	0.164453	1.076827	2.927573
RARS	0.447201	0.164522	-0.59486	6.235304
SMAD2	0.447201	0.164552	1.287762	1.794264
L2HGDH	0.447201	0.164558	-0.73629	4.621373
TNK2	0.447708	0.164781	-0.86113	4.123874

PLA2G4E-A	0.447758	0.164835	-2.46399	3.51484
STC2	0.448082	0.165016	-2.04969	2.249918
GUF1	0.448082	0.165027	-0.53036	6.012874
PTGES2-AS	0.448322	0.165169	1.756289	1.941487
RORB	0.448322	0.165188	2.037888	2.445196
INTS4P2	0.448373	0.165244	1.007514	3.592626
WNT4	0.448508	0.16533	-0.83563	4.596049
TTC24	0.44891	0.165519	2.411544	1.152889
LOC101928	0.44891	0.16555	2.515907	0.822184
DENND3	0.449052	0.165639	0.792334	5.255003
JAKMIP3	0.449062	0.165681	1.628651	2.10414
TERF2	0.449062	0.165716	0.577058	5.456099
NDUFS6	0.449098	0.165765	-0.57169	5.467781
SNORD14E	0.449597	0.165986	1.371327	2.643915
EPHX1	0.449957	0.166159	-1.69797	2.150635
LOC100131	0.449957	0.166204	1.529149	3.244587
ZNF701	0.449957	0.166228	1.544692	2.047683
TLX1NB	0.450841	0.166627	-6.3016	0.665793
ATP6V0B	0.450841	0.166628	-1.51265	2.309296
RPS6KA6	0.450927	0.166696	-1.57772	3.109254
HTT	0.451025	0.166769	-0.53808	8.035337
LINC01873	0.451176	0.166861	-2.20142	1.664793
SCGB2B3P	0.451846	0.167153	2.670273	0.469515
TIPRL	0.451846	0.167182	-0.69933	4.623511
SOX10	0.451914	0.167244	1.207661	2.354081
SNW1	0.451994	0.16731	-0.57407	6.464029
RPS25	0.452317	0.167467	-0.55018	7.692844
HCLS1	0.452544	0.167587	2.19207	1.691536
ZACN	0.452851	0.167754	-2.64844	0.906029
MIR17HG	0.452851	0.167774	-1.66239	3.059331
RPL19	0.453561	0.168074	-0.54309	9.566114
COX8A	0.453832	0.168211	-0.52799	5.564713
SPRN	0.45385	0.168255	0.813961	4.107429
ANAPC13	0.454125	0.168393	1.4592	1.795591
TMEM164	0.454145	0.16844	0.97255	3.362341
SEH1L	0.454145	0.1685	-1.05204	3.85334
SUN2	0.454145	0.168511	-1.76271	2.153399
HRH2	0.454232	0.168582	2.451878	0.809386
EMILIN3	0.454232	0.168617	2.246692	1.701445
MAST4	0.454415	0.168722	-1.54072	2.621339
C5orf17	0.454451	0.168793	-3.03756	1.196766
PLPP5	0.454451	0.168809	0.791107	4.163095
ANKRD20A	0.454464	0.168876	1.193565	2.820024
SIX5	0.454464	0.168947	0.812719	3.821341
APLN	0.454464	0.168953	1.245771	2.462662

UTP4	0.454464	0.168966	-1.219	3.694699
ZNF532	0.454464	0.168997	-0.64393	6.17256
IL7R	0.45457	0.169074	2.186351	1.060286
TMEM181	0.455297	0.169389	0.505336	6.743844
TAB2	0.455297	0.169418	-1.39346	2.401398
KIF1C	0.455552	0.16955	-0.58741	9.087899
PLA2R1	0.455634	0.169629	-1.72643	2.575784
CYP2B6	0.455634	0.169698	2.519213	1.501883
RAP1A	0.455634	0.169747	1.52849	1.459729
ANKRD61	0.455634	0.169785	1.164126	3.153692
PLEKHA8P1	0.455634	0.169804	1.349079	2.239455
RTKN	0.455634	0.169842	1.159086	2.74401
SNAP47	0.455634	0.169884	0.859042	3.720544
PNMA2	0.455634	0.169909	1.680312	2.642986
DIAPH2-AS	0.455634	0.169912	1.519149	2.484708
ETHE1	0.45603	0.170138	0.537334	5.24923
CDS2	0.45603	0.170147	0.580117	6.423109
PLPPR2	0.45603	0.170171	-3.93776	0.651755
YTHDC2	0.456457	0.170367	-0.53648	6.712349
ARNTL2-AS	0.456476	0.170424	-1.27482	3.136818
GLI2	0.456476	0.170448	1.083237	2.960957
1-Mar	0.4566	0.170542	1.014567	3.715915
RFXAP	0.4566	0.170569	0.938627	3.581995
KCTD4	0.456847	0.170725	-1.92846	2.451255
RPL7A	0.456847	0.170735	-0.5648	8.904915
NBR1	0.456899	0.170791	2.138652	1.503108
BTG1	0.456932	0.170841	0.530941	7.350895
RRS1	0.456934	0.170878	-0.7204	4.444302
LONRF1	0.456948	0.170966	-1.08645	5.100601
VWA5B2	0.456948	0.17098	1.905029	1.360915
EHD4	0.456948	0.17107	-0.68763	6.211473
RPL23	0.456948	0.1711	-0.59058	8.875752
OBSCN	0.456948	0.171114	-0.84687	4.748866
TROAP	0.456948	0.17112	1.106431	2.831962
SLC35F2	0.456948	0.171178	-0.71906	5.242494
GBAP1	0.456948	0.171191	0.800886	4.003709
TRPV1	0.456948	0.171217	1.760897	1.370417
OXR1	0.456948	0.171335	-1.44349	2.320963
SNORA73A	0.456948	0.171339	-0.6123	9.282202
CTAGE6	0.456948	0.171363	-2.6538	0.757112
MRPL36	0.456948	0.171365	-1.01428	3.044348
KLK7	0.457476	0.171637	-2.23452	1.3521
CD3E	0.457476	0.171712	1.262622	3.021818
HOXA3	0.457476	0.171722	2.099224	0.515444
ZNF852	0.457476	0.171745	0.853943	3.878308

LMNTD2	0.457476	0.171748	1.058274	2.850034
CNN3	0.458101	0.17202	-1.91758	2.343541
CSNK2A3	0.458518	0.172244	-0.69018	4.863442
LGALS9C	0.458518	0.172262	-1.28781	2.607262
PDK4	0.458518	0.172333	1.137508	8.074785
DUXAP9	0.458518	0.172349	1.525624	3.127176
FAM160B1	0.458518	0.172364	-1.20221	4.13886
SNORD116	0.458518	0.172488	-0.95149	5.818973
UFM1	0.458518	0.172501	1.241557	2.523908
RAPGEFL1	0.458518	0.172557	-0.92922	4.756669
ADGRB3	0.458518	0.172691	2.030976	1.42292
RAI1-AS1	0.458518	0.172701	-1.44051	3.064534
PEX14	0.458518	0.172718	-0.69514	4.846256
TSHR	0.458518	0.172746	1.887355	2.140952
GTF2IP1	0.458518	0.172785	-0.63113	5.552901
LINC00200	0.458518	0.17284	2.528997	0.730249
FNDC1	0.458518	0.17287	1.336746	3.098294
LINC01004	0.458518	0.172901	0.539588	6.914264
MIR4426	0.458518	0.172945	-0.56957	6.978038
WNK2	0.458518	0.17296	1.602856	1.683571
THRA	0.458518	0.172986	1.095545	3.549759
MAN1B1-D	0.458518	0.173016	-2.22171	0.481644
TBX5-AS1	0.458518	0.173034	1.943916	1.522888
CCDC130	0.458518	0.173054	0.67175	4.631749
PKN2-AS1	0.458518	0.173092	-2.78302	1.064537
NSUN3	0.458518	0.173115	1.402939	2.885012
TMEM87A	0.458518	0.173137	0.831153	4.060208
MRPS16	0.458518	0.173142	-0.5636	5.746827
SIRT5	0.458619	0.173217	-2.35046	1.207056
PJA2	0.458642	0.173286	-0.60634	7.838265
CCDC88B	0.458642	0.173364	0.64822	5.220874
PLAC1	0.458642	0.17337	-6.20332	0.592535
ZNF687	0.458642	0.173398	-2.4078	1.364491
LINC01279	0.458642	0.17347	1.074571	4.255541
RP1L1	0.458642	0.173495	-1.48992	3.11479
LINC00858	0.458642	0.173523	2.443746	1.265481
HS2ST1	0.458642	0.173523	-0.70422	5.162484
LOC101241	0.458715	0.173646	1.935709	1.797775
RORC	0.458715	0.173654	2.035497	2.453758
ATXN7L3B	0.458715	0.173662	0.549252	6.82028
RN7SL2	0.459093	0.173907	-0.7173	14.59183
AKT1	0.459093	0.173914	1.626971	0.99579
ADCY1	0.459093	0.173972	1.321686	2.854165
PSORS1C3	0.459093	0.173988	2.459765	1.111018
MAP1LC3B	0.459093	0.173991	-1.43801	2.348033

APPL2	0.459106	0.174033	1.871157	1.584005
CENPV	0.459309	0.174174	1.322671	2.810679
TRIM56	0.459309	0.174185	0.524204	6.732677
SMARCD2	0.459619	0.17434	-0.55665	6.746726
LARP1	0.460386	0.174703	-0.53255	8.94834
EIF2B1	0.460386	0.174791	-0.54311	5.343265
WNT9A	0.460386	0.174795	-1.89504	1.800326
STAU2-AS1	0.460386	0.1748	1.697211	1.571347
FANCF	0.460386	0.174835	0.790274	3.649113
LOC101929	0.460386	0.174854	-1.44104	2.833993
SLED1	0.460554	0.174955	-1.68223	2.047166
WDR13	0.460653	0.175062	0.926751	3.090553
CEL	0.460653	0.175068	-2.2156	0.763333
SNORA5C	0.460921	0.175207	1.084111	2.740824
NAPSA	0.461725	0.17555	2.893713	0.501574
NHS-AS1	0.462037	0.175706	1.927178	1.671114
PTPN3	0.462156	0.175789	-2.572	1.463543
MED12L	0.462255	0.175894	1.315301	2.495552
TCTA	0.462255	0.175901	0.663249	4.258462
NKD1	0.462503	0.176055	0.924993	4.352439
FOXO3B	0.462503	0.176113	-0.58041	5.86811
LINC00997	0.462503	0.176113	0.70687	3.835413
GMDS-DT	0.462503	0.176145	0.923018	3.025332
EEF2K	0.462644	0.176236	0.531888	6.512236
MAP3K11	0.46348	0.176592	0.502051	5.530098
HS3ST1	0.463756	0.176747	-1.02679	4.110315
TRMT12	0.463756	0.176773	0.963006	3.956353
ESYT2	0.464123	0.17695	-0.55551	7.721223
SYNGR2	0.464344	0.177112	0.540251	6.660915
RUFY2	0.464344	0.177143	-1.16811	3.66509
OTULIN	0.464344	0.177147	-0.61158	6.369041
SNORA33	0.465197	0.177511	-0.74603	5.637008
HIST1H3J	0.465225	0.177559	-1.40405	2.919212
NDUFA1	0.465237	0.177613	-0.53797	5.626278
RTP4	0.465237	0.177639	2.041678	1.416056
FGR	0.465245	0.17768	2.231823	1.546321
SNORD116	0.466057	0.178027	-0.86635	5.881166
PCAT19	0.466354	0.178205	1.439328	1.917258
CHPF2	0.466354	0.178226	0.803862	4.106498
WDR41	0.466354	0.178289	-0.54258	6.103563
LINC00927	0.466354	0.178292	-6.13939	0.548725
LOC100507	0.46643	0.178358	0.744683	4.158131
CA2	0.466562	0.178447	1.920062	1.337746
STXBP5L	0.46686	0.178632	-6.12495	0.535019
MRPS28	0.46686	0.178636	-0.93942	4.115671

MAGT1	0.467066	0.178753	-0.52711	6.728277
FBXO8	0.467291	0.178893	-0.81508	4.232808
LCAT	0.467291	0.178924	1.41969	3.152279
OSBPL5	0.467291	0.178953	2.768235	0.674657
CEP63	0.467313	0.178999	1.936122	1.884789
FLG2	0.467626	0.179171	-1.84629	2.998523
PTP4A1	0.467626	0.179195	-0.66444	7.769
AP1S1	0.467714	0.179266	0.620784	4.742365
GRIN3A	0.468061	0.179437	1.625352	1.919711
TMEM200(	0.468234	0.17957	2.993816	0.468794
PLA2G16	0.468234	0.17958	2.82797	0.795463
LINC01022	0.468238	0.179619	2.691713	0.806053
CTAGE9	0.468338	0.179695	-2.14486	1.121812
SNHG11	0.468737	0.179886	1.217727	2.554058
PAPPA	0.468771	0.179937	0.82333	7.309578
ID2	0.469061	0.180087	0.835503	4.346585
FAM209A	0.46948	0.180393	-2.6295	0.617111
KCNV2	0.46948	0.180433	-1.58942	2.188631
NUS1	0.46948	0.180468	-0.68203	5.748464
ANKAR	0.46948	0.180495	0.903	3.828089
QRICH2	0.46948	0.180498	-1.27362	3.385921
GLB1L2	0.46948	0.180502	1.292999	3.80108
CRISPLD1	0.46948	0.180543	-2.85838	1.198902
GPR151	0.46948	0.180567	-6.11329	0.529721
SMIM20	0.46948	0.18059	0.845326	3.628821
SYPL2	0.469702	0.180726	2.521822	0.655339
ADAMTS1	0.469702	0.18078	0.788031	6.755888
ARSB	0.469702	0.180789	1.325245	2.933011
CYYR1	0.469735	0.180868	1.395761	3.384199
THCAT155	0.469735	0.180878	2.67142	1.252331
SHC2	0.46976	0.180926	1.222135	3.127652
SNORA21	0.470002	0.181058	0.636937	8.64435
USP12-AS1	0.470002	0.181107	1.39194	2.070363
CIDECP1	0.470002	0.181146	-1.90083	1.213684
KIAA0556	0.470002	0.181171	-0.5995	6.266115
LRP2BP	0.470027	0.181219	-0.83149	4.144052
C2CD5	0.470127	0.181296	2.074512	1.521709
STK17A	0.47068	0.181547	-0.5686	6.043698
NOA1	0.47124	0.181801	0.558288	5.055081
GANC	0.471281	0.181862	-0.60467	5.608263
IGSF9B	0.471281	0.181893	1.301188	2.254761
SLC9A4	0.471569	0.182043	-1.74056	4.362828
RPL29	0.472392	0.182399	-0.6061	7.590994
RFX3	0.472681	0.182548	2.133049	1.312634
VPS13D	0.473232	0.1828	-1.87715	0.938156

CTAGE15	0.473488	0.182945	-2.84665	0.61633
TRIM52	0.473488	0.182975	0.664916	4.602766
SELENOT	0.473616	0.183087	-0.52804	5.669198
CCDC146	0.473616	0.183101	-0.60114	5.246075
INPP4A	0.474254	0.18339	1.667251	1.620757
ABHD15	0.474254	0.183425	0.876836	3.52741
MSR1	0.475049	0.183771	1.8751	1.680166
MYO5B	0.475588	0.184018	-0.56408	8.42092
PIEZO1	0.475606	0.184063	-0.5249	7.626761
SDHB	0.475616	0.184106	-0.58024	5.545072
C2orf27A	0.476062	0.184317	-3.71391	0.96038
RAB1B	0.476254	0.184433	-0.54199	7.210776
SH2D6	0.476254	0.184468	2.465238	1.079066
SPATA24	0.47643	0.184577	1.596025	1.793702
SNORA50A	0.47643	0.184614	-1.28927	3.452225
LINC02210	0.47655	0.184699	-1.25554	3.646594
ST6GALNA(	0.476632	0.184769	2.485213	0.672554
GNPAT	0.476766	0.18486	1.589597	1.582592
RNF5P1	0.476811	0.184931	1.656501	1.373486
RBM15	0.476811	0.184955	0.974138	2.801461
KCNJ2	0.477412	0.185226	-0.97505	3.565152
LINC02502	0.477421	0.185269	-6.04208	0.475092
SCYL2	0.477515	0.185344	0.992155	2.641043
YARS	0.477528	0.185387	-0.60399	6.763448
AQP11	0.4785	0.185803	1.164952	2.78171
PSD	0.478715	0.185926	2.97004	0.535252
SOCS3	0.478796	0.18603	-1.21864	6.05621
BSG	0.478796	0.186038	-2.33787	1.061549
LINC01572	0.478796	0.186091	0.909755	3.530127
ANKRD17	0.478796	0.186115	0.624626	4.443037
PHLDB1	0.478796	0.186151	2.457809	0.775026
OLFM5P	0.478799	0.186196	2.597758	0.606177
SLC25A40	0.478799	0.18623	-0.76023	4.316758
ZC3H3	0.478977	0.186338	-0.9048	4.089322
RNF148	0.479248	0.186482	2.215313	1.226404
IFNK	0.480188	0.186892	1.275389	2.704514
LOC101927	0.480188	0.186925	-1.68566	2.543727
RHOT2	0.480797	0.187202	0.602045	5.906326
LINC01235	0.480831	0.187257	2.152626	0.968336
MGC16025	0.480831	0.187293	-1.39794	2.631575
EGR4	0.481115	0.187442	-3.23109	0.611706
SPOUT1	0.481138	0.18749	0.570709	5.249599
C16orf70	0.481248	0.187589	-1.10821	4.182779
ADGRF2	0.481248	0.187611	-1.66771	2.778062
BNIP2	0.481567	0.187807	-0.54152	6.014665

WHAMMP:	0.481567	0.187813	-1.81483	2.449386
TMEM120I	0.481865	0.187969	0.596764	4.675588
SLC35A4	0.481935	0.188035	-0.51122	5.818751
HIST1H1C	0.482311	0.188221	-0.54146	6.446348
LOC28378ɛ	0.482313	0.188278	0.70472	5.741587
DNHD1	0.482313	0.188334	0.546189	5.631866
IFI44	0.482313	0.18835	1.104825	4.338136
FKBP3	0.482313	0.188378	-0.57636	5.782425
TWSG1	0.482574	0.188519	0.554452	5.343329
LINC-PINT	0.482711	0.188611	0.988647	4.13682
TGFB3	0.482891	0.188721	0.724407	5.028909
AGFG2	0.482919	0.188819	-0.62279	6.169076
ACAD8	0.482919	0.188848	0.672593	4.304319
CRYM	0.482919	0.188856	-1.4614	2.312682
NFYC-AS1	0.482919	0.188888	-1.27431	2.72543
SPATA1	0.483017	0.188966	1.259733	2.905896
THRAP3	0.483273	0.189105	-0.53477	8.734789
ZSWIM6	0.483339	0.18917	-0.54861	6.538032
TMEM75	0.483813	0.189395	-0.78893	4.762991
SMAD4	0.483864	0.189454	0.495832	6.81867
SNORD13	0.484545	0.18976	0.667132	6.452461
DIS3L2	0.484575	0.189884	-1.66152	2.073665
UBE2B	0.484575	0.189955	-0.52969	5.80861
ASMTL-AS1	0.484575	0.189962	1.629509	1.208872
UBA2	0.484575	0.190014	-0.51722	7.047022
TNIK	0.484575	0.190047	2.465695	1.285109
TMED7	0.484575	0.190052	-0.57013	6.548885
TRAF4	0.484575	0.190062	0.660651	5.128683
SNORD10	0.484575	0.190092	-0.58899	10.44133
SH3BP5-AS	0.484575	0.190125	1.037972	4.207811
DNAJC2	0.485287	0.190444	-2.23191	1.136312
PFDN4	0.485287	0.190525	-1.09002	3.655541
TOPBP1	0.485287	0.190557	-0.56257	6.189011
TXN2	0.485287	0.190604	-0.57717	5.457137
SSTR1	0.485287	0.190635	1.67399	3.666719
TEF	0.485287	0.190641	2.19795	1.083004
PIP4P2	0.485287	0.19068	1.683067	2.635548
PSORS1C2	0.485287	0.190786	2.41142	0.815869
FGD4	0.485287	0.190801	1.042166	2.901498
ZNF224	0.485287	0.190831	1.091383	2.535701
MRPL1	0.485287	0.190837	-1.01817	3.860059
RFTN2	0.48551	0.191001	1.037629	3.762368
TDP2	0.48551	0.191003	0.514223	5.285691
CELF2-AS2	0.485679	0.191147	1.554499	1.962433
ZNF134	0.485679	0.191148	-1.26741	3.653624

ZBTB37	0.485846	0.191282	-1.4711	2.114424
HSPA14	0.485846	0.191315	-0.72346	4.19916
MUTYH	0.485846	0.191332	2.934834	0.518406
CBR3-AS1	0.485901	0.191393	-1.30977	2.703849
ZNF519	0.485901	0.19145	-1.12624	3.74037
FBXO48	0.485901	0.191472	-2.0569	1.545339
ECHDC3	0.486022	0.191559	-1.73353	2.866583
CLCF1	0.486175	0.191678	-2.10333	0.81243
PIGP	0.486175	0.191698	1.785093	0.90865
FAM53B	0.486532	0.191878	0.522614	6.292278
HAS2	0.486988	0.192097	1.333966	2.561416
GTPBP4	0.487419	0.192307	-0.60527	6.137288
TIA1	0.48748	0.192423	2.504792	0.985926
TUBB1	0.48748	0.192443	1.469524	1.785285
FDX2	0.48748	0.192462	1.113142	2.514337
LBHD1	0.48748	0.192489	1.267741	2.208542
PDCD11	0.487646	0.192594	-0.518	6.683666
POLG2	0.487646	0.192633	0.773295	3.693276
TFAP2B	0.487697	0.192747	-2.14413	2.315827
ZNF493	0.487697	0.192765	1.653273	1.966196
HIST1H4A	0.487697	0.192772	-1.30927	3.35037
SNORA79	0.487746	0.192831	-1.50946	2.277128
CDC42BPB	0.487789	0.192894	-0.51238	8.611521
KLHL28	0.487789	0.192927	1.253229	2.019304
OCLM	0.487934	0.193024	-1.95868	2.710793
C15orf53	0.488004	0.193112	-2.59352	0.461221
CS	0.488004	0.193133	-0.57865	7.567458
KDR	0.488004	0.19317	0.700872	4.994883
LAMC2	0.488184	0.193286	0.910015	4.489908
TCFL5	0.488184	0.193326	-1.30642	2.642011
EFR3B	0.488184	0.193447	1.243543	2.261867
MACROD2-	0.488184	0.193468	1.870429	1.679987
JRK	0.488184	0.193478	-0.6861	4.679616
PALM3	0.488184	0.193478	-1.82391	5.015809
POU5F1	0.488269	0.193552	2.870996	1.72451
SLC25A15	0.488713	0.193767	0.690924	4.180074
MBNL2	0.489422	0.194119	2.032347	1.068381
ITGB1	0.489422	0.194163	1.545624	1.64605
CAVIN3	0.489422	0.194188	1.73892	2.229552
FAM50B	0.489422	0.194207	1.445081	1.848132
STK4	0.489546	0.194296	0.499973	7.011968
LOC72973Z	0.489719	0.194404	0.638498	5.083579
MIR3173	0.489821	0.194484	-2.77431	0.784239
CCR1	0.490005	0.194631	1.374688	2.314599
PRCC	0.490005	0.194661	-0.51283	5.58719

FAAH	0.490005	0.194676	0.766692	4.131931
TMED2	0.490228	0.194805	-0.53628	8.389937
LINC02381	0.490299	0.194873	-1.27079	3.175989
RNF183	0.490415	0.194958	1.686182	1.95945
HCG4B	0.490916	0.19522	1.702054	1.907394
FAM186A	0.490916	0.195292	1.490121	1.959232
LRFN4	0.490916	0.195292	0.81275	3.901546
SNORD133	0.490916	0.195317	-0.58424	8.511208
FOXQ1	0.490938	0.195365	0.62005	5.398555
AOAH-IT1	0.491154	0.195491	1.961637	1.112619
DIRAS1	0.491155	0.195531	1.341636	2.753071
SUCLG2	0.491292	0.195625	0.609682	5.511747
FOXCUT	0.491865	0.195893	1.678091	1.842293
DDI1	0.491886	0.195957	3.329668	0.538645
RNASEH2B	0.491886	0.195981	1.98112	0.74008
MUS81	0.492052	0.196088	0.606622	4.541239
S100A13	0.492247	0.196205	-1.15492	2.570396
FH	0.492438	0.196321	-0.58993	5.385076
ADSS	0.492731	0.196586	-0.55975	6.021269
AK1	0.492731	0.196627	0.643224	4.836927
SPRED1	0.492731	0.196651	0.591166	5.804164
SLC25A33	0.492731	0.196784	1.260504	2.194971
MIR4668	0.492731	0.196785	-2.5472	1.766068
PGBD4	0.492731	0.196787	-1.82731	1.67787
TLL2	0.492731	0.196791	1.54994	2.486985
BMI1	0.492731	0.196792	1.558844	1.550935
RSAD2	0.492731	0.196799	1.513073	2.216221
ELOVL7	0.492731	0.196837	2.631823	1.197911
ATP6V1B1	0.492966	0.196971	1.679403	1.126615
IL10RB	0.493025	0.197034	-0.62819	5.106695
SEMA6A-A:	0.493374	0.197214	1.561737	1.980584
PGBD5	0.493395	0.197265	1.304512	2.20276
URAHP	0.493395	0.197339	-2.01889	1.926082
ZC3H7A	0.493395	0.197367	0.50993	6.0768
ITPKB-IT1	0.493395	0.197382	1.558963	1.797671
FKBP1AP1	0.49416	0.197774	0.627984	4.404033
AGRP	0.49416	0.197791	1.537363	1.762289
CHKA	0.49416	0.197838	2.704227	1.318052
TREML2	0.49416	0.197848	2.283234	0.988877
SNORA23	0.494225	0.197914	-0.52049	8.134815
HTRA3	0.494389	0.19802	1.579104	1.780846
DTD2	0.494454	0.198086	-0.73654	4.51126
RNF112	0.494622	0.198193	1.743974	1.768399
ARL10	0.494644	0.198242	-1.24465	3.559339
SURF4	0.494671	0.198293	1.440278	1.077961

TMEM201	0.494821	0.198393	-0.92314	3.566196
NACC2	0.49506	0.198529	0.513295	5.340633
KCNK1	0.495285	0.198692	-0.72455	5.261922
QRSL1	0.495285	0.198702	0.648038	4.756741
MTMR9LP	0.495285	0.19874	1.392789	3.34192
SKP1	0.495412	0.198831	-1.43849	2.166233
PSMD3	0.495685	0.198981	-0.52489	6.090556
SIDT2	0.496269	0.199255	0.617953	5.652371
HDHD2	0.496389	0.199344	-0.57098	5.295638
RNA5S9	0.49648	0.199475	-0.95806	8.850654
JMJD8	0.49648	0.199573	-0.90136	3.899438
APPL1	0.49648	0.199575	-0.52555	7.126764
PBX2	0.49648	0.199601	0.865141	4.088434
NCOA5	0.49648	0.199644	0.508074	5.657683
NFIA	0.49648	0.199664	1.62752	0.940915
MYLK-AS2	0.49648	0.199667	2.315768	1.037772
DUSP10	0.49648	0.199713	-1.70121	3.081506
YME1L1	0.49648	0.19977	1.601991	1.227655
ZYG11B	0.49648	0.199827	-0.53512	6.136658
DCLK1	0.49648	0.199887	1.252788	2.473874
SOD2	0.49648	0.199915	-0.63104	6.654933
BLID	0.49648	0.199941	2.641921	0.461159
SPSB1	0.49648	0.199943	-0.64292	4.924612
MCCC1	0.49689	0.200155	-1.80114	1.503919
LOC28333	0.49689	0.200215	0.899997	3.491546
EXTL3-AS1	0.49689	0.200229	-3.7121	0.441248
RNA18SN5	0.496999	0.200313	-0.64774	12.11909
LOC105447	0.497017	0.200374	1.982267	1.609672
SMIM15	0.497017	0.200401	-0.49033	5.847699
RNU12	0.497145	0.200493	0.711545	7.390204
FBXO7	0.497223	0.200565	1.693105	1.586247
RPL27A	0.497476	0.200707	-0.54343	9.024354
UCA1	0.49763	0.200845	-1.99306	1.940485
IL11RA	0.49763	0.20085	1.971832	1.3899
LOC102724	0.497838	0.200974	-2.14009	1.031936
SNORD12	0.498186	0.201155	0.634873	5.608663
KDM4A	0.498293	0.201238	0.501805	6.431192
ALG11	0.498621	0.201411	0.917393	3.69974
ZNF783	0.49898	0.201629	0.811621	4.526657
CRADD	0.49898	0.201637	-1.12327	4.099298
LOC101927	0.499154	0.201748	1.842277	2.55612
ZNF790	0.499564	0.201954	-1.20318	2.535813
SNORA75	0.499812	0.202095	-0.7289	5.017098
EIF5B	0.499843	0.202186	-0.51123	8.901927
LOC101929	0.499843	0.202219	2.567438	0.527301

NUDT14	0.499843	0.202235	0.802985	3.529697
TUG1	0.499843	0.202269	-1.44127	3.122322
SNX9	0.499862	0.202317	-0.54232	7.266064
PARGP1	0.500034	0.202439	0.639111	4.359029
PRKCB	0.500034	0.202475	1.260193	3.457468
SH3RF3-AS	0.500034	0.202509	1.648929	1.690356
TNFRSF10A	0.500048	0.202582	-0.83746	4.426714
PHKB	0.500048	0.202595	-2.26361	1.125961
LOC101928	0.500125	0.202667	1.306456	2.583833
TTF1	0.500633	0.202933	0.761994	3.633265
FRAT1	0.500633	0.202954	1.221737	2.613114
HSPA1B	0.500635	0.202995	-1.43741	4.509602
OPLAH	0.501179	0.203256	0.694911	5.348286
DYNC1LI1	0.50119	0.203302	0.54566	5.614915
NLGN2	0.501405	0.203436	0.671677	4.526645
UQCC2	0.501405	0.203492	-0.72755	4.452584
C2orf48	0.501405	0.20351	-1.98063	1.446546
CSTF3	0.501456	0.203572	0.615636	4.665317
ACVR2B-AS	0.501469	0.203618	1.515029	2.059307
JPT2	0.501549	0.203691	-0.55806	6.767866
DVL2	0.501571	0.20374	-0.58461	5.185407
NMNAT1	0.501721	0.203842	2.087835	0.620638
FJX1	0.501981	0.203988	-1.98723	1.621266
IFT172	0.502224	0.204166	0.525919	5.634948
VGF	0.502224	0.204168	2.279966	0.863985
MYL12A	0.502627	0.204373	1.336114	1.973499
PDXP	0.502842	0.204515	1.266166	2.101088
FAXC	0.502842	0.204549	-1.48958	2.778512
DRICH1	0.502842	0.204615	-2.28281	1.279797
SPAG7	0.502842	0.204623	-0.53838	5.514334
DGKH	0.502867	0.204674	-0.96019	3.012181
PCP4	0.503246	0.204884	2.985149	0.911776
PKD2	0.503246	0.20491	0.612895	5.398778
ZNF252P-A	0.503272	0.204961	1.454045	2.25673
ERI3-IT1	0.503339	0.205029	0.911819	2.713025
SNORD81	0.503386	0.205127	-2.49604	1.132508
BRIX1	0.503386	0.20513	-0.81977	4.574783
CBX5	0.503583	0.205251	1.270522	2.274623
HTR3B	0.503858	0.205426	-3.81922	0.515328
PDIA2	0.503858	0.205445	-2.09135	4.433342
ACTR3	0.504262	0.205688	-2.42622	1.096107
NUDCD3	0.504262	0.205691	-0.48711	6.906635
ACSF3	0.504477	0.20582	-2.50649	0.649567
C19orf38	0.50565	0.206339	2.638176	0.724609
AHSA2P	0.505847	0.206479	0.517543	6.448838

BTBD1	0.505847	0.206502	-2.65559	0.799575
ASCC2	0.506053	0.206655	-2.21822	0.726285
COX6B2	0.506053	0.206668	-2.86588	0.959356
PCDHB5	0.506272	0.206825	1.857009	1.198427
USP38	0.506272	0.206839	2.384014	1.56201
COL24A1	0.506299	0.206894	1.6424	2.519976
UBE2S	0.506299	0.206932	0.864265	3.599368
FDFT1	0.506739	0.207153	0.744551	4.100485
RUFY1	0.50704	0.207317	1.189666	2.70767
GLMN	0.507059	0.207388	0.796772	4.073468
SMG9	0.507059	0.207447	0.5819	4.601484
COX7A2	0.507059	0.207467	1.825837	1.379248
MAP3K8	0.507059	0.207504	-1.37945	2.238632
HCFC2	0.507059	0.20753	-0.50932	5.85239
FAM107B	0.507115	0.207634	2.090108	0.890341
KANSL2	0.507115	0.207635	-0.70076	4.259187
LOC284581	0.507285	0.207746	1.158341	2.95533
APBB1IP	0.507407	0.207837	0.972743	3.773322
ZNF749	0.508161	0.208187	0.88201	3.743843
SNORD116	0.508366	0.208397	-0.94462	3.990335
NOTCH1	0.508366	0.20842	-0.61015	6.717242
SPSB3	0.508366	0.208455	-0.56186	5.567628
FBXO36	0.508366	0.208573	-1.38324	2.218992
RAB11B	0.508366	0.208582	-0.47821	5.8626
RNASEL	0.508366	0.208596	0.544632	4.958946
ASPN	0.508366	0.208607	1.809494	1.171712
METTL7A	0.508366	0.208623	0.679065	6.916763
ESR2	0.508366	0.208642	1.608691	1.163659
MSL1	0.508534	0.208775	-0.51794	7.223362
FAM86C2P	0.508534	0.208861	-1.32579	2.614792
MMRN2	0.508534	0.208879	0.857968	4.365649
ZNF266	0.508534	0.20888	2.013446	0.692038
CCDC32	0.508534	0.208917	2.078398	1.147631
ZBED6CL	0.508801	0.209091	0.809681	3.327095
BCLAF3	0.508801	0.209109	0.462799	5.707823
FANCE	0.508822	0.209159	-1.12404	3.606026
METTL27	0.509037	0.209369	-2.39811	0.534901
LHX4-AS1	0.509037	0.209387	1.051395	2.49493
WDR53	0.509037	0.209535	-1.16402	3.274333
CRTAP	0.509037	0.209537	0.494869	6.745419
MX2	0.509037	0.209542	-0.68341	4.773686
TXNL4B	0.509037	0.209551	1.504575	2.194414
TMEM158	0.509037	0.209569	1.377222	2.371377
MTM1	0.509037	0.209585	0.601214	5.476758
DGCR11	0.509037	0.209618	-0.81786	4.324829

MST1L	0.509068	0.209679	-3.31089	0.847217
AVPI1	0.509068	0.209721	-1.27223	3.936371
TNFAIP6	0.509068	0.20979	-3.78245	1.012902
KMT2C	0.509068	0.209796	-0.49092	9.659218
SNORA37	0.50912	0.209858	-0.64045	4.926854
LINC01590	0.509396	0.210013	-1.93975	0.717741
ELP5	0.509524	0.210107	-1.0028	3.204437
C6orf62	0.509692	0.210218	-0.51842	6.866209
NSUN7	0.509719	0.21027	-0.78599	3.849979
GTPBP1	0.510469	0.210621	-0.55028	6.376977
WISP2	0.51051	0.210679	-2.91206	0.882039
CLPTM1L	0.510593	0.210755	0.553353	4.675264
TOMM20	0.511009	0.210968	-0.50262	7.003373
GNAQ	0.511053	0.211028	0.468072	7.495739
CASP8	0.511585	0.211326	1.591286	1.652314
NKAIN3-IT1	0.511585	0.211348	2.341417	1.009325
XRN1	0.511585	0.211372	-0.95196	3.411519
TAF1C	0.511778	0.211518	1.700387	1.37512
CFDP1	0.511778	0.211556	-0.51813	6.89627
OIP5-AS1	0.511778	0.211583	-0.50518	5.750929
KCNA3	0.511778	0.211684	1.51293	2.956001
PLN	0.511778	0.211713	1.26673	2.949094
ZNF579	0.511778	0.21178	1.03939	2.657617
SNORA2A	0.511778	0.211807	-1.31866	2.17811
TRIM8	0.511778	0.21182	0.551042	6.686778
SLC36A4	0.511778	0.211824	1.602452	1.765004
DENND4B	0.512107	0.212002	0.477618	5.628907
HOXA2	0.512484	0.2122	-1.88192	1.599984
NRARP	0.513254	0.21256	0.567348	5.822958
USP2	0.513572	0.212734	-2.7183	0.951021
LVRN	0.513757	0.21288	1.953488	1.374754
ABCD4	0.513757	0.212893	1.466012	1.622885
MAPK8	0.513883	0.212987	-2.03539	0.79812
ELMOD3	0.514137	0.213138	-1.39542	2.244226
FAM131C	0.514137	0.213196	-2.5216	0.413446
PTGS2	0.514137	0.213217	-1.25567	3.562184
DRAIC	0.514202	0.213298	-2.99517	0.936205
SRY	0.514202	0.213328	-2.01792	1.426304
IARS2	0.514293	0.213407	-0.52171	6.910073
ATP5MGL	0.514296	0.21345	-2.61322	1.118585
SNORD116	0.514425	0.213545	-0.82523	5.379694
TRIM21	0.514578	0.213651	0.712259	4.402641
FAM229A	0.514809	0.213788	1.60802	1.769297
POMGNT2	0.514814	0.213832	1.314369	2.900269
LOC103611	0.514833	0.21389	0.706015	3.894383

SNORD116	0.514833	0.213923	-0.84657	6.779588
HSD17B12	0.515111	0.21408	-0.52303	6.344746
ISM1	0.515168	0.214185	-1.32481	2.182706
CLIC2	0.515168	0.214187	1.073471	2.703308
FBXL19-AS:	0.515211	0.214247	1.19485	2.218912
HIPK1-AS1	0.515238	0.21434	1.971347	0.460844
UBE3A	0.515238	0.214342	2.383832	0.945962
SLC30A9	0.515745	0.214594	-0.49498	7.113866
PARP14	0.516033	0.214756	0.548147	7.708653
CLDN12	0.5161	0.214826	1.831189	1.49106
SNAP29	0.516101	0.214876	-0.46316	5.81972
NR1D1	0.516101	0.214925	-0.99373	4.301204
IGFBP6	0.516101	0.214989	-0.83026	4.428301
DNAJB7	0.516101	0.215003	1.761468	1.026586
PITPNA-AS	0.516101	0.215035	-1.6039	1.996807
IDH3A	0.516142	0.215118	-0.52627	6.316761
FGFBP3	0.516142	0.215136	1.244151	2.003611
AKNA	0.516464	0.215312	1.777186	1.447758
NAV2	0.516792	0.21549	1.438074	1.78536
RPL18A	0.517036	0.215658	-0.56093	7.634703
RBM26-AS:	0.517036	0.215676	-1.94396	1.347068
HEATR5B	0.517038	0.215719	-0.4795	6.816096
FN3K	0.5172	0.215828	1.087757	4.228019
SERPINE2	0.517374	0.215943	-1.91987	2.31326
C9orf66	0.517559	0.216062	-2.35793	0.837464
AP3S1	0.517897	0.216245	-0.58955	6.12694
SERPINB9P	0.517958	0.216312	-1.54031	2.334536
SNHG1	0.518103	0.216449	-0.742	4.641076
SAMHD1	0.518103	0.216457	0.561139	6.823503
GSTM4	0.518173	0.216584	2.076184	1.072051
ANXA6	0.518173	0.216601	1.845079	1.561542
ISG15	0.518173	0.216612	1.211668	1.911302
ZNF252P	0.51836	0.216732	0.538494	5.14493
CNIH4	0.518361	0.216788	-1.81618	1.974239
LOC101927	0.518361	0.216817	1.948607	1.783983
NRAS	0.518389	0.21687	-0.54038	6.392662
JPH3	0.518464	0.216963	2.342044	1.116557
RNU5F-1	0.518464	0.216985	-1.07701	4.329402
HIST1H4F	0.518601	0.217107	0.632949	4.15577
AARS2	0.518601	0.217127	-0.52961	5.348756
CAPZB	0.518783	0.217245	0.709233	3.838675
SPRYD4	0.518937	0.21744	-0.70932	4.077224
LOR	0.518937	0.217462	-1.80233	1.910785
EXOC4	0.518937	0.217465	-0.53118	5.696502
HMGCL	0.518937	0.217478	1.8928	1.148351

ZNF606	0.519075	0.217578	-1.08764	3.103239
BAMBI	0.519121	0.217639	1.476435	2.142211
SNORA38	0.519132	0.217686	-1.94333	1.91581
CCT6P3	0.519133	0.217854	-0.85991	4.078028
SLMAP	0.519133	0.217871	-1.14144	2.350823
RNF168	0.519133	0.217916	-0.55199	6.878118
TMEM17	0.519133	0.217968	-2.05872	1.791107
NT5DC2	0.519133	0.218011	-2.042	0.772638
SLC25A38	0.519133	0.218022	0.811554	4.13643
C1RL-AS1	0.519133	0.218074	0.518807	5.800922
PLAC9	0.519133	0.218085	0.952943	3.253354
STT3B	0.519133	0.218147	-0.50776	7.856565
NPIPB13	0.519133	0.218201	0.483562	7.515041
SLCO3A1	0.519133	0.218226	-0.66125	4.378225
CDR1	0.519133	0.218249	1.015006	4.332334
ABR	0.519133	0.218262	1.852	1.907068
LOC10013(	0.519133	0.218347	1.065501	3.087867
PPIP5K2	0.519133	0.218349	1.908188	0.712692
APC2	0.519133	0.218391	0.817203	3.468147
LOC10537ℇ	0.519133	0.218401	2.020935	1.476734
SHROOM1	0.519482	0.21859	1.751398	0.874639
MIR155HG	0.519669	0.21872	1.852277	1.018654
FSTL3	0.519669	0.218753	1.212163	3.434462
ZNF184	0.519831	0.218863	0.645766	4.318039
JDP2	0.519876	0.218924	1.681066	0.8639
LRRC37A11	0.520142	0.219078	-4.80649	0.824043
NUP133	0.520367	0.219215	-0.50671	5.45367
ZFP41	0.520824	0.21945	0.655338	4.046074
FAM78B	0.521278	0.219721	2.047668	0.805732
POC5	0.521278	0.219726	1.517068	0.854668
ATXN7L1	0.521353	0.219842	1.031402	3.298345
VIT	0.521353	0.219859	-2.77713	1.757786
C5orf34	0.521353	0.219884	-1.34243	2.553621
SP3	0.52158	0.220022	2.132928	1.171653
CA13	0.521603	0.220074	0.573202	5.146916
ATF1	0.5217	0.220182	-0.84205	4.076593
PTGR1	0.5217	0.22024	-2.11322	0.713561
LOC100132	0.5217	0.220241	-1.34854	4.15583
FOXN3-AS2	0.521774	0.220315	1.350807	2.114468
SNORA78	0.521776	0.220386	1.042173	3.427711
LOC648987	0.521776	0.2204	1.914866	1.043554
ABCA4	0.521988	0.220532	1.403965	1.508333
IARS	0.522213	0.220709	2.077521	1.261973
ADGRA3	0.522213	0.220712	0.629957	5.111108
LOC101927	0.522359	0.220816	1.785504	1.523324

PFKP	0.522506	0.22092	-0.97567	3.719429
FYB2	0.523122	0.221223	-2.31871	1.497812
MFSD6L	0.523229	0.221319	1.749959	1.706316
DBT	0.523229	0.221353	-0.55295	5.979572
NCK2	0.523439	0.221484	-2.82022	0.34972
PUS10	0.523664	0.221622	0.796583	4.364194
BTN3A2	0.52375	0.221701	1.659872	1.035704
NOC2L	0.523936	0.221822	-0.56986	6.214879
ARHGEF33	0.524075	0.221929	2.043395	0.906189
PLEKHA3	0.524075	0.221966	0.721055	3.924663
KDSR	0.524092	0.222015	-0.66171	6.004664
MAPKAPK2	0.524117	0.222068	-2.01735	1.163764
CKS2	0.524124	0.222114	1.118415	3.34211
TUBG1	0.524194	0.222186	-0.68852	4.847473
EIF2AK3	0.524437	0.222337	0.854677	3.954349
EPHB4	0.524437	0.222424	0.477126	6.691648
SCARNA1	0.524437	0.222452	-0.6129	5.180078
DCUN1D4	0.524437	0.222479	1.668886	1.001753
STK24	0.524437	0.222501	-1.18187	3.391394
MIR1537	0.524931	0.222754	2.087079	0.680211
AQP7	0.525052	0.222847	1.967671	0.975654
NKX6-1	0.52538	0.223029	1.722561	2.086986
RNVU1-19	0.526075	0.223367	-3.01964	1.470882
LINC02586	0.526369	0.223534	-3.86459	0.693421
ZNF718	0.526465	0.22367	1.006092	2.500215
RBM6	0.526465	0.223813	0.471042	6.841007
ZNF607	0.526465	0.223838	-1.82036	1.587234
NIFK	0.526465	0.22385	-0.90912	4.981974
SLC11A2	0.526465	0.22386	1.215723	2.015273
MAP3K4	0.526465	0.223906	-1.19881	3.015052
LINC00299	0.526465	0.223957	1.864585	0.593564
ZNF563	0.526465	0.223962	-1.70678	2.234397
SNORD134	0.526465	0.223988	-1.87393	1.684591
CTPS2	0.526465	0.224001	1.528181	1.147365
DCP1A	0.526537	0.224113	1.688954	1.602174
HLA-DQA1	0.526537	0.224121	1.455657	3.915021
ARL17A	0.526537	0.224171	0.773115	4.431404
FAM153C	0.526537	0.224202	-2.29929	0.941269
RASSF9	0.526573	0.224326	-0.94139	4.176629
ZG16	0.526573	0.224339	2.009672	0.711319
SYNPO2L	0.526573	0.224429	-1.85021	1.991141
GALNT12	0.526573	0.224449	-0.66602	6.284405
SYNPO	0.526573	0.224469	0.644606	4.768706
NEFL	0.526573	0.224497	-2.36184	2.124603
SCAF8	0.526573	0.224516	0.968893	2.844962

TXNDC9	0.526635	0.224586	-0.6242	4.831745
N4BP2L2	0.526668	0.224642	0.466782	7.044671
CNOT8	0.526996	0.224825	1.948525	1.247551
NDUFA4	0.527067	0.224898	-0.52022	5.643781
KMT2E-AS1	0.527091	0.224951	0.494017	5.513108
NCBP3	0.527262	0.225066	-0.47106	7.215628
DOCK4	0.527643	0.225374	0.53503	5.673394
RAB6C	0.527643	0.225384	1.038932	2.771792
ATRN	0.527643	0.225398	0.467793	5.655788
NUDT19	0.527643	0.225423	-0.65298	4.695885
RER1	0.527643	0.225443	-0.49385	6.702243
RBM12B-A	0.527909	0.225636	0.86224	3.082689
KYNU	0.527909	0.225653	-2.15724	2.077926
MRPS31P5	0.527909	0.225685	1.941356	1.478217
MNDA	0.528076	0.225831	1.519116	2.463819
CSF1	0.528076	0.225842	2.196883	1.122049
MRPL19	0.528169	0.225943	-0.48738	6.111816
HIST1H1D	0.528169	0.225967	-0.54869	5.306914
VPS13A	0.528275	0.226083	0.752292	3.937324
LMTK2	0.528275	0.226098	-0.50624	7.517485
SNORD33	0.528275	0.226206	0.518406	7.609875
SNORD11	0.528275	0.226293	0.675261	3.983284
HACD1	0.528275	0.226296	-1.48691	1.951714
WBP2	0.528275	0.226298	-0.49487	6.52804
MYH7B	0.528275	0.226343	0.70218	3.727604
CCDC155	0.528275	0.226355	2.401631	0.722676
PLA2G5	0.528376	0.226491	2.06042	0.749551
SATB2	0.528376	0.226495	1.591187	1.473067
CRTC3-AS1	0.528376	0.226526	2.116855	1.025947
HID1-AS1	0.528393	0.226593	2.461425	1.229776
SIRT7	0.528393	0.226619	-0.57734	5.605816
AQP1	0.528414	0.226671	1.311104	1.975503
TIMM21	0.528558	0.226776	-0.69099	4.552485
RPPH1	0.528584	0.226855	-0.50636	12.65323
RAPGEF1	0.528584	0.226873	1.129827	3.351069
TRIL	0.528856	0.227111	0.947642	3.647249
ARMT1	0.528856	0.227112	1.884043	1.216044
LATS1	0.528856	0.227118	2.001907	0.929149
PIN4	0.529055	0.227261	2.001377	1.067002
LOC100289	0.529055	0.227324	-2.5958	1.242824
RTN1	0.529055	0.227331	1.874306	0.564977
RASGRP1	0.529272	0.227468	-2.05425	0.690652
COX19	0.529297	0.227522	0.511985	4.992505
ZNF713	0.529306	0.227568	-0.98424	2.742352
TRRAP	0.529438	0.227668	1.834351	0.89774

SNORA46	0.529537	0.227815	-0.87973	4.022129
SRC	0.529537	0.22782	1.458184	1.296608
SMG5	0.529537	0.227855	-0.48176	7.139542
DDX21	0.529537	0.227882	-0.86023	3.386268
WDR59	0.529579	0.227943	-0.48353	5.861806
DDX10	0.529631	0.228028	-0.52924	5.809193
FHDC1	0.529631	0.228051	-0.54283	6.011916
RPRD1B	0.529959	0.22824	-0.47267	5.44536
PIGM	0.529959	0.228278	0.826462	4.116953
SNORA16B	0.530085	0.228375	-1.97443	1.542836
SLC39A6	0.530291	0.228507	-0.54564	5.07475
SNORD67	0.530604	0.228685	0.632952	4.898954
RNMT	0.530619	0.228735	-1.02277	3.572591
FAM84B	0.530714	0.228847	-0.51372	6.890578
FAM111A-I	0.530714	0.228861	1.303363	2.005342
SNORD12B	0.530939	0.229036	-0.65402	6.361135
SNORA73B	0.530939	0.229068	-0.54533	9.920571
KLF13	0.530939	0.229088	-0.83517	3.784266
SERINC2	0.531105	0.229202	-1.17853	3.172985
LOC10028!	0.531202	0.229287	-1.39955	2.443504
ZNF22	0.531225	0.229394	0.998079	3.290581
SETMAR	0.531225	0.229424	-1.93182	2.048789
FRG1	0.531225	0.229426	-2.43743	0.77465
MIR570	0.531244	0.229477	0.910577	2.957524
SEL1L3	0.531251	0.229523	2.232526	1.098207
RNVU1-7	0.531283	0.2296	-0.53969	6.892654
DEGS2	0.531283	0.229651	-0.76309	4.754062
APOPT1	0.531283	0.229666	0.955141	2.693789
MED12	0.531604	0.229911	0.439802	6.751546
WWC2	0.531604	0.22992	-0.52458	5.882474
PTGIS	0.531604	0.229934	1.168536	3.128142
SCARNA21	0.531877	0.230095	-0.51887	7.659808
ANP32B	0.531988	0.230268	0.612735	9.05376
FAM98A	0.531988	0.230304	-1.96292	1.79401
ARID5B	0.531988	0.230337	0.665049	4.5728
HMGN5	0.531988	0.230343	0.565057	6.08173
ANP32C	0.531988	0.230361	1.736609	1.381424
CALHM5	0.531988	0.230402	2.070782	1.258823
TYSND1	0.532049	0.230471	-1.66101	1.358394
SEPT7P2	0.532881	0.230875	0.618943	4.280167
ZNF658B	0.533312	0.231129	-1.40761	2.572096
PLCB2	0.533312	0.231148	2.106112	0.616598
ADAMTS5	0.533637	0.23137	1.097156	3.900018
PDSS1	0.533637	0.231375	0.790699	4.026328
IL18RAP	0.533949	0.231609	1.499004	2.174119

PRC1-AS1	0.533949	0.231724	-2.63195	1.034448
PBOV1	0.533949	0.231749	1.691233	2.173706
MPLKIP	0.533949	0.231773	-0.73229	4.056232
DYNC1LI2	0.533949	0.231776	0.687023	3.756187
GEM	0.533949	0.231804	2.197465	1.460248
SRPX2	0.533949	0.231813	-0.51656	5.918694
LOC100335	0.53402	0.231914	0.993527	3.266155
VSIG2	0.53402	0.231931	1.059596	5.437663
LOC155060	0.53416	0.232034	0.686293	4.083994
FTX	0.534209	0.232099	0.71786	4.735205
NDUFA8	0.534357	0.232207	-0.67422	4.089064
LINC01798	0.534408	0.232272	1.985913	0.50426
ACOX2	0.534998	0.232609	1.105674	2.42166
ADGRL2	0.534998	0.232618	-1.6309	2.538039
SLC26A2	0.534998	0.232659	-0.49142	5.782846
LINC00612	0.53502	0.232712	1.791254	0.552794
ATP5F1A	0.535638	0.233027	1.166375	1.802473
BSDC1	0.535638	0.233072	0.750281	3.740933
CNRIP1	0.535638	0.233111	2.255558	0.541948
ESRRA	0.5357	0.233181	0.896128	3.104646
PCSK6-AS1	0.535956	0.233364	-2.30424	1.03568
ESAM	0.535956	0.233379	-0.74136	3.919848
INSYN2	0.536049	0.233476	0.709757	4.354093
MRPS23	0.536049	0.233507	-0.69651	4.89417
ZNF439	0.536795	0.233875	-1.01607	3.724264
LINC00578	0.537155	0.234076	1.612485	1.520229
DDX41	0.537452	0.234248	-0.49906	6.085218
RGPD3	0.538071	0.234562	-1.31368	2.003113
BCAM	0.538225	0.234672	-1.00553	3.016965
LOC101927	0.538538	0.234852	-1.28962	2.405324
RNF122	0.53857	0.23491	1.170606	2.51457
SAMD15	0.539264	0.235284	1.464163	1.619878
MCEMP1	0.539264	0.235321	-3.06651	1.036461
MSC	0.539264	0.235344	1.584132	2.068481
SLC45A1	0.539669	0.235582	1.748408	1.14671
PLEKHG5	0.539669	0.235608	-1.5183	1.780341
MRTO4	0.539753	0.235688	-0.57634	4.932571
PCDHB19P	0.539789	0.235782	1.515047	1.93903
TMEM243	0.539789	0.235791	0.798162	3.150341
RBM43	0.540078	0.235961	0.874823	3.769684
SYNE1	0.540112	0.236057	1.915393	1.296401
MYSM1	0.540112	0.236073	-0.48493	7.450333
MFSD14A	0.540112	0.236107	-0.50699	5.775959
FNIP2	0.540154	0.23617	0.495055	5.765108
MCOLN2	0.540224	0.236244	1.310383	2.484929

LDLRAD4	0.54063	0.236499	2.108143	1.089505
FAM3D	0.54063	0.236555	0.550483	6.750181
ELOVL2	0.54063	0.236624	2.134576	0.770194
IL5	0.54063	0.236682	2.503861	0.682361
USP18	0.54063	0.236715	1.143656	2.70215
FBXO34	0.54063	0.236728	-0.72005	3.770389
ACOT2	0.54063	0.236728	2.087183	0.922206
RABGGTB	0.540805	0.236889	1.262109	2.739502
FBXO5	0.540805	0.236892	-1.93615	0.464894
HRC	0.540884	0.237046	1.851857	0.765389
DNMBP	0.540884	0.237056	0.51058	6.572808
GUSBP4	0.540884	0.237058	0.571936	4.727331
MEIS1	0.541052	0.237176	-0.74523	6.16477
ACSM1	0.5411	0.237241	1.664083	1.978316
SNORA64	0.54123	0.237401	-0.58491	7.484684
SETDB2	0.54123	0.237421	1.129499	2.335247
SUSD6	0.54123	0.237429	-0.49427	7.187022
C8orf59	0.541279	0.237495	2.72306	0.746933
SNHG18	0.541318	0.237564	-0.90775	3.894529
CHP2	0.541318	0.2376	2.21103	1.589328
NCAM2	0.541496	0.237743	1.730363	1.158055
FLJ37035	0.541496	0.237802	2.525065	0.573546
SEMG1	0.541496	0.237809	2.985666	1.264925
OSGEP	0.541686	0.237936	0.565593	4.391797
CD5	0.541799	0.238041	0.802942	3.773946
LRRC58	0.541799	0.238108	-0.6707	6.455401
GOLGA6L9	0.541799	0.238118	-2.65877	0.709264
THBS1	0.541883	0.238199	0.797611	7.645587
SBSPON	0.541935	0.238308	-1.29959	3.194933
MTRNR2L1	0.541935	0.238325	-2.25245	0.904648
MMP16	0.541935	0.238357	1.382463	1.897123
EFL1P1	0.541935	0.238411	1.496296	0.713354
KIAA0753	0.541935	0.238453	-0.46489	5.759619
PDPR	0.541935	0.238485	-0.74028	5.588203
WIPF2	0.542081	0.238593	-0.48304	6.935181
PCARE	0.542091	0.238641	-2.49928	0.813518
ARRDC5	0.542102	0.23869	1.471237	1.860696
PTCH1	0.542199	0.238776	1.30438	2.388897
LOC10028[illegible]	0.542298	0.238864	-2.279	0.873837
PPP1R35	0.542303	0.23891	0.992379	3.015904
KIZ-AS1	0.542532	0.239055	1.495847	1.776707
CSTF2T	0.543145	0.239369	0.551907	5.512226
LOC10192[illegible]	0.543287	0.239476	-2.27446	1.466297
ST5	0.543584	0.239651	-0.98707	3.387233
CLDN17	0.543609	0.239706	-3.23127	0.894301

GRID1	0.543788	0.239829	1.301542	1.897107
NHSL2	0.544265	0.240083	1.081518	3.024414
FXR2	0.544603	0.240276	-0.48705	5.711119
LPGAT1	0.544794	0.240425	0.452198	6.4948
HNRNPL	0.544794	0.240449	0.660703	4.343031
CAMLG	0.545333	0.240752	-0.55374	4.578693
ARRDC4	0.545333	0.240775	0.655498	4.972983
RAB40B	0.545341	0.240862	0.694346	4.02897
LINC01979	0.545341	0.24088	1.454564	1.915053
SPPL2A	0.545341	0.240911	0.489272	5.158378
CXCL14	0.545348	0.240958	-0.58997	6.737759
HSPA9	0.545419	0.241034	-0.48765	8.223302
PLLP	0.545508	0.241118	0.807732	4.200824
SARS	0.545553	0.241182	-0.46822	7.080266
LRP4	0.545972	0.24145	-0.73019	5.718856
POLR2G	0.545972	0.241497	-0.6686	4.114354
DPH2	0.545972	0.241541	1.461924	1.891763
LIFR	0.545972	0.241567	-2.75377	1.177995
RBPMS-AS:	0.545972	0.241588	0.936956	2.681091
COL5A3	0.546017	0.24168	0.548491	5.766673
ACACA	0.546017	0.241697	-2.12184	1.007652
SCAND1	0.546017	0.241741	1.802482	0.858147
FTSJ1	0.546027	0.241789	1.853257	0.550027
ANKHD1	0.546124	0.241881	0.925638	2.768722
LOC10106(	0.546124	0.241921	1.80239	1.257322
LINC00886	0.54657	0.242182	1.421666	2.88647
TM9SF4	0.54657	0.242207	-0.46458	6.916251
OGFR	0.546819	0.242361	0.602922	5.509594
FAM133CP	0.546943	0.242461	1.896271	1.477912
GPR174	0.546998	0.242532	1.492549	1.233792
SENP5	0.546998	0.242573	-1.71129	2.001402
PRR7-AS1	0.547087	0.242657	1.798801	0.722013
AGRN	0.547368	0.242827	-2.57188	0.647813
KLHL42	0.548112	0.243201	0.501559	5.333722
WDR82	0.548244	0.243304	-0.47291	7.018649
LINC00342	0.548328	0.243386	0.655942	5.623006
CTAGE7P	0.54843	0.243475	1.436628	1.625017
LINC01612	0.548458	0.24354	2.233173	0.716201
AHCYL1	0.548458	0.243588	1.645817	1.916367
ZSCAN2	0.548458	0.243621	1.002619	3.003435
ZBTB7B	0.548472	0.243673	-1.41746	1.531689
LTV1	0.548472	0.24378	-0.64978	5.825576
LINC00630	0.548472	0.243799	-1.06479	3.100003
DIO3OS	0.548472	0.243805	2.274811	1.272005
TAF2	0.548575	0.243932	-0.49298	6.51141

KDM6B	0.548575	0.243948	-0.52095	6.938835
TRIM58	0.548575	0.243984	-2.85098	0.488109
MAT2A	0.548877	0.244224	-0.68951	7.879907
HLA-E	0.548877	0.244235	0.752031	3.62905
GPR22	0.548877	0.244252	-0.88954	3.363453
PDLIM1	0.548949	0.244328	0.455356	7.437573
LINC00548	0.548982	0.244435	2.18121	0.671684
ACOX1	0.548982	0.244459	-2.12073	1.353762
TOE1	0.548982	0.244476	0.785936	3.155473
RNF4	0.549457	0.244736	1.34113	2.022792
C1orf174	0.549457	0.244777	0.581834	4.163015
LINC00939	0.549526	0.244852	1.855521	1.279953
NLN	0.549708	0.244977	-0.55306	5.116642
ANKRD34A	0.54972	0.245027	1.426932	1.598744
SUFU	0.550126	0.245285	-0.718	4.081447
ADA	0.550126	0.245298	-1.05197	3.582502
CLYBL	0.550149	0.245393	1.786359	0.596595
LINC00235	0.550149	0.245397	1.679448	0.943523
LOC10272⁴	0.55039	0.245549	1.298472	2.323752
TMEM147-	0.550663	0.245725	-0.91593	2.882569
MAT2B	0.550663	0.245778	1.203101	3.303291
FOXD2-AS1	0.550663	0.245843	1.559082	1.615621
MKNK1	0.550663	0.245886	-1.98628	1.424455
RYR2	0.550663	0.245894	0.990046	4.266157
COIL	0.550724	0.245966	-0.53448	5.150065
C1GALT1C1	0.550734	0.246015	-2.80976	1.396619
TMEM140	0.550743	0.246063	0.957045	3.616276
CWC15	0.550855	0.246158	-0.54315	5.397908
ACTR1A	0.550871	0.246211	-0.47682	6.316513
KYAT1	0.550871	0.246283	-3.07581	0.668879
SH3GL2	0.550871	0.246311	-4.07632	0.731734
LCLAT1	0.550871	0.246344	2.391143	0.719571
TBP	0.550912	0.246407	2.022396	0.90249
NCKAP5-AS	0.551016	0.246542	-2.53904	1.082564
ZNF695	0.551016	0.246542	1.614619	1.70796
PTS	0.551815	0.246944	-0.69517	4.063313
FIP1L1	0.551898	0.247069	1.913161	1.796699
PSMA2	0.551898	0.247071	-0.49662	5.458446
MAPK14	0.551927	0.247172	-1.53336	1.470267
KLHDC1	0.551927	0.247194	1.075197	2.511973
MTPAP	0.551927	0.247218	-0.54422	5.031993
DOCK6	0.552117	0.247348	0.443155	7.046341
NSMCE1	0.552303	0.247489	-0.58147	5.152528
WASL	0.552303	0.247521	-0.49749	7.570978
SNAPC5	0.552343	0.247584	0.754685	3.237842

ETFDH	0.552347	0.247647	-1.76405	1.106357
XXYLT1	0.552347	0.247675	-1.81323	0.995173
CCDC90B	0.552494	0.247786	2.236044	0.893344
COQ8A	0.552518	0.247841	0.721626	5.352227
FAF1	0.553021	0.248111	-0.4401	6.621299
TNFRSF13C	0.553223	0.248247	2.093235	1.206194
DCTN6	0.553516	0.248441	-0.83781	4.40662
CHMP4A	0.553516	0.248468	-0.46601	5.973321
ABHD13	0.553851	0.248776	-0.51603	5.425595
GIGYF1	0.553851	0.248784	0.443187	7.298814
FUZ	0.553851	0.248798	-2.27599	0.626229
AGK	0.553851	0.248811	-0.61557	4.43296
DPYD	0.553851	0.24888	-0.70697	5.851154
FAXDC2	0.553851	0.248888	0.539477	4.577204
SIX1	0.555074	0.249482	1.970199	1.318568
NT5C2	0.555928	0.249936	-2.19982	0.412535
PIK3IP1-AS	0.555928	0.249956	1.804005	0.835114
GLRX5	0.556517	0.250321	0.630635	4.460278
DDX1	0.556517	0.25033	-0.48612	7.083682
SYNJ2-IT1	0.556517	0.250362	-1.48635	1.725473
RN7SK	0.556517	0.250401	-0.46557	14.27483
LOC101927	0.556611	0.250488	-2.94826	0.565657
LINC00987	0.557145	0.250824	0.520435	4.768365
HTT-AS	0.557145	0.250865	1.975524	0.58147
LURAP1	0.557145	0.25094	-1.79734	1.437364
RGCC	0.557145	0.250949	0.927381	3.616906
STYX	0.557145	0.250954	-1.91023	0.716628
ZNF212	0.557558	0.251185	-0.57649	5.107718
TRIP6	0.55825	0.251587	-0.47347	6.086734
UBASH3B	0.55825	0.251588	-0.88362	3.796775
TPST1	0.558273	0.251677	-0.59293	4.929779
ZGLP1	0.558273	0.251689	1.827083	1.368218
ANAPC1P1	0.558696	0.251925	0.815765	2.749488
LRBA	0.558768	0.252055	2.101697	1.120611
RPRD1A	0.558768	0.252087	1.387484	1.951466
TSPYL2	0.558768	0.25213	0.729338	6.126939
GPR173	0.558768	0.252138	1.549457	2.209785
NAA30	0.558776	0.252225	-0.48348	5.501994
MYH15	0.558776	0.252232	1.830492	1.192542
PDE1A	0.558887	0.252389	2.168702	1.39188
CSNK1G2-A	0.558887	0.252407	1.085002	1.700752
COX11	0.558887	0.252486	-0.82472	3.20501
IQGAP3	0.558887	0.252492	0.499118	4.971683
ARHGEF5	0.558887	0.252509	-0.51025	6.40081
NBPF14	0.559341	0.252779	-0.61224	5.613169

SNORD28	0.559341	0.252823	-0.88689	4.103438
RPS16P5	0.559341	0.25285	-1.0587	4.399701
SGMS1-AS1	0.559392	0.252942	-1.74401	1.321573
ZNF225	0.559392	0.252982	0.718607	3.473426
ATP8A1	0.559392	0.253009	1.803173	1.306681
MLYCD	0.559487	0.253101	0.789676	3.939882
ZC3H6	0.559487	0.253142	-0.51784	6.186605
DIMT1	0.559576	0.253272	-0.66375	4.527836
KIAA1841	0.559576	0.253273	1.01554	3.006169
1-Sep	0.559615	0.253336	0.860771	3.431265
CCDC47	0.559863	0.253494	-0.44471	7.751497
LAMC3	0.560039	0.253638	0.842568	3.929654
PM20D2	0.560039	0.253732	-0.55913	5.44977
EIF3M	0.560039	0.253744	-0.73	4.311521
SPDYE3	0.560039	0.253755	-0.92359	2.87873
C11orf71	0.560077	0.253817	1.214213	2.506671
C3orf35	0.560155	0.253898	1.488471	2.06944
ZNF569	0.560189	0.253963	-1.74479	1.269887
SETD9	0.560189	0.254021	-2.69004	0.769576
S100B	0.560189	0.25405	1.153372	2.301579
MIR3936H(	0.560358	0.254177	1.014751	2.203175
KDM2B	0.560358	0.254217	1.487534	1.347359
PXN	0.560685	0.254411	-1.26259	1.773095
SNORD113	0.560975	0.254588	1.695212	1.394381
SPEN	0.560995	0.254672	-0.45084	8.736333
BCAN	0.560995	0.254688	1.479332	1.421758
HOXB-AS1	0.561582	0.255	1.812719	1.075981
KIAA1551	0.562264	0.255355	0.490245	7.43514
PLCB4	0.562335	0.255454	2.528746	0.724503
HIST1H2BE	0.562335	0.255478	-0.85938	3.643305
ZNF91	0.562358	0.255535	-2.08406	0.9026
MLLT1	0.562382	0.255591	-0.4611	6.093649
SNORD35B	0.562663	0.255764	0.672924	3.990767
HYMAI	0.5628	0.255943	-1.36633	2.904503
TRIM41	0.5628	0.255979	-1.30068	2.363305
FBXO6	0.5628	0.256131	1.045389	2.803868
RMRP	0.5628	0.256149	-0.44376	11.99632
ERLIN1	0.5628	0.256155	-2.35877	1.298271
AGBL2	0.5628	0.25616	1.410204	1.598047
GPR89B	0.5628	0.256163	0.820798	3.138224
C12orf49	0.5628	0.256202	0.439161	5.655991
FZD9	0.5628	0.256237	2.147195	0.799025
FMO4	0.562833	0.256297	1.182185	3.451408
SCAMP1-A:	0.562835	0.256344	1.108286	2.34381
LOC10574:	0.562916	0.256426	-1.25061	2.63659

ZNF775	0.562962	0.256493	1.186572	1.869132
ZNF442	0.563116	0.256609	-1.99854	1.175416
PSMD6	0.563366	0.256793	-1.87038	1.159981
RAB28	0.563366	0.256814	-2.00099	1.882738
MRPS35	0.563542	0.256959	2.320618	0.692874
ADGRD1	0.563542	0.256985	1.063099	3.910365
RANGAP1	0.563767	0.257146	-0.8818	3.140153
CHD3	0.563767	0.257179	1.071112	3.211591
RNA28SN5	0.563858	0.257267	0.532141	16.46569
NAF1	0.564094	0.25742	-1.29774	2.347986
CEP44	0.564139	0.257486	-0.68887	4.452342
LRFN5	0.564201	0.25761	1.728256	0.797536
VEZF1	0.564201	0.257613	0.456621	6.143856
GRID2IP	0.564201	0.257652	-1.0655	2.940576
CD69	0.564753	0.257949	1.086877	2.934354
LOC100507	0.564895	0.25806	1.731653	0.6439
LINC01372	0.565099	0.258213	1.607689	1.01246
C6orf99	0.565099	0.258271	1.959776	1.144176
SLC25A29	0.565099	0.25829	1.343519	1.534452
TM9SF2	0.565481	0.258511	0.425145	7.810019
MRPS22	0.565686	0.25865	-0.48572	5.579011
LOC100499	0.56584	0.258767	1.486003	1.162507
COPS9	0.56601	0.25889	-1.80478	1.964741
LTC4S	0.566344	0.259116	1.600528	1.384308
NAP1L1	0.566344	0.259135	-0.92793	2.852367
CHMP4B	0.566569	0.259284	-0.46921	7.619978
MIR1281	0.567158	0.259599	1.147038	2.965403
BRINP1	0.567233	0.259679	-1.76046	1.658181
POU6F1	0.567801	0.259992	-1.90083	1.379016
LINC02384	0.567801	0.260064	2.529868	0.436085
EIF1B-AS1	0.567801	0.260077	-2.04807	1.195695
ITFG2	0.568217	0.260345	0.587768	4.463498
MIR421	0.568217	0.26036	-1.92485	0.952381
LLGL1	0.568271	0.260448	-0.63526	4.848974
AMD1	0.568271	0.2605	1.656322	1.471328
C1QTNF8	0.568271	0.260553	2.372947	1.536654
ZNF561	0.568271	0.260574	-0.47506	5.90097
PIGF	0.568271	0.260635	1.66763	1.580573
FZD1	0.568271	0.260661	-0.55055	5.253826
P4HB	0.568418	0.260774	-0.46274	9.910831
SEC11A	0.568562	0.260932	1.721971	1.37582
FMOD	0.568562	0.260936	0.991736	4.68616
GPR52	0.568562	0.260978	0.929887	3.270771
KIF19	0.568597	0.261041	2.168203	0.848036
LOC286297	0.568984	0.261308	-1.93057	2.776259

BGN	0.568984	0.261347	0.671571	5.548552
PLCL1	0.568984	0.261356	1.333283	3.276907
SOCS6	0.569008	0.261463	-0.50288	6.281854
PRPF39	0.569008	0.261575	-0.4473	5.634654
ANKRD16	0.569008	0.261582	1.845413	1.157802
SAP30	0.569008	0.261588	0.613637	4.170521
NCAPH2	0.569008	0.261598	-1.59739	1.173961
TRIM68	0.569264	0.261763	-2.05371	1.050912
HNRNPDL	0.569264	0.261846	-0.9068	3.083379
LBR	0.569264	0.26187	1.595754	0.84549
JTB	0.569264	0.2619	0.476311	6.052607
TP53I13	0.569268	0.261948	0.782093	3.560841
LINC00443	0.56928	0.262	1.554059	1.739211
MIR32	0.569629	0.262241	-3.04063	0.829904
C1QTNF6	0.569629	0.262277	1.745327	1.037326
KDM7A	0.569629	0.262299	-0.48423	8.025877
LINC00641	0.569866	0.262459	-0.48849	5.136401
USP30-AS1	0.569866	0.262532	1.848165	1.235022
AEBP2	0.569866	0.262546	-1.28509	2.105387
LOC101929	0.570323	0.262803	-2.01886	1.796173
H3F3C	0.570372	0.262872	-2.03827	1.190393
PIK3C2B	0.570412	0.262962	0.437897	7.169457
PIGS	0.570412	0.262983	-0.52211	5.187962
SCML1	0.570458	0.263073	2.397299	0.430869
DOLK	0.570458	0.26314	-0.87127	3.431483
PAK4	0.570458	0.263143	1.854539	0.80768
ACOXL	0.570602	0.263295	1.915025	1.355693
LNPEP	0.570602	0.263365	-2.17112	0.777936
STXBP4	0.570602	0.26339	0.651048	4.568166
DEXI	0.570602	0.263408	0.708592	4.105372
ZNF441	0.570602	0.26344	0.940881	3.780293
FAM86EP	0.570676	0.26352	-2.574	0.567723
MIR320C1	0.5707	0.263578	-2.49242	1.070989
NFKBIZ	0.571265	0.263885	-1.28508	2.046218
LCOR	0.571513	0.264046	-0.46721	6.330915
DNAJC9	0.571701	0.264179	-0.4924	5.149067
SRRM4	0.571839	0.264289	-1.74537	2.05716
RTL10	0.572063	0.264479	-0.50479	5.316727
IQCH-AS1	0.572063	0.264516	2.155772	0.799304
MTRNR2L9	0.572063	0.264532	-1.48918	1.978404
ZNF561-AS	0.572094	0.264592	0.934773	2.606106
APOOL	0.572142	0.264703	-0.52583	5.745068
XPO7	0.572142	0.264745	-0.48609	6.507641
SNORD116	0.572142	0.264754	-0.93029	3.147945
SGSH	0.572169	0.264812	-0.64166	4.431444

SLC22A15	0.572467	0.264997	-0.59398	5.120499
PCNX4	0.573342	0.265469	0.497769	5.307836
SNORD100	0.573342	0.265537	0.838282	2.713495
GCNT7	0.573342	0.265541	1.224053	2.753049
TMEM132(	0.573669	0.265739	1.565177	1.991744
UHRF1	0.573691	0.265795	1.838402	0.882617
HEYL	0.574021	0.26605	1.297234	2.631192
SLC22A14	0.574021	0.266126	-2.88372	0.64694
UFL1	0.574021	0.266154	-0.49442	6.992986
APMAP	0.574021	0.266177	0.413688	5.516669
FAM95C	0.574021	0.266216	0.680906	3.942127
COPZ2	0.574021	0.266228	-0.9518	2.589969
PPARD	0.574088	0.266305	-1.86622	2.230408
HERPUD2	0.574373	0.266492	-0.51073	5.596589
OST4	0.574373	0.26653	-0.48482	5.341837
NUP50	0.574443	0.266609	-1.24172	2.053641
MRPL27	0.574464	0.266666	0.587747	4.20769
KSR2	0.574792	0.266868	1.141703	2.679412
CDT1	0.574792	0.266949	-0.8194	3.362915
MIS18BP1	0.574792	0.266997	0.466701	6.068426
NR2C1	0.574792	0.267004	0.947507	3.07544
PKHD1L1	0.575201	0.267241	1.115722	3.658869
CCDC173	0.575511	0.267431	-2.17077	1.397766
RPS6KB1	0.575528	0.267486	-2.36476	0.961181
MSANTD2	0.575655	0.267591	0.664887	3.977257
NMI	0.575843	0.267747	0.551106	4.531966
UPK2	0.575843	0.267772	-2.93341	0.563002
CAPN15	0.576031	0.267906	0.442932	6.274652
PNP	0.576135	0.268001	-0.53504	6.342001
TMCO5A	0.576364	0.268154	2.560879	0.631781
PHAX	0.576402	0.268219	-0.44879	5.674804
FRS3	0.576536	0.268363	-1.41909	2.131332
USE1	0.576536	0.268393	0.631786	3.419191
TMCO1-AS	0.576536	0.268421	1.851177	1.212916
SNORA47	0.576633	0.268517	0.997429	6.546089
SNHG19	0.576633	0.26856	-1.09571	2.58558
GUSBP11	0.577008	0.268781	0.529724	4.812296
GS1-124K5	0.577015	0.268831	-1.25891	3.123207
SLFN12L	0.57739	0.269053	1.211626	2.977146
PRR14L	0.577559	0.269241	-0.44099	8.004234
C16orf54	0.577559	0.26925	1.571592	1.932267
TSTD2	0.577559	0.269272	0.610529	4.008363
ZSWIM9	0.577563	0.269321	0.567148	4.812881
SKI	0.57785	0.269501	-0.43289	7.068588
COPG1	0.577906	0.269574	0.436693	7.598829

DCC	0.577966	0.269649	1.524042	2.197879
SLC7A11	0.578026	0.269724	-0.6599	4.95836
RAB31	0.57847	0.269978	-0.56764	6.239307
CHMP4C	0.578728	0.270145	-0.57377	5.59343
SLC25A24	0.579059	0.270346	1.155414	2.179768
FAM122C	0.579148	0.270444	-1.88585	0.787541
LPAR2	0.579148	0.270482	0.668149	3.357093
MGAM	0.579205	0.270587	1.754318	0.807455
RASEF	0.579205	0.270602	0.439049	6.393904
BACE1-AS	0.579621	0.270844	-2.05638	0.814687
MYH9	0.579762	0.270956	0.413321	11.18399
PGGT1B	0.579929	0.271098	-0.48855	5.085285
LINC00909	0.579929	0.271167	0.799947	3.350027
HIST2H2AC	0.579929	0.271175	-0.63623	4.363767
FRYL	0.579968	0.271279	0.408419	8.034978
LAMTOR1	0.579968	0.271287	-0.49886	5.870931
GGT1	0.579968	0.271334	1.865291	0.928052
ERGIC1	0.580065	0.271427	0.450298	7.420451
TMEM214	0.580159	0.271518	1.679891	0.958951
KHDC4	0.580243	0.271604	0.428721	6.34685
BRD4	0.580296	0.271676	-0.44785	6.330781
ZNF90	0.580307	0.271747	-1.67402	1.633816
MORN2	0.580307	0.271805	-1.43903	2.341187
OPA3	0.580307	0.271822	0.422636	5.473777
ATXN1L	0.580331	0.27188	-0.44847	6.445833
EPHA8	0.580775	0.272135	-2.87839	0.894713
EFS	0.580824	0.272206	-1.4853	1.655239
SDE2	0.58106	0.272363	-0.46055	5.538202
DPEP2	0.58107	0.272415	1.574646	0.966209
MIS12	0.581294	0.272567	1.382471	1.940027
USP32P2	0.58146	0.272692	1.121651	3.482557
SVBP	0.581526	0.27277	1.257961	1.794869
GPR82	0.581562	0.272872	0.628986	3.666515
ZFP64	0.581562	0.272968	0.982957	2.787384
BAK1	0.581562	0.27298	0.585914	4.699341
RAD54L2	0.581562	0.273022	-0.4959	5.680492
IMP4	0.581562	0.273081	0.580276	4.530151
LYSMD1	0.581562	0.273119	-1.14652	2.844316
EXD3	0.581562	0.273166	0.629474	3.614349
HARBI1	0.581562	0.273242	0.984042	2.186901
PDCD2L	0.581562	0.273258	1.029066	2.680229
SLC12A9	0.581562	0.273258	0.802039	3.154862
SPA17	0.581774	0.273409	1.324969	1.847454
SETD1A	0.581774	0.273452	0.493927	6.120157
LMNB1	0.581892	0.273567	-1.21174	2.378631

ING2	0.581892	0.27366	-1.93121	1.195497
MUT	0.581892	0.273662	-0.44187	6.066512
MED22	0.581892	0.273721	0.537231	4.435095
SNORD66	0.581892	0.273743	1.253915	1.182682
ARHGEF9	0.582021	0.273913	1.732529	0.675754
N4BP2	0.582021	0.273917	-0.46878	6.344978
ACVR2B	0.582021	0.273955	0.546253	4.832774
PCDH12	0.582021	0.273992	0.986467	2.853072
ZZEF1	0.582021	0.274039	0.41119	8.044935
MACC1-AS	0.58211	0.274128	0.733808	3.110164
SRGAP2C	0.582204	0.274229	0.787653	3.617591
KIAA2013	0.582204	0.274267	0.434409	5.325917
RASGRP3	0.582321	0.274369	-2.52327	0.791562
SSTR2	0.582379	0.274444	1.765992	1.122563
HLTF	0.582389	0.274495	1.618922	1.451017
KLHL18	0.582503	0.274596	-0.57814	5.099528
LIMS2	0.582524	0.274654	1.777138	0.980577
CRTC2	0.582721	0.274794	0.44136	5.51454
STX6	0.58304	0.274991	-1.65705	2.095051
MIR100HG	0.583439	0.275255	0.864422	4.529201
FITM1	0.583439	0.275274	-2.64062	0.869437
LTBP4	0.58354	0.275369	1.338567	2.45392
HIST1H3I	0.583564	0.275465	-0.64399	4.652329
PRELID2	0.583564	0.275475	2.043261	0.869993
MFSD4A	0.58369	0.275582	-1.58405	5.334285
ROCK1	0.584126	0.275835	0.406916	8.306585
RIOK2	0.58424	0.275936	-1.31871	2.233241
ILF3-DT	0.584392	0.276055	0.83822	3.439373
LDB2	0.584425	0.276118	1.743893	1.06316
SLC8A2	0.58459	0.276243	-2.51045	0.707664
HMGXB3	0.584816	0.276397	0.420704	5.737937
ZNF684	0.584877	0.276519	1.083372	2.275592
PTDSS2	0.584877	0.276541	-1.38208	1.990064
GMCL1	0.584877	0.276568	-0.53455	4.547684
PLA2G4B	0.585042	0.276694	-1.72147	1.762824
OSTM1	0.585259	0.276844	0.475961	4.862331
RAB39B	0.585757	0.277127	2.308786	0.736368
PON3	0.586357	0.27747	1.86801	1.419916
TRPC6	0.586357	0.277569	0.788595	3.292218
DDX3Y	0.586357	0.277641	-0.91869	4.860326
ABRAXAS2	0.586357	0.277721	-0.57201	5.329844
SEM1	0.586357	0.277783	-0.4594	6.397596
ZC3H14	0.586357	0.277804	-1.29241	1.797224
UBE2Q2P1	0.586357	0.277866	-0.53866	5.138709
LOC389247	0.586357	0.277882	1.679498	1.394809

APOL1	0.586357	0.277884	-3.09135	1.666218
CRYGS	0.586357	0.277885	-2.03863	0.766305
TOR1AIP2	0.586377	0.277942	-0.4288	6.390632
SERF2	0.586426	0.278013	1.120194	2.006741
ZNF624	0.586468	0.278082	-0.67257	4.362433
LOC73018З	0.586468	0.27818	1.119001	2.04066
HMGCR	0.586468	0.278205	1.068101	2.603435
GOLGA7B	0.586468	0.278223	-0.98102	3.311938
FRG1CP	0.586624	0.278344	0.600836	4.102465
PPP4R3A	0.586707	0.278469	-0.54266	4.522979
MALL	0.586707	0.278479	-0.68818	7.486656
EFCAB13	0.586797	0.27863	1.100593	3.276471
CHMP2A	0.586797	0.278662	1.523724	0.967645
SSSCA1-AS	0.586797	0.278664	1.382919	1.747807
PCDHGB8P	0.587146	0.278895	-1.45382	3.029424
SV2C	0.587146	0.278933	-2.0199	0.983425
MTERF1	0.587146	0.278973	-2.17696	0.565094
PLEK	0.587209	0.279062	1.058253	3.004553
CASD1	0.587209	0.279098	0.554764	4.696
SRPK2	0.587808	0.27943	-1.32334	2.000997
CENPB	0.587914	0.279552	0.435983	6.762946
SNORD3A	0.587914	0.279576	0.498171	10.37527
COX7A2L	0.588041	0.279706	0.489492	5.394146
TUFT1	0.588041	0.279765	-2.59344	1.129179
LINC01089	0.588041	0.279804	0.582822	4.538741
TAS2R3	0.588041	0.279827	-1.8685	0.608569
TRIB1	0.58826	0.280029	-1.21205	2.614354
ARHGAP35	0.58826	0.280058	-0.42574	7.874177
FXYD7	0.58826	0.280178	-2.82096	1.143794
STYK1	0.58826	0.280202	-0.61813	5.038657
MACROD1	0.58826	0.280215	0.653621	3.906092
PLEKHH2	0.58826	0.280217	0.630784	5.970448
MIR31HG	0.588432	0.280346	-0.70526	3.919785
RPS11	0.588563	0.28048	-0.42999	8.74779
SNORA49	0.588563	0.280504	-0.45757	7.386038
MYO6	0.588582	0.280561	-1.38012	2.650277
GRTP1	0.588691	0.280712	-1.13584	1.966471
SCARA3	0.588691	0.280772	-1.02797	3.59229
WBP11	0.588691	0.280845	-0.44155	6.629469
LINC01061	0.588691	0.280847	-0.69726	3.740859
MRPL44	0.588691	0.280851	-0.54264	4.6886
MALAT1	0.589051	0.281071	-0.47047	16.65177
LOC10193(	0.589163	0.281198	-2.89114	0.658515
GP1BA	0.589163	0.28122	-0.9544	3.4184
PKP3	0.589882	0.281681	-1.38723	1.792626

C1QBP	0.589882	0.281705	-0.454	5.960198
CCNE1	0.589882	0.281706	0.88573	3.279446
LRIG1	0.59011	0.281863	0.695876	6.281725
LOC72960З	0.59015	0.281967	0.624752	4.279302
SLU7	0.59015	0.28205	-0.42621	6.807118
TTC3P1	0.59015	0.282093	0.887669	2.815795
LOC64284€	0.59015	0.282129	0.767668	3.860807
FAT3	0.59015	0.282138	1.486932	2.488898
HSP90AA1	0.59015	0.282204	-2.06995	0.377347
SNX29	0.59015	0.282217	-0.4826	6.385715
CLCC1	0.590484	0.282424	1.830644	0.81091
SEPSECS	0.59081	0.282628	0.556088	4.843601
CCDC170	0.590957	0.282808	1.257296	1.86504
SLC4A1AP	0.590957	0.282832	-0.45569	6.232811
CCL13	0.590957	0.282842	1.927683	1.33257
PSAPL1	0.591027	0.282923	-1.17816	2.982906
UBE2J1	0.591052	0.283018	0.433543	6.727443
CDC42SE1	0.591052	0.283031	-1.28902	2.20582
BIN3-IT1	0.591584	0.283345	-1.52579	2.581039
C17orf107	0.591584	0.283382	1.199583	2.645357
BUB1B	0.591657	0.2835	-0.57195	4.795513
TPI1P3	0.591657	0.283512	0.752723	3.387788
YIPF3	0.591676	0.283616	0.445165	6.264061
ZNF124	0.591676	0.283625	1.162444	1.807027
CHRNE	0.591676	0.283692	-1.35933	1.312664
S1PR2	0.591676	0.283713	0.983082	2.812574
ZFP36L2	0.591844	0.283842	0.461928	8.201664
NR2C2	0.592001	0.283965	-2.88477	0.539882
GUSBP3	0.592113	0.284066	1.149803	2.480522
PTPN12	0.592681	0.284394	1.538623	0.645101
FGFR1OP	0.592681	0.284435	1.193509	2.453026
BTBD19	0.592697	0.284491	-0.89842	3.661991
C1orf112	0.592877	0.284625	-0.73802	3.598719
ANKRD24	0.59368	0.285059	0.901705	2.486729
SRRD	0.593802	0.285165	-0.80408	3.597974
UBE2D3	0.594216	0.285412	-0.45107	5.263983
RCBTB1	0.594372	0.28561	0.52661	4.617364
UBE2M	0.594372	0.285668	-0.51265	5.502036
OR7E2P	0.594372	0.285788	1.395618	1.391853
DSCC1	0.594372	0.285809	-1.35224	1.728642
PFDN1	0.594372	0.28581	-0.54243	5.013068
SPTLC3	0.594372	0.285823	-0.56904	4.366994
TRPM5	0.594372	0.285832	1.301354	2.040489
MYL12B	0.594372	0.285872	-2.19757	1.314483
GK5	0.594848	0.286149	0.81851	3.074697

MAPRE1	0.595215	0.286374	-0.42696	6.707248
SLC6A16	0.595271	0.286449	1.274105	2.228751
SYCP2L	0.595434	0.286576	1.350009	1.385949
LCORL	0.595572	0.286691	0.571215	4.057151
NFKBIE	0.595636	0.286827	0.865031	2.967539
LOC10192ε	0.595636	0.286919	-1.43002	2.237301
LRRC45	0.595636	0.286953	0.622956	4.199874
GTF2H1	0.595636	0.286987	2.294739	0.461226
SH2B2	0.595636	0.287057	1.670622	1.164576
IPO9	0.595636	0.287109	-0.45043	6.624202
LOC10028ς	0.595636	0.287128	-0.93075	3.405225
ZNF8	0.595636	0.287149	0.453837	4.721392
TBCD	0.595636	0.287156	0.452791	6.03414
JMJD6	0.596177	0.287508	-1.29488	2.503691
CNOT6	0.596177	0.287598	-0.42556	6.600051
ERP44	0.596177	0.287623	-0.43864	6.989495
RPL32P3	0.596177	0.287638	0.664487	4.088641
KCNAB2	0.596177	0.287658	1.654123	1.174736
BRAT1	0.596234	0.287734	0.582599	4.64558
THBS2	0.596857	0.288083	-0.79145	6.311638
RPS6	0.597002	0.288241	-0.4366	10.38997
DRG2	0.597002	0.28825	-0.56201	4.463518
LRRC4B	0.597328	0.288456	1.743172	1.281557
PHB2	0.597419	0.288548	1.316101	1.406449
HMBOX1	0.597534	0.288652	0.696522	3.333598
FBXL13	0.597586	0.28876	1.749525	1.124774
PHC1	0.597586	0.288774	-0.69304	5.23776
FLJ42102	0.598002	0.289023	2.695666	0.56997
MCRIP1	0.59823	0.289188	-2.56505	0.636202
FAAP20	0.59823	0.289268	0.855844	2.636021
BUB3	0.59823	0.289279	0.52303	4.561228
TMEM258	0.598535	0.289474	-0.68052	3.478398
ZGPAT	0.598638	0.289707	1.471857	1.265282
ZNF367	0.598638	0.289759	0.700404	3.141052
GRHL2	0.598638	0.289788	-0.48725	7.162535
COL18A1-A	0.598638	0.289804	1.27561	1.660462
ENO4	0.598638	0.289813	1.374417	1.312729
ZDHHC20	0.598638	0.289893	-1.92351	1.378891
LINC01644	0.598638	0.289895	1.729091	0.531695
PYGO1	0.598638	0.289916	1.112119	2.908892
EBF4	0.598638	0.289961	1.146903	2.230834
ATAD3C	0.599014	0.290192	-1.65608	1.812015
NAGLU	0.599086	0.290347	0.572617	4.136802
LINC01504	0.599086	0.290381	1.496454	0.728525
CHAC2	0.599086	0.290442	-1.07313	3.136009

EBF2	0.599086	0.290442	1.518142	2.197206
C3	0.599086	0.290469	0.759548	5.437599
PDZD8	0.59927	0.290649	0.425384	7.800367
DIAPH2	0.59927	0.290724	0.913745	2.796196
NPFF	0.59927	0.29073	-1.80784	0.531521
ZEB1-AS1	0.59927	0.290752	1.285081	1.930585
C3orf52	0.599721	0.29102	-2.33798	0.817835
ALKBH7	0.599995	0.291254	-0.68853	3.58279
LOC101929	0.599995	0.291272	1.608439	1.107824
FRMPD1	0.599995	0.291299	-2.09402	1.090039
SMIM26	0.600077	0.291387	-0.78687	2.925276
LOC93429	0.600269	0.291529	-1.89019	1.426858
TIMM29	0.60051	0.291694	-0.98773	2.918606
MRPL37	0.600713	0.291842	-0.47218	5.227654
SNORD76	0.60127	0.292161	-0.53269	7.588557
OXCT2	0.601485	0.292314	2.185201	0.516341
SPECC1L	0.601789	0.292511	-1.70438	1.433774
SNORD58A	0.601912	0.292619	-0.53912	5.944047
SGMS2	0.602163	0.29279	-1.7922	1.580737
ZBTB42	0.602286	0.292898	-0.66272	3.77599
EGR1	0.603046	0.293402	-1.01211	7.962605
FAM174B	0.603046	0.293411	0.729786	4.200449
RAB40A	0.603046	0.293415	1.543208	1.739234
LOC101927	0.603602	0.293742	-2.07989	0.49978
DCAF15	0.603602	0.293796	-0.52049	4.972349
ABCC9	0.603602	0.293832	1.988304	1.365244
DLG1-AS1	0.604006	0.294139	-2.11213	0.522568
C19orf44	0.604006	0.294182	1.44213	0.855848
RHBDD3	0.604006	0.294198	0.821396	2.785786
MRPL40	0.604006	0.294224	-0.57847	4.309377
ATL2	0.60401	0.294275	-1.34544	2.32894
CHST6	0.604071	0.294407	-0.69125	3.951835
ADGRL1	0.604071	0.294412	1.698909	1.261381
SLC31A2	0.604071	0.294461	-0.71207	4.147019
APEH	0.604071	0.29454	0.397038	6.000693
ARHGAP6	0.604071	0.29455	1.881286	0.798934
SMU1	0.60427	0.294696	-0.39889	6.457141
AGPAT4	0.604399	0.294807	-0.45652	5.340696
RBMX	0.604511	0.294911	0.406207	5.369253
GSE1	0.604796	0.295099	-1.55026	1.449725
AKR1B10	0.60494	0.295218	0.467198	7.467632
CD58	0.604957	0.295275	0.719755	3.155403
MIATNB	0.605012	0.295351	0.757255	3.043694
MCMDC2	0.605323	0.295552	-1.57954	1.702129
UNC119B	0.605376	0.295627	0.423966	5.826768

MIR3176	0.605529	0.295777	1.917878	0.765878
NEURL1	0.605529	0.2958	0.926117	3.009751
ZNF629	0.605812	0.295987	0.472588	5.319926
PHYKPL	0.605852	0.296088	1.417518	2.090841
HCRTR1	0.605852	0.296105	-1.88695	0.9541
CUEDC1	0.606197	0.296368	1.931584	0.543473
PAXIP1	0.606197	0.296372	-0.48875	4.840953
PRKX-AS1	0.606335	0.296488	-2.19267	1.051409
ANKRD36B	0.606547	0.296641	0.447046	5.427521
HOXB4	0.606603	0.296718	0.708673	3.733031
ZNF354A	0.606649	0.296828	0.644798	3.969077
SNORD96A	0.606649	0.296839	0.64129	3.731806
COX10-AS1	0.606745	0.296961	-0.73924	3.851625
MIR3175	0.606745	0.297004	1.358657	1.594983
HELB	0.606745	0.297033	0.822049	3.715336
PPP1R3D	0.606839	0.297128	-0.68255	3.930483
HTR3C	0.607404	0.297454	-3.63901	0.772858
YRDC	0.607475	0.297538	-1.06134	3.22935
MIR3671	0.607493	0.297596	2.00026	1.39076
KLHL3	0.607675	0.297734	1.575896	1.254025
VPS29	0.60817	0.29805	1.705826	1.252579
ISCU	0.60817	0.298097	1.371809	1.269327
VGLL4	0.60817	0.298125	0.600217	3.790905
CACNA2D1	0.608334	0.298254	0.648862	3.91078
QSOX1	0.608377	0.298325	-0.49022	7.27953
NUCKS1	0.608385	0.298378	-0.41648	8.735432
ZNF362	0.609079	0.298767	0.468827	4.933985
MANEA-DT	0.609561	0.29914	1.317308	1.981042
DOCK9-AS1	0.609561	0.299146	-1.45709	1.736134
CHRNB1	0.609561	0.299152	-1.11751	2.538867
CSRNP1	0.60959	0.299215	-0.5956	5.868885
DPP9-AS1	0.609595	0.299333	0.965155	3.30556
LINC01589	0.609595	0.299339	1.937186	1.15511
CLDN20	0.609595	0.299366	0.736688	2.864303
FAM111A	0.609615	0.299425	1.34889	1.648344
APOBEC3F	0.610545	0.299932	0.838778	2.265856
USP6	0.610731	0.300073	1.840229	1.267952
CCDC186	0.610849	0.30018	-0.41012	7.769811
LOC642852	0.611404	0.300502	0.557021	4.418841
OTOGL	0.611502	0.3006	1.664491	1.096824
RBPMS	0.611528	0.300662	0.774636	3.490656
SDHAP3	0.611626	0.30076	0.781835	3.508985
NOP53	0.611998	0.300992	0.426362	7.603999
TMEM38B	0.612073	0.30123	0.717818	3.623408
RNU2-1	0.612073	0.301255	-0.99862	3.261569

LYRM4	0.612073	0.301273	-1.75397	0.951876
CYBC1	0.612073	0.301285	0.700966	2.995753
ZNF846	0.612073	0.301287	-0.66977	4.65177
SPINT2	0.612073	0.301327	0.677394	4.135045
SNORD35A	0.612258	0.301478	0.545668	5.230326
PANK2	0.612258	0.301517	-1.00194	2.467438
POMT1	0.612306	0.301621	1.439352	1.125526
MINOS1P1	0.612306	0.301677	0.841422	3.243779
RAG1	0.612306	0.301731	1.244787	2.098481
BAZ1A	0.612306	0.301739	1.326305	1.532872
CASP14	0.612368	0.301819	2.04536	0.480823
SNORA70	0.612415	0.301892	-0.44629	7.720194
LINC00526	0.612465	0.301966	1.748508	1.146718
NORAD	0.612726	0.302145	-0.41258	9.497205
ADAM6	0.612807	0.302238	2.657752	0.881756
MAFK	0.612807	0.302284	0.548411	5.184347
FCRL3	0.612917	0.302425	1.753669	0.750897
RBM4B	0.612917	0.302531	-1.14026	2.33983
ZNF836	0.612917	0.302589	0.637843	4.207691
UBE2Q1	0.612917	0.30261	-0.42039	5.616371
ZNF277	0.612917	0.302626	0.462236	5.023729
FAHD2A	0.612917	0.302676	0.71376	3.817859
ZNF438	0.612917	0.302685	-1.51774	1.840694
LOC64690:	0.613249	0.302899	1.571536	1.016191
TVP23B	0.613949	0.30334	1.093887	2.023153
8-Sep	0.613949	0.303344	1.628287	1.315692
SMTNL1	0.614468	0.303672	1.691933	0.738405
TBCB	0.614468	0.3037	1.026935	1.748336
SCARA5	0.614567	0.303799	1.384263	2.599972
SNHG25	0.614761	0.303945	0.834109	2.848485
SYF2	0.615145	0.304203	0.867332	2.143901
A2M-AS1	0.615145	0.304234	1.787052	1.342688
EDAR	0.615155	0.304297	-0.54501	4.422961
TMEM237	0.615155	0.304339	-1.83188	0.749603
SH2B3	0.615155	0.304389	1.647848	1.53467
EIF2S3	0.615273	0.304497	-0.40531	8.030196
ACTA2	0.615375	0.304597	1.474017	1.195021
EXTL3	0.615381	0.30465	0.456682	4.65437
PCDHB14	0.615638	0.304827	0.65348	3.412929
LOC10537!	0.615801	0.304984	1.644948	1.385523
IRF9	0.615801	0.305024	0.513938	5.334396
ZNF622	0.615801	0.305105	0.439537	5.213881
CWC22	0.615801	0.305107	-0.40041	6.127814
AIMP2	0.615834	0.305209	-0.88253	3.05013
METTL24	0.615834	0.305223	2.083229	0.816784

MYL9	0.61603	0.30537	1.816928	1.250902
HNRNPA3F	0.616082	0.305483	0.868304	2.493557
AHR	0.616082	0.305512	0.420378	7.191277
UNC5A	0.616082	0.305592	1.686601	1.086274
TMEM242	0.616082	0.305597	0.753718	3.948986
CDC37	0.616082	0.305646	0.396137	7.480851
CDC42BPA	0.616101	0.305732	0.950815	2.493536
NRIP2	0.616101	0.30582	0.570145	4.041295
C9orf64	0.616101	0.305846	1.055139	2.409737
BTBD3	0.616101	0.305855	1.731501	0.980812
MOSPD2	0.616148	0.305928	-1.19146	1.597251
TERC	0.616464	0.306162	0.446813	5.78504
RIMBP2	0.616464	0.306277	0.957435	3.273538
WAS	0.616464	0.30628	0.812298	2.628419
POFUT2	0.616464	0.306319	0.980493	1.769906
SLC35D1	0.616464	0.306335	0.388079	5.777897
SFRP4	0.616856	0.306614	1.901693	1.258083
CST3	0.616856	0.306635	0.417287	5.954719
CST7	0.616856	0.306679	1.272732	1.859625
LINC00893	0.616863	0.306733	-0.53033	4.305883
TTPAL	0.617409	0.30707	-3.20747	0.582663
ZNF425	0.617409	0.307104	-1.39637	2.045551
SMIM13	0.617661	0.30728	0.642452	4.224579
SNX27	0.618358	0.307676	0.388924	6.680209
TMEM178I	0.618368	0.30779	-0.93519	3.480724
LETM1	0.618368	0.307791	-0.41914	7.32869
TBRG1	0.618368	0.307832	0.893828	2.023144
INA	0.618424	0.307948	-1.8451	1.319894
HMGN4	0.618424	0.30796	0.443762	4.875674
PRPF31	0.618613	0.308104	-1.59799	1.547506
VAMP2	0.618631	0.308163	-0.43499	5.943635
LOC100507	0.618781	0.308288	0.810022	3.451649
SMS	0.619051	0.308499	1.089466	1.58817
RNA45SN5	0.619051	0.308523	0.476275	15.36888
RPL28	0.619271	0.308726	-0.86302	3.519903
BCKDHB	0.619271	0.308733	-0.96945	3.14678
DNAL4	0.619388	0.308841	0.60467	3.671463
SYBU	0.619396	0.308895	1.993734	0.942062
HRASLS2	0.619556	0.309107	1.651902	1.371781
GMEB2	0.619556	0.309108	-0.55381	4.958745
FBXO4	0.619556	0.309126	1.732216	0.676766
LRRC61	0.619887	0.309377	-1.95227	0.400549
GDNF	0.619887	0.309391	1.874632	1.144788
UHRF1BP1	0.620178	0.309587	0.396732	6.108166
UBALD2	0.620275	0.309712	-0.41893	6.018789

LOC100507	0.620275	0.309735	1.232577	1.80389
SNHG7	0.620523	0.309918	-0.85347	2.563727
FAM222B	0.620523	0.309996	1.289921	0.795895
ZNF705E	0.620523	0.31001	-1.64364	1.309628
SLC5A3	0.621151	0.310374	-0.47268	6.695183
PIM3	0.621511	0.310605	-0.51861	6.470922
DOP1B	0.621682	0.31074	-0.45241	7.61113
LINC01588	0.622203	0.311051	-0.78364	3.067684
SNORA19	0.622515	0.311258	-0.80783	3.152166
IRF2BPL	0.622531	0.311316	-0.5465	4.708716
CHST1	0.622609	0.311405	-2.442	1.162042
PHLPP1	0.622667	0.311502	-0.42313	5.718502
SNORA18	0.622667	0.311535	-0.46394	6.906392
INCENP	0.622671	0.311587	2.178957	0.927329
MIR6814	0.622681	0.311643	-1.29885	1.950459
BCAR1	0.622764	0.311735	0.827254	2.44609
DHDH	0.623272	0.31204	1.724446	0.488018
MGST3	0.623313	0.312111	0.492117	5.111787
GDF7	0.623694	0.312352	-1.92068	0.898764
ATP1A4	0.624448	0.31278	-2.88787	0.821004
FAM151A	0.624713	0.312973	0.840729	2.133195
XK	0.624713	0.313014	0.619327	4.242846
VWA3B	0.624897	0.313219	1.864876	1.184082
SNCG	0.624897	0.313255	-1.42491	2.86233
FAM200B	0.624897	0.313258	0.513529	4.413366
SNORD9	0.625049	0.313428	0.508938	4.960722
NIF3L1	0.625049	0.313436	1.725854	0.700036
PPP1CC	0.625049	0.313508	-0.51849	4.77629
NEK9	0.625049	0.313543	0.387659	6.786106
VCP	0.625049	0.313592	-0.40992	8.413206
COX6B1	0.625049	0.313638	-0.44256	7.085898
PIGL	0.625214	0.313804	0.577035	3.864376
C17orf97	0.625214	0.313847	0.569622	3.896155
METTL16	0.625214	0.313923	-0.4087	5.254237
INKA1	0.625214	0.313924	-1.06666	2.454601
TEPSIN	0.625235	0.313999	0.562411	3.883533
RBSN	0.625235	0.314105	-2.30343	0.560493
EIF4G1	0.625235	0.314169	-1.41744	0.770933
DUSP23	0.625235	0.314175	-1.06781	3.090078
TMPRSS2	0.625235	0.314217	1.311338	1.524936
RAB32	0.625235	0.314266	1.066014	1.803647
INPP5A	0.625235	0.314289	0.483177	4.740861
BOP1	0.625272	0.314358	0.394034	5.391143
DMXL1	0.62631	0.314931	-1.30861	1.694217
LINC00511	0.626413	0.315054	1.527885	0.689186

RPS21	0.626413	0.315084	-0.43295	7.512063
PRR12	0.626721	0.315324	0.39963	6.34357
TRIB2	0.626721	0.31534	0.694025	3.781294
UBN2	0.626726	0.315395	-0.46521	7.563824
RTN4RL1	0.626726	0.315444	-1.98489	0.809795
PRIM2	0.626746	0.315505	0.691363	2.829984
ELOVL5	0.626785	0.315575	-1.24492	2.39948
VOPP1	0.626828	0.315691	1.025537	2.26223
RAMP2	0.626828	0.315699	1.316515	1.514197
CCDC112	0.626925	0.315798	1.152496	0.957015
NRSN2-AS1	0.627056	0.315974	1.143506	2.483601
HOXA4	0.627056	0.315985	-1.17449	2.291841
GMPPA	0.627056	0.316017	1.270035	1.583864
NBPF9	0.627696	0.316393	-1.91367	0.499374
PPP1R2B	0.627696	0.316495	-0.6547	3.82508
DTX3L	0.627696	0.316664	0.405221	6.844371
STMND1	0.627696	0.316666	-2.75924	0.764665
RTTN	0.627696	0.31668	-0.54327	4.64488
TMED1	0.627696	0.316691	-1.47316	1.173128
CCDC28A	0.627696	0.316695	-0.56867	4.442446
ZNF148	0.6279	0.316849	-0.3969	7.006034
CLPTM1	0.628171	0.317037	1.230376	0.976779
LRRN1	0.628362	0.317199	0.962727	2.416785
LINC00337	0.628362	0.317257	-2.79801	0.574075
GSS	0.628362	0.317286	-0.43383	5.166288
PDS5A	0.629008	0.317702	-0.39189	7.547249
ASPH	0.629008	0.317714	0.589247	3.973557
BRD7P3	0.629026	0.317774	1.507038	1.490812
GEMIN6	0.629182	0.317904	0.720259	3.415304
SNHG15	0.629209	0.317968	-0.999	2.888119
PDCD6	0.629248	0.318039	0.710836	3.372806
PAFAH1B2	0.629289	0.318111	-2.0421	0.752137
GTSE1-DT	0.629513	0.318275	1.860539	1.117941
MESTIT1	0.629665	0.318512	1.288993	1.01518
SAFB2	0.629665	0.318552	0.381874	7.320442
ATP6V0E1	0.629665	0.318556	-0.44187	5.853836
SNX18	0.629665	0.31866	1.756205	1.67502
FAM102A	0.629665	0.318672	-0.55042	5.594323
DNMBP-AS	0.629665	0.318689	-2.17163	1.092108
DDOST	0.629665	0.318709	0.401675	6.635245
LIPH	0.629914	0.318886	0.445703	6.171601
BTBD18	0.630047	0.319032	-0.8639	2.758271
MIPEP	0.630047	0.319148	-0.48654	4.608857
SNORA13	0.630047	0.319154	0.475848	5.464115
TGIF1	0.630047	0.319157	0.882659	2.264481

NEMF	0.630049	0.319224	-1.82631	1.232678
ISX	0.630049	0.31926	2.567328	0.552596
CXCL6	0.630144	0.31936	-2.45774	3.023644
IRF2	0.630244	0.319507	-0.40824	6.132816
EPHB6	0.630244	0.319562	-1.75486	0.724191
RNU5E-1	0.630244	0.319677	-0.66611	6.550768
SLC22A3	0.630244	0.319719	-0.84187	4.205006
TIPARP	0.630244	0.319754	-2.09185	1.522053
MAP1LC3A	0.630244	0.319788	1.112505	0.92666
NPIPB15	0.630244	0.3198	-1.27671	2.991328
LYRM9	0.630244	0.319818	1.100462	2.319923
SPTLC1	0.630386	0.319955	-1.02012	2.094201
DIRC3	0.630386	0.319992	1.517727	1.473326
SAAL1	0.630535	0.320119	0.801658	3.254611
PLAC8L1	0.631277	0.320547	1.157363	2.028972
CNR1	0.631283	0.320601	1.967382	0.718131
PRPF38A	0.631698	0.320902	-0.38838	5.988483
CAD	0.631698	0.320914	1.31711	0.659651
SLC20A1	0.631785	0.32101	-0.67437	6.423474
OR7E47P	0.631816	0.321093	1.649239	0.657138
ZNF527	0.631816	0.321151	0.630943	3.76335
GNG5	0.631816	0.321179	-0.45181	5.581822
FGD6	0.631913	0.32128	0.394007	6.446816
TRAPPC8	0.63227	0.321624	-0.40224	7.026509
PTPN7	0.63227	0.321641	1.603004	0.652304
ANKLE2	0.63227	0.321644	-0.40969	7.169799
AGGF1	0.63227	0.321699	-0.40702	6.383048
ACBD3	0.63227	0.321717	-0.40592	8.217521
EEF1G	0.632667	0.322011	-0.44301	9.265351
KCNK15-AS	0.632667	0.322022	1.837903	1.219457
SNORD30	0.632861	0.322171	1.234195	1.33638
ZNF680	0.632928	0.322257	-0.61068	3.93009
MAP2	0.632937	0.322313	-1.13818	3.456414
KIF9-AS1	0.63299	0.322391	1.38454	1.108874
CTNNA1	0.633037	0.322545	-1.29786	2.379263
ICAM1	0.633037	0.322577	0.658892	3.620466
ZNF879	0.633037	0.32258	-1.42953	2.213733
NRSN1	0.633037	0.32262	2.222464	0.626801
ATXN1	0.633194	0.32276	-2.32416	0.653642
ZNF271P	0.633194	0.322844	1.91008	0.729305
GLUL	0.633194	0.322872	-0.61436	5.708313
FAM49A	0.633194	0.323002	0.819046	3.620181
ABCA11P	0.633194	0.323052	0.940519	2.790081
LARP1B	0.633194	0.323195	-0.57824	3.809475
C3AR1	0.633194	0.323242	1.362664	2.179124

LINC02449	0.633194	0.323259	-1.96386	0.574411
NT5M	0.633194	0.323286	1.384616	0.828597
TTC19	0.633194	0.323298	-2.39836	1.134187
ZNF75D	0.633194	0.323302	0.903828	3.525437
SRSF3	0.633194	0.323315	-0.93832	3.19565
WBP1L	0.633272	0.323407	0.943869	2.584787
TTC37	0.633787	0.323721	-0.39595	7.22576
SNX16	0.634319	0.324044	-2.17409	0.709367
C17orf75	0.634414	0.324144	-0.70881	3.190382
NINL	0.63457	0.324314	-0.42168	6.21832
ZNF324B	0.63457	0.324348	-0.95597	2.770138
ADCY2	0.63457	0.324428	1.513252	1.815879
FARP2	0.63457	0.324465	0.572524	3.868827
MIR604	0.63457	0.324481	-1.23908	1.721542
GORASP2	0.635202	0.324876	-1.98933	0.550262
PARP4	0.635202	0.324928	0.373814	8.265449
GTF3C2	0.635202	0.324987	-1.56996	1.634865
ATP10A	0.635202	0.32501	0.859381	3.228254
IGIP	0.635389	0.325199	0.529856	4.416992
GRWD1	0.635389	0.325208	-0.54531	4.906793
SEC31B	0.635401	0.325266	0.428267	5.177989
ANKRD13C	0.635491	0.32539	0.429797	6.031466
NRSN2	0.635491	0.325444	-0.92492	2.512075
NACAD	0.635491	0.325466	1.426899	1.476923
CC2D2B	0.635878	0.325716	1.476516	1.5384
FLJ12825	0.636032	0.325847	-1.66513	1.261863
HEPHL1	0.636059	0.325912	-1.05155	5.064983
ZBTB43	0.636156	0.326013	-2.12272	0.675024
ZNF469	0.636333	0.326165	0.910083	3.676308
OSTCP1	0.636333	0.326207	1.802733	1.17099
GOLGA8B	0.636438	0.326312	0.707749	3.065477
RAD21	0.636474	0.326382	-0.40952	8.124548
PDXDC2P-N	0.636512	0.326485	0.397088	7.464548
PRICKLE2	0.636512	0.326505	0.587426	4.721273
PNMA1	0.636624	0.326761	0.505342	4.695278
SNORD114	0.636624	0.32681	1.366358	0.787932
LRP1-AS	0.636624	0.326899	-2.57554	0.974557
LINC01252	0.636624	0.326923	-1.96463	0.936379
PIAS1	0.636624	0.326961	-0.40088	6.509725
DDX47	0.636624	0.326962	-2.39455	0.639759
RIN3	0.636624	0.32697	0.58975	4.716981
ECHDC2	0.636624	0.326988	-1.38844	1.995964
APTR	0.636624	0.327026	0.657428	3.306908
LINC01273	0.636659	0.327128	-3.09443	0.506528
C19orf54	0.636659	0.327148	0.525407	4.31715

CCDC89	0.636792	0.327267	1.223126	1.590722
MIR4454	0.637372	0.327659	1.686295	0.56734
GBP2	0.637372	0.327669	0.419665	6.879425
GKN1	0.637423	0.327747	-2.87062	4.797334
SPHK1	0.637479	0.327827	-1.46697	0.996259
MYLK4	0.63751	0.327895	-0.93434	2.774211
RUSC2	0.637567	0.328021	-1.44944	1.054734
ZNF543	0.637567	0.328027	-0.57081	4.488823
ZNF714	0.638055	0.32833	-0.54642	4.868321
LINC01599	0.638163	0.328437	-1.43287	2.019482
INTS4	0.638376	0.328599	0.394683	5.042838
LINC00102	0.638431	0.328678	1.838377	0.940403
FLOT2	0.638554	0.328794	-0.46761	6.390406
SET	0.639058	0.329116	0.616883	3.973636
LINC00839	0.639058	0.329157	1.166231	1.646512
RBKS	0.639136	0.329253	1.724555	0.472912
CDK4	0.639136	0.3293	0.496686	4.937845
FAM185A	0.63922	0.329396	1.532692	1.136053
ELK2AP	0.639472	0.329612	1.105594	3.481804
HNRNPUL2	0.639472	0.329634	-0.37818	5.942443
ARL16	0.639472	0.329681	-0.56035	3.999394
MAP6	0.639926	0.330024	1.758101	1.139937
PLPP2	0.639926	0.330085	1.723873	1.052889
HIP1R	0.639926	0.330107	0.443531	6.033971
SLC2A1-AS	0.639926	0.330122	-1.72328	1.662259
FLJ37453	0.639942	0.330182	0.890803	2.575682
UGT8	0.641149	0.330896	1.820545	1.103234
ZNF581	0.641149	0.330937	-0.87045	2.947522
STAG2	0.641149	0.330961	0.917858	2.175882
XKR8	0.64128	0.33108	0.65703	2.94031
LOC10050(	0.641305	0.331145	1.634024	1.57143
PLAGL1	0.64156	0.331329	-0.95604	3.226167
STBD1	0.641624	0.331414	1.044484	1.85598
LRRC3B	0.641751	0.331578	1.706954	1.030612
PPP1R3B	0.641751	0.331619	-1.68723	1.088872
NUDT3	0.641751	0.331636	0.717288	2.886872
CFAP54	0.642194	0.331957	1.279421	2.749749
ZNF704	0.642194	0.331978	0.49508	5.163086
PDF	0.642194	0.33202	-1.5503	1.709157
HLA-DQB1	0.642281	0.332118	1.900679	0.698325
MPV17	0.642489	0.332277	-0.61359	3.695905
CAPN3	0.642506	0.332338	-1.13227	2.685399
MYO3B-AS	0.643124	0.332721	-2.72253	0.73918
SELENOW	0.643124	0.332762	0.376482	6.049268
FAM122B	0.64314	0.332825	-0.70498	3.224541

CMC2	0.64314	0.332874	-0.87481	3.366444
CNPY2	0.643419	0.333071	0.457656	4.422168
METTL14	0.643551	0.333191	0.395474	5.366517
ITPRID2	0.643824	0.333446	0.541381	4.615311
LOC101927	0.643824	0.333449	-0.95373	2.981266
LINC00963	0.643824	0.333489	0.580783	3.74945
NAT14	0.643991	0.333628	1.439244	1.059327
SNORD38A	0.644111	0.333742	0.608403	4.58492
FSD1	0.644299	0.333892	1.850882	0.887098
IL1R2	0.644361	0.333976	1.448486	0.792291
DENND1B	0.644439	0.334143	-0.87678	3.342056
FAM81B	0.644439	0.334152	1.553712	0.590675
FCHSD2	0.644439	0.334196	0.465444	5.621164
POLRMT	0.644439	0.334225	0.394484	5.480827
SLC7A11-A	0.64465	0.334424	-1.05216	2.625217
C17orf51	0.64465	0.334461	0.603635	3.919675
CCPG1	0.64465	0.334569	1.839866	0.807659
ZNF329	0.64465	0.334571	-0.87392	3.227209
CPSF2	0.64465	0.33461	-0.39028	6.84884
LOC101928	0.64465	0.334648	1.1729	1.639369
LENG8-AS1	0.644681	0.334762	0.588897	3.748601
SWI5	0.644681	0.334768	-0.65714	3.591503
GPD2	0.644747	0.334892	1.40547	1.455089
RELB	0.644747	0.334918	0.6915	3.530294
LMBRD1	0.644747	0.334959	-0.40703	5.669124
DLGAP1-AS	0.645018	0.335152	1.982482	0.4315
GRIK3	0.646165	0.335801	1.623446	1.76862
SRSF2	0.646322	0.335935	0.505058	4.245114
EP400P1	0.646701	0.336234	0.63425	3.322659
KPNA6	0.646701	0.336242	-0.3987	7.616489
RNY1	0.646701	0.336289	-0.45024	8.665232
MDFI	0.647254	0.336638	-1.62952	1.095549
CPSF6	0.647254	0.336734	1.453098	2.268382
SNHG9	0.647254	0.336756	-1.78753	0.869872
RNF217-AS	0.647254	0.336798	-2.01953	1.441121
DPY19L2P4	0.647254	0.336858	1.437633	0.783496
TEX264	0.647254	0.336935	1.753641	0.633854
FAM118A	0.647254	0.336943	-1.85071	0.994681
PCDHB2	0.647318	0.337029	1.093781	1.914691
SLC38A6	0.647654	0.337256	-1.27757	1.467851
GSN	0.647867	0.33742	0.765582	2.392573
SNX8	0.647912	0.337496	0.460687	5.028597
CELF2	0.64795	0.337568	1.125223	2.473785
BMPER	0.648052	0.337674	1.636906	1.214925
GPR162	0.648103	0.337789	1.603514	0.911897

HULC	0.648103	0.337805	-2.72695	0.524777
APOD	0.648491	0.338081	0.780315	4.040546
LINC00954	0.648491	0.338113	1.112943	2.127839
CRIM1-DT	0.648925	0.338391	1.176726	1.078965
FAM162B	0.649161	0.338567	1.514127	0.92456
MIS18A	0.649161	0.33868	-0.64781	3.533531
MIR5003	0.649161	0.338689	1.72634	0.703229
ZNF350	0.649161	0.338725	-0.90616	3.277833
SPATA2L	0.649235	0.338816	0.744656	2.887979
EEF1D	0.649553	0.339068	-2.15853	1.655457
BMS1P2	0.649553	0.339087	0.411447	6.397368
ACTL10	0.649569	0.339148	1.210235	2.078384
DOCK7	0.651303	0.340111	1.189016	1.773153
PPM1A	0.651303	0.340164	-0.84235	4.166741
MIR5192	0.651303	0.340212	-1.92477	1.487243
MRPL54	0.651311	0.340269	-0.65672	3.608901
ZNF506	0.651468	0.340447	1.197198	1.62818
PMM2	0.651468	0.340456	0.362626	6.291505
PARP1	0.651965	0.340769	0.352038	7.097065
AKIRIN1	0.651967	0.340823	1.166834	1.924794
TTBK2	0.652142	0.340967	-0.76103	4.888086
SFRP1	0.652164	0.341031	-0.68746	4.215177
CAPN10	0.652166	0.341085	-1.69295	1.005752
SNORD45A	0.652337	0.341227	0.430564	8.72587
DCPS	0.652437	0.341332	0.597929	3.239094
CNTN3	0.652702	0.341524	1.539145	1.840799
CGRRF1	0.65275	0.341623	1.008897	2.80781
SCART1	0.65275	0.341655	-0.51215	4.935537
LOC101927	0.653301	0.342009	-1.25612	1.762094
HBS1L	0.653301	0.342049	-0.65278	3.246727
A1BG	0.653385	0.342167	1.51686	0.668721
RPS18P9	0.653385	0.342199	0.968008	1.776914
NOL8	0.653458	0.342331	1.375575	1.384294
ZBTB46	0.653458	0.342343	0.866795	3.176521
ASCL1	0.653632	0.342487	1.759347	0.663461
PDP2	0.653702	0.342577	-0.53809	4.737631
RAI14	0.654074	0.342824	-1.66849	1.154774
TMEM161I	0.654665	0.343188	1.231012	2.704672
HSD17B14	0.654667	0.343242	1.288101	0.983152
PACSIN2	0.654774	0.343351	0.817383	3.464777
TMEM115	0.654792	0.343426	0.399547	4.854911
CLDN7	0.654792	0.343466	-0.86221	3.046234
MRPL45P2	0.654922	0.343635	-0.77588	3.589971
NACA	0.654922	0.343641	-0.50749	4.65298
ARFGEF2	0.654955	0.343711	-0.36565	7.854724

COL19A1	0.654963	0.343768	2.115956	1.039699
YKT6	0.655281	0.343988	-0.38981	6.634183
KCND1	0.65622	0.344606	0.965893	2.128108
NDC80	0.65622	0.344651	-0.81008	3.493581
MED31	0.65622	0.344668	-1.57488	1.597177
SSR2	0.65622	0.344694	0.350958	5.559911
YBX3	0.656554	0.344996	-0.69771	3.61575
LINC02443	0.656554	0.345013	-2.52969	0.605539
ITIH3	0.656554	0.345028	-2.94858	0.671691
TNFSF10	0.656569	0.345089	-0.71518	3.005285
SNORD1C	0.656681	0.345201	0.619551	4.742934
SLC5A1	0.656854	0.345346	1.252238	1.387785
FA2H	0.656892	0.345419	0.611792	4.274804
KIDINS220	0.657963	0.346084	-0.40132	8.100246
ISL1	0.657963	0.346089	1.613454	2.008957
LOC10192ε	0.658131	0.346231	1.791941	0.461386
SNORA69	0.658752	0.346611	0.946584	2.577701
TSEN54	0.658753	0.346683	-0.54047	4.200885
KANTR	0.658753	0.346718	0.570061	3.754176
LINC02541	0.659359	0.347092	-2.0694	0.49432
HOXA5	0.659359	0.347143	0.946405	3.105554
KCTD20	0.659813	0.347436	-0.94305	2.912665
MYO19	0.659825	0.347499	1.214986	1.725946
RIBC2	0.659825	0.347549	-1.51737	1.325095
ENAM	0.659933	0.347694	1.498016	1.94865
DDX11-AS1	0.659933	0.347718	1.395337	0.928641
SCRG1	0.659933	0.347767	1.459216	1.571007
SNORD45C	0.660241	0.348021	0.443569	6.419933
FBL	0.660241	0.348035	0.379827	6.870948
LPP-AS2	0.660411	0.348179	0.672495	3.192483
DNM1P46	0.660422	0.348238	1.284938	2.088294
EVI5L	0.660502	0.348334	1.53818	1.362766
TPM3P9	0.660526	0.348512	0.490654	4.662767
ARMH3	0.660526	0.348535	-0.40105	5.991952
UBR1	0.660526	0.348537	0.414327	6.403301
NPM1	0.660526	0.34856	-0.84901	2.43355
LOC10030:	0.660591	0.348658	-0.9525	2.586044
SNORD32A	0.660591	0.348702	0.593977	3.705291
LRRTM2	0.660838	0.348886	-0.51648	6.11317
TPCN1	0.660896	0.34897	-1.64785	1.629723
SPTA1	0.661079	0.34912	1.863285	1.097155
PLIN4	0.661297	0.349289	-1.12662	2.515475
GSPT2	0.661369	0.349388	-1.07474	2.592661
SNORA6	0.661369	0.349472	-0.44964	8.020491
AK4	0.661369	0.349487	1.477396	1.123466

ZW10	0.661376	0.349545	-0.47577	4.921136
PSME4	0.662556	0.350276	-0.38293	7.351459
PLPP3	0.662556	0.350293	0.433947	5.321259
OTUD5	0.662556	0.35038	-0.75519	2.73617
DIP2A-IT1	0.662556	0.350406	0.872205	2.378124
RNF216P1	0.662556	0.350491	-1.41554	1.403927
GOLGA8R	0.662556	0.3505	1.503868	1.361161
PRR14	0.662556	0.350575	-0.47068	4.849671
KLF5	0.662556	0.350598	-0.51183	6.950914
NEK8	0.662667	0.35075	0.837477	2.704292
RELL2	0.662667	0.350764	-2.08064	0.651884
UBTF	0.662993	0.351008	1.150641	2.170154
SPINK1	0.662993	0.351044	1.28312	3.32637
LINC00662	0.66309	0.351149	0.900627	2.581959
MESD	0.663387	0.35136	-0.47822	4.666927
ANP32A-IT	0.663467	0.351456	0.744202	3.609181
SLC27A5	0.663682	0.351634	0.937346	2.194183
ELL	0.663682	0.351677	-0.43541	5.099276
OMG	0.664287	0.352071	-0.99132	2.819968
GPR155	0.664287	0.352108	-1.72384	0.697933
PTDSS1	0.664287	0.352159	0.94972	2.011988
TMA16	0.66444	0.352294	-0.47372	4.428842
CRKL	0.664526	0.352394	-0.38351	6.975941
NUP88	0.664534	0.352571	-0.39024	5.532609
FGD5-AS1	0.664534	0.352577	1.279191	1.627661
GLIPR1	0.664534	0.352581	0.530472	4.164566
SNORA71D	0.664534	0.352613	-0.51714	5.72075
NOD1	0.664561	0.352718	0.404499	4.716847
LOC284412	0.664561	0.352825	0.721515	2.720375
PIP5K1P1	0.664561	0.352865	1.499642	1.229502
NEMP1	0.664561	0.352887	-1.93444	0.553337
FER1L5	0.664561	0.352897	-1.22772	2.142439
LINC01354	0.664568	0.352954	1.435282	0.535679
DSTNP2	0.665138	0.353375	-1.49871	0.924161
SNORA30	0.665138	0.353384	-0.98414	2.518301
SPIN2A	0.665138	0.35349	1.054239	1.912424
JPT1	0.665138	0.353576	0.991423	1.691792
DMRTA2	0.665138	0.353579	1.108083	1.832072
WASHC5	0.665138	0.35358	-0.39788	5.961794
TMEM167I	0.665267	0.353703	-0.37453	5.188547
PRXL2A	0.665287	0.353787	1.638666	1.080422
SEC14L1	0.665287	0.353821	-1.12326	2.335307
TLR7	0.665521	0.354075	1.540492	0.772374
HNRNPA0	0.665521	0.354122	0.358037	6.685023
CD2BP2	0.665521	0.354131	1.817575	0.526602

TMEM260	0.665521	0.354161	0.411401	4.850806
SNORA77	0.665529	0.354219	-1.56445	1.129174
ZNF283	0.66575	0.354391	1.504446	0.69866
TEDC1	0.665833	0.354489	1.373611	1.07878
HIST1H4J	0.665962	0.354636	1.005419	2.314938
DHRS13	0.665962	0.354665	-0.73101	2.69936
TLE3	0.665985	0.354774	-2.06024	0.49034
KPNA7	0.665985	0.354785	1.286113	1.854963
ADGRB1	0.666084	0.354892	-1.16511	2.588652
ANXA11	0.666665	0.355284	-0.71686	3.757341
LOC10050(	0.666665	0.355333	1.047458	1.756628
EGR3	0.666665	0.355364	-2.57737	0.762787
LNCTAM34	0.666758	0.355467	1.160531	2.41621
JPH1	0.667032	0.355672	1.362427	1.441888
LOC10192[	0.667032	0.355749	1.202505	1.871508
CDK7	0.667032	0.355776	-1.24855	2.854466
ASAH2B	0.667113	0.355873	-2.03857	1.102577
MFGE8	0.667204	0.355975	-1.46331	1.206207
IPO5	0.667235	0.356046	-0.3651	7.299322
MIR3170	0.667465	0.356223	1.94825	0.808857
MIR6769A	0.667499	0.356295	1.593925	1.700206
C1orf116	0.66766	0.356602	-1.35294	1.864385
SNU13	0.66766	0.356622	1.206555	0.991275
SLC5A6	0.66766	0.356623	1.301428	1.013315
CNTNAP3B	0.66766	0.356641	0.845498	2.71622
SMG1P5	0.66766	0.356652	0.721848	2.710142
CRB3	0.668163	0.356998	-1.81393	0.93866
ESD	0.668163	0.357028	-0.50756	4.543253
TJP3	0.66874	0.357404	0.902211	2.286045
SVIP	0.66874	0.357445	0.516044	4.631477
CAPNS1	0.668797	0.357529	-1.12088	1.724693
ZNF346	0.668921	0.357688	1.703778	0.819997
LRRC37A8I	0.668921	0.357805	1.275926	1.53155
PXMP2	0.668921	0.357821	-0.84452	3.536647
XPA	0.668921	0.357936	1.319492	1.405634
DUSP16	0.668921	0.357975	-0.41083	7.011132
CDH23	0.668921	0.35799	-1.36677	1.367126
ZNF358	0.668921	0.357996	0.514886	4.236999
KRI1	0.668921	0.358029	-0.38624	6.003193
IDH3B	0.668974	0.358112	-1.13713	2.315404
LOC10028[	0.669102	0.358261	1.381438	0.676322
FAM198B	0.669102	0.358336	1.651117	0.6643
DGLUCY	0.669102	0.358343	1.073204	2.170825
TSHZ3	0.669205	0.358482	0.648225	4.055931
MYO1G	0.669205	0.358507	0.951332	2.811203

NRG4	0.66947	0.358703	0.711875	3.252818
SNORD116	0.669597	0.358849	-0.92619	3.538583
NADSYN1	0.669597	0.358879	-0.41603	6.477463
FNBP4	0.669657	0.358982	-0.35906	8.196514
ATAD3B	0.669657	0.35902	0.591958	3.426786
PPM1L	0.66978	0.35914	-0.90573	2.622919
ZNF616	0.669979	0.359301	0.485325	3.972017
RNVU1-3	0.670086	0.359509	1.851365	1.019872
AMZ2P1	0.670086	0.359565	0.72041	2.809797
RASSF1	0.670086	0.359578	-1.38232	1.466724
RBM23	0.670086	0.359664	-1.48502	1.3323
SHCBP1	0.670086	0.359786	-0.62577	3.265581
SUN1	0.670086	0.359857	0.936259	2.23647
PTBP3	0.670086	0.359861	-1.25991	1.839246
FAF2	0.670086	0.359899	-0.36464	6.338294
KDM7A-DT	0.670086	0.359984	0.540469	4.754621
MYO18A	0.670086	0.360002	1.621993	0.87281
SPDYE6	0.670086	0.360042	-0.55273	4.245818
STUM	0.670086	0.360082	1.402896	2.109674
CHD2	0.670086	0.360167	-0.36475	8.924299
NHLH1	0.670086	0.360272	0.897219	2.480945
RMST	0.670086	0.360311	-1.77988	2.434424
TMEM254	0.670086	0.360314	-1.08995	2.59387
RPL30	0.670086	0.360314	-0.38144	7.944047
TANC2	0.670086	0.360336	-0.37425	6.996463
EZH1	0.670512	0.360619	-0.38507	6.526413
MIR3162	0.670672	0.360803	1.770192	1.729904
OAT	0.670672	0.360813	-0.67424	3.272062
MOB1B	0.671247	0.361177	-1.48295	1.068539
COLEC10	0.671405	0.361328	-2.47482	0.717821
SH2D1B	0.671405	0.361371	1.222369	1.844657
FRG2C	0.671494	0.361482	-1.57318	0.713078
NUP37	0.671494	0.36158	-0.64433	3.636668
TRIM28	0.671494	0.361592	0.347022	6.871671
SLC25A3	0.671494	0.361637	-0.82824	2.410817
C20orf27	0.67161	0.361754	1.911454	1.387103
MKNK1-AS	0.671843	0.361964	0.63898	2.811894
CARMIL3	0.671843	0.362034	-1.1312	2.119041
MCIDAS	0.671843	0.362042	1.764575	0.568288
DDX12P	0.672004	0.362234	0.508324	4.329575
POLR2C	0.672004	0.362238	0.390876	5.28333
NCOA2	0.672321	0.362496	-0.39255	7.424264
NBPF20	0.672321	0.362517	0.675514	2.849694
ZNF791	0.672581	0.362776	-0.5245	5.124467
SLC22A4	0.672581	0.362777	0.950638	1.867903

FAM122A	0.672581	0.362821	0.456183	4.944338
ZNF768	0.673015	0.36311	0.416062	4.885828
BRD3	0.673071	0.363212	0.376732	6.130822
C17orf100	0.673071	0.363249	-1.46235	1.989497
TIMM44	0.673715	0.363651	0.374483	5.136787
IQSEC3	0.673873	0.363791	1.174174	1.336649
GPR4	0.674158	0.363999	1.016626	2.271292
NKTR	0.674288	0.364124	0.360248	8.68807
AMY2B	0.674403	0.364321	0.60888	3.665508
DLSTP1	0.674403	0.36433	0.641339	3.531205
ZC3H15	0.674403	0.36435	-0.38851	7.033785
SCARNA7	0.674753	0.364594	0.342626	7.918504
JAK1	0.674926	0.364742	0.34137	7.726834
PKDREJ	0.675229	0.364961	0.929715	1.658627
LRRC8C	0.675747	0.365296	0.518527	4.448619
TRIR	0.675781	0.365368	-0.40595	6.774763
LMAN1	0.675844	0.365457	-0.36663	8.160301
EEFSEC	0.676039	0.365685	-0.5232	3.901168
HIST1H3E	0.676039	0.365709	-0.88545	2.552681
FOXJ1	0.676039	0.365727	1.396855	1.176432
ZNF276	0.676089	0.365808	-1.75112	1.356501
ZC3H7B	0.676216	0.365942	0.349005	6.785526
LINC02145	0.676216	0.365987	1.85585	0.963987
GLS	0.676393	0.366137	0.369674	5.654368
UTP3	0.67653	0.366267	-0.51612	5.134958
IRF2BP1	0.676716	0.366451	0.462037	4.368091
SRL	0.676716	0.366512	1.310843	1.768206
ADAMTSL4	0.676716	0.366554	-0.69771	4.022734
FADS3	0.676716	0.366613	0.843475	2.907493
CCDC26	0.676716	0.366642	-2.34239	1.089002
ATP6V1B2	0.676803	0.366743	-0.3779	6.325519
ZSCAN31	0.677678	0.367316	-1.66846	1.533966
NEXN-AS1	0.677678	0.367327	1.500872	1.158865
ATP5F1D	0.677856	0.367479	-1.5386	0.607878
SECTM1	0.6781	0.36769	0.630382	3.416486
LTBP2	0.6781	0.367721	0.365285	6.207094
TMEM108	0.678118	0.367835	1.988743	0.896324
EVC	0.678118	0.367866	1.357012	1.672972
FAM86B3P	0.678118	0.367926	1.065167	2.094688
NAA16	0.678118	0.367991	0.393162	5.030211
ZNF880	0.678118	0.368005	0.568401	3.623691
SCARNA9L	0.6782	0.368105	0.559818	3.75976
ELP6	0.678202	0.368185	0.576196	3.765359
GUCA1C	0.678202	0.368265	2.30178	0.744154
LOC72867[illegible]	0.678202	0.36827	-1.58821	1.072814

DCAF7	0.678824	0.368709	0.329522	6.476884
ATAD2	0.678824	0.368718	-0.38986	6.408713
HNRNPF	0.679109	0.368979	0.650641	2.949582
OCEL1	0.679109	0.368983	0.797073	2.168624
MIR570HG	0.679368	0.369263	-0.95327	2.59204
CAP2	0.679368	0.369318	-0.90355	3.167405
GCNT4	0.679368	0.369331	-0.86811	3.075947
LRCH1	0.679368	0.369344	0.625078	3.864138
DHX8	0.679423	0.369461	-0.56817	3.438956
CNTLN	0.679423	0.369483	0.7257	3.859117
PTGER3	0.679481	0.36957	-1.73435	3.59877
TP53I11	0.679924	0.369906	1.149834	0.895348
LINC01238	0.679924	0.369921	1.05772	2.253802
ANKRD50	0.680074	0.370058	-0.90724	3.334488
TOP3B	0.680568	0.370432	1.043847	2.021992
LOC100134	0.680568	0.370437	0.721116	2.671204
IMPACT	0.680579	0.370498	0.367847	5.787256
LINC00473	0.680606	0.370568	-2.42453	1.016748
ZCCHC4	0.680673	0.37066	0.572928	3.927335
IRAK3	0.680692	0.370753	1.583735	0.596631
LPIN3	0.680692	0.37078	-2.14948	0.827975
VEGFA	0.681216	0.371121	-1.00715	2.883433
LOC100287	0.681338	0.371287	-1.89522	0.886181
MSN	0.681338	0.371301	-0.44972	8.315798
TRMT61B	0.681338	0.371376	0.453902	4.011644
OXCT2P1	0.681338	0.371609	1.518953	1.015202
MIR4802	0.681338	0.371636	-1.29991	1.856789
TMEM232	0.681338	0.371695	1.539424	0.948835
TCHP	0.681338	0.371759	-1.15159	1.833821
NAV2-AS4	0.681338	0.371779	1.523082	1.574519
C12orf71	0.681338	0.371781	-1.69046	0.940293
S100Z	0.681338	0.371829	1.637495	1.12641
YDJC	0.681338	0.371831	0.645548	3.376627
PRAM1	0.681338	0.371849	1.348108	0.60066
LINC00597	0.681424	0.371952	0.791114	2.514208
ZNRD1ASP	0.681666	0.372191	0.733172	2.896751
LOC284009	0.681666	0.372231	1.067579	1.686633
ARID5A	0.681666	0.372292	0.658808	3.068697
LINC00506	0.681666	0.372319	1.609556	1.099649
TBX18	0.681666	0.372426	1.382392	1.839436
HAUS7	0.681666	0.372468	1.190522	0.984385
DZIP1L	0.681666	0.37247	0.584502	3.328619
DENND1A	0.681679	0.372533	0.604766	3.161485
SAP18	0.681747	0.372625	-0.40326	6.341656
SNAI3-AS1	0.682118	0.372883	1.056186	2.136522

SNORD89	0.682286	0.373053	0.353053	7.630045
INPPL1	0.682286	0.373109	0.371408	6.317598
CDKN2AIPN	0.682286	0.373141	-0.65881	3.072707
ZNF831	0.682359	0.373236	0.760713	2.860498
LOC101928	0.682497	0.373367	1.318306	0.622039
POLB	0.682845	0.373612	-0.60736	4.434876
AGPAT3	0.682864	0.373678	-1.30499	1.222866
R3HCC1L	0.683132	0.37388	1.392938	1.813537
ABCB8	0.683315	0.374036	-1.48293	0.560289
MORC2	0.683726	0.374316	-1.25409	1.984364
EFR3A	0.683954	0.374505	-0.36403	7.059534
UBE2Z	0.683954	0.374552	-0.35953	6.5293
ERCC6L2	0.684051	0.374672	-0.34476	5.674257
RAD23B	0.684051	0.374715	-0.41748	5.038197
ZNF385B	0.684132	0.374815	1.404663	0.517374
LOC102723	0.684487	0.375065	0.566468	3.5994
RAB35	0.684539	0.375149	0.797729	2.037422
PHF12	0.684688	0.375286	0.413026	4.428736
TRIM16L	0.684786	0.375409	-0.44439	4.436445
SMARCE1	0.684786	0.375451	-0.345	6.954777
NFIX	0.685079	0.375667	0.727387	2.189103
PLEKHG6	0.685411	0.375904	1.212281	1.648269
RRAGA	0.685704	0.376121	-0.35489	5.382432
ID3	0.685932	0.376301	0.679247	2.760689
NECTIN3	0.685992	0.37639	1.098272	1.88033
CFLAR-AS1	0.686269	0.376683	0.501341	3.949388
FAM92A	0.686269	0.376694	-1.84876	0.625429
MED17	0.686269	0.376728	0.393192	4.832416
AKR7A2	0.686269	0.376764	0.386946	4.929558
NUP160	0.686541	0.377001	-0.35542	6.309266
ANKRD33B	0.686541	0.377065	0.593052	4.37555
MPHOSPH9	0.686541	0.377092	-1.60914	1.113071
C8orf33	0.686541	0.377359	-0.40457	4.895482
CLN5	0.686541	0.37742	-0.51601	4.156935
CHCHD2	0.686541	0.377542	-0.35261	6.553185
RPAP1	0.686541	0.377623	-0.45058	4.95824
SECISBP2	0.686541	0.377644	1.51796	0.990219
KIAA1147	0.686541	0.377645	-0.9736	3.439886
ZNF326	0.686541	0.37766	-0.3589	6.987787
MIR650	0.686541	0.377749	1.10301	1.970873
LDOC1	0.686541	0.377761	-0.78439	3.188099
PCNT	0.686541	0.377768	0.707038	3.459884
KLC1	0.686541	0.377812	-0.45416	4.726813
AGAP5	0.686541	0.377812	-0.79757	3.157039
HNRNPA3	0.686541	0.377838	-0.36082	8.766461

DAAM2	0.686541	0.377859	1.47166	1.067067
UBE2E4P	0.68657	0.37793	0.903175	2.171858
FUT10	0.686623	0.378015	0.394805	4.565575
EHBP1L1	0.686735	0.378133	-0.40154	9.139952
NPIPB5	0.686739	0.378191	0.417265	6.533779
COPG2	0.686853	0.378388	-0.63634	3.798991
ACP1	0.686853	0.378418	1.539929	1.013867
ZNF197	0.686853	0.37842	0.416627	4.989982
PCGF1	0.686874	0.378523	0.578244	3.240642
B4GALT7	0.686874	0.378543	0.548804	3.5983
SOCS2	0.687261	0.378817	1.457863	0.922577
P2RY13	0.687261	0.378911	1.472467	1.114313
SEC14L5	0.687261	0.378966	-1.12872	2.555054
HSP90B2P	0.687261	0.378979	0.633866	3.169694
MTHFR	0.687395	0.379108	0.333562	5.716955
LINC00324	0.68743	0.379271	-0.88956	2.210524
KBTBD6	0.68743	0.379295	0.709929	3.354086
USP48	0.68743	0.379346	0.336625	6.717357
POM121C	0.68743	0.379397	-0.37591	6.311311
TMED10	0.68743	0.379406	-0.34321	8.345843
SNORD114	0.687543	0.37955	1.165994	1.354906
KIRREL1	0.687543	0.37958	-1.72819	0.678289
LOC10192ε	0.687739	0.379776	-1.23671	2.026663
AGPAT5	0.687739	0.379846	-0.39095	5.717959
ZNF585B	0.687739	0.379855	0.408893	4.777176
PIGK	0.688089	0.380104	0.569496	4.39556
ABHD1	0.68816	0.380199	1.271368	1.648267
CBX3	0.68832	0.380394	0.889246	1.636586
PDSS2	0.68832	0.380399	-0.52994	4.957671
LOC441455	0.688411	0.380553	-2.11636	0.473929
FAM90A1	0.688411	0.380561	0.960972	2.298499
SNORD114	0.688574	0.380707	-3.36909	0.696558
BATF3	0.689232	0.381126	1.376684	1.725512
PMS2P2	0.68931	0.381226	-1.39397	0.594911
MLLT10P1	0.689419	0.381341	1.26838	1.283465
MMGT1	0.689442	0.38141	0.390723	5.527906
PEA15	0.689603	0.381595	-1.8352	0.461031
PLEKHA5	0.689603	0.381652	-0.46792	5.622193
MAP7	0.689603	0.381704	1.206818	0.592952
ADAM20P1	0.689603	0.38176	-1.04845	2.704463
LINC01547	0.689603	0.381778	1.532263	1.360522
LOC100419	0.689645	0.381889	-0.43822	5.000187
H2AFX	0.689645	0.381913	-0.48099	4.409185
KRBOX1	0.689726	0.382026	-2.01567	0.722235
CDS1	0.689726	0.38207	-0.37074	6.497138

SH2D3C	0.689775	0.382212	1.031627	2.568549
SGTB	0.689775	0.382233	0.773728	3.402783
ZNF865	0.689775	0.382265	0.4989	4.347238
RAPGEF6	0.689833	0.382353	0.717411	2.895883
EP300	0.689876	0.382445	-0.34934	8.746568
UAP1L1	0.689876	0.382489	0.466656	4.414525
VPS4A	0.689899	0.382696	-0.35264	6.267877
IFRD2	0.689899	0.382698	0.50334	4.668247
CCDC136	0.689899	0.3827	0.897025	2.427581
C11orf1	0.689899	0.382725	0.699344	2.642964
CTSW	0.689917	0.382829	1.064418	1.684417
NCF1B	0.689917	0.382846	-1.27613	1.111112
EZR-AS1	0.689971	0.383009	-0.89183	3.186595
GAS7	0.689971	0.383061	-1.04398	2.180979
ADRA2C	0.689971	0.383086	-2.17276	1.082742
PRRC2C	0.689971	0.3831	-0.35505	10.37066
USP9Y	0.690205	0.383286	-0.78843	7.165423
ARHGEF6	0.690226	0.383353	1.435335	0.479416
IKZF1	0.690236	0.383445	1.34482	0.937371
RNF212B	0.690236	0.383539	-1.28618	2.678134
FBXL15	0.690236	0.38357	0.577286	3.001973
AIM2	0.690236	0.383614	1.558793	0.907782
TDRP	0.690236	0.383638	-1.35522	1.383191
PATE4	0.690701	0.384128	-2.0945	0.767864
TAS2R4	0.690701	0.384131	-1.9589	0.817852
LINGO3	0.690701	0.384146	1.408796	1.542848
AGO4	0.690701	0.38416	-0.35072	6.462559
SOX6	0.690701	0.384314	-2.27267	0.757228
FAH	0.690701	0.384347	0.630348	3.366951
DNAJC17	0.690701	0.38436	-0.55926	3.932953
ZBTB7A	0.690701	0.384462	-1.27092	1.198151
KIF2A	0.690701	0.384506	-1.32457	1.863211
CXCL12	0.690701	0.384522	0.850109	2.678506
CEP89	0.690701	0.38456	0.400317	4.941121
SLC35E1	0.690701	0.384568	-0.34969	6.584382
ZDHHC11	0.690831	0.384837	0.603115	3.512385
PDZRN3	0.690831	0.384839	-0.70638	4.147855
IFI16	0.690831	0.384863	1.078915	1.483089
LINC00970	0.690831	0.384864	1.49521	0.698688
LCN10	0.690869	0.384946	1.415075	2.566671
AGBL1	0.690869	0.385088	1.733187	1.579134
TMEM161I	0.690869	0.385178	0.808749	2.447006
HGS	0.690869	0.385207	-0.34667	6.972257
FADS6	0.690869	0.385241	-1.56638	1.576105
CORO2A	0.690869	0.385258	-1.6956	0.871658

RPL7	0.690869	0.385277	-0.38164	8.113186
PRICKLE4	0.690873	0.385381	-0.49881	4.504027
TUSC2	0.690873	0.385391	0.606259	3.598062
KMT5C	0.690903	0.385464	0.595779	3.315068
LINC00174	0.690975	0.38556	-0.42573	4.836555
ARSI	0.691013	0.385638	-1.07966	2.191823
MGLL	0.69116	0.385816	-1.47166	1.051457
B3GALT6	0.69116	0.385832	0.506123	3.515615
NAAA	0.691416	0.386031	-1.14004	2.867964
DNAJC30	0.69149	0.386127	-0.72648	3.00436
FOXP4-AS1	0.691571	0.386237	1.135402	1.71404
PPARGC1B	0.691571	0.386351	1.259762	1.306539
RNF207	0.691571	0.386391	0.401806	5.237623
ZBED6	0.691571	0.386397	-0.39604	7.192616
LOC10050€	0.692136	0.386834	0.449554	5.213785
ARHGAP21	0.692136	0.386857	0.317439	7.60578
CHD5	0.692136	0.386888	-1.87898	1.265183
POC1B	0.692136	0.386937	1.581989	0.570312
TMPO	0.692239	0.387051	0.42478	4.966997
RBM17	0.692273	0.387126	0.59361	3.50413
CCNL2	0.692362	0.387231	-0.35063	7.196671
LOC100128	0.692384	0.387331	-0.38475	5.007423
LOC646214	0.692384	0.387356	0.702499	3.796182
CXorf40B	0.69292	0.387712	0.496106	3.754024
SNX30	0.693099	0.387896	-0.37283	6.171126
LOC10192€	0.693099	0.387948	-1.1986	1.663002
PAXX	0.693099	0.38798	0.578247	3.349256
ATP5PB	0.693159	0.38807	-0.35675	6.927443
NAT9	0.693259	0.388182	-1.96107	0.811254
INHBA	0.693672	0.38847	-1.46271	3.66366
TMEM198I	0.693757	0.388573	1.341392	0.745213
REM2	0.693833	0.388672	1.203895	1.492884
NSD3	0.693925	0.388787	-0.35563	6.229842
ERVW-1	0.693925	0.388836	-1.6445	0.574707
LOC10537(	0.694171	0.389076	-0.89137	2.487515
DDHD2	0.694171	0.38927	1.418441	0.773699
PDIA3	0.694171	0.389335	0.319592	8.082029
TNFSF8	0.694171	0.389337	0.996383	2.666907
CSKMT	0.694171	0.389366	0.726491	2.134591
AP1G1	0.694171	0.38937	0.848473	1.785047
TSR2	0.694171	0.389375	-0.37345	5.755459
EBPL	0.694171	0.389424	-1.61035	1.228196
C11orf49	0.694171	0.38951	-1.34537	0.914792
ATP5PD	0.694171	0.389536	-1.70568	0.55213
HEBP1	0.694206	0.389612	-0.45869	4.660429

NDUFAF3	0.694493	0.389829	-1.4754	1.274445
ZCCHC3	0.694521	0.389902	0.534957	4.281598
NIPAL3	0.695336	0.390416	-0.35897	6.195365
FNTA	0.69548	0.390553	-1.43451	1.557026
KANK1	0.695544	0.390645	-0.58003	3.510744
TIMM13	0.695564	0.390713	-0.33592	5.857668
ARID3A	0.696119	0.39108	-0.46084	4.554966
HLCS	0.696121	0.391245	1.006088	1.804106
CUL1	0.696121	0.391277	-0.34669	6.426387
PLBD1-AS1	0.696121	0.391289	1.135878	1.698229
TKFC	0.696121	0.391307	0.370377	4.950796
NR2F2	0.696222	0.391427	0.728022	4.461239
MAGOHB	0.696222	0.391477	0.557206	3.096758
DEGS1	0.696343	0.391709	0.419404	4.601108
PIPOX	0.696343	0.391741	-1.45531	1.105578
DNER	0.696343	0.391828	-1.78581	2.356353
LOC101927	0.696343	0.391845	0.887534	2.701781
BMPR1A	0.696343	0.391926	-0.38521	5.09013
CDK8	0.696343	0.391942	-0.41492	4.931521
SON	0.696343	0.391944	-0.44297	4.72325
CD27-AS1	0.696343	0.391996	0.611824	2.809789
LOC101927	0.696361	0.392104	1.125767	1.500403
RNU4-2	0.696361	0.392134	0.415113	10.34046
KCNAB3	0.696361	0.392219	1.146438	1.539799
IQCD	0.696361	0.392296	0.914816	1.907923
TCF7L2	0.696361	0.392367	0.964256	1.45214
SEC14L1P1	0.696361	0.392375	0.730807	2.338571
ZCCHC14	0.696361	0.392401	-0.33577	6.499936
MYO18B	0.696436	0.3925	1.324553	1.914823
CSRP1	0.696566	0.392667	1.472071	0.716325
KPNA1	0.696566	0.392686	0.723852	2.702477
VAPA	0.696573	0.392746	-1.49385	1.951197
CROCCP3	0.697118	0.393137	0.622131	4.225794
DNAJC21	0.697118	0.393167	0.81256	2.102337
LOC645513	0.697235	0.393289	-0.62863	3.539454
BHLHA15	0.697279	0.393426	-1.252	2.293299
GTF2E1	0.697279	0.393427	0.505219	4.198522
GFPT2	0.697286	0.393487	-0.96658	2.279198
RIN2	0.697387	0.393601	-1.73322	0.567233
LCE3D	0.697397	0.393663	-1.86027	1.845604
SHANK2-AS	0.697447	0.393748	0.677672	2.428165
LOC100130	0.697455	0.393808	0.830557	2.246258
WIZ	0.69748	0.393901	-0.36438	5.197748
CRSP8P	0.69748	0.393935	1.113585	1.4594
EPB41L4A-.	0.697597	0.394058	-0.50415	4.095321

ACTRT3	0.697626	0.394131	1.265624	1.001816
PEAR1	0.697644	0.394234	0.586313	3.328848
TSNARE1	0.697644	0.394254	0.518795	3.651141
CASP2	0.697746	0.394374	-1.75566	0.937534
DDB1	0.697746	0.394452	-0.33273	7.703381
PRKRA-AS1	0.697746	0.394481	1.02797	2.644262
UTP20	0.698049	0.394715	-0.3564	6.425241
MED25	0.698049	0.394858	-0.41668	5.199683
ADAMTS15	0.698049	0.394866	0.737822	3.341058
PRPF19	0.698049	0.394894	-0.34384	6.099362
SMNDC1	0.698049	0.394936	-0.41539	5.284084
MAP3K21	0.698199	0.395079	0.40785	4.634974
PPP1R12B	0.698199	0.395134	0.851363	2.816607
CDRT4	0.698413	0.395311	0.63582	2.916688
SCARNA27	0.698456	0.395406	0.775133	2.196675
PATZ1	0.698456	0.395448	0.994031	2.214685
FANCA	0.698599	0.395586	-1.67718	0.618413
HMGN2	0.698798	0.395755	-0.33601	6.380458
EBLN2	0.698806	0.395816	0.616069	3.710834
TP53RK	0.698822	0.395882	-0.5951	4.108052
FAM228A	0.69897	0.396103	1.746375	0.451413
LOC100506	0.69897	0.396104	1.023616	1.576296
UAP1	0.69897	0.396151	-0.32825	6.332192
PER1	0.69897	0.396193	-0.4455	7.344014
ZNF428	0.699464	0.396576	0.771853	2.320293
SLC37A4	0.699464	0.396601	1.130029	0.921423
APBA3	0.699464	0.396643	0.479937	3.766014
PEX13	0.699542	0.396879	-0.34521	5.933736
NGRN	0.699542	0.396919	-1.28768	1.341995
ABCC5-AS1	0.699542	0.396985	-1.1229	2.248868
RNF31	0.699542	0.397007	0.6786	2.688847
SLC11A1	0.699542	0.397147	0.867878	3.441171
CCDC157	0.699542	0.397169	-1.46604	1.514347
PLS3	0.699542	0.397177	-1.38402	1.950299
VSIR	0.699542	0.397191	0.327355	5.707455
KAT6A	0.699542	0.397219	-0.33909	7.410867
FABP4	0.699542	0.397271	1.373264	0.666339
TBCA	0.699542	0.39731	0.860329	3.124742
EFCAB14	0.700334	0.397816	-0.3391	8.294788
GCHFR	0.700343	0.397977	-0.92895	2.964494
DCLRE1B	0.700343	0.39798	0.510136	3.588917
SEC62	0.700343	0.397992	0.3858	7.847983
TSPAN7	0.700629	0.398211	0.614556	3.336563
SCARNA16	0.700852	0.398395	0.509113	4.263111
ANK2	0.700856	0.398457	0.758443	3.446121

MMS22L	0.700856	0.398511	-0.4982	4.393412
MBD6	0.701072	0.39869	0.318102	6.674456
PCDHB16	0.701234	0.398858	0.797157	2.7528
TMED6	0.701234	0.399019	-1.45063	2.89086
RNF113B	0.701234	0.399048	1.240875	1.242777
PAK2	0.701234	0.399059	-0.33769	7.672772
IDO1	0.701234	0.39909	-1.89912	1.187112
PLEKHG2	0.701234	0.399123	-0.48161	5.769565
CTCF	0.701315	0.399255	0.352556	4.932284
TTC39C-AS	0.701315	0.399282	1.561032	0.838252
SPIN1	0.701555	0.399511	-0.36529	6.346479
HLX	0.701555	0.399533	1.031846	1.426848
SNORD88A	0.701623	0.399692	-1.29026	1.361665
GPN2	0.701623	0.399753	0.493515	3.793472
LINC00278	0.701623	0.39979	-0.78978	4.057086
USF3	0.701623	0.399799	-0.34466	6.917357
DPYS	0.701961	0.400049	-2.60812	0.848897
ARGFXP2	0.702158	0.400263	-0.95327	2.28663
MALT1	0.702158	0.400274	-1.68421	0.956571
TMEM255I	0.702535	0.400546	1.111948	1.421484
HUS1	0.70268	0.400686	-1.41907	1.527935
SRP19	0.7036	0.401307	0.480447	4.168685
AMDHD1	0.7036	0.401325	1.939581	0.666128
LINC01569	0.703732	0.401457	1.057431	1.132972
CYCS	0.703776	0.401539	-0.35132	7.669197
C2orf68	0.70391	0.401672	0.338514	5.584155
PIPSL	0.703994	0.401793	0.911045	2.332033
MIR103A2	0.703994	0.401834	-3.22839	0.853815
HS6ST2-AS	0.704109	0.401957	-1.73807	0.54716
ADAM15	0.704121	0.402041	1.423249	1.057004
LOC10012[illegible]	0.704121	0.402078	-0.54476	4.259848
PHACTR2-[illegible]	0.704164	0.40216	0.907887	2.867551
RASL11B	0.704191	0.402263	1.43629	1.182708
CCDC93	0.704191	0.402289	0.312405	6.853977
CCNE2	0.70449	0.402517	0.495772	4.219267
NDUFV3	0.704693	0.40269	0.414992	4.488341
SPICE1	0.705249	0.40315	0.325369	5.962524
MYCL	0.705249	0.40322	-1.61126	1.730415
TCEAL2	0.705249	0.403241	-3.22471	0.585261
SLC25A4	0.705249	0.403252	-0.58534	4.698823
UGP2	0.705249	0.403293	-0.39777	5.144038
PIH1D1	0.705386	0.403565	-0.38277	4.868141
TMEM45A	0.705386	0.403575	-0.77421	3.928927
ANKRA2	0.705386	0.403692	0.350988	5.37555
CDKN2A	0.705386	0.403703	0.579029	3.737497

PCDH7	0.705386	0.403711	-1.60255	0.601556
FIS1	0.705386	0.403732	0.363751	5.844321
PSMD4	0.705386	0.40378	-0.33866	6.541678
FBXO22	0.705386	0.403829	-1.72623	1.63243
MIR145	0.705413	0.403953	1.201429	1.234422
FAM193A	0.705413	0.403958	-1.06774	2.125075
KCNB1	0.705716	0.404189	1.447478	1.575563
ANKRD46	0.705747	0.404375	-1.40283	0.931968
PPIH	0.705747	0.404394	-0.53258	3.62841
SNORD59B	0.705747	0.404461	0.629624	2.723951
STX17	0.705747	0.404539	-0.33914	6.917749
TRUB1	0.705747	0.404554	-0.54103	4.375763
ACTN4	0.705747	0.40456	-0.35036	9.711745
LOC64465(	0.705747	0.404607	0.965232	1.555678
LAMA5	0.706093	0.404862	-0.35472	7.630981
GOLPH3	0.706242	0.405005	0.30372	6.814963
PANX1	0.706872	0.405424	0.424801	4.565235
FBXW9	0.707124	0.405625	-0.84119	2.614332
ACKR3	0.707173	0.405739	-0.8521	4.147792
ABCA1	0.707173	0.405837	0.387294	6.593306
SERAC1	0.707173	0.405865	-0.56252	3.740015
MRPS6	0.707173	0.405923	-0.3729	5.010019
MUC4	0.707173	0.405944	-0.7313	9.085214
MIR324	0.707173	0.405998	-1.32091	0.866328
ZC3H12C	0.707276	0.406113	-0.74944	4.192484
UBAP2L	0.707382	0.406241	-0.63114	3.916448
LOX	0.707382	0.406289	1.027678	1.551961
DARS2	0.707401	0.406357	0.304706	5.419422
MEX3A	0.707401	0.406539	-0.58272	4.033364
RNF139	0.707401	0.406571	-0.39033	4.917739
MIR4728	0.707401	0.406627	-1.90699	0.644325
MFSD9	0.707401	0.406689	-0.46681	4.408889
KDM1B	0.707401	0.406727	-0.35556	5.621759
CEBPB-AS1	0.707401	0.40673	1.087945	1.421304
ERG	0.707401	0.406758	-1.47601	1.057288
PIK3CB	0.707784	0.407062	-0.39416	5.28961
MPHOSPH(	0.707784	0.407141	-0.50097	4.771908
VWA8	0.707784	0.40715	0.414102	5.026787
HELZ2	0.708036	0.407353	0.457169	3.778952
UQCC3	0.708585	0.407731	-0.62253	3.71007
WWP2	0.708585	0.407876	0.673791	3.273006
MTMR9	0.708585	0.407885	-0.36252	5.195413
EPN1	0.708585	0.407904	-0.832	2.976323
MINAR1	0.708585	0.407956	0.950125	2.266386
MZT2B	0.70887	0.408177	-0.72888	3.162201

TTC14	0.708976	0.408295	0.47248	4.423435
PET117	0.709639	0.408753	-1.53077	0.852731
STARD13-A	0.709639	0.408793	1.546989	0.873748
PIK3C2A	0.7099	0.409001	-0.32759	7.604447
SEC23A	0.710054	0.409167	0.314075	6.114588
GYG2P1	0.710054	0.409283	1.109962	1.18411
HIRA	0.710054	0.409299	0.310454	6.04161
H2AFY	0.710054	0.409375	1.362333	0.747213
DNM1	0.710054	0.409414	-1.28108	1.324263
UBA5	0.710054	0.409619	-0.51904	4.107911
ATL3	0.710054	0.409639	1.069062	1.12473
FAM87B	0.710054	0.40967	1.279927	1.496842
CCDC152	0.710054	0.409707	0.963127	1.43337
RTL8A	0.710054	0.409708	-0.62078	3.194952
GAS1	0.710054	0.409722	-1.40358	1.531777
ARPC2	0.710464	0.410138	1.095297	0.845026
PAN3-AS1	0.710464	0.410149	0.559777	3.225118
RAPGEF2	0.710464	0.410178	0.327097	6.73877
EDEM1	0.710464	0.410189	-0.34347	7.222333
MTFMT	0.710678	0.410404	-0.53581	3.541989
ASPM	0.710678	0.410427	-0.44278	4.952015
CRTAC1	0.711028	0.410691	-1.7834	1.217447
WNT6	0.711028	0.410744	-1.59502	1.514135
NCOA4	0.711815	0.411308	1.181893	1.728208
S100A1	0.711815	0.411314	-1.0013	2.462535
ICE1	0.711983	0.411469	-0.33881	6.352472
BRMS1L	0.712001	0.411537	-0.43913	4.371018
FAM133B	0.7122	0.41171	-1.91285	0.792338
JMY	0.712203	0.411769	-0.337	6.377234
PTK6	0.712234	0.411863	-1.3423	1.394396
COX5B	0.712234	0.411959	-0.36545	7.248587
SNORD116	0.712234	0.412004	-0.55039	5.098108
SPIN4	0.712234	0.412018	-0.51602	4.567853
CERK	0.712297	0.412141	0.313283	6.328858
LINC02099	0.712297	0.41217	-1.89455	1.065553
RUVBL1	0.71292	0.412623	-0.33504	5.248887
LOC100129	0.71292	0.412646	0.59375	2.970299
SHROOM4	0.71328	0.412912	0.948976	2.6782
SKA2	0.71343	0.413056	1.401397	0.606121
ZNF776	0.713598	0.413217	0.3277	5.37543
LSP1P4	0.713598	0.413285	-1.04968	1.900991
NMD3	0.713598	0.413327	-0.34469	6.212964
SNORA14A	0.713634	0.413414	0.527367	2.963709
INPP5E	0.713634	0.413493	-0.56796	3.600187
NHSL1	0.713634	0.413608	-0.47907	4.669244

LOC10050€	0.713634	0.413642	-0.74358	3.164349
PFKL	0.713634	0.41375	0.890248	1.901508
ZNF671	0.713634	0.413807	0.756376	3.216448
RIF1	0.713634	0.413895	1.184594	1.194392
FAM117B	0.713634	0.413908	0.313199	5.504456
CCSAP	0.713634	0.413975	-0.46028	4.636236
GPATCH1	0.713634	0.414035	0.378329	4.6482
ZCCHC2	0.713634	0.414063	0.412908	4.210825
COL18A1	0.713634	0.41414	0.923301	2.049721
TGFA-IT1	0.713634	0.414155	-1.65143	0.514133
HMOX1	0.713634	0.414189	-0.53752	4.515431
MYPOP	0.713634	0.414215	0.567834	3.20657
HIST1H2AI	0.713692	0.414307	-0.59172	3.642625
OCLN	0.713871	0.414553	-1.20237	1.489708
HSPBAP1	0.713871	0.414554	0.472299	3.458036
PRKY	0.713871	0.414584	0.506742	5.99676
HSBP1L1	0.713917	0.414669	0.485346	3.88385
RTN4	0.713918	0.414774	-0.51329	4.300334
LOC541473	0.713918	0.414785	1.644899	0.627905
LOC100129	0.714103	0.414975	1.104901	1.250407
GKAP1	0.714103	0.415033	1.623509	0.852571
SLC2A12	0.714103	0.415066	-0.76297	4.433086
ZADH2	0.71417	0.415195	0.607448	2.460673
ZSCAN5A	0.71417	0.415244	1.072043	0.90467
MMD	0.71417	0.415279	0.651309	2.917379
CCAT2	0.714244	0.41542	1.231884	0.962131
WDR55	0.714244	0.415437	0.361421	5.543423
TPGS1	0.714548	0.415761	0.600547	2.384036
MED27	0.714548	0.415831	-1.57021	0.968456
LCMT2	0.714548	0.415845	-0.76581	2.93039
LRRCC1	0.714548	0.415906	0.363985	4.700176
STARD5	0.714548	0.415953	0.508737	3.749354
ECH1	0.714548	0.415961	0.343128	6.726886
OVOL3	0.714698	0.416107	-1.69589	0.498785
COQ10B	0.714851	0.416258	0.424808	4.913083
SLC4A7	0.714851	0.416312	0.589953	3.125104
TESK2	0.714875	0.416467	-0.47354	4.287814
CDCA4	0.714875	0.41647	-1.20491	0.768267
LINC01006	0.714875	0.416517	0.703892	2.902306
PPM1M	0.714875	0.416592	1.245036	0.549238
SNORD16	0.714875	0.416615	-0.42248	5.271203
MRPL23-AS	0.715137	0.416825	1.322068	0.648983
NME5	0.715168	0.416935	1.489027	0.741695
VWA1	0.715168	0.416959	-1.36071	1.483278
C10orf90	0.715244	0.417061	-2.2101	0.666573

HIST1H2BI	0.715324	0.417166	0.45566	4.31442
TRIP13	0.716051	0.417648	0.817651	2.12939
RETSAT	0.716111	0.417741	0.331234	6.614166
FSTL4	0.716174	0.417836	-1.13883	2.29327
6-Mar	0.716213	0.417917	-1.69925	0.35044
PCAT2	0.716606	0.418204	-1.8298	0.491112
ZNF789	0.716823	0.418438	0.467376	3.515902
SNORD97	0.716823	0.418446	0.358224	9.421041
APCDD1	0.716894	0.418546	-0.41095	5.561937
IL6ST	0.71708	0.418713	1.338509	0.791534
SEC16A	0.717129	0.418874	0.763489	2.439113
RPL12	0.717129	0.418911	-0.33305	8.716517
LOC10666C	0.717129	0.418973	1.131645	0.930576
MBD5	0.717129	0.418974	0.365486	5.188919
SESN3	0.71715	0.419044	0.792794	2.155443
SDCBP2-AS	0.717386	0.419258	-0.95633	2.327468
ADAMTS16	0.717386	0.419298	1.457616	0.671255
IER3IP1	0.717408	0.419405	0.295454	5.724597
NUFIP2	0.717408	0.419472	-0.33499	8.327061
LINC02159	0.717408	0.419486	-1.5918	0.70936
LOC105375	0.717672	0.419698	1.126867	1.640585
CHSY3	0.717963	0.419926	1.173683	1.838361
CAVIN1	0.717974	0.420148	0.369396	7.542434
IDE	0.717974	0.420149	-0.39211	5.563811
CARS2	0.717974	0.420159	-0.35751	5.129504
CHST10	0.717974	0.420166	1.107992	1.760018
TPP2	0.717975	0.420224	-0.32154	6.638545
LOC100506	0.718247	0.420451	-0.87332	2.143307
PCIF1	0.718247	0.4205	-0.38316	4.93773
ZC3H8	0.718477	0.420741	-0.38535	5.175033
ERRFI1	0.718477	0.420799	-0.4782	7.097222
RUBCN	0.718477	0.420809	0.601968	2.620564
DBIL5P	0.718516	0.42089	-1.66238	0.733622
FAM13A-A	0.718628	0.421085	0.503602	3.531883
CFAP57	0.718628	0.421099	1.229132	0.784491
BBS7	0.718628	0.42113	0.667997	2.163471
PP2D1	0.718787	0.421281	-1.22927	2.11053
ATP1B1	0.718873	0.421442	0.328602	9.401865
SAMM50	0.718873	0.421448	-0.35821	5.160826
ADD1	0.719119	0.421651	-0.63413	2.595948
FAHD2B	0.719224	0.421834	-1.02727	2.490282
FOXO1	0.719224	0.421922	0.380257	5.987134
SMIM19	0.719224	0.421926	-0.95835	1.564169
DICER1-AS	0.719224	0.421946	-1.21592	1.663296
NDUFAF6	0.720299	0.422635	0.558213	3.59089

KNDC1	0.720487	0.422812	1.420834	1.25358
FCHO2	0.720487	0.422862	-1.69776	0.623816
UBL4A	0.720693	0.423041	-0.51003	3.940093
RDH10	0.721305	0.423481	0.363308	6.114899
EXOC6	0.721305	0.423517	1.403881	0.853157
FAM216A	0.721343	0.423598	-0.9993	2.370363
PTCD3	0.721416	0.423739	0.301044	6.592023
HTR7P1	0.721416	0.423758	-0.89619	2.050808
ENKD1	0.721497	0.423909	0.574058	3.113361
ARID1A	0.721497	0.423922	-0.44837	4.085404
VMP1	0.721886	0.424279	-0.39354	8.127455
C16orf71	0.721886	0.424286	1.300202	1.44401
KRT79	0.721886	0.424366	-1.28556	1.764617
LOC101927	0.721886	0.424499	-1.48023	0.618169
SLC19A2	0.721886	0.424538	-0.41806	4.740648
ELMO3	0.721886	0.424614	0.406843	5.283574
DBF4	0.721886	0.42473	-0.51907	4.043299
SNAPC3	0.721886	0.424759	-0.34961	6.05185
SNX32	0.721886	0.424772	0.882906	1.422162
SNX29P2	0.721886	0.424777	0.880809	1.717694
LOC100128	0.721886	0.424793	-1.27594	1.767042
AZGP1	0.721913	0.424868	-1.77393	5.225744
FYTTD1	0.721976	0.424963	0.520964	3.141424
ZNF552	0.722278	0.425255	-0.44376	4.986507
CLASP1	0.722278	0.425313	0.70052	2.189829
IFIT5	0.722278	0.425317	-0.71069	3.866958
LARP7	0.722323	0.425431	0.979423	1.305357
ACTR1B	0.722323	0.425486	0.319287	5.542371
SCARNA9	0.722323	0.425519	0.321104	6.228941
XPO1	0.722615	0.42575	-0.32862	7.786311
SCIN	0.722723	0.425872	0.749368	2.560759
ANKRD28	0.723042	0.426118	0.580214	2.964937
LOC100507	0.72315	0.426277	1.523865	0.622746
RGPD4	0.72315	0.426299	-1.1412	1.920804
UQCRQ	0.723298	0.426445	-0.35858	6.257839
MAK	0.723307	0.426508	1.16273	1.734738
LOC101928	0.723352	0.426594	0.842093	1.925507
PRDM4	0.723955	0.427008	-0.32108	6.195417
PPP3CB-AS	0.724103	0.427157	0.825958	2.634164
INPP5F	0.724103	0.427252	-0.49826	3.99736
MIEF1	0.724103	0.427316	-1.86116	0.873377
FLJ32255	0.724103	0.42733	0.610156	3.176137
SCD5	0.72506	0.427954	0.74472	3.446979
CYP2B7P	0.725181	0.428084	1.498443	1.123742
TIMM50	0.725422	0.4283	0.359657	4.650123

MAK16	0.725422	0.428343	-0.42998	4.701285
ZMYM2	0.725564	0.428486	-1.48797	0.539296
DNAJB9	0.726293	0.428975	0.364378	5.107545
MYOM3	0.726448	0.429126	0.979662	1.302196
ATF5	0.726838	0.429479	1.008848	1.697024
TANGO6	0.726838	0.429511	-0.39266	4.70501
MYBPC3	0.726838	0.429533	1.017721	2.007112
IL2RB	0.727292	0.429885	0.671436	3.446242
SNRPA	0.727292	0.429919	0.337317	5.265342
LOC101928	0.727445	0.430068	0.733072	1.59638
MAGED2	0.727825	0.430352	1.054018	0.88018
NAA80	0.72789	0.430449	1.408235	0.588109
HAO1	0.72798	0.430561	1.809325	0.695522
WBP1	0.727992	0.430627	0.56651	2.958859
PITPNM2-A	0.72842	0.43094	-0.73739	2.952314
HOMEZ	0.728462	0.431024	-0.41138	4.827559
GAB2	0.728525	0.43112	-1.02448	1.866152
CFAP97	0.728788	0.431334	-0.4556	4.788844
GOT1	0.729493	0.431873	-0.35129	5.386418
C14orf132	0.729493	0.431882	-2.41309	1.380717
LAMA1	0.729493	0.432011	-1.07094	2.755062
C16orf58	0.729493	0.432062	0.325526	5.746121
SEMA5A	0.729493	0.432235	0.363648	6.148314
CDC5L	0.729493	0.432252	-0.3046	6.653025
DHRS11	0.729493	0.432252	-1.54679	1.030578
SORT1	0.729493	0.432255	-0.63157	2.975923
EPHA1-AS1	0.729493	0.432341	-0.6031	4.33868
NUP54	0.729493	0.432381	1.129142	0.859351
ITGA10	0.729493	0.432402	1.093455	1.297558
EML6	0.730112	0.432828	-0.37614	4.872047
L3HYPDH	0.730396	0.433132	0.51325	3.215153
CDK19	0.730396	0.433136	-1.40546	1.287084
GPR37	0.730396	0.433216	-1.21038	2.311103
OTUD4	0.730396	0.433233	-0.32484	6.81065
AXL	0.730466	0.433333	-1.24331	1.780165
A2MP1	0.730524	0.433427	1.266771	0.793688
DUOX2	0.730672	0.433585	-0.48173	7.869469
ANAPC15	0.730672	0.433643	1.987531	0.502972
LAG3	0.730672	0.433693	1.296202	0.532193
DGKE	0.73078	0.433922	-0.52245	4.671657
NKPD1	0.73078	0.433948	-1.13772	1.6915
GNG12-AS1	0.73078	0.434033	0.666398	3.146745
ZNF213-AS	0.73078	0.434045	0.725427	2.232782
MLLT10	0.73078	0.434084	-0.90799	1.988182
AHSA1	0.73078	0.434112	0.291304	6.4987

FBXW4	0.730949	0.434271	-0.34329	6.185317
DDX27	0.731232	0.434498	0.294994	6.992282
EXOC3	0.731257	0.434613	-0.32366	7.096125
ZNF488	0.731257	0.434688	0.682634	2.163177
PRR36	0.731257	0.434691	1.134712	1.017238
SLC10A7	0.73128	0.434764	1.14643	1.373054
CFAP43	0.731523	0.434968	1.168596	0.943708
MTOR-AS1	0.731658	0.43519	-1.59393	0.663097
MGMT	0.731658	0.435203	-0.6644	3.199372
PSTPIP2	0.731658	0.435226	-0.69206	3.935788
LSG1	0.732366	0.435758	-0.35793	5.582541
HCG17	0.732366	0.435765	-0.97448	1.522479
FANK1	0.732676	0.436009	-0.60273	3.590554
ARCN1	0.73279	0.436211	-0.87623	2.201909
PTAR1	0.73279	0.436216	0.305922	7.301243
MRPL20	0.73279	0.436255	-0.34062	5.077062
FAM201A	0.73281	0.436326	-1.3706	0.923763
ZNF554	0.732884	0.43643	-0.47695	3.774257
TSPAN32	0.733157	0.436652	1.105501	1.322579
PRMT2	0.733179	0.436725	0.3463	5.757568
GPALPP1	0.733596	0.437081	-0.75242	2.932468
CDHR2	0.733596	0.437092	-1.22069	1.188108
PGM5P2	0.734315	0.437633	0.709253	2.927052
MST1P2	0.734315	0.437639	-0.57511	2.94147
LINC01909	0.734321	0.437702	1.326737	0.714509
ZNF626	0.734886	0.438098	-1.01763	2.588007
XPNPEP2	0.734927	0.43819	1.238631	1.154609
ZNF513	0.734927	0.438242	1.088295	1.40252
CEACAM16	0.735288	0.438562	1.279748	0.81036
CDC26	0.735288	0.438576	-0.49822	3.607636
TONSL	0.735429	0.43872	-0.47802	4.261641
KIAA0895L	0.735429	0.43878	0.314024	5.127311
LOC101593	0.735489	0.439016	1.078965	0.887687
FRAS1	0.735489	0.439049	0.443647	5.383619
SNORD61	0.735489	0.439073	-0.78543	2.761639
LOC100505	0.735489	0.43908	0.637212	2.982058
SH3D21	0.735489	0.439158	0.730955	2.276029
EPRS	0.735489	0.439173	-0.31725	7.9554
SIM2	0.735692	0.439354	-0.39515	4.603375
GPM6B	0.735887	0.43961	-0.68942	3.434225
SF3A2	0.735887	0.439646	0.320224	5.131375
HMG20A	0.735887	0.439648	1.639248	0.527935
SEMA6B	0.73597	0.439758	0.701605	2.450192
TSC22D4	0.735981	0.439824	-1.40471	0.519342
MIR6870	0.736028	0.439912	1.3903	0.487892

HCG27	0.736255	0.440107	1.570114	0.677358
RRH	0.73634	0.440236	1.223009	1.020644
NAP1L2	0.73634	0.440277	0.943643	1.988201
NUP50-DT	0.736827	0.44067	-1.063	1.796046
MIR5695	0.736827	0.440688	1.15486	0.894811
TRNP1	0.73687	0.440829	0.320205	6.52963
REC114	0.73687	0.440833	-1.58655	1.1077
TRUB2	0.736909	0.440916	0.434375	4.467651
KDM5B	0.736959	0.441027	-2.01814	0.457666
GSK3A	0.736959	0.441065	-0.33695	5.309144
ZNF496	0.736967	0.44114	0.487577	3.753317
SPRYD3	0.736967	0.441189	-0.35327	6.246863
MAMDC2-/	0.737133	0.441349	1.052093	1.921838
KIF7	0.737218	0.44148	0.588072	3.34637
HIST1H2BK	0.737218	0.44155	-0.82866	3.221377
FGF7P3	0.737218	0.441578	-1.15696	2.014144
MED7	0.73726	0.441793	1.284824	0.778192
FGF7	0.73726	0.441823	0.805016	4.58062
WDR45	0.73726	0.441836	0.772004	1.884499
RPA4	0.73726	0.441862	0.776417	2.20252
PFKFB4	0.73726	0.441902	-0.96281	2.295876
OVGP1	0.737415	0.442074	-1.00034	1.708156
RNF113A	0.737415	0.442114	0.404402	4.136481
HIST1H3F	0.737642	0.442349	-0.54323	3.532043
P3H2	0.737642	0.442406	-1.1732	1.789972
AGFG1	0.737642	0.442517	1.190848	1.283624
PURA	0.737642	0.44253	-0.32227	6.291101
ZNF264	0.737642	0.442549	-0.31324	6.906243
CYP51A1	0.73784	0.442729	0.54885	3.887719
DBN1	0.73784	0.442788	0.79211	1.261008
RBBP9	0.737845	0.44285	-0.39279	4.639477
HERC2P9	0.738074	0.443214	0.37736	4.341702
ZNF726	0.738074	0.443223	1.032872	1.995297
9-Sep	0.738074	0.443273	-0.3581	5.474044
CDC6	0.738074	0.443281	0.473364	3.8054
RFT1	0.738074	0.443287	-0.38656	4.64124
LOC407835	0.73898	0.443891	-0.93794	1.86223
OSBPL3	0.739068	0.444004	-1.07131	1.644269
LRRC55	0.739365	0.444242	1.619514	0.859055
BFAR	0.739428	0.444339	0.632176	2.869126
RPL36A	0.739458	0.444418	-0.70102	3.375653
FASTKD5	0.739825	0.444698	-0.44311	4.568279
CYP2C18	0.740709	0.445289	1.144001	1.274317
VRK2	0.741089	0.445578	-1.18915	2.026232
LOC101927	0.741509	0.445938	-1.00922	2.391098

NELFE	0.741509	0.445983	-1.24975	1.538326
DIO2-AS1	0.741509	0.44601	-1.39582	1.43527
C11orf91	0.741853	0.446278	-1.27571	2.264889
LOC202181	0.742007	0.44643	-0.69304	4.289467
FBXL3	0.742016	0.446582	-0.29714	6.463946
CDK2AP2	0.742016	0.446606	0.929713	1.587763
ERN1	0.742016	0.446659	0.342368	5.863779
NELFB	0.742016	0.446676	0.297607	5.601994
DRG1	0.74224	0.446871	-0.36867	4.937259
SUZ12P1	0.742355	0.447044	-0.44376	4.07583
THEM6	0.742355	0.44706	-0.5527	4.04744
TUBBP5	0.742436	0.44717	-0.9259	2.492734
TMEM236	0.742911	0.447651	1.212362	0.79578
GATA2	0.742911	0.447663	1.18278	0.537334
FOS	0.742911	0.447675	-0.78364	8.598539
NPAT	0.742911	0.447766	0.345957	5.286606
BOD1	0.742911	0.44781	0.85917	1.982811
TTLL7	0.742911	0.447817	-0.76351	3.828772
NUTM2A-A	0.74318	0.448039	-0.73325	2.983328
SPATA33	0.743514	0.448342	0.956361	1.256601
SAYSD1	0.743514	0.448381	0.490838	3.309153
SHLD2P3	0.743514	0.448421	-0.65501	2.66631
KIAA1211L	0.743759	0.448629	-0.34421	5.563212
MAU2	0.743995	0.448845	-0.31981	6.234581
PRORSD1P	0.743995	0.448892	-1.43307	1.364763
NBPF15	0.744164	0.449054	1.171554	1.15174
PLGRKT	0.744222	0.44915	-0.49207	3.8062
KLHL41	0.74424	0.449239	1.03943	1.503981
LINCR-0001	0.74424	0.449281	0.975553	1.298199
FOXK2	0.744506	0.449501	-0.29632	6.184598
EIF2B2	0.744749	0.449731	-0.444	4.495905
MBOAT1	0.744749	0.449769	1.185482	0.739088
C2CD2	0.744805	0.449894	0.599277	2.853241
INAVA	0.744805	0.449923	0.768462	1.794766
ALS2	0.745084	0.450152	0.32675	5.153525
IVD	0.745153	0.450263	1.741043	0.56968
RBM19	0.745153	0.450315	0.404647	3.950983
LINC01389	0.745303	0.450547	1.188903	1.324887
CTD-2350J1	0.745303	0.450556	1.201261	1.291312
CXCL3	0.745303	0.450632	1.244544	1.770171
AGPS	0.745303	0.450647	0.3136	6.833279
RASAL2-AS	0.745404	0.450769	-1.53393	0.666284
UGT1A4	0.745448	0.450911	-1.08693	1.75905
HNRNPUL1	0.745448	0.450964	0.452129	3.957458
MTMR8	0.745448	0.450976	-0.56053	3.105198

CYC1	0.745451	0.451038	-0.30732	5.978532
NSMAF	0.745468	0.451109	0.89279	0.935693
SMURF2P1	0.745732	0.451329	-0.36089	5.092953
FLJ37201	0.74581	0.451437	0.965962	1.841244
TRG-AS1	0.745867	0.451577	-1.21473	1.895615
GZF1	0.745867	0.451592	0.978846	1.816533
CCDC191	0.745945	0.4517	0.390963	4.778687
FOXP1	0.746443	0.452064	0.444388	4.517687
TBC1D13	0.746443	0.452147	-1.27977	1.859451
SMCR8	0.746443	0.452183	0.294714	6.117019
ARHGEF17	0.746527	0.452294	0.400029	5.216257
TXLNA	0.746536	0.452457	0.285368	7.014534
STRIP2	0.746536	0.452461	1.390372	1.08468
LYRM4-AS1	0.746536	0.452481	0.850181	2.282421
DDX24	0.746822	0.452715	0.570837	3.155272
MNT	0.74692	0.452834	-0.34135	6.541461
TTF2	0.746932	0.453042	-0.2963	5.562158
GRAP	0.746932	0.453064	0.951512	1.129057
NBPF12	0.746932	0.453077	0.341854	5.398554
PTPN21	0.746932	0.453109	-0.34424	5.585927
PDLIM5	0.746932	0.45324	-0.69312	3.518321
PPP1R15B	0.746932	0.453245	-0.2938	7.131963
CCDC39	0.746932	0.453266	-0.71116	3.087007
MIPEPP3	0.747032	0.453387	-1.16985	1.56288
TMEM199	0.747079	0.453476	-0.44729	3.97185
GABRA3	0.747249	0.453639	-1.75539	1.595256
LRP5L	0.747313	0.453739	0.710613	2.528377
MMP7	0.747594	0.45397	1.239272	2.19537
ULK2	0.748004	0.454279	-0.67988	2.921551
REG1A	0.748096	0.454396	-1.75284	5.605485
SNORA92	0.748236	0.454551	0.888258	1.23744
PRSS16	0.748236	0.454602	0.55152	3.183128
CEP192	0.74836	0.454768	0.276954	6.980563
TXNRD2	0.74836	0.454799	-0.83085	2.810592
CCL2	0.748493	0.45494	0.884002	1.534877
ZNF383	0.748545	0.455032	-0.76987	2.910214
XYLT1	0.748974	0.455354	-0.29812	6.605827
COG8	0.749091	0.455485	0.433173	3.927645
LRRC25	0.749253	0.455645	1.32248	0.587682
PRRG2	0.749272	0.455717	-1.8282	0.483521
METTL21EI	0.749398	0.455855	1.418482	0.468734
TEX261	0.749482	0.455967	0.263592	5.883087
BAG5	0.749493	0.456034	-1.5657	0.563895
EHD2	0.750005	0.456428	-0.45033	6.530094
ATP6AP2	0.750005	0.456498	-0.32422	6.122592

SKAP2	0.750005	0.456527	0.528296	2.7834
GPR137B	0.750589	0.456964	0.553483	4.26703
PLCXD3	0.750589	0.457005	-1.55703	2.528315
ADAD2	0.75063	0.457131	-1.42921	0.875897
COPRS	0.75063	0.457151	-0.5337	3.372814
GOLGA6L3	0.750736	0.457307	-1.02483	1.443307
FUNDC1	0.750736	0.457337	-0.53957	3.758672
SLC35C1	0.751269	0.457758	0.440892	3.466823
ALG1	0.751269	0.457888	0.527819	3.21276
JMJD1C-AS	0.751269	0.457951	1.16051	0.988733
CPNE2	0.751269	0.45801	-0.46794	4.447048
TSIX	0.751269	0.458148	-1.29907	2.545209
RAB3GAP2	0.751269	0.458173	-0.30343	6.576911
CAPN13	0.751269	0.458254	0.718944	4.088075
GPR180	0.751269	0.458256	-0.3853	4.488974
RASA4CP	0.751269	0.458263	0.461476	3.517447
BTBD10	0.751269	0.45827	-0.68131	3.221744
NTN3	0.752008	0.4588	0.899381	1.163203
SHPK	0.752008	0.458843	0.338177	5.022805
NSD2	0.752365	0.459198	-0.41966	5.272068
CHD6	0.752365	0.45921	-0.29197	7.490264
PRSS30P	0.752365	0.459243	1.260931	0.598381
MIR548V	0.752509	0.459392	0.893004	1.890526
KLRC4	0.752514	0.45949	1.190889	0.498774
CLPP	0.752514	0.459517	-0.49693	3.825126
LOC642361	0.752701	0.459693	0.95288	1.028761
ZNF767P	0.75295	0.459906	0.340652	4.671731
ADCK2	0.753096	0.460086	0.370085	4.393028
SH3BP5L	0.753096	0.460117	-0.3644	5.368396
NUTM2B	0.753176	0.460227	-0.76162	2.747858
POLR2J2	0.753177	0.460362	1.125188	0.872158
RPS18	0.753177	0.460408	0.496921	5.956842
HUS1B	0.753177	0.460418	1.040437	1.628249
GPI	0.753177	0.460471	-0.62476	3.945475
TPRG1-AS2	0.753196	0.460548	-1.34687	1.923114
NFKBID	0.753196	0.460605	0.46225	3.513683
FUNDC2	0.753329	0.460762	0.346266	4.66155
C1orf216	0.753329	0.460865	0.563438	2.579459
DGCR6L	0.753329	0.460869	-0.46159	3.6071
FRS2	0.753412	0.461029	1.22393	0.549565
GGT7	0.753412	0.461042	0.499909	3.549318
SNORA66	0.753836	0.461363	0.455067	5.418441
UGT1A3	0.754483	0.461863	-1.43985	1.181397
DUSP3	0.754483	0.461934	-0.29157	6.589393
NR4A2	0.754483	0.461942	-0.60889	5.415723

WAPL	0.754533	0.462034	-0.28728	7.701442
LOC101928	0.754753	0.46229	1.326853	1.120489
BAZ2B	0.754753	0.462291	0.753799	2.345449
PNPT1	0.755033	0.462524	0.305245	5.458945
HIST2H2AB	0.755104	0.462628	-0.57143	3.14878
LOC730668	0.755531	0.462951	1.2311	0.557543
C9orf3	0.755899	0.463301	0.671748	3.141376
SMARCA5	0.755899	0.463318	0.286842	7.435608
TMEM63B	0.755899	0.463388	-0.30865	6.396605
OFD1	0.755899	0.463421	-0.28434	6.713158
FAM110B	0.75598	0.463544	0.921501	2.219802
STAG1	0.75598	0.463593	-0.30481	6.771042
SARNP	0.756029	0.463685	1.183762	0.626063
LINC00667	0.756115	0.463974	0.478691	4.487654
FBXW7	0.756115	0.464004	-0.50279	3.553964
DISC2	0.756115	0.464023	-1.11881	1.973865
MPRIP	0.756115	0.464111	-1.43918	1.051018
C2orf76	0.756115	0.464197	-0.76643	2.551477
FAM161B	0.756115	0.464334	0.428433	3.704936
TMPRSS9	0.756115	0.464337	-0.58349	2.965331
EXOSC7	0.756115	0.464413	-0.6735	2.83767
KLF3-AS1	0.756115	0.464421	-0.97949	1.79362
MON2	0.756115	0.464441	0.796796	1.762194
SAT1	0.756115	0.464479	0.446155	5.146537
SNORD91B	0.756115	0.464499	-0.43654	5.236515
CHAD	0.756115	0.464534	0.440177	4.034598
SHC4	0.756153	0.464645	1.361667	0.8235
MIR941-1	0.756153	0.464767	-1.27538	0.990658
TXLNGY	0.756153	0.464801	0.529447	5.662784
BBOX1	0.756153	0.464802	0.458796	4.167096
TAMM41	0.756289	0.465001	0.59333	3.156683
USP42	0.756289	0.465008	0.353618	5.04004
GLOD5	0.756822	0.465544	1.113229	1.146477
ETAA1	0.756822	0.465564	-0.32976	5.17565
CSDC2	0.756822	0.465565	-1.03211	1.981211
SLC30A1	0.756822	0.465648	-0.42203	3.979093
RAB33B	0.756822	0.465727	0.426057	4.11243
ETFRF1	0.756822	0.465727	0.404913	4.13804
DPY19L1	0.756822	0.465765	0.411734	4.381632
RABGAP1	0.757329	0.466227	-0.29217	7.602026
KRT35	0.757329	0.466241	-1.99259	0.967398
ARHGAP31	0.757329	0.466276	1.179027	1.328926
CES3	0.757329	0.466323	-1.35119	1.19938
SMIM7	0.757415	0.466436	-0.37036	4.647693
ZNF230	0.757425	0.466566	0.670894	2.924317

CAHM	0.757425	0.46661	1.070067	0.916596
INHBB	0.757425	0.466697	0.893862	2.218328
PDXK	0.757425	0.466784	-0.30482	7.522206
WASF3	0.757425	0.466821	1.478175	0.727626
PLA2G4A	0.757425	0.466875	-1.24432	1.138808
PASK	0.757425	0.466914	-0.6712	3.165635
DCK	0.757425	0.466934	0.356499	4.686622
UQCRHL	0.757483	0.467031	0.341402	5.184576
RBPMS2	0.757736	0.467248	0.967219	2.537652
TESMIN	0.75794	0.467435	-1.82574	0.490721
CEND1	0.758183	0.467646	-1.29079	1.421086
DNPEP	0.758217	0.467731	0.313676	4.75628
SCUBE2	0.758217	0.46779	0.969911	1.741625
LOC728024	0.75823	0.46786	0.86054	1.848854
LRPPRC	0.758342	0.468023	-0.28589	7.513244
SDHAF1	0.758342	0.468052	0.570865	2.764706
OR7E14P	0.758793	0.468391	-0.8482	2.606447
ITGAD	0.758947	0.468565	-1.48866	0.895982
GNL3	0.758947	0.468665	0.833835	1.65304
SLC5A5	0.758947	0.468706	1.112449	2.235273
CCDC127	0.758947	0.468785	0.621887	3.307911
GOLGA8A	0.758947	0.468794	0.317956	5.790825
STX1B	0.759156	0.468985	-1.03381	1.818873
ATG16L2	0.759172	0.469084	-0.3302	5.63126
EPM2A	0.759172	0.469117	-1.09116	1.481875
ENPEP	0.759281	0.469287	0.739779	2.506681
FOXA1	0.759281	0.469377	-0.318	5.887648
TFAP2A-AS	0.759281	0.469522	-1.14873	0.709762
CDC37L1	0.759281	0.469564	-0.33993	5.296965
DCUN1D2	0.759281	0.469574	0.448077	4.350804
TMEM266	0.759281	0.469627	0.96204	0.973499
MIR4265	0.759281	0.469716	1.050505	1.207884
SERTAD3	0.759281	0.469771	-1.07964	1.158812
MINOS1	0.759281	0.469787	-1.31328	1.042724
WFIKKN1	0.759281	0.469846	1.078653	1.01108
LOC100506	0.759281	0.469906	0.836086	1.849262
SEC23IP	0.759281	0.469923	0.421026	3.479109
CES2	0.759643	0.470209	-1.25099	1.575438
FZD6	0.759677	0.470291	1.289795	0.694651
CFAP46	0.759733	0.470436	0.727117	3.180215
CD300LB	0.759733	0.470448	1.347795	1.065309
DONSON	0.759749	0.47052	-0.56282	3.598385
B4GALNT3	0.759801	0.470721	-0.43192	6.965416
LOC100133	0.759801	0.470722	-0.41435	4.051551
BTN2A1	0.759801	0.470737	-1.69101	0.652706

CHD1	0.759871	0.470856	-0.30932	7.372182
REEP5	0.759871	0.470903	-0.29827	6.616414
MFSD8	0.759962	0.471075	0.314162	5.155643
TCEAL4	0.759962	0.471083	1.050029	1.107446
ALKBH4	0.759999	0.471168	0.592376	2.721037
LINC01465	0.760064	0.471269	0.731617	2.268029
HSCB	0.760071	0.471335	0.626566	3.222547
PPP2R2D	0.76011	0.471421	-0.90442	3.102186
NUDT11	0.760463	0.471783	1.151107	1.337211
NUDT18	0.760463	0.471795	-0.58135	3.132275
FREM2	0.760463	0.471961	0.758404	3.090082
KCNJ8	0.760463	0.471974	0.844266	2.094609
CCDC85A	0.760463	0.472051	1.157449	0.889925
CDKAL1	0.760463	0.472068	0.295425	5.307873
IQSEC2	0.760463	0.472083	0.693275	2.049059
HCG25	0.760463	0.472132	0.86724	1.92273
MIR27B	0.760735	0.472363	-0.85934	2.163103
CCDC134	0.760853	0.472498	0.9013	1.837066
C1QTNF2	0.761239	0.4728	1.111683	0.761682
MCPH1-AS	0.761546	0.473058	-0.95748	2.196972
COL7A1	0.761546	0.473113	-0.3252	8.303118
NMT2	0.761574	0.47324	0.601635	2.52457
BRWD1	0.761574	0.473254	-0.29164	7.0276
KALRN	0.761725	0.47341	0.709873	2.535098
SAV1	0.761725	0.473599	0.362534	4.886919
TRADD	0.761725	0.473702	0.382573	3.884521
RPS14P3	0.761725	0.473711	-0.74066	2.49222
SLC25A44	0.761725	0.473776	0.756885	2.205545
CCNL1	0.761725	0.473935	-0.39231	5.322293
PTENP1	0.761725	0.473942	0.421305	4.704325
GLI3	0.761725	0.473957	-0.39883	4.762625
ZNF354B	0.761725	0.473959	0.527621	3.978848
LOC100506	0.761725	0.473966	0.67729	2.176816
ZNF404	0.761725	0.474026	0.960798	1.030503
EAPP	0.761885	0.474245	-0.35178	5.491172
ZNF443	0.761885	0.47425	-0.70014	2.945391
MRPL18	0.762418	0.474643	-0.31855	5.018522
VRK1	0.762442	0.47472	-0.36786	4.441392
TNFRSF9	0.762453	0.474801	-1.25927	0.819536
ATP6V1C2	0.762453	0.47485	-1.86075	0.627352
PPARGC1A	0.762554	0.475032	0.589146	3.603159
NKX6-2	0.762554	0.475036	-1.71415	0.518163
LOC105379	0.762805	0.475313	0.976458	0.835303
SERPINE1	0.762805	0.475317	0.547041	5.76826
AGAP3	0.763014	0.475552	-0.66911	2.281764

SNORD55	0.763014	0.475635	0.399264	6.453241
HASPIN	0.763014	0.475652	-0.63903	3.213437
UBE4B	0.763014	0.475694	-1.00438	1.833941
PDCD7	0.763435	0.476019	-0.3438	5.505481
LRRC8D	0.763713	0.476266	0.806751	1.811771
FBLIM1	0.763713	0.476357	1.053847	1.274879
FBRSL1	0.763713	0.476377	0.27906	5.88251
PANK4	0.763857	0.476594	0.851229	0.961999
KDM5A	0.763857	0.476677	-0.29123	7.974095
ZNF792	0.763857	0.476771	0.532169	2.994873
KHDRBS1	0.763857	0.4768	0.748405	2.238289
SGF29	0.763857	0.476811	-0.52855	3.282765
RAP2C-AS1	0.763857	0.476838	0.474675	3.272514
MAML3	0.763968	0.476996	-0.28293	6.267669
RWDD2B	0.763968	0.477032	-0.64192	3.737812
TP53I3	0.764069	0.477157	0.853338	1.362677
HUNK	0.764096	0.477235	0.713781	2.103274
OVCA2	0.764199	0.477361	0.769589	1.37747
TNNI1	0.76442	0.477612	0.702017	2.75132
LOC653712	0.76442	0.477623	-1.08997	2.726148
MMP13	0.764421	0.477686	1.286339	0.599567
ODF3B	0.764441	0.477831	-0.55409	3.197468
ERCC4	0.764441	0.477859	-0.41528	4.161994
ZNF71	0.764441	0.477907	-0.89672	2.517318
PCBD2	0.764441	0.477988	0.379128	4.775426
KIAA0319L	0.764441	0.47803	0.267156	7.078196
WNT3A	0.764441	0.478069	-1.27654	0.782436
WDR81	0.76456	0.47822	0.619922	3.598944
PLK5	0.76456	0.478323	1.213296	0.917589
MIR3975	0.76456	0.478361	-1.04324	2.403945
CH17-340M	0.76456	0.478451	0.958075	1.29276
RAB1A	0.76456	0.478477	0.330028	4.911921
TRMT44	0.76456	0.478542	-0.35919	5.158802
UNK	0.76456	0.478578	-0.29196	5.925534
L3MBTL1	0.764597	0.478724	-1.38149	0.65755
NAP1L4	0.764597	0.478755	-0.48142	5.461086
GFOD1	0.764597	0.478786	-0.97545	2.100489
CXCL9	0.764643	0.478877	0.957146	1.985627
LOC100131	0.764839	0.479062	1.341907	1.67639
GPRIN2	0.765032	0.479311	0.500372	3.057413
POLI	0.765032	0.479399	0.346641	4.66298
UNKL	0.765032	0.47942	-0.65944	3.102865
CEBPD	0.765032	0.479431	-0.31625	6.401417
ACVR1B	0.765454	0.479811	0.538267	2.966309
MIR4742	0.765454	0.479819	-1.71415	0.545854

KLF16	0.765567	0.479952	0.337938	4.746962
ZNF512	0.765706	0.480146	1.045322	1.040219
CTDSPL2	0.765706	0.480163	-0.35075	6.117841
RPL36AL	0.765876	0.480393	-0.27879	7.610799
NFX1	0.765876	0.48043	-0.40072	4.426294
MSL2	0.765876	0.480487	0.422463	3.538879
APBA1	0.765876	0.480518	0.743435	2.595398
FADS1	0.766158	0.480881	0.490832	3.884865
LAMB4	0.766158	0.480923	-1.29044	2.44335
ZC3H11A	0.766158	0.481021	-0.28668	8.342488
CORO1C	0.766158	0.481045	-0.97538	1.848366
TMX1	0.766158	0.48109	-0.30168	6.210932
CDC23	0.766158	0.481118	-0.43083	4.904956
TMEM183/	0.766158	0.481231	-0.29142	6.039217
BEND7	0.766158	0.481251	1.055105	0.906038
SNORA70G	0.766158	0.481253	0.73382	2.547322
ZNF491	0.766266	0.481392	0.817071	2.069368
MPV17L2	0.766266	0.48146	-1.03386	1.403527
RILPL1	0.766266	0.481522	0.391444	4.289169
CACNA2D4	0.766266	0.481611	0.815973	1.851064
MSRB2	0.766266	0.481658	0.417292	3.9558
PICALM	0.766266	0.481694	1.143206	0.88706
TDP1	0.766752	0.482135	0.971498	1.057597
TERF2IP	0.766752	0.482147	-0.28188	6.076802
GOLGA3	0.766752	0.482185	0.260985	7.686132
LAMB2P1	0.766781	0.482266	1.288346	1.238576
SNORA26	0.766798	0.482339	0.307915	8.082416
TMUB1	0.7671	0.482591	-0.55272	3.19238
POLR1A	0.767352	0.482811	-0.2844	6.508514
MBTPS1	0.767777	0.483188	0.263307	7.096494
DIABLO	0.767777	0.483215	1.017584	1.067856
RDH16	0.767777	0.483338	-1.54044	0.705393
WDR37	0.767777	0.483354	0.342343	4.994578
FBXO46	0.767777	0.483458	-0.37999	5.277811
ZBTB20-AS	0.767777	0.483502	-1.17281	1.28718
RIC1	0.767777	0.48353	1.253163	0.950969
GCC1	0.767777	0.483576	0.301003	4.967647
CNTN2	0.767842	0.483679	-0.79706	2.385322
CRLF1	0.767887	0.48377	-1.37343	1.253344
TRIP11	0.76803	0.483931	-0.28655	7.867859
XRN2	0.76803	0.483984	0.651254	2.574632
SSRP1	0.768843	0.48457	-0.28428	7.308146
LINC00910	0.768843	0.484621	-0.76483	2.4065
ATF7IP	0.769042	0.484809	-1.13707	2.137576
ZBTB6	0.769134	0.484949	-0.37076	4.575693

EPPK1	0.769134	0.48505	-0.5213	7.276778
CARM1	0.769134	0.485054	-0.3058	6.060447
TACC1	0.769345	0.48525	0.783363	2.041987
ST7-AS2	0.769628	0.48549	0.720252	2.991677
LINC01521	0.769756	0.485633	0.903715	1.862965
ZNF699	0.770002	0.485888	-0.72687	3.077149
SNORA52	0.770002	0.485913	0.500947	4.124502
LINC01082	0.770165	0.486102	1.10362	0.486288
ISG20L2	0.770165	0.486141	0.296081	5.263596
NET1	0.770176	0.486247	-1.03727	1.634955
GPR75	0.770176	0.486273	0.578632	2.489553
LINC01138	0.770229	0.486368	-0.85312	2.916527
PFDN2	0.770766	0.486839	-0.35129	4.780419
BPTF	0.770766	0.486858	-0.4746	3.695285
BIRC6-AS2	0.770766	0.486899	-1.1391	1.898146
LPP	0.770766	0.487032	0.434553	3.89249
RIPK2	0.770766	0.487081	-0.7673	3.151168
SIMC1	0.770766	0.487082	-1.3916	1.14848
TRAF2	0.770869	0.487237	-0.35492	4.267499
SNORD80	0.770869	0.487272	-0.40792	3.72707
GHR	0.770947	0.487384	-1.09118	1.565576
MIR21	0.771118	0.487603	-0.86439	2.606912
GHET1	0.771118	0.487659	1.272716	0.649193
MITD1	0.771118	0.487679	0.32165	4.837463
SH3YL1	0.77128	0.487877	-0.35811	4.766612
MB21D2	0.77128	0.487906	-0.71817	2.675861
MLLT11	0.771312	0.48799	0.711515	2.695262
FRG1HP	0.77176	0.488335	-0.51041	4.003485
FARP1	0.771974	0.488582	0.794314	1.654533
C4orf33	0.771974	0.488649	-1.24248	0.799921
AGBL5	0.771974	0.488668	-1.18684	0.889145
ANXA2R	0.771974	0.488783	0.719045	2.05014
WIPI1	0.771974	0.488801	-0.31815	5.283429
CELP	0.771974	0.488846	0.96409	1.102234
KIF3C	0.77211	0.489026	-0.50813	3.350719
SRGAP2	0.77211	0.489085	0.562897	3.611413
NOP53-AS1	0.77211	0.48912	0.867562	1.236337
BORCS7	0.772167	0.489249	-0.76668	2.389704
SHLD1	0.772167	0.489281	1.14228	0.990299
FOXN2	0.772245	0.489393	0.345375	5.269182
GAPDH	0.773441	0.490213	-0.95493	2.699619
LOC646762	0.773555	0.490404	0.371565	4.220424
TRAPPC2L	0.773555	0.490511	-0.42226	4.003424
CYB5R2	0.773555	0.490613	-1.21351	1.049068
MIR3165	0.773555	0.490683	1.575079	0.488624

RBM48	0.773555	0.490693	-0.44989	3.833402
ZNF17	0.773555	0.490742	-0.55053	4.023649
NKAP	0.773555	0.490785	0.286262	4.785275
ZRANB2-AS	0.773555	0.490812	1.56984	0.431377
ZDHHC17	0.773555	0.490855	0.337683	5.133641
MROH1	0.773555	0.490912	-0.94442	1.515845
LINC01963	0.773717	0.491078	-0.62591	2.741744
LOC100190	0.773856	0.491229	0.273918	5.767397
LOC339666	0.773953	0.491403	0.855703	1.514693
EMG1	0.773953	0.491455	-0.35407	4.734412
TFB1M	0.773953	0.49154	0.505136	4.055963
EMC3-AS1	0.773953	0.491541	0.549475	3.551973
FAM129A	0.774273	0.491807	-0.2825	7.4463
TBC1D10B	0.774276	0.491923	0.257607	6.213216
GTF2IP20	0.774276	0.491934	-0.44127	3.534627
STX18-IT1	0.774369	0.49209	-1.5566	0.664987
ABCC2	0.774369	0.492206	0.722717	2.055071
POLK	0.774369	0.492271	0.278506	5.595476
ABCA17P	0.774369	0.492323	1.199076	1.29713
FAM151B	0.774369	0.49234	-1.57775	1.630994
FAM98B	0.774369	0.492375	0.306222	5.486634
SORBS2	0.774369	0.492432	0.750457	2.139684
LDLRAP1	0.774558	0.492616	0.283884	5.157922
CHTF18	0.775192	0.493112	-0.41255	3.898965
IRX2	0.775192	0.493144	-1.08051	1.871048
PLEKHG7	0.775286	0.493267	0.68863	2.73945
FUBP1	0.775407	0.493407	0.826077	2.631828
TMX4	0.775421	0.493484	0.266377	6.427352
MMP14	0.775421	0.493579	0.267039	7.618864
ACSS3	0.775421	0.493623	0.832536	1.798555
F2RL3	0.775421	0.493667	0.572249	3.525581
SVIL	0.775433	0.493785	-0.28898	6.392713
CERNA1	0.775433	0.493923	1.069802	0.567527
CLK4	0.775433	0.493988	0.331254	5.865332
ZNF653	0.775433	0.494013	0.439201	2.942769
HGSNAT	0.775433	0.494026	-0.27643	6.709565
LRRC57	0.775433	0.494105	0.331989	4.579438
ZCRB1	0.775433	0.494172	-0.30958	5.637863
CCDC102A	0.775433	0.494177	0.542823	3.036859
MRPL12	0.775591	0.494415	0.381499	4.672012
LOC642366	0.775591	0.494416	1.340785	0.895512
PACRG-AS3	0.775591	0.494466	-1.29701	1.198027
ZFAND2A	0.775611	0.49457	0.373051	3.766429
POLR3A	0.775611	0.494674	-0.2685	5.587352
LINC02102	0.775611	0.494697	-1.16454	1.328944

CPLANE1	0.775611	0.49476	-0.37056	6.508664
POLR2K	0.775611	0.494864	0.339355	4.231419
OSMR	0.775611	0.495041	-0.33792	5.585255
ABHD18	0.775611	0.495113	0.376611	4.678401
PCSK4	0.775611	0.495219	0.687167	2.428343
ZNF860	0.775611	0.495283	-0.49358	3.595552
ELOVL1	0.775611	0.495325	-1.21711	1.045728
CUTALP	0.775611	0.49535	0.411117	4.531611
SMO	0.775611	0.495366	-0.44186	3.846999
DECR1	0.775611	0.495433	-0.28348	5.880697
CMYA5	0.775611	0.495458	0.400409	4.269294
ANAPC1	0.775611	0.495466	-0.27573	5.531286
PPID	0.775611	0.495522	0.284236	5.538263
BRF1	0.775611	0.495547	-0.59668	2.529688
COX7A1	0.775805	0.495734	-0.59509	2.819013
EEF1DP3	0.775836	0.495816	0.803825	1.922005
TEDDM1	0.776042	0.496052	0.955487	1.035041
HIST1H2BC	0.776042	0.496073	-0.31081	6.542256
SYS1	0.776184	0.496227	0.5774	2.500781
BST2	0.776332	0.496385	0.555858	3.910562
N4BP2L2-IT	0.776372	0.496473	0.290902	5.418017
CYB5B	0.776496	0.496625	0.267526	5.745819
LCMT1	0.776496	0.496678	0.792188	1.60888
CASKIN2	0.776779	0.496964	0.51541	2.516164
GRIK1	0.776779	0.497058	1.261816	0.921432
SCMH1	0.776779	0.497087	-1.04564	1.752791
HAND2	0.776779	0.497111	1.448701	0.983273
LOC100499	0.776779	0.497259	1.108307	1.27006
MGC16275	0.776779	0.497291	-1.12979	1.293707
LRIT3	0.776779	0.497299	-1.43964	0.486442
C15orf65	0.777198	0.497631	1.262842	0.746025
FAM228B	0.777236	0.497718	1.038325	1.12547
SDHAF2	0.777361	0.497861	0.37826	4.188295
GNAS	0.777723	0.498161	-1.11765	1.045561
ACADM	0.777723	0.498436	1.436655	0.660152
ZFR	0.777723	0.498443	-0.26746	7.39182
PNRC2	0.777723	0.498474	0.249705	7.066911
CIAO1	0.777723	0.498519	-0.27538	6.056924
LINC00092	0.777723	0.49857	1.363549	0.845011
VTRNA1-3	0.777723	0.498579	-0.55394	3.613018
IFITM1	0.777723	0.498645	0.452451	4.701688
TAF3	0.777723	0.498719	0.291218	5.385738
MIR29C	0.777723	0.498757	0.699391	2.342406
BBS1	0.777723	0.498827	0.322409	4.65878
SNORD70	0.777723	0.498859	-0.70678	2.422928

CNOT11	0.777723	0.498912	-0.28024	6.268222
ERI1	0.777948	0.499119	-0.36572	4.601712
SLC46A2	0.777988	0.499208	0.522801	2.601757
HPF1	0.778056	0.499324	-0.76044	2.95669
EEF1AKMT	0.778056	0.499397	-1.08045	1.644486
KIAA1143	0.778056	0.499503	-0.30628	5.151844
MRPL14	0.778056	0.499535	0.362456	4.271586
ZNF331	0.778056	0.499566	1.219918	0.915283
DNM3OS	0.778226	0.499831	0.896406	2.032321
LRRC41	0.778226	0.49985	-0.2724	6.012726
GATA6-AS1	0.778226	0.49987	0.891636	3.401333
LINC02476	0.778226	0.499928	1.526458	1.188486
SPAG16	0.778312	0.500201	-0.7686	3.65135
RRS1-AS1	0.778312	0.500212	-1.21021	2.044764
GSTM2	0.778312	0.500252	-1.22959	1.323432
MFN2	0.778312	0.500287	0.532738	2.296393
GAPDHS	0.778312	0.50033	0.476327	3.115181
RP9P	0.778312	0.500361	-0.55931	3.352842
PPM1N	0.778536	0.500594	0.74136	2.085749
PPP2R2A	0.778536	0.500681	-0.70668	2.077962
PHYH	0.778536	0.500694	1.158653	0.577859
IKZF4	0.778571	0.50078	-0.47096	4.321669
DDX55	0.778636	0.500885	0.26408	5.51628
WARS2-IT1	0.778744	0.501021	1.314479	1.008336
FLJ42627	0.778744	0.501081	0.291536	5.334437
GLIPR2	0.77881	0.501186	0.801595	2.508295
SMAP1	0.779242	0.501532	-1.13858	0.716475
TECTA	0.779242	0.501663	-1.52517	0.772893
NCBP2	0.779242	0.501694	0.69518	1.929242
SCARB2	0.779242	0.501717	0.368029	4.113602
C2CD4B	0.779504	0.501949	1.042835	2.122006
WFDC5	0.779587	0.502088	-1.14318	1.62316
IGHMBP2	0.779587	0.502224	0.298587	4.90815
ZNF652	0.779587	0.502268	0.92884	0.779101
SCN9A	0.779587	0.502341	0.738559	2.489718
MRPL4	0.779587	0.502376	-1.21893	1.164039
SLC7A5P1	0.779587	0.502462	0.834862	1.796782
GUSBP1	0.779587	0.50249	0.393904	4.32937
ERO1B	0.779587	0.502507	-0.69123	5.294392
ATP2B1-AS	0.779661	0.502618	0.717579	2.512239
LOC10192[illegible]	0.779829	0.50279	1.22968	0.842962
GPBP1L1	0.779868	0.502878	-0.25847	7.705925
LOC38990[illegible]	0.780193	0.503231	-0.40845	3.791147
MIR7849	0.780193	0.503253	1.288403	0.722554
SNORA74B	0.780193	0.503277	-0.44532	4.8512

VMAC	0.78026	0.503384	0.437173	3.486775
RASL11A	0.780606	0.503674	0.716462	1.855104
SPG11	0.780606	0.503769	0.668438	1.923327
POM121	0.780606	0.503803	-0.31936	4.855795
JAGN1	0.780606	0.503859	0.396128	3.917675
FLT4	0.780757	0.50402	0.814463	1.473004
CACYBP	0.780878	0.504168	-0.86791	1.906024
LCMT1-AS2	0.780878	0.504225	0.436999	3.736709
NDUFB10	0.781006	0.504371	-0.27016	5.509123
MSANTD4	0.781264	0.5046	0.473293	4.388639
TBCK	0.781336	0.50471	0.699884	1.925488
SNORA51	0.781483	0.504868	0.32552	5.206458
IQCC	0.781585	0.504998	0.93477	0.942149
PDS5B	0.78169	0.505216	0.296532	6.552032
VTRNA2-1	0.78169	0.505293	0.350005	6.045312
KLC4	0.78169	0.505361	0.939106	0.609711
SNHG6	0.78169	0.505382	0.319538	4.776503
LSM8	0.78169	0.505388	0.239433	5.889211
VTN	0.78169	0.505445	-1.22505	0.635337
SNIP1	0.781695	0.505529	-0.36645	5.017498
SPINDOC	0.781695	0.505575	0.45065	2.977722
EIF4EBP2	0.78207	0.505956	-0.25869	8.284978
MIR320E	0.78207	0.506011	0.818354	1.551831
PGM1	0.78207	0.506018	-0.52878	3.81018
SNORA104	0.78207	0.506071	0.891092	1.571284
MRPL48	0.782296	0.506281	0.376218	4.170192
AKR1C6P	0.782326	0.506367	-1.8279	1.113316
TMEM185B	0.782326	0.506429	-0.27253	5.460329
ISG20	0.782326	0.506535	1.217148	0.4404
CUL5	0.782326	0.506554	-0.26304	6.439309
CDC73	0.782458	0.506703	-0.26002	6.740809
DCHS2	0.783153	0.507223	0.671713	3.142443
SS18L1	0.783153	0.50728	0.9755	1.049676
TRIM38	0.783613	0.507704	-0.27598	6.793227
CHM	0.783613	0.507705	-0.31674	5.055548
LNCOC1	0.783783	0.507878	-1.29917	1.042343
POTEE	0.783841	0.507979	0.540596	2.092914
RBMS2	0.784197	0.508274	0.282277	6.259643
AQP9	0.78425	0.508431	-1.64378	0.843729
CHADL	0.78425	0.508435	0.790972	1.817137
SNORD2	0.784548	0.508741	0.409545	3.79548
TTC23	0.784548	0.508755	-0.75097	2.60415
NATD1	0.78464	0.508878	-0.37206	4.402989
TMEM116	0.784769	0.509074	-1.58024	0.790347
ARNT2	0.784769	0.509089	0.730493	1.970274

MAP10	0.785233	0.509568	0.644271	2.129301
DARS-AS1	0.785233	0.509576	-1.22252	1.396878
EFNB3	0.785233	0.509581	-0.58193	2.641228
PRRX2	0.785233	0.509658	0.837829	0.93778
C15orf40	0.785233	0.509708	-0.9087	2.04576
ADARB2	0.785301	0.509816	0.579957	2.015798
MRPL38	0.785491	0.510025	0.27849	5.119087
TCF7	0.785491	0.510067	0.794652	1.407916
BLOC1S5	0.785609	0.510207	0.971791	1.165966
GPR137C	0.785621	0.510278	1.011433	0.954281
TBC1D12	0.785907	0.510562	0.26108	5.402006
LINC01176	0.785907	0.510621	-0.52509	3.104727
HMGN3-AS	0.785907	0.510655	-0.74837	2.630842
PLEKHF2	0.786115	0.510918	-0.39015	5.227063
ZNF649	0.786115	0.510977	-0.59105	3.399721
LGALS3	0.786115	0.511063	-0.33919	5.376901
SNRNP48	0.786115	0.511076	0.292234	5.12903
CD160	0.786115	0.511109	-1.49254	1.189937
MIR429	0.786124	0.511178	0.835301	1.491292
CLN6	0.786534	0.511547	0.389487	4.112105
DCUN1D3	0.786534	0.511622	-0.4141	4.893575
SLC16A7	0.786534	0.511697	-0.86017	1.869647
MEF2D	0.786534	0.511729	-0.83291	1.90056
C16orf74	0.786534	0.511834	-0.77432	1.958986
ARAP1	0.786534	0.511859	0.675677	1.74961
JUN	0.786534	0.511904	-0.34031	8.506171
PHKA2	0.786534	0.511954	0.283585	5.316381
DNAH17	0.786762	0.512166	0.427062	3.222618
OMA1	0.788335	0.513301	0.271639	5.358538
TNFRSF13E	0.788335	0.513339	0.933215	1.71057
CTNNB1	0.788335	0.513468	0.664684	1.627086
FCF1	0.788335	0.513486	-0.31318	5.296121
SNORD14A	0.788335	0.51351	-0.43454	4.972521
TSEN2	0.788346	0.51358	0.697279	2.459311
CCDC78	0.788512	0.513791	0.608343	2.198259
TFB2M	0.788512	0.513851	-0.3876	4.164943
WDR1	0.788512	0.513928	0.242165	6.37507
NAGS	0.788512	0.513944	-1.13471	1.186494
WDR43	0.788618	0.514138	-0.27279	7.124035
SRGAP1	0.788618	0.514163	0.259608	6.734788
DGKI	0.788618	0.514216	0.612789	2.34905
CASP10	0.788618	0.514269	-1.53609	0.551861
CFAP70	0.788619	0.514333	0.591974	2.80196
RNF103	0.788833	0.514634	0.44237	3.425432
TMED8	0.788833	0.514717	0.479061	3.673727

DPY19L1P1	0.788833	0.514723	0.780118	2.517351
WRN	0.788833	0.514729	-0.33643	5.88172
LOC101927	0.788993	0.514998	-0.92384	1.39403
NPHP4	0.788993	0.51501	-1.33392	0.737727
FAM84A	0.788993	0.515024	-0.28882	7.081697
PSTK	0.789344	0.515318	-1.03104	1.704089
LITAF	0.789401	0.51542	-1.10599	0.98918
TMEM125	0.789401	0.515534	0.417657	3.64959
SHANK1	0.789401	0.515547	-1.57898	0.676016
PTPN4	0.789418	0.515622	0.247445	5.845948
DYNLL1	0.789571	0.515786	0.974425	0.797812
ASH1L	0.78965	0.515901	-0.26618	8.808213
CUEDC2	0.78974	0.516056	0.32498	4.241909
KLHL9	0.78974	0.516088	0.288604	5.320172
PHF8	0.790142	0.516424	0.828013	1.828595
OGFRP1	0.790142	0.516539	0.66117	1.879851
SNORA20	0.790142	0.516543	0.303588	7.652453
EIF4E2	0.790285	0.5167	-0.38978	3.861221
TNFAIP8	0.790308	0.516833	-0.96738	1.693267
LOC100240	0.790308	0.516844	0.933897	1.243802
GAL3ST4	0.790317	0.516913	-0.65074	2.967082
MIR6883	0.790483	0.517086	0.916417	0.71464
TBX1	0.790531	0.517181	-1.23926	1.022125
7-Mar	0.790983	0.517621	-1.14932	1.969326
SMPD2	0.790983	0.517657	-0.45512	3.520048
MAB21L2	0.790983	0.517669	-0.42013	3.927012
SCAND2P	0.791081	0.517797	-0.7031	2.598342
UBE2C	0.791128	0.517892	0.839183	1.528902
SELENOP	0.791221	0.518017	1.142115	0.737013
METRNL	0.791371	0.51818	-0.55838	3.754477
BICRA	0.791445	0.518292	-0.29754	4.874252
ABCD3	0.79164	0.518484	0.255489	6.862701
LOC100270	0.791698	0.518586	0.958801	1.040451
MFSD6	0.791792	0.518827	-0.26339	7.910987
PPAT	0.791792	0.518876	0.359219	4.313849
LMO7	0.791792	0.518916	0.581476	2.791717
TRIM14	0.791792	0.518968	-0.28611	5.67103
AK9	0.791792	0.519032	0.439244	3.748486
THOC3	0.791792	0.519034	-0.43488	4.498241
NOP58	0.791792	0.519096	-0.29244	6.706264
DSCR9	0.79272	0.519769	1.169845	0.441617
ARFGEF1	0.792791	0.5199	-0.2624	8.212443
PCDHB3	0.792791	0.51995	-1.14938	1.163632
NEIL1	0.792791	0.520008	0.857552	1.182033
VASN	0.792958	0.520182	-1.19708	0.464681

BOLA1	0.793023	0.520289	-0.81509	1.957777
INTS10	0.793035	0.520361	0.237436	6.392913
IRF3	0.7932	0.520558	0.855322	0.838805
DAPK3	0.7932	0.520657	0.295354	5.653879
HDAC1	0.7932	0.520662	0.232895	7.127243
TET2-AS1	0.793251	0.520818	-0.55816	3.594844
RIMS2	0.793251	0.520824	-1.32553	1.168931
SNORD22	0.793311	0.520967	-0.31485	7.855829
CTBS	0.793311	0.521025	0.357821	4.543939
ABLIM2	0.793311	0.521056	-0.70392	3.11081
ATMIN	0.793492	0.521381	0.5871	2.231077
NPTN-IT1	0.793492	0.52141	-0.39659	4.780058
TH2LCRR	0.793492	0.521467	0.818258	1.61816
CEBPZ	0.793492	0.521493	-0.27618	6.390412
SOS2	0.793492	0.521496	-0.24852	6.780707
TMEM61	0.793991	0.521908	1.011437	1.019551
DOCK11	0.793991	0.521953	0.344194	5.040355
SNRNP40	0.794032	0.522044	0.309406	4.80453
LIMS1	0.794198	0.522266	0.318843	4.161129
SNORA53	0.794198	0.522282	-0.28477	7.261095
LINC00900	0.794407	0.522484	0.902017	0.658971
DYNC1H1	0.794439	0.522569	-0.25261	10.00573
REREP3	0.794738	0.522892	0.712655	1.481303
SEC24A	0.794738	0.523041	-0.26807	5.904198
INE1	0.794738	0.523082	-0.37021	4.511129
DR1	0.794738	0.523094	-0.25569	6.371556
CEP19	0.794738	0.523095	0.665237	1.592539
CXADR	0.794738	0.523152	0.716813	1.761281
AKAP8L	0.794797	0.523265	0.847847	1.187764
PKD1L1	0.794797	0.52332	0.72299	3.065433
GABPB2	0.795338	0.52382	-0.3233	4.960621
CALCOCO2	0.795338	0.523858	0.762894	1.944637
ZNF609	0.795338	0.523894	0.234492	7.232098
EIF3D	0.795338	0.523936	-0.25306	7.748338
STAG3L1	0.795338	0.524002	0.44165	3.259249
FKBP2	0.795338	0.524063	-1.01343	1.097556
EMC9	0.795386	0.52421	0.566416	2.528638
VPS16	0.795386	0.524332	-0.82085	1.948406
LACTB	0.795386	0.524389	0.697572	2.453912
YJU2	0.795386	0.524453	0.324742	4.129267
PPIA	0.795386	0.5245	-0.9298	1.751124
BAHCC1	0.795386	0.524534	-0.53696	4.830767
FABP3	0.795386	0.524545	-1.10732	2.154641
ITGB4	0.795844	0.524912	0.945393	0.807636
RNF146	0.795959	0.525108	1.003635	1.127518

SLC1A3	0.795959	0.525117	0.951947	1.391621
SNORD52	0.796326	0.525529	0.752356	1.40919
KIAA0513	0.796326	0.525637	0.770096	1.891245
MAGEE1	0.796326	0.52567	-1.17796	0.845842
VAV3	0.796326	0.525812	0.695576	3.014747
LINC01679	0.796326	0.52586	-0.79982	1.501214
PABPC1L	0.796326	0.525915	0.340556	4.208888
CDK16	0.796326	0.525976	-0.68267	2.514727
ERICH1	0.796326	0.526107	-0.66612	2.662097
EIF2B3	0.796326	0.526124	-1.07612	1.65211
UHRF1BP1	0.796326	0.526161	0.260228	5.166929
IFNGR2	0.796326	0.526234	-0.43065	4.763991
LOC101929	0.796326	0.526273	-0.63917	2.21152
ZNF77	0.796326	0.526365	0.447757	2.964527
CRYBA1	0.796326	0.526425	-1.47542	0.41066
REPIN1	0.796326	0.526426	0.691338	1.562665
COLCA1	0.796326	0.526432	1.019187	0.795026
SCX	0.796326	0.526506	0.604819	2.101405
SEMA3D	0.796326	0.526576	1.074229	1.506282
ZBTB32	0.796326	0.526584	0.714314	1.994105
SMG1P7	0.79652	0.526807	-0.45334	3.473303
MLH1	0.79652	0.526894	-1.36208	1.460786
LOC100130	0.79652	0.526912	-0.6706	2.376382
GNG3	0.79652	0.526971	-1.1945	0.887221
ZNF100	0.796557	0.527059	1.105159	0.974541
PNPLA8	0.796569	0.527132	-0.98268	1.712464
MBTD1	0.796584	0.527206	-0.27402	6.155036
FOXP1-AS1	0.796942	0.527508	-0.56635	3.60786
GJB7	0.796969	0.527635	0.87663	1.006574
TMEM38A	0.796969	0.527655	0.56453	2.347426
LPAR6	0.797306	0.527943	-0.82742	1.888005
RNF19A	0.797355	0.52804	-0.54162	2.775331
GPS2	0.797415	0.528144	-0.25557	6.411016
SPDYE1	0.797594	0.528327	-0.34569	4.674473
JADE2	0.797598	0.528421	0.851389	1.1036
BTBD9	0.797598	0.528489	0.844418	1.175014
ASXL3	0.797598	0.528539	0.70515	2.495142
SPAG8	0.797598	0.528634	1.275668	0.450111
SNORD117	0.797598	0.528653	-0.90563	1.770678
POMZP3	0.797975	0.529017	1.174998	0.770299
FLJ30679	0.797975	0.529044	-2.475	0.559938
SYT9	0.797975	0.529096	1.116623	0.643223
PLA2G15	0.798216	0.529321	0.416273	3.056391
SOX13	0.798449	0.52954	0.258032	5.395324
SLC43A3	0.798455	0.529608	0.742217	0.952732

THEM5	0.799082	0.53009	-0.74296	1.877232
C16orf86	0.799082	0.530154	0.688386	1.855587
2-Mar	0.799355	0.530458	-1.06357	0.917168
SLC20A2	0.799355	0.530464	-1.03824	1.824525
GEMIN4	0.79952	0.530639	-0.32515	4.385226
FARSB	0.799788	0.530965	-0.70337	2.369807
CD1C	0.799788	0.530983	0.993813	0.806966
PPWD1	0.799788	0.531148	0.84043	1.532843
DNALI1	0.799788	0.53116	0.656677	2.819456
LDHAL6B	0.799788	0.531234	0.861864	1.612908
SLC25A51	0.799788	0.531354	-0.55324	3.573645
ZNF81	0.799788	0.531423	0.308355	4.756436
ATF4	0.799788	0.531434	0.455407	3.377551
RGS17	0.799788	0.531546	0.891848	1.517871
USP3	0.799788	0.53156	0.985486	0.644168
PALLD	0.799788	0.53157	0.385128	3.865358
CPT2	0.799788	0.531627	-0.35433	4.659187
ZNF37A	0.799788	0.531666	0.853503	1.363123
PFN4	0.799788	0.531723	1.118919	0.560844
CTSG	0.799866	0.53184	0.845753	1.417296
SNORD121	0.800216	0.532138	-1.26236	0.730485
NIT2	0.800686	0.532515	-0.36798	4.153345
GTF3C3	0.800843	0.532703	-0.36423	4.051628
TRAF3IP1	0.800843	0.532791	-1.04977	1.340214
CC2D1A	0.800843	0.532814	0.248501	6.350207
GRK2	0.800896	0.532955	-0.26117	7.284249
PRR34	0.800896	0.533005	0.553059	2.611579
ATP5IF1	0.800896	0.533134	0.392627	3.81136
ZNF720	0.800896	0.533179	-0.53162	4.143416
SLC16A14	0.800896	0.533267	-0.6308	3.055183
EIF5A	0.800896	0.53329	-0.46498	3.870159
SMG1	0.800896	0.533304	-0.25296	8.808677
DMTF1	0.800909	0.533422	0.515072	3.455967
LRRC3	0.800909	0.533442	0.621683	2.229781
ARHGEF7	0.801458	0.533873	0.709285	2.278253
UBFD1	0.801492	0.534018	-0.25393	5.819656
CIRBP	0.801492	0.534025	-1.21436	1.034563
RPL23AP87	0.801501	0.534096	-1.22387	0.964561
SLC24A1	0.801864	0.534403	0.874858	1.330126
OSCP1	0.80243	0.534878	0.933519	1.445477
LOC10192[illegible]	0.80243	0.53491	-1.30795	0.942433
NAA35	0.802448	0.534987	-0.26019	5.688749
MPHOSPH[illegible]	0.802655	0.53519	0.257252	7.452007
LGALS2	0.802822	0.535476	0.572799	2.727855
CCDC144C[illegible]	0.802822	0.535524	0.693492	1.472499

PLEKHH3	0.802822	0.535545	-1.07235	1.377602
RNF32	0.802822	0.535567	-0.83371	1.993916
TTTY15	0.802822	0.535627	-0.51639	4.99548
BYSL	0.802862	0.535745	0.395972	3.413822
LOC101928	0.802862	0.535783	0.99629	0.961487
THSD4	0.802962	0.535936	-0.2921	4.808432
MRPL3	0.802962	0.53598	-0.2509	5.950059
CLCN5	0.803173	0.536186	-1.44392	0.503492
LEMD2	0.803203	0.536343	-0.47308	3.006158
EPC2	0.803203	0.536449	-0.29347	5.47331
ZNF263	0.803203	0.536517	0.287164	5.03202
ACTR3-AS1	0.803203	0.536524	0.702768	2.050417
GPATCH8	0.803203	0.536592	0.666549	2.390757
GNL1	0.803203	0.536597	-0.99481	1.686825
SEC61A1	0.803538	0.536885	0.224225	7.746399
SNORD71	0.803732	0.53708	0.341159	4.368449
L3MBTL4-A	0.803881	0.537274	-1.00463	1.221843
LOC101929	0.803881	0.53731	-0.93621	1.623149
PCK2	0.803939	0.53745	0.577609	1.615667
FOXC2	0.803939	0.537619	1.185124	0.950913
MYCBP2-AS	0.803939	0.537622	0.708527	2.315918
CDC123	0.803939	0.537648	-0.25053	5.589428
LOC286437	0.803939	0.537674	0.46651	3.556409
TPM3	0.804072	0.53785	0.385291	5.41936
HIST1H4E	0.804072	0.537894	0.290403	4.981614
TMEM167A	0.804229	0.538133	0.225284	7.226763
ARV1	0.804229	0.538172	0.336221	4.396417
C5	0.804229	0.538248	0.998044	0.46854
KIAA1328	0.804229	0.538259	0.520615	4.120031
AMPD3	0.804452	0.538473	-0.77574	2.049321
ORC5	0.804455	0.538587	-0.70715	2.33956
SYP	0.804455	0.538606	0.807999	1.826575
DNAJC11	0.804747	0.538866	0.239073	6.319993
RPS16	0.804798	0.539012	-0.26778	7.641366
SIN3B	0.804798	0.539031	0.515802	2.210632
THYN1	0.804861	0.539185	-1.23731	0.753649
HIST1H4L	0.804861	0.539203	-0.44335	3.681468
LOC101927	0.804884	0.539284	1.105562	0.621611
ZC3H13	0.804926	0.539416	0.24626	8.503512
INPP5B	0.804926	0.53953	0.658717	1.987474
ATP5F1E	0.804926	0.539572	0.745382	1.141438
SPCS1	0.804926	0.539573	-0.26335	5.68814
RASAL2	0.804927	0.539639	-0.36817	4.10448
BEST1	0.804942	0.539714	-1.26663	1.36026
KCNJ15	0.804983	0.539825	-0.92264	1.551055

FBXO31	0.804983	0.5401	0.621051	2.188617
PTPRE	0.804983	0.540125	-0.97773	1.525749
TUBD1	0.804983	0.540236	1.182852	0.427603
MIR4458H(	0.804983	0.540249	-0.48485	3.50056
PPM1K	0.804983	0.540303	-0.24755	5.717423
KCNC3	0.804983	0.54032	0.390878	3.946739
CEP170B	0.804983	0.540332	0.727571	1.229034
NTRK1	0.804983	0.540481	1.263633	0.767761
SLCO4A1-A	0.804983	0.540491	-0.5729	2.719646
WDR5B	0.804983	0.540509	-0.37073	4.084195
PPARG	0.804983	0.540544	0.791146	1.707974
NBEAL1	0.804983	0.540601	0.228728	7.640127
RAB3C	0.804983	0.540669	1.308238	0.54154
FAM168B	0.804983	0.54072	-0.23926	7.196749
RASSF1-AS	0.805175	0.540928	-1.06931	1.025486
LINC01697	0.805175	0.540999	0.891993	1.468161
PSPC1	0.805175	0.541044	0.27836	4.860845
CNNM1	0.805239	0.541153	0.589509	2.388862
MBL1P	0.805473	0.541391	0.978875	0.559564
YBX1	0.805473	0.54144	0.269442	5.048617
RIN1	0.805623	0.541747	0.320639	4.161273
SAMD5	0.805623	0.541842	0.357533	7.231686
CCAR2	0.805623	0.541897	-1.16167	0.712673
CDC42SE2	0.805623	0.541897	0.786125	1.14475
SHLD2P1	0.805623	0.541974	0.671753	2.184152
EXOC3L1	0.805623	0.541978	0.9121	1.047107
LOC10192(	0.805623	0.541998	-1.21702	1.400265
IGFBPL1	0.805677	0.542099	1.090855	0.764724
STEAP2	0.805705	0.542183	1.09007	0.556766
TMEM80	0.805749	0.542278	-0.61027	1.898158
AOX1	0.805749	0.542344	0.616061	2.890658
LOC10192(	0.805915	0.542521	-0.58173	2.57037
ZBTB41	0.806298	0.542851	-0.29209	5.919351
PRKRIP1	0.806298	0.542909	-1.49063	0.519332
FAM72D	0.806855	0.543349	0.739547	1.424454
MIR614	0.807095	0.543618	-1.49283	0.820162
OXNAD1	0.807095	0.543642	0.268077	5.366271
PABPC4L	0.807109	0.543717	-0.85497	2.061262
DNAJC8	0.80752	0.544111	-0.24372	6.268904
BCRP2	0.80752	0.544124	0.870331	1.00652
FPGT	0.807534	0.544229	-0.80782	1.97515
CHD7	0.807534	0.544341	-0.25885	7.267351
URB1	0.807534	0.544342	-0.26786	6.604285
SLC35F1	0.807534	0.544395	1.340498	0.938077
SNORD113	0.808047	0.544807	1.460741	0.597987

CREG2	0.808234	0.545045	1.065391	0.927921
LYL1	0.808234	0.54508	0.719906	1.795158
TMEM184B	0.808234	0.545129	0.727846	1.730337
UBXN2B	0.808357	0.545333	0.254891	5.338853
EDC4	0.808357	0.545463	0.219965	5.921048
C4orf47	0.808357	0.545471	-1.19284	1.466706
RBL2	0.808357	0.545542	-0.26227	7.403477
BEND6	0.808357	0.545608	1.121699	0.457865
HINT1	0.808357	0.54562	0.682538	0.681273
FADD	0.808357	0.545677	0.359578	4.315924
LINC00471	0.808357	0.545736	-1.03143	1.844452
STK32C	0.808698	0.546031	0.714931	1.755274
ATP1A2	0.808748	0.546131	0.506574	3.549437
ST8SIA6	0.808961	0.54634	-1.06279	2.029055
LOC101927	0.809181	0.546575	0.565342	3.087606
SPDYE7P	0.809181	0.546629	-0.44642	3.376527
ARR3	0.809181	0.546802	-0.89016	1.985488
OPA1-AS1	0.809181	0.546875	0.800615	1.848451
STX7	0.809181	0.546875	0.227097	6.646502
SLC5A4	0.809181	0.546882	0.97639	0.41473
ABCA13	0.809327	0.547076	-0.47645	4.820165
HLA-DQB1-	0.809327	0.547112	-0.90929	1.155129
LINC00982	0.809346	0.547191	-1.23845	4.025868
RPS19	0.809351	0.547334	-0.23893	8.183606
UBR3	0.809351	0.547344	-0.23999	7.302799
LRFN3	0.809351	0.54739	-0.60883	2.923946
SEMA5A-AS	0.809379	0.547475	-1.09741	1.901331
MIR3138	0.80941	0.547573	0.854906	1.487117
LZTS1-AS1	0.80941	0.547673	1.16969	1.156171
NTPCR	0.80941	0.547736	-0.34176	4.163189
COL20A1	0.80941	0.547758	-1.56189	1.737512
RUFY3	0.809582	0.54794	-0.67128	3.285977
DAPK2	0.80961	0.548024	0.443195	3.900632
SNORD95	0.80965	0.548129	0.428065	3.815821
CARMN	0.80965	0.548183	1.035859	0.673678
JHY	0.809906	0.548449	-0.74401	2.446891
USB1	0.809906	0.548504	-0.62995	2.79334
YLPM1	0.809906	0.548553	0.220202	8.356893
NFE2L3	0.810094	0.548746	0.277517	4.910721
LOC389641	0.810284	0.54894	0.729271	1.912946
ACBD6	0.810445	0.549175	0.229465	5.313809
ZNF668	0.810445	0.549181	0.667029	1.65532
SNORD17	0.81086	0.549527	0.236073	8.688116
ASAP1-IT2	0.810961	0.549662	0.737279	1.696563
SEC13	0.81099	0.549747	0.800278	1.648009

TTLL4	0.811067	0.549927	0.24578	5.100191
PSMB4	0.811067	0.54993	-0.27317	4.925876
ING5	0.811121	0.550033	0.306477	5.009895
SLX4IP	0.811171	0.550132	0.3692	3.62973
BMPR2	0.811219	0.550231	0.232825	7.806641
PWARSN	0.811417	0.550501	-0.56165	3.234219
MIR1304	0.811417	0.55051	-1.24486	1.372836
RIPK1	0.811417	0.550562	0.726895	1.563456
SDK2	0.811488	0.55071	-0.45191	4.153012
TCEA3	0.811488	0.550742	0.254765	6.796295
SIK2	0.811494	0.550811	-0.27225	6.182356
SPRY4-AS1	0.812068	0.551267	1.052135	0.704346
EFNA1	0.81221	0.551447	0.83592	1.732741
ACSL1	0.81221	0.551533	-0.5422	2.784017
USP49	0.81221	0.551561	-0.3687	4.212734
CCDC141	0.812532	0.551846	0.650271	2.569156
PNO1	0.812532	0.551911	-0.41007	4.036111
PRPF18	0.812548	0.552015	-0.24826	5.489123
MNAT1	0.812548	0.552053	-1.55973	1.325673
SOGA1	0.812888	0.55235	-0.25098	5.704776
FRMPD3	0.813209	0.552635	0.962713	1.025544
COA1	0.813312	0.55277	0.300696	4.177455
GABRR2	0.813368	0.552894	-1.40629	0.871908
KBTBD13	0.813368	0.55294	-1.38519	0.890061
MNS1	0.813598	0.553162	0.337964	3.771922
FLCN	0.813768	0.553346	0.287351	4.555001
EFNB1	0.813768	0.553468	0.261487	5.874122
CMAHP	0.813768	0.553528	0.379375	3.626131
GGT8P	0.813768	0.5537	0.422948	3.699225
MAFIP	0.813768	0.553712	-0.41219	4.7476
SLC35A5	0.813768	0.553769	0.326901	4.64829
TTC9C	0.813768	0.553803	-0.59082	3.18124
SGPP1	0.813768	0.553831	-0.40107	3.479944
C2orf16	0.813768	0.553914	-0.92091	1.720498
CTD-2201I	0.813768	0.554057	-0.77166	1.850497
LOC389831	0.813768	0.554076	0.694842	2.625237
DNASE1L1	0.813768	0.554106	-0.68922	2.815517
PPP1R12A	0.813768	0.554169	0.53461	1.802636
MPZL1	0.813768	0.554201	-0.71229	1.70945
HERC2	0.813862	0.554447	0.230172	6.398799
NTS	0.813862	0.554506	-1.36459	1.125549
LOC10192	0.813862	0.554515	0.839933	1.462118
FLT1	0.813862	0.554668	0.318514	4.534813
GREM2	0.813862	0.554715	0.694075	3.79505
LINC00663	0.813862	0.554718	0.887459	1.20227

HADHA	0.813862	0.554726	-0.2346	7.971245
AGAP12P	0.813871	0.554808	0.278294	4.60429
C2CD4D-AS	0.813871	0.554882	0.759139	1.774776
SV2A	0.813871	0.554945	0.904348	1.323231
RALGAPA1	0.813871	0.555032	-0.23528	6.489887
PPIB	0.813871	0.555106	0.224974	6.779413
GTF2E2	0.813871	0.555194	-0.28461	5.953947
TMEM102	0.813871	0.555194	-0.40207	3.269661
VSIG10	0.814251	0.555519	-0.23958	5.754495
RHOH	0.814287	0.555609	0.780794	1.471456
LOC10254(	0.814429	0.555773	-1.02385	1.792555
ELF1	0.814442	0.555847	0.935388	1.008341
CCDC142	0.814455	0.555922	0.35869	4.144231
TM2D1	0.81446	0.555991	-0.27054	5.321675
ZBTB16	0.81487	0.556337	0.599666	3.29385
C9orf85	0.814891	0.556479	0.346897	4.137534
OVCH1	0.814891	0.556484	0.959014	1.405123
SUSD2	0.815058	0.556663	0.462487	2.707353
RNF219	0.815261	0.556868	0.361689	4.239165
SMAD1	0.815318	0.556974	0.750613	0.874465
LINC01948	0.815443	0.557124	1.105561	0.784138
ZNF564	0.815506	0.557234	0.306314	4.509473
ZNF799	0.815596	0.557361	0.638996	1.688029
TBCC	0.815651	0.557504	-0.38847	3.820036
POLA1	0.815651	0.557645	-0.31961	5.302743
MRTFB	0.815651	0.557655	1.038	0.578467
SNORA12	0.815651	0.557717	-0.25053	5.834409
EMC4	0.815651	0.557729	-0.65925	2.155693
NARS	0.816189	0.558163	-0.22775	7.98563
MN1	0.816298	0.558304	-0.49363	4.324954
SPIRE1	0.816348	0.558536	-1.55524	1.186322
LINC00638	0.816348	0.558574	1.02531	0.422316
IL20	0.816348	0.558597	1.194238	0.647347
SNORA41	0.816348	0.558666	0.459429	2.384802
MIR181A2	0.816348	0.558699	0.991322	1.276043
HMGN3	0.816348	0.558735	-1.30331	1.209598
TSSK3	0.81667	0.559022	0.528156	3.162092
CNOT7	0.816776	0.55916	-0.26786	5.206529
ASNSD1	0.817004	0.559445	-0.29704	4.799734
EVL	0.817004	0.559448	-0.2847	5.280701
SART1	0.817472	0.559835	0.240711	6.838286
MIER2	0.817483	0.559909	-0.32248	5.053749
GNB4	0.818033	0.560352	-0.28347	5.846749
CDK17	0.818063	0.560439	0.805857	1.354431
C6orf106	0.818435	0.56076	0.433343	2.990115

TTC7A	0.818628	0.560985	0.865878	1.509883
ROR1-AS1	0.818628	0.561025	0.744886	1.878199
ERVH48-1	0.818767	0.561186	0.444803	2.616105
SPG7	0.818958	0.561384	0.252148	5.252785
TSSC4	0.819003	0.561529	0.724136	1.348001
RNF180	0.819003	0.561547	0.881135	1.706482
PDCL3P4	0.819104	0.561683	0.463623	2.418589
LOC100289	0.819144	0.561876	0.504892	2.737511
ACOT11	0.819144	0.561905	-0.71554	2.41173
CSAD	0.819144	0.561909	-0.57651	2.721856
LOC101929	0.819366	0.562139	0.376494	3.372866
MCM3AP	0.819366	0.562212	0.209682	7.397367
CCNC	0.819366	0.56226	0.424507	3.016844
STARD7	0.819375	0.562333	0.222507	6.937433
WDR60	0.819425	0.562481	-0.23842	6.588679
TOMM22	0.819425	0.5625	-0.2665	5.878202
SF3B4	0.819427	0.562632	-0.25877	5.424227
AMH	0.819427	0.562635	0.623394	1.619262
ZNF284	0.819701	0.563031	-0.41656	3.495165
SRSF9	0.819701	0.563063	-0.26983	6.255814
PDCL	0.819701	0.563081	-0.25348	5.017231
COA7	0.819701	0.563105	0.345946	3.759505
ZNF600	0.819701	0.563229	0.328892	4.254097
ALKAL2	0.819701	0.563249	0.557215	2.207109
C9orf41-AS	0.819701	0.563326	0.402315	3.190377
NCOR1	0.819701	0.563386	0.294703	4.412218
LOC285819	0.819701	0.56342	0.956958	0.452446
RPL3	0.8198	0.563556	0.271956	5.210194
ST14	0.8198	0.563621	0.237192	8.165725
MTREX	0.81981	0.563694	-0.23248	6.950112
CAPRIN1	0.820387	0.564241	-0.29669	6.186206
BET1	0.820387	0.564267	0.756629	0.994933
ZNF335	0.820387	0.564344	-0.22335	5.76186
DFFA	0.820387	0.564388	0.467505	2.524994
RECQL5	0.820387	0.564423	-0.25545	5.707204
USP46	0.820581	0.564674	-1.46664	0.325845
SNX29P1	0.820581	0.564689	-0.97832	1.571671
SUMO4	0.820764	0.564933	0.623726	2.150977
MLIP	0.820764	0.564948	0.912422	0.964876
THOC6	0.820786	0.565088	0.850408	1.193647
ZNF572	0.820786	0.565135	0.739783	2.109072
DDX23	0.820786	0.565206	0.214417	7.359172
MCPH1	0.820786	0.56531	-0.62213	2.044527
AP3D1	0.820786	0.565374	-0.77229	2.668223
DAB1	0.820786	0.565402	-1.91877	0.518309

PAPLN	0.820786	0.565429	0.294747	4.966134
TMEM238	0.820894	0.565606	0.346025	3.616765
IFT57	0.820894	0.56568	0.233503	5.457752
CREBZF	0.820894	0.565719	-0.52883	3.841888
RPS6KA2-A	0.820894	0.565809	0.880486	1.132499
RPS6KA5	0.820894	0.565836	-0.61607	2.81223
RP9	0.82102	0.565989	0.476885	3.030896
ZDHHC8	0.821161	0.566209	-0.47419	3.316839
NKAIN1	0.821161	0.566219	-1.3402	1.233244
LOC10027(	0.821178	0.566316	-1.37173	0.766696
GRIP2	0.821178	0.56646	-0.68014	3.647059
CCNH	0.821178	0.566488	1.004913	0.791227
COL18A1-A	0.821178	0.566497	0.608874	2.261603
ABHD17B	0.821526	0.566803	-0.62497	2.997913
TTLL11	0.822277	0.567402	0.731933	1.655932
MIR7854	0.822277	0.567455	-1.0188	1.505247
MIR4441	0.822341	0.567589	-0.92093	1.592183
PRDX4	0.822341	0.567633	0.290605	5.082756
CMC1	0.822341	0.567751	0.536237	2.726451
KAT6B	0.822341	0.567766	-0.53048	3.245514
TBK1	0.822651	0.5681	-0.24227	5.827969
IL27RA	0.822651	0.568113	0.442802	2.767783
LPCAT3	0.822893	0.568359	0.247497	5.455638
HSD17B7	0.822893	0.568414	-0.81945	2.192291
LRCH4	0.823142	0.568652	-0.34674	3.588524
HEIH	0.82323	0.56878	-0.46916	3.787033
SLC1A4	0.823399	0.569069	0.49995	1.827691
DNM1P35	0.823399	0.569087	-0.89919	1.039151
ASTN2	0.823399	0.569133	-0.81313	2.23881
HERC3	0.823399	0.569227	-0.38196	3.860359
COMMD2	0.823399	0.56923	-0.30262	4.873535
SNORD29	0.823554	0.56942	0.541623	1.714465
PWWP2B	0.823554	0.56947	0.604083	1.84669
NSUN6	0.823596	0.569632	0.278169	4.547964
MIR548AR	0.823596	0.569633	0.711006	1.379348
LOC101929	0.824545	0.570356	1.08298	0.655356
TBC1D23	0.824645	0.570503	-1.30748	0.714532
COL28A1	0.824645	0.57062	0.332363	4.829391
TMEM41B	0.824645	0.570692	-0.23282	5.787882
JOSD1	0.824645	0.570752	0.247899	6.104208
ADGRE5	0.824645	0.570821	0.896203	1.086191
ITGA6	0.824645	0.571014	0.835768	0.955086
DUSP6	0.824645	0.571016	0.573085	2.138661
MIR3619	0.824645	0.571032	0.647905	1.550561
TWF1	0.824645	0.57109	-1.17948	0.635864

LINC00999	0.824645	0.571093	-0.58061	3.282109
NDUFA6-D	0.824892	0.571371	0.8167	0.871195
PHF5A	0.824892	0.571397	-0.3361	3.927165
WFDC12	0.825049	0.571574	-1.24439	1.118362
ATG9B	0.825154	0.571758	-0.90591	1.588237
FBH1	0.825154	0.571779	0.347855	3.35739
VAC14	0.825513	0.572096	-0.24282	5.1233
SNORA62	0.825688	0.572284	0.285333	8.033782
S1PR3	0.82603	0.572587	0.556963	2.970749
HACD4	0.826146	0.572757	0.437534	2.439461
MEG3	0.826146	0.572802	0.405413	6.257228
SLC6A8	0.826213	0.572978	-0.80562	2.38982
TAS2R31	0.826213	0.572982	0.557975	2.63895
ALDOA	0.826218	0.573052	0.539089	2.361156
FARSA	0.826376	0.573229	-0.2743	5.286088
SNORD38B	0.826521	0.573492	-0.48045	2.774269
MUL1	0.826521	0.573498	-0.38583	4.314156
TNS1	0.826521	0.573553	0.794617	1.260089
TBC1D20	0.826521	0.573716	-0.26551	4.820365
LOC101927	0.826521	0.573728	1.078307	1.048728
CASP3	0.826521	0.573752	-1.09824	1.033848
RFC3	0.826521	0.573829	0.41332	2.737732
MACC1	0.826521	0.574009	-0.23556	7.653867
ERBIN	0.826521	0.574053	-0.77704	2.041195
RNF215	0.826521	0.574055	0.475552	2.230423
TFR2	0.826521	0.574066	-2.15778	0.54439
TSSK4	0.826618	0.574233	0.701453	1.194003
CHERP	0.826618	0.574309	0.250229	5.410823
MIR4435-2	0.826618	0.574334	-0.70939	2.830463
LIMA1	0.826847	0.57456	-0.8211	1.634585
LOC101929	0.82734	0.574972	0.830027	1.656697
MIR8085	0.82734	0.575132	-1.17882	0.636404
TMEM190	0.82734	0.57515	-0.9392	0.643405
TBC1D8	0.82734	0.575171	-0.24356	6.753816
MMP27	0.828118	0.575779	-1.22914	1.757178
OSBP	0.828293	0.576032	0.214094	7.41864
MCTS1	0.828293	0.57623	0.610598	1.821642
ETFA	0.828293	0.576274	0.718196	0.762287
SPTBN4	0.828293	0.576307	-0.95213	1.778203
LINC01000	0.828293	0.576339	0.22026	8.186582
OTUD7B	0.828293	0.576343	0.219183	6.593178
ANP32A	0.828293	0.57637	0.257079	6.981868
LINC01473	0.828416	0.576523	-0.82698	1.693869
RCC1L	0.828608	0.576724	0.436747	3.028317
CYP2R1	0.828676	0.576838	0.352323	3.423685

COQ9	0.828777	0.577021	-0.23317	5.460822
ATXN10	0.828777	0.577042	0.66933	1.378888
CCDC144B	0.82895	0.57728	0.296083	6.351954
ASF1A	0.82895	0.577297	-0.29081	4.472769
NCF1	0.829296	0.577771	0.758194	1.559185
MTBP	0.829296	0.577851	-0.54642	3.082248
RBBP7	0.829296	0.577852	0.273839	4.623985
NDRG2	0.829296	0.577874	-1.00225	1.333863
SLC15A1	0.829296	0.577874	0.34625	3.826245
TMED9	0.829974	0.578414	-0.24148	6.956387
DPCD	0.830064	0.578544	-0.51891	3.624854
LOC283177	0.830121	0.578651	0.956708	1.651483
FBXL22	0.830162	0.578747	0.643279	1.655217
SF3B1	0.830299	0.578954	-0.22127	8.978517
C2CD4D	0.830299	0.578977	-1.03952	1.042648
CACNG8	0.830334	0.579069	0.916062	0.804009
BAIAP2L1	0.830334	0.579209	-0.23739	7.18661
NCAPG2	0.830334	0.579266	-0.81338	1.515314
ZNF717	0.830334	0.57927	0.75525	1.305737
PDK3	0.830526	0.579539	0.206486	5.831397
ADAMTS8	0.830526	0.579568	-1.16927	1.506683
ZNF45	0.830526	0.579605	-0.36981	4.125262
METTL6	0.830589	0.579834	0.718167	1.091687
YIPF2	0.830589	0.579904	0.228627	5.160497
SETD2	0.830589	0.57994	-0.21852	8.438701
GINS4	0.830589	0.579945	0.383394	3.0804
PAN3	0.830589	0.579993	-0.22248	6.870988
PGAM1P5	0.830589	0.580067	0.78565	1.519614
MTG1	0.830589	0.580121	0.250233	5.224598
TP73-AS1	0.830915	0.580421	0.45237	2.585263
METTL22	0.830915	0.580541	-0.47606	3.71933
LOC101928	0.830915	0.58055	0.793747	1.679295
SBNO2	0.831035	0.580727	0.663488	1.589399
LINC00654	0.831035	0.580769	0.749574	1.882948
DDX58	0.831044	0.580842	-0.25641	5.599067
PML	0.831357	0.581128	-0.45915	4.06067
ANKRD19P	0.831772	0.581486	-0.61945	1.918539
GPR153	0.831856	0.581612	-0.40127	4.893045
AGL	0.832073	0.581831	-0.60849	1.896047
FBXW11	0.832095	0.581914	0.798223	1.210332
NDUFAB1	0.832158	0.582093	-0.27117	4.818365
VEGFC	0.832158	0.582097	0.489877	2.281018
ZFP69B	0.832158	0.582207	0.573195	2.593777
ZSCAN9	0.832158	0.582227	0.811644	0.918101
ANKHD1-EI	0.83222	0.582344	-1.31033	0.526984

PRSS53	0.83222	0.582406	0.591816	1.660516
SPDL1	0.832323	0.582545	-0.30305	4.271722
MAP9	0.83233	0.582639	0.469864	3.411763
MTUS1	0.83233	0.582685	0.415479	3.386742
CUL9	0.83248	0.582857	-0.22769	6.618892
DGUOK	0.832616	0.58302	-1.01782	1.837518
CLN8	0.832631	0.58311	-0.56497	4.36898
DDX31	0.832631	0.583254	-0.34282	4.111164
ALKBH3	0.832631	0.583327	-0.29426	4.170407
DBP	0.832631	0.583424	-0.4074	3.555902
CYB5R4	0.832631	0.58349	-0.283	4.968883
LOC728485	0.832631	0.583493	-2.19178	0.689663
ACOT7	0.832631	0.583528	0.723716	1.615572
PRKCZ	0.832631	0.58357	-0.42824	3.085983
PPP1R26-A	0.832863	0.5838	0.635034	2.241403
SNORD116	0.833131	0.584056	-0.84025	4.780067
CBWD2	0.83318	0.584157	-0.26029	5.040834
DELE1	0.833377	0.584389	0.508037	2.97755
MORF4L1	0.833377	0.584431	0.719821	1.789211
ACOX3	0.833385	0.58459	0.635956	1.512474
ELAVL1	0.833385	0.584756	0.222664	5.687902
MTMR4	0.833385	0.584776	0.204827	6.30928
GGA1	0.833385	0.584819	-0.72787	1.563782
RABGGTA	0.833385	0.584933	0.698022	0.700871
ERI2	0.833385	0.584958	0.336732	4.159681
LOC102723	0.833385	0.585018	1.140263	0.90101
NDUFAF8	0.833385	0.585114	-0.31396	4.282701
SLC36A2	0.833385	0.585151	0.957722	1.050491
MUC19	0.833385	0.585246	0.861024	1.339788
PLPP6	0.833385	0.585258	0.262015	4.75138
ANP32AP1	0.833385	0.585274	0.692273	0.826048
PLEKHG4	0.833385	0.585313	-0.99498	1.295678
TACR2	0.833952	0.585909	0.55257	2.369897
ABHD3	0.833952	0.585919	-0.54764	2.784953
STARD13	0.833952	0.585935	0.661312	2.14456
COL21A1	0.833952	0.585982	0.478104	2.834383
PCID2	0.834037	0.586151	-1.13787	0.697537
AP4M1	0.834037	0.586238	0.373197	3.547324
IQCJ-SCHIP	0.834037	0.586245	0.85098	0.801856
SNORD45B	0.834212	0.586482	-0.31648	4.572255
AFMID	0.834212	0.586572	0.57699	1.970305
LINC00115	0.834212	0.586618	0.343819	3.191127
SNORD50B	0.834212	0.586699	0.678046	1.511134
TYRO3P	0.834212	0.586762	0.625966	1.885453
POLR2H	0.834212	0.586773	0.659691	1.423903

ZNF318	0.834449	0.587007	0.222201	6.298817
TMEM168	0.834545	0.587142	-0.8473	1.160168
BBS4	0.834593	0.587307	-1.01203	0.956785
FEN1	0.834593	0.587311	-0.35319	3.945275
MYO1D	0.834608	0.587453	-0.24723	6.204973
PI4K2B	0.834608	0.587457	-0.22431	5.89729
ACKR4	0.834654	0.587557	0.725288	0.898995
LSM11	0.834729	0.587705	0.369891	3.951167
TCEA2	0.834729	0.587745	0.621588	2.426371
LINC00513	0.834795	0.587984	0.356313	4.060167
ZNF696	0.834795	0.588007	-0.48151	2.975217
ZGRF1	0.834795	0.588037	-0.28618	4.800012
MRPL28	0.834795	0.588062	-0.36973	4.209559
LINC01132	0.834968	0.588251	0.824491	0.477106
NAB1	0.834982	0.588329	-0.24273	7.418787
LINC01001	0.835274	0.588602	-0.71485	2.120752
GINS2	0.835331	0.588771	-0.64896	2.159726
SAT2	0.835331	0.588778	0.235533	4.610675
BACH1-IT2	0.835634	0.589059	-0.84595	2.038229
NACC1	0.835904	0.589317	-0.2297	6.076394
LYST	0.836071	0.589503	0.424514	3.450176
UBE2QL1	0.836487	0.589907	-1.08042	1.192153
ST7-OT4	0.836487	0.590009	0.41556	2.712522
ABHD11-AS	0.836487	0.590049	0.828804	1.769457
COG3	0.836487	0.590173	-0.24371	6.353091
PMS2CL	0.836487	0.590174	-0.39624	3.871531
CIAO2A	0.836487	0.590269	0.376192	3.592744
TMEM225	0.836487	0.59027	0.965543	0.911681
GABARAPL	0.836845	0.590642	-0.31025	5.513657
CCDC34	0.836845	0.590659	0.401361	3.256203
FZD2	0.836866	0.590756	0.935864	0.527271
LUC7L2	0.836866	0.590809	-0.44295	4.273162
RSAD1	0.837163	0.591092	0.489408	1.950376
HP1BP3	0.837163	0.591154	-0.21302	7.841569
YPEL2	0.837325	0.591336	0.204583	5.98298
TFAP4	0.83741	0.591482	0.348956	4.157086
BSN	0.83741	0.591532	-0.55502	2.511175
TMEM35A	0.837429	0.591613	-1.32875	0.630027
PVT1	0.837451	0.591696	-0.31743	4.526737
MCM8-AS1	0.837829	0.592031	-1.24696	0.487731
ANKRD13B	0.837847	0.592112	0.411728	3.007503
LPCAT2	0.838131	0.592381	-0.29576	5.253993
MTDH	0.838227	0.592607	-0.21398	8.185985
TIRAP	0.838227	0.592612	0.464663	3.035595
ATF7	0.838227	0.592657	0.582765	1.500283

UGT1A5	0.838227	0.59272	0.524682	2.489115
TFDP2	0.838362	0.592883	0.728071	1.168346
SNORD20	0.838556	0.593135	0.405383	3.024361
ABALON	0.838556	0.593156	0.707037	1.256722
MYL5	0.838609	0.593271	-0.48615	2.685098
PPHLN1	0.838609	0.59333	0.828296	1.445877
EMC7	0.838644	0.593422	-0.22638	5.830972
WDR83OS	0.838694	0.593526	-0.53383	3.112466
DDX20	0.838822	0.593684	-0.33224	4.150729
MYH16	0.838988	0.593871	0.723354	0.693826
MSRB1	0.838988	0.593938	-0.49204	3.554937
CHUK	0.839229	0.594308	0.222333	5.10134
TCERG1L	0.839229	0.594324	-1.07834	0.955242
TRAIP	0.839229	0.594344	-0.6913	1.874409
CCDC137	0.839229	0.59438	-0.26802	4.925572
DPP7	0.839677	0.594766	-0.26887	5.099264
USO1	0.839814	0.595033	0.564516	2.408203
TMEM91	0.839814	0.59505	0.746569	1.804751
SNRNP25	0.839814	0.595067	-0.55528	3.345764
MCF2L-AS1	0.839863	0.59517	-1.20461	0.933147
CXCR6	0.839936	0.595289	-0.51993	3.795163
LOC101928	0.839977	0.595386	0.472531	1.58299
NUP43	0.839984	0.595503	0.401252	3.289641
FBXO3	0.839984	0.595621	-0.33853	5.024345
FRMD4A	0.839984	0.595653	-0.24939	6.263055
ERICH5	0.839984	0.595663	0.669599	1.835463
NFRKB	0.840021	0.595758	0.583649	1.905774
FTO-IT1	0.840044	0.5959	-0.94349	0.805285
CCDC168	0.840044	0.595984	0.804159	1.607998
FYCO1	0.840044	0.596014	-0.22244	7.319167
WDR34	0.840044	0.596085	0.244697	4.643511
SPDYE8P	0.840044	0.596114	-0.69698	1.812169
LOC100287	0.840044	0.596182	-1.03607	1.157025
TXNDC15	0.840107	0.596295	0.214667	5.55102
CRYBB3	0.840531	0.596664	-0.68577	1.377288
ERVK13-1	0.841185	0.597196	-0.24769	6.233265
LOC101927	0.841556	0.597528	-0.85419	1.757414
ITGB5	0.84158	0.597613	-0.22196	6.986436
SAMD12	0.841847	0.59798	-0.5661	2.772976
CDK6	0.841847	0.598054	-1.35583	0.951189
SLC30A4	0.841847	0.598161	-0.29966	4.968041
AKR1C1	0.841847	0.5982	-0.35243	5.071135
TNKS	0.841847	0.598339	-0.20963	6.861143
GNPTAB	0.841847	0.598345	-0.21333	6.89904
SP2-AS1	0.841847	0.598386	0.700577	1.183621

OGDH	0.841847	0.598387	0.753647	0.875899
PPP2R5B	0.841847	0.598451	0.277744	4.209848
GLUD1P3	0.841847	0.598485	0.53437	1.738942
POM121L8	0.841854	0.598558	0.702897	1.5057
ARHGAP39	0.841886	0.598649	-0.86206	1.766761
C20orf194	0.841976	0.598781	-0.25881	5.935305
FBXL4	0.842264	0.599146	0.541733	2.728414
NSDHL	0.842264	0.599187	-1.13662	0.611173
CCDC91	0.842264	0.599248	-0.19759	5.844236
MIR3128	0.842264	0.599295	-1.09467	0.745726
APC	0.842264	0.599388	-0.6861	2.641306
GCA	0.842264	0.59946	0.447581	3.364762
LOC44115!	0.842264	0.599463	-0.45656	2.573167
ZNF558	0.842324	0.599611	0.550676	2.527966
WDR88	0.842324	0.599642	-1.05027	1.159652
NCOA6	0.842585	0.599896	-0.23834	6.193332
SNORA14B	0.84273	0.600068	-0.28024	6.78576
SF3B6	0.842742	0.600145	-0.33596	5.927863
GCSHP3	0.84275	0.600264	-0.42113	3.417571
RNY3	0.84275	0.6003	-0.29023	8.930188
PRPS1	0.84275	0.600384	-0.84323	1.70567
MAN2A1	0.84275	0.600424	0.200949	7.034112
SENP3	0.842778	0.600512	-0.54183	2.428854
NUCB1	0.842859	0.600638	0.197519	7.113087
TRIP4	0.842891	0.600737	-0.24858	5.37933
DIPK2A	0.842891	0.600837	-0.60099	2.229552
SCNN1A	0.842891	0.600909	-0.22109	6.545235
ICAM5	0.842891	0.600968	0.666073	1.975166
TSTD3	0.842891	0.601057	0.329487	3.855404
TWIST2	0.842891	0.60107	0.578188	2.27598
PABPC4-AS	0.842938	0.601172	0.402356	2.141271
SPOCK1	0.84297	0.601313	0.522147	2.493301
HRAT5	0.84297	0.601332	-1.51294	1.404778
PDE6A	0.843119	0.601511	-0.48034	2.553686
FAM76B	0.843119	0.601574	0.24276	5.047295
SLC15A4	0.843166	0.601745	-0.26605	4.434422
NUDT15	0.843166	0.601781	0.325153	3.999496
TIMM10B	0.843166	0.601813	-0.21357	5.772349
LOC14547	0.843239	0.601933	0.465963	2.442653
LIG3	0.843455	0.602156	0.47151	2.539793
STOM	0.843627	0.602347	-0.98799	0.571356
SDCBP2	0.843682	0.602513	-0.5277	2.373123
ROBO3	0.843682	0.602523	-0.55444	2.512941
LPCAT4	0.843784	0.60276	0.198969	6.417442
REEP3	0.843784	0.60277	-0.22712	6.350017

SNPH	0.843784	0.602839	0.389465	3.795773
GUCY2D	0.843784	0.602869	0.604426	1.718872
TMEM104	0.844035	0.603205	0.224428	4.664139
RSPH6A	0.844035	0.603227	0.921264	0.716476
QPCT	0.844035	0.603283	0.74357	1.293356
DOCK4-AS1	0.844035	0.603321	-0.5997	3.056859
GPR21	0.844135	0.603558	-0.56717	3.03475
LYG2	0.844135	0.603572	0.994099	0.468183
ACAT1	0.844135	0.603598	-0.29916	5.323454
PIAS2	0.844152	0.603688	-0.30288	5.093298
NEXMIF	0.844152	0.603747	-1.45678	1.31098
PPP1R37	0.844342	0.604004	0.231707	5.273501
SNORD144	0.844342	0.60402	-0.926	1.213899
CENPT	0.844405	0.604133	-0.21894	5.465367
KLRA1P	0.844519	0.604283	0.670254	1.825789
ADAM11	0.844547	0.604372	0.748862	0.823687
GLCE	0.844756	0.604589	0.217648	5.922088
FAM27B	0.844811	0.604698	0.573763	2.097836
ITIH2	0.844851	0.604794	0.933124	0.585021
HIST3H2A	0.845247	0.605147	-0.61665	1.541021
KBTBD2	0.845267	0.60523	-0.20857	5.879777
C15orf41	0.845273	0.605342	-0.80579	1.499372
MIR4677	0.845273	0.605371	-1.43288	0.690116
ENTHD1	0.845286	0.605449	0.719521	1.130788
TMEM132E	0.845316	0.605538	-0.9783	0.452787
SCARNA3	0.845749	0.605917	-0.41763	2.951133
RORA	0.845841	0.606051	-0.57447	3.312007
CD3G	0.845853	0.606168	-0.73148	1.924703
PSMD8	0.845853	0.606275	-0.21342	6.649383
LOC100129	0.845853	0.606359	-1.05807	1.291646
MIR26A2	0.845853	0.606383	-1.4482	1.142141
OGFOD2	0.845853	0.606419	0.779187	1.015361
EFCAB5	0.845853	0.606471	-0.91098	1.123096
RANBP2	0.845853	0.606539	-0.21722	8.86785
RBP5	0.846186	0.606847	-0.39905	3.437322
RHEB	0.846251	0.606969	-0.44651	4.29282
HIST1H1E	0.846251	0.607176	-0.22418	6.736596
GPR171	0.846251	0.607211	0.795663	1.177628
ACO2	0.846251	0.607223	0.234456	6.439251
HMGA2	0.846251	0.607236	-0.58128	2.534022
KDM4B	0.846385	0.607401	-0.20787	6.620572
TOP2B	0.846515	0.607603	-0.20614	8.196392
C9orf72	0.846515	0.607631	0.539078	2.546645
AATF	0.846526	0.607729	-0.48533	2.69012
APOLD1	0.846526	0.607776	0.701622	0.591446

UROS	0.846589	0.607957	0.25212	4.091271
VWCE	0.846589	0.607958	0.507205	1.981895
MED23	0.84677	0.608204	-0.64882	2.534977
TRDMT1	0.84677	0.608344	0.31414	3.541936
P3H2-AS1	0.84677	0.608402	-1.29718	0.868412
UBOX5-AS1	0.84677	0.608408	-0.66106	1.829366
RAB30	0.84677	0.608483	0.736725	1.042623
LOC100506	0.84677	0.6085	0.601048	1.137555
IL17RB	0.847006	0.608739	0.435364	3.191016
VRK3	0.847082	0.608862	0.731439	0.73557
VPS18	0.847307	0.609092	0.24591	4.739961
MED13L	0.84744	0.609256	-0.19971	8.388363
ACYP2	0.847586	0.60943	-0.81159	1.504479
MRTFA-AS1	0.847676	0.609598	-0.46784	3.956139
SH3GL1P2	0.847676	0.609665	-0.62151	2.191678
SNORA44	0.847676	0.609701	-0.28966	6.637264
LOC101928	0.847707	0.609792	-1.30246	0.517704
SAPCD2	0.848134	0.610167	-0.30291	4.12362
E2F4	0.848139	0.610297	-0.20391	5.865145
SLC52A3	0.848139	0.610324	-0.43271	2.720757
ALKBH5	0.848139	0.610377	0.183887	6.406464
SNORD127	0.848393	0.610628	-0.76463	1.365401
CKMT1B	0.848431	0.610724	0.307454	4.263978
CYP17A1	0.848486	0.610883	0.903649	0.935649
OAS3	0.848486	0.610902	-0.20959	6.209277
SYNGAP1	0.84853	0.611002	0.329628	3.723363
DHX34	0.848618	0.611134	-0.24753	4.662414
NPHP3	0.848627	0.611209	-0.37234	3.488985
ANKRD23	0.849106	0.611624	0.421287	2.926911
ZNF517	0.849192	0.611754	-0.68833	1.381797
POLE	0.849226	0.611952	0.195019	6.55753
TADA2B	0.849226	0.611968	-0.19979	5.574387
STAG3L5P	0.849226	0.611985	0.505687	2.558296
RAD1	0.84937	0.612299	0.867275	0.38396
C6orf47	0.84937	0.612309	0.596723	0.756526
HSPA8	0.84937	0.612341	0.190923	8.031768
FAHD2CP	0.84937	0.612364	-1.28227	0.579376
SNORD63	0.849472	0.612506	-0.3859	3.534702
ANKRD12	0.849502	0.612627	-0.65379	2.110152
TMEM186	0.849502	0.612768	0.39435	3.369156
ZNF141	0.849502	0.61278	-0.32456	4.413827
CLCA3P	0.849502	0.612927	0.521522	2.489538
CUX1	0.849502	0.613001	0.554784	1.73246
PRKAB1	0.849502	0.613048	0.214818	5.812286
BAG4	0.849502	0.613069	0.623099	1.295499

PFKFB3	0.849502	0.613078	0.605246	1.179539
WEE2-AS1	0.849513	0.613204	0.783691	1.086442
PROSER3	0.849513	0.613224	0.264677	4.144592
ST18	0.849689	0.61353	0.549553	2.337415
CDK9	0.849689	0.613577	-0.23932	5.743654
ZNF833P	0.849689	0.613623	-1.18353	0.639167
TM9SF1	0.849689	0.61365	-1.72883	0.971937
TTC6	0.849689	0.613784	0.371931	4.326723
SCN2B	0.849689	0.613828	-0.8432	0.718309
SLC7A5P2	0.849689	0.613854	-0.24619	5.378372
PSCA	0.849689	0.613962	-0.73584	2.095386
NRF1	0.849689	0.614022	0.809997	0.598597
SETD5	0.849689	0.614159	-0.49379	2.984297
EXOC2	0.849689	0.614164	0.741553	0.968512
LOC101928	0.849689	0.61425	1.012657	0.366609
SNORD15A	0.849689	0.61425	-0.23446	7.795564
GRB10	0.849689	0.614314	-1.08316	1.123198
PMS2P7	0.849746	0.614502	-0.6119	2.002489
RNF26	0.849746	0.614518	-0.26865	4.643363
PTPN2	0.849746	0.614719	0.48889	2.114972
RPS4Y1	0.849746	0.614753	-0.46805	7.300967
STK39	0.849746	0.614779	-0.20796	7.576049
SNORD3D	0.849746	0.614791	-0.29799	4.897299
LLPH	0.849746	0.614837	0.259976	4.779945
RABL3	0.850878	0.615858	0.262876	5.089178
CREBRF	0.850878	0.615894	0.265934	5.053592
FAM171A2	0.850878	0.616004	-0.78321	1.530128
LOC100128	0.850878	0.616033	0.467412	2.357759
EIPR1	0.850878	0.616036	0.27162	4.172638
SUMO1P3	0.850878	0.61607	0.292508	3.670422
KLF7	0.850892	0.616157	-0.69991	2.352523
HDHD3	0.850892	0.616218	0.437029	2.173131
LOC91450	0.851026	0.616384	0.64138	0.911941
CNOT3	0.851144	0.616558	0.32948	3.461102
LINC00112	0.851144	0.616607	0.727214	0.829367
RANBP17	0.851365	0.616836	0.46977	3.27736
ZNF547	0.851439	0.617028	0.69374	1.649485
WRAP73	0.851439	0.617064	-0.42149	3.267041
C11orf95	0.851439	0.617097	0.270261	4.119042
NUMBL	0.851522	0.617232	0.683807	1.154293
ABL1	0.851522	0.617333	0.353	3.211846
MKRN2OS	0.851522	0.617532	0.315159	3.263664
FEM1C	0.851522	0.617568	-0.22643	6.661098
RAB6A	0.851522	0.617582	-0.56047	1.778548
MPP5	0.851522	0.617612	-0.75849	1.811148

CREBL2	0.851522	0.617705	0.224179	5.539944
ZMIZ2	0.851522	0.617735	0.558111	1.303688
CCT6P1	0.851522	0.617778	-0.3437	3.47428
ATN1	0.851576	0.617908	0.659264	0.679526
RALY	0.851576	0.61796	0.696733	1.844167
DDX5	0.851576	0.618024	-0.20338	9.950071
C1GALT1	0.851684	0.618171	-0.22174	6.689027
TLNRD1	0.85171	0.618259	0.224575	5.011473
RFX7	0.851775	0.618406	-0.23965	6.457228
PCF11	0.851775	0.618505	-0.20508	7.374926
NSRP1	0.851775	0.618695	-0.86224	1.693113
PRDM11	0.851775	0.618719	-0.49218	3.130416
SFXN5	0.851775	0.618734	0.233853	4.230729
SERTAD4-A	0.851775	0.618746	0.786147	0.807608
ZNF862	0.851775	0.618859	0.244173	4.721838
ABHD16B	0.851775	0.618906	-0.52413	2.614692
MIRLET7F1	0.851775	0.61899	0.504192	2.55377
RAPH1	0.851775	0.618996	-0.21641	6.868278
ZNF786	0.852018	0.619242	-0.38173	3.366472
HIST1H3G	0.852024	0.619315	-0.289	4.215806
IL6	0.852147	0.619473	-1.26192	0.875851
LRFN1	0.852423	0.619743	-0.90602	1.31496
LMF1	0.852463	0.619875	0.652119	0.869304
FOCAD	0.852463	0.619912	-0.21555	5.60937
NRBP2	0.852463	0.620037	-0.2089	5.834638
LRIF1	0.852463	0.620048	0.585158	2.471944
INO80C	0.85264	0.620367	-0.73572	1.803943
FAM210B	0.85264	0.620468	0.217442	6.101058
RAPGEF3	0.85264	0.620473	-1.054	0.735911
PART1	0.85264	0.620502	-0.61778	2.289177
UBIAD1	0.85264	0.62054	-0.35125	3.227812
CELF1	0.85264	0.620591	0.182216	5.486293
ANKRD27	0.852847	0.62085	-0.2071	6.16419
LOC100505	0.852847	0.620944	0.544726	2.258439
LACC1	0.852847	0.62099	-0.84839	0.561311
SBDS	0.852847	0.621019	-0.20403	6.828496
EPG5	0.852892	0.621168	-0.19906	6.68051
MIR4706	0.852892	0.621189	-1.17393	0.63185
ZNF426	0.8531	0.62141	-0.67696	1.886949
CCER2	0.853139	0.621508	-0.8499	1.36844
PSMD6-AS:	0.85315	0.621585	0.261669	4.269812
CYS1	0.853164	0.621664	0.492985	1.580607
EXOSC4	0.853272	0.621827	-0.47491	3.027598
HEATR3	0.853272	0.621881	-0.28021	4.808864
ZNF251	0.853299	0.621974	-0.76647	1.261918

ZNF490	0.853299	0.622066	-0.28496	4.52063
RNF126	0.853299	0.622108	-0.28593	5.190568
OCLNP1	0.853998	0.622687	0.231119	5.247069
RPS17	0.854014	0.622768	0.704227	1.489675
SDF2	0.85414	0.622929	0.626859	0.924586
IL15RA	0.854181	0.623028	0.930069	0.942829
NKX2-3	0.85445	0.623293	-1.36738	0.948201
THUMPD3-	0.854501	0.623427	-1.49451	0.861402
NSFP1	0.854501	0.623476	-0.88414	0.705418
SNORD104	0.854501	0.623538	0.241186	5.93089
GNPTG	0.854748	0.623791	0.27923	4.664526
ZHX3	0.854748	0.623857	0.193601	6.530549
CD177	0.854937	0.624119	-1.0158	2.076032
MRC2	0.854937	0.624133	0.213999	6.631645
THAP6	0.855229	0.624416	-0.83381	1.705176
MYO9A	0.855436	0.624636	-0.2082	7.43376
LOC10099(	0.855469	0.624729	0.728699	0.490509
DMPK	0.85562	0.624929	0.486728	2.273972
CNOT1	0.85562	0.625025	-0.39896	3.113059
LINC00996	0.85562	0.625047	0.835838	1.4168
MEGF6	0.855786	0.625277	0.203075	5.978641
UCN2	0.855786	0.625307	-0.69488	1.509107
PNKP	0.855892	0.625506	0.219261	4.838947
CDRT1	0.855892	0.625523	0.532809	1.805323
NAV1	0.856213	0.625896	-0.41885	2.716121
ACSM3	0.856213	0.625966	0.769385	1.522855
ZBTB24	0.856213	0.626011	-0.30459	3.774052
HCFC1-AS1	0.856213	0.62606	0.885176	1.227489
CTSH	0.856213	0.626105	0.195332	5.468175
TAF5L	0.856291	0.626232	-0.27538	4.21156
HTR2B	0.856291	0.626301	0.35807	3.242136
USP1	0.856389	0.626483	0.496174	1.380166
PDCD4-AS1	0.856389	0.626511	0.416801	1.976585
C10orf88	0.856494	0.62671	0.331029	3.183482
BTBD6	0.856494	0.626778	0.203626	5.332063
PROSER1	0.856494	0.626805	-0.19922	6.010605
SMCHD1	0.856494	0.626887	0.18547	7.793421
COX4I2	0.856494	0.626935	0.802552	0.785385
SESN2	0.856659	0.627125	-0.2538	5.371588
MRPS30	0.857014	0.627489	0.240291	4.720499
TTC13	0.857014	0.627523	0.841243	0.644215
TMA7	0.857047	0.627617	-0.20422	6.929824
CD276	0.857413	0.627991	0.552832	1.518365
GPATCH2L	0.857413	0.628024	-1.2091	0.91328
LSM6	0.857442	0.628115	0.329226	3.694412

GINM1	0.857686	0.628363	0.25611	5.04574
MAP1LC3B	0.858453	0.628995	-0.20446	5.872415
C8orf82	0.8585	0.629099	-0.3688	3.661439
IL1R1	0.858548	0.629309	0.544745	1.51814
PSMC2	0.858548	0.629334	-0.23822	5.219353
NEK2	0.858548	0.629427	0.733568	1.439852
MPC1	0.858548	0.629527	-0.44797	3.415089
LINC01310	0.858548	0.629551	-1.41836	1.22527
MGC27345	0.858548	0.629575	-0.21297	5.035117
ZAP70	0.858548	0.629621	0.6392	1.142196
DUBR	0.8588	0.629874	0.404741	2.55092
ZNF394	0.858916	0.63009	0.228147	4.898545
BRF2	0.858916	0.630099	0.404449	2.678059
DUT	0.858969	0.630208	0.656661	0.788101
ZNF397	0.858984	0.630288	0.257251	5.056604
CA5BP1	0.859273	0.630569	-0.40018	2.992441
SNORD14C	0.859403	0.630735	0.23405	7.200742
ZZZ3	0.859668	0.63101	-0.19245	5.490665
MTOR	0.859668	0.631151	-0.18932	7.214583
SEPSECS-AS	0.859668	0.631159	0.582655	1.097029
CD81-AS1	0.859668	0.631308	0.697837	0.611636
LOC100996	0.859668	0.631435	-0.96114	0.431483
ZNF578	0.859668	0.631629	0.879209	0.831362
RNF25	0.859668	0.631758	0.294984	3.492446
SDC1	0.859668	0.631783	0.467384	1.480763
TNFRSF11E	0.859668	0.631813	0.696896	1.509346
LRP3	0.859668	0.631872	0.477469	2.758194
TMEM150(	0.859668	0.631916	-0.72788	2.602354
C8orf88	0.859668	0.631983	0.743924	0.709336
PPP4R2	0.859668	0.63207	-1.00558	0.783884
NUDC	0.859668	0.63221	0.182615	6.803165
NOL7	0.859668	0.632233	-0.68053	1.453649
TIGD7	0.859668	0.63228	-0.54211	3.088807
MAP3K6	0.859668	0.632292	-1.13382	1.094199
SCAMP4	0.859668	0.632354	-0.19325	5.456163
RXYLT1	0.859668	0.63237	-0.84625	0.630279
FRA10AC1	0.859668	0.632424	0.199169	5.440687
TP53TG1	0.859668	0.632496	0.403574	3.006266
CRYZL1	0.859668	0.632528	-0.318	4.491124
HEATR1	0.859668	0.632651	-0.20288	6.516393
BCL2L11	0.859668	0.632693	-0.58642	3.076973
GRIPAP1	0.859668	0.63271	0.172255	6.62975
TACR1	0.859668	0.63274	0.621146	1.604428
UBE3C	0.85967	0.632811	-0.19109	7.205162
PHPT1	0.859719	0.632917	-0.97364	1.485857

DNAH10	0.859837	0.633073	-1.21905	0.736093
LINC00671	0.859848	0.633151	0.918142	0.94217
KCNMA1-A	0.85992	0.633379	0.69433	1.648557
LINC01091	0.85992	0.633419	0.687012	0.700351
CASKIN1	0.85992	0.633434	-0.81791	1.43458
NR2F2-AS1	0.85992	0.633482	0.680983	2.113201
LOC103344	0.860036	0.633643	0.312165	4.080501
RNF6	0.860036	0.633707	-0.79796	0.728148
RPS27A	0.860197	0.633937	0.533028	1.911446
LINC01364	0.860197	0.633965	0.653897	1.026348
ARRDC1-AS	0.860478	0.634242	0.655881	0.553583
ABHD17C	0.860628	0.634422	0.213192	6.335198
LOC101929	0.860667	0.634521	0.56724	1.759609
GPRC5A	0.860735	0.63464	0.311683	6.945351
ZNF529-AS	0.860766	0.634754	0.747011	1.210586
DPY19L3	0.860766	0.634803	-1.18213	0.547271
KDM3B	0.860838	0.634925	0.183137	7.757042
TRIM37	0.860937	0.635083	-0.51667	2.800965
FAM114A1	0.860937	0.635199	0.300312	3.338752
SPTY2D1OS	0.860937	0.635208	-0.95486	1.673723
POLR2L	0.860998	0.635403	0.185762	5.358256
ANKIB1	0.860998	0.635505	-0.19929	6.597994
CACNA1C-I	0.860998	0.63553	0.743602	0.512084
MIR4442	0.860998	0.635645	0.406077	2.246035
TOB1-AS1	0.860998	0.635689	0.811383	0.840384
LAP3	0.860998	0.63577	0.187847	5.422217
SORCS2	0.860998	0.635782	-0.29135	4.050829
FBXO40	0.860998	0.635857	-1.4247	0.992679
TRIO	0.860998	0.63588	0.417921	1.733883
RSBN1L	0.861227	0.63612	0.17938	5.660601
ALX3	0.861594	0.636489	-0.45022	2.629816
C14orf28	0.861594	0.63656	0.345983	3.444541
SETD1B	0.861594	0.636624	-0.18826	7.338813
ELL3	0.861594	0.63667	0.227155	4.221395
PDK1	0.86183	0.636935	-0.97637	0.783656
PRR13	0.86183	0.637008	-0.73297	1.850524
HNRNPA2B	0.86183	0.637053	-0.81746	1.657901
PPP1R9B	0.861898	0.637173	-0.19439	6.974212
SPATA25	0.861913	0.637254	0.595425	1.493137
CCDC167	0.861919	0.637328	-0.41661	2.677804
KLHL17	0.861981	0.637444	0.308343	3.456009
CTSF	0.862121	0.637617	0.428323	3.356952
MIR4676	0.862672	0.638095	0.861165	1.085191
GJA4	0.862774	0.638247	0.599545	2.002375
DEPDC1	0.862774	0.638542	0.575036	1.805207

ZNF691	0.862774	0.638566	0.794727	1.084735
SLC2A5	0.862774	0.638578	-0.81356	1.273132
CDC42	0.862774	0.638586	0.295634	3.588361
EXOC3-AS1	0.862774	0.638677	0.67767	0.716275
HGH1	0.862774	0.638721	0.251458	3.80292
SLC6A14	0.862774	0.638729	0.336407	5.013201
MIR657	0.862926	0.638929	-1.04829	0.64825
DZIP3	0.862926	0.638998	0.243304	4.427511
HECW2	0.862926	0.639052	-0.66357	2.124383
MTMR11	0.863385	0.63962	0.541644	1.778275
CCDC85B	0.863385	0.639633	-0.31243	4.272529
STPG2	0.863385	0.639691	0.739996	1.432852
TAGLN	0.863385	0.639858	0.679612	0.970983
DCTN5	0.863385	0.639877	-1.03444	0.699367
DUSP28	0.863385	0.639905	-0.60175	2.064686
MIR6758	0.863385	0.639992	0.522944	1.579732
ARHGAP24	0.863385	0.640019	0.412236	3.182332
DLG4	0.863385	0.640046	-0.92269	1.737522
MAP4	0.863385	0.640138	-0.29838	4.180676
MCM9	0.863385	0.640161	0.188188	5.50946
LZTR1	0.86339	0.640235	0.165047	5.720175
CD36	0.863757	0.640577	0.601802	2.079762
SLC25A35	0.863805	0.640751	-0.61959	1.911896
TRAK2	0.863805	0.640752	0.179193	6.52363
DNAJB2	0.864298	0.641335	-0.51433	2.887356
XRRA1	0.864298	0.641342	-0.77985	1.675616
PCDH1	0.864298	0.641359	0.205165	4.726628
LPP-AS1	0.864298	0.641398	0.47378	1.932797
CFAP58-DT	0.86438	0.641593	0.871091	0.984025
ADPGK-AS1	0.86438	0.641599	0.547969	0.773153
RGS16	0.864445	0.641717	0.61271	1.522817
SMC5	0.864481	0.641814	-0.1908	7.953304
GZMB	0.864512	0.641953	-0.85859	1.647685
PPP6R1	0.864512	0.641977	0.192772	7.458381
SRGAP2B	0.865017	0.642482	0.349245	3.965197
TSR3	0.865017	0.642492	-0.26011	4.650327
TRAM1	0.865152	0.642697	0.557759	1.374526
C1orf56	0.865152	0.642732	-0.3498	3.182767
LOC728392	0.86525	0.642963	-0.46892	2.551233
TMEM81	0.86525	0.642988	-0.75874	1.59233
MED9	0.86525	0.643015	0.292087	3.656455
STEAP3	0.865274	0.643203	0.459151	2.270033
GPR65	0.865274	0.643212	0.597732	1.689475
UTRN	0.865274	0.643243	0.18969	9.19993
DDX19A	0.865595	0.643553	0.182225	5.464924

EIF3F	0.866056	0.64404	-0.17826	5.94251
DCTN4	0.866056	0.644084	-0.91663	0.800924
COQ5	0.866056	0.644105	0.256218	4.440364
GTSE1	0.866371	0.64441	0.308706	3.0385
NKIRAS1	0.86643	0.644531	-0.36086	3.599785
ORAI1	0.86643	0.644629	-0.673	1.325602
B4GALT2	0.86643	0.644666	-0.78084	1.352946
UBA1	0.86643	0.644735	-0.44513	3.375507
TPPP	0.866501	0.644857	0.22318	5.819058
EIF2D	0.866621	0.645082	0.317893	3.1077
RGMB	0.866621	0.645087	-0.2718	5.61985
VCPIP1	0.866661	0.645198	-0.18684	6.551575
LINC01128	0.866661	0.645388	-0.5263	2.694458
CELA2B	0.866661	0.645414	-0.78115	2.191762
OBSL1	0.866661	0.645475	-0.58475	1.691875
NIM1K	0.866661	0.645614	0.725738	0.528019
SNX13	0.866661	0.645667	-0.1994	6.536696
SUCLG1	0.866661	0.645794	-0.19	6.461384
PLD1	0.866661	0.645826	0.602921	1.238144
TAF6L	0.866661	0.645888	0.256699	3.616442
LRRC37A2	0.866661	0.645951	0.454385	2.568445
C3orf38	0.866661	0.645981	-0.26971	3.959964
HERC2P2	0.866661	0.646011	0.202561	6.766045
ZNF528-AS	0.866661	0.64603	0.448716	2.413798
PFN1	0.866846	0.646238	-0.19719	7.940229
ZNF853	0.866899	0.646348	0.574417	1.715572
MROH9	0.86693	0.646441	-1.02155	0.756267
HTRA2	0.866971	0.646541	0.616469	1.310069
ATP5MD	0.867584	0.647081	-0.53175	1.933506
SYT10	0.867584	0.647139	1.057559	0.889614
BMS1P20	0.86766	0.647266	-0.20919	5.755423
USP40	0.867758	0.647448	-0.19204	6.735719
PYM1	0.867758	0.64748	-0.91969	1.285437
TRIP12	0.868058	0.647774	-0.94745	0.997039
SENP8	0.868068	0.647852	0.614758	1.730768
SNORA81	0.868685	0.64845	0.340559	2.734236
CTNNBIP1	0.868685	0.648453	-1.12576	0.797524
GTF3A	0.8687	0.648535	-0.22603	4.865411
EIF3G	0.868778	0.648663	0.172251	6.308087
ADAT3	0.869053	0.649001	0.476633	1.710754
GLA	0.869053	0.649013	-0.63254	2.474401
SERINC1	0.869053	0.649139	-0.19339	7.394613
RING1	0.869053	0.64915	-0.66108	1.629087
COG7	0.869164	0.64932	0.197927	4.831345
KIN	0.869164	0.649374	-0.65395	1.919719

SLC18B1	0.869385	0.64967	0.222207	4.963916
TMSB10	0.869385	0.64968	-0.18882	9.200615
SEC61B	0.869565	0.649884	-0.24173	4.772567
TRIM25	0.869704	0.650059	0.171208	6.885051
SPCS2	0.869861	0.650246	0.179882	5.734932
ABCF2	0.869923	0.650393	-0.62005	3.230642
NDUFS1	0.869923	0.650485	0.574185	1.264206
MIR200A	0.869923	0.650554	-0.89666	1.084171
RDH11	0.869923	0.650587	-0.79507	1.553787
ERBB2	0.869923	0.65066	-0.78669	1.809093
CTDSP2	0.869923	0.650889	-0.18897	7.578112
SHPRH	0.869923	0.650941	-0.94648	1.159686
GMFB	0.869923	0.650946	-0.19078	5.751258
RARRES1	0.869923	0.650968	0.630216	1.227204
NANP	0.869923	0.650998	-0.34238	3.257964
RHBDL2	0.870144	0.651308	0.619159	0.620419
KNTC1	0.870144	0.651325	-0.21473	5.882593
COMMD8	0.870144	0.651375	-0.26988	4.305178
ANK3	0.870152	0.651481	0.195	5.662457
NAXE	0.870152	0.651522	-0.22528	4.521972
CHSY1	0.870285	0.651692	0.185114	5.849792
GPAA1	0.870318	0.651843	-0.2139	5.125766
CCDC61	0.870318	0.651857	0.281736	3.779598
RAB6B	0.870978	0.652422	-0.37	2.772078
LINC00938	0.871066	0.652607	-0.22064	4.578728
NME3	0.871066	0.652629	0.416666	2.56991
TMEM208	0.871137	0.652753	0.287995	3.768968
AHI1	0.871187	0.652902	0.5341	1.740087
LOC10272(	0.871187	0.652931	0.684546	0.686298
LRRC56	0.871325	0.653105	-1.31632	0.553509
GUCA1B	0.871379	0.653385	0.425384	2.502208
PFN1P2	0.871379	0.653403	-0.30806	3.950883
DOCK10	0.871379	0.65356	0.496551	1.941058
ZBTB34	0.871379	0.653597	0.257209	4.519124
ATF3	0.871379	0.653635	0.855625	0.787095
MRPS21	0.871379	0.653669	0.517282	1.586887
RNY5	0.871379	0.653708	-0.25944	7.482902
TIGD2	0.871379	0.653711	0.303294	3.160542
MIER1	0.871641	0.653978	0.422908	2.978231
CSGALNAC	0.871924	0.654295	0.723077	0.921451
SFTPD	0.871924	0.654331	-0.84381	0.41999
WDFY3-AS:	0.872037	0.654568	-0.85983	1.213057
TMOD4	0.872037	0.654572	0.769548	0.730248
LINC00571	0.872037	0.654695	-0.80259	1.6234
LOC10192ɛ	0.872037	0.654808	-0.53686	2.941563

IRAIN	0.872037	0.654829	0.376098	2.861256
GAS5	0.872037	0.65484	-0.22024	5.608135
TOB2P1	0.872254	0.655135	0.737774	0.746246
RIDA	0.872254	0.655185	0.284474	3.712636
GATAD1	0.872254	0.655215	0.452832	2.173512
MT1L	0.872255	0.655286	0.696198	0.888837
NPIPB3	0.872413	0.655503	0.198776	4.57406
SNORD136	0.872413	0.655614	-1.32773	0.530219
ZNF385C	0.872413	0.655617	0.538403	0.793571
MRPL30	0.872749	0.65594	0.262283	4.175363
FAHD1	0.872822	0.656066	0.313284	3.922581
BMP8A	0.872881	0.656212	0.289436	3.515249
RPTOR	0.872881	0.656252	-0.86193	1.544255
RGS18	0.872905	0.65634	0.613298	1.150355
THAP2	0.872928	0.656672	-0.54384	2.486385
DIPK1A	0.872928	0.65668	-0.25166	4.384388
CKAP5	0.872928	0.656777	-0.55	1.721916
SULT1C4	0.872928	0.656869	0.549468	1.425231
NEMP2	0.872928	0.65691	0.362957	3.965218
GATB	0.872928	0.656918	0.242709	4.336812
FAM13B	0.872928	0.656984	-1.07064	0.939756
CFAP45	0.872928	0.656992	0.467988	1.94417
MTMR6	0.872928	0.65709	-0.22819	4.936372
LOC10272[illegible]	0.872928	0.657151	-0.57391	2.668297
LOC28329[illegible]	0.872928	0.657206	0.665299	0.490275
PUS3	0.872928	0.657206	-0.72499	1.590125
MIGA2	0.873091	0.6574	-0.21223	5.809125
HDAC6	0.873113	0.657491	-0.17359	5.893315
DPY19L2	0.873113	0.657584	0.449565	2.423428
MGA	0.873113	0.657628	0.572593	0.739525
LOC10192[illegible]	0.873743	0.658174	0.650304	0.644971
HIST1H2BJ	0.874213	0.658669	-0.29597	4.559555
PDCD10	0.874213	0.658669	-0.59159	2.800705
JMJD1C	0.874244	0.658806	-0.33777	3.535433
PPP1R3E	0.874244	0.658886	-0.31293	4.104883
MCTP2	0.874244	0.658941	0.295719	3.282525
SLC16A8	0.874244	0.658976	0.427793	2.028593
TRPV6	0.874296	0.659086	0.430203	1.974052
OARD1	0.874296	0.659157	0.20434	5.102062
SYNM	0.874382	0.659301	0.512528	2.772526
CSNK1G2	0.874382	0.659427	-0.17594	5.856713
LIN52	0.874382	0.659528	0.369691	2.679666
SUPT6H	0.874382	0.659543	-0.18142	8.246491
FUT8	0.874382	0.659579	-0.69509	1.066006
INSIG2	0.874382	0.659755	-0.22235	5.159481

CIRBP-AS1	0.874382	0.659765	-0.50813	2.724097
GNA12	0.874382	0.65987	0.550725	1.386617
HIST1H4K	0.874382	0.659894	-0.56024	3.204733
FMO6P	0.874382	0.660054	0.549613	1.922214
HIST1H2AM	0.874382	0.660112	-0.29323	4.411795
OR51E1	0.874382	0.660123	0.868206	1.319608
ADM5	0.874382	0.660392	0.664562	1.204747
MBLAC2	0.874382	0.660462	0.313485	3.923219
NUDT21	0.874382	0.660529	-0.19103	6.895138
TAOK3	0.874382	0.66053	0.155388	7.174832
FAM208B	0.874382	0.660549	-0.17784	7.32864
PIK3C3	0.874382	0.660614	0.595322	1.273046
MMP3	0.874382	0.660637	0.774845	1.296545
ZNF688	0.874382	0.660731	-0.72005	0.74029
ASB7	0.874382	0.660749	-0.27978	3.983198
SMAD5-AS	0.874382	0.660779	-0.59178	1.782947
MAPK11	0.874476	0.661013	0.632876	1.422156
MIR210	0.874476	0.66105	-0.86802	0.77271
ZNF544	0.874476	0.661063	0.164187	5.581392
WBP4	0.874606	0.661232	0.224786	5.05192
GRIK1-AS2	0.874774	0.661435	0.73176	0.542906
CXCL2	0.874774	0.661554	-0.57071	3.400153
ALG6	0.874774	0.661572	-0.39094	3.716247
TYMS	0.874792	0.66169	0.307976	3.067406
BCL6B	0.874792	0.661727	0.398085	2.256944
SCARNA22	0.875005	0.661959	-0.31723	4.149827
SNORD116	0.875064	0.662075	-0.54347	2.63549
THAP10	0.875314	0.662335	0.623117	1.184527
B4GALT6	0.875417	0.662694	0.360244	2.927766
CDAN1	0.875417	0.662696	0.204585	4.748378
IL10RB-DT	0.875417	0.662696	0.665065	0.844386
KPNA5	0.875417	0.662696	-0.27427	4.009763
LRRC47	0.875656	0.662948	-0.18105	5.671429
HDAC2-AS2	0.875657	0.663019	-0.91732	0.646262
SP4	0.876173	0.663481	0.431117	3.554188
CTSD	0.876192	0.663567	-0.18575	8.139189
SCO1	0.876481	0.663857	-0.19992	5.183629
CSPP1	0.876922	0.664261	-0.60203	1.899676
SMC2	0.877169	0.664555	0.639193	1.164161
SETSIP	0.877169	0.664591	-0.44308	2.169208
TXNDC12	0.877208	0.664724	0.243951	4.031672
STT3A-AS1	0.877208	0.664865	-0.93758	1.42026
LRG1	0.877208	0.664892	-0.38073	3.921574
FERMT1	0.877208	0.664905	0.19125	6.73318
LOC100505	0.877553	0.665237	-1.0735	0.657135

C19orf53	0.877844	0.665529	-0.18528	5.538437
NGFR	0.878121	0.665813	-0.42859	3.179241
ARSK	0.878121	0.665881	-0.41547	2.647481
AKT1S1	0.878368	0.666208	0.399392	2.046991
TMEM86B	0.878368	0.666251	-0.28445	3.793643
KCNA7	0.878368	0.666282	-0.97746	0.530055
HIPK3	0.87861	0.666536	0.42689	2.235529
LOC10013(	0.878835	0.666779	0.311809	3.173593
LHFPL4	0.879137	0.667079	-0.84537	1.551103
SNORA16A	0.879253	0.667244	-0.33892	3.997378
SIRT2	0.879253	0.66731	0.603535	0.743299
KIF1BP	0.879291	0.667409	0.208833	5.214635
CCDC106	0.879595	0.66789	0.351727	2.827641
MCAT	0.879595	0.667961	0.569652	1.224717
ZNF319	0.879595	0.668027	-0.43345	3.117381
COPS8	0.879595	0.668033	-1.09285	0.579221
LOC10272?	0.879595	0.668082	0.735338	1.520812
MEGF11	0.879595	0.668295	0.243549	3.787751
LOC10050(	0.879595	0.66833	0.437151	3.469824
LRRC37A	0.879595	0.668414	0.527496	1.092525
FAM171B	0.879595	0.668477	0.436122	2.881237
MTFR1	0.879595	0.668561	0.720057	0.645472
CXCL10	0.879595	0.66857	-0.87067	1.279532
CCDC71L	0.879595	0.66868	0.31421	3.671755
HMGN2P4(	0.879595	0.668741	0.540211	1.148171
MRPL52	0.879595	0.668772	0.465643	1.881081
CIR1	0.879595	0.668857	0.170989	5.604299
GLI4	0.879595	0.668859	-0.32603	3.37526
S100A6	0.879595	0.668912	0.181838	9.40855
SMARCC1	0.879595	0.668923	0.160192	8.426635
DIPK1B	0.879787	0.66914	0.318257	2.80685
ABCC10	0.879817	0.669349	-0.3995	2.415944
METTL21A	0.879817	0.669385	0.586449	1.769046
ESRG	0.879817	0.669412	-0.53063	1.660738
RNVU1-15	0.879817	0.669447	-0.66217	1.228794
SMIM10	0.879898	0.66958	0.559016	1.609059
HIST1H2BH	0.879923	0.669732	0.221375	4.29035
LOC14584!	0.879923	0.669773	0.82348	0.893791
TMBIM4	0.879923	0.669813	0.746451	0.945049
HTATSF1P2	0.879943	0.670027	0.370264	3.214045
CYP1B1	0.879943	0.670089	0.508124	3.095348
HEATR6	0.879943	0.670098	0.22262	4.001069
ARL1	0.879943	0.670114	0.575254	1.193582
CSRP3	0.880327	0.670511	0.636115	1.055462
DHX37	0.880327	0.670568	-0.23263	4.957261

USP36	0.880327	0.67062	0.174588	6.46945
EEF1A1	0.88036	0.670766	-0.2375	11.03698
HOTAIRM1	0.88036	0.670802	-0.52794	2.704987
PRSS8	0.88036	0.670859	-0.19809	6.582044
EFCAB14-A	0.880547	0.671073	-1.07825	1.1127
LOC10013:	0.880594	0.67118	-0.46551	1.78222
MAFG	0.880713	0.671515	-0.8533	0.544346
CD80	0.880713	0.671521	0.682067	0.946827
CRY1	0.880713	0.671545	-0.26397	4.802828
INTS1	0.880713	0.671556	-0.18412	6.865698
IER5L	0.880791	0.671687	-0.39476	3.648895
DMD	0.880883	0.671877	0.615224	1.73684
METTL3	0.880883	0.671919	-0.1747	5.537533
ARID4A	0.880883	0.672039	-0.95486	1.264369
MON1B	0.880883	0.672042	0.323784	2.762443
FGF14-IT1	0.880961	0.672233	0.557099	1.524366
IFRD1	0.880961	0.672245	0.454479	1.546225
GNL2	0.880972	0.672385	-0.17978	6.652999
UBE2L3	0.880972	0.672498	-0.61562	2.062719
BTF3	0.880972	0.672511	0.424566	2.390191
PARPBP	0.880972	0.672539	-0.35779	3.009031
NALCN	0.881368	0.673011	-0.95807	1.251894
RAB11FIP2	0.881368	0.673117	-0.23976	5.813819
TMEM123	0.881368	0.673148	-0.18979	7.496031
GOT1L1	0.881368	0.673317	-1.17771	0.822456
DCAF16	0.881368	0.673331	0.167481	5.881039
ZNF774	0.881368	0.673397	0.410263	1.814196
GNPDA1	0.881368	0.673422	0.230247	4.05756
NKG7	0.881368	0.673465	-0.66687	1.492288
OR7E12P	0.881368	0.673556	0.540215	2.422267
AFF1-AS1	0.881368	0.673562	0.274272	3.028974
TMEM183I	0.881368	0.673626	-0.42194	2.389091
SNORD44	0.881396	0.673719	0.429712	2.173751
FAM222A	0.881588	0.67394	0.330139	3.55277
C1orf220	0.881588	0.674027	0.33587	2.512915
TMBIM6	0.881588	0.67408	0.492312	1.83756
ZNF175	0.881839	0.674343	-0.48832	3.211348
PSPH	0.882298	0.674766	-0.28628	3.879584
ETS2	0.88233	0.674862	0.499897	1.050393
LOC10260€	0.882359	0.674956	-0.61624	1.810087
UBE2MP1	0.882384	0.675046	-0.62153	1.375153
AIRN	0.882473	0.675254	0.376455	2.785439
PKHD1	0.882473	0.675317	0.681689	1.033985
SIPA1L1	0.882473	0.675329	-0.67093	0.910446
ZFAT-AS1	0.882563	0.675468	-0.75432	0.701907

SCAF11	0.882801	0.675732	-0.16917	8.453522
C12orf10	0.882801	0.675967	0.241869	3.964138
SLC35F5	0.882801	0.675978	-0.54334	2.373346
INSRR	0.882801	0.675993	-1.26589	0.901084
LINC01913	0.882801	0.676008	0.848964	1.262774
ASCC1	0.883353	0.676539	0.460031	1.714003
WBP2NL	0.883353	0.676747	0.56939	1.244206
FAM98C	0.883353	0.67676	-0.23992	4.045979
ZNF782	0.883353	0.676806	0.190597	4.987055
ZNF185	0.883353	0.676852	-1.04657	1.170337
FAM167A	0.883353	0.676861	-0.41322	2.967077
ZNF593	0.883395	0.676965	0.331467	2.940357
SLITRK6	0.883395	0.677036	0.336567	3.250305
INHBE	0.883779	0.677501	0.719622	0.495576
MIR95	0.883779	0.677549	0.775093	1.489258
MAFG-DT	0.883779	0.677575	-0.67212	1.777142
XAB2	0.883779	0.677617	-0.18398	5.190421
MED16	0.883808	0.67771	-0.17433	5.441787
NUDT5	0.884204	0.678085	-0.21674	4.527707
DPYSL3	0.884266	0.678205	0.495966	1.855955
POU5F1P4	0.884534	0.678482	-0.84857	1.182742
CAND1.11	0.884776	0.678739	-0.64932	1.540371
HIST1H2AE	0.884966	0.678957	-0.32678	3.353895
CAMTA1	0.885293	0.679322	0.444939	2.094216
DNMT3A	0.885293	0.679419	0.388649	2.386539
ZNF354C	0.885293	0.679475	0.297375	3.798727
AFF1	0.885293	0.679494	0.248627	3.665639
FBXO38	0.88549	0.679718	0.292186	2.633985
SPRR2G	0.885534	0.679822	-1.19399	0.880298
KCNA10	0.885773	0.680078	0.799134	0.75183
C7orf31	0.885843	0.68036	0.341648	3.086745
RGMB-AS1	0.885843	0.680391	-1.21982	1.632707
SNX20	0.885843	0.68048	0.578372	0.846028
NCKAP5L	0.885843	0.680509	0.205696	4.591111
SLC38A1	0.885843	0.68053	0.21101	3.978879
CARD18	0.885843	0.680819	-1.03133	0.985508
WDR97	0.885843	0.680857	0.599049	1.183601
TTC17	0.885843	0.680881	0.200711	5.232877
FURIN	0.885843	0.680932	0.510912	0.681968
UST	0.885843	0.680939	0.238053	3.894097
TOP1MT	0.885843	0.680941	0.489856	1.64321
SERP1	0.885843	0.680992	-0.17588	6.843714
CBWD6	0.886205	0.681342	0.438244	2.49963
PIGU	0.886223	0.681428	-0.27255	3.841693
SNAI2	0.886225	0.681555	0.215818	5.116797

MATN3	0.886225	0.681632	0.771649	0.521653
NBPF3	0.886225	0.681646	0.557385	0.905197
PDLIM3	0.886277	0.681813	0.588768	1.056175
CHST14	0.886277	0.681893	-0.44225	2.344988
SRPRB	0.886277	0.6819	-0.21315	5.684909
FAM117A	0.886423	0.682085	-0.25962	3.475522
GSTCD	0.886538	0.682248	-0.81994	1.272652
SMIM25	0.886538	0.682356	-0.95697	0.648661
AEN	0.886538	0.682389	0.215358	4.300961
SNORD111	0.887262	0.683071	0.34587	2.002735
MIR6887	0.887262	0.68309	-0.74229	1.654192
RSL1D1	0.887339	0.683345	-0.1597	7.362401
COQ4	0.887339	0.683348	0.266213	3.417812
RHOA	0.887339	0.683384	-0.79507	0.75064
LOC93622	0.887339	0.683545	0.229838	4.163242
AMIGO3	0.887339	0.683578	-0.34763	3.6145
DDB2	0.887339	0.683656	-0.24661	3.986119
IWS1	0.887339	0.68369	0.150441	7.206516
HMCES	0.887339	0.683724	0.659865	0.766158
DEFB131B	0.887412	0.683852	-0.81867	1.273
SERTAD1	0.88747	0.683969	-0.32342	3.252312
KATNA1	0.887498	0.684062	0.418675	2.24625
LZTS2	0.887527	0.684217	-0.24336	5.073825
MIR27A	0.887527	0.684228	-1.02087	0.985594
ISM1-AS1	0.887547	0.684436	0.665975	0.554062
GJD3	0.887547	0.684489	0.203994	4.40168
MAP2K2	0.887547	0.684493	-0.17297	6.735676
MAP2K4P1	0.887547	0.684532	-0.66369	1.030141
SYCE2	0.887547	0.684603	-0.85868	0.592554
RPL21P44	0.887645	0.684779	0.402944	2.924898
COL9A3	0.887645	0.684823	0.568971	2.111258
CXCL8	0.887719	0.684958	-0.93712	3.040206
HIST1H3D	0.887719	0.685129	-0.2552	3.933599
GPSM1	0.887719	0.685172	-0.60714	2.117765
C7	0.887719	0.685234	0.440324	4.171295
MIR3139	0.887719	0.685285	0.607458	1.249179
TFCP2L1	0.887719	0.685311	-0.19144	7.854437
WDR33	0.887771	0.685439	0.149948	6.603476
PRPF4B	0.887771	0.685562	-0.16509	8.046599
SLC9A1	0.887771	0.685567	0.377302	1.94914
DPF2	0.887913	0.685749	0.187787	4.762022
ITGB7	0.888132	0.686043	-0.54255	1.991558
SP140	0.888132	0.686192	0.779167	0.473979
TMEM241	0.888132	0.686236	0.226965	4.131296
UNC50	0.888132	0.686251	0.184707	4.664345

NLRX1	0.888132	0.686278	-0.56188	2.155608
PMPCB	0.888202	0.686403	0.171652	5.881959
MACROD2	0.888324	0.686569	-0.41867	2.243753
TACO1	0.888367	0.686675	-0.25322	3.821115
NDUFA10	0.888439	0.686844	-0.1757	7.167348
IFT80	0.888439	0.686989	-0.70304	1.458447
MRPS11	0.888439	0.687071	-0.63123	1.137547
CHMP6	0.888439	0.687092	0.182386	4.709805
LOC10050!	0.888439	0.687117	-0.47969	2.295712
CCDC122	0.888439	0.687162	-0.43032	3.068512
LY9	0.888727	0.687457	0.540157	1.125761
HEXA-AS1	0.888736	0.687536	0.637344	1.314659
SNORD83A	0.888812	0.687712	0.305986	2.599026
PPIL4	0.888812	0.687739	0.154411	6.350195
SNORD42B	0.889126	0.688054	-0.75724	1.209743
LONRF2	0.889428	0.688368	-0.48124	4.009976
SPACA6	0.889428	0.688431	-0.2986	3.80633
KLB	0.889534	0.688585	-0.68987	2.150483
YY1	0.889603	0.688711	-0.1596	6.946431
RARA-AS1	0.889717	0.688872	-0.61331	2.233365
ST7	0.88973	0.689009	0.432513	2.217334
PHF2	0.88973	0.689025	0.151154	6.319509
LOC10012{	0.889854	0.689193	0.248707	3.786829
ZC3HAV1	0.889902	0.689303	0.147571	6.417271
DPY19L4	0.889932	0.68942	-0.18356	5.478602
GNAI2	0.889932	0.68947	0.450602	2.132279
RUNX1	0.890372	0.689989	-0.6146	2.510782
TRIM9	0.890372	0.690026	-0.86781	1.186512
SNORA40	0.890372	0.690028	-0.20621	5.837199
RRP9	0.890442	0.690154	-0.3014	3.499229
AP3S2	0.891027	0.690711	0.51982	1.760794
NOCT	0.891027	0.690773	-0.28151	3.680035
ZNF500	0.891027	0.690839	0.59934	0.539557
EGOT	0.891027	0.690896	-0.49404	2.356563
NDUFAF7	0.891059	0.690993	-0.92079	0.747124
PLPBP	0.891262	0.691222	-0.18586	5.432546
SNORD24	0.891285	0.691312	0.268615	3.021983
CTBP2	0.891756	0.691788	0.184739	4.489905
VN1R1	0.891756	0.691822	-0.6733	1.404631
LOC101927	0.891877	0.692023	-0.89629	1.469363
MC1R	0.891877	0.692085	-0.45392	2.51198
FUT9	0.891877	0.692133	0.524806	4.656715
SNORA4	0.891959	0.692269	-0.27438	3.564198
NARF	0.891976	0.692382	-1.08251	0.768249
POLR1D	0.891976	0.692434	-0.23147	4.729863

CBWD5	0.891976	0.692499	-0.57116	2.10177
PWP2	0.892182	0.692912	0.329431	2.987749
LINC00628	0.892182	0.692921	0.511434	1.050822
SNHG8	0.892182	0.692948	-0.96453	0.839892
SNRNP35	0.892182	0.692962	0.464882	1.608017
POGK	0.892182	0.69302	-0.80523	0.525837
LOC171391	0.892262	0.693258	-0.71818	1.227612
SNORA60	0.892262	0.693335	0.294645	3.707225
LOC101928	0.892262	0.693399	0.677657	1.119827
LUCAT1	0.892262	0.693419	-1.04029	0.896029
PLEKHG4B	0.892262	0.693444	-0.45213	3.168429
SWAP70	0.892462	0.693716	-0.79421	1.208435
CHRM3	0.892462	0.693744	-0.86644	1.413922
TMEM220-	0.892944	0.69419	0.516426	1.513365
OXGR1	0.892991	0.6943	-0.94856	1.259116
PHF20L1	0.893207	0.69454	-0.48581	2.208949
TRAP1	0.89328	0.694675	0.39356	2.063435
SZT2	0.89328	0.694741	0.139603	7.321528
HIST1H2BG	0.893406	0.694912	-0.26703	4.219656
POLR3D	0.893683	0.695246	0.164175	5.439974
TUBE1	0.893683	0.695343	0.31061	4.085077
ATP6V0C	0.893683	0.695344	0.464158	0.742227
C6orf226	0.893695	0.695497	0.463784	1.130717
DMAC1	0.893695	0.69552	0.196921	4.177518
TCEA1	0.893695	0.69567	-0.76352	1.353127
HEY2	0.893695	0.695732	-0.72311	1.73409
RRP8	0.893695	0.695758	0.194982	4.507728
KMT2D	0.893695	0.695788	-0.15508	8.992821
AASS	0.893798	0.695962	-0.18852	6.168028
IFT20	0.893798	0.696012	0.594869	0.956563
SNRK-AS1	0.893884	0.696152	-0.38749	2.761414
INTS5	0.894357	0.696593	-0.22913	4.134048
SPAG5	0.894561	0.69686	0.19305	4.420307
KCTD1	0.894561	0.696958	-0.2381	3.954193
GCNA	0.894561	0.697087	0.357975	2.671256
B3GNT10	0.894561	0.697153	0.443889	1.40644
MIRLET7A1	0.894561	0.697319	-0.56668	2.14549
FER	0.894561	0.697348	0.39189	2.908844
SLC7A14	0.894561	0.697349	-0.91241	1.101051
RAMP2-AS	0.894561	0.697438	0.78012	1.062888
SEMA6A	0.894561	0.697442	0.663317	0.928941
GCNT2	0.894561	0.697477	0.211001	4.755281
SAMD8	0.894567	0.697554	-0.71512	0.911264
TJP2	0.894771	0.697785	-0.20813	5.209972
ATP7A	0.894825	0.697905	-0.87507	0.547113

FIG4	0.894825	0.697972	0.21258	4.984318
RPL13AP20	0.894963	0.698153	0.266779	3.058385
LOC100288	0.895439	0.698596	-0.59359	1.356334
RAD51B	0.89563	0.698818	0.294616	2.759752
MRPL23	0.895825	0.699078	-0.29242	3.564395
YTHDF1	0.895825	0.699115	0.143301	5.551449
DCDC2B	0.895856	0.699212	0.323968	2.74873
SHISA9	0.895906	0.699324	0.588183	0.682415
PSMG4	0.896467	0.699863	-1.02779	0.725738
FEM1A	0.896467	0.700057	-0.16668	5.655303
B3GAT3	0.896467	0.700073	0.55823	1.383632
ZBTB49	0.896467	0.700078	-0.42376	2.998246
SNORD94	0.896467	0.700124	0.178939	5.546598
CLEC2B	0.896533	0.700256	-0.23666	3.881611
EXOSC2	0.896533	0.700335	0.3872	1.804926
TBPL1	0.896533	0.700393	-0.86316	1.159683
CMIP	0.896688	0.700587	0.227039	4.13921
CPNE8	0.896756	0.700827	-0.24141	4.991451
CRIPAK	0.896756	0.700858	-0.18781	4.852836
RABGEF1	0.896756	0.700858	-0.27495	3.755855
RNF24	0.896783	0.701009	0.43929	1.022769
RPAP2	0.896783	0.701024	0.171213	5.441721
TINAGL1	0.896809	0.701164	0.560027	0.92182
PCDHB13	0.896809	0.701191	0.387209	2.444238
MAGI1-AS1	0.896817	0.701269	-0.40184	3.358172
GABARAP	0.896879	0.701391	-0.15409	6.890576
FHAD1	0.897079	0.701643	-0.68327	1.846767
MAX	0.897079	0.701897	0.439026	1.71967
LOC100506	0.897079	0.701898	0.303892	2.800019
TMEM108-	0.897079	0.702003	0.706258	0.515716
HIST1H2BC	0.897079	0.702021	-0.29004	3.652759
ABHD6	0.897079	0.702036	-0.24285	3.734378
LOC101928	0.897079	0.702116	-0.55602	1.731084
GABPB1-AS	0.897079	0.702181	0.178685	6.183473
ARHGEF28	0.897079	0.702201	-0.94198	0.930817
ORC4	0.89708	0.702274	0.496621	1.196907
FAU	0.897192	0.702503	-0.16747	7.425224
DKFZP586I	0.897192	0.702507	-0.18834	5.269575
FOXO3	0.897216	0.702599	0.442247	2.40068
THOC7-AS1	0.897313	0.702864	0.371276	1.996771
BCL2L1	0.897313	0.702994	0.619382	0.693755
LINC01137	0.897313	0.703039	0.41996	1.781613
IL4R	0.897313	0.703119	0.168973	5.119148
TOMM6	0.897313	0.703153	-0.19281	5.188079
MAPK8IP1I	0.897313	0.703175	0.591713	2.040202

CCNF	0.897313	0.703304	-0.23499	3.894361
DNAJB11	0.897313	0.703394	-0.16088	6.561253
MITF	0.897313	0.703403	0.542649	1.092957
LINC02076	0.897313	0.703413	-0.53672	1.395052
RNF111	0.897313	0.703485	0.354192	2.174284
SNORD36C	0.897313	0.703616	-0.45498	2.250214
ANKS1A	0.897313	0.70362	0.141838	6.230716
KCNK6	0.897351	0.703794	-0.18048	7.385943
CD40LG	0.897351	0.703795	0.572747	0.916039
CLK1	0.897501	0.703985	-0.17963	5.836167
MCRS1	0.897548	0.704095	0.583868	0.909663
FOXJ3	0.897777	0.704347	-0.60701	1.924357
PIGZ	0.897781	0.704482	0.349343	2.308569
RBM4	0.897781	0.704496	0.212016	4.833937
MOSMO	0.897833	0.704609	0.191455	4.517095
PISD	0.89787	0.704711	0.150135	5.266604
BMS1P4	0.897927	0.704863	-0.21368	4.448453
MRPL15	0.897927	0.704901	0.164833	5.090017
GOLGA8T	0.898155	0.705224	-0.65225	1.779719
CTSO	0.898155	0.705226	0.516632	1.161035
FAM180A	0.898387	0.705481	-0.5686	2.168723
SGSM3	0.898481	0.705627	-0.88012	1.175152
JRKL	0.898487	0.705852	0.531397	0.864945
CCDC174	0.898487	0.705875	0.149123	5.613126
LINC01126	0.898487	0.705905	-0.64819	0.694549
CLIC4	0.898487	0.706021	0.168535	7.090764
NUTF2	0.898487	0.706068	-0.20435	4.760439
SNORA98	0.898487	0.706078	-1.11802	0.808028
SRD5A3	0.898487	0.706142	-0.18182	5.022959
GOLT1B	0.898501	0.706225	0.154017	5.014971
ZCCHC8	0.898743	0.706488	-0.17888	5.450255
SLC44A1	0.898848	0.706643	-0.25113	4.733152
FLJ31356	0.898903	0.706759	0.206816	3.857186
SOS1	0.899032	0.706973	-0.15194	7.485198
QPCTL	0.899032	0.707006	0.590153	1.006263
GALE	0.899088	0.707123	0.393781	1.611881
LOC100132	0.899395	0.707438	-0.50189	2.110929
GPIHBP1	0.899468	0.707568	-0.97911	0.884549
TTC5	0.899468	0.707658	0.255781	3.5274
TOP1	0.899468	0.707782	-0.15926	8.384441
CASQ1	0.899468	0.707808	-0.50978	1.483494
GTDC1	0.899468	0.707859	0.459899	1.5569
RAB11B-AS	0.899501	0.707958	-0.45167	2.431325
SNED1	0.899604	0.708112	0.189397	5.502029
SNORD116	0.899633	0.708266	-0.43429	2.973602

LRRC37B	0.899633	0.708392	-0.34676	3.963482
LINC01311	0.899633	0.708412	0.371116	1.912082
UTP18	0.899633	0.708593	-0.17841	5.221663
MIR548K	0.899633	0.708594	0.724874	0.65388
EID1	0.899633	0.708615	0.152341	6.639177
TAGLN2	0.899633	0.708645	0.458089	1.371687
ASB9P1	0.89965	0.708731	-0.8243	0.665738
OSBPL10-A	0.899775	0.708959	0.408793	2.273648
ATP13A3	0.899775	0.708975	-0.17565	7.321632
RPS2	0.899901	0.70927	-0.1692	8.755967
RBM5-AS1	0.899901	0.709364	-0.17181	5.02076
SLC39A3	0.899901	0.709411	0.27986	2.546642
LOC101929	0.899901	0.709474	-0.75095	0.815199
DPH1	0.899901	0.709574	0.214107	3.886317
LOC100288	0.899901	0.709593	0.398779	2.115094
NDUFB4	0.899901	0.709629	-0.55392	1.909338
OTUD1	0.899901	0.709754	0.205317	4.581986
FKSG29	0.899901	0.709822	0.450072	1.844227
GTF2IRD2	0.899901	0.709863	0.404217	2.216994
RRAS	0.899901	0.709962	0.239246	3.330004
FDPS	0.899901	0.710014	-0.75432	1.384443
DCAF4L1	0.899901	0.710022	0.522519	0.691937
CEP126	0.900342	0.710578	0.351529	3.348467
MAGOH2P	0.900342	0.710602	-0.67315	1.373023
LINC00678	0.900342	0.710606	0.824745	0.529254
CCDC160	0.900342	0.710662	-0.7728	1.563185
CCS	0.90085	0.711261	0.206308	4.350182
UFC1	0.90085	0.711279	-0.1587	5.292782
KCNJ14	0.90085	0.711282	-0.39495	2.106528
TBL1XR1	0.901002	0.711475	-0.16053	8.247195
HNRNPA1	0.901049	0.711585	-0.25374	3.994919
LYPLA2P2	0.901085	0.711687	0.481633	0.612272
FOXJ2	0.901303	0.711931	-0.15468	5.760141
HPS3	0.901357	0.712048	0.31132	2.863987
NXPE1	0.901425	0.712211	0.519934	1.660635
CEP72	0.901425	0.712247	0.213123	4.185897
AFG3L2	0.901922	0.712769	-0.14788	7.109308
F8A1	0.901922	0.712785	-0.49897	2.363276
CSNK1A1	0.901981	0.712905	-0.97405	0.667683
MFSD4B	0.901997	0.712991	0.147828	5.025158
SLC30A7	0.902035	0.713207	-0.47997	1.822102
ZNF524	0.902035	0.713234	0.264549	3.551691
CPA6	0.902035	0.71326	-0.85086	0.66106
ABRAXAS1	0.902035	0.713318	0.25133	3.430028
USP34	0.902035	0.713409	-0.15328	8.870293

KRT75	0.902035	0.713459	-0.92912	1.750123
EDARADD	0.90211	0.713592	-1.0087	0.91275
RNY4	0.90223	0.71376	-0.19565	10.05849
GBP1	0.902307	0.713895	-0.22242	5.152773
SNORA24	0.902307	0.714022	-0.20999	7.180405
ST13P4	0.902307	0.71404	-0.19441	4.520871
SF3B2	0.902395	0.714183	0.136057	8.352576
PYGO2	0.902484	0.714327	0.161835	5.09343
COX4I1	0.902928	0.714751	0.141608	8.106868
HSF2BP	0.903038	0.714911	-0.69918	0.961775
ILKAP	0.903117	0.71506	0.194422	5.084342
LINC00950	0.903117	0.715212	0.401378	1.540795
TIPARP-AS1	0.903117	0.715233	0.392189	1.794966
TMEM9B-A	0.903117	0.715266	-0.50665	1.771726
SETX	0.903331	0.715509	-0.14901	8.296231
SMYD5	0.903359	0.715669	0.217793	4.167238
PCDHB4	0.903359	0.715677	0.457713	1.827117
NDUFV2-AS	0.903567	0.715916	-0.68874	1.010842
TRA2B	0.903659	0.716166	-0.37249	2.426402
HELZ	0.903659	0.716178	0.134987	7.514485
FAN1	0.903659	0.716208	0.371545	2.171827
NLK	0.903689	0.716305	0.161829	4.864608
MIR26A1	0.903698	0.716407	0.498438	0.538772
PLD6	0.903698	0.716458	-0.65302	2.054268
SLC35E3	0.903772	0.71659	0.233737	3.83772
CCNB1	0.90405	0.716921	-0.2336	4.880732
SNORA7B	0.90405	0.716957	0.160146	5.239197
POLA2	0.904065	0.717081	-0.20836	4.106652
U2SURP	0.904065	0.717306	-0.15025	7.704411
SNORD14B	0.904065	0.717333	-0.18346	5.287663
TMEM8A	0.904065	0.717367	0.175672	5.790789
MCM6	0.904065	0.717368	-0.18958	5.11259
ITPKB	0.904065	0.717421	-0.16426	6.01907
KLF12	0.904065	0.717591	-0.25219	4.318014
COL14A1	0.904065	0.717606	-0.25105	7.34898
NABP2	0.904065	0.717772	0.193186	3.939499
ERCC2	0.904065	0.717785	-0.27734	3.498107
SLC4A5	0.904065	0.717875	0.326193	2.754827
GGA2	0.904065	0.71792	-0.16535	6.185576
RAPGEF4	0.904065	0.717921	0.558469	0.710563
CASC10	0.90416	0.718091	-0.91391	0.910572
FNDC9	0.90416	0.718143	0.526538	1.250495
SLC8A1-AS	0.904297	0.718325	0.355061	2.354517
RASSF5	0.904519	0.718574	-0.52165	2.471122
RBMX2	0.904542	0.718666	-0.19433	4.703869

SRM	0.904936	0.719053	-0.21452	4.840578
ALG10	0.905012	0.719186	-0.45402	2.860643
PPT1	0.905163	0.719459	0.393412	1.694982
LRMDA	0.905163	0.719491	0.436725	2.53862
SCARNA28	0.905163	0.719574	-0.23345	5.000753
DLGAP4-AS	0.905163	0.719599	-0.40898	2.13633
TMEM97	0.905215	0.719714	0.249887	4.004185
PRKCE	0.905293	0.719878	-0.17022	4.901129
TTC21B	0.905293	0.719923	0.168795	4.897724
ADCY3	0.905758	0.720574	-0.17702	4.951996
STAB2	0.905758	0.720592	-0.41412	2.12433
TRPC5	0.905758	0.720626	-0.72919	0.792409
HSPE1	0.905758	0.720785	-0.43524	2.365232
TSPYL4	0.905758	0.720793	0.160552	5.656655
MAP3K2	0.905758	0.720801	0.136033	7.508529
GTPBP6	0.905758	0.720806	0.27845	3.151186
USP24	0.905775	0.721029	-0.14113	7.945523
SH3BP5	0.905775	0.721056	0.34669	2.203456
FAM136A	0.905775	0.721094	0.135016	5.640675
ZNF223	0.905775	0.721113	0.298894	2.972281
CHAF1A	0.905786	0.721242	-0.15069	5.689819
CTU1	0.905786	0.721269	-0.47438	2.115739
NCL	0.905901	0.72145	0.148979	10.25894
ZNF697	0.905901	0.721571	0.276576	3.583471
PNKD	0.905901	0.72158	0.280856	2.631073
SCAF1	0.906082	0.72183	0.144429	6.495309
CDYL	0.906082	0.721871	-0.43694	2.402828
ATG2B	0.906369	0.722173	-0.14467	7.217615
VPS26B	0.906424	0.72229	0.135987	6.136836
CNIH3	0.906485	0.722412	-0.75524	0.972333
SFPQ	0.906585	0.722566	0.129192	9.155289
FOXP2	0.906686	0.722739	-0.73457	1.750583
PAWR	0.906686	0.722793	0.137805	6.623924
TRIM66	0.906811	0.722982	0.147915	6.342343
MRPL43	0.906811	0.72304	0.401969	1.859768
POU5F2	0.906926	0.72333	0.275759	2.44926
MFAP1	0.906926	0.723399	-0.14802	6.580704
SGTA	0.906926	0.723458	0.140135	5.626468
XRCC2	0.906926	0.723516	0.40273	2.330107
ACKR2	0.906926	0.723521	-0.34894	2.214196
LINC01105	0.906926	0.723572	-0.97456	2.111699
INTS3	0.907113	0.723928	0.261853	3.578032
CAB39	0.907113	0.723936	0.200761	3.866564
MIR5047	0.907113	0.723981	0.140444	5.120564
HEATR5A	0.907113	0.724034	0.128646	6.231367

TTC30B	0.907113	0.724089	0.432035	1.937538
TMEM39B	0.907224	0.72425	0.2653	2.953364
LOC101928	0.907985	0.724932	0.525564	1.194159
FAM229B	0.907994	0.725013	0.42859	2.00267
HIP1	0.908443	0.72565	0.475619	1.020437
USP3-AS1	0.908443	0.725657	0.384057	2.396072
ADAMTS4	0.908443	0.725723	-0.38016	4.837891
ZNF674-AS	0.908443	0.725809	-0.41751	2.084569
FRMPD2B	0.908443	0.725844	-0.88646	0.964919
CCDC40	0.908443	0.725937	-1.04701	0.953135
DIP2A	0.908443	0.725952	0.3965	1.195924
FAM241A	0.908443	0.725996	0.176662	4.64995
BIN3	0.908443	0.726034	-0.17699	5.289081
SNORA5A	0.908519	0.72622	-0.19622	4.486861
NDUFA9	0.908519	0.726241	0.13191	5.550789
FLJ36000	0.908736	0.726559	0.754139	0.92714
TRAF7	0.908736	0.726562	-0.16193	6.167248
KLHL31	0.908775	0.726667	0.3361	1.666451
TXK	0.909565	0.72738	-0.44471	2.856839
ELOA2	0.909565	0.727476	0.51419	1.222842
ABLIM3	0.909565	0.727537	-0.53763	1.485166
LOC641367	0.909565	0.727593	0.363544	2.60911
LMNB2	0.909627	0.727717	0.135804	7.038757
SLF2	0.90977	0.727905	0.174697	4.837999
FBXO30	0.909781	0.728344	-0.17434	5.199279
KHDRBS3	0.909781	0.728395	-0.32085	2.596174
MRS2P2	0.909781	0.728395	-0.51968	2.243145
COX10	0.909781	0.728429	-0.19367	4.47966
DNAJC3	0.909781	0.728464	-0.14019	7.109188
9-Mar	0.909781	0.728568	-0.29387	2.914841
ZNF514	0.909781	0.728654	0.378438	1.400806
SNTA1	0.909781	0.728743	0.229897	3.554802
NPTXR	0.909781	0.728831	0.402224	1.937863
STIMATE	0.909781	0.72889	0.160239	4.408071
RNASEH2C	0.909781	0.728956	0.188105	4.065117
NRDE2	0.909781	0.728972	0.136308	5.148477
VCL	0.909781	0.728991	0.354865	2.174593
KTN1-AS1	0.909781	0.729023	0.480492	1.268453
NFIA-AS1	0.909781	0.729074	0.290363	2.825143
GRK3	0.909781	0.729104	-0.21765	4.791766
NSMCE1-D	0.909781	0.729166	0.389949	2.328584
LOC101927	0.909904	0.729409	-0.95879	0.571731
UNC13B	0.909904	0.729412	0.126941	7.474492
CFAP58	0.909905	0.729516	-0.4913	1.618865
ANAPC16	0.909905	0.729645	0.255586	2.902489

GLIDR	0.909905	0.729679	0.516001	0.537377
CSNK1E	0.909905	0.729707	-0.71541	1.765419
USP35	0.910104	0.730016	-0.18668	4.638501
FZD8	0.910104	0.73008	0.44003	1.245507
RAD50	0.910104	0.730088	0.125604	7.823251
CROCC	0.910313	0.73033	-0.1496	6.00079
TTC12	0.910441	0.730506	-0.24469	3.904761
PYROXD1	0.910618	0.73075	-0.2053	4.712914
SNORD103	0.910618	0.730932	0.24434	3.003666
NUP210	0.910618	0.730964	0.186169	5.269707
CCDC126	0.910618	0.731055	0.303545	3.311882
SNORA65	0.910618	0.731175	-0.20977	7.197745
DUS1L	0.910618	0.731208	-0.14912	5.936434
SPATS2L	0.910618	0.731223	0.548455	0.643829
HIST1H3H	0.910618	0.731456	-0.23123	3.751749
RANBP6	0.910618	0.731544	-0.35543	2.535873
EPHX3	0.910618	0.731567	-0.77107	0.490128
GJB3	0.910618	0.731604	-0.53132	2.037017
EIF5	0.910618	0.731688	0.288202	2.875665
FLRT1	0.910618	0.731704	-0.30695	3.965479
PDCD4	0.910618	0.731745	0.523667	0.67942
TSPYL5	0.910618	0.731755	0.379269	2.575184
TBC1D25	0.910732	0.732096	-0.20393	4.456843
MIR4296	0.910732	0.732107	0.32294	1.950115
RNF150	0.910732	0.732113	-0.41164	2.393934
SIRT1	0.910732	0.732141	0.279341	3.29618
BCL7C	0.910747	0.732278	-0.60659	1.496086
ZNF529	0.910747	0.732352	0.395965	2.170707
CLK3	0.910747	0.73244	-0.57652	0.923254
PCDH20	0.910747	0.73251	0.556791	1.122602
FTO	0.910747	0.73263	-0.15604	5.771127
FNIP1	0.910747	0.732658	0.458339	1.540376
TSC22D3	0.910747	0.732739	0.214757	5.453142
TMEM37	0.910747	0.732784	0.274647	2.999843
DNASE1	0.910747	0.732896	-0.20723	4.218913
CXCL16	0.910747	0.733099	-0.2841	3.651401
WDR20	0.910747	0.733135	-0.33857	3.134434
MACF1	0.910747	0.733187	-0.15104	10.02183
RGMA	0.910747	0.733239	-0.67616	0.893862
ZSCAN21	0.910747	0.733297	0.331397	2.125071
EIF4A2	0.910747	0.733328	-0.13945	8.541531
TSNAX	0.910747	0.733335	-0.15219	5.141743
KLHL2	0.910747	0.733477	-0.49393	1.231999
FBXO10	0.910747	0.733481	-0.31877	3.741306
ODC1	0.910793	0.733592	-0.70116	1.89058

TEN1-CDK3	0.911203	0.73417	0.416655	1.278005
TARDBP	0.911203	0.734197	-0.1329	7.833115
SOX18	0.911203	0.734255	0.386576	1.710491
ADCY10P1	0.911203	0.734306	-0.32034	3.553692
ARHGEF12	0.911203	0.734335	-0.71297	1.459956
GUCY1B2	0.911203	0.734567	0.394032	1.734167
SNORA10	0.911203	0.734646	-0.1714	6.029172
CPEB2-DT	0.911203	0.734677	-1.00638	0.469018
MTRF1	0.911203	0.734714	0.185761	4.08779
SUGP2	0.911203	0.734747	0.155677	4.950575
GTF2F1	0.911203	0.734757	-0.14402	6.62421
RPL23AP82	0.911203	0.734865	-0.76188	0.563193
ZIK1	0.911203	0.734886	-0.60504	1.803456
FAM89A	0.911203	0.734956	-0.42711	2.427534
RFFL	0.91122	0.735043	-0.51705	1.661904
RIOX1	0.911298	0.735181	0.237798	3.121388
OR52B6	0.911298	0.735282	0.513463	0.870646
ZFPL1	0.911298	0.735327	-0.21832	4.146993
GOLGA1	0.9113	0.735403	0.122334	5.936749
WNT2B	0.911358	0.735523	-0.72234	0.949
UNC13A	0.911462	0.735737	0.459948	1.469207
CCL22	0.911462	0.735862	0.286669	2.501623
GCN1	0.911462	0.735892	-0.13467	8.081201
ZNF777	0.911462	0.735929	0.250257	4.136595
PRICKLE2-A	0.911462	0.736053	-0.79541	0.631641
ZNF174	0.911462	0.736094	0.365079	1.609523
SNORD14D	0.911462	0.736124	-0.26005	4.403232
ZNRF2P1	0.911546	0.736326	0.24937	2.931348
ATXN7L2	0.911546	0.736378	0.28392	2.716778
MIR644A	0.911546	0.736414	0.453453	1.117516
ZNF575	0.911662	0.736604	0.309737	1.755727
LOC100996	0.911662	0.736712	-0.48789	1.630733
FAM241B	0.911662	0.736729	0.465442	1.192644
SH3GL1P1	0.911944	0.737031	-0.39243	2.602405
FAM160B2	0.911971	0.737126	-0.17527	6.036033
FMO5	0.91212	0.737321	0.438512	2.572769
RASIP1	0.91226	0.737769	-0.27494	3.597137
LOC101928	0.91226	0.737852	-0.41891	2.731974
GNAZ	0.91226	0.737856	0.396383	2.342069
NPIPB11	0.91226	0.737943	0.23305	2.905309
RPL6	0.91226	0.737946	0.409728	0.896413
ICE2	0.91226	0.737998	0.191342	3.899726
CYHR1	0.91226	0.738021	0.182431	3.982403
FASTKD3	0.91226	0.738052	-0.61714	1.380741
CHPF	0.91226	0.738106	0.296589	2.635112

MIR581	0.91226	0.738242	-0.71477	0.617545
RSRP1	0.91226	0.738246	0.122551	6.615157
SSBP3-AS1	0.912463	0.738485	0.256755	3.549544
ANKRD36C	0.912482	0.738574	0.163343	7.05095
KMT2B	0.912546	0.738767	0.12333	7.50617
C2orf69	0.912546	0.738774	-0.20117	4.635074
XPO4	0.912564	0.73891	-0.1455	6.086647
ACSL4	0.912564	0.738992	-1.1057	0.7945
UBAP1L	0.912564	0.73901	-0.50719	1.948961
UQCRBP1	0.912572	0.739132	0.393858	1.325849
KIAA1109	0.912572	0.739164	0.12422	8.578411
BACH1	0.912659	0.739308	0.221123	3.426866
USP12	0.912803	0.739618	-0.14082	5.844078
GBP4	0.912803	0.739642	-0.22354	5.123402
RNF44	0.912803	0.739647	0.125294	5.812533
RASA1	0.912831	0.739762	0.190164	3.637897
CFAP53	0.912831	0.739946	-0.50474	1.848282
EBF3	0.912831	0.739972	0.389276	1.483069
C16orf46	0.912831	0.740131	-0.81813	0.478508
ANGPTL8	0.912831	0.740142	0.30238	2.501758
GABPB1-IT	0.912831	0.740173	0.199187	4.093279
NOP16	0.912831	0.740188	-0.46777	2.236598
MXD3	0.912851	0.740278	-0.42158	1.376703
POLR3K	0.912925	0.740411	0.228354	3.042979
SNORD116	0.913142	0.740661	-0.2539	5.477744
SETD7	0.913534	0.741053	-0.13568	6.860787
KLRB1	0.913559	0.741147	0.434079	1.269837
LOC72809!	0.913585	0.741243	0.585139	0.93008
LAMTOR5	0.913729	0.741434	0.182269	4.622788
MOCS3	0.913964	0.741698	-0.2498	3.431775
PRSS48	0.914113	0.741893	0.186696	3.670283
C4orf46	0.914118	0.741971	-0.88683	1.066632
DDX59	0.914118	0.742121	0.152612	4.906019
CEBPG	0.914118	0.742183	0.329664	1.835755
ZNF646	0.914118	0.742205	0.137608	5.407404
SETD3	0.914118	0.742267	0.141769	5.326768
MFAP3	0.914195	0.742438	0.398689	2.160385
TMEM173	0.914195	0.742478	-0.57436	0.876914
MEG8	0.914448	0.742757	0.390923	1.59102
AQP6	0.914511	0.742883	0.589972	0.499738
TFEB	0.915199	0.743543	-0.64404	0.893352
MSTO2P	0.915199	0.74359	0.415377	0.9851
FAM86FP	0.9152	0.743665	-0.55408	1.380264
MLKL	0.91528	0.743831	0.213866	3.21439
H6PD	0.91528	0.743878	0.280365	2.630258

PEAK3	0.915352	0.744011	0.485374	1.269407
RALGDS	0.915369	0.744098	-0.3573	2.807136
C9orf84	0.915432	0.744247	0.483509	1.385593
OR5C1	0.915432	0.74437	0.46895	1.546057
CD1A	0.915432	0.744373	0.3509	2.052312
THUMPD3	0.915554	0.744546	0.396864	0.913219
LOC101927	0.915669	0.744762	-0.3035	3.584521
HNRNPAB	0.915669	0.744788	0.402271	0.93946
ATAD5	0.915806	0.744989	0.135525	5.36608
NFAM1	0.915806	0.745047	0.257288	2.99548
CSMD1	0.91592	0.745215	0.417182	2.217752
CENPI	0.915933	0.745369	0.265651	2.518907
MELTF	0.915933	0.745485	0.252337	2.629218
RNPC3	0.915933	0.745539	0.145013	5.322478
MAATS1	0.915933	0.74567	-0.6603	1.941602
PAK3	0.915933	0.745673	-1.11295	0.752291
FAM120C	0.915933	0.745723	0.394122	2.027151
CCZ1P-OR7	0.915933	0.745744	0.169191	4.25991
LINC00869	0.915992	0.745904	0.313147	2.110245
PIGV	0.915992	0.746044	0.484481	0.501348
WHAMMP3	0.915992	0.746106	0.397117	1.798978
LOC729970	0.915992	0.746127	0.51094	1.1282
LOC100132	0.915992	0.746194	0.48063	1.291342
BORCS6	0.915992	0.746237	-0.34406	3.070463
EEF2	0.916014	0.746374	0.120437	10.99727
NHLH2	0.916014	0.746428	0.456148	0.897374
APOBEC3G	0.916014	0.746565	-0.29841	3.309295
GPR55	0.916014	0.746595	-0.58576	1.180189
SMYD4	0.916014	0.746662	0.165896	4.455362
PIK3R4	0.916014	0.7467	0.145115	5.270312
MORC2-AS	0.916124	0.746864	0.392969	1.928416
SPOPL	0.916368	0.747166	0.125693	5.311609
FAM21EP	0.916368	0.747212	0.303158	1.378196
KIF26A	0.916396	0.747309	-0.47925	2.642122
COL6A6	0.916527	0.74749	-0.38319	2.72938
REST	0.916547	0.747581	-0.49979	1.567923
SRRM2	0.916721	0.747797	-0.13109	11.20558
ATP2B4	0.916779	0.748021	-0.57182	1.206079
LINC01410	0.916779	0.748085	-0.43469	2.617871
IL20RA	0.916779	0.748145	0.540323	0.899024
NDUFS3	0.916779	0.748158	-0.17902	4.733244
TECPR1	0.916779	0.748215	0.123079	5.878492
SMARCAD1	0.916967	0.748443	-0.43486	1.337464
NOB1	0.917047	0.748582	0.446569	0.810331
ATAD1	0.91706	0.748668	0.136755	6.394467

MIR133A1I	0.917327	0.74896	-0.24566	3.282268
RNF166	0.917333	0.749066	-0.6175	1.25867
RBM41	0.917333	0.749189	0.123085	5.680106
LRRC46	0.917333	0.749194	0.33283	1.926243
STRN3	0.917333	0.749286	-0.24052	3.431285
TMEM50B	0.917333	0.749388	0.438371	0.990985
USP22	0.917333	0.74941	-0.12931	7.480128
VPS26A	0.917383	0.749526	-0.30029	2.813183
ZNF528	0.917419	0.749653	-0.17585	4.348129
RAB8A	0.917419	0.749704	0.11997	6.921288
PFDN5	0.917471	0.74982	-0.40083	1.838845
LINC00894	0.917707	0.750088	0.135856	5.006719
CCZ1B	0.918385	0.75077	0.146079	5.292579
SNHG28	0.918385	0.7508	-0.38261	2.165638
ZNF32-AS2	0.918385	0.750898	0.328876	2.083981
TBCE	0.918385	0.750995	0.597953	0.959078
CYB5D2	0.918385	0.751014	0.321514	2.269236
ZNF322	0.918607	0.751271	-0.47854	1.688627
BIRC6	0.918607	0.75144	-0.12633	9.159316
FIGN	0.918607	0.751531	-0.40485	2.595386
MCOLN1	0.918607	0.751553	0.166939	3.942622
SYMPK	0.918607	0.751567	0.114811	6.459779
LOC221946	0.918627	0.751658	0.213977	4.291058
ZFP1	0.918734	0.751859	-0.21664	3.702462
CCT3	0.918734	0.751961	0.309729	2.279639
C8G	0.918734	0.751969	-0.88529	1.186781
ZNF192P1	0.919091	0.752336	0.294548	2.008635
NR3C1	0.919182	0.752555	-0.36019	3.152167
LOC389765	0.919182	0.752626	0.179457	3.808766
HIST1H2AJ	0.919182	0.752691	-0.21831	4.73366
SELENOO	0.919182	0.752708	-0.16991	4.414437
OSBPL10	0.919425	0.753155	0.331573	1.543206
PSMB9	0.919425	0.753198	0.478588	0.454458
BUD13	0.919425	0.75321	0.332777	2.010847
SPC25	0.919425	0.753266	0.368851	1.563117
SNORD5	0.919425	0.753403	-0.48243	2.410646
GPR183	0.919425	0.753411	0.149632	4.695461
NUTM2B-A	0.919425	0.753428	0.119737	5.743474
TMEM42	0.919505	0.753659	-0.39834	2.090625
LINC00853	0.919505	0.753805	0.423261	1.30897
RNU5A-1	0.919505	0.753811	-0.17551	9.116244
ELP1	0.919505	0.753921	0.121011	6.298477
SRFBP1	0.919505	0.753944	-0.17954	4.748529
CABLES2	0.919505	0.753983	0.264919	3.194424
LMBR1	0.919505	0.754079	0.120445	6.122386

ZNF451	0.919505	0.75409	-0.15072	5.481307
LOC101927	0.919585	0.754296	0.376541	1.776216
FAM45BP	0.919585	0.754304	-0.35181	2.69575
MDH2	0.919882	0.754814	-0.6695	0.840525
SH3D19	0.919882	0.75492	-0.47356	1.235304
IMPG2	0.919882	0.754939	0.369487	1.11662
PJVK	0.919882	0.754998	0.39656	1.470206
RHPN1	0.919882	0.755013	-0.35901	2.880324
HSPA5	0.919882	0.755034	-0.1367	8.929532
C1QL3	0.919882	0.755069	0.427614	1.477233
CYP2A6	0.920019	0.755257	0.418105	0.892903
LEF1	0.920019	0.755331	0.475085	0.669306
SLC47A1	0.920276	0.755616	0.38349	1.305378
FRK	0.920326	0.755835	0.117405	5.935453
TIMP4	0.920326	0.755851	0.48755	1.132745
DNAH9	0.920326	0.755916	-0.70713	1.603374
RCL1	0.920326	0.755962	0.389592	1.65668
RABL6	0.920326	0.75603	0.220504	3.290612
DBF4B	0.920495	0.756243	0.326232	1.824573
MIR4312	0.9208	0.7566	-0.79057	1.063601
SREK1	0.9208	0.756769	-0.18497	5.425345
MIR4774	0.9208	0.756873	-0.97556	0.350619
OSM	0.9208	0.756944	-1.15486	0.883088
ILVBL	0.9208	0.756948	-0.1406	5.713702
HNRNPM	0.9208	0.757014	0.416226	1.366335
ZNF343	0.9208	0.757081	0.471232	1.247897
CHD9	0.9208	0.757124	-0.65164	0.756225
PPM1D	0.9208	0.757165	0.126777	4.869677
ALMS1	0.920949	0.757362	0.121824	6.873104
GUSBP5	0.921071	0.757644	-0.64558	0.496335
MIR26B	0.921071	0.757653	0.328116	1.910512
LOC101927	0.921071	0.757747	-0.73671	0.869008
SNX19	0.921071	0.757761	-0.18005	5.011886
RNF20	0.921208	0.758007	-0.12504	7.126901
APOA1-AS	0.921208	0.758023	0.331469	1.358472
CCDC24	0.921439	0.758337	-0.28112	3.210729
DENND6B	0.921439	0.758416	0.223579	2.880267
WDR49	0.921439	0.758445	0.416874	1.805541
NEIL3	0.921439	0.758512	0.295006	2.621732
MIR4804	0.921567	0.758692	0.591574	0.492213
PSMG3	0.921808	0.759004	-0.42867	1.346402
ALPK3	0.921808	0.759166	0.16026	4.344676
SMARCC2	0.921808	0.759179	-0.35191	2.915331
KCNMA1	0.921808	0.759257	-0.67734	1.069731
ZNRF2	0.921808	0.759264	-0.12181	6.098364

SAMD9	0.92191	0.759423	-0.75883	0.628358
COG1	0.92191	0.759499	0.13556	4.939378
AACSP1	0.92191	0.759572	-0.96455	0.809492
YAE1	0.921971	0.759696	0.331783	2.071953
LOC100131	0.922043	0.759882	0.468676	1.669455
MYH3	0.922043	0.760069	-0.3294	3.094963
C19orf71	0.922043	0.76008	0.250422	2.276299
LAMTOR4	0.922043	0.760116	0.155867	4.203525
INF2	0.922043	0.760143	0.241382	2.738053
MRPS31	0.922043	0.760204	-0.14457	4.715386
OGFR-AS1	0.922094	0.760399	0.401057	0.873832
ARHGEF10	0.922094	0.760422	0.372046	1.235551
LOC100132	0.922094	0.76047	-0.32473	2.77267
LMCD1	0.922274	0.760732	-0.27978	2.862471
SNRPG	0.922274	0.760768	0.43835	1.057573
MYO15A	0.922378	0.76099	-0.4454	2.327819
TRMT13	0.922378	0.761065	-0.14259	4.868282
ETV6	0.922378	0.761078	0.114694	6.556197
LCNL1	0.922625	0.761474	0.434577	1.366673
FTH1P3	0.922625	0.7615	-0.2258	3.646176
EIF4E	0.922625	0.761509	-0.95856	0.507881
IKZF5	0.922625	0.76158	0.342413	2.019095
CDC42EP1	0.922727	0.761806	0.122832	6.739083
OGN	0.922727	0.761814	-0.82352	0.591248
DYNLL2	0.923299	0.762361	-0.13369	5.881445
GZMK	0.923444	0.762925	0.529436	0.516112
DUSP4	0.923444	0.762961	0.269244	3.809991
CCNB2	0.923444	0.762988	-0.24019	3.736307
LOC643387	0.923444	0.762992	0.375317	0.755955
CRAMP1	0.923444	0.763149	0.108505	6.356822
TSPO	0.923444	0.763176	0.550982	0.51231
SIAH1	0.923444	0.763179	0.234296	2.51917
SNORA94	0.923444	0.763313	-0.36421	2.608577
CYTH1	0.923444	0.763327	0.163571	3.840969
EFCAB2	0.923444	0.76335	0.474862	0.660479
UQCRC1	0.923444	0.763397	0.116187	6.601317
TRMT6	0.923444	0.763604	-0.64321	1.258929
NECTIN2	0.923444	0.763663	-0.13259	5.690735
MRPL35	0.923444	0.763732	0.182363	3.822633
XPNPEP3	0.923444	0.763842	-0.1269	5.854309
PCDHB10	0.923444	0.763885	0.373845	0.90689
FBXO33	0.923444	0.764069	0.156558	4.723729
SNTB2	0.923444	0.764154	0.117154	6.570341
SMA5	0.923444	0.764171	0.357359	1.295249
NRIP3	0.923444	0.764288	-0.48178	1.842881

TMEM182	0.923444	0.764373	0.317224	2.378163
CRYL1	0.923444	0.764463	0.137569	4.820451
CCBE1	0.923444	0.764597	-0.53985	2.522154
FLAD1	0.923444	0.764603	0.215648	2.441605
FAM120A	0.923444	0.764609	0.238575	3.358792
RPL23AP53	0.923444	0.764649	-0.16049	4.988845
GDAP2	0.923444	0.764655	-0.14101	5.790959
MSS51	0.923444	0.764667	-0.34762	2.729414
RAB11FIP5	0.923444	0.764714	-0.16714	4.615914
LINC00940	0.923444	0.764725	0.459642	1.016184
SLC8B1	0.923535	0.765064	0.155137	5.02653
CISD1	0.923535	0.765204	-0.25035	3.692197
FUT5	0.923535	0.765222	0.409353	0.737045
NPEPL1	0.923535	0.765283	-0.56481	1.000618
MIIP	0.923535	0.765317	0.191924	2.980424
ARL11	0.923535	0.765327	-0.36715	2.636289
NFYC	0.923535	0.765394	0.319689	1.978738
LOC102724	0.923535	0.765683	0.503172	0.503397
PRAF2	0.923535	0.765722	-0.23517	3.342728
GTF2H2B	0.923535	0.765776	-0.48718	0.936937
FLJ42393	0.923535	0.765796	0.165733	4.46106
RRNAD1	0.923535	0.765816	-0.67578	1.013223
GPAT4	0.923535	0.76584	0.108361	6.385892
CARD6	0.923535	0.765909	0.118319	6.086763
PTGFRN	0.923535	0.765962	0.111608	7.657018
NGLY1	0.923535	0.765997	0.383366	1.602002
SNORD111	0.923616	0.766147	-0.18952	4.46357
SULF2	0.923616	0.766214	-0.79897	0.743735
FAM227B	0.92368	0.766399	0.430641	0.969717
REXO1	0.92368	0.766416	0.115407	5.53338
CUTC	0.923798	0.766705	0.214281	3.54689
NUTM2A	0.923798	0.766981	-0.51388	1.879268
USP13	0.923798	0.766998	0.165132	4.728532
ORMDL1	0.923798	0.76711	-0.49446	1.448829
SNORD116	0.923798	0.767159	-0.2128	6.473297
NSD1	0.923798	0.767179	0.211665	3.465572
11-Sep	0.923798	0.767221	0.384504	1.430943
RPS29	0.923798	0.767348	-0.27113	3.34317
SNORA3A	0.923798	0.767353	-0.15004	7.103529
SNORD21	0.923798	0.767415	-0.15725	6.332555
LRCH3	0.923798	0.767456	0.114498	5.552289
BMP7-AS1	0.923798	0.767463	-0.47298	1.749912
ZNF214	0.923798	0.767487	0.343797	1.794104
CENPQ	0.923849	0.767614	0.338519	2.065915
PDCD1LG2	0.923849	0.76768	0.313824	1.678982

FAM3B	0.923849	0.76788	0.354774	1.015073
TCF4	0.923849	0.767923	0.26854	2.298141
C8orf76	0.923849	0.768056	0.353605	1.180495
KATNB1	0.923849	0.768068	-0.14686	4.429963
NDEL1	0.923849	0.768078	-0.43596	2.429985
LINC01481	0.923849	0.768169	-0.63311	0.504257
ZNF808	0.923849	0.768203	0.117783	5.596434
PALD1	0.923851	0.768279	-0.20641	3.801899
FBXO9	0.923858	0.768391	-0.36647	2.604123
LGALS9B	0.923858	0.768475	-0.41322	2.483182
METTL18	0.923858	0.768509	-0.31158	2.428199
TFAP2E	0.924314	0.768964	-0.46695	1.762497
TBX19	0.924496	0.769191	0.218805	3.074197
ABHD12	0.924503	0.769337	-0.15002	4.782917
TMEM267	0.924503	0.76938	0.240616	2.703151
IDI2	0.924503	0.769442	0.362905	1.468242
CHIAP2	0.924503	0.769495	-0.86174	1.073193
DDX6	0.924511	0.769577	0.416974	0.845884
HIST1H4I	0.924597	0.769781	0.256234	2.50047
F12	0.924597	0.769799	0.202517	2.548339
RABEP2	0.924833	0.77014	0.135264	5.174681
C16orf91	0.924833	0.770145	-0.41125	1.951806
TMCO4	0.925224	0.770545	0.128018	5.08125
LINC01560	0.92526	0.77079	0.288567	1.952001
NADK2	0.92526	0.770791	0.293009	1.696299
ARGLU1	0.92526	0.770829	0.120505	8.199602
ZNF891	0.92526	0.770932	0.182342	3.174846
GKN2	0.92526	0.770951	0.427501	2.750547
TIGD6	0.92526	0.771131	0.428364	0.54348
GALK1	0.92526	0.771167	0.161951	3.490144
RRP36	0.92526	0.771175	0.144732	4.794475
USP54	0.925274	0.771438	-0.11988	7.365933
MRPL51	0.925274	0.771446	-0.13848	6.198266
FAM20B	0.925274	0.771567	-0.12545	6.333207
LRRC1	0.925274	0.771606	-0.12944	6.143919
SWSAP1	0.925274	0.771677	-0.65338	0.431871
EP400	0.925274	0.771701	-0.11732	7.964655
POU5F1P5	0.925274	0.771711	0.25463	2.173044
LINC01355	0.925313	0.771826	0.144655	4.480088
HNRNPA1F	0.925313	0.772016	0.107835	7.855619
EXOG	0.925313	0.772073	0.464499	0.971733
LYRM1	0.925313	0.772307	-0.56949	0.946389
NDUFS7	0.925313	0.772312	-0.18513	4.287268
SNORD77	0.925313	0.772401	-0.46191	1.15287
LINC00467	0.925313	0.772422	-0.43461	1.532433

ZFYVE9	0.925313	0.772494	0.367131	0.769197
GMPR	0.925313	0.772625	-0.43945	2.887754
CITED4	0.925313	0.772638	0.158835	3.57211
RPL21P28	0.925313	0.772669	-0.13847	5.713002
SS18L2	0.925313	0.772795	0.220287	2.89744
SMAD3	0.925313	0.772831	-0.2406	3.903392
IGF2R	0.925313	0.772834	0.102483	8.690645
UCHL3	0.925313	0.772888	0.307092	1.462779
MAPK13	0.925313	0.772979	-0.60288	1.033431
ZBTB20-AS	0.925313	0.773018	-0.38402	2.421517
ZNF236	0.92541	0.773174	0.359343	1.110248
UBE2F	0.925433	0.773282	0.342705	1.396515
C9orf40	0.925433	0.773343	0.246844	2.757023
SSC4D	0.925655	0.773623	-0.63546	1.211801
JAK2	0.925655	0.773678	-0.12751	7.118632
CDC34	0.925684	0.773813	0.121453	4.955531
PTRHD1	0.925684	0.774005	0.238643	2.603065
RAB27A	0.925684	0.774024	-0.38372	2.383371
THAP8	0.925684	0.774035	0.202468	2.781569
PI4KA	0.925684	0.774078	0.103348	6.83081
TNRC18P1	0.925832	0.774276	-0.35172	2.377127
PHIP	0.925938	0.77444	-0.12055	8.127438
SLC35G6	0.926077	0.774684	-0.59996	0.707765
MIR6504	0.926077	0.774706	-0.76242	1.000104
IQCB1	0.926143	0.774837	-0.32864	2.570283
TPH1	0.92617	0.77499	0.442041	1.566446
SNORD36A	0.92617	0.775009	0.140312	5.782621
CCDC18	0.926209	0.775117	-0.6642	1.111204
CLDN18	0.926294	0.775263	0.333542	2.710212
SUGT1P3	0.926441	0.775461	0.352407	1.386388
CAPN2	0.926611	0.775743	-0.23202	4.097475
CASC19	0.926611	0.775787	-0.35823	2.847215
GLTPD2	0.926611	0.775829	0.239071	2.688431
TRIM4	0.926629	0.775918	0.292356	2.310018
SCARNA6	0.926733	0.776142	0.112368	5.985868
HSPA13	0.926733	0.776217	-0.262	4.358385
HM13	0.926733	0.776231	-0.20656	4.61435
LMBR1L	0.92676	0.776361	0.398165	1.255365
ISOC1	0.92676	0.776524	0.194348	4.396314
ESPNP	0.92676	0.77655	0.433589	1.281216
STAR	0.92676	0.776588	0.321437	1.793493
SPRED2	0.92676	0.776681	0.237345	2.179952
ROS1	0.92676	0.776704	0.439393	1.180879
ABCB10	0.926868	0.776896	0.116257	4.792143
SNORD142	0.926868	0.77705	-0.60237	1.287395

ETV3	0.926868	0.777111	-0.16789	4.599858
CLDND2	0.926868	0.777155	0.371049	0.737998
SNORD140	0.926868	0.777174	-0.45453	1.108363
SRP72	0.926868	0.77726	0.203872	2.39787
TSHZ1	0.926868	0.777319	0.228345	2.403577
MIR3064	0.927118	0.777662	0.177972	3.437945
MIR6069	0.927118	0.77768	-0.65229	1.269816
SNORD18A	0.927407	0.777997	0.412218	0.737165
KIAA2026	0.927416	0.77808	-0.11335	7.331555
GAMT	0.927507	0.778366	-0.52615	1.423324
ZNF341-AS	0.927507	0.778393	-0.79545	1.248249
FPGS	0.927507	0.778422	0.354372	1.395497
CAPN12	0.927507	0.778566	0.181821	3.821802
UGT1A6	0.927507	0.778657	0.19455	4.127075
NDUFB2	0.927507	0.77873	-0.15795	4.325939
ERFE	0.927507	0.778734	0.367859	1.632951
HOXA-AS2	0.927507	0.778801	-0.5989	1.026979
ENDOG	0.927507	0.778833	-0.51086	1.156073
PDLIM7	0.927521	0.778919	0.31806	1.802006
AK7	0.927558	0.779026	0.181529	3.176716
ADAL	0.927611	0.779145	0.302581	1.701802
NOL6	0.927836	0.77947	0.151726	3.720045
PTCH2	0.927836	0.779524	0.137134	6.945573
TVP23A	0.927836	0.77956	-0.33219	2.566477
USP45	0.927941	0.779797	0.169367	5.162905
ATP2C2	0.927941	0.779798	0.24524	2.473223
CREBBP	0.927944	0.779969	0.37057	0.899899
SRBD1	0.927944	0.780165	-0.12785	5.238842
DZANK1	0.927944	0.780237	-0.36187	1.756318
FGGY	0.927944	0.780309	-0.47484	1.599334
TBC1D32	0.927944	0.780317	0.480273	0.577089
EIF4G3	0.927944	0.780382	-0.25994	3.232421
SERPIND1	0.927944	0.780384	0.227698	3.126618
FAM214A	0.927944	0.780402	0.163739	3.383012
PELO	0.928024	0.780546	-0.16771	4.772568
MBTPS2	0.928024	0.780684	-0.15587	4.441822
CACNG4	0.928024	0.780695	0.358623	0.908947
SPATS2	0.928197	0.781259	0.274258	2.187668
GPRC5D	0.928197	0.781372	-0.34569	2.758242
PCDHB8	0.928197	0.781399	-0.68014	1.296389
LOC101928	0.928197	0.781418	-0.58992	0.987952
MAML1	0.928197	0.781517	0.098842	6.177328
CEP295	0.928197	0.781582	-0.12369	6.300165
CYREN	0.928197	0.781657	-0.69781	1.361778
RNF170	0.928197	0.781693	-0.61319	0.871012

AKAP7	0.928197	0.781708	-0.42674	2.328984
IGBP1	0.928197	0.781955	0.109279	6.099332
MIR3916	0.928197	0.781975	0.174191	3.553005
WNT8B	0.928197	0.782169	0.406541	0.831664
TSC22D2	0.928197	0.782347	0.344125	2.509621
LOC64602‹	0.928197	0.782432	0.439569	0.427499
VPS36	0.928197	0.782463	0.305929	1.154773
PKIA-AS1	0.928197	0.782501	-0.43549	2.310178
LRRC8A	0.928197	0.782525	-0.58994	2.017509
CCDC7	0.928197	0.782576	-0.18935	4.240908
DAZAP2	0.928197	0.782582	-0.40927	2.693457
AKAP5	0.928197	0.782583	0.207583	2.828609
KIF24	0.928197	0.782601	-0.20311	3.750357
SLC35E4	0.928197	0.78261	-0.43593	2.290597
OR10A5	0.928197	0.782622	-0.76643	0.901865
NF1	0.928197	0.782645	0.330451	1.484634
ZNF814	0.928424	0.78295	-0.13109	5.427889
ZNF727	0.928424	0.782987	0.409557	0.677763
TTTY14	0.928455	0.783087	-0.81926	0.949245
EXOSC8	0.92851	0.783246	0.173476	3.876033
C11orf96	0.92851	0.783285	-0.3889	3.496712
FANCC	0.928711	0.78353	-0.31852	3.344272
ING3	0.928759	0.783769	-0.20179	3.384847
EPC1	0.928759	0.783802	-0.31425	2.033413
ARHGEF18	0.928759	0.783914	0.252425	2.159275
ALYREF	0.928759	0.783936	-0.12861	4.888907
ALG12	0.928759	0.78397	0.154643	4.17145
CAPN10-D1	0.928759	0.784021	0.21214	2.967382
RNASEH2B	0.928892	0.784209	-0.36432	1.95417
ST8SIA6-AS	0.928893	0.784353	-0.84939	1.197004
LOC10272‹	0.928893	0.78436	-0.16297	3.795991
UBE2E3	0.928975	0.784574	-0.43172	1.738155
SLC4A11	0.928975	0.78458	0.203705	2.636536
CDK2AP1	0.929209	0.784913	-0.55544	0.997706
CMTR1	0.929209	0.785095	0.114982	6.057082
LOC10012‹	0.929209	0.785157	-0.51773	1.26858
DPYD-AS1	0.929209	0.785174	0.308691	1.393799
RNPS1	0.929209	0.785206	-0.31784	2.547091
SEMA4C	0.929209	0.785229	-0.14611	4.814973
PRKACA	0.929225	0.785358	-0.32865	2.488117
FAM66C	0.929225	0.785479	0.351445	1.396372
LOC10144‹	0.929225	0.785509	-1.10666	0.660334
SLC2A3	0.929225	0.785543	-0.21829	5.040774
BTBD2	0.929296	0.785824	-0.12256	5.828208
CERS5	0.929296	0.786154	-0.30234	2.86276

DYNLRB1	0.929296	0.786291	0.443396	0.685274
RNU5B-1	0.929296	0.78632	-0.15562	8.504923
CNTD1	0.929296	0.786344	-0.47371	1.983813
P2RX5-TAX	0.929296	0.786384	-0.51123	1.274307
CASTOR2	0.929296	0.78646	-0.12021	5.248073
LOC100131	0.929296	0.78665	0.193428	2.900944
HIST3H2BB	0.929296	0.786707	0.224163	2.534786
SLC44A2	0.929296	0.7868	-0.33987	2.86177
RAB30-AS1	0.929296	0.786818	0.176942	3.813089
DTX1	0.929296	0.786946	-0.27999	3.164022
ESYT3	0.929296	0.787003	0.228405	2.682303
USP51	0.929296	0.787025	-0.65932	0.690427
FAM72B	0.929296	0.787297	0.446644	0.507671
C17orf67	0.929296	0.787351	0.316809	1.574454
FNDC8	0.929296	0.787376	-0.60082	1.464604
FDX1	0.929296	0.787413	0.118241	4.878624
SCAP	0.929296	0.787439	0.105862	6.399951
IVNS1ABP	0.929296	0.787472	-0.12107	7.75642
SNORD116	0.929296	0.787534	-0.16497	5.096067
GLRX3	0.929296	0.787542	-0.5383	1.316459
LOC399715	0.929296	0.787611	-0.35065	3.139878
TCF4-AS1	0.929296	0.787686	-0.67971	0.895523
LINC01114	0.929296	0.787804	0.472119	0.615319
MRPL41	0.929296	0.787823	0.167007	4.611647
FLOT1	0.929296	0.787827	0.22286	3.863193
SNORD92	0.929296	0.787849	0.176365	2.934679
SCYL3	0.929296	0.787925	-0.57087	0.706688
PABPN1	0.929296	0.787933	0.114319	5.299873
RAD54L	0.929296	0.787937	-0.60448	0.563014
NPTX2	0.92952	0.788202	-0.5489	0.746436
SNORD49A	0.929557	0.788308	0.183977	2.836703
WWP1	0.929557	0.788384	-0.10812	6.893837
MAP3K5	0.929822	0.788684	-0.12194	6.63106
ZNF33A	0.929982	0.788895	-0.35879	2.478333
ANGPTL1	0.930262	0.789216	0.134053	5.172331
GALNT11	0.930262	0.789283	-0.29145	2.649658
LIMD1	0.930455	0.789523	0.113611	5.392789
TAF11	0.93089	0.789997	-0.48854	1.501532
LRPAP1	0.93089	0.790072	-0.22141	4.30642
SNTB1	0.93089	0.790118	-0.30094	4.517916
ADO	0.930891	0.790194	0.146731	4.120362
APOBEC2	0.930938	0.79031	0.243843	2.070404
MANEA	0.930976	0.790438	0.127238	4.86334
LINC01721	0.930976	0.790492	-1.23495	0.65771
ZNF204P	0.931239	0.790791	-0.42936	1.917587

PTPRCAP	0.931556	0.791136	0.184785	3.210859
NLGN4Y	0.931612	0.791305	-0.65253	1.063702
CCNA2	0.931612	0.791335	0.13041	4.291468
ATP1A1-AS	0.931972	0.791715	0.2503	2.360436
IGFN1	0.932113	0.791936	0.255253	2.086626
ZNF35	0.932113	0.791987	-0.20838	3.666319
IFNGR1	0.932136	0.792131	-0.12235	6.31453
STX18	0.932136	0.792161	0.116739	5.199785
ACSL3	0.932136	0.792232	-0.6991	1.070053
LOC100505	0.932357	0.792496	0.423038	0.558826
WDR4	0.932402	0.79261	-0.98951	0.726652
SLC4A2	0.932634	0.792933	0.273843	2.032109
HCAR1	0.932634	0.793033	0.385533	1.011892
SNORA28	0.932634	0.793033	-0.2022	3.654938
ANKRD49	0.932787	0.793268	-0.16515	4.592624
GTF2B	0.932787	0.793315	-0.12281	4.864128
GRAMD1C	0.932809	0.793409	0.251674	1.644035
TBC1D5	0.933015	0.79366	0.160475	3.389743
ACAD10	0.933085	0.793803	0.340876	0.873956
BCCIP	0.933085	0.79387	0.16024	3.832984
SLC26A10	0.933133	0.794033	0.343116	0.954798
DDA1	0.933133	0.794077	0.114628	4.658012
NEDD4L	0.933133	0.794138	0.164586	3.695779
LNX1-AS1	0.933628	0.794635	-0.449	1.943526
DGKZ	0.934051	0.795198	-0.59856	0.741723
RNPEPL1	0.934051	0.795222	0.102566	5.889752
ASH1L-AS1	0.934051	0.795222	0.170064	3.142185
ZNF644	0.934206	0.795429	-0.117	6.311357
ISCA1	0.934255	0.795651	0.135288	4.258378
ZNF407	0.934255	0.795684	-0.1868	3.461078
CACNB2	0.934255	0.795698	0.381754	0.990631
TBC1D22A-	0.934285	0.79581	-0.23561	4.40331
NIPA2	0.934285	0.795921	0.335849	1.528499
TDRD7	0.934285	0.79595	0.255412	2.086534
CHRNG	0.934291	0.796031	0.224142	2.829812
RABIF	0.934366	0.796171	0.218514	3.706472
BAZ1B	0.934392	0.796269	0.091699	7.985278
HYAL1	0.934401	0.796373	0.260322	1.469457
HLA-L	0.934401	0.7965	-0.19265	3.35416
LOC102724	0.934401	0.796506	-0.43479	1.464213
CCNT2-AS1	0.934401	0.796632	0.293846	1.835448
NRG1	0.934401	0.796736	-0.45273	2.620771
DNAH14	0.934401	0.796821	-0.25465	3.361044
KMT5B	0.934401	0.796855	0.301721	1.602224
ARL14EP	0.934401	0.796882	0.189643	3.578372

GAK	0.934481	0.797026	0.190885	3.537842
LOC101927	0.934716	0.797309	-0.71252	1.227637
HEXD	0.934716	0.797378	0.152522	3.715101
TCAP	0.934821	0.797727	0.30571	0.776595
RHBDL3	0.934821	0.797731	0.324712	1.620009
CLASP2	0.934821	0.797772	-0.14298	4.721682
SNORD116	0.934821	0.797787	0.234636	2.649221
ZNF137P	0.934821	0.797973	0.194479	2.634557
MXRA7	0.934821	0.797979	-0.27574	3.629297
LOC100129	0.934821	0.798005	0.174571	3.336388
TUBGCP2	0.934821	0.798073	0.317634	1.385071
SLC22A23	0.934848	0.798172	0.159532	3.665771
AHCY	0.934887	0.798281	0.368736	0.963979
RBL1	0.934897	0.79842	-0.40163	2.142846
PTK2	0.934897	0.798473	0.330958	0.964634
C2CD6	0.934897	0.798517	-0.62832	1.31511
KLHDC3	0.935581	0.799311	-0.47206	0.924166
BAZ2A	0.935581	0.799459	0.289155	1.44763
EAF2	0.935581	0.799464	0.247838	2.476465
BCAR3	0.935581	0.799669	-0.31978	2.358558
LIPT2	0.935581	0.799793	-0.51931	0.962938
NHLRC2	0.935581	0.799848	-0.11631	6.229371
TMEM69	0.935581	0.79987	-0.19308	3.913878
CYP27B1	0.935581	0.799886	-0.45962	1.077951
RMI1	0.935581	0.799951	-0.15112	4.115393
TIMM8B	0.935581	0.799987	0.341757	0.901907
SNORD46	0.935581	0.800052	0.116687	6.595212
ALOX12P2	0.935581	0.800054	-0.30834	2.587686
POF1B	0.935581	0.800213	0.118741	6.876647
IKBKB	0.935581	0.800223	0.293565	0.703311
LOC100996	0.935581	0.800643	0.254225	2.624427
KBTBD7	0.935581	0.800756	0.161129	3.700634
CDC42EP4	0.935581	0.800758	-0.13663	6.001001
NTHL1	0.935581	0.800758	0.252855	2.442484
LINC01336	0.935581	0.800773	-0.57507	1.660116
LOC102723	0.935581	0.800828	0.275023	0.939086
WASH2P	0.935581	0.800868	0.176792	3.954736
ABRACL	0.935581	0.800915	-0.18163	4.132594
SNORA8	0.935581	0.800957	-0.12821	5.952634
SLC25A5-A	0.935581	0.801018	0.239543	2.241646
ZNF815P	0.935581	0.801032	-0.42259	1.892595
RBM44	0.935581	0.801071	0.207956	3.227781
PTPRD	0.935924	0.801504	-0.52236	0.676884
NEFM	0.935924	0.801516	-0.69538	0.451537
ADHFE1	0.936048	0.801698	-0.47596	2.998335

SNORA32	0.936155	0.801913	0.114961	4.722932
IDS	0.936155	0.802012	-0.52115	1.097158
NDUFB2-A!	0.936155	0.802057	0.283645	2.469823
DNAJC3-DT	0.936155	0.802093	-0.42955	2.141804
PIGH	0.936158	0.802273	0.223454	2.466456
COL6A4P2	0.936158	0.80231	0.342021	1.640154
PPIG	0.936158	0.802327	-0.10467	8.087399
MED10	0.936158	0.802399	-0.2307	3.827047
RPL31	0.936415	0.802696	-0.32677	3.082913
ERLIN2	0.936536	0.802875	0.095594	5.988941
ASPDH	0.936785	0.803209	-0.39321	1.668731
WHAMM	0.936785	0.803259	0.088437	5.628312
DNASE2	0.936785	0.803316	-0.16781	4.046015
TIMM17A	0.936937	0.803523	-0.14267	4.601825
ZNF574	0.937322	0.803929	0.134455	3.9924
AGAP1	0.937555	0.804205	0.256196	1.84211
MID1IP1	0.937565	0.804289	0.176044	2.785915
NDUFA7	0.937611	0.804558	-0.14739	4.235601
SNHG12	0.937611	0.804576	0.124857	4.256708
PSMC5	0.937611	0.804672	0.26914	1.629111
CCDC71	0.937611	0.804689	0.173754	2.907611
HIF1AN	0.937611	0.805069	-0.10497	6.822762
BRPF3	0.937611	0.805097	0.094412	6.328103
RBM33	0.937611	0.805105	-0.10275	8.419523
FGD3	0.937611	0.805133	-0.53521	1.125099
HRAT92	0.937611	0.805181	0.235772	1.812632
TUBGCP3	0.937611	0.805206	0.273902	2.154505
MPO	0.937611	0.805259	-0.99008	1.238814
ORAI3	0.937611	0.805285	-0.26116	3.349148
BRIP1	0.937611	0.805455	0.153374	4.22245
SLFNL1-AS:	0.937611	0.805487	0.197581	3.259259
ZNF587B	0.937611	0.805552	-0.09936	5.796982
TOMM34	0.937611	0.80558	0.139812	4.037716
ARMCX1	0.937611	0.805669	0.199614	2.679378
CA14	0.937611	0.805722	0.269042	1.816164
ZBTB3	0.937611	0.805821	0.197996	2.841482
CYTOR	0.937611	0.805848	0.190854	2.804761
CLEC19A	0.93781	0.806161	0.435019	0.846525
SMIM10L1	0.93781	0.80617	-0.15568	4.181994
DHX38	0.938049	0.806505	-0.09975	7.243728
ERBB3	0.938049	0.806527	-0.10681	8.746058
PTOV1-AS1	0.93822	0.806751	-0.17985	3.835472
PRKAR1A	0.938254	0.806856	0.193155	1.996565
NOC4L	0.938294	0.806966	-0.22174	3.449351
BOD1L1	0.938399	0.807186	0.087257	8.345562

LINC00921	0.938399	0.807208	0.173486	2.558995
CLHC1	0.938465	0.807341	-0.40628	2.281437
NISCH	0.93904	0.807924	0.08617	5.846593
UBE2O	0.93904	0.808016	-0.10413	6.048482
ZBED4	0.93904	0.808064	-0.10711	5.956011
SHF	0.939133	0.808269	0.221449	2.44261
CENPC	0.939133	0.808354	-0.11386	6.185416
TSC22D1	0.939133	0.808373	-0.17898	5.112181
PKNOX2	0.939277	0.808572	-0.53942	1.61345
LOC101929	0.939311	0.808717	-0.44504	1.576292
DPRXP4	0.939311	0.808754	-0.39273	1.734257
KL	0.939468	0.808983	-0.3479	2.242062
GADD45GI	0.939468	0.809067	-0.12898	5.055406
SAMD11	0.939468	0.809118	-0.42185	2.10946
CLBA1	0.939485	0.809208	-0.18915	3.245465
CNST	0.939649	0.809426	-0.12798	4.658895
MASP2	0.939873	0.809695	-0.26782	3.083809
SNORD88C	0.939956	0.809842	0.232565	1.023449
HIST1H2AL	0.940042	0.810024	-0.19759	3.74787
WASH8P	0.940042	0.810069	0.128411	4.27218
TECPR2	0.940222	0.8103	0.145304	3.453525
PIGO	0.940551	0.810769	0.204272	2.096174
NCBP1	0.940551	0.810807	0.087335	6.474246
PAPOLA	0.940551	0.810812	-0.28973	2.432902
TASP1	0.940689	0.811007	0.118535	4.601127
PLEKHM3	0.940836	0.811395	-0.22674	4.827988
PWAR5	0.940836	0.811405	0.143599	4.441666
STARD7-AS	0.940836	0.811479	-0.1902	3.166316
PNISR	0.940836	0.811523	0.292958	1.014915
PAQR6	0.940836	0.811645	-0.4661	1.540528
CBX8	0.940836	0.811674	-0.27281	2.459015
CEP250	0.940836	0.811722	-0.09612	7.246759
CASC6	0.940836	0.811821	0.383936	1.019895
FBXW2	0.940836	0.8119	0.085444	7.034184
BMS1	0.940836	0.811934	-0.09904	7.168119
GRSF1	0.940836	0.811981	-0.32714	1.829881
MRNIP	0.940836	0.812065	0.317751	0.607964
TNRC6B	0.940836	0.812125	0.141123	3.801226
CEP104	0.940902	0.812321	0.082162	5.965069
KANK3	0.940902	0.812335	0.206053	2.71454
LRIG3	0.940902	0.812475	0.184774	2.971834
ATPAF1	0.940902	0.812522	-0.98919	0.750846
EPM2AIP1	0.940902	0.812563	0.091657	5.799684
TRAF3IP2-A	0.941	0.812782	0.196459	2.656596
ZFYVE28	0.941	0.8128	-0.51447	0.701494

TMEM132I	0.941117	0.813052	0.370514	0.493414
MAIP1	0.941117	0.813053	0.112703	3.989055
EFCAB7	0.941331	0.813314	0.223293	2.878043
MRPL57	0.941565	0.813654	-0.15184	4.237262
SCG2	0.941565	0.813669	0.38277	0.763696
RBM28	0.941644	0.813863	0.168997	3.30063
LMF2	0.941644	0.813915	-0.11879	5.102889
CATSPERG	0.941644	0.813966	-0.29818	3.119759
CSNK2A1	0.941818	0.814217	-0.69684	0.60809
INSYN1	0.941818	0.81431	-0.47223	1.202727
TMEM94	0.941818	0.814345	0.088323	6.088894
NEDD9	0.941821	0.814498	-0.33137	2.558855
ARL6IP6	0.941821	0.814542	0.171723	2.861963
ARL15	0.941821	0.814577	0.12755	4.233467
SNORA48	0.941881	0.814705	-0.11528	7.551477
E2F1	0.942027	0.814908	-0.2828	2.940524
SLC38A2	0.942028	0.815117	-0.43709	1.994486
DSCAML1	0.942028	0.81518	0.257127	1.963371
CFAP126	0.942028	0.815378	-0.14102	4.617228
RBM25	0.942028	0.81546	-0.10686	9.214408
SACM1L	0.942028	0.81549	-0.10131	6.230418
UBE2Q1-AS	0.942028	0.815563	-0.52008	1.407487
GTPBP2	0.942028	0.815637	0.135742	3.869393
LINC01585	0.942028	0.815701	-0.7861	0.616054
SNORA9	0.942028	0.815726	-0.2339	3.329585
SIPA1L2	0.942028	0.815767	0.087805	7.974749
SENP2	0.942028	0.815873	-0.09847	6.068749
DPH6	0.942028	0.815882	0.170663	2.729105
SNORD87	0.942028	0.815901	0.100676	7.540623
SETBP1	0.942253	0.816172	-0.14625	5.563421
GET4	0.942331	0.816315	0.094613	5.549976
ZNF14	0.942568	0.816835	0.190872	3.213687
NUFIP1	0.942568	0.816836	0.113755	3.950776
ZSCAN25	0.942568	0.816852	0.102986	4.536577
CPLX1	0.942568	0.816923	-0.54533	0.90324
GPATCH3	0.942568	0.816927	0.102921	4.473632
DBH	0.942568	0.816988	-0.91372	0.7068
VAMP5	0.942568	0.817055	-0.23965	2.948461
ANKRD55	0.942632	0.817187	0.302472	1.386839
DNAJC13	0.942786	0.817619	-0.09498	7.320048
ALG1L9P	0.942786	0.817641	-0.65691	0.928618
STK38L	0.942786	0.817649	-0.10451	6.977432
LOC441666	0.942786	0.817664	0.222069	2.44037
MYO1H	0.942786	0.817702	-0.46463	1.163828
CCL19	0.942878	0.817859	0.374489	0.714413

MAP3K13	0.943092	0.818121	0.125893	4.42439
RPL13AP3	0.943159	0.818255	0.278801	0.673822
ZC3HAV1L	0.943587	0.818836	0.136429	3.744812
SNORD84	0.943587	0.818849	0.144444	3.359858
TTC39C	0.943587	0.818896	0.179977	2.822132
UBE2V1	0.943587	0.819007	0.260562	1.578559
HTR6	0.943587	0.819066	-0.64599	0.512332
SLC38A7	0.943587	0.819151	0.220391	1.55495
ANKRD62	0.943587	0.819161	-0.28837	2.723655
MCCC2	0.943682	0.819347	0.078927	5.846374
ZNF486	0.943682	0.819397	0.192413	3.05942
MEDAG	0.943696	0.819624	-0.31035	3.340341
FAAH2	0.943696	0.81983	-0.14828	3.607643
KMO	0.943696	0.819848	-0.46934	1.594287
LOC100505	0.943696	0.819879	0.121592	3.805246
TRIM23	0.943696	0.819889	-0.3375	2.01212
TBC1D3P1-	0.943696	0.819914	0.242323	1.48469
MIR647	0.943696	0.819944	0.259318	1.643691
CTDNEP1	0.943703	0.820027	0.222744	1.798378
OR10AD1	0.943757	0.820172	-0.67608	0.377549
LMO3	0.943757	0.820495	-0.68575	1.548264
PSMA3-AS1	0.943757	0.820553	0.238572	1.866034
HIST1H4C	0.943757	0.820583	0.092892	5.169126
LINC01347	0.943757	0.820584	0.175616	3.097794
PACRGL	0.943757	0.820646	-0.63121	0.756071
MIR5690	0.943757	0.820769	0.149181	4.856871
LHX4	0.943757	0.820796	0.217943	1.880413
MRGPRF-A	0.943757	0.820808	0.303214	0.913902
HIST1H2AH	0.943757	0.820874	-0.14689	4.231073
CCNK	0.943757	0.820945	-0.11243	6.074237
NRDC	0.943757	0.82099	0.306607	0.774934
MDN1	0.943791	0.821142	-0.09352	8.470953
EDRF1-AS1	0.943791	0.821189	0.256744	1.380095
FNBP1	0.943791	0.82125	0.088833	7.034284
SNORD8	0.943859	0.82151	0.091279	7.6035
ZFP82	0.943859	0.821531	0.287737	1.515146
ZBTB1	0.943859	0.821538	0.110547	4.663775
OPN1SW	0.944051	0.821782	0.368595	0.494932
SLC25A37	0.944116	0.821978	-0.27329	2.860765
TEKT4P2	0.944116	0.822069	0.247599	1.142229
ASB1	0.944116	0.822125	0.089662	5.07786
TLL1	0.944116	0.822144	0.283482	1.250128
GADD45G	0.944242	0.82233	0.218756	1.596567
PRNCR1	0.944244	0.822409	-0.0942	5.740986
SCARNA10	0.944405	0.822625	-0.09263	7.248049

THRB-AS1	0.944465	0.822754	0.263071	1.781169
LOC101926	0.944735	0.823083	-0.23576	3.421654
SNORD19	0.944735	0.823176	0.237879	1.21572
CDC20	0.944735	0.8233	0.135678	3.468335
LTBP1	0.944735	0.823308	0.206673	2.156897
GPLD1	0.944735	0.823431	-0.24784	3.184261
ABCF3	0.944735	0.823449	-0.13859	5.652259
C3orf62	0.944909	0.823684	0.152042	3.873071
TNFRSF21	0.944909	0.823913	0.099729	6.909149
TMSB4Y	0.944909	0.823989	0.201465	2.568497
KLHL15	0.944909	0.824022	-0.15139	3.955284
PTRH2	0.944909	0.824178	0.198626	2.225083
ZC2HC1A	0.944909	0.824274	-0.14366	4.859361
MAB21L1	0.944909	0.824435	0.28543	1.77035
CARNMT1	0.944909	0.824492	-0.10366	5.466742
ZC3H4	0.944909	0.824526	-0.09612	7.06909
CHL1	0.944909	0.82466	-0.50399	2.05412
ACAP1	0.944909	0.824667	0.107057	4.226791
B9D2	0.944909	0.824681	0.294233	1.095477
GRASP	0.944909	0.824699	0.295878	0.878479
RNVU1-14	0.944909	0.82472	0.22233	3.01852
KIFC1	0.944909	0.824748	0.176399	2.956112
SNORD65	0.94524	0.825114	0.220962	1.883069
PCMTD1	0.945287	0.825303	0.100879	4.859402
NDUFAF4	0.945287	0.825308	0.180004	2.9631
NODAL	0.945399	0.825482	-0.4521	1.016386
FFAR2	0.945647	0.825791	0.269908	1.341152
MIR5194	0.945647	0.825851	-0.48578	1.513803
IGDCC3	0.945872	0.826155	-0.53184	0.961192
CCDC183	0.945872	0.826202	-0.2858	2.671219
ZNF483	0.945956	0.826396	-0.21342	3.002273
NPRL2	0.945956	0.826428	0.129232	3.364221
VPS13B	0.946045	0.826636	-0.46404	1.62761
SH2D3A	0.946045	0.826659	0.084453	5.272965
PIGA	0.946167	0.826842	-0.42463	1.831937
ZNF598	0.946201	0.826949	-0.11014	5.958252
ATAD3A	0.94635	0.827155	-0.57111	0.504814
AFG1L	0.946373	0.827252	0.182215	2.983139
PRKAB2	0.946378	0.827334	0.299699	1.268449
MIRLET7BH	0.946525	0.827632	-0.43116	1.084544
WNT10A	0.946525	0.827668	0.161809	3.177899
CYB5D1	0.946525	0.827692	0.079604	5.760299
STK11IP	0.946525	0.827843	0.098309	4.407958
GAS8-AS1	0.946525	0.827845	-0.31016	2.221621
HCN3	0.946714	0.828129	0.315444	0.884281

ACADSB	0.946714	0.828451	-0.12423	5.117817
RPS3A	0.946714	0.828499	-0.10693	5.677751
KLHDC7A	0.946714	0.82851	-0.31597	2.530119
SNORD90	0.946714	0.828553	0.135555	3.488438
RPS20	0.946714	0.828565	0.18179	2.080481
PPP1R18	0.946714	0.828604	-0.24641	2.686285
LOC10013(	0.946714	0.828667	0.16242	3.428551
MICU3	0.946714	0.828741	0.243682	2.23412
ZKSCAN2	0.946714	0.828777	0.106584	3.728835
TCOF1	0.946834	0.828959	0.249009	1.334542
NDST2	0.946853	0.829131	-0.09512	5.408916
PGLS	0.946853	0.82923	0.090838	4.881861
SMIM18	0.946853	0.829361	-0.55635	1.141691
TANK	0.946853	0.829419	0.187446	2.49312
GPX2	0.946853	0.829508	0.148472	2.669604
ZNF449	0.946853	0.829539	-0.1391	4.034882
ATPAF2	0.946853	0.829623	0.144758	3.953146
TMEM141	0.946853	0.829629	-0.10692	5.052395
CAPN11	0.946853	0.829749	0.341504	0.742363
SRRM5	0.946853	0.829788	-0.27645	2.790405
CUX2	0.946853	0.82982	-0.68883	0.928194
ZMPSTE24	0.946874	0.829915	-0.09351	6.489415
TSSC2	0.946887	0.830002	0.25048	0.964589
THAP11	0.946966	0.830149	-0.14383	4.269339
KRBA2	0.947136	0.830472	0.199903	1.770065
FANCD2	0.947136	0.830536	-0.6407	0.723085
CCR2	0.947136	0.830542	-0.39179	1.584476
ZNF391	0.947136	0.830635	0.216284	0.820263
SLC35B4	0.947136	0.830681	-0.1348	4.617335
TMEM50A	0.947147	0.830767	0.077125	5.75232
URM1	0.947461	0.831208	0.170296	2.159953
PSD4	0.947461	0.831441	0.082926	6.801859
SAP25	0.947461	0.831568	0.177136	2.166353
HIST1H2AG	0.947461	0.831612	-0.13006	4.55869
LOC90246	0.947461	0.831734	0.305136	0.884964
NFKBIB	0.947461	0.831854	0.123315	3.601553
DLG3	0.947461	0.83191	0.159854	2.698295
MIR6773	0.947461	0.83194	0.246078	1.340666
PPFIA1	0.947461	0.831987	0.318662	0.576001
ZDBF2	0.947461	0.832007	0.310917	0.575606
NFIB	0.947461	0.832016	-0.41889	1.497257
RERE-AS1	0.947461	0.832076	0.201222	1.758231
ZNF605	0.947461	0.832175	-0.66948	0.556577
CHKB-DT	0.947461	0.832286	0.244202	1.256037
ZDHHC24	0.947461	0.83252	0.174359	1.851781

TAF13	0.947461	0.83252	0.094583	4.523535
SCARNA18	0.947461	0.832542	-0.22868	2.365664
ZNF662	0.947461	0.832584	-0.59806	1.336923
MALSU1	0.947461	0.832633	-0.1461	3.648065
ADAMTSL1	0.947461	0.832736	-0.36836	2.565089
RABGAP1L	0.947461	0.832762	0.073146	6.673169
GOLPH3L	0.947461	0.832769	-0.0955	6.372738
TNRC6A	0.947461	0.832808	-0.08897	7.705171
ZNF215	0.947674	0.833144	0.137806	2.935319
CENPBD1	0.947674	0.833242	-0.20023	2.608398
RRN3P3	0.947674	0.833371	-0.20623	3.838955
LINC00863	0.947674	0.833484	-0.3299	1.681931
C8orf44	0.947674	0.833489	0.212236	2.417359
FBF1	0.947674	0.833521	-0.12437	4.412077
MOB3A	0.947674	0.833532	-0.08419	5.840024
C5AR2	0.947713	0.833644	-0.66725	1.543215
SNORD83B	0.947852	0.833843	0.183329	2.759969
WWC2-AS1	0.947933	0.834018	-0.52485	1.149557
ZNF300P1	0.947933	0.834094	0.238533	1.612412
HSD17B7P2	0.947933	0.834144	-0.26405	2.564627
IFIH1	0.947939	0.834336	-0.12312	4.675792
RAD9A	0.947939	0.83439	0.22932	1.625059
SNORA50C	0.947939	0.834411	0.090172	6.627098
SUSD5	0.947939	0.834457	0.298644	0.966218
CFAP20	0.948335	0.83494	0.107302	4.119191
PDE7A	0.948335	0.835092	0.258321	0.839424
SDCCAG8	0.948335	0.835107	-0.34088	2.447156
CCDC124	0.948335	0.835112	0.278014	0.827447
CDKN2C	0.948444	0.83537	0.219036	1.584078
KIF16B	0.948444	0.83543	0.189058	1.999235
ST20	0.948444	0.835474	0.311672	0.566692
SMG1P1	0.948444	0.835561	0.076235	6.009977
RUVBL1-AS	0.948444	0.835618	-0.64018	0.673092
SNHG3	0.948444	0.83567	-0.30003	2.682325
TYW1B	0.948476	0.835775	0.104257	4.098306
C10orf95	0.94874	0.836205	-0.397	0.709913
AKR7L	0.94874	0.83624	0.199379	1.933504
B3GLCT	0.94874	0.836258	-0.16453	3.811634
CPPED1	0.94874	0.836315	-0.30587	1.903102
SLC1A5	0.948759	0.836408	0.091578	4.633712
MAPK8IP1	0.949115	0.836799	-0.31686	2.589994
LINC01959	0.949471	0.837189	0.201439	1.281124
CDKN1B	0.949841	0.837593	0.074396	6.439195
HCFC1	0.950301	0.838166	-0.09068	7.020939
ZNF333	0.950301	0.838178	0.183718	2.620578

GGN	0.950301	0.838386	0.251349	0.82831
THOC1	0.950301	0.838418	0.071213	5.59264
PILRB	0.950301	0.838452	0.252908	1.302971
NPIPA1	0.950301	0.83847	0.22253	1.157273
NLRP1	0.950301	0.838537	0.11525	3.418287
ST8SIA4	0.950341	0.838719	0.165826	3.093056
SNORD53	0.950341	0.838726	-0.30608	1.968864
C19orf67	0.95042	0.838907	0.252445	0.659901
NDUFB7	0.95042	0.83895	0.077461	5.460249
LOC33905[illegible]	0.950426	0.839042	-0.25211	2.459771
ACTG1P4	0.950426	0.839109	-0.50576	1.016195
SLC13A4	0.950736	0.83946	0.102513	4.368816
STAU2	0.950928	0.839706	-0.3321	1.648996
PRIM1	0.950944	0.839797	0.136075	3.106295
ADRA1D	0.950971	0.839899	-0.54835	0.510782
LRP1	0.951045	0.840041	0.073392	9.684964
FAM149B1	0.951444	0.840563	-0.10949	4.975033
NDUFS2	0.951444	0.840764	0.226793	1.107543
POMT2	0.951444	0.840772	0.096655	4.083174
VGLL1	0.951444	0.840811	-0.71798	0.454405
MIB1	0.951444	0.840823	-0.08489	7.436973
TMEM131	0.951444	0.840856	0.070108	7.523037
PANK3	0.951543	0.841021	0.07032	7.347602
OCIAD1	0.951585	0.841173	0.232995	1.530162
ABHD8	0.951585	0.841255	0.175825	2.275275
GOLGA8O	0.951585	0.84133	0.208099	1.917944
ZFP2	0.951585	0.841365	0.261152	1.229878
UBAP2	0.951598	0.841454	0.106287	3.954802
SFRP2	0.951698	0.841689	0.14346	5.007776
LARS2-AS1	0.951698	0.841697	-0.44441	0.830148
ZBED8	0.95174	0.841932	-0.9036	0.626916
SND1	0.95174	0.841942	-0.08544	7.6768
TMEM18	0.95174	0.841965	-0.13755	4.462807
TUBA1B	0.951857	0.842146	0.070073	7.524225
ACTR5	0.951866	0.842231	-0.20166	3.167394
UST-AS1	0.952024	0.842448	-0.39308	1.102654
MAF1	0.952089	0.842673	-0.09537	5.383572
MAGEF1	0.952089	0.842755	0.083179	5.092965
SIK3	0.952089	0.842797	-0.2278	2.234241
TMEM72	0.952089	0.84292	-0.6568	0.806915
KLHDC2	0.952089	0.842936	-0.09331	5.464443
LOC10050[illegible]	0.952089	0.843166	-0.36519	1.352397
FBXO2	0.952089	0.84318	-0.26642	2.19594
DYRK1A	0.952089	0.843239	-0.15767	4.345121
CNGA1	0.952089	0.843241	0.23143	1.176939

THOP1	0.952089	0.843276	-0.15133	4.65418
FAM131A	0.952124	0.843385	0.192162	1.813471
GEN1	0.953066	0.844296	0.13605	2.836917
LOC101928	0.953176	0.844543	-0.36075	1.570027
LMNA	0.953176	0.844548	-0.31113	2.786647
KIF5B	0.953417	0.844839	-0.08623	8.734327
ZNF24	0.95349	0.844981	0.076255	6.64944
DKC1	0.95353	0.845094	0.234741	1.555505
TSPYL6	0.953628	0.845392	-0.3333	2.324023
ZNF845	0.953628	0.845428	-0.12105	4.237014
RNF139-AS	0.953628	0.845539	0.179748	2.355603
LOXL4	0.953628	0.84558	-0.21833	2.975821
ZNF132	0.953628	0.845632	-0.22983	2.68697
UBN1	0.953628	0.845644	-0.32506	2.244687
CCL24	0.953857	0.845988	0.227495	1.146936
HSPA12A	0.953857	0.846094	0.152535	3.082346
RPUSD1	0.953857	0.846103	-0.16889	3.175276
SELENOS	0.953857	0.846156	0.11281	3.811454
UQCR11	0.953899	0.84627	-0.1027	5.736037
MSL3	0.953943	0.846436	0.138136	3.421723
ALK	0.953943	0.846464	-0.33584	1.339505
PTGES2	0.954036	0.846624	-0.34886	1.883417
MAGEH1	0.954225	0.846911	-0.30675	1.615214
RBM14	0.954225	0.847046	0.103291	3.879406
IRS2	0.954225	0.847088	0.105855	5.337093
EXOC3L2	0.954225	0.847213	-0.62797	0.649408
SNCA-AS1	0.954225	0.847249	-1.00157	0.828713
6-Sep	0.954225	0.847256	0.203048	1.621732
MIR320B2	0.954258	0.847362	-0.46114	1.085197
LINC01578	0.954535	0.847699	-0.13164	3.517323
CCT8	0.954535	0.847762	0.140898	2.531813
SPATA21	0.954779	0.848117	-0.37367	1.065843
PRSS23	0.954779	0.848163	-0.37684	2.062804
RPL23P8	0.954779	0.848211	-0.2789	1.597409
FLRT2	0.954854	0.848355	0.141129	4.087646
NBPF1	0.954917	0.8485	0.092702	4.105369
PATL1	0.954917	0.848566	0.0705	6.601799
LINC00672	0.955263	0.849018	0.164312	2.266612
NAT10	0.955263	0.849028	-0.25864	2.111904
CPXM2	0.955563	0.849447	0.149972	3.607994
TMEM203	0.955563	0.8495	-0.18664	3.591849
LOC101927	0.955563	0.84958	-0.59562	0.679195
C1orf195	0.955563	0.849604	0.156778	2.662436
STK40	0.955747	0.849946	-0.30667	2.068943
ZBTB46-AS	0.955747	0.850071	-0.4044	1.483394

GGPS1	0.955747	0.850231	0.11185	2.939492
YIPF4	0.955747	0.850232	-0.07992	5.937199
AURKAP1	0.955747	0.850243	0.163344	2.279002
ACP7	0.955747	0.850261	0.154014	2.644733
RECQL4	0.955747	0.850317	0.099649	4.043128
SLC22A13	0.955747	0.850437	-0.31902	1.817595
UTP23	0.955747	0.850464	-0.13232	4.322726
LOC100288	0.955765	0.850585	-0.20693	2.664512
FADS2	0.955765	0.850636	0.243248	0.577806
NLRP9	0.955829	0.85081	0.219005	1.515358
GPRC5C	0.955829	0.850847	0.21067	1.452847
HIGD2A	0.955881	0.850971	0.107638	3.292824
TMEM92-A	0.955942	0.851103	0.241576	0.554648
PLEC	0.955986	0.85122	-0.1183	5.095493
TRIM62	0.95601	0.851318	0.090911	3.659129
ZNF136	0.956053	0.851434	-0.20757	3.350978
SFTPA2	0.956317	0.851746	0.29009	0.622275
BCR	0.956453	0.852019	0.165784	1.416358
PNN	0.956453	0.852022	0.065526	9.363592
CT62	0.956726	0.852343	-0.32617	1.475689
SLC30A5	0.956732	0.852446	-0.08269	5.449904
CEMP1	0.956732	0.852578	0.118505	2.96179
ROCK1P1	0.956732	0.852738	-0.14273	3.225046
TIMMDC1	0.956732	0.852747	0.068734	5.872622
FUBP3	0.956732	0.852825	-0.08067	6.698236
BRE-AS1	0.956732	0.85299	-0.47966	0.671435
KDELR2	0.956732	0.853008	0.138055	2.162838
NRXN3	0.956732	0.853019	-0.53001	1.13203
CHCHD5	0.956732	0.853046	0.206309	0.702473
RAB36	0.957151	0.853532	-0.19836	2.940428
UTP6	0.957151	0.853577	0.063661	6.031151
KLF15	0.957151	0.853782	-0.33626	3.882318
CIZ1	0.957151	0.853842	0.224817	0.559738
SLC25A16	0.957151	0.853849	-0.16407	3.853345
RBM15B	0.957151	0.853905	-0.07883	6.355836
ATF6	0.957151	0.854092	0.062687	6.643056
GPR135	0.957151	0.854117	-0.304	1.833202
ZNF396	0.957151	0.854169	-0.32891	1.722475
UBE2W	0.957151	0.854195	0.142117	3.312165
AAK1	0.957469	0.854648	-0.0782	7.786414
PEX12	0.957469	0.854686	-0.2626	2.395367
DLST	0.957469	0.854712	-0.26194	3.329532
POLN	0.957796	0.855081	-0.18018	2.923048
BLOC1S2	0.957799	0.855227	0.198878	1.542749
MRPL20-AS	0.957799	0.855275	-0.16616	3.592456

TAF1D	0.957799	0.855316	-0.08923	6.556235
MGAT1	0.957975	0.855551	0.098058	3.673193
COMT	0.957986	0.855655	0.147976	3.010026
PTPRA	0.957986	0.855776	-0.53651	0.932664
RPP30	0.957986	0.855824	0.142886	2.937901
SMG8	0.957986	0.855871	-0.11239	4.244064
WHAMMP:	0.958016	0.855982	0.074869	5.131293
SBF2-AS1	0.958016	0.856054	0.15113	3.03086
LOC10192	0.958073	0.856181	-0.31726	2.037035
NEDD4	0.95817	0.856346	0.164399	2.10918
MTMR3	0.958177	0.856475	0.166478	1.910716
UBR4	0.958177	0.856529	-0.07885	9.282573
NAGA	0.958177	0.856586	-0.11041	4.274613
HSPB7	0.958251	0.856729	-0.42081	2.200935
GINS1	0.958634	0.857149	-0.15301	3.971241
PDZRN3-A	0.958692	0.857278	0.215852	1.478048
SLC16A1	0.958752	0.857432	0.170408	1.397676
LIX1L	0.958752	0.857487	0.097659	4.654529
SNORD27	0.958972	0.857761	-0.09805	7.217669
STARD3NL	0.95913	0.858033	0.114806	3.283974
LIG1	0.95913	0.858058	0.149362	2.767076
TCTEX1D2	0.959173	0.858175	0.12854	2.010556
DPH7	0.95937	0.858447	0.093704	3.477541
CC2D1B	0.95937	0.858506	0.06397	5.768252
MIR4755	0.959455	0.85866	0.163284	2.003934
KNOP1	0.959835	0.859078	-0.09035	4.841549
CCDC150	0.959898	0.859356	0.154805	1.866931
LOC14578	0.959898	0.859385	0.149784	2.348014
SH3BGRL	0.959898	0.859519	-0.08369	5.980575
NMT1	0.959898	0.859553	-0.07341	6.507794
KMT2E	0.959898	0.859624	-0.43142	0.91409
SNORD26	0.959898	0.859715	-0.10294	7.951176
C18orf54	0.959898	0.859723	0.239365	0.538994
DHX30	0.959898	0.859756	0.162154	1.277258
INTS6-AS1	0.960017	0.85994	-0.26723	2.124204
PNPLA3	0.960078	0.860073	0.186262	1.628286
CEMIP	0.960416	0.860454	-0.7759	0.632587
CACTIN	0.960585	0.860683	0.236234	0.88388
TOP1P1	0.960707	0.860945	0.098838	3.525633
ATG14	0.960707	0.860948	0.058889	6.05459
KIF15	0.960735	0.861051	-0.09692	4.470047
MYL6	0.960833	0.861216	0.131249	2.386379
PRR22	0.960886	0.861342	0.174681	1.726465
UGT1A1	0.960949	0.861476	-0.19484	2.463141
UBA6-AS1	0.960963	0.861566	0.122019	2.922337

DCLRE1C	0.961231	0.861885	-0.46073	0.736301
GABRP	0.961334	0.862055	-0.23265	3.463087
GLE1	0.961545	0.862357	-0.11466	4.260387
SH3BP2	0.961545	0.862399	0.155507	1.878798
ABCB1	0.961795	0.862847	0.147228	3.069227
CTC-338M1	0.961795	0.862852	-0.31916	1.683055
BRI3	0.961795	0.862891	-0.1433	3.751752
SMG1P3	0.961795	0.862936	0.062251	5.859545
CLUHP3	0.961852	0.863065	0.086421	4.53403
LOC102723	0.962165	0.863456	0.189737	1.416038
KLHL26	0.962165	0.86374	-0.22343	2.480424
FAM133DP	0.962165	0.863799	-0.20956	3.373779
MLANA	0.962165	0.863806	0.123396	2.895868
MYCBPAP	0.962165	0.863842	-0.41387	0.658773
BRAP	0.962165	0.864013	-0.07833	5.148478
GCLC	0.962165	0.864077	-0.45953	0.487032
STXBP3	0.962165	0.864119	0.057509	5.799657
LAMP3	0.962165	0.864173	-0.25171	3.230788
TADA1	0.962165	0.864176	-0.17154	3.861272
TRAF3IP2	0.962165	0.864274	-0.28182	1.903696
LONP2	0.962165	0.864281	-0.1361	3.441975
NIPBL-DT	0.962178	0.864569	0.08967	4.322345
UQCRFS1	0.962178	0.864666	0.061638	5.861622
PSKH1	0.962178	0.864682	-0.09029	4.885233
IAH1	0.962178	0.864864	0.10505	3.605378
RFX3-AS1	0.962178	0.864917	0.177631	1.488843
LSM7	0.962178	0.864944	0.094447	3.554634
ATP6V1C1	0.962178	0.864962	0.063708	5.598833
TOM1L1	0.962178	0.86498	-0.08617	5.570999
RAB3A	0.962178	0.865033	0.198258	1.455179
LOC100506	0.962178	0.865072	0.24495	0.496728
METRN	0.962195	0.865165	-0.37151	1.49994
HOXA7	0.962207	0.865284	-0.59482	0.657366
MBLAC1	0.962207	0.865332	0.133271	2.053884
DRAM2	0.96223	0.865471	-0.10919	4.549572
ERVFRD-1	0.96223	0.865577	0.137364	3.329596
LOC101927	0.96223	0.86564	0.167592	1.726514
SCARNA23	0.96223	0.865685	-0.10971	4.537405
LRRC59	0.96223	0.865742	-0.08021	7.108109
DNAJC24	0.962327	0.865961	-0.14338	3.672062
ACADL	0.962327	0.86605	0.192593	1.451115
LINC01725	0.962327	0.866256	-0.57194	0.731642
RRAS2	0.962327	0.866278	0.191004	0.569347
CAPS2	0.962327	0.866286	-0.49381	0.727376
CCSER2	0.962327	0.866297	-0.41125	1.403243

SMG1P2	0.962535	0.866759	0.113307	3.357745
TMED3	0.962535	0.866766	-0.16869	2.74832
NEDD8	0.962535	0.866829	-0.12232	3.661238
YPEL4	0.962535	0.866865	0.140207	2.182258
C12orf73	0.962535	0.866874	0.117621	2.131656
CCZ1	0.962906	0.86731	0.075208	5.204103
MORF4L2-/	0.962906	0.867364	-0.44272	1.035921
GCH1	0.963003	0.867547	-0.50187	1.374472
RAB27B	0.963003	0.867634	-0.08111	7.650082
ZNF571-AS	0.963003	0.86779	-0.51881	0.636425
CLEC16A	0.963003	0.867842	0.068198	4.981407
UBTD1	0.963003	0.867868	0.126051	2.877461
CLIP4	0.963003	0.867978	0.243418	0.633914
SDF2L1	0.963003	0.867997	0.105591	2.954931
SOWAHA	0.963083	0.868227	0.151106	1.129249
VPS26C	0.963083	0.868253	-0.08219	5.452216
MYLIP	0.963083	0.868373	0.093614	4.325796
SOX9	0.963083	0.868382	0.062944	6.708942
SLC31A1	0.963442	0.868783	-0.07579	6.240023
NUP98	0.963478	0.868894	0.190925	0.855497
BRICD5	0.96358	0.869094	0.210058	0.834146
ZNF467	0.96358	0.869197	-0.17673	3.072789
C19orf57	0.96358	0.86922	-0.26956	2.352353
USP4	0.963869	0.869623	-0.36363	1.610535
TAF7	0.963869	0.86991	-0.07203	6.648365
PUS1	0.963869	0.869928	-0.18924	2.361092
ZNF597	0.963869	0.869943	-0.24712	3.106068
CARD8-AS1	0.963869	0.869962	0.162798	1.817343
SNAPC4	0.963869	0.870056	0.063245	5.039255
MRPL17	0.963869	0.870069	0.07722	4.473801
PRPF8	0.963869	0.870106	-0.08074	6.415605
NECAB1	0.963875	0.870204	-0.51393	1.220792
C5AR1	0.963875	0.870328	0.123206	2.91492
C8orf37	0.963875	0.870345	0.168687	1.722546
LINC02256	0.963995	0.870531	0.142471	2.688184
CWF19L2	0.96406	0.870704	-0.07229	5.654963
SF3A3	0.96406	0.870747	0.054027	6.458253
HAVCR1P1	0.964368	0.87111	0.19139	0.917116
SNORA71A	0.964368	0.871201	-0.1174	5.252846
USP17L7	0.964368	0.871278	-0.69028	0.436839
PRDX5	0.964368	0.871337	-0.23675	1.768409
LOC100506	0.964718	0.871731	0.05954	5.635121
PIKFYVE	0.964727	0.871889	0.057945	6.618902
LSM10	0.964727	0.871896	0.110205	3.075008
SALL4	0.964829	0.872066	-0.72653	0.983335

TRIM39	0.964848	0.872241	0.151241	1.860307
ANGEL2	0.964848	0.872429	-0.1987	2.412325
CLCN3	0.964848	0.87243	0.138241	2.334226
ZFYVE26	0.964848	0.872498	0.054468	6.406012
TULP4	0.964848	0.872535	0.062789	4.96704
AP4S1	0.964848	0.872553	-0.16829	2.781941
TAS2R60	0.964875	0.872655	0.20362	1.236011
DANCR	0.964916	0.872858	0.066796	4.902416
MECP2	0.964916	0.872927	-0.07627	5.943968
MEIOC	0.964916	0.872965	-0.43423	0.965087
PGAP1	0.964916	0.873005	-0.07538	6.282599
MRS2	0.965042	0.873197	0.242402	0.594707
GPR158	0.96508	0.873309	-0.56628	0.614347
SNORA72	0.965473	0.873743	-0.11	4.927242
CCND3	0.965518	0.873862	0.159411	2.046151
MZT1	0.96555	0.874016	-0.10697	3.842051
MRPL32	0.96555	0.874047	0.075617	4.65744
TNFRSF12A	0.965646	0.874213	-0.1751	4.1252
OR2A7	0.96572	0.874414	0.21648	0.601748
SELENOK	0.96572	0.874436	-0.09453	4.59619
GNAT2	0.965952	0.874724	0.091716	3.34848
RARS2	0.966332	0.875147	-0.06577	5.768471
PHKG2	0.966335	0.875228	0.127382	1.312195
CYTH3	0.966338	0.875309	0.059003	5.608613
CDC14A	0.966432	0.875497	-0.24565	1.880959
NFXL1	0.966432	0.87563	-0.6472	0.481175
DDX50	0.966432	0.875677	0.052626	5.574433
CTSB	0.966432	0.875742	0.165086	1.355023
E2F7	0.966432	0.875785	-0.13354	3.198443
DDX51	0.966681	0.876144	0.080193	4.791343
GPATCH2	0.966681	0.87628	0.069371	4.330982
MED21	0.966681	0.876362	0.141915	0.888742
RNF2	0.966681	0.876391	-0.09626	4.810565
EXO5	0.966681	0.876402	-0.14655	2.761097
NEXN	0.966844	0.876628	0.218419	0.556091
FANCG	0.966871	0.876731	-0.11884	3.770831
ALKBH1	0.966976	0.877133	-0.19994	3.04829
ZRANB2	0.966976	0.877189	-0.26944	1.776835
RNF216-IT1	0.966976	0.877218	0.150835	1.40632
VARS	0.966976	0.877227	-0.27741	2.076283
DHX15	0.966976	0.877329	-0.06597	7.695084
SBF1	0.966976	0.877398	-0.06626	7.515786
ZNF672	0.966976	0.877429	-0.08375	4.972547
ELMOD2	0.966976	0.877478	-0.0924	5.417686
SUMO2	0.966976	0.877531	0.148212	1.62895

TWF2	0.966991	0.877623	-0.09658	5.165482
C22orf39	0.96706	0.877764	-0.12618	3.274022
DSTN	0.967324	0.878082	0.179234	1.410108
SUPT16H	0.967382	0.878213	-0.06871	7.516728
XXYLT1-AS1	0.96757	0.878462	0.120725	1.217491
SNORA54	0.967622	0.878598	-0.089	5.906322
SNORD102	0.967622	0.878857	0.072264	4.334163
SCFD2	0.967622	0.879018	0.059787	5.038043
OPHN1	0.967622	0.879054	-0.07795	5.390366
RCOR3	0.967622	0.879103	0.224835	0.700262
TARBP2	0.967622	0.879222	0.20418	1.016192
POU5F1P3	0.967622	0.879232	0.204178	0.707403
WAC	0.967622	0.879265	-0.21399	2.020168
WASH3P	0.967622	0.879327	-0.11059	3.934794
CENPF	0.967622	0.879401	0.057646	7.118846
TLR9	0.967622	0.87944	0.187893	0.683736
EIF4G2	0.967622	0.87945	-0.13942	2.923682
WASH7P	0.96786	0.879764	0.061644	4.446685
ANKS1B	0.96786	0.879823	0.12895	2.164187
AKAP11	0.967958	0.880054	-0.07161	7.568461
SNORD11B	0.967958	0.880164	-0.29888	1.79609
PRPF6	0.967958	0.880187	-0.07136	7.147323
ZNF830	0.967958	0.880226	-0.10966	4.523137
LOC729218	0.968204	0.880584	0.083024	3.798812
ATP9A	0.968204	0.880606	0.053767	7.50531
ANKRD52	0.968502	0.880955	0.054248	6.694939
USP11	0.968538	0.881095	-0.07055	5.357637
TM7SF3	0.968538	0.881196	-0.07015	6.570694
IFITM2	0.968538	0.881321	0.107429	4.226878
MLF2	0.968538	0.881615	0.101272	2.480657
RND1	0.968538	0.881621	0.124384	2.100648
LINC01012	0.968538	0.881659	-0.31517	0.538435
B9D1	0.968538	0.881676	-0.37547	1.006089
CLSTN2	0.968538	0.881761	-0.22117	2.569264
COMMD3	0.968538	0.881773	0.136574	1.819367
SNORA80A	0.968538	0.881773	-0.13203	3.413314
RBCK1	0.968548	0.881861	-0.36612	1.6627
RNPEP	0.96869	0.882168	-0.07627	6.710308
RPL15	0.96869	0.882203	0.13636	1.912618
PCNX1	0.96869	0.882265	-0.17156	2.484588
WASHC1	0.96869	0.882304	0.114892	2.353483
EPB41L1	0.968806	0.882691	-0.13673	3.444214
HSPA6	0.968806	0.882809	-0.41304	1.809021
SRCAP	0.968806	0.882886	0.049517	8.523002
TNPO2	0.968806	0.88303	0.129057	1.943751

CCM2L	0.968806	0.883123	-0.29748	1.652911
MEX3B	0.968806	0.883388	0.119692	1.374858
ECHS1	0.968806	0.883396	-0.07141	6.317963
UTY	0.968806	0.88344	-0.18641	3.21225
ASXL2	0.968806	0.883462	-0.06263	7.392655
FDXR	0.968806	0.883564	0.083282	3.318671
ALPK2	0.968806	0.883586	-0.22275	2.534523
SCARNA20	0.968806	0.883693	-0.18209	3.132158
GPAT2	0.968806	0.883736	0.131478	2.365684
GTF2H4	0.968806	0.883743	-0.28538	0.569374
DEAF1	0.968806	0.883763	-0.30975	0.807111
KCNMB4	0.968806	0.883775	0.15138	1.46997
ZRSR2	0.968806	0.883852	-0.07777	5.64625
CES1P2	0.968806	0.883901	0.179658	0.775639
CHCHD1	0.968806	0.883991	-0.10717	4.043948
NYAP1	0.968806	0.884018	-0.4201	1.119612
ZNF787	0.968806	0.884057	0.071034	4.185883
USH2A	0.96888	0.884283	-0.20253	2.798924
FIZ1	0.96888	0.88436	0.106561	2.614635
PARS2	0.96888	0.884428	-0.26068	2.457157
IRGQ	0.96888	0.884438	-0.06974	6.297914
METTL2A	0.969196	0.884806	0.06869	4.061924
LOC646471	0.969278	0.885122	0.062604	4.389618
BBS5	0.969278	0.885137	-0.21915	3.00569
TMCC3	0.969278	0.88519	-0.38195	1.003836
AHCTF1	0.969278	0.885195	-0.06649	6.972299
ACP4	0.969322	0.88532	-0.35539	1.488134
PIAS4	0.969322	0.885442	0.061027	4.959568
FAM193B	0.969322	0.885546	0.060314	4.984017
PCDH17	0.969322	0.885673	0.089216	3.329209
TIMM8A	0.969322	0.885685	0.132252	2.517092
ALKBH3-AS	0.969322	0.885736	0.138116	1.523129
BDP1	0.969322	0.885784	-0.08936	5.149003
IFITM10	0.969791	0.886291	0.076696	3.590272
NICN1	0.969858	0.886431	0.061831	4.297248
TAF4	0.969933	0.88667	0.071131	4.22589
USP27X	0.969933	0.88668	-0.20103	1.993083
ITGA3	0.969933	0.886795	0.054304	6.96822
FOXN3-AS1	0.969933	0.886814	-0.24703	2.084294
UCK2	0.969944	0.886905	0.051353	5.607755
MPC2	0.969944	0.886981	0.110998	1.716008
ZNF765	0.969998	0.887208	-0.08798	4.584249
POLR2J3	0.969998	0.887233	0.076839	3.586086
TG	0.969998	0.887419	0.125715	2.148986
NADK	0.969998	0.887422	-0.20962	2.435079

PREPL	0.969998	0.887423	0.107452	2.360623
AKAP12	0.970039	0.887547	0.131438	1.741138
HOXB2	0.970039	0.887617	-0.10723	4.013749
SUPT7L	0.970155	0.887803	-0.44005	1.370858
RTN4IP1	0.970334	0.888075	0.070047	3.809496
SARAF	0.970334	0.888124	0.133901	1.407369
WAC-AS1	0.970497	0.888508	0.046329	5.643142
STC1	0.970497	0.888562	-0.23629	4.157241
HYDIN	0.970497	0.888607	-0.39485	1.476476
CCT2	0.970497	0.888615	-0.43342	1.043751
L3MBTL2	0.970497	0.888666	0.053815	5.04891
HIST1H2AE	0.970552	0.88885	-0.0785	5.457938
CLUAP1	0.970552	0.888873	0.116365	1.486422
SYCE1L	0.970614	0.889014	0.153672	1.06648
GOLGA2P1	0.970614	0.88909	-0.5047	0.571667
SLC3A2	0.970614	0.889228	-0.22911	1.787208
GNRH1	0.970614	0.889244	0.108794	2.429335
ROM1	0.970866	0.889555	-0.24904	2.044438
PIBF1	0.971069	0.889888	0.049203	5.487445
PAXIP1-AS:	0.971069	0.889942	0.141837	1.12975
MEA1	0.971069	0.889976	-0.0676	5.589112
TOP3A	0.971152	0.890131	0.064153	4.491219
C14orf119	0.971163	0.890364	-0.10639	3.955967
IGSF6	0.971163	0.890632	-0.09037	4.224721
NPAS4	0.971163	0.890636	-0.49593	0.91265
SNORA1	0.971163	0.890673	-0.09908	4.935937
SLC16A12	0.971163	0.890677	0.168436	0.565902
CEP68	0.971163	0.890701	0.054521	5.709822
TMED10P1	0.971163	0.890819	0.099241	3.042419
SRSF4	0.971163	0.890833	-0.06157	7.705068
NUP93	0.971163	0.89086	-0.14381	2.836631
ZNF740	0.971163	0.890928	0.047951	5.799323
ERH	0.971345	0.891239	0.056443	5.300491
MMAA	0.971345	0.891252	-0.12948	3.7083
VPS33A	0.971358	0.891342	-0.08948	4.540124
FTSJ3	0.971491	0.89168	-0.06162	6.125551
PIK3CD	0.971491	0.891712	0.058031	4.422549
DLL1	0.971491	0.891815	0.085207	3.452318
SIRPA	0.971491	0.891816	0.121424	0.940399
JPX	0.971491	0.891858	-0.12961	3.27136
ANAPC2	0.971584	0.89204	-0.09103	4.836365
ENSA	0.971584	0.892101	0.095877	3.095467
GTF2H5	0.971608	0.892251	0.06457	4.15512
RCOR2	0.971608	0.89228	-0.30255	1.56766
BHLHE22	0.971759	0.892532	0.138697	1.422473

RPLP1	0.971759	0.8926	0.138851	0.730276
MIR181A2I	0.971759	0.892713	0.099474	2.775647
WASH5P	0.971759	0.892733	-0.17306	2.058701
RHOQ	0.97179	0.892847	0.051557	5.461961
ZNF106	0.97179	0.892919	-0.06564	6.402682
ANKRD39	0.971823	0.893056	0.087769	2.637562
WDR64	0.971823	0.893158	-0.67111	1.207202
DPH3P1	0.971823	0.893186	-0.37542	1.468688
SLAMF8	0.971962	0.893392	0.122515	2.3372
SYCP2	0.971991	0.893616	-0.15238	3.231973
NDFIP1	0.971991	0.89364	-0.05794	6.731598
CPAMD8	0.971991	0.893656	-0.30116	1.826057
SULT2A1	0.972034	0.893774	-0.68761	1.804319
SEC63	0.972073	0.893908	0.048385	7.489779
PXN-AS1	0.972073	0.894143	0.158125	0.636793
CYP8B1	0.972073	0.894146	0.122208	1.477646
PLSCR1	0.972073	0.894202	-0.06461	5.752012
MAN1A2	0.972073	0.894204	0.044192	7.948609
R3HDM2	0.972128	0.894361	-0.05853	7.034389
COL22A1	0.972128	0.894509	0.10127	2.419155
POLR2D	0.972128	0.89451	0.056769	4.6827
MMP17	0.972128	0.894569	0.105955	1.176128
ESCO2	0.972304	0.89481	0.090571	2.797507
CD300E	0.972338	0.894968	0.100768	2.998199
BCAT2	0.972338	0.894998	-0.37545	0.742739
MED15	0.972355	0.89524	-0.12517	3.388024
LOC100507	0.972355	0.895386	0.129483	1.657167
GSAP	0.972355	0.895461	-0.08303	4.938019
METTL25	0.972355	0.89547	-0.11907	3.4048
LIN37	0.972355	0.895473	0.096267	2.891825
NOL9	0.972355	0.895486	-0.05902	6.005114
MMP24	0.972431	0.895636	-0.10856	4.088593
C19orf24	0.972754	0.896011	0.069557	3.317491
XKR6	0.972833	0.896198	-0.17707	2.310197
PPP1R16A	0.972833	0.896242	0.059386	4.102171
FAM169A	0.973058	0.896539	0.15912	0.576685
KIAA0100	0.973058	0.896699	0.04226	7.880998
LRRC8B	0.973058	0.896831	0.111981	2.226489
DPAGT1	0.973058	0.896948	0.058659	4.50346
SNORD82	0.973058	0.897015	0.076106	2.793616
LINC00852	0.973058	0.897016	-0.2119	2.603982
CD52	0.973058	0.897141	0.099872	2.027146
TPR	0.973058	0.89718	-0.05828	8.725749
STS	0.973058	0.897192	0.09409	3.986716
NCAPD2	0.973058	0.897238	0.045772	6.398147

RPS24	0.973089	0.89751	-0.40004	0.991587
GALNT15	0.973089	0.897525	-0.23237	2.440728
PVR	0.973089	0.897588	0.104023	2.026347
ADNP	0.973089	0.897718	-0.22959	2.431293
TNRC6C-AS	0.973089	0.897735	0.097459	3.039062
C9orf78	0.973089	0.897738	0.04621	6.291358
DAGLA	0.973153	0.897877	0.059191	4.076298
HPS6	0.973155	0.897976	0.057123	3.991467
AP2S1	0.973155	0.898092	0.086775	2.460325
STAG3L2	0.973155	0.898157	-0.07549	4.686314
MED28	0.973155	0.898194	0.053641	5.878902
LINC01303	0.973219	0.898393	0.116257	2.294659
CDYL2	0.973219	0.89841	0.076822	3.128821
FAM13A	0.973291	0.898634	0.08858	2.639137
CHCHD6	0.973291	0.898711	-0.16456	2.938166
TSPYL1	0.973291	0.898713	-0.05904	7.756025
CALML6	0.973501	0.898986	0.132872	0.905595
ZSWIM1	0.973562	0.899121	0.052965	4.056996
LOC101927	0.973613	0.899248	-0.1008	3.754804
BROX	0.973877	0.89958	-0.22921	2.001501
FOXK1	0.973877	0.899691	0.041924	7.277872
LINC01535	0.973877	0.899777	0.145713	0.715049
RPL13AP6	0.973877	0.899807	0.096846	1.560309
NPAS1	0.974062	0.900056	0.15065	0.708934
CASC2	0.974239	0.900299	-0.38862	1.026208
EFNB2	0.974368	0.900498	0.047908	6.950024
GXYLT2	0.974368	0.900576	0.102801	1.866918
CATSPER2F	0.974469	0.900748	-0.23032	2.005287
KDELC2	0.974548	0.901218	0.043974	5.443244
GPR15	0.974548	0.90125	-0.45973	0.724604
ITGAE	0.974548	0.901381	-0.09464	3.967166
CIC	0.974548	0.901396	-0.06409	5.479567
NOD2	0.974548	0.901404	-0.37997	0.98022
ELK4	0.974548	0.901427	-0.05833	7.134571
MRGBP	0.974548	0.90145	-0.11482	3.805606
TMEM184C	0.974548	0.901453	-0.0625	5.306059
PCAT1	0.974838	0.9018	-0.21846	1.735419
RTL1	0.974973	0.902004	-0.32686	2.367727
PLEKHO2	0.974993	0.902102	0.135273	0.548284
JAZF1	0.975027	0.902212	-0.11098	3.729021
LOC105377	0.975323	0.902565	0.161248	0.666859
CAPN1	0.975664	0.90296	-0.27451	1.618522
ZNF660	0.975747	0.903202	0.121735	1.828148
GTF2A2	0.975747	0.903361	0.069248	3.970645
MICALCL	0.975747	0.903466	0.070299	3.499805

THEM4	0.975747	0.903466	0.064643	3.607028
GALNS	0.975747	0.903492	-0.08486	4.403021
ANKRD9	0.975747	0.90351	0.046648	4.59622
EWSR1	0.975866	0.9037	-0.14301	3.867837
MAP6D1	0.975883	0.903803	-0.36784	0.52921
C19orf25	0.975883	0.903873	0.065218	3.725156
GOLGA6L4	0.975988	0.90405	0.081743	2.252648
FAM192A	0.976099	0.904231	-0.05227	6.70693
PRKD2	0.976127	0.904337	0.077549	1.940295
WDR70	0.976335	0.904676	-0.05754	5.63588
DAGLB	0.976335	0.904688	-0.22724	1.579906
EXOC8	0.976335	0.904793	0.046176	5.205817
MYOZ1	0.976335	0.904846	0.113859	1.912148
MMACHC	0.976498	0.905076	-0.14012	2.548086
DDX18	0.976684	0.905391	-0.05584	6.430635
TUBG2	0.976684	0.90544	-0.09353	3.965028
LINC01484	0.976684	0.905486	-0.47053	1.894242
WNT5B	0.976741	0.905633	-0.30463	1.447374
LINC00649	0.976741	0.905742	-0.12501	3.571364
SNORA74A	0.976741	0.905776	0.042911	6.38945
MTRNR2L1	0.976768	0.90595	0.091647	1.697705
SRGAP2D	0.976768	0.905959	0.042086	5.813237
BCRP3	0.976776	0.90606	0.150652	0.556741
ZNF709	0.976776	0.906125	-0.11236	3.062045
CCDC28B	0.976891	0.906364	0.120749	0.687873
TAF5	0.976891	0.906402	-0.12399	3.061342
DCST2	0.976891	0.906557	-0.31557	1.66147
SF3B5	0.976891	0.906559	0.051796	4.700444
EIF3A	0.976891	0.906627	-0.0535	10.14802
MLLT3	0.976916	0.906742	0.167109	0.525604
MAP3K14	0.976916	0.906809	-0.08863	5.170507
TMEM268	0.976954	0.907061	0.05149	4.511349
SCTR	0.976954	0.90719	0.103023	1.476369
PRPF3	0.976954	0.907215	-0.05913	6.440933
DLEU2L	0.976954	0.907349	0.078427	1.934222
GPR107	0.976954	0.907396	-0.2944	1.785848
GTF3C6	0.976954	0.907407	-0.06027	5.726407
SLC25A25	0.976954	0.907478	0.073756	2.065047
NUTM2G	0.976954	0.907506	-0.09205	4.055294
MSMP	0.976954	0.907556	-0.12093	3.510834
ZNF248	0.977306	0.907995	-0.2838	1.811893
TRAPPC13	0.977306	0.908042	-0.34766	0.999309
DDIAS	0.977424	0.908343	0.07045	2.70662
PPM1G	0.977424	0.908364	-0.0543	7.246283
ZNF841	0.977424	0.908403	-0.05726	6.237988

GALNT18	0.977424	0.908467	0.051332	4.105601
GLOD4	0.97745	0.908651	0.043647	5.922797
CCDC14	0.97745	0.908697	-0.29029	1.079565
GREB1L	0.97745	0.908729	0.142414	0.974034
ALDH3A1	0.977518	0.908872	-0.27642	1.642234
SSPO	0.977588	0.909016	0.099888	2.224134
ANKRD11	0.977978	0.909458	0.067189	3.591461
NFASC	0.978072	0.909625	0.050521	1.077805
HIST1H3A	0.978108	0.909826	-0.15217	2.671506
SEC22A	0.978108	0.909919	-0.07252	4.636119
ALCAM	0.978108	0.910223	0.095283	1.629898
ARRDC3-AS	0.978108	0.910223	0.06739	2.440568
KAT2A	0.978108	0.910249	0.042556	5.311692
IP6K1	0.978108	0.910279	0.10726	1.586096
RYBP	0.978108	0.910294	0.042417	6.032043
PPFIBP1	0.978108	0.910315	0.104632	0.649937
TTN	0.978108	0.910371	0.08438	2.505938
AHCTF1P1	0.978356	0.910681	0.042093	6.056501
GLG1	0.978553	0.911206	0.098697	1.456836
ZMYM3	0.978553	0.91121	-0.218	0.92607
ELMSAN1	0.978553	0.911244	0.075701	2.275223
LOC101929	0.978553	0.911259	0.072974	3.027528
ATP23	0.978553	0.911261	0.083676	1.313775
SNORA68	0.978941	0.911959	0.041107	7.375327
CCDC84	0.978941	0.91202	0.108853	1.083194
RNF138	0.978941	0.912039	0.175113	0.543852
LOC102724	0.978941	0.912109	-0.099	3.413922
MED26	0.978941	0.912283	0.051732	4.262304
ANGPTL7	0.978941	0.912329	0.07372	2.982023
SLC25A42	0.978941	0.912334	-0.09431	3.383859
SNORD25	0.978941	0.912357	0.09121	1.246082
WWC2-AS2	0.978941	0.912555	0.095952	1.040571
ANKS6	0.978941	0.912651	-0.0553	5.812924
KPNA2	0.978941	0.912684	-0.07818	5.194247
TBC1D9B	0.978941	0.91275	0.08427	1.976879
UQCRC2	0.978941	0.912778	-0.05356	6.906682
RPLP2	0.978941	0.912815	-0.06061	9.408182
CD2AP	0.978941	0.91289	-0.0489	8.181097
ZNF432	0.978941	0.912921	-0.08499	4.444594
ANGPTL3	0.978941	0.912983	-0.14737	2.638101
CALR	0.978941	0.913195	-0.04881	9.849565
THOC2	0.978941	0.913522	-0.05019	7.83892
EIF3L	0.978941	0.913544	-0.19974	1.869137
TVP23C	0.978941	0.913576	-0.05121	5.82332
SNORD36B	0.978941	0.913609	-0.16377	3.054152

LOC10272З	0.978941	0.913663	-0.4648	0.852693
LRGUK	0.978941	0.913671	0.089209	1.520276
VWA5B1	0.978941	0.913728	-0.37087	1.201429
NOM1	0.978941	0.913747	0.036381	6.117431
TIGIT	0.978941	0.913762	0.113199	1.340673
HERC1	0.979193	0.914077	0.03567	8.438106
LOC100506	0.979398	0.914348	0.076128	3.196158
UCKL1	0.979451	0.914479	-0.28539	1.278114
RPF1	0.979451	0.914557	-0.07274	4.737996
ZNF614	0.979451	0.91472	0.055309	3.495101
DDX28	0.979451	0.914789	0.070585	2.885302
SHKBP1	0.979451	0.914794	0.037279	5.298806
ZSCAN23	0.979544	0.91496	0.100165	1.733343
MAOA	0.979606	0.915098	-0.3179	1.067741
NSMCE2	0.97997	0.915517	0.052848	3.990395
NDST1	0.980008	0.915632	-0.10434	3.148497
ZSCAN22	0.980208	0.915993	0.051819	4.036082
NCAPD3	0.980208	0.916057	-0.05112	5.483338
ATP5F1C	0.980208	0.916077	-0.35781	1.038502
ZSCAN20	0.980208	0.916256	0.070521	3.210212
KLC2	0.980208	0.916287	-0.61947	0.79124
SLC10A1	0.980208	0.916318	0.059958	3.130982
ZNF337	0.980208	0.916451	0.071004	1.76729
ATR	0.980208	0.916562	0.037434	6.523173
LYPLAL1	0.980208	0.916623	0.065206	1.648954
PRR34-AS1	0.980208	0.916671	-0.07536	4.183811
MEIS1-AS2	0.980208	0.916692	0.070872	2.70537
SFT2D3	0.98027	0.916829	0.046842	3.968178
MAP4K4	0.980322	0.916957	0.068681	3.385488
FAM53B-A	0.980937	0.917612	0.088357	1.422698
ZNHIT1	0.981084	0.91784	0.038119	4.950719
DKFZP434k	0.981084	0.918082	-0.30006	1.899209
DHRS12	0.981084	0.918138	-0.16503	2.398812
TTL	0.981084	0.918359	-0.04727	5.751329
ZHX2	0.981084	0.91846	-0.05377	5.241989
UBXN4	0.981084	0.918462	-0.04506	8.142612
FOSB	0.981084	0.918484	-0.27158	1.974097
COX17	0.981084	0.918539	-0.1041	4.473765
SLC25A19	0.981084	0.918551	0.135595	0.607678
ZFYVE16	0.981084	0.918582	-0.22693	1.913294
SELENOH	0.981084	0.918717	0.034795	5.203673
ZNF7	0.981084	0.918788	-0.18999	2.08438
RNASE2	0.981084	0.91884	-0.29824	0.593691
TCHH	0.981084	0.918862	-0.11846	3.595376
USPL1	0.981149	0.919002	0.041671	5.101415

ASCC3	0.981167	0.919099	0.033128	6.722268
ZBTB44	0.981228	0.919241	0.04485	4.979518
DMAC2L	0.981228	0.919315	0.083834	2.036134
LOC10028ɛ	0.981275	0.919546	-0.22073	1.86027
CHCHD3	0.981275	0.919708	-0.36259	0.92189
NFYB	0.981275	0.919715	0.038966	4.832186
ESCO1	0.981275	0.919787	-0.05143	6.476849
PGRMC2	0.981275	0.920046	0.033463	7.079133
B3GNT4	0.981275	0.920066	0.093742	1.49206
MIR200B	0.981275	0.920087	0.072969	1.492332
SLC51A	0.981275	0.920134	-0.21429	2.274178
ST7-AS1	0.981275	0.920139	0.102382	0.99563
ALMS1-IT1	0.981275	0.920154	0.07687	2.222405
ARF4	0.981591	0.920554	0.033783	7.20981
TSPAN14	0.981591	0.920685	-0.08417	3.83765
ABTB2	0.981591	0.920689	-0.07925	4.525765
SNORA61	0.981648	0.920821	0.042502	6.970322
MED14	0.981658	0.920911	0.034199	6.500822
CCDC115	0.981769	0.921152	-0.22305	1.705663
CRELD1	0.981769	0.921225	-0.15906	2.804854
LOC65227ϵ	0.981769	0.921281	0.04888	4.762419
DAB2IP	0.981769	0.921374	-0.05139	5.37971
SHOC2	0.981769	0.921465	0.063029	1.184603
PMS2P9	0.981769	0.921492	0.039762	5.004956
PUM2	0.982061	0.921846	-0.26916	0.936176
PGP	0.982259	0.922111	0.044435	3.728574
TARBP1	0.982506	0.922486	0.032782	6.146512
RNU6ATAC	0.982506	0.922594	0.041179	4.653944
NR2F6	0.982506	0.922693	0.031069	5.847724
REXO1L2P	0.982506	0.922971	-0.60128	0.586746
HLA-DOB	0.982506	0.922992	0.037964	0.520017
PI15	0.982506	0.922992	-0.2022	3.633819
MIR1205	0.982506	0.922997	0.048687	1.556711
TTC7B	0.982506	0.923193	0.05336	3.95233
LOC44002ɛ	0.982506	0.923436	0.089623	0.868154
TNFAIP8L2	0.982506	0.923453	0.076449	0.807988
TOP2A	0.982506	0.923475	0.034061	6.848673
LOC10537ɛ	0.982506	0.923563	0.069534	2.713466
SOCS2-AS1	0.982506	0.923571	-0.33052	0.671374
SCARNA8	0.982506	0.923672	-0.06083	5.123279
ZNF503-AS	0.982506	0.923881	0.099724	1.361746
AGAP6	0.982506	0.923904	-0.08284	4.174537
ZUP1	0.982506	0.924081	0.039632	4.338107
MCF2L2	0.982506	0.924101	0.052797	2.828147
C6orf163	0.982506	0.924142	-0.12282	2.194021

SNORD119	0.982506	0.924144	0.038566	4.833289
VHL	0.982506	0.924234	-0.24049	1.478645
ATXN2	0.982506	0.924374	0.091651	0.490501
PDE6D	0.982506	0.924502	-0.25592	1.751768
TREM1	0.982506	0.924528	0.012142	0.779404
LOC10099€	0.982506	0.924536	-0.46649	0.603857
ARHGAP45	0.982506	0.92455	0.060695	1.729341
SLC12A4	0.982506	0.924579	-0.45505	0.682284
ZNF530	0.982506	0.92461	-0.35455	0.643319
AGBL3	0.982506	0.924722	-0.19881	2.286947
WDR73	0.982506	0.924803	-0.26096	1.141007
GNB1L	0.982506	0.924825	0.070924	1.073762
PRMT6	0.982506	0.92489	0.044363	3.547509
NUTM2F	0.982659	0.925113	0.079696	1.963049
KLRG1	0.983208	0.925731	0.071314	0.601357
SAMD1	0.983208	0.925819	-0.05311	5.73574
SMIM3	0.983208	0.925869	-0.08179	3.663178
CENPU	0.983662	0.926404	0.056143	1.417557
AKAP17A	0.983662	0.926456	0.077263	1.193404
PARP9	0.983864	0.926726	-0.07968	3.949615
PTMAP11	0.983887	0.926878	-0.07901	4.422868
DCAF5	0.983887	0.926907	-0.10172	3.376933
AJM1	0.983924	0.927022	-0.1762	2.542275
STAM	0.983924	0.927101	-0.36068	1.305737
TAF12	0.984108	0.927378	0.047506	2.476101
WARS2-AS:	0.984108	0.927459	0.047601	3.288785
ELAC1	0.984108	0.927601	0.066756	2.643326
THAP4	0.984108	0.927607	0.036496	4.66678
NAIF1	0.984108	0.927703	-0.11832	2.22009
ZNF19	0.984108	0.927752	0.077363	1.772818
PTPN11	0.984125	0.927848	-0.04342	6.922809
C11orf65	0.984696	0.928552	0.034425	1.055861
CDCA5	0.984696	0.928592	-0.11618	3.212602
CCDC9	0.984696	0.928626	-0.05394	5.927172
HINT3	0.984798	0.928801	0.033597	6.202896
ELF3	0.984803	0.928914	-0.10608	3.657866
PTGER1	0.984803	0.928966	0.063343	0.514174
RASSF3	0.984904	0.929186	-0.32519	0.456343
LOC10192ε	0.984904	0.92925	0.086345	1.18558
TMEM179E	0.984904	0.929301	0.037518	4.04983
PRRC2B	0.984936	0.929544	-0.04044	8.775401
ZNHIT2	0.984936	0.929577	0.063127	2.412667
UBALD1	0.984936	0.929582	-0.07468	4.03432
MAP3K14-/	0.984936	0.92965	-0.31726	0.911
MIR22HG	0.984956	0.929788	-0.10173	3.053509

CDKL1	0.984956	0.929861	0.045374	2.942068
TCP1	0.984956	0.929908	-0.20916	1.353599
FBXO11	0.985028	0.930056	0.049149	1.67082
CA9	0.985043	0.93015	-0.20514	4.8537
PMS2P3	0.985171	0.930351	0.031342	4.51377
CCDC183-A	0.985199	0.93061	0.060556	2.632658
ADAMTS12	0.985199	0.930713	-0.13849	3.016863
FCHSD1	0.985199	0.930729	0.040422	4.409029
ZER1	0.985199	0.930766	-0.04808	5.785327
LOC100129	0.985199	0.930806	-0.40121	1.515002
LMBRD2	0.985199	0.930856	-0.04841	5.235341
PCCA-AS1	0.985589	0.931304	-0.11546	2.754169
DOC2B	0.985956	0.931731	-0.12152	3.159413
MSI1	0.986323	0.932158	-0.21462	1.915467
KIF26B	0.986331	0.932245	-0.199	1.672216
TEX22	0.98641	0.932399	-0.18882	2.013685
BCL6	0.98655	0.932612	0.051091	2.752953
CYP2F1	0.986619	0.932757	0.019195	0.871185
LOC100130	0.986665	0.932888	0.094865	0.758812
CYB561	0.986665	0.932982	-0.28771	0.945213
RNU4ATAC	0.986665	0.93304	-0.06213	5.021428
NOMO1	0.986894	0.933336	0.03194	4.349847
ATP6V0D1	0.986985	0.933506	-0.04378	5.230366
GIT2	0.986985	0.9337	-0.09759	2.539569
HIST1H2BL	0.986985	0.933719	0.039928	3.593406
ZNF446	0.986985	0.933775	0.04598	1.972618
PMS2P5	0.986985	0.93393	0.028671	1.884224
CCNYL1	0.986985	0.933973	0.043048	1.84882
EFHC1	0.986985	0.933983	-0.12567	2.256542
TSC22D1-A	0.987012	0.934088	-0.19579	2.195951
FKBP15	0.987023	0.934242	-0.04121	6.552906
CSRNP2	0.987023	0.934319	-0.34006	1.048277
BTBD7	0.987023	0.934338	0.090558	0.717559
SNRPA1	0.987128	0.934517	-0.07576	4.896598
BAIAP2-DT	0.987181	0.934676	-0.07105	3.575008
PDXDC1	0.987181	0.934728	0.027613	1.800133
OGA	0.987309	0.934933	0.069984	1.701008
EID2	0.987309	0.935096	-0.08038	3.176301
PPM1J	0.987309	0.935154	-0.23695	1.1112
RNVU1-6	0.987309	0.935168	-0.25232	0.571642
GPR18	0.987438	0.935405	-0.16075	1.67135
CISD2	0.987438	0.935451	-0.08811	3.523908
CTBP1	0.987465	0.935735	-0.12354	2.836584
CBX1	0.987465	0.935764	0.046867	1.847829
SLX4	0.987465	0.935816	-0.05091	4.903411

KHDC1	0.987465	0.935964	0.049867	1.598738
RNF144A	0.987465	0.935982	0.028382	4.737452
QSOX2	0.987465	0.935987	0.032604	4.202476
CTTN	0.987465	0.936036	-0.13632	2.687501
ARL3	0.987486	0.936225	0.042198	4.014919
DLC1	0.987486	0.936231	0.043056	1.833639
ATXN7	0.987486	0.936335	0.060872	2.678706
STK11	0.987486	0.936376	0.025401	6.563988
SNORD34	0.98754	0.936507	-0.10018	2.798698
CCDC22	0.987651	0.936693	-0.05664	4.197986
MCUB	0.987719	0.936871	0.034218	2.392279
MARS2	0.987719	0.936985	-0.11892	3.1503
GTF2A1	0.987719	0.937001	-0.00303	0.857483
PARTICL	0.987719	0.937077	0.018545	1.360051
HSD17B1	0.98797	0.937562	-0.07467	3.177586
ZNF582	0.98797	0.937569	0.036641	1.623184
GALNT3	0.98797	0.937596	0.024622	7.113723
BLCAP	0.98797	0.937636	-0.06302	4.360478
SHQ1	0.988231	0.938044	0.036532	3.96901
MIR7161	0.988231	0.938073	-0.31027	1.198844
ACOT8	0.988231	0.938123	0.028547	3.891828
CFLAR	0.988379	0.938403	0.026925	6.433283
MIR491	0.988379	0.938532	-0.38429	0.617554
GEMIN8P4	0.988379	0.938538	-0.29723	0.852861
TMEM14EI	0.988379	0.938584	-0.04994	4.590161
NTN5	0.988734	0.939068	0.034796	1.081609
DTWD1	0.988734	0.939106	0.045207	1.098163
THUMPD1	0.988734	0.939161	-0.21948	1.611585
RPS3	0.988851	0.939359	-0.23824	1.045665
TRABD	0.988851	0.939432	-0.05187	5.470588
MRPS26	0.988873	0.939535	-0.06227	4.368093
ZNF287	0.988873	0.939733	0.03562	2.346471
MELTF-AS1	0.988873	0.939836	0.032663	0.974409
ZNF133	0.988873	0.939839	0.04265	1.826479
GOT2	0.988873	0.93987	0.040028	0.920948
ARSD	0.988873	0.939934	-0.06485	6.000147
ATIC	0.988926	0.940064	-0.04457	5.190506
PITRM1-AS	0.988951	0.940217	-0.0793	4.033097
DDTL	0.988951	0.940414	0.045307	2.67363
IGF2BP2-A:	0.988951	0.940472	0.02574	2.621296
GALNT2	0.988951	0.940484	0.05237	1.478622
RETREG2	0.988951	0.940489	-0.04022	5.944166
TES	0.989243	0.940965	0.030991	3.528173
MIR98	0.989243	0.940981	0.02997	1.093593
TRIM11	0.989243	0.941318	0.020425	5.006424

MRPL34	0.989243	0.941342	0.031413	3.337815
ERCC6L	0.989243	0.941373	0.024149	3.210072
CYFIP1	0.989243	0.941382	0.032584	2.3312
NPIPB4	0.989243	0.94167	0.066025	1.919609
ARMH4	0.989243	0.941712	0.028794	2.009674
NOXRED1	0.989243	0.941742	0.050149	0.724853
SF3A1	0.989243	0.941803	-0.03419	7.448755
PA2G4P4	0.989243	0.94181	0.029111	3.45874
HERC4	0.989243	0.941811	-0.10489	3.207911
BEND3P3	0.989243	0.941858	0.028059	4.814377
LAGE3	0.989243	0.941933	0.027052	1.515502
CENPH	0.989243	0.941968	0.035439	3.122085
ZBTB39	0.989455	0.94225	-0.07997	3.968377
LOC10192§	0.989518	0.942391	0.042455	0.726207
SGK494	0.989542	0.942493	0.021183	5.140825
FAM8A1	0.989783	0.942842	-0.03465	5.58874
HIC2	0.989783	0.942913	-0.04825	4.814094
SLC2A9	0.989783	0.943208	0.031517	1.055233
TMEM191/	0.989783	0.943251	0.037287	0.883933
FZR1	0.989783	0.943327	-0.14052	2.373081
SLC9C1	0.989783	0.943355	-0.21156	2.069965
C1orf21	0.989783	0.943371	-0.03594	7.001455
RPA3	0.989783	0.943376	0.023521	3.711477
FAM32A	0.989783	0.943444	-0.03638	5.885901
CITED2	0.989795	0.943536	0.025778	3.65287
RSF1	0.989922	0.943738	0.020967	7.453765
PPP1R3F	0.990037	0.943927	0.045699	2.179647
ZNF33BP1	0.990185	0.944166	-0.21386	1.192288
GATD1	0.990185	0.944313	0.020316	6.586584
LRP1B	0.990185	0.94436	0.027758	1.751887
TPRN	0.990185	0.944389	-0.04632	4.789285
H3F3AP4	0.990203	0.944486	-0.04107	6.37935
RTRAF	0.990205	0.944613	-0.03399	6.02262
ZNF763	0.990205	0.944722	0.037031	1.887726
C10orf111	0.990205	0.944729	-0.34808	0.771197
LPAR5	0.990216	0.94482	-0.36363	0.624618
ZNF324	0.990257	0.944939	0.026085	3.827835
DNAJC14	0.990421	0.945277	-0.0354	5.530462
HBA2	0.990421	0.945369	-0.20516	1.589131
TAOK1	0.990421	0.945496	-0.06153	4.374503
ITGA2B	0.990421	0.945527	-0.00351	1.037145
SNAP23	0.990421	0.945561	-0.23777	0.428922
ZNF580	0.990421	0.945577	0.043102	1.812336
MLLT6	0.990557	0.945887	-0.06046	3.54749
PPIL3	0.990557	0.9459	-0.12896	2.036751

BICRAL	0.990557	0.946009	-0.03409	5.899659
SLC41A1	0.990557	0.946119	-0.04703	5.645835
MYO1E	0.990557	0.946226	0.020446	7.813267
FARS2	0.990557	0.946237	-0.05664	4.066053
ZNF165	0.990557	0.946506	-0.10148	3.495163
LRRC75B	0.990557	0.946597	0.021008	1.853521
BLOC1S3	0.990557	0.946601	-0.08301	3.22825
SND1-IT1	0.990557	0.946605	0.026352	3.754537
TMEM160	0.990557	0.946639	0.027373	2.129724
OLFML2A	0.990557	0.946669	0.038541	2.750498
ZNF766	0.990866	0.947129	-0.04611	4.632139
LUC7L	0.990866	0.947281	0.043873	0.814263
QTRT1	0.990866	0.947286	0.026088	4.528228
CCDC86	0.990866	0.947379	-0.04588	4.216815
SNORD68	0.990866	0.947453	0.023015	4.164498
USF2	0.990866	0.947524	0.004483	1.035932
NHEJ1	0.990866	0.9477	0.032192	3.69261
SNRNP200	0.990866	0.947788	-0.03193	8.562008
CTRL	0.990866	0.947823	0.037414	2.617613
MTRNR2L2	0.990866	0.947887	-0.07179	4.510394
POLE4	0.990866	0.947921	0.020352	3.204211
TULP2	0.990866	0.947928	-0.38553	0.674994
DEF6	0.990905	0.9481	-0.03861	5.933385
UBE2I	0.990905	0.94815	-0.22686	1.180536
TMEM30B	0.990905	0.948285	0.020022	6.44347
GDE1	0.990905	0.948286	-0.03225	5.9171
DHTKD1	0.991097	0.948551	-0.03247	5.708566
MRFAP1L1	0.991167	0.948766	-0.03135	6.878801
IPW	0.991167	0.948888	0.03198	2.497333
COQ3	0.991167	0.948954	-0.07576	2.895008
C12orf66	0.991167	0.949005	-0.01131	1.354859
ZNF316	0.991167	0.949019	0.016697	5.895783
SHANK2-AS	0.991332	0.949297	0.013434	2.163742
FOXO6	0.991332	0.949337	0.065803	1.111
ATP5MC3	0.991375	0.949459	-0.15446	2.132355
DMWD	0.991529	0.949686	-0.03428	5.692913
LOC340512	0.99159	0.949834	0.041874	1.974715
LOC100129	0.99159	0.949905	0.037556	1.173732
HIST1H4B	0.991646	0.95004	0.021038	4.713884
SRGAP2-AS	0.991855	0.950376	0.024709	1.913976
LEPROTL1	0.991855	0.9504	-0.05494	4.409819
HSF1	0.99191	0.950534	0.018137	5.425703
SLC25A5	0.992124	0.950895	0.017919	6.924283
AREL1	0.992124	0.951001	0.017417	6.115412
ZMYM4	0.992124	0.95111	0.01745	7.096726

PDE4B	0.992124	0.951144	-0.40016	0.693264
KIF18A	0.992124	0.95116	-0.11519	2.674379
IFNAR1	0.992124	0.951221	-0.03105	6.488825
LOC28605[illegible]	0.992559	0.951718	0.075518	0.677298
VDR	0.993303	0.952512	0.026566	1.303231
RNF152	0.993483	0.952765	0.019897	4.511284
CDKN1A	0.99368	0.953034	0.02927	2.914288
SNHG4	0.993918	0.953356	0.009553	2.517105
ACAD9	0.993918	0.953424	-0.19794	1.073925
PLBD1	0.993977	0.953561	-0.03717	6.995582
CNPPD1	0.994097	0.953822	0.01678	6.321608
ZNF280C	0.994097	0.953936	-0.05939	3.495617
TPX2	0.994097	0.954096	-0.03814	5.481591
RPS15AP1[illegible]	0.994097	0.954136	0.014687	3.442919
PTBP2	0.994097	0.954152	0.021498	2.993758
CLTB	0.994097	0.954278	0.024458	2.846539
PSMD12	0.994097	0.95443	-0.08145	3.691184
TRMT1L	0.994097	0.954473	-0.00096	1.137547
BORCS5	0.994097	0.954478	-0.20595	1.420133
KIF5A	0.994097	0.954481	0.059658	1.015918
CPLANE2	0.994124	0.9546	-0.11955	2.501139
TAF10	0.994124	0.954668	0.01583	5.140118
PRSS3	0.994153	0.954777	0.010228	1.620358
SLC9A5	0.994209	0.954911	-0.1073	2.064962
PRICKLE2-[illegible]	0.994278	0.955057	0.027138	2.036315
ZNF451-AS	0.994311	0.95517	0.00404	2.024142
KLHL36	0.994409	0.955345	-0.14461	0.821934
SDHAP2	0.994578	0.955588	0.014698	5.949462
ELOF1	0.994623	0.955712	-0.04715	3.964798
FAM208A	0.994812	0.955973	-0.03519	5.370931
ALG2	0.994923	0.956161	0.051141	1.088092
HIST1H2BB	0.994987	0.956303	-0.05131	4.470351
CAMK2D	0.995018	0.956414	0.038391	1.242766
NAP1L5	0.995055	0.9566	-0.07043	3.500429
MTMR12	0.995055	0.95661	-0.17615	1.498393
HDGF	0.99531	0.95699	-0.05665	3.720803
GON7	0.99531	0.957146	-0.10514	2.89615
PSD3	0.99531	0.957167	0.013874	6.369739
PSMA1	0.99531	0.957283	-0.07496	2.795392
AMBRA1	0.99531	0.957334	0.019674	3.234882
LINC01635	0.99531	0.957459	-0.32602	0.701876
PTEN	0.99531	0.957479	0.010142	2.726919
LCN12	0.99531	0.957626	-0.318	0.779021
AK3	0.99531	0.957724	-0.17041	2.057194
SDHAF4	0.99531	0.957806	0.011279	3.505053

ATP6V1FNI	0.99531	0.957883	-0.08318	3.331554
LATS2	0.99531	0.957885	-0.04001	5.44322
SNORA36C	0.99531	0.957904	0.002674	1.160727
STAG3L3	0.995675	0.958407	-0.05214	4.160355
PHF20	0.995675	0.958416	-0.02765	5.891576
SLC25A6	0.995686	0.958539	-0.08343	6.710153
LRRC2	0.995686	0.958588	0.010819	1.69152
MRPL53	0.995853	0.958928	-0.06748	4.08484
HINT2	0.995853	0.959088	0.01974	3.16966
SART3	0.995853	0.959107	0.013369	6.663212
PKD1P6-NI	0.995853	0.959146	-0.06057	3.789944
CD99P1	0.995853	0.959152	-0.3277	0.571754
AKAP6	0.995912	0.959357	-0.05329	3.865594
LOC100505	0.995912	0.95937	-0.04008	4.860852
RAD18	0.9963	0.95988	-0.03818	4.679966
TMEM86A	0.9963	0.959906	0.022514	2.965608
ANKRD40	0.996388	0.960076	0.015529	5.874366
DUS3L	0.996388	0.960155	-0.01135	2.21853
LOC729867	0.996388	0.960232	-0.11885	1.431868
LAMB3	0.996453	0.960467	0.005076	2.956014
WDPCP	0.996453	0.960512	0.028789	1.515111
SNORA2B	0.996453	0.960537	-0.11811	2.556206
RNU4-1	0.996617	0.960807	0.012988	8.302937
BBOF1	0.996617	0.96086	0.014123	3.890802
SNORD93	0.996617	0.960956	0.014562	1.883971
MAML2	0.996617	0.961101	-0.02747	6.839928
LOC101927	0.996617	0.961103	-0.18602	1.638025
POLR1C	0.996617	0.961288	-0.05369	4.206104
TTC30A	0.996617	0.961427	-0.10491	2.793395
OMD	0.996617	0.961498	-0.12862	2.959184
LOC100652	0.996617	0.961588	-0.1343	2.429018
CEP170P1	0.996617	0.96163	0.01972	3.171044
ZNF737	0.996617	0.96166	0.020848	4.246548
RRP7BP	0.996617	0.961663	0.013109	4.855858
FBXL17	0.996699	0.961823	0.014858	5.183539
MOK	0.996749	0.961953	-0.1468	2.37867
PEX7	0.996878	0.962299	-0.139	2.594451
CFAP157	0.996878	0.96231	-0.21386	1.515318
PHF7	0.996878	0.962319	-0.11656	2.352368
NR1H2	0.997022	0.962602	-0.18084	1.652685
CAPN7	0.997022	0.962686	0.013491	6.168077
PI4KB	0.997022	0.9627	-0.01709	1.056585
F8	0.997431	0.963176	-0.16546	2.064773
GDI1	0.997587	0.963408	-0.02647	6.234648
TMEM45B	0.997698	0.963596	0.011631	7.26016

KIAA0825	0.998121	0.964097	0.019318	2.844539
WDFY1	0.998121	0.964166	-0.0252	6.696215
ZNF594	0.998161	0.964285	-0.04867	3.867557
LRRC27	0.998209	0.964484	-0.24056	1.159356
CAPZA2	0.998209	0.964493	0.011284	6.792354
SLC2A4	0.998223	0.964601	0.013065	2.875558
TOMM7	0.998223	0.964762	0.012015	5.896531
BBS2	0.998223	0.964811	-0.03191	5.557629
ZBTB25	0.998223	0.964883	0.011486	2.539644
RNF145	0.998223	0.964911	0.009095	3.263461
LAPTM4B	0.998223	0.965045	-0.03047	4.818983
HMGA1	0.998223	0.965073	-0.15313	1.684486
OPN3	0.998688	0.965603	-0.08077	2.489503
FKBP5	0.998754	0.965748	-0.03007	6.151001
TAS1R3	0.998812	0.965885	-0.06899	3.08126
BAP1	0.999101	0.966246	-0.02654	6.702864
FRG1BP	0.999238	0.966548	0.008637	4.978546
STX19	0.999238	0.966619	-0.03793	4.414597
ADAT2	0.999238	0.966621	-0.00116	3.201408
CIP2A	0.999498	0.96699	0.01293	3.79219
DERA	0.999498	0.967035	-0.08586	3.206931
SNORD12C	0.999609	0.967228	0.00859	4.193801
PDRG1	0.999609	0.967304	0.004873	3.10826
DEPTOR	0.999752	0.967523	-0.11436	2.260893
MUC16	0.99986	0.967709	-0.09562	3.516454
UPF3A	0.999925	0.967852	-0.16823	1.449996
LOC100507	0.99997	0.967977	-0.14222	2.305455
SERINC4	0.999973	0.968124	0.032181	2.008719
ANKRD18A	0.999973	0.968221	0.009986	5.289322
ZSCAN12	0.999973	0.968223	-0.07919	2.199435
GPR143	1	0.968385	-0.1792	1.085113
ZNF599	1	0.968656	-0.11864	1.549418
SERTAD4	1	0.968891	0.015724	3.441041
RPL13AP5	1	0.968899	0.00845	5.550736
C2CD3	1	0.969007	0.012261	3.321163
HTATIP2	1	0.969302	-0.24389	0.887498
BRD9	1	0.969332	-0.0894	1.947867
PTK2B	1	0.969614	-0.21712	0.837029
CENPBD1P	1	0.969979	-0.04305	3.749245
IL1B	1	0.970148	-0.14934	2.363656
ANAPC5	1	0.970344	0.001166	2.320799
SOD1	1	0.970676	0.008569	6.942132
TTLL5	1	0.97075	0.008104	6.365116
LOC101928	1	0.971076	-0.1222	1.302754
SNRPF	1	0.97115	-0.02925	5.968994

CLSTN3	1	0.971167	-0.03052	4.680693
ANO6	1	0.971256	-0.24364	0.664726
COA4	1	0.971365	0.011084	4.262413
BLOC1S4	1	0.971368	-0.00029	2.819695
LOC101927	1	0.971597	0.018156	2.92574
RTCB	1	0.972418	0.006165	6.411808
PPIC	1	0.972433	-0.02349	5.898108
SUGT1P4-S	1	0.972523	-0.14165	1.63914
ATG2A	1	0.972595	0.006075	5.662156
DCAF13P3	1	0.972647	-0.07404	3.124084
LRRC69	1	0.972674	-0.18825	1.224227
CHML	1	0.972714	-0.02389	5.336466
PAQR3	1	0.97288	-0.0401	4.320172
LARS2	1	0.973012	-0.0267	4.840244
EBAG9	1	0.973102	-0.24793	0.829026
ZKSCAN4	1	0.973413	-0.19668	0.847653
SEMA4B	1	0.973543	-0.09019	2.600105
ZNF721	1	0.973715	0.007853	6.322917
WDR11	1	0.973886	-0.01961	6.715331
TMF1	1	0.974349	0.006099	7.504477
IRS1	1	0.974435	-0.02469	6.027383
TMEM60	1	0.974577	-2.86E-05	2.620641
PCNX3	1	0.975132	0.00452	6.634504
TRIM13	1	0.975132	-0.12706	1.484816
LANCL2	1	0.975272	0.008999	4.327515
SSH1	1	0.975398	0.007821	5.909779
ICA1L	1	0.975873	-0.37339	0.623037
PCP2	1	0.97607	-0.22011	0.900051
IMPDH2	1	0.976468	-0.02018	7.219837
CLK2P1	1	0.976589	-0.18129	0.946976
TAPT1	1	0.976728	-0.02569	4.845505
KIAA0754	1	0.977093	-0.02161	6.65197
LINC00899	1	0.977624	-0.12536	1.427282
PPP1R11	1	0.977891	-0.05827	4.682132
XRCC1	1	0.977997	0.009015	4.514225
TMEM170E	1	0.978221	-0.0656	2.669788
CCND2	1	0.978579	0.002347	7.59037
CANT1	1	0.978639	-0.19115	1.849467
EIF1B	1	0.978768	0.008092	3.981826
PNRC1	1	0.978906	0.004134	7.376133
MTG2	1	0.978943	-0.00041	4.392728
CCP110	1	0.979126	-0.19306	0.892805
IPO7	1	0.979451	0.003444	7.809001
NT5DC1	1	0.979452	-0.02845	4.903514
CSPG4	1	0.979481	0.004111	5.551505

LOC101928	1	0.979492	-0.17768	0.95325
BRD1	1	0.979519	0.011823	3.294128
RGP1	1	0.979679	0.003433	4.001325
MT2A	1	0.979769	-0.037	5.940696
FAM199X	1	0.979835	-0.01671	6.800299
ACTR8	1	0.97991	-0.01929	5.106936
LOC102031	1	0.980169	-0.12534	1.726477
ZNF732	1	0.980431	-0.1268	1.963759
SNORD60	1	0.980587	-0.0273	6.22768
MRPL50	1	0.980652	-0.01737	5.055891
ZFYVE19	1	0.980777	-0.18944	1.529445
ENTPD7	1	0.980781	-0.01849	5.97984
ZMYM6	1	0.980888	-0.01878	5.644265
NPM3	1	0.981068	0.000263	3.619436
RNF13	1	0.981244	-0.12826	1.383929
TEFM	1	0.981461	-0.06384	3.242764
ZSCAN29	1	0.981822	-0.01781	5.903371
MKI67	1	0.981847	-0.06616	3.25073
RNASEH2A	1	0.981957	0.009907	3.183433
NPC2	1	0.98196	-0.01744	4.919708
LOC100505	1	0.982051	-0.15818	1.363463
ACSM5	1	0.982173	-0.3598	0.553733
KHSRP	1	0.982316	0.001823	7.415583
ULBP3	1	0.982477	-0.05625	3.274508
ITIH5	1	0.982662	-0.1793	1.361126
KANSL3	1	0.982773	-0.18002	1.185986
INTS6L	1	0.982931	0.00349	4.673053
CASC3	1	0.983016	-0.01588	7.348604
PAPOLG	1	0.983669	0.002053	5.427015
REEP6	1	0.983945	-0.02543	3.843003
DGCR6	1	0.984079	-0.22444	1.455974
TMEM51-A	1	0.984304	-0.05646	3.428119
RBM27	1	0.984336	0.001564	7.041773
SNORA71B	1	0.984469	-0.03643	4.673994
TCF25	1	0.98447	-0.00017	7.457478
KCNQ1	1	0.984705	-0.15733	2.038701
SYTL4	1	0.984769	-0.12217	1.957141
RNF165	1	0.985057	-0.21934	0.897687
CCDC97	1	0.985272	-0.01492	5.834416
FAM104A	1	0.985338	-0.22696	1.214004
DHRS7B	1	0.985468	-0.04503	3.153802
PAXIP1-AS1	1	0.985516	-0.11182	2.342873
SNORD37	1	0.985525	-0.07414	2.499971
ALDH1A1	1	0.985635	-0.03636	7.168193
VTI1A	1	0.985713	-0.01481	6.290305

MED1	1	0.985779	0.000206	6.985613
CDK13	1	0.985913	-0.20644	0.799914
RUNX1-IT1	1	0.986194	-0.15976	1.974186
ZNF485	1	0.986253	-0.07342	2.469666
PCNX2	1	0.986281	-0.01632	5.504135
RBM22	1	0.986377	0.000911	5.553024
TMEM245	1	0.986405	-0.01594	6.478398
SLC16A11	1	0.986461	-0.09506	2.061957
TMX3	1	0.986567	0.000966	5.664414
ADAM10	1	0.986735	-4.40E-05	7.466324
DHX57	1	0.986828	-0.0247	4.973852
KCNJ10	1	0.986946	-0.11528	1.603643
RBM42	1	0.98708	-0.0242	5.561293
VPS53	1	0.987437	-0.01662	6.425834
PLCXD1	1	0.987786	-0.05927	3.607982
KIF20A	1	0.987847	-0.03429	3.634044
RERE	1	0.988969	-0.09399	2.704947
PTGES3	1	0.989356	-0.02124	4.664399
PM20D1	1	0.989439	-0.05446	3.714361
MORN1	1	0.990138	-0.09688	1.589184
KLHL35	1	0.990159	-0.26385	0.922552
MEX3C	1	0.990284	-0.00181	5.609063
SOX12	1	0.990284	-0.01521	4.365825
KLHL20	1	0.990336	-0.0167	5.154983
LTO1	1	0.990525	-0.01892	4.382596
INAFM1	1	0.990598	-0.09237	1.793355
TLCD2	1	0.990848	-0.02576	6.866034
UXS1	1	0.99108	-0.18684	1.147984
PLXNB2	1	0.991307	-0.00314	9.015468
SUB1	1	0.991801	-0.00222	6.731663
SLC16A10	1	0.991908	-0.10874	2.773263
PCDHB17P	1	0.992143	-0.30463	0.48005
MIR661	1	0.992146	-0.24422	0.868904
ELOB	1	0.992515	-0.16668	1.657218
AGAP4	1	0.992525	-0.11619	1.435913
SNORA27	1	0.992589	-0.00363	6.053262
CLEC18B	1	0.992976	-0.12256	1.912207
XRCC4	1	0.993142	-0.20598	0.602389
COX20	1	0.993165	-0.09037	1.736664
SPCS3	1	0.99329	-0.00324	6.897852
LOC100287	1	0.993339	-0.14949	1.10104
RNF43	1	0.993506	-0.05884	3.304384
ATP6AP1L	1	0.993739	-0.02605	3.911111
MIR2278	1	0.994583	-0.12069	1.24884
LOC100126	1	0.994587	-0.0577	2.741023

FAM120AC	1	0.994686	-0.01443	5.329342
NCOA7	1	0.995107	-0.05082	2.506262
ZNF516	1	0.995276	-0.00353	6.414322
TTYH2	1	0.995543	-0.09506	0.937132
EIF2B5	1	0.995571	-0.01149	6.276799
PPP2R3B	1	0.996029	-0.08071	2.240061
CDC40	1	0.996474	-0.0072	5.74976
ZSCAN12P1	1	0.996747	-0.08628	2.033648
OSBPL11	1	0.996925	-0.0088	5.331897
LINC01559	1	0.99693	-0.04323	4.721975
TACC3	1	0.996941	-0.01502	4.906475
MEF2A	1	0.997028	-0.10691	1.486715
CCNT2	1	0.997464	-0.21055	0.509542
ALG10B	1	0.997501	-0.02899	3.141384
CASZ1	1	0.997739	-0.01436	5.737374
SRSF10	1	0.997775	-0.02771	3.69129
ACIN1	1	0.998035	-0.02257	3.363084
STEAP1	1	0.998328	-0.0355	3.359554
TUFM	1	0.998389	-0.00964	7.122201
TJAP1	1	0.998441	-0.19731	0.591658
HEXIM1	1	0.998502	-0.00686	6.316851
ADPRM	1	0.998762	-0.03156	3.109848
RGPD2	1	0.998765	-0.1701	0.812101
DHRS7	1	0.998833	-0.01436	5.635769
KLHL25	1	0.998835	-0.03237	4.077767
C5orf15	1	0.999066	-0.00979	5.031187
NOC3L	1	0.999382	-0.00857	5.677371
GOLGA6L1(	1	0.999804	-0.05064	2.082432
UBR2	1	0.999898	-0.00697	7.109752
MKLN1-AS	1	0.999999	-0.0655	2.180169
CHEK2P2	1	1	-0.43723	0.431054
GATA5	1	1	-0.32083	0.713315
LOC101927	1	1	-0.26065	0.511563
PCGF3	1	1	-0.24007	0.625568
LRRC37A6F	1	1	-0.21603	0.90902
SNORA29	1	1	-0.20753	0.487979
EPN2	1	1	-0.19645	0.794362
LINC01603	1	1	-0.19093	0.663166
ERCC1	1	1	-0.18421	0.59513
MIR616	1	1	-0.18155	1.072085
MYH7	1	1	-0.18058	0.665026
STAG3L5P-	1	1	-0.18032	0.900987
SPESP1	1	1	-0.17912	0.767843
OR2D3	1	1	-0.17861	1.326255
GALT	1	1	-0.17659	0.626403

RBM39	1	1	-0.17505	0.465384
LINC01189	1	1	-0.17458	0.516504
EZR	1	1	-0.14753	1.155705
FAM41C	1	1	-0.14503	0.749267
TMEM151I	1	1	-0.14456	0.763889
FAM45A	1	1	-0.14179	0.889463
SDS	1	1	-0.13792	0.50189
RUNDC3A-/	1	1	-0.13753	0.50989
EPX	1	1	-0.13585	0.567316
PID1	1	1	-0.13174	0.70734
C1QTNF12	1	1	-0.12963	0.752991
MINK1	1	1	-0.12919	1.18459
CCM2	1	1	-0.12725	0.597547
TMEM250	1	1	-0.12723	1.133773
DBH-AS1	1	1	-0.12537	0.855608
UVSSA	1	1	-0.12487	1.220131
LINC01118	1	1	-0.12071	0.690552
MIR200C	1	1	-0.1205	0.510359
TTLL13P	1	1	-0.11639	0.561249
NIPBL	1	1	-0.11522	0.642798
SP5	1	1	-0.11457	1.161962
CCR4	1	1	-0.11314	1.507269
TCTN3	1	1	-0.11196	1.214875
MATN1-AS	1	1	-0.11164	0.470587
MIR646HG	1	1	-0.09758	0.602928
CNOT2	1	1	-0.09714	0.961084
LOC101928	1	1	-0.09536	1.347596
UBE2E1	1	1	-0.09514	1.455567
NOTCH4	1	1	-0.09507	0.658685
YY2	1	1	-0.09502	1.763846
MFSD5	1	1	-0.09473	1.281998
EEF1E1	1	1	-0.09374	1.376723
FKBP11	1	1	-0.09343	1.708621
CCDC154	1	1	-0.08998	1.327314
NUMA1	1	1	-0.08959	0.78524
IFT43	1	1	-0.08839	0.783892
SNORD114	1	1	-0.08834	1.273526
LIN7A	1	1	-0.08781	0.940728
DLGAP1-AS	1	1	-0.08687	1.140669
RBFOX3	1	1	-0.08562	0.90741
PDGFB	1	1	-0.08546	1.636574
SAP30BP	1	1	-0.08329	1.399262
MOB3C	1	1	-0.08154	1.778917
CXXC5	1	1	-0.07986	1.669071
CD7	1	1	-0.07698	1.638945

DHX9	1	1	-0.07657	1.501712
PDIA5	1	1	-0.07478	0.52339
SMARCD3	1	1	-0.07472	2.2569
ZNF638	1	1	-0.07348	1.303021
SERPINA3	1	1	-0.07271	1.519455
SRPRA	1	1	-0.07185	1.25307
NUDT17	1	1	-0.07077	1.338309
STX18-AS1	1	1	-0.06876	2.198025
TTC39A-AS	1	1	-0.06873	1.736545
DCAF10	1	1	-0.06784	1.818203
ATRX	1	1	-0.06746	0.755081
RACGAP1	1	1	-0.06658	1.813495
SNORD6	1	1	-0.06577	1.022505
CMC4	1	1	-0.06509	1.958197
ZNF708	1	1	-0.06446	1.133619
NDST3	1	1	-0.06342	0.94407
NDUFAF4P	1	1	-0.06333	1.086762
SIX4	1	1	-0.06228	2.535867
C10orf143	1	1	-0.06212	1.199323
LEAP2	1	1	-0.06202	1.788479
PCGF2	1	1	-0.06089	0.533847
TMUB2	1	1	-0.05953	1.715931
PIN4P1	1	1	-0.05877	1.800437
TIAF1	1	1	-0.05855	1.943248
NCOA7-AS1	1	1	-0.05835	0.530787
STRADA	1	1	-0.05649	2.003161
KIAA0895	1	1	-0.05588	1.402563
G0S2	1	1	-0.05544	2.754998
SNORD135	1	1	-0.05346	2.631712
MIR5687	1	1	-0.05312	0.785081
PCSK9	1	1	-0.05312	0.617939
CEP164	1	1	-0.05272	2.456163
LEO1	1	1	-0.05014	2.546248
MROH2A	1	1	-0.04826	0.503881
EFHC2	1	1	-0.04701	1.63121
GPX7	1	1	-0.04654	2.139263
RAB26	1	1	-0.04612	0.753692
ETFB	1	1	-0.04415	1.906144
NEFH	1	1	-0.04389	2.158609
SPG21	1	1	-0.04301	0.819257
CKMT2-AS1	1	1	-0.04294	1.77466
LOC100422	1	1	-0.04245	1.57794
FITM2	1	1	-0.04185	1.82147
M6PR	1	1	-0.04152	1.698005
SECISBP2L	1	1	-0.0411	0.686829

ZNF384	1	1	-0.04035	0.860563
TMEM68	1	1	-0.04031	2.61112
RAB18	1	1	-0.03975	2.737958
ZNF784	1	1	-0.03916	2.335806
LINC00312	1	1	-0.03892	1.694185
ZBTB4	1	1	-0.03887	2.743595
ZMYND8	1	1	-0.03838	2.483151
A1BG-AS1	1	1	-0.03813	0.527182
NDN	1	1	-0.03811	2.48038
ZNF628	1	1	-0.0381	3.112091
HIST2H2BC	1	1	-0.03641	2.078924
DENND2D	1	1	-0.03572	2.711677
MIR29B2	1	1	-0.03542	1.003318
ASAH1	1	1	-0.03541	1.513027
POLR2E	1	1	0.035291	0.657356
C2orf92	1	1	-0.03418	1.405916
AR	1	1	-0.0341	1.528419
STRC	1	1	-0.03399	0.72622
NAGPA	1	1	-0.03392	2.620705
SNORD43	1	1	-0.03275	1.363991
CYB5R3	1	1	-0.03257	1.611119
ADSL	1	1	-0.03228	0.621094
SPAG9	1	1	-0.03194	2.802853
GDPD4	1	1	-0.03039	1.102147
LIMD2	1	1	-0.03033	2.361165
CCDC60	1	1	-0.03016	1.597508
SLC39A1	1	1	-0.02945	0.562079
BOC	1	1	-0.02875	2.824258
ARNTL2	1	1	-0.02799	1.041545
TRMT61A	1	1	-0.02687	3.681389
DSE	1	1	-0.02602	1.037085
ATXN2L	1	1	-0.02556	2.436066
MMP25-AS	1	1	-0.02522	2.476759
SOX17	1	1	0.024899	1.327477
GCKR	1	1	-0.02466	3.049831
GZMA	1	1	-0.0223	2.065211
LINC00260	1	1	-0.02132	2.529554
PHF11	1	1	-0.02122	1.904507
USP7	1	1	-0.02097	3.343181
SRSF8	1	1	-0.02083	2.286414
DHODH	1	1	-0.02082	3.812992
DNAJB12	1	1	-0.02043	2.516294
REL	1	1	-0.02021	0.84459
C19orf48	1	1	-0.02007	1.676711
TRIM27	1	1	-0.02005	2.94926

LOC10272З	1	1	-0.02003	1.531533
RASSF10	1	1	-0.01979	4.228641
LOC101928	1	1	-0.01816	1.040362
KDM4C	1	1	-0.01785	3.892696
FAM214B	1	1	-0.01777	1.537752
SLC45A4	1	1	-0.01636	3.413681
CA3	1	1	-0.01626	1.90136
KIAA1656	1	1	-0.01527	2.483196
CAMK4	1	1	-0.01488	2.364859
SMC6	1	1	-0.01485	1.391286
PLXDC2	1	1	-0.01422	0.813345
LINC01145	1	1	0.014154	0.944493
C1QTNF7	1	1	-0.01369	0.70697
ZNF304	1	1	0.013464	1.115575
SRSF12	1	1	-0.01319	2.366256
GJB4	1	1	-0.01171	2.741557
RNFT1	1	1	-0.01126	3.951228
HCG18	1	1	-0.01121	4.560389
SCARNA11	1	1	-0.01112	2.862786
DROSHA	1	1	-0.01105	0.559934
VSTM2L	1	1	-0.01096	1.482551
TLK1	1	1	-0.0109	3.359648
MAGOH	1	1	-0.01061	4.177801
DERL2	1	1	-0.01041	1.664306
RUNX3	1	1	0.010025	1.479444
AKR7A2P1	1	1	-0.00941	2.071097
HBB	1	1	-0.00936	3.162586
PRR11	1	1	-0.00892	4.264009
UTP25	1	1	-0.00873	5.489029
RAB5C	1	1	-0.00854	0.424487
EPS15	1	1	-0.00837	5.082393
HIST4H4	1	1	-0.0083	3.389412
ASAP1	1	1	-0.00827	1.340838
FBXL8	1	1	-0.00804	2.202335
PLEKHA8	1	1	-0.00776	5.803381
AP5M1	1	1	-0.00746	3.661767
C2orf42	1	1	-0.00706	3.296468
GNAO1	1	1	-0.00701	2.431613
CAMKMT	1	1	-0.007	3.548659
C1orf123	1	1	0.006565	1.955771
FAM86HP	1	1	-0.00606	2.170283
STAT1	1	1	-0.00599	5.39175
TIMELESS	1	1	-0.00564	5.616502
DDX46	1	1	-0.00558	1.446301
C12orf45	1	1	-0.00556	3.32443

MIR1207	1	1	-0.00554	1.896618
F3	1	1	-0.00395	3.841738
LINC01278	1	1	0.00346	4.909774
MBNL1	1	1	-0.00335	3.188231
TCF7L1	1	1	0.002913	4.150492
ZNF25	1	1	-0.00258	4.20179
MRPS36	1	1	-0.00205	3.039808
MTMR7	1	1	-0.00158	3.434671
LOC101927	1	1	-0.00141	1.789474
THNSL1	1	1	-0.00119	1.84048
ARRDC2	1	1	0.001057	0.627021
PFAS	1	1	-0.00102	4.92319
TUT1	1	1	0.000811	4.738455
GPRC5D-AS	1	1	0.000756	3.215959
TMPO-AS1	1	1	0.000713	0.498159
MPL	1	1	-0.00045	2.074645
UBE2D2	1	1	0.000356	2.707915
