# Supplementary material for: Transcriptomic Profiling of Laser Capture Microdissected FFPE Esophageal Tissues Identifies Candidate Genes and Pathways Associated with Esophageal Adenocarcinoma
Source: Int J Mol Sci. 2026 Sep 14;27(18):8164. doi: 10.3390/ijms27188164 (PMC13607975; doi:10.3390/ijms27188164)
Supplement: Supplementary file 1 [file ijms-27-08164-s001.zip › Supplementary Table S3_WT vs AC-DE.pdf]

| GeneSymb | FDR | pval | log2FC | log2CPM |
|---|---|---|---|---|
| DAPL1 | 6.13E-22 | 4.67E-26 | -10.5371 | 4.132894 |
| TIAM1 | 6.66E-18 | 1.02E-21 | -4.17191 | 8.002787 |
| EHD3 | 7.24E-18 | 1.68E-21 | -4.09381 | 6.637627 |
| CAPNS2 | 7.24E-18 | 2.26E-21 | -7.04622 | 4.453135 |
| HOXA13 | 7.24E-18 | 2.76E-21 | 7.932394 | 3.970182 |
| PITX1 | 3.54E-17 | 1.62E-20 | -4.72029 | 8.695274 |
| HCG22 | 1.68E-16 | 8.99E-20 | -7.53117 | 5.538934 |
| TTC9 | 2.77E-16 | 1.69E-19 | -4.49222 | 7.257618 |
| TMPRSS11 | 2.80E-16 | 1.92E-19 | -8.11567 | 6.19948 |
| FRMD6 | 2.90E-16 | 2.21E-19 | -6.06069 | 4.335844 |
| IL20RB | 3.37E-16 | 2.91E-19 | -5.61864 | 6.699051 |
| GRHL3 | 3.37E-16 | 3.14E-19 | -5.76882 | 5.199037 |
| PPL | 3.37E-16 | 3.34E-19 | -4.94225 | 12.29858 |
| TMEM154 | 6.17E-16 | 6.58E-19 | -4.34906 | 7.540023 |
| KRT15 | 7.37E-16 | 8.43E-19 | -7.08261 | 9.648221 |
| N4BP3 | 1.24E-15 | 1.51E-18 | -4.04267 | 5.550234 |
| CYSRT1 | 1.25E-15 | 1.62E-18 | -5.08046 | 6.076067 |
| HOXC10 | 1.25E-14 | 1.72E-17 | 9.938175 | 4.82482 |
| RHCG | 1.27E-14 | 1.86E-17 | -7.7467 | 4.199052 |
| SPRR3 | 1.27E-14 | 1.93E-17 | -8.03176 | 4.703373 |
| CAPN14 | 1.89E-14 | 3.03E-17 | -7.50183 | 9.203647 |
| SLURP1 | 2.88E-14 | 4.83E-17 | -7.95487 | 6.240728 |
| SCNN1B | 2.21E-13 | 3.93E-16 | -6.43973 | 6.046273 |
| GLTP | 2.21E-13 | 4.03E-16 | -4.72709 | 9.054846 |
| FAT2 | 4.31E-13 | 8.21E-16 | -5.25609 | 9.472983 |
| S100A14 | 6.61E-13 | 1.31E-15 | -4.70566 | 10.76856 |
| ZNF750 | 6.92E-13 | 1.42E-15 | -4.24276 | 7.563444 |
| MISP | 8.39E-13 | 1.79E-15 | 6.444936 | 6.729179 |
| VSIG10L | 2.10E-12 | 4.65E-15 | -5.44825 | 8.192816 |
| MPP7 | 2.53E-12 | 5.78E-15 | -3.35398 | 7.645646 |
| MAB21L3 | 3.90E-12 | 9.22E-15 | -5.50831 | 5.275566 |
| TENT5B | 5.22E-12 | 1.27E-14 | -3.7695 | 5.663089 |
| SLURP2 | 6.76E-12 | 1.70E-14 | -7.3992 | 3.545625 |
| CLIC3 | 8.25E-12 | 2.14E-14 | -4.85949 | 6.014047 |
| ADH7 | 1.12E-11 | 3.00E-14 | -8.06248 | 2.869221 |
| NDUFA4L2 | 1.87E-11 | 5.14E-14 | -5.04995 | 6.926646 |
| PAX9 | 2.49E-11 | 7.03E-14 | -5.30596 | 7.278148 |
| CSTB | 3.38E-11 | 9.80E-14 | -5.97256 | 12.12155 |
| TP53AIP1 | 4.17E-11 | 1.24E-13 | -7.951 | 2.763711 |
| F5 | 7.44E-11 | 2.27E-13 | 6.786009 | 5.61031 |
| PPP1R3C | 8.19E-11 | 2.56E-13 | -4.74785 | 7.721275 |
| SLC25A21 | 8.51E-11 | 2.72E-13 | -5.18272 | 4.521148 |
| SUSD4 | 8.58E-11 | 2.81E-13 | -5.34694 | 4.802096 |
| DSG1-AS1 | 9.11E-11 | 3.06E-13 | -8.73037 | 2.396397 |

| | | | | |
|---|---|---|---|---|
| HOXB5 | 1.19E-10 | 4.07E-13 | 8.008193 | 3.605055 |
| BARX2 | 1.25E-10 | 4.37E-13 | -4.8229 | 7.603635 |
| GNA15 | 1.25E-10 | 4.49E-13 | -3.44733 | 5.817598 |
| GRHL1 | 1.34E-10 | 4.89E-13 | -5.19163 | 8.866766 |
| EVPLL | 1.34E-10 | 4.99E-13 | -6.06674 | 3.78543 |
| ARHGAP40 | 1.34E-10 | 5.12E-13 | -4.96188 | 5.625677 |
| ANXA1 | 3.02E-10 | 1.17E-12 | -4.67658 | 12.59721 |
| CDX2 | 3.49E-10 | 1.38E-12 | 7.281128 | 2.910022 |
| CYP4F22 | 4.83E-10 | 1.95E-12 | -6.08357 | 5.81587 |
| CRYBG1 | 7.75E-10 | 3.19E-12 | -3.22406 | 9.511839 |
| PRR15 | 8.21E-10 | 3.44E-12 | 5.806492 | 4.062833 |
| SASH1 | 9.52E-10 | 4.06E-12 | -2.82759 | 8.80382 |
| FUT4 | 1.08E-09 | 4.71E-12 | 3.673959 | 5.604207 |
| ELOVL4 | 1.35E-09 | 5.98E-12 | -4.90198 | 5.021473 |
| TINCR | 1.44E-09 | 6.48E-12 | -4.65051 | 4.245616 |
| C5orf66 | 1.95E-09 | 8.94E-12 | -2.99803 | 7.09628 |
| DSG1 | 2.47E-09 | 1.15E-11 | -6.69692 | 8.16098 |
| OTOP2 | 2.69E-09 | 1.27E-11 | -5.9621 | 4.529589 |
| KRT13 | 3.05E-09 | 1.46E-11 | -7.90435 | 6.564441 |
| HSPB1 | 3.12E-09 | 1.52E-11 | -3.48682 | 10.03439 |
| DSP | 4.42E-09 | 2.19E-11 | -3.84678 | 11.71609 |
| EMP1 | 5.03E-09 | 2.53E-11 | -4.42534 | 12.02291 |
| EPHA1 | 5.21E-09 | 2.66E-11 | -3.13337 | 6.897136 |
| DLEU1 | 5.26E-09 | 2.73E-11 | 6.382794 | 2.803244 |
| CELSR2 | 5.45E-09 | 2.87E-11 | -3.17817 | 7.29481 |
| PADI3 | 6.14E-09 | 3.28E-11 | -4.39761 | 5.025444 |
| SOX7 | 7.63E-09 | 4.13E-11 | -3.51184 | 5.891075 |
| SLC9A3R1 | 8.41E-09 | 4.62E-11 | -2.79598 | 8.051136 |
| CDHR1 | 9.59E-09 | 5.34E-11 | -5.3903 | 4.56651 |
| EVPL | 1.13E-08 | 6.45E-11 | -4.34971 | 10.56847 |
| KRT1 | 1.13E-08 | 6.46E-11 | -9.58362 | 8.295338 |
| DHCR24 | 1.18E-08 | 6.86E-11 | -3.22871 | 8.68472 |
| ADGRF4 | 1.19E-08 | 7.05E-11 | -4.38919 | 5.032507 |
| GBP6 | 1.19E-08 | 7.10E-11 | -8.41886 | 10.40098 |
| TRIM31 | 1.30E-08 | 7.83E-11 | 7.235338 | 3.272271 |
| CTTNBP2 | 1.89E-08 | 1.15E-10 | -3.60343 | 6.367672 |
| IGFL1 | 2.21E-08 | 1.37E-10 | -6.07716 | 4.906008 |
| LGALS7B | 2.39E-08 | 1.50E-10 | -6.30845 | 4.588662 |
| TRIM16 | 2.42E-08 | 1.53E-10 | -2.79482 | 7.471776 |
| MIR194-2H | 2.60E-08 | 1.67E-10 | 5.964163 | 3.300125 |
| LTB4R | 2.60E-08 | 1.68E-10 | -3.09069 | 6.498445 |
| SDR9C7 | 2.83E-08 | 1.87E-10 | -6.23213 | 3.590627 |
| CERS3-AS1 | 2.83E-08 | 1.87E-10 | -6.50177 | 3.488392 |
| GSDMC | 3.01E-08 | 2.02E-10 | -5.34822 | 6.514274 |
| NECTIN1 | 3.09E-08 | 2.09E-10 | -3.70274 | 8.267548 |

| | | | | |
|---|---|---|---|---|
| RRAGD | 3.34E-08 | 2.29E-10 | -2.62143 | 6.159913 |
| SERPINB13 | 3.85E-08 | 2.67E-10 | -8.57881 | 2.255433 |
| PRSS27 | 4.10E-08 | 2.88E-10 | -4.80556 | 6.537688 |
| FRMD6-AS: | 4.23E-08 | 3.00E-10 | -5.46045 | 3.850914 |
| SERPINB2 | 4.47E-08 | 3.20E-10 | -6.93641 | 2.37958 |
| MKNK2 | 4.71E-08 | 3.41E-10 | -2.72586 | 7.714234 |
| LGALS4 | 5.89E-08 | 4.31E-10 | 4.757209 | 5.994364 |
| ATP13A4 | 6.42E-08 | 4.74E-10 | -4.5325 | 6.937516 |
| RAET1E | 7.04E-08 | 5.26E-10 | -5.42605 | 6.443856 |
| BCL11A | 7.28E-08 | 5.49E-10 | -3.39775 | 4.568746 |
| IL12A | 7.49E-08 | 5.71E-10 | -6.54303 | 2.459399 |
| IGLON5 | 7.65E-08 | 5.89E-10 | -4.14296 | 4.036768 |
| A2ML1 | 8.35E-08 | 6.49E-10 | -7.18207 | 9.733455 |
| RNF222 | 8.39E-08 | 6.58E-10 | -5.41678 | 4.836717 |
| NIPAL1 | 9.55E-08 | 7.63E-10 | -3.44172 | 6.607102 |
| KCTD11 | 9.55E-08 | 7.64E-10 | -3.43212 | 6.860334 |
| AHDC1 | 1.01E-07 | 8.19E-10 | -2.74082 | 7.596896 |
| FLG | 1.03E-07 | 8.40E-10 | -6.15107 | 9.289929 |
| ASPG | 1.10E-07 | 9.05E-10 | -3.93544 | 3.951855 |
| MPZL2 | 1.19E-07 | 9.93E-10 | -3.22416 | 7.878237 |
| PKP1 | 1.24E-07 | 1.04E-09 | -5.61144 | 3.892955 |
| FAM162A | 1.26E-07 | 1.07E-09 | -2.55563 | 6.518826 |
| TPRG1 | 1.47E-07 | 1.25E-09 | -4.2312 | 6.566473 |
| MYO7B | 1.48E-07 | 1.27E-09 | 4.80853 | 5.673288 |
| C10orf99 | 1.60E-07 | 1.40E-09 | -6.52272 | 5.870218 |
| KRT4 | 1.60E-07 | 1.40E-09 | -9.81894 | 14.55576 |
| ANKRD20A | 1.72E-07 | 1.53E-09 | -6.07123 | 6.119134 |
| BTBD11 | 1.72E-07 | 1.53E-09 | -5.11294 | 5.902454 |
| SNX24 | 1.83E-07 | 1.65E-09 | -2.90774 | 5.463666 |
| AEBP1 | 2.21E-07 | 2.00E-09 | 3.467414 | 8.134802 |
| STON2 | 2.95E-07 | 2.70E-09 | -2.52696 | 6.801543 |
| SFRP4 | 3.01E-07 | 2.78E-09 | 6.67201 | 5.745644 |
| FAM83A-A: | 3.13E-07 | 2.91E-09 | -5.46067 | 5.12455 |
| ALOX12 | 3.32E-07 | 3.11E-09 | -4.23974 | 7.315767 |
| FAM25A | 3.75E-07 | 3.55E-09 | -6.63834 | 6.404867 |
| CTSZ | 3.88E-07 | 3.70E-09 | 2.70831 | 6.488098 |
| MAB21L4 | 4.04E-07 | 3.87E-09 | -5.28875 | 3.593014 |
| NUAK2 | 4.11E-07 | 3.98E-09 | -3.59929 | 7.093672 |
| CLCA4 | 4.16E-07 | 4.06E-09 | -8.3913 | 2.083119 |
| LOC38960: | 4.41E-07 | 4.33E-09 | 6.682295 | 4.331171 |
| EPCAM | 4.49E-07 | 4.45E-09 | 3.594093 | 7.894575 |
| SLC10A6 | 5.13E-07 | 5.12E-09 | -4.81068 | 4.804626 |
| FBXO27 | 5.66E-07 | 5.69E-09 | -3.8775 | 3.570801 |
| KLK11 | 5.90E-07 | 5.98E-09 | -4.0272 | 3.769621 |
| SDC3 | 6.12E-07 | 6.25E-09 | 2.807033 | 6.161754 |

| | | | | |
|---|---|---|---|---|
| LGALS7 | 6.20E-07 | 6.39E-09 | -7.2774 | 2.731452 |
| GJB2 | 6.20E-07 | 6.43E-09 | -5.61412 | 10.9432 |
| A2M | 6.27E-07 | 6.54E-09 | 3.494971 | 10.10439 |
| TACSTD2 | 6.32E-07 | 6.65E-09 | -3.81098 | 10.35409 |
| LGI2 | 6.58E-07 | 6.97E-09 | 6.221905 | 3.406267 |
| ERO1A | 6.97E-07 | 7.44E-09 | -3.43081 | 10.03417 |
| C6orf222 | 7.24E-07 | 7.82E-09 | 6.382886 | 4.022532 |
| PGM2 | 7.24E-07 | 7.83E-09 | -2.80864 | 6.887788 |
| KLC3 | 7.30E-07 | 7.96E-09 | -3.19502 | 4.840324 |
| PGLYRP3 | 7.44E-07 | 8.16E-09 | -5.04745 | 4.426927 |
| DIO2 | 7.50E-07 | 8.29E-09 | -6.0877 | 3.095442 |
| HCAR2 | 7.72E-07 | 8.59E-09 | -4.4988 | 4.661024 |
| MIR205 | 7.96E-07 | 8.92E-09 | -7.99004 | 1.718655 |
| SOX15 | 8.37E-07 | 9.44E-09 | -4.28733 | 4.443234 |
| SPIRE2 | 9.31E-07 | 1.06E-08 | 2.837453 | 4.775333 |
| GJB6 | 9.64E-07 | 1.11E-08 | -6.15837 | 4.730688 |
| LOC10537З | 9.64E-07 | 1.12E-08 | 7.534681 | 2.435781 |
| LINC02487 | 9.64E-07 | 1.12E-08 | -6.04139 | 7.620157 |
| LINC00958 | 9.79E-07 | 1.14E-08 | -5.43516 | 3.781869 |
| LBH | 9.79E-07 | 1.15E-08 | 3.438209 | 6.070645 |
| DEFB1 | 1.03E-06 | 1.22E-08 | -4.11248 | 3.653055 |
| PALMD | 1.06E-06 | 1.27E-08 | -2.42634 | 6.488485 |
| FAM83C | 1.20E-06 | 1.43E-08 | -5.24135 | 5.859942 |
| FOXE1 | 1.29E-06 | 1.55E-08 | -4.36488 | 4.061834 |
| CCNG2 | 1.32E-06 | 1.60E-08 | -2.56869 | 7.543356 |
| GPX3 | 1.34E-06 | 1.63E-08 | -2.59641 | 8.243256 |
| TMC5 | 1.37E-06 | 1.68E-08 | 4.787328 | 3.861417 |
| FTLP10 | 1.39E-06 | 1.71E-08 | -5.41764 | 3.700235 |
| TMEM184/ | 1.45E-06 | 1.80E-08 | -2.72211 | 7.75861 |
| TGM3 | 1.58E-06 | 1.97E-08 | -8.4069 | 10.73257 |
| IFFO2 | 1.71E-06 | 2.16E-08 | -3.14673 | 8.042812 |
| CSTA | 1.71E-06 | 2.16E-08 | -7.75629 | 10.45292 |
| P2RY1 | 1.83E-06 | 2.34E-08 | -4.01963 | 6.482606 |
| C3 | 1.83E-06 | 2.34E-08 | 3.873183 | 8.158518 |
| ZNF431 | 1.87E-06 | 2.41E-08 | -2.52698 | 7.076082 |
| APOC1 | 2.00E-06 | 2.60E-08 | 7.994104 | 1.72437 |
| GPR157 | 2.00E-06 | 2.60E-08 | -3.61062 | 4.31162 |
| LOC442497 | 2.22E-06 | 2.91E-08 | 9.293251 | 2.936422 |
| EREG | 2.38E-06 | 3.13E-08 | -3.49383 | 6.939981 |
| FABP5 | 2.59E-06 | 3.44E-08 | -4.97436 | 9.977805 |
| TTC22 | 2.65E-06 | 3.53E-08 | -3.3775 | 5.358444 |
| CYP4F29P | 2.71E-06 | 3.63E-08 | -5.19647 | 4.63586 |
| ARHGAP23 | 2.78E-06 | 3.75E-08 | -2.77477 | 5.312428 |
| ZNF812P | 2.90E-06 | 3.94E-08 | -4.70429 | 5.192329 |
| LYZ | 2.90E-06 | 3.96E-08 | 5.997835 | 10.22642 |

| | | | | |
|---|---|---|---|---|
| RHPN2 | 2.90E-06 | 3.97E-08 | 3.563114 | 6.21455 |
| ELL2 | 3.06E-06 | 4.24E-08 | -2.26248 | 8.669246 |
| MUC21 | 3.06E-06 | 4.25E-08 | -6.01699 | 2.513756 |
| KRT78 | 3.09E-06 | 4.31E-08 | -6.50875 | 5.619267 |
| NECTIN4 | 3.14E-06 | 4.41E-08 | -3.26413 | 8.46525 |
| CSGALNAC | 3.15E-06 | 4.44E-08 | 2.395307 | 6.185971 |
| RHOV | 3.15E-06 | 4.46E-08 | -4.81667 | 6.791765 |
| BHLHE40 | 3.17E-06 | 4.52E-08 | -2.67925 | 9.737799 |
| ESRP2 | 3.23E-06 | 4.62E-08 | -2.20334 | 7.099766 |
| C6orf132 | 3.29E-06 | 4.74E-08 | -2.30843 | 9.383639 |
| NPEPPS | 3.31E-06 | 4.80E-08 | -2.23575 | 8.605419 |
| CDR2 | 3.40E-06 | 4.95E-08 | 2.555428 | 5.50916 |
| GRPEL2 | 3.54E-06 | 5.18E-08 | -3.22923 | 7.220283 |
| RAB10 | 3.58E-06 | 5.27E-08 | -2.74653 | 9.549295 |
| B3GNT8 | 3.60E-06 | 5.32E-08 | -2.72591 | 4.644719 |
| FAM83A | 3.70E-06 | 5.50E-08 | -5.82019 | 6.361498 |
| CRNN | 3.73E-06 | 5.57E-08 | -8.45848 | 12.02798 |
| TRIM29 | 3.91E-06 | 5.87E-08 | -3.34578 | 10.2033 |
| ZBED2 | 4.01E-06 | 6.06E-08 | -3.15617 | 4.389818 |
| GAN | 4.05E-06 | 6.15E-08 | -2.10981 | 6.67787 |
| LOC154761 | 4.18E-06 | 6.37E-08 | -2.75711 | 4.859304 |
| RNF141 | 4.28E-06 | 6.55E-08 | -2.41089 | 7.422461 |
| CERS4 | 4.37E-06 | 6.73E-08 | -3.6024 | 5.216464 |
| RSPO3 | 4.92E-06 | 7.62E-08 | 5.455234 | 6.087796 |
| CELSR3 | 5.14E-06 | 8.00E-08 | 3.565191 | 4.966944 |
| AQP2 | 5.15E-06 | 8.05E-08 | 6.658709 | 3.820074 |
| LYPD3 | 5.15E-06 | 8.09E-08 | -5.82589 | 9.001609 |
| AGT | 5.78E-06 | 9.11E-08 | 5.336095 | 3.979424 |
| PERP | 5.93E-06 | 9.40E-08 | -3.21645 | 11.23675 |
| SYCN | 5.99E-06 | 9.53E-08 | -6.37364 | 1.897583 |
| CXCL5 | 6.14E-06 | 9.83E-08 | 7.262457 | 4.534835 |
| PRRG4 | 6.23E-06 | 1.00E-07 | -3.01264 | 7.307471 |
| DUOXA1 | 6.33E-06 | 1.02E-07 | -5.34238 | 2.459874 |
| CCL2 | 6.50E-06 | 1.05E-07 | 3.664805 | 3.833243 |
| JUP | 6.57E-06 | 1.07E-07 | -3.01457 | 4.316973 |
| SFN | 6.66E-06 | 1.09E-07 | -3.94631 | 9.570878 |
| TIMP1 | 6.89E-06 | 1.13E-07 | 3.460964 | 6.740786 |
| EMILIN2 | 6.93E-06 | 1.15E-07 | 2.464689 | 4.713718 |
| SFXN3 | 6.94E-06 | 1.15E-07 | 2.582854 | 5.418471 |
| KIAA1257 | 6.98E-06 | 1.16E-07 | 4.874701 | 2.220722 |
| JSRP1 | 7.17E-06 | 1.20E-07 | 6.118466 | 1.841402 |
| NUCB2 | 8.06E-06 | 1.36E-07 | -2.31864 | 8.343576 |
| BNIP3 | 8.06E-06 | 1.36E-07 | -2.20043 | 6.365925 |
| LYPD2 | 8.93E-06 | 1.52E-07 | -5.36337 | 4.736576 |
| ALDH3B2 | 8.93E-06 | 1.52E-07 | -5.40901 | 2.434667 |

| | | | | |
|---|---|---|---|---|
| RNF225 | 9.32E-06 | 1.60E-07 | -5.5077 | 2.731044 |
| ALDH9A1 | 9.47E-06 | 1.63E-07 | -2.27936 | 7.48127 |
| TXNDC17 | 9.96E-06 | 1.72E-07 | -2.79361 | 5.119658 |
| FSTL1 | 1.04E-05 | 1.81E-07 | 2.519065 | 7.707379 |
| FKBP10 | 1.05E-05 | 1.84E-07 | 4.227383 | 6.196809 |
| EPS8 | 1.05E-05 | 1.84E-07 | 2.372924 | 6.762561 |
| PARD6G-AS | 1.06E-05 | 1.87E-07 | -3.00199 | 3.68164 |
| GPR87 | 1.06E-05 | 1.87E-07 | -4.44263 | 5.206527 |
| LOC101928 | 1.06E-05 | 1.88E-07 | -2.77962 | 4.688966 |
| FAM110C | 1.07E-05 | 1.91E-07 | -2.43545 | 6.828061 |
| VIL1 | 1.13E-05 | 2.03E-07 | 6.441749 | 5.827841 |
| NCCRP1 | 1.13E-05 | 2.04E-07 | -6.63965 | 8.263891 |
| SCNN1G | 1.13E-05 | 2.05E-07 | -3.71555 | 5.707908 |
| ACPP | 1.14E-05 | 2.06E-07 | -4.45363 | 3.450637 |
| NOTCH3 | 1.17E-05 | 2.13E-07 | -2.05097 | 8.35512 |
| MAP1A | 1.17E-05 | 2.14E-07 | 2.769444 | 6.071408 |
| CASP1P2 | 1.25E-05 | 2.30E-07 | -4.78261 | 3.299472 |
| IPPK | 1.27E-05 | 2.34E-07 | -2.31454 | 6.306279 |
| RNF224 | 1.30E-05 | 2.40E-07 | -3.56796 | 3.227171 |
| SH3RF2 | 1.30E-05 | 2.42E-07 | -2.31664 | 6.608074 |
| ITGBL1 | 1.30E-05 | 2.42E-07 | 4.734298 | 4.083953 |
| PHLDB3 | 1.30E-05 | 2.44E-07 | -2.43205 | 6.233635 |
| RAB3D | 1.31E-05 | 2.46E-07 | -2.34054 | 7.558635 |
| KPRP | 1.31E-05 | 2.48E-07 | -4.76947 | 4.648735 |
| S100A9 | 1.42E-05 | 2.69E-07 | -5.71972 | 11.47318 |
| HOXA11 | 1.46E-05 | 2.78E-07 | 6.922751 | 1.950437 |
| ONECUT3 | 1.51E-05 | 2.90E-07 | 7.041786 | 3.157822 |
| FZD10 | 1.55E-05 | 2.99E-07 | -4.60429 | 5.588385 |
| ANKRD20A | 1.56E-05 | 3.00E-07 | -3.80362 | 4.825674 |
| LOC101928 | 1.61E-05 | 3.13E-07 | 8.697248 | 2.361986 |
| TMPRSS11 | 1.64E-05 | 3.20E-07 | -6.87756 | 1.772294 |
| RCN3 | 1.64E-05 | 3.20E-07 | 2.521968 | 4.73896 |
| ARL4D | 1.65E-05 | 3.23E-07 | -3.8525 | 5.308055 |
| HOXC-AS2 | 1.65E-05 | 3.25E-07 | 5.972836 | 1.994512 |
| NRTN | 1.70E-05 | 3.35E-07 | -2.94982 | 5.434943 |
| TGFBI | 1.73E-05 | 3.42E-07 | 2.693212 | 6.315902 |
| LINC00551 | 1.84E-05 | 3.67E-07 | -4.92514 | 3.464804 |
| AGR3 | 1.85E-05 | 3.69E-07 | 6.062261 | 2.831036 |
| IL12A-AS1 | 1.86E-05 | 3.74E-07 | -4.48699 | 2.791478 |
| PROM2 | 1.86E-05 | 3.74E-07 | -2.50429 | 5.225678 |
| LOC101927 | 2.03E-05 | 4.09E-07 | -3.43838 | 3.609899 |
| PPP1R13B | 2.09E-05 | 4.24E-07 | -1.97553 | 6.552952 |
| GSTA4 | 2.11E-05 | 4.30E-07 | -2.38684 | 5.577745 |
| TMPRSS11 | 2.11E-05 | 4.31E-07 | -7.38187 | 8.92046 |
| C5orf66-AS | 2.16E-05 | 4.42E-07 | -4.12666 | 4.195391 |

| | | | | |
|---|---|---|---|---|
| DOCK5 | 2.17E-05 | 4.48E-07 | 1.997976 | 7.686282 |
| RBM47 | 2.17E-05 | 4.49E-07 | -2.29166 | 5.819753 |
| ADGRL2 | 2.24E-05 | 4.64E-07 | -4.95771 | 2.170541 |
| PADI1 | 2.24E-05 | 4.66E-07 | -5.88462 | 9.015059 |
| HNF4A-AS1 | 2.28E-05 | 4.75E-07 | 5.92833 | 2.443972 |
| FNDC1 | 2.36E-05 | 4.96E-07 | 3.752905 | 5.336452 |
| PTN | 2.41E-05 | 5.08E-07 | -3.48492 | 6.036604 |
| WASF2 | 2.43E-05 | 5.14E-07 | -2.06219 | 5.652703 |
| TSPAN15 | 2.53E-05 | 5.35E-07 | 3.612073 | 5.510846 |
| SOD3 | 2.56E-05 | 5.45E-07 | 4.333026 | 6.312939 |
| TMPRSS11 | 2.56E-05 | 5.47E-07 | -5.17701 | 7.992875 |
| CASTOR1 | 2.57E-05 | 5.50E-07 | -3.21243 | 4.199273 |
| HSD17B11 | 2.63E-05 | 5.65E-07 | 2.971413 | 4.711624 |
| ARF6 | 2.97E-05 | 6.41E-07 | -2.14852 | 8.805329 |
| NTSR1 | 3.12E-05 | 6.76E-07 | 6.099602 | 2.872798 |
| PPP2R2C | 3.14E-05 | 6.83E-07 | -4.23448 | 2.794963 |
| BICD2 | 3.16E-05 | 6.89E-07 | -2.32821 | 5.119583 |
| KREMEN1 | 3.17E-05 | 6.92E-07 | -2.3956 | 6.353816 |
| KLRG2 | 3.17E-05 | 6.95E-07 | -8.26602 | 1.967026 |
| LINC01395 | 3.21E-05 | 7.06E-07 | -4.95292 | 2.025848 |
| TSPAN1 | 3.21E-05 | 7.09E-07 | 4.401996 | 5.596439 |
| HOXC11 | 3.33E-05 | 7.38E-07 | 8.111315 | 1.841629 |
| CST6 | 3.35E-05 | 7.46E-07 | -4.82638 | 2.043752 |
| TMC1 | 3.47E-05 | 7.75E-07 | -5.33331 | 2.160994 |
| SULT2B1 | 3.70E-05 | 8.30E-07 | -4.72013 | 3.510521 |
| S100A11 | 3.73E-05 | 8.39E-07 | -2.61949 | 9.670278 |
| C1R | 3.88E-05 | 8.74E-07 | 2.708465 | 7.946696 |
| ANKS4B | 3.97E-05 | 9.00E-07 | 5.284787 | 3.70743 |
| SLC6A19 | 3.97E-05 | 9.02E-07 | 7.749009 | 2.726152 |
| LNX1 | 4.41E-05 | 1.01E-06 | -3.87629 | 3.551215 |
| NAGK | 4.51E-05 | 1.03E-06 | -2.03075 | 6.813339 |
| SESTD1 | 4.63E-05 | 1.06E-06 | 2.239427 | 6.578256 |
| FAM83B | 4.63E-05 | 1.07E-06 | -2.39845 | 7.570702 |
| PGLYRP4 | 4.63E-05 | 1.07E-06 | -4.53357 | 3.987419 |
| CLDN3 | 4.67E-05 | 1.08E-06 | 4.878141 | 3.436912 |
| SHH | 4.72E-05 | 1.10E-06 | 6.407013 | 3.388505 |
| MREG | 4.77E-05 | 1.11E-06 | -3.52836 | 6.056713 |
| DEPP1 | 4.78E-05 | 1.12E-06 | 3.423408 | 6.845244 |
| IGSF9B | 4.88E-05 | 1.15E-06 | 3.717376 | 4.437316 |
| PAQR7 | 4.88E-05 | 1.15E-06 | -2.21988 | 5.196366 |
| COL10A1 | 4.91E-05 | 1.16E-06 | 3.004972 | 4.582053 |
| CRYZL2P | 4.92E-05 | 1.17E-06 | 2.421286 | 6.12018 |
| ARHGEF38 | 4.92E-05 | 1.17E-06 | 4.979014 | 4.534215 |
| CNFN | 5.06E-05 | 1.21E-06 | -6.22348 | 8.981676 |
| WDFY2 | 5.06E-05 | 1.21E-06 | -1.91071 | 6.229879 |

| | | | | |
|---|---|---|---|---|
| C9orf50 | 5.06E-05 | 1.21E-06 | 7.375294 | 1.198787 |
| FGF11 | 5.11E-05 | 1.23E-06 | -4.13976 | 3.726146 |
| LINC00887 | 5.22E-05 | 1.26E-06 | -4.09881 | 4.398529 |
| FGFR3 | 5.23E-05 | 1.27E-06 | -4.60781 | 3.30783 |
| LINC02210 | 5.23E-05 | 1.27E-06 | -3.56793 | 3.262069 |
| P3H3 | 5.23E-05 | 1.27E-06 | 2.445941 | 4.623399 |
| LINC01814 | 5.26E-05 | 1.29E-06 | 3.812687 | 3.424305 |
| SIAH2 | 5.29E-05 | 1.30E-06 | -1.88722 | 6.282927 |
| MUC13 | 5.31E-05 | 1.31E-06 | 6.492421 | 8.432139 |
| PIK3AP1 | 5.34E-05 | 1.32E-06 | 3.656361 | 4.512262 |
| KLK9 | 5.34E-05 | 1.32E-06 | -4.30877 | 2.635652 |
| KIAA1211 | 5.43E-05 | 1.35E-06 | 3.258234 | 4.863519 |
| LOC440434 | 5.55E-05 | 1.39E-06 | -1.83294 | 6.860959 |
| FAM129B | 5.55E-05 | 1.39E-06 | -2.76786 | 4.002884 |
| ARPC1B | 5.55E-05 | 1.39E-06 | 2.412673 | 6.24904 |
| FAM160A1 | 5.81E-05 | 1.46E-06 | -2.51534 | 6.619987 |
| AHNAK | 5.97E-05 | 1.50E-06 | -1.89797 | 13.51692 |
| SPINK5 | 5.98E-05 | 1.51E-06 | -4.78216 | 3.854937 |
| LPAR3 | 6.21E-05 | 1.58E-06 | -5.33391 | 3.76984 |
| DNAH6 | 6.42E-05 | 1.63E-06 | 4.308179 | 4.826997 |
| EPB41L4A | 6.51E-05 | 1.66E-06 | -2.02188 | 7.009795 |
| SLC16A9 | 6.55E-05 | 1.68E-06 | -3.25547 | 5.216021 |
| RSAD2 | 6.55E-05 | 1.69E-06 | 3.864088 | 4.392831 |
| EMP2 | 6.55E-05 | 1.69E-06 | -2.05539 | 8.248452 |
| AXIN2 | 6.57E-05 | 1.70E-06 | 4.581589 | 7.366324 |
| SULT1C2 | 6.60E-05 | 1.71E-06 | 6.237873 | 2.403111 |
| NIPAL2 | 6.71E-05 | 1.74E-06 | -2.21337 | 6.161968 |
| DUOX1 | 6.74E-05 | 1.76E-06 | -4.09281 | 3.166452 |
| LOC729737 | 6.87E-05 | 1.80E-06 | -3.19051 | 5.158072 |
| FAM20C | 6.94E-05 | 1.82E-06 | 2.384615 | 5.648194 |
| PDZD2 | 6.95E-05 | 1.83E-06 | -2.15732 | 6.693183 |
| OSTF1 | 7.14E-05 | 1.88E-06 | -2.18525 | 6.553321 |
| MIR210HG | 7.22E-05 | 1.91E-06 | -3.38782 | 5.013391 |
| PMM1 | 7.34E-05 | 1.95E-06 | -2.30784 | 5.651284 |
| TMEM63C | 7.46E-05 | 1.99E-06 | -3.07062 | 4.009864 |
| HKDC1 | 7.49E-05 | 2.00E-06 | 5.08267 | 5.744976 |
| SLC9A3-AS | 7.51E-05 | 2.01E-06 | -2.16882 | 5.328952 |
| COL6A3 | 7.53E-05 | 2.02E-06 | 2.597791 | 3.651262 |
| HOXB8 | 7.53E-05 | 2.03E-06 | 7.209658 | 3.474635 |
| MAP3K20 | 7.84E-05 | 2.11E-06 | 2.132537 | 6.914758 |
| DEGS2 | 8.21E-05 | 2.22E-06 | -3.342 | 4.267058 |
| ERMP1 | 8.23E-05 | 2.23E-06 | -1.8789 | 7.114085 |
| BICDL2 | 8.37E-05 | 2.28E-06 | -2.14055 | 6.940351 |
| SEPT5-GP1 | 8.37E-05 | 2.29E-06 | -3.87446 | 3.115811 |
| RASA3 | 8.37E-05 | 2.29E-06 | 2.978347 | 5.700698 |

| | | | | |
|---|---|---|---|---|
| ALDH1B1 | 8.71E-05 | 2.39E-06 | 2.749194 | 4.957548 |
| PAK6 | 8.91E-05 | 2.45E-06 | -5.12368 | 1.97914 |
| ICAM1 | 8.99E-05 | 2.48E-06 | 2.996389 | 5.570492 |
| IFI30 | 9.09E-05 | 2.51E-06 | 2.433381 | 6.14326 |
| FGF2 | 9.34E-05 | 2.59E-06 | 2.810057 | 4.837129 |
| HOXC8 | 9.37E-05 | 2.60E-06 | 4.225769 | 2.871009 |
| LOC283045 | 9.50E-05 | 2.65E-06 | -5.27385 | 3.428956 |
| FRMD6-AS1 | 9.71E-05 | 2.71E-06 | -3.844 | 3.259247 |
| STOX2 | 9.80E-05 | 2.75E-06 | -2.46817 | 4.584811 |
| GALNT6 | 9.95E-05 | 2.80E-06 | 2.396744 | 5.65544 |
| TM4SF20 | 0.000103 | 2.91E-06 | 8.615397 | 5.887321 |
| ARL14 | 0.000108 | 3.06E-06 | 3.683629 | 3.065532 |
| RGS12 | 0.000113 | 3.19E-06 | -2.46201 | 4.680975 |
| TYRO3 | 0.000115 | 3.26E-06 | -2.01487 | 4.972982 |
| CCDC158 | 0.000117 | 3.34E-06 | 4.731468 | 2.258386 |
| IGFN1 | 0.000118 | 3.36E-06 | 4.964839 | 5.977186 |
| PCP4L1 | 0.000119 | 3.40E-06 | -3.79314 | 3.681551 |
| ETV5 | 0.000121 | 3.49E-06 | 2.517914 | 4.916427 |
| RANBP9 | 0.000122 | 3.52E-06 | -1.97178 | 8.052206 |
| UGT1A7 | 0.000124 | 3.59E-06 | -5.17083 | 4.164126 |
| SPAG17 | 0.000124 | 3.59E-06 | -3.3466 | 5.425619 |
| VPS4B | 0.000124 | 3.59E-06 | -2.08635 | 7.997503 |
| SYNGR1 | 0.000124 | 3.63E-06 | -2.61012 | 4.785949 |
| RFTN1 | 0.000124 | 3.63E-06 | 2.055266 | 5.701063 |
| RAB38 | 0.000128 | 3.75E-06 | -3.62935 | 4.877414 |
| HOXC9 | 0.000134 | 3.95E-06 | 5.353683 | 2.895585 |
| FGFBP1 | 0.000134 | 3.95E-06 | -4.73513 | 6.66891 |
| LGI3 | 0.000134 | 3.95E-06 | -3.9746 | 2.539662 |
| PAIP2B | 0.000137 | 4.05E-06 | -2.77145 | 5.917586 |
| TEC | 0.000148 | 4.39E-06 | -1.79666 | 5.752354 |
| SLC6A11 | 0.000149 | 4.43E-06 | -3.2901 | 4.167689 |
| EPHA10 | 0.000158 | 4.70E-06 | 3.109811 | 3.614497 |
| AGBL4 | 0.000158 | 4.73E-06 | 7.179073 | 1.029439 |
| IQGAP3 | 0.000158 | 4.74E-06 | 1.93369 | 6.125815 |
| SPTBN1 | 0.000159 | 4.78E-06 | 1.695424 | 7.700173 |
| ITGB3 | 0.00016 | 4.81E-06 | 2.782076 | 4.639956 |
| TFAP2C | 0.00016 | 4.84E-06 | -3.32796 | 5.471797 |
| BDKRB2 | 0.00016 | 4.85E-06 | -2.2653 | 5.984758 |
| ZNF770 | 0.00016 | 4.85E-06 | -1.87903 | 7.650169 |
| THBS1 | 0.000161 | 4.89E-06 | 3.310775 | 9.834996 |
| TCP11L2 | 0.000165 | 5.04E-06 | -2.08027 | 5.758711 |
| HOXA11-AS | 0.000165 | 5.04E-06 | 7.732465 | 1.511491 |
| EPS8L2 | 0.000172 | 5.28E-06 | -2.04651 | 8.831724 |
| SULT1C3 | 0.000175 | 5.37E-06 | 5.624537 | 3.0357 |
| ZNF703 | 0.000178 | 5.49E-06 | 2.446853 | 6.025833 |

| | | | | |
|---|---|---|---|---|
| ANKRD31 | 0.000179 | 5.52E-06 | -3.08029 | 3.863967 |
| CREB3L1 | 0.000186 | 5.76E-06 | 4.383361 | 7.926067 |
| LY6G6C | 0.000186 | 5.77E-06 | -5.82857 | 1.360563 |
| DUOXA2 | 0.000187 | 5.82E-06 | -3.65501 | 3.909015 |
| MIR4697H( | 0.000201 | 6.27E-06 | 2.934609 | 4.945543 |
| NDRG1 | 0.000203 | 6.33E-06 | -3.88875 | 6.610439 |
| CTSK | 0.000207 | 6.48E-06 | 2.327867 | 5.519323 |
| ANKRD20A | 0.000209 | 6.56E-06 | -2.17887 | 5.673256 |
| ANXA4 | 0.000212 | 6.67E-06 | 1.727868 | 6.803721 |
| TGM1 | 0.000212 | 6.68E-06 | -5.74393 | 9.95136 |
| ANKFN1 | 0.000218 | 6.89E-06 | 5.041262 | 2.111146 |
| ITPRIP | 0.000219 | 6.93E-06 | -2.30018 | 4.409512 |
| HSPB6 | 0.000231 | 7.34E-06 | 3.082264 | 5.867519 |
| SEMA3F | 0.000231 | 7.35E-06 | -2.62049 | 6.947786 |
| FAR2P1 | 0.000235 | 7.49E-06 | 8.859245 | 2.529038 |
| TGM2 | 0.000236 | 7.55E-06 | 3.559451 | 6.214759 |
| POLR2J4 | 0.000237 | 7.60E-06 | -1.76791 | 6.289532 |
| LOC254896 | 0.000248 | 7.97E-06 | 4.99833 | 2.066546 |
| UQCRQ | 0.000252 | 8.12E-06 | -1.78582 | 5.87706 |
| ZDHHC21 | 0.000255 | 8.25E-06 | -1.87095 | 7.404756 |
| LINC02584 | 0.000256 | 8.30E-06 | 6.274671 | 2.405028 |
| PDGFRB | 0.000262 | 8.51E-06 | 2.030095 | 7.138973 |
| S100A8 | 0.000262 | 8.54E-06 | -5.81638 | 11.6256 |
| TMEM63A | 0.000263 | 8.59E-06 | 2.015476 | 7.426416 |
| KAT2B | 0.000264 | 8.62E-06 | -1.8571 | 6.76654 |
| BGN | 0.000274 | 8.97E-06 | 2.392772 | 6.995432 |
| USP6NL | 0.000274 | 8.99E-06 | -2.36414 | 4.352876 |
| MTCL1 | 0.000278 | 9.16E-06 | 2.3978 | 6.486009 |
| CARD14 | 0.000287 | 9.47E-06 | -5.67746 | 1.274337 |
| TSPAN8 | 0.00029 | 9.60E-06 | 5.655193 | 7.517712 |
| PTPRZ1 | 0.000296 | 9.80E-06 | -3.1922 | 3.720186 |
| RBP7 | 0.000304 | 1.01E-05 | -3.10011 | 3.121684 |
| CAMSAP1 | 0.000311 | 1.04E-05 | -1.65608 | 7.495102 |
| PKP3 | 0.000328 | 1.09E-05 | -4.48195 | 1.400909 |
| IFIT2 | 0.000339 | 1.13E-05 | 4.120395 | 4.768573 |
| C3orf80 | 0.000352 | 1.18E-05 | -3.55479 | 3.353697 |
| TTYH3 | 0.000352 | 1.18E-05 | 2.083038 | 6.630615 |
| SERPINA3 | 0.000353 | 1.19E-05 | 4.402744 | 4.945065 |
| RELCH | 0.000355 | 1.20E-05 | -1.82928 | 7.908731 |
| FSTL3 | 0.000358 | 1.21E-05 | 2.741758 | 4.850101 |
| TCAF2P1 | 0.00036 | 1.22E-05 | -3.68016 | 2.582451 |
| FUT3 | 0.00036 | 1.23E-05 | -3.44837 | 2.960863 |
| KLHL30 | 0.00036 | 1.23E-05 | 5.505748 | 1.308516 |
| NKD1 | 0.000361 | 1.23E-05 | 5.316346 | 8.354513 |
| EPN2-AS1 | 0.000361 | 1.23E-05 | -2.31048 | 4.263324 |

| | | | | |
|---|---|---|---|---|
| KLK13 | 0.000363 | 1.24E-05 | -5.67637 | 9.872951 |
| LY6D | 0.000364 | 1.25E-05 | -6.29686 | 8.227792 |
| CFTR | 0.000365 | 1.26E-05 | 3.747831 | 4.337071 |
| PPP1R1B | 0.000372 | 1.28E-05 | 6.0346 | 2.938355 |
| COL4A1 | 0.000375 | 1.30E-05 | 2.030833 | 8.360435 |
| ME1 | 0.000391 | 1.36E-05 | -2.29795 | 7.051011 |
| MSX2 | 0.0004 | 1.39E-05 | 4.731031 | 3.411762 |
| CFB | 0.0004 | 1.39E-05 | 3.560192 | 3.380117 |
| COL4A2 | 0.0004 | 1.40E-05 | 2.105629 | 8.630873 |
| IFI44L | 0.000402 | 1.41E-05 | 4.482823 | 6.423097 |
| SP100 | 0.000405 | 1.42E-05 | 1.951251 | 5.860798 |
| LINC01002 | 0.000412 | 1.45E-05 | -2.75941 | 3.730073 |
| ALPP | 0.000413 | 1.46E-05 | 7.708599 | 1.475599 |
| OVOL1-AS1 | 0.000415 | 1.47E-05 | -5.01865 | 3.189805 |
| ATP11B | 0.000415 | 1.47E-05 | -1.67832 | 8.72297 |
| GPT2 | 0.000415 | 1.47E-05 | -3.86266 | 3.371859 |
| FRMD4B | 0.000417 | 1.48E-05 | -1.75088 | 7.368616 |
| CCDC85C | 0.000427 | 1.52E-05 | -1.75304 | 6.17743 |
| SBSN | 0.000427 | 1.52E-05 | -6.68019 | 9.251573 |
| DENND2C | 0.000429 | 1.53E-05 | -4.22525 | 3.03993 |
| METTL7B | 0.000429 | 1.54E-05 | 4.685609 | 2.001119 |
| SIGLEC9 | 0.000429 | 1.54E-05 | 5.752445 | 0.945728 |
| PROX1-AS1 | 0.00043 | 1.55E-05 | 7.157295 | 2.081486 |
| LDLRAD4-A | 0.000442 | 1.59E-05 | 3.970215 | 2.914953 |
| GRIN2D | 0.000446 | 1.61E-05 | 3.096924 | 4.639477 |
| KIAA1755 | 0.000448 | 1.62E-05 | 3.117921 | 3.726444 |
| TRAM2 | 0.000454 | 1.65E-05 | 1.852718 | 6.052982 |
| VDAC2 | 0.000458 | 1.67E-05 | -3.39379 | 3.6939 |
| DRAM1 | 0.000463 | 1.68E-05 | 2.360204 | 4.657132 |
| GADL1 | 0.000476 | 1.74E-05 | 5.915207 | 2.688245 |
| TOLLIP | 0.000478 | 1.75E-05 | -1.57941 | 6.129968 |
| KRT6A | 0.000478 | 1.75E-05 | -5.6563 | 13.03551 |
| SVEP1 | 0.000479 | 1.76E-05 | 2.633504 | 6.162994 |
| TMOD3 | 0.000483 | 1.78E-05 | -1.81548 | 8.891351 |
| ZNF365 | 0.000493 | 1.82E-05 | -3.65237 | 4.784054 |
| NNMT | 0.000497 | 1.84E-05 | 3.491458 | 5.713427 |
| MSR1 | 0.000499 | 1.85E-05 | 3.647416 | 3.364075 |
| OGFRL1 | 0.000512 | 1.90E-05 | -1.71077 | 6.362608 |
| AQP3 | 0.000515 | 1.92E-05 | -3.85807 | 10.11864 |
| GJB5 | 0.000517 | 1.93E-05 | -3.63518 | 4.448102 |
| ZNFX1 | 0.000521 | 1.95E-05 | 1.579333 | 7.652069 |
| FBN1 | 0.000521 | 1.95E-05 | 2.287884 | 8.130001 |
| CRCT1 | 0.000525 | 1.97E-05 | -5.8825 | 7.448524 |
| TSPAN5 | 0.000526 | 1.98E-05 | -2.23973 | 5.08185 |
| SLPI | 0.000531 | 2.00E-05 | -2.99435 | 8.585715 |

| | | | | |
|---|---|---|---|---|
| KRT10 | 0.000532 | 2.01E-05 | -2.90584 | 6.678298 |
| UBXN10 | 0.000535 | 2.03E-05 | 3.106227 | 3.402329 |
| IL1RL2 | 0.000535 | 2.03E-05 | -2.5326 | 4.179849 |
| DLGAP1-AS | 0.000547 | 2.08E-05 | 3.002408 | 3.409515 |
| MSMO1 | 0.00055 | 2.09E-05 | -3.87201 | 2.943917 |
| DYNLT3 | 0.00055 | 2.10E-05 | -1.95942 | 6.90336 |
| BICC1 | 0.000557 | 2.13E-05 | 3.141241 | 5.019825 |
| LINC01594 | 0.000559 | 2.14E-05 | 5.738749 | 2.28377 |
| FAM102B | 0.000561 | 2.15E-05 | 1.746819 | 5.494517 |
| MCAM | 0.000567 | 2.18E-05 | 2.254968 | 6.556804 |
| VIM | 0.000567 | 2.18E-05 | 1.934351 | 7.984275 |
| ENDOU | 0.000568 | 2.19E-05 | -5.76504 | 1.746593 |
| RIPK4 | 0.000571 | 2.20E-05 | -3.18886 | 7.408332 |
| ONECUT2 | 0.000574 | 2.22E-05 | 4.876909 | 4.714346 |
| LGALSL | 0.000579 | 2.25E-05 | -1.73555 | 6.031845 |
| MICALL1 | 0.00058 | 2.25E-05 | -1.65477 | 7.349578 |
| ENTPD5 | 0.000582 | 2.27E-05 | -1.90773 | 4.807694 |
| PGM2L1 | 0.000582 | 2.27E-05 | 2.77276 | 5.66196 |
| SRY | 0.000585 | 2.29E-05 | -7.26216 | 1.095615 |
| EMB | 0.00059 | 2.31E-05 | 1.794731 | 5.611818 |
| IL2RG | 0.000596 | 2.34E-05 | 2.557145 | 4.342483 |
| NOTUM | 0.000605 | 2.38E-05 | 9.079382 | 6.471071 |
| UBE2Q2P2 | 0.000615 | 2.42E-05 | -2.1248 | 6.029407 |
| CMAS | 0.000617 | 2.43E-05 | -1.89875 | 6.140883 |
| AADACP1 | 0.000626 | 2.48E-05 | 6.484788 | 2.093743 |
| COL12A1 | 0.000639 | 2.53E-05 | 2.865231 | 7.351424 |
| CWH43 | 0.000644 | 2.56E-05 | -4.94252 | 1.576902 |
| SPATA13-A | 0.00065 | 2.58E-05 | 4.189721 | 2.291411 |
| DUSP7 | 0.000653 | 2.60E-05 | -2.32823 | 6.612582 |
| MUC3A | 0.000653 | 2.61E-05 | 4.525518 | 7.770914 |
| TMPRSS11 | 0.000654 | 2.62E-05 | -5.76273 | 6.592469 |
| LOC72855 | 0.000671 | 2.69E-05 | -2.24296 | 6.132412 |
| SERPINF1 | 0.000672 | 2.70E-05 | 2.264742 | 5.760746 |
| DHRS1 | 0.000684 | 2.75E-05 | -3.41636 | 2.31682 |
| GFPT2 | 0.000689 | 2.78E-05 | 2.526545 | 4.369307 |
| HTR1D | 0.000702 | 2.84E-05 | 7.730043 | 1.482846 |
| CYBB | 0.000718 | 2.90E-05 | 2.417665 | 5.111405 |
| TLN2 | 0.000729 | 2.96E-05 | 2.696814 | 6.332178 |
| RDH12 | 0.00074 | 3.00E-05 | -3.71246 | 3.597558 |
| GFOD2 | 0.00074 | 3.01E-05 | -1.99878 | 5.943616 |
| ZIC5 | 0.000756 | 3.08E-05 | 7.958711 | 1.709671 |
| POM121L9 | 0.000768 | 3.14E-05 | 4.524626 | 2.422295 |
| TIMP2 | 0.000788 | 3.22E-05 | 2.154206 | 7.924063 |
| ARHGAP32 | 0.000806 | 3.31E-05 | -1.59677 | 5.607168 |
| TFPI | 0.000829 | 3.41E-05 | 2.169035 | 4.663572 |

| | | | | |
|---|---|---|---|---|
| MIR600HG | 0.000829 | 3.41E-05 | -1.66268 | 5.586793 |
| PROCR | 0.000847 | 3.49E-05 | 2.307432 | 3.852821 |
| ISG15 | 0.000856 | 3.54E-05 | 3.233322 | 3.683828 |
| BIRC3 | 0.000856 | 3.54E-05 | 3.03492 | 5.977913 |
| CPNE3 | 0.000875 | 3.63E-05 | -1.68847 | 7.981712 |
| PLXNC1 | 0.000882 | 3.67E-05 | 2.746152 | 4.48359 |
| IL1RN | 0.000886 | 3.69E-05 | -4.17951 | 6.93197 |
| ACAP3 | 0.000894 | 3.73E-05 | -1.65793 | 6.575429 |
| FBLN5 | 0.000894 | 3.74E-05 | 2.159266 | 5.248068 |
| DNAH5 | 0.000898 | 3.76E-05 | 3.744585 | 5.017032 |
| KRT32 | 0.000898 | 3.76E-05 | -5.90642 | 1.567274 |
| KCNE3 | 0.000912 | 3.83E-05 | 3.542703 | 5.719696 |
| IRF6 | 0.000919 | 3.87E-05 | -3.49186 | 2.899977 |
| ZBTB7C | 0.000948 | 4.00E-05 | -2.57913 | 6.861712 |
| PARM1 | 0.000948 | 4.00E-05 | 2.469388 | 5.406651 |
| MAL2 | 0.000963 | 4.07E-05 | -2.10235 | 9.151725 |
| TIMP3 | 0.000967 | 4.10E-05 | 1.846785 | 7.998056 |
| GRN | 0.000967 | 4.10E-05 | -1.5923 | 8.307321 |
| ALPK3 | 0.000979 | 4.16E-05 | 1.750339 | 5.50696 |
| JCAD | 0.000981 | 4.19E-05 | 1.795743 | 5.901324 |
| DST | 0.000981 | 4.19E-05 | -1.90937 | 8.197738 |
| OVOL1 | 0.000983 | 4.20E-05 | -3.92178 | 6.048346 |
| UBE2G1 | 0.000991 | 4.25E-05 | -1.79813 | 6.721754 |
| LOC10099( | 0.000992 | 4.26E-05 | -3.50889 | 3.185319 |
| CD72 | 0.000992 | 4.26E-05 | 3.074772 | 2.618168 |
| SQLE | 0.001021 | 4.40E-05 | -2.21923 | 7.080353 |
| RALA | 0.001023 | 4.41E-05 | -1.63316 | 7.711486 |
| ARID5A | 0.001034 | 4.47E-05 | 2.317005 | 4.425246 |
| VWA3A | 0.001051 | 4.55E-05 | 5.511172 | 2.915393 |
| KRT2 | 0.001054 | 4.57E-05 | -6.12489 | 2.327859 |
| CCDC80 | 0.001058 | 4.60E-05 | 2.486092 | 4.851403 |
| SLC19A3 | 0.001059 | 4.61E-05 | 5.065436 | 3.114459 |
| PI3 | 0.001059 | 4.62E-05 | -4.67546 | 9.626462 |
| CHMP1B2F | 0.001066 | 4.66E-05 | 4.05011 | 3.741101 |
| FAM25BP | 0.001066 | 4.66E-05 | -4.57132 | 2.013395 |
| SLCO1B3 | 0.001079 | 4.73E-05 | 5.932239 | 3.431752 |
| RPS6KB2 | 0.001084 | 4.76E-05 | -1.56342 | 6.385733 |
| CEMIP2 | 0.001088 | 4.79E-05 | 3.581575 | 2.741956 |
| ALKAL1 | 0.001088 | 4.79E-05 | 6.904995 | 0.806466 |
| STRN | 0.001091 | 4.81E-05 | -1.67352 | 8.595313 |
| ATP6V1D | 0.001105 | 4.88E-05 | -1.73238 | 6.338443 |
| SPEG | 0.001106 | 4.90E-05 | 2.96327 | 5.644273 |
| ZNF275 | 0.001108 | 4.92E-05 | 2.262604 | 4.750519 |
| MAP1B | 0.001131 | 5.03E-05 | 2.257362 | 8.061055 |
| HES2 | 0.001137 | 5.06E-05 | -2.84308 | 5.498136 |

| | | | | |
|---|---|---|---|---|
| FGF3 | 0.001147 | 5.12E-05 | 9.430342 | 3.071113 |
| SYT11 | 0.001147 | 5.12E-05 | 2.868573 | 4.545933 |
| EEA1 | 0.001147 | 5.13E-05 | -1.58748 | 8.462942 |
| STMP1 | 0.001148 | 5.15E-05 | -1.8222 | 6.333184 |
| VAMP8 | 0.001148 | 5.16E-05 | -1.4794 | 6.459005 |
| MIR6510 | 0.001164 | 5.24E-05 | -4.72801 | 2.085014 |
| KRTAP5-AS | 0.001167 | 5.26E-05 | 7.099328 | 0.970366 |
| FBXO41 | 0.001168 | 5.27E-05 | 1.778501 | 4.703407 |
| MBD2 | 0.001188 | 5.37E-05 | -1.61267 | 8.073735 |
| DCDC1 | 0.001188 | 5.38E-05 | 8.156887 | 1.883306 |
| PGGHG | 0.001189 | 5.39E-05 | 2.930553 | 6.17924 |
| ZNF395 | 0.001193 | 5.42E-05 | -1.63301 | 7.529645 |
| HERC2P4 | 0.001216 | 5.53E-05 | -2.35996 | 4.757782 |
| SERPINB3 | 0.001223 | 5.57E-05 | -6.68698 | 10.34394 |
| RCOR1 | 0.001225 | 5.59E-05 | -2.03145 | 7.699107 |
| BAIAP2L2 | 0.001238 | 5.66E-05 | 2.268088 | 5.180233 |
| PTGIS | 0.001249 | 5.72E-05 | 3.310588 | 5.062069 |
| SRMS | 0.001253 | 5.75E-05 | 3.309494 | 3.692507 |
| LRRFIP2 | 0.001257 | 5.78E-05 | -1.7328 | 5.207004 |
| SPTBN2 | 0.001257 | 5.78E-05 | -2.68208 | 8.490927 |
| FENDRR | 0.001257 | 5.80E-05 | 2.578625 | 5.029379 |
| ECM1 | 0.001259 | 5.82E-05 | -5.61325 | 0.711236 |
| TAB3 | 0.001284 | 5.94E-05 | -1.56445 | 7.051792 |
| SCGB1A1 | 0.001288 | 5.97E-05 | -4.86319 | 2.465233 |
| ERG28 | 0.001293 | 6.00E-05 | -1.71681 | 5.126384 |
| ASCL2 | 0.0013 | 6.05E-05 | 4.695431 | 3.96174 |
| SERPINB1 | 0.0013 | 6.05E-05 | -2.93949 | 4.641891 |
| AHNAK2 | 0.001307 | 6.09E-05 | -1.91653 | 10.32513 |
| SCEL-AS1 | 0.001345 | 6.29E-05 | -5.29122 | 1.63633 |
| BRCA2 | 0.001345 | 6.29E-05 | 1.673583 | 5.51428 |
| HSD17B14 | 0.001348 | 6.32E-05 | 3.497797 | 2.828005 |
| PIWIL4 | 0.00135 | 6.34E-05 | 3.401154 | 3.742258 |
| TMEM163 | 0.001353 | 6.36E-05 | 4.950993 | 2.94839 |
| ITGA1 | 0.001363 | 6.42E-05 | 2.506173 | 6.548598 |
| BAG2 | 0.001384 | 6.53E-05 | 2.791237 | 3.717712 |
| COL5A2 | 0.001404 | 6.63E-05 | 1.95419 | 8.084233 |
| GDPD5 | 0.001414 | 6.69E-05 | 3.464599 | 3.285684 |
| LCP2 | 0.001433 | 6.80E-05 | 2.003496 | 5.143705 |
| SLC7A1 | 0.001433 | 6.80E-05 | -1.65825 | 8.154043 |
| PRPH | 0.001459 | 6.94E-05 | 6.802261 | 0.733177 |
| ST6GALNA( | 0.001481 | 7.05E-05 | 4.491063 | 2.504415 |
| FGL2 | 0.001508 | 7.19E-05 | 2.475008 | 7.107691 |
| DCHS1 | 0.00153 | 7.31E-05 | 1.998466 | 5.450938 |
| JAK3 | 0.001559 | 7.46E-05 | 2.212599 | 4.631076 |
| ESPL1 | 0.001585 | 7.60E-05 | -2.89293 | 6.149882 |

| | | | | |
|---|---|---|---|---|
| TALDO1 | 0.001603 | 7.70E-05 | -1.58047 | 7.379279 |
| NPR2 | 0.001612 | 7.76E-05 | 3.254547 | 4.108745 |
| C12orf29 | 0.001612 | 7.77E-05 | -2.16817 | 4.600015 |
| CBR3 | 0.001621 | 7.82E-05 | -2.69102 | 4.295215 |
| COL16A1 | 0.001631 | 7.88E-05 | 1.768313 | 7.038903 |
| RNF11 | 0.001638 | 7.93E-05 | -1.58521 | 7.202827 |
| ANKRD36B | 0.001641 | 7.95E-05 | 1.829677 | 6.528724 |
| ANTXR1 | 0.001653 | 8.03E-05 | 2.216921 | 7.438137 |
| LINC01356 | 0.001666 | 8.10E-05 | 5.42268 | 1.24535 |
| C20orf203 | 0.001713 | 8.34E-05 | 5.134157 | 0.960346 |
| LEFTY1 | 0.001727 | 8.43E-05 | 4.944538 | 3.026369 |
| CDS1 | 0.001727 | 8.45E-05 | -1.61647 | 6.158243 |
| PCP4 | 0.001727 | 8.45E-05 | 6.305277 | 4.120163 |
| SH3BP1 | 0.00173 | 8.48E-05 | -2.00973 | 4.734466 |
| RAET1L | 0.001738 | 8.53E-05 | -3.61506 | 4.117008 |
| KLF2 | 0.001741 | 8.56E-05 | 2.34405 | 5.136149 |
| SPTLC2 | 0.001746 | 8.59E-05 | -1.56789 | 7.871748 |
| PLXND1 | 0.001762 | 8.69E-05 | 1.703962 | 6.355097 |
| MIR4279 | 0.001773 | 8.75E-05 | -4.18594 | 2.137009 |
| MRC1 | 0.001782 | 8.81E-05 | 2.715841 | 6.115052 |
| NEAT1 | 0.001802 | 8.93E-05 | -1.87985 | 14.62551 |
| TRIOBP | 0.001803 | 8.95E-05 | -2.41847 | 5.870655 |
| COL4A4 | 0.001803 | 8.96E-05 | 3.270678 | 5.07644 |
| GCNT3 | 0.001829 | 9.10E-05 | 3.326565 | 6.219657 |
| CACNA2D3 | 0.001849 | 9.22E-05 | -2.95526 | 3.712831 |
| POTEF | 0.001891 | 9.45E-05 | 6.732558 | 3.18417 |
| NOS2 | 0.001891 | 9.45E-05 | 5.361413 | 2.747159 |
| NCKAP5 | 0.001893 | 9.48E-05 | -2.25267 | 4.672555 |
| ANXA8L1 | 0.001905 | 9.55E-05 | -7.09626 | 0.962115 |
| TCIM | 0.001914 | 9.61E-05 | 3.74102 | 6.795522 |
| DACT3 | 0.001921 | 9.66E-05 | 6.753719 | 0.679105 |
| CEP290 | 0.001921 | 9.68E-05 | 1.739155 | 6.07293 |
| MEGF9 | 0.001923 | 9.70E-05 | -1.81523 | 6.537631 |
| DLEU7 | 0.001942 | 9.81E-05 | 3.568325 | 3.072649 |
| DSG3 | 0.001963 | 9.93E-05 | -4.87959 | 11.22723 |
| ARHGEF37 | 0.001964 | 9.96E-05 | -1.80773 | 6.148599 |
| TWIST1 | 0.001971 | 0.0001 | 3.163371 | 3.907641 |
| TFF3 | 0.001982 | 0.000101 | 4.734646 | 3.231761 |
| COL15A1 | 0.002001 | 0.000102 | 1.910941 | 6.827397 |
| SNX31 | 0.002001 | 0.000102 | -3.80675 | 3.466243 |
| LOC64333[illegible] | 0.002017 | 0.000103 | 6.998726 | 0.882545 |
| SPINK7 | 0.002034 | 0.000104 | -4.74854 | 5.502899 |
| HSPA1A | 0.002038 | 0.000104 | -4.34522 | 5.82335 |
| P3H4 | 0.002076 | 0.000106 | 2.593471 | 3.624307 |
| PRPSAP1 | 0.002096 | 0.000108 | -1.41207 | 5.932245 |

| | | | | |
|---|---|---|---|---|
| AGFG2 | 0.002127 | 0.000109 | -1.69966 | 5.891082 |
| EXOC6B | 0.00213 | 0.00011 | -1.5217 | 7.331743 |
| VGLL3 | 0.00213 | 0.00011 | 2.186417 | 4.548105 |
| TMEM263 | 0.002149 | 0.000111 | 1.752717 | 5.373951 |
| DPYSL2 | 0.002166 | 0.000112 | 2.518229 | 4.208851 |
| SLC40A1 | 0.002166 | 0.000112 | 2.004624 | 6.323819 |
| GOLGA6L1 | 0.002186 | 0.000113 | -2.1577 | 4.999862 |
| DUSP5 | 0.002201 | 0.000114 | -2.83838 | 7.683993 |
| THEMIS2 | 0.002225 | 0.000116 | 6.736533 | 0.692649 |
| AGR2 | 0.002225 | 0.000116 | 4.953173 | 9.365818 |
| S100A2 | 0.002227 | 0.000116 | -4.43518 | 8.893481 |
| FAM157A | 0.002259 | 0.000118 | -2.71618 | 4.010148 |
| ARHGAP42 | 0.002268 | 0.000119 | 2.454514 | 5.467875 |
| NID2 | 0.002273 | 0.000119 | 1.987656 | 5.110748 |
| MMP9 | 0.002383 | 0.000125 | 4.237751 | 3.003754 |
| BPIFB1 | 0.002427 | 0.000128 | 6.260648 | 3.044274 |
| PRDM1 | 0.002439 | 0.000129 | -1.99661 | 6.308495 |
| CAMK1D | 0.002439 | 0.000129 | -2.08781 | 5.189269 |
| SI | 0.002439 | 0.000129 | 7.817393 | 2.770708 |
| C1QB | 0.002443 | 0.000129 | 2.387066 | 5.736203 |
| ITGA5 | 0.002453 | 0.00013 | 2.155346 | 6.894402 |
| EBP | 0.002475 | 0.000131 | -1.80171 | 5.467617 |
| SERTAD2 | 0.002475 | 0.000132 | -1.89282 | 7.549874 |
| TENT5A | 0.002475 | 0.000132 | 1.908188 | 6.256653 |
| KLK7 | 0.002484 | 0.000132 | -4.88842 | 1.078586 |
| BHLHE41 | 0.002513 | 0.000134 | 2.475226 | 5.08967 |
| CDKN2B | 0.00252 | 0.000135 | -4.23515 | 2.566829 |
| PCK1 | 0.00252 | 0.000135 | 3.441964 | 3.163152 |
| GLIS2 | 0.00252 | 0.000135 | 3.020475 | 2.677198 |
| CYP2J2 | 0.002535 | 0.000136 | -2.42623 | 4.377722 |
| GPR78 | 0.002586 | 0.000139 | -4.72098 | 1.000334 |
| CYP4Z2P | 0.002586 | 0.000139 | -3.73584 | 3.412135 |
| ITPKC | 0.002586 | 0.000139 | -1.74026 | 7.522534 |
| PKDCC | 0.002593 | 0.00014 | 4.686764 | 4.732729 |
| C1QA | 0.002593 | 0.00014 | 2.20209 | 4.922578 |
| CLIP1 | 0.002594 | 0.00014 | -4.34175 | 1.707967 |
| CDR2L | 0.002616 | 0.000142 | 1.872877 | 5.396907 |
| CLCA2 | 0.002631 | 0.000143 | -5.81087 | 9.03611 |
| FAM219B | 0.002634 | 0.000143 | -1.66427 | 6.725189 |
| ANKRD35 | 0.002649 | 0.000144 | -4.12395 | 1.876603 |
| DUSP27 | 0.002649 | 0.000145 | 6.671876 | 5.489598 |
| FADS1 | 0.002649 | 0.000145 | 1.802515 | 4.931192 |
| EPN3 | 0.002717 | 0.000148 | -2.18304 | 5.967635 |
| TCAF2 | 0.002723 | 0.000149 | -2.28206 | 4.353977 |
| SCD | 0.002734 | 0.00015 | -2.87375 | 9.43946 |

| | | | | |
|---|---|---|---|---|
| PARD6G | 0.002759 | 0.000151 | -2.42513 | 5.299632 |
| KRT16P1 | 0.002772 | 0.000152 | -4.36462 | 3.843765 |
| RARG | 0.002773 | 0.000153 | -2.59015 | 2.928038 |
| HIST1H1A | 0.002778 | 0.000153 | -3.08521 | 3.051739 |
| LINC00857 | 0.002804 | 0.000155 | 3.133138 | 2.553597 |
| ARMC9 | 0.002804 | 0.000155 | 3.263258 | 4.250996 |
| ASB4 | 0.002815 | 0.000156 | 9.036906 | 2.695941 |
| GPC1 | 0.002821 | 0.000156 | -1.39387 | 6.82737 |
| TRANK1 | 0.002834 | 0.000157 | 2.090894 | 6.524511 |
| LOC101927 | 0.002859 | 0.000159 | -2.81519 | 3.633193 |
| IRX3 | 0.002882 | 0.00016 | -3.53632 | 5.074664 |
| LURAP1L-A | 0.002885 | 0.000161 | 5.913135 | 1.053476 |
| ALDOC | 0.002908 | 0.000162 | -2.33627 | 4.572916 |
| FSCN1 | 0.002921 | 0.000163 | -1.99449 | 6.269985 |
| SIGLEC1 | 0.002929 | 0.000164 | 2.980471 | 4.090707 |
| EPS8L1 | 0.002935 | 0.000164 | -4.41679 | 3.484243 |
| SLC12A7 | 0.002941 | 0.000165 | 2.61202 | 5.565205 |
| MMP2 | 0.002959 | 0.000166 | 3.004666 | 4.291214 |
| RXFP4 | 0.002959 | 0.000166 | 6.553148 | 0.523545 |
| GPC4 | 0.002969 | 0.000167 | 2.319884 | 4.953006 |
| GPD1L | 0.002971 | 0.000168 | -1.69388 | 6.399259 |
| SLC12A6 | 0.003007 | 0.00017 | -2.68119 | 3.759524 |
| HOXB3 | 0.003007 | 0.00017 | 2.143805 | 5.518827 |
| MEAK7 | 0.003007 | 0.00017 | -1.86885 | 6.640984 |
| PADI2 | 0.00302 | 0.000171 | 2.94933 | 4.206916 |
| TNN | 0.003029 | 0.000172 | 4.251743 | 1.376617 |
| SPNS2 | 0.003303 | 0.000188 | -1.9282 | 6.695219 |
| CARD11 | 0.003317 | 0.000189 | 2.952209 | 4.97245 |
| EPHA3 | 0.003328 | 0.00019 | 4.329982 | 3.27526 |
| KRT6B | 0.003369 | 0.000192 | -5.04754 | 10.0955 |
| C2orf91 | 0.003425 | 0.000196 | 5.72882 | 2.959585 |
| DPP4 | 0.00346 | 0.000198 | 3.27428 | 5.234603 |
| TMEM119 | 0.003553 | 0.000204 | 3.418439 | 3.389594 |
| COLEC12 | 0.003554 | 0.000204 | 2.524521 | 4.406433 |
| MALL | 0.003608 | 0.000207 | -2.57567 | 7.06506 |
| CABCOCO1 | 0.003622 | 0.000208 | 6.668164 | 0.629413 |
| PLXNA4 | 0.003629 | 0.000209 | 3.610001 | 4.349239 |
| IVL | 0.003634 | 0.00021 | -5.92627 | 9.469126 |
| FCGBP | 0.003642 | 0.00021 | 3.893518 | 8.855384 |
| C1orf216 | 0.003645 | 0.000211 | 1.992468 | 3.707524 |
| NFE2L3 | 0.00365 | 0.000212 | 1.555227 | 5.878475 |
| RECQL5 | 0.00365 | 0.000212 | -1.42125 | 5.377665 |
| FOXN1 | 0.003663 | 0.000213 | -3.23567 | 4.530557 |
| KIAA0232 | 0.00369 | 0.000215 | -3.18013 | 2.662202 |
| HMCN1 | 0.003714 | 0.000216 | 2.668831 | 7.646498 |

| | | | | |
|---|---|---|---|---|
| ILDR2 | 0.003731 | 0.000218 | 3.543907 | 2.912122 |
| LHFPL2 | 0.00375 | 0.000219 | 1.867713 | 6.06971 |
| CAST | 0.003754 | 0.00022 | -1.96642 | 4.406547 |
| FAT1 | 0.003754 | 0.00022 | 1.542969 | 9.770893 |
| COL6A4P1 | 0.003825 | 0.000224 | 4.773196 | 1.398525 |
| PDE3B | 0.003825 | 0.000224 | 1.765463 | 4.969724 |
| GALNT10 | 0.003856 | 0.000227 | 1.754396 | 6.181549 |
| ADCY5 | 0.003871 | 0.000228 | 4.896084 | 1.991063 |
| C10orf25 | 0.003915 | 0.000231 | 4.07365 | 1.202253 |
| TMTC3 | 0.003924 | 0.000231 | -1.5104 | 6.656735 |
| TMTC2 | 0.003926 | 0.000232 | -1.4861 | 6.11608 |
| MBOAT2 | 0.00393 | 0.000232 | -2.2461 | 7.888787 |
| PODN | 0.004016 | 0.000238 | 5.779464 | 1.573297 |
| SEMA3A | 0.004049 | 0.00024 | 2.95974 | 4.605273 |
| ZNRF1 | 0.00405 | 0.00024 | -1.44911 | 5.682565 |
| SDR16C5 | 0.004068 | 0.000242 | -1.9818 | 5.722278 |
| HTRA3 | 0.004142 | 0.000247 | 2.707087 | 2.872592 |
| CDR1 | 0.004144 | 0.000247 | 1.75487 | 5.071995 |
| THBD | 0.004199 | 0.000251 | -1.49442 | 6.013292 |
| FMO9P | 0.004199 | 0.000251 | -6.48269 | 0.472221 |
| LOC10012ε | 0.004236 | 0.000253 | -2.11954 | 4.554653 |
| ZNF888 | 0.004294 | 0.000257 | 1.811427 | 4.759758 |
| B3GNTL1 | 0.004311 | 0.000259 | 3.298526 | 2.780847 |
| ARAP2 | 0.004327 | 0.00026 | -1.48237 | 7.466649 |
| LOC10192€ | 0.004327 | 0.00026 | 3.05607 | 1.950302 |
| C1orf74 | 0.004346 | 0.000262 | -2.44665 | 5.127509 |
| ARL8B | 0.004368 | 0.000263 | -1.56897 | 7.542712 |
| FOXD4L5 | 0.004402 | 0.000266 | 5.831421 | 1.027207 |
| ADAP1 | 0.004414 | 0.000267 | 4.730069 | 1.066214 |
| BNIPL | 0.004414 | 0.000267 | -3.80594 | 1.837228 |
| HRASLS | 0.0045 | 0.000273 | -4.40142 | 1.74392 |
| PTRH1 | 0.004539 | 0.000275 | 3.050815 | 1.707503 |
| IMPA2 | 0.004564 | 0.000277 | -1.78742 | 7.08361 |
| NEK5 | 0.004564 | 0.000278 | 3.241077 | 2.454274 |
| PCOLCE | 0.004564 | 0.000278 | 2.465082 | 3.713374 |
| C12orf75 | 0.004564 | 0.000278 | 2.025149 | 5.270177 |
| BST2 | 0.00461 | 0.000281 | 2.577757 | 5.554529 |
| FUT6 | 0.004621 | 0.000282 | -3.05453 | 2.385894 |
| C3orf67 | 0.004624 | 0.000283 | -3.03043 | 5.093321 |
| TMEM45B | 0.004624 | 0.000283 | -1.75616 | 6.747712 |
| RNF208 | 0.004684 | 0.000287 | -2.18659 | 3.301342 |
| TGFB1 | 0.004707 | 0.000289 | -1.56204 | 7.031861 |
| DUXAP8 | 0.004814 | 0.000296 | 3.767662 | 2.81086 |
| TMEM47 | 0.004848 | 0.000299 | 2.504043 | 4.959361 |
| PMEPA1 | 0.004861 | 0.0003 | 4.132208 | 2.4415 |

| | | | | |
|---|---|---|---|---|
| MAGI2-AS3 | 0.004861 | 0.0003 | 2.135015 | 5.087331 |
| LOC105377 | 0.004861 | 0.0003 | 5.118032 | 1.020043 |
| ATP6V1E2 | 0.004919 | 0.000304 | 4.071896 | 1.530037 |
| BST1 | 0.00495 | 0.000307 | 4.578825 | 0.58317 |
| FAM83G | 0.004959 | 0.000308 | -1.86771 | 6.014225 |
| FSIP2 | 0.004959 | 0.000308 | 2.222757 | 5.156121 |
| GDF15 | 0.005043 | 0.000314 | 3.208474 | 4.798521 |
| CD9 | 0.005119 | 0.000319 | -1.56707 | 9.038589 |
| DLEU7-AS1 | 0.005125 | 0.00032 | 4.054284 | 2.105595 |
| PHLDA1 | 0.005141 | 0.000321 | -1.62957 | 8.535427 |
| YWHAZ | 0.00515 | 0.000322 | -1.72647 | 5.129451 |
| HELZ2 | 0.005159 | 0.000323 | 1.636097 | 4.712534 |
| ADGRF2 | 0.005162 | 0.000323 | -3.87844 | 2.463171 |
| SERPINB11 | 0.005163 | 0.000324 | -6.71627 | 0.654758 |
| MIR944 | 0.005163 | 0.000324 | -7.1768 | 1.016142 |
| GID8 | 0.005163 | 0.000325 | -1.43318 | 7.528673 |
| ADRA2A | 0.005164 | 0.000325 | 3.449693 | 3.289593 |
| DOK6 | 0.005164 | 0.000326 | 2.573268 | 3.152254 |
| LPIN1 | 0.005164 | 0.000326 | -2.75949 | 3.162394 |
| JARID2 | 0.005258 | 0.000332 | -1.60939 | 5.045347 |
| FOXI2 | 0.005291 | 0.000335 | -5.9508 | 1.086875 |
| TNFRSF1B | 0.005293 | 0.000335 | 1.828933 | 4.965555 |
| ZIC2 | 0.005301 | 0.000336 | 4.630639 | 1.822244 |
| ITPK1-AS1 | 0.005347 | 0.00034 | 2.611274 | 3.366141 |
| COL1A1 | 0.005347 | 0.00034 | 1.980229 | 10.55602 |
| CDX1 | 0.005405 | 0.000344 | 6.660307 | 2.980865 |
| NAMPT | 0.005405 | 0.000344 | -1.92389 | 9.658858 |
| TTPA | 0.005447 | 0.000347 | 6.563414 | 1.665943 |
| LOC10013( | 0.005456 | 0.000349 | -2.50742 | 3.400892 |
| GDPD3 | 0.005456 | 0.000349 | -2.09084 | 5.222047 |
| GTPBP3 | 0.005456 | 0.000349 | -4.56788 | 1.722698 |
| HES1 | 0.00548 | 0.000351 | -1.57976 | 7.29319 |
| MARVELD1 | 0.005521 | 0.000355 | 1.759904 | 5.140745 |
| ANKRD10 | 0.005521 | 0.000355 | 2.396808 | 3.845364 |
| KLF8 | 0.005526 | 0.000355 | -3.37657 | 2.531573 |
| ARNT2 | 0.005617 | 0.000362 | 3.23641 | 4.021033 |
| COL1A2 | 0.005621 | 0.000363 | 1.965845 | 10.1814 |
| AGMO | 0.005621 | 0.000364 | 6.484741 | 0.490298 |
| ZCCHC24 | 0.005621 | 0.000364 | 1.909249 | 5.366792 |
| LUM | 0.005621 | 0.000364 | 2.151717 | 7.470648 |
| SDS | 0.005621 | 0.000364 | 3.347873 | 2.772654 |
| LAMC1 | 0.005646 | 0.000366 | 1.453737 | 8.220949 |
| CPED1 | 0.005671 | 0.000368 | 2.510091 | 4.26033 |
| HOTTIP | 0.0057 | 0.000371 | 4.225284 | 2.137051 |
| DZIP3 | 0.005703 | 0.000371 | 1.373297 | 5.276157 |

| | | | | |
|---|---|---|---|---|
| ADAMTS2 | 0.005748 | 0.000375 | 1.949712 | 5.492418 |
| FRZB | 0.005764 | 0.000376 | 3.009032 | 3.248874 |
| NES | 0.005784 | 0.000378 | 1.58691 | 5.741524 |
| TSPEAR-AS | 0.005869 | 0.000384 | 5.24796 | 3.327976 |
| GPSM2 | 0.005869 | 0.000384 | -1.36493 | 6.277453 |
| EMX2OS | 0.005926 | 0.000388 | 3.897239 | 2.264299 |
| SNORD57 | 0.005929 | 0.000389 | 2.624159 | 7.849958 |
| PCDHB7 | 0.005943 | 0.00039 | 3.346414 | 1.917099 |
| HABP4 | 0.005949 | 0.000391 | 2.002156 | 4.689717 |
| STEAP2 | 0.005951 | 0.000392 | 3.238609 | 2.194085 |
| FAM171A1 | 0.005985 | 0.000395 | 2.290722 | 4.560755 |
| DNM3OS | 0.006008 | 0.000397 | 2.96866 | 3.755174 |
| GABRP | 0.006048 | 0.0004 | -3.46059 | 2.812782 |
| LINC01605 | 0.006048 | 0.0004 | -2.70559 | 2.606436 |
| MAST2 | 0.006067 | 0.000402 | 1.310584 | 6.537165 |
| BCYRN1 | 0.006094 | 0.000404 | -2.18665 | 4.159362 |
| TMC3 | 0.006135 | 0.000407 | 5.096803 | 0.96162 |
| KCNK6 | 0.006138 | 0.000408 | -1.37869 | 7.041563 |
| SLC22A31 | 0.006145 | 0.000409 | 6.859931 | 0.793526 |
| GLS | 0.006145 | 0.000409 | 1.541211 | 6.575149 |
| ITPR2 | 0.006145 | 0.00041 | -1.29201 | 8.550127 |
| CALM1 | 0.006233 | 0.000416 | -1.38247 | 9.487106 |
| DLEU1-AS1 | 0.006292 | 0.000421 | 3.710109 | 2.092702 |
| MYOC | 0.006292 | 0.000421 | 6.326675 | 4.260065 |
| LOC101927 | 0.00637 | 0.000427 | -2.89734 | 2.254413 |
| TNRC18 | 0.00637 | 0.000427 | 1.337062 | 8.827579 |
| HK2 | 0.006383 | 0.000429 | -1.93476 | 8.466466 |
| MIR6814 | 0.006432 | 0.000432 | -3.87566 | 1.570934 |
| PEX3 | 0.00647 | 0.000435 | -1.3842 | 5.28082 |
| DNAH7 | 0.006504 | 0.000439 | 2.825578 | 4.207991 |
| ST3GAL2 | 0.006504 | 0.000439 | 1.978817 | 4.260191 |
| ZNF385D | 0.006504 | 0.00044 | 4.368841 | 2.164446 |
| ZBTB38 | 0.006504 | 0.00044 | 1.399822 | 7.305126 |
| HEYL | 0.006538 | 0.000442 | 3.415193 | 4.564981 |
| TLN1 | 0.006568 | 0.000445 | 1.45403 | 9.12232 |
| GSTP1 | 0.006634 | 0.00045 | -1.71495 | 8.468143 |
| LAD1 | 0.006669 | 0.000453 | -1.83995 | 8.805631 |
| ARF5 | 0.006743 | 0.000458 | -1.28097 | 5.922386 |
| NPAS2 | 0.006746 | 0.000459 | -1.36443 | 7.067772 |
| OXTR | 0.006784 | 0.000462 | 4.711704 | 3.521053 |
| CD164L2 | 0.00684 | 0.000467 | -3.90745 | 2.871085 |
| LOC550113 | 0.006874 | 0.000469 | -4.9351 | 2.373197 |
| PPDPF | 0.006879 | 0.00047 | -1.40846 | 8.322905 |
| MUC5B | 0.006942 | 0.000475 | 3.626695 | 7.785236 |
| CACNA1C-/ | 0.006961 | 0.000477 | 4.252963 | 1.940667 |

| | | | | |
|---|---|---|---|---|
| TNR | 0.007006 | 0.000481 | 4.738499 | 0.707067 |
| CD93 | 0.007035 | 0.000483 | 1.667765 | 6.344002 |
| DNAH14 | 0.007076 | 0.000487 | 2.012061 | 4.868676 |
| POU3F1 | 0.007076 | 0.000487 | -3.61426 | 2.034074 |
| MEIS1-AS3 | 0.007083 | 0.000488 | 4.259678 | 1.790612 |
| SEC16B | 0.007097 | 0.000489 | 2.374311 | 5.18688 |
| ADAMTSL5 | 0.0071 | 0.00049 | -1.73473 | 4.312036 |
| FPR3 | 0.007122 | 0.000492 | 2.535616 | 3.712456 |
| RAP2A | 0.007142 | 0.000494 | -1.58687 | 6.717671 |
| CAPN8 | 0.007157 | 0.000496 | 3.766837 | 6.646471 |
| CLIP1-AS1 | 0.007246 | 0.000503 | -2.30156 | 3.954458 |
| HEPH | 0.007273 | 0.000505 | 7.798326 | 1.544193 |
| LINC00278 | 0.007301 | 0.000508 | -3.802 | 3.549716 |
| AFDN | 0.007301 | 0.000508 | -1.46091 | 5.498083 |
| LOC10013( | 0.007301 | 0.000509 | -1.68772 | 5.916066 |
| SLC28A2 | 0.007309 | 0.00051 | 3.520672 | 2.095634 |
| IKZF2 | 0.007313 | 0.00051 | -3.04479 | 2.304359 |
| HOOK1 | 0.007375 | 0.000515 | -1.6718 | 6.99745 |
| WDR91 | 0.007413 | 0.000519 | -1.35556 | 5.942941 |
| JPH2 | 0.007413 | 0.000519 | 3.667687 | 4.303252 |
| SSC5D | 0.007436 | 0.000522 | 1.966699 | 4.083127 |
| EP300-AS1 | 0.007436 | 0.000522 | -3.66764 | 1.9062 |
| PHGR1 | 0.007436 | 0.000522 | 4.937053 | 2.554882 |
| PLOD3 | 0.007504 | 0.000528 | 2.229009 | 4.868087 |
| HTR2A | 0.007511 | 0.000529 | 6.562852 | 0.525385 |
| KSR1 | 0.007533 | 0.000531 | -1.3639 | 6.163797 |
| LOXL2 | 0.007539 | 0.000532 | 2.501106 | 5.183541 |
| CELSR1 | 0.007539 | 0.000533 | -1.87513 | 7.887254 |
| CD24 | 0.007615 | 0.000539 | -2.33188 | 4.147207 |
| SRR | 0.00782 | 0.000554 | -5.43574 | 0.552628 |
| CRYBG3 | 0.00784 | 0.000556 | -1.2487 | 8.218191 |
| MXRA5 | 0.007846 | 0.000557 | 1.691382 | 8.377678 |
| DSG2 | 0.007883 | 0.00056 | 1.744836 | 7.893809 |
| HEBP2 | 0.007883 | 0.000561 | -1.43381 | 7.139245 |
| PMVK | 0.007916 | 0.000563 | -1.565 | 5.380425 |
| DYNAP | 0.00795 | 0.000567 | -7.12424 | 0.980462 |
| KMT5A | 0.007971 | 0.000569 | -1.25598 | 6.622072 |
| PABPC1 | 0.008008 | 0.000572 | -1.51719 | 11.45704 |
| ANKRD37 | 0.008008 | 0.000572 | -3.07915 | 3.91527 |
| ANAPC7 | 0.008056 | 0.000577 | -1.6657 | 4.50004 |
| SPRR1B | 0.008056 | 0.000577 | -5.09267 | 9.142893 |
| PRKCH | 0.008066 | 0.000578 | -1.4689 | 6.283991 |
| DNAH2 | 0.008066 | 0.000579 | 2.543207 | 3.948574 |
| TRPM2-AS | 0.008086 | 0.000581 | 4.767171 | 1.208331 |
| KIF3B | 0.008109 | 0.000583 | 1.342525 | 6.741111 |

| | | | | |
|---|---|---|---|---|
| HCAR3 | 0.008146 | 0.000587 | -3.40101 | 3.325747 |
| TP53INP2 | 0.008165 | 0.000589 | -1.58075 | 7.145575 |
| CDK5R1 | 0.008188 | 0.000591 | -2.14973 | 4.176815 |
| RBBP8NL | 0.008225 | 0.000594 | -2.80218 | 3.100345 |
| GID4 | 0.008239 | 0.000596 | -1.58209 | 5.422901 |
| IGF1 | 0.008239 | 0.000596 | 5.062368 | 1.439858 |
| LOC10192{ | 0.008289 | 0.000601 | 7.039943 | 0.914712 |
| IL36A | 0.00831 | 0.000603 | -4.07416 | 6.247721 |
| CACNB1 | 0.00831 | 0.000603 | 2.518018 | 2.829139 |
| ABLIM1 | 0.00831 | 0.000604 | -1.62965 | 4.748414 |
| CAPN6 | 0.008362 | 0.000609 | 2.611006 | 4.598917 |
| SLC9A9 | 0.008372 | 0.00061 | -1.94226 | 4.863984 |
| MYO15B | 0.008375 | 0.000611 | 1.534346 | 7.29725 |
| ABCC3 | 0.008378 | 0.000612 | 1.920165 | 6.803061 |
| ARHGEF4 | 0.008378 | 0.000612 | -2.63903 | 4.428342 |
| RRN3P2 | 0.008378 | 0.000613 | 2.328506 | 4.582577 |
| ACER1 | 0.008382 | 0.000614 | -3.84446 | 2.370537 |
| ZNF618 | 0.008383 | 0.000615 | 1.703174 | 5.662901 |
| SNX33 | 0.008418 | 0.000618 | -1.28581 | 6.977526 |
| FAR1 | 0.00848 | 0.000623 | -1.32915 | 8.393602 |
| TRABD2A | 0.008567 | 0.00063 | 4.026186 | 1.501955 |
| DOP1B | 0.008616 | 0.000635 | -1.38065 | 7.360393 |
| GNAI3 | 0.008616 | 0.000635 | -1.58681 | 7.80907 |
| CBLB | 0.008616 | 0.000636 | 1.348516 | 6.250497 |
| CACNG8 | 0.008687 | 0.000641 | 3.980585 | 3.265585 |
| SCCPDH | 0.008696 | 0.000643 | 1.919734 | 4.683752 |
| AACS | 0.008732 | 0.000646 | -1.44505 | 6.247933 |
| PROX1 | 0.008759 | 0.000649 | 4.818756 | 4.207977 |
| FAM157C | 0.008759 | 0.000649 | -1.81065 | 4.641527 |
| CHST5 | 0.008796 | 0.000653 | 5.631181 | 1.4096 |
| MLXIP | 0.008848 | 0.000657 | 1.327749 | 7.732771 |
| CYB5R1 | 0.008946 | 0.000665 | -1.29028 | 5.757809 |
| IER3 | 0.009057 | 0.000674 | -3.03093 | 4.142349 |
| CMBL | 0.009079 | 0.000677 | 2.195421 | 4.350516 |
| CILP2 | 0.009156 | 0.000683 | 6.450074 | 0.462059 |
| AKR1B15 | 0.009185 | 0.000686 | -3.15757 | 2.190697 |
| KRT3 | 0.009195 | 0.000687 | -3.64909 | 3.206999 |
| BCL9 | 0.009201 | 0.000689 | 1.342558 | 5.985435 |
| AKR1C4 | 0.009201 | 0.000689 | 7.131265 | 1.006767 |
| LINC01279 | 0.009235 | 0.000693 | 2.207893 | 5.320488 |
| FOXL1 | 0.009339 | 0.000701 | 3.709057 | 2.317364 |
| NBPF14 | 0.009339 | 0.000702 | -1.54783 | 5.368368 |
| DGCR11 | 0.009355 | 0.000704 | -1.84073 | 4.07155 |
| ABCA9 | 0.009355 | 0.000704 | 2.729769 | 4.548465 |
| ATP10A | 0.009375 | 0.000707 | 2.30116 | 4.469766 |

| | | | | |
|---|---|---|---|---|
| VASP | 0.00941 | 0.00071 | 1.274836 | 6.951232 |
| VIT | 0.009456 | 0.000714 | -6.60229 | 1.490629 |
| PLN | 0.009478 | 0.000717 | 3.069344 | 4.601169 |
| IGFBP4 | 0.009494 | 0.000718 | 1.994707 | 7.847406 |
| FAM83H-A | 0.009509 | 0.00072 | -1.8887 | 5.492239 |
| LBX2-AS1 | 0.00959 | 0.000727 | 3.012886 | 2.659791 |
| JMJD7 | 0.009671 | 0.000734 | -1.79429 | 4.076409 |
| BATF2 | 0.009671 | 0.000735 | 5.335712 | 0.634294 |
| DDIT4 | 0.009671 | 0.000736 | -1.86488 | 8.95846 |
| RGS16 | 0.009764 | 0.000743 | 2.723617 | 3.134065 |
| VSNL1 | 0.009788 | 0.000746 | -2.12848 | 5.576743 |
| MAN1A1 | 0.00981 | 0.000748 | 1.585071 | 6.663866 |
| GPA33 | 0.009843 | 0.000752 | 3.823205 | 2.800821 |
| HOXB-AS3 | 0.009844 | 0.000754 | 4.866558 | 1.958484 |
| ADAMTS6 | 0.009844 | 0.000754 | 2.599411 | 3.918569 |
| NINJ1 | 0.009844 | 0.000754 | 1.78755 | 4.285379 |
| SH3PXD2A- | 0.009868 | 0.000757 | -2.39864 | 5.049084 |
| TMPRSS11I | 0.009879 | 0.000758 | -4.34582 | 5.770359 |
| COL4A3 | 0.009907 | 0.000761 | 2.607774 | 4.116726 |
| PDGFA | 0.00998 | 0.000768 | 6.37003 | 0.403724 |
| PTPRH | 0.00998 | 0.000768 | 4.137967 | 2.503399 |
| MACO1 | 0.009986 | 0.00077 | -1.57391 | 4.417108 |
| EPGN | 0.009986 | 0.00077 | -4.44266 | 2.074192 |
| CFH | 0.00999 | 0.000771 | 1.980892 | 6.927513 |
| ANO1 | 0.00999 | 0.000772 | 2.065078 | 4.535734 |
| C3orf70 | 0.010018 | 0.000775 | 2.937083 | 3.707473 |
| MVD | 0.010018 | 0.000776 | -1.70667 | 5.238602 |
| MAP9 | 0.010055 | 0.000779 | 2.381344 | 4.922658 |
| LRRN4CL | 0.010073 | 0.000782 | 3.338587 | 2.519055 |
| QSOX1 | 0.010073 | 0.000782 | -1.63281 | 6.976041 |
| SYCP2 | 0.010122 | 0.000787 | 2.214935 | 4.862405 |
| ABCC2 | 0.01022 | 0.000795 | 3.730452 | 4.557325 |
| DDX60L | 0.01024 | 0.000798 | 1.893793 | 4.481493 |
| TENM2 | 0.010457 | 0.000815 | -4.34639 | 1.831388 |
| HECTD1 | 0.01046 | 0.000816 | -1.36243 | 9.722252 |
| BICDL1 | 0.01048 | 0.000819 | -1.62584 | 6.01047 |
| POLR2A | 0.010554 | 0.000826 | -1.26722 | 9.168891 |
| LINC01819 | 0.010554 | 0.000826 | 5.128704 | 4.096566 |
| LOC101927 | 0.010554 | 0.000827 | -2.8737 | 2.311721 |
| ROCK2 | 0.010582 | 0.00083 | 1.275149 | 8.054401 |
| LTBP2 | 0.010716 | 0.000841 | 1.431208 | 7.049854 |
| SPRR2A | 0.010725 | 0.000843 | -4.99331 | 9.361513 |
| HERC5 | 0.010732 | 0.000844 | 2.26907 | 4.118855 |
| KLHL42 | 0.010768 | 0.000848 | 1.190021 | 5.939381 |
| F13A1 | 0.010822 | 0.000853 | 1.904884 | 5.71139 |

| | | | | |
|---|---|---|---|---|
| MMP3 | 0.010826 | 0.000855 | 4.946845 | 4.807871 |
| RASSF4 | 0.010826 | 0.000855 | 2.71577 | 4.489096 |
| USP18 | 0.010865 | 0.000859 | 2.108404 | 3.622609 |
| ATP13A4-A | 0.010865 | 0.000859 | -5.34292 | 1.203095 |
| RAPGEFL1 | 0.010893 | 0.000863 | -2.43166 | 4.419652 |
| SLC52A3 | 0.010897 | 0.000864 | 1.856283 | 4.146109 |
| SLC45A3 | 0.010938 | 0.000868 | 2.898817 | 3.444356 |
| HSPA4 | 0.010953 | 0.00087 | -1.29579 | 8.160703 |
| KATNBL1 | 0.011025 | 0.000877 | -1.42806 | 5.670877 |
| EMILIN1 | 0.011025 | 0.000877 | 2.390346 | 6.653394 |
| ANKRD44 | 0.011025 | 0.000878 | 1.789656 | 4.380574 |
| FRRS1L | 0.011037 | 0.00088 | 5.826371 | 1.015456 |
| SRD5A1 | 0.011086 | 0.000885 | -2.06681 | 4.791195 |
| DPY19L1 | 0.011108 | 0.000887 | 1.760498 | 5.438717 |
| CTGF | 0.011108 | 0.000888 | 2.169575 | 7.187529 |
| BNC1 | 0.011109 | 0.000889 | -4.27609 | 0.802065 |
| NCR3LG1 | 0.011109 | 0.00089 | 1.478042 | 4.936869 |
| NTNG2 | 0.011126 | 0.000892 | 3.389875 | 1.778091 |
| LINC00312 | 0.011149 | 0.000895 | 2.554599 | 3.480042 |
| SPATA17 | 0.01116 | 0.000897 | 3.519156 | 1.571846 |
| HOXC-AS3 | 0.01116 | 0.000897 | 5.116087 | 0.479626 |
| SEC24D | 0.01122 | 0.000903 | 1.428953 | 6.313004 |
| LINC00578 | 0.011254 | 0.000907 | 3.363464 | 3.114152 |
| KCNMB1 | 0.011299 | 0.000911 | 3.355466 | 3.67747 |
| SELP | 0.01131 | 0.000913 | 3.269817 | 2.242352 |
| MPEG1 | 0.011333 | 0.000915 | 1.789519 | 5.126962 |
| THAP3 | 0.011418 | 0.000923 | 5.155449 | 0.486122 |
| NDST1 | 0.011558 | 0.000935 | -2.57104 | 2.555188 |
| ARSB | 0.011564 | 0.000937 | 2.177938 | 3.797682 |
| EPN2-IT1 | 0.011608 | 0.000941 | -2.54962 | 2.99584 |
| IRF8 | 0.011611 | 0.000942 | 2.471137 | 4.668364 |
| FTL | 0.011659 | 0.000948 | 1.363276 | 9.46572 |
| COL9A2 | 0.011659 | 0.000949 | 3.040256 | 4.715859 |
| MRPS10 | 0.011659 | 0.00095 | -1.30518 | 5.933444 |
| LINC01088 | 0.011659 | 0.000951 | 5.146958 | 0.93266 |
| THOC3 | 0.011659 | 0.000951 | -2.33599 | 4.03994 |
| PLA2G4D | 0.011665 | 0.000952 | -3.53226 | 3.598734 |
| BZW1 | 0.011771 | 0.000962 | -3.68649 | 1.300223 |
| TPTE2P6 | 0.011771 | 0.000963 | 5.282961 | 0.58051 |
| METAP1 | 0.011791 | 0.000965 | -1.46798 | 6.72003 |
| HSH2D | 0.011889 | 0.000974 | 4.710404 | 0.872173 |
| RTL8C | 0.011899 | 0.000976 | -1.29859 | 6.225077 |
| KRT16P3 | 0.011903 | 0.000978 | -4.03091 | 4.047561 |
| BCL2L15 | 0.011903 | 0.000978 | 3.131536 | 4.290703 |
| RNASE7 | 0.011908 | 0.000979 | -2.9988 | 3.656572 |

| | | | | |
|---|---|---|---|---|
| CIPC | 0.011932 | 0.000982 | -1.29967 | 5.763655 |
| ZDHHC5 | 0.012004 | 0.000989 | -1.22761 | 7.993375 |
| LAPTM5 | 0.012051 | 0.000994 | 1.677981 | 6.17845 |
| LOC10012ᵋ | 0.012059 | 0.000995 | -1.63231 | 6.456514 |
| SNORD113 | 0.012062 | 0.000996 | 2.506782 | 4.207432 |
| UGT1A5 | 0.012062 | 0.000997 | -4.64734 | 1.516073 |
| ATCAY | 0.012085 | 0.001 | 6.522982 | 0.499175 |
| ATP8B2 | 0.012102 | 0.001003 | 1.548477 | 5.456461 |
| NAPSB | 0.012197 | 0.001011 | 3.045473 | 2.510435 |
| RIC8B | 0.012536 | 0.00104 | 1.854289 | 4.040804 |
| SOX21 | 0.012656 | 0.001051 | -3.06183 | 4.086565 |
| ANKRD36 | 0.012664 | 0.001053 | 1.356027 | 7.407468 |
| PDLIM2 | 0.012782 | 0.001064 | -2.31683 | 3.869072 |
| KRT24 | 0.012975 | 0.001081 | -4.34443 | 6.400345 |
| NOTCH2NL | 0.01302 | 0.001086 | -1.68803 | 5.429322 |
| SPON2 | 0.01302 | 0.001087 | 2.509319 | 2.362721 |
| SPOCK1 | 0.013066 | 0.001091 | 2.402396 | 3.961384 |
| KRT6C | 0.013066 | 0.001092 | -5.39858 | 10.1486 |
| MICALL2 | 0.0131 | 0.001097 | 1.342438 | 6.103899 |
| CDC42EP2 | 0.0131 | 0.001097 | 2.359355 | 3.240807 |
| NT5DC3 | 0.013124 | 0.0011 | 1.513868 | 6.027351 |
| TMC7 | 0.013137 | 0.001102 | 5.206591 | 0.438209 |
| PDE3A | 0.013255 | 0.001113 | 4.323069 | 2.06254 |
| EEPD1 | 0.013263 | 0.001115 | 1.608545 | 5.285705 |
| CASTOR3 | 0.013349 | 0.001123 | 2.146983 | 4.564217 |
| LHFPL6 | 0.013457 | 0.001133 | 1.664513 | 5.267721 |
| ATP10D | 0.013497 | 0.001138 | -1.36368 | 7.053838 |
| RAB27B | 0.013507 | 0.001139 | -1.74905 | 7.175373 |
| SUCLG1 | 0.013512 | 0.001142 | -1.3212 | 6.138065 |
| ANGPTL2 | 0.013512 | 0.001142 | 1.462805 | 5.43207 |
| KIFC3 | 0.013522 | 0.001144 | -3.21568 | 1.959927 |
| ABCC9 | 0.013542 | 0.001147 | 4.186491 | 3.417148 |
| DGKA | 0.013542 | 0.001148 | -2.34041 | 3.145967 |
| FMNL2 | 0.013552 | 0.001149 | 1.27071 | 6.858085 |
| MYOM3 | 0.013677 | 0.001161 | 3.344178 | 3.241414 |
| S1PR3 | 0.013712 | 0.001165 | 2.850086 | 4.822389 |
| PRKCA | 0.013871 | 0.00118 | 2.282831 | 6.497142 |
| LPIN2 | 0.013936 | 0.001186 | 2.169002 | 7.968806 |
| HMGCR | 0.01398 | 0.001191 | -3.44337 | 1.423611 |
| CRYBG2 | 0.013992 | 0.001195 | -2.79318 | 8.183609 |
| C11orf42 | 0.013992 | 0.001195 | 4.528839 | 0.54291 |
| MEIS3P1 | 0.013992 | 0.001197 | 2.002111 | 3.560209 |
| FER1L6-AS: | 0.013992 | 0.001197 | 4.566365 | 2.467777 |
| WDFY4 | 0.013992 | 0.001197 | 1.730717 | 4.627325 |
| OTUD6B-A: | 0.014039 | 0.001203 | 1.453951 | 4.3894 |

| | | | | |
|---|---|---|---|---|
| SARM1 | 0.014054 | 0.001205 | 2.18478 | 3.064148 |
| HOXC-AS1 | 0.014202 | 0.001219 | 5.09114 | 0.981245 |
| MMP25 | 0.014275 | 0.001226 | 2.547878 | 3.15466 |
| PPP1R3G | 0.014365 | 0.001235 | -3.13031 | 2.643181 |
| LPCAT1 | 0.014365 | 0.001236 | 2.969559 | 3.805347 |
| CAMTA1 | 0.014381 | 0.001238 | 2.016475 | 3.268305 |
| CYP4A11 | 0.014493 | 0.001249 | -3.106 | 2.291711 |
| COL3A1 | 0.014676 | 0.001267 | 1.782563 | 10.67696 |
| FER1L4 | 0.014676 | 0.001267 | 1.857005 | 5.847909 |
| CFAP43 | 0.014979 | 0.001294 | 3.033397 | 2.455443 |
| DIP2B | 0.015104 | 0.001307 | -1.41483 | 7.858147 |
| LMOD1 | 0.015104 | 0.001308 | 3.050805 | 7.634768 |
| MIR31HG | 0.015116 | 0.00131 | -2.26422 | 3.55566 |
| TSPAN18 | 0.015253 | 0.001323 | 1.799591 | 4.191025 |
| ZFHX4 | 0.015266 | 0.001325 | 1.727293 | 5.199879 |
| CD207 | 0.015302 | 0.001329 | -3.99732 | 1.696116 |
| TTLL12 | 0.015313 | 0.001331 | -1.62402 | 7.240981 |
| SIPA1L3 | 0.015345 | 0.001337 | 1.204831 | 6.975339 |
| TTLL6 | 0.015345 | 0.001337 | 4.896094 | 0.846455 |
| MTURN | 0.015345 | 0.001338 | 1.58436 | 7.015234 |
| EPB41L5 | 0.015419 | 0.001345 | 4.051692 | 1.440386 |
| CDA | 0.015506 | 0.001355 | -2.91342 | 6.019591 |
| CLDN7 | 0.015506 | 0.001356 | -3.10495 | 2.616371 |
| PLUT | 0.015506 | 0.001357 | 5.967376 | 1.157185 |
| RARRES2 | 0.015577 | 0.001364 | 2.428407 | 4.499937 |
| RIOK3 | 0.015591 | 0.001366 | -1.52641 | 8.492264 |
| CTNNA3 | 0.015645 | 0.001372 | 6.504202 | 0.507249 |
| RXRA | 0.015667 | 0.001375 | -2.06822 | 3.276275 |
| TRIM31-AS | 0.015736 | 0.001383 | 3.876532 | 1.808848 |
| SH3PXD2A | 0.01574 | 0.001385 | -1.26511 | 9.192038 |
| SIX3 | 0.01574 | 0.001386 | 5.193675 | 0.530791 |
| DCTN1-AS1 | 0.01574 | 0.001388 | 3.994709 | 0.771492 |
| B3GNT5 | 0.01574 | 0.001388 | -1.81092 | 7.483227 |
| SOWAHC | 0.015758 | 0.001391 | -2.13286 | 7.361412 |
| WNT4 | 0.015758 | 0.001392 | -1.81829 | 4.351901 |
| SESN2 | 0.015793 | 0.001396 | -1.44541 | 5.036603 |
| CDCA7 | 0.01583 | 0.001401 | 4.809583 | 1.135931 |
| IFI35 | 0.01584 | 0.001403 | 1.865755 | 4.073357 |
| MMP21 | 0.015875 | 0.001408 | 5.357421 | 0.660265 |
| HS3ST3A1 | 0.015875 | 0.001408 | -2.2238 | 4.020024 |
| SYT15 | 0.01592 | 0.001413 | -2.84357 | 1.910271 |
| GMPR2 | 0.015993 | 0.001422 | -3.52787 | 1.478203 |
| GALNT17 | 0.015993 | 0.001422 | 3.116091 | 3.100606 |
| TM4SF19-A | 0.016069 | 0.00143 | -3.08472 | 2.54952 |
| MME | 0.016087 | 0.001433 | 5.409419 | 1.243841 |

| | | | | |
|---|---|---|---|---|
| TIFA | 0.016252 | 0.001449 | -1.94241 | 4.925301 |
| BNIP3L | 0.016258 | 0.001451 | -1.38292 | 7.607686 |
| PLXNA2 | 0.01627 | 0.001453 | -1.13347 | 7.837133 |
| NRIP1 | 0.016337 | 0.00146 | -1.38564 | 7.818356 |
| DNAJB1 | 0.016354 | 0.001464 | -1.79636 | 7.050955 |
| LOC101927 | 0.016354 | 0.001464 | 3.037117 | 2.78741 |
| HS3ST5 | 0.016379 | 0.001468 | 5.766347 | 0.971111 |
| SEMA3C | 0.016379 | 0.001469 | 1.744477 | 6.071963 |
| HS1BP3 | 0.016402 | 0.001473 | 1.409453 | 4.563322 |
| LGALS1 | 0.016402 | 0.001475 | 1.604843 | 4.827792 |
| KCNN4 | 0.016402 | 0.001477 | 2.524902 | 5.200529 |
| FNDC10 | 0.016402 | 0.001477 | -2.51425 | 2.147259 |
| LINC02516 | 0.016402 | 0.001479 | -2.91743 | 2.302867 |
| NEBL | 0.016402 | 0.00148 | -1.67918 | 5.979264 |
| PECAM1 | 0.016402 | 0.001481 | 1.403315 | 7.11329 |
| PGS1 | 0.016402 | 0.001482 | 5.701054 | 0.925941 |
| CPE | 0.016402 | 0.001482 | 1.696274 | 5.455934 |
| HOXB13 | 0.01641 | 0.001484 | 6.572709 | 0.56112 |
| KIF3A | 0.016455 | 0.00149 | 5.202028 | 0.550512 |
| MMP12 | 0.016472 | 0.001493 | 4.381303 | 2.958338 |
| SYNPO | 0.016535 | 0.001499 | 1.605804 | 5.598348 |
| COTL1 | 0.016675 | 0.001513 | 1.632778 | 6.503102 |
| LAMC2 | 0.016691 | 0.001516 | 2.108853 | 5.567058 |
| SLC5A4 | 0.016742 | 0.001522 | 3.019789 | 1.898961 |
| KCNH8 | 0.016791 | 0.001528 | 3.333608 | 3.861398 |
| GLDC | 0.016791 | 0.001529 | -3.08131 | 2.985714 |
| HOXB6 | 0.016802 | 0.001531 | 3.468173 | 5.040617 |
| KLK10 | 0.016804 | 0.001534 | -2.39824 | 2.897788 |
| LINC01770 | 0.016804 | 0.001534 | 6.436317 | 0.465219 |
| STEAP1 | 0.016809 | 0.001536 | 2.052979 | 4.821611 |
| FABP5P3 | 0.016928 | 0.001548 | -3.76948 | 1.15196 |
| FRY | 0.016937 | 0.00155 | 1.900612 | 6.087423 |
| RMND5A | 0.016971 | 0.001555 | -1.20084 | 7.768038 |
| SPPL3 | 0.017048 | 0.001563 | -1.26785 | 6.517064 |
| CCDC71L | 0.017059 | 0.001565 | 2.13774 | 5.058739 |
| CST1 | 0.017158 | 0.001576 | 5.006546 | 0.399154 |
| GUCY1A1 | 0.017164 | 0.001577 | 4.013573 | 1.257419 |
| CCDC200 | 0.017204 | 0.001582 | 2.629846 | 2.804083 |
| CALM2 | 0.017229 | 0.001586 | 1.675857 | 5.106837 |
| PLAC8L1 | 0.017229 | 0.001587 | -4.29464 | 0.815885 |
| KIF26B | 0.017268 | 0.001592 | 2.389749 | 3.373717 |
| C15orf56 | 0.017318 | 0.001598 | -3.10554 | 2.340912 |
| HOXA10 | 0.017433 | 0.00161 | 6.192437 | 0.292488 |
| FAM174A | 0.017524 | 0.001621 | -1.61712 | 4.607327 |
| GRHL2 | 0.017524 | 0.001621 | -1.35437 | 6.931566 |

| | | | | |
|---|---|---|---|---|
| HOXB4 | 0.017571 | 0.001627 | 1.888639 | 4.737976 |
| TNNC1 | 0.017638 | 0.001635 | 4.282434 | 1.271407 |
| JAG1 | 0.017689 | 0.001641 | -1.20858 | 8.374656 |
| ING1 | 0.017745 | 0.001647 | 2.362974 | 2.655428 |
| MSH4 | 0.017862 | 0.001659 | 4.098772 | 1.947394 |
| IGFBP7 | 0.017879 | 0.001662 | 2.377454 | 3.006493 |
| FGF20 | 0.017982 | 0.001673 | 7.074928 | 0.964544 |
| EVI2B | 0.018008 | 0.001677 | 1.552855 | 5.418857 |
| NHSL1 | 0.018042 | 0.001682 | -1.70833 | 4.344804 |
| SH3BP4 | 0.018067 | 0.001685 | 1.622917 | 6.385967 |
| SOBP | 0.018144 | 0.001694 | 2.635078 | 4.166244 |
| KIF1C | 0.018155 | 0.001696 | -1.18505 | 8.937304 |
| ROR2 | 0.018211 | 0.001704 | 2.105699 | 4.102484 |
| CHDH | 0.018211 | 0.001704 | 2.395818 | 4.096927 |
| PARP14 | 0.018256 | 0.00171 | 1.387543 | 8.433713 |
| PITHD1 | 0.018353 | 0.001723 | -1.362 | 6.256649 |
| FEZF1-AS1 | 0.018353 | 0.001723 | 4.729156 | 1.060939 |
| BAMBI | 0.018353 | 0.001723 | 4.568097 | 5.013786 |
| SGCB | 0.018357 | 0.001725 | 1.33848 | 5.688644 |
| LCP1 | 0.01844 | 0.001734 | 1.732629 | 6.234731 |
| LFNG | 0.01848 | 0.001739 | 4.408576 | 1.568983 |
| ENO1 | 0.018486 | 0.001741 | -1.33385 | 6.883921 |
| SFTA2 | 0.018541 | 0.001748 | -3.89724 | 1.216645 |
| C15orf62 | 0.018541 | 0.001749 | -1.45476 | 4.667323 |
| FBN2 | 0.018592 | 0.001755 | 2.404034 | 4.296279 |
| GPRIN3 | 0.018692 | 0.001766 | 1.7895 | 5.610906 |
| ZEB2-AS1 | 0.018736 | 0.001772 | 7.098858 | 0.96462 |
| FAM133CP | 0.018736 | 0.001773 | 3.451704 | 2.972144 |
| KBTBD11 | 0.018902 | 0.001791 | 5.450675 | 1.691519 |
| PDILT | 0.018956 | 0.001797 | -7.11268 | 3.508768 |
| CCDC26 | 0.019081 | 0.00181 | -6.8998 | 0.792865 |
| SERPINE1 | 0.019164 | 0.00182 | 2.241242 | 7.14981 |
| PDGFRL | 0.019164 | 0.001821 | 3.697324 | 1.699199 |
| ADAMTS2( | 0.019468 | 0.001852 | 6.588473 | 0.5487 |
| NMRAL2P | 0.019619 | 0.001868 | -2.47898 | 3.944719 |
| ETFBKMT | 0.019619 | 0.001869 | 5.204837 | 0.935562 |
| CXCL3 | 0.019638 | 0.001872 | 3.442125 | 3.716652 |
| GLI2 | 0.019682 | 0.001878 | 1.983706 | 3.820591 |
| CFAP100 | 0.019694 | 0.001881 | 4.09279 | 1.79722 |
| ADGRA2 | 0.0197 | 0.001883 | 1.759401 | 5.635178 |
| LOC44030( | 0.019814 | 0.001895 | -1.15225 | 6.619258 |
| STX5 | 0.019822 | 0.001897 | 6.554042 | 0.550331 |
| BLVRB | 0.019822 | 0.001899 | -1.79152 | 5.277479 |
| OXCT1 | 0.019859 | 0.001905 | -1.4395 | 6.194033 |
| SLC25A43 | 0.019859 | 0.001906 | -1.41487 | 5.366469 |

| | | | | |
|---|---|---|---|---|
| ASPHD2 | 0.019859 | 0.001907 | 1.681444 | 3.872653 |
| SLC22A15 | 0.019884 | 0.001911 | -1.3179 | 4.935324 |
| HSPB8 | 0.019884 | 0.001914 | -1.6845 | 6.281319 |
| MGAT5 | 0.019884 | 0.001914 | 1.191763 | 7.348239 |
| PCAT14 | 0.019898 | 0.001917 | 5.7062 | 0.88911 |
| CLRN3 | 0.019957 | 0.001924 | 4.406754 | 3.289483 |
| ATP2C2 | 0.020007 | 0.001931 | -4.32951 | 1.617444 |
| VAT1L | 0.020007 | 0.001932 | 4.131186 | 1.336201 |
| GPC6-AS2 | 0.020041 | 0.001937 | 3.810605 | 1.432582 |
| LOC10012[illegible] | 0.020045 | 0.001939 | 4.560982 | 1.200077 |
| CALML3 | 0.020173 | 0.001952 | -4.0761 | 8.483652 |
| CYP4F35P | 0.020176 | 0.001954 | -3.14197 | 3.755378 |
| FABP12 | 0.020271 | 0.001966 | -4.19037 | 0.731535 |
| KCTD12 | 0.020271 | 0.001967 | 1.388752 | 6.804806 |
| TMC2 | 0.02029 | 0.00197 | 3.362289 | 1.097279 |
| FMNL1 | 0.020304 | 0.001973 | 1.519307 | 5.160974 |
| MIR608 | 0.02032 | 0.001976 | 6.247058 | 0.33977 |
| ANXA9 | 0.020323 | 0.001979 | -1.51354 | 5.134449 |
| PLEKHO1 | 0.020323 | 0.001979 | 3.262425 | 1.769944 |
| HSBP1 | 0.020373 | 0.001986 | -1.28802 | 6.565834 |
| AQP5 | 0.020398 | 0.00199 | 3.389759 | 3.832944 |
| ABCC5 | 0.020401 | 0.001993 | -1.13912 | 7.271454 |
| VLDLR | 0.020401 | 0.001994 | -4.12476 | 1.230625 |
| TNS3 | 0.020401 | 0.001995 | 1.875242 | 7.366243 |
| SLC27A4 | 0.020425 | 0.001999 | -1.40812 | 5.662818 |
| ZNF512B | 0.020442 | 0.002002 | -1.12769 | 5.798705 |
| MYO1F | 0.02053 | 0.002013 | 1.504967 | 5.16589 |
| TET1 | 0.02053 | 0.002014 | 1.586217 | 4.289848 |
| SRP54 | 0.020536 | 0.002016 | 5.013384 | 0.410237 |
| FAM198A | 0.020587 | 0.002022 | 2.92585 | 3.129903 |
| LOC10037[illegible] | 0.020592 | 0.002025 | 3.643665 | 1.133137 |
| ZMIZ1 | 0.020592 | 0.002026 | -1.13846 | 8.28215 |
| HTR1B | 0.0206 | 0.002028 | 5.098322 | 1.419105 |
| ITSN1 | 0.020637 | 0.002034 | 1.633665 | 3.742197 |
| ZXDA | 0.020732 | 0.002044 | 2.170733 | 3.432177 |
| PEAK1 | 0.020877 | 0.00206 | 1.1938 | 7.566546 |
| ZNF124 | 0.020888 | 0.002063 | 2.461698 | 2.964621 |
| LOC38828[illegible] | 0.020956 | 0.002073 | -3.46408 | 1.23667 |
| MKRN3 | 0.020956 | 0.002073 | -3.24401 | 2.026761 |
| CASQ2 | 0.020988 | 0.002078 | 4.406593 | 2.742744 |
| LAMB2 | 0.021022 | 0.002084 | 1.311284 | 7.407436 |
| CEACAM1[illegible] | 0.021022 | 0.002084 | 7.148616 | 0.96835 |
| ACAP2 | 0.021119 | 0.002095 | -1.21736 | 7.988862 |
| LINC01730 | 0.02116 | 0.002101 | 3.760824 | 1.047357 |
| FRRS1 | 0.021207 | 0.002107 | -2.38656 | 5.035436 |

| | | | | |
|---|---|---|---|---|
| WNT5A | 0.02135 | 0.002125 | -2.16302 | 3.972131 |
| ARHGEF17 | 0.02135 | 0.002127 | 1.437999 | 6.044256 |
| WNT7B | 0.02135 | 0.002127 | -2.84399 | 4.45681 |
| LRCH1 | 0.021502 | 0.002144 | 1.63838 | 4.721452 |
| LINC02078 | 0.021502 | 0.002148 | 4.760489 | 1.06267 |
| MYCBP2 | 0.021502 | 0.002148 | -1.10043 | 9.07738 |
| ST6GALNA( | 0.021502 | 0.00215 | -1.56176 | 4.609387 |
| ZNF37BP | 0.021502 | 0.00215 | 1.356421 | 6.319439 |
| LINC00941 | 0.021548 | 0.002157 | 3.810687 | 1.732385 |
| SRPX2 | 0.021548 | 0.002158 | -1.23072 | 5.733007 |
| MUC16 | 0.021575 | 0.002162 | 3.302413 | 6.068794 |
| IGFBP1 | 0.021606 | 0.002167 | 7.450447 | 1.273762 |
| FREM1 | 0.021726 | 0.002181 | 2.304626 | 2.58003 |
| DENND3 | 0.021865 | 0.002196 | 1.467247 | 5.908137 |
| LINC02544 | 0.021992 | 0.002211 | 3.848305 | 1.688673 |
| TLR1 | 0.022055 | 0.002219 | 2.216218 | 2.662659 |
| AHR | 0.022077 | 0.002223 | 1.375128 | 7.968379 |
| MAF | 0.022082 | 0.002225 | -1.4635 | 5.882624 |
| HOXB9 | 0.022082 | 0.002226 | 4.160086 | 3.891752 |
| GBE1 | 0.022172 | 0.002237 | -1.16938 | 6.050766 |
| PGAM2 | 0.022174 | 0.002239 | -1.67207 | 4.722472 |
| LGALS9C | 0.02221 | 0.002245 | -3.00873 | 2.292824 |
| OR7E91P | 0.02221 | 0.002246 | -3.35474 | 1.833739 |
| STAMBPL1 | 0.022228 | 0.00225 | 2.12144 | 4.706276 |
| CCDC82 | 0.02244 | 0.002274 | 1.176372 | 6.159954 |
| TMEM176/ | 0.02244 | 0.002274 | 2.830519 | 4.163086 |
| TBX4 | 0.02246 | 0.002278 | 6.627864 | 0.622027 |
| ULBP2 | 0.022519 | 0.002286 | -2.77006 | 3.159239 |
| KLHL6 | 0.022548 | 0.002292 | 2.29659 | 4.896909 |
| BDKRB1 | 0.022548 | 0.002292 | -2.92837 | 2.218905 |
| UNC13A | 0.022621 | 0.002301 | 2.544925 | 3.033346 |
| CALD1 | 0.022669 | 0.002309 | 2.034485 | 8.26131 |
| PCDHB18P | 0.022669 | 0.002311 | 3.179541 | 2.094615 |
| ADAMTS12 | 0.022669 | 0.002311 | 1.829161 | 4.327374 |
| SNORD67 | 0.022767 | 0.002323 | 1.729674 | 5.828309 |
| ACAA2 | 0.022831 | 0.002334 | 1.312263 | 5.745495 |
| EPHA2 | 0.022831 | 0.002335 | -1.58243 | 8.650687 |
| TCN1 | 0.022831 | 0.002336 | -2.77047 | 5.435611 |
| NBEA | 0.022831 | 0.002337 | 1.988089 | 5.760193 |
| MALRD1 | 0.022971 | 0.002353 | 5.160985 | 2.645944 |
| CCDC190 | 0.023182 | 0.002376 | -3.26498 | 2.480616 |
| CDC42BPA | 0.023225 | 0.002383 | 2.160866 | 3.552718 |
| HS6ST1 | 0.023225 | 0.002384 | -1.2147 | 5.474762 |
| KLHDC8B | 0.023335 | 0.002397 | -2.02431 | 4.02042 |
| RNF187 | 0.023422 | 0.002409 | -1.19437 | 6.424881 |

| | | | | |
|---|---|---|---|---|
| RAPGEF5 | 0.023422 | 0.00241 | -1.22136 | 7.106531 |
| LINC00327 | 0.023468 | 0.002416 | 4.730333 | 1.02137 |
| HUNK | 0.023518 | 0.002423 | 2.901857 | 3.880929 |
| ARHGEF38- | 0.023626 | 0.002436 | 3.76099 | 1.891946 |
| SLAIN2 | 0.023836 | 0.002459 | -1.09243 | 7.243068 |
| SGPP2 | 0.024032 | 0.002482 | -1.24904 | 5.738429 |
| SLC6A4 | 0.024385 | 0.00252 | -1.9724 | 2.755521 |
| SACS-AS1 | 0.024498 | 0.002533 | 5.809186 | 0.988771 |
| XDH | 0.024526 | 0.002538 | -2.46955 | 6.449265 |
| SELENOM | 0.024824 | 0.002571 | 2.755994 | 4.53618 |
| ATP5PO | 0.02484 | 0.002576 | -1.15752 | 6.26131 |
| PLEK | 0.02484 | 0.002576 | 2.341516 | 4.160686 |
| THSD4 | 0.025045 | 0.0026 | -1.5401 | 4.464337 |
| ZNF462 | 0.025085 | 0.002606 | -1.46554 | 7.236539 |
| IRS2 | 0.0251 | 0.002609 | 1.738428 | 6.519225 |
| POFUT1 | 0.025247 | 0.002626 | 1.089632 | 6.040011 |
| LRRK2 | 0.025319 | 0.002636 | -1.48099 | 6.670285 |
| PDK4 | 0.025396 | 0.002646 | 3.50848 | 10.2551 |
| NAP1L3 | 0.025396 | 0.002648 | 3.925363 | 1.846878 |
| STYK1 | 0.025518 | 0.002662 | -1.82594 | 4.728677 |
| JAKMIP3 | 0.025575 | 0.00267 | 2.571696 | 3.069918 |
| UTP14C | 0.025662 | 0.002681 | 1.078273 | 5.947275 |
| ERLIN1 | 0.025662 | 0.002683 | -5.26125 | 1.023274 |
| TBX15 | 0.02576 | 0.002695 | 4.005634 | 1.841317 |
| CSNK1G1 | 0.025775 | 0.0027 | -1.13209 | 6.560347 |
| ADAM20 | 0.025775 | 0.002701 | -2.0249 | 3.04309 |
| FAM126A | 0.025867 | 0.002712 | -1.2002 | 6.377763 |
| DRD1 | 0.02589 | 0.002717 | 4.695591 | 1.628003 |
| TMPRSS13 | 0.025896 | 0.002719 | -3.34652 | 2.344715 |
| DNAJC5 | 0.026192 | 0.002752 | -1.10564 | 7.752431 |
| C2CD4D-AS | 0.026277 | 0.002763 | 2.251998 | 2.956726 |
| EGLN3 | 0.02633 | 0.002771 | -2.50358 | 3.827913 |
| PRRT2 | 0.026364 | 0.002777 | 3.202744 | 2.317387 |
| STARD3NL | 0.026398 | 0.002782 | 1.417631 | 4.202805 |
| ARG2 | 0.026556 | 0.002801 | -1.65256 | 4.739239 |
| CPTP | 0.026574 | 0.002806 | -1.23235 | 4.735503 |
| OBSCN | 0.026574 | 0.002807 | -1.867 | 4.500374 |
| PPP2CA | 0.026604 | 0.002814 | -1.32967 | 7.230574 |
| GNS | 0.026604 | 0.002814 | 1.34815 | 7.03656 |
| PPP1R13L | 0.026694 | 0.002826 | -5.20739 | 1.871892 |
| ANLN | 0.026759 | 0.002835 | 4.129997 | 0.556664 |
| DUXAP9 | 0.026829 | 0.002844 | 3.163258 | 4.708887 |
| LLGL1 | 0.026859 | 0.002849 | -1.38845 | 4.657445 |
| ADAM21 | 0.026874 | 0.002853 | -1.93944 | 3.000039 |
| NBPF15 | 0.026887 | 0.002856 | 2.740625 | 2.507696 |

| | | | | |
|---|---|---|---|---|
| LOC72789€ | 0.027058 | 0.002877 | 2.474264 | 4.964581 |
| C7 | 0.027124 | 0.002886 | 3.688183 | 6.865474 |
| FLG2 | 0.027204 | 0.002896 | -2.17021 | 2.89477 |
| DPEP2 | 0.027482 | 0.002928 | 2.759264 | 2.019491 |
| TRIM16L | 0.027521 | 0.002936 | -1.79958 | 4.080964 |
| PODXL2 | 0.027521 | 0.002936 | 2.050799 | 4.527815 |
| HERC2P3 | 0.027521 | 0.002938 | 1.299053 | 4.778848 |
| LRRC37A4F | 0.027528 | 0.002942 | -1.13766 | 7.004803 |
| RNF186 | 0.027528 | 0.002943 | 7.867823 | 1.587648 |
| PRSS22 | 0.027644 | 0.002958 | -1.61838 | 6.50941 |
| CRTAC1 | 0.027674 | 0.002963 | -4.10723 | 0.91684 |
| SPRR4 | 0.027706 | 0.002969 | -3.77531 | 2.174329 |
| ACE | 0.02773 | 0.002973 | 2.25733 | 2.81563 |
| LINC01206 | 0.027753 | 0.002978 | -3.70993 | 2.569815 |
| PIP4P2 | 0.027836 | 0.002991 | 2.227649 | 3.303932 |
| C4BPA | 0.027836 | 0.002991 | 4.281839 | 1.450848 |
| LOC101928 | 0.02807 | 0.003018 | -3.64435 | 2.715469 |
| SNORD105 | 0.028103 | 0.003024 | 2.134973 | 4.651175 |
| ABCA5 | 0.02816 | 0.003032 | -1.77184 | 3.823527 |
| S100A12 | 0.028228 | 0.003042 | -3.0915 | 4.507703 |
| PKN2 | 0.028261 | 0.003049 | -1.10104 | 7.844957 |
| RYR2 | 0.028261 | 0.00305 | 2.498797 | 5.619883 |
| TTC21B | 0.028336 | 0.00306 | 1.265591 | 5.704031 |
| PITPNA | 0.028346 | 0.003063 | -1.08796 | 7.198709 |
| MIR3142H( | 0.028576 | 0.00309 | 4.024665 | 2.093159 |
| PXYLP1 | 0.028615 | 0.003097 | 5.395638 | 0.678646 |
| SERINC5 | 0.028683 | 0.003107 | -1.12163 | 7.697799 |
| P4HB | 0.028683 | 0.003108 | -1.16238 | 9.728091 |
| NDC80 | 0.028756 | 0.003121 | 1.967321 | 5.143109 |
| ABI3BP | 0.028756 | 0.003121 | 1.675328 | 5.786462 |
| FLOT1 | 0.028756 | 0.003123 | -2.33961 | 3.163812 |
| KRTAP10-9 | 0.028764 | 0.003127 | 7.225658 | 1.094685 |
| TPRG1L | 0.028764 | 0.003128 | -1.35563 | 5.916327 |
| GNMT | 0.028778 | 0.003132 | -4.61929 | 2.520073 |
| FAM157B | 0.028829 | 0.003139 | -2.91558 | 3.0627 |
| SLC1A1 | 0.028841 | 0.003143 | 2.033098 | 5.634772 |
| PRDX2 | 0.029069 | 0.00317 | -1.08847 | 6.087344 |
| PLEKHA3 | 0.029131 | 0.003179 | 1.471236 | 4.609919 |
| GLP2R | 0.029182 | 0.003187 | 4.151013 | 1.065113 |
| USH1C | 0.029256 | 0.003197 | 3.505105 | 3.630444 |
| SLC25A25-/ | 0.029304 | 0.003205 | 1.529157 | 5.182694 |
| TLR3 | 0.029362 | 0.003213 | -2.25687 | 3.661088 |
| MMP10 | 0.029381 | 0.003217 | 4.555489 | 1.833612 |
| TNFRSF10L | 0.029498 | 0.003233 | 1.902184 | 4.573982 |
| DKK4 | 0.029772 | 0.003267 | -4.46453 | 1.739304 |

| | | | | |
|---|---|---|---|---|
| PIP4K2A | 0.029772 | 0.003269 | 1.202314 | 5.779886 |
| MUC12 | 0.029772 | 0.003269 | 3.217529 | 4.428758 |
| GSTM5 | 0.029785 | 0.003273 | 3.397707 | 1.07112 |
| UBAP1 | 0.03003 | 0.003302 | -1.77082 | 3.426083 |
| GSTA1 | 0.030129 | 0.003316 | -4.00444 | 3.029049 |
| TGIF1 | 0.030191 | 0.003325 | 1.877879 | 3.131939 |
| LINC01209 | 0.030285 | 0.003337 | 6.981592 | 0.885462 |
| RPS27L | 0.030474 | 0.003361 | 1.344062 | 5.447464 |
| DSG2-AS1 | 0.030519 | 0.003368 | 4.311523 | 1.028337 |
| GNA13 | 0.030563 | 0.003375 | 1.487858 | 4.354064 |
| ACP7 | 0.030578 | 0.003379 | -3.56836 | 1.871197 |
| ABHD5 | 0.030578 | 0.003381 | -1.12446 | 6.254315 |
| SPON1 | 0.030624 | 0.003389 | 1.599844 | 6.366599 |
| NRROS | 0.030671 | 0.003396 | 2.833572 | 1.776208 |
| HMCN2 | 0.030693 | 0.003401 | 2.323476 | 5.59946 |
| CCDC57 | 0.03075 | 0.00341 | 1.235327 | 5.480185 |
| TF | 0.030926 | 0.003431 | -2.57454 | 3.514238 |
| TMEM79 | 0.031068 | 0.00345 | -2.52754 | 2.444678 |
| HNF4G | 0.031077 | 0.003456 | 2.462444 | 4.498595 |
| SH3BGRL2 | 0.031077 | 0.003458 | -1.25048 | 8.251251 |
| PGM5P2 | 0.031077 | 0.003459 | 2.070501 | 4.055292 |
| ANPEP | 0.031077 | 0.003461 | 2.345131 | 4.387677 |
| FOXQ1 | 0.031077 | 0.003465 | 1.534627 | 6.189289 |
| MIR145 | 0.031077 | 0.003465 | 3.411841 | 3.106499 |
| DNM1P41 | 0.031077 | 0.003467 | -1.82925 | 2.577912 |
| UVRAG | 0.031132 | 0.003476 | 1.195303 | 5.525236 |
| ITGA9 | 0.031173 | 0.003483 | 1.857784 | 4.744881 |
| STRIP2 | 0.031177 | 0.003486 | 3.709515 | 3.14283 |
| CGN | 0.031218 | 0.003493 | 1.461111 | 8.147408 |
| LOC10013( | 0.031302 | 0.003504 | 5.473722 | 0.746823 |
| CYGB | 0.031382 | 0.003516 | 2.172686 | 3.493867 |
| KRTAP10-1 | 0.03143 | 0.003523 | 7.551782 | 1.36931 |
| PRTG | 0.031534 | 0.003537 | 2.423538 | 2.655517 |
| SERPINH1 | 0.031541 | 0.003541 | 6.270083 | 0.343212 |
| KRT5 | 0.031638 | 0.003554 | -5.08364 | 12.26658 |
| MORC3 | 0.031718 | 0.003565 | -1.18889 | 7.150196 |
| CEACAM1 | 0.031781 | 0.003575 | -3.07882 | 2.914652 |
| FGFR2 | 0.031858 | 0.003586 | 4.806876 | 1.72289 |
| C5orf67 | 0.03205 | 0.003611 | 6.198451 | 0.297847 |
| TMEM95 | 0.03205 | 0.003612 | -3.71971 | 1.529057 |
| TMCC1-AS: | 0.032269 | 0.003639 | 3.272808 | 1.302181 |
| DDX60 | 0.032985 | 0.003723 | 2.378691 | 6.240796 |
| ATP5PB | 0.033002 | 0.003727 | -1.05746 | 6.742219 |
| CDSN | 0.033096 | 0.003742 | 4.523544 | 2.158997 |
| LRP11 | 0.033096 | 0.003748 | -1.25716 | 6.811211 |

| | | | | |
|---|---|---|---|---|
| ZNF426 | 0.033096 | 0.003749 | -3.04325 | 1.441217 |
| ABO | 0.033096 | 0.00375 | -1.51661 | 6.144789 |
| SLC15A1 | 0.033096 | 0.00375 | -2.54903 | 3.043029 |
| LOC10013( | 0.033147 | 0.003759 | -1.6413 | 4.543129 |
| SDC2 | 0.033282 | 0.003777 | 1.830595 | 4.836794 |
| FBXO42 | 0.033285 | 0.003779 | -1.24745 | 5.837109 |
| APMAP | 0.033403 | 0.003798 | 1.030772 | 6.054681 |
| NDRG4 | 0.033403 | 0.003799 | -2.56428 | 1.741902 |
| RNF114 | 0.033403 | 0.003801 | 1.084396 | 5.758819 |
| ZNF185 | 0.033468 | 0.003811 | -4.55276 | 0.748005 |
| DHRS3 | 0.033772 | 0.003849 | -1.32196 | 6.872607 |
| SPRR2B | 0.033772 | 0.00385 | -3.64928 | 1.527696 |
| LRP10 | 0.034022 | 0.003881 | -1.21341 | 7.895075 |
| TDRD9 | 0.034044 | 0.003886 | 3.954612 | 1.041453 |
| BMP8B | 0.034157 | 0.003902 | 2.753381 | 2.387347 |
| PAG1 | 0.03422 | 0.003912 | 1.51423 | 6.1835 |
| NAV2-AS5 | 0.034418 | 0.003937 | 3.879097 | 1.953765 |
| PARVA | 0.034427 | 0.003944 | 1.275086 | 6.855103 |
| GPR160 | 0.034427 | 0.003946 | 1.95387 | 4.737595 |
| WDR45B | 0.034427 | 0.003946 | -1.21387 | 7.285177 |
| ZFHX3 | 0.034428 | 0.003949 | 1.242282 | 5.392539 |
| CNGA1 | 0.034434 | 0.003952 | -6.35027 | 0.387436 |
| ZNF253 | 0.034439 | 0.003955 | 1.492503 | 4.411422 |
| KRT79 | 0.034651 | 0.003983 | -3.50775 | 1.416671 |
| LINC00885 | 0.034651 | 0.003985 | -3.25112 | 0.956576 |
| KRT16 | 0.034899 | 0.004016 | -3.84817 | 9.977097 |
| LINC00540 | 0.034906 | 0.004019 | 5.471017 | 1.685587 |
| KRT35 | 0.034937 | 0.004028 | -6.71007 | 0.64268 |
| RCSD1 | 0.034937 | 0.004029 | 1.578221 | 4.722068 |
| FGF19 | 0.034937 | 0.004031 | 6.410238 | 3.560863 |
| CACNA2D4 | 0.035086 | 0.004051 | 2.285018 | 3.04435 |
| CNTNAP1 | 0.035237 | 0.004073 | 1.632814 | 4.673241 |
| TMIE | 0.035237 | 0.004074 | -3.2812 | 1.603908 |
| PLBD1 | 0.035237 | 0.004076 | -1.3487 | 6.606194 |
| HGF | 0.035246 | 0.00408 | 4.026811 | 0.782184 |
| ENPP1 | 0.035294 | 0.004088 | 2.629921 | 3.837359 |
| SNORD116 | 0.035303 | 0.004092 | 2.093379 | 6.663396 |
| HSPA12A | 0.035334 | 0.004098 | 1.954138 | 4.380266 |
| STARD9 | 0.035372 | 0.004105 | 1.576788 | 6.176064 |
| MIR595 | 0.035491 | 0.004122 | 4.793286 | 0.789158 |
| SLC2A1 | 0.035724 | 0.004152 | -1.85887 | 8.713591 |
| ITM2B | 0.035745 | 0.004158 | -1.08466 | 8.922426 |
| CPXM2 | 0.035745 | 0.00416 | 2.015679 | 4.966 |
| RAB32 | 0.035763 | 0.004164 | 2.310079 | 2.875716 |
| RCN1 | 0.035791 | 0.00417 | 1.131882 | 6.411421 |

| | | | | |
|---|---|---|---|---|
| DNAJB6 | 0.035795 | 0.004174 | -1.2635 | 6.183694 |
| MAPK6 | 0.035838 | 0.004181 | -1.37866 | 8.044231 |
| SCUBE1 | 0.035992 | 0.004203 | 3.379582 | 4.133841 |
| FLVCR1 | 0.035992 | 0.004205 | 1.244117 | 4.980034 |
| PLS1 | 0.03607 | 0.004217 | 2.490237 | 2.277236 |
| TEP1 | 0.036138 | 0.004227 | 1.076396 | 6.923373 |
| IKBIP | 0.036159 | 0.004233 | 2.81944 | 2.51997 |
| TPM4 | 0.0363 | 0.004252 | -1.20852 | 7.030493 |
| ANKMY2 | 0.0363 | 0.004255 | 1.2692 | 5.026727 |
| MARCKS | 0.036357 | 0.004264 | -1.19354 | 8.318678 |
| LOC10013( | 0.036384 | 0.00427 | -1.87477 | 3.659954 |
| LINC01260 | 0.036425 | 0.004278 | 5.444547 | 0.726876 |
| PLA2G4E-A | 0.036461 | 0.004285 | -4.25517 | 3.278888 |
| C5AR1 | 0.036511 | 0.004293 | 2.028734 | 4.275823 |
| WNT11 | 0.036515 | 0.004297 | 5.254985 | 5.822572 |
| KRTAP10-3 | 0.036534 | 0.004302 | 6.473267 | 0.505194 |
| BMP8A | 0.036563 | 0.004308 | 1.525541 | 4.440478 |
| OSR1 | 0.036744 | 0.004334 | 2.420905 | 3.103185 |
| SFRP2 | 0.036744 | 0.004337 | 1.622361 | 6.086003 |
| FBLN1 | 0.036744 | 0.004338 | 1.504104 | 5.917319 |
| EPB41L4A- | 0.036841 | 0.004352 | -3.01264 | 1.602085 |
| PDLIM7 | 0.036901 | 0.004362 | 2.317169 | 3.248755 |
| C22orf46 | 0.037043 | 0.004381 | 1.29125 | 6.132864 |
| FOSL2 | 0.037455 | 0.004433 | -1.22835 | 9.567267 |
| SPRR2C | 0.037567 | 0.004451 | -4.21409 | 4.428077 |
| NMRK1 | 0.037567 | 0.004452 | -2.85058 | 1.48034 |
| KLF5 | 0.037735 | 0.004475 | -1.56622 | 6.669942 |
| ARRDC3 | 0.038064 | 0.004516 | -1.21592 | 8.098386 |
| TRPM8 | 0.038148 | 0.00453 | 3.792889 | 0.626874 |
| CDC42BPG | 0.038148 | 0.004532 | -1.44727 | 6.645404 |
| GAS2L2 | 0.038148 | 0.004535 | 3.605514 | 1.11234 |
| MAST3 | 0.038198 | 0.004545 | 1.216237 | 4.909783 |
| PRB2 | 0.038198 | 0.004547 | 4.34332 | 1.50554 |
| GALM | 0.038288 | 0.004561 | 1.633442 | 3.876053 |
| LRP6 | 0.03843 | 0.00458 | 1.098302 | 7.162093 |
| AFF4 | 0.038593 | 0.004603 | -1.0777 | 9.039852 |
| ZNF32 | 0.038598 | 0.004606 | 4.415204 | 1.15709 |
| ABCD3 | 0.0386 | 0.00461 | -1.05005 | 6.441675 |
| GOLGA6L3 | 0.0386 | 0.004613 | -3.14363 | 1.070959 |
| SCARNA17 | 0.038656 | 0.004622 | 1.216426 | 7.486638 |
| DOCK11 | 0.038762 | 0.004638 | 1.240179 | 5.751953 |
| RGS20 | 0.038837 | 0.00465 | -4.45166 | 1.348332 |
| CHP1 | 0.038855 | 0.004655 | -1.22977 | 7.879852 |
| LINC01790 | 0.038855 | 0.004658 | 6.184098 | 0.280208 |
| RPS6 | 0.0389 | 0.004666 | -1.07454 | 10.22641 |

| | | | | |
|---|---|---|---|---|
| CACNA2D1 | 0.038965 | 0.004677 | 1.619152 | 4.740113 |
| LOC10050 | 0.038979 | 0.004682 | 5.871749 | 1.048832 |
| C2orf72 | 0.03909 | 0.004699 | 3.183919 | 3.897523 |
| SGTB | 0.03909 | 0.004701 | 1.490855 | 4.069421 |
| EPB41L2 | 0.039179 | 0.004715 | 1.735213 | 3.300404 |
| ARNTL2-AS | 0.039394 | 0.004746 | -2.37201 | 2.90073 |
| XCL1 | 0.039394 | 0.004746 | 6.371082 | 0.40452 |
| LILRA6 | 0.039405 | 0.004754 | 4.535206 | 0.901529 |
| SORT1 | 0.039405 | 0.004755 | -1.75891 | 2.695251 |
| CYP4V2 | 0.039405 | 0.004757 | 1.551831 | 4.914263 |
| GRK5 | 0.039507 | 0.004772 | 1.296277 | 5.671947 |
| RNF152 | 0.039646 | 0.004792 | -1.49182 | 4.062824 |
| CPQ | 0.039666 | 0.004797 | 1.783856 | 4.507031 |
| PGAM1 | 0.039724 | 0.004808 | -1.87441 | 3.455076 |
| PGA5 | 0.039724 | 0.00481 | -8.42065 | 9.619805 |
| GALNT12 | 0.039769 | 0.004819 | -1.24217 | 6.138252 |
| FAM83E | 0.039947 | 0.004848 | 1.693955 | 4.052285 |
| SEMA4D | 0.039947 | 0.004849 | -1.09703 | 6.293495 |
| CHRNA5 | 0.039947 | 0.004852 | 3.438853 | 0.915954 |
| MROH2B | 0.039947 | 0.004853 | 3.867532 | 1.715093 |
| ANKRD44-I | 0.039949 | 0.004856 | 1.598875 | 3.565833 |
| SOX8 | 0.039992 | 0.004864 | 4.221985 | 1.740618 |
| RN7SL1 | 0.040048 | 0.004874 | -1.23475 | 14.74922 |
| ZNF620 | 0.040308 | 0.004909 | 7.738646 | 1.521643 |
| ID3 | 0.04032 | 0.004913 | 1.922894 | 3.784367 |
| CYP21A1P | 0.040377 | 0.004926 | 4.712685 | 0.676136 |
| DSCC1 | 0.040377 | 0.004926 | 1.68458 | 3.198854 |
| WFDC2 | 0.040642 | 0.004962 | 4.225152 | 1.751671 |
| MSTO2P | 0.040785 | 0.004982 | 2.386992 | 2.352418 |
| STAB1 | 0.040841 | 0.004992 | 1.488919 | 6.709619 |
| SCAMP2 | 0.040951 | 0.00501 | -1.18238 | 6.22462 |
| FADS3 | 0.040951 | 0.005013 | 1.775544 | 3.734804 |
| ACTC1 | 0.040951 | 0.005015 | 4.409492 | 2.35836 |
| CDH17 | 0.041047 | 0.00503 | 6.641283 | 0.574239 |
| LRRC18 | 0.041058 | 0.005034 | 3.917662 | 1.123206 |
| TSPAN13 | 0.041077 | 0.00504 | 1.477414 | 6.019828 |
| PEBP1 | 0.041242 | 0.005063 | -1.04971 | 7.056559 |
| HELB | 0.041312 | 0.005075 | 1.91407 | 4.679126 |
| DSCAS | 0.041372 | 0.005086 | -2.76693 | 1.689861 |
| SLC16A13 | 0.041481 | 0.005102 | -1.69999 | 3.185975 |
| SCPEP1 | 0.041505 | 0.005108 | -1.13909 | 5.877548 |
| PPP1R14C | 0.041505 | 0.005111 | -2.61222 | 5.065288 |
| SLC6A1 | 0.04153 | 0.00512 | -3.07564 | 2.672751 |
| CLMP | 0.04153 | 0.005121 | 1.367583 | 5.378809 |
| EPHB1 | 0.041575 | 0.00513 | 3.184946 | 2.387675 |

| | | | | |
|---|---|---|---|---|
| BNC2 | 0.041585 | 0.005134 | 3.707449 | 1.436282 |
| TGFBR2 | 0.041782 | 0.005162 | 3.298902 | 0.959388 |
| KCMF1 | 0.041782 | 0.005167 | -1.09555 | 7.064382 |
| DNAJC15 | 0.041782 | 0.005168 | 1.503206 | 5.577824 |
| SYNDIG1 | 0.04187 | 0.005183 | 3.107901 | 0.985917 |
| BTNL3 | 0.04187 | 0.005185 | 4.608591 | 2.519068 |
| MMP13 | 0.041917 | 0.005194 | 4.434083 | 3.194762 |
| RELB | 0.041976 | 0.005205 | 1.407436 | 4.179179 |
| TET2 | 0.042064 | 0.005219 | -1.04839 | 7.126301 |
| MLH3 | 0.042173 | 0.005235 | 4.302049 | 1.473709 |
| LMAN2 | 0.042182 | 0.005242 | -1.1208 | 7.037577 |
| ZMAT1 | 0.042182 | 0.005246 | 5.172666 | 0.48841 |
| FLJ46906 | 0.042182 | 0.005247 | -1.94028 | 3.441824 |
| PLEKHA7 | 0.042182 | 0.00525 | -1.11281 | 7.705818 |
| USP50 | 0.042326 | 0.005271 | 4.474045 | 1.441601 |
| PDLIM3 | 0.042398 | 0.005283 | 2.518866 | 2.448601 |
| MRPS7 | 0.042679 | 0.005321 | -1.16081 | 5.375447 |
| STRBP | 0.042679 | 0.005328 | -2.69493 | 2.164133 |
| WHAMMP: | 0.042679 | 0.005332 | 2.009937 | 2.966847 |
| COMP | 0.042679 | 0.005334 | 2.926655 | 3.108204 |
| RPL5 | 0.042679 | 0.005337 | -1.05454 | 9.254454 |
| SH3RF3 | 0.042679 | 0.005337 | 1.482602 | 4.21932 |
| DOCK2 | 0.042717 | 0.005345 | 1.908762 | 5.468652 |
| SLFN13 | 0.042814 | 0.005361 | 1.363933 | 5.380763 |
| SNORD89 | 0.042826 | 0.00537 | 1.690826 | 8.673737 |
| GALNT1 | 0.042826 | 0.005374 | -1.29879 | 7.911046 |
| MNS1 | 0.042826 | 0.005375 | 1.219816 | 4.461664 |
| SNORD116 | 0.042826 | 0.005375 | 2.185538 | 7.154529 |
| SPINK4 | 0.042836 | 0.00538 | 4.999638 | 2.457604 |
| RILP | 0.04285 | 0.005385 | 3.899378 | 1.121783 |
| PRKAR2B | 0.042973 | 0.005404 | 1.908202 | 4.201502 |
| KRT16P2 | 0.042973 | 0.005408 | -3.57207 | 3.615781 |
| SLC27A1 | 0.042973 | 0.00541 | 1.392242 | 5.07166 |
| ZDHHC18 | 0.043015 | 0.005419 | -1.24272 | 5.706167 |
| LOXL1 | 0.043024 | 0.005425 | 2.041914 | 4.033063 |
| LINC02532 | 0.043024 | 0.005426 | 4.708709 | 1.09223 |
| MTF1 | 0.043566 | 0.005498 | -1.10433 | 6.713578 |
| EPHA1-AS1 | 0.043732 | 0.005525 | -1.67094 | 4.062732 |
| BMP2K | 0.043732 | 0.005526 | -1.19038 | 5.260641 |
| ZNF654 | 0.043746 | 0.005531 | -1.10735 | 6.110609 |
| LINC01169 | 0.043967 | 0.005562 | 6.39465 | 0.436879 |
| RNF215 | 0.044363 | 0.005616 | 1.676994 | 3.147251 |
| TIPARP | 0.044364 | 0.005619 | -4.48202 | 1.234945 |
| KIF7 | 0.044405 | 0.005628 | 1.513833 | 4.123151 |
| DHRS11 | 0.044432 | 0.005634 | -4.32666 | 0.690311 |

| | | | | |
|---|---|---|---|---|
| CP | 0.044764 | 0.00568 | 5.447775 | 0.719073 |
| PNMA2 | 0.04492 | 0.005703 | 2.728945 | 3.731422 |
| STX19 | 0.04509 | 0.005728 | -1.8918 | 3.898534 |
| IFT74 | 0.045488 | 0.005782 | -6.58536 | 0.545459 |
| SYNM | 0.045516 | 0.005789 | 3.024897 | 4.788244 |
| LINC01614 | 0.045534 | 0.005795 | 4.311196 | 0.402328 |
| PLP2 | 0.045627 | 0.00581 | -1.5405 | 6.701658 |
| ABCA6 | 0.045758 | 0.00583 | 2.182986 | 5.348955 |
| LOC101928 | 0.046041 | 0.00587 | -1.53677 | 4.116807 |
| ALDH3A2 | 0.046136 | 0.005886 | -3.19731 | 1.622024 |
| PREX1 | 0.046347 | 0.005919 | 1.39227 | 6.076242 |
| NLRP1 | 0.046347 | 0.005922 | -1.68315 | 2.893458 |
| FAM102A | 0.046347 | 0.005923 | -1.1594 | 5.438314 |
| N4BP1 | 0.046674 | 0.005971 | -1.04446 | 7.359819 |
| NCAM2 | 0.046674 | 0.005972 | 3.092628 | 2.404156 |
| TNFRSF14-. | 0.04685 | 0.006 | 1.512953 | 4.151164 |
| GPRIN1 | 0.04685 | 0.006004 | 2.408785 | 3.364536 |
| DESI1 | 0.04685 | 0.006005 | -1.05834 | 6.622444 |
| SNORD114 | 0.046875 | 0.006012 | 2.67759 | 2.63946 |
| SIPA1L2 | 0.046887 | 0.006017 | -1.23157 | 7.567907 |
| LOC103021 | 0.046902 | 0.006023 | -1.56246 | 3.59894 |
| LPL | 0.046906 | 0.006027 | 2.712529 | 2.820547 |
| SNORA11 | 0.046952 | 0.006036 | 1.841083 | 7.192732 |
| AMZ2P1 | 0.046978 | 0.006047 | 1.752517 | 3.681804 |
| CXCL9 | 0.046978 | 0.006049 | 2.225033 | 3.0545 |
| LINC00589 | 0.046978 | 0.00605 | 4.321843 | 0.406565 |
| RAC2 | 0.047006 | 0.006058 | 1.559215 | 3.820385 |
| CACNA1C-/ | 0.047009 | 0.006062 | 4.1494 | 0.702107 |
| LOC105375 | 0.04703 | 0.006068 | 4.42331 | 0.829048 |
| ALS2CL | 0.047129 | 0.006084 | -1.9928 | 3.619271 |
| HOPX | 0.047162 | 0.006092 | -3.7074 | 1.349018 |
| RSPH3 | 0.04726 | 0.006108 | -1.13639 | 5.534183 |
| SIGLEC14 | 0.047339 | 0.006122 | 3.059659 | 0.784721 |
| GYS2 | 0.048193 | 0.006236 | -3.02198 | 3.419772 |
| OTUD3 | 0.048551 | 0.006291 | 1.315575 | 5.803961 |
| HSPA2 | 0.048551 | 0.006292 | -1.54831 | 5.670955 |
| KCNG1 | 0.048551 | 0.006294 | -2.60302 | 1.723186 |
| PTPRJ | 0.048595 | 0.006303 | 1.751181 | 6.49944 |
| TMEM248 | 0.048823 | 0.006336 | 1.003813 | 7.182675 |
| PCSK7 | 0.048959 | 0.006358 | 1.054511 | 7.19093 |
| MAP2K7 | 0.049043 | 0.006372 | 3.843719 | 0.415415 |
| LIF | 0.049061 | 0.006379 | 3.70064 | 1.443551 |
| FUCA2 | 0.049061 | 0.006382 | 1.251128 | 5.325136 |
| ADAMTS10 | 0.049152 | 0.0064 | 2.346991 | 3.081552 |
| THBS2 | 0.049152 | 0.006401 | 1.491412 | 7.620289 |

| | | | | |
|---|---|---|---|---|
| MROH8 | 0.049521 | 0.006453 | -3.69791 | 1.332902 |
| RNF213 | 0.049569 | 0.006463 | 1.126363 | 9.526061 |
| STAT1 | 0.049591 | 0.00647 | 1.148125 | 6.191142 |
| PELI1 | 0.049645 | 0.006481 | -1.14576 | 7.953855 |
| TCF3 | 0.049773 | 0.006501 | 2.162382 | 2.966125 |
| SYDE2 | 0.050051 | 0.006542 | 2.083493 | 4.167721 |
| VAT1 | 0.050123 | 0.006555 | -1.10412 | 6.931023 |
| SNORD100 | 0.050161 | 0.006564 | 1.888503 | 3.62601 |
| FGF18 | 0.050184 | 0.00657 | 3.6105 | 2.136618 |
| TOX3 | 0.050263 | 0.006585 | 3.625631 | 1.888796 |
| POLR3G | 0.050397 | 0.006606 | -1.69696 | 3.984972 |
| LOC100505 | 0.050434 | 0.006615 | 4.955515 | 1.310086 |
| SAP30L-AS | 0.05053 | 0.006631 | 1.950907 | 3.555757 |
| MANBA | 0.050595 | 0.006643 | 1.191166 | 5.499289 |
| GAL3ST2 | 0.050755 | 0.006668 | 5.291133 | 0.611809 |
| KRT7 | 0.050862 | 0.006688 | 2.279365 | 7.341176 |
| POC5 | 0.050862 | 0.006694 | 2.547319 | 1.780076 |
| FNDC4 | 0.050862 | 0.006695 | -1.48267 | 4.239228 |
| ATM | 0.050862 | 0.006698 | 1.104582 | 7.597236 |
| PRSS41 | 0.050881 | 0.006704 | 6.947068 | 0.864579 |
| LETM1 | 0.051 | 0.006724 | -1.01788 | 7.177321 |
| LAG3 | 0.051109 | 0.006744 | 2.808982 | 1.688182 |
| GOLGA8A | 0.051109 | 0.006746 | -1.21966 | 5.289929 |
| IFI44 | 0.051152 | 0.006756 | 2.225464 | 5.397687 |
| CST7 | 0.051211 | 0.006767 | 2.770697 | 3.212599 |
| CDCP1 | 0.051214 | 0.006772 | -1.2939 | 7.237593 |
| GIMAP4 | 0.051258 | 0.006784 | 1.462399 | 4.78781 |
| CCKAR | 0.051258 | 0.006785 | -5.38258 | 3.008309 |
| LINC00365 | 0.051268 | 0.00679 | 4.102459 | 2.385242 |
| ACTR10 | 0.05159 | 0.006837 | -1.03519 | 5.764829 |
| NMUR2 | 0.051638 | 0.006847 | 4.269653 | 0.512852 |
| HOXB7 | 0.052222 | 0.006929 | 2.302146 | 4.724537 |
| ZNF263 | 0.052279 | 0.00694 | 1.139448 | 5.699677 |
| IDNK | 0.052283 | 0.006945 | 2.762263 | 1.360346 |
| ABHD4 | 0.052399 | 0.006964 | -1.25839 | 5.419455 |
| TRAF5 | 0.052568 | 0.006991 | 3.933272 | 0.507986 |
| INSYN2B | 0.052741 | 0.007021 | 2.658003 | 2.664142 |
| ZNF70 | 0.052741 | 0.007025 | 1.305355 | 4.223379 |
| VILL | 0.052741 | 0.007026 | 1.381432 | 4.872087 |
| LINC00538 | 0.052787 | 0.007036 | 6.921886 | 0.849683 |
| CD248 | 0.052805 | 0.007042 | 1.604529 | 4.74576 |
| ZBTB47 | 0.05288 | 0.007056 | 1.224828 | 5.501067 |
| TNXB | 0.053077 | 0.007087 | 2.111009 | 3.74264 |
| UNC13C | 0.053741 | 0.007179 | 4.33842 | 0.700974 |
| DRAP1 | 0.053788 | 0.007192 | -1.17302 | 6.253836 |

| | | | | |
|---|---|---|---|---|
| GRIN2B | 0.053788 | 0.007194 | 4.061695 | 2.081001 |
| GAS6 | 0.053801 | 0.0072 | 1.402935 | 6.728166 |
| LOC101928 | 0.054371 | 0.00728 | 4.11431 | 1.290053 |
| SPTB | 0.054455 | 0.007296 | -1.21467 | 4.648453 |
| LINC02538 | 0.054479 | 0.007303 | -3.2857 | 3.617065 |
| INTS6 | 0.0548 | 0.00735 | -1.20065 | 5.092592 |
| ANXA2P2 | 0.054852 | 0.007361 | -1.15118 | 7.838594 |
| LZTS3 | 0.054978 | 0.007387 | 2.057971 | 5.497649 |
| LOC101929 | 0.054978 | 0.007389 | -2.59697 | 2.473118 |
| ALX4 | 0.054978 | 0.007391 | 2.799101 | 0.880898 |
| IFIT5 | 0.055028 | 0.007404 | 1.266702 | 4.975222 |
| ABCA1 | 0.055028 | 0.007406 | 1.104849 | 7.196199 |
| ARFGEF3 | 0.055066 | 0.007415 | 1.690687 | 6.949459 |
| TPM1 | 0.055079 | 0.007421 | 3.149444 | 3.228497 |
| PRKAR1B | 0.055136 | 0.007433 | 3.429912 | 0.275839 |
| FYN | 0.055221 | 0.007449 | 1.527238 | 4.520957 |
| MIR4298 | 0.055242 | 0.00746 | 5.469692 | 0.627127 |
| CNKSR3 | 0.055242 | 0.007463 | -1.20238 | 6.489502 |
| CRTAP | 0.055242 | 0.007469 | 1.06255 | 7.268953 |
| TMCO3 | 0.055242 | 0.007473 | 1.123659 | 6.570515 |
| WDR90 | 0.055242 | 0.007476 | 1.123785 | 5.460026 |
| ZEB2 | 0.055242 | 0.007477 | 2.036734 | 4.92049 |
| PABPC3 | 0.05528 | 0.007486 | -1.78876 | 2.914154 |
| SMCHD1 | 0.055731 | 0.007551 | 1.339152 | 8.651611 |
| FHOD1 | 0.055756 | 0.007559 | 1.252922 | 4.733493 |
| SRGN | 0.055806 | 0.00757 | 3.553455 | 0.659033 |
| SMILR | 0.055905 | 0.007591 | -3.82416 | 2.380662 |
| PGA3 | 0.055905 | 0.007595 | -9.94517 | 11.53954 |
| TRPV2 | 0.055905 | 0.007599 | 1.702805 | 3.176823 |
| LOC101928 | 0.055905 | 0.007603 | 5.68152 | 0.902221 |
| OR13H1 | 0.055905 | 0.007605 | 6.966881 | 0.88872 |
| ANKRD20A | 0.056369 | 0.007672 | -1.88173 | 4.203214 |
| ULK4 | 0.05645 | 0.007688 | 1.14845 | 5.065706 |
| GLIS3 | 0.056458 | 0.007693 | 2.34276 | 2.750936 |
| UGGT2 | 0.056612 | 0.007718 | 1.157437 | 5.920976 |
| ASPRV1 | 0.056773 | 0.007746 | 3.289993 | 1.386652 |
| SLC22A23 | 0.056773 | 0.007753 | -1.75732 | 3.105955 |
| NPIPB11 | 0.056773 | 0.007759 | 1.647814 | 3.929312 |
| CKMT1B | 0.056773 | 0.007762 | -1.56109 | 3.684266 |
| LINC01429 | 0.056773 | 0.007762 | 3.257799 | 1.189911 |
| TXNIP | 0.056998 | 0.007797 | 1.680818 | 6.748343 |
| PRXL2C | 0.057038 | 0.007807 | 1.633954 | 3.537043 |
| MAP1LC3A | 0.057091 | 0.007818 | 2.397022 | 1.966914 |
| CD8B | 0.057178 | 0.007835 | 3.993833 | 0.740842 |
| CATIP-AS2 | 0.057182 | 0.00784 | 3.673553 | 0.806044 |

| | | | | |
|---|---|---|---|---|
| ARHGAP27 | 0.057182 | 0.007844 | -1.01192 | 5.595007 |
| TNFRSF10C | 0.057202 | 0.007853 | 2.62187 | 2.678157 |
| ASXL1 | 0.057202 | 0.007858 | 1.057911 | 7.005621 |
| ADRB2 | 0.057202 | 0.007862 | -2.11347 | 3.933889 |
| PRDM6 | 0.057202 | 0.007864 | 2.477476 | 1.792154 |
| CALHM5 | 0.057211 | 0.00787 | 2.894822 | 2.118333 |
| IQSEC1 | 0.057225 | 0.007876 | 1.172822 | 4.821805 |
| MADD | 0.057367 | 0.0079 | 3.980292 | 1.249295 |
| LPGAT1 | 0.057445 | 0.007915 | 1.082725 | 7.052335 |
| S100A10 | 0.057482 | 0.007926 | -1.46328 | 9.890132 |
| RHPN1 | 0.057482 | 0.007929 | 1.597461 | 4.091107 |
| LGALS3 | 0.057715 | 0.007968 | -1.4432 | 5.071813 |
| MYO1G | 0.057715 | 0.00797 | 2.035557 | 3.779107 |
| CDH26 | 0.057715 | 0.007974 | -2.23479 | 3.15129 |
| SORL1 | 0.057796 | 0.00799 | 1.635017 | 8.238549 |
| LINC01630 | 0.057878 | 0.00801 | 6.939062 | 0.794249 |
| ZNF684 | 0.057878 | 0.00801 | 1.653574 | 2.873105 |
| FGD5 | 0.05795 | 0.008024 | 1.746625 | 5.506176 |
| PPBPP2 | 0.058036 | 0.008041 | 6.346205 | 0.408456 |
| PHLPP1 | 0.058042 | 0.008046 | -1.11558 | 5.535567 |
| CTHRC1 | 0.058546 | 0.00812 | 2.676762 | 1.998896 |
| PCED1B-AS | 0.058562 | 0.008127 | 2.416725 | 3.024951 |
| EPHX4 | 0.058632 | 0.008141 | 4.735252 | 0.741503 |
| LINC01281 | 0.058741 | 0.008161 | 5.495429 | 0.783207 |
| SREK1IP1 | 0.058913 | 0.008189 | -1.00767 | 6.344289 |
| GORAB-AS1 | 0.058939 | 0.008197 | 2.555711 | 1.724948 |
| PPP1R3B | 0.058958 | 0.008204 | -3.75939 | 0.794196 |
| STARD13-A | 0.059219 | 0.008247 | 3.256731 | 2.356209 |
| ARRDC4 | 0.059219 | 0.00825 | 1.352631 | 5.61401 |
| LINC00920 | 0.059301 | 0.008266 | 3.292051 | 0.910537 |
| ST8SIA6 | 0.059376 | 0.008281 | -3.64436 | 1.618834 |
| EBF3 | 0.059404 | 0.008289 | 2.687471 | 3.172503 |
| CXorf38 | 0.059523 | 0.00831 | 1.169005 | 4.873988 |
| LRRC66 | 0.059648 | 0.008335 | 3.190331 | 1.92414 |
| SNORD87 | 0.059648 | 0.008338 | 1.612304 | 8.636113 |
| DNAJC18 | 0.059648 | 0.008341 | 1.680876 | 3.239682 |
| KCNQ1OT1 | 0.059648 | 0.008346 | 1.459395 | 9.805518 |
| HCP5 | 0.059656 | 0.008352 | 1.458937 | 5.046506 |
| TDRD1 | 0.059771 | 0.008372 | -4.35275 | 1.274886 |
| CKMT1A | 0.059805 | 0.008381 | -2.12268 | 3.339975 |
| ATOX1 | 0.059878 | 0.008396 | -1.6113 | 3.661249 |
| ECHDC3 | 0.05988 | 0.008401 | -2.52935 | 2.684565 |
| LEXM | 0.059944 | 0.008415 | -5.38944 | 0.468045 |
| VPS9D1 | 0.060017 | 0.008429 | -1.30799 | 4.601542 |
| SOX2 | 0.060081 | 0.008443 | -2.42735 | 5.598515 |

| | | | | |
|---|---|---|---|---|
| H2AFV | 0.060267 | 0.008474 | 1.069744 | 6.482432 |
| LMCD1-AS: | 0.060465 | 0.008506 | 2.484071 | 1.71139 |
| KCNJ18 | 0.060777 | 0.008557 | 7.333631 | 1.178623 |
| INHBB | 0.060777 | 0.008559 | 4.012024 | 4.90453 |
| LOC10050 | 0.061004 | 0.008596 | 3.208239 | 0.851178 |
| SNORD114 | 0.061014 | 0.008602 | 2.556699 | 2.219024 |
| THBS3 | 0.061023 | 0.008608 | 3.629411 | 1.095549 |
| FBXL19-AS: | 0.06109 | 0.008622 | 2.096561 | 3.080117 |
| KANSL1 | 0.061186 | 0.00864 | -1.76978 | 2.925955 |
| PARVB | 0.061324 | 0.008664 | 2.821897 | 1.515973 |
| RNF10 | 0.061383 | 0.008678 | -0.94314 | 7.748662 |
| SPRR2E | 0.061455 | 0.008692 | -4.18 | 7.718819 |
| BCAN | 0.061485 | 0.008706 | 2.454447 | 2.355333 |
| XKRX | 0.061485 | 0.008709 | -2.60938 | 2.882842 |
| TSPAN11 | 0.061485 | 0.008717 | 1.89888 | 3.689449 |
| MTHFD1 | 0.061485 | 0.008723 | -0.99455 | 6.372226 |
| COX7C | 0.061485 | 0.008723 | -0.99712 | 7.537034 |
| ANKRD34B | 0.061485 | 0.008725 | 6.148919 | 0.25992 |
| AMH | 0.061649 | 0.008753 | 2.58161 | 3.129252 |
| SLC11A1 | 0.061649 | 0.008757 | 2.119607 | 4.534887 |
| KANK2 | 0.061681 | 0.008767 | 2.044554 | 4.99845 |
| LOC28314( | 0.061721 | 0.008777 | 2.278539 | 3.85199 |
| GPC6 | 0.061862 | 0.008802 | 2.346078 | 4.933124 |
| CACNA1S | 0.061877 | 0.008809 | 4.25406 | 0.672528 |
| KNOP1 | 0.061991 | 0.008829 | 1.069688 | 5.614738 |
| PPP1R2B | 0.062023 | 0.008839 | -1.50225 | 3.605795 |
| DDI2 | 0.062044 | 0.008847 | -0.96588 | 5.969587 |
| ZFHX2 | 0.06205 | 0.008852 | 1.440735 | 4.212051 |
| SNORD33 | 0.062147 | 0.008871 | 1.486361 | 8.422295 |
| FCHSD2 | 0.062227 | 0.008887 | 0.998575 | 6.1138 |
| PPP2R5A | 0.062594 | 0.008945 | -1.23369 | 4.880948 |
| SLC2A13 | 0.062594 | 0.008953 | 1.558194 | 5.062689 |
| KANK1 | 0.062594 | 0.008954 | -1.52478 | 3.263442 |
| ZNF826P | 0.062624 | 0.008963 | 3.66962 | 1.080928 |
| LAMB1 | 0.062657 | 0.008972 | 1.217587 | 8.103696 |
| H3F3B | 0.062795 | 0.008997 | -1.02766 | 9.091552 |
| KBTBD12 | 0.062795 | 0.009001 | 2.61103 | 2.507437 |
| SNTN | 0.063131 | 0.009054 | 6.56395 | 0.531129 |
| IFIH1 | 0.063139 | 0.00906 | 1.162337 | 5.527921 |
| RACK1 | 0.063279 | 0.009089 | -0.95228 | 9.214514 |
| SNORA67 | 0.063279 | 0.009093 | -1.23006 | 6.174399 |
| FCER1G | 0.063279 | 0.009095 | 2.283381 | 3.571639 |
| LINC01733 | 0.063348 | 0.00911 | -6.91774 | 0.802322 |
| MAST4 | 0.063396 | 0.009122 | -1.84176 | 2.527564 |
| FAR2 | 0.063446 | 0.009133 | 4.067217 | 0.613587 |

| | | | | |
|---|---|---|---|---|
| DPYSL4 | 0.063501 | 0.009146 | 3.0553 | 1.354545 |
| PTX3 | 0.063546 | 0.009158 | 4.16679 | 2.801772 |
| 5-Mar | 0.063606 | 0.009178 | -0.97741 | 5.968595 |
| PLEKHG3 | 0.063606 | 0.009178 | -1.14286 | 6.967403 |
| TMEM45A | 0.063606 | 0.009181 | -1.89478 | 3.654171 |
| UBL3 | 0.0638 | 0.009217 | -1.05134 | 7.293004 |
| TMEM255I | 0.0638 | 0.009219 | 2.479018 | 2.577527 |
| TRNP1 | 0.0638 | 0.009226 | -1.37398 | 5.981851 |
| C9 | 0.0638 | 0.009232 | -3.3444 | 2.051702 |
| SNORD116 | 0.0638 | 0.009233 | 1.982306 | 6.708068 |
| GCOM1 | 0.063818 | 0.009241 | -1.85 | 4.074703 |
| NGB | 0.063834 | 0.009248 | 5.730068 | 0.971998 |
| KRT86 | 0.063848 | 0.009259 | 4.232815 | 1.974571 |
| PTPRG | 0.063848 | 0.00926 | 1.106979 | 6.432512 |
| SNORD116 | 0.064154 | 0.009309 | 2.030849 | 5.268147 |
| GNB2 | 0.064193 | 0.009319 | -1.04277 | 6.477718 |
| ABCD2 | 0.064381 | 0.009352 | 3.138243 | 1.311861 |
| COX6B1 | 0.064381 | 0.009356 | -1.01596 | 6.943569 |
| KIF3C | 0.064422 | 0.009367 | 1.517665 | 4.566799 |
| NAA20 | 0.065316 | 0.009502 | -2.2115 | 2.674113 |
| HACD3 | 0.065321 | 0.009515 | 1.03418 | 5.881002 |
| SCARNA6 | 0.065321 | 0.009521 | 1.344409 | 6.875522 |
| SAT1 | 0.065321 | 0.009522 | 1.502873 | 5.998161 |
| ZFP69 | 0.065321 | 0.009523 | 1.532719 | 3.570351 |
| NLRC5 | 0.065497 | 0.009553 | 1.394739 | 6.52013 |
| SCAPER | 0.065503 | 0.009559 | -1.20685 | 4.098377 |
| DIP2C | 0.065584 | 0.009583 | 1.111861 | 6.076729 |
| TSG1 | 0.065584 | 0.009584 | 5.323128 | 1.12233 |
| LRRN4 | 0.065584 | 0.009586 | 3.175242 | 1.495863 |
| LINC01160 | 0.06562 | 0.009597 | 3.936666 | 0.714624 |
| COL6A1 | 0.06562 | 0.009601 | 1.225338 | 8.467652 |
| FAM71D | 0.065748 | 0.009625 | 3.023164 | 1.461075 |
| FGF7 | 0.065865 | 0.009647 | 2.417612 | 5.967961 |
| DNAH12 | 0.066052 | 0.00968 | 3.230252 | 1.605694 |
| DLX6 | 0.066441 | 0.009742 | 4.92481 | 0.704454 |
| SLC30A1 | 0.066618 | 0.009778 | 1.094946 | 4.877778 |
| PVRIG | 0.066618 | 0.009779 | 1.561848 | 4.516838 |
| MIR100HG | 0.066618 | 0.009783 | 1.855135 | 5.430766 |
| LEPROTL1 | 0.066641 | 0.009795 | 0.988409 | 5.115577 |
| HTRA1 | 0.066641 | 0.009797 | 1.386442 | 5.303356 |
| SRSF6 | 0.066798 | 0.009825 | 1.675857 | 3.281469 |
| SEMA4G | 0.067051 | 0.009867 | 2.339683 | 2.55117 |
| SAMD4B | 0.067089 | 0.009882 | -1.02188 | 5.498419 |
| MIR6512 | 0.067089 | 0.009883 | -2.9069 | 1.575913 |
| CC2D2B | 0.067284 | 0.009917 | 2.277542 | 2.317654 |

| | | | | |
|---|---|---|---|---|
| RCBTB1 | 0.067343 | 0.009931 | 1.146803 | 5.177356 |
| PNMT | 0.067343 | 0.009936 | 6.213783 | 0.313866 |
| PARP12 | 0.067883 | 0.010023 | 3.11291 | 1.557937 |
| LSAMP | 0.067883 | 0.010026 | 1.822976 | 6.096337 |
| GRPEL1 | 0.068033 | 0.010053 | -1.29704 | 4.926006 |
| ATP6V1A | 0.06819 | 0.010081 | -1.08532 | 7.216984 |
| GPRC5A | 0.068222 | 0.010091 | 1.841799 | 8.122079 |
| SIT1 | 0.068281 | 0.010105 | 3.350341 | 0.442275 |
| SOX4 | 0.068389 | 0.010126 | 1.204967 | 6.993769 |
| TXN | 0.06848 | 0.010145 | -2.31043 | 1.828334 |
| SLC30A4 | 0.068485 | 0.010159 | -1.12938 | 4.739878 |
| POLR2L | 0.068485 | 0.01016 | -1.26318 | 4.902627 |
| HK1 | 0.068485 | 0.010162 | -1.59789 | 2.714231 |
| HECA | 0.068598 | 0.010187 | -0.97463 | 7.370047 |
| ADGRB3 | 0.068598 | 0.010189 | 2.779206 | 2.258679 |
| RAMP1 | 0.06905 | 0.010261 | 4.341407 | 1.282084 |
| IFITM1 | 0.06905 | 0.010266 | 1.702236 | 5.697349 |
| HRNR | 0.069097 | 0.010279 | -1.22419 | 4.387023 |
| RAB3B | 0.069202 | 0.0103 | 2.878797 | 3.01431 |
| LINC00861 | 0.069362 | 0.010329 | 2.299892 | 3.178148 |
| SSH2 | 0.069362 | 0.010334 | 2.749932 | 2.230734 |
| LINC01599 | 0.069439 | 0.010351 | -3.11822 | 1.717475 |
| EI24 | 0.069461 | 0.010359 | 3.717393 | 0.819528 |
| MXI1 | 0.069837 | 0.010422 | -1.9261 | 2.586131 |
| CATSPERB | 0.069837 | 0.010426 | 3.904452 | 2.310378 |
| ZNF44 | 0.069881 | 0.010438 | 2.519367 | 0.80815 |
| TCIRG1 | 0.070008 | 0.010462 | 1.522378 | 3.775923 |
| CELF3 | 0.07025 | 0.010509 | 7.172239 | 1.046073 |
| LOC100128 | 0.07025 | 0.010509 | 4.260434 | 1.216735 |
| NXPH3 | 0.07027 | 0.010524 | 2.646191 | 2.562885 |
| PLEKHM1P | 0.07027 | 0.010526 | -1.04689 | 6.414205 |
| PKIG | 0.07027 | 0.010528 | 3.54698 | 1.42103 |
| LPCAT4 | 0.070351 | 0.010546 | -1.03459 | 6.024597 |
| KIAA1217 | 0.070422 | 0.010562 | -1.12585 | 7.424543 |
| RTL6 | 0.070525 | 0.010583 | 1.033833 | 5.592346 |
| SLC3A1 | 0.070728 | 0.010629 | 3.476638 | 1.317225 |
| IL18RAP | 0.070728 | 0.01063 | 2.248148 | 2.961191 |
| EID3 | 0.070728 | 0.010631 | 1.601601 | 3.736838 |
| NRK | 0.070728 | 0.010634 | 3.507256 | 2.202338 |
| RRAGA | 0.070827 | 0.010655 | -1.03265 | 5.205549 |
| ZNF675 | 0.070873 | 0.010667 | -1.58004 | 4.516081 |
| TNKS1BP1 | 0.070991 | 0.01069 | -0.99435 | 9.231507 |
| HSD17B2 | 0.071096 | 0.010712 | 2.427987 | 2.676554 |
| ALOX12B | 0.071187 | 0.010731 | -2.40934 | 3.892844 |
| TNS1 | 0.07147 | 0.010779 | 2.574157 | 2.640984 |

| | | | | |
|---|---|---|---|---|
| SYNPO2 | 0.071673 | 0.010815 | 2.124923 | 7.370405 |
| MUC5AC | 0.07176 | 0.010833 | 3.96048 | 11.2345 |
| ZNF780B | 0.071782 | 0.010842 | 1.096336 | 5.829191 |
| ASXL3 | 0.071918 | 0.01087 | 2.75675 | 4.168755 |
| MIR32 | 0.071918 | 0.010874 | -5.52256 | 0.601441 |
| MYH1 | 0.071943 | 0.010883 | 4.725711 | 0.672374 |
| PYGL | 0.072029 | 0.01091 | -3.37303 | 1.067622 |
| TBC1D16 | 0.072029 | 0.010911 | 1.388104 | 3.486229 |
| FOXO1 | 0.072029 | 0.010916 | 1.013988 | 6.531911 |
| GIPC2 | 0.072029 | 0.010922 | 2.798916 | 1.061483 |
| PSMC6 | 0.072029 | 0.010924 | -1.24658 | 6.575419 |
| FCN1 | 0.072029 | 0.010929 | 2.547027 | 1.74101 |
| TMEM256 | 0.072046 | 0.010937 | -1.8049 | 4.23534 |
| TTBK1 | 0.072132 | 0.01096 | 2.806008 | 1.898663 |
| RBM8A | 0.072132 | 0.010966 | -0.99905 | 7.371795 |
| FAM177B | 0.072132 | 0.010967 | 2.652413 | 2.819633 |
| DCLK1 | 0.072159 | 0.010976 | 2.118921 | 3.32715 |
| GVQW3 | 0.072175 | 0.010984 | 1.844065 | 2.614261 |
| RSC1A1 | 0.072222 | 0.010997 | -1.24331 | 4.904511 |
| RNF175 | 0.072386 | 0.011027 | 4.312634 | 0.8608 |
| SNORD116 | 0.072445 | 0.011042 | 2.001206 | 7.086574 |
| F2RL1 | 0.072555 | 0.011064 | 1.152967 | 5.480832 |
| VPS37B | 0.072658 | 0.011085 | -1.01117 | 7.271069 |
| ZNF367 | 0.072731 | 0.011102 | 1.500569 | 3.847551 |
| MYH15 | 0.073057 | 0.011157 | 2.708312 | 2.085343 |
| PHGDH | 0.07312 | 0.011172 | -1.24872 | 5.257454 |
| SCARNA5 | 0.073262 | 0.0112 | 1.364535 | 8.130135 |
| CCSER1 | 0.073271 | 0.011207 | 3.023229 | 2.828928 |
| ACTA2-AS1 | 0.073272 | 0.011218 | 2.802463 | 3.399082 |
| ZNF600 | 0.073272 | 0.011218 | 1.227142 | 4.956907 |
| MIR5706 | 0.073377 | 0.01124 | -4.00247 | 0.466188 |
| KCNE4 | 0.073463 | 0.011258 | 2.433368 | 2.947259 |
| CCR1 | 0.073544 | 0.011277 | 2.282986 | 3.208485 |
| SDHA | 0.073665 | 0.011301 | 2.91874 | 0.715255 |
| NHP2 | 0.073675 | 0.011308 | -2.42002 | 1.761609 |
| OXGR1 | 0.073968 | 0.011358 | -3.86022 | 0.8248 |
| ZXDB | 0.074021 | 0.011372 | 1.181551 | 4.303421 |
| NELFA | 0.074185 | 0.011403 | -1.06139 | 4.908082 |
| MFHAS1 | 0.074225 | 0.011415 | 1.630201 | 5.486697 |
| LRRC8A | 0.074371 | 0.011443 | -3.16986 | 1.528028 |
| SF3B3 | 0.074647 | 0.011491 | -0.9341 | 7.915709 |
| ZSCAN16 | 0.0748 | 0.01152 | 1.819361 | 3.500094 |
| CEP135 | 0.074835 | 0.011531 | 1.139217 | 4.741059 |
| RNF223 | 0.074919 | 0.01155 | -2.27724 | 3.376622 |
| ZNF326 | 0.07498 | 0.011565 | -0.92281 | 6.84833 |

| | | | | |
|---|---|---|---|---|
| LINC02563 | 0.075008 | 0.01158 | 6.558637 | 0.577732 |
| WTAPP1 | 0.075008 | 0.011581 | 3.316994 | 1.127774 |
| SNORD116 | 0.075027 | 0.01159 | 1.930947 | 7.545199 |
| PPIF | 0.075258 | 0.011631 | -1.17684 | 6.172099 |
| DSEL | 0.075261 | 0.011637 | 1.951821 | 3.660853 |
| ALMS1 | 0.075265 | 0.011644 | 0.915263 | 7.467118 |
| PDZRN4 | 0.075471 | 0.011681 | 5.054109 | 0.936807 |
| PRKG1 | 0.075483 | 0.011689 | 2.121738 | 3.222894 |
| CLIC4 | 0.075702 | 0.011728 | 1.064319 | 7.76493 |
| TLR6 | 0.075718 | 0.011737 | 2.316853 | 3.26514 |
| SLCO1B1 | 0.075787 | 0.011754 | 5.225203 | 0.579322 |
| LOC101929 | 0.075787 | 0.011759 | 2.670075 | 2.073851 |
| SCN3A | 0.075895 | 0.011782 | 6.1863 | 0.299059 |
| SUMO1P1 | 0.076053 | 0.011814 | 6.338769 | 0.390349 |
| CDS2 | 0.076053 | 0.011822 | 1.031273 | 6.884275 |
| RALB | 0.076053 | 0.011824 | -1.10116 | 7.462037 |
| LINC02363 | 0.076564 | 0.011916 | 3.493643 | 1.242271 |
| PKD2 | 0.076564 | 0.011919 | 1.220994 | 5.970543 |
| SNORD116 | 0.076564 | 0.01192 | 2.019478 | 8.512294 |
| PCDHGB8P | 0.076575 | 0.011928 | -1.81088 | 2.927707 |
| ZNF30 | 0.076806 | 0.01197 | 4.423283 | 0.501355 |
| YWHAQ | 0.076818 | 0.011978 | -1.01145 | 7.900075 |
| SNORD102 | 0.076865 | 0.011991 | 1.409626 | 5.276621 |
| FASN | 0.07705 | 0.012025 | -1.14282 | 8.180207 |
| STK32C | 0.07708 | 0.012045 | 2.345967 | 3.046899 |
| LINC00434 | 0.07708 | 0.012048 | 4.459823 | 0.520895 |
| PCDHB15 | 0.07708 | 0.01206 | 2.497324 | 2.372344 |
| VWDE | 0.07708 | 0.012067 | 2.488865 | 3.288829 |
| OAS2 | 0.07708 | 0.012068 | 1.321969 | 4.343465 |
| STX12 | 0.07708 | 0.012068 | -0.97057 | 6.820315 |
| LOC285593 | 0.07708 | 0.012074 | 3.578806 | 0.903787 |
| LENG8 | 0.07708 | 0.012077 | 1.190196 | 5.519367 |
| CADM4 | 0.077226 | 0.012111 | -1.35406 | 4.683586 |
| RAB40B | 0.077226 | 0.012112 | 1.312377 | 4.616434 |
| SNORD119 | 0.077284 | 0.012127 | 1.483689 | 5.847388 |
| GOSR2 | 0.077308 | 0.012136 | 1.046552 | 5.308365 |
| ZNF80 | 0.077606 | 0.012189 | 3.875076 | 0.709 |
| REXO2 | 0.077718 | 0.012218 | -1.08234 | 6.164396 |
| SLC35F6 | 0.077718 | 0.012224 | -1.0244 | 6.378046 |
| FOXF1 | 0.077718 | 0.012225 | 1.500869 | 4.755022 |
| MIGA2 | 0.07781 | 0.012245 | -0.93911 | 5.610542 |
| LOC100287 | 0.077836 | 0.012255 | 3.863337 | 0.71816 |
| RTKN2 | 0.077895 | 0.01227 | -1.72345 | 5.46024 |
| LINC00619 | 0.078287 | 0.012338 | 6.330299 | 0.398064 |
| MAST4-AS1 | 0.078405 | 0.012362 | -2.54488 | 1.913145 |

| | | | | |
|---|---|---|---|---|
| BLK | 0.078811 | 0.012432 | 3.31995 | 1.607249 |
| PLEKHS1 | 0.078923 | 0.012456 | 3.69763 | 1.857555 |
| COLGALT1 | 0.078997 | 0.012474 | 1.15524 | 6.200026 |
| CREB3 | 0.079188 | 0.01251 | 1.220816 | 4.85376 |
| EVA1B | 0.079247 | 0.012525 | -2.43229 | 2.691821 |
| KLF3 | 0.079247 | 0.012531 | -1.07391 | 8.319763 |
| NCF1C | 0.079525 | 0.012581 | 2.049064 | 2.098037 |
| DES | 0.079829 | 0.012636 | 2.801866 | 8.966153 |
| SEMA3G | 0.079863 | 0.012647 | 2.364523 | 3.291006 |
| ACTG2 | 0.079922 | 0.012663 | 2.818727 | 3.653781 |
| SNORD114 | 0.080284 | 0.012726 | 4.341726 | 0.800509 |
| ANKRD13A | 0.080297 | 0.012734 | -0.94529 | 6.960431 |
| TMEM109 | 0.080313 | 0.012743 | -0.94018 | 6.218168 |
| HAVCR2 | 0.080319 | 0.01275 | 2.266695 | 3.385534 |
| CASC15 | 0.080333 | 0.012758 | 2.489378 | 3.093173 |
| LINC00494 | 0.080379 | 0.012774 | 2.834991 | 2.163193 |
| HOXC13 | 0.080379 | 0.012778 | 3.99847 | 1.340515 |
| SNORD116 | 0.080733 | 0.01284 | 1.961582 | 7.958782 |
| FAM84A | 0.080751 | 0.012849 | -1.65005 | 6.70619 |
| BCHE | 0.080901 | 0.012883 | 3.968858 | 2.15678 |
| TLR2 | 0.080901 | 0.012885 | 1.975568 | 4.163087 |
| LHFPL3 | 0.080903 | 0.012892 | 3.82034 | 1.716265 |
| PLK1 | 0.081041 | 0.01292 | 1.146385 | 4.563409 |
| ADIPOR2 | 0.081046 | 0.012927 | -1.03765 | 7.087979 |
| NOL12 | 0.0811 | 0.012942 | -1.09836 | 5.12874 |
| RASL10B | 0.081164 | 0.012958 | 2.814361 | 1.291308 |
| SDK1 | 0.081277 | 0.012982 | 1.458505 | 4.725164 |
| SNORD23 | 0.081482 | 0.013022 | 2.487065 | 1.852563 |
| TAGLN | 0.081482 | 0.013028 | 2.44568 | 2.27943 |
| EPHB3 | 0.081596 | 0.013052 | -1.32519 | 6.43234 |
| SLC24A5 | 0.081626 | 0.013063 | 3.769717 | 1.045212 |
| EGFR | 0.081639 | 0.013071 | -1.17025 | 7.785511 |
| KLF9 | 0.08164 | 0.013081 | 1.268403 | 7.073907 |
| THSD7A | 0.08164 | 0.013089 | 1.478007 | 4.082913 |
| SGCE | 0.08164 | 0.01309 | 4.942873 | 0.371645 |
| BAIAP2 | 0.081663 | 0.0131 | -1.62522 | 4.379648 |
| LINC01103 | 0.081735 | 0.013118 | 6.29719 | 0.355931 |
| ADGRL3 | 0.081814 | 0.013142 | 2.160105 | 4.865889 |
| OSCP1 | 0.081814 | 0.013143 | 2.367678 | 2.604392 |
| GPR26 | 0.081922 | 0.013167 | 4.050661 | 1.215194 |
| MGST1 | 0.081984 | 0.013183 | -3.03066 | 1.578253 |
| LOC10192ℇ | 0.081984 | 0.013189 | 3.099967 | 0.351202 |
| RASSF8 | 0.082053 | 0.013206 | 2.844639 | 1.337264 |
| ALPG | 0.082229 | 0.013241 | 3.908922 | 2.296279 |
| IL10RA | 0.082315 | 0.013261 | 2.552427 | 0.657438 |

| | | | | |
|---|---|---|---|---|
| NOTCH2 | 0.082475 | 0.013293 | -1.05256 | 8.175407 |
| LOC101927 | 0.082581 | 0.013317 | -1.67517 | 2.800493 |
| WSCD1 | 0.082765 | 0.013353 | 2.696346 | 1.876215 |
| RAMAC | 0.082765 | 0.013359 | -1.41609 | 3.213907 |
| HKR1 | 0.082859 | 0.01338 | 1.049829 | 5.994802 |
| LOC100652 | 0.083276 | 0.013454 | 1.815227 | 3.71032 |
| KIAA1549L | 0.083434 | 0.013486 | -1.30425 | 4.119559 |
| CARD6 | 0.083562 | 0.013513 | -1.06565 | 5.719719 |
| HDAC3 | 0.083584 | 0.013531 | -0.99118 | 5.879059 |
| ENGASE | 0.083584 | 0.013532 | 1.175303 | 5.833118 |
| INKA1 | 0.083584 | 0.013536 | -2.09307 | 2.22045 |
| CAVIN3 | 0.083598 | 0.013544 | 2.57769 | 3.145712 |
| PLEKHG5 | 0.083616 | 0.013554 | -2.03342 | 1.638656 |
| RBX1 | 0.083661 | 0.013567 | -1.14316 | 4.70431 |
| NEGR1 | 0.083981 | 0.013626 | 1.813135 | 4.37965 |
| ZSWIM5 | 0.084186 | 0.013665 | 1.5701 | 3.46149 |
| ZNF225 | 0.084242 | 0.013681 | 1.29772 | 4.029463 |
| NEU1 | 0.084327 | 0.013701 | 4.587649 | 0.632408 |
| TRPS1 | 0.084591 | 0.01375 | -2.96636 | 1.26587 |
| EFNA5 | 0.084777 | 0.013787 | -1.51343 | 5.794936 |
| PRR36 | 0.085294 | 0.013879 | 2.881806 | 2.433455 |
| MMP7 | 0.085294 | 0.013887 | 3.593616 | 4.30886 |
| GLDN | 0.085294 | 0.013894 | 2.377541 | 2.396311 |
| ADM | 0.085294 | 0.013897 | -1.77451 | 6.611466 |
| ARL10 | 0.085637 | 0.01396 | -1.65855 | 3.449058 |
| CCDC153 | 0.085649 | 0.013969 | 2.690388 | 1.459752 |
| ARHGEF35 | 0.085649 | 0.013977 | -1.63602 | 4.069169 |
| MIR4458H | 0.085649 | 0.013981 | -1.54933 | 3.222002 |
| PPP4R1-AS | 0.085693 | 0.013995 | -3.30754 | 0.811229 |
| HLA-DMB | 0.085707 | 0.014004 | 3.272676 | 1.173101 |
| KRTAP10-1 | 0.086111 | 0.014083 | 6.182545 | 1.360138 |
| PLEKHH2 | 0.086111 | 0.014083 | 1.438101 | 6.689031 |
| LOC102724 | 0.086197 | 0.014103 | 3.029341 | 1.863796 |
| ZNF728 | 0.086207 | 0.014112 | 3.928277 | 1.679813 |
| UGCG | 0.086427 | 0.014154 | -1.20316 | 7.654181 |
| BOK | 0.08648 | 0.01417 | -0.95864 | 5.223986 |
| KCTD3 | 0.086574 | 0.014192 | -0.92365 | 7.773513 |
| CCDC150 | 0.08673 | 0.014224 | 2.07863 | 3.218804 |
| RAB19 | 0.087465 | 0.014356 | 3.252669 | 0.999248 |
| CPD | 0.087465 | 0.014358 | 1.41124 | 4.139751 |
| MYL9 | 0.0875 | 0.01437 | 3.024707 | 2.40068 |
| PLA2G4B | 0.087891 | 0.014441 | -2.8777 | 1.534955 |
| MIR3137 | 0.087912 | 0.014451 | -3.69528 | 0.424881 |
| C11orf80 | 0.087996 | 0.014472 | -2.24078 | 2.448139 |
| HBA1 | 0.088073 | 0.014494 | 3.464668 | 0.970899 |

| | | | | |
|---|---|---|---|---|
| TNS4 | 0.088073 | 0.014498 | -2.0676 | 7.962443 |
| HYDIN2 | 0.088122 | 0.014518 | 2.053358 | 3.561101 |
| HSD3BP4 | 0.088122 | 0.014524 | 2.179978 | 3.321327 |
| POLG2 | 0.088122 | 0.014526 | 1.178756 | 4.143135 |
| NPTX1 | 0.08815 | 0.014537 | 3.569625 | 1.317399 |
| RPL22 | 0.088252 | 0.014564 | -1.00134 | 8.777343 |
| TRIM7 | 0.088252 | 0.014567 | -2.48836 | 2.473082 |
| TBX2 | 0.088323 | 0.014591 | 1.858493 | 4.431334 |
| BMS1P22 | 0.088323 | 0.014593 | 3.062218 | 1.269196 |
| ZNF496 | 0.088414 | 0.014614 | 1.340268 | 4.458682 |
| OSBPL7 | 0.088417 | 0.014622 | 2.009458 | 4.892713 |
| SLC24A3 | 0.088918 | 0.014711 | -1.27492 | 5.901906 |
| BLID | 0.089443 | 0.014805 | 3.542253 | 1.31907 |
| WEE1 | 0.089678 | 0.014851 | -1.74398 | 3.837622 |
| PCDH12 | 0.089695 | 0.01486 | 1.846163 | 3.656366 |
| ITGA11 | 0.089798 | 0.014884 | 2.468861 | 4.724441 |
| LOC101927 | 0.089854 | 0.0149 | 5.833913 | 1.03973 |
| ALS2 | 0.08991 | 0.014916 | 0.976028 | 5.692967 |
| RORA-AS1 | 0.089957 | 0.014931 | -1.52528 | 4.244797 |
| COX8A | 0.089958 | 0.014938 | -0.93147 | 5.476783 |
| VPS25 | 0.090089 | 0.014967 | -0.96146 | 5.158549 |
| CAVIN4 | 0.09017 | 0.014987 | 3.094283 | 1.056813 |
| TIMM10 | 0.090492 | 0.015048 | -1.178 | 4.17214 |
| LOC101927 | 0.090554 | 0.015065 | 2.685699 | 1.461901 |
| CPOX | 0.090565 | 0.015073 | -1.00909 | 5.405348 |
| DCN | 0.090626 | 0.015091 | 2.07439 | 4.06834 |
| TTC3P1 | 0.090819 | 0.01513 | 1.438583 | 3.371845 |
| CENPF | 0.09087 | 0.015148 | 1.150627 | 7.898241 |
| RASSF2 | 0.09087 | 0.015152 | 2.534866 | 1.550633 |
| CLUH | 0.09102 | 0.015184 | -0.98884 | 7.147061 |
| PLEKHA5 | 0.091646 | 0.015298 | -1.01169 | 5.490651 |
| MAP3K21 | 0.091646 | 0.015313 | 1.156707 | 5.25741 |
| SYCP2L | 0.091646 | 0.015317 | 2.683535 | 2.552542 |
| PARVG | 0.091646 | 0.015323 | 3.596202 | 0.699236 |
| HTT | 0.091646 | 0.015328 | -0.85777 | 7.977791 |
| CELF2-AS1 | 0.091646 | 0.01533 | 1.395274 | 3.615884 |
| RNF125 | 0.091753 | 0.015359 | 1.386667 | 3.971514 |
| PDZK1P1 | 0.091753 | 0.015368 | 1.506583 | 3.540852 |
| MSI2 | 0.091753 | 0.015376 | 1.325492 | 4.060088 |
| BCAS1 | 0.091753 | 0.015376 | 2.658537 | 2.580663 |
| ACSL5 | 0.091868 | 0.015402 | 2.98939 | 1.927238 |
| CDO1 | 0.092034 | 0.015444 | 4.125473 | 0.631948 |
| SH3RF1 | 0.092034 | 0.01545 | 0.965117 | 6.3096 |
| SNAPC3 | 0.092034 | 0.015455 | -0.83111 | 5.941359 |
| ATP6V1B1 | 0.092034 | 0.015458 | 2.370014 | 1.829534 |

| | | | | |
|---|---|---|---|---|
| LOC100131 | 0.092044 | 0.015467 | 2.820817 | 1.61241 |
| GM2A | 0.092239 | 0.015507 | -1.65266 | 2.737021 |
| C1QTNF1 | 0.092245 | 0.015515 | 2.751148 | 0.67363 |
| EXOSC5 | 0.092248 | 0.015527 | -1.36472 | 3.295277 |
| RGS2 | 0.092248 | 0.015533 | 1.703768 | 6.222945 |
| PCYOX1 | 0.092248 | 0.015536 | -1.00972 | 7.398206 |
| RNASEK | 0.092251 | 0.015546 | -3.19408 | 1.339793 |
| SLC51B | 0.092251 | 0.015551 | 2.981606 | 0.845015 |
| EIF4EBP1 | 0.09243 | 0.015588 | -1.02382 | 5.348472 |
| UBE2D4 | 0.093101 | 0.015708 | -1.10106 | 4.920938 |
| TNNC2 | 0.093111 | 0.015717 | 3.059689 | 1.188559 |
| CRAT37 | 0.093248 | 0.015747 | 4.732671 | 2.228726 |
| RHOJ | 0.093644 | 0.015821 | 1.316663 | 3.579106 |
| CRTC2 | 0.093687 | 0.015836 | 0.894989 | 5.950769 |
| NGEF | 0.093828 | 0.015867 | 2.242631 | 2.259586 |
| MIR4637 | 0.093915 | 0.015892 | 5.085481 | 0.467485 |
| ANXA5 | 0.093915 | 0.015896 | 0.962959 | 7.634243 |
| UCP2 | 0.093992 | 0.015916 | 1.651125 | 4.178046 |
| LOC653653 | 0.094004 | 0.015925 | 2.025669 | 2.656202 |
| NID1 | 0.094007 | 0.015933 | 1.363436 | 7.394071 |
| KCTD9 | 0.09404 | 0.015946 | -0.9814 | 7.259605 |
| TET3 | 0.094653 | 0.016062 | -0.95282 | 7.82384 |
| UNC93B1 | 0.094653 | 0.016064 | 1.00996 | 5.745468 |
| TCF21 | 0.094669 | 0.016074 | 2.074285 | 3.651142 |
| DVL2 | 0.094726 | 0.016098 | -1.04432 | 5.07972 |
| DDX41 | 0.094726 | 0.016098 | -0.85467 | 6.015051 |
| HHIPL1 | 0.09473 | 0.016106 | 3.069369 | 1.166887 |
| TMX1 | 0.094731 | 0.016113 | -0.92797 | 6.050259 |
| PPP2R3A | 0.09476 | 0.016126 | -1.3548 | 5.294037 |
| NACA4P | 0.094837 | 0.016151 | -1.01979 | 4.619483 |
| MBOAT1 | 0.094837 | 0.016153 | 2.747895 | 1.96143 |
| CCDC30 | 0.094997 | 0.016188 | 2.320606 | 3.153537 |
| LOC100294 | 0.094997 | 0.016195 | 1.299014 | 4.11453 |
| CD1A | 0.095015 | 0.016207 | -2.72953 | 1.312974 |
| SEC22C | 0.095015 | 0.016212 | 1.074307 | 4.934523 |
| LINC01477 | 0.095163 | 0.01625 | 6.271893 | 0.355789 |
| ADGRG7 | 0.095163 | 0.016252 | 6.992607 | 0.834265 |
| USP17L15 | 0.095212 | 0.016271 | 5.03492 | 1.391554 |
| TUBA1C | 0.095212 | 0.016275 | -2.07611 | 1.951474 |
| KIAA2012 | 0.095388 | 0.016312 | 3.594541 | 0.57645 |
| CDC6 | 0.095608 | 0.016357 | 1.427539 | 4.575943 |
| TOR3A | 0.095608 | 0.016365 | 1.111541 | 4.632048 |
| MYOCD | 0.095636 | 0.016377 | 5.122532 | 0.479779 |
| FKBP3 | 0.095647 | 0.016391 | -0.93484 | 5.710596 |
| ENO4 | 0.095647 | 0.016393 | 2.137452 | 2.046213 |

| | | | | |
|---|---|---|---|---|
| PELI2 | 0.095963 | 0.016455 | 1.737215 | 5.554045 |
| GVINP1 | 0.096075 | 0.016487 | 1.326146 | 4.342944 |
| NR2F1 | 0.096075 | 0.016488 | 1.530955 | 5.086416 |
| CELF2-AS2 | 0.096108 | 0.016501 | 2.12868 | 2.612198 |
| NPR1 | 0.096262 | 0.016535 | 1.794503 | 2.85896 |
| CAMK2N2 | 0.096292 | 0.016554 | 2.673571 | 1.221353 |
| APOD | 0.096292 | 0.016561 | 2.025695 | 5.119142 |
| ZFPM2 | 0.096292 | 0.016562 | 2.006728 | 4.189332 |
| SLC39A14 | 0.096686 | 0.016637 | 2.44227 | 2.506115 |
| MSH6 | 0.097038 | 0.016705 | 4.347078 | 0.381527 |
| COQ8B | 0.097278 | 0.016754 | 2.93507 | 0.436677 |
| LINC01269 | 0.097529 | 0.016808 | -2.8516 | 1.699601 |
| ANO8 | 0.097529 | 0.016812 | -0.99424 | 5.467218 |
| FOXG1 | 0.097979 | 0.016897 | 4.060127 | 0.588419 |
| RHOB | 0.098167 | 0.016937 | 1.240689 | 7.43435 |
| JAM3 | 0.098237 | 0.016957 | 3.863009 | 0.545017 |
| MIA3 | 0.098237 | 0.016964 | 0.885095 | 7.239715 |
| PHF13 | 0.098373 | 0.016995 | -1.01976 | 5.169424 |
| NOD1 | 0.098389 | 0.017005 | 0.928475 | 5.190404 |
| ZC3H7A | 0.09862 | 0.017053 | 0.91048 | 6.49133 |
| LINC01615 | 0.098796 | 0.017091 | 3.172425 | 0.979545 |
| TAL1 | 0.098847 | 0.017107 | 4.756014 | 0.243972 |
| FAM155A | 0.098974 | 0.017137 | 2.128471 | 2.215737 |
| LRP2 | 0.099093 | 0.017165 | 2.325632 | 2.027025 |
| CDC42EP5 | 0.099099 | 0.017174 | 3.258101 | 1.205135 |
| IFT88 | 0.099213 | 0.017228 | 2.939917 | 0.806482 |
| ARVCF | 0.099213 | 0.017236 | 1.585099 | 3.805926 |
| MAPK8IP1I | 0.099213 | 0.017237 | -3.56706 | 1.106835 |
| SUCNR1 | 0.099213 | 0.017242 | 3.486559 | 0.897079 |
| SNORD13 | 0.099213 | 0.017243 | 1.203428 | 6.985089 |
| CYP27A1 | 0.099213 | 0.017245 | 1.809054 | 3.31758 |
| KIAA1671 | 0.099213 | 0.017246 | -0.97547 | 8.370017 |
| STXBP5-AS | 0.099222 | 0.017255 | -2.12187 | 1.982788 |
| OCM | 0.099252 | 0.017268 | 6.666162 | 0.647986 |
| NACAD | 0.099288 | 0.017282 | 2.432843 | 2.427049 |
| HLA-DRA | 0.099392 | 0.017308 | 1.29873 | 5.495895 |
| GNPDA1 | 0.099521 | 0.017344 | 1.112715 | 4.727389 |
| HCG27 | 0.099521 | 0.017345 | 2.907451 | 1.825153 |
| SLC9A9-AS | 0.099828 | 0.017406 | -3.19698 | 1.082917 |
| MCC | 0.099843 | 0.017417 | -1.56818 | 3.137231 |
| DGAT1 | 0.100468 | 0.017541 | 0.924164 | 5.483977 |
| CCDC160 | 0.100468 | 0.017547 | -3.82511 | 1.088849 |
| ENPP4 | 0.100468 | 0.017554 | 2.324349 | 4.057891 |
| NOS3 | 0.100468 | 0.017562 | -1.49067 | 4.697254 |
| GREB1 | 0.100468 | 0.01757 | 1.829098 | 3.93684 |

| | | | | |
|---|---|---|---|---|
| LNX2 | 0.100468 | 0.017571 | -0.94135 | 6.31595 |
| ZFPM2-AS1 | 0.100482 | 0.017582 | 2.560442 | 1.832172 |
| BBOX1 | 0.100519 | 0.017601 | -2.11117 | 3.400395 |
| UCHL1 | 0.100519 | 0.017607 | 3.09303 | 0.981889 |
| MAFB | 0.100519 | 0.017611 | -1.03121 | 6.229143 |
| GRB7 | 0.100525 | 0.017638 | -1.81489 | 2.556919 |
| FBXO3 | 0.100525 | 0.017645 | -1.05654 | 4.83494 |
| C17orf67 | 0.100525 | 0.017652 | 2.131058 | 2.861884 |
| PLEKHA6 | 0.100525 | 0.017654 | 1.037793 | 7.185737 |
| MIOS | 0.100525 | 0.017655 | -0.9137 | 6.328453 |
| SNORD116 | 0.100525 | 0.017663 | 1.77643 | 7.384773 |
| ZNF589 | 0.100525 | 0.017666 | -1.11561 | 6.012252 |
| LIPC | 0.100769 | 0.017716 | 4.417378 | 0.414372 |
| LOC10012 | 0.101367 | 0.017829 | 1.048856 | 5.752481 |
| LINC01695 | 0.101467 | 0.017854 | 3.102473 | 1.423744 |
| ZEB1-AS1 | 0.101519 | 0.017871 | 2.008929 | 2.651234 |
| KLF12 | 0.101696 | 0.01791 | 1.240493 | 5.266464 |
| EFNB3 | 0.101964 | 0.017965 | -2.09177 | 2.279221 |
| SYNE2 | 0.102215 | 0.018017 | -2.99766 | 1.459166 |
| DISP1 | 0.102506 | 0.018076 | 1.62441 | 3.829743 |
| ADCY9 | 0.103 | 0.018171 | 1.132484 | 6.335446 |
| CES2 | 0.10322 | 0.018221 | -2.80835 | 1.283556 |
| AMOT | 0.10322 | 0.018226 | 2.101416 | 2.641525 |
| CACNB2 | 0.103419 | 0.018269 | 2.576439 | 2.520951 |
| PXDN | 0.103478 | 0.018287 | 1.263629 | 6.105363 |
| THEM5 | 0.103646 | 0.018331 | 1.56614 | 3.133628 |
| ZFPM1 | 0.103646 | 0.018333 | 1.130553 | 4.243844 |
| PTGIR | 0.10369 | 0.01835 | 2.19732 | 1.780234 |
| CEP128 | 0.10369 | 0.018356 | 0.948987 | 5.4019 |
| FHDC1 | 0.103818 | 0.018387 | -1.15413 | 5.855189 |
| SMG9 | 0.103833 | 0.018397 | 0.981495 | 5.023097 |
| COMTD1 | 0.104234 | 0.018477 | -1.27123 | 4.123707 |
| ZNF239 | 0.104328 | 0.018501 | 3.485166 | 1.027028 |
| PPFIA2-AS1 | 0.104373 | 0.018517 | 3.847507 | 0.649338 |
| MIR181A1I | 0.104477 | 0.018543 | 3.273234 | 0.998325 |
| SLC38A10 | 0.104596 | 0.018577 | 0.925311 | 5.371407 |
| SUSD2 | 0.104596 | 0.01858 | 1.671939 | 3.642975 |
| TSPEAR-AS | 0.104606 | 0.01859 | 5.913869 | 0.125499 |
| TXLNB | 0.10467 | 0.018615 | 1.917649 | 2.625108 |
| CADM3-AS | 0.10467 | 0.018625 | 3.694283 | 0.923281 |
| EFCAB13 | 0.10467 | 0.018626 | 1.6868 | 3.909152 |
| DENND1A | 0.104705 | 0.018642 | 1.321988 | 3.787793 |
| MAGEC1 | 0.104705 | 0.018648 | -6.1777 | 1.157178 |
| EHBP1 | 0.104899 | 0.018697 | -3.89435 | 0.428533 |
| IL22RA1 | 0.104899 | 0.018705 | -1.67721 | 5.035691 |

| | | | | |
|---|---|---|---|---|
| CLEC11A | 0.104899 | 0.018713 | 1.903748 | 2.79491 |
| NALCN | 0.104899 | 0.018714 | 2.656657 | 3.337073 |
| CHIC2 | 0.105117 | 0.018761 | -0.97154 | 5.141837 |
| TMEM159 | 0.105227 | 0.018793 | -2.73405 | 1.280631 |
| DENND1B | 0.105227 | 0.018797 | -1.45153 | 3.196953 |
| RAB25 | 0.105643 | 0.018879 | -1.82474 | 2.589789 |
| ZNF597 | 0.105791 | 0.018914 | 1.464181 | 4.188184 |
| KIF1B | 0.105957 | 0.018951 | -0.87573 | 8.122372 |
| DAG1 | 0.10604 | 0.018974 | 3.134247 | 0.9077 |
| PHTF1 | 0.106366 | 0.019041 | 1.236949 | 4.635676 |
| SREBF2 | 0.106397 | 0.019063 | -1.22586 | 4.943252 |
| TSG101 | 0.106397 | 0.019063 | -0.88421 | 5.901444 |
| NMNAT3 | 0.106528 | 0.019101 | 3.125192 | 1.175825 |
| AFF1 | 0.106528 | 0.019102 | -1.30756 | 3.180727 |
| ZNF853 | 0.106969 | 0.01919 | 1.924149 | 2.732971 |
| PFKFB4 | 0.106983 | 0.0192 | -2.15583 | 2.023682 |
| IGDCC4 | 0.107035 | 0.019218 | 1.690993 | 3.317028 |
| PIAS3 | 0.10715 | 0.019247 | -0.92754 | 6.426778 |
| KAZALD1 | 0.107174 | 0.019259 | 2.069174 | 2.389996 |
| HIF1A-AS2 | 0.107174 | 0.019267 | -2.20402 | 2.720263 |
| ZNF208 | 0.107307 | 0.019299 | 2.04503 | 3.373774 |
| LOC10140[illegible] | 0.107367 | 0.019318 | 2.681072 | 1.34442 |
| CAPZA1 | 0.107367 | 0.019326 | -0.99473 | 7.664975 |
| PLAT | 0.107475 | 0.019354 | -3.83045 | 1.632318 |
| RNF133 | 0.107526 | 0.019371 | 2.537513 | 1.504869 |
| LOC28507[illegible] | 0.107608 | 0.019394 | -0.9219 | 5.78471 |
| LOC10272[illegible] | 0.107632 | 0.019407 | -1.7903 | 2.853407 |
| RAB20 | 0.107779 | 0.019442 | 2.087015 | 4.123382 |
| KIAA1211L | 0.108311 | 0.019546 | -0.94552 | 5.412142 |
| CFAP57 | 0.108353 | 0.019562 | 2.809713 | 2.108994 |
| RTL8B | 0.108479 | 0.019593 | -1.31594 | 3.589821 |
| STK24 | 0.108494 | 0.019604 | -1.45455 | 3.318508 |
| AQP1 | 0.10859 | 0.019634 | 2.194198 | 2.828085 |
| ITPR3 | 0.10859 | 0.019641 | 0.954158 | 8.872883 |
| BTBD16 | 0.10859 | 0.019646 | 2.610723 | 2.513942 |
| TMEM52 | 0.10859 | 0.019654 | 3.602037 | 0.714138 |
| TSTD2 | 0.108815 | 0.019703 | 1.069135 | 4.469446 |
| ASF1B | 0.109079 | 0.019759 | 1.487873 | 2.742405 |
| COA3 | 0.109188 | 0.019788 | -1.07354 | 4.621931 |
| SELE | 0.109188 | 0.019796 | 2.670298 | 2.716808 |
| FAM222B | 0.109207 | 0.019807 | 2.049936 | 1.478687 |
| MTMR1 | 0.109246 | 0.019823 | -1.11222 | 4.385562 |
| FZD8 | 0.109419 | 0.019862 | 2.29225 | 2.564229 |
| LINC02297 | 0.109608 | 0.019905 | -6.57954 | 0.542184 |
| DRAIC | 0.10965 | 0.019925 | -4.32328 | 0.738318 |

| | | | | |
|---|---|---|---|---|
| RPE65 | 0.10965 | 0.019934 | -2.83446 | 3.125821 |
| VTI1B | 0.10965 | 0.019938 | -1.02286 | 5.689009 |
| TNS2 | 0.109728 | 0.01996 | 2.386575 | 1.905333 |
| COX19 | 0.109808 | 0.019983 | 0.910261 | 5.402091 |
| DAD1 | 0.10987 | 0.020003 | -0.84652 | 5.904913 |
| ZNF681 | 0.109974 | 0.02003 | -1.55161 | 3.789686 |
| PGM1 | 0.11017 | 0.020076 | -1.24882 | 3.616582 |
| PCDHB11 | 0.11017 | 0.020083 | 2.790023 | 1.735024 |
| C18orf25 | 0.110191 | 0.020102 | -2.99866 | 1.580173 |
| ECHS1 | 0.110191 | 0.020103 | -0.96432 | 6.060043 |
| LINC00926 | 0.110269 | 0.020126 | 2.026779 | 2.154816 |
| PALD1 | 0.110499 | 0.020182 | 1.426338 | 4.858019 |
| UCA1 | 0.110499 | 0.020189 | 2.729565 | 4.374939 |
| ADCY7 | 0.110499 | 0.020193 | -0.92141 | 6.075403 |
| IGSF22 | 0.110769 | 0.020266 | 1.991423 | 1.984001 |
| ZNF469 | 0.110769 | 0.020266 | 2.300251 | 4.899841 |
| LINC00467 | 0.110769 | 0.020268 | 1.853682 | 2.880321 |
| SF3B6 | 0.111264 | 0.020371 | -1.02037 | 5.748359 |
| TMSB4X | 0.111264 | 0.020376 | 0.91643 | 8.698797 |
| LINC00612 | 0.111319 | 0.020394 | 2.600582 | 1.280995 |
| CARD18 | 0.111353 | 0.020415 | -3.97857 | 0.580113 |
| PEAR1 | 0.111353 | 0.020417 | 1.237952 | 3.908062 |
| ZNF710 | 0.111355 | 0.020426 | -0.89046 | 5.875955 |
| GAL | 0.11142 | 0.020453 | 3.273118 | 1.005648 |
| LINC00674 | 0.11142 | 0.020455 | 0.896025 | 7.048896 |
| RNF220 | 0.111621 | 0.0205 | 0.918292 | 5.482713 |
| CCDC136 | 0.111776 | 0.020541 | 1.961321 | 3.351635 |
| PLSCR1 | 0.111776 | 0.020547 | 1.018942 | 6.487941 |
| ACP6 | 0.111776 | 0.020555 | 1.37571 | 4.499692 |
| SLC7A5P2 | 0.111842 | 0.020575 | 1.111171 | 6.239563 |
| PDK2 | 0.112045 | 0.020621 | 3.456992 | 1.124082 |
| CCDC141 | 0.112127 | 0.020652 | 2.08515 | 3.722065 |
| CEP126 | 0.112127 | 0.020653 | 1.851625 | 4.487268 |
| LOC646471 | 0.112307 | 0.020695 | -1.30869 | 3.974691 |
| ZNF783 | 0.112461 | 0.020732 | 1.018439 | 4.852859 |
| SPINK1 | 0.112461 | 0.02074 | 2.969345 | 4.896649 |
| LRRTM2 | 0.112928 | 0.020835 | -0.99608 | 6.001944 |
| RP9P | 0.113016 | 0.02086 | 1.091224 | 4.283524 |
| SRSF10 | 0.113037 | 0.020872 | 1.287907 | 4.581974 |
| DIO3 | 0.113038 | 0.020881 | 3.72008 | 1.253147 |
| XACT | 0.113214 | 0.020922 | 6.471765 | 0.502956 |
| CRIM1 | 0.113405 | 0.020966 | 0.872238 | 7.419652 |
| SLC2A10 | 0.113577 | 0.021007 | 0.981373 | 5.184051 |
| LOC10192[illegible] | 0.113635 | 0.021026 | 2.548197 | 3.467322 |
| MAP3K20-[illegible] | 0.113754 | 0.02106 | 1.647153 | 3.281621 |

| | | | | |
|---|---|---|---|---|
| SNX29P2 | 0.113754 | 0.021065 | 1.969369 | 2.621291 |
| AKAP8 | 0.114026 | 0.021125 | 0.952776 | 6.524382 |
| RAC1 | 0.114211 | 0.021175 | -2.24127 | 2.141897 |
| AXL | 0.114211 | 0.021176 | -2.93802 | 1.46551 |
| ADGRF1 | 0.114263 | 0.021195 | -1.87913 | 4.809135 |
| LINC01359 | 0.114353 | 0.021223 | 2.491738 | 2.305367 |
| PRRX2 | 0.114353 | 0.021231 | 2.371976 | 2.106915 |
| RGS3 | 0.114353 | 0.021238 | 1.880167 | 2.506733 |
| CSK | 0.114501 | 0.021275 | -1.60537 | 2.266516 |
| ITIH6 | 0.114501 | 0.021288 | 5.12206 | 0.501773 |
| FAM3D | 0.114501 | 0.021291 | -2.25134 | 5.898911 |
| ATP1A2 | 0.11453 | 0.021305 | 1.888583 | 4.647865 |
| MEDAG | 0.11463 | 0.021333 | 2.454273 | 5.223243 |
| PLA2G4E | 0.11463 | 0.021348 | -3.29459 | 4.256648 |
| CRIP2 | 0.11463 | 0.021355 | -1.40151 | 4.674944 |
| LOC101929 | 0.11463 | 0.021359 | -2.45342 | 1.307487 |
| CD2 | 0.114893 | 0.021416 | 1.825081 | 3.298151 |
| LOC100505 | 0.114998 | 0.021445 | -1.14087 | 4.53576 |
| ARHGAP6 | 0.114998 | 0.021454 | 2.924632 | 1.759003 |
| GJA1 | 0.115245 | 0.021508 | -1.28505 | 7.538361 |
| CYTOR | 0.115611 | 0.021586 | 1.395169 | 3.665485 |
| FAM214A | 0.115641 | 0.021603 | -1.61761 | 2.857209 |
| FRG1CP | 0.115641 | 0.021609 | 1.112564 | 4.599271 |
| SNORD8 | 0.115774 | 0.021642 | 1.490506 | 8.611315 |
| TYRP1 | 0.115969 | 0.021688 | 3.043502 | 1.756903 |
| MSANTD4 | 0.115986 | 0.0217 | 1.375977 | 5.132876 |
| RBMS2 | 0.116552 | 0.021815 | 0.884653 | 6.763767 |
| WNK1 | 0.116793 | 0.021869 | -1.35795 | 4.158513 |
| STAT5B | 0.117063 | 0.021928 | 0.855407 | 6.305935 |
| MT1X | 0.117307 | 0.021983 | -1.25578 | 5.406235 |
| DLGAP1-AS | 0.117401 | 0.022009 | 3.132292 | 1.41177 |
| ARHGEF25 | 0.117478 | 0.022033 | 3.463544 | 1.884477 |
| LHCGR | 0.117597 | 0.02207 | 3.86896 | 0.940824 |
| EFNA2 | 0.117597 | 0.022073 | 3.576311 | 1.66161 |
| PDGFRA | 0.117836 | 0.02213 | 1.098383 | 7.290265 |
| KIAA1614 | 0.117836 | 0.022149 | 1.687491 | 2.78746 |
| LINC00271 | 0.117836 | 0.022151 | 3.653407 | 1.47179 |
| SPRR2D | 0.117836 | 0.022154 | -3.71112 | 6.967496 |
| HADHA | 0.117902 | 0.022175 | -0.85752 | 7.811147 |
| LOC101927 | 0.118031 | 0.022208 | 3.773853 | 0.857616 |
| LRFN5 | 0.118049 | 0.022221 | 2.367889 | 1.477955 |
| ZBED5-AS1 | 0.118092 | 0.022238 | -1.73875 | 1.885161 |
| CENPV | 0.118144 | 0.022257 | 1.718281 | 3.321695 |
| SNORD12 | 0.118176 | 0.022272 | 1.395705 | 6.293984 |
| SOD2 | 0.118287 | 0.022302 | 1.388496 | 7.843849 |

| | | | | |
|---|---|---|---|---|
| TRIM21 | 0.11856 | 0.022362 | 1.133617 | 4.857662 |
| HMGCS2 | 0.11865 | 0.022388 | 4.072045 | 0.275749 |
| IGFBP2 | 0.118696 | 0.022406 | -1.57012 | 3.273078 |
| CYP4F62P | 0.118712 | 0.022423 | 6.363796 | 0.424448 |
| CXCL16 | 0.118712 | 0.022427 | 1.113115 | 4.509966 |
| LONRF2 | 0.118737 | 0.022441 | 2.369685 | 5.900494 |
| TRIM2 | 0.118887 | 0.022478 | 3.248624 | 1.833357 |
| A2M-AS1 | 0.118968 | 0.022502 | 2.372035 | 1.991324 |
| QSOX2 | 0.119073 | 0.022531 | 0.953842 | 4.847277 |
| PI4K2A | 0.119096 | 0.022546 | -0.93679 | 5.854575 |
| PIM2 | 0.119096 | 0.022554 | 1.523599 | 5.440951 |
| CCDC6 | 0.119103 | 0.022564 | -0.91739 | 8.112617 |
| DENND2D | 0.119568 | 0.022662 | -1.63106 | 2.262149 |
| DLGAP4 | 0.119659 | 0.022688 | 1.105405 | 4.328963 |
| PDE1A | 0.119673 | 0.0227 | 2.877099 | 2.185271 |
| SAMD4A | 0.119914 | 0.022755 | 3.336526 | 0.512373 |
| IGF2 | 0.120133 | 0.02282 | 3.189011 | 0.797902 |
| GNG12 | 0.120133 | 0.022823 | -0.92765 | 7.770081 |
| SCARNA9 | 0.120133 | 0.022834 | 0.963359 | 6.767238 |
| DNALI1 | 0.120133 | 0.022841 | 2.064755 | 3.964682 |
| LINC01989 | 0.120133 | 0.022848 | 4.69894 | 0.214373 |
| SNORD50A | 0.120133 | 0.022851 | 1.627529 | 3.1849 |
| KIZ-AS1 | 0.12037 | 0.022905 | 2.11074 | 2.452577 |
| HEXB | 0.120712 | 0.02298 | 1.604699 | 2.622042 |
| CCDC69 | 0.120745 | 0.022995 | 1.022917 | 5.772461 |
| PECR | 0.121516 | 0.023154 | 1.540392 | 3.017612 |
| LOC103908 | 0.121516 | 0.02316 | -3.26357 | 0.576599 |
| ZNF280C | 0.121593 | 0.023184 | 1.048407 | 4.232621 |
| OR51I1 | 0.121692 | 0.023213 | 6.325937 | 0.397277 |
| SNORD116 | 0.12172 | 0.023235 | 1.763402 | 3.750968 |
| KIRREL3 | 0.12172 | 0.023248 | -4.78483 | 0.513857 |
| SMAP2 | 0.12172 | 0.023252 | 1.131759 | 4.902351 |
| S1PR1 | 0.12172 | 0.023255 | 1.374033 | 4.439296 |
| ANO6 | 0.121738 | 0.023268 | 2.092724 | 2.004141 |
| ABCD1 | 0.121906 | 0.023309 | 1.395378 | 3.644019 |
| SNORA80E | 0.122476 | 0.023428 | 1.239319 | 8.100631 |
| MAST1 | 0.122852 | 0.023509 | 2.772654 | 1.405779 |
| ETHE1 | 0.122852 | 0.023518 | -1.00877 | 4.712665 |
| CYBA | 0.123043 | 0.023564 | 1.124284 | 5.337093 |
| FDPSP2 | 0.123255 | 0.023614 | -0.85931 | 5.964019 |
| SPHK1 | 0.123266 | 0.02363 | -2.77217 | 0.75349 |
| CASD1 | 0.123266 | 0.023644 | 1.033963 | 5.166992 |
| TMEM170E | 0.123266 | 0.023644 | 1.356837 | 3.597362 |
| LINC01134 | 0.123331 | 0.023666 | 3.595634 | 1.094142 |
| DVL1 | 0.123331 | 0.023675 | -0.90792 | 6.543889 |

| | | | | |
|---|---|---|---|---|
| CLMAT3 | 0.123491 | 0.023716 | 2.057613 | 1.499354 |
| IFNE | 0.123643 | 0.023755 | -2.55563 | 2.608616 |
| SEMA4B | 0.123643 | 0.023764 | -1.76065 | 2.144168 |
| RPS6KA2-IT | 0.123709 | 0.023786 | 2.329305 | 1.641443 |
| TIGAR | 0.123819 | 0.023816 | 0.913315 | 5.208497 |
| SHISA2 | 0.123898 | 0.023841 | 3.782797 | 0.894206 |
| ZSCAN23 | 0.123899 | 0.02386 | 1.607173 | 2.729597 |
| SEC14L5 | 0.123899 | 0.02386 | -2.0027 | 2.34766 |
| GLO1 | 0.123923 | 0.023874 | -1.02031 | 6.406127 |
| IER5 | 0.12393 | 0.023885 | -0.97737 | 6.525765 |
| TNFRSF12A | 0.124271 | 0.02396 | 1.328441 | 5.101469 |
| MGAM2 | 0.124598 | 0.024043 | 4.045897 | 2.559417 |
| MAFK | 0.124598 | 0.024047 | 1.044351 | 5.664525 |
| NYNRIN | 0.124598 | 0.024061 | -0.89857 | 5.921884 |
| ITGAX | 0.124598 | 0.024061 | 2.530623 | 1.735143 |
| SNORD114 | 0.124599 | 0.024071 | 2.51746 | 1.764708 |
| TWSG1 | 0.124865 | 0.024132 | 0.945856 | 5.758739 |
| MMD | 0.124923 | 0.024153 | 1.504305 | 3.644075 |
| SLC2A3 | 0.124928 | 0.024163 | 1.493074 | 6.16188 |
| TPP1 | 0.12511 | 0.024208 | 0.881938 | 6.84737 |
| HMGN4 | 0.125114 | 0.024218 | 0.875301 | 5.295003 |
| DNAH3 | 0.125226 | 0.024249 | 1.922737 | 3.123859 |
| CNPPD1 | 0.125304 | 0.024274 | -0.8364 | 6.073549 |
| ULK1 | 0.125431 | 0.024309 | -0.83075 | 6.455175 |
| GAS6-AS1 | 0.125431 | 0.024335 | 1.749412 | 3.499019 |
| STS | 0.125431 | 0.024335 | 1.150487 | 4.734504 |
| LOC101929 | 0.125431 | 0.024347 | 1.158564 | 4.83621 |
| ANGPTL4 | 0.125431 | 0.024349 | -3.35402 | 1.034838 |
| TBC1D8B | 0.125431 | 0.024356 | 1.065662 | 4.405432 |
| ZNF432 | 0.125539 | 0.024386 | 1.055243 | 5.204298 |
| LINC00964 | 0.125625 | 0.024413 | -2.93712 | 2.204736 |
| POFUT2 | 0.125996 | 0.024494 | 1.649821 | 2.393971 |
| MIR142 | 0.126089 | 0.024522 | 3.081888 | 1.03407 |
| UQCRFS1 | 0.126109 | 0.024536 | -0.87074 | 5.583643 |
| PRKCA-AS1 | 0.126207 | 0.024564 | 1.473645 | 2.997384 |
| NRBP1 | 0.126424 | 0.024616 | -0.86549 | 7.641496 |
| GIMAP7 | 0.126778 | 0.024695 | 1.673169 | 4.260588 |
| USP32 | 0.12702 | 0.024752 | -0.90847 | 7.198123 |
| MIDN | 0.127074 | 0.024772 | -0.93133 | 7.846074 |
| FHL1 | 0.127263 | 0.024821 | 2.988723 | 1.787119 |
| SNORD2 | 0.127263 | 0.024831 | 1.190588 | 4.432987 |
| IFITM2 | 0.127263 | 0.024839 | 1.81749 | 5.458982 |
| EHF | 0.127263 | 0.024847 | -1.91197 | 3.003102 |
| OXSR1 | 0.12739 | 0.024887 | -0.97426 | 7.780226 |
| GJD3 | 0.12739 | 0.024892 | -1.04857 | 4.004172 |

| | | | | |
|---|---|---|---|---|
| PIGR | 0.127792 | 0.02498 | 3.539509 | 9.35826 |
| PARGP1 | 0.127816 | 0.024994 | 0.949213 | 4.728043 |
| MAML3 | 0.127949 | 0.025039 | -0.89568 | 6.111731 |
| ZDHHC23 | 0.127949 | 0.02504 | 1.491799 | 4.586431 |
| WDR27 | 0.128074 | 0.025074 | -1.394 | 3.928241 |
| RASEF | 0.128147 | 0.025098 | 0.996349 | 6.898532 |
| FMOD | 0.128153 | 0.025109 | 1.962493 | 5.604697 |
| AP3S1 | 0.128509 | 0.025189 | -0.95918 | 6.051437 |
| TACR1 | 0.128546 | 0.025206 | 1.76788 | 2.476372 |
| ZBTB5 | 0.128769 | 0.025268 | -0.86733 | 5.973205 |
| PCDHB10 | 0.128769 | 0.025269 | 2.453306 | 2.340604 |
| BRF2 | 0.128993 | 0.025325 | 1.441432 | 3.475523 |
| RTL5 | 0.128993 | 0.025333 | 1.524066 | 3.802773 |
| BMI1 | 0.129021 | 0.025348 | 2.073975 | 2.167981 |
| SIX1 | 0.129492 | 0.025451 | 2.750502 | 2.161526 |
| UNC5B-AS1 | 0.129492 | 0.02546 | -1.86589 | 2.686782 |
| PPP1R1A | 0.129673 | 0.025506 | 3.752276 | 2.347587 |
| UBE2E2 | 0.129728 | 0.025526 | -1.12426 | 4.679717 |
| GNG11 | 0.129732 | 0.025537 | 1.328307 | 3.226302 |
| TIGIT | 0.129964 | 0.0256 | 1.689593 | 2.355492 |
| LIMD2 | 0.129964 | 0.025605 | 1.696999 | 3.506689 |
| LOC10013[illegible] | 0.129964 | 0.025613 | 3.128512 | 0.719449 |
| MYLK4 | 0.129997 | 0.025629 | -1.79886 | 2.563772 |
| A4GNT | 0.130203 | 0.02568 | 3.973095 | 2.496971 |
| CBR3-AS1 | 0.130229 | 0.025694 | -1.87448 | 2.559597 |
| TFF1 | 0.130326 | 0.025723 | 3.326418 | 6.378032 |
| ADRA2B | 0.130555 | 0.025779 | 2.61625 | 1.313867 |
| CCDC162P | 0.130598 | 0.025797 | 2.33616 | 2.552541 |
| NLGN1 | 0.130841 | 0.025861 | 2.743481 | 2.116058 |
| ITGA4 | 0.130841 | 0.025874 | 1.561342 | 4.853867 |
| CS | 0.130841 | 0.025877 | -0.87287 | 7.51728 |
| ID2 | 0.130841 | 0.025885 | 1.233479 | 4.804742 |
| ZSWIM4 | 0.130928 | 0.025912 | -0.91497 | 6.247975 |
| CTPS2 | 0.131254 | 0.025987 | 1.934603 | 1.652352 |
| FNDC3B | 0.131279 | 0.026008 | 1.769688 | 2.379994 |
| SNORD116 | 0.131279 | 0.026016 | 1.62617 | 5.422776 |
| UNC5C | 0.131279 | 0.026036 | 1.786208 | 3.572097 |
| 1-Sep | 0.131279 | 0.026041 | 1.369857 | 3.965038 |
| SNORD116 | 0.131279 | 0.02605 | 1.982031 | 4.188364 |
| ZNF333 | 0.131279 | 0.026058 | 1.565445 | 3.597574 |
| TRAK1 | 0.131279 | 0.026062 | 0.834873 | 6.110375 |
| KLK8 | 0.131389 | 0.026095 | -3.533 | 0.928609 |
| RBFOX3 | 0.131389 | 0.026104 | 1.967555 | 2.153274 |
| FAT3 | 0.131452 | 0.026126 | 3.106836 | 4.014299 |
| DARS-AS1 | 0.131656 | 0.026177 | -3.40096 | 1.047813 |

| | | | | |
|---|---|---|---|---|
| SPINK13 | 0.13173 | 0.026202 | 4.49423 | 0.559233 |
| DKFZP434A | 0.13184 | 0.026233 | 4.144418 | 0.675449 |
| CEP112 | 0.131918 | 0.026259 | 2.123196 | 1.386529 |
| CLVS1 | 0.13203 | 0.026294 | -2.84865 | 1.672625 |
| DUSP1 | 0.13203 | 0.026301 | -1.24351 | 8.74218 |
| NPTX2 | 0.132236 | 0.026359 | 2.401162 | 2.431982 |
| PRO1804 | 0.132236 | 0.026363 | -1.6214 | 2.713456 |
| IPMK | 0.132363 | 0.026398 | -0.93601 | 5.73043 |
| PCDHB14 | 0.13243 | 0.026421 | 1.722256 | 4.309975 |
| RRAS | 0.132443 | 0.026434 | 1.17679 | 4.028361 |
| ODAM | 0.13252 | 0.02646 | 3.676226 | 1.613313 |
| TMEM30B | 0.13273 | 0.026512 | -1.0739 | 6.113094 |
| ADAMTS9-. | 0.132806 | 0.026542 | 2.06319 | 2.537208 |
| RNF180 | 0.132806 | 0.026553 | 2.315302 | 2.864897 |
| KRTAP12-3 | 0.132806 | 0.026561 | 6.077137 | 0.236319 |
| TENM3 | 0.132806 | 0.02658 | -1.74223 | 5.430831 |
| KRTAP10-4 | 0.132806 | 0.026584 | 6.077134 | 0.236319 |
| SNORD117 | 0.132806 | 0.026587 | 1.695726 | 3.152734 |
| CAPNS1 | 0.132862 | 0.026609 | -1.68523 | 1.574977 |
| LOC100422 | 0.133125 | 0.026672 | 6.097902 | 0.237663 |
| FEM1B | 0.133551 | 0.026767 | -0.83855 | 7.519991 |
| WFDC21P | 0.133622 | 0.026807 | -2.4765 | 4.599243 |
| LINC01061 | 0.133622 | 0.02681 | -1.16599 | 3.628236 |
| PTCHD1 | 0.133622 | 0.026812 | 3.423252 | 1.06257 |
| SERPINB5 | 0.133671 | 0.026832 | -1.71819 | 8.737305 |
| SUSD5 | 0.13385 | 0.02688 | 2.587633 | 2.536298 |
| ADCY3 | 0.13385 | 0.026906 | 0.84211 | 5.61263 |
| EFS | 0.13385 | 0.026908 | -2.16862 | 1.489168 |
| RAP2B | 0.13385 | 0.026909 | -0.97751 | 7.695571 |
| DENND4B | 0.134082 | 0.026966 | 0.864835 | 6.028547 |
| PDX1 | 0.134268 | 0.027013 | 2.445843 | 3.017115 |
| ZNF213-AS | 0.134358 | 0.027042 | 1.548306 | 2.939981 |
| RNF148 | 0.134371 | 0.027054 | 2.562689 | 1.764027 |
| TSPAN14 | 0.134403 | 0.027071 | -1.24632 | 3.497229 |
| AKR1B10 | 0.134726 | 0.027146 | -2.00799 | 6.700604 |
| ZNF579 | 0.135061 | 0.027224 | 1.444815 | 3.135902 |
| IL17RB | 0.135529 | 0.027329 | 1.365838 | 3.926349 |
| RAB23 | 0.135533 | 0.02734 | 2.929489 | 0.633246 |
| SRRM2-AS1 | 0.136043 | 0.027458 | -1.98646 | 2.292717 |
| SLC45A1 | 0.136043 | 0.027464 | 2.481791 | 1.885991 |
| SPAG1 | 0.136079 | 0.027481 | 3.280663 | 0.302375 |
| ZNF445 | 0.136085 | 0.027493 | 0.929862 | 6.386685 |
| HAND2 | 0.136754 | 0.027638 | 3.321355 | 2.575357 |
| FOXJ1 | 0.13752 | 0.027804 | 2.998144 | 2.61244 |
| PRSS33 | 0.137729 | 0.027856 | 4.305631 | 2.266278 |

| | | | | |
|---|---|---|---|---|
| PLEKHA8P1 | 0.13775 | 0.027871 | 1.713547 | 2.727663 |
| PLIN4 | 0.13791 | 0.027919 | 1.560236 | 3.896493 |
| SHC2 | 0.13791 | 0.027925 | 1.695642 | 3.687646 |
| PDCL3P4 | 0.137986 | 0.027952 | 1.436988 | 3.175165 |
| ZNF624 | 0.137986 | 0.027969 | -1.41153 | 4.171768 |
| COMMD8 | 0.137986 | 0.027972 | -1.50133 | 3.966577 |
| HNMT | 0.138525 | 0.028094 | 1.993959 | 3.85089 |
| GLUL | 0.138525 | 0.028111 | -1.16106 | 5.572538 |
| TRABD2B | 0.138525 | 0.028113 | 1.72636 | 2.606752 |
| ATP1B3 | 0.138615 | 0.028142 | -1.0593 | 7.151798 |
| PGK1 | 0.138615 | 0.028158 | -0.99271 | 8.251201 |
| PCDHB12 | 0.138615 | 0.028162 | 2.80483 | 1.499469 |
| RBMX | 0.138625 | 0.028185 | 0.761805 | 5.737791 |
| ZNF223 | 0.138625 | 0.028186 | 1.349783 | 3.758203 |
| B3GNT7 | 0.138711 | 0.028217 | 1.587388 | 4.426472 |
| DOCK9-AS1 | 0.138711 | 0.028224 | -2.11508 | 1.577241 |
| PHACTR4 | 0.13878 | 0.028259 | -1.16268 | 4.001514 |
| NR2F6 | 0.13878 | 0.028259 | -0.95921 | 5.549418 |
| ADAP2 | 0.138798 | 0.028274 | 1.250053 | 3.67998 |
| RNF150 | 0.139003 | 0.028326 | 1.55035 | 3.570018 |
| FRMD8P1 | 0.139134 | 0.028363 | 4.870879 | 0.654619 |
| ITK | 0.139213 | 0.02839 | 1.320991 | 3.978666 |
| FBXW4P1 | 0.139454 | 0.02845 | -1.31082 | 4.224957 |
| KRT14 | 0.139468 | 0.028463 | -2.91746 | 9.840825 |
| RBM12B | 0.139484 | 0.028477 | 0.832625 | 6.12535 |
| PPP1CB | 0.139565 | 0.028504 | -1.45508 | 2.654299 |
| LINC00840 | 0.139903 | 0.028584 | 4.672234 | 0.903403 |
| TMEM8B | 0.139927 | 0.0286 | 1.739198 | 1.984643 |
| CNRIP1 | 0.139966 | 0.028618 | 3.058049 | 1.29526 |
| UPB1 | 0.140209 | 0.028679 | 4.442534 | 0.353624 |
| CLDN1 | 0.140504 | 0.02875 | -1.04099 | 6.529245 |
| MYRFL | 0.140504 | 0.02876 | 3.126468 | 1.230434 |
| KDF1 | 0.140959 | 0.028865 | -1.50214 | 3.791584 |
| RPL7A | 0.140959 | 0.028875 | -0.90838 | 8.838269 |
| VSTM4 | 0.14105 | 0.028907 | 1.411865 | 4.001432 |
| KLRA1P | 0.14105 | 0.028915 | 1.83835 | 2.74186 |
| FOXF2 | 0.141281 | 0.028973 | 1.660446 | 3.457642 |
| ZDHHC17 | 0.141505 | 0.02903 | 0.844119 | 5.584319 |
| ANKRD36B | 0.141569 | 0.029054 | 1.894042 | 4.36474 |
| ZBTB46 | 0.141775 | 0.029113 | 1.269358 | 3.634749 |
| RPL35 | 0.141775 | 0.029125 | -0.85354 | 8.546554 |
| PRKX | 0.141775 | 0.029129 | -1.02668 | 6.215343 |
| FIGNL2 | 0.142011 | 0.029188 | 2.894903 | 0.866523 |
| MRAP2 | 0.142308 | 0.02926 | 2.455376 | 2.55519 |
| PRAM1 | 0.142377 | 0.029285 | 2.277853 | 1.379425 |

| | | | | |
|---|---|---|---|---|
| LINC00466 | 0.142485 | 0.029326 | 3.695739 | 0.553668 |
| ZNF714 | 0.142485 | 0.029335 | -1.1746 | 4.709025 |
| RHBDD1 | 0.142485 | 0.02934 | 2.936935 | 0.747443 |
| NPIPB13 | 0.1429 | 0.029436 | 0.926571 | 7.954609 |
| MIR6887 | 0.14291 | 0.029451 | -3.17975 | 1.209269 |
| GARS-DT | 0.14291 | 0.02946 | 0.974933 | 5.309355 |
| NPSR1-AS1 | 0.143005 | 0.029493 | 5.37661 | 1.281621 |
| RTN1 | 0.143005 | 0.029501 | 2.473188 | 1.16766 |
| FAM214B | 0.14315 | 0.029542 | -2.32606 | 0.994292 |
| RHOD | 0.143483 | 0.029622 | -1.91936 | 2.102784 |
| SKINT1L | 0.143507 | 0.029637 | 2.978921 | 1.32625 |
| IMP3 | 0.143606 | 0.02967 | -1.16625 | 5.016537 |
| SH2B3 | 0.143606 | 0.029691 | 2.160563 | 2.1275 |
| G0S2 | 0.143606 | 0.029709 | 2.22221 | 4.336793 |
| DOCK4 | 0.143606 | 0.02971 | 0.852203 | 6.038717 |
| GUSBP11 | 0.143606 | 0.029713 | 1.007949 | 5.276133 |
| PPP4R1 | 0.143998 | 0.029805 | -1.33896 | 2.952951 |
| IGFBP5 | 0.144494 | 0.029918 | 1.330214 | 9.805709 |
| LOC10192¢ | 0.144505 | 0.029937 | 2.578653 | 0.732271 |
| HSPBAP1 | 0.144505 | 0.029943 | 1.021916 | 3.948556 |
| ABCA13 | 0.144564 | 0.029966 | -1.6423 | 4.510674 |
| RPL37 | 0.144587 | 0.029982 | -0.84919 | 8.406722 |
| EMP3 | 0.144787 | 0.030045 | 2.0372 | 1.543458 |
| ABCB1 | 0.144787 | 0.030045 | 1.750956 | 4.21174 |
| ZNF704 | 0.144946 | 0.030089 | 1.344692 | 5.879462 |
| MIR614 | 0.145032 | 0.030141 | 2.140139 | 2.53652 |
| GPRIN2 | 0.145032 | 0.030141 | -1.46513 | 2.42562 |
| FBXO34 | 0.145032 | 0.030154 | -1.28372 | 3.630698 |
| TMEM141 | 0.145032 | 0.030154 | -0.92563 | 4.818865 |
| ALDH3A1 | 0.145032 | 0.030162 | -2.32952 | 1.178515 |
| TSPAN12 | 0.145052 | 0.030178 | 2.087591 | 3.693626 |
| IL17D | 0.145065 | 0.030192 | 2.187756 | 1.137321 |
| BEST4 | 0.145065 | 0.030202 | 2.96677 | 0.822476 |
| ARFGAP1 | 0.145193 | 0.03024 | 1.970064 | 1.749968 |
| POU3F3 | 0.145262 | 0.030266 | 3.672364 | 1.092638 |
| INSYN1 | 0.145407 | 0.030308 | -2.35175 | 0.800838 |
| MESD | 0.145407 | 0.030318 | -1.01995 | 4.535508 |
| TMEM240 | 0.145767 | 0.030409 | 2.631975 | 1.12609 |
| LOC10050€ | 0.145767 | 0.030415 | 1.94662 | 2.778597 |
| COBLL1 | 0.146061 | 0.030504 | -2.83684 | 1.447457 |
| LOC642852 | 0.146061 | 0.03053 | 1.00477 | 4.865744 |
| SNORD116 | 0.146061 | 0.030544 | 1.703507 | 4.291175 |
| RNASET2 | 0.146061 | 0.030552 | 0.985032 | 5.359006 |
| NPIPB5 | 0.146061 | 0.030554 | 0.978496 | 7.037647 |
| LOC55420€ | 0.146061 | 0.030561 | 2.967349 | 0.629714 |

| | | | | |
|---|---|---|---|---|
| LOC101928 | 0.146061 | 0.030566 | -1.87469 | 2.656373 |
| SRSF9 | 0.146061 | 0.030577 | -0.86853 | 6.102449 |
| CLEC14A | 0.146061 | 0.030577 | 1.47464 | 2.947444 |
| CNOT1 | 0.146337 | 0.030646 | -1.76283 | 2.760794 |
| CYP4X1 | 0.146404 | 0.030671 | -1.50812 | 4.416186 |
| EXOC5 | 0.146589 | 0.030721 | -0.79936 | 6.997517 |
| NTRK3 | 0.14693 | 0.030803 | 3.862367 | 0.952286 |
| FAM49A | 0.147194 | 0.030881 | 1.252193 | 4.096202 |
| DCDC2C | 0.147194 | 0.030882 | 3.213011 | 1.143209 |
| HIST2H2AE | 0.147194 | 0.030893 | 1.106227 | 4.090337 |
| SH3GL3 | 0.147403 | 0.030954 | -3.87389 | 0.82195 |
| COX4I1 | 0.147403 | 0.030959 | -0.8104 | 7.816559 |
| INHBA | 0.147416 | 0.030976 | 2.543011 | 5.934994 |
| WNT9A | 0.147416 | 0.030984 | -2.02969 | 1.721003 |
| LOC101928 | 0.147535 | 0.031025 | 3.721189 | 0.82396 |
| PTCHD4 | 0.147535 | 0.031032 | 1.998807 | 1.796387 |
| QRICH2 | 0.147568 | 0.03105 | -1.51243 | 3.314171 |
| HIST1H4A | 0.147691 | 0.031087 | -1.58001 | 3.267147 |
| USP42 | 0.148022 | 0.03117 | 0.866339 | 5.495621 |
| LOR | 0.148022 | 0.031179 | -2.06125 | 1.808517 |
| LINC00426 | 0.148259 | 0.031255 | 2.364154 | 1.258015 |
| EPHX1 | 0.148259 | 0.031263 | -1.88398 | 2.067827 |
| SHISA3 | 0.148259 | 0.031273 | 3.60253 | 1.499435 |
| CCNT2 | 0.148259 | 0.031274 | 2.209236 | 1.886137 |
| NUAK1 | 0.148295 | 0.031296 | 1.156327 | 4.151814 |
| HSPA7 | 0.148295 | 0.031305 | 1.44505 | 3.84497 |
| RIPK2 | 0.14836 | 0.03133 | 1.400452 | 4.353871 |
| PNPT1 | 0.148415 | 0.031367 | 0.779236 | 5.884761 |
| LDHA | 0.148415 | 0.031369 | -2.66832 | 2.434884 |
| FBXO30 | 0.148415 | 0.031375 | 0.910427 | 5.901728 |
| FCRL3 | 0.148417 | 0.031389 | 2.558111 | 1.506974 |
| EIF5A2 | 0.148417 | 0.031398 | 1.671538 | 3.434675 |
| MAP6 | 0.148689 | 0.031477 | 2.533361 | 1.911287 |
| LIPE | 0.148689 | 0.031478 | -1.06432 | 4.166661 |
| TTC41P | 0.148775 | 0.031508 | 2.600063 | 1.107689 |
| NBEAL2 | 0.148825 | 0.03153 | -1.09388 | 9.190202 |
| ELP5 | 0.149067 | 0.031592 | -1.56576 | 3.056873 |
| HLA-DQA1 | 0.149236 | 0.031658 | 1.973875 | 4.553749 |
| RASGRF2 | 0.149236 | 0.031661 | 1.272079 | 4.080472 |
| GATM | 0.149236 | 0.031668 | -0.98199 | 5.393508 |
| SF3A2 | 0.149236 | 0.031674 | 0.883326 | 5.613428 |
| OAF | 0.14933 | 0.031705 | 0.917983 | 5.083286 |
| PROX2 | 0.149384 | 0.031728 | 3.869843 | 0.725022 |
| CRISPLD2 | 0.149444 | 0.031752 | 1.252199 | 7.15551 |
| RUNDC1 | 0.149687 | 0.031821 | 0.797058 | 5.77183 |

| | | | | |
|---|---|---|---|---|
| ATP6AP1 | 0.149687 | 0.031826 | -0.88202 | 6.272791 |
| MYADM | 0.150318 | 0.031972 | 2.523218 | 2.575661 |
| RNF128 | 0.150333 | 0.031987 | 1.84997 | 2.982354 |
| LOC28441: | 0.150514 | 0.032037 | 1.525134 | 3.424382 |
| RAPGEF4-A | 0.150516 | 0.032049 | 3.332221 | 1.404354 |
| PATL2 | 0.150648 | 0.032088 | 2.041969 | 2.501049 |
| DMAC1 | 0.150665 | 0.032111 | -1.11196 | 3.766095 |
| ARHGEF39 | 0.150665 | 0.032115 | 1.490024 | 3.379972 |
| IRF1 | 0.150722 | 0.032138 | 1.478618 | 5.813895 |
| TRIM47 | 0.151057 | 0.032221 | 0.97702 | 4.400592 |
| NHLH1 | 0.151087 | 0.032239 | -2.27292 | 1.523215 |
| TCHP | 0.15124 | 0.032283 | -1.93377 | 1.646215 |
| TYK2 | 0.151319 | 0.032315 | 0.923515 | 6.411922 |
| TSPYL2 | 0.151319 | 0.032323 | 1.142249 | 6.585383 |
| GPR37 | 0.151781 | 0.032434 | -2.15147 | 2.093243 |
| NACA | 0.152043 | 0.032501 | -0.97967 | 4.542939 |
| AGMAT | 0.152247 | 0.032563 | 1.461202 | 2.972258 |
| SNORA25 | 0.152247 | 0.032568 | 0.853399 | 6.105874 |
| EMC3 | 0.15234 | 0.0326 | -0.82406 | 5.121279 |
| RFX3 | 0.152572 | 0.032661 | 2.092904 | 1.538439 |
| PRKD1 | 0.152601 | 0.032679 | 2.20725 | 3.181754 |
| TBC1D1 | 0.153022 | 0.03278 | 2.226703 | 1.925331 |
| WDR41 | 0.153069 | 0.032829 | -0.78981 | 6.071283 |
| WFDC12 | 0.153069 | 0.03283 | -3.43099 | 0.776454 |
| SNORD12C | 0.153069 | 0.032837 | 1.284124 | 5.072904 |
| CUBN | 0.153069 | 0.032844 | 2.102043 | 3.096158 |
| VANGL2 | 0.153069 | 0.032869 | -1.32645 | 5.60498 |
| SH3RF3-AS | 0.153069 | 0.032871 | 1.966812 | 2.162094 |
| CCDC8 | 0.153069 | 0.032872 | 2.06024 | 2.985389 |
| GIPC1 | 0.15332 | 0.032938 | -2.02587 | 1.808738 |
| SLC5A12 | 0.153408 | 0.032968 | 2.926368 | 0.328303 |
| CSGALNAC | 0.153531 | 0.033006 | 2.328203 | 2.088866 |
| NEPRO | 0.153904 | 0.033098 | 3.212525 | 0.616178 |
| FHAD1 | 0.154043 | 0.03314 | 2.167626 | 3.53849 |
| SMG1P3 | 0.154661 | 0.033285 | 0.903295 | 6.468752 |
| RAB8B | 0.154816 | 0.03333 | 0.946139 | 5.677427 |
| HCN2 | 0.154816 | 0.033342 | -1.96141 | 1.893735 |
| LINC00877 | 0.154856 | 0.033362 | 2.788956 | 1.483118 |
| NTMT1 | 0.155406 | 0.033493 | 3.091276 | 0.312247 |
| MYH7B | 0.155754 | 0.033579 | 1.23936 | 4.257113 |
| SLC7A5 | 0.155815 | 0.033621 | -0.93887 | 6.958583 |
| ADCY1 | 0.155815 | 0.033633 | 1.693067 | 3.353392 |
| MIR378J | 0.155815 | 0.033639 | 3.935905 | 0.665458 |
| FZD10-DT | 0.155815 | 0.03364 | -1.87259 | 2.884603 |
| MIR4778 | 0.155863 | 0.033679 | 4.933116 | 0.378344 |

| | | | | |
|---|---|---|---|---|
| IQGAP1 | 0.155863 | 0.033684 | -0.81535 | 10.04453 |
| HAS2 | 0.155863 | 0.033696 | 1.919896 | 3.207319 |
| IRAK3 | 0.155863 | 0.033702 | 2.600049 | 1.43755 |
| PFKP | 0.155863 | 0.03371 | -1.39563 | 3.611791 |
| FADS2 | 0.155911 | 0.033732 | 1.904092 | 1.58556 |
| SMIM5 | 0.156207 | 0.033808 | -1.13759 | 5.454744 |
| SPATA5 | 0.156874 | 0.033974 | 1.105881 | 4.276407 |
| RN7SL2 | 0.156874 | 0.033976 | -0.96137 | 14.55236 |
| PATJ | 0.156896 | 0.034001 | -0.83499 | 8.791844 |
| SNORD52 | 0.156896 | 0.034005 | 1.923209 | 2.329779 |
| ZC3H12D | 0.157056 | 0.034052 | 1.690411 | 2.767135 |
| LHFPL3-AS: | 0.157081 | 0.034069 | 3.093972 | 2.986728 |
| CYTIP | 0.157222 | 0.034112 | 1.487624 | 5.257757 |
| C3orf67-AS | 0.157222 | 0.034129 | -2.8872 | 2.394606 |
| DGKD | 0.157222 | 0.034135 | 2.568628 | 0.349707 |
| MARCO | 0.157341 | 0.034184 | 2.459964 | 2.057623 |
| FAM86B2 | 0.157341 | 0.034188 | 3.134051 | 0.374217 |
| LOC72874: | 0.157341 | 0.034197 | 1.405782 | 3.62652 |
| ATL1 | 0.157427 | 0.034228 | -4.46945 | 1.388399 |
| CD1D | 0.157629 | 0.034284 | 2.317872 | 1.721236 |
| WIF1 | 0.157698 | 0.034311 | 4.41042 | 3.4043 |
| CCDC159 | 0.157698 | 0.034328 | 1.094648 | 4.251688 |
| LOC10192ε | 0.157698 | 0.034335 | 2.688555 | 1.19712 |
| C1orf115 | 0.157747 | 0.034358 | 1.370778 | 4.859585 |
| TAS2R60 | 0.159005 | 0.034644 | -3.35989 | 0.525711 |
| SULT1C2P1 | 0.159185 | 0.034701 | 2.916453 | 1.010274 |
| ZBTB18 | 0.159185 | 0.034709 | 1.743105 | 3.409587 |
| COL27A1 | 0.159185 | 0.034719 | 0.986347 | 6.796932 |
| CDH5 | 0.159477 | 0.034805 | 1.054687 | 4.857233 |
| THEM4 | 0.159477 | 0.034807 | 1.176796 | 4.380056 |
| LOC10012ε | 0.159672 | 0.034862 | -0.91191 | 4.878396 |
| CLUAP1 | 0.159693 | 0.034879 | 1.745355 | 2.560747 |
| LOC10192ς | 0.159773 | 0.034908 | 2.569363 | 0.511854 |
| GOLGA6L5 | 0.159993 | 0.034972 | -1.33864 | 4.450533 |
| FSTL4 | 0.159993 | 0.034981 | -2.41591 | 2.017362 |
| MRGPRX3 | 0.160034 | 0.035002 | 2.832413 | 0.309687 |
| DUOX2 | 0.160154 | 0.035046 | -1.7447 | 7.536096 |
| SH3BGRL3 | 0.160154 | 0.035059 | -1.02713 | 7.65253 |
| PRRT3-AS1 | 0.160154 | 0.035079 | 2.383585 | 1.039523 |
| UGT1A6 | 0.160154 | 0.03508 | -1.96098 | 3.505154 |
| RTL8A | 0.160154 | 0.035089 | -1.17997 | 3.055284 |
| E2F2 | 0.160155 | 0.035102 | -1.27625 | 4.177948 |
| GPR39 | 0.160451 | 0.035179 | 1.695672 | 2.350133 |
| RP1L1 | 0.160505 | 0.035203 | -1.52864 | 3.079164 |
| PRKAR2A | 0.160682 | 0.035254 | -0.80444 | 6.390003 |

| | | | | |
|---|---|---|---|---|
| CEACAM8 | 0.161049 | 0.035347 | 3.427587 | 1.951672 |
| TM6SF2 | 0.161183 | 0.035389 | 2.9171 | 0.536678 |
| PCGF2 | 0.161538 | 0.035479 | 2.327051 | 1.956282 |
| LOC157273 | 0.161825 | 0.035557 | 4.01206 | 0.783756 |
| TEAD1 | 0.161825 | 0.035567 | -0.79222 | 7.858366 |
| KRTAP10-7 | 0.161965 | 0.03561 | 5.452346 | 0.629489 |
| UBE2B | 0.161995 | 0.035633 | -0.83455 | 5.757547 |
| TMEM140 | 0.161995 | 0.035641 | 1.452724 | 4.16039 |
| LOC285762 | 0.162298 | 0.03572 | 2.741477 | 1.064178 |
| HOOK3 | 0.162309 | 0.035735 | -0.80695 | 8.548818 |
| GFM1 | 0.162455 | 0.035779 | 2.999915 | 0.748286 |
| ERICH4 | 0.162595 | 0.035823 | 3.997689 | 0.571992 |
| OTOGL | 0.162857 | 0.035893 | 2.344753 | 1.766765 |
| AQP7 | 0.163034 | 0.035954 | 3.038775 | 2.051239 |
| ARHGDIB | 0.163034 | 0.03596 | 0.958999 | 5.608449 |
| EPCAM-DT | 0.163034 | 0.035969 | 3.409606 | 0.812458 |
| ADH1A | 0.163587 | 0.036103 | 2.677657 | 1.184404 |
| C2CD4A | 0.163666 | 0.03614 | 2.801001 | 1.358641 |
| LOC100130 | 0.163666 | 0.036146 | 1.567454 | 2.906571 |
| RFK | 0.163692 | 0.036164 | 1.435312 | 4.742061 |
| RAB1B | 0.163812 | 0.036203 | -0.77339 | 7.18508 |
| OST4 | 0.16386 | 0.036226 | -0.97499 | 5.22718 |
| UBLCP1 | 0.163867 | 0.03624 | -0.88129 | 5.289727 |
| GIMAP2 | 0.164034 | 0.03629 | 1.934832 | 2.715592 |
| SMG1P1 | 0.164034 | 0.03631 | 0.847436 | 6.578664 |
| ABHD17B | 0.164034 | 0.036315 | -1.66479 | 2.733203 |
| F2RL2 | 0.164071 | 0.03635 | 4.828916 | 0.807629 |
| LYVE1 | 0.164071 | 0.036365 | 2.043489 | 2.924035 |
| NLGN2 | 0.164071 | 0.036378 | 1.038601 | 4.939505 |
| PDZD7 | 0.164071 | 0.036384 | 3.050698 | 0.960546 |
| MNX1 | 0.164071 | 0.036385 | 2.960988 | 0.895434 |
| DZANK1 | 0.164234 | 0.036444 | 1.385103 | 2.763811 |
| CHST2 | 0.164234 | 0.036446 | 1.809207 | 3.400106 |
| LINC01001 | 0.164349 | 0.036484 | -2.14604 | 1.795507 |
| MESTIT1 | 0.164463 | 0.036522 | 2.197828 | 1.832912 |
| LOC654841 | 0.164772 | 0.036603 | 2.627875 | 0.794213 |
| IL13RA1 | 0.164779 | 0.036624 | -0.83948 | 7.605326 |
| OSBPL1A | 0.164779 | 0.03663 | 1.256831 | 3.918915 |
| ZFP36 | 0.164913 | 0.036673 | -1.07365 | 9.219873 |
| CLEC7A | 0.165268 | 0.036764 | 2.377142 | 1.853208 |
| RRN3P1 | 0.165419 | 0.036817 | 1.365743 | 3.8331 |
| MST1L | 0.165419 | 0.03683 | 2.367325 | 2.930705 |
| HCFC2 | 0.165419 | 0.036835 | -0.85236 | 5.788521 |
| SAMD10 | 0.165459 | 0.036857 | -1.40173 | 3.922538 |
| C11orf95 | 0.165584 | 0.0369 | 1.070357 | 4.740732 |

| | | | | |
|---|---|---|---|---|
| MIOX | 0.165584 | 0.03691 | 4.021286 | 0.983003 |
| CYP2U1 | 0.165709 | 0.036954 | 1.741128 | 3.766533 |
| SUCLG2-AS | 0.165709 | 0.036963 | 2.606912 | 0.871295 |
| ARHGAP18 | 0.16573 | 0.03698 | 1.622244 | 6.053756 |
| EPHB6 | 0.16605 | 0.037064 | -2.74619 | 0.523337 |
| IER5L | 0.166154 | 0.0371 | 0.999869 | 4.472228 |
| NRG1 | 0.166533 | 0.037198 | -2.03267 | 2.229988 |
| FRG1HP | 0.166657 | 0.037248 | 1.078911 | 4.924242 |
| PPP1R16B | 0.166657 | 0.037251 | 3.725649 | 0.366046 |
| DZIP1L | 0.166695 | 0.037272 | 1.427468 | 4.041019 |
| CCAT1 | 0.166946 | 0.037341 | -1.34177 | 5.113596 |
| EFCC1 | 0.166959 | 0.037356 | 2.072398 | 2.263327 |
| S100A16 | 0.16698 | 0.037374 | -2.02874 | 1.602203 |
| AP5Z1 | 0.16715 | 0.037438 | 0.899483 | 5.550488 |
| CASC19 | 0.16715 | 0.037442 | -2.06337 | 2.422946 |
| ARL1 | 0.16715 | 0.03745 | 1.927461 | 2.171043 |
| SWI5 | 0.167346 | 0.037507 | -1.23108 | 3.446598 |
| CLDND1 | 0.167508 | 0.037556 | -2.23849 | 1.468273 |
| CD180 | 0.167508 | 0.037569 | 3.034942 | 1.476177 |
| MORN3 | 0.16778 | 0.037658 | 2.524586 | 1.289455 |
| ARHGAP45 | 0.16778 | 0.037666 | 1.698762 | 2.812949 |
| PHLPP2 | 0.16778 | 0.037668 | 2.430107 | 0.70474 |
| NR4A1 | 0.167965 | 0.037722 | 1.793456 | 3.281448 |
| SNORD32A | 0.168054 | 0.037755 | 1.301635 | 4.333617 |
| PSORS1C1 | 0.168054 | 0.037768 | 2.822921 | 0.789398 |
| RORA | 0.168537 | 0.037889 | -1.76997 | 3.009933 |
| VTRNA1-2 | 0.168748 | 0.03795 | 1.343794 | 7.296089 |
| TSFM | 0.168853 | 0.038006 | 2.189462 | 1.254065 |
| MID1IP1 | 0.168853 | 0.038009 | 1.306645 | 3.58739 |
| SSH3 | 0.168853 | 0.038014 | -0.90144 | 6.407793 |
| HPDL | 0.168853 | 0.038025 | 1.793803 | 1.668795 |
| LY75 | 0.168964 | 0.038063 | 1.623645 | 3.181522 |
| FAM83F | 0.16916 | 0.03812 | -1.0977 | 5.26594 |
| NIPSNAP3A | 0.169169 | 0.038144 | -0.98018 | 4.621525 |
| APOBEC3F | 0.169169 | 0.038148 | 1.234545 | 2.69732 |
| CHST6 | 0.16956 | 0.038249 | -1.30037 | 3.795675 |
| MIR4668 | 0.169576 | 0.038265 | -2.69985 | 1.654289 |
| LINC00323 | 0.169576 | 0.038278 | 2.601875 | 0.625997 |
| ADAM19 | 0.169963 | 0.038378 | 0.72807 | 5.933661 |
| UBE2V2 | 0.170255 | 0.038457 | -0.88113 | 5.908854 |
| DANT2 | 0.170525 | 0.038533 | 1.513075 | 4.396131 |
| GATA6 | 0.170525 | 0.038544 | 2.446563 | 5.560242 |
| DNTTIP1 | 0.170575 | 0.038569 | 0.970872 | 4.87146 |
| TMEM63B | 0.170736 | 0.038619 | -0.74004 | 6.304835 |
| CTD-2270F | 0.170736 | 0.038631 | 3.39887 | 0.961031 |

| | | | | |
|---|---|---|---|---|
| TOX | 0.170827 | 0.038665 | 2.390307 | 4.213407 |
| RAB9B | 0.170858 | 0.038685 | 2.046917 | 2.403457 |
| DBR1 | 0.17086 | 0.038698 | 1.039128 | 3.980077 |
| MUC19 | 0.170871 | 0.038717 | 2.342848 | 2.493012 |
| TCF7L2 | 0.170871 | 0.03873 | 1.581111 | 2.035909 |
| LOC643201 | 0.170871 | 0.03874 | 3.128282 | 1.958607 |
| ATG16L2 | 0.170871 | 0.038753 | -0.90764 | 5.485343 |
| ROMO1 | 0.171072 | 0.038811 | -0.99188 | 4.522624 |
| ZNF629 | 0.171316 | 0.03888 | 0.827523 | 5.696927 |
| PACS2 | 0.171447 | 0.038926 | -2.92257 | 1.414328 |
| BORCS5 | 0.171447 | 0.038935 | -2.27342 | 0.951836 |
| RNF167 | 0.171583 | 0.038988 | -0.7487 | 5.954311 |
| AOX1 | 0.171583 | 0.038992 | 1.824515 | 3.871079 |
| COL6A2 | 0.171847 | 0.039074 | 1.366939 | 4.617288 |
| EIF5A | 0.171847 | 0.039079 | -1.31745 | 3.639339 |
| NR2E3 | 0.17198 | 0.039122 | -1.80279 | 2.825191 |
| ERCC6 | 0.17215 | 0.039174 | -0.82976 | 5.625018 |
| NAALADL2 | 0.172189 | 0.039196 | 1.357965 | 4.271261 |
| PIP4K2B | 0.172391 | 0.039255 | 1.32077 | 4.81446 |
| SOWAHB | 0.172498 | 0.039293 | -1.28791 | 3.980894 |
| ARMH1 | 0.172573 | 0.039323 | 2.849718 | 0.836711 |
| LATS2 | 0.172755 | 0.039377 | 0.795757 | 6.024132 |
| SPAG5 | 0.172808 | 0.039403 | 0.954806 | 5.001708 |
| EFNA3 | 0.173082 | 0.039478 | -1.51861 | 5.143595 |
| MANSC1 | 0.173515 | 0.03959 | -0.85238 | 5.953059 |
| BCL10 | 0.174024 | 0.03972 | -0.94827 | 5.599257 |
| PCBP1 | 0.174062 | 0.039742 | -0.79569 | 7.729698 |
| NUDT19 | 0.174445 | 0.039842 | -1.03533 | 4.611879 |
| ANKRD18B | 0.174574 | 0.039885 | -2.44379 | 4.70226 |
| ZNF514 | 0.174578 | 0.039912 | 1.788223 | 2.39146 |
| LXN | 0.174578 | 0.039921 | 1.382564 | 3.238613 |
| CROCCP2 | 0.174578 | 0.039926 | 0.96648 | 4.950941 |
| CDC20 | 0.174615 | 0.039948 | 1.126756 | 4.177338 |
| CBX2 | 0.17473 | 0.040003 | -1.15895 | 3.354286 |
| UTY | 0.17473 | 0.04001 | -2.41927 | 2.662881 |
| LOC65316( | 0.17473 | 0.040014 | 1.411507 | 2.805399 |
| SYNE3 | 0.174853 | 0.040057 | 1.476697 | 3.915727 |
| ACTL10 | 0.174853 | 0.040082 | 1.881751 | 2.764413 |
| PNMA8B | 0.174853 | 0.040082 | 2.533782 | 1.103348 |
| LOC25739( | 0.174909 | 0.040119 | 3.312671 | 0.338562 |
| KHDC4 | 0.174909 | 0.040123 | 0.784684 | 6.719949 |
| GOLGA2P7 | 0.174909 | 0.040135 | -1.23721 | 3.894098 |
| TTC39A | 0.175035 | 0.040184 | -2.90976 | 1.543137 |
| TGM4 | 0.175035 | 0.04019 | 2.965371 | 1.121934 |
| GCNT1 | 0.175438 | 0.040296 | 2.330671 | 1.727553 |

| | | | | |
|---|---|---|---|---|
| IL1B | 0.175545 | 0.040334 | 1.513258 | 3.424547 |
| RPL13P5 | 0.175598 | 0.040375 | 2.053404 | 1.179237 |
| ZNF483 | 0.175598 | 0.040379 | 1.43295 | 4.049376 |
| MVB12B | 0.175598 | 0.040387 | 1.315767 | 3.705195 |
| TRIB2 | 0.175822 | 0.040452 | 1.153227 | 4.25521 |
| SELENOI | 0.175889 | 0.040484 | -0.83968 | 7.186274 |
| KCNK15 | 0.175889 | 0.040494 | 2.465773 | 1.263786 |
| GADD45G | 0.176188 | 0.040584 | 1.896248 | 2.748955 |
| ZNF713 | 0.176188 | 0.040603 | 1.133891 | 3.80269 |
| CHMP4A | 0.176188 | 0.040616 | -0.75646 | 5.927928 |
| HRC | 0.176188 | 0.040617 | 2.372066 | 1.335834 |
| KBTBD8 | 0.176272 | 0.040649 | 1.622563 | 2.782832 |
| S1PR2 | 0.176381 | 0.040688 | 1.477122 | 3.347225 |
| POMGNT2 | 0.176952 | 0.040833 | 1.455943 | 3.236214 |
| SNORD135 | 0.177049 | 0.040869 | 1.540777 | 3.685309 |
| ANKRD36C | 0.177067 | 0.040887 | 0.924304 | 7.633104 |
| IQSEC3 | 0.177137 | 0.040916 | 2.194758 | 2.228676 |
| ZNF350-AS | 0.177142 | 0.040931 | 3.418976 | 0.296192 |
| PPP1R14A | 0.177221 | 0.040963 | 3.898975 | 0.724523 |
| SLC44A2 | 0.177244 | 0.040981 | -1.41816 | 2.567169 |
| APOBEC3D | 0.177459 | 0.041045 | 1.597744 | 2.265279 |
| CKAP4 | 0.177523 | 0.041073 | -0.78388 | 7.32718 |
| ANKRD22 | 0.177735 | 0.041136 | -1.04597 | 6.17748 |
| CCDC73 | 0.177767 | 0.041157 | -2.12211 | 1.867145 |
| CBX7 | 0.17793 | 0.041208 | 1.087944 | 5.668208 |
| AMIGO2 | 0.177945 | 0.041225 | 3.87398 | 0.395899 |
| MINDY1 | 0.177971 | 0.041255 | -1.21361 | 3.421541 |
| HIBADH | 0.177971 | 0.041258 | 0.935337 | 5.131085 |
| KIF4A | 0.17804 | 0.041288 | 1.494838 | 4.539428 |
| CHERP | 0.178232 | 0.04136 | 0.846944 | 5.901328 |
| SRD5A3 | 0.178232 | 0.041391 | -0.977 | 4.802525 |
| ARHGAP27 | 0.178232 | 0.041393 | -0.85227 | 7.149279 |
| GOLGB1 | 0.178232 | 0.041394 | 2.634062 | 0.537099 |
| SP4 | 0.178232 | 0.0414 | 1.416503 | 4.336504 |
| FZD5 | 0.178421 | 0.041457 | 0.920548 | 6.129157 |
| AGO1 | 0.179278 | 0.041674 | 3.436094 | 1.016627 |
| COL19A1 | 0.179278 | 0.041684 | 3.369707 | 2.227932 |
| RBM20 | 0.179317 | 0.041711 | -2.02554 | 5.01298 |
| PGM3 | 0.179317 | 0.04172 | 2.62267 | 1.526093 |
| L3MBTL4 | 0.179372 | 0.041747 | -1.37209 | 3.739216 |
| ZNF235 | 0.179372 | 0.041761 | 1.081722 | 4.36461 |
| RHOQ | 0.179553 | 0.041823 | 0.848728 | 6.038223 |
| RCAN1 | 0.179553 | 0.04183 | 1.310561 | 3.243647 |
| ZNF345 | 0.179623 | 0.04186 | -1.66881 | 2.227099 |
| CAMK1 | 0.179924 | 0.041944 | 1.230244 | 3.479166 |

| | | | | |
|---|---|---|---|---|
| WFDC5 | 0.179985 | 0.041972 | -2.81161 | 1.308968 |
| ASTN2-AS1 | 0.180009 | 0.041991 | 2.219158 | 1.618007 |
| ASPN | 0.180011 | 0.042016 | 2.067305 | 1.612299 |
| TRPC6 | 0.180011 | 0.042019 | 1.357186 | 3.852959 |
| IRAK1BP1 | 0.180136 | 0.042062 | 1.261824 | 2.986708 |
| SENP3 | 0.180475 | 0.042155 | -1.49747 | 2.178368 |
| LINC00330 | 0.180725 | 0.042227 | -2.29124 | 1.734809 |
| MEOX2 | 0.180749 | 0.042246 | 2.147803 | 1.909962 |
| OAS3 | 0.180976 | 0.04233 | 0.797735 | 6.855818 |
| RIPOR2 | 0.180976 | 0.042336 | 2.893647 | 1.049629 |
| SLC17A5 | 0.180976 | 0.042347 | 0.955263 | 4.939869 |
| MKRN2OS | 0.180976 | 0.042355 | -1.47003 | 2.709347 |
| METAP1D | 0.181023 | 0.042379 | 1.830903 | 3.10652 |
| INPPL1 | 0.181074 | 0.042405 | 0.799929 | 6.726199 |
| ELK2AP | 0.181432 | 0.042503 | 2.35566 | 4.633973 |
| ZNF165 | 0.181571 | 0.042549 | 1.069002 | 4.259168 |
| BEND7 | 0.18179 | 0.042615 | 1.911013 | 1.638769 |
| ATP5F1B | 0.181926 | 0.04268 | -0.88841 | 8.314922 |
| COL4A2-AS | 0.181926 | 0.042691 | 2.787867 | 0.576915 |
| COL24A1 | 0.181926 | 0.042695 | 2.154486 | 3.152256 |
| GABRE | 0.181926 | 0.042705 | -1.53268 | 6.021543 |
| ZNF302 | 0.181926 | 0.042719 | 2.041142 | 1.665566 |
| ESPN | 0.181926 | 0.042729 | -1.18579 | 5.136179 |
| SNORD56 | 0.182081 | 0.04278 | 1.161047 | 5.853237 |
| OTUB2 | 0.182125 | 0.042804 | -3.66883 | 0.544954 |
| ALDH2 | 0.182369 | 0.042875 | 2.392811 | 0.622752 |
| LINC01310 | 0.182408 | 0.042904 | -4.1972 | 0.859592 |
| CIR1 | 0.182408 | 0.042912 | 0.739029 | 6.063883 |
| NLGN1-AS1 | 0.1826 | 0.042971 | 3.073432 | 1.531748 |
| HLA-DOA | 0.182634 | 0.042993 | 3.430614 | 1.220199 |
| RNA5-8SN5 | 0.182881 | 0.043065 | -1.01958 | 12.55551 |
| LTBR | 0.183044 | 0.043118 | -1.14398 | 4.25146 |
| ZNF768 | 0.183181 | 0.043172 | 0.892788 | 5.329489 |
| GOT1 | 0.183181 | 0.043178 | -0.8072 | 5.283462 |
| DPF2 | 0.183297 | 0.043219 | 0.863124 | 5.289677 |
| SPAG16 | 0.18353 | 0.043288 | -1.60873 | 3.437954 |
| GAS5-AS1 | 0.183548 | 0.043306 | 2.389397 | 1.425725 |
| CREBRF | 0.183712 | 0.043364 | 1.018399 | 5.650648 |
| LMCD1 | 0.183712 | 0.043374 | 1.106369 | 3.703647 |
| PAH | 0.183712 | 0.043387 | 3.171769 | 1.355997 |
| MUC2 | 0.183734 | 0.043406 | 3.444002 | 5.617979 |
| AGBL2 | 0.184097 | 0.043506 | 2.035806 | 2.26728 |
| ZSCAN31 | 0.184623 | 0.043644 | -2.66541 | 1.325349 |
| MTAP | 0.184664 | 0.043685 | -0.98032 | 5.963064 |
| PFKFB3 | 0.184664 | 0.043685 | 1.863894 | 2.102962 |

| | | | | |
|---|---|---|---|---|
| RPS23 | 0.184664 | 0.043696 | -0.81343 | 8.417209 |
| NBPF25P | 0.18555 | 0.04392 | -0.82485 | 5.611177 |
| UFM1 | 0.185758 | 0.043983 | 1.480087 | 2.910945 |
| SERPINE2 | 0.18577 | 0.044 | -1.97708 | 2.245335 |
| LINC01144 | 0.186215 | 0.04412 | 2.256357 | 0.788193 |
| ADGRA3 | 0.187178 | 0.044365 | 0.992602 | 5.517179 |
| ZNF441 | 0.187178 | 0.044377 | 1.277045 | 4.209727 |
| PDZD11 | 0.187578 | 0.044492 | -0.97916 | 4.056473 |
| BCL6B | 0.187578 | 0.0445 | 1.346049 | 2.971169 |
| CD3E | 0.187595 | 0.044518 | 1.688193 | 3.550738 |
| ORMDL2 | 0.187666 | 0.044558 | -1.01678 | 4.191462 |
| VPS13D | 0.187666 | 0.044588 | -2.05658 | 0.845907 |
| ROBO3 | 0.187666 | 0.044592 | 1.353391 | 3.598287 |
| NUDCD2 | 0.187666 | 0.044592 | -1.41931 | 3.643594 |
| KRTAP10-8 | 0.187897 | 0.044662 | 5.016143 | 0.992991 |
| ASB5 | 0.188146 | 0.044738 | 3.512475 | 1.567851 |
| LAMB4 | 0.188146 | 0.04475 | -2.75576 | 2.154538 |
| RPL12 | 0.188181 | 0.044781 | -0.7858 | 8.617049 |
| MAOB | 0.188181 | 0.044819 | 1.522325 | 4.80083 |
| GLUD1 | 0.188181 | 0.044822 | -0.77523 | 7.59286 |
| HIST1H3E | 0.188181 | 0.044827 | -1.49043 | 2.400801 |
| NAT14 | 0.188181 | 0.04483 | 1.948406 | 1.619786 |
| RPL10A | 0.188316 | 0.044876 | -0.81363 | 8.124088 |
| LOC101241 | 0.188522 | 0.04494 | 1.798631 | 1.955522 |
| ZNF594 | 0.188835 | 0.045038 | 1.075781 | 4.621452 |
| TCEAL7 | 0.188835 | 0.045048 | 2.043026 | 1.033912 |
| SSNA1 | 0.188835 | 0.045057 | -0.832 | 4.665149 |
| MIR3681H | 0.188993 | 0.045118 | 3.863664 | 0.689013 |
| VEZF1 | 0.188993 | 0.045124 | 0.764149 | 6.490164 |
| CGAS | 0.189084 | 0.04516 | 1.28855 | 3.187813 |
| SPATA13 | 0.189155 | 0.045191 | 3.398215 | 1.238896 |
| CLDN34 | 0.189407 | 0.045272 | -3.74437 | 0.699849 |
| TMEM41B | 0.189407 | 0.045286 | -0.8105 | 5.645028 |
| PIGK | 0.189407 | 0.045295 | 1.12145 | 4.917392 |
| KDM7A | 0.189593 | 0.045354 | -0.85227 | 7.953198 |
| SNORD9 | 0.189678 | 0.045389 | 1.396824 | 5.704416 |
| CITED2 | 0.189703 | 0.045409 | -1.33381 | 3.248602 |
| ASPHD1 | 0.189754 | 0.045436 | 2.223186 | 4.045769 |
| MBP | 0.189772 | 0.045455 | -0.7608 | 5.784754 |
| TMEM87B | 0.190245 | 0.045588 | 0.757656 | 7.075531 |
| TMBIM4 | 0.190245 | 0.045597 | 2.112496 | 1.998985 |
| MYL6 | 0.190428 | 0.045672 | 1.349434 | 3.225946 |
| PRICKLE2-A | 0.190428 | 0.045683 | 1.580892 | 3.055756 |
| AKAP6 | 0.190428 | 0.045684 | 1.278632 | 4.763257 |
| EMC7 | 0.190563 | 0.045766 | -0.74937 | 5.706121 |

| | | | | |
|---|---|---|---|---|
| EGFR-AS1 | 0.190563 | 0.045768 | -1.36037 | 3.844728 |
| TIGD1 | 0.190563 | 0.045771 | 1.161906 | 3.907035 |
| NDUFB1 | 0.190563 | 0.045775 | -1.3137 | 5.053318 |
| DNAH1 | 0.190592 | 0.045806 | 0.953627 | 6.28051 |
| CA3-AS1 | 0.190592 | 0.045811 | -2.38042 | 2.492402 |
| PTGER2 | 0.190672 | 0.045854 | 1.991471 | 2.652034 |
| ADGRD1 | 0.190672 | 0.045866 | 1.845914 | 4.68978 |
| SNORD84 | 0.190672 | 0.045873 | 1.283203 | 4.172297 |
| FIRRE | 0.190673 | 0.045888 | 2.443185 | 2.449381 |
| TPST1 | 0.191262 | 0.046045 | -0.8797 | 4.880423 |
| MFSD13A | 0.191434 | 0.046103 | 1.396996 | 2.65091 |
| LOC72861З | 0.191434 | 0.046115 | 1.555516 | 3.607972 |
| MGLL | 0.191614 | 0.046173 | -1.99229 | 0.906966 |
| ARMCX4 | 0.191655 | 0.046198 | 1.049835 | 4.33297 |
| MERTK | 0.191797 | 0.046246 | 0.960632 | 5.51198 |
| SYTL4 | 0.191891 | 0.046284 | -2.3435 | 1.426558 |
| TPRG1-AS2 | 0.192217 | 0.046377 | -2.61785 | 1.669334 |
| TRMT11 | 0.192222 | 0.046394 | 0.801703 | 5.041259 |
| PGLS | 0.192222 | 0.046408 | -0.85031 | 4.600062 |
| SNORA20 | 0.192495 | 0.046488 | 1.043659 | 8.254074 |
| WNT5B | 0.192543 | 0.046514 | -2.94784 | 0.92218 |
| PLEKHA2 | 0.192611 | 0.046545 | -0.81666 | 7.090214 |
| C11orf96 | 0.192668 | 0.046587 | 1.828449 | 4.907249 |
| SLC41A2 | 0.192668 | 0.046589 | 1.624718 | 5.294823 |
| SALRNA2 | 0.192746 | 0.046622 | 2.496052 | 0.639591 |
| AKR1C3 | 0.192872 | 0.046671 | 2.776282 | 0.363722 |
| SNORD116 | 0.192872 | 0.0467 | 1.843785 | 5.412446 |
| PLA2G12A | 0.192872 | 0.04671 | -0.79276 | 6.037893 |
| PPIC | 0.192872 | 0.046712 | -0.82222 | 5.67235 |
| AQP9 | 0.192882 | 0.04673 | 2.809874 | 3.121265 |
| CCNI | 0.192882 | 0.046743 | -0.75841 | 8.137876 |
| PRR15L | 0.192976 | 0.046781 | 1.393765 | 5.523537 |
| ADAMTS9 | 0.193045 | 0.046812 | 1.080129 | 6.272354 |
| TMEM265 | 0.193186 | 0.046861 | -0.99277 | 4.571968 |
| SNORD27 | 0.193322 | 0.046935 | 1.158498 | 8.064376 |
| ADD3 | 0.193322 | 0.046943 | 2.483901 | 1.293234 |
| ANKAR | 0.193322 | 0.046944 | 1.059072 | 4.130894 |
| PLCE1 | 0.193322 | 0.046965 | 2.330555 | 0.759254 |
| DCAF12 | 0.193322 | 0.046979 | -0.79308 | 6.680756 |
| SST | 0.193322 | 0.046982 | -3.46589 | 2.262791 |
| MYO5B | 0.193455 | 0.04704 | -0.94865 | 8.339672 |
| GOLGA2 | 0.193455 | 0.047048 | -0.75984 | 8.404773 |
| PTGDS | 0.193455 | 0.047059 | 1.255468 | 4.813794 |
| KCNJ15 | 0.193697 | 0.047138 | -2.36567 | 1.241694 |
| TBX18 | 0.193697 | 0.047147 | 2.42395 | 2.813631 |

| | | | | |
|---|---|---|---|---|
| CLSTN3 | 0.193949 | 0.047223 | 0.797356 | 5.254589 |
| SNORA80B | 0.194347 | 0.047335 | 1.546687 | 2.855593 |
| MIR6069 | 0.194476 | 0.047381 | -2.75663 | 0.871366 |
| HMGXB3 | 0.194532 | 0.04741 | 0.702939 | 6.060751 |
| MIR4480 | 0.194534 | 0.04743 | -2.55994 | 1.035649 |
| CDK7 | 0.194534 | 0.047452 | -1.90928 | 2.689336 |
| RPL36AL | 0.194534 | 0.047455 | -0.73667 | 7.510566 |
| TPI1 | 0.195226 | 0.047639 | -1.52236 | 3.140808 |
| RPS26P11 | 0.195231 | 0.047658 | -1.27485 | 3.96956 |
| MYLIP | 0.195231 | 0.04767 | 0.910462 | 4.919553 |
| MYCT1 | 0.195286 | 0.047698 | 1.489452 | 2.331139 |
| LINC01606 | 0.195457 | 0.047755 | 3.877911 | 1.171645 |
| FAIM2 | 0.195572 | 0.047797 | 2.220281 | 1.682449 |
| ABCC1 | 0.195771 | 0.047861 | -0.87152 | 5.949324 |
| CCP110 | 0.19584 | 0.047893 | 1.799442 | 2.034929 |
| KDELC1 | 0.195928 | 0.047929 | 1.954936 | 1.897471 |
| TYMS | 0.19632 | 0.04804 | 1.321998 | 3.831373 |
| DACT3-AS1 | 0.19643 | 0.048083 | 2.705624 | 0.439467 |
| CYP2E1 | 0.19643 | 0.048097 | -1.54938 | 4.17799 |
| HRAT92 | 0.196439 | 0.048114 | 1.368162 | 2.590929 |
| COMMD1 | 0.196467 | 0.048136 | -0.98999 | 4.30344 |
| STK4 | 0.196537 | 0.048206 | 0.713643 | 7.306851 |
| LOC40094( | 0.196537 | 0.048209 | -3.39427 | 1.01321 |
| GLCCI1 | 0.196537 | 0.048227 | 1.012936 | 4.641302 |
| MTLN | 0.196537 | 0.048235 | -1.43457 | 2.767422 |
| ADCY2 | 0.196537 | 0.048254 | 2.535323 | 2.817003 |
| CYSTM1 | 0.196537 | 0.048263 | 1.670608 | 6.20571 |
| ABALON | 0.196537 | 0.048266 | 2.011588 | 2.239759 |
| KLHL15 | 0.196537 | 0.048273 | 0.917646 | 4.644393 |
| COQ9 | 0.196685 | 0.048324 | -0.74819 | 5.338648 |
| LINC00476 | 0.196709 | 0.048367 | 1.162728 | 3.649671 |
| SEC23A | 0.196709 | 0.04837 | 0.839984 | 6.575588 |
| RCAN2 | 0.196709 | 0.048376 | 2.888092 | 0.443886 |
| GFER | 0.196709 | 0.048402 | 1.065148 | 3.715945 |
| OVCH2 | 0.196709 | 0.048411 | 2.90709 | 1.622335 |
| USP12 | 0.196709 | 0.04842 | 0.747681 | 6.434255 |
| GBP1P1 | 0.197095 | 0.04853 | -1.25024 | 3.534464 |
| AMFR | 0.197127 | 0.048553 | -0.815 | 6.784752 |
| RPS2P32 | 0.197216 | 0.04859 | 2.739019 | 1.35959 |
| MITF | 0.19801 | 0.048801 | 2.372341 | 2.431798 |
| KIFC2 | 0.198216 | 0.048878 | -0.88391 | 6.033516 |
| PSORS1C2 | 0.198216 | 0.048882 | 2.749503 | 1.305302 |
| PRAG1 | 0.198506 | 0.048983 | 0.863495 | 5.328712 |
| RBM3 | 0.198506 | 0.048984 | -0.73485 | 7.730397 |
| TMEM41A | 0.198783 | 0.049067 | -0.78983 | 5.188367 |

| | | | | |
|---|---|---|---|---|
| SLC16A7 | 0.198984 | 0.049147 | -2.25822 | 1.562425 |
| ZDHHC2 | 0.198984 | 0.049153 | 1.33784 | 4.567741 |
| RNASE6 | 0.198984 | 0.049162 | 2.140606 | 2.307376 |
| DUSP4 | 0.199087 | 0.049203 | 1.670615 | 4.855775 |
| DCBLD2 | 0.199087 | 0.049218 | 0.778385 | 5.864367 |
| KHDRBS1 | 0.199115 | 0.04924 | 1.23307 | 2.725856 |
| ACTA2 | 0.199288 | 0.049298 | 1.848591 | 1.65467 |
| NOS1AP | 0.199431 | 0.049359 | 1.678513 | 2.319383 |
| SULT1C4 | 0.199431 | 0.049366 | 1.950373 | 2.449201 |
| SNORD35B | 0.199431 | 0.049379 | 1.177372 | 4.493109 |
| EHBP1L1 | 0.199633 | 0.049447 | -0.76078 | 9.072565 |
| CXCL13 | 0.199633 | 0.049459 | 3.41847 | 1.271857 |
| FAAH | 0.199934 | 0.049551 | 0.926135 | 4.417699 |
| FGD1 | 0.199934 | 0.049565 | -0.84844 | 4.314418 |
| HSD17B3 | 0.200045 | 0.049607 | 2.603106 | 1.06853 |
| PRDM11 | 0.200136 | 0.049645 | 1.110339 | 4.050547 |
| SLC6A15 | 0.200219 | 0.049681 | -2.47806 | 1.063693 |
| ATP2A2 | 0.200251 | 0.049704 | -0.89649 | 7.95935 |
| PPM1F | 0.200393 | 0.049755 | -0.69133 | 5.938755 |
| CEP72 | 0.200418 | 0.049776 | 1.266316 | 4.96436 |
| RNF217 | 0.200466 | 0.049803 | -1.29626 | 3.017961 |
| AIDA | 0.2005 | 0.049832 | 0.892869 | 4.818119 |
| NBAT1 | 0.2005 | 0.049842 | 2.224163 | 2.555604 |
| STN1 | 0.200848 | 0.049961 | -0.85855 | 6.139148 |
| RUNX1 | 0.200848 | 0.049966 | 1.42755 | 3.663484 |
| SPOPL | 0.200848 | 0.049982 | 0.703821 | 5.768152 |
| SMYD2 | 0.200848 | 0.04999 | -0.85415 | 5.074533 |
| C15orf41 | 0.201308 | 0.05012 | -2.23109 | 1.187797 |
| CAVIN1 | 0.201395 | 0.050157 | 0.792173 | 7.949234 |
| TCHH | 0.20183 | 0.050281 | -1.64697 | 3.166338 |
| CD70 | 0.201888 | 0.050325 | 2.687599 | 1.802934 |
| PAXIP1-AS: | 0.201888 | 0.050326 | 2.229213 | 2.51079 |
| APOBR | 0.202002 | 0.05037 | 1.211259 | 4.523845 |
| BMT2 | 0.20241 | 0.050487 | -1.00688 | 5.101232 |
| PGM5 | 0.202411 | 0.050507 | 1.880975 | 4.933186 |
| SARS | 0.202411 | 0.050527 | -0.71065 | 7.054196 |
| SERPINB8 | 0.202411 | 0.050533 | -1.93105 | 1.826942 |
| SLC6A16 | 0.202716 | 0.050625 | 1.652608 | 2.712886 |
| SNORA5C | 0.202866 | 0.050678 | 1.301586 | 3.097422 |
| ERICH2 | 0.202887 | 0.050699 | 3.056861 | 0.363566 |
| MIR4802 | 0.202887 | 0.050729 | -2.13919 | 1.66076 |
| VRTN | 0.202887 | 0.05073 | -4.23044 | 0.791692 |
| TARSL2 | 0.202887 | 0.050745 | 0.878694 | 5.130845 |
| CNTRL | 0.202936 | 0.050773 | 0.863017 | 6.610573 |
| MMP27 | 0.20303 | 0.050812 | -3.76389 | 1.371324 |

| | | | | |
|---|---|---|---|---|
| CCDC12 | 0.203208 | 0.050872 | -1.30301 | 2.375442 |
| MRPS36 | 0.203266 | 0.050902 | -1.84043 | 2.533419 |
| ZNF766 | 0.203851 | 0.051064 | 0.872178 | 5.2605 |
| ZNF792 | 0.20414 | 0.051159 | 1.302734 | 3.638422 |
| KDM7A-DT | 0.20414 | 0.051172 | -1.14069 | 4.178281 |
| LNX1-AS1 | 0.20414 | 0.051183 | -2.53374 | 1.49423 |
| IDH3A | 0.204148 | 0.0512 | -0.80198 | 6.274872 |
| CNPY4 | 0.204358 | 0.051278 | 1.29101 | 3.325385 |
| LOC100507 | 0.204358 | 0.051284 | 3.071566 | 0.705991 |
| MATN3 | 0.204577 | 0.051355 | 2.578454 | 1.783457 |
| SMIM15 | 0.204583 | 0.051372 | -0.80537 | 5.793312 |
| GXYLT2 | 0.204806 | 0.051444 | 1.503467 | 2.805496 |
| SNORD63 | 0.204855 | 0.051484 | 1.467427 | 4.677346 |
| ISCA1 | 0.204855 | 0.051487 | 0.890883 | 4.823491 |
| OR7E47P | 0.204913 | 0.051517 | 2.244516 | 1.244572 |
| TOR4A | 0.205194 | 0.051603 | 0.972662 | 5.456501 |
| RGS14 | 0.205238 | 0.05163 | -0.97312 | 4.007886 |
| CCDC9B | 0.205499 | 0.051712 | -1.10495 | 7.421042 |
| SLC16A3 | 0.205645 | 0.051778 | -1.62481 | 3.215598 |
| GABARAPL | 0.205645 | 0.051779 | -0.72171 | 5.631521 |
| AOAH-IT1 | 0.205726 | 0.051816 | 2.131184 | 1.503646 |
| ACER2 | 0.206271 | 0.05197 | 1.464283 | 4.208589 |
| FAM222A | 0.206271 | 0.051986 | -1.40841 | 3.003833 |
| CYP1B1 | 0.206271 | 0.052 | 1.711729 | 4.046267 |
| ZNF564 | 0.206309 | 0.052025 | 0.900633 | 5.007137 |
| DDA1 | 0.206552 | 0.052102 | 0.821214 | 5.18522 |
| TRAF4 | 0.206632 | 0.052138 | 0.919766 | 5.470337 |
| INO80C | 0.206657 | 0.05216 | -1.83968 | 1.542132 |
| DUSP11 | 0.206921 | 0.052243 | -0.8478 | 5.23922 |
| TICAM2 | 0.207425 | 0.052386 | 4.858452 | 0.333002 |
| ZBTB34 | 0.207479 | 0.052423 | 1.029779 | 5.125717 |
| SMCR5 | 0.207479 | 0.052431 | -1.01555 | 3.768793 |
| NREP | 0.207747 | 0.052526 | 2.491463 | 1.19478 |
| PI15 | 0.207747 | 0.052531 | 1.926422 | 5.059172 |
| POTEE | 0.208276 | 0.052683 | 1.552626 | 2.882354 |
| COX5A | 0.208276 | 0.052713 | -0.89559 | 5.335445 |
| ZCCHC3 | 0.208276 | 0.052726 | 0.884216 | 4.659687 |
| DIO2-AS1 | 0.208276 | 0.052763 | -2.46841 | 1.21509 |
| LOC101927 | 0.208276 | 0.052766 | 1.854195 | 1.806009 |
| FAM111A | 0.208276 | 0.052771 | 1.985705 | 2.307478 |
| TRIM4 | 0.208276 | 0.052783 | -2.09849 | 1.64876 |
| STUM | 0.208276 | 0.052791 | 2.334726 | 3.039966 |
| VAMP3 | 0.20835 | 0.052826 | -0.72367 | 6.655381 |
| COX6A1 | 0.208899 | 0.052981 | -0.84876 | 5.711731 |
| SOX21-AS1 | 0.208936 | 0.053006 | -1.75202 | 3.6774 |

| | | | | |
|---|---|---|---|---|
| ZNF135 | 0.208965 | 0.053029 | 2.977511 | 1.113124 |
| CARD17 | 0.209183 | 0.053115 | -2.11173 | 1.879253 |
| TINAGL1 | 0.209183 | 0.053123 | 1.943088 | 1.889432 |
| LOC38983 | 0.209183 | 0.053135 | -1.08433 | 6.310537 |
| SPEF2 | 0.209183 | 0.053149 | 1.994634 | 1.939917 |
| ABHD15 | 0.209455 | 0.053234 | 1.056446 | 3.840405 |
| PABPC1L | 0.209511 | 0.053264 | 0.913474 | 4.698041 |
| SULT4A1 | 0.209545 | 0.053289 | 3.368965 | 0.786922 |
| GUK1 | 0.209649 | 0.053331 | -1.22886 | 3.344003 |
| LINC02411 | 0.209664 | 0.053387 | -4.13988 | 1.421063 |
| LRCH3 | 0.209664 | 0.053392 | 0.699639 | 6.011115 |
| MIR1247 | 0.209664 | 0.053395 | 3.60124 | 0.979362 |
| SLC26A10 | 0.209664 | 0.053399 | 1.798307 | 1.907256 |
| FAM238C | 0.209783 | 0.053456 | -1.98674 | 2.407018 |
| TFCP2L1 | 0.209783 | 0.053461 | -0.95857 | 7.642709 |
| FAM155A-I | 0.209971 | 0.053529 | 1.661575 | 2.460393 |
| SNORD94 | 0.209971 | 0.053566 | 1.105567 | 6.240253 |
| CHMP4C | 0.209971 | 0.053578 | -0.92069 | 5.522992 |
| ABCA10 | 0.209971 | 0.053583 | 1.40603 | 4.942752 |
| ABCA3 | 0.209971 | 0.0536 | 1.452789 | 3.66353 |
| KCNMA1-A | 0.209971 | 0.053605 | 2.251572 | 1.187777 |
| GLUD2 | 0.209971 | 0.053632 | -1.06599 | 3.14818 |
| SLC46A2 | 0.209971 | 0.053637 | -1.72438 | 1.9137 |
| HSPB7 | 0.21039 | 0.053776 | 2.372847 | 4.006792 |
| LOC33987 | 0.21039 | 0.053779 | 1.521271 | 2.864249 |
| GLIPR1 | 0.21039 | 0.053792 | 1.029659 | 4.640725 |
| HLA-B | 0.21041 | 0.053813 | 0.990827 | 8.165578 |
| TARID | 0.210502 | 0.053853 | 2.95315 | 1.801352 |
| PRSS8 | 0.210663 | 0.05391 | -0.86751 | 6.40545 |
| NACC2 | 0.210918 | 0.053991 | 0.843275 | 5.709132 |
| ORM2 | 0.211981 | 0.054279 | 3.151024 | 1.84565 |
| SLCO1B7 | 0.212096 | 0.054325 | 3.806324 | 0.656437 |
| FEZ1 | 0.212319 | 0.054411 | 2.050189 | 2.571964 |
| PIGM | 0.212319 | 0.054415 | 1.105415 | 4.493406 |
| MSANTD2 | 0.21253 | 0.054485 | 0.825208 | 4.254848 |
| ITLN1 | 0.212697 | 0.054544 | 3.088397 | 2.618635 |
| TACC2 | 0.212886 | 0.054609 | -1.26282 | 2.698963 |
| DPY19L2P4 | 0.213036 | 0.054675 | 2.259633 | 1.508878 |
| LOC28392 | 0.213036 | 0.054693 | 0.869741 | 5.383581 |
| YWHAH | 0.213036 | 0.054696 | 0.707021 | 6.815604 |
| HLA-H | 0.213369 | 0.054825 | 1.460027 | 3.474213 |
| DPYD-AS1 | 0.213369 | 0.05483 | -1.95525 | 0.801831 |
| ARAP1 | 0.213369 | 0.05483 | 1.270151 | 2.27575 |
| PHETA2 | 0.213554 | 0.054894 | 1.479102 | 2.455458 |
| SPTSSB | 0.213567 | 0.054915 | -1.24643 | 4.045117 |

| | | | | |
|---|---|---|---|---|
| SH3TC1 | 0.213567 | 0.054948 | -0.77976 | 5.837999 |
| ZNF749 | 0.213567 | 0.054965 | 0.988929 | 4.013403 |
| NPDC1 | 0.213567 | 0.054975 | 1.255429 | 4.962978 |
| MZB1 | 0.213567 | 0.054979 | 2.032784 | 3.148184 |
| KIAA0754 | 0.213758 | 0.055046 | 0.729554 | 7.184952 |
| CNP | 0.213758 | 0.05506 | -0.76593 | 6.241988 |
| APBB1IP | 0.214149 | 0.055177 | 1.307769 | 4.20352 |
| LOC101929 | 0.214194 | 0.055205 | -2.32888 | 2.061037 |
| ICOS | 0.214378 | 0.055269 | 2.357793 | 1.440369 |
| CREBL2 | 0.214617 | 0.055347 | 0.771184 | 5.996894 |
| PCNX2 | 0.214751 | 0.055398 | 0.781637 | 6.066645 |
| ARHGEF19 | 0.214966 | 0.05547 | -1.06177 | 4.126188 |
| UBC | 0.215115 | 0.055525 | -0.76962 | 10.25532 |
| LTC4S | 0.215332 | 0.055597 | 2.013515 | 1.895979 |
| L2HGDH | 0.215387 | 0.055628 | -1.0737 | 4.550103 |
| ST5 | 0.2159 | 0.055777 | -1.46756 | 3.261992 |
| CYB5R2 | 0.216092 | 0.055851 | -2.69919 | 0.762739 |
| GDNF-AS1 | 0.216092 | 0.055874 | 2.914984 | 0.789804 |
| TAF6 | 0.216092 | 0.055876 | 1.907139 | 1.581228 |
| SNORD59A | 0.216103 | 0.055895 | 1.118433 | 6.04052 |
| RFX8 | 0.216556 | 0.056029 | 2.445376 | 1.889563 |
| C3AR1 | 0.216611 | 0.056093 | 2.215377 | 3.016354 |
| NR2C2 | 0.216611 | 0.056097 | -2.78473 | 0.424112 |
| ADAD2 | 0.216611 | 0.0561 | -3.01145 | 0.606846 |
| CCR10 | 0.216611 | 0.056113 | 2.345547 | 0.999842 |
| RTP4 | 0.216611 | 0.056138 | 2.063294 | 1.726264 |
| LOC101929 | 0.216611 | 0.056142 | 2.900514 | 0.964538 |
| LCN2 | 0.216628 | 0.056163 | -1.27895 | 8.082929 |
| TP53I13 | 0.21683 | 0.056232 | 1.041857 | 3.913065 |
| RER1 | 0.217204 | 0.056345 | -0.69671 | 6.68922 |
| ZNF660 | 0.217268 | 0.056378 | 1.850717 | 2.990683 |
| GUCY2C | 0.217275 | 0.056397 | 1.332565 | 4.094239 |
| ELK3 | 0.217587 | 0.056494 | -0.75394 | 6.115702 |
| FGFBP3 | 0.217907 | 0.056618 | 1.28689 | 2.240724 |
| KCND1 | 0.217907 | 0.056653 | 1.319106 | 2.549723 |
| FAM228B | 0.217907 | 0.056654 | 1.825325 | 1.797367 |
| UNC13B | 0.217907 | 0.056661 | -0.74366 | 7.214815 |
| CHMP6 | 0.217907 | 0.056676 | -0.82844 | 4.398278 |
| CYB5RL | 0.217907 | 0.056705 | 1.269643 | 2.92046 |
| LOC646762 | 0.217907 | 0.056762 | 0.903012 | 4.688603 |
| OXCT2 | 0.217907 | 0.056785 | 2.268242 | 0.775345 |
| PPM1H | 0.217907 | 0.056786 | 1.577117 | 4.177341 |
| ASB8 | 0.217907 | 0.056788 | -0.77564 | 4.697497 |
| SLC36A1 | 0.217907 | 0.056794 | 0.912542 | 5.097463 |
| CCR8 | 0.217907 | 0.056803 | 2.700138 | 0.518316 |

| | | | | |
|---|---|---|---|---|
| PPARGC1A | 0.217907 | 0.056811 | 1.43947 | 4.327782 |
| DNER | 0.217907 | 0.056814 | -2.68928 | 2.159767 |
| XYLB | 0.217907 | 0.056839 | 0.912101 | 4.903489 |
| PROSER3 | 0.217907 | 0.056843 | 0.926058 | 4.672355 |
| PLAUR | 0.217979 | 0.05689 | 2.247859 | 1.325719 |
| ZNF304 | 0.217979 | 0.056914 | 1.736698 | 2.178287 |
| PSMC3IP | 0.217979 | 0.056918 | 2.404844 | 0.889522 |
| IFT57 | 0.217979 | 0.056928 | 0.736596 | 5.887979 |
| CA8 | 0.218091 | 0.056974 | 2.148448 | 2.579941 |
| RBPMS | 0.218275 | 0.057039 | 1.185751 | 3.944409 |
| PNP | 0.218359 | 0.057077 | -0.88432 | 6.272863 |
| LINC01342 | 0.218955 | 0.05725 | 2.703357 | 2.558054 |
| SNORA32 | 0.219132 | 0.057313 | 0.896393 | 5.302966 |
| ALDH4A1 | 0.219174 | 0.057353 | -3.12797 | 0.49695 |
| CNTLN | 0.219174 | 0.057357 | 1.250554 | 4.386508 |
| PTK6 | 0.219191 | 0.057394 | -1.87785 | 1.246048 |
| MNAT1 | 0.219191 | 0.057403 | -3.22755 | 1.042768 |
| NHSL2 | 0.219191 | 0.057412 | 1.486631 | 3.511881 |
| LINC01278 | 0.219198 | 0.057431 | 0.814212 | 5.48318 |
| HLA-F-AS1 | 0.219359 | 0.057501 | 1.931782 | 1.382442 |
| RAD51D | 0.219359 | 0.057506 | 2.342 | 1.949163 |
| PTGS1 | 0.219562 | 0.057576 | -2.22722 | 1.343084 |
| UCKL1-AS1 | 0.219691 | 0.057632 | -0.90051 | 4.293684 |
| LOX | 0.219691 | 0.05767 | 1.828261 | 2.259996 |
| TYW1 | 0.219691 | 0.057675 | 0.755853 | 5.799832 |
| ARHGAP27 | 0.219691 | 0.057691 | -1.73408 | 1.975032 |
| SOX9 | 0.219691 | 0.057694 | 0.740208 | 7.214043 |
| DNAJB3 | 0.219721 | 0.057718 | -2.42937 | 1.833196 |
| KY | 0.220098 | 0.057834 | 2.040649 | 0.918751 |
| PDE11A | 0.220187 | 0.057875 | 2.802428 | 1.767737 |
| SNORA14A | 0.220187 | 0.057891 | 1.059883 | 3.448277 |
| FALEC | 0.220274 | 0.057931 | -1.67115 | 2.327884 |
| ACVR2B | 0.220935 | 0.058121 | 0.857175 | 5.191804 |
| NR2F2 | 0.220944 | 0.058141 | 1.45116 | 5.13359 |
| FGF9 | 0.220989 | 0.058171 | 2.859707 | 0.913544 |
| SPINDOC | 0.220989 | 0.058186 | 1.08553 | 3.513937 |
| CCDC170 | 0.220991 | 0.058203 | 1.927708 | 2.540616 |
| MRFAP1L1 | 0.221278 | 0.058312 | -0.73124 | 6.688152 |
| KLHL21 | 0.221278 | 0.058315 | -0.79112 | 6.196208 |
| THSD4-AS2 | 0.221278 | 0.058329 | -1.33712 | 4.367346 |
| ZNF212 | 0.22128 | 0.058347 | -0.81394 | 5.073971 |
| MUC6 | 0.221359 | 0.058393 | 3.027849 | 9.552333 |
| CYR61 | 0.221359 | 0.058402 | 0.961385 | 6.19388 |
| UPK1B | 0.221379 | 0.058424 | -1.66119 | 2.841706 |
| LRRC25 | 0.221726 | 0.058532 | 2.433887 | 1.463366 |

| | | | | |
|---|---|---|---|---|
| PLAC8 | 0.22173 | 0.05855 | 1.953901 | 1.57694 |
| DERA | 0.221964 | 0.058629 | -1.57302 | 2.785782 |
| SENP8 | 0.222082 | 0.058677 | -2.32274 | 0.940152 |
| MKNK1 | 0.222292 | 0.058766 | -1.92661 | 1.368157 |
| RPSAP9 | 0.222292 | 0.058766 | 2.329773 | 1.125166 |
| ZNF10 | 0.222469 | 0.05883 | 1.035596 | 4.216071 |
| ITPKA | 0.222469 | 0.058847 | 2.722412 | 2.343314 |
| MAP2K6 | 0.222635 | 0.058909 | 1.144063 | 3.241771 |
| C2orf70 | 0.222635 | 0.058925 | 2.929214 | 0.31512 |
| LOC101928 | 0.222868 | 0.059005 | -1.58573 | 1.922689 |
| LINC01978 | 0.222868 | 0.059029 | 1.681003 | 1.939021 |
| BIRC2 | 0.222868 | 0.059037 | 1.664441 | 1.721959 |
| SPR | 0.222928 | 0.05907 | 1.137214 | 3.849216 |
| PDZK1IP1 | 0.223066 | 0.059124 | -1.24199 | 5.499024 |
| VPS35 | 0.223527 | 0.059277 | -0.70226 | 7.333814 |
| SUGT1P3 | 0.223527 | 0.05928 | 1.513748 | 2.18516 |
| C2CD3 | 0.223613 | 0.05932 | 1.01812 | 4.006921 |
| MTA2 | 0.22367 | 0.059352 | -0.74105 | 7.446376 |
| KIAA1107 | 0.22368 | 0.059372 | 0.936658 | 3.531692 |
| RIMKLA | 0.223808 | 0.059423 | 1.509176 | 3.095508 |
| POLR3GL | 0.223851 | 0.059451 | 0.940599 | 5.641983 |
| ACE2 | 0.223983 | 0.059504 | -1.63245 | 2.733923 |
| ZNF45 | 0.223983 | 0.05952 | 0.891854 | 4.877186 |
| SNORD14C | 0.224137 | 0.059579 | 1.083024 | 7.859955 |
| AVIL | 0.224664 | 0.059736 | 1.317909 | 3.584361 |
| ZDHHC13 | 0.224999 | 0.059848 | -2.47532 | 0.970979 |
| CRADD | 0.224999 | 0.059865 | -1.39093 | 4.030796 |
| LINC01225 | 0.224999 | 0.059898 | 2.871328 | 1.673315 |
| ATP13A1 | 0.224999 | 0.059901 | 0.734441 | 6.154401 |
| ZBTB39 | 0.224999 | 0.059911 | 0.917383 | 4.633675 |
| ARL3 | 0.225411 | 0.060038 | 0.836351 | 4.580187 |
| LOC148696 | 0.225628 | 0.060112 | 0.787551 | 5.929104 |
| KCNK5 | 0.225691 | 0.060147 | 1.286702 | 4.715311 |
| ANKRD36B | 0.225834 | 0.060202 | -1.01189 | 5.466523 |
| ZKSCAN2 | 0.226012 | 0.060267 | 0.957643 | 4.338454 |
| USP27X | 0.22611 | 0.06031 | 1.282797 | 2.889995 |
| CFAP45 | 0.226259 | 0.060383 | 1.347588 | 2.627065 |
| PLCXD1 | 0.226259 | 0.060384 | -1.25027 | 3.259208 |
| HDAC2-AS2 | 0.226758 | 0.060534 | 1.507423 | 1.753849 |
| RNA18SN5 | 0.226776 | 0.060557 | -0.91273 | 12.07705 |
| SMG6 | 0.226826 | 0.060587 | -0.66421 | 7.114757 |
| MAP1S | 0.226984 | 0.06066 | 2.484902 | 1.239011 |
| MIR155HG | 0.226984 | 0.060664 | 2.160354 | 1.444669 |
| ATP11A | 0.227143 | 0.060724 | 2.689663 | 1.260399 |
| OXCT2P1 | 0.227421 | 0.060815 | 2.047902 | 1.611331 |

| | | | | |
|---|---|---|---|---|
| PPFIBP2 | 0.227502 | 0.060854 | -1.12099 | 4.521305 |
| VWA7 | 0.227563 | 0.060888 | 2.928668 | 1.170201 |
| RNA5S9 | 0.227602 | 0.060916 | -1.35466 | 8.756831 |
| ZNF22 | 0.22798 | 0.061046 | 1.289363 | 3.692248 |
| ZSCAN25 | 0.22798 | 0.061052 | 0.769856 | 5.038941 |
| FAM78A | 0.227991 | 0.061072 | 1.159751 | 3.401614 |
| POLR3D | 0.228109 | 0.061121 | 0.797862 | 5.935937 |
| HEATR6 | 0.228192 | 0.061161 | 0.805562 | 4.473006 |
| 3-Mar | 0.22835 | 0.06122 | 1.605018 | 3.066822 |
| PNPLA2 | 0.228368 | 0.061243 | 0.708833 | 5.948558 |
| TMEM19 | 0.228433 | 0.061278 | -0.77256 | 5.812086 |
| FADS6 | 0.228752 | 0.06138 | -2.61712 | 1.356328 |
| LENG8-AS1 | 0.228831 | 0.061419 | 1.075538 | 4.221967 |
| DNASE2 | 0.228952 | 0.061469 | 0.804763 | 4.67202 |
| HCLS1 | 0.228996 | 0.061498 | 2.168433 | 1.947817 |
| DND1 | 0.229403 | 0.061625 | 1.240179 | 2.766732 |
| SLC1A4 | 0.229805 | 0.061751 | 1.35934 | 2.491155 |
| RPL29 | 0.229944 | 0.061806 | -0.83285 | 7.563644 |
| KCNJ5 | 0.230076 | 0.061864 | 2.030131 | 1.35747 |
| SCG2 | 0.230076 | 0.061885 | 2.661321 | 2.327543 |
| FAM171A2 | 0.230076 | 0.061894 | -1.8701 | 1.270779 |
| BAZ2B | 0.230139 | 0.06194 | 1.130435 | 2.750902 |
| SLC39A8 | 0.230139 | 0.061946 | 2.352974 | 0.787583 |
| CNNM1 | 0.230174 | 0.061973 | 1.548575 | 3.170719 |
| FAM84B | 0.230282 | 0.062046 | -0.76711 | 6.857724 |
| PLCL1 | 0.230282 | 0.062058 | 1.630771 | 3.721995 |
| THUMPD3 | 0.230282 | 0.062066 | 1.722257 | 1.805486 |
| PIEZO2 | 0.230282 | 0.06208 | 0.932835 | 5.065414 |
| ZFP36L2 | 0.230282 | 0.062089 | 0.790321 | 8.563288 |
| HSPA6 | 0.231167 | 0.062346 | 2.032752 | 3.314651 |
| BLMH | 0.231241 | 0.062385 | -0.88385 | 5.297422 |
| MMADHC | 0.231241 | 0.062401 | -0.71667 | 5.943387 |
| PTPN9 | 0.231371 | 0.062453 | 0.685146 | 5.585631 |
| P3H2-AS1 | 0.231651 | 0.062547 | -3.49277 | 0.535222 |
| HBEGF | 0.231889 | 0.062648 | -1.39929 | 6.260786 |
| ZNF385C | 0.231889 | 0.062663 | 1.844863 | 1.686181 |
| APOBEC4 | 0.231889 | 0.062664 | 1.73715 | 1.842582 |
| TMX4 | 0.232175 | 0.062762 | 0.759859 | 6.859257 |
| WRN | 0.232175 | 0.062777 | 0.761338 | 6.550837 |
| LINC01133 | 0.232194 | 0.0628 | 1.063657 | 5.699123 |
| CCDC78 | 0.232195 | 0.062818 | 1.338777 | 2.807763 |
| UTP23 | 0.232479 | 0.062946 | 0.831106 | 4.956433 |
| RFC3 | 0.232479 | 0.062947 | 1.0338 | 3.254299 |
| MIR3174 | 0.232479 | 0.062948 | -2.29419 | 0.442947 |
| QSER1 | 0.232771 | 0.063044 | -0.76307 | 7.638943 |

| | | | | |
|---|---|---|---|---|
| ARHGEF5 | 0.23278 | 0.063065 | -0.81691 | 6.349455 |
| ENPEP | 0.23278 | 0.063085 | 1.535537 | 3.200155 |
| ELOA-AS1 | 0.23278 | 0.0631 | 2.558962 | 1.01055 |
| FAM86DP | 0.232871 | 0.063142 | 1.165601 | 3.753211 |
| HS3ST1 | 0.232988 | 0.063192 | -1.25685 | 4.055089 |
| GFOD1 | 0.233149 | 0.063253 | -1.93175 | 1.874764 |
| NME3 | 0.233368 | 0.063331 | 1.227844 | 3.208565 |
| GALNT14 | 0.233368 | 0.063348 | -2.84527 | 0.656007 |
| BCLAF3 | 0.233375 | 0.063375 | 0.661683 | 5.986909 |
| RALGAPA1 | 0.233375 | 0.063386 | -1.28214 | 2.825525 |
| TTC19 | 0.233444 | 0.063422 | -2.80948 | 0.98916 |
| RPL39 | 0.233471 | 0.063453 | -0.82612 | 6.53653 |
| NPLOC4 | 0.233471 | 0.063465 | -0.65741 | 6.50035 |
| TBCC | 0.234007 | 0.063629 | 0.81656 | 4.526731 |
| ZNF394 | 0.234189 | 0.063705 | 0.878242 | 5.418327 |
| N4BP2L2 | 0.234189 | 0.063714 | 0.661128 | 7.323732 |
| LINC02123 | 0.234336 | 0.063772 | -4.7696 | 1.243222 |
| CD1C | 0.234366 | 0.063798 | 2.32599 | 1.820658 |
| PHF20 | 0.234383 | 0.063823 | 0.693145 | 6.403381 |
| DCLRE1A | 0.234383 | 0.063838 | 2.408531 | 0.835531 |
| LINC00869 | 0.234561 | 0.063916 | 1.38809 | 2.888437 |
| CEP170P1 | 0.234561 | 0.063922 | 1.050121 | 3.870377 |
| NCLN | 0.23469 | 0.063975 | 0.800694 | 6.097304 |
| USP17L2 | 0.234734 | 0.064005 | 2.39886 | 0.903788 |
| UBQLN2 | 0.234736 | 0.064024 | -0.72087 | 5.793088 |
| MIR604 | 0.234933 | 0.064096 | -1.99594 | 1.539457 |
| MIR3909 | 0.235223 | 0.0642 | -2.25081 | 0.886442 |
| COQ8A | 0.235223 | 0.06425 | 1.092685 | 5.780061 |
| FBRS | 0.235223 | 0.064254 | -0.67937 | 7.15248 |
| MMP16 | 0.235223 | 0.064257 | 2.039568 | 2.583584 |
| DNAJA2 | 0.235223 | 0.064264 | -0.66939 | 7.233363 |
| LINC00886 | 0.235338 | 0.064319 | 1.945034 | 3.512878 |
| RAD23B | 0.235338 | 0.064332 | -0.75378 | 4.978563 |
| SV2A | 0.235399 | 0.064366 | 1.774778 | 2.034837 |
| LOC10050[illegible] | 0.235768 | 0.064485 | 1.888884 | 2.637647 |
| SNORD41 | 0.235917 | 0.064544 | 1.088135 | 5.554615 |
| MIR151A | 0.236387 | 0.064695 | 2.501818 | 0.554949 |
| PLIN5 | 0.236387 | 0.064708 | -2.03762 | 3.579099 |
| PLAGL2 | 0.236612 | 0.064788 | -0.69576 | 6.19913 |
| RPS7 | 0.236688 | 0.064827 | -0.76059 | 7.520988 |
| CXADRP2 | 0.236948 | 0.064916 | 2.2181 | 0.601651 |
| DLL4 | 0.23739 | 0.065055 | 1.395634 | 2.778883 |
| SLC25A23 | 0.237653 | 0.065165 | -0.73778 | 7.006651 |
| CLOCK | 0.237653 | 0.065191 | -1.21871 | 3.570855 |
| DMAC2L | 0.237653 | 0.065199 | -2.16725 | 1.458089 |

| | | | | |
|---|---|---|---|---|
| RHOBTB3 | 0.237653 | 0.065224 | 1.138994 | 5.889013 |
| GPATCH2 | 0.237653 | 0.065233 | 0.750659 | 4.834175 |
| UBA6 | 0.237653 | 0.065236 | -0.79841 | 7.343576 |
| MCM6 | 0.237719 | 0.065272 | 0.801302 | 5.752241 |
| ENSA | 0.237927 | 0.065363 | -1.21239 | 2.704833 |
| GOLT1A | 0.237927 | 0.065366 | 2.485183 | 1.474373 |
| FOXD2-AS1 | 0.238126 | 0.065451 | 1.643975 | 1.919292 |
| SNORD3D | 0.238126 | 0.065456 | 1.305648 | 5.911265 |
| FLJ31356 | 0.238128 | 0.06548 | -1.03261 | 3.47071 |
| VN1R1 | 0.238128 | 0.065499 | 1.546006 | 2.587518 |
| LHX4-AS1 | 0.238128 | 0.065517 | 1.229774 | 2.815042 |
| IHH | 0.238128 | 0.06553 | 2.436301 | 2.34143 |
| CBX8 | 0.238223 | 0.065574 | 1.250294 | 3.378075 |
| HEIH | 0.238332 | 0.065622 | -1.09554 | 3.623376 |
| TMEM92-A | 0.238334 | 0.06565 | 1.978667 | 1.630456 |
| DTX3L | 0.238334 | 0.065659 | 0.743885 | 7.203616 |
| HSP90B1 | 0.239078 | 0.065887 | 0.759301 | 9.825701 |
| LNP1 | 0.239078 | 0.0659 | 1.670019 | 2.15909 |
| UBE2W | 0.239708 | 0.066092 | -1.8505 | 2.739155 |
| ZNF697 | 0.239981 | 0.066186 | 1.427826 | 4.44512 |
| BCAP31 | 0.239987 | 0.06623 | -1.55332 | 1.985517 |
| HNRNPA1F | 0.239987 | 0.066235 | -1.57812 | 2.552324 |
| CELA2B | 0.239987 | 0.066242 | -1.85817 | 1.932162 |
| MT1M | 0.24002 | 0.066279 | 2.03889 | 1.926759 |
| LINC01588 | 0.24002 | 0.066302 | -1.40813 | 2.907914 |
| CNTNAP3B | 0.24002 | 0.066306 | 1.252301 | 3.166706 |
| MSX1 | 0.240204 | 0.066375 | 2.853248 | 1.145549 |
| ERN2 | 0.240263 | 0.066424 | 3.059816 | 0.553851 |
| B2M | 0.240263 | 0.066434 | 0.897223 | 6.718468 |
| MSRB3 | 0.240263 | 0.06645 | 2.55959 | 0.505302 |
| UBAP2 | 0.240263 | 0.066465 | -1.06435 | 3.594137 |
| LINC00342 | 0.240484 | 0.066544 | 0.999481 | 6.021231 |
| RGS1 | 0.24082 | 0.066656 | 1.289086 | 6.320131 |
| TMEM181 | 0.240993 | 0.066722 | 0.696735 | 7.024912 |
| SNAP47 | 0.241021 | 0.066765 | 0.924163 | 3.957996 |
| TSPAN7 | 0.241021 | 0.066767 | -1.48054 | 2.647169 |
| ASB1 | 0.241581 | 0.066953 | 0.684266 | 5.534138 |
| GANC | 0.241581 | 0.066959 | -0.81954 | 5.584387 |
| YPEL3 | 0.241729 | 0.067018 | -1.31466 | 3.311462 |
| DEGS1 | 0.241783 | 0.067058 | 0.835065 | 5.007887 |
| SPIN4 | 0.241783 | 0.06707 | -0.96018 | 4.469574 |
| LINC01416 | 0.241969 | 0.06714 | 3.297771 | 0.924513 |
| PDXP | 0.242259 | 0.067239 | 1.456793 | 2.44832 |
| FIS1 | 0.24226 | 0.067257 | -0.74944 | 5.477909 |
| PCDH20 | 0.242287 | 0.067283 | 2.453724 | 2.491096 |

| | | | | |
|---|---|---|---|---|
| BMP4 | 0.242429 | 0.067341 | 2.999934 | 1.169985 |
| LOC10192 | 0.242477 | 0.067374 | -2.07303 | 1.137585 |
| SNORD116 | 0.242477 | 0.067392 | 1.424894 | 4.408968 |
| VTRNA2-1 | 0.242477 | 0.06741 | 1.130595 | 6.681309 |
| PLCD3 | 0.243215 | 0.067634 | 0.8804 | 6.127427 |
| PHF12 | 0.243571 | 0.06777 | 0.721168 | 4.763901 |
| ELFN2 | 0.243571 | 0.06777 | 2.257646 | 1.737268 |
| PARP1 | 0.244017 | 0.067917 | 0.655232 | 7.425528 |
| SNHG17 | 0.244017 | 0.067931 | 2.434546 | 1.595274 |
| FLJ37035 | 0.244062 | 0.067962 | 2.483915 | 0.768414 |
| PROSER2-A | 0.244187 | 0.068016 | -1.3333 | 3.856477 |
| SSU72 | 0.244239 | 0.068049 | -0.66394 | 6.522738 |
| CTSO | 0.244353 | 0.068099 | 1.935707 | 2.16534 |
| LOC72921 | 0.244482 | 0.068154 | -1.01336 | 3.470561 |
| RNF212B | 0.244592 | 0.068203 | -1.92365 | 2.516214 |
| TRMT44 | 0.244984 | 0.068331 | -0.77934 | 5.069729 |
| CACNA1C-I | 0.245591 | 0.068519 | 2.012137 | 1.367305 |
| SNORA5A | 0.245618 | 0.068545 | 0.885493 | 5.179419 |
| SRXN1 | 0.245703 | 0.068588 | -0.79623 | 5.812767 |
| DLX3 | 0.245808 | 0.068636 | -1.97861 | 2.381428 |
| ZNF696 | 0.246057 | 0.06874 | 0.996774 | 3.809811 |
| NDUFA13 | 0.246057 | 0.068743 | -0.64378 | 6.127494 |
| TCTEX1D2 | 0.246639 | 0.068924 | -1.4211 | 1.567871 |
| TMEM126I | 0.246742 | 0.068984 | 2.610694 | 0.571756 |
| CLEC4E | 0.246742 | 0.068991 | 2.467776 | 2.123465 |
| MIR28 | 0.247384 | 0.069189 | 2.894003 | 0.967361 |
| NDNF | 0.247838 | 0.069335 | 1.624711 | 3.999884 |
| PGC | 0.247962 | 0.069406 | -4.63021 | 10.69184 |
| TMEM35B | 0.247962 | 0.069407 | 1.64579 | 2.891509 |
| LPP-AS1 | 0.248122 | 0.069471 | 1.494581 | 2.706047 |
| LOC10192 | 0.248222 | 0.069518 | -3.38474 | 0.274921 |
| GATA2-AS1 | 0.248418 | 0.069592 | 1.887041 | 2.204937 |
| IFNGR1 | 0.248627 | 0.069669 | 0.731251 | 6.887981 |
| PPIH | 0.248916 | 0.069769 | 0.779157 | 4.358128 |
| LMF1 | 0.249029 | 0.06982 | 2.207814 | 2.005204 |
| RWDD2A | 0.249076 | 0.069853 | 1.827849 | 2.218869 |
| NBR2 | 0.249076 | 0.069898 | 0.843001 | 4.299363 |
| NDUFA10 | 0.249076 | 0.069906 | -0.66397 | 7.05721 |
| BCO1 | 0.249076 | 0.069921 | 1.874039 | 2.06773 |
| ABHD12 | 0.249076 | 0.069928 | -0.76344 | 4.625033 |
| ZNF781 | 0.249358 | 0.070026 | 2.246943 | 1.538027 |
| PORCN | 0.249499 | 0.070097 | -3.26675 | 0.604619 |
| EN2 | 0.249499 | 0.070131 | 3.215419 | 2.29518 |
| GUSBP9 | 0.249499 | 0.070138 | 1.816101 | 2.710942 |
| LURAP1L | 0.249499 | 0.070142 | 1.29296 | 3.387476 |

| | | | | |
|---|---|---|---|---|
| PGAM4 | 0.249617 | 0.070205 | -1.49135 | 3.44765 |
| S100A3 | 0.249617 | 0.070213 | -1.51306 | 2.135492 |
| GABARAP | 0.249844 | 0.070298 | -0.66163 | 6.774047 |
| TENT5C | 0.249844 | 0.070343 | 1.362169 | 6.339028 |
| KLHDC1 | 0.249844 | 0.070344 | 1.383416 | 2.923244 |
| CCDC59 | 0.249844 | 0.070354 | 2.055105 | 0.707476 |
| FMN1 | 0.249844 | 0.070372 | 0.855449 | 4.604238 |
| GIMAP1 | 0.250211 | 0.070495 | 2.096565 | 1.770809 |
| CA12 | 0.250893 | 0.070706 | -3.0323 | 0.529015 |
| ZC3H12B | 0.251099 | 0.070783 | 1.35521 | 3.396112 |
| SSR2 | 0.251135 | 0.070812 | 0.67817 | 5.900857 |
| PCDHB5 | 0.251311 | 0.070881 | 2.065814 | 1.582709 |
| CYP27C1 | 0.251376 | 0.070919 | -1.54851 | 3.377326 |
| GAPDH | 0.251524 | 0.07098 | -1.567 | 2.542868 |
| RCC2 | 0.251789 | 0.071074 | -1.79606 | 1.451128 |
| TRIM17 | 0.251838 | 0.071106 | 1.921778 | 2.304292 |
| SF1 | 0.251838 | 0.071126 | 2.032284 | 1.111697 |
| SCARNA9L | 0.252018 | 0.071196 | 1.018169 | 4.210341 |
| TTTY15 | 0.252135 | 0.071265 | -2.00119 | 4.622615 |
| YTHDF1 | 0.252135 | 0.071283 | 0.634654 | 5.958008 |
| LCAL1 | 0.252135 | 0.071287 | 2.457888 | 0.597625 |
| ANK2 | 0.252227 | 0.071335 | 1.377475 | 4.037771 |
| PA2G4 | 0.252227 | 0.071351 | -0.68233 | 7.448142 |
| SLC48A1 | 0.252408 | 0.071421 | -0.74534 | 5.083972 |
| NHLRC1 | 0.252466 | 0.071457 | 2.003773 | 1.33675 |
| CPA2 | 0.252497 | 0.071485 | -4.28718 | 3.912898 |
| TASP1 | 0.252735 | 0.071572 | 0.75178 | 5.083171 |
| CD80 | 0.252821 | 0.071615 | 2.016505 | 1.896538 |
| SMC6 | 0.252946 | 0.07167 | -1.84104 | 0.929263 |
| ADAM11 | 0.253471 | 0.071838 | 1.927113 | 1.697566 |
| ABCC6P1 | 0.253635 | 0.071904 | 2.34308 | 1.562105 |
| KCTD7 | 0.253684 | 0.071941 | 1.341824 | 2.673469 |
| HEPHL1 | 0.253684 | 0.071956 | -1.54543 | 4.941564 |
| MAN2A2 | 0.253714 | 0.072 | 0.796305 | 6.320584 |
| CHST10 | 0.253714 | 0.072004 | 1.609937 | 2.280643 |
| CTSC | 0.253733 | 0.072028 | 0.868421 | 5.37231 |
| IWS1 | 0.253989 | 0.07212 | 0.654725 | 7.62444 |
| NRON | 0.254495 | 0.072283 | 1.890875 | 1.725132 |
| POLR2J3 | 0.254665 | 0.072354 | -1.07009 | 3.241477 |
| ETV6 | 0.254665 | 0.072383 | 0.686512 | 7.00746 |
| GAS1 | 0.254665 | 0.072391 | 1.658186 | 2.96983 |
| SFT2D2 | 0.254665 | 0.072409 | -0.90644 | 5.288996 |
| SDHAP3 | 0.255196 | 0.072591 | 1.121923 | 3.914787 |
| PCDHB16 | 0.255196 | 0.072619 | 1.485577 | 3.387337 |
| PPP1R12B | 0.255196 | 0.072619 | 1.403563 | 3.366765 |

| | | | | |
|---|---|---|---|---|
| LOC10013( | 0.256177 | 0.072948 | 1.842015 | 2.31609 |
| BRK1 | 0.256177 | 0.072951 | -0.69256 | 6.120145 |
| TREML2 | 0.256177 | 0.072958 | 2.098235 | 1.15315 |
| TOE1 | 0.256177 | 0.072976 | 0.942034 | 3.436519 |
| PRDM8 | 0.256298 | 0.07303 | 2.630137 | 0.722154 |
| RNF157 | 0.256529 | 0.073115 | 1.663085 | 3.003085 |
| AK7 | 0.256729 | 0.073192 | -1.17579 | 2.759357 |
| NEXN-AS1 | 0.257079 | 0.073311 | 1.988783 | 1.678155 |
| FZD4 | 0.257343 | 0.073406 | 1.012278 | 4.976117 |
| B3GNT9 | 0.257527 | 0.073478 | 1.144395 | 3.313982 |
| TMEM223 | 0.25773 | 0.073589 | 1.193608 | 2.96518 |
| PZP | 0.25773 | 0.073603 | 1.490677 | 3.160236 |
| DMKN | 0.25773 | 0.073608 | -1.84339 | 2.105736 |
| LIG4 | 0.25773 | 0.073615 | 2.06915 | 0.573315 |
| OSGEPL1 | 0.258195 | 0.073767 | 1.244291 | 3.308739 |
| FAM30A | 0.258385 | 0.073841 | 1.874598 | 3.708924 |
| KRTAP10-1 | 0.25881 | 0.073982 | 4.345916 | 1.303014 |
| GNA14 | 0.258958 | 0.074045 | 1.51016 | 2.544955 |
| SLC16A2 | 0.259191 | 0.074131 | 1.025042 | 3.949407 |
| GAU1 | 0.259443 | 0.074223 | 2.389013 | 1.152762 |
| UBL4B | 0.259483 | 0.074255 | -3.82612 | 0.677119 |
| LRRC4B | 0.259483 | 0.074274 | 2.110518 | 1.758271 |
| MEI1 | 0.259595 | 0.074326 | 1.993167 | 2.413089 |
| PPCS | 0.259804 | 0.074405 | 1.893679 | 1.398149 |
| LARP1 | 0.259822 | 0.07443 | -0.63325 | 8.97235 |
| SNX27 | 0.259879 | 0.074466 | 0.621984 | 6.971331 |
| LRGUK | 0.259987 | 0.074517 | 1.764372 | 2.613731 |
| ODF3B | 0.260041 | 0.074552 | 1.080859 | 4.117089 |
| EMBP1 | 0.26019 | 0.074615 | 1.458294 | 2.827694 |
| PRR12 | 0.260353 | 0.07469 | 0.694567 | 6.673823 |
| SF3A3 | 0.260353 | 0.074701 | 0.647952 | 6.910105 |
| FAM118B | 0.260453 | 0.07475 | 0.827966 | 4.385587 |
| TMEM132I | 0.260484 | 0.074779 | 2.086608 | 1.934537 |
| ADPRHL2 | 0.260604 | 0.074833 | -0.75698 | 4.487124 |
| CNTNAP3 | 0.260639 | 0.074863 | -1.28003 | 3.996947 |
| LINC01117 | 0.260641 | 0.074883 | -2.89615 | 0.636351 |
| RAVER1 | 0.260687 | 0.074916 | 0.776781 | 5.59038 |
| DLGAP3 | 0.26096 | 0.075014 | 2.384605 | 0.626073 |
| DTHD1 | 0.261022 | 0.075054 | 1.962733 | 1.934222 |
| MAZ | 0.261022 | 0.075091 | 2.399125 | 0.288862 |
| ATP6V0A2 | 0.261022 | 0.075092 | 0.725594 | 5.905142 |
| PHAX | 0.261072 | 0.075126 | -0.65787 | 5.660067 |
| PPP1R14B | 0.261685 | 0.075323 | -0.86694 | 5.776752 |
| LINC00431 | 0.261734 | 0.075357 | 2.471757 | 0.379267 |
| SNORD114 | 0.26188 | 0.075419 | -4.35963 | 0.512638 |

| | | | | |
|---|---|---|---|---|
| PRKRA-AS1 | 0.261907 | 0.075458 | 1.363567 | 3.067026 |
| NNT-AS1 | 0.261907 | 0.075466 | 1.257701 | 3.660817 |
| TNFAIP2 | 0.262044 | 0.075526 | -0.80127 | 8.190726 |
| PTCD3 | 0.262238 | 0.075602 | 0.665653 | 6.950627 |
| OLFML1 | 0.26225 | 0.075637 | 1.183539 | 3.535615 |
| SPATA2 | 0.26225 | 0.075645 | 2.224997 | 0.706043 |
| LINC01226 | 0.262266 | 0.07567 | 3.04441 | 2.585557 |
| CAP2 | 0.262443 | 0.075757 | 1.076818 | 4.185494 |
| RANBP10 | 0.262443 | 0.075761 | -0.70836 | 5.848614 |
| APP | 0.262621 | 0.075832 | 2.218942 | 0.892046 |
| LOC392232 | 0.262977 | 0.075955 | -2.31038 | 2.78973 |
| ANKRD13B | 0.263228 | 0.076048 | 0.950064 | 3.474464 |
| PLCL2 | 0.263345 | 0.076101 | 2.568915 | 0.630787 |
| AREG | 0.263438 | 0.076148 | -1.13601 | 5.894317 |
| ZACN | 0.263655 | 0.076231 | -2.19448 | 0.856482 |
| TNFAIP8L2 | 0.263803 | 0.076294 | 1.606153 | 1.726726 |
| FANCE | 0.264385 | 0.076483 | -1.01936 | 3.643602 |
| ABLIM3 | 0.264765 | 0.076618 | -2.23147 | 1.110985 |
| ABHD1 | 0.264765 | 0.076633 | 1.736864 | 2.165856 |
| B4GALNT4 | 0.265375 | 0.07683 | -1.22187 | 4.801913 |
| CFDP1 | 0.26551 | 0.076889 | -0.65607 | 6.907269 |
| SLC16A1 | 0.265575 | 0.076928 | -2.19017 | 0.822184 |
| ZFAND6 | 0.265732 | 0.077009 | 1.720742 | 1.168782 |
| SLC45A4 | 0.265732 | 0.077014 | 0.961701 | 4.07394 |
| OSBPL10 | 0.26635 | 0.077217 | 1.417325 | 2.304874 |
| UNC5CL | 0.26635 | 0.077234 | 1.181966 | 4.655771 |
| KBTBD13 | 0.26644 | 0.07728 | -2.562 | 0.649321 |
| SYPL2 | 0.266684 | 0.077371 | 2.666334 | 1.014197 |
| EPC2 | 0.26695 | 0.077469 | 0.650615 | 6.057246 |
| GCNA | 0.267191 | 0.077559 | 1.071965 | 3.236171 |
| NAA30 | 0.267474 | 0.077679 | -0.70502 | 5.482238 |
| SASH3 | 0.267474 | 0.077682 | 1.087079 | 3.566313 |
| EDEM3 | 0.267487 | 0.077723 | 0.665381 | 7.433542 |
| LOC10666( | 0.267487 | 0.077727 | 1.823496 | 1.586508 |
| SNORD82 | 0.267569 | 0.077771 | 1.171981 | 3.543507 |
| MKI67 | 0.267889 | 0.077884 | 1.282376 | 4.144194 |
| PTBP3 | 0.267998 | 0.077936 | -1.56005 | 1.746189 |
| NAPSA | 0.268689 | 0.078168 | 2.998025 | 0.805374 |
| C12orf54 | 0.268689 | 0.078182 | -2.89893 | 0.703749 |
| RIMS3 | 0.268689 | 0.078199 | 0.968642 | 3.882391 |
| C1RL | 0.268842 | 0.078264 | 1.448397 | 2.787437 |
| GRM3 | 0.268849 | 0.078286 | 2.39714 | 0.671055 |
| ZNF836 | 0.26937 | 0.078458 | 0.933184 | 4.569396 |
| ANKRD9 | 0.269492 | 0.078514 | -0.99304 | 4.280594 |
| PARP9 | 0.269619 | 0.078572 | 0.961694 | 4.64328 |

| | | | | |
|---|---|---|---|---|
| CTRL | 0.269661 | 0.078605 | -1.28966 | 2.23242 |
| NDUFA6-D | 0.269775 | 0.078664 | 1.924415 | 1.691658 |
| MAB21L2 | 0.269775 | 0.078721 | -0.97517 | 3.790207 |
| FUCA1 | 0.269775 | 0.078724 | 0.729698 | 4.928139 |
| TAB2 | 0.269775 | 0.078731 | -1.22554 | 2.420886 |
| CRIPT | 0.269775 | 0.078741 | 0.740759 | 5.448761 |
| BTF3 | 0.270075 | 0.078849 | -1.59615 | 1.774213 |
| SLC6A8 | 0.270086 | 0.078931 | -2.15151 | 2.080265 |
| CLMN | 0.270086 | 0.078933 | 0.809923 | 7.016399 |
| HIST1H4E | 0.270086 | 0.078936 | 0.687474 | 5.356117 |
| MAP10 | 0.270086 | 0.078951 | 1.55869 | 2.878041 |
| NRSN2 | 0.270086 | 0.078955 | 0.959948 | 3.439171 |
| TNKS2 | 0.270087 | 0.078976 | -0.69674 | 7.699939 |
| PRKRA | 0.271164 | 0.079312 | 1.947014 | 0.593476 |
| H1FX-AS1 | 0.271754 | 0.079505 | 1.396562 | 2.398431 |
| MAP3K1 | 0.271892 | 0.079566 | -0.7069 | 7.258879 |
| CMYA5 | 0.271995 | 0.079617 | 1.007356 | 4.794016 |
| ARHGAP21 | 0.272288 | 0.079724 | 0.673283 | 7.9617 |
| MIR210 | 0.272411 | 0.07978 | -2.28312 | 0.49495 |
| FADD | 0.272671 | 0.079877 | 0.789328 | 4.716118 |
| ZSCAN12P1 | 0.272765 | 0.079925 | 1.406659 | 2.979073 |
| AVL9 | 0.27279 | 0.079954 | 0.668619 | 6.745046 |
| CNDP1 | 0.272862 | 0.080016 | 2.744204 | 0.77707 |
| OTULINL | 0.272862 | 0.080016 | 1.019735 | 4.566934 |
| DENND2A | 0.27342 | 0.080214 | 1.178651 | 3.923225 |
| RNF41 | 0.27342 | 0.080222 | 1.991865 | 1.050232 |
| SNORD71 | 0.273443 | 0.080249 | 1.227434 | 5.066989 |
| KRT37 | 0.273508 | 0.080289 | -3.88213 | 0.539388 |
| TOP1P1 | 0.273704 | 0.080367 | 0.862643 | 4.077684 |
| ASAH1 | 0.274438 | 0.080604 | -1.833 | 1.058006 |
| LOC100132 | 0.274606 | 0.080674 | 1.52946 | 1.320003 |
| ZNF653 | 0.27463 | 0.080704 | 1.035579 | 3.45049 |
| TSPYL1 | 0.27463 | 0.080723 | -0.74073 | 7.572099 |
| LINC00294 | 0.275306 | 0.080943 | 0.876546 | 4.278788 |
| CD300E | 0.275536 | 0.08107 | 1.799307 | 4.192301 |
| C19orf71 | 0.275536 | 0.081075 | 1.132018 | 2.915102 |
| LRP5 | 0.275536 | 0.081094 | 1.455262 | 2.330002 |
| FBXO4 | 0.275536 | 0.081094 | 2.03496 | 1.095054 |
| TRMT9B | 0.275656 | 0.08117 | 3.150934 | 0.633442 |
| TAOK2 | 0.275656 | 0.081172 | 0.77053 | 4.383694 |
| TMEM165 | 0.275684 | 0.081251 | -1.34527 | 2.51107 |
| GCC1 | 0.275684 | 0.081261 | 0.701729 | 5.344945 |
| TLR7 | 0.275684 | 0.081269 | 2.260302 | 1.440582 |
| PSTPIP1 | 0.275684 | 0.081278 | 0.97962 | 4.028002 |
| BRSK1 | 0.275684 | 0.081296 | 1.492588 | 3.053182 |

| | | | | |
|---|---|---|---|---|
| CLASP1 | 0.275684 | 0.081369 | -1.63061 | 1.459435 |
| COX5B | 0.275684 | 0.081376 | -0.7159 | 7.18619 |
| FNIP2 | 0.275684 | 0.081383 | 0.686325 | 6.045023 |
| KCNJ12 | 0.275684 | 0.081385 | 3.126244 | 1.486583 |
| LOC441204 | 0.275684 | 0.08139 | 2.103407 | 0.562571 |
| KCNJ8 | 0.27572 | 0.081422 | 1.780413 | 2.891985 |
| SULT2A1 | 0.275728 | 0.081445 | -4.4167 | 1.265029 |
| SCARNA7 | 0.275799 | 0.0815 | 0.716138 | 8.289225 |
| MIR1-1HG- | 0.275799 | 0.08152 | 2.875854 | 1.235484 |
| HECTD4 | 0.275799 | 0.081529 | -0.63858 | 8.528238 |
| LINC00696 | 0.276111 | 0.081642 | -2.28012 | 2.030366 |
| SECTM1 | 0.276289 | 0.081716 | 1.052506 | 3.851372 |
| FBXO45 | 0.276356 | 0.081757 | -0.70272 | 5.893414 |
| ERAP1 | 0.276459 | 0.081808 | -0.87674 | 4.835332 |
| NUP107 | 0.276944 | 0.081973 | 0.696709 | 6.184777 |
| LAPTM4B | 0.277043 | 0.082024 | 0.8491 | 5.426185 |
| DBNDD1 | 0.277089 | 0.082058 | -2.08921 | 1.311373 |
| GPATCH1 | 0.277615 | 0.082235 | 0.690558 | 4.981516 |
| EDRF1 | 0.278144 | 0.082413 | 2.467514 | 0.375274 |
| FAM210A | 0.278359 | 0.082526 | -1.70564 | 1.754818 |
| LOC143666 | 0.278359 | 0.08254 | -1.60133 | 2.556284 |
| CTSS | 0.278359 | 0.082557 | 1.994406 | 0.755192 |
| UBE2R2 | 0.278359 | 0.082573 | -0.67286 | 7.146698 |
| CD7 | 0.278359 | 0.082583 | 1.454549 | 2.583009 |
| CHL1 | 0.278444 | 0.082644 | -2.65697 | 1.591878 |
| OR7E2P | 0.278444 | 0.082655 | 1.657095 | 1.791818 |
| FZD2 | 0.278444 | 0.082679 | 2.230585 | 1.453997 |
| ZNF358 | 0.278444 | 0.082693 | 0.868695 | 4.616722 |
| STK10 | 0.278477 | 0.082724 | 0.721772 | 6.141684 |
| ARMH4 | 0.278488 | 0.082759 | 1.678592 | 3.105142 |
| SPATA12 | 0.278488 | 0.08277 | -1.17092 | 3.75642 |
| SOCS2 | 0.278527 | 0.082802 | 2.038151 | 1.55149 |
| OTOAP1 | 0.278755 | 0.082891 | 2.077178 | 1.01539 |
| GNL3 | 0.278789 | 0.082938 | -2.06162 | 0.815217 |
| C14orf132 | 0.278789 | 0.082944 | -3.6749 | 1.162888 |
| MIR3679 | 0.278882 | 0.082993 | 2.050786 | 1.427397 |
| PRDX5 | 0.278996 | 0.083068 | -1.52854 | 1.424331 |
| GPR180 | 0.278996 | 0.08307 | 0.710535 | 5.133893 |
| LOC101929 | 0.279025 | 0.083099 | -1.56805 | 2.781734 |
| APC | 0.27949 | 0.083259 | -1.5007 | 2.434572 |
| N4BP2L2-IT | 0.279534 | 0.083314 | 0.742075 | 5.828048 |
| CRACR2B | 0.279534 | 0.083332 | 2.229906 | 1.539557 |
| LINC01126 | 0.279534 | 0.083353 | 1.695161 | 1.87937 |
| MAPK1 | 0.279534 | 0.083394 | -0.63662 | 6.977421 |
| SORBS3 | 0.279534 | 0.083397 | 1.090473 | 3.045121 |

| | | | | |
|---|---|---|---|---|
| DNAJA4 | 0.279534 | 0.083412 | 1.985721 | 1.075248 |
| BAG3 | 0.279534 | 0.083452 | -0.76887 | 7.048035 |
| FLJ42393 | 0.279534 | 0.083461 | 0.782621 | 4.943117 |
| HIST1H4I | 0.279534 | 0.083464 | 1.045426 | 3.090812 |
| SCAMP1-A | 0.279649 | 0.083519 | 1.356613 | 2.714442 |
| EGLN1 | 0.279904 | 0.083617 | -0.66211 | 6.991619 |
| MAGEA1 | 0.279976 | 0.08366 | 3.912194 | 0.450358 |
| LINC02595 | 0.280285 | 0.083774 | -1.20126 | 3.214188 |
| SERPINA5 | 0.280381 | 0.08384 | 2.667668 | 2.363614 |
| PPP1R11 | 0.280381 | 0.083845 | -0.98163 | 4.416171 |
| HCG11 | 0.280412 | 0.083876 | 0.8317 | 4.783619 |
| MIR648 | 0.280873 | 0.084069 | 1.71543 | 0.898387 |
| STARD4-AS | 0.280873 | 0.084116 | 1.238492 | 4.195879 |
| PLXDC1 | 0.280873 | 0.084146 | 1.084559 | 4.915496 |
| PYGO1 | 0.280873 | 0.084148 | 1.328057 | 3.263219 |
| TBX6 | 0.280873 | 0.08415 | -1.40083 | 2.710131 |
| TNFSF8 | 0.280873 | 0.084156 | 1.366123 | 3.105925 |
| ISM1-AS1 | 0.280873 | 0.084164 | 1.747908 | 1.280738 |
| SPRED1 | 0.280896 | 0.084192 | 0.719782 | 6.057257 |
| ANKS1A | 0.280988 | 0.084241 | 0.662786 | 6.656337 |
| HFM1 | 0.281014 | 0.08427 | 2.194107 | 1.340102 |
| ATP8B5P | 0.281014 | 0.084291 | 2.680963 | 0.544646 |
| NSUN7 | 0.281343 | 0.084416 | -1.0233 | 3.805409 |
| SLC6A14 | 0.281343 | 0.084433 | 1.557381 | 5.953422 |
| LOC101927 | 0.281367 | 0.084462 | -2.26251 | 1.149389 |
| MIR553 | 0.281498 | 0.084536 | 1.839997 | 1.094709 |
| GREB1L | 0.281498 | 0.084553 | 2.022866 | 2.168461 |
| JPX | 0.281498 | 0.084573 | 0.885573 | 3.923885 |
| PHF14 | 0.281498 | 0.084594 | 0.720757 | 6.430329 |
| LRP1B | 0.281498 | 0.084626 | 1.748996 | 2.875415 |
| DIXDC1 | 0.281498 | 0.08463 | 1.997056 | 1.175933 |
| EFR3B | 0.281529 | 0.08466 | 1.297352 | 2.513348 |
| ZBTB42 | 0.281726 | 0.084752 | -0.93603 | 3.726153 |
| CYTH1 | 0.281726 | 0.084763 | -1.29432 | 3.391037 |
| RALGAPA2 | 0.281763 | 0.084802 | 0.692652 | 7.804282 |
| BATF | 0.281763 | 0.08484 | 1.732544 | 1.827467 |
| ARL2BP | 0.281763 | 0.084852 | -0.7501 | 5.410523 |
| LIPE-AS1 | 0.281763 | 0.08486 | -1.5869 | 2.143737 |
| SNORD55 | 0.281799 | 0.084892 | 1.127246 | 7.066078 |
| TCERG1L | 0.282316 | 0.085069 | -2.34818 | 0.693438 |
| RAI1-AS1 | 0.282582 | 0.085171 | -1.25818 | 3.091382 |
| CTNNB1 | 0.282811 | 0.085261 | 1.400343 | 2.230478 |
| FOXK1 | 0.283112 | 0.085374 | 0.683125 | 7.757525 |
| PITPNM2-A | 0.283167 | 0.085412 | -1.02523 | 2.893037 |
| PKD1P1 | 0.283185 | 0.085439 | 0.676926 | 6.856807 |

| | | | | |
|---|---|---|---|---|
| NAGA | 0.283337 | 0.085506 | 0.747262 | 4.846444 |
| VPS35L | 0.283932 | 0.085708 | 2.150995 | 1.674287 |
| ATAD5 | 0.284133 | 0.08579 | 0.635541 | 5.777676 |
| SMYD3 | 0.284204 | 0.085833 | 2.203967 | 0.930783 |
| KLKB1 | 0.284282 | 0.085878 | 2.277664 | 0.643549 |
| ADAMTS8 | 0.284315 | 0.08591 | 1.867585 | 3.049552 |
| LSM14B | 0.284381 | 0.085951 | -0.67655 | 5.903672 |
| GIHCG | 0.285187 | 0.086217 | 2.289791 | 1.002584 |
| CCDC122 | 0.28519 | 0.086254 | 1.010494 | 3.902767 |
| RAB39B | 0.28519 | 0.086261 | 2.469538 | 1.098452 |
| DPP9 | 0.285268 | 0.086306 | 0.642623 | 6.391743 |
| VAX2 | 0.285758 | 0.086476 | 2.48654 | 1.005752 |
| USP15 | 0.28636 | 0.086681 | -0.85668 | 4.421667 |
| APC2 | 0.286967 | 0.086902 | 1.096448 | 3.838637 |
| RIOK1 | 0.286967 | 0.086908 | -1.00342 | 4.611865 |
| LINC01264 | 0.287079 | 0.086975 | -4.59023 | 0.686735 |
| FUT10 | 0.287079 | 0.086986 | 0.686133 | 4.890046 |
| GDF6 | 0.28746 | 0.087123 | 2.568929 | 0.693126 |
| TMEM75 | 0.287647 | 0.087228 | -0.83039 | 4.785517 |
| LOC101927 | 0.287647 | 0.087246 | 1.636602 | 2.016984 |
| FAM216B | 0.287647 | 0.08725 | 2.830791 | 1.246106 |
| EXT1 | 0.287647 | 0.087283 | 0.713006 | 6.452261 |
| PCDHB13 | 0.287647 | 0.087289 | 1.559851 | 3.328141 |
| FXR2 | 0.28769 | 0.087333 | -0.71459 | 5.688606 |
| TNFRSF11E | 0.28769 | 0.087346 | 1.757338 | 2.327379 |
| LOC283335 | 0.28773 | 0.08738 | 1.043105 | 3.783228 |
| ATP23 | 0.28785 | 0.087439 | -3.00818 | 0.665164 |
| ZNF774 | 0.28795 | 0.087556 | 1.117257 | 2.356853 |
| PRSS53 | 0.28795 | 0.087569 | 1.502045 | 2.376688 |
| ATG9B | 0.28795 | 0.08757 | -2.10599 | 1.316995 |
| UBR1 | 0.28795 | 0.087572 | 0.698156 | 6.729525 |
| CRIP1 | 0.28795 | 0.087579 | 0.825541 | 4.280293 |
| RAB11FIP5 | 0.288152 | 0.087663 | 0.709498 | 5.184948 |
| TMEM245 | 0.288152 | 0.087684 | 0.651419 | 6.962703 |
| BLOC1S3 | 0.28824 | 0.087766 | 0.846267 | 3.83716 |
| RPS3A | 0.28824 | 0.087774 | -0.72292 | 5.518868 |
| SPTLC3 | 0.28824 | 0.087777 | -0.91587 | 4.296574 |
| SLC35D2 | 0.288315 | 0.087822 | 1.80936 | 1.665968 |
| NUP210 | 0.288877 | 0.088019 | 0.846043 | 5.789969 |
| FNIP1 | 0.288877 | 0.088037 | -2.07024 | 0.856361 |
| TRIM22 | 0.289198 | 0.088157 | 2.100486 | 1.37772 |
| APEX2 | 0.289262 | 0.088198 | 2.291704 | 0.533799 |
| GSDMB | 0.289544 | 0.088314 | 2.436454 | 0.882137 |
| LIFR-AS1 | 0.289544 | 0.088328 | -2.48187 | 1.380011 |
| PPP4R1L | 0.289737 | 0.088431 | 0.869449 | 4.264798 |

| | | | | |
|---|---|---|---|---|
| CXCL2 | 0.289737 | 0.088431 | 1.634928 | 4.71936 |
| AVPR1A | 0.289998 | 0.088535 | 1.43254 | 2.85321 |
| SNHG6 | 0.289998 | 0.088555 | 0.878358 | 5.255671 |
| ZSCAN5A | 0.290139 | 0.088636 | 1.829014 | 1.552573 |
| MIR3616 | 0.290139 | 0.088643 | 3.326038 | 0.984544 |
| CXCL14 | 0.29038 | 0.088755 | -1.24008 | 6.572393 |
| TMEM59L | 0.29038 | 0.08876 | 2.053254 | 1.069933 |
| LINC01106 | 0.290472 | 0.088861 | 1.096745 | 3.992015 |
| UBXN2A | 0.290472 | 0.088862 | -0.80928 | 5.032725 |
| WTIP | 0.290472 | 0.088863 | 1.361908 | 2.902386 |
| CKB | 0.290472 | 0.088877 | -1.41991 | 5.999202 |
| USP9Y | 0.290478 | 0.088908 | -1.86075 | 6.898986 |
| SH3KBP1 | 0.290478 | 0.088923 | 1.34842 | 2.094135 |
| PRDX6 | 0.290511 | 0.088955 | -0.6505 | 7.369559 |
| EID2B | 0.29117 | 0.08918 | 2.305125 | 0.877381 |
| SNORA54 | 0.291261 | 0.089229 | 0.951873 | 6.607104 |
| EHD4-AS1 | 0.291619 | 0.089361 | -1.47999 | 3.118175 |
| PXDC1 | 0.291683 | 0.089403 | 0.830723 | 4.894005 |
| TRIM3 | 0.291981 | 0.089517 | -2.88709 | 0.620601 |
| ANO7 | 0.292416 | 0.089673 | 1.550275 | 2.797738 |
| EEF2K | 0.292433 | 0.089725 | 0.660326 | 6.75852 |
| MIEN1 | 0.292433 | 0.089728 | -0.68854 | 4.760459 |
| RASSF3 | 0.292433 | 0.089744 | 1.885004 | 1.613952 |
| CYP51A1 | 0.292681 | 0.089843 | -1.09443 | 3.325541 |
| RCCD1 | 0.292815 | 0.089906 | 2.787767 | 0.483175 |
| MIR3173 | 0.292825 | 0.089932 | -2.34401 | 0.720113 |
| NBPF12 | 0.293693 | 0.090234 | 0.798931 | 5.818447 |
| PRICKLE2 | 0.293693 | 0.090243 | 0.817683 | 5.036846 |
| ADGRE4P | 0.293698 | 0.090267 | 1.896478 | 1.3923 |
| MAP2K1 | 0.293818 | 0.090326 | -0.68776 | 6.542019 |
| SPINK9 | 0.293935 | 0.090385 | 2.99489 | 0.569029 |
| DNAJC25 | 0.293989 | 0.090424 | 1.828804 | 0.789087 |
| MED15 | 0.294573 | 0.090644 | -0.96606 | 3.151696 |
| ACRBP | 0.294573 | 0.090648 | 1.532249 | 1.999216 |
| KCNIP2 | 0.294574 | 0.090673 | 2.523163 | 0.767695 |
| ZNF776 | 0.294574 | 0.090693 | 0.659791 | 5.717003 |
| AKAP5 | 0.294743 | 0.090787 | 1.058792 | 3.450848 |
| C1orf198 | 0.294743 | 0.09079 | 2.127491 | 0.780545 |
| LOC101927 | 0.294889 | 0.090904 | -1.80395 | 1.213656 |
| HACD2 | 0.294889 | 0.090905 | -0.82713 | 5.235801 |
| YTHDC2 | 0.294889 | 0.090941 | -0.61841 | 6.743733 |
| ELF3 | 0.294889 | 0.090948 | -1.50591 | 3.259175 |
| EIF5AL1 | 0.294889 | 0.090951 | -1.17507 | 4.715909 |
| C8orf88 | 0.294889 | 0.09097 | 2.157521 | 1.715522 |
| YARS | 0.294907 | 0.091004 | -0.68142 | 6.790489 |

| | | | | |
|---|---|---|---|---|
| INTS10 | 0.294907 | 0.091021 | 0.609703 | 6.744538 |
| LINC01979 | 0.295173 | 0.091125 | 1.503519 | 2.176495 |
| ACY3 | 0.295946 | 0.091398 | 2.370733 | 0.73569 |
| CTTNBP2N | 0.295946 | 0.091421 | -0.67461 | 7.101723 |
| KCTD15 | 0.295946 | 0.091432 | -1.27178 | 2.059678 |
| GAS6-DT | 0.296049 | 0.091486 | 2.355598 | 0.975127 |
| ALMS1P1 | 0.296064 | 0.091513 | 2.485094 | 1.132873 |
| RPL4 | 0.296482 | 0.091671 | -0.72603 | 9.872472 |
| PROSER2 | 0.296482 | 0.091688 | -1.17283 | 3.928041 |
| EPS15L1 | 0.296509 | 0.091731 | -0.82411 | 4.011427 |
| LOC150776 | 0.296509 | 0.091741 | 0.799751 | 4.823089 |
| NCDN | 0.29692 | 0.091891 | -3.31902 | 0.558351 |
| HK3 | 0.297181 | 0.091994 | 1.611814 | 1.874973 |
| POLR1D | 0.297265 | 0.092043 | -0.86931 | 4.566213 |
| SEC62 | 0.297705 | 0.092221 | 0.774459 | 8.235936 |
| SNORA73A | 0.297705 | 0.092225 | -0.81336 | 9.264381 |
| COL17A1 | 0.297773 | 0.09227 | -0.91195 | 7.950712 |
| TIMP4 | 0.297773 | 0.092311 | 2.030765 | 2.210778 |
| RPL24 | 0.297773 | 0.092329 | -0.68714 | 7.890677 |
| JTB | 0.297773 | 0.092336 | 0.703563 | 6.352344 |
| MMP15 | 0.297872 | 0.092392 | 0.916743 | 4.902652 |
| RPS13 | 0.297872 | 0.092425 | -0.68203 | 7.616788 |
| LOC100996 | 0.297872 | 0.092435 | 2.085295 | 1.413462 |
| SNORD90 | 0.297956 | 0.092484 | 1.367849 | 4.366023 |
| ADO | 0.29804 | 0.092533 | 1.028779 | 4.767034 |
| LOC105370 | 0.29812 | 0.09258 | -1.34897 | 2.367899 |
| LINC00243 | 0.298213 | 0.092656 | 2.661386 | 0.521062 |
| PABPC5 | 0.298213 | 0.092664 | 2.506429 | 0.89898 |
| DOCK3 | 0.298213 | 0.092693 | -0.98398 | 5.083739 |
| IKBKG | 0.298213 | 0.092702 | -2.88814 | 0.497015 |
| PKDREJ | 0.298213 | 0.092723 | 1.291452 | 2.059774 |
| OAS1 | 0.29824 | 0.092754 | 1.682381 | 1.935405 |
| GNAT2 | 0.298361 | 0.092814 | -1.02009 | 3.01403 |
| IDO2 | 0.298965 | 0.093025 | 2.846178 | 0.5378 |
| RPL28 | 0.299038 | 0.093071 | -1.12529 | 3.457471 |
| KHDRBS3 | 0.299093 | 0.093111 | 1.156866 | 3.478558 |
| SPTBN5 | 0.299358 | 0.093216 | 1.128403 | 5.778713 |
| SFRP5 | 0.299462 | 0.093271 | 2.759545 | 0.797456 |
| BUB3 | 0.299541 | 0.093336 | 0.729829 | 4.851082 |
| ACSS3 | 0.299541 | 0.093352 | 1.633337 | 2.491902 |
| MIR30E | 0.299541 | 0.093364 | 2.507208 | 0.283597 |
| PLPPR2 | 0.299752 | 0.093457 | -3.47065 | 0.53556 |
| RIPK3 | 0.299752 | 0.093475 | 0.894446 | 4.217012 |
| INTS13 | 0.299771 | 0.093516 | -0.65505 | 5.982802 |
| TMEM243 | 0.299771 | 0.093548 | 0.967172 | 3.443192 |

| | | | | |
|---|---|---|---|---|
| HILPDA | 0.299771 | 0.093554 | -2.89147 | 0.97904 |
| DPY19L2P1 | 0.299771 | 0.093573 | 1.757425 | 1.175587 |
| TTN | 0.299945 | 0.09365 | 1.465831 | 3.447092 |
| TRPM5 | 0.300041 | 0.093703 | 1.960618 | 2.733393 |
| GSAP | 0.300179 | 0.093787 | 0.773543 | 5.517961 |
| ISM1 | 0.300179 | 0.093792 | 1.340007 | 3.435547 |
| UBIAD1 | 0.300244 | 0.093835 | 0.812703 | 3.913654 |
| SNORD60 | 0.300244 | 0.093888 | 1.094788 | 7.001904 |
| SIX4 | 0.300244 | 0.093895 | 1.611522 | 3.644647 |
| RBPMS2 | 0.300244 | 0.093904 | 1.886798 | 3.366296 |
| RPL23AP53 | 0.300747 | 0.094097 | 0.66251 | 5.529868 |
| GJB7 | 0.300747 | 0.094119 | 1.732444 | 1.728599 |
| TMEM237 | 0.300747 | 0.094132 | -2.10776 | 0.634309 |
| LOC10192 | 0.300747 | 0.094153 | 2.365408 | 1.031065 |
| SCARNA22 | 0.300862 | 0.094211 | -0.95117 | 3.988564 |
| COX7B | 0.300948 | 0.094261 | -0.78197 | 5.657574 |
| MIR103A2 | 0.300976 | 0.094293 | -3.85753 | 0.6825 |
| C9orf139 | 0.301013 | 0.094327 | 1.93242 | 1.084614 |
| MIR215 | 0.301082 | 0.094372 | 4.216167 | 0.191708 |
| ATP6V1G1 | 0.301194 | 0.09443 | -0.63926 | 6.570783 |
| CTSH | 0.301523 | 0.094579 | 0.636873 | 5.851339 |
| TUBB4B | 0.301523 | 0.094592 | -0.72771 | 7.52933 |
| PTK2B | 0.301523 | 0.094608 | 1.820928 | 2.005965 |
| TRIP13 | 0.301523 | 0.094625 | 1.322638 | 2.628361 |
| TNFSF10 | 0.301642 | 0.094686 | -1.12387 | 2.907991 |
| EXOC8 | 0.301749 | 0.094742 | 0.653533 | 5.663838 |
| CALML3-AS | 0.301918 | 0.094818 | -2.62119 | 0.787255 |
| TFAP2B | 0.301951 | 0.094852 | -1.96359 | 2.282004 |
| PDIA4 | 0.302279 | 0.094978 | 0.712681 | 8.018683 |
| PGRMC2 | 0.302399 | 0.095038 | -0.64226 | 6.897613 |
| WDR78 | 0.302415 | 0.095083 | 2.275254 | 0.618686 |
| ZBTB20 | 0.302415 | 0.095105 | 0.975109 | 3.793538 |
| BMPR1A | 0.302415 | 0.09512 | 0.633406 | 5.695523 |
| RPS6KA6 | 0.302415 | 0.095136 | -1.62095 | 3.070723 |
| ATP6V1C1 | 0.302457 | 0.095172 | 0.641224 | 6.04247 |
| NWD2 | 0.302548 | 0.095243 | 2.343492 | 0.571361 |
| LINC00501 | 0.302548 | 0.095247 | 2.635369 | 0.733735 |
| IRF3 | 0.302674 | 0.095309 | 1.932301 | 1.653499 |
| ELK4 | 0.303308 | 0.095532 | 0.640956 | 7.629298 |
| PINK1 | 0.30344 | 0.095614 | -0.7463 | 4.642704 |
| RBPJ | 0.30344 | 0.09562 | 2.178079 | 0.780422 |
| CDK19 | 0.303583 | 0.095714 | -2.03671 | 1.122606 |
| RPS2 | 0.303583 | 0.095723 | -0.65567 | 8.646713 |
| GOLGA6L2 | 0.303583 | 0.095734 | 2.139944 | 1.123968 |
| ADAMTS1 | 0.303602 | 0.095763 | 0.842433 | 6.984227 |

| | | | | |
|---|---|---|---|---|
| PREX2 | 0.303655 | 0.095803 | 1.320449 | 3.582783 |
| CA13 | 0.303933 | 0.095914 | -0.75288 | 4.683751 |
| DPY19L2 | 0.304161 | 0.096009 | 0.977507 | 2.876068 |
| NRIP2 | 0.304438 | 0.096133 | 0.891354 | 4.406962 |
| DLG2 | 0.304438 | 0.096143 | 1.726698 | 1.829369 |
| FAM186A | 0.304773 | 0.09631 | 1.381044 | 2.111208 |
| TXNDC12 | 0.304773 | 0.096327 | 0.883694 | 4.543203 |
| RBP1 | 0.304773 | 0.096331 | -1.63999 | 3.292394 |
| LOC202181 | 0.304773 | 0.09637 | 0.789174 | 5.079776 |
| DSCR9 | 0.304773 | 0.096378 | 1.985086 | 1.067587 |
| LINC01655 | 0.304773 | 0.096398 | 2.576995 | 1.035424 |
| TCEAL4 | 0.304773 | 0.096417 | 1.620008 | 1.64363 |
| MARVELD3 | 0.304773 | 0.096439 | 1.152932 | 2.508047 |
| DIAPH2-AS | 0.304773 | 0.096459 | 1.301927 | 2.592247 |
| CDCA5 | 0.304773 | 0.096481 | 1.010083 | 3.935993 |
| NAGPA-AS1 | 0.304868 | 0.096535 | 2.875392 | 1.282696 |
| LINC02495 | 0.305239 | 0.096675 | 2.789052 | 0.88905 |
| TRAF2 | 0.305433 | 0.09676 | 0.683364 | 4.885975 |
| NPAT | 0.305484 | 0.0968 | 0.695823 | 5.640546 |
| LAMP2 | 0.305527 | 0.096836 | -0.70659 | 6.77354 |
| LOC100506 | 0.305632 | 0.096893 | 1.519603 | 2.653602 |
| HID1 | 0.30596 | 0.09702 | 1.264526 | 5.777087 |
| ZBTB7B | 0.306209 | 0.097175 | -1.56342 | 1.463202 |
| AMBRA1 | 0.306209 | 0.097191 | 0.93448 | 3.860737 |
| FCHSD1 | 0.306209 | 0.097259 | 0.97031 | 5.0653 |
| ADAM1A | 0.306209 | 0.097263 | 1.113347 | 3.613612 |
| KRT75 | 0.306209 | 0.097283 | -2.98303 | 1.36196 |
| SSTR5-AS1 | 0.306209 | 0.097293 | 2.346319 | 0.550386 |
| FAM111A-I | 0.306209 | 0.097303 | 1.64333 | 2.45746 |
| PKHD1 | 0.306209 | 0.097304 | 2.336044 | 2.254471 |
| MIR34AHG | 0.306209 | 0.09733 | 0.758753 | 5.600265 |
| LOC101928 | 0.306209 | 0.097348 | -2.43805 | 0.494256 |
| CT62 | 0.306209 | 0.097356 | -1.53226 | 1.164404 |
| RNU6ATAC | 0.30626 | 0.097395 | 0.955956 | 5.299741 |
| LOC101927 | 0.306369 | 0.097453 | 2.730223 | 0.939158 |
| KPNA3 | 0.30643 | 0.097496 | -0.67824 | 6.538692 |
| PRR5L | 0.306451 | 0.097526 | 2.001951 | 1.875655 |
| TMC6 | 0.30654 | 0.097578 | 2.456265 | 0.581988 |
| FGD6 | 0.306632 | 0.097631 | 0.638635 | 6.745588 |
| PDF | 0.307024 | 0.097793 | -1.69352 | 1.640051 |
| CFAP73 | 0.307024 | 0.097812 | 2.411179 | 0.499346 |
| UQCR11 | 0.307024 | 0.097825 | -0.6796 | 5.592028 |
| BRAT1 | 0.307141 | 0.0979 | 0.845602 | 4.976874 |
| TUBB1 | 0.307141 | 0.09791 | 1.48577 | 2.018415 |
| TP53INP1 | 0.307495 | 0.098049 | 2.596569 | 0.522374 |

| | | | | |
|---|---|---|---|---|
| LOC400867 | 0.307495 | 0.098069 | 2.287104 | 0.740509 |
| SAFB2 | 0.307608 | 0.098142 | 0.631114 | 7.620673 |
| MMP14 | 0.307608 | 0.098152 | 0.649702 | 7.982469 |
| PTPRN2 | 0.307771 | 0.098228 | 2.966236 | 1.172651 |
| LINC01484 | 0.307772 | 0.098251 | -3.21329 | 1.360442 |
| XPO4 | 0.307943 | 0.09833 | 0.591215 | 6.583584 |
| HERC2P9 | 0.307962 | 0.098359 | 0.771595 | 4.725377 |
| NKX6-1 | 0.308221 | 0.098473 | 2.121262 | 2.62313 |
| ALG11 | 0.308221 | 0.098489 | 0.813674 | 3.835004 |
| ZNF287 | 0.308487 | 0.098597 | 1.150261 | 3.086119 |
| ARMCX1 | 0.308551 | 0.098641 | 1.017167 | 3.27525 |
| ACBD7 | 0.309065 | 0.098833 | 2.169863 | 0.761714 |
| NXPH4 | 0.309065 | 0.098853 | -2.02643 | 2.244045 |
| ZNF75D | 0.3091 | 0.098888 | 0.989502 | 3.780547 |
| RPS6KL1 | 0.309187 | 0.098939 | 0.954873 | 3.533918 |
| C4orf19 | 0.309257 | 0.099002 | 2.661145 | 0.707335 |
| CRYM-AS1 | 0.309257 | 0.099009 | -1.02734 | 3.150821 |
| AUNIP | 0.309298 | 0.099067 | 1.968228 | 0.681891 |
| LRRC45 | 0.309298 | 0.099069 | 0.983848 | 4.600338 |
| PCDH17 | 0.309335 | 0.099104 | 0.938924 | 3.930188 |
| GNA12 | 0.309419 | 0.099154 | 1.430248 | 2.04035 |
| IL6 | 0.309712 | 0.099272 | 2.05849 | 2.502872 |
| FAM50A | 0.309789 | 0.09932 | 0.655561 | 6.386888 |
| PLS3 | 0.310245 | 0.09949 | -1.93663 | 1.802888 |
| CYP8B1 | 0.310802 | 0.099693 | -2.24296 | 0.893496 |
| ZSCAN18 | 0.310963 | 0.099774 | -2.09153 | 1.434328 |
| SLC6A9 | 0.310963 | 0.099792 | -2.18701 | 0.829748 |
| STX6 | 0.311505 | 0.099989 | -1.49458 | 2.088567 |
| ERICH3 | 0.311608 | 0.100046 | 2.221125 | 2.220929 |
| SH3TC2 | 0.311827 | 0.10014 | -0.8815 | 4.862564 |
| EIF3M | 0.311827 | 0.100164 | -0.87236 | 4.301 |
| NADSYN1 | 0.312002 | 0.100244 | -0.68684 | 6.442144 |
| LOC101928 | 0.312161 | 0.100319 | 1.176892 | 2.018476 |
| CPLX1 | 0.312171 | 0.100346 | 1.609765 | 2.027487 |
| SCAND2P | 0.31239 | 0.10044 | -1.0623 | 2.513592 |
| UBA7 | 0.312449 | 0.100495 | 0.932206 | 5.429834 |
| MELTF-AS1 | 0.312449 | 0.100507 | 1.60461 | 1.935546 |
| KDELR3 | 0.312502 | 0.100548 | 1.37104 | 3.769842 |
| VPS29 | 0.312614 | 0.100607 | 1.840768 | 1.61987 |
| B3GNT3 | 0.312634 | 0.100638 | 0.744423 | 6.439602 |
| HPS4 | 0.313076 | 0.100804 | -1.71337 | 1.228128 |
| MCUR1 | 0.313755 | 0.101046 | -0.69379 | 5.993881 |
| FOXA3 | 0.313814 | 0.101089 | 1.837856 | 2.491411 |
| LOC100131 | 0.313879 | 0.101134 | 2.123425 | 2.429475 |
| TMEM120B | 0.314008 | 0.1012 | 0.65634 | 4.883661 |

| | | | | |
|---|---|---|---|---|
| SEPT7P2 | 0.314217 | 0.101291 | 0.788085 | 4.556293 |
| GRAP | 0.314434 | 0.101385 | 1.556332 | 1.685044 |
| LOC101928 | 0.31455 | 0.101457 | 1.475123 | 3.772813 |
| DNAJC16 | 0.31455 | 0.101487 | 2.775405 | 0.678442 |
| ANXA6 | 0.31455 | 0.101499 | 1.889552 | 1.851832 |
| FAM72C | 0.31455 | 0.101518 | 2.470274 | 0.304692 |
| FOXN3-AS1 | 0.314818 | 0.101629 | -1.39282 | 1.774768 |
| FNDC3A | 0.314916 | 0.10169 | 1.375183 | 2.575857 |
| PLXNB1 | 0.314916 | 0.101728 | -1.50179 | 2.34402 |
| ENDOG | 0.314916 | 0.101732 | -1.54342 | 0.903719 |
| SLC9A7P1 | 0.314935 | 0.101777 | 2.346031 | 1.114186 |
| FLT1 | 0.314935 | 0.10181 | 0.736145 | 4.923477 |
| SLC39A3 | 0.314935 | 0.10181 | 0.953757 | 3.062258 |
| CYP7B1 | 0.31498 | 0.101858 | -1.18206 | 4.081941 |
| SLC1A5 | 0.31498 | 0.101882 | 0.922641 | 5.235406 |
| NLRX1 | 0.31498 | 0.101897 | -1.47679 | 1.923295 |
| RNF115 | 0.314991 | 0.101936 | 0.713427 | 5.479623 |
| TNIK | 0.314991 | 0.101948 | 2.058382 | 1.273282 |
| DDX10 | 0.315026 | 0.101984 | 0.731827 | 6.523333 |
| TTC30A | 0.315047 | 0.102015 | 0.910899 | 3.440738 |
| LYG1 | 0.315335 | 0.102132 | 1.933609 | 1.663423 |
| ZFAND2A | 0.315446 | 0.102203 | 0.779798 | 4.15111 |
| LINC-PINT | 0.315446 | 0.102216 | 0.939731 | 4.313154 |
| LRRC4 | 0.315497 | 0.102269 | -0.81736 | 5.118744 |
| LOC100288 | 0.315497 | 0.10228 | 1.528774 | 2.266098 |
| TMEM33 | 0.316282 | 0.102559 | -0.64807 | 6.931064 |
| CKS2 | 0.316327 | 0.102598 | 1.236126 | 3.640434 |
| ABLIM2 | 0.31636 | 0.102633 | -1.21539 | 2.984341 |
| ILVBL | 0.316367 | 0.102659 | -0.61552 | 5.606613 |
| DDX58 | 0.316383 | 0.102716 | 0.729016 | 6.219922 |
| RNF122 | 0.316383 | 0.102719 | 1.054262 | 2.649949 |
| SNORA73B | 0.316383 | 0.102736 | -0.7555 | 9.903274 |
| ELMO3 | 0.316851 | 0.102919 | -0.75365 | 4.895765 |
| TRAM1L1 | 0.316851 | 0.102937 | 2.535826 | 0.505778 |
| ZNF252P | 0.317368 | 0.10313 | 0.662679 | 5.389291 |
| TRPM4 | 0.317368 | 0.103181 | 1.6234 | 1.620792 |
| RPL27 | 0.317368 | 0.103195 | -0.63928 | 8.323832 |
| LAMTOR1 | 0.317368 | 0.103213 | -0.61534 | 5.88772 |
| LINC00423 | 0.317368 | 0.103225 | -4.24626 | 0.82435 |
| DENND5B | 0.317563 | 0.103313 | 2.065802 | 0.928959 |
| SNORD61 | 0.317809 | 0.103418 | 1.316099 | 3.898908 |
| SPRYD4 | 0.318209 | 0.103572 | -0.93912 | 4.04022 |
| LINC02585 | 0.318339 | 0.103638 | 2.115331 | 0.516385 |
| MIR1914 | 0.31867 | 0.103782 | -1.40509 | 1.781877 |
| PWAR5 | 0.31867 | 0.103795 | 0.907063 | 5.014939 |

| | | | | |
|---|---|---|---|---|
| LCLAT1 | 0.318763 | 0.103849 | 2.379561 | 0.918345 |
| CC2D2A | 0.318934 | 0.103929 | 0.655416 | 5.532508 |
| ZMYM5 | 0.318975 | 0.103981 | -0.96335 | 4.076836 |
| SNORD111 | 0.318975 | 0.103991 | 1.319173 | 2.712066 |
| RPS6KA2 | 0.319171 | 0.104079 | 1.480388 | 2.80588 |
| IL36G | 0.319359 | 0.104165 | -2.86168 | 0.45976 |
| TTLL4 | 0.319617 | 0.104274 | 0.638914 | 5.462307 |
| CLDN17 | 0.320004 | 0.10444 | -3.30585 | 0.767888 |
| FAM50B | 0.320004 | 0.104467 | 1.473569 | 2.104484 |
| YME1L1 | 0.320004 | 0.104473 | 1.786772 | 1.567658 |
| MEP1A | 0.320097 | 0.10455 | 3.908862 | 1.288773 |
| SMS | 0.320097 | 0.104558 | 1.488075 | 2.037134 |
| DUXAP10 | 0.320097 | 0.104602 | 2.56783 | 0.518483 |
| GPR137B | 0.320097 | 0.10461 | 1.113381 | 4.788708 |
| RASSF10 | 0.320097 | 0.104625 | -1.09783 | 3.906285 |
| LOC10013( | 0.32029 | 0.104731 | 1.575613 | 1.675089 |
| CNPY3 | 0.32029 | 0.104737 | 0.852027 | 5.256766 |
| TBC1D23 | 0.321518 | 0.105167 | -2.35739 | 0.496411 |
| ARAP3 | 0.321518 | 0.105188 | 0.825961 | 4.761073 |
| SMIM10 | 0.321533 | 0.105217 | 1.634411 | 2.420002 |
| ADGRE5 | 0.321572 | 0.105271 | 1.749094 | 1.77542 |
| TRAM2-AS: | 0.321572 | 0.105284 | 2.726905 | 0.32251 |
| MCU | 0.321572 | 0.105309 | 1.138435 | 3.918427 |
| PFDN5 | 0.321572 | 0.105329 | 1.087473 | 2.665948 |
| RARS | 0.321572 | 0.105375 | -0.61865 | 6.283273 |
| ERBB2 | 0.321572 | 0.105377 | -1.66713 | 1.592597 |
| MAPK1IP1I | 0.32166 | 0.105476 | -0.62582 | 7.528663 |
| EIF1 | 0.32166 | 0.105477 | -0.6174 | 7.909171 |
| OGFOD2 | 0.32166 | 0.105499 | 1.448762 | 1.545438 |
| USP11 | 0.32166 | 0.105504 | 0.621275 | 5.842187 |
| SOX12 | 0.321759 | 0.105585 | 0.684708 | 4.862732 |
| CNTN2 | 0.321759 | 0.105586 | -1.15633 | 2.297854 |
| CNNM4 | 0.321796 | 0.105637 | 0.641622 | 5.695871 |
| RPS8 | 0.321796 | 0.105647 | -0.69605 | 9.206418 |
| COL14A1 | 0.322226 | 0.105821 | 0.893013 | 8.074011 |
| ROCK1 | 0.322226 | 0.105837 | 0.585303 | 8.568376 |
| KIAA1551 | 0.322313 | 0.10589 | 0.665797 | 7.706189 |
| HIST1H2BK | 0.322371 | 0.105934 | -1.32901 | 3.095658 |
| OSTM1 | 0.322627 | 0.106043 | 0.665915 | 5.136838 |
| PGAP1 | 0.322722 | 0.106105 | -0.63038 | 6.148084 |
| UGP2 | 0.322722 | 0.106123 | -0.72575 | 5.088699 |
| GJA3 | 0.32294 | 0.106221 | -1.78094 | 1.593145 |
| TET2-AS1 | 0.32294 | 0.106244 | -1.04357 | 3.476231 |
| FAM76B | 0.323039 | 0.10634 | 0.611067 | 5.392628 |
| RAMP3 | 0.323039 | 0.106381 | 1.612762 | 1.840227 |

| | | | | |
|---|---|---|---|---|
| PDE12 | 0.323039 | 0.106398 | -0.62413 | 5.764575 |
| LOC10019( | 0.323039 | 0.106404 | 0.686302 | 6.149857 |
| CD27-AS1 | 0.323039 | 0.106414 | 1.006917 | 3.217381 |
| SCARNA4 | 0.323039 | 0.106424 | 1.018876 | 8.358527 |
| TOMM70 | 0.323154 | 0.106488 | -0.59299 | 6.84511 |
| EZR-AS1 | 0.323154 | 0.106511 | -1.156 | 3.123461 |
| KCTD5 | 0.323455 | 0.106635 | -0.63729 | 5.525454 |
| KLHL6-AS1 | 0.323698 | 0.10674 | 2.242472 | 1.000065 |
| LINC01446 | 0.323885 | 0.106826 | 3.066981 | 1.776757 |
| SLC25A15 | 0.323885 | 0.106851 | 0.782185 | 4.416382 |
| ATP4A | 0.324287 | 0.107008 | -3.68257 | 8.58219 |
| TTLL13P | 0.32435 | 0.107054 | 1.755294 | 1.609866 |
| TMED6 | 0.324482 | 0.107122 | -2.0433 | 2.73877 |
| ELOA | 0.324716 | 0.107224 | -0.61709 | 7.041835 |
| ARF3 | 0.325034 | 0.107375 | -0.6346 | 7.607983 |
| ZRANB1 | 0.325034 | 0.107379 | -0.64463 | 7.103921 |
| MRPS31P5 | 0.325174 | 0.10745 | 1.783752 | 1.648756 |
| SPATA46 | 0.325349 | 0.107532 | -1.49586 | 1.707207 |
| BMP2 | 0.325421 | 0.107581 | 0.920657 | 4.859614 |
| TNRC6C-AS | 0.325749 | 0.107721 | 0.946371 | 3.637154 |
| REEP6 | 0.325749 | 0.107739 | -1.03485 | 3.549366 |
| ITGA3 | 0.325935 | 0.107825 | 0.662535 | 7.429075 |
| SVBP | 0.325961 | 0.107876 | 1.38089 | 2.100136 |
| NTN4 | 0.325961 | 0.107884 | 1.025266 | 5.376219 |
| FAM98A | 0.326236 | 0.107999 | -1.67822 | 1.787571 |
| FAM120C | 0.326488 | 0.108122 | -2.12063 | 1.343277 |
| MFN1 | 0.326488 | 0.108133 | -0.60052 | 7.257107 |
| BMPER | 0.326564 | 0.108183 | 1.930929 | 1.615164 |
| TFIP11 | 0.327127 | 0.108394 | 2.366986 | 1.563929 |
| PDLIM5 | 0.327207 | 0.108458 | -1.32929 | 3.358757 |
| C1QTNF6 | 0.327207 | 0.108471 | 2.061211 | 1.498791 |
| TAAR1 | 0.327478 | 0.108585 | 3.634976 | 0.518603 |
| SYNGR2 | 0.328152 | 0.108856 | 0.611908 | 6.872802 |
| SULF2 | 0.328152 | 0.108878 | -2.68284 | 0.403997 |
| CNTN3 | 0.328152 | 0.108884 | 2.052289 | 2.430211 |
| TMEM151/ | 0.328182 | 0.108919 | 2.34403 | 1.289186 |
| MCM3 | 0.328214 | 0.108955 | 2.147051 | 0.79181 |
| MAOA | 0.32829 | 0.109005 | -2.10398 | 0.665487 |
| PRKCB | 0.3284 | 0.109066 | 1.382632 | 3.775455 |
| LOC441242 | 0.328434 | 0.109103 | 1.036478 | 2.559134 |
| EXTL2 | 0.328669 | 0.109206 | 2.161983 | 0.588994 |
| CLDN23 | 0.32908 | 0.109367 | 1.201602 | 3.522565 |
| PCDHB19P | 0.329222 | 0.10944 | 1.905419 | 2.443708 |
| COL21A1 | 0.32967 | 0.109628 | 1.474073 | 3.62063 |
| PDE5A | 0.32967 | 0.109639 | 1.778479 | 1.818707 |

| | | | | |
|---|---|---|---|---|
| YBX3P1 | 0.329778 | 0.1097 | -0.82064 | 3.65464 |
| SNORD20 | 0.329806 | 0.109734 | 1.267881 | 3.706543 |
| LINC01100 | 0.329925 | 0.109799 | -2.40277 | 1.476285 |
| AOC4P | 0.32996 | 0.109848 | 2.351994 | 1.328152 |
| LOC10272[illegible] | 0.32996 | 0.109876 | -1.98055 | 1.22415 |
| FAM81A | 0.32996 | 0.109898 | 1.80203 | 1.871171 |
| SRGAP2D | 0.32996 | 0.109922 | 0.689887 | 6.297156 |
| PAQR6 | 0.32996 | 0.109938 | -1.72947 | 1.224035 |
| DUBR | 0.32996 | 0.109961 | 1.295135 | 3.23818 |
| TULP3 | 0.329974 | 0.109991 | -0.62368 | 5.76354 |
| SEPHS2 | 0.330159 | 0.110078 | 0.635169 | 5.915912 |
| TTC28 | 0.33066 | 0.11027 | 0.707727 | 6.076797 |
| MX2 | 0.331327 | 0.110518 | 0.752511 | 5.541763 |
| SPAG7 | 0.331355 | 0.110553 | -0.58653 | 5.556688 |
| ZNF229 | 0.331564 | 0.110648 | 2.928523 | 0.294126 |
| OSGEPL1-A | 0.331564 | 0.110673 | 2.181318 | 0.648675 |
| ACBD4 | 0.331672 | 0.110752 | 2.353598 | 0.475893 |
| POLR2I | 0.331672 | 0.11076 | -0.69668 | 4.47205 |
| F3 | 0.331922 | 0.110875 | -1.33385 | 3.446773 |
| TPX2 | 0.331922 | 0.110894 | 0.712436 | 6.008485 |
| ADPGK-AS1 | 0.331922 | 0.110932 | 1.614391 | 1.502501 |
| SFMBT1 | 0.331922 | 0.110962 | 0.650655 | 5.895788 |
| GALNT15 | 0.331922 | 0.110988 | 1.522451 | 3.536369 |
| SNORD68 | 0.331922 | 0.110995 | 1.108181 | 4.915675 |
| SUDS3 | 0.331928 | 0.111022 | -0.59796 | 7.405984 |
| ST18 | 0.331946 | 0.111055 | 1.928992 | 3.419515 |
| F11 | 0.331946 | 0.111079 | 2.482175 | 0.754789 |
| 6-Sep | 0.332065 | 0.111144 | 1.3566 | 2.394785 |
| RAF1 | 0.332218 | 0.111245 | -0.56089 | 7.218792 |
| KDM4B | 0.332218 | 0.11129 | -0.58376 | 6.553817 |
| SERP2 | 0.332218 | 0.111295 | 2.345945 | 0.790341 |
| ARMC7 | 0.332218 | 0.111296 | 2.275877 | 0.762556 |
| FAM90A25 | 0.332717 | 0.111489 | 2.514548 | 0.366162 |
| RASD2 | 0.332936 | 0.111588 | 2.039482 | 1.605785 |
| EIF2S1 | 0.333004 | 0.111636 | -0.64583 | 6.623898 |
| NCOA2 | 0.333048 | 0.111676 | -0.58892 | 7.421206 |
| LOC101927 | 0.333273 | 0.111777 | 1.657131 | 1.26392 |
| LOC101928 | 0.333462 | 0.111865 | 1.971343 | 2.393871 |
| FLJ37453 | 0.333759 | 0.111991 | 0.96177 | 2.812326 |
| RB1 | 0.33389 | 0.11206 | -0.57673 | 7.282334 |
| SLC16A11 | 0.334023 | 0.11213 | -1.39704 | 1.703525 |
| LIX1L | 0.334082 | 0.112175 | 0.868628 | 5.220035 |
| UBE2S | 0.334169 | 0.11223 | 0.881425 | 3.806069 |
| RAB7A | 0.334397 | 0.112332 | -0.64391 | 8.40838 |
| CROCCP3 | 0.335454 | 0.112719 | 0.796808 | 4.508073 |

| | | | | |
|---|---|---|---|---|
| FAM92A | 0.335454 | 0.112738 | 1.621819 | 1.978086 |
| TSPY26P | 0.335467 | 0.112768 | 1.619956 | 1.178984 |
| SNORA77 | 0.335505 | 0.112835 | 1.508397 | 2.442924 |
| GFPT1 | 0.335505 | 0.112842 | 1.725791 | 1.425167 |
| UPK2 | 0.335505 | 0.112858 | -2.5475 | 0.472567 |
| A2MP1 | 0.335534 | 0.112893 | 1.987134 | 1.411387 |
| FOXD2 | 0.33559 | 0.112937 | 1.899771 | 2.318505 |
| MIR762HG | 0.335875 | 0.113077 | 2.525307 | 0.429446 |
| TSC22D2 | 0.335875 | 0.113085 | -1.71385 | 1.899051 |
| USP36 | 0.335875 | 0.11311 | 0.632141 | 6.861522 |
| SLC25A53 | 0.336136 | 0.113224 | 1.324031 | 2.441153 |
| LOC10050[illegible] | 0.336161 | 0.113267 | 1.492102 | 2.349338 |
| NDN | 0.336161 | 0.1133 | 1.010173 | 3.160883 |
| TOGARAM[illegible] | 0.336161 | 0.113309 | 1.855248 | 1.504514 |
| SBK1 | 0.336189 | 0.113344 | 1.191657 | 2.146597 |
| PLA2G5 | 0.336227 | 0.113382 | 2.204009 | 1.039685 |
| H3F3AP4 | 0.336283 | 0.113427 | -0.70679 | 6.201141 |
| TFAP2E | 0.3363 | 0.113458 | 1.147695 | 2.639516 |
| RASAL3 | 0.336319 | 0.113511 | 1.079834 | 3.448306 |
| RETREG1 | 0.336319 | 0.113516 | 1.772667 | 1.089203 |
| RPS18P9 | 0.336339 | 0.113571 | 1.142441 | 2.064067 |
| NOL7 | 0.336339 | 0.113574 | 1.251633 | 2.441793 |
| SDHAP1 | 0.336458 | 0.11364 | -0.56794 | 6.08084 |
| RHBDD3 | 0.336494 | 0.113689 | 0.998336 | 3.083908 |
| EHD4 | 0.336494 | 0.113706 | -0.75033 | 6.237638 |
| CYP27B1 | 0.336494 | 0.113729 | 1.338896 | 2.022635 |
| PPM1B | 0.33658 | 0.113784 | 0.825026 | 4.449667 |
| SNORD59B | 0.337114 | 0.11399 | 1.109949 | 3.185838 |
| ADAM17 | 0.337257 | 0.114064 | -0.61604 | 6.839299 |
| RELL1 | 0.337834 | 0.114285 | 0.765845 | 4.859101 |
| UQCRBP1 | 0.338091 | 0.114397 | 1.345025 | 1.989915 |
| SNORD45B | 0.338115 | 0.114431 | 0.812245 | 5.258783 |
| PMF1 | 0.338238 | 0.114519 | 2.050381 | 0.742261 |
| NEK2 | 0.338238 | 0.114525 | 1.675064 | 2.178085 |
| STC1 | 0.338284 | 0.114566 | 1.264767 | 5.113655 |
| LINC00865 | 0.338672 | 0.114755 | 1.354354 | 2.25733 |
| IL1RAP | 0.338672 | 0.114785 | -2.17478 | 0.814025 |
| TMEM204 | 0.338672 | 0.114841 | 2.069927 | 0.417333 |
| LCE3D | 0.338672 | 0.114853 | -2.5043 | 1.685844 |
| STK32A | 0.338672 | 0.114874 | 1.972971 | 0.782905 |
| TM4SF1 | 0.338672 | 0.114877 | -0.69695 | 8.482843 |
| FAM206A | 0.338672 | 0.114878 | -0.77505 | 4.767428 |
| TXN2 | 0.339 | 0.115023 | -0.65163 | 5.485894 |
| ADRB1 | 0.339 | 0.115041 | 2.17377 | 0.888774 |
| TMEM187 | 0.339018 | 0.115114 | -0.90146 | 2.952126 |

| | | | | |
|---|---|---|---|---|
| CHRM3 | 0.339018 | 0.115122 | 1.927594 | 2.925919 |
| RAB22A | 0.339018 | 0.115124 | -0.60841 | 7.702007 |
| LOC101927 | 0.339018 | 0.11515 | 2.522081 | 0.812204 |
| ORMDL3 | 0.339753 | 0.115426 | 0.63618 | 5.81822 |
| NFKBIE | 0.339788 | 0.115465 | 1.265961 | 3.420704 |
| CXCL10 | 0.339788 | 0.115523 | 1.583925 | 2.51458 |
| MARS2 | 0.339788 | 0.11554 | 1.031143 | 3.890581 |
| PIK3R1 | 0.339788 | 0.115541 | 0.691853 | 5.767991 |
| USP12-AS1 | 0.339869 | 0.115595 | 1.295652 | 2.239708 |
| CHMP7 | 0.339935 | 0.115643 | 1.815983 | 0.504996 |
| ARHGEF3 | 0.339935 | 0.115669 | -0.83228 | 3.339341 |
| MAK16 | 0.340181 | 0.115779 | 0.641744 | 5.320304 |
| C16orf54 | 0.340555 | 0.115945 | 1.669763 | 2.234747 |
| SFRP1 | 0.340555 | 0.116004 | 1.023393 | 5.15405 |
| DENR | 0.340555 | 0.116005 | -0.58718 | 6.939239 |
| GPRC5D | 0.340555 | 0.11601 | 1.2867 | 3.739239 |
| CTSW | 0.340632 | 0.116062 | 1.395371 | 2.091658 |
| EBLN3P | 0.34105 | 0.11625 | 0.620124 | 6.879596 |
| SCNN1A | 0.34105 | 0.11629 | -0.8801 | 6.371915 |
| PLPP5 | 0.34105 | 0.116307 | 0.830585 | 4.378663 |
| RABAC1 | 0.34105 | 0.116309 | 0.725702 | 4.414493 |
| MRPL47 | 0.341246 | 0.116401 | -1.76868 | 0.619621 |
| SMIM3 | 0.341412 | 0.116484 | 0.771027 | 4.234798 |
| LRRFIP1 | 0.3418 | 0.116642 | -0.7297 | 4.639701 |
| TP53BP2 | 0.342235 | 0.116828 | -2.04953 | 1.242912 |
| NIPA2 | 0.342235 | 0.116843 | -1.81415 | 0.930705 |
| STAM | 0.342393 | 0.116936 | -2.68481 | 0.819686 |
| CXCR1 | 0.342393 | 0.116967 | 2.004638 | 1.643326 |
| ATRIP | 0.342393 | 0.116975 | 2.205313 | 0.536125 |
| DDX24 | 0.342474 | 0.117029 | 1.199333 | 3.715305 |
| C22orf34 | 0.342571 | 0.117107 | 1.303982 | 2.488858 |
| MARK2 | 0.342571 | 0.117114 | -1.6056 | 1.944873 |
| ARL14EP | 0.342785 | 0.117214 | 1.064732 | 4.22685 |
| ABHD8 | 0.342786 | 0.11724 | 0.989191 | 2.852386 |
| SNAPC4 | 0.343011 | 0.117343 | 0.618818 | 5.466343 |
| RTN4 | 0.343158 | 0.117419 | -0.82862 | 4.240907 |
| FAM241A | 0.343442 | 0.117543 | -0.754 | 4.369391 |
| SNORA14B | 0.343691 | 0.117654 | 0.904739 | 7.529818 |
| FER | 0.343808 | 0.117737 | -1.11946 | 2.422796 |
| DHRS7 | 0.343808 | 0.117756 | -0.766 | 5.426356 |
| MRGBP | 0.343808 | 0.117773 | 0.784238 | 4.394101 |
| NOP10 | 0.344271 | 0.117958 | -0.6526 | 5.211022 |
| VCP | 0.34432 | 0.118001 | -0.57752 | 8.419975 |
| SMCO2 | 0.344992 | 0.118257 | -1.20647 | 2.334412 |
| RUFY3 | 0.345077 | 0.118318 | -0.83141 | 3.270566 |

| | | | | |
|---|---|---|---|---|
| VSIG10 | 0.345077 | 0.118339 | -0.8154 | 5.610482 |
| HLTF | 0.345094 | 0.118371 | 1.633859 | 1.675062 |
| LNPK | 0.345158 | 0.118419 | 1.96454 | 0.45541 |
| KIF13B | 0.345172 | 0.118451 | 0.642047 | 8.295775 |
| SPATA6L | 0.345623 | 0.118632 | 1.082614 | 3.587859 |
| CDK14 | 0.345722 | 0.118692 | 1.966537 | 1.182855 |
| ENDOD1 | 0.345841 | 0.118759 | -0.71734 | 7.00031 |
| DIPK1A | 0.345894 | 0.118806 | -0.76056 | 4.264417 |
| VSIG8 | 0.345894 | 0.11883 | -1.29128 | 3.384845 |
| RPL37A | 0.346174 | 0.118953 | -0.63813 | 8.474114 |
| MIR644A | 0.346754 | 0.119178 | -2.07654 | 0.474363 |
| MRPS28 | 0.346875 | 0.119272 | -0.67898 | 4.230592 |
| PRR7 | 0.346875 | 0.119282 | 2.066632 | 0.972786 |
| PRPF3 | 0.346875 | 0.119301 | 0.614052 | 6.917943 |
| DPYD | 0.346875 | 0.119326 | -0.74011 | 5.887894 |
| ZBED3-AS1 | 0.347298 | 0.119498 | 2.011277 | 1.053616 |
| PHKG2 | 0.347332 | 0.119536 | 1.138257 | 1.957672 |
| EPHA8 | 0.347408 | 0.119589 | -2.5092 | 0.817782 |
| AMN | 0.34741 | 0.119616 | 1.31031 | 3.574194 |
| SVIL-AS1 | 0.347472 | 0.119664 | -1.38831 | 2.47419 |
| SMURF2 | 0.347518 | 0.119706 | 0.649474 | 5.607457 |
| KLC1 | 0.347687 | 0.119791 | -0.75524 | 4.68028 |
| PIGZ | 0.347695 | 0.11983 | 1.138883 | 2.908598 |
| MIR627 | 0.347695 | 0.119847 | -1.88455 | 0.893415 |
| LOC101927 | 0.347731 | 0.119885 | 1.410163 | 2.511386 |
| TPM3 | 0.347813 | 0.11994 | 0.825214 | 5.836193 |
| RSPRY1 | 0.347816 | 0.119968 | 1.372899 | 2.130859 |
| LOC100129 | 0.347897 | 0.120045 | -0.86437 | 4.199447 |
| ST6GALNA( | 0.347897 | 0.120049 | 1.694853 | 2.224587 |
| RPL27A | 0.347913 | 0.120081 | -0.67361 | 9.036152 |
| ADORA2B | 0.348001 | 0.120137 | -1.01004 | 3.600285 |
| IRX6 | 0.348275 | 0.120263 | -2.98357 | 0.657317 |
| NPAS1 | 0.348275 | 0.120307 | 1.464681 | 1.493565 |
| ZBTB20-AS | 0.348275 | 0.120312 | 1.729289 | 1.309042 |
| GRAMD1C | 0.348313 | 0.120351 | 1.182845 | 2.287591 |
| PRR34 | 0.348325 | 0.120382 | 1.127747 | 3.121467 |
| DPH2 | 0.348664 | 0.120526 | 1.333489 | 2.030151 |
| KAT2A | 0.348833 | 0.120611 | 0.604413 | 5.741457 |
| LOC101929 | 0.348989 | 0.120701 | -1.24565 | 2.398634 |
| TMEM80 | 0.348989 | 0.120718 | -1.19091 | 1.747829 |
| GNAO1 | 0.349184 | 0.120812 | 1.246793 | 3.252478 |
| SNORD17 | 0.34944 | 0.120927 | 0.686332 | 9.088798 |
| ZNF254 | 0.349521 | 0.120982 | -1.35425 | 1.582933 |
| FLJ44635 | 0.349674 | 0.121094 | 1.574736 | 1.888703 |
| RCC1L | 0.349674 | 0.121096 | 0.986632 | 3.506857 |

| | | | | |
|---|---|---|---|---|
| ZBTB2 | 0.349674 | 0.121152 | 0.66634 | 4.305026 |
| INPP4A | 0.349674 | 0.121154 | 1.628855 | 1.826186 |
| THOC6 | 0.349674 | 0.121168 | -1.99746 | 0.399277 |
| FOXO6 | 0.349865 | 0.121261 | 1.541169 | 2.018528 |
| IQCH | 0.350696 | 0.121576 | 2.405056 | 0.659107 |
| UBE2U | 0.35103 | 0.121718 | 3.111351 | 0.984793 |
| ZAP70 | 0.35117 | 0.121794 | 1.588956 | 1.846533 |
| ACKR3 | 0.351348 | 0.121882 | -1.09326 | 4.10168 |
| DCAF15 | 0.351635 | 0.122071 | -0.6685 | 4.976509 |
| TIGD7 | 0.351635 | 0.122096 | 0.93638 | 3.908421 |
| LOC642366 | 0.351635 | 0.122096 | 2.770739 | 2.082313 |
| F11R | 0.351635 | 0.122096 | -0.57899 | 8.318248 |
| DTD1 | 0.351635 | 0.122122 | -0.67924 | 5.094302 |
| ZNF440 | 0.351635 | 0.122142 | -0.623 | 5.584881 |
| TRIM44 | 0.351669 | 0.122181 | 0.664634 | 8.065916 |
| PLEKHN1 | 0.351854 | 0.122272 | -2.02537 | 0.47946 |
| MIR4505 | 0.352097 | 0.122383 | -1.8332 | 0.827975 |
| ISCU | 0.352121 | 0.122419 | 1.564213 | 1.586597 |
| RASGRP1 | 0.352181 | 0.122466 | -1.91176 | 0.631028 |
| DCP1B | 0.35228 | 0.122528 | 0.957459 | 3.717167 |
| MIR4441 | 0.352682 | 0.122694 | 1.240762 | 2.644985 |
| URM1 | 0.352698 | 0.122727 | 1.123665 | 2.818887 |
| AEN | 0.352791 | 0.122786 | 0.740662 | 4.736225 |
| IQCJ-SCHIP | 0.352975 | 0.122877 | 1.721622 | 1.451076 |
| RUNX1-IT1 | 0.353214 | 0.122987 | 1.761602 | 3.190685 |
| LDLRAD4 | 0.353286 | 0.123039 | 1.889678 | 1.246347 |
| CRAMP1 | 0.353388 | 0.123101 | 0.612188 | 6.765455 |
| FAM126B | 0.353493 | 0.123165 | -0.61218 | 7.000855 |
| LINC00963 | 0.353867 | 0.123361 | 0.710228 | 3.997361 |
| LDB2 | 0.353867 | 0.123366 | 1.867828 | 1.375308 |
| LINC00622 | 0.353867 | 0.123376 | 2.655115 | 0.323742 |
| CMTR1 | 0.354129 | 0.123512 | 0.644901 | 6.482955 |
| HELZ | 0.354129 | 0.123521 | 0.533373 | 7.866329 |
| SCN1B | 0.3542 | 0.123573 | 1.994991 | 0.821604 |
| HECW2 | 0.35451 | 0.123708 | -1.26806 | 1.969371 |
| VEGFA | 0.354743 | 0.123817 | -1.47536 | 2.762873 |
| NDUFS6 | 0.354774 | 0.123856 | -0.60579 | 5.512488 |
| MAP2K2 | 0.354774 | 0.123885 | -0.55499 | 6.667213 |
| S100G | 0.354774 | 0.123909 | 2.139034 | 0.701196 |
| DBT | 0.354975 | 0.124026 | -0.58789 | 6.02899 |
| NUTM2F | 0.354975 | 0.124049 | -1.59051 | 1.492975 |
| LZTS2 | 0.354975 | 0.12406 | 0.671405 | 5.650421 |
| CHST7 | 0.355018 | 0.124102 | 1.30465 | 2.45698 |
| EMC2 | 0.355184 | 0.124187 | -0.59396 | 6.192891 |
| CACNA1C-/ | 0.355331 | 0.124265 | 1.779018 | 2.021604 |

| | | | | |
|---|---|---|---|---|
| IRF9 | 0.355337 | 0.124295 | 0.808629 | 5.680958 |
| TRIM8 | 0.355476 | 0.124371 | 0.592599 | 6.882259 |
| ICAM3 | 0.355799 | 0.124511 | 0.849576 | 3.437542 |
| ATIC | 0.355828 | 0.124565 | 0.651823 | 5.68023 |
| OR7E14P | 0.355828 | 0.124575 | -1.29428 | 2.493978 |
| VPS53 | 0.355882 | 0.124621 | -0.59056 | 6.282602 |
| BTN2A3P | 0.356197 | 0.124758 | 1.219915 | 2.858526 |
| CETN2 | 0.356215 | 0.1248 | -0.75903 | 4.786989 |
| ARPC5L | 0.356215 | 0.124827 | -0.6345 | 6.120111 |
| CD163 | 0.356215 | 0.124851 | 3.523749 | 1.380358 |
| FCER1A | 0.356215 | 0.124874 | -1.59772 | 1.792808 |
| METTL25 | 0.356524 | 0.125009 | -1.22039 | 3.087346 |
| LOC653712 | 0.356534 | 0.125039 | -1.50866 | 2.612929 |
| TTC14 | 0.356659 | 0.12511 | 0.657386 | 4.693032 |
| HLA-L | 0.356733 | 0.125164 | 0.804926 | 3.98064 |
| KLHL31 | 0.357201 | 0.125355 | 1.139993 | 2.248383 |
| CSTF2T | 0.357216 | 0.125388 | 0.632646 | 5.731139 |
| RASA4B | 0.35789 | 0.125651 | 1.377192 | 2.208633 |
| SEMA6B | 0.358095 | 0.125751 | 1.245037 | 2.960981 |
| ITPKB-IT1 | 0.358133 | 0.125791 | 1.526895 | 1.999557 |
| B4GALT4-A | 0.358225 | 0.125856 | 1.929995 | 0.628681 |
| C1RL-AS1 | 0.358225 | 0.125878 | 0.626181 | 6.032247 |
| C9orf16 | 0.358256 | 0.125917 | -0.7918 | 4.425066 |
| LOC100506 | 0.358856 | 0.126155 | 1.544589 | 2.105362 |
| SCART1 | 0.359296 | 0.126337 | -0.88517 | 4.856257 |
| ZBTB11 | 0.359374 | 0.126392 | -0.5613 | 6.094657 |
| PI4K2B | 0.359398 | 0.126427 | -0.65705 | 5.807516 |
| SLC44A3-A | 0.359698 | 0.12656 | 1.472481 | 2.340033 |
| HMGB1 | 0.359884 | 0.126653 | 0.693271 | 4.561653 |
| MAMDC4 | 0.36019 | 0.126796 | 0.720295 | 4.544533 |
| TPT1-AS1 | 0.36019 | 0.126816 | 0.893479 | 3.652894 |
| DHX32 | 0.360248 | 0.126872 | -0.59615 | 6.08406 |
| FOXC1 | 0.360248 | 0.126891 | -0.8567 | 5.463026 |
| TXNDC8 | 0.360369 | 0.126961 | -3.18109 | 0.757585 |
| ZNF252P-A | 0.360479 | 0.127033 | 1.442125 | 2.489313 |
| ASAP1-IT2 | 0.360479 | 0.127059 | 1.31779 | 2.209065 |
| OXR1 | 0.360479 | 0.127082 | -0.98958 | 2.419022 |
| TTC17 | 0.360496 | 0.127134 | 0.649398 | 5.624333 |
| PTGS2 | 0.360496 | 0.127174 | 1.237908 | 4.774362 |
| LOC100506 | 0.360496 | 0.127196 | -1.06511 | 3.195847 |
| CST2 | 0.360496 | 0.127198 | 2.735285 | 1.192692 |
| KYNU | 0.360543 | 0.12726 | -1.72856 | 2.087811 |
| LOC93429 | 0.360543 | 0.12727 | -1.94088 | 1.353207 |
| TTC39B | 0.360753 | 0.127371 | 2.062912 | 1.980594 |
| TES | 0.360758 | 0.127408 | 0.746561 | 4.0347 |

| | | | | |
|---|---|---|---|---|
| BAZ1B | 0.360758 | 0.127456 | 0.577588 | 8.381475 |
| S100A13 | 0.360758 | 0.127456 | -1.11339 | 2.579469 |
| CBFA2T2 | 0.361098 | 0.127619 | 1.317405 | 1.591604 |
| SNORA8 | 0.361098 | 0.127636 | 0.716948 | 6.519979 |
| CCDC127 | 0.361098 | 0.127672 | 0.881646 | 3.637645 |
| TMEM232 | 0.361098 | 0.127686 | 1.690568 | 1.258475 |
| P2RX1 | 0.361701 | 0.127927 | 1.494821 | 2.335264 |
| LRRC75A-A | 0.361878 | 0.128017 | 2.121798 | 0.736013 |
| LINC01003 | 0.362044 | 0.128103 | -1.25008 | 1.863343 |
| UGT1A4 | 0.362322 | 0.128229 | -1.98282 | 1.551044 |
| CARMN | 0.362332 | 0.12826 | 2.63202 | 1.891089 |
| FBH1 | 0.362461 | 0.128339 | 0.887049 | 3.818398 |
| TPRXL | 0.362461 | 0.128361 | -1.37323 | 3.845494 |
| IL4R | 0.362763 | 0.128499 | -0.62437 | 4.888887 |
| MID1IP1-A | 0.362763 | 0.128523 | 1.612213 | 1.997799 |
| LINC00896 | 0.362847 | 0.128581 | 1.543342 | 1.646419 |
| ELMO1 | 0.36298 | 0.12866 | 1.443674 | 1.850433 |
| RPRD1A | 0.36298 | 0.128686 | 1.37865 | 2.178986 |
| LOC101928 | 0.36298 | 0.128711 | 2.397955 | 0.4017 |
| EMD | 0.363982 | 0.129094 | -0.65394 | 4.897298 |
| ACOT2 | 0.364072 | 0.129186 | 1.917475 | 1.10227 |
| ZNF787 | 0.364072 | 0.129204 | 0.652305 | 4.623285 |
| PDK3 | 0.364072 | 0.129209 | 0.698511 | 6.251779 |
| CENPI | 0.364115 | 0.129256 | 0.982271 | 3.062034 |
| LRP5L | 0.364115 | 0.129295 | 1.247825 | 3.041993 |
| KCNA5 | 0.364115 | 0.129335 | 2.423843 | 1.369807 |
| HHEX | 0.364115 | 0.129359 | 1.988005 | 0.741042 |
| PRDM4 | 0.364115 | 0.129363 | -0.65564 | 6.140826 |
| CMAHP | 0.364343 | 0.129472 | 0.81631 | 4.03201 |
| CGGBP1 | 0.36448 | 0.129577 | -2.2412 | 1.811611 |
| SNORD99 | 0.36448 | 0.129638 | 0.885188 | 5.797821 |
| TMSB4Y | 0.36448 | 0.129651 | -1.94632 | 1.972288 |
| MYLK-AS2 | 0.36448 | 0.129659 | 2.24096 | 1.214754 |
| VASH1-AS1 | 0.36448 | 0.129676 | 2.099456 | 0.348163 |
| ARHGAP15 | 0.36448 | 0.129721 | 1.080524 | 3.196821 |
| GUF1 | 0.36448 | 0.12974 | -0.56153 | 6.063152 |
| DNM1P35 | 0.36448 | 0.129756 | 1.248961 | 2.032215 |
| DUSP6 | 0.36448 | 0.129795 | 1.271534 | 2.717621 |
| RNFT1-DT | 0.36448 | 0.129817 | 1.618564 | 0.853118 |
| MAX | 0.36448 | 0.129826 | -1.78291 | 1.0873 |
| MLPH | 0.364607 | 0.129934 | 2.656567 | 1.04813 |
| B4GALT5 | 0.364607 | 0.129952 | -0.64104 | 7.363593 |
| PCGF6 | 0.364607 | 0.129955 | 2.26651 | 0.633469 |
| SORCS3 | 0.364633 | 0.130004 | 2.059287 | 0.70075 |
| CD40LG | 0.364633 | 0.13002 | 2.067589 | 1.964579 |

| | | | | |
|---|---|---|---|---|
| EBF4 | 0.365074 | 0.130205 | 1.331278 | 2.563058 |
| RARRES3 | 0.365366 | 0.130346 | 1.421304 | 3.163431 |
| SAP30 | 0.365366 | 0.130365 | 0.695596 | 4.396 |
| HPS3 | 0.365423 | 0.130439 | 1.098181 | 3.469413 |
| MDGA1 | 0.365423 | 0.130443 | -0.91202 | 4.621594 |
| IRF2 | 0.365423 | 0.130525 | -0.54888 | 6.149944 |
| TFF2 | 0.365423 | 0.130532 | 2.40766 | 6.453654 |
| SRBD1 | 0.365423 | 0.130566 | 0.630298 | 5.74869 |
| POMGNT1 | 0.365423 | 0.130568 | 2.165786 | 1.059942 |
| DQX1 | 0.365423 | 0.13058 | 0.889562 | 3.953667 |
| TADA2B | 0.365789 | 0.130739 | -0.56478 | 5.511782 |
| TAF1B | 0.365804 | 0.130772 | -0.64885 | 4.589087 |
| DPYSL3 | 0.366049 | 0.130887 | 1.580083 | 2.672596 |
| DNAJC7 | 0.366146 | 0.13095 | -1.9005 | 0.702777 |
| NDUFA7 | 0.366257 | 0.131038 | -0.77536 | 4.072881 |
| CT83 | 0.366257 | 0.131045 | 2.64807 | 1.20348 |
| FAM135A | 0.366272 | 0.131078 | -0.9446 | 3.733147 |
| SLC35F2 | 0.366288 | 0.131112 | -0.62168 | 5.324866 |
| KLLN | 0.366418 | 0.131199 | 1.186806 | 2.233499 |
| GUCY2D | 0.366418 | 0.131215 | -1.53205 | 1.074812 |
| SERPINB9 | 0.366676 | 0.131335 | -0.93139 | 6.723993 |
| ARX | 0.366758 | 0.131392 | 2.691727 | 1.803537 |
| SLC9A5 | 0.366823 | 0.131443 | 0.912992 | 2.692473 |
| SCN9A | 0.366918 | 0.131506 | 1.254302 | 2.985737 |
| PDE6C | 0.367326 | 0.13168 | -1.89704 | 1.339413 |
| ANGPTL8 | 0.367767 | 0.131916 | -1.20416 | 2.034052 |
| B4GALT6 | 0.367767 | 0.131944 | 1.079729 | 3.500919 |
| CEP192 | 0.367767 | 0.131948 | 0.66778 | 7.351264 |
| CDC42EP3 | 0.367767 | 0.131951 | 1.966644 | 0.624022 |
| TMEM253 | 0.367767 | 0.131978 | -1.05056 | 1.994551 |
| AMBP | 0.367907 | 0.132077 | 1.928597 | 1.012417 |
| FLJ16779 | 0.367907 | 0.132101 | 1.863397 | 0.795822 |
| LPIN3 | 0.367907 | 0.132112 | -2.61513 | 0.680036 |
| LOC728024 | 0.36797 | 0.132168 | 1.17008 | 2.211638 |
| RARRES1 | 0.36797 | 0.132191 | 1.721967 | 2.021167 |
| ITCH | 0.368077 | 0.132257 | 2.226416 | 0.561639 |
| SLIT1 | 0.368153 | 0.132313 | 1.452094 | 1.358112 |
| SLC25A6 | 0.368677 | 0.132529 | -1.24827 | 6.364917 |
| SNX20 | 0.368679 | 0.132564 | 1.674136 | 1.594037 |
| RINT1 | 0.368679 | 0.132586 | -0.65432 | 5.26948 |
| LINC00950 | 0.368998 | 0.132752 | 1.514141 | 2.342445 |
| TNK2 | 0.368998 | 0.132759 | -0.77059 | 4.184507 |
| 11-Sep | 0.368998 | 0.132785 | 1.573756 | 2.25667 |
| PCDHB4 | 0.369004 | 0.132816 | 1.502871 | 2.599899 |
| ARHGAP31 | 0.369052 | 0.132861 | 0.703868 | 5.337841 |

| | | | | |
|---|---|---|---|---|
| HEXA | 0.369298 | 0.132978 | 0.638274 | 5.409019 |
| SMIM24 | 0.369302 | 0.133007 | 1.996488 | 4.528055 |
| MAATS1 | 0.369364 | 0.133058 | 1.505059 | 3.132716 |
| MOB3B | 0.369417 | 0.133105 | 0.805534 | 5.313639 |
| ZNF561 | 0.369458 | 0.133148 | -0.62904 | 5.908405 |
| COA1 | 0.369552 | 0.133252 | 0.653208 | 4.522168 |
| PSD3 | 0.369552 | 0.133255 | -0.66061 | 6.187568 |
| MDFIC | 0.369552 | 0.133266 | -2.5586 | 0.927483 |
| PSORS1C3 | 0.369819 | 0.133391 | 2.478669 | 1.420929 |
| FLCN | 0.369926 | 0.133458 | 0.665955 | 4.914236 |
| IPO9 | 0.370077 | 0.13354 | 0.547097 | 7.201509 |
| PSMD7 | 0.370358 | 0.13367 | -0.54575 | 7.251419 |
| C9orf163 | 0.371116 | 0.133972 | 2.203941 | 0.805289 |
| THRA | 0.37144 | 0.134117 | 1.050508 | 3.737033 |
| PKD1P6-NF | 0.371549 | 0.134185 | 0.71157 | 4.311978 |
| JARID2-AS1 | 0.371808 | 0.134306 | -1.18684 | 1.786152 |
| C2CD6 | 0.371916 | 0.134398 | -2.24835 | 0.955481 |
| ISG20L2 | 0.371916 | 0.134402 | 0.574277 | 5.565725 |
| COA7 | 0.372176 | 0.134626 | 0.784132 | 4.160244 |
| BTBD1 | 0.372176 | 0.134629 | -2.51008 | 0.708442 |
| FDXR | 0.372176 | 0.134636 | -1.1809 | 2.933185 |
| IDI2 | 0.372176 | 0.134641 | 1.394247 | 2.193124 |
| MRPL43 | 0.372176 | 0.134647 | -1.94127 | 1.211086 |
| ARID4A | 0.372176 | 0.134692 | -1.92595 | 1.030736 |
| KRT76 | 0.372176 | 0.134725 | -2.29865 | 0.537801 |
| SLC6A6 | 0.372176 | 0.134726 | 1.915292 | 0.567965 |
| LINC00938 | 0.372176 | 0.134774 | -0.62801 | 4.498519 |
| RBM43 | 0.372176 | 0.13478 | 0.931738 | 4.003869 |
| SLC26A2 | 0.372363 | 0.134899 | -0.7453 | 5.75228 |
| BMP6 | 0.372363 | 0.134904 | 1.245361 | 2.105468 |
| GNAL | 0.372607 | 0.135034 | -1.67086 | 1.738509 |
| ZNF671 | 0.372607 | 0.13505 | 0.888367 | 3.477821 |
| TSPYL5 | 0.372802 | 0.135156 | 1.343124 | 3.306601 |
| LOC200772 | 0.372802 | 0.135177 | 2.509071 | 1.75441 |
| TSR2 | 0.372973 | 0.135273 | -0.56861 | 5.751361 |
| IQGAP2 | 0.372973 | 0.135296 | 1.06968 | 3.503022 |
| RACGAP1 | 0.372988 | 0.135329 | -1.59614 | 1.404942 |
| LINC01232 | 0.373112 | 0.135403 | 1.268264 | 1.708247 |
| ETFB | 0.373151 | 0.135489 | -1.80593 | 1.446802 |
| TMEM65 | 0.373151 | 0.135509 | -0.62821 | 5.930601 |
| SMAD5-AS | 0.373151 | 0.135556 | -1.23973 | 1.618337 |
| PGLYRP1 | 0.373151 | 0.135562 | 1.837514 | 0.792773 |
| MIR7854 | 0.373151 | 0.135565 | -1.92421 | 1.289021 |
| TPMT | 0.373151 | 0.135589 | 0.707986 | 5.261537 |
| LOC34009( | 0.373151 | 0.135616 | 2.456846 | 0.858066 |

| | | | | |
|---|---|---|---|---|
| TFAP2A | 0.373525 | 0.135781 | -1.16551 | 2.927527 |
| ZNF69 | 0.373542 | 0.135816 | 1.919293 | 2.753732 |
| LRIG1 | 0.373542 | 0.135868 | 0.924062 | 6.612267 |
| POR | 0.373542 | 0.135872 | -0.57376 | 6.130248 |
| RPL18A | 0.374131 | 0.136124 | -0.6525 | 7.660503 |
| ZNF865 | 0.374131 | 0.136144 | 0.659051 | 4.604777 |
| TEDC2 | 0.374223 | 0.13625 | 1.549226 | 1.368082 |
| MIR497HG | 0.374223 | 0.136254 | 1.860435 | 0.615992 |
| MIR320C1 | 0.374223 | 0.13627 | -2.43296 | 0.978326 |
| POLK | 0.374223 | 0.136291 | 0.596798 | 5.921538 |
| WDPCP | 0.374983 | 0.136597 | 1.281981 | 2.295034 |
| NUP214 | 0.375181 | 0.136713 | -0.5653 | 7.581412 |
| ZNF680 | 0.375181 | 0.136736 | 0.684803 | 4.630573 |
| OLFML2A | 0.375181 | 0.136754 | -1.69482 | 2.260136 |
| UGT8 | 0.375354 | 0.136846 | 1.73956 | 1.297061 |
| DCLK3 | 0.37565 | 0.136993 | 1.902806 | 0.51715 |
| CYC1 | 0.37565 | 0.137011 | -0.65714 | 5.917056 |
| SNORD113 | 0.375694 | 0.137056 | 2.887082 | 0.714041 |
| MRPS15 | 0.375703 | 0.137088 | -0.6323 | 5.569606 |
| CHN2 | 0.375836 | 0.137165 | 1.703989 | 1.739344 |
| CAVIN2 | 0.375972 | 0.137246 | 0.809604 | 4.981242 |
| PUM2 | 0.375972 | 0.137272 | 1.206295 | 1.725325 |
| HEATR5B | 0.376248 | 0.137401 | -0.51777 | 6.868962 |
| PDPR | 0.376305 | 0.137451 | 0.811304 | 6.412922 |
| FANK1 | 0.376429 | 0.137548 | -0.85982 | 3.548877 |
| SH3PXD2B | 0.376429 | 0.137553 | 0.691044 | 6.776832 |
| ZNF136 | 0.376433 | 0.137584 | 0.827347 | 3.996147 |
| SMPD3 | 0.376442 | 0.137616 | 0.887135 | 4.650599 |
| OSER1 | 0.376505 | 0.137686 | -0.54842 | 6.027886 |
| CLIC2 | 0.376505 | 0.137696 | 1.291733 | 3.056039 |
| FAM66B | 0.376711 | 0.1378 | 1.716504 | 0.67357 |
| B3GALT2 | 0.376836 | 0.137875 | -0.75767 | 4.148596 |
| GOLGA8J | 0.376906 | 0.137929 | 2.146189 | 0.52506 |
| PAXIP1 | 0.376982 | 0.137998 | 0.622185 | 5.468518 |
| TMEM266 | 0.376982 | 0.138014 | 1.360433 | 1.37385 |
| DDX18 | 0.377248 | 0.13814 | 0.541744 | 6.862725 |
| KRT31 | 0.377271 | 0.138178 | -2.1375 | 1.558315 |
| FREM2 | 0.377277 | 0.138232 | 1.485875 | 3.754033 |
| SMARCA4 | 0.377277 | 0.138237 | -0.87091 | 3.190439 |
| SZT2 | 0.377368 | 0.138321 | 0.530211 | 7.668734 |
| MIR101-1 | 0.377368 | 0.138328 | 2.610075 | 1.000832 |
| KYAT1 | 0.377413 | 0.138392 | -2.14408 | 0.64288 |
| MLLT10 | 0.377413 | 0.13843 | -1.41117 | 1.859591 |
| SUSD6 | 0.377413 | 0.138431 | -0.55749 | 7.228808 |
| KCTD6 | 0.377559 | 0.138529 | 2.013625 | 0.765914 |

| | | | | |
|---|---|---|---|---|
| C1orf21 | 0.377559 | 0.138542 | -0.64324 | 6.847294 |
| CDKN2A | 0.377616 | 0.138592 | -1.43211 | 3.068008 |
| TMBIM1 | 0.37767 | 0.13864 | -0.68874 | 8.567415 |
| ADAMTS17 | 0.377908 | 0.138757 | -0.98511 | 4.061126 |
| RIN2 | 0.37808 | 0.138848 | -1.84798 | 0.479431 |
| ACTR5 | 0.378354 | 0.138978 | 0.941662 | 3.88141 |
| TAS1R3 | 0.378364 | 0.13901 | -0.99053 | 2.821022 |
| RNF169 | 0.378398 | 0.139052 | -0.55968 | 7.602958 |
| ZNF429 | 0.378405 | 0.139106 | -0.96115 | 4.268858 |
| GSDMA | 0.378405 | 0.139112 | -1.50466 | 1.242571 |
| TTC39C-AS | 0.378417 | 0.139145 | 1.780585 | 1.163721 |
| SMDT1 | 0.378451 | 0.139202 | -1.35271 | 3.1017 |
| ITIH2 | 0.378451 | 0.139223 | 1.628672 | 1.09671 |
| PTPN12 | 0.378451 | 0.139277 | 1.616485 | 0.866004 |
| RNF157-AS | 0.378451 | 0.139279 | 2.36847 | 0.526651 |
| CD74 | 0.378451 | 0.139302 | 1.129607 | 2.473486 |
| NF1 | 0.378678 | 0.139414 | 1.401248 | 2.222681 |
| CNOT6 | 0.378937 | 0.139539 | -0.53677 | 6.627987 |
| GNAZ | 0.379016 | 0.139604 | 1.580636 | 3.228204 |
| SLC22A13 | 0.379016 | 0.139671 | -1.51458 | 1.50466 |
| TSPAN33 | 0.379016 | 0.139677 | 1.057517 | 2.968794 |
| CXCL8 | 0.379016 | 0.139683 | 2.330573 | 4.999395 |
| SLC2A9 | 0.37937 | 0.139842 | -2.15463 | 0.542207 |
| S100A7 | 0.379581 | 0.139949 | -2.65331 | 7.779052 |
| RNU5D-1 | 0.379684 | 0.140016 | -1.01999 | 5.064977 |
| GOLGA8T | 0.379731 | 0.140062 | 1.251051 | 2.787601 |
| KCND3-AS1 | 0.380075 | 0.140218 | 1.824917 | 1.392971 |
| MYCBPAP | 0.38021 | 0.140297 | 1.352641 | 1.535475 |
| SLC30A9 | 0.38026 | 0.140372 | -0.56492 | 7.153211 |
| NFKBID | 0.38026 | 0.140374 | 0.758559 | 3.844294 |
| LOC652276 | 0.380711 | 0.14059 | 0.672229 | 5.228509 |
| M6PR | 0.380711 | 0.140598 | 1.134903 | 2.425008 |
| MICAL3 | 0.380766 | 0.140697 | 0.533091 | 6.239754 |
| ZNF571-AS | 0.380766 | 0.140701 | 1.945197 | 1.945204 |
| NKAIN3 | 0.380766 | 0.140705 | 2.459183 | 0.389051 |
| PAGR1 | 0.380802 | 0.140751 | 0.582455 | 6.04831 |
| MAP2 | 0.380802 | 0.140776 | 1.153262 | 4.588321 |
| RPLP0P2 | 0.380883 | 0.140836 | 1.874014 | 1.626745 |
| PRICKLE4 | 0.380931 | 0.140882 | -0.81346 | 4.444191 |
| YBX1 | 0.38142 | 0.141092 | 0.659755 | 5.415362 |
| DEK | 0.381516 | 0.141157 | 1.490577 | 1.198304 |
| LGI4 | 0.381545 | 0.141197 | 1.0071 | 3.099042 |
| RAB14 | 0.381666 | 0.141298 | -0.6127 | 7.576587 |
| NBPF1 | 0.381666 | 0.141301 | 0.626393 | 4.519684 |
| AKR1B1 | 0.381666 | 0.141329 | 0.866741 | 4.138566 |

| | | | | |
|---|---|---|---|---|
| ATP4B | 0.381749 | 0.141405 | -3.24423 | 7.022432 |
| ENO2 | 0.381749 | 0.14146 | 0.726209 | 5.285753 |
| FAM86C1 | 0.381749 | 0.141463 | 2.38347 | 0.371937 |
| CDKL5 | 0.381749 | 0.141476 | 2.026073 | 0.517386 |
| MYC | 0.381974 | 0.141588 | -0.79118 | 7.017258 |
| FOXP1-AS1 | 0.38203 | 0.141638 | -1.00998 | 3.502882 |
| LUC7L | 0.382093 | 0.141714 | 1.481371 | 1.656327 |
| WDR54 | 0.382093 | 0.14172 | 1.084415 | 2.801083 |
| SMIM12 | 0.382207 | 0.141791 | 2.200244 | 0.747312 |
| SIK1 | 0.38238 | 0.14189 | -1.16108 | 6.042458 |
| IMPDH2 | 0.38238 | 0.141942 | -0.65258 | 7.054444 |
| CLIP3 | 0.38238 | 0.141943 | 1.901961 | 0.659179 |
| CORO6 | 0.382391 | 0.141976 | 1.437687 | 2.05436 |
| SNORA94 | 0.382791 | 0.142154 | -1.2277 | 2.378215 |
| MIR744 | 0.383068 | 0.142286 | 2.800907 | 0.911661 |
| PALLD | 0.383562 | 0.142498 | 0.80832 | 4.265775 |
| RHOBTB2 | 0.383718 | 0.142586 | 2.038252 | 0.923247 |
| LINC02197 | 0.383851 | 0.142664 | 3.030892 | 0.848512 |
| MYCL | 0.383858 | 0.142696 | -1.93218 | 1.625988 |
| NDUFA6 | 0.383889 | 0.142737 | -1.12376 | 5.161471 |
| CEP295 | 0.383931 | 0.14281 | 0.573966 | 6.778632 |
| SPRY2 | 0.383931 | 0.142829 | 0.816614 | 4.378839 |
| LOC10013( | 0.383931 | 0.142859 | 2.172677 | 0.827805 |
| UQCRC1 | 0.383931 | 0.14287 | -0.54995 | 6.421061 |
| PLVAP | 0.383931 | 0.142935 | 0.74258 | 5.517689 |
| STARD7 | 0.383931 | 0.142943 | 0.510537 | 7.23756 |
| EFHC1 | 0.383931 | 0.142957 | 0.911826 | 2.899351 |
| ERP44 | 0.384029 | 0.143023 | -0.52656 | 7.026907 |
| HNF1A-AS1 | 0.384379 | 0.143211 | 1.990072 | 3.963751 |
| OSGIN2 | 0.384379 | 0.143212 | 2.141415 | 0.582597 |
| RENBP | 0.384447 | 0.14329 | 1.11106 | 2.217434 |
| CTSE | 0.384447 | 0.143296 | 2.907464 | 0.466744 |
| ZNF404 | 0.384589 | 0.143396 | 1.541449 | 1.557882 |
| BATF3 | 0.384589 | 0.143473 | 1.594359 | 2.072421 |
| MTMR6 | 0.384589 | 0.143481 | 0.520654 | 5.417961 |
| LINC02097 | 0.384589 | 0.143491 | 2.084348 | 1.341459 |
| LINC01436 | 0.384589 | 0.143504 | 1.868967 | 1.030169 |
| SMARCD2 | 0.384589 | 0.143525 | -0.5375 | 6.81595 |
| ERCC4 | 0.384724 | 0.143605 | 0.71369 | 4.819695 |
| CHPF | 0.384844 | 0.143705 | 0.851109 | 3.083246 |
| DICER1 | 0.384844 | 0.143736 | -0.85551 | 3.055717 |
| GLB1L2 | 0.384844 | 0.143737 | 1.221315 | 3.990972 |
| ZNF763 | 0.385008 | 0.143845 | 1.089992 | 2.564649 |
| SCARNA28 | 0.385008 | 0.143857 | 0.746398 | 5.619955 |
| ADAMTS4 | 0.385052 | 0.143903 | 1.190842 | 5.797446 |

| | | | | |
|---|---|---|---|---|
| LOC38919 | 0.385241 | 0.144003 | -2.56296 | 1.502848 |
| FGR | 0.385404 | 0.144118 | 1.775961 | 1.476785 |
| MIR4513 | 0.385404 | 0.144123 | -1.71883 | 1.06049 |
| RP2 | 0.385528 | 0.144198 | -0.66654 | 5.402374 |
| SNORD111 | 0.385535 | 0.144414 | 0.994535 | 5.223646 |
| DIRC3 | 0.385535 | 0.144418 | 1.617439 | 1.762108 |
| PNRC2 | 0.385535 | 0.144439 | 0.526382 | 7.364996 |
| DPT | 0.385535 | 0.144442 | -1.25262 | 3.086984 |
| IPW | 0.385535 | 0.144446 | 1.030226 | 3.16208 |
| RPL39L | 0.385535 | 0.14448 | 1.39346 | 1.696175 |
| ITGB4 | 0.385535 | 0.144489 | 1.45264 | 1.280568 |
| VTN | 0.385535 | 0.144496 | 1.399381 | 1.733509 |
| ESAM | 0.385535 | 0.144511 | -0.72455 | 3.971237 |
| PRR14L | 0.385535 | 0.144523 | -0.52936 | 8.040861 |
| ARHGEF34 | 0.385535 | 0.14454 | -0.69279 | 7.886558 |
| RNA28SN5 | 0.385535 | 0.144554 | -0.61707 | 16.06966 |
| LOC100287 | 0.385548 | 0.144591 | 1.552342 | 0.956419 |
| PANX1 | 0.385548 | 0.144617 | 0.778754 | 4.934111 |
| HLX | 0.3859 | 0.144779 | 1.227063 | 1.726198 |
| DPY19L1P2 | 0.385914 | 0.144813 | 1.685 | 1.719204 |
| MMAA | 0.386029 | 0.144886 | 0.68818 | 4.238997 |
| SNAPC5 | 0.386214 | 0.144985 | 0.813912 | 3.455081 |
| XYLT1 | 0.386457 | 0.145126 | -0.52741 | 6.593916 |
| MYO5C | 0.386457 | 0.145135 | 0.733175 | 7.413889 |
| DOCK7 | 0.386479 | 0.145172 | -1.71615 | 0.816726 |
| RFLNB | 0.386909 | 0.145364 | 0.752114 | 3.956732 |
| FTO-IT1 | 0.387112 | 0.145479 | 1.230832 | 1.766632 |
| TBC1D12 | 0.387112 | 0.145499 | 0.525879 | 5.691803 |
| WAS | 0.38712 | 0.145531 | 0.963049 | 2.90533 |
| CFAP20 | 0.387362 | 0.145652 | -0.748 | 3.870261 |
| PER2 | 0.387771 | 0.145851 | -0.68366 | 7.363194 |
| CCNB3 | 0.387771 | 0.145889 | 2.092278 | 0.401294 |
| LOC100505 | 0.387771 | 0.145894 | 1.074961 | 2.725483 |
| AKAP12 | 0.387897 | 0.145991 | 1.41496 | 2.59046 |
| JOSD1 | 0.387897 | 0.146001 | 0.586426 | 6.436543 |
| TK1 | 0.38801 | 0.146073 | 0.641695 | 4.091478 |
| ZNF467 | 0.38802 | 0.146106 | -1.2488 | 2.769803 |
| SNHG18 | 0.38824 | 0.146219 | -1.11945 | 3.850023 |
| LOC101928 | 0.388284 | 0.146265 | -1.50283 | 1.201109 |
| PNKP | 0.388507 | 0.146466 | 0.578945 | 5.175013 |
| COL7A1 | 0.388507 | 0.146485 | -0.77135 | 8.206475 |
| NHLRC2 | 0.388507 | 0.146485 | 0.535489 | 6.68189 |
| TBX5-AS1 | 0.388507 | 0.146488 | 1.915014 | 1.732172 |
| SNRNP48 | 0.388507 | 0.146497 | 0.5958 | 5.446862 |
| NFYB | 0.388704 | 0.146601 | 0.60489 | 5.26118 |

| | | | | |
|---|---|---|---|---|
| KLHL2 | 0.388819 | 0.146728 | -1.43567 | 0.994655 |
| EID1 | 0.388819 | 0.146753 | 0.589232 | 7.015553 |
| ESYT3 | 0.388819 | 0.146761 | 1.196209 | 3.385834 |
| TMEM54 | 0.388819 | 0.146763 | -0.67609 | 4.95714 |
| CYP3A5 | 0.389007 | 0.146863 | 0.829323 | 6.552468 |
| FITM1 | 0.389633 | 0.147129 | -2.59011 | 0.765597 |
| PSMB10 | 0.389708 | 0.147187 | 0.787284 | 4.124895 |
| LINC00475 | 0.389973 | 0.147319 | 1.827788 | 1.144516 |
| TPSG1 | 0.389973 | 0.147347 | 2.219081 | 1.067955 |
| CHMP5 | 0.390234 | 0.147475 | 2.005437 | 1.394416 |
| ZC3H12C | 0.390292 | 0.147527 | 0.730719 | 4.961586 |
| GRAMD2A | 0.390325 | 0.147585 | 1.097173 | 2.13802 |
| ATG2B | 0.390325 | 0.147599 | -0.52489 | 7.151957 |
| TBX21 | 0.390391 | 0.147654 | 1.846976 | 0.4631 |
| ZNRF2P1 | 0.390526 | 0.147739 | 0.898803 | 3.434004 |
| SNORA57 | 0.390526 | 0.147764 | 0.663708 | 5.793393 |
| LOC28493( | 0.390774 | 0.147888 | 2.691971 | 1.820828 |
| PDCD7 | 0.391353 | 0.148163 | -0.58454 | 5.487589 |
| LRPAP1 | 0.391353 | 0.148195 | -0.87489 | 4.134783 |
| NDUFS4 | 0.391353 | 0.148219 | -0.61986 | 5.807725 |
| ASIC1 | 0.391353 | 0.148226 | 2.02673 | 0.426025 |
| CCDC192 | 0.391364 | 0.14826 | 1.765883 | 0.917198 |
| SDR39U1 | 0.391441 | 0.148319 | 1.65778 | 1.489631 |
| MSC | 0.391574 | 0.148399 | 1.825408 | 2.491333 |
| LIMA1 | 0.391623 | 0.148459 | 1.013284 | 2.516231 |
| BARX1 | 0.391623 | 0.148478 | 2.169294 | 3.480493 |
| FAM122C | 0.391874 | 0.148603 | 1.414362 | 2.032328 |
| OAT | 0.391885 | 0.148637 | -1.0331 | 3.19565 |
| DYRK1A | 0.39197 | 0.148719 | -0.89154 | 4.145599 |
| ZMYM4 | 0.39197 | 0.148739 | 0.535292 | 7.497446 |
| CARM1 | 0.39197 | 0.148759 | -0.54283 | 6.043696 |
| LOC101928 | 0.392051 | 0.148819 | -0.71537 | 3.599958 |
| KCNMB2-A | 0.392294 | 0.148941 | 2.74734 | 0.774714 |
| MRNIP | 0.392533 | 0.149062 | 1.33498 | 1.223495 |
| SMARCA5 | 0.392852 | 0.149213 | 0.529334 | 7.719371 |
| CFLAR-AS1 | 0.393032 | 0.149336 | 0.692398 | 4.226331 |
| DCBLD1 | 0.393032 | 0.149376 | 0.654822 | 4.904072 |
| PRKY | 0.393032 | 0.149427 | -1.24724 | 5.39965 |
| DLG3-AS1 | 0.393032 | 0.149429 | 1.594962 | 0.871856 |
| SBSPON | 0.393032 | 0.149431 | 1.322898 | 4.471692 |
| DCUN1D5 | 0.393119 | 0.149494 | 0.59999 | 6.238158 |
| KLHL18 | 0.393519 | 0.149676 | -0.59274 | 5.150671 |
| ZSCAN20 | 0.393543 | 0.149716 | 1.08219 | 3.914439 |
| POGK | 0.393551 | 0.149749 | 1.54421 | 1.609884 |
| SNORA12 | 0.393694 | 0.149888 | 0.641625 | 6.398616 |

| | | | | |
|---|---|---|---|---|
| PLGRKT | 0.393694 | 0.149891 | -0.75037 | 3.77009 |
| RNF216-IT1 | 0.393694 | 0.149893 | 1.182317 | 2.069108 |
| SHROOM3 | 0.394036 | 0.150075 | 0.607416 | 8.26346 |
| TTC38 | 0.394036 | 0.150083 | 0.814915 | 4.433179 |
| LINC00346 | 0.394184 | 0.15017 | 1.162209 | 3.060971 |
| GLIS3-AS1 | 0.394545 | 0.150366 | 2.685394 | 0.579416 |
| RTKN | 0.394545 | 0.150367 | 0.955629 | 2.832254 |
| POU5F1B | 0.394651 | 0.150438 | 1.893927 | 0.913751 |
| C4orf46 | 0.395121 | 0.150647 | -2.09694 | 0.791344 |
| PLEKHH3 | 0.395164 | 0.150693 | -1.54615 | 1.24761 |
| FAM241B | 0.396017 | 0.151049 | 1.461827 | 1.905477 |
| AGO2 | 0.396061 | 0.151096 | 1.436864 | 0.812333 |
| LINC00562 | 0.396072 | 0.15113 | 2.066781 | 0.681043 |
| EPB41L3 | 0.396168 | 0.151226 | 2.036052 | 0.678653 |
| DTX2P1-UF | 0.396168 | 0.151227 | -0.58771 | 6.041904 |
| TMEM220 | 0.396245 | 0.151289 | 0.764836 | 4.228702 |
| DLGAP4-AS | 0.396245 | 0.151345 | 0.955321 | 2.896033 |
| TENM4 | 0.396245 | 0.151347 | -0.68574 | 5.337529 |
| RRP1 | 0.396461 | 0.15146 | -0.64058 | 5.720748 |
| C2orf40 | 0.396551 | 0.151544 | 2.180313 | 1.875326 |
| TPTE2P1 | 0.396551 | 0.151555 | 2.129453 | 1.115349 |
| HPS5 | 0.397075 | 0.151785 | -2.52274 | 1.10821 |
| RPS28 | 0.397159 | 0.151848 | -0.88461 | 5.502118 |
| SNHG3 | 0.397321 | 0.15194 | -1.28978 | 2.414208 |
| HLA-DQB1- | 0.397491 | 0.152058 | 1.331839 | 2.223084 |
| EEF1A1 | 0.397491 | 0.152066 | -0.56424 | 10.98805 |
| TRDMT1 | 0.397631 | 0.152165 | 0.734871 | 3.922365 |
| ADAMTS7 | 0.397631 | 0.15218 | 1.015306 | 3.065194 |
| CAMK4 | 0.397657 | 0.15222 | 1.130627 | 3.10967 |
| MICU3 | 0.397742 | 0.152283 | 1.410811 | 3.05755 |
| PSMB2 | 0.397934 | 0.152387 | 1.768054 | 0.815739 |
| PBRM1 | 0.398017 | 0.152482 | -0.53804 | 8.084235 |
| PDIA2 | 0.398017 | 0.152484 | -2.01985 | 4.39247 |
| CMTM8 | 0.398017 | 0.15251 | 1.383453 | 2.679813 |
| CYCS | 0.398127 | 0.152582 | -0.59412 | 7.648754 |
| DAPK3 | 0.398465 | 0.152742 | 0.602121 | 5.973629 |
| AMIGO1 | 0.398992 | 0.152974 | 1.276108 | 2.356493 |
| LIMS1 | 0.399169 | 0.153073 | 0.606857 | 4.469076 |
| ZNF57 | 0.399641 | 0.153284 | -0.70267 | 4.050645 |
| DELE1 | 0.399774 | 0.153365 | 0.927963 | 3.386143 |
| TRAF3IP2 | 0.3998 | 0.153406 | -1.32721 | 1.623327 |
| LINC00858 | 0.400085 | 0.153559 | 1.87149 | 1.121982 |
| JAK1 | 0.400085 | 0.153581 | 0.51235 | 7.975849 |
| UBE2E1 | 0.400085 | 0.153642 | -1.60867 | 1.061001 |
| NOC2L | 0.400085 | 0.153678 | -0.52441 | 6.291584 |

| | | | | |
|---|---|---|---|---|
| FAM177A1 | 0.400085 | 0.153748 | 2.000858 | 0.644108 |
| RSF1 | 0.400085 | 0.153749 | 0.53606 | 7.853789 |
| NEU3 | 0.400085 | 0.153754 | 0.768417 | 4.151323 |
| C11orf24 | 0.400085 | 0.153759 | 1.072028 | 3.471903 |
| MAD2L1 | 0.400185 | 0.153828 | 0.796557 | 4.325574 |
| LSG1 | 0.400325 | 0.153912 | 0.590856 | 6.15258 |
| ETNK1 | 0.400386 | 0.153989 | 0.522514 | 6.956597 |
| PDE4DIP | 0.400386 | 0.153997 | 0.798804 | 4.760888 |
| MROH6 | 0.401276 | 0.15437 | -1.52372 | 1.46279 |
| ICMT | 0.401759 | 0.154586 | -0.51254 | 6.363297 |
| PPIP5K2 | 0.401762 | 0.154618 | 1.665004 | 0.767393 |
| CEBPB-AS1 | 0.40183 | 0.154675 | 1.358162 | 1.808072 |
| ABCG2 | 0.401913 | 0.154758 | 2.624407 | 1.023068 |
| GIMAP8 | 0.401913 | 0.154768 | 0.82889 | 4.075662 |
| VAMP1 | 0.40193 | 0.154805 | 1.701368 | 1.096628 |
| INPP5A | 0.402012 | 0.154867 | 0.633176 | 4.991765 |
| ATP11A-AS | 0.402482 | 0.155098 | 1.022507 | 3.415942 |
| LOC10192 | 0.402482 | 0.15511 | 1.769266 | 0.677047 |
| AFAP1-AS1 | 0.402482 | 0.15514 | 0.940772 | 4.303652 |
| SPCS1 | 0.402655 | 0.155238 | -0.59189 | 5.636378 |
| ZSCAN21 | 0.402805 | 0.155326 | 1.243918 | 2.798291 |
| DHX34 | 0.403032 | 0.155445 | 0.60479 | 5.199487 |
| DAZAP2 | 0.403238 | 0.155555 | -1.22956 | 2.47662 |
| LIN7C | 0.403274 | 0.155614 | -0.53455 | 6.764861 |
| HOXD10 | 0.403274 | 0.15563 | 2.106965 | 1.473768 |
| BIVM | 0.403316 | 0.155677 | -1.71201 | 0.925116 |
| LOC10192 | 0.403577 | 0.155819 | 0.999712 | 2.609187 |
| FAAP20 | 0.403577 | 0.155861 | 1.050561 | 2.945573 |
| TEX41 | 0.403577 | 0.15587 | 2.091959 | 0.750799 |
| AGAP5 | 0.404341 | 0.156196 | -0.81908 | 3.179615 |
| GFI1 | 0.4049 | 0.156443 | 3.188067 | 0.235719 |
| LGALS3BP | 0.40516 | 0.156607 | 0.567066 | 7.500406 |
| SNORA38B | 0.40516 | 0.156611 | 1.045817 | 4.722732 |
| KPNA4 | 0.40516 | 0.156636 | -0.56631 | 8.098031 |
| C16orf87 | 0.405225 | 0.156693 | -0.76863 | 4.056776 |
| ST8SIA3 | 0.405225 | 0.156723 | 2.279651 | 1.365613 |
| CLHC1 | 0.405357 | 0.156835 | 1.132928 | 3.165471 |
| SCGB2B3P | 0.405357 | 0.156836 | 2.186756 | 0.384257 |
| TRPV1 | 0.405557 | 0.156944 | 1.52184 | 1.469549 |
| MID1 | 0.405567 | 0.156979 | 1.799572 | 1.808083 |
| LAMP1 | 0.405865 | 0.157143 | -0.58072 | 8.067539 |
| CYP4Z1 | 0.405865 | 0.157164 | -1.5626 | 1.634152 |
| PLA2G16 | 0.405865 | 0.157187 | 1.973425 | 0.486207 |
| LINC00654 | 0.406352 | 0.157406 | 1.528335 | 2.534612 |
| MAPK15 | 0.406479 | 0.157486 | -0.91289 | 4.276875 |

| | | | | |
|---|---|---|---|---|
| PIP5K1B | 0.406494 | 0.157523 | 2.484881 | 0.315311 |
| PBOV1 | 0.406615 | 0.157601 | 1.441571 | 2.255671 |
| NR0B2 | 0.40669 | 0.157661 | 2.326148 | 0.515412 |
| ACSL3 | 0.407261 | 0.157916 | -1.9804 | 0.772901 |
| LOC101927 | 0.407261 | 0.157945 | 1.350051 | 2.645138 |
| TRIP12 | 0.407427 | 0.15804 | -1.66179 | 0.819182 |
| LOC100131 | 0.40777 | 0.158204 | 0.849893 | 3.398091 |
| CELF2 | 0.407889 | 0.158281 | 1.216393 | 2.742069 |
| RANBP17 | 0.407962 | 0.158341 | 1.208228 | 3.891717 |
| FAM107B | 0.40803 | 0.158398 | 1.690512 | 0.893273 |
| WNT2B | 0.408686 | 0.158688 | -1.73762 | 0.72087 |
| ADGRF5 | 0.408686 | 0.158729 | 1.857474 | 0.593389 |
| GLYCTK-AS | 0.408686 | 0.158747 | 1.496175 | 1.667703 |
| LOC105372 | 0.409757 | 0.159194 | -2.27634 | 0.530801 |
| MAP3K10 | 0.409762 | 0.159227 | 0.794368 | 4.077042 |
| FAM167B | 0.41002 | 0.159359 | 2.005658 | 0.488067 |
| TKT | 0.410145 | 0.159438 | -1.23195 | 2.175102 |
| NRADDP | 0.410543 | 0.159624 | -1.75553 | 1.195102 |
| SNORA78 | 0.410675 | 0.159707 | 0.852027 | 3.520926 |
| CSPG4 | 0.410857 | 0.159809 | 0.556683 | 5.967795 |
| UCP3 | 0.410946 | 0.159879 | 1.757933 | 0.834 |
| GOLGA8S | 0.410946 | 0.159906 | 2.024421 | 0.660838 |
| PRRG1 | 0.411088 | 0.16001 | 1.444603 | 1.173895 |
| IGFLR1 | 0.411088 | 0.160051 | 1.441106 | 1.612545 |
| C16orf74 | 0.411088 | 0.160056 | -1.26719 | 1.834453 |
| JAK2 | 0.412578 | 0.160667 | -0.55886 | 7.032632 |
| SNORD38A | 0.412876 | 0.160815 | 0.910464 | 4.946126 |
| ST8SIA6-AS | 0.413095 | 0.160977 | -2.66414 | 0.839349 |
| FAM87B | 0.413095 | 0.16099 | 1.515054 | 1.847103 |
| NUP188 | 0.413095 | 0.160994 | -0.54135 | 7.02187 |
| MYEF2 | 0.413494 | 0.161194 | 2.16441 | 1.648965 |
| RGL4 | 0.413494 | 0.161213 | 0.672638 | 5.098851 |
| PLCD4 | 0.41362 | 0.161293 | 0.769222 | 4.215038 |
| C17orf51 | 0.413624 | 0.161327 | 0.851077 | 4.241401 |
| TMA7 | 0.41406 | 0.161528 | -0.53348 | 6.881225 |
| KLHL3 | 0.414159 | 0.161598 | 1.634972 | 1.52515 |
| ALK | 0.414686 | 0.161836 | 1.258926 | 2.215414 |
| KLHL14 | 0.414901 | 0.161951 | 1.92497 | 0.666693 |
| FRA10AC1 | 0.415123 | 0.162069 | 0.520521 | 5.755547 |
| MIR3654 | 0.415577 | 0.162278 | -0.76832 | 5.187321 |
| ACACB | 0.415644 | 0.162336 | 1.075259 | 6.461655 |
| FER1L6 | 0.415984 | 0.162501 | 1.268574 | 4.994118 |
| MED17 | 0.416082 | 0.162571 | 0.591943 | 5.100647 |
| SNHG7 | 0.416415 | 0.162759 | -1.12633 | 2.50317 |
| SPRED2 | 0.416415 | 0.16281 | 0.999108 | 2.735173 |

| | | | | |
|---|---|---|---|---|
| DISC2 | 0.416415 | 0.162816 | -1.60917 | 1.841092 |
| CCDC50 | 0.416415 | 0.162843 | 1.017907 | 4.080508 |
| UNC119B | 0.416415 | 0.162859 | -0.56081 | 5.506513 |
| TUBBP5 | 0.416423 | 0.162894 | -1.32519 | 2.391251 |
| SHANK2 | 0.416544 | 0.162973 | 0.847856 | 4.551397 |
| CEL | 0.416643 | 0.163048 | 1.470298 | 2.090452 |
| CRLF3 | 0.416643 | 0.1631 | -1.44026 | 2.032279 |
| PDE4B | 0.416643 | 0.163108 | 1.682053 | 1.788983 |
| GCA | 0.416671 | 0.16315 | 1.023456 | 3.868787 |
| ALG1 | 0.416734 | 0.163207 | 0.826694 | 3.54815 |
| EIF3H | 0.416902 | 0.163304 | -0.60805 | 7.696998 |
| CLNK | 0.416993 | 0.1634 | 1.870924 | 1.041183 |
| MELTF | 0.416993 | 0.163403 | 0.964097 | 3.167634 |
| HOXA3 | 0.416996 | 0.163436 | 1.878395 | 0.576606 |
| TRAPPC2L | 0.41707 | 0.163525 | -0.72544 | 3.952182 |
| NIN | 0.41707 | 0.163529 | -0.58928 | 4.951256 |
| DHX58 | 0.417298 | 0.163659 | 0.803449 | 3.574781 |
| KLHL26 | 0.417298 | 0.163682 | 0.949928 | 3.188137 |
| CLDND2 | 0.417554 | 0.163814 | 1.40646 | 1.395811 |
| GATAD1 | 0.417651 | 0.163892 | 1.146167 | 2.738271 |
| SFXN2 | 0.417651 | 0.163916 | 0.778069 | 3.980146 |
| UTP25 | 0.417677 | 0.163958 | 0.533798 | 5.895978 |
| ZNF354C | 0.417825 | 0.164048 | 0.7919 | 4.228273 |
| SCFD2 | 0.418021 | 0.164156 | -0.57825 | 4.869631 |
| SEC1P | 0.418159 | 0.164242 | 2.133415 | 0.269699 |
| KIF2A | 0.418185 | 0.164303 | -1.35155 | 1.835706 |
| PLTP | 0.418185 | 0.164316 | 1.909526 | 1.607561 |
| PDE6A | 0.418352 | 0.164414 | -1.11695 | 2.393091 |
| FCGR1B | 0.418371 | 0.164453 | -2.18225 | 0.66997 |
| KCTD21 | 0.418437 | 0.164529 | -0.74554 | 4.126437 |
| CHTF18 | 0.418437 | 0.164543 | 0.6489 | 4.508045 |
| NIT1 | 0.41852 | 0.164612 | 0.701468 | 3.775642 |
| PCDHB9 | 0.41852 | 0.164639 | 1.272235 | 2.47966 |
| RGS7BP | 0.418642 | 0.16475 | 2.29675 | 0.461912 |
| MCCC1 | 0.418642 | 0.164752 | -1.19377 | 1.581187 |
| FAM171B | 0.418689 | 0.164802 | 0.840605 | 3.264613 |
| FLRT3 | 0.418942 | 0.164933 | 1.865623 | 1.104108 |
| LOC101928 | 0.418961 | 0.164973 | 1.795417 | 1.905468 |
| TBC1D25 | 0.419805 | 0.165337 | -0.70393 | 4.34191 |
| DNPEP | 0.420388 | 0.165626 | 0.524135 | 5.017576 |
| PI4KA | 0.420388 | 0.165639 | 0.501485 | 7.176215 |
| NEXN | 0.420388 | 0.165688 | 2.158577 | 1.734768 |
| PTENP1 | 0.420388 | 0.165695 | -0.73533 | 4.321013 |
| BRD3 | 0.420558 | 0.165794 | 0.511981 | 6.361039 |
| CTNNBIP1 | 0.42073 | 0.165894 | -2.51409 | 0.53157 |

| | | | | |
|---|---|---|---|---|
| MSL1 | 0.420828 | 0.165964 | -0.48094 | 7.30444 |
| IL10 | 0.420981 | 0.166058 | 2.149199 | 1.140085 |
| BEND3 | 0.420981 | 0.166089 | 0.693224 | 3.861789 |
| MITD1 | 0.421091 | 0.166181 | 0.541817 | 5.110731 |
| DHDH | 0.421091 | 0.166197 | 1.704014 | 0.648255 |
| RNASEH2B | 0.42112 | 0.16624 | 1.678537 | 0.780335 |
| PAFAH1B1 | 0.421202 | 0.166309 | -0.52692 | 7.913042 |
| GP1BA | 0.421202 | 0.166337 | -0.7593 | 3.504282 |
| KDM2A | 0.421202 | 0.166369 | -0.88895 | 3.138526 |
| SSBP3-AS1 | 0.42136 | 0.166482 | -1.24752 | 3.079791 |
| IFT172 | 0.42136 | 0.166512 | 0.542609 | 5.811853 |
| MIR5009 | 0.42136 | 0.166528 | 2.76617 | 1.206033 |
| HEATR3 | 0.421413 | 0.166584 | -0.66222 | 4.73641 |
| BRI3 | 0.421413 | 0.166625 | 0.841077 | 4.387439 |
| FDPS | 0.421413 | 0.166645 | -1.80093 | 1.12874 |
| MTRNR2L9 | 0.421542 | 0.166728 | -1.42641 | 1.961731 |
| DCAF16 | 0.421635 | 0.166797 | 0.529611 | 6.21429 |
| NIPAL3 | 0.421781 | 0.166887 | -0.54061 | 6.198742 |
| SLA | 0.421968 | 0.166993 | 1.643542 | 1.08929 |
| NAAA | 0.422006 | 0.16704 | -1.13001 | 2.873284 |
| SAYSD1 | 0.422024 | 0.167079 | 0.737746 | 3.607515 |
| MTRNR2L1 | 0.422025 | 0.167128 | -1.6444 | 0.923459 |
| UBE3A | 0.422025 | 0.167144 | 1.837183 | 0.832383 |
| LOC100268 | 0.422028 | 0.167185 | 1.906151 | 1.609677 |
| CCL11 | 0.422028 | 0.16721 | 1.556075 | 2.62103 |
| DHX29 | 0.422231 | 0.167322 | -0.49863 | 6.735595 |
| TM9SF3 | 0.422339 | 0.167397 | 0.539462 | 8.978264 |
| C20orf202 | 0.42235 | 0.167434 | 2.878981 | 0.710221 |
| LCAT | 0.422596 | 0.167598 | 1.068294 | 3.165124 |
| C19orf38 | 0.422596 | 0.16761 | 1.838902 | 0.436879 |
| NRDC | 0.422596 | 0.167654 | 1.425559 | 1.487283 |
| DPYS | 0.422596 | 0.16772 | -2.42928 | 0.760085 |
| LINC00630 | 0.422596 | 0.167753 | 0.761815 | 3.947933 |
| MLYCD | 0.422596 | 0.167754 | 0.877246 | 4.184782 |
| KRT17 | 0.422596 | 0.167756 | -1.46761 | 9.127992 |
| RPS14P3 | 0.422637 | 0.167805 | 0.794094 | 3.261037 |
| TMEM200A | 0.422787 | 0.167897 | 1.624972 | 0.951998 |
| REEP3 | 0.422972 | 0.168003 | 0.573348 | 6.865794 |
| MIR3176 | 0.422998 | 0.168052 | 1.722302 | 0.926439 |
| ILKAP | 0.422998 | 0.168079 | 0.564538 | 5.42434 |
| PFKL | 0.422998 | 0.168134 | 1.512186 | 2.487305 |
| APOOL | 0.422998 | 0.168146 | -0.5456 | 5.803201 |
| CNIH1 | 0.422998 | 0.168174 | -0.6551 | 5.573759 |
| WDR20 | 0.423151 | 0.168267 | -1.01923 | 2.956275 |
| TNFRSF25 | 0.423157 | 0.168302 | -0.87319 | 3.957977 |

| | | | | |
|---|---|---|---|---|
| MIR5579 | 0.423161 | 0.168336 | 2.233027 | 0.353168 |
| CHSY3 | 0.423264 | 0.168435 | 1.32051 | 2.13327 |
| SHC3 | 0.423264 | 0.1685 | 0.953895 | 4.182695 |
| PCDHGC4 | 0.423264 | 0.168503 | -1.96931 | 0.498484 |
| RPS26 | 0.423264 | 0.168506 | -0.7517 | 5.014276 |
| SPDYE7P | 0.423321 | 0.168561 | -0.80367 | 3.304518 |
| FAM220A | 0.423504 | 0.168675 | -0.68616 | 4.130118 |
| HIST1H2BH | 0.423504 | 0.168698 | 0.670549 | 4.681241 |
| PLXNA3 | 0.423803 | 0.168849 | 0.533078 | 6.04841 |
| KCTD20 | 0.423941 | 0.168937 | -1.08557 | 2.885771 |
| MRPL20 | 0.423983 | 0.168986 | -0.69856 | 5.013349 |
| SNORD104 | 0.424377 | 0.169198 | 0.842071 | 6.425161 |
| ARMC2-AS | 0.424377 | 0.169207 | 1.616212 | 1.078605 |
| ZNF799 | 0.424506 | 0.16933 | 1.121744 | 2.124038 |
| PPP1R3F | 0.424506 | 0.169334 | -1.17954 | 1.824987 |
| SLC5A5 | 0.424506 | 0.169356 | 2.539979 | 3.504394 |
| 2-Mar | 0.424732 | 0.169479 | -1.51861 | 0.791515 |
| RAD18 | 0.424965 | 0.169632 | 0.605209 | 5.138201 |
| SUMO2 | 0.424965 | 0.169636 | -1.80787 | 1.106299 |
| DIS3 | 0.42514 | 0.169739 | -1.04913 | 3.143323 |
| HLA-J | 0.425383 | 0.169868 | 1.22041 | 2.049979 |
| EMILIN3 | 0.425507 | 0.169966 | 1.371077 | 1.422177 |
| DHRS12 | 0.425507 | 0.169987 | -0.98328 | 2.177455 |
| KLHL11 | 0.425507 | 0.170034 | 0.479193 | 5.724138 |
| NAA25 | 0.425507 | 0.170047 | -0.54496 | 6.97161 |
| PROB1 | 0.426109 | 0.17032 | 1.17938 | 2.288193 |
| CLK2P1 | 0.426115 | 0.170355 | 1.381841 | 1.821026 |
| EPN2 | 0.426334 | 0.170475 | -2.05134 | 0.381111 |
| CEP44 | 0.426637 | 0.170629 | -0.62173 | 4.526316 |
| MAFIP | 0.426847 | 0.170745 | -0.83487 | 4.658626 |
| LINC02384 | 0.427246 | 0.171009 | 2.532996 | 0.636101 |
| SDHAP2 | 0.427246 | 0.17101 | -0.50028 | 5.830766 |
| TMEM116 | 0.427246 | 0.171015 | -1.94213 | 0.668578 |
| CPSF3 | 0.427246 | 0.171035 | -0.55274 | 5.942233 |
| CIP2A | 0.427502 | 0.17117 | 0.822158 | 4.359286 |
| MMP17 | 0.427913 | 0.171396 | 1.65466 | 2.169959 |
| LOC10013C | 0.427913 | 0.1714 | 0.871432 | 3.476732 |
| CD48 | 0.42793 | 0.171439 | 1.902923 | 1.038354 |
| FNBP1 | 0.427976 | 0.171491 | 0.605074 | 7.44839 |
| LEAP2 | 0.428069 | 0.17156 | -1.8514 | 1.333082 |
| SLC35A1 | 0.4281 | 0.171605 | 1.591194 | 1.145394 |
| MFSD6 | 0.428172 | 0.171667 | -0.50941 | 7.894654 |
| TRAIP | 0.428311 | 0.171755 | 0.835073 | 2.629833 |
| STT3A-AS1 | 0.429043 | 0.172089 | -1.67172 | 1.243863 |
| PLA2G3 | 0.429043 | 0.172114 | -1.26769 | 4.030755 |

| | | | | |
|---|---|---|---|---|
| OIP5-AS1 | 0.429228 | 0.172248 | -0.50942 | 5.816294 |
| MBLAC2 | 0.429228 | 0.172254 | -0.92762 | 3.525151 |
| AMOTL1 | 0.42929 | 0.172314 | -1.67481 | 1.265332 |
| COMMD3 | 0.42929 | 0.172372 | 1.274954 | 2.576416 |
| WDR36 | 0.42929 | 0.172396 | -0.54072 | 6.349372 |
| LINC00265 | 0.42929 | 0.172416 | -0.67013 | 4.718264 |
| RIOX1 | 0.42929 | 0.172442 | 0.815661 | 3.582678 |
| EXD3 | 0.42942 | 0.172527 | 0.695275 | 3.826946 |
| TIMM44 | 0.429507 | 0.172637 | 0.529247 | 5.376232 |
| XRCC2 | 0.429507 | 0.172658 | 1.254503 | 2.982047 |
| AGL | 0.429507 | 0.172661 | -1.25958 | 1.732018 |
| CYTH3 | 0.429624 | 0.172744 | 0.54994 | 5.999993 |
| NLK | 0.429624 | 0.172789 | 0.512294 | 5.190979 |
| CORT | 0.429624 | 0.172806 | 1.97498 | 1.485222 |
| NLRP14 | 0.429985 | 0.172989 | 2.001574 | 0.438207 |
| MRPL51 | 0.429985 | 0.173016 | -0.56154 | 6.11396 |
| STK39 | 0.430321 | 0.173185 | -0.50448 | 7.54244 |
| LINC01600 | 0.430649 | 0.173349 | 2.284575 | 1.63738 |
| EZH2 | 0.430726 | 0.173413 | 1.825744 | 0.865564 |
| SOX9-AS1 | 0.430983 | 0.173549 | 1.722065 | 1.548836 |
| GBP1 | 0.431267 | 0.173723 | 0.705664 | 5.74373 |
| FIGN | 0.431267 | 0.173735 | 0.957281 | 3.371358 |
| C1orf226 | 0.431267 | 0.173762 | 0.836447 | 4.1296 |
| TEX45 | 0.431401 | 0.173849 | 2.066179 | 0.428894 |
| MYH16 | 0.431525 | 0.173962 | 1.51031 | 1.272511 |
| KRTCAP2 | 0.431525 | 0.173978 | 0.552361 | 5.160003 |
| ATAD3C | 0.431525 | 0.173998 | 1.263149 | 3.04621 |
| CCDC96 | 0.431646 | 0.174079 | -0.98105 | 2.165487 |
| ADM2 | 0.43169 | 0.17413 | 0.839168 | 3.436486 |
| INPP5E | 0.431732 | 0.174194 | 0.650642 | 4.258117 |
| ARHGAP10 | 0.431732 | 0.174213 | -0.63721 | 7.116137 |
| TLCD2 | 0.43192 | 0.174322 | -0.73446 | 6.670712 |
| HERC3 | 0.432401 | 0.174549 | -0.68869 | 3.809198 |
| MTG2 | 0.432557 | 0.174644 | 0.583958 | 4.818077 |
| TECTA | 0.432838 | 0.174791 | 1.341355 | 1.908813 |
| RBM48 | 0.433041 | 0.174906 | 0.725132 | 4.506767 |
| LINC01214 | 0.433049 | 0.174942 | -2.55677 | 1.003894 |
| TMEM178I | 0.433124 | 0.175006 | -1.0958 | 3.449204 |
| UST | 0.433166 | 0.175081 | 0.767264 | 4.332813 |
| ZBTB37 | 0.433166 | 0.175101 | 0.828828 | 3.057516 |
| LPAR6 | 0.433166 | 0.175122 | -1.02504 | 1.846028 |
| TWISTNB | 0.433406 | 0.175252 | 0.583932 | 5.063313 |
| LY86 | 0.433594 | 0.175377 | 1.616753 | 1.293594 |
| LOC100131 | 0.433594 | 0.175394 | 1.731525 | 0.465069 |
| REXO1 | 0.433692 | 0.175492 | 0.513023 | 5.877084 |

| | | | | |
|---|---|---|---|---|
| LINC01569 | 0.433692 | 0.1755 | 1.434818 | 1.552619 |
| BIK | 0.434114 | 0.175703 | -0.78248 | 4.517272 |
| SNORA22 | 0.434755 | 0.176006 | 0.60376 | 5.98135 |
| FLG-AS1 | 0.434755 | 0.176052 | -2.17045 | 1.247104 |
| R3HDM4 | 0.434755 | 0.176062 | -0.51969 | 6.199724 |
| MICAL2 | 0.434911 | 0.176187 | 2.155073 | 0.42677 |
| NEFL | 0.434911 | 0.176192 | -1.83697 | 2.139333 |
| MTRF1 | 0.434923 | 0.176254 | 0.682349 | 4.500577 |
| HDAC4 | 0.434923 | 0.176263 | 0.87897 | 7.562427 |
| HSD17B6 | 0.435029 | 0.176339 | 2.000543 | 1.707854 |
| IFT140 | 0.435246 | 0.17646 | 0.741974 | 5.735409 |
| MPHOSPH8 | 0.435509 | 0.1766 | 0.544141 | 7.757267 |
| NOP16 | 0.436099 | 0.176873 | -1.16995 | 2.054481 |
| SAP30BP | 0.436177 | 0.176937 | -1.94246 | 0.932055 |
| CDH6 | 0.43627 | 0.177039 | 1.000246 | 4.319479 |
| ST14 | 0.43627 | 0.177043 | -0.55535 | 7.93127 |
| CFAP46 | 0.43627 | 0.177075 | 1.200243 | 3.665604 |
| RHOF | 0.43687 | 0.177355 | 2.068751 | 1.702281 |
| YBX3 | 0.43687 | 0.177385 | -0.67576 | 3.669332 |
| RNF149 | 0.436885 | 0.177424 | -0.56864 | 7.093928 |
| RPA3 | 0.437024 | 0.177514 | 0.75166 | 4.228735 |
| EIF1B | 0.437136 | 0.177601 | 0.579189 | 4.403591 |
| FAM45BP | 0.437136 | 0.177645 | -0.94074 | 2.549054 |
| MIER1 | 0.437136 | 0.177663 | 0.778469 | 3.332662 |
| MTRNR2L2 | 0.437136 | 0.177693 | -0.78568 | 4.316518 |
| JPT1 | 0.437149 | 0.177746 | 1.229234 | 2.028638 |
| INHBA-AS1 | 0.437149 | 0.177804 | 2.036035 | 0.539422 |
| KCNMA1 | 0.437149 | 0.177805 | 1.440454 | 2.147113 |
| PNPLA6 | 0.437149 | 0.177831 | 1.602792 | 0.690437 |
| AARS | 0.437323 | 0.177935 | 0.548829 | 6.996854 |
| MYRF | 0.437462 | 0.178058 | 1.65051 | 2.125802 |
| CYP2G1P | 0.437462 | 0.178059 | 2.391276 | 0.711311 |
| PUSL1 | 0.437749 | 0.178209 | -0.74507 | 3.652046 |
| KL | 0.437897 | 0.178328 | -1.3675 | 1.966798 |
| TOP2A | 0.437897 | 0.178336 | 0.63096 | 7.299658 |
| BLZF1 | 0.438133 | 0.178494 | -0.53884 | 6.024454 |
| LOC10192S | 0.438133 | 0.178499 | 1.596484 | 1.344601 |
| STK40 | 0.438197 | 0.178558 | -1.29728 | 1.80893 |
| CFAP157 | 0.438269 | 0.178621 | 1.185727 | 2.327975 |
| TTC33 | 0.438582 | 0.178782 | 0.729506 | 4.745983 |
| SHB | 0.438618 | 0.17883 | -0.523 | 6.50115 |
| SEC14L2 | 0.438795 | 0.178942 | -1.2882 | 2.646871 |
| NKILA | 0.438795 | 0.178987 | 1.403659 | 1.46421 |
| BRWD3 | 0.438795 | 0.179002 | -0.50994 | 7.6612 |
| OSMR-AS1 | 0.439198 | 0.1792 | 1.440374 | 1.404037 |

| | | | | |
|---|---|---|---|---|
| CCDC102A | 0.439229 | 0.179276 | 0.884083 | 3.399777 |
| NPIPA1 | 0.439229 | 0.17928 | 1.189318 | 1.785674 |
| DNAJC27-A | 0.439578 | 0.179456 | 2.010387 | 0.636897 |
| ZNF793 | 0.439701 | 0.17954 | -0.72843 | 3.880081 |
| LOC101927 | 0.43997 | 0.179683 | -2.25401 | 1.169212 |
| LOC101927 | 0.439988 | 0.179724 | 1.644805 | 1.116005 |
| GPR4 | 0.440007 | 0.179795 | 1.01392 | 2.465953 |
| MIR4312 | 0.440007 | 0.179798 | -2.04794 | 0.775255 |
| LOC100289 | 0.440232 | 0.179937 | -0.70385 | 3.505979 |
| CCND2 | 0.440232 | 0.179958 | 0.660966 | 8.073561 |
| PCGF1 | 0.440388 | 0.180074 | 0.714551 | 3.48618 |
| TELO2 | 0.440388 | 0.180089 | 0.613432 | 4.821327 |
| PSMD8 | 0.440527 | 0.180179 | -0.5642 | 6.592886 |
| UBXN7 | 0.44054 | 0.180218 | -0.48366 | 7.039046 |
| COBL | 0.440655 | 0.180309 | 0.699619 | 3.825232 |
| LINC00471 | 0.440655 | 0.180332 | -1.59609 | 1.695075 |
| MIR320D1 | 0.440882 | 0.1805 | 1.873913 | 0.596928 |
| NT5C1A | 0.440882 | 0.180526 | 3.111312 | 1.182839 |
| FIBIN | 0.440882 | 0.180548 | 1.454468 | 2.713642 |
| HOXB2 | 0.440882 | 0.18056 | 0.776302 | 4.600468 |
| LSM10 | 0.441301 | 0.180773 | 0.811006 | 3.583821 |
| DNAJB11 | 0.441301 | 0.180798 | 0.52303 | 7.022246 |
| KIAA1328 | 0.441428 | 0.180884 | 0.770207 | 4.434914 |
| DTX4 | 0.441441 | 0.180923 | 1.631331 | 1.298982 |
| MTSS1 | 0.441593 | 0.181019 | -2.11893 | 1.083015 |
| C12orf73 | 0.44181 | 0.181168 | 0.905043 | 2.675087 |
| FAF1 | 0.44181 | 0.181175 | -0.48161 | 6.676409 |
| UHRF1BP1 | 0.442083 | 0.181321 | 0.49162 | 5.437967 |
| CUEDC1 | 0.442162 | 0.181387 | 1.811879 | 0.664145 |
| LINC00999 | 0.442218 | 0.181486 | -0.85753 | 3.231052 |
| KATNBL1P6 | 0.442218 | 0.1815 | -1.16289 | 2.139937 |
| OGFR | 0.442218 | 0.181519 | 0.613031 | 5.690171 |
| FLJ42627 | 0.442218 | 0.181545 | 0.582443 | 5.645247 |
| RPL34-AS1 | 0.442225 | 0.181581 | 1.888899 | 1.167211 |
| JPH1 | 0.442317 | 0.181653 | 1.626596 | 1.82782 |
| RPL23 | 0.442756 | 0.181867 | -0.53068 | 8.959176 |
| SRP19 | 0.442947 | 0.182004 | 0.705152 | 4.463283 |
| KMT2C | 0.442947 | 0.182036 | -0.48265 | 9.731441 |
| AP5B1 | 0.442947 | 0.182046 | -0.52501 | 5.934813 |
| LMBRD2 | 0.443024 | 0.182112 | -0.56956 | 5.115868 |
| EDEM1 | 0.443118 | 0.182184 | -0.51337 | 7.232453 |
| FARSB | 0.443125 | 0.182236 | -1.04221 | 2.289333 |
| ZNF366 | 0.443125 | 0.18228 | 0.837621 | 3.240996 |
| ZNF607 | 0.443125 | 0.182315 | -1.11156 | 1.698367 |
| SEC22B | 0.443125 | 0.18236 | 0.477006 | 5.976475 |

| | | | | |
|---|---|---|---|---|
| PAPLN | 0.443125 | 0.18236 | 0.579512 | 5.272792 |
| CDYL | 0.443125 | 0.18239 | -1.28699 | 2.178802 |
| SOX6 | 0.443212 | 0.182459 | -2.33327 | 0.653829 |
| BRAP | 0.44332 | 0.182546 | 0.474133 | 5.547084 |
| CYP20A1 | 0.44332 | 0.182571 | 0.539269 | 6.159351 |
| RNY5 | 0.44355 | 0.1827 | 0.85629 | 8.188801 |
| NEFH | 0.443678 | 0.182835 | -1.29419 | 1.810923 |
| ARSJ | 0.443678 | 0.182852 | 0.632248 | 4.054784 |
| NR2C1 | 0.443678 | 0.182854 | 0.943511 | 3.271695 |
| MIR548I2 | 0.443695 | 0.182895 | 2.034046 | 1.011503 |
| DLEU2 | 0.443898 | 0.183012 | 0.767055 | 3.708755 |
| FAM151A | 0.443917 | 0.183054 | 0.955611 | 2.380904 |
| CCDC38 | 0.443931 | 0.183094 | 2.03589 | 0.593239 |
| TMEM190 | 0.444016 | 0.1832 | 1.270246 | 1.609539 |
| GOLGA6L4 | 0.444016 | 0.18321 | -0.98287 | 1.944418 |
| C12orf71 | 0.444016 | 0.18323 | -1.89757 | 0.839857 |
| LOC10192E | 0.444021 | 0.183266 | -1.74902 | 0.709359 |
| AP2S1 | 0.444056 | 0.183319 | -0.83788 | 2.193319 |
| SH3GLB1 | 0.444056 | 0.183369 | -1.39364 | 1.958522 |
| CCL21 | 0.444056 | 0.183382 | 1.61341 | 1.236751 |
| HRASLS2 | 0.444159 | 0.183459 | 1.6667 | 1.613248 |
| MYOZ1 | 0.44418 | 0.183501 | -1.62544 | 1.427618 |
| NUPL2 | 0.444299 | 0.183584 | 0.610117 | 4.612133 |
| CTR9 | 0.444379 | 0.183651 | -0.48432 | 7.003995 |
| PNLIPRP3 | 0.444447 | 0.183713 | -2.02499 | 2.052542 |
| SALL4 | 0.444466 | 0.183755 | 1.821668 | 2.319982 |
| SAA2 | 0.444975 | 0.183999 | 2.80113 | 0.712311 |
| FAM19A2 | 0.44526 | 0.184157 | 1.534781 | 1.310849 |
| CLK3 | 0.44526 | 0.184217 | -1.25643 | 0.750938 |
| BAHCC1 | 0.44526 | 0.184251 | 0.51663 | 5.412582 |
| GREM2 | 0.44526 | 0.184252 | 1.454675 | 4.480364 |
| MRPS5 | 0.445274 | 0.184292 | -0.47291 | 6.666849 |
| DIPK2A | 0.445322 | 0.184346 | -1.00193 | 2.138595 |
| SGK2 | 0.445529 | 0.184528 | 1.784093 | 1.439249 |
| BYSL | 0.445529 | 0.184532 | 0.737154 | 3.762592 |
| FBXW5 | 0.445529 | 0.184534 | -0.52996 | 6.253204 |
| TCEAL2 | 0.445547 | 0.184575 | 2.132757 | 2.432815 |
| TRIM39 | 0.4456 | 0.184631 | -1.89942 | 1.312587 |
| C15orf61 | 0.445954 | 0.184833 | 1.828379 | 0.664548 |
| SWSAP1 | 0.445954 | 0.184854 | 1.176877 | 1.230457 |
| SH3BP5-AS | 0.445954 | 0.18488 | 0.856837 | 4.306374 |
| DENND4C | 0.44596 | 0.184931 | -0.80344 | 2.954888 |
| CDC42BPB | 0.44596 | 0.18495 | -0.4888 | 8.687715 |
| SRGAP2B | 0.445974 | 0.18499 | 0.805056 | 4.380909 |
| ZNF646 | 0.445975 | 0.185024 | 0.518554 | 5.747099 |

| | | | | |
|---|---|---|---|---|
| FSIP2-AS2 | 0.445988 | 0.185064 | 3.066959 | 0.816383 |
| RB1-DT | 0.446284 | 0.185221 | 2.283459 | 1.057571 |
| PLPP2 | 0.446972 | 0.18554 | 1.975052 | 1.443881 |
| DAAM2 | 0.447116 | 0.185657 | 1.679822 | 1.393432 |
| LOC100287 | 0.447116 | 0.185672 | -1.66447 | 0.719531 |
| ARHGAP17 | 0.447116 | 0.18578 | 1.81941 | 0.841692 |
| EIF4A3 | 0.447116 | 0.185794 | -0.59418 | 6.293552 |
| STAU1 | 0.447116 | 0.185832 | 1.530567 | 1.357682 |
| LRFN4 | 0.447116 | 0.185834 | 0.752308 | 4.054005 |
| VRK1 | 0.447116 | 0.185877 | -0.63951 | 4.406439 |
| LOC729683 | 0.447116 | 0.1859 | 1.513172 | 0.911202 |
| SLFNL1-AS1 | 0.447116 | 0.185906 | 0.918972 | 3.805741 |
| PRH1 | 0.44738 | 0.18605 | 2.092753 | 0.41388 |
| CLN8 | 0.447413 | 0.186098 | -1.13601 | 4.227796 |
| PCDHB8 | 0.447549 | 0.186189 | 1.654972 | 2.534738 |
| KLHDC10 | 0.448312 | 0.18654 | -0.46955 | 6.470657 |
| FAM193A | 0.448358 | 0.186594 | -1.2106 | 2.082256 |
| SNHG28 | 0.448503 | 0.186688 | -1.24825 | 1.937893 |
| SLC10A1 | 0.448558 | 0.186756 | -0.9876 | 2.821851 |
| MIR4523 | 0.448558 | 0.18678 | 1.305013 | 0.84678 |
| LRP3 | 0.448651 | 0.186853 | 0.999021 | 3.223657 |
| PTRH2 | 0.448663 | 0.1869 | 1.069021 | 2.843083 |
| DNAH8 | 0.448663 | 0.186943 | 1.801371 | 1.107119 |
| VWA3B | 0.448663 | 0.18696 | 1.796502 | 1.403184 |
| ZNF695 | 0.448782 | 0.187044 | 1.60336 | 1.928803 |
| SOGA3 | 0.448955 | 0.18715 | 1.325149 | 1.87189 |
| CCDC130 | 0.449573 | 0.187442 | 0.612724 | 4.774271 |
| SNORA58 | 0.449711 | 0.187535 | 0.727756 | 6.627914 |
| N4BP2 | 0.449711 | 0.187568 | -0.48503 | 6.408821 |
| SLITRK3 | 0.450024 | 0.187744 | 2.308234 | 1.552401 |
| PSMB4 | 0.450024 | 0.187767 | 0.498173 | 5.409648 |
| ELF4 | 0.450034 | 0.187812 | 1.568026 | 1.123886 |
| SGK494 | 0.450034 | 0.187851 | 0.531922 | 5.53261 |
| RNF144B | 0.450034 | 0.187874 | -0.59879 | 6.33228 |
| PTGR1 | 0.450061 | 0.18792 | -1.64488 | 0.709402 |
| AKIRIN2 | 0.450374 | 0.188085 | -0.60406 | 5.370097 |
| WNT3 | 0.450745 | 0.188283 | 1.57638 | 1.097638 |
| ANKRD40 | 0.450745 | 0.188308 | 0.503865 | 6.256763 |
| FAM162B | 0.450824 | 0.188376 | 1.553148 | 1.140783 |
| NUTM2A-A | 0.450833 | 0.188414 | 0.748014 | 3.737991 |
| SLAIN1 | 0.451083 | 0.188553 | 2.174928 | 1.140143 |
| FLRT2 | 0.451477 | 0.188784 | 0.696039 | 4.526132 |
| GLA | 0.451477 | 0.188786 | 1.020985 | 3.357754 |
| ATP1B1 | 0.451496 | 0.188846 | -0.52863 | 9.13793 |
| SNORD130 | 0.451496 | 0.188877 | -2.18664 | 0.74 |

| | | | | |
|---|---|---|---|---|
| NECAP1 | 0.451496 | 0.188897 | 1.75378 | 0.944392 |
| LIN52 | 0.451598 | 0.188975 | 0.789193 | 3.061024 |
| LOC100132 | 0.45163 | 0.189022 | -0.93667 | 4.27441 |
| FZR1 | 0.452229 | 0.189308 | -1.13727 | 2.094469 |
| NEK8 | 0.452264 | 0.189357 | 0.88415 | 2.922709 |
| STK17B | 0.452523 | 0.1895 | 0.602917 | 6.430885 |
| SNORD116 | 0.452825 | 0.189672 | 1.567283 | 1.195182 |
| MIR1183 | 0.452825 | 0.189695 | 1.890516 | 1.066305 |
| VPS37D | 0.453127 | 0.189856 | 1.844186 | 0.632404 |
| REM1 | 0.453201 | 0.189932 | 2.020743 | 0.770154 |
| GRIK5 | 0.453201 | 0.189956 | 2.004941 | 0.570208 |
| HPCA | 0.453225 | 0.190001 | 2.211976 | 0.68672 |
| LOC101929 | 0.453601 | 0.190193 | 1.965122 | 1.525088 |
| TOMM22 | 0.453754 | 0.190297 | -0.48317 | 5.872982 |
| GIGYF1 | 0.453754 | 0.190326 | 0.500048 | 7.492203 |
| B3GNT2 | 0.453811 | 0.190385 | -0.48118 | 5.984468 |
| LRRC8C | 0.454033 | 0.190543 | 0.611752 | 4.669977 |
| TPCN2 | 0.454033 | 0.190567 | 0.523524 | 5.528512 |
| CBWD6 | 0.454033 | 0.190582 | 1.015836 | 2.985127 |
| SAV1 | 0.454804 | 0.190949 | 0.600833 | 5.175539 |
| REL | 0.454804 | 0.190975 | -1.6196 | 0.44503 |
| DSTNP2 | 0.454808 | 0.191011 | 1.193496 | 1.993869 |
| METRN | 0.454812 | 0.191047 | 1.283439 | 2.43201 |
| EZR | 0.454851 | 0.191098 | -1.35128 | 0.845264 |
| TNNI1 | 0.455041 | 0.19123 | 1.226676 | 3.258776 |
| IL10RB | 0.455041 | 0.191247 | -0.51763 | 5.204123 |
| HAUS7 | 0.455091 | 0.191303 | 1.504848 | 1.356058 |
| KMT5C | 0.455412 | 0.191502 | 0.854084 | 3.636658 |
| SMARCA2 | 0.455412 | 0.191512 | 1.326684 | 1.143973 |
| DDX3X | 0.455412 | 0.191542 | -0.618 | 4.956611 |
| SNORA9 | 0.45594 | 0.191799 | 0.846635 | 3.996785 |
| MYH3 | 0.456002 | 0.191894 | 0.797656 | 3.758945 |
| RALBP1 | 0.456002 | 0.191897 | -0.60818 | 8.44177 |
| RTTN | 0.456002 | 0.191929 | 0.524829 | 5.232098 |
| F12 | 0.456428 | 0.192176 | -1.04032 | 2.17057 |
| CEP89 | 0.456428 | 0.192193 | 0.614613 | 5.22041 |
| NDUFV2-AS | 0.456428 | 0.192218 | 1.273903 | 1.968239 |
| ZDHHC24 | 0.456428 | 0.192248 | 0.994451 | 2.415621 |
| SLC6A7 | 0.456435 | 0.192286 | 2.851789 | 0.741002 |
| NMI | 0.456474 | 0.192376 | 0.560164 | 4.706252 |
| SNORA37 | 0.456474 | 0.1924 | -0.64241 | 4.979683 |
| DOCK9-DT | 0.456474 | 0.192407 | -1.03864 | 2.655263 |
| UGT1A10 | 0.456582 | 0.192487 | 1.798375 | 2.488356 |
| QRFPR | 0.456942 | 0.192673 | -1.78817 | 1.411015 |
| ANKRD23 | 0.456965 | 0.192718 | 0.827373 | 3.311511 |

| | | | | |
|---|---|---|---|---|
| ST7-OT3 | 0.457013 | 0.192834 | -1.33569 | 1.918981 |
| TEX13B | 0.457013 | 0.192887 | -1.72766 | 0.593497 |
| BAIAP2-DT | 0.457013 | 0.192892 | 0.642514 | 4.061312 |
| ROR1-AS1 | 0.457013 | 0.192936 | 1.314499 | 2.417732 |
| RAMP2 | 0.457013 | 0.192956 | 1.22524 | 1.648856 |
| COG7 | 0.457013 | 0.192972 | 0.558814 | 5.165749 |
| MFSD6L | 0.457013 | 0.192982 | 1.523808 | 1.824723 |
| IGSF6 | 0.457022 | 0.193041 | -0.70868 | 4.068454 |
| LINC01039 | 0.457022 | 0.193119 | 2.042167 | 0.781911 |
| PLAC9 | 0.457022 | 0.193128 | 0.724552 | 3.314634 |
| CD276 | 0.457022 | 0.193145 | 1.304589 | 2.096515 |
| HTRA4 | 0.457022 | 0.19316 | 1.566539 | 0.797212 |
| TEKT3 | 0.457203 | 0.193274 | 2.711225 | 0.294263 |
| KLRG1 | 0.457203 | 0.193306 | 1.455488 | 1.376878 |
| TMOD4 | 0.457257 | 0.193364 | 1.397511 | 1.191005 |
| NCF1B | 0.457426 | 0.193504 | 1.306026 | 2.236169 |
| DECR1 | 0.457426 | 0.193532 | -0.48085 | 5.883196 |
| TACR2 | 0.457426 | 0.193549 | 1.351477 | 3.020074 |
| GLCE | 0.457426 | 0.193575 | -0.56356 | 5.694584 |
| TPRKB | 0.457434 | 0.19362 | -0.58458 | 4.599272 |
| LINC00260 | 0.457434 | 0.19366 | 0.801301 | 3.072323 |
| MSH3 | 0.457434 | 0.193682 | -0.51479 | 5.505429 |
| DDX31 | 0.457709 | 0.193834 | -0.87648 | 3.977939 |
| CCDC71 | 0.458153 | 0.194057 | 0.787853 | 3.374982 |
| CHMP2B | 0.458154 | 0.194174 | 1.406739 | 0.953887 |
| UBE2V1 | 0.458154 | 0.194177 | -1.55546 | 1.067138 |
| BET1 | 0.458154 | 0.194178 | 1.574331 | 1.636148 |
| SNORD114 | 0.458154 | 0.194197 | 1.452727 | 2.186707 |
| GUSBP4 | 0.45827 | 0.194281 | 0.560411 | 4.892098 |
| PROC | 0.458506 | 0.19444 | 1.612437 | 1.012449 |
| TANC2 | 0.458506 | 0.194451 | -0.4807 | 7.029778 |
| SCAMP4 | 0.458545 | 0.194502 | -0.46899 | 5.430022 |
| KLF3-AS1 | 0.458576 | 0.194551 | -1.2766 | 1.712863 |
| CLEC4D | 0.459005 | 0.194779 | 2.043699 | 0.617915 |
| MYL5 | 0.459005 | 0.194803 | -0.88873 | 2.598302 |
| NXPE3 | 0.459125 | 0.194888 | 1.525629 | 0.903621 |
| MUS81 | 0.45916 | 0.194963 | 0.551981 | 4.682965 |
| POU2AF1 | 0.45916 | 0.194973 | 1.141027 | 4.777531 |
| CCDC154 | 0.459344 | 0.195146 | 1.442077 | 2.251054 |
| LINC00673 | 0.459344 | 0.195199 | -0.73714 | 5.256026 |
| MIR4742 | 0.459344 | 0.195227 | -1.85281 | 0.452481 |
| ZNF451 | 0.459344 | 0.195236 | 0.481727 | 5.914507 |
| ZSWIM6 | 0.459344 | 0.195237 | -0.50313 | 6.619376 |
| BEND6 | 0.459344 | 0.195262 | 1.889733 | 1.023945 |
| HARBI1 | 0.459471 | 0.19535 | 0.968778 | 2.372023 |

| | | | | |
|---|---|---|---|---|
| SNX8 | 0.459472 | 0.195386 | 0.598373 | 5.268616 |
| SSTR1 | 0.459561 | 0.195466 | 1.738282 | 3.986386 |
| ZNF578 | 0.459561 | 0.195494 | 1.900416 | 1.599241 |
| KIF13A | 0.459585 | 0.19556 | 0.618363 | 4.505497 |
| ERI3-IT1 | 0.459585 | 0.195592 | 0.785381 | 2.829282 |
| FKBP1A | 0.459585 | 0.19562 | 1.656772 | 1.327625 |
| ARHGAP5-/ | 0.459585 | 0.195644 | -1.19591 | 2.609179 |
| ZNF214 | 0.459802 | 0.195811 | 0.987315 | 2.278484 |
| TIRAP | 0.459802 | 0.195881 | 0.763849 | 3.362274 |
| SRPRA | 0.459802 | 0.195907 | 1.34734 | 2.093186 |
| BBS1 | 0.459802 | 0.195909 | 0.535017 | 4.925812 |
| MGAT4A | 0.459802 | 0.195912 | 0.781431 | 4.522514 |
| C1QTNF7 | 0.459827 | 0.195957 | 1.7317 | 1.712158 |
| ZHX2 | 0.459875 | 0.196025 | 0.479776 | 5.633792 |
| SETD1A | 0.459875 | 0.196048 | 0.518312 | 6.298364 |
| FAS-AS1 | 0.460329 | 0.196277 | 1.825008 | 0.508837 |
| CYS1 | 0.460467 | 0.196374 | 1.34804 | 2.221075 |
| H2AFY2 | 0.460467 | 0.196406 | -0.65119 | 4.382858 |
| SDR42E1 | 0.460486 | 0.196449 | -0.68406 | 4.007191 |
| ST8SIA4 | 0.460687 | 0.19657 | 0.780962 | 3.563008 |
| LOC100128 | 0.460801 | 0.196702 | 1.427298 | 1.145671 |
| FTX | 0.460801 | 0.196714 | 0.663426 | 4.889131 |
| RAB33B | 0.460801 | 0.196723 | 0.645907 | 4.39429 |
| EIF3G | 0.460841 | 0.196776 | 0.46334 | 6.601408 |
| CYB5R4 | 0.4609 | 0.196869 | -0.5677 | 4.936991 |
| HEG1 | 0.4609 | 0.196882 | -0.61859 | 7.591974 |
| ZKSCAN3 | 0.4609 | 0.196906 | 1.751561 | 0.731648 |
| BRD7P3 | 0.460985 | 0.196983 | 1.395812 | 1.634581 |
| MIR3975 | 0.460985 | 0.197013 | -1.32563 | 2.329757 |
| PHLDB1 | 0.461164 | 0.197125 | 1.656378 | 0.486477 |
| TNFRSF9 | 0.461596 | 0.197414 | 1.329863 | 1.907074 |
| HCG26 | 0.461596 | 0.197494 | 1.03629 | 2.983488 |
| RPPH1 | 0.461596 | 0.197507 | -0.48988 | 12.72724 |
| FAM86HP | 0.461596 | 0.197517 | 0.97616 | 2.803898 |
| GIF | 0.461596 | 0.197519 | -2.65193 | 7.593592 |
| LINC00337 | 0.461596 | 0.19752 | -2.24566 | 0.517259 |
| CYP2S1 | 0.462258 | 0.197839 | 0.658556 | 5.745532 |
| SCARNA12 | 0.462384 | 0.197928 | -0.47069 | 6.9396 |
| HLA-DRB1 | 0.462767 | 0.198127 | 1.240127 | 4.280944 |
| LINC00960 | 0.462919 | 0.198228 | 1.874128 | 0.553676 |
| PLD6 | 0.463125 | 0.198351 | 1.170658 | 3.024839 |
| RERE | 0.463337 | 0.198506 | -1.22986 | 2.381044 |
| MCTP2 | 0.463337 | 0.198512 | -0.92346 | 2.896454 |
| GPR158 | 0.463346 | 0.198552 | 1.936666 | 1.914597 |
| ABHD3 | 0.463493 | 0.19865 | -1.09599 | 2.649967 |

| | | | | |
|---|---|---|---|---|
| PMS2P3 | 0.463532 | 0.198702 | 0.632935 | 4.958902 |
| FRAT2 | 0.463735 | 0.198827 | 0.570459 | 4.911993 |
| ARRDC5 | 0.463735 | 0.19886 | 1.169822 | 1.899789 |
| NSD1 | 0.463842 | 0.198941 | -0.75041 | 3.173373 |
| RPLP2 | 0.463931 | 0.199015 | -0.52506 | 9.309932 |
| MMP25-AS | 0.46413 | 0.199135 | 0.982908 | 3.134526 |
| MB21D2 | 0.46424 | 0.199218 | 0.764331 | 3.428107 |
| SGPL1 | 0.464306 | 0.199315 | -0.58536 | 7.664166 |
| LIFR | 0.464306 | 0.199341 | -1.9237 | 1.188955 |
| VSTM2L | 0.464306 | 0.199371 | 1.931386 | 2.736276 |
| CCDC157 | 0.464306 | 0.199387 | -1.68091 | 1.426253 |
| ZNRF3-AS1 | 0.464563 | 0.199544 | 1.083196 | 2.260362 |
| PIMREG | 0.464563 | 0.199569 | 2.541711 | 0.769684 |
| WIPF2 | 0.46492 | 0.199794 | -0.45447 | 7.016755 |
| PRSS3 | 0.46492 | 0.199797 | -1.56211 | 1.20386 |
| KLHL24 | 0.46492 | 0.199828 | 0.526167 | 6.983521 |
| BRICD5 | 0.464963 | 0.199882 | 1.388096 | 1.55646 |
| MPHOSPH1 | 0.465414 | 0.200112 | -0.47662 | 6.960918 |
| SNORD21 | 0.465595 | 0.200237 | 0.758825 | 6.937891 |
| ARRDC1-AS | 0.465595 | 0.20026 | 1.401856 | 1.071506 |
| SNORD103 | 0.4656 | 0.200298 | 0.871691 | 3.493048 |
| PNMA1 | 0.465972 | 0.200502 | 0.543321 | 4.881739 |
| TMCO1-AS | 0.465972 | 0.200529 | 1.496825 | 1.218332 |
| LOC100289 | 0.466036 | 0.200592 | 1.008595 | 3.194468 |
| SMPD2 | 0.466127 | 0.200667 | -0.79456 | 3.453004 |
| TNFAIP6 | 0.466139 | 0.200708 | 1.919514 | 2.805596 |
| NEK9 | 0.466455 | 0.200899 | 0.528086 | 7.021764 |
| SLC10A7 | 0.466455 | 0.200938 | 1.417774 | 1.75864 |
| SNAP29 | 0.466455 | 0.20095 | -0.4637 | 5.888572 |
| PPP1R36 | 0.466474 | 0.201015 | 1.737996 | 1.836719 |
| ICE1 | 0.466474 | 0.201044 | 0.467773 | 6.845786 |
| HDHD2 | 0.466474 | 0.201076 | -0.52411 | 5.372382 |
| SPSB3 | 0.466474 | 0.201101 | -0.49116 | 5.654003 |
| TEPSIN | 0.466483 | 0.201146 | 0.62716 | 4.089565 |
| TBC1D9B | 0.466483 | 0.201176 | -1.3442 | 1.566763 |
| TTLL10 | 0.466539 | 0.201235 | -1.8003 | 0.934765 |
| PPP3R1 | 0.466564 | 0.201296 | -0.50011 | 7.18969 |
| FAM201A | 0.466564 | 0.201317 | 1.319699 | 2.05279 |
| FBXO7 | 0.466743 | 0.201458 | 1.295672 | 1.568121 |
| GNRH1 | 0.466743 | 0.201491 | -1.25231 | 2.026817 |
| GRHPR | 0.466743 | 0.201512 | -0.51834 | 5.306105 |
| GBA2 | 0.466743 | 0.201537 | 0.476105 | 6.068013 |
| RPE | 0.466852 | 0.201659 | 1.896101 | 0.612002 |
| FN3K | 0.466852 | 0.20167 | 1.002579 | 4.390815 |
| PSMA7 | 0.466852 | 0.201797 | -0.52508 | 6.738696 |

| | | | | |
|---|---|---|---|---|
| PIAS1 | 0.466852 | 0.201827 | -0.48383 | 6.552522 |
| LINC01481 | 0.466852 | 0.201847 | 1.207128 | 1.327726 |
| TMC8 | 0.466852 | 0.201857 | 0.87567 | 4.049915 |
| PLAGL1 | 0.466852 | 0.201874 | -0.95977 | 3.245505 |
| CHUK | 0.466852 | 0.201898 | 0.543756 | 5.418505 |
| MIR365B | 0.466852 | 0.201904 | 2.45663 | 0.291267 |
| TMEM26 | 0.46703 | 0.202017 | 1.829241 | 0.466171 |
| MAPKAP1 | 0.467032 | 0.202067 | 1.749843 | 0.535262 |
| NDUFA1 | 0.467032 | 0.202089 | -0.56448 | 5.678778 |
| MINK1 | 0.467061 | 0.202137 | -1.64877 | 0.795156 |
| S100A5 | 0.467097 | 0.202188 | 1.748363 | 0.609741 |
| CFAP44 | 0.467198 | 0.202267 | 0.592892 | 5.13005 |
| CEMP1 | 0.467347 | 0.202368 | -0.85249 | 2.66796 |
| FABP3 | 0.467484 | 0.202462 | 1.059474 | 3.171688 |
| CHD5 | 0.468313 | 0.202857 | 1.666732 | 2.755105 |
| SMA5 | 0.468393 | 0.202928 | 1.038895 | 1.776453 |
| YY1 | 0.46847 | 0.202997 | -0.4783 | 6.905309 |
| ORAI1 | 0.468495 | 0.203047 | 0.944538 | 2.103303 |
| ZNF765 | 0.468495 | 0.203094 | 0.565987 | 5.0389 |
| TLX1NB | 0.468495 | 0.20313 | -2.94293 | 0.622648 |
| THNSL2 | 0.468495 | 0.203181 | 1.670073 | 0.42388 |
| ARHGAP44 | 0.468495 | 0.203186 | 1.172852 | 3.325547 |
| ECHDC2 | 0.468624 | 0.203277 | -1.18981 | 2.022204 |
| SNORD113 | 0.468735 | 0.203361 | 1.694223 | 1.625932 |
| GZMA | 0.46879 | 0.203421 | 1.098245 | 2.772175 |
| COX6C | 0.469036 | 0.203597 | -0.58117 | 6.681237 |
| LOC101928 | 0.469036 | 0.203607 | -1.25806 | 2.755397 |
| VTRNA1-1 | 0.469036 | 0.203658 | 0.768899 | 7.81023 |
| FAM27E2 | 0.469036 | 0.203671 | 1.426868 | 1.391875 |
| NUDT17 | 0.4692 | 0.203782 | 1.018262 | 1.976385 |
| MPC1 | 0.4692 | 0.203828 | -0.71423 | 3.376444 |
| HIST1H3J | 0.4692 | 0.203849 | -1.01735 | 3.010672 |
| GTF2E1 | 0.469233 | 0.203899 | 0.651441 | 4.447556 |
| NAV2-AS4 | 0.469523 | 0.204061 | 1.629805 | 1.856031 |
| WASL | 0.469691 | 0.20417 | -0.49265 | 7.641432 |
| CAPN5 | 0.469749 | 0.204231 | -0.56991 | 6.168837 |
| CDH20 | 0.469849 | 0.204334 | 1.828405 | 0.917512 |
| SPTAN1 | 0.469849 | 0.204346 | -1.22302 | 1.59037 |
| SLC46A1 | 0.469889 | 0.204425 | 1.858264 | 0.794916 |
| ST20 | 0.469889 | 0.204435 | 1.575666 | 1.315394 |
| KDM4C | 0.470253 | 0.204658 | -0.74213 | 3.695629 |
| KDELR2 | 0.470253 | 0.204665 | 0.864186 | 2.673412 |
| MEGF6 | 0.470399 | 0.204765 | 0.580156 | 6.325582 |
| CTDSPL | 0.470614 | 0.204894 | 2.418933 | 0.532202 |
| RRM1 | 0.470656 | 0.204976 | -0.46161 | 6.539313 |

| | | | | |
|---|---|---|---|---|
| DCHS2 | 0.470656 | 0.205044 | 1.428208 | 3.812636 |
| CBARP | 0.470656 | 0.205051 | -1.0216 | 2.397995 |
| MRPL54 | 0.470656 | 0.205059 | -0.89143 | 3.571765 |
| TMEM254 | 0.470656 | 0.205092 | -1.01188 | 2.620416 |
| SIX5 | 0.470741 | 0.205165 | 0.78531 | 3.997835 |
| RNY4 | 0.471079 | 0.205373 | 0.807501 | 10.71029 |
| GON7 | 0.471079 | 0.205417 | -0.90473 | 2.670251 |
| UBXN2B | 0.471079 | 0.205449 | 0.503931 | 5.618859 |
| CLEC3B | 0.471079 | 0.205456 | 1.450369 | 1.481841 |
| EIF3I | 0.471079 | 0.205491 | -0.57635 | 6.679812 |
| SLC9C2 | 0.471233 | 0.205594 | 2.022859 | 0.413137 |
| UTP6 | 0.471361 | 0.20571 | 0.442151 | 6.357429 |
| COX11 | 0.471361 | 0.205757 | 0.678708 | 3.950181 |
| HMGN1 | 0.471361 | 0.205798 | -0.53196 | 6.876286 |
| GPCPD1 | 0.471361 | 0.205818 | -0.56122 | 6.977 |
| PPP1R35 | 0.471361 | 0.20583 | 0.789737 | 3.096178 |
| CDKAL1 | 0.471484 | 0.205947 | 0.47632 | 5.555711 |
| TTC5 | 0.471484 | 0.205955 | 0.744702 | 3.942343 |
| STARD5 | 0.471498 | 0.205998 | -0.73409 | 3.327082 |
| DGKB | 0.471516 | 0.206041 | 2.013661 | 1.538298 |
| TCF4 | 0.471977 | 0.206289 | 0.975206 | 2.828379 |
| DPY19L1P1 | 0.471977 | 0.206315 | 1.038959 | 2.850779 |
| CEP68 | 0.472535 | 0.206594 | 0.478943 | 6.062198 |
| OSGIN1 | 0.472854 | 0.20677 | -0.8392 | 3.033378 |
| MGC2889 | 0.473346 | 0.207021 | -1.54293 | 1.727929 |
| MOB1B | 0.473446 | 0.207101 | 1.066247 | 2.073947 |
| MRPL44 | 0.473487 | 0.207155 | -0.58137 | 4.734497 |
| HORMAD2 | 0.473735 | 0.2073 | -1.50077 | 1.247565 |
| ALOX12-AS | 0.473876 | 0.207397 | -1.85419 | 1.118395 |
| ZSCAN2 | 0.474078 | 0.207522 | 0.997419 | 3.206318 |
| NPIPB3 | 0.47418 | 0.207615 | 0.559789 | 4.907364 |
| DGCR6 | 0.47418 | 0.207675 | -2.04609 | 1.039551 |
| TMEM258 | 0.47418 | 0.207717 | -0.85961 | 3.46016 |
| PRADC1 | 0.47418 | 0.207719 | -1.00573 | 2.852646 |
| CRYL1 | 0.47418 | 0.207747 | -0.56053 | 4.625723 |
| AIM2 | 0.474182 | 0.207797 | 1.716011 | 1.184583 |
| CLTB | 0.474182 | 0.207821 | -1.18809 | 2.493632 |
| INSIG1 | 0.475193 | 0.2083 | -1.53183 | 1.831705 |
| POLN | 0.475195 | 0.208416 | -1.02179 | 2.692978 |
| MRS2 | 0.475195 | 0.208419 | 1.479572 | 1.327836 |
| LOC10012( | 0.475195 | 0.208441 | 1.727816 | 0.74979 |
| TSHZ3 | 0.475195 | 0.208509 | 0.637453 | 4.226616 |
| PMAIP1 | 0.475195 | 0.20851 | -0.73614 | 4.888472 |
| SHLD2P1 | 0.475195 | 0.208518 | 0.941621 | 2.508944 |
| SPACA4 | 0.475397 | 0.208643 | 1.448974 | 0.766576 |

| | | | | |
|---|---|---|---|---|
| MIR378H | 0.475874 | 0.208888 | 2.505205 | 0.649434 |
| JCHAIN | 0.475947 | 0.209026 | 1.10368 | 7.634403 |
| COX10 | 0.475947 | 0.20905 | -0.55421 | 4.420709 |
| TBX19 | 0.475947 | 0.209081 | -0.88529 | 2.736247 |
| CCDC191 | 0.475947 | 0.209095 | 0.674672 | 5.098191 |
| PLCE1-AS2 | 0.475947 | 0.209102 | 1.317944 | 1.749255 |
| NIPA1 | 0.476115 | 0.209229 | 1.227231 | 1.062143 |
| SHROOM4 | 0.476115 | 0.209251 | 1.121059 | 2.981585 |
| 1-Mar | 0.476115 | 0.209284 | 0.864489 | 3.830024 |
| RAB6B | 0.476138 | 0.209331 | 0.747823 | 3.412825 |
| ITGAE | 0.47616 | 0.209377 | -0.72372 | 3.80743 |
| CSNK1A1P1 | 0.476274 | 0.209464 | -1.5377 | 1.01967 |
| PATE3 | 0.476345 | 0.209531 | 2.120108 | 0.347803 |
| TK2 | 0.476434 | 0.20962 | 1.184132 | 1.654397 |
| ITGB7 | 0.476434 | 0.209642 | -1.05113 | 1.865658 |
| ZNF711 | 0.476516 | 0.209737 | 0.629122 | 4.181871 |
| TMEM14A | 0.476516 | 0.209751 | -0.76065 | 4.581994 |
| REPIN1 | 0.47662 | 0.209834 | 1.024854 | 1.908257 |
| PLEKHM2 | 0.476749 | 0.209927 | -0.46548 | 6.830481 |
| UBALD2 | 0.477476 | 0.210318 | -0.47392 | 6.069524 |
| ASB16 | 0.477476 | 0.210319 | 0.800451 | 3.21152 |
| ADAMTS18 | 0.47749 | 0.210362 | 1.583513 | 1.46436 |
| ZBTB11-AS | 0.477579 | 0.210437 | -1.0114 | 3.022378 |
| MIR941-1 | 0.477887 | 0.210652 | -1.27466 | 0.957601 |
| XKR8 | 0.477887 | 0.210698 | 0.745521 | 3.164651 |
| SCAP | 0.477887 | 0.210762 | -0.49786 | 6.245771 |
| TNRC6B | 0.477887 | 0.210781 | 0.673386 | 4.221264 |
| CECR7 | 0.477887 | 0.210784 | -1.12584 | 3.338947 |
| LOC100506 | 0.477887 | 0.210792 | 0.799849 | 2.412507 |
| THSD7B | 0.478193 | 0.210963 | -0.97947 | 3.403836 |
| ANP32B | 0.47871 | 0.211256 | -0.52322 | 8.655831 |
| LINC02245 | 0.47871 | 0.211264 | 2.415756 | 0.641366 |
| CCNY | 0.479 | 0.211429 | -1.82681 | 0.461016 |
| LINC00266 | 0.479015 | 0.211487 | -1.27123 | 1.479374 |
| E2F1 | 0.479015 | 0.211508 | 0.74433 | 3.552244 |
| NRAS | 0.479083 | 0.211575 | 0.717836 | 7.102722 |
| KRBA1 | 0.479385 | 0.211759 | 1.592043 | 0.786371 |
| UBE2D3 | 0.479385 | 0.211781 | -0.48295 | 5.319526 |
| HNRNPM | 0.47939 | 0.21182 | -1.78025 | 0.768058 |
| NDFIP1 | 0.479427 | 0.211873 | 0.563784 | 7.178647 |
| HRH2 | 0.47944 | 0.211936 | 1.896158 | 0.681637 |
| STARD13 | 0.47944 | 0.211952 | 1.22133 | 2.651301 |
| ZNF785 | 0.480575 | 0.212511 | 0.587662 | 4.146705 |
| DLX5 | 0.480575 | 0.212528 | -1.42861 | 1.734185 |
| GALK1 | 0.480575 | 0.212603 | -0.85107 | 3.181094 |

| | | | | |
|---|---|---|---|---|
| CPNE8 | 0.480575 | 0.212604 | -0.58384 | 4.936616 |
| MAT1A | 0.480575 | 0.212637 | 2.527014 | 0.355863 |
| ARL15 | 0.480691 | 0.212725 | -0.66494 | 4.007154 |
| MNDA | 0.480811 | 0.212834 | 1.220165 | 2.502673 |
| ZC3HAV1L | 0.480811 | 0.212851 | 0.635886 | 4.148133 |
| LYPLA2P2 | 0.480862 | 0.21291 | 1.689032 | 1.414819 |
| ITGA10 | 0.481056 | 0.213039 | -1.7125 | 0.433591 |
| ADAMTS3 | 0.481056 | 0.21307 | 1.440615 | 1.612911 |
| SENCR | 0.481233 | 0.213263 | 1.657854 | 0.79399 |
| TUBG1 | 0.481233 | 0.213284 | -0.52757 | 4.95993 |
| CATSPER3 | 0.481233 | 0.213286 | -0.80109 | 2.964149 |
| TDRP | 0.481233 | 0.213294 | -1.39892 | 1.339613 |
| CYP2W1 | 0.481303 | 0.213362 | 1.177967 | 1.566059 |
| LAPTM4A | 0.481387 | 0.213436 | -0.48655 | 7.479599 |
| LINC01011 | 0.481569 | 0.213554 | 1.45352 | 0.865006 |
| MTRNR2L3 | 0.481873 | 0.213725 | -1.29155 | 2.38909 |
| PIK3CD | 0.482134 | 0.213902 | 0.566014 | 4.817605 |
| SH2B2 | 0.482134 | 0.213928 | 1.363631 | 1.208792 |
| LOC101927 | 0.482134 | 0.213951 | 1.293276 | 1.956844 |
| ANAPC1P1 | 0.482686 | 0.214233 | -0.87029 | 2.150131 |
| CARHSP1 | 0.483034 | 0.214424 | -1.12219 | 2.095471 |
| PDS5B | 0.48318 | 0.214526 | 0.489288 | 6.807776 |
| GRID2IP | 0.483197 | 0.21457 | 0.803079 | 3.814454 |
| PHF23 | 0.483375 | 0.21471 | -1.08677 | 1.771813 |
| BRINP1 | 0.483375 | 0.214723 | -1.30203 | 1.706072 |
| LAMC3 | 0.483562 | 0.214843 | -0.94425 | 3.267744 |
| MBD5 | 0.483721 | 0.214957 | 0.492959 | 5.415252 |
| CPNE2 | 0.483721 | 0.214987 | -0.58948 | 4.461464 |
| RNMT | 0.483961 | 0.215131 | 0.599545 | 4.32602 |
| LOC101928 | 0.483969 | 0.215171 | -1.19919 | 1.56505 |
| ADCK5 | 0.484171 | 0.215298 | 0.783444 | 3.658623 |
| MIR548AR | 0.484477 | 0.215471 | 1.071682 | 1.765771 |
| PPM1K | 0.484566 | 0.215547 | -0.60901 | 5.656778 |
| MYO1E | 0.484624 | 0.215613 | 0.558676 | 8.227169 |
| FEZ2 | 0.484624 | 0.215647 | 1.87161 | 0.425659 |
| SLC16A8 | 0.484991 | 0.215847 | 1.087206 | 2.557383 |
| SNORD7 | 0.485152 | 0.215956 | 1.739971 | 0.943763 |
| SLC20A1 | 0.485449 | 0.216125 | 0.722698 | 7.175206 |
| PMM2 | 0.485965 | 0.216392 | 0.456532 | 6.495874 |
| HOXD1 | 0.486104 | 0.216496 | -1.5756 | 0.948763 |
| SNN | 0.486104 | 0.216534 | -0.58056 | 4.363813 |
| CABLES2 | 0.486104 | 0.216565 | 0.880902 | 3.685446 |
| VAPB | 0.48628 | 0.216715 | -0.7088 | 3.42259 |
| KPNA7 | 0.48628 | 0.216717 | 1.228097 | 2.033432 |
| FAM8A1 | 0.486411 | 0.216813 | 0.482156 | 5.978362 |

| | | | | |
|---|---|---|---|---|
| ZNF879 | 0.486655 | 0.216959 | -1.10692 | 2.278621 |
| DNAJA1 | 0.486946 | 0.217131 | 0.776416 | 3.942999 |
| EEF1G | 0.486946 | 0.217163 | -0.57261 | 9.285064 |
| SNORA64 | 0.487469 | 0.217433 | -0.52952 | 7.567534 |
| LINC01426 | 0.487753 | 0.217612 | 1.576979 | 0.713632 |
| CRB3 | 0.487753 | 0.217634 | -1.47329 | 0.936402 |
| HSBP1L1 | 0.487762 | 0.217708 | -0.73958 | 3.473788 |
| RNVU1-1 | 0.487762 | 0.217747 | -1.12567 | 1.912594 |
| ATF7IP | 0.487762 | 0.217751 | 0.966872 | 3.100758 |
| APIP | 0.487762 | 0.217787 | 0.633525 | 3.598535 |
| MRPL40 | 0.487788 | 0.217869 | -0.61896 | 4.347344 |
| RNPEP | 0.487788 | 0.217873 | -0.51887 | 6.62025 |
| ZFP69B | 0.487856 | 0.21794 | 0.898404 | 2.944646 |
| BCL3 | 0.487953 | 0.218021 | -0.59377 | 5.533404 |
| ACTN4 | 0.487976 | 0.218068 | -0.46698 | 9.743493 |
| ERCC8 | 0.488067 | 0.218146 | 1.527435 | 1.176511 |
| POLR3K | 0.48813 | 0.218212 | 0.748357 | 3.46489 |
| CD5 | 0.488191 | 0.2183 | 0.812646 | 3.96844 |
| EXOC3 | 0.488191 | 0.218382 | -0.46971 | 7.117691 |
| HIP1R | 0.488191 | 0.218407 | -0.57884 | 5.696759 |
| SALRNA3 | 0.488191 | 0.218458 | 2.002302 | 0.533332 |
| AHCYL1 | 0.488191 | 0.21851 | 1.241137 | 1.906404 |
| ZNF789 | 0.488191 | 0.218514 | 0.66217 | 3.784335 |
| GATB | 0.488191 | 0.218552 | -0.65163 | 4.065179 |
| AFDN-DT | 0.488191 | 0.218567 | -0.86583 | 2.796265 |
| RNF144A-A | 0.488191 | 0.218574 | 1.433017 | 0.899684 |
| ANP32AP1 | 0.488216 | 0.218622 | 1.332713 | 1.308975 |
| MBTPS1 | 0.488264 | 0.218694 | 0.460657 | 7.34976 |
| FGD2 | 0.488264 | 0.218718 | 0.761455 | 4.036242 |
| SF3B5 | 0.488525 | 0.218872 | -0.77615 | 4.461273 |
| NFE2L1 | 0.488943 | 0.219097 | 0.436416 | 8.421011 |
| SHLD2 | 0.489119 | 0.219239 | 0.491606 | 6.027774 |
| LOC646903 | 0.489119 | 0.21925 | 1.443365 | 1.187568 |
| GHITM | 0.4892 | 0.219324 | -0.53 | 7.359265 |
| RTCA | 0.489284 | 0.219399 | 1.913315 | 0.628594 |
| KCNAB2 | 0.489345 | 0.219489 | 1.495277 | 1.28278 |
| CATSPER4 | 0.489345 | 0.2195 | 2.412943 | 0.489662 |
| ARID2 | 0.489403 | 0.219564 | -0.44601 | 7.41638 |
| STAG3L5P- | 0.489637 | 0.219706 | 1.110037 | 1.609636 |
| COQ4 | 0.489795 | 0.219814 | -0.78173 | 3.089983 |
| TTC30B | 0.489938 | 0.219919 | 1.012765 | 2.403729 |
| SULT1A1 | 0.489938 | 0.219953 | 1.587497 | 0.862317 |
| USP13 | 0.489983 | 0.220038 | 0.536834 | 5.065936 |
| GARS | 0.489983 | 0.220048 | 1.456172 | 1.310921 |
| C9orf152 | 0.490029 | 0.220106 | 1.003252 | 4.654978 |

| | | | | |
|---|---|---|---|---|
| PLCB2 | 0.490032 | 0.220152 | 1.596896 | 0.503299 |
| SRC | 0.490032 | 0.220182 | 1.029218 | 1.266762 |
| RBM5-AS1 | 0.490039 | 0.220245 | -0.53159 | 4.962196 |
| AMY2B | 0.490039 | 0.22026 | 0.747981 | 3.922266 |
| VWCE | 0.490119 | 0.220333 | 0.980571 | 2.398924 |
| NGRN | 0.490321 | 0.220461 | 1.025639 | 2.312676 |
| AHCTF1 | 0.490672 | 0.220665 | 0.465201 | 7.365594 |
| MAGOH | 0.490672 | 0.220734 | 0.562358 | 4.599389 |
| MAGI1-AS1 | 0.490672 | 0.22075 | 0.90886 | 4.123067 |
| HTR2B | 0.490672 | 0.220769 | 0.718943 | 3.593845 |
| TUSC1 | 0.490914 | 0.220915 | -0.82842 | 2.914038 |
| KCNK3 | 0.491302 | 0.221127 | 1.190617 | 1.828043 |
| MAK | 0.491609 | 0.221303 | 1.33635 | 2.042655 |
| SNORD96A | 0.491933 | 0.221486 | 0.798303 | 4.000798 |
| PSMG4 | 0.492419 | 0.22182 | -1.71701 | 0.563969 |
| BRWD1-AS | 0.492419 | 0.221856 | 1.875111 | 0.498373 |
| KIF14 | 0.492419 | 0.221888 | 1.653298 | 0.823329 |
| EDAR | 0.492419 | 0.221891 | 0.591112 | 5.048722 |
| SNORD35A | 0.492419 | 0.221893 | 0.679663 | 5.479632 |
| CARNMT1 | 0.492551 | 0.22199 | -0.51637 | 5.388378 |
| TTC39A-AS | 0.492847 | 0.222161 | -1.45514 | 1.36328 |
| SLC24A1 | 0.49324 | 0.222389 | 1.080499 | 1.602931 |
| ZC3H13 | 0.49324 | 0.222413 | 0.483779 | 8.778049 |
| AHSA2P | 0.493567 | 0.222598 | 0.504374 | 6.609237 |
| PSPC1 | 0.494023 | 0.222841 | 0.509266 | 5.131582 |
| NKX2-3 | 0.494036 | 0.222885 | -2.17119 | 0.758025 |
| CAPN10-DT | 0.494208 | 0.223022 | 0.800405 | 3.427941 |
| SNAI1 | 0.494208 | 0.223067 | 1.501291 | 1.406959 |
| TPGS1 | 0.494208 | 0.223075 | 0.796688 | 2.656976 |
| IP6K1 | 0.49452 | 0.223254 | 1.052896 | 2.199913 |
| RPS4Y1 | 0.49463 | 0.223341 | -1.45356 | 7.036266 |
| FA2H | 0.494706 | 0.223413 | 0.786895 | 4.553863 |
| CCDC28B | 0.494944 | 0.223559 | 1.488057 | 1.478487 |
| LSM7 | 0.494976 | 0.223611 | 0.59343 | 3.94601 |
| PRSS12 | 0.495252 | 0.223773 | 0.650418 | 4.291096 |
| RFFL | 0.495426 | 0.223889 | -1.26806 | 1.470493 |
| PLEKHA4 | 0.495511 | 0.223965 | 1.746924 | 0.725385 |
| MIR421 | 0.495581 | 0.224039 | 1.040095 | 1.992477 |
| TMEM132E | 0.495581 | 0.224072 | 1.821786 | 0.725097 |
| ELOB | 0.495613 | 0.224125 | -1.87661 | 1.230728 |
| SNORA3B | 0.495772 | 0.224235 | 0.586099 | 8.233259 |
| MRPS11 | 0.496126 | 0.224432 | -1.38483 | 0.946785 |
| DMRTA1 | 0.496148 | 0.22448 | -1.39369 | 1.848974 |
| CD4 | 0.496218 | 0.224609 | 1.470106 | 1.116564 |
| LINC01235 | 0.496218 | 0.224612 | 1.563122 | 0.841718 |

| | | | | |
|---|---|---|---|---|
| ZFYVE19 | 0.496218 | 0.224646 | -1.30099 | 1.245712 |
| DOP1A | 0.496218 | 0.224663 | 1.492893 | 0.441709 |
| PTOV1-AS2 | 0.496428 | 0.224801 | 0.558392 | 4.129206 |
| CYP4F12 | 0.496428 | 0.224834 | 1.846498 | 0.724204 |
| GALNT4 | 0.496525 | 0.224963 | 1.660803 | 1.8435 |
| LOC101928 | 0.496525 | 0.224976 | 1.748745 | 0.649936 |
| PAK1IP1 | 0.496525 | 0.225011 | -0.58297 | 5.020536 |
| EFCAB14-A | 0.496525 | 0.225029 | -1.88049 | 0.922808 |
| MPC2 | 0.497189 | 0.225392 | -1.26771 | 1.324861 |
| ZNF329 | 0.497189 | 0.225406 | -0.73008 | 3.307008 |
| GALNT5 | 0.497361 | 0.225537 | 0.569741 | 6.36333 |
| GPRC5D-AS | 0.497361 | 0.225563 | -0.74268 | 3.012532 |
| NDUFS7 | 0.497361 | 0.225598 | -0.57704 | 4.214956 |
| AKT1 | 0.497415 | 0.22571 | 1.376154 | 1.071067 |
| LGALS9B | 0.497415 | 0.225717 | -1.27316 | 2.256175 |
| RNU11 | 0.497415 | 0.225736 | 0.648573 | 9.179139 |
| GABPB1-IT | 0.497596 | 0.225886 | 0.533684 | 4.411225 |
| NSD2 | 0.497596 | 0.225912 | -0.4591 | 5.329774 |
| TBL3 | 0.497596 | 0.225932 | -0.5307 | 4.704932 |
| PM20D2 | 0.497647 | 0.226028 | 0.444912 | 5.998687 |
| STX17 | 0.497647 | 0.226031 | -0.49588 | 6.933686 |
| CWC27 | 0.497685 | 0.226086 | -0.70814 | 3.608138 |
| STX18-IT1 | 0.497723 | 0.226141 | 1.053279 | 1.618508 |
| LPP | 0.498056 | 0.22633 | 0.62636 | 4.15849 |
| LOC389765 | 0.498159 | 0.22646 | 0.610996 | 4.177224 |
| LINC00299 | 0.498159 | 0.226469 | 1.527052 | 0.581757 |
| DPH7 | 0.498159 | 0.226561 | 0.600516 | 3.871176 |
| GOT1L1 | 0.498159 | 0.226562 | -2.48746 | 0.560896 |
| ZNF718 | 0.498159 | 0.226623 | -0.98358 | 1.776514 |
| CACHD1 | 0.498159 | 0.226636 | 1.418406 | 0.841986 |
| TRAPPC4 | 0.498159 | 0.226643 | 0.652607 | 3.3113 |
| CENPH | 0.498455 | 0.226816 | 0.673712 | 3.578764 |
| LINC01530 | 0.4985 | 0.226874 | 1.504848 | 0.680663 |
| C1orf56 | 0.498583 | 0.22695 | -0.69136 | 3.121201 |
| UBE2C | 0.498935 | 0.227177 | 1.209861 | 1.917895 |
| APOL1 | 0.498935 | 0.227186 | -1.96067 | 1.703087 |
| SAMD8 | 0.499238 | 0.227386 | -1.66296 | 0.68278 |
| APBA3 | 0.499238 | 0.2274 | 0.614093 | 3.999492 |
| SLC25A44 | 0.499361 | 0.227494 | 0.974011 | 2.506676 |
| KCTD19 | 0.499501 | 0.227596 | 1.582458 | 0.648066 |
| TMEM74B | 0.499546 | 0.227666 | 1.819246 | 1.106503 |
| FAXDC2 | 0.499546 | 0.227693 | 0.646417 | 4.80857 |
| ARHGAP29 | 0.499835 | 0.227863 | 0.55927 | 5.288223 |
| MS4A8 | 0.500528 | 0.228217 | 1.866248 | 1.1999 |
| SPRY4-IT1 | 0.501141 | 0.228576 | 1.328458 | 1.5627 |

| | | | | |
|---|---|---|---|---|
| TIPRL | 0.501141 | 0.228596 | -0.55007 | 4.728568 |
| HLA-DPB1 | 0.501141 | 0.228673 | 1.078304 | 3.994214 |
| INTS1 | 0.501141 | 0.228681 | 0.46912 | 7.305284 |
| DNAL4 | 0.501141 | 0.228687 | -0.62593 | 3.247211 |
| ZNRD1ASP | 0.501547 | 0.228922 | 0.881471 | 3.166991 |
| SUCLG2 | 0.501547 | 0.228949 | -0.48115 | 5.134873 |
| E2F7 | 0.501708 | 0.229095 | 0.744978 | 3.763341 |
| ZNF777 | 0.501708 | 0.22913 | 0.824207 | 4.608502 |
| GSS | 0.501708 | 0.229162 | -0.53899 | 5.194056 |
| PRIM1 | 0.501708 | 0.229175 | 0.71738 | 3.548331 |
| NKX2-8 | 0.50174 | 0.229228 | 2.330372 | 0.418389 |
| CATSPERG | 0.501783 | 0.229322 | 0.797725 | 3.773137 |
| CWF19L2 | 0.501783 | 0.229324 | 0.44474 | 6.037278 |
| SDHB | 0.502032 | 0.229476 | -0.48332 | 5.644282 |
| KPNA6 | 0.50267 | 0.229806 | -0.42091 | 7.684539 |
| TMEM208 | 0.502812 | 0.229909 | 0.594957 | 4.080244 |
| RORB | 0.503068 | 0.230065 | 1.307263 | 2.194279 |
| ZNF732 | 0.503156 | 0.230143 | -1.36165 | 1.62419 |
| CAPZB | 0.50354 | 0.230357 | 0.58178 | 3.945974 |
| TMEM269 | 0.503546 | 0.23042 | 1.454364 | 0.710387 |
| PKIA-AS1 | 0.503546 | 0.230488 | -1.29406 | 2.084388 |
| RASSF5 | 0.503546 | 0.230498 | -0.97615 | 2.363725 |
| SRRM4 | 0.503546 | 0.230514 | -1.4744 | 2.07063 |
| CTNNA1 | 0.503571 | 0.230563 | -1.17631 | 2.400721 |
| ST3GAL6 | 0.503707 | 0.230727 | 2.106027 | 0.651844 |
| TMEM158 | 0.503707 | 0.230731 | 1.350828 | 2.590097 |
| MFSD3 | 0.503707 | 0.230741 | 2.082942 | 0.632605 |
| ZNF446 | 0.503938 | 0.2309 | 1.089891 | 2.647832 |
| TEX22 | 0.503938 | 0.230924 | -1.20334 | 1.736079 |
| WARS2-AS: | 0.504166 | 0.23108 | 0.777284 | 3.804769 |
| ZNF391 | 0.504166 | 0.231105 | 1.284602 | 1.49631 |
| U2SURP | 0.504333 | 0.23122 | 0.443176 | 8.117115 |
| ZSWIM8-A: | 0.504375 | 0.231284 | -0.72238 | 3.269279 |
| LINC01082 | 0.504375 | 0.231341 | 1.709923 | 0.951507 |
| HLA-DRB5 | 0.504375 | 0.231354 | 1.528559 | 4.519666 |
| C2orf76 | 0.504538 | 0.231468 | -0.7861 | 2.572531 |
| CSF2RB | 0.50466 | 0.231565 | 0.792924 | 4.08397 |
| HUWE1 | 0.50466 | 0.231601 | -0.42152 | 9.91931 |
| RPL9 | 0.50485 | 0.231726 | -1.48224 | 0.702172 |
| SATB1 | 0.50532 | 0.232017 | 1.58443 | 0.442676 |
| KAZN | 0.50532 | 0.232019 | -0.94292 | 2.862828 |
| SNED1 | 0.505771 | 0.232265 | 0.485834 | 5.800268 |
| KIAA0087 | 0.506278 | 0.232631 | 2.049499 | 0.414747 |
| COG1 | 0.506278 | 0.232632 | 0.506777 | 5.270717 |
| RTN4RL2 | 0.506278 | 0.23264 | 1.3688 | 1.558416 |

| | | | | |
|---|---|---|---|---|
| ZNF706 | 0.506278 | 0.232652 | -1.15747 | 2.241882 |
| EEF1A2 | 0.506476 | 0.232781 | -1.40404 | 2.760585 |
| ZNF818P | 0.506502 | 0.232832 | -0.90141 | 3.235548 |
| MORC2 | 0.506697 | 0.23296 | 0.871016 | 2.902997 |
| NCKAP5-AS | 0.506736 | 0.233024 | -1.64964 | 1.122357 |
| WWC2 | 0.506736 | 0.233087 | 0.47421 | 6.440994 |
| DHRSX | 0.506736 | 0.233094 | 1.563437 | 1.222018 |
| PANK2 | 0.507175 | 0.233346 | -0.76358 | 2.551068 |
| LSP1P4 | 0.507175 | 0.233373 | 0.77941 | 2.71373 |
| SLC25A38 | 0.507446 | 0.233536 | 0.720752 | 4.273736 |
| PCBP1-AS1 | 0.507988 | 0.233825 | 0.677487 | 3.405967 |
| ANXA2 | 0.508435 | 0.234069 | -0.7548 | 4.199328 |
| TRAPPC13 | 0.509041 | 0.234387 | 1.253972 | 1.850033 |
| ADGB | 0.509041 | 0.234429 | 1.964141 | 0.582149 |
| BHLHA15 | 0.509041 | 0.23448 | -1.20709 | 2.293407 |
| KNDC1 | 0.509041 | 0.234503 | 1.800211 | 1.746874 |
| DDX27 | 0.509051 | 0.234547 | 0.442998 | 7.221067 |
| ANKRD19P | 0.509184 | 0.234647 | 0.794517 | 2.633653 |
| ELF1 | 0.509271 | 0.234726 | 1.27116 | 1.348145 |
| IDO1 | 0.509411 | 0.234829 | 1.249116 | 2.384281 |
| GLS2 | 0.509687 | 0.234995 | 2.471346 | 0.446852 |
| SCIN | 0.509867 | 0.235117 | -1.30808 | 1.869324 |
| DOK7 | 0.50997 | 0.235239 | 1.362492 | 0.801031 |
| YBX2 | 0.50997 | 0.235263 | 0.961019 | 2.787844 |
| RNF146 | 0.50997 | 0.235281 | 1.347645 | 1.50807 |
| TTYH2 | 0.510026 | 0.235346 | 1.068473 | 1.575777 |
| KCTD17 | 0.510508 | 0.235607 | 1.507288 | 0.826707 |
| ISL1 | 0.510669 | 0.23572 | 1.880393 | 2.450801 |
| ALG10B | 0.510729 | 0.235849 | 0.729854 | 3.655604 |
| EIF3E | 0.510729 | 0.235857 | -0.50996 | 8.008156 |
| CPZ | 0.510729 | 0.235884 | -1.77662 | 0.780398 |
| DDRGK1 | 0.510729 | 0.23591 | 0.598017 | 6.359424 |
| TSR1 | 0.510729 | 0.235944 | -0.47422 | 6.529972 |
| MCOLN2 | 0.510729 | 0.23603 | 1.1528 | 2.603368 |
| AQP7P3 | 0.510729 | 0.236038 | 1.283126 | 1.725563 |
| LINC01521 | 0.510729 | 0.236091 | 0.95821 | 2.065854 |
| SGTA | 0.510729 | 0.236098 | 0.428705 | 5.912205 |
| RPL19 | 0.510815 | 0.236177 | -0.47824 | 9.656467 |
| FAM110B | 0.510965 | 0.236285 | 1.143053 | 2.53794 |
| MAPRE1 | 0.511206 | 0.236436 | 0.441432 | 7.214845 |
| SIRT7 | 0.511425 | 0.236576 | -0.50128 | 5.693745 |
| ACBD3 | 0.51146 | 0.236631 | -0.44078 | 8.279005 |
| MRM2 | 0.51175 | 0.236804 | 0.488569 | 4.950519 |
| POLR2J | 0.512091 | 0.237001 | -0.509 | 5.01844 |
| FBXO2 | 0.512182 | 0.237082 | 0.77697 | 2.801011 |

| | | | | |
|---|---|---|---|---|
| ARL6IP5 | 0.512356 | 0.237202 | 0.511833 | 6.045782 |
| ERBB3 | 0.512616 | 0.237361 | -0.45721 | 8.693597 |
| NAB2 | 0.512726 | 0.237451 | -0.4654 | 5.534854 |
| GBP2 | 0.51281 | 0.237529 | 0.560621 | 7.120411 |
| RHOG | 0.512924 | 0.237675 | -0.5194 | 4.94219 |
| MPPED2 | 0.512924 | 0.237699 | -1.24175 | 2.078507 |
| NT5M | 0.512924 | 0.237699 | 1.455842 | 1.057766 |
| OTUD7B | 0.513049 | 0.237796 | 0.437834 | 6.851955 |
| CHI3L1 | 0.513071 | 0.237846 | 1.198711 | 5.248324 |
| SCN7A | 0.513921 | 0.238317 | 2.062045 | 0.535122 |
| CCDC9 | 0.513921 | 0.238318 | 0.511044 | 6.339689 |
| WDR4 | 0.513988 | 0.238421 | 1.730634 | 2.052317 |
| CSRNP2 | 0.513988 | 0.238427 | -1.72644 | 0.710332 |
| P2RY11 | 0.514042 | 0.238491 | -1.2214 | 1.507246 |
| MANF | 0.514124 | 0.238569 | -0.53129 | 5.586524 |
| COPS9 | 0.514369 | 0.238722 | -1.22361 | 2.052275 |
| TVP23C | 0.514431 | 0.23879 | -0.44568 | 5.753685 |
| RLF | 0.514751 | 0.23899 | -0.46516 | 7.120457 |
| MED1 | 0.514751 | 0.239032 | 0.415318 | 7.323315 |
| MFSD14B | 0.514751 | 0.239081 | -0.44704 | 6.529022 |
| ABHD2 | 0.514751 | 0.239133 | 0.97694 | 3.125611 |
| XPNPEP2 | 0.514751 | 0.239134 | 1.465131 | 1.45882 |
| LINC01963 | 0.514983 | 0.239315 | 0.755646 | 3.467679 |
| USPL1 | 0.514983 | 0.23932 | 0.478458 | 5.457129 |
| BAZ1A | 0.515025 | 0.239394 | -1.55625 | 0.560953 |
| AKAP10 | 0.515025 | 0.239448 | -0.42918 | 6.187144 |
| PAPOLG | 0.515025 | 0.239476 | 0.418302 | 5.764986 |
| CSNK2A2 | 0.515025 | 0.239522 | -0.43263 | 5.551244 |
| POU4F1 | 0.515025 | 0.23955 | 1.792693 | 0.669914 |
| UQCRHL | 0.515025 | 0.239576 | -0.55122 | 4.906612 |
| WDR26 | 0.515232 | 0.239734 | -1.91862 | 0.528126 |
| ATP1A1 | 0.515232 | 0.239774 | 0.706189 | 4.697934 |
| MIS18BP1 | 0.515232 | 0.23979 | 0.465559 | 6.231804 |
| LOC101927 | 0.515372 | 0.239894 | -1.02756 | 3.013859 |
| ATF6 | 0.515544 | 0.240059 | 0.4657 | 6.984261 |
| ANKRD34A | 0.515544 | 0.24011 | 1.227088 | 1.680016 |
| AGAP4 | 0.515544 | 0.240119 | 1.012712 | 2.097786 |
| KCNB2 | 0.515544 | 0.240168 | 1.899838 | 0.896899 |
| DKK2 | 0.515544 | 0.240171 | 1.770431 | 1.415427 |
| ARHGAP26 | 0.515672 | 0.24027 | 1.364427 | 1.637975 |
| MRPL48 | 0.515789 | 0.240402 | 0.591366 | 4.44199 |
| GTF2IRD2 | 0.515789 | 0.240403 | -1.12551 | 1.730742 |
| MGA | 0.515922 | 0.240504 | 1.233514 | 1.215514 |
| HOXD4 | 0.515935 | 0.240549 | 1.545577 | 0.64867 |
| TRIM52 | 0.516267 | 0.240744 | 0.56046 | 4.718082 |

| | | | | |
|---|---|---|---|---|
| PDCL3 | 0.516552 | 0.240916 | -0.52899 | 5.263055 |
| KNTC1 | 0.516734 | 0.241114 | 0.454995 | 6.325163 |
| TAF4 | 0.516734 | 0.241116 | 0.530777 | 4.596001 |
| IMP4 | 0.516734 | 0.241119 | 0.532383 | 4.671943 |
| PLPP3 | 0.516825 | 0.241201 | 0.527322 | 5.534372 |
| REREP3 | 0.516936 | 0.241306 | 1.277753 | 1.971689 |
| UGT1A1 | 0.516936 | 0.241331 | -1.03375 | 2.236411 |
| RHEB | 0.517043 | 0.241448 | 0.546742 | 4.860996 |
| EXOSC7 | 0.517043 | 0.241499 | -0.96909 | 2.774163 |
| GNPTG | 0.517043 | 0.241499 | 0.593953 | 4.984514 |
| MEF2C-AS1 | 0.517117 | 0.241608 | 1.492469 | 0.720299 |
| DBF4 | 0.517117 | 0.241613 | 0.556846 | 4.637831 |
| GIPC3 | 0.517362 | 0.241767 | 1.111303 | 1.823622 |
| RPTN | 0.517405 | 0.241865 | -1.3661 | 4.094955 |
| ABL2 | 0.517405 | 0.241866 | 1.526152 | 0.503046 |
| HAS2-AS1 | 0.517899 | 0.242136 | 1.901663 | 0.861776 |
| LSM3 | 0.518057 | 0.242266 | -0.51447 | 5.708569 |
| CCDC24 | 0.518057 | 0.242318 | 0.578058 | 3.725813 |
| SPACA6 | 0.518057 | 0.242329 | 0.692076 | 4.40613 |
| MIR4720 | 0.518109 | 0.242392 | -1.55499 | 0.595845 |
| C1orf116 | 0.51833 | 0.242535 | -1.02797 | 1.929764 |
| ELAVL1 | 0.5184 | 0.242607 | 0.416269 | 5.933926 |
| NKAP | 0.518483 | 0.24272 | 0.458027 | 5.021858 |
| GPR65 | 0.518483 | 0.242725 | 1.014475 | 2.068784 |
| PDIA3P1 | 0.518506 | 0.242776 | 0.475515 | 5.895168 |
| LOC101927 | 0.519085 | 0.243086 | -1.65346 | 0.606951 |
| TEDDM1 | 0.519232 | 0.243195 | 1.371799 | 1.442593 |
| NTF4 | 0.519494 | 0.243428 | -1.38169 | 1.320159 |
| TRIO | 0.519494 | 0.24344 | 1.037355 | 2.218265 |
| LOC100996 | 0.519494 | 0.243474 | -1.39732 | 1.393431 |
| MIR548U | 0.519494 | 0.243481 | 1.198597 | 1.305583 |
| GOLPH3L | 0.519494 | 0.243515 | -0.43614 | 6.325904 |
| TEN1-CDK3 | 0.520105 | 0.243845 | -1.42454 | 0.752124 |
| MACF1 | 0.520105 | 0.243913 | 0.430226 | 10.42808 |
| ZG16B | 0.520105 | 0.243921 | 1.63773 | 1.257918 |
| SETMAR | 0.520237 | 0.244073 | -1.1226 | 2.18086 |
| LOC101929 | 0.520237 | 0.244079 | -0.6868 | 3.033906 |
| CYB5B | 0.520237 | 0.244141 | 0.516157 | 6.029138 |
| NEMF | 0.520237 | 0.24417 | -1.3352 | 1.274834 |
| ZNF224 | 0.520237 | 0.244181 | 0.779476 | 2.549708 |
| GJB3 | 0.520339 | 0.244339 | -1.26743 | 1.848381 |
| C5orf17 | 0.520339 | 0.244342 | -1.89778 | 1.234451 |
| PRKAB1 | 0.520339 | 0.244364 | -0.47446 | 5.621074 |
| SCARNA1 | 0.520339 | 0.244387 | -0.54602 | 5.264482 |
| SLFN12 | 0.521162 | 0.244814 | 1.538707 | 0.723476 |

| | | | | |
|---|---|---|---|---|
| SCN4A | 0.521263 | 0.244901 | 1.43835 | 0.768834 |
| TDGF1P3 | 0.521375 | 0.244993 | 2.191797 | 0.904226 |
| ERCC1 | 0.521615 | 0.245145 | 1.219352 | 1.324561 |
| CCDC85A | 0.521634 | 0.245194 | 1.562424 | 1.294938 |
| KBTBD6 | 0.521635 | 0.245235 | 0.787782 | 3.579809 |
| MSH5-SAP( | 0.522148 | 0.245549 | 1.319294 | 0.782512 |
| MED12 | 0.522148 | 0.245568 | 0.401127 | 6.889871 |
| SNRNP200 | 0.522148 | 0.245595 | 0.428775 | 8.921301 |
| ZNF25 | 0.522214 | 0.245666 | 0.549761 | 4.612893 |
| NAGLU | 0.522219 | 0.245708 | 0.539453 | 4.283672 |
| LRRC37A6F | 0.522748 | 0.245997 | 1.414863 | 1.841894 |
| WDR53 | 0.5229 | 0.246108 | -0.79405 | 3.393163 |
| TMEM161A | 0.523133 | 0.246262 | 1.487029 | 0.620279 |
| C5orf63 | 0.523133 | 0.246297 | 0.685048 | 4.234861 |
| GOLM1 | 0.523316 | 0.24645 | 2.148827 | 0.514914 |
| CDYL2 | 0.523316 | 0.246497 | 0.672005 | 3.568532 |
| INA | 0.523316 | 0.246531 | -1.4034 | 1.353408 |
| COX6B2 | 0.523316 | 0.246619 | 1.27245 | 2.238277 |
| DIRAS3 | 0.523316 | 0.246634 | -0.91742 | 2.919627 |
| LOC101928 | 0.523316 | 0.246721 | -1.56182 | 0.407538 |
| UNC50 | 0.523316 | 0.246722 | 0.482045 | 4.958911 |
| LINC01504 | 0.523316 | 0.24674 | 1.351567 | 0.822668 |
| TTPAL | 0.523316 | 0.246743 | -2.22472 | 0.557328 |
| ZNF19 | 0.523519 | 0.246878 | 1.233161 | 2.516805 |
| VNN1 | 0.523527 | 0.246922 | 1.408983 | 2.300999 |
| TPM3P9 | 0.523567 | 0.24698 | 0.542609 | 4.856665 |
| ALX3 | 0.523674 | 0.247071 | -0.80391 | 2.558005 |
| SNORA104 | 0.523704 | 0.247125 | 1.285618 | 1.978988 |
| TOMM34 | 0.523711 | 0.247213 | 0.529324 | 4.378242 |
| SNORD116 | 0.523711 | 0.24723 | 1.818617 | 0.799387 |
| ZNF197 | 0.523711 | 0.247259 | 0.499455 | 5.190259 |
| FUZ | 0.523711 | 0.247288 | -1.69792 | 0.622597 |
| FAM228A | 0.523886 | 0.247411 | 1.382421 | 0.408785 |
| RC3H2 | 0.523925 | 0.247469 | -0.59457 | 4.258273 |
| HNRNPA1 | 0.524192 | 0.247635 | -0.62537 | 3.925013 |
| MXRA7 | 0.524913 | 0.248019 | 0.755608 | 4.256516 |
| S100B | 0.524913 | 0.248118 | -1.04211 | 1.488088 |
| CCDC15 | 0.524913 | 0.248138 | -0.65064 | 4.237935 |
| ZSCAN22 | 0.524913 | 0.248141 | -0.58196 | 3.871397 |
| THNSL1 | 0.524913 | 0.248175 | 1.02696 | 2.488577 |
| ATG2A | 0.525091 | 0.2483 | 0.412188 | 5.992682 |
| TMEM260 | 0.525093 | 0.248341 | 0.480363 | 5.046744 |
| KIF5A | 0.525204 | 0.248435 | 1.34059 | 1.777303 |
| IGIP | 0.525204 | 0.248473 | 0.66174 | 4.660684 |
| SNORD91B | 0.525432 | 0.248621 | 0.72435 | 5.912843 |

| | | | | |
|---|---|---|---|---|
| LINC02076 | 0.525855 | 0.248861 | -1.13063 | 1.244484 |
| LRRC37A3 | 0.525856 | 0.248902 | 1.531432 | 0.705471 |
| PARD6B | 0.525981 | 0.249045 | 0.479192 | 5.990696 |
| SNORD95 | 0.525981 | 0.24905 | 0.840913 | 4.216071 |
| HBA2 | 0.525981 | 0.249081 | 1.210061 | 2.418912 |
| DCPS | 0.526272 | 0.249259 | 0.591326 | 3.404364 |
| SDCCAG8 | 0.526469 | 0.249393 | 0.898893 | 3.160356 |
| DIO3OS | 0.526503 | 0.249449 | 1.860694 | 1.253666 |
| LOC10013⁄ | 0.526639 | 0.249554 | 0.914352 | 2.962226 |
| LANCL2 | 0.526681 | 0.249639 | 0.45594 | 4.679625 |
| LUC7L3 | 0.526681 | 0.249654 | 0.505083 | 5.558796 |
| TICAM1 | 0.526926 | 0.24981 | -0.48997 | 5.523984 |
| HEATR1 | 0.527021 | 0.249929 | 0.460669 | 6.957553 |
| SNORA16B | 0.527021 | 0.249935 | -1.21453 | 1.639454 |
| SMG1P2 | 0.527255 | 0.250086 | 0.744496 | 3.830622 |
| KCNE2 | 0.527332 | 0.250163 | -2.27758 | 4.172673 |
| TBL2 | 0.527562 | 0.250313 | 0.52458 | 5.200735 |
| ARHGEF33 | 0.527593 | 0.250391 | 1.713772 | 0.932791 |
| H1FX | 0.527593 | 0.250423 | 0.458066 | 6.070965 |
| MIR4426 | 0.527593 | 0.250468 | -0.45884 | 7.084209 |
| MSL3P1 | 0.527593 | 0.250524 | 0.968518 | 2.53279 |
| GPR137C | 0.527593 | 0.250528 | 1.459521 | 1.385087 |
| RPL23AP32 | 0.528197 | 0.250855 | 1.678393 | 1.490781 |
| MMRN1 | 0.528735 | 0.251187 | 1.3342 | 3.545236 |
| ZC3H7B | 0.528735 | 0.251191 | 0.432592 | 6.983562 |
| IL1RAPL1 | 0.529453 | 0.251573 | 1.508259 | 0.432857 |
| PRSS30P | 0.529635 | 0.251701 | 1.857832 | 1.1382 |
| FAHD1 | 0.529635 | 0.25174 | -0.7246 | 3.593046 |
| SCARNA3 | 0.529786 | 0.251852 | 0.715832 | 3.593744 |
| AKAP1 | 0.529884 | 0.251976 | 1.032756 | 1.946825 |
| FOXH1 | 0.529884 | 0.25198 | 1.494886 | 0.294072 |
| LOC10192¦ | 0.529944 | 0.252049 | 0.818591 | 2.958091 |
| MTMR3 | 0.529944 | 0.252131 | -0.88864 | 1.608174 |
| ZNF428 | 0.529944 | 0.252219 | 0.857916 | 2.546108 |
| TNFRSF17 | 0.529944 | 0.252221 | 1.400166 | 1.454553 |
| BCAR3 | 0.529944 | 0.252238 | -0.80627 | 2.240223 |
| ABCA17P | 0.529944 | 0.252278 | 1.673824 | 1.82391 |
| KLK1 | 0.529944 | 0.252344 | 0.833009 | 2.178342 |
| SPIN3 | 0.529944 | 0.252345 | 0.602033 | 4.071804 |
| MUT | 0.529944 | 0.252414 | -0.40809 | 6.153056 |
| ACOX3 | 0.529944 | 0.252417 | -1.24656 | 0.927073 |
| AHCTF1P1 | 0.529944 | 0.252453 | 0.447354 | 6.395127 |
| SNX30 | 0.530108 | 0.252571 | -0.46285 | 6.210858 |
| TMEM51-A | 0.530567 | 0.25283 | -0.72846 | 3.24458 |
| MINOS1P1 | 0.530596 | 0.252884 | 0.810205 | 3.417463 |

| | | | | |
|---|---|---|---|---|
| GTPBP6 | 0.530844 | 0.253043 | -0.88346 | 2.78577 |
| IGFBPL1 | 0.53113 | 0.253239 | 1.697588 | 1.326063 |
| CLN6 | 0.53113 | 0.25326 | 0.627098 | 4.398841 |
| IL6ST | 0.531318 | 0.253391 | 1.608982 | 1.106237 |
| NAA38 | 0.531349 | 0.253481 | -0.5483 | 4.053446 |
| RAPGEF2 | 0.531349 | 0.253515 | 0.415762 | 6.938086 |
| PEX14 | 0.531349 | 0.253527 | -0.4748 | 4.978306 |
| MIR3140 | 0.531455 | 0.253676 | 1.442966 | 1.100183 |
| SEMA3D | 0.531455 | 0.25368 | 1.56578 | 1.994202 |
| DISP2 | 0.531455 | 0.253699 | 1.157016 | 2.557398 |
| XAB2 | 0.531888 | 0.254006 | 0.442732 | 5.609434 |
| CXCR4 | 0.531888 | 0.254018 | 1.185593 | 1.714342 |
| LINC00668 | 0.531888 | 0.254027 | 1.910306 | 1.170977 |
| FTO | 0.532099 | 0.254168 | 0.479311 | 6.206002 |
| MCM9 | 0.532371 | 0.254395 | -0.41707 | 5.355088 |
| KIAA2026 | 0.532371 | 0.254424 | -0.42592 | 7.29495 |
| OGFOD1 | 0.532371 | 0.25444 | -0.43427 | 5.880632 |
| SLC25A17 | 0.532371 | 0.254469 | -0.73523 | 3.275562 |
| ACKR4 | 0.532371 | 0.254501 | 1.151106 | 1.257834 |
| RAB30-AS1 | 0.532374 | 0.254543 | 0.721766 | 4.249659 |
| CMIP | 0.532869 | 0.25482 | -0.6214 | 3.885014 |
| METTL24 | 0.533144 | 0.255022 | 1.752605 | 0.792323 |
| AP4B1-AS1 | 0.533144 | 0.255035 | 1.421365 | 1.435237 |
| TMEM88 | 0.533144 | 0.255074 | 1.490907 | 0.868877 |
| LOC642943 | 0.534002 | 0.255558 | 1.930329 | 0.913108 |
| NECAB1 | 0.534002 | 0.255592 | 1.47297 | 2.275891 |
| WBP2 | 0.534002 | 0.255606 | -0.41514 | 6.628947 |
| WDFY3 | 0.534072 | 0.255711 | -0.40606 | 8.07574 |
| KLHL41 | 0.534072 | 0.255721 | 1.147797 | 1.74566 |
| SYBU | 0.534233 | 0.255839 | 1.790458 | 1.102585 |
| SLC10A5 | 0.534319 | 0.255921 | -1.08542 | 1.612566 |
| MTSS1L | 0.534332 | 0.255968 | -0.4959 | 6.867465 |
| EPB41L4A-. | 0.534365 | 0.256049 | -0.61464 | 4.114711 |
| B4GALT2 | 0.534365 | 0.256065 | -1.15312 | 1.257155 |
| ADCY6 | 0.53446 | 0.256169 | 0.432025 | 6.635314 |
| KIF19 | 0.53446 | 0.256192 | 1.935629 | 0.909259 |
| BORCS8 | 0.53451 | 0.256284 | 0.637826 | 3.26881 |
| PIP5K1C | 0.53451 | 0.256313 | 0.880158 | 2.288338 |
| RGS18 | 0.53451 | 0.256346 | 1.16207 | 1.588681 |
| ERRFI1 | 0.53451 | 0.256382 | 0.600359 | 7.714833 |
| SNHG9 | 0.53451 | 0.25642 | -1.26016 | 0.916681 |
| WDR1 | 0.534697 | 0.25655 | -0.42701 | 6.192173 |
| VPS9D1-AS | 0.535142 | 0.256849 | -0.59381 | 4.644185 |
| NSDHL | 0.535142 | 0.256906 | -1.57461 | 0.482809 |
| SNORA55 | 0.535142 | 0.256916 | 0.685814 | 7.069818 |

| | | | | |
|---|---|---|---|---|
| ATP6V0D1 | 0.535142 | 0.256927 | -0.54118 | 5.120374 |
| LARP4B | 0.535411 | 0.257097 | -0.44368 | 7.883913 |
| GART | 0.536418 | 0.257621 | 1.482806 | 0.961467 |
| RORC | 0.536648 | 0.257773 | 1.477624 | 2.35332 |
| PFN2 | 0.536649 | 0.257814 | -1.49171 | 1.354676 |
| DR1 | 0.536977 | 0.258043 | -0.47811 | 6.365718 |
| TMED10 | 0.536977 | 0.258054 | -0.42413 | 8.393414 |
| PPP2R2A | 0.537004 | 0.258167 | -0.9714 | 2.021665 |
| H1F0 | 0.537004 | 0.25818 | -0.5404 | 8.185879 |
| BTG2 | 0.537004 | 0.258293 | 0.550101 | 7.978191 |
| SAMM50 | 0.537004 | 0.258308 | -0.51278 | 5.172496 |
| MYO6 | 0.537004 | 0.258317 | -1.04275 | 2.728684 |
| MCUB | 0.537004 | 0.258332 | 0.912604 | 2.981048 |
| MRPS26 | 0.537004 | 0.258353 | 0.520655 | 4.782968 |
| VIRMA | 0.537053 | 0.258417 | 0.442528 | 5.336489 |
| CRELD1 | 0.53743 | 0.25864 | -0.92399 | 2.594978 |
| MAP3K6 | 0.53745 | 0.25869 | -1.57286 | 0.978729 |
| CDK17 | 0.537914 | 0.258954 | 1.021551 | 1.622594 |
| ECE1 | 0.538223 | 0.259181 | 1.347474 | 0.848566 |
| RPL7 | 0.538223 | 0.259185 | -0.47304 | 8.153235 |
| C9orf40 | 0.538224 | 0.259227 | -0.76589 | 2.449193 |
| HEBP1 | 0.538427 | 0.259366 | -0.61819 | 4.663392 |
| NUP58 | 0.538517 | 0.25945 | -1.67267 | 0.972798 |
| IFT46 | 0.538954 | 0.259702 | 1.680308 | 0.380669 |
| TRIM66 | 0.539036 | 0.259782 | -0.46305 | 6.18575 |
| SGF29 | 0.539195 | 0.2599 | 0.64772 | 3.924468 |
| SYNE1 | 0.539206 | 0.259946 | 1.580394 | 1.315603 |
| LLGL2 | 0.53984 | 0.260293 | 0.859103 | 3.409333 |
| SAMHD1 | 0.539863 | 0.260345 | 0.535804 | 6.981522 |
| SCRN2 | 0.539872 | 0.260412 | 1.335959 | 1.065045 |
| DDX3Y | 0.539872 | 0.260432 | -1.21188 | 4.794843 |
| YY2 | 0.539891 | 0.260482 | 0.859019 | 2.343463 |
| UBR5-AS1 | 0.539992 | 0.260572 | -1.5366 | 0.475189 |
| IFNA8 | 0.540095 | 0.260667 | -2.64156 | 0.706418 |
| ECSIT | 0.540095 | 0.260704 | -1.35506 | 2.017758 |
| POLR2K | 0.540223 | 0.260807 | 0.521985 | 4.483402 |
| CASP8 | 0.540518 | 0.260991 | 1.389672 | 1.766702 |
| C10orf143 | 0.540689 | 0.2612 | 1.12942 | 1.898956 |
| NIF3L1 | 0.540689 | 0.261209 | 1.392567 | 0.685854 |
| TIMM10B | 0.540689 | 0.261237 | 0.414498 | 6.19074 |
| GAR1 | 0.540689 | 0.261297 | 1.768026 | 0.492225 |
| H2AFZ | 0.540689 | 0.261346 | -0.45195 | 5.621569 |
| SNORD110 | 0.540689 | 0.261395 | 0.735138 | 4.386511 |
| DMD | 0.540689 | 0.261396 | 1.470258 | 2.39677 |
| MRPL1 | 0.540689 | 0.261403 | -0.81171 | 3.943702 |

| | | | | |
|---|---|---|---|---|
| RNPS1 | 0.540973 | 0.26162 | 0.757828 | 3.167156 |
| FAM229B | 0.540973 | 0.261623 | 1.020662 | 2.475245 |
| HHIP-AS1 | 0.541019 | 0.261688 | 2.018472 | 0.322765 |
| MCEE | 0.541019 | 0.261729 | -0.58808 | 4.125215 |
| NPIPB4 | 0.541019 | 0.261782 | 0.891225 | 2.46314 |
| RAB28 | 0.541019 | 0.261842 | -1.24386 | 1.985268 |
| MFSD4B | 0.541019 | 0.261913 | 0.444447 | 5.315282 |
| PPARG | 0.541019 | 0.261921 | 1.206534 | 2.141754 |
| HIPK3 | 0.541019 | 0.261934 | 0.970072 | 2.693412 |
| SNORA60 | 0.54108 | 0.262005 | 0.799167 | 4.140622 |
| APPL1 | 0.541164 | 0.262086 | -0.44676 | 7.225129 |
| TRAF3IP2-/ | 0.541655 | 0.262366 | 0.627289 | 3.013424 |
| PSMC2 | 0.541817 | 0.2625 | 0.424658 | 5.650489 |
| FBXL15 | 0.541817 | 0.262527 | 0.604135 | 3.178182 |
| NEK6 | 0.542082 | 0.262696 | 1.363245 | 1.772367 |
| PAPSS1 | 0.542151 | 0.262771 | -0.42263 | 6.174765 |
| GPR173 | 0.542301 | 0.262886 | 1.138481 | 2.169934 |
| HGD | 0.542301 | 0.262949 | 1.25421 | 2.941522 |
| UNK | 0.542301 | 0.262968 | -0.41423 | 5.95926 |
| CASC2 | 0.542336 | 0.263026 | 1.231621 | 1.870516 |
| PHF3 | 0.54334 | 0.263554 | 0.663303 | 3.05056 |
| MIR548V | 0.543684 | 0.263762 | 1.021902 | 2.148767 |
| UAP1L1 | 0.543802 | 0.263861 | 0.515442 | 4.601535 |
| TNNI3 | 0.543842 | 0.263935 | 1.696491 | 0.684347 |
| MIR26A2 | 0.543842 | 0.263964 | -1.96305 | 0.999461 |
| C5AR2 | 0.543932 | 0.264049 | -1.82524 | 1.257267 |
| ZNF506 | 0.543974 | 0.26411 | 1.12936 | 1.784619 |
| MICALCL | 0.544003 | 0.264166 | 0.640727 | 3.927224 |
| SLC25A34 | 0.544172 | 0.264326 | 0.947755 | 1.895593 |
| ESPNP | 0.544172 | 0.26437 | 1.440743 | 2.033015 |
| PPP2R3B | 0.544172 | 0.264373 | -0.94336 | 2.003308 |
| FAM95B1 | 0.544324 | 0.264488 | -1.11402 | 1.620056 |
| TSL | 0.545139 | 0.264925 | 1.58458 | 0.339091 |
| MRPS35 | 0.545185 | 0.265022 | 1.654606 | 0.486842 |
| VN1R5 | 0.545185 | 0.265052 | 2.216128 | 1.075733 |
| COL23A1 | 0.545185 | 0.265096 | 1.142844 | 1.875971 |
| SELENOP | 0.545185 | 0.265114 | 1.470563 | 1.091557 |
| UMAD1 | 0.545503 | 0.26531 | 1.38806 | 0.867958 |
| NFKBIA | 0.545596 | 0.265397 | -0.47867 | 7.636864 |
| UQCRC2 | 0.545702 | 0.26549 | -0.39878 | 6.859165 |
| ADGRB1 | 0.545773 | 0.265582 | -0.91997 | 2.660915 |
| NKTR | 0.545773 | 0.26561 | 0.422637 | 8.876442 |
| C11orf54 | 0.545773 | 0.265685 | 1.603751 | 0.496322 |
| NEDD8 | 0.545773 | 0.265691 | -0.6039 | 3.557249 |
| PHIP | 0.545791 | 0.265742 | 0.394385 | 8.501315 |

| | | | | |
|---|---|---|---|---|
| EIF2AK4 | 0.546934 | 0.26634 | 0.440516 | 6.570203 |
| ZCCHC2 | 0.546977 | 0.266402 | 0.492372 | 4.412317 |
| AMER1 | 0.54708 | 0.266494 | -0.42013 | 5.175325 |
| SLC35E2B | 0.547136 | 0.266563 | 1.807872 | 0.601831 |
| ADAMTS16 | 0.54715 | 0.266612 | 1.512131 | 0.866084 |
| DNAJC9-AS | 0.547554 | 0.26685 | 1.150182 | 1.711048 |
| NR3C1 | 0.547821 | 0.267035 | -0.68377 | 3.094942 |
| DNMT3A | 0.547821 | 0.267142 | 0.728877 | 2.715028 |
| CASC3 | 0.547821 | 0.267154 | 0.392802 | 7.680464 |
| ZDHHC20 | 0.547821 | 0.267194 | -1.15947 | 1.485934 |
| LCOR | 0.547821 | 0.267206 | -0.40542 | 6.427186 |
| MCCC2 | 0.547821 | 0.267231 | -0.40233 | 5.743839 |
| PRPH2 | 0.547976 | 0.267348 | 0.99609 | 2.068459 |
| NPTXR | 0.548014 | 0.267409 | 1.236687 | 2.562467 |
| MIR3138 | 0.548123 | 0.267503 | -1.5694 | 0.73132 |
| MUCL3 | 0.548257 | 0.26761 | 1.168917 | 5.711914 |
| USF3 | 0.548458 | 0.267751 | -0.39304 | 6.979362 |
| TMEM177 | 0.548475 | 0.2678 | 1.64208 | 0.577622 |
| GPN1 | 0.548515 | 0.267862 | 1.669507 | 0.680868 |
| HMGA2 | 0.54907 | 0.268175 | 0.810278 | 3.270988 |
| POLRMT | 0.549396 | 0.268376 | 0.429308 | 5.65266 |
| RPS16P5 | 0.549445 | 0.268454 | -0.71571 | 4.533704 |
| SGSM3 | 0.549445 | 0.268491 | -1.47146 | 1.016338 |
| HTR6 | 0.549445 | 0.268538 | 1.151082 | 1.318701 |
| ATP9A | 0.549445 | 0.268568 | 0.401799 | 7.814206 |
| YPEL5 | 0.550454 | 0.269103 | -1.24198 | 1.700453 |
| RTCB | 0.55055 | 0.269191 | -0.39918 | 6.338414 |
| LINC01578 | 0.550667 | 0.269301 | -0.62318 | 3.406009 |
| GK3P | 0.550667 | 0.269332 | -0.64708 | 3.167847 |
| KCNB1 | 0.550847 | 0.269463 | 1.745014 | 1.978215 |
| EAPP | 0.550883 | 0.269522 | -0.51458 | 5.50033 |
| NFYC | 0.550933 | 0.269628 | 0.95783 | 2.47328 |
| CASKIN1 | 0.550933 | 0.26963 | -1.09962 | 1.364385 |
| CNTNAP2 | 0.551033 | 0.269722 | 1.718181 | 1.26649 |
| MED27 | 0.551161 | 0.269826 | -1.24174 | 0.99018 |
| METTL16 | 0.551425 | 0.27002 | 0.399841 | 5.729 |
| TAF5 | 0.551425 | 0.27004 | 0.617052 | 3.54318 |
| TTC23 | 0.551722 | 0.270227 | -0.76611 | 2.627961 |
| CEPT1 | 0.551741 | 0.270278 | 0.87238 | 1.506067 |
| DUSP8 | 0.552157 | 0.270524 | 1.42714 | 0.412603 |
| ZNF791 | 0.552365 | 0.270668 | 0.41348 | 5.646468 |
| WDR3 | 0.552785 | 0.270933 | -0.42891 | 6.28535 |
| CPSF2 | 0.552785 | 0.270995 | -0.40918 | 6.91852 |
| CCDC47 | 0.552785 | 0.271 | -0.38785 | 7.848279 |
| TTC9C | 0.553173 | 0.271268 | 0.661586 | 3.85111 |

| | | | | |
|---|---|---|---|---|
| TUBGCP6 | 0.553173 | 0.271275 | 0.458238 | 6.658683 |
| MIEF1 | 0.553322 | 0.27143 | -1.58422 | 0.855767 |
| LOC100128 | 0.553322 | 0.271471 | 1.305482 | 1.873108 |
| DTX1 | 0.553322 | 0.271532 | -0.67324 | 3.084268 |
| BFAR | 0.553322 | 0.271554 | 0.810737 | 3.145022 |
| ZNF775 | 0.553322 | 0.271559 | 1.098952 | 2.017412 |
| WDR18 | 0.553361 | 0.271656 | -0.52915 | 4.423992 |
| RNU12 | 0.553361 | 0.271689 | 0.654879 | 7.543692 |
| GRM6 | 0.553361 | 0.271705 | 1.425437 | 0.964304 |
| WASHC1 | 0.553615 | 0.271871 | 0.771896 | 2.822526 |
| IDE | 0.55362 | 0.271916 | -0.51883 | 5.587343 |
| TRMO | 0.553718 | 0.272006 | 0.608309 | 3.61979 |
| THAP8 | 0.554153 | 0.272262 | 0.641243 | 3.148794 |
| CEND1 | 0.554243 | 0.272382 | -1.10827 | 1.443403 |
| ACTR3 | 0.554243 | 0.272391 | -1.30174 | 1.202835 |
| B3GALT6 | 0.554375 | 0.272498 | 0.524405 | 3.682911 |
| REG4 | 0.554696 | 0.2727 | 1.861501 | 0.662431 |
| LOC283177 | 0.554696 | 0.27274 | 2.034307 | 2.571171 |
| HOXC5 | 0.554838 | 0.272853 | 1.615478 | 0.687374 |
| PHLDA2 | 0.554924 | 0.272937 | 1.352103 | 2.169045 |
| FH | 0.555154 | 0.273093 | -0.47893 | 5.489415 |
| LINC00239 | 0.555213 | 0.273164 | 1.733367 | 0.989271 |
| MAGT1 | 0.555234 | 0.273216 | -0.45656 | 6.822171 |
| NCMAP | 0.555284 | 0.273283 | 1.235782 | 2.396096 |
| TPI1P2 | 0.555589 | 0.273476 | -1.21285 | 0.545522 |
| HEXD | 0.555647 | 0.273623 | 0.626021 | 4.103061 |
| ZNF174 | 0.555647 | 0.27363 | 0.961536 | 2.063656 |
| NAIF1 | 0.555647 | 0.273679 | 0.726377 | 2.746623 |
| TIMM13 | 0.555647 | 0.273755 | -0.45497 | 5.887286 |
| RNF219 | 0.555647 | 0.273791 | 0.545392 | 4.492909 |
| SAAL1 | 0.555647 | 0.273794 | 0.753341 | 3.412801 |
| NFAM1 | 0.555647 | 0.273828 | 0.710122 | 3.37945 |
| SNORD88C | 0.555647 | 0.273843 | 1.024627 | 1.531573 |
| PA2G4P4 | 0.556085 | 0.274162 | 0.617502 | 3.888532 |
| LAMTOR5 | 0.556085 | 0.274166 | -0.53979 | 4.41947 |
| CLEC18B | 0.556085 | 0.274192 | -1.26981 | 1.596907 |
| COPG2IT1 | 0.556085 | 0.274228 | 0.567975 | 4.124661 |
| DNAJC1 | 0.556199 | 0.274327 | 0.421563 | 5.648539 |
| INAFM1 | 0.55623 | 0.274385 | 0.846227 | 2.364664 |
| MNT | 0.556556 | 0.274588 | -0.43506 | 6.58192 |
| GSN-AS1 | 0.556801 | 0.274784 | 0.457626 | 5.07982 |
| VRK3 | 0.556801 | 0.274855 | 1.179308 | 1.103146 |
| NCOA5 | 0.556801 | 0.274875 | 0.413534 | 5.768086 |
| ELMO1-AS1 | 0.556801 | 0.274879 | 1.693519 | 0.465263 |
| GALNT2 | 0.556965 | 0.275002 | 0.94211 | 2.036486 |

| | | | | |
|---|---|---|---|---|
| NCAPD3 | 0.557157 | 0.275149 | 0.426923 | 5.847525 |
| TESK1 | 0.557157 | 0.275182 | 0.548607 | 4.689903 |
| GOLGA8R | 0.557182 | 0.275252 | 1.196618 | 1.371024 |
| CLCN3 | 0.557182 | 0.275279 | -1.29559 | 1.915317 |
| MIR5094 | 0.557337 | 0.275398 | 2.169356 | 0.356169 |
| INIP | 0.557416 | 0.275479 | -0.53774 | 4.304153 |
| GUCY1B2 | 0.55742 | 0.275524 | 1.107181 | 2.27365 |
| NAALADL1 | 0.557923 | 0.275815 | 1.365031 | 0.658079 |
| PGRMC1 | 0.558031 | 0.275911 | 1.315157 | 0.474981 |
| CAPS2 | 0.55822 | 0.276047 | 1.237031 | 1.574147 |
| LOC28329 | 0.558221 | 0.27609 | 1.773521 | 1.234075 |
| PRPF40B | 0.558317 | 0.27618 | 1.305613 | 0.84806 |
| GPAT3 | 0.558528 | 0.276327 | -1.6183 | 0.397836 |
| LAP3 | 0.559036 | 0.276621 | 0.441246 | 5.695624 |
| PPP1CC | 0.559119 | 0.276705 | -0.65484 | 4.785358 |
| CCDC39 | 0.559274 | 0.276829 | 0.666168 | 3.788016 |
| NDUFAF2 | 0.559274 | 0.276867 | -0.49629 | 4.348418 |
| CASC4 | 0.55928 | 0.276912 | 0.81084 | 3.373775 |
| TACC3 | 0.559461 | 0.277044 | 0.452427 | 5.266599 |
| ZNF284 | 0.559488 | 0.27713 | 0.572874 | 4.059938 |
| ITGA7 | 0.559488 | 0.277143 | 1.491061 | 0.92589 |
| TRIM58 | 0.559538 | 0.27721 | 1.110375 | 1.568869 |
| GATD1 | 0.559738 | 0.277352 | -0.4031 | 6.50748 |
| SIRT2 | 0.560084 | 0.277567 | 1.113372 | 1.163562 |
| LOC44115 | 0.560234 | 0.277684 | -0.86578 | 2.481209 |
| KLHL35 | 0.560272 | 0.277745 | 1.00572 | 1.608977 |
| NDUFB7 | 0.560313 | 0.277811 | -0.42396 | 5.347629 |
| ZNF232 | 0.560313 | 0.277867 | 0.547342 | 3.777522 |
| PAXIP1-AS | 0.560313 | 0.277916 | 0.840612 | 2.943383 |
| PWARSN | 0.560313 | 0.27797 | 0.631257 | 3.87617 |
| LOC10050 | 0.560313 | 0.277979 | 1.267608 | 1.090618 |
| ABRAXAS2 | 0.560556 | 0.278142 | -0.47348 | 5.425695 |
| NDUFS8 | 0.560556 | 0.278185 | -0.45328 | 4.625474 |
| C2CD2 | 0.560789 | 0.278343 | -0.74797 | 2.393055 |
| CCDC90B | 0.560794 | 0.278417 | 1.415933 | 0.700857 |
| TMEM123 | 0.560794 | 0.278431 | 0.409617 | 7.904324 |
| ZNF184 | 0.56081 | 0.278554 | 0.512255 | 4.418326 |
| JMJD1C-AS | 0.56081 | 0.278573 | 1.232439 | 1.188896 |
| GDE1 | 0.56081 | 0.278618 | 0.409465 | 6.262224 |
| CASP4 | 0.56081 | 0.278643 | 1.747866 | 0.531586 |
| ROCK1P1 | 0.56081 | 0.278653 | 0.628629 | 3.726817 |
| ATN1 | 0.561904 | 0.279355 | 1.160645 | 1.087394 |
| LOC10192 | 0.561904 | 0.279379 | -1.22777 | 1.919957 |
| ALDH1A3 | 0.561904 | 0.279416 | -1.0088 | 2.921343 |
| SLC25A25 | 0.561904 | 0.279432 | -1.0476 | 1.746083 |

| | | | | |
|---|---|---|---|---|
| ZNF318 | 0.561904 | 0.279456 | 0.422423 | 6.548168 |
| LOC101927 | 0.561904 | 0.279473 | -1.63607 | 1.007955 |
| LSM5 | 0.561904 | 0.279499 | 1.503821 | 0.641057 |
| FAM234B | 0.561904 | 0.279539 | -0.58606 | 3.485596 |
| VASH1 | 0.562013 | 0.279636 | 0.475812 | 5.239157 |
| LINC01515 | 0.562292 | 0.279826 | 1.621236 | 1.125867 |
| SUCLA2 | 0.562292 | 0.279861 | -0.42421 | 5.738578 |
| MIR548O | 0.562365 | 0.27994 | -1.63282 | 0.582624 |
| WASH2P | 0.562387 | 0.279994 | 0.785289 | 4.433422 |
| NDUFS3 | 0.5625 | 0.280093 | -0.51262 | 4.682061 |
| NAT10 | 0.562667 | 0.280219 | 0.727441 | 2.682299 |
| CES3 | 0.562716 | 0.280286 | -1.32164 | 1.173716 |
| AQR | 0.562778 | 0.28036 | -0.40804 | 6.867873 |
| LCA5L | 0.562822 | 0.280428 | 1.031657 | 2.077395 |
| ANAPC13 | 0.562822 | 0.280513 | 0.924692 | 1.696391 |
| SLC25A5 | 0.562822 | 0.280548 | -0.4982 | 6.806577 |
| C5orf51 | 0.562822 | 0.280553 | -0.52405 | 6.012253 |
| BTBD10 | 0.562996 | 0.280716 | -0.64374 | 3.280436 |
| NOP14-AS1 | 0.562996 | 0.280787 | -0.59085 | 3.764406 |
| GEM | 0.562996 | 0.28081 | 1.543437 | 1.262869 |
| NBL1 | 0.562996 | 0.280834 | 0.704161 | 2.390006 |
| ESCO2 | 0.562996 | 0.28086 | 0.886924 | 3.355964 |
| MT1H | 0.562996 | 0.280904 | 1.560673 | 2.094074 |
| SMAP1 | 0.562996 | 0.280952 | -1.27485 | 0.653318 |
| SOS1 | 0.562996 | 0.281017 | -0.38894 | 7.479299 |
| LOC101928 | 0.562996 | 0.281026 | 1.120542 | 2.0248 |
| TBC1D30 | 0.563145 | 0.281144 | 1.024672 | 3.842644 |
| ZNF32-AS2 | 0.563296 | 0.281261 | 1.046049 | 2.625206 |
| SAMD15 | 0.563357 | 0.281335 | 1.089521 | 1.619588 |
| TFAP4 | 0.563445 | 0.281422 | 0.558489 | 4.420781 |
| SYMPK | 0.563559 | 0.281546 | 0.392613 | 6.737306 |
| GRIP2 | 0.563559 | 0.281565 | -0.87062 | 3.623767 |
| PRKAG2 | 0.563762 | 0.28171 | 1.049149 | 2.102321 |
| HCFC1 | 0.563762 | 0.281752 | 0.402902 | 7.387205 |
| SMAGP | 0.564112 | 0.281993 | -1.13003 | 0.81945 |
| DDX23 | 0.564112 | 0.282037 | 0.408614 | 7.603765 |
| NKAIN1 | 0.564112 | 0.282056 | -1.49057 | 1.171018 |
| TOR1A | 0.56469 | 0.282388 | 0.474252 | 4.636514 |
| TROAP | 0.565073 | 0.282657 | 0.751832 | 2.823383 |
| ZNF485 | 0.565073 | 0.282684 | 0.888226 | 3.083331 |
| DKFZP586I | 0.565073 | 0.282709 | -0.49386 | 5.232442 |
| CYBC1 | 0.56509 | 0.28276 | 0.600932 | 3.110344 |
| CA11 | 0.565303 | 0.28291 | -0.60951 | 4.265798 |
| A1BG | 0.565433 | 0.283059 | 1.430386 | 0.809168 |
| TJP2 | 0.565433 | 0.283061 | -0.48846 | 5.181242 |

| | | | | |
|---|---|---|---|---|
| C2orf68 | 0.565477 | 0.283126 | 0.43189 | 5.785628 |
| BSDC1 | 0.565528 | 0.283205 | 0.534096 | 3.797933 |
| MIR1343 | 0.565528 | 0.283238 | 1.617678 | 0.322515 |
| IZUMO1 | 0.566061 | 0.283548 | -0.95993 | 1.69992 |
| CACNA2D3 | 0.566189 | 0.283655 | -1.48023 | 1.06588 |
| BCL2 | 0.566213 | 0.283711 | 0.600069 | 3.695963 |
| LOC10050! | 0.566236 | 0.283765 | -1.04059 | 1.910428 |
| PANK3 | 0.566239 | 0.28381 | -0.38673 | 7.255185 |
| ADAM23 | 0.566285 | 0.283876 | -1.00049 | 5.358261 |
| TIA1 | 0.566386 | 0.28397 | 1.429715 | 0.538633 |
| FBXO9 | 0.566589 | 0.284115 | -0.83533 | 2.495113 |
| ENKD1 | 0.566602 | 0.284198 | 0.747403 | 3.38065 |
| TLNRD1 | 0.566602 | 0.284208 | 0.499777 | 5.300633 |
| DGCR6L | 0.566674 | 0.284324 | -0.58257 | 3.623928 |
| PDCD2L | 0.566674 | 0.28433 | 0.982683 | 2.854146 |
| FAM104A | 0.56686 | 0.284467 | -1.57956 | 0.869883 |
| TTC3 | 0.566874 | 0.284517 | 0.438424 | 6.288902 |
| TCEA2 | 0.567184 | 0.284753 | 0.953063 | 2.79984 |
| CERNA1 | 0.567184 | 0.284785 | 1.319457 | 0.840055 |
| APOL6 | 0.567184 | 0.284802 | 0.418268 | 7.139631 |
| CIZ1 | 0.567333 | 0.28492 | 1.293917 | 1.193839 |
| RSRP1 | 0.567776 | 0.28526 | -0.40145 | 6.494023 |
| MACROD2- | 0.567776 | 0.285263 | 1.280217 | 1.534844 |
| KCNMA1-A | 0.567776 | 0.28529 | 1.254865 | 2.126038 |
| LINC00997 | 0.567776 | 0.285316 | 0.542951 | 3.920855 |
| MAP3K2 | 0.567785 | 0.28541 | 0.371254 | 7.766282 |
| MED26 | 0.567785 | 0.285415 | 0.44091 | 4.588149 |
| NR1H2 | 0.567785 | 0.28545 | 0.84087 | 2.244926 |
| LINC01909 | 0.567969 | 0.285586 | 1.353555 | 0.891275 |
| PTPRVP | 0.568005 | 0.285668 | 1.498768 | 1.018255 |
| FLNA | 0.568005 | 0.285691 | 1.605728 | 0.747388 |
| ITPRID2 | 0.568442 | 0.285954 | 0.625405 | 4.831677 |
| LINC00628 | 0.568762 | 0.286158 | 1.077255 | 1.499978 |
| HPCAL1 | 0.568793 | 0.286217 | 0.996566 | 2.753548 |
| IGBP1 | 0.568998 | 0.286388 | -0.39018 | 5.990356 |
| OTUD1 | 0.568998 | 0.286457 | 0.535618 | 4.901834 |
| KIF12 | 0.568998 | 0.286487 | 1.246873 | 2.908014 |
| RBP2 | 0.568998 | 0.286494 | 1.297991 | 0.78168 |
| SGIP1 | 0.569319 | 0.286699 | 1.308447 | 1.284298 |
| CSTF3 | 0.569336 | 0.286751 | 0.475881 | 4.760291 |
| A1BG-AS1 | 0.569483 | 0.286868 | 1.147 | 1.145062 |
| C1QTNF2 | 0.569691 | 0.287091 | 1.148366 | 0.964704 |
| NBPF3 | 0.569691 | 0.287099 | 1.249533 | 1.423362 |
| OARD1 | 0.569691 | 0.28712 | -0.46436 | 4.918115 |
| IFITM10 | 0.569691 | 0.287177 | -0.65801 | 3.38447 |

| | | | | |
|---|---|---|---|---|
| MYOM2 | 0.569691 | 0.287218 | 1.378523 | 0.672274 |
| HLF | 0.569691 | 0.287233 | -0.74162 | 5.534293 |
| GDF11 | 0.569698 | 0.28728 | 0.602273 | 3.572693 |
| PTPRC | 0.5698 | 0.287375 | 0.700257 | 3.821417 |
| SYNJ2BP | 0.569945 | 0.287492 | 0.417049 | 6.519178 |
| TBCA | 0.570109 | 0.287637 | -0.9811 | 2.455823 |
| PFN1P2 | 0.570109 | 0.287661 | 0.466682 | 4.423412 |
| CASC20 | 0.570121 | 0.287711 | -2.28255 | 0.81129 |
| COX10-AS1 | 0.570323 | 0.28788 | -0.59392 | 3.948839 |
| CDKN1A | 0.570323 | 0.287925 | -0.90329 | 2.641029 |
| ANP32A-IT | 0.570323 | 0.287943 | 0.742565 | 3.793714 |
| CYB5D2 | 0.57035 | 0.288 | -1.12836 | 1.819376 |
| ZNF460 | 0.57036 | 0.288049 | -0.38578 | 7.763379 |
| LOC171391 | 0.570797 | 0.28832 | -1.10777 | 1.125716 |
| SNORD91A | 0.570797 | 0.288357 | -1.08367 | 1.707962 |
| TESK2 | 0.57094 | 0.288472 | -0.60843 | 4.296172 |
| HIF1A | 0.571019 | 0.288586 | -0.78511 | 4.996562 |
| FKBP4 | 0.571019 | 0.288599 | -0.471 | 7.401835 |
| LOC389831 | 0.571064 | 0.288692 | 0.964537 | 2.957847 |
| LINC01132 | 0.571064 | 0.288749 | 1.165175 | 0.755678 |
| SYNPO2L | 0.571064 | 0.288758 | -1.06799 | 2.132353 |
| COG5 | 0.571064 | 0.2888 | 0.610495 | 3.584823 |
| NIPBL-DT | 0.571064 | 0.288839 | 0.507088 | 4.672093 |
| CCND1 | 0.571111 | 0.288954 | 0.520016 | 9.179781 |
| TBKBP1 | 0.571111 | 0.288972 | -0.54683 | 4.65103 |
| DYNC1LI1 | 0.571111 | 0.289131 | 0.389774 | 5.695485 |
| SLC2A4RG | 0.571111 | 0.289132 | -0.41474 | 5.380415 |
| SLITRK5 | 0.571111 | 0.289142 | 1.482079 | 1.462495 |
| CTSB | 0.571111 | 0.289161 | -1.20092 | 0.962567 |
| ARHGAP35 | 0.571111 | 0.28918 | -0.37816 | 7.969658 |
| RBM28 | 0.571111 | 0.289211 | 0.586849 | 3.653758 |
| ITIH5 | 0.571165 | 0.289282 | -1.68192 | 0.972494 |
| ZNF524 | 0.57129 | 0.289389 | -0.59639 | 3.292612 |
| LOXL4 | 0.57168 | 0.28963 | 0.757496 | 3.573707 |
| MAPKAPK5 | 0.57171 | 0.289689 | 0.573842 | 3.520656 |
| TMEM229B | 0.571778 | 0.289767 | 1.219002 | 1.845053 |
| ZNF608 | 0.571961 | 0.28992 | 0.546606 | 6.120405 |
| GCNT2 | 0.571961 | 0.289947 | -0.70589 | 4.47654 |
| CCDC22 | 0.572006 | 0.290013 | -0.4598 | 4.123918 |
| SGCA | 0.572016 | 0.290097 | -2.32706 | 0.796226 |
| OSGEP | 0.572016 | 0.290105 | 0.46033 | 4.501313 |
| RNF138 | 0.572019 | 0.290193 | 1.267849 | 1.157696 |
| SEPSECS-AS | 0.572019 | 0.290194 | 0.995375 | 1.456564 |
| KHDC1 | 0.57234 | 0.290401 | 0.876262 | 2.127397 |
| NUFIP2 | 0.572811 | 0.290683 | 0.373164 | 8.766716 |

| | | | | |
|---|---|---|---|---|
| ARHGAP26 | 0.572871 | 0.290787 | 1.200417 | 1.818102 |
| DDX19A | 0.572871 | 0.290845 | 0.410279 | 5.722097 |
| RPUSD1 | 0.572871 | 0.290845 | 0.572508 | 3.646578 |
| GRASP | 0.573697 | 0.291312 | 1.267837 | 1.493333 |
| RNF138P1 | 0.573697 | 0.291371 | 0.46305 | 4.83405 |
| IL1R2 | 0.573697 | 0.291395 | 1.639205 | 1.079607 |
| TTC37 | 0.573939 | 0.291562 | -0.37331 | 7.313678 |
| XRRA1 | 0.574603 | 0.291943 | -1.14246 | 1.584462 |
| MRPL46 | 0.574717 | 0.292084 | -0.58795 | 3.73782 |
| PPAT | 0.574717 | 0.292089 | 0.514631 | 4.547549 |
| DCUN1D2 | 0.57474 | 0.292144 | 0.602861 | 4.59916 |
| FBXO46 | 0.575115 | 0.292419 | -0.43579 | 5.333421 |
| CACUL1 | 0.575115 | 0.292461 | -0.38089 | 7.222686 |
| DIPK2B | 0.575115 | 0.292466 | 0.617877 | 3.666755 |
| PPARD | 0.575153 | 0.29253 | -1.11318 | 2.362925 |
| CCDC114 | 0.575313 | 0.292655 | 1.069094 | 1.355328 |
| TBX1 | 0.575397 | 0.292745 | 1.367621 | 2.177105 |
| GZMK | 0.575397 | 0.292814 | 1.786675 | 1.295197 |
| CALHM6 | 0.575397 | 0.292829 | 1.899964 | 0.525901 |
| GPR162 | 0.575453 | 0.292901 | 1.454939 | 1.039854 |
| ANKH | 0.575458 | 0.292948 | 0.398943 | 6.255925 |
| MYCBP2-A! | 0.575595 | 0.293064 | -0.99181 | 1.741912 |
| ARMC1 | 0.575595 | 0.293105 | -0.8956 | 2.022183 |
| PLA2G15 | 0.576044 | 0.293378 | 0.546308 | 3.275637 |
| PIGA | 0.576092 | 0.293446 | -1.22654 | 1.623013 |
| SPPL2A | 0.576147 | 0.29357 | 0.403051 | 5.27439 |
| S100A7A | 0.576147 | 0.293578 | -1.44161 | 2.501635 |
| GORASP2 | 0.576147 | 0.293606 | 1.041497 | 1.531542 |
| RBM14 | 0.576672 | 0.293991 | 0.499517 | 4.215021 |
| SBF2 | 0.576672 | 0.294004 | -0.37077 | 7.306884 |
| C2CD4D | 0.576672 | 0.294005 | 0.907675 | 1.859495 |
| CLEC16A | 0.577068 | 0.294251 | 0.445377 | 5.306816 |
| SUFU | 0.577108 | 0.294315 | -0.51314 | 4.203231 |
| ADARB2 | 0.577183 | 0.294398 | -1.05287 | 1.4889 |
| ACKR2 | 0.577303 | 0.294503 | -0.69788 | 2.149903 |
| APOBEC3C | 0.577371 | 0.294582 | -0.49569 | 5.000956 |
| WASH8P | 0.577581 | 0.29482 | 0.56085 | 4.632693 |
| RPS17 | 0.577581 | 0.294837 | -1.1608 | 0.931109 |
| LOC14578: | 0.577581 | 0.294847 | 0.865852 | 2.864713 |
| IREB2 | 0.577581 | 0.294891 | -0.44363 | 6.899638 |
| RBM4 | 0.577581 | 0.294909 | 0.495796 | 5.128811 |
| WDR6 | 0.57774 | 0.295034 | 0.411299 | 6.466862 |
| TGS1 | 0.577784 | 0.2951 | 1.228397 | 0.93093 |
| LOC44017: | 0.577889 | 0.295198 | -1.63355 | 1.882409 |
| PARP4 | 0.578063 | 0.295331 | 0.377544 | 8.421508 |

| | | | | |
|---|---|---|---|---|
| SNORD45A | 0.578181 | 0.295486 | 0.577219 | 8.972355 |
| CBY3 | 0.578181 | 0.295493 | 1.577186 | 0.887033 |
| SDHAF4 | 0.578181 | 0.295524 | -0.59688 | 3.345483 |
| RANGAP1 | 0.578196 | 0.295575 | -0.62926 | 3.254257 |
| BSN | 0.578864 | 0.295961 | 0.683867 | 3.162375 |
| PTPRA | 0.578927 | 0.29605 | 1.192029 | 1.78366 |
| SPTSSA | 0.578927 | 0.296081 | -0.50783 | 5.368743 |
| CFAP54 | 0.579097 | 0.296212 | 1.031973 | 2.809563 |
| RNF5P1 | 0.57962 | 0.296524 | 0.975905 | 1.17449 |
| RALGAPA1 | 0.579645 | 0.296637 | -0.38919 | 6.514909 |
| LACTB2 | 0.579645 | 0.296656 | 0.558537 | 3.555152 |
| RGMA | 0.579645 | 0.29667 | -1.15143 | 0.767061 |
| FKBP14 | 0.579775 | 0.29678 | -1.71451 | 0.813079 |
| FASTKD3 | 0.579847 | 0.296861 | 0.921289 | 2.139531 |
| TVP23A | 0.57989 | 0.296966 | -0.85943 | 2.434179 |
| SEL1L | 0.57989 | 0.296972 | 0.425093 | 7.119218 |
| CCT6P1 | 0.579966 | 0.297055 | 0.533518 | 3.992707 |
| TTTY14 | 0.579985 | 0.297132 | -1.81826 | 0.722295 |
| GDI2 | 0.579985 | 0.297165 | 1.251468 | 0.511039 |
| BTBD8 | 0.579985 | 0.297197 | 0.884587 | 2.233503 |
| MTPAP | 0.580601 | 0.297566 | 0.457397 | 5.584143 |
| TTC7B | 0.580601 | 0.297601 | 0.565532 | 4.347413 |
| PPIL4 | 0.58062 | 0.297655 | 0.370385 | 6.598705 |
| MAGEE1 | 0.581204 | 0.297999 | 0.955862 | 1.697946 |
| NSUN5P2 | 0.581225 | 0.298054 | 1.038196 | 1.150738 |
| PLCB4 | 0.58183 | 0.298409 | 1.735914 | 0.431199 |
| PML | 0.582027 | 0.298554 | 0.463344 | 4.582811 |
| PSMB5 | 0.582071 | 0.298643 | 1.165249 | 1.401089 |
| TSSC4 | 0.582071 | 0.298695 | 1.080383 | 1.6996 |
| LRFN3 | 0.582071 | 0.298728 | 0.602536 | 3.55287 |
| HSPA13 | 0.582071 | 0.298754 | 0.712805 | 4.964373 |
| ITGB8 | 0.582156 | 0.298842 | -0.45382 | 7.361811 |
| SATB1-AS1 | 0.582655 | 0.299145 | 1.624647 | 0.838294 |
| CAT | 0.582655 | 0.299187 | 0.405342 | 5.928353 |
| SHANK3 | 0.582781 | 0.299296 | 0.442557 | 5.784363 |
| C11orf91 | 0.582836 | 0.299368 | -1.26601 | 2.255853 |
| TIMM21 | 0.583087 | 0.299542 | -0.44925 | 4.6951 |
| UGT1A3 | 0.58326 | 0.299706 | -1.67657 | 1.091375 |
| PBDC1 | 0.58326 | 0.299722 | -1.81067 | 0.759627 |
| TUBA3D | 0.58326 | 0.299764 | -0.94744 | 1.748202 |
| PLK3 | 0.583422 | 0.299995 | -0.50925 | 5.847289 |
| MYO1C | 0.583422 | 0.300029 | 1.286105 | 0.40767 |
| PSKH1 | 0.583422 | 0.300053 | -0.47015 | 4.820087 |
| KRTAP12-2 | 0.583422 | 0.300061 | 2.625838 | 0.260955 |
| ZNF33BP1 | 0.583422 | 0.30007 | 1.040847 | 1.8768 |

| | | | | |
|---|---|---|---|---|
| SH2D6 | 0.583545 | 0.300177 | 1.332232 | 0.601719 |
| HSF2BP | 0.583737 | 0.300321 | 1.111048 | 1.809257 |
| DPYD-AS2 | 0.58378 | 0.300428 | -1.35357 | 0.868523 |
| KRBOX1 | 0.58378 | 0.300467 | -1.40747 | 0.755733 |
| THOC1 | 0.58378 | 0.300476 | 0.449884 | 5.919931 |
| YJU2 | 0.583878 | 0.300576 | 0.534261 | 4.390327 |
| SNORD66 | 0.583878 | 0.300616 | 1.173169 | 1.32584 |
| RRP15 | 0.584076 | 0.300762 | 0.417443 | 5.469643 |
| SBF1 | 0.584125 | 0.300832 | -0.36751 | 7.485985 |
| C19orf44 | 0.584538 | 0.301089 | 1.122209 | 0.847412 |
| LYPLA2 | 0.584979 | 0.301361 | -0.37046 | 5.569681 |
| EEF1AKMT | 0.585258 | 0.301549 | 0.841594 | 2.486732 |
| CFAP61 | 0.585973 | 0.302002 | 2.140887 | 0.682483 |
| LOC101927 | 0.585973 | 0.30201 | -1.05578 | 1.709665 |
| TEAD3 | 0.585973 | 0.302092 | -0.39043 | 6.60108 |
| NRG4 | 0.585973 | 0.302096 | 0.667695 | 3.40574 |
| ATF2 | 0.586094 | 0.30222 | 1.557471 | 0.564159 |
| MN1 | 0.586094 | 0.302248 | 0.555706 | 4.913652 |
| FAM83H | 0.5861 | 0.302404 | -0.80358 | 7.206298 |
| BLOC1S2 | 0.5861 | 0.3025 | 0.896947 | 2.016511 |
| MEF2D | 0.5861 | 0.302521 | 0.830581 | 2.693102 |
| PRIM2 | 0.5861 | 0.302525 | 0.59775 | 2.948695 |
| ABI2 | 0.5861 | 0.302535 | 1.368141 | 0.51776 |
| MPL | 0.5861 | 0.302569 | 0.8828 | 2.644662 |
| ATP6V0E1 | 0.5861 | 0.302594 | -0.42976 | 5.932603 |
| TRUB1 | 0.5861 | 0.302652 | 0.457889 | 4.920856 |
| ZNF529 | 0.5861 | 0.302653 | 0.829734 | 2.551116 |
| BAIAP2L1 | 0.586345 | 0.302837 | 0.444309 | 7.630172 |
| HIRIP3 | 0.586345 | 0.3029 | 0.628941 | 4.39957 |
| MSI1 | 0.586345 | 0.302917 | -1.01407 | 1.705551 |
| HIST1H4K | 0.586345 | 0.302969 | 0.939923 | 4.035565 |
| SERINC1 | 0.586345 | 0.303003 | 0.410048 | 7.804843 |
| CENPU | 0.586463 | 0.303109 | -1.54625 | 0.993718 |
| SF3B4 | 0.586822 | 0.303347 | 0.380005 | 5.83838 |
| METRNL | 0.586822 | 0.303416 | -0.67882 | 3.764353 |
| GTF3A | 0.586822 | 0.303483 | 0.440398 | 5.299578 |
| SCYL3 | 0.586822 | 0.303566 | 1.107698 | 1.485662 |
| TSC22D1 | 0.586822 | 0.303576 | 0.482213 | 5.552915 |
| RNU4ATAC | 0.586822 | 0.303592 | 0.64329 | 5.514954 |
| SETBP1 | 0.586822 | 0.303609 | 0.567265 | 6.045765 |
| GOLGA2P6 | 0.586822 | 0.303652 | -1.89054 | 1.427866 |
| FLRT1 | 0.587875 | 0.304273 | -0.72655 | 3.875819 |
| FTSJ3 | 0.587875 | 0.304287 | 0.371945 | 6.46123 |
| FDX2 | 0.588109 | 0.304453 | 0.698157 | 2.469003 |
| MIR454 | 0.588157 | 0.304522 | 1.798822 | 0.383042 |

| | | | | |
|---|---|---|---|---|
| LOC100507 | 0.588253 | 0.304617 | 1.734587 | 0.906722 |
| NCKAP5L | 0.588547 | 0.304819 | 0.440792 | 4.854204 |
| SVIP | 0.588547 | 0.304858 | 0.515763 | 4.795265 |
| PNPLA3 | 0.588588 | 0.304925 | 1.081594 | 2.228864 |
| PLA2R1 | 0.588624 | 0.30506 | -0.99934 | 2.727714 |
| MORC2-AS | 0.588624 | 0.305128 | 0.832608 | 2.313653 |
| KCNN1 | 0.588624 | 0.305147 | 1.608598 | 0.741336 |
| GCSHP3 | 0.588624 | 0.305148 | 0.602126 | 4.001411 |
| GRK2 | 0.588624 | 0.305168 | -0.39372 | 7.31538 |
| ZNF398 | 0.588754 | 0.30528 | -0.6985 | 2.577786 |
| LRP1-AS | 0.588948 | 0.305425 | -1.60046 | 1.030372 |
| VPS36 | 0.588956 | 0.305474 | 1.030678 | 1.654089 |
| ELMOD2 | 0.589024 | 0.305592 | -0.43118 | 5.370959 |
| CNGB3 | 0.589024 | 0.305627 | 1.679776 | 1.172843 |
| GSK3A | 0.589024 | 0.305644 | 0.358322 | 5.734845 |
| CTSF | 0.589188 | 0.305774 | 0.896239 | 3.787273 |
| EPN1 | 0.589589 | 0.306035 | -0.63561 | 3.071646 |
| USP2 | 0.589589 | 0.306114 | -1.39114 | 1.063574 |
| TRPV6 | 0.589589 | 0.306193 | -1.07766 | 1.501861 |
| GRK3 | 0.589589 | 0.306217 | 0.504591 | 5.258998 |
| GGA2 | 0.589589 | 0.306251 | 0.398492 | 6.577042 |
| EPB41 | 0.589589 | 0.306252 | 1.159593 | 1.554008 |
| RFXAP | 0.589638 | 0.306322 | 0.598388 | 3.574926 |
| EPB41L4B | 0.589834 | 0.306469 | 0.669815 | 3.961172 |
| KANSL2 | 0.589907 | 0.306552 | 0.409454 | 4.824587 |
| FAM72B | 0.589965 | 0.306627 | 1.319079 | 1.042359 |
| DTD2 | 0.590009 | 0.306776 | -0.46278 | 4.662989 |
| MRPS22 | 0.590009 | 0.306784 | -0.40137 | 5.683163 |
| EFHB | 0.590009 | 0.306785 | 1.601393 | 0.493671 |
| S100A1 | 0.590199 | 0.306929 | -0.90584 | 2.501807 |
| GRB2 | 0.590561 | 0.307162 | 1.610475 | 0.673831 |
| ZNF841 | 0.590775 | 0.307318 | 0.451131 | 6.618181 |
| SCUBE2 | 0.590777 | 0.307364 | -1.78491 | 0.874778 |
| JRKL | 0.591362 | 0.307714 | 0.906459 | 1.211952 |
| MZT1 | 0.591546 | 0.3079 | 0.50571 | 4.263379 |
| FGFR1OP | 0.591546 | 0.3079 | 0.863022 | 2.458467 |
| CFAP65 | 0.591773 | 0.308063 | -1.35231 | 1.348772 |
| LOC101927 | 0.591839 | 0.308143 | -1.04817 | 2.115492 |
| MIS12 | 0.591854 | 0.308195 | -1.22097 | 0.982598 |
| EIF2D | 0.591998 | 0.308316 | 0.611818 | 3.41205 |
| BPGM | 0.592116 | 0.308435 | 1.584556 | 0.584774 |
| PPP3R2 | 0.592116 | 0.308467 | 1.772489 | 0.619153 |
| ARL6IP6 | 0.592221 | 0.308567 | -0.63323 | 2.630392 |
| ZNF561-AS | 0.592512 | 0.308764 | 0.681001 | 2.64862 |
| TMPRSS9 | 0.592569 | 0.308878 | 0.665802 | 3.627204 |

| | | | | |
|---|---|---|---|---|
| HACE1 | 0.592569 | 0.308884 | -0.96309 | 2.2636 |
| MYPOP | 0.594071 | 0.309737 | 0.639187 | 3.411022 |
| MAPRE2 | 0.594071 | 0.309757 | 1.441774 | 0.548075 |
| MIIP | 0.594139 | 0.309856 | 0.560296 | 3.304237 |
| RMST | 0.594139 | 0.309936 | -1.72795 | 2.394368 |
| ZNF623 | 0.594139 | 0.309939 | 1.397678 | 0.99428 |
| GPI | 0.594139 | 0.309974 | -0.73423 | 3.957732 |
| CHD9 | 0.594329 | 0.310118 | -1.24654 | 0.599934 |
| CCER2 | 0.594968 | 0.310497 | -1.07946 | 1.307511 |
| MANEA-DT | 0.595017 | 0.310568 | 1.114884 | 2.061125 |
| ZFC3H1 | 0.595213 | 0.310716 | -0.36633 | 8.538163 |
| NRAV | 0.595213 | 0.310807 | -0.53706 | 3.97255 |
| SNAI2 | 0.595213 | 0.310829 | -0.5344 | 4.903614 |
| GAS7 | 0.595213 | 0.31086 | -0.81159 | 2.260382 |
| TYRO3P | 0.595213 | 0.310933 | 0.973927 | 2.243191 |
| APCDD1 | 0.595213 | 0.310942 | 0.796152 | 6.278583 |
| PREB | 0.595356 | 0.311104 | 0.417927 | 5.316739 |
| C11orf53 | 0.595356 | 0.311108 | 1.569109 | 0.461503 |
| ZNF581 | 0.595867 | 0.31142 | -0.85699 | 2.971042 |
| SLC4A1AP | 0.596141 | 0.311617 | -0.37885 | 6.336614 |
| MTERF1 | 0.596141 | 0.311662 | 0.947772 | 1.515618 |
| SNORA13 | 0.596141 | 0.3117 | 0.583865 | 5.690981 |
| RTL1 | 0.596204 | 0.311778 | -1.33705 | 2.095269 |
| LIG3 | 0.596225 | 0.311835 | 0.724296 | 2.832996 |
| FBXO40 | 0.596399 | 0.311994 | -2.01679 | 0.826156 |
| TUFM | 0.596399 | 0.312019 | -0.39566 | 7.058352 |
| NFATC2IP | 0.596399 | 0.312062 | 0.381084 | 6.419735 |
| ABHD14A | 0.596601 | 0.312213 | 1.207779 | 0.696275 |
| SLC43A3 | 0.597167 | 0.312554 | 0.982738 | 1.23206 |
| NCBP3 | 0.597178 | 0.312635 | -0.36036 | 7.332881 |
| DUSP16 | 0.597178 | 0.312652 | -0.38406 | 7.099021 |
| LOC101928 | 0.597655 | 0.312998 | 1.537447 | 0.484287 |
| DTWD1 | 0.597655 | 0.313001 | 1.070572 | 1.705121 |
| PRXL2A | 0.597655 | 0.313057 | 1.412606 | 1.148707 |
| MTCP1 | 0.597655 | 0.313083 | 1.345086 | 1.264989 |
| MYBPC3 | 0.597972 | 0.313295 | 1.093996 | 2.249506 |
| SPG7 | 0.598387 | 0.313602 | 0.431889 | 5.494018 |
| RP9 | 0.598387 | 0.313649 | 0.735395 | 3.335741 |
| RHOT2 | 0.598387 | 0.313693 | 0.428129 | 5.979016 |
| ZNF439 | 0.598387 | 0.313695 | -0.5201 | 3.921441 |
| ACTR3-AS1 | 0.598526 | 0.313813 | -0.97874 | 1.489114 |
| CKMT2-AS1 | 0.598788 | 0.313996 | -1.10498 | 1.477617 |
| DISC1-IT1 | 0.598861 | 0.31408 | 1.43461 | 0.981701 |
| TCP11L1 | 0.599098 | 0.31425 | 1.485246 | 0.281192 |
| RBFADN | 0.599194 | 0.314419 | 1.431405 | 0.313823 |

| | | | | |
|---|---|---|---|---|
| SNORA50A | 0.599194 | 0.31443 | -0.71611 | 3.634759 |
| PPFIA1 | 0.599194 | 0.314437 | 1.24962 | 1.121608 |
| LOC101928 | 0.599208 | 0.31449 | 1.221558 | 0.747859 |
| TMEM183A | 0.599334 | 0.314603 | -0.36022 | 6.093388 |
| SNORD51 | 0.599471 | 0.314727 | 1.434067 | 0.724098 |
| LINC00623 | 0.599471 | 0.314765 | 1.271683 | 1.019697 |
| ITGB1 | 0.599477 | 0.314815 | 1.006204 | 1.549314 |
| TMEM121 | 0.599511 | 0.314878 | -1.78429 | 0.583094 |
| LOC104054 | 0.599632 | 0.315015 | 1.685305 | 0.591826 |
| CRYM | 0.599632 | 0.315033 | -0.64272 | 2.532509 |
| MROH9 | 0.599735 | 0.315197 | -1.31613 | 0.660246 |
| MYDGF | 0.599735 | 0.315247 | 0.439509 | 5.087031 |
| LOC100129 | 0.599735 | 0.315257 | -1.41987 | 1.185273 |
| ZNF586 | 0.599735 | 0.315322 | -1.39382 | 0.647893 |
| LOC105375 | 0.599735 | 0.315329 | 1.08492 | 1.807123 |
| HOXA7 | 0.599735 | 0.315361 | -1.71612 | 0.416849 |
| STEAP3 | 0.599763 | 0.315422 | -1.03108 | 1.792794 |
| SNORD93 | 0.599784 | 0.315516 | 1.076903 | 2.558733 |
| MCRIP2 | 0.599784 | 0.315535 | 0.740602 | 2.874912 |
| RUSC2 | 0.599784 | 0.315607 | -1.01815 | 1.119358 |
| IGFL4 | 0.599784 | 0.315658 | 1.509214 | 1.756667 |
| FMO2 | 0.599784 | 0.315661 | -1.0309 | 2.798975 |
| FBXL13 | 0.600425 | 0.316044 | 1.051084 | 0.975824 |
| FAM43A | 0.600724 | 0.316248 | -0.55592 | 5.134059 |
| UFL1 | 0.600732 | 0.316298 | -0.36007 | 7.118828 |
| TPTEP1 | 0.601025 | 0.316498 | 1.14486 | 1.286172 |
| SBF2-AS1 | 0.601025 | 0.316544 | -0.80343 | 2.743531 |
| DICER1-AS1 | 0.601408 | 0.316791 | -1.04099 | 1.69603 |
| CSRNP1 | 0.601427 | 0.316847 | -0.50935 | 5.960587 |
| AJUBA | 0.601718 | 0.317048 | -1.09592 | 2.02099 |
| HIST2H2BE | 0.601718 | 0.317092 | -0.44958 | 5.184594 |
| CDH22 | 0.601959 | 0.317265 | 1.152385 | 1.801781 |
| OSMR | 0.601964 | 0.317319 | 0.47161 | 6.080134 |
| RIPOR1 | 0.601964 | 0.317359 | 0.837524 | 1.582891 |
| SDF2L1 | 0.602717 | 0.317802 | 0.644012 | 3.36182 |
| LYNX1 | 0.602721 | 0.31785 | -1.26764 | 1.390089 |
| PHYKPL | 0.602823 | 0.317959 | 0.988043 | 2.045451 |
| LINC00511 | 0.602823 | 0.317997 | 1.256668 | 0.729397 |
| GPR82 | 0.602823 | 0.318042 | 0.522005 | 3.778082 |
| DEUP1 | 0.60334 | 0.318401 | 1.415924 | 1.425186 |
| ZBTB4 | 0.60334 | 0.318406 | -0.84243 | 2.518689 |
| CTBP1-DT | 0.603622 | 0.318601 | 0.548528 | 3.289823 |
| TPR | 0.60422 | 0.318963 | 0.355711 | 9.054132 |
| SERINC4 | 0.604605 | 0.319227 | -0.82585 | 1.767661 |
| WDR73 | 0.604605 | 0.319258 | 0.81836 | 1.713317 |

| | | | | |
|---|---|---|---|---|
| PCED1A | 0.604649 | 0.31935 | 0.627134 | 2.987106 |
| CORO1C | 0.604649 | 0.319425 | -0.92727 | 1.866776 |
| FOXCUT | 0.604649 | 0.319504 | -1.25704 | 0.729228 |
| MGAM | 0.604649 | 0.319514 | 1.462794 | 0.806874 |
| ZNF283 | 0.604649 | 0.31956 | 1.237773 | 0.72156 |
| TSSK6 | 0.604649 | 0.319614 | 1.104156 | 0.851841 |
| DLX6-AS1 | 0.604649 | 0.319616 | 1.873012 | 0.908875 |
| GAB2 | 0.604649 | 0.31965 | 0.692582 | 2.619981 |
| WSB1 | 0.604943 | 0.319865 | -0.40361 | 5.582852 |
| TRMT13 | 0.604943 | 0.319898 | -0.39455 | 4.853095 |
| NLRP9 | 0.604952 | 0.319949 | 1.057662 | 2.079718 |
| RGCC | 0.604981 | 0.320038 | 0.698559 | 3.677043 |
| KLF17 | 0.604981 | 0.320056 | 1.560181 | 0.803689 |
| RAB6A | 0.605149 | 0.320227 | -0.92615 | 1.69485 |
| C9orf85 | 0.605149 | 0.320237 | -0.75276 | 3.784246 |
| LOC10013( | 0.605274 | 0.32035 | 0.597157 | 3.791716 |
| LOC10272( | 0.605457 | 0.320492 | 0.739656 | 1.931826 |
| SHROOM1 | 0.605524 | 0.32058 | 1.038394 | 0.663072 |
| DPY30 | 0.605524 | 0.320655 | -0.45718 | 4.421797 |
| CPSF1 | 0.605524 | 0.320667 | 0.378712 | 6.461091 |
| BMPR2 | 0.606017 | 0.321016 | 0.361088 | 8.017482 |
| FOXO3B | 0.606017 | 0.321113 | -0.37747 | 6.011625 |
| SNORA30 | 0.606017 | 0.321127 | -0.84624 | 2.576235 |
| GTF2A2 | 0.606017 | 0.321221 | 0.56453 | 4.362047 |
| PTPN11 | 0.606017 | 0.321235 | 0.3732 | 7.254089 |
| ITPRIPL2 | 0.606017 | 0.321259 | 0.539488 | 3.175999 |
| PDP2 | 0.606017 | 0.321317 | -0.46257 | 4.831936 |
| DYNLL2 | 0.606017 | 0.321342 | -0.39749 | 5.863866 |
| GIGYF2 | 0.606017 | 0.321343 | -1.2249 | 1.890343 |
| GDI1 | 0.606245 | 0.32151 | 0.349678 | 6.54562 |
| FBXO8 | 0.606354 | 0.32164 | -0.53015 | 4.378438 |
| JADE2 | 0.606354 | 0.321689 | 1.080724 | 1.385206 |
| PFDN2 | 0.606354 | 0.321707 | 0.421235 | 5.24537 |
| FUNDC2 | 0.606608 | 0.321888 | 0.410946 | 4.844433 |
| SLC2A4 | 0.606867 | 0.322071 | 0.945762 | 3.501573 |
| GOLGA7B | 0.606903 | 0.322137 | -0.7846 | 3.392864 |
| HOMEZ | 0.606905 | 0.322184 | -0.4309 | 4.893832 |
| MRPL28 | 0.607318 | 0.322519 | -0.47727 | 4.237088 |
| PYGB | 0.607318 | 0.322558 | 0.39215 | 7.761652 |
| PRMT2 | 0.607318 | 0.322607 | 0.380355 | 5.928469 |
| SNORD36C | 0.607318 | 0.322617 | 0.91767 | 3.004626 |
| NEMP2 | 0.607318 | 0.322661 | 0.476146 | 4.176583 |
| ESRG | 0.607318 | 0.322681 | -0.80079 | 1.606003 |
| SNORD101 | 0.607927 | 0.323065 | -0.63542 | 2.449346 |
| TBC1D13 | 0.607927 | 0.323097 | -1.11394 | 1.883469 |

| | | | | |
|---|---|---|---|---|
| MIR4677 | 0.608371 | 0.323379 | -1.64002 | 0.593734 |
| OR13D1 | 0.608669 | 0.323585 | -1.42544 | 0.5354 |
| SCX | 0.609011 | 0.323812 | 0.791747 | 2.36608 |
| RASSF9 | 0.609172 | 0.323952 | -0.73394 | 4.275062 |
| BBS5 | 0.609172 | 0.323991 | 0.680703 | 3.560589 |
| SRSF11 | 0.609336 | 0.324125 | -1.80175 | 0.588975 |
| BLOC1S4 | 0.609905 | 0.324474 | -0.58349 | 2.670834 |
| ZNF554 | 0.609912 | 0.324539 | -0.51651 | 3.82184 |
| SEMA5A-A: | 0.609912 | 0.324571 | -0.95838 | 1.941929 |
| HSD11B2 | 0.610215 | 0.324809 | 0.670162 | 3.921968 |
| LOC285819 | 0.610215 | 0.324825 | 1.150839 | 0.658449 |
| FICD | 0.610257 | 0.324894 | 0.780435 | 2.23951 |
| LDHAL6B | 0.61033 | 0.324979 | 1.016264 | 1.865613 |
| HPX | 0.610351 | 0.325046 | -1.15946 | 0.991959 |
| IRS1 | 0.610351 | 0.325083 | 0.458754 | 6.398684 |
| CEP152 | 0.610687 | 0.325309 | 1.105166 | 0.946834 |
| CDC42SE1 | 0.610928 | 0.325484 | -0.69393 | 2.378753 |
| SRPRB | 0.611291 | 0.325723 | -0.43657 | 5.680175 |
| ANKS1B | 0.611346 | 0.32587 | 0.749734 | 2.605412 |
| LOC399815 | 0.611346 | 0.325888 | 1.248361 | 0.945283 |
| RPL23A | 0.611346 | 0.325914 | -0.40082 | 7.696539 |
| PPM1M | 0.611346 | 0.325939 | 1.182638 | 0.668433 |
| EXOC3L1 | 0.611586 | 0.326122 | 1.146903 | 1.37345 |
| NPTN-IT1 | 0.611586 | 0.326172 | -0.42123 | 4.846303 |
| FXR1 | 0.611586 | 0.326207 | 1.225331 | 0.511268 |
| AKT1S1 | 0.611866 | 0.326403 | 0.872094 | 2.450772 |
| SPATA1 | 0.611892 | 0.326505 | 0.70721 | 2.782654 |
| LOC153684 | 0.611892 | 0.326513 | 0.755217 | 3.078657 |
| PJVK | 0.611892 | 0.326557 | 1.143607 | 2.024819 |
| DGKZ | 0.612063 | 0.326731 | 0.798187 | 1.361674 |
| JUNB | 0.612063 | 0.326742 | -0.48045 | 7.998998 |
| TBC1D31 | 0.612382 | 0.326981 | 1.284254 | 1.035909 |
| STMND1 | 0.612382 | 0.327005 | -1.74899 | 0.792167 |
| UBE2D2 | 0.612439 | 0.327082 | -0.64835 | 2.538105 |
| PAFAH1B2 | 0.612511 | 0.327167 | -1.28472 | 0.815186 |
| SAMD5 | 0.613075 | 0.327554 | -0.66236 | 6.902348 |
| PLXNA1 | 0.613075 | 0.327562 | 0.408235 | 7.259384 |
| FBXO17 | 0.613346 | 0.327776 | 1.34824 | 0.623798 |
| GTF2H5 | 0.613346 | 0.3278 | -0.48506 | 4.025266 |
| CEP41 | 0.613499 | 0.327929 | 1.462997 | 0.510924 |
| MOSMO | 0.613681 | 0.328073 | 0.447851 | 4.793602 |
| CTAGE7P | 0.613818 | 0.328193 | 1.002492 | 1.572627 |
| TXLNA | 0.614108 | 0.328395 | 0.34988 | 7.195266 |
| ASCC1 | 0.614128 | 0.328452 | 0.862794 | 2.070478 |
| TMCC1 | 0.614501 | 0.328699 | -0.50505 | 3.666298 |

| | | | | |
|---|---|---|---|---|
| SLC31A2 | 0.614737 | 0.328874 | -0.47494 | 4.284913 |
| LINC00513 | 0.614737 | 0.328976 | 0.518228 | 4.2981 |
| GPR3 | 0.614737 | 0.329043 | 1.026366 | 1.290849 |
| AP4M1 | 0.614737 | 0.329046 | 0.538518 | 3.786824 |
| BSG | 0.614737 | 0.329059 | -0.91477 | 1.272565 |
| DHX57 | 0.615128 | 0.329389 | 0.432807 | 5.328607 |
| MIATNB | 0.615128 | 0.329404 | 0.692014 | 3.187049 |
| RPRD2 | 0.615128 | 0.329431 | 1.495069 | 0.505048 |
| HIF3A | 0.615128 | 0.329456 | 1.988807 | 0.842066 |
| LOC10028‹ | 0.615156 | 0.329534 | -0.73862 | 2.621885 |
| RBP5 | 0.615156 | 0.329608 | 0.536669 | 3.971646 |
| EMC4 | 0.615156 | 0.329612 | -0.79876 | 2.144244 |
| LOC100131 | 0.61524 | 0.329703 | 0.97164 | 3.119335 |
| BCRP3 | 0.615448 | 0.329886 | 1.443012 | 1.283064 |
| ZNF813 | 0.615448 | 0.329909 | -0.59912 | 3.640115 |
| SLC25A37 | 0.615902 | 0.330199 | 0.690815 | 3.43647 |
| PSMD2 | 0.616024 | 0.330317 | -0.35104 | 5.441178 |
| GPR35 | 0.616024 | 0.330361 | 1.290965 | 1.012284 |
| ACVR1B | 0.616024 | 0.330405 | -0.74701 | 2.535017 |
| NRSN2-AS1 | 0.61614 | 0.330516 | 0.78936 | 2.470191 |
| ARSD | 0.61614 | 0.330561 | -0.5165 | 5.907393 |
| MFSD4A-A: | 0.616223 | 0.330653 | 1.376193 | 1.238471 |
| MAPK8IP1 | 0.616456 | 0.33085 | 0.732099 | 3.198696 |
| LINC01364 | 0.616456 | 0.330872 | 1.047408 | 1.365544 |
| PWWP2B | 0.616706 | 0.331077 | 0.770339 | 2.090486 |
| ZNF230 | 0.616706 | 0.3311 | 0.691304 | 3.10772 |
| DLG4 | 0.61695 | 0.331278 | -1.03546 | 1.710477 |
| PCCA-AS1 | 0.616999 | 0.331351 | -0.76905 | 2.583829 |
| QPCTL | 0.617101 | 0.331453 | -1.26743 | 0.469053 |
| FITM2 | 0.617279 | 0.331608 | 0.678276 | 2.275668 |
| MEG9 | 0.617279 | 0.331703 | 1.231539 | 0.621718 |
| RAB27A | 0.617279 | 0.331709 | -0.74452 | 2.30733 |
| ANKRD24 | 0.617279 | 0.331743 | 0.909265 | 2.682707 |
| CCNC | 0.617279 | 0.331826 | -0.64587 | 2.669574 |
| EIF4EBP2 | 0.617279 | 0.331831 | -0.35259 | 8.334015 |
| CITED4 | 0.617519 | 0.332014 | -0.57859 | 3.362707 |
| ERVK13-1 | 0.617519 | 0.332054 | 0.3969 | 6.653738 |
| FAM98C | 0.617614 | 0.332152 | -0.45801 | 4.037768 |
| IL10RB-DT | 0.617686 | 0.332238 | 1.137975 | 1.217898 |
| NAPG | 0.6177 | 0.332293 | 0.440794 | 6.420941 |
| ALMS1-IT1 | 0.617709 | 0.332387 | 0.73133 | 2.679416 |
| PTP4A2 | 0.617709 | 0.332391 | 0.546182 | 4.51361 |
| XPO5 | 0.618027 | 0.33261 | -0.37746 | 6.531225 |
| NTN1 | 0.618097 | 0.332726 | 0.516221 | 5.797363 |
| RDH16 | 0.618097 | 0.332742 | -1.2518 | 0.711109 |

| | | | | |
|---|---|---|---|---|
| CEACAM22 | 0.61865 | 0.333105 | 1.575631 | 0.604812 |
| MCF2L | 0.61865 | 0.333167 | 1.056354 | 1.517698 |
| HSP90AB4I | 0.61865 | 0.33322 | 0.75752 | 3.782191 |
| DNAAF1 | 0.61865 | 0.333228 | 0.972437 | 0.424507 |
| SPEM2 | 0.618843 | 0.333379 | 2.262756 | 0.29433 |
| CRYGS | 0.618853 | 0.333432 | -1.17754 | 0.859438 |
| ZBED3 | 0.619071 | 0.333596 | 0.74086 | 2.132471 |
| PGBD2 | 0.619161 | 0.333692 | 0.898878 | 1.312804 |
| VCPIP1 | 0.619547 | 0.333947 | 0.354504 | 6.927236 |
| MBTD1 | 0.619881 | 0.334174 | -0.37761 | 6.196826 |
| ATG14 | 0.620127 | 0.334354 | -0.35454 | 5.980501 |
| C19orf25 | 0.620142 | 0.334454 | 0.568476 | 4.115896 |
| SNORA63 | 0.620142 | 0.334485 | 0.470038 | 7.703284 |
| LOC101928 | 0.620142 | 0.334504 | -0.71997 | 2.678837 |
| SKIV2L | 0.620165 | 0.33466 | -0.69775 | 2.344723 |
| RNF31 | 0.620165 | 0.334671 | 0.604976 | 2.815287 |
| OR5C1 | 0.620165 | 0.334709 | -1.3686 | 0.992008 |
| FANCD2 | 0.620165 | 0.334736 | 1.032573 | 1.497111 |
| TM2D1 | 0.620165 | 0.334753 | -0.36572 | 5.368987 |
| PCLO | 0.620343 | 0.334897 | 1.332267 | 1.056889 |
| ATP5MGL | 0.620567 | 0.335064 | -1.0324 | 1.315045 |
| PDS5A | 0.620624 | 0.335142 | -0.37989 | 7.630625 |
| PCSK1N | 0.620721 | 0.335243 | 1.595188 | 1.815826 |
| FRMD3 | 0.620721 | 0.335289 | 1.426831 | 1.370513 |
| TARBP1 | 0.62171 | 0.335871 | 0.446238 | 6.487276 |
| PLEKHA1 | 0.62173 | 0.335931 | -0.92534 | 1.705811 |
| INTS12 | 0.62173 | 0.335977 | 1.063249 | 1.263472 |
| ZNF71 | 0.62182 | 0.336105 | 0.545088 | 3.181404 |
| LOC100507 | 0.62182 | 0.33612 | 0.843879 | 2.911935 |
| JMJD1C | 0.621845 | 0.336195 | 0.487948 | 4.022804 |
| HTR5A-AS1 | 0.621845 | 0.336228 | -2.06166 | 1.373131 |
| TRIM28 | 0.621882 | 0.336341 | 0.34449 | 7.02015 |
| MAP3K4 | 0.621882 | 0.336343 | -0.73517 | 3.164801 |
| TRIM62 | 0.622271 | 0.336601 | 0.471009 | 3.981068 |
| LOC284581 | 0.622713 | 0.336887 | 0.913493 | 3.018021 |
| ZNF503-AS | 0.622821 | 0.337022 | -1.01077 | 1.050567 |
| CYCSP52 | 0.622821 | 0.337062 | 1.370322 | 0.66743 |
| CRTC3-AS1 | 0.622821 | 0.337157 | 1.271044 | 0.738188 |
| OLFM4 | 0.622821 | 0.337257 | 1.462271 | 5.632074 |
| PYROXD2 | 0.622821 | 0.337264 | 0.600881 | 3.004937 |
| BCAR1 | 0.622821 | 0.337305 | 0.683281 | 2.541722 |
| ARHGAP39 | 0.622821 | 0.337314 | -0.90993 | 1.760823 |
| AKR1C6P | 0.622821 | 0.337326 | -1.74067 | 1.059044 |
| SNHG22 | 0.622966 | 0.337507 | -0.75325 | 3.071138 |
| DIP2A-IT1 | 0.622966 | 0.337514 | -0.98595 | 1.726617 |

| | | | | |
|---|---|---|---|---|
| ITGA9-AS1 | 0.622966 | 0.337547 | 1.652307 | 1.052405 |
| IFRD1 | 0.623061 | 0.337646 | 0.763653 | 1.847244 |
| KIAA1522 | 0.62325 | 0.337796 | -0.81656 | 1.771762 |
| EIF3D | 0.623283 | 0.337861 | -0.37416 | 7.785478 |
| SLC35B3 | 0.623294 | 0.337915 | 1.353029 | 0.517302 |
| MIR3916 | 0.623464 | 0.338054 | 0.49389 | 3.850808 |
| CSNK1G2-A | 0.62373 | 0.338246 | 0.730109 | 1.683184 |
| FEM1A | 0.624012 | 0.338461 | -0.36754 | 5.661451 |
| CPLANE2 | 0.624012 | 0.338494 | -0.88703 | 2.291424 |
| AZGP1 | 0.62423 | 0.338766 | -1.5221 | 5.25219 |
| RPL23AP82 | 0.62423 | 0.338811 | -1.06701 | 0.481325 |
| SLC35E4 | 0.62423 | 0.338821 | 0.937024 | 3.054415 |
| UBAC1 | 0.62423 | 0.338872 | -0.36774 | 5.37821 |
| MEGF11 | 0.62423 | 0.338883 | -0.52098 | 3.568497 |
| ELFN1 | 0.62423 | 0.338898 | 1.380554 | 0.710558 |
| SLC8B1 | 0.624267 | 0.338966 | 0.445336 | 5.314204 |
| MIR4442 | 0.624473 | 0.339125 | -0.7426 | 1.885939 |
| RPS4X | 0.624726 | 0.33931 | -0.40077 | 9.824022 |
| SNTB1 | 0.625207 | 0.339619 | 0.781976 | 5.181173 |
| BOP1 | 0.625302 | 0.339737 | 0.398005 | 5.545056 |
| PAXX | 0.625302 | 0.339787 | 0.480327 | 3.459147 |
| ZNF614 | 0.625302 | 0.339813 | 0.487449 | 3.841813 |
| PFN4 | 0.625549 | 0.340021 | 1.325174 | 0.807579 |
| TSSC2 | 0.625549 | 0.340042 | 1.141088 | 1.522922 |
| PRNCR1 | 0.625686 | 0.340165 | -0.33754 | 5.735129 |
| TOR1AIP2 | 0.626546 | 0.34068 | -0.35637 | 6.496237 |
| DAPK2 | 0.626581 | 0.340747 | -0.66486 | 3.536674 |
| HERC2P10 | 0.626657 | 0.340871 | 1.402737 | 0.411072 |
| RBM45 | 0.626657 | 0.340884 | 0.614247 | 3.239335 |
| MECOM | 0.627458 | 0.341367 | 0.509981 | 5.26533 |
| RDH14 | 0.627469 | 0.341465 | 0.840982 | 1.343107 |
| ZNF251 | 0.627469 | 0.341469 | 0.881345 | 2.023989 |
| PFKFB2 | 0.627557 | 0.341565 | -0.40214 | 6.623816 |
| TRMT5 | 0.62829 | 0.342174 | -0.5817 | 4.466451 |
| WDR66 | 0.62829 | 0.342227 | -1.21997 | 0.882518 |
| NTN3 | 0.62829 | 0.342229 | 0.888027 | 1.308996 |
| DIRC2 | 0.62829 | 0.342237 | -0.41031 | 5.68536 |
| TMEM164 | 0.62829 | 0.342248 | 0.554345 | 3.311686 |
| RPIA | 0.62829 | 0.342251 | -0.40784 | 4.787904 |
| TAF7 | 0.628492 | 0.342409 | 0.420726 | 7.017933 |
| AKR1C1 | 0.628557 | 0.342492 | -0.61709 | 5.040829 |
| PYM1 | 0.628669 | 0.342622 | -1.00588 | 1.266208 |
| OR7D2 | 0.628669 | 0.342649 | -1.59269 | 1.163187 |
| SARAF | 0.628742 | 0.342737 | 0.863416 | 1.886253 |
| PRRG2 | 0.628745 | 0.342799 | -1.63625 | 0.436513 |

| | | | | |
|---|---|---|---|---|
| DHRS7B | 0.628745 | 0.342834 | 0.594104 | 3.597622 |
| EIF3J-DT | 0.629259 | 0.343239 | 1.192192 | 0.717919 |
| SPACA6P-A | 0.629259 | 0.343269 | 0.989515 | 1.226693 |
| HTATSF1P2 | 0.629259 | 0.343299 | 0.695465 | 3.54272 |
| PLA2G6 | 0.629259 | 0.343306 | -1.19534 | 1.028629 |
| FLOT2 | 0.629281 | 0.343421 | -0.3846 | 6.49486 |
| ENTPD7 | 0.629281 | 0.343431 | 0.372736 | 6.299204 |
| MON1A | 0.629281 | 0.343528 | 1.086758 | 0.744268 |
| COA5 | 0.629281 | 0.34355 | 0.409452 | 4.825877 |
| CEP250 | 0.629281 | 0.343558 | 0.397169 | 7.611683 |
| TXNL4B | 0.629392 | 0.343667 | -1.16796 | 1.159824 |
| SRF | 0.629456 | 0.34375 | 0.433043 | 4.582726 |
| INHBE | 0.629563 | 0.34389 | 1.465359 | 0.977561 |
| ABT1 | 0.629563 | 0.343904 | -0.50705 | 4.42616 |
| GAL3ST1 | 0.629624 | 0.343985 | 1.438163 | 0.708353 |
| ZNF106 | 0.629869 | 0.344167 | 0.350753 | 6.729269 |
| LRRC19 | 0.629891 | 0.344227 | 1.033757 | 2.082159 |
| TC2N | 0.630328 | 0.344604 | 1.105993 | 1.112649 |
| NTS | 0.630328 | 0.34461 | -1.64055 | 1.023391 |
| P2RY13 | 0.630328 | 0.34461 | 1.140651 | 1.108594 |
| VRK2 | 0.630455 | 0.344763 | -1.00143 | 2.071463 |
| ZNF852 | 0.630455 | 0.344776 | 0.492671 | 3.857052 |
| LINC02288 | 0.630647 | 0.344946 | 1.455143 | 0.973766 |
| RPS12 | 0.630647 | 0.344977 | -0.42151 | 8.629758 |
| BEND3P3 | 0.630776 | 0.345095 | 0.447048 | 5.156659 |
| GGT7 | 0.630815 | 0.345179 | 0.567807 | 3.747588 |
| LINC01137 | 0.630815 | 0.345213 | 0.772197 | 2.117292 |
| RETSAT | 0.630916 | 0.345316 | -0.3467 | 6.425578 |
| MIR548L | 0.630995 | 0.345439 | 1.456279 | 0.466023 |
| CLBA1 | 0.630995 | 0.345456 | -0.58654 | 3.16926 |
| ARHGAP28 | 0.631172 | 0.345648 | 0.671923 | 2.472466 |
| CBLN3 | 0.631172 | 0.345648 | 1.04701 | 1.563217 |
| LOC10028 | 0.631198 | 0.345711 | 0.886493 | 2.506554 |
| CFAP58 | 0.631412 | 0.345876 | -1.02932 | 1.483624 |
| BMS1 | 0.631787 | 0.34613 | 0.343083 | 7.503779 |
| ALKBH7 | 0.631974 | 0.346281 | -0.46525 | 3.714827 |
| RAB31 | 0.632541 | 0.346677 | 0.486808 | 6.818819 |
| BMP3 | 0.632541 | 0.346696 | 1.817725 | 1.114051 |
| MTMR9LP | 0.632541 | 0.346736 | 0.747512 | 3.163196 |
| LOC22194 | 0.632688 | 0.346865 | -0.71353 | 4.006578 |
| PICALM | 0.632864 | 0.347031 | 1.074426 | 1.009083 |
| ARFGEF1 | 0.632864 | 0.347128 | -0.3439 | 8.26678 |
| ZBTB20-AS | 0.632864 | 0.347135 | 0.669788 | 3.007665 |
| WWC3 | 0.632864 | 0.347154 | -0.38759 | 6.784008 |
| CDRT1 | 0.632907 | 0.347226 | -0.97927 | 1.327401 |

| | | | | |
|---|---|---|---|---|
| RYBP | 0.633339 | 0.347549 | -0.35311 | 5.966633 |
| PURA | 0.633339 | 0.347581 | 0.476211 | 6.783896 |
| PHF11 | 0.633339 | 0.347615 | 0.805151 | 2.425974 |
| TM9SF1 | 0.633339 | 0.347656 | -1.70972 | 0.90782 |
| TMEM199 | 0.633375 | 0.347724 | 0.405076 | 4.454303 |
| ATP7A | 0.63359 | 0.34789 | -1.2258 | 0.441339 |
| ATP6V1C2 | 0.6336 | 0.347944 | -1.30633 | 0.664761 |
| GATA5 | 0.633608 | 0.348045 | 1.690255 | 1.832098 |
| PPP1R26 | 0.633608 | 0.348088 | 0.372434 | 5.149762 |
| PLAA | 0.633608 | 0.348127 | -0.45003 | 5.927725 |
| CRKL | 0.633608 | 0.348141 | -0.34291 | 7.072392 |
| SHROOM2 | 0.633665 | 0.348221 | -0.61983 | 5.371738 |
| CPNE7 | 0.633827 | 0.348382 | -1.71165 | 0.489206 |
| KIF11 | 0.633827 | 0.348436 | 0.439492 | 5.907494 |
| PLPP6 | 0.633827 | 0.348455 | -0.43669 | 4.557829 |
| MFSD5 | 0.63394 | 0.348566 | -1.30501 | 0.98121 |
| PLET1 | 0.63408 | 0.348691 | -0.87375 | 2.644309 |
| XRCC1 | 0.634491 | 0.349018 | 0.408049 | 4.841484 |
| TPSB2 | 0.634491 | 0.349062 | -0.71073 | 2.242969 |
| PIBF1 | 0.634491 | 0.34909 | 0.354577 | 5.76888 |
| LYRM9 | 0.634491 | 0.34914 | 0.887003 | 2.395028 |
| QPCT | 0.634491 | 0.349159 | 1.191435 | 1.723547 |
| NCAPH2 | 0.634659 | 0.3493 | -0.88758 | 1.307835 |
| ZFAT-AS1 | 0.63467 | 0.349354 | -0.99039 | 0.631906 |
| CCDC171 | 0.634958 | 0.349621 | 0.537987 | 4.078943 |
| KIF18A | 0.634958 | 0.349643 | 0.796533 | 3.253931 |
| MIR3175 | 0.634958 | 0.349675 | 0.887409 | 1.538893 |
| CHD2 | 0.634958 | 0.349706 | -0.34679 | 9.012856 |
| LRRCC1 | 0.635071 | 0.349864 | 0.394963 | 4.868251 |
| HNRNPA3 | 0.635071 | 0.34991 | -0.35825 | 8.849099 |
| CCDC88C | 0.635071 | 0.349913 | -0.39292 | 7.03792 |
| SLFN5 | 0.635249 | 0.35006 | 0.394084 | 6.100646 |
| LINC01589 | 0.635698 | 0.350356 | 1.369649 | 1.007908 |
| GLUD1P3 | 0.635712 | 0.350412 | 0.757579 | 2.007669 |
| ERF | 0.635766 | 0.35049 | 1.139549 | 1.020153 |
| MGC16275 | 0.63593 | 0.350629 | -0.82318 | 1.366629 |
| FBN3 | 0.636182 | 0.350817 | 1.598879 | 0.884811 |
| SEC24A | 0.636208 | 0.350879 | -0.37027 | 5.949181 |
| SUMO1P3 | 0.636451 | 0.351062 | 0.466767 | 3.905173 |
| PRKX-AS1 | 0.636464 | 0.35115 | -1.15185 | 1.180885 |
| ZKSCAN4 | 0.636464 | 0.351166 | 0.912245 | 1.419986 |
| ADORA2A-/ | 0.636536 | 0.351254 | 0.946912 | 1.702706 |
| MIR1276 | 0.636993 | 0.351555 | -1.41287 | 0.490652 |
| COQ2 | 0.637008 | 0.351612 | -0.41483 | 4.195504 |
| MRPS16 | 0.637105 | 0.351714 | -0.3473 | 5.897145 |

| | | | | |
|---|---|---|---|---|
| PDXDC1 | 0.6375 | 0.352021 | 0.837321 | 2.326903 |
| ANOS1 | 0.6375 | 0.352041 | 0.646792 | 3.562598 |
| MIR640 | 0.6375 | 0.352078 | 1.08189 | 0.319176 |
| KIAA1147 | 0.637649 | 0.352281 | 0.950598 | 4.39634 |
| LOC10028٤ | 0.637649 | 0.352303 | 0.58654 | 3.149051 |
| LOC10192٤ | 0.637649 | 0.352306 | 0.657128 | 1.816811 |
| SMA4 | 0.638002 | 0.352552 | 0.444874 | 4.922203 |
| UBAP2L | 0.638002 | 0.352598 | 0.412616 | 4.461969 |
| ELL | 0.63809 | 0.352704 | 0.356194 | 5.558294 |
| AP5M1 | 0.63809 | 0.352744 | -0.69351 | 3.477858 |
| BRIP1 | 0.638175 | 0.352884 | 0.450803 | 4.512858 |
| DUS3L | 0.638175 | 0.352935 | -0.65308 | 2.053897 |
| FRS2 | 0.638175 | 0.352937 | 1.138228 | 0.651401 |
| FBRSL1 | 0.638256 | 0.353073 | 0.377292 | 6.07857 |
| CFAP300 | 0.638256 | 0.353079 | 1.179161 | 0.774276 |
| TAPBPL | 0.638465 | 0.353243 | 0.538038 | 4.082419 |
| GRSF1 | 0.638622 | 0.353379 | -0.7733 | 1.7255 |
| SNX32 | 0.640031 | 0.354207 | 0.837425 | 1.559581 |
| TBXAS1 | 0.640265 | 0.354411 | -1.53363 | 0.576593 |
| SPRR2F | 0.640265 | 0.354498 | -1.55346 | 4.375576 |
| LOC10192٤ | 0.640265 | 0.354507 | 0.86142 | 1.94333 |
| TUG1 | 0.640265 | 0.354532 | -0.7422 | 3.320546 |
| RPS5 | 0.640382 | 0.354645 | -0.36385 | 7.647228 |
| AGAP6 | 0.641195 | 0.355237 | 0.427558 | 4.543704 |
| DONSON | 0.641195 | 0.35524 | 0.428205 | 4.128862 |
| MAT2B | 0.641195 | 0.355273 | -0.74064 | 2.531793 |
| METTL6 | 0.641195 | 0.355294 | -1.07495 | 0.542837 |
| PRR16 | 0.641195 | 0.35534 | 1.453136 | 0.925608 |
| WIZ | 0.641767 | 0.355706 | 0.33641 | 5.620957 |
| GLTPD2 | 0.641804 | 0.35582 | -0.82888 | 2.361832 |
| SLC6A17 | 0.641804 | 0.355824 | 1.201314 | 0.957109 |
| SNORA49 | 0.641943 | 0.356043 | 0.418608 | 7.892361 |
| USP53 | 0.641943 | 0.356128 | -0.37897 | 7.853249 |
| S100A6 | 0.641943 | 0.35615 | 0.454253 | 9.694052 |
| INTS11 | 0.641943 | 0.356173 | 1.146322 | 0.934435 |
| CSNK1A1 | 0.641943 | 0.356179 | 1.203235 | 1.593636 |
| CPT1A | 0.641943 | 0.356195 | 0.361016 | 5.943221 |
| SPA17 | 0.641985 | 0.356267 | 0.960118 | 1.834187 |
| ITIH4 | 0.642009 | 0.356329 | 1.43498 | 0.328106 |
| FAM227B | 0.642282 | 0.356641 | 0.97312 | 1.360089 |
| MIR573 | 0.642282 | 0.35665 | -0.96389 | 1.039228 |
| ZNF134 | 0.642282 | 0.356675 | -0.54962 | 3.89497 |
| ATF5 | 0.642282 | 0.35668 | 0.839725 | 1.803698 |
| POLB | 0.642282 | 0.356725 | -0.52159 | 4.523711 |
| SHANK2-AS | 0.642417 | 0.356849 | -0.82478 | 1.926529 |

| | | | | |
|---|---|---|---|---|
| CASTOR2 | 0.642562 | 0.357002 | 0.443275 | 5.645086 |
| DMWD | 0.642562 | 0.357067 | 0.33853 | 5.999408 |
| LOC440028 | 0.642562 | 0.357082 | 1.048143 | 1.445699 |
| C5orf22 | 0.642562 | 0.357131 | 1.128031 | 1.019064 |
| HHIP | 0.642562 | 0.357174 | 1.131811 | 2.450324 |
| SEMA4C | 0.642763 | 0.357338 | 0.39268 | 5.191315 |
| KLRC4 | 0.642763 | 0.357384 | 1.340464 | 0.73501 |
| SOWAHA | 0.642955 | 0.35754 | -1.30122 | 0.753205 |
| LRRK1 | 0.64297 | 0.357597 | 0.371131 | 6.048468 |
| TMEM147- | 0.643043 | 0.357796 | -0.5589 | 3.026962 |
| CDC26 | 0.643043 | 0.357807 | 0.458297 | 4.13616 |
| TAS2R3 | 0.643043 | 0.357827 | 0.932151 | 1.526534 |
| MZF1-AS1 | 0.643043 | 0.357854 | 0.467363 | 4.315739 |
| TAF3 | 0.643043 | 0.357883 | 0.360484 | 5.566743 |
| IFNK | 0.643053 | 0.357938 | 0.704102 | 2.569549 |
| SLC5A6 | 0.643362 | 0.358217 | 0.900353 | 0.964743 |
| RPS29 | 0.643362 | 0.35823 | -0.64186 | 3.276968 |
| CANT1 | 0.643362 | 0.358257 | -1.16295 | 1.585063 |
| PNO1 | 0.643487 | 0.358412 | 0.476076 | 4.54937 |
| PTPRB | 0.643487 | 0.358469 | 1.318381 | 0.811534 |
| BBIP1 | 0.643487 | 0.358473 | -0.95677 | 1.789752 |
| ECH1 | 0.643524 | 0.358543 | -0.35495 | 6.529629 |
| QRFP | 0.643672 | 0.358675 | 1.246468 | 0.352384 |
| FBXL14 | 0.644242 | 0.359042 | 0.498499 | 3.933092 |
| RAB18 | 0.644321 | 0.359135 | -0.93328 | 2.48525 |
| SNORD24 | 0.64458 | 0.359363 | 0.557287 | 3.312863 |
| THAP12 | 0.64458 | 0.359377 | 1.328662 | 0.711651 |
| TBRG1 | 0.644735 | 0.359513 | 0.661364 | 2.071336 |
| MATN1-AS | 0.645133 | 0.359784 | 1.031459 | 1.036002 |
| LINC01348 | 0.645782 | 0.360195 | -1.25033 | 1.255879 |
| PLD3 | 0.64609 | 0.360416 | -0.85386 | 1.264105 |
| SLC25A35 | 0.646095 | 0.360469 | -0.83005 | 1.874031 |
| LRP4-AS1 | 0.646095 | 0.36055 | 1.404362 | 1.528742 |
| CCDC152 | 0.646095 | 0.360566 | 1.071301 | 1.669323 |
| H6PD | 0.646193 | 0.36067 | -0.76764 | 2.310317 |
| CD63 | 0.64622 | 0.360735 | 0.803225 | 1.604436 |
| C1QL3 | 0.646434 | 0.360903 | 0.986091 | 1.918432 |
| PANO1 | 0.646843 | 0.361181 | -1.42733 | 0.831157 |
| SPIRE1 | 0.647204 | 0.361483 | -1.20526 | 1.221793 |
| MPO | 0.647204 | 0.36152 | -1.8334 | 1.019721 |
| NUDT15 | 0.647204 | 0.361531 | 0.451833 | 4.216488 |
| TTN-AS1 | 0.647328 | 0.36167 | -0.76386 | 2.717044 |
| PRMT9 | 0.647328 | 0.361728 | 1.110264 | 0.36578 |
| GTF2IRD2B | 0.647328 | 0.361748 | 0.58985 | 4.296438 |
| RNF216P1 | 0.647552 | 0.361923 | 0.740282 | 2.23359 |

| | | | | |
|---|---|---|---|---|
| MIR1304 | 0.64763 | 0.362019 | -1.17116 | 1.366859 |
| TMED9 | 0.64763 | 0.362065 | -0.37676 | 6.987489 |
| MYO18A | 0.647764 | 0.362189 | 1.244602 | 0.829058 |
| FAM81B | 0.647832 | 0.362277 | 1.498762 | 0.702737 |
| SNORA4 | 0.647986 | 0.362412 | 0.656678 | 4.131432 |
| SLC25A2 | 0.648458 | 0.362743 | 1.24234 | 1.12645 |
| SNORA24 | 0.648458 | 0.362882 | -0.5438 | 7.132157 |
| RNF165 | 0.648458 | 0.362927 | -1.34675 | 0.612597 |
| ZSWIM1 | 0.648458 | 0.362937 | 0.387815 | 4.348515 |
| KAT6B | 0.648458 | 0.362942 | 0.561949 | 3.836069 |
| TRAPPC8 | 0.648458 | 0.362972 | -0.36607 | 7.119775 |
| SLC7A11 | 0.648473 | 0.36303 | 0.464516 | 5.546648 |
| C16orf72 | 0.648564 | 0.363198 | -0.31488 | 7.012378 |
| C12orf76 | 0.648564 | 0.363212 | 0.918089 | 1.751893 |
| GK5 | 0.648564 | 0.363229 | 0.583709 | 3.121837 |
| KIF5B | 0.648878 | 0.363455 | 0.355432 | 9.073591 |
| LMAN1 | 0.649165 | 0.363665 | -0.40484 | 8.224326 |
| TOMM20 | 0.649409 | 0.363851 | 0.361916 | 7.492371 |
| KCND2 | 0.649674 | 0.364058 | -1.08026 | 2.359074 |
| AGPAT4-IT | 0.649674 | 0.364099 | -0.66005 | 3.155679 |
| NAV1 | 0.649709 | 0.364203 | 0.517269 | 3.235552 |
| PTPRF | 0.649709 | 0.364218 | -1.02925 | 0.531472 |
| FRMD4A | 0.649715 | 0.36427 | -0.37129 | 6.299389 |
| THAP4 | 0.650418 | 0.364714 | -0.37007 | 4.592929 |
| DHX37 | 0.650445 | 0.364779 | 0.360852 | 5.345357 |
| P3H2 | 0.650518 | 0.364931 | -0.99222 | 1.831634 |
| PDZRN3-AS | 0.650518 | 0.364991 | 0.833456 | 1.904464 |
| SNORD83B | 0.650518 | 0.365012 | 0.833885 | 3.253709 |
| RUFY2 | 0.650518 | 0.365056 | -0.63008 | 3.8552 |
| TMED2 | 0.650518 | 0.365067 | -0.35204 | 8.532522 |
| BCAM | 0.650571 | 0.365147 | -0.60872 | 3.167607 |
| KCTD13 | 0.650672 | 0.365253 | 1.505983 | 0.564541 |
| CA14 | 0.650918 | 0.365459 | -0.97173 | 1.440546 |
| PRH2 | 0.650918 | 0.365498 | 1.1966 | 0.357971 |
| ALCAM | 0.650918 | 0.36554 | 1.054477 | 2.252999 |
| LINC02611 | 0.650994 | 0.365657 | 1.333966 | 0.629326 |
| HACD4 | 0.650994 | 0.365682 | 0.744516 | 2.758379 |
| TDG | 0.651026 | 0.365749 | -0.445 | 5.701646 |
| IQCB1 | 0.651056 | 0.365816 | 0.51349 | 3.055291 |
| TUFT1 | 0.651453 | 0.366088 | -1.34752 | 1.243146 |
| TEX10 | 0.651491 | 0.36616 | 1.180963 | 0.601387 |
| WNK4 | 0.651801 | 0.366384 | -0.56309 | 5.062119 |
| SNORA68 | 0.651846 | 0.366459 | 0.444541 | 7.714894 |
| HIST1H2BC | 0.652118 | 0.366706 | -0.40683 | 6.585842 |
| FRG1JP | 0.652118 | 0.366711 | -0.69457 | 2.932826 |

| | | | | |
|---|---|---|---|---|
| DFFA | 0.652229 | 0.366823 | 0.633579 | 2.765854 |
| LPCAT2 | 0.652243 | 0.366902 | -0.44689 | 5.272464 |
| ATE1-AS1 | 0.652243 | 0.366993 | 1.323352 | 0.792201 |
| GRIK3 | 0.652243 | 0.367006 | 1.051827 | 1.6314 |
| TPP2 | 0.652243 | 0.367079 | 0.342514 | 7.055637 |
| YAF2 | 0.652243 | 0.367079 | 0.434348 | 4.638355 |
| SNHG8 | 0.652448 | 0.367244 | -1.05189 | 0.812343 |
| RPL22L1 | 0.65267 | 0.367419 | 0.574365 | 5.424175 |
| RPGR | 0.653243 | 0.367814 | 0.442318 | 5.353935 |
| NPY6R | 0.653243 | 0.367841 | 0.778213 | 2.255944 |
| CRIPAK | 0.653388 | 0.367973 | -0.46557 | 4.826597 |
| SNORD45C | 0.653591 | 0.368137 | 0.491017 | 6.607433 |
| PLEKHF1 | 0.653913 | 0.368402 | -0.66846 | 3.033189 |
| THAP9-AS1 | 0.653913 | 0.368418 | -0.66536 | 2.782155 |
| TMEM160 | 0.653974 | 0.368502 | 0.723562 | 2.596203 |
| FRMPD3 | 0.654328 | 0.368753 | 1.192974 | 1.30741 |
| USP46-AS1 | 0.654328 | 0.368801 | -0.55387 | 2.615343 |
| TP53I11 | 0.654351 | 0.368864 | 1.205384 | 1.109059 |
| DHX9 | 0.654424 | 0.368955 | 0.809294 | 2.03366 |
| HIVEP1 | 0.654867 | 0.369255 | -0.35014 | 7.120636 |
| ZNF547 | 0.655041 | 0.369414 | 0.830231 | 1.876372 |
| ANAPC1 | 0.655041 | 0.369453 | 0.341649 | 5.929256 |
| LOC100272 | 0.655064 | 0.369538 | -0.93494 | 1.000404 |
| CRSP8P | 0.655064 | 0.369575 | 0.911011 | 1.51733 |
| NOC3L | 0.655064 | 0.369655 | 0.35526 | 5.982749 |
| TCP10L | 0.655064 | 0.369686 | -1.23822 | 1.69286 |
| LOC100506 | 0.655064 | 0.369715 | 0.776192 | 2.08233 |
| SHC4 | 0.655413 | 0.370009 | 1.121099 | 0.839948 |
| SIK2 | 0.655413 | 0.37005 | 0.358899 | 6.590855 |
| ZNF510 | 0.655413 | 0.370062 | 1.214994 | 0.583753 |
| ATP13A3 | 0.655443 | 0.370129 | 0.383772 | 7.709972 |
| GSTM4 | 0.656146 | 0.370576 | 1.376983 | 0.882544 |
| SPTA1 | 0.656324 | 0.370726 | 1.399826 | 0.998182 |
| GRIN3B | 0.656386 | 0.370829 | -0.59933 | 2.396888 |
| LOC100506 | 0.656386 | 0.370862 | 1.334675 | 0.395102 |
| LOC101930 | 0.656612 | 0.371039 | -1.30261 | 0.786985 |
| SDC1 | 0.656974 | 0.371294 | -0.86045 | 1.073924 |
| MAP3K11 | 0.657024 | 0.371372 | 0.347406 | 5.606323 |
| CDC14A | 0.657385 | 0.371626 | 0.695064 | 2.418038 |
| NELFCD | 0.657706 | 0.371858 | 0.360497 | 6.184503 |
| FAM27E3 | 0.658011 | 0.372081 | 0.694926 | 2.74612 |
| OTUD5 | 0.658214 | 0.372245 | -0.52867 | 2.854797 |
| FRAT1 | 0.658281 | 0.372336 | 0.700212 | 2.513478 |
| EXOSC3 | 0.658281 | 0.372384 | 0.982945 | 0.564902 |
| NAA15 | 0.658431 | 0.372519 | -0.37003 | 7.297746 |

| | | | | |
|---|---|---|---|---|
| ULBP3 | 0.659066 | 0.372932 | -0.6579 | 3.121849 |
| MACC1 | 0.659066 | 0.373033 | 0.378677 | 8.060652 |
| CC2D1A | 0.659066 | 0.373135 | -0.33415 | 6.20411 |
| PQLC1 | 0.659066 | 0.373206 | 1.272823 | 1.054347 |
| LINC01266 | 0.659066 | 0.373222 | 1.304768 | 1.568365 |
| TGFB3 | 0.659066 | 0.373254 | 0.439661 | 5.045231 |
| NGFR | 0.659066 | 0.373294 | -0.5671 | 3.187322 |
| KALRN | 0.659066 | 0.373357 | 0.702203 | 2.699653 |
| ASPM | 0.659066 | 0.373366 | 0.487808 | 5.487922 |
| MIR5092 | 0.659066 | 0.373411 | 1.255311 | 0.912712 |
| NSMCE1 | 0.659066 | 0.373431 | -0.38404 | 5.292844 |
| KTN1-AS1 | 0.659117 | 0.373557 | -1.12656 | 0.784512 |
| CCDC146 | 0.659117 | 0.373657 | -0.39396 | 5.388676 |
| ZNRF2 | 0.659117 | 0.373742 | -0.30836 | 6.11572 |
| MAT2A | 0.659117 | 0.37379 | -0.45047 | 8.024658 |
| NCBP2 | 0.659117 | 0.373794 | 0.701893 | 2.099455 |
| EXOC4 | 0.659117 | 0.373797 | -0.34513 | 5.839759 |
| LINC01105 | 0.659117 | 0.373811 | -1.78927 | 1.907537 |
| TACO1 | 0.659664 | 0.374172 | -0.43989 | 3.826626 |
| CMC1 | 0.659676 | 0.374231 | 0.74878 | 3.008906 |
| MIR6801 | 0.659676 | 0.374279 | 1.627421 | 1.083523 |
| SPATA33 | 0.659721 | 0.374495 | 0.880378 | 1.382262 |
| GDF7 | 0.659721 | 0.374516 | -1.33938 | 0.936543 |
| LINS1 | 0.659721 | 0.374525 | -0.45344 | 4.307948 |
| MBTPS2 | 0.659721 | 0.374618 | 0.373315 | 4.810484 |
| TDP2 | 0.659721 | 0.374674 | 0.354795 | 5.362121 |
| SUGP1 | 0.659721 | 0.374675 | -0.34454 | 5.413201 |
| CCDC88B | 0.659721 | 0.374762 | 0.408333 | 5.258546 |
| MIR4482 | 0.659721 | 0.374767 | 1.221755 | 0.739266 |
| PILRB | 0.659721 | 0.374855 | 0.961078 | 1.786566 |
| METTL27 | 0.659721 | 0.374909 | 0.974536 | 1.523835 |
| MZT2A | 0.659721 | 0.374912 | -0.56547 | 3.882849 |
| MSL2 | 0.659721 | 0.374937 | 0.445991 | 3.703497 |
| FUNDC1 | 0.659721 | 0.374958 | -0.52057 | 3.82454 |
| TMF1 | 0.659811 | 0.375059 | 0.33384 | 7.794677 |
| OCLN | 0.659824 | 0.375117 | -0.94473 | 1.54285 |
| GDPGP1 | 0.660033 | 0.375287 | 0.643571 | 1.823549 |
| POLD2 | 0.660065 | 0.375391 | 1.081025 | 1.087197 |
| TPRN | 0.660065 | 0.375472 | -0.45504 | 4.715258 |
| TRIP11 | 0.660065 | 0.375574 | -0.33394 | 7.935028 |
| SCARB2 | 0.660065 | 0.375584 | 0.439467 | 4.304148 |
| PDE7B | 0.660065 | 0.375598 | 0.679213 | 3.062689 |
| SMC5-AS1 | 0.660065 | 0.375606 | -0.79891 | 1.836065 |
| ITFG1 | 0.660195 | 0.375823 | -0.66277 | 1.872103 |
| WASHC2A | 0.660195 | 0.375854 | -1.2848 | 0.843009 |

| | | | | |
|---|---|---|---|---|
| SRSF12 | 0.660195 | 0.375879 | 0.640829 | 2.804999 |
| GTPBP4 | 0.660195 | 0.375966 | 0.372145 | 6.659828 |
| NUTM2A | 0.660195 | 0.375973 | -0.98819 | 1.760897 |
| PTCH2 | 0.660195 | 0.375982 | 0.522583 | 7.290315 |
| CELF1 | 0.660465 | 0.376223 | 0.309758 | 5.68833 |
| DLAT | 0.660465 | 0.376237 | -0.3372 | 6.062539 |
| C11orf1 | 0.660572 | 0.376348 | 0.729354 | 2.83395 |
| BRD1 | 0.660579 | 0.376402 | -0.54158 | 3.164599 |
| LINC01585 | 0.660931 | 0.376681 | -1.30076 | 0.46992 |
| FRG2C | 0.660931 | 0.376704 | -1.04805 | 0.776653 |
| CCDC144N | 0.661134 | 0.37687 | 1.173877 | 0.630522 |
| FXYD3 | 0.661369 | 0.377087 | 0.816238 | 2.146835 |
| COPS6 | 0.661369 | 0.377104 | -0.36631 | 6.447975 |
| MSRB1 | 0.661504 | 0.377238 | 0.610823 | 4.167506 |
| NDUFAF6 | 0.661504 | 0.377327 | 0.462239 | 3.701442 |
| RTF1 | 0.661504 | 0.377334 | -0.31749 | 7.383209 |
| TTF2 | 0.661504 | 0.377383 | -0.33497 | 5.631995 |
| LOC10050€ | 0.661614 | 0.377496 | 0.641087 | 3.122426 |
| HIST1H2BE | 0.661736 | 0.377711 | -0.4914 | 3.811837 |
| POM121L1 | 0.661736 | 0.377745 | 1.300204 | 0.547903 |
| NUP160 | 0.661736 | 0.377762 | 0.332227 | 6.730493 |
| FAM20B | 0.661736 | 0.377767 | 0.325903 | 6.669314 |
| KIAA0355 | 0.661839 | 0.377876 | -0.47305 | 5.32454 |
| RAB17 | 0.661848 | 0.377989 | 1.228713 | 0.429487 |
| SYT7 | 0.661848 | 0.378006 | 1.685843 | 0.439636 |
| AACSP1 | 0.661848 | 0.378033 | -1.58896 | 0.642629 |
| GPR108 | 0.661927 | 0.378223 | -0.3242 | 5.866649 |
| LOC40332: | 0.661927 | 0.378225 | 0.996514 | 0.550644 |
| LOC10012! | 0.661927 | 0.378255 | 0.840216 | 3.842844 |
| ZNF574 | 0.661927 | 0.37828 | 0.453239 | 4.289139 |
| RIF1 | 0.662188 | 0.378525 | 0.87656 | 1.229314 |
| SLC12A9 | 0.662188 | 0.37853 | 0.548082 | 3.192556 |
| TAGLN2 | 0.662283 | 0.378635 | 0.921293 | 1.742854 |
| TAMM41 | 0.662397 | 0.378751 | -0.72481 | 2.706874 |
| CCDC93 | 0.662482 | 0.378849 | 0.329957 | 7.010728 |
| SQSTM1 | 0.662688 | 0.379018 | 0.555309 | 2.995272 |
| SLC9A2 | 0.662689 | 0.379069 | 0.887054 | 4.361189 |
| LINC00174 | 0.662733 | 0.379145 | -0.40849 | 4.916095 |
| AKIRIN1 | 0.662898 | 0.37933 | 0.760471 | 1.883292 |
| LOC10192: | 0.662898 | 0.37934 | -0.97254 | 1.668058 |
| COL18A1-A | 0.663708 | 0.379897 | 0.704178 | 2.476717 |
| ERICH1 | 0.663708 | 0.379905 | 0.555222 | 3.275553 |
| FARP1 | 0.664272 | 0.380278 | 0.909897 | 1.886694 |
| CDKL2 | 0.664482 | 0.380449 | -0.68263 | 2.356411 |
| TMPRSS2 | 0.664556 | 0.380542 | -0.91531 | 0.741548 |

| | | | | |
|---|---|---|---|---|
| C2orf42 | 0.664822 | 0.380745 | 0.466135 | 3.652958 |
| HIST1H2BL | 0.66493 | 0.38088 | 0.551313 | 3.982192 |
| C8orf44 | 0.66493 | 0.380908 | 0.78025 | 2.852742 |
| RIMBP2 | 0.665063 | 0.381035 | 0.734331 | 3.335647 |
| LOC10272‹ | 0.665253 | 0.381195 | -0.62865 | 3.291591 |
| HCAR1 | 0.665269 | 0.381255 | 1.135211 | 1.508179 |
| FAM120AC | 0.665384 | 0.381417 | 0.30818 | 5.609833 |
| ADAMTSL1 | 0.665384 | 0.381446 | 0.9392 | 3.316618 |
| RERE-AS1 | 0.665384 | 0.381472 | 0.759015 | 2.165255 |
| SLAMF8 | 0.66588 | 0.381808 | 0.746087 | 2.785477 |
| PAX8-AS1 | 0.66618 | 0.382031 | -0.3669 | 7.261885 |
| TNFAIP1 | 0.666477 | 0.382274 | -0.33839 | 6.13852 |
| UQCC3 | 0.666477 | 0.382352 | -0.46364 | 3.816893 |
| BNIP2 | 0.666477 | 0.382353 | 0.326419 | 6.494123 |
| CARF | 0.666615 | 0.382522 | 1.160225 | 0.445675 |
| MLEC | 0.666615 | 0.382582 | -1.10553 | 0.528573 |
| LINC02099 | 0.666615 | 0.382585 | -1.44245 | 1.080799 |
| SMG7-AS1 | 0.666638 | 0.382649 | 1.055232 | 1.158706 |
| MIR1205 | 0.666729 | 0.382752 | -0.96231 | 1.29331 |
| ATP10B | 0.666773 | 0.382828 | -0.70791 | 6.575575 |
| E2F3 | 0.667167 | 0.383105 | 1.160055 | 0.888639 |
| LOC10192¢ | 0.667193 | 0.383237 | -1.37563 | 0.58725 |
| BTBD6 | 0.667193 | 0.383288 | -0.35536 | 5.196601 |
| POMZP3 | 0.667193 | 0.383358 | 1.578429 | 1.171881 |
| CTBP2 | 0.667193 | 0.383372 | 0.398376 | 4.733595 |
| APLF | 0.667193 | 0.383374 | -0.39976 | 5.010149 |
| PLA2G2F | 0.667291 | 0.383481 | 1.763531 | 1.650364 |
| RAB41 | 0.667399 | 0.383594 | 1.226936 | 0.542464 |
| SNORD47 | 0.667493 | 0.383699 | 0.585348 | 5.924437 |
| VENTX | 0.667501 | 0.383755 | 1.114179 | 0.724553 |
| CLK4 | 0.667532 | 0.383824 | -0.36036 | 5.672119 |
| PFN1 | 0.667561 | 0.383891 | -0.33566 | 7.973686 |
| EFNB2 | 0.667959 | 0.38417 | 0.392826 | 7.25434 |
| PIK3IP1-AS | 0.668372 | 0.384481 | 1.070214 | 0.663429 |
| LOC10027( | 0.668372 | 0.384518 | 0.965267 | 1.199688 |
| EPB41L1 | 0.668372 | 0.384624 | -0.52791 | 3.370862 |
| ORMDL1 | 0.668372 | 0.38465 | -0.82312 | 1.371686 |
| LOC33905¢ | 0.668372 | 0.384663 | -0.60088 | 2.396951 |
| TAF9B | 0.668383 | 0.38472 | -0.41522 | 5.359322 |
| RAB11FIP2 | 0.668584 | 0.384887 | 0.424771 | 6.247957 |
| SMC2 | 0.669032 | 0.3852 | 1.002941 | 1.495416 |
| WDR35 | 0.669032 | 0.385363 | 1.190242 | 0.442406 |
| LOC91450 | 0.669032 | 0.385382 | 0.942459 | 1.218243 |
| HIST4H4 | 0.669032 | 0.385397 | -0.53987 | 3.262778 |
| CLCF1 | 0.669032 | 0.385432 | 0.751817 | 1.686324 |

| | | | | |
|---|---|---|---|---|
| POLR2G | 0.669032 | 0.385504 | -0.46796 | 4.244283 |
| TCTA | 0.669032 | 0.385547 | 0.399104 | 4.283559 |
| RSBN1 | 0.669032 | 0.385553 | -1.01582 | 0.574434 |
| LPAR2 | 0.669151 | 0.38572 | 0.43964 | 3.398637 |
| EIF4G1 | 0.669151 | 0.385759 | -0.85138 | 0.870304 |
| PTPRD | 0.669151 | 0.385774 | 0.919197 | 1.323407 |
| STK35 | 0.66923 | 0.385871 | -0.32512 | 6.961657 |
| MEIOC | 0.669484 | 0.386114 | 1.039348 | 1.690359 |
| STAR | 0.669484 | 0.386119 | -0.98721 | 1.397896 |
| ASNA1 | 0.669622 | 0.386249 | -0.35331 | 4.545392 |
| SIPA1L1 | 0.669995 | 0.386516 | -0.87643 | 0.857378 |
| ZNF354B | 0.67014 | 0.386677 | 0.596361 | 4.185314 |
| OR52I2 | 0.67014 | 0.386702 | -1.80625 | 0.54621 |
| SCRG1 | 0.670165 | 0.386767 | 1.024092 | 1.522361 |
| PSMC5 | 0.670185 | 0.386836 | -0.84734 | 1.315996 |
| SNORD76 | 0.670185 | 0.386881 | 0.507799 | 8.171409 |
| DDIAS | 0.670326 | 0.387029 | 0.690765 | 3.153095 |
| CABLES1 | 0.670326 | 0.387065 | 0.861846 | 2.173476 |
| MIR4714 | 0.670454 | 0.387205 | 1.007232 | 1.099739 |
| S1PR4 | 0.670454 | 0.387283 | 1.067154 | 1.141789 |
| ERI1 | 0.670454 | 0.387292 | 0.357636 | 5.037988 |
| LOC101928 | 0.670526 | 0.387385 | -0.95636 | 1.318323 |
| CDKN2C | 0.670549 | 0.387449 | 0.7122 | 1.951154 |
| FTH1 | 0.670838 | 0.387703 | -0.40153 | 10.38308 |
| THOC2 | 0.670838 | 0.387732 | 0.331339 | 8.150397 |
| MRTFA-AS1 | 0.670838 | 0.387769 | 0.43315 | 4.459898 |
| SERTAD1 | 0.670866 | 0.387836 | -0.55985 | 3.233831 |
| SYN2 | 0.671054 | 0.387996 | 1.325491 | 1.691615 |
| SNORD74 | 0.671518 | 0.388316 | 1.017257 | 0.834497 |
| ROM1 | 0.671534 | 0.388376 | -0.76765 | 1.920015 |
| SUOX | 0.671701 | 0.388524 | 1.618253 | 0.326363 |
| POLR1A | 0.671852 | 0.388705 | 0.312789 | 6.894205 |
| CRACR2A | 0.671852 | 0.388714 | 0.684634 | 2.780937 |
| RAB11B-AS | 0.671951 | 0.388838 | -0.72394 | 2.38651 |
| ATMIN | 0.671951 | 0.388918 | -0.72854 | 1.802645 |
| FAM166A | 0.671951 | 0.388924 | -1.27845 | 0.794416 |
| FDX1 | 0.672004 | 0.389006 | 0.389035 | 5.149384 |
| SLC35B4 | 0.672128 | 0.389169 | 0.416034 | 5.004039 |
| SH3GL1P2 | 0.672128 | 0.38918 | 0.721119 | 2.875941 |
| TYSND1 | 0.67214 | 0.389239 | 0.747243 | 2.23411 |
| TMEM91 | 0.672296 | 0.389472 | -1.17224 | 1.202767 |
| SHQ1 | 0.672296 | 0.389492 | 0.470094 | 4.315269 |
| TSHR | 0.672296 | 0.389517 | 1.052675 | 1.844023 |
| TFRC | 0.672296 | 0.389534 | 1.292416 | 0.398261 |
| PRKCI | 0.672377 | 0.389632 | 0.364006 | 7.533963 |

| | | | | |
|---|---|---|---|---|
| PGA4 | 0.672501 | 0.389755 | -2.36473 | 7.166152 |
| ZNF687 | 0.67261 | 0.38995 | -0.91175 | 1.595136 |
| PTTG3P | 0.67261 | 0.38998 | 1.258554 | 0.761152 |
| LOC10537ε | 0.67261 | 0.389998 | 0.661399 | 3.140158 |
| DNAJC3-DT | 0.67261 | 0.390067 | 0.620998 | 2.707938 |
| GZMB | 0.67261 | 0.390075 | 1.07916 | 2.57593 |
| LINC01291 | 0.672622 | 0.390175 | 1.347802 | 0.639266 |
| PAPPA | 0.672622 | 0.39023 | -0.47338 | 6.82082 |
| MAN2A1 | 0.672622 | 0.39025 | -0.32128 | 6.914771 |
| PROSER1 | 0.672622 | 0.390287 | 0.343789 | 6.383364 |
| MAMDC2-/ | 0.67266 | 0.390405 | 1.070684 | 2.118064 |
| LINC00314 | 0.67266 | 0.390449 | 1.670451 | 0.847874 |
| IL21R-AS1 | 0.67266 | 0.390519 | -1.67122 | 0.769569 |
| RPEL1 | 0.67266 | 0.390552 | 0.817414 | 0.88361 |
| CNTD1 | 0.67266 | 0.390576 | 0.732851 | 2.621849 |
| GNG5 | 0.67266 | 0.390616 | -0.50166 | 5.634288 |
| XRN2 | 0.672686 | 0.390683 | 0.677078 | 2.756645 |
| CMTM6 | 0.67275 | 0.390771 | -0.32054 | 7.098191 |
| HAAO | 0.672853 | 0.390883 | 0.724567 | 2.648011 |
| LOC102724 | 0.67318 | 0.391124 | 1.433765 | 0.71449 |
| ZNF516 | 0.673315 | 0.39127 | -0.36309 | 6.363019 |
| PDCD6IP | 0.673315 | 0.391351 | 0.441832 | 3.617655 |
| EVC | 0.673315 | 0.391393 | -1.13577 | 0.774404 |
| SYP | 0.673315 | 0.391407 | 0.876703 | 2.031122 |
| TRIM56 | 0.673448 | 0.391569 | 0.316671 | 6.782625 |
| IPO8 | 0.673448 | 0.391587 | 0.431136 | 3.99188 |
| CD160 | 0.673466 | 0.391649 | 1.011428 | 2.18065 |
| C2orf16 | 0.673703 | 0.391867 | 0.744988 | 2.473139 |
| PIKFYVE | 0.673703 | 0.391906 | 0.300071 | 6.869737 |
| RAB44 | 0.673703 | 0.392082 | -0.86539 | 1.106187 |
| ARMH3 | 0.673703 | 0.392103 | -0.33512 | 6.098508 |
| LOC101928 | 0.673703 | 0.392108 | 1.437233 | 1.385447 |
| UBXN6 | 0.673703 | 0.392135 | -1.05247 | 0.922165 |
| IMPG2 | 0.673703 | 0.392157 | 0.712434 | 1.398009 |
| CERCAM | 0.673703 | 0.392243 | 1.219315 | 0.448501 |
| SCML2 | 0.673703 | 0.392263 | 1.240814 | 0.446075 |
| CNOT3 | 0.673703 | 0.392301 | 0.53187 | 3.717137 |
| INPP5B | 0.673806 | 0.392412 | 0.809505 | 2.234942 |
| PACS1 | 0.673845 | 0.392485 | -0.30191 | 6.764766 |
| PPA2 | 0.67392 | 0.392581 | 1.236866 | 0.579024 |
| UBE2E4P | 0.673959 | 0.392655 | 0.734357 | 2.252711 |
| ANAPC16 | 0.674181 | 0.392873 | -0.6124 | 2.644246 |
| PPID | 0.674181 | 0.392887 | 0.332418 | 5.709675 |
| ZNF8 | 0.674366 | 0.393046 | 0.399771 | 4.8505 |
| RNF26 | 0.674662 | 0.39327 | 0.345369 | 5.035931 |

| | | | | |
|---|---|---|---|---|
| BARHL1 | 0.674854 | 0.393433 | 1.08873 | 0.304745 |
| GCFC2 | 0.674876 | 0.393512 | 0.914773 | 0.819659 |
| SHKBP1 | 0.674876 | 0.393588 | 0.319846 | 5.567014 |
| SLC38A3 | 0.674876 | 0.393601 | -1.1378 | 1.004737 |
| MLLT10P1 | 0.675028 | 0.393741 | 0.985833 | 1.305855 |
| SEMA6A-A: | 0.675114 | 0.393847 | -1.03066 | 0.978511 |
| CASC21 | 0.675114 | 0.393894 | 1.565936 | 0.391323 |
| IGBP1P1 | 0.67531 | 0.394074 | 1.271229 | 0.505171 |
| SNORA6 | 0.67531 | 0.394115 | -0.40222 | 8.113869 |
| GAS5 | 0.67531 | 0.394162 | -0.35255 | 5.641432 |
| TSC22D4 | 0.675372 | 0.39425 | -1.07179 | 0.540937 |
| GPALPP1 | 0.675518 | 0.394387 | 0.478045 | 3.534975 |
| SMTNL1 | 0.675539 | 0.394451 | 1.030951 | 0.594913 |
| PTRHD1 | 0.675743 | 0.394621 | -0.69165 | 2.324798 |
| IMPAD1 | 0.675991 | 0.394817 | -0.36312 | 7.433323 |
| RTF2 | 0.676235 | 0.395011 | 1.22048 | 0.407914 |
| NCF1 | 0.676338 | 0.395123 | 0.882022 | 1.782813 |
| OTULIN | 0.676371 | 0.395273 | -0.30114 | 6.557443 |
| CDC23 | 0.676371 | 0.395317 | -0.39504 | 4.992878 |
| PARP6 | 0.676371 | 0.395358 | -0.34473 | 6.045481 |
| YKT6 | 0.676371 | 0.395432 | -0.33564 | 6.733821 |
| PSPH | 0.676371 | 0.395451 | 0.44985 | 4.332548 |
| PRICKLE2-A | 0.676371 | 0.395452 | 0.981382 | 1.379263 |
| ZNF337 | 0.676699 | 0.395695 | 0.65315 | 2.164599 |
| FBXL3 | 0.676728 | 0.395763 | -0.30804 | 6.546044 |
| ANKIB1 | 0.676826 | 0.395878 | 0.332962 | 6.966044 |
| SCARNA10 | 0.676826 | 0.395924 | 0.306917 | 7.561754 |
| RIDA | 0.67702 | 0.396157 | -0.49352 | 3.486847 |
| GKN1 | 0.67702 | 0.396186 | 2.088807 | 6.903795 |
| LINC00515 | 0.67702 | 0.396193 | 1.021347 | 1.199765 |
| LSAMP-AS1 | 0.677234 | 0.396369 | 1.095682 | 0.860031 |
| DGLUCY | 0.677337 | 0.396543 | 0.817654 | 2.213671 |
| GPR151 | 0.677337 | 0.396577 | -2.0756 | 0.578462 |
| CRISPLD1 | 0.677337 | 0.396604 | 1.015576 | 2.343152 |
| KANSL3 | 0.677337 | 0.396641 | -1.09717 | 0.95245 |
| TMEM186 | 0.677337 | 0.396688 | 0.605691 | 3.63862 |
| CHMP1B | 0.677489 | 0.396828 | -0.40835 | 6.916635 |
| MTMR7 | 0.677972 | 0.397207 | 0.587338 | 3.860796 |
| WNK2 | 0.677972 | 0.397334 | 1.049209 | 1.567764 |
| MRPS31 | 0.677972 | 0.397357 | -0.354 | 4.72028 |
| NEURL1 | 0.677972 | 0.397359 | -0.7066 | 2.402995 |
| HMGN3 | 0.677972 | 0.397403 | -1.09105 | 1.239458 |
| LINC00898 | 0.677972 | 0.397421 | -1.55564 | 1.391568 |
| SMIM6 | 0.677995 | 0.397517 | 0.979956 | 1.866192 |
| ZSCAN9 | 0.677995 | 0.397551 | 0.816043 | 1.05193 |

| | | | | |
|---|---|---|---|---|
| SSC4D | 0.677995 | 0.39761 | 0.877602 | 1.94416 |
| NKX6-2 | 0.677995 | 0.397644 | -1.30672 | 0.529077 |
| LINC01959 | 0.677995 | 0.397693 | -1.10568 | 0.917667 |
| MALT1 | 0.678056 | 0.397814 | -1.08578 | 1.032108 |
| ABHD18 | 0.678056 | 0.397832 | 0.356092 | 4.819476 |
| HIST1H2BD | 0.678216 | 0.398018 | 0.700454 | 1.86475 |
| SORCS2 | 0.678216 | 0.39803 | -0.39066 | 4.091428 |
| DUSP14 | 0.678421 | 0.398202 | -1.02048 | 2.29606 |
| ZNF416 | 0.678566 | 0.398338 | -0.49793 | 3.345902 |
| RECQL4 | 0.678884 | 0.398613 | 0.426211 | 4.337984 |
| LDLRAD2 | 0.678884 | 0.398628 | 0.811469 | 1.840374 |
| RDH11 | 0.679085 | 0.398833 | -0.91958 | 1.531496 |
| SPDYE5 | 0.679085 | 0.398958 | 0.37448 | 4.84384 |
| OCIAD1 | 0.679085 | 0.39897 | -1.14284 | 1.127444 |
| MRPL53 | 0.679085 | 0.398972 | -0.45944 | 4.014999 |
| MTRNR2L4 | 0.679085 | 0.399005 | 1.202424 | 0.976442 |
| NCK1 | 0.679567 | 0.39934 | -1.2407 | 1.011087 |
| DALRD3 | 0.679713 | 0.399478 | 1.362923 | 0.286708 |
| FEM1C | 0.679888 | 0.399726 | -0.3378 | 6.705124 |
| SYS1 | 0.679888 | 0.39977 | 0.621172 | 2.683057 |
| CHSY1 | 0.679888 | 0.399813 | 0.319289 | 6.056595 |
| RAI1 | 0.679888 | 0.399825 | -0.33135 | 6.876601 |
| LOC100996 | 0.679888 | 0.39984 | 0.621908 | 2.440677 |
| ACO2 | 0.680105 | 0.400019 | -0.3424 | 6.296348 |
| PPP3CB-AS | 0.680167 | 0.400107 | -0.69999 | 2.082974 |
| LOC100507 | 0.680167 | 0.400159 | -1.56151 | 0.55664 |
| CLPTM1L | 0.680169 | 0.400258 | 0.37776 | 4.744171 |
| OVOL2 | 0.680169 | 0.400265 | 1.02824 | 1.23229 |
| MCRIP1 | 0.680169 | 0.400316 | -1.28983 | 0.741101 |
| FLJ20021 | 0.680199 | 0.400428 | 1.080612 | 0.907668 |
| LOC340512 | 0.680199 | 0.400437 | -0.98239 | 1.67973 |
| C8orf37 | 0.680345 | 0.400608 | 0.782925 | 2.155962 |
| CLDN10 | 0.680345 | 0.400653 | -1.52101 | 0.408426 |
| SNORA92 | 0.680345 | 0.400679 | 0.717917 | 1.296995 |
| PACRG-AS3 | 0.680366 | 0.400743 | -1.05702 | 1.228845 |
| DDX20 | 0.680442 | 0.40084 | 0.378511 | 4.585596 |
| RFX3-AS1 | 0.680581 | 0.400973 | -1.38762 | 1.057337 |
| PAXBP1 | 0.680893 | 0.401218 | 1.40143 | 0.403692 |
| ARMT1 | 0.680893 | 0.401261 | 1.119719 | 0.969359 |
| ADIRF | 0.681026 | 0.401407 | -0.50242 | 5.132037 |
| PCBP2-OT1 | 0.681026 | 0.401494 | 0.638461 | 2.12705 |
| LRRC37A8P | 0.681026 | 0.401625 | 1.117554 | 1.619563 |
| MSN | 0.681026 | 0.401692 | 0.374953 | 8.794585 |
| SNORD5 | 0.681026 | 0.401697 | 0.606549 | 2.995373 |
| SPG21 | 0.681026 | 0.401723 | -1.37494 | 0.511039 |

| | | | | |
|---|---|---|---|---|
| SOCS2-AS1 | 0.681026 | 0.401755 | 1.015474 | 1.318253 |
| IGSF21 | 0.681026 | 0.401778 | 1.000781 | 0.963389 |
| YLPM1 | 0.681026 | 0.401807 | 0.360589 | 8.572503 |
| LOC101927 | 0.681249 | 0.40199 | -1.07289 | 0.7744 |
| LOC644936 | 0.681259 | 0.40208 | 1.045224 | 0.75681 |
| SP5 | 0.681259 | 0.4021 | 1.165671 | 1.904869 |
| STX7 | 0.681294 | 0.402316 | 0.303888 | 6.827124 |
| LOC101927 | 0.681294 | 0.402381 | 0.908424 | 1.148587 |
| PTPRE | 0.681294 | 0.402382 | 0.769088 | 2.290475 |
| ZNF470 | 0.681294 | 0.40241 | -0.45211 | 4.23617 |
| LINC00310 | 0.681294 | 0.402422 | -0.94198 | 1.648753 |
| CLASP2 | 0.681294 | 0.402432 | -0.38717 | 4.71331 |
| CARNS1 | 0.681309 | 0.402492 | -0.94754 | 0.380082 |
| SEM1 | 0.681516 | 0.402718 | 0.423576 | 6.904902 |
| MALAT1 | 0.681516 | 0.402719 | -0.31817 | 16.78831 |
| MECP2 | 0.681542 | 0.402822 | 0.360051 | 6.279021 |
| LOC100303 | 0.681542 | 0.402838 | -0.55254 | 2.744992 |
| TMEM97 | 0.681583 | 0.402914 | 0.531692 | 4.298784 |
| SNORD46 | 0.681949 | 0.403222 | 0.431923 | 6.894447 |
| CST3 | 0.681949 | 0.403292 | 0.319132 | 6.057683 |
| TSPAN32 | 0.681949 | 0.403397 | 1.003848 | 1.443574 |
| SNORD112 | 0.681949 | 0.403417 | 1.096573 | 0.836359 |
| PLXDC2 | 0.681949 | 0.403427 | 1.047187 | 1.398818 |
| C16orf91 | 0.681949 | 0.403542 | 0.692788 | 2.546742 |
| C20orf194 | 0.681949 | 0.403544 | 0.340045 | 6.329245 |
| COPG2 | 0.681949 | 0.403546 | -0.42049 | 3.936022 |
| PIK3R4 | 0.682562 | 0.403961 | 0.337641 | 5.505468 |
| FBXL4 | 0.682996 | 0.40427 | 0.594063 | 2.915365 |
| ZNF341-AS | 0.683001 | 0.40441 | -1.07626 | 1.165088 |
| METTL3 | 0.683001 | 0.404522 | 0.337865 | 5.898291 |
| LOC101926 | 0.683001 | 0.404626 | 0.959679 | 0.795368 |
| CCDC84 | 0.683001 | 0.404637 | 0.947183 | 1.621332 |
| NME8 | 0.683001 | 0.404658 | 1.064571 | 0.954561 |
| TEX261 | 0.683001 | 0.404682 | 0.285953 | 6.036411 |
| NKPD1 | 0.683001 | 0.404683 | 0.768224 | 2.506093 |
| LOC100289 | 0.683001 | 0.404689 | -1.01989 | 1.455272 |
| PTGES2-AS | 0.68312 | 0.404935 | 1.038343 | 1.726651 |
| SNORD98 | 0.68312 | 0.404964 | 1.006623 | 0.706947 |
| RPS6KB1 | 0.68312 | 0.404973 | -1.0699 | 1.118694 |
| LINC01801 | 0.68312 | 0.405027 | 1.379402 | 1.07513 |
| SPNS3 | 0.68312 | 0.405043 | 0.972328 | 0.783617 |
| SH2D3C | 0.68312 | 0.405129 | -0.96703 | 1.834562 |
| GCHFR | 0.68312 | 0.405205 | -0.76886 | 3.040792 |
| GPAT4 | 0.68312 | 0.405234 | 0.306212 | 6.616871 |
| KIF20A | 0.68312 | 0.405235 | 0.443894 | 3.992675 |

| | | | | |
|---|---|---|---|---|
| CTD-2201I: | 0.68312 | 0.405307 | 0.903673 | 2.667304 |
| SUPT6H | 0.68312 | 0.405332 | 0.305833 | 8.593633 |
| TUSC8 | 0.683151 | 0.405417 | -1.44734 | 0.625525 |
| NFIA-AS1 | 0.683151 | 0.405501 | 0.583753 | 3.119883 |
| SNORD11 | 0.683151 | 0.405507 | 0.613545 | 4.122978 |
| CDCA4 | 0.683331 | 0.405666 | -0.89697 | 0.816136 |
| UNC13D | 0.683586 | 0.405888 | -0.3561 | 6.088302 |
| SLC35C2 | 0.683586 | 0.405921 | 0.517983 | 3.170762 |
| PPP2R5B | 0.683646 | 0.40601 | 0.41406 | 4.423371 |
| C8orf74 | 0.683775 | 0.406138 | -1.54019 | 0.508022 |
| CAPN7 | 0.683878 | 0.406328 | 0.311106 | 6.44268 |
| ZADH2 | 0.683878 | 0.406335 | -0.54822 | 2.076331 |
| SCARNA14 | 0.683878 | 0.406356 | -0.94644 | 0.991989 |
| SMIM25 | 0.684395 | 0.406735 | 0.975042 | 1.431391 |
| SPCS2 | 0.684395 | 0.406767 | 0.349561 | 5.959169 |
| POLR3A | 0.684442 | 0.406847 | 0.293375 | 5.955918 |
| NUDC | 0.68455 | 0.407022 | 0.309512 | 7.005536 |
| PET117 | 0.68455 | 0.407045 | -0.9405 | 0.943877 |
| DEXI | 0.68455 | 0.407068 | 0.421614 | 4.119559 |
| FEN1 | 0.684716 | 0.407287 | 0.495293 | 4.447427 |
| NUMBL | 0.684716 | 0.407306 | -1.06988 | 0.622222 |
| LINC01679 | 0.684716 | 0.407323 | -0.85489 | 1.497487 |
| PLEKHM3 | 0.684928 | 0.407537 | 0.534602 | 5.317407 |
| PKN2-AS1 | 0.684928 | 0.407553 | -1.04687 | 1.26468 |
| RASIP1 | 0.685038 | 0.407671 | -0.54197 | 3.568705 |
| UBE2F | 0.685192 | 0.407815 | -1.02851 | 1.016604 |
| AEBP2 | 0.685241 | 0.40793 | -0.55309 | 2.327095 |
| FRY-AS1 | 0.685241 | 0.407949 | 0.87413 | 1.228189 |
| RBM41 | 0.685387 | 0.408088 | 0.309989 | 5.907603 |
| DNAJC10 | 0.68556 | 0.408291 | -1.11344 | 0.387237 |
| RNASEH2A | 0.68556 | 0.408419 | 0.478194 | 3.539593 |
| CCNF | 0.68556 | 0.408426 | 0.397562 | 4.300918 |
| PRRT3 | 0.68556 | 0.408467 | 0.623692 | 2.955983 |
| TERC | 0.68556 | 0.408485 | -0.33376 | 5.546528 |
| SMG1 | 0.68556 | 0.408504 | 0.295737 | 9.176558 |
| CREG2 | 0.685662 | 0.408617 | 1.220454 | 1.229733 |
| NUP133 | 0.685728 | 0.40875 | 0.278793 | 5.891778 |
| PCF11-AS1 | 0.685728 | 0.408761 | 0.752036 | 2.193023 |
| GABPB1-AS | 0.685759 | 0.408883 | -0.33081 | 6.068998 |
| URAHP | 0.685759 | 0.408936 | -0.70789 | 2.204374 |
| UBB | 0.685759 | 0.408951 | 1.48768 | 0.617981 |
| VWA2 | 0.685759 | 0.408989 | 0.558663 | 5.405157 |
| ABCB6 | 0.686188 | 0.409373 | -0.33729 | 4.97773 |
| LMTK3 | 0.686188 | 0.409414 | -0.42265 | 4.347591 |
| PP7080 | 0.686188 | 0.40942 | 0.576902 | 5.879997 |

| | | | | |
|---|---|---|---|---|
| BBS2 | 0.686188 | 0.409454 | -0.34082 | 5.526186 |
| DHX8 | 0.686212 | 0.409551 | -0.56836 | 3.49368 |
| BDH2 | 0.686212 | 0.409573 | 0.424951 | 4.697057 |
| CCDC142 | 0.686352 | 0.409758 | 0.415065 | 4.321596 |
| TMED7 | 0.686352 | 0.409876 | -0.43957 | 6.663216 |
| FANCF | 0.686352 | 0.409896 | 0.468912 | 3.64673 |
| MUM1 | 0.686352 | 0.409927 | 0.938577 | 0.617165 |
| CLCA3P | 0.686352 | 0.409948 | -0.83821 | 2.042536 |
| OCLNP1 | 0.686352 | 0.40997 | 0.383459 | 5.470218 |
| L3MBTL4-A | 0.686638 | 0.410221 | -0.67155 | 1.316277 |
| RPL3 | 0.686638 | 0.410246 | -0.40749 | 5.023127 |
| SIGIRR | 0.686783 | 0.410384 | -1.12775 | 0.679643 |
| ZNF532 | 0.686882 | 0.410496 | 0.338218 | 6.69044 |
| IRGQ | 0.686891 | 0.410553 | 0.301664 | 6.598317 |
| NKG7 | 0.68719 | 0.410831 | 0.801675 | 2.191771 |
| GTF3C6 | 0.68719 | 0.410837 | -0.34714 | 5.702065 |
| ZNF846 | 0.687266 | 0.410935 | -0.47021 | 4.780848 |
| TTLL5 | 0.687434 | 0.411126 | -0.31408 | 6.329202 |
| CAPN2 | 0.687434 | 0.41114 | -0.37672 | 4.121798 |
| GBP3 | 0.687514 | 0.41124 | 0.531634 | 5.920797 |
| EXTL3-AS1 | 0.687776 | 0.411449 | -1.26016 | 0.593979 |
| LOC645752 | 0.688021 | 0.411648 | 1.226874 | 1.027362 |
| GTSE1 | 0.688023 | 0.411702 | 0.545421 | 3.306083 |
| MLH1 | 0.688256 | 0.411938 | -1.1865 | 1.468914 |
| MOB3A | 0.688256 | 0.411947 | -0.28762 | 5.853357 |
| LINC01195 | 0.688888 | 0.412377 | 1.815974 | 0.536851 |
| RAD9A | 0.689079 | 0.412544 | 0.705736 | 1.991404 |
| VAC14-AS1 | 0.689152 | 0.412641 | 0.935049 | 1.355803 |
| FBLIM1 | 0.689403 | 0.412849 | 0.807366 | 1.3227 |
| POLA1 | 0.689403 | 0.412903 | 0.330287 | 5.70837 |
| THUMPD1 | 0.689403 | 0.412948 | 0.779226 | 2.171562 |
| GALNTL6-A | 0.689598 | 0.41313 | -1.9238 | 1.082901 |
| ZNF215 | 0.689598 | 0.41323 | 0.503928 | 3.249152 |
| LOC93622 | 0.689598 | 0.413237 | -0.40632 | 3.996667 |
| PTBP2 | 0.689598 | 0.413325 | -0.6533 | 2.809854 |
| TMEM150C | 0.689598 | 0.413328 | -0.70576 | 2.640814 |
| PDE4D | 0.689645 | 0.413414 | 0.480968 | 4.414586 |
| CORIN | 0.689645 | 0.413491 | 1.414296 | 0.479291 |
| XXYLT1 | 0.689645 | 0.413514 | -0.8665 | 1.15568 |
| ZNF300P1 | 0.689877 | 0.413705 | 0.941927 | 2.093797 |
| DDX12P | 0.689905 | 0.413831 | 0.437666 | 4.452332 |
| ZCWPW2 | 0.689905 | 0.413878 | -0.82247 | 2.715972 |
| FMO6P | 0.689905 | 0.413949 | -0.95343 | 1.432778 |
| USO1 | 0.689905 | 0.413984 | 0.658573 | 2.615239 |
| PIGF | 0.689905 | 0.414021 | 0.902186 | 1.341403 |

| | | | | |
|---|---|---|---|---|
| PIGV | 0.689905 | 0.414067 | 0.792471 | 0.731356 |
| ZBTB43 | 0.689905 | 0.414094 | -1.327 | 0.728863 |
| LOC101928 | 0.689905 | 0.414143 | -1.34686 | 0.851172 |
| NOTCH4 | 0.689983 | 0.414242 | 0.945506 | 1.188357 |
| MFSD1 | 0.689995 | 0.41435 | 1.101338 | 0.76196 |
| REC114 | 0.689995 | 0.414355 | -1.12975 | 1.162684 |
| LOC101929 | 0.690194 | 0.414526 | 1.305273 | 0.831175 |
| MKNK1-AS | 0.690509 | 0.414871 | 0.521129 | 2.914062 |
| ZNF592 | 0.690509 | 0.414897 | 0.293966 | 6.436121 |
| NAA35 | 0.690509 | 0.414914 | 0.285889 | 6.049604 |
| AFG1L | 0.690509 | 0.414942 | 0.585789 | 3.32904 |
| PLEKHG1 | 0.690509 | 0.415033 | -0.30281 | 6.984994 |
| FOXN2 | 0.690509 | 0.41511 | 0.414442 | 5.457947 |
| LOC105378 | 0.690509 | 0.415115 | 0.995306 | 1.090399 |
| HMGCS1 | 0.690509 | 0.415137 | -1.17445 | 0.991629 |
| SAR1A | 0.690582 | 0.415233 | 1.146605 | 1.107357 |
| PHKA2 | 0.690592 | 0.415294 | 0.348165 | 5.493565 |
| CXorf21 | 0.690592 | 0.415345 | 0.841934 | 1.42865 |
| LOC100996 | 0.690821 | 0.415535 | 1.645268 | 1.360423 |
| GPR153 | 0.690835 | 0.415596 | -0.50499 | 4.921278 |
| SUPT20H | 0.691058 | 0.415783 | 0.544571 | 2.299458 |
| MIRLET7BH | 0.691077 | 0.415847 | 0.782935 | 1.682865 |
| HDHD3 | 0.691228 | 0.416014 | -0.65977 | 1.8306 |
| LAMP3 | 0.691228 | 0.416102 | 0.617443 | 3.761959 |
| SLC38A1 | 0.691228 | 0.416111 | 0.377754 | 4.201138 |
| LOC389247 | 0.691228 | 0.416193 | 1.012398 | 1.209409 |
| STIP1 | 0.691228 | 0.416225 | 1.168691 | 0.767505 |
| POLR3B | 0.691228 | 0.416254 | 1.104292 | 1.008393 |
| TONSL | 0.691454 | 0.416443 | 0.402052 | 4.751746 |
| CSKMT | 0.691486 | 0.416637 | 0.579851 | 2.21894 |
| CAMLG | 0.691486 | 0.416671 | -0.3521 | 4.723787 |
| STAU2-AS1 | 0.691486 | 0.416723 | 0.767399 | 1.237083 |
| FABP2 | 0.691486 | 0.416735 | 1.368371 | 0.86984 |
| TMX3 | 0.691486 | 0.416774 | 0.332495 | 5.956058 |
| ASB9P1 | 0.691486 | 0.416778 | 0.752612 | 1.30202 |
| TMEM161B | 0.69222 | 0.417273 | 0.684243 | 2.55392 |
| ZNF530 | 0.692245 | 0.417341 | 0.880683 | 1.23743 |
| LOC101929 | 0.692289 | 0.417444 | -0.85827 | 2.017292 |
| MBD6 | 0.692289 | 0.417478 | 0.288679 | 6.80621 |
| ARMC5 | 0.692289 | 0.417526 | -0.81527 | 2.003557 |
| DDHD2 | 0.692806 | 0.417931 | 1.074792 | 0.785386 |
| MRM3 | 0.692806 | 0.417944 | -0.80002 | 1.225934 |
| LOC339666 | 0.69294 | 0.418085 | -1.00398 | 0.90584 |
| LRP4 | 0.69294 | 0.418223 | 0.658764 | 6.448795 |
| MXD3 | 0.69294 | 0.418231 | -0.74549 | 1.30768 |

| | | | | |
|---|---|---|---|---|
| GOLGA6L9 | 0.69294 | 0.418235 | -1.28148 | 0.831089 |
| HSF1 | 0.693145 | 0.418412 | 0.303169 | 5.692397 |
| KBTBD2 | 0.693198 | 0.418497 | 0.302829 | 6.23262 |
| EEF2 | 0.69321 | 0.418557 | -0.30519 | 10.91992 |
| RPS21 | 0.69325 | 0.418634 | -0.34826 | 7.622716 |
| HERC4 | 0.693619 | 0.418909 | 0.420334 | 3.570689 |
| BMP7 | 0.694237 | 0.419336 | -0.81701 | 5.22817 |
| EFCAB5 | 0.694424 | 0.419502 | -0.89118 | 1.124828 |
| LCMT1-AS2 | 0.694424 | 0.419563 | -0.50669 | 3.434809 |
| CAMK1G | 0.694424 | 0.419626 | 0.933542 | 0.695799 |
| INPP1 | 0.694424 | 0.41966 | 0.972553 | 0.810785 |
| ZFP41 | 0.694442 | 0.419724 | 0.401534 | 4.074052 |
| FAM153A | 0.694511 | 0.419855 | 1.477898 | 1.052474 |
| CCT6A | 0.694511 | 0.419872 | 1.189281 | 0.610692 |
| CFAP74 | 0.694729 | 0.420089 | 1.317698 | 0.548655 |
| SHANK2-AS | 0.694729 | 0.420109 | 0.580353 | 2.539775 |
| MFAP1 | 0.694802 | 0.420219 | -0.28749 | 6.617586 |
| OSM | 0.694802 | 0.420278 | 1.281724 | 1.918976 |
| ATAD3B | 0.694802 | 0.420312 | 0.470752 | 3.526689 |
| ARMC6 | 0.695 | 0.420485 | 0.88947 | 0.998618 |
| ARHGAP1 | 0.69501 | 0.420544 | -0.29739 | 7.480157 |
| FAM86B3P | 0.695067 | 0.420631 | 0.833351 | 2.15581 |
| TM9SF2 | 0.695084 | 0.420695 | 0.30714 | 7.90266 |
| LINC02502 | 0.695196 | 0.420816 | -2.00163 | 0.529596 |
| RNF112 | 0.695212 | 0.420883 | 0.951841 | 1.521446 |
| KXD1 | 0.695212 | 0.420946 | -0.88353 | 0.701823 |
| MIR4534 | 0.695212 | 0.420986 | 1.462748 | 0.392678 |
| CCT3 | 0.695212 | 0.421037 | -0.68271 | 1.973137 |
| LOC401312 | 0.69547 | 0.421246 | -1.79521 | 0.563241 |
| ADAR | 0.695895 | 0.421609 | 1.16913 | 0.875923 |
| PATZ1 | 0.695895 | 0.421664 | 0.89294 | 2.340681 |
| TRAPPC12 | 0.695895 | 0.421757 | 0.339855 | 5.551499 |
| SH3BP2 | 0.695895 | 0.421799 | -0.84972 | 1.587584 |
| PTGER4 | 0.695895 | 0.421802 | 0.481795 | 4.340251 |
| IKZF1 | 0.695895 | 0.421822 | 1.107917 | 0.991148 |
| SQOR | 0.696061 | 0.421976 | 1.116197 | 0.80837 |
| PKD1L3 | 0.696164 | 0.422097 | 0.8616 | 1.862314 |
| NAF1 | 0.696164 | 0.422144 | -0.57616 | 2.563389 |
| ZRANB2-AS | 0.696188 | 0.422212 | 1.009331 | 0.929565 |
| NR4A2 | 0.696221 | 0.42232 | 0.649902 | 6.098708 |
| FAM199X | 0.696221 | 0.422338 | 0.295202 | 7.078219 |
| EPM2A | 0.696419 | 0.42256 | 0.839844 | 2.309074 |
| UHRF1BP1 | 0.696419 | 0.422579 | 0.327447 | 6.225239 |
| WASHC5 | 0.696419 | 0.422703 | 0.302882 | 6.382521 |
| UNC5A | 0.696419 | 0.422723 | 1.091204 | 0.951357 |

| | | | | |
|---|---|---|---|---|
| CHML | 0.696419 | 0.422724 | 0.314902 | 5.627092 |
| BCR | 0.69644 | 0.422789 | -0.74561 | 1.158585 |
| MIR8085 | 0.696445 | 0.422898 | -0.91386 | 0.66853 |
| UEVLD | 0.696445 | 0.422918 | 1.114991 | 0.470571 |
| USP3 | 0.696445 | 0.422981 | 0.966308 | 0.761965 |
| GPR143 | 0.696445 | 0.423013 | 0.954771 | 1.698352 |
| MAPKAPK3 | 0.696445 | 0.423063 | 1.229512 | 0.733257 |
| XPOT | 0.696445 | 0.423111 | -0.35337 | 7.500366 |
| LOC100287 | 0.696571 | 0.42324 | -0.6439 | 2.511316 |
| TAF1D | 0.696644 | 0.423338 | 0.365041 | 6.899646 |
| LPP-AS2 | 0.696965 | 0.423586 | 0.536735 | 3.287026 |
| FBXO11 | 0.6971 | 0.423721 | 0.692345 | 2.09818 |
| LOC100126 | 0.697146 | 0.423808 | 0.523067 | 3.13909 |
| LCNL1 | 0.697146 | 0.423855 | -1.04555 | 0.915629 |
| DDAH1 | 0.697258 | 0.423977 | 0.582253 | 2.287978 |
| RPL19P12 | 0.697285 | 0.424048 | 0.594775 | 2.334971 |
| GNPNAT1 | 0.697285 | 0.424121 | 0.401282 | 5.526259 |
| LEO1 | 0.697285 | 0.424195 | -0.55434 | 2.428174 |
| LUCAT1 | 0.697285 | 0.424226 | 0.888778 | 1.695376 |
| LOC101928 | 0.697285 | 0.424267 | -0.46801 | 3.236892 |
| ZNF362 | 0.697285 | 0.424312 | 0.372604 | 5.041234 |
| LINC00992 | 0.69746 | 0.424521 | 1.506374 | 0.977437 |
| DEAF1 | 0.69746 | 0.424525 | -0.81909 | 0.678016 |
| SNORA79 | 0.697585 | 0.424654 | -0.68612 | 2.496086 |
| SRL | 0.697849 | 0.424868 | -0.95348 | 0.919036 |
| VAMP2 | 0.697898 | 0.424951 | -0.31251 | 6.069764 |
| THYN1 | 0.698358 | 0.425308 | -0.94891 | 0.792528 |
| MRPS21 | 0.698358 | 0.425337 | 0.797438 | 1.889808 |
| TMEM81 | 0.698401 | 0.425503 | -0.8968 | 1.569487 |
| CD36 | 0.698401 | 0.425543 | 0.766118 | 2.324253 |
| SCARNA11 | 0.698401 | 0.425546 | 0.523758 | 3.246299 |
| ACAD9 | 0.698401 | 0.425611 | 0.74861 | 1.582341 |
| HIST1H3A | 0.698401 | 0.42563 | -0.67607 | 2.545221 |
| UBA1 | 0.698626 | 0.42582 | -0.49383 | 3.421587 |
| SAMD12 | 0.69876 | 0.425955 | 0.531896 | 3.348871 |
| ARC | 0.698916 | 0.426131 | -0.91917 | 1.922939 |
| TM7SF3 | 0.698916 | 0.426206 | 0.302701 | 6.873313 |
| SOCS1 | 0.698916 | 0.42621 | 0.722415 | 2.43601 |
| ADCK2 | 0.699127 | 0.426392 | -0.45335 | 4.144391 |
| SUPT3H | 0.699202 | 0.426491 | 1.387976 | 0.609594 |
| TUSC2 | 0.69925 | 0.426573 | 0.491895 | 3.701509 |
| C17orf107 | 0.69936 | 0.426694 | -0.78104 | 1.86828 |
| NCOA7 | 0.699473 | 0.426837 | 0.553936 | 2.913999 |
| PDSS2 | 0.699473 | 0.426984 | -0.36265 | 5.09117 |
| REM2 | 0.699473 | 0.427074 | -0.9034 | 0.739145 |

| | | | | |
|---|---|---|---|---|
| GSTCD | 0.699473 | 0.427096 | -0.87556 | 1.267331 |
| PLEK2 | 0.699473 | 0.427104 | 0.513198 | 3.999872 |
| CUL4B | 0.699473 | 0.427119 | 0.677203 | 1.826196 |
| MAGEH1 | 0.699473 | 0.427151 | 0.722251 | 2.173615 |
| SNORA72 | 0.699473 | 0.427217 | 0.442818 | 5.322171 |
| LINC00663 | 0.699473 | 0.427243 | 0.994413 | 1.453629 |
| STRN3 | 0.699589 | 0.42746 | -0.47091 | 3.41962 |
| CRYZL1 | 0.699589 | 0.427463 | 0.354736 | 4.907631 |
| INTS8 | 0.699589 | 0.427474 | 1.111781 | 0.426289 |
| NCOA4 | 0.699776 | 0.427641 | -1.09082 | 0.920222 |
| SNORD3C | 0.699786 | 0.427739 | 0.456497 | 8.490142 |
| ZNF503 | 0.699786 | 0.4278 | -0.39762 | 4.712801 |
| CNN3 | 0.699786 | 0.42788 | -0.65163 | 2.638744 |
| PRR22 | 0.699786 | 0.427882 | 0.821032 | 2.181591 |
| ERO1B | 0.699786 | 0.427934 | -0.66134 | 5.35478 |
| RAP2C-AS1 | 0.699786 | 0.427967 | -0.55229 | 2.941489 |
| INHA | 0.700114 | 0.428221 | 1.223408 | 0.499704 |
| TTLL11 | 0.700221 | 0.42834 | 0.638025 | 1.789131 |
| MRPS25 | 0.700358 | 0.428521 | -0.292 | 6.395897 |
| UFC1 | 0.700358 | 0.42858 | 0.301929 | 5.626069 |
| MYO1H | 0.700358 | 0.428584 | 0.802784 | 1.781451 |
| ALG2 | 0.700929 | 0.428986 | -0.89131 | 0.824595 |
| IGF2BP3 | 0.701041 | 0.429108 | 0.547916 | 4.99359 |
| CXCR6 | 0.701249 | 0.42937 | -0.41961 | 3.895792 |
| RAB12 | 0.701249 | 0.429479 | -0.35841 | 6.258206 |
| HEXIM2 | 0.701249 | 0.429489 | 1.187293 | 0.819828 |
| KCNAB3 | 0.701249 | 0.429493 | 0.825661 | 1.534994 |
| DDX28 | 0.701249 | 0.429543 | -0.46726 | 2.759589 |
| MIR200B | 0.701249 | 0.429603 | 0.620477 | 1.861733 |
| VWA8 | 0.701249 | 0.42961 | 0.319565 | 5.131394 |
| BCL6 | 0.701308 | 0.429699 | -0.65499 | 2.562553 |
| AGPAT4 | 0.701405 | 0.429882 | -0.30829 | 5.474339 |
| AP3D1 | 0.701405 | 0.429895 | -0.64862 | 2.740288 |
| RASSF1-AS | 0.701405 | 0.429919 | -0.91742 | 1.050754 |
| MIR646HG | 0.701435 | 0.429991 | 0.967301 | 1.147834 |
| PLPP7 | 0.701838 | 0.430335 | 1.170442 | 0.304688 |
| DNAJC13 | 0.701838 | 0.430391 | 0.280927 | 7.620698 |
| RBM6 | 0.701838 | 0.430399 | 0.283116 | 6.898325 |
| OMD | 0.701841 | 0.4305 | 0.618856 | 3.443135 |
| SLC7A4 | 0.701841 | 0.430507 | -0.76363 | 2.169576 |
| LOC10537S | 0.70187 | 0.430579 | -1.21374 | 0.526324 |
| ZNF81 | 0.70206 | 0.430749 | 0.352818 | 4.927489 |
| TLL2 | 0.702088 | 0.43082 | 0.871682 | 2.291197 |
| PLEC | 0.702518 | 0.431175 | 0.360523 | 5.441689 |
| PRPF6 | 0.702518 | 0.431191 | 0.315949 | 7.456673 |

| | | | | |
|---|---|---|---|---|
| CHPT1 | 0.702659 | 0.431394 | 0.400594 | 4.658925 |
| LINC01554 | 0.702659 | 0.431398 | 1.000195 | 0.468986 |
| KANSL1-AS | 0.702659 | 0.431437 | 1.273093 | 0.559947 |
| PAQR3 | 0.702767 | 0.431557 | -0.39391 | 4.269326 |
| ZNF316 | 0.70309 | 0.431809 | 0.295617 | 6.157318 |
| PLEKHO2 | 0.703373 | 0.43211 | 0.924137 | 0.992673 |
| SLX4IP | 0.703373 | 0.432127 | 0.421665 | 3.803402 |
| LAMA3 | 0.703373 | 0.432188 | 1.73755 | 0.248432 |
| SLC11A2 | 0.703373 | 0.432199 | 0.809273 | 1.97728 |
| MCTS1 | 0.703373 | 0.432251 | -0.84843 | 1.355365 |
| PRPF8 | 0.70346 | 0.432358 | -0.34256 | 6.401787 |
| ZNF727 | 0.703572 | 0.432481 | 1.183911 | 1.155734 |
| C17orf75 | 0.703588 | 0.432544 | 0.389147 | 3.734397 |
| ATF3 | 0.703671 | 0.432653 | 1.180596 | 1.091743 |
| KIAA0895L | 0.703671 | 0.432703 | 0.328801 | 5.279966 |
| FMO4 | 0.703743 | 0.432842 | 0.664602 | 3.344756 |
| SLC22A4 | 0.703743 | 0.432854 | 0.693513 | 1.902159 |
| LRG1 | 0.703966 | 0.433061 | -0.57467 | 3.914315 |
| DERL2 | 0.703966 | 0.433099 | -0.95324 | 1.398969 |
| ECPAS | 0.704296 | 0.433355 | -0.28666 | 8.292008 |
| BUB1B | 0.704316 | 0.433421 | 0.389721 | 5.314475 |
| RPS6KA5 | 0.704578 | 0.433636 | 0.596687 | 3.441144 |
| GABRA3 | 0.704753 | 0.433797 | -1.40455 | 1.618569 |
| POLA2 | 0.704795 | 0.433933 | 0.363763 | 4.487451 |
| SH3BGRL | 0.704795 | 0.433979 | 0.297484 | 6.285911 |
| INF2 | 0.704795 | 0.433985 | 0.559948 | 3.037727 |
| NUP54 | 0.704858 | 0.434124 | 0.872321 | 0.887821 |
| CDC34 | 0.704858 | 0.43413 | -0.30248 | 4.878927 |
| TTC12 | 0.705129 | 0.434351 | 0.407693 | 4.31751 |
| GAD1 | 0.705232 | 0.434468 | 0.842643 | 1.803356 |
| FBL | 0.705551 | 0.434719 | -0.3036 | 6.679404 |
| SLC25A3 | 0.705691 | 0.434859 | -0.62758 | 2.505489 |
| TMEM38A | 0.705853 | 0.435012 | -0.75125 | 1.912166 |
| MIR29B2 | 0.705993 | 0.435152 | 0.75907 | 1.454422 |
| ASMTL-AS1 | 0.706076 | 0.435257 | 0.793299 | 0.953989 |
| MIR133A1I | 0.706325 | 0.435501 | 0.400891 | 3.689017 |
| VWF | 0.706325 | 0.435518 | 0.329787 | 7.716048 |
| CCNYL1 | 0.706694 | 0.435828 | -0.7604 | 1.634105 |
| ADSL | 0.706694 | 0.435854 | -1.22721 | 0.324134 |
| LIPK | 0.706978 | 0.436083 | -1.41268 | 0.823641 |
| NDUFB2-A | 0.70699 | 0.436144 | -1.03672 | 2.05777 |
| LRRC1 | 0.707173 | 0.436311 | -0.32963 | 6.154476 |
| C1QTNF8 | 0.707411 | 0.436512 | 1.324003 | 1.092465 |
| C1QBP | 0.70753 | 0.436644 | -0.3463 | 6.0758 |
| HOTAIRM1 | 0.70753 | 0.436744 | -0.77064 | 2.668919 |

| | | | | |
|---|---|---|---|---|
| PMS2P7 | 0.70753 | 0.436767 | 0.493537 | 2.542669 |
| YEATS2 | 0.70753 | 0.436825 | -0.30137 | 7.276619 |
| SEC63 | 0.70753 | 0.436921 | 0.280569 | 7.733873 |
| SNORD11B | 0.70753 | 0.43694 | 0.69715 | 2.353351 |
| ARHGEF18 | 0.70753 | 0.436962 | -0.69576 | 1.880898 |
| CWC22 | 0.707728 | 0.437139 | 0.27091 | 6.531271 |
| SLC30A5 | 0.708042 | 0.437387 | 0.279792 | 5.743658 |
| C1QTNF12 | 0.708055 | 0.437449 | 0.785732 | 1.229604 |
| ZNF658B | 0.708273 | 0.437683 | -0.52008 | 2.840297 |
| MRPL20-AS | 0.708273 | 0.437692 | 0.463546 | 4.008848 |
| HS3ST4 | 0.708431 | 0.437843 | -0.87644 | 1.000766 |
| UACA | 0.708612 | 0.438009 | -0.45714 | 3.649798 |
| SMLR1 | 0.708871 | 0.438223 | 1.319916 | 0.459003 |
| CDKN1B | 0.708959 | 0.438332 | -0.27588 | 6.39377 |
| GNG7 | 0.709318 | 0.438607 | 0.601054 | 3.062331 |
| HUS1 | 0.709325 | 0.438666 | -0.84024 | 1.653248 |
| PDRG1 | 0.709716 | 0.439014 | 0.552464 | 3.50675 |
| LINC01547 | 0.709716 | 0.439016 | 1.095771 | 1.325016 |
| SLC15A4 | 0.709829 | 0.43914 | 0.312635 | 4.810374 |
| ZMPSTE24 | 0.70995 | 0.439269 | 0.322369 | 6.811748 |
| TAF12 | 0.710034 | 0.439402 | -0.64046 | 2.289269 |
| CALM3 | 0.710034 | 0.439429 | 0.272726 | 7.804593 |
| UBR2 | 0.710131 | 0.439543 | 0.274124 | 7.372575 |
| CCDC115 | 0.71059 | 0.439881 | -0.69961 | 1.597027 |
| ZYG11B | 0.710598 | 0.43994 | -0.28529 | 6.308034 |
| SCAF1 | 0.710686 | 0.440172 | 0.28074 | 6.696981 |
| BRI3BP | 0.710686 | 0.440208 | 0.48057 | 3.202875 |
| TNRC6A | 0.710686 | 0.440214 | 0.287626 | 8.007029 |
| SMARCD3 | 0.710686 | 0.440262 | -0.64098 | 2.117109 |
| NAV2 | 0.710686 | 0.440266 | 0.854393 | 1.637352 |
| CCDC149 | 0.710701 | 0.440349 | 1.138263 | 0.747608 |
| BBS10 | 0.710701 | 0.440383 | -0.40852 | 4.087279 |
| MLLT6 | 0.710756 | 0.44048 | 0.471623 | 3.929856 |
| GPBP1L1 | 0.710756 | 0.440535 | -0.28034 | 7.786746 |
| LOC100132 | 0.710756 | 0.440637 | 1.232605 | 0.662341 |
| MEIS2 | 0.710756 | 0.440682 | 0.658432 | 1.614759 |
| LOC400706 | 0.710756 | 0.440715 | 1.175579 | 2.613298 |
| CPLANE1 | 0.710756 | 0.440767 | 0.291993 | 6.91514 |
| ADAMTS7F | 0.710756 | 0.440796 | 1.092385 | 0.75122 |
| CACYBP | 0.710904 | 0.440943 | 0.518084 | 2.524158 |
| TEFM | 0.710974 | 0.44104 | 0.397038 | 3.580549 |
| LOC100289 | 0.711042 | 0.441136 | -0.96015 | 1.04954 |
| NDUFS2 | 0.711348 | 0.441398 | 0.75301 | 1.491675 |
| VWA5A | 0.711348 | 0.441435 | 1.077312 | 0.912315 |
| CBX5 | 0.711593 | 0.441665 | 0.694418 | 2.149425 |

| | | | | |
|---|---|---|---|---|
| KLHL9 | 0.711593 | 0.441695 | 0.312446 | 5.478867 |
| RNU5B-1 | 0.711685 | 0.441807 | 0.473781 | 8.93951 |
| ORC4 | 0.711705 | 0.441873 | 0.859351 | 1.527333 |
| HNRNPDL | 0.711796 | 0.441984 | -0.48673 | 3.260374 |
| DENND6A | 0.711965 | 0.442159 | -0.26616 | 6.109394 |
| PCSK6-AS1 | 0.711965 | 0.442198 | 0.815997 | 1.999578 |
| TDRD3 | 0.712087 | 0.442327 | 0.894195 | 0.705683 |
| ZNF146 | 0.712194 | 0.442448 | 0.960254 | 0.73685 |
| CMC2 | 0.712344 | 0.442763 | -0.40475 | 3.57124 |
| SNORA74B | 0.712344 | 0.442768 | 0.419038 | 5.347187 |
| TMSB10 | 0.712344 | 0.442786 | -0.30254 | 9.246247 |
| TPCN1 | 0.712344 | 0.442799 | -0.89318 | 1.780868 |
| RASA1 | 0.712344 | 0.442813 | -0.38835 | 3.49381 |
| LOC72973[illegible] | 0.712626 | 0.443043 | -0.37343 | 4.73788 |
| GNAS | 0.712678 | 0.443129 | 0.660368 | 1.74116 |
| DPRXP4 | 0.713033 | 0.443404 | -0.6779 | 1.682626 |
| PER1 | 0.713133 | 0.443565 | -0.35299 | 7.456203 |
| STT3B | 0.713133 | 0.443575 | -0.30112 | 8.012399 |
| PCARE | 0.713142 | 0.443635 | -1.17353 | 0.953117 |
| SLU7 | 0.713183 | 0.443715 | -0.28016 | 6.945207 |
| ZNF383 | 0.713681 | 0.444105 | 0.41391 | 3.478379 |
| EEF1D | 0.713681 | 0.444133 | -1.23893 | 1.774837 |
| PDE4A | 0.713748 | 0.444245 | 1.236701 | 0.495607 |
| MT2A | 0.713748 | 0.444334 | 0.439564 | 6.305855 |
| LINC00324 | 0.713748 | 0.444441 | -0.72082 | 2.284254 |
| HASPIN | 0.713748 | 0.444482 | -0.5968 | 3.272544 |
| SNORD54 | 0.713748 | 0.444502 | 0.583482 | 3.245133 |
| RPS27 | 0.713748 | 0.44453 | 0.356084 | 7.246526 |
| GOLGA5 | 0.713748 | 0.444638 | -0.30636 | 6.371677 |
| TRIM68 | 0.713748 | 0.444678 | -0.80939 | 1.259654 |
| TAS2R19 | 0.713748 | 0.444681 | 0.954072 | 0.765755 |
| GABARAPL | 0.713748 | 0.444804 | 0.37082 | 5.939802 |
| CTC1 | 0.713748 | 0.444848 | 1.186646 | 0.412233 |
| CEBPZ | 0.713748 | 0.444881 | 0.310534 | 6.772568 |
| SLC8A1-AS | 0.713748 | 0.444882 | 0.566273 | 2.598839 |
| CHCHD5 | 0.713752 | 0.444939 | -1.30335 | 0.353822 |
| GLOD4 | 0.713754 | 0.444995 | -0.29611 | 5.880351 |
| MDH2 | 0.714298 | 0.445389 | -0.87938 | 0.790113 |
| KDM1B | 0.714402 | 0.445508 | 0.269457 | 6.007538 |
| SNORD49A | 0.71441 | 0.445635 | 0.580004 | 3.175167 |
| MMACHC | 0.71441 | 0.445672 | 0.448549 | 2.928393 |
| NKAPP1 | 0.71441 | 0.445676 | 0.613501 | 2.497442 |
| CCDC174 | 0.714971 | 0.446164 | 0.288185 | 5.817457 |
| ACTR1A | 0.714971 | 0.446233 | -0.29393 | 6.465098 |
| MIB2 | 0.714971 | 0.446338 | 1.033164 | 0.676043 |

| | | | | |
|---|---|---|---|---|
| VPS26B | 0.714971 | 0.446353 | -0.27923 | 6.063913 |
| SHCBP1 | 0.714971 | 0.44642 | 0.385351 | 3.786034 |
| POLE | 0.714971 | 0.446466 | 0.298454 | 6.749082 |
| CCT8 | 0.714971 | 0.446507 | 0.557947 | 2.869617 |
| SLC46A3 | 0.714971 | 0.446514 | 1.138548 | 1.103526 |
| SRCAP | 0.714971 | 0.446516 | 0.285876 | 8.768918 |
| RPL13AP5 | 0.715152 | 0.446701 | -0.29994 | 5.520376 |
| PDE10A | 0.715152 | 0.446788 | 1.318667 | 0.507261 |
| PSMA6 | 0.715152 | 0.446841 | -0.88053 | 0.403089 |
| SPESP1 | 0.715152 | 0.446867 | 0.94289 | 1.369067 |
| HIST2H2BC | 0.715152 | 0.446902 | 0.606085 | 2.502931 |
| HOXB-AS1 | 0.715513 | 0.447182 | 0.968799 | 0.851738 |
| C1orf87 | 0.715514 | 0.447237 | -1.38009 | 0.63363 |
| YIF1A | 0.715826 | 0.447487 | 0.944662 | 0.868797 |
| SPC25 | 0.716029 | 0.447785 | -0.97308 | 1.176267 |
| FOXRED1 | 0.716029 | 0.447863 | 0.773161 | 2.174326 |
| PTDSS1 | 0.716029 | 0.447866 | -0.81562 | 1.38659 |
| TBC1D3P1- | 0.716029 | 0.447873 | 0.678739 | 1.814715 |
| SIDT2 | 0.716029 | 0.447943 | 0.324809 | 5.659657 |
| POP4 | 0.716029 | 0.448021 | 0.803018 | 0.970617 |
| C2orf48 | 0.716029 | 0.448095 | 0.694373 | 2.340074 |
| FAM86EP | 0.716029 | 0.448129 | -0.94181 | 0.747996 |
| DTX3 | 0.716029 | 0.448228 | 1.093716 | 0.597404 |
| TIMM29 | 0.716029 | 0.448255 | -0.41738 | 3.141662 |
| TBC1D2 | 0.716029 | 0.448289 | -0.8251 | 1.001834 |
| NALT1 | 0.716029 | 0.448307 | 0.962596 | 1.022457 |
| MEX3A | 0.716029 | 0.448323 | 0.455103 | 4.584818 |
| LINC01635 | 0.716045 | 0.448388 | -1.05077 | 0.514474 |
| WDR33 | 0.716075 | 0.448474 | 0.265175 | 6.795967 |
| MTMR12 | 0.716075 | 0.448516 | -0.82491 | 1.324676 |
| SZRD1 | 0.716319 | 0.448723 | 1.097592 | 1.377479 |
| DNHD1 | 0.716606 | 0.449019 | 0.337905 | 5.682329 |
| MGAT2 | 0.716606 | 0.449042 | 0.371536 | 4.780668 |
| MIR4676 | 0.716606 | 0.44912 | 1.122556 | 1.377049 |
| C12orf49 | 0.716606 | 0.449121 | 0.286661 | 5.731145 |
| RBL2 | 0.716687 | 0.449262 | -0.27952 | 7.485825 |
| CAD | 0.716687 | 0.449281 | 1.036735 | 0.698921 |
| HIST1H2AC | 0.716855 | 0.449441 | -0.28729 | 5.620259 |
| MRM1 | 0.716952 | 0.449557 | -1.17899 | 0.595651 |
| TAS2R4 | 0.717182 | 0.449756 | 0.841639 | 1.731252 |
| ANKRD11 | 0.717501 | 0.450136 | -0.53081 | 3.442092 |
| BTBD3 | 0.717501 | 0.450155 | 1.027647 | 0.760297 |
| HHIPL2 | 0.717501 | 0.450161 | 1.303141 | 0.703003 |
| CKAP5 | 0.717501 | 0.450174 | 0.582971 | 2.284871 |
| RPL13AP3 | 0.71774 | 0.450379 | -1.02278 | 0.33059 |

| | | | | |
|---|---|---|---|---|
| EIF1AX-AS1 | 0.717743 | 0.450436 | 1.061899 | 0.744841 |
| STAG3L3 | 0.717805 | 0.450529 | -0.38569 | 4.111733 |
| LINC00909 | 0.718266 | 0.450931 | 0.441566 | 3.327455 |
| CCDC32 | 0.718266 | 0.450949 | 1.110285 | 0.765615 |
| GAS1RR | 0.718266 | 0.450983 | 1.443167 | 0.789143 |
| STAG1 | 0.718338 | 0.451088 | 0.278318 | 7.146915 |
| LOC72908C | 0.718338 | 0.451138 | -1.36067 | 1.171927 |
| KATNAL2 | 0.718493 | 0.451342 | 0.46181 | 3.212732 |
| FABP4 | 0.718493 | 0.451344 | 1.00956 | 0.668334 |
| CENPBD1 | 0.718605 | 0.451469 | -0.50414 | 2.568962 |
| MIR200A | 0.71901 | 0.451779 | 0.815172 | 1.826049 |
| OBSCN-AS1 | 0.71901 | 0.451833 | -0.71743 | 1.38525 |
| GOLT1B | 0.719012 | 0.451985 | 0.388596 | 5.270183 |
| PLEKHG2 | 0.719012 | 0.451991 | 0.32009 | 6.224265 |
| FAM43B | 0.719012 | 0.451999 | -0.94324 | 0.473661 |
| TEKT4P2 | 0.719027 | 0.452063 | 0.737216 | 1.502368 |
| SNORA15 | 0.719274 | 0.452318 | 1.005422 | 0.72479 |
| HIST2H2AC | 0.719274 | 0.452332 | 0.343169 | 4.874695 |
| SNORD28 | 0.719274 | 0.452406 | -0.47414 | 4.287133 |
| SREK1 | 0.719274 | 0.452438 | 0.367336 | 5.805831 |
| SLC4A5 | 0.719553 | 0.452668 | 0.591526 | 3.037633 |
| MYH9 | 0.719723 | 0.45283 | 0.269718 | 11.26181 |
| NAALADL2- | 0.720255 | 0.45322 | -1.28402 | 0.520106 |
| GIT2 | 0.720257 | 0.453276 | 0.523742 | 2.949756 |
| RND1 | 0.72033 | 0.453452 | 0.93169 | 2.649729 |
| ZNF442 | 0.72033 | 0.453474 | 0.753457 | 2.079027 |
| RNU4-1 | 0.72033 | 0.453486 | 0.366653 | 8.609269 |
| MIR544B | 0.720347 | 0.453552 | 1.14226 | 0.665194 |
| CUTC | 0.720624 | 0.453878 | 0.486396 | 3.828888 |
| CABIN1 | 0.720624 | 0.453901 | 0.780703 | 1.166998 |
| WAC-AS1 | 0.720624 | 0.453954 | 0.269838 | 5.879807 |
| PPP6R1 | 0.720624 | 0.454025 | 0.284366 | 7.642199 |
| NSMCE1-D | 0.720624 | 0.454027 | -0.84962 | 1.928913 |
| DGKI | 0.720624 | 0.454056 | 0.722802 | 2.570801 |
| C6orf106 | 0.720639 | 0.454241 | 0.41912 | 3.132041 |
| LINC00504 | 0.720639 | 0.454281 | -0.75922 | 1.193405 |
| ZNF436-AS | 0.720639 | 0.454322 | 0.553851 | 2.471994 |
| NSMF | 0.720639 | 0.454348 | 1.22238 | 0.43435 |
| SLC35E1 | 0.720639 | 0.454423 | -0.27304 | 6.699825 |
| TXLNGY | 0.720639 | 0.454442 | -0.82678 | 5.192429 |
| NUCKS1 | 0.720639 | 0.45448 | 0.283442 | 9.153022 |
| HTRA2 | 0.720639 | 0.454529 | -0.87726 | 0.836073 |
| KMT2E | 0.720639 | 0.454559 | 0.906134 | 1.551068 |
| SPATA21 | 0.720735 | 0.454692 | -0.87361 | 0.939115 |
| GALNTL6 | 0.720735 | 0.45473 | 1.695544 | 1.102634 |

| | | | | |
|---|---|---|---|---|
| MFN2 | 0.720922 | 0.454913 | 0.530418 | 2.446367 |
| MIR3936H( | 0.720922 | 0.454998 | 0.574689 | 2.140583 |
| PTPRR | 0.720922 | 0.455013 | 1.545645 | 0.619895 |
| LOC10192( | 0.721078 | 0.455166 | -1.0302 | 0.443772 |
| KCNA7 | 0.721762 | 0.455653 | 0.846401 | 1.220025 |
| TRAK2 | 0.721969 | 0.455894 | 0.30481 | 6.725795 |
| LINC02026 | 0.721969 | 0.455972 | -1.55637 | 0.595498 |
| DLG3 | 0.721969 | 0.456023 | -0.57758 | 2.493487 |
| DLEU2L | 0.721969 | 0.45612 | 0.635234 | 2.332468 |
| TMEM125 | 0.721969 | 0.456221 | 0.377846 | 3.775362 |
| TRHDE | 0.721969 | 0.456225 | -1.37398 | 0.740529 |
| LOC10050( | 0.721969 | 0.456245 | -1.62873 | 0.545731 |
| TPSAB1 | 0.721969 | 0.456275 | 0.529814 | 3.348452 |
| SUMO4 | 0.721969 | 0.456278 | 0.672643 | 2.336442 |
| PARP8 | 0.721997 | 0.456352 | 1.013659 | 0.813706 |
| LGALS2 | 0.722105 | 0.456522 | 0.644488 | 2.92928 |
| ATAD1 | 0.722105 | 0.456529 | 0.308279 | 6.615775 |
| C17orf97 | 0.722312 | 0.456716 | -0.41246 | 3.567542 |
| BCDIN3D | 0.722514 | 0.456901 | -0.60398 | 2.577777 |
| PLXNB2 | 0.722514 | 0.456954 | -0.27297 | 9.003017 |
| ZSCAN29 | 0.722588 | 0.457157 | 0.283627 | 6.173785 |
| AXIN1 | 0.722588 | 0.457185 | 1.074101 | 0.594316 |
| SMIM14 | 0.722588 | 0.457218 | -0.88315 | 0.675053 |
| DNAH9 | 0.722588 | 0.457298 | -0.90195 | 1.561048 |
| ADGRL4 | 0.722588 | 0.457306 | 0.560858 | 3.786359 |
| CHTF8 | 0.722588 | 0.457331 | -0.86206 | 1.552653 |
| SLCO4A1 | 0.722765 | 0.457538 | -0.40331 | 5.654013 |
| HSPA1B | 0.722765 | 0.457553 | -0.62779 | 4.755311 |
| SLC25A28 | 0.722987 | 0.45779 | 0.301987 | 5.006705 |
| FLJ22447 | 0.722987 | 0.45788 | 0.967469 | 0.739132 |
| ANK3 | 0.722987 | 0.457954 | -0.36791 | 5.523917 |
| PIAS4 | 0.722987 | 0.457958 | 0.30714 | 5.209195 |
| LOC38964: | 0.722987 | 0.457969 | 0.59987 | 2.001056 |
| UBE2Q2 | 0.723028 | 0.45805 | 0.773607 | 0.910447 |
| PKM | 0.723171 | 0.458204 | -0.82962 | 1.728332 |
| EIF2B1 | 0.723171 | 0.458251 | -0.28063 | 5.517994 |
| ROR1 | 0.723207 | 0.458329 | 0.550782 | 4.093326 |
| PRSS23 | 0.723392 | 0.458547 | 0.530377 | 2.552627 |
| ZNF541 | 0.723392 | 0.458556 | -0.9014 | 0.91423 |
| SEC14L1P1 | 0.72353 | 0.458724 | 0.606139 | 2.438637 |
| CENPQ | 0.72353 | 0.458754 | 0.719716 | 2.399975 |
| PNPO | 0.723851 | 0.459013 | 0.287106 | 5.69853 |
| DOC2B | 0.724048 | 0.459323 | -0.69732 | 3.016922 |
| UBFD1 | 0.724048 | 0.459377 | 0.286007 | 6.178856 |
| UBR3 | 0.724048 | 0.459383 | -0.25946 | 7.386539 |

| | | | | |
|---|---|---|---|---|
| ESR2 | 0.724048 | 0.459468 | 0.942454 | 0.99058 |
| TNRC18P1 | 0.724048 | 0.459502 | -0.51574 | 2.378201 |
| PRKAA2 | 0.724048 | 0.459504 | 0.409224 | 4.768738 |
| HMGN2 | 0.724048 | 0.459524 | -0.26403 | 6.495231 |
| RBM12B-A | 0.724191 | 0.45967 | 0.399715 | 3.005094 |
| EXOSC8 | 0.724492 | 0.459916 | 0.47005 | 4.166307 |
| DPY19L4 | 0.724568 | 0.46002 | 0.298699 | 5.819906 |
| HERC2P7 | 0.724864 | 0.460263 | 0.419047 | 3.395506 |
| HSPE1 | 0.724873 | 0.460348 | 0.57425 | 2.911775 |
| SLK | 0.724873 | 0.460379 | -0.77988 | 1.74672 |
| BACH1-IT2 | 0.724895 | 0.460459 | 0.574483 | 2.692356 |
| TMEM221 | 0.724895 | 0.460571 | -0.90713 | 2.049666 |
| RPS3 | 0.724895 | 0.460641 | 0.812009 | 1.599128 |
| PABPC4-AS | 0.724895 | 0.460708 | 0.484829 | 2.321335 |
| GDPD4 | 0.724895 | 0.460714 | -1.20716 | 0.789594 |
| CTBP1 | 0.724895 | 0.460724 | -0.47875 | 2.773075 |
| RPS6KA3 | 0.7249 | 0.460783 | 0.278238 | 7.233346 |
| XPO7 | 0.725066 | 0.460943 | 0.272707 | 6.941643 |
| CAMK2D | 0.725336 | 0.461231 | 0.68192 | 1.642562 |
| RRAGB | 0.725336 | 0.461283 | -0.90684 | 0.831411 |
| TTTY10 | 0.725336 | 0.461334 | 1.427413 | 0.500566 |
| GRPR | 0.725336 | 0.461336 | 1.272297 | 0.994241 |
| HIST1H3I | 0.725363 | 0.461409 | -0.48433 | 4.764239 |
| SNORD37 | 0.725448 | 0.461518 | 0.510306 | 2.892844 |
| LRRC14 | 0.725455 | 0.461578 | 1.031822 | 1.057836 |
| IFI16 | 0.725468 | 0.461641 | 0.848301 | 1.540443 |
| GTF3C3 | 0.725873 | 0.461954 | 0.339843 | 4.474645 |
| SLF2 | 0.725891 | 0.462021 | 0.344362 | 5.061665 |
| SETD9 | 0.725903 | 0.462106 | -1.04481 | 0.953815 |
| SCARNA2 | 0.725903 | 0.462139 | 0.300114 | 7.66401 |
| LOC220729 | 0.726105 | 0.462331 | -0.30332 | 5.787345 |
| IKZF4 | 0.726105 | 0.462426 | -0.4347 | 4.402252 |
| LOC642846 | 0.726105 | 0.462494 | 0.473793 | 3.874998 |
| LOC100287 | 0.726105 | 0.462508 | -0.79446 | 1.21575 |
| THRB-AS1 | 0.726105 | 0.462545 | -0.79775 | 1.460047 |
| BTAF1 | 0.726799 | 0.463089 | -0.26603 | 8.073956 |
| CNOT10 | 0.726799 | 0.463097 | 0.74785 | 1.010495 |
| BRWD1 | 0.726818 | 0.463165 | -0.27537 | 7.122949 |
| NDEL1 | 0.726893 | 0.463268 | -0.58385 | 2.432736 |
| DCAF7 | 0.727025 | 0.463447 | 0.254143 | 6.583681 |
| NDUFB10 | 0.727025 | 0.463463 | 0.27388 | 5.868646 |
| HLA-E | 0.727085 | 0.463563 | 0.429233 | 3.622587 |
| CDK6 | 0.727085 | 0.463624 | 0.903028 | 1.816273 |
| SBNO2 | 0.727085 | 0.463668 | 0.670038 | 1.749831 |
| GAS8-AS1 | 0.727165 | 0.463774 | -0.64251 | 2.162189 |

| | | | | |
|---|---|---|---|---|
| PPP1R18 | 0.72717 | 0.463833 | 0.573527 | 3.182004 |
| CEBPG | 0.727276 | 0.463956 | 0.625578 | 2.12374 |
| HMGN5 | 0.727418 | 0.46417 | -0.33783 | 5.786678 |
| USP45 | 0.727418 | 0.464191 | 0.353247 | 5.394953 |
| SNORA26 | 0.727418 | 0.464212 | 0.376352 | 8.268766 |
| LOC101927 | 0.727715 | 0.464458 | 0.883406 | 1.397823 |
| ETFRF1 | 0.727878 | 0.464634 | -0.35984 | 3.910846 |
| DNAJB7 | 0.727878 | 0.46472 | 0.97531 | 0.778486 |
| CROT | 0.727878 | 0.464728 | -0.52642 | 2.196164 |
| COX7A2L | 0.727962 | 0.464872 | 0.272195 | 5.435719 |
| CA5B | 0.727962 | 0.464925 | 0.370885 | 4.27923 |
| TXNDC9 | 0.727962 | 0.464948 | -0.31336 | 5.01682 |
| ACTR8 | 0.728099 | 0.465091 | -0.31692 | 5.081217 |
| MRPL38 | 0.728173 | 0.465194 | -0.28669 | 4.98246 |
| GTDC1 | 0.728195 | 0.465264 | 0.702007 | 1.810551 |
| INE1 | 0.728411 | 0.465505 | -0.36703 | 4.587318 |
| TSACC | 0.728411 | 0.465513 | 0.83713 | 1.023446 |
| B4GALNT3 | 0.728694 | 0.465849 | -0.40381 | 7.050451 |
| ZNF616 | 0.728694 | 0.465862 | 0.384381 | 4.075462 |
| PANDAR | 0.728694 | 0.465905 | 0.817375 | 1.200632 |
| RRN3P3 | 0.728694 | 0.465933 | 0.484872 | 4.286077 |
| SMNDC1 | 0.728694 | 0.465978 | -0.33591 | 5.393294 |
| LOC286297 | 0.728694 | 0.466027 | -0.78494 | 3.034956 |
| LIPH | 0.729073 | 0.466324 | 0.294576 | 6.246752 |
| NEDD9 | 0.729128 | 0.466422 | 0.596325 | 3.088987 |
| FRG1 | 0.729128 | 0.466471 | 0.930649 | 1.781855 |
| NOL6 | 0.729132 | 0.466529 | 0.428801 | 3.99065 |
| ALKBH3-AS | 0.729171 | 0.46661 | 0.788244 | 1.956458 |
| ZNF622 | 0.729623 | 0.466954 | 0.293334 | 5.290279 |
| NRIP3 | 0.729821 | 0.467188 | 0.579226 | 2.389035 |
| SERPINB6 | 0.729821 | 0.467192 | -0.38724 | 4.928265 |
| THUMPD3- | 0.729833 | 0.467255 | -1.08379 | 0.913929 |
| USP7 | 0.730066 | 0.467473 | 0.412084 | 3.672702 |
| AKAP7 | 0.730066 | 0.467516 | 0.620227 | 2.897978 |
| INO80D | 0.730797 | 0.468112 | -0.26823 | 8.283749 |
| LOC100129 | 0.730797 | 0.468123 | -1.26494 | 1.294027 |
| FAM161B | 0.730797 | 0.468159 | -0.4104 | 3.449283 |
| BACH1 | 0.730797 | 0.468224 | -0.46059 | 3.24256 |
| SNORA81 | 0.730797 | 0.468272 | 0.585732 | 3.008035 |
| PMS2P1 | 0.730797 | 0.468319 | 0.413795 | 3.946505 |
| LOC100507 | 0.730914 | 0.468516 | 0.437565 | 3.624818 |
| C4orf33 | 0.730914 | 0.468554 | -0.92358 | 0.846525 |
| BRAF | 0.730914 | 0.468575 | -0.2848 | 6.143486 |
| KCNK9 | 0.730914 | 0.468616 | 1.386335 | 0.590619 |
| FERMT1 | 0.731064 | 0.468768 | 0.321585 | 6.939265 |

| | | | | |
|---|---|---|---|---|
| EFHD2 | 0.731146 | 0.468907 | 0.344474 | 7.38648 |
| TRPA1 | 0.731146 | 0.468932 | 0.631112 | 4.191407 |
| SNORD80 | 0.731303 | 0.469105 | 0.397543 | 4.191726 |
| DNMBP-AS | 0.731303 | 0.469144 | -0.86363 | 1.302407 |
| RNFT1 | 0.731371 | 0.469282 | -0.37432 | 3.893414 |
| LOC10019 | 0.731371 | 0.469299 | 0.983729 | 0.554881 |
| LOC10012 | 0.731622 | 0.469548 | 0.841889 | 1.93374 |
| LOC10192 | 0.731622 | 0.469595 | 0.900204 | 1.397627 |
| LINC01590 | 0.731622 | 0.469628 | -0.72804 | 0.922921 |
| NMT2 | 0.731626 | 0.469689 | 0.467003 | 2.611231 |
| ZNF830 | 0.731626 | 0.469742 | 0.314391 | 4.83966 |
| CAPN15 | 0.73195 | 0.470074 | 0.278954 | 6.341092 |
| EFNB1 | 0.73195 | 0.470087 | 0.40006 | 6.09138 |
| SDHAF3 | 0.73195 | 0.470117 | 0.552777 | 3.482922 |
| FIP1L1 | 0.732175 | 0.470317 | -1.15004 | 0.578898 |
| DCUN1D3 | 0.73231 | 0.47046 | 0.370726 | 5.354266 |
| LRRIQ1 | 0.732404 | 0.470601 | 0.891685 | 1.435806 |
| DSCAML1 | 0.732404 | 0.470645 | 0.750811 | 2.347922 |
| SNORD36A | 0.732404 | 0.470702 | 0.411264 | 6.059852 |
| LINC00954 | 0.732404 | 0.470743 | 0.613597 | 2.041136 |
| CLPP | 0.732438 | 0.470821 | -0.35656 | 3.945588 |
| KSR2 | 0.732539 | 0.470942 | -0.77855 | 1.937345 |
| KRI1 | 0.732593 | 0.471108 | 0.291414 | 6.411331 |
| UBE2Q1 | 0.732593 | 0.471141 | 0.249945 | 6.012166 |
| MCM7 | 0.732593 | 0.471144 | 0.540933 | 2.365191 |
| ADAMTS1 | 0.732625 | 0.471347 | 0.956217 | 0.579056 |
| KCNC3 | 0.732625 | 0.471401 | 0.445357 | 4.125616 |
| PIAS2 | 0.732625 | 0.471413 | -0.31267 | 5.174216 |
| HSD17B7P | 0.732625 | 0.47143 | 0.461922 | 2.999647 |
| RADIL | 0.732625 | 0.471444 | 1.077601 | 0.71581 |
| ESYT2 | 0.732779 | 0.471655 | 0.27214 | 8.177146 |
| ZBTB32 | 0.732779 | 0.471662 | 0.686612 | 2.144397 |
| EIF4H | 0.732779 | 0.471711 | 1.066161 | 0.801907 |
| LINC01091 | 0.732921 | 0.471869 | 0.942149 | 0.964757 |
| MIR95 | 0.732921 | 0.471913 | -1.28175 | 0.835868 |
| TSHZ1 | 0.732981 | 0.472069 | -0.52175 | 2.194295 |
| PRORSD1P | 0.732981 | 0.472119 | 0.724856 | 2.183231 |
| EPC1 | 0.732981 | 0.47212 | -0.66226 | 1.963301 |
| TACC1 | 0.733028 | 0.472209 | 0.717696 | 2.170237 |
| RND2 | 0.733028 | 0.472262 | 0.850754 | 0.936457 |
| ZNF346 | 0.733033 | 0.472321 | 0.98874 | 0.650914 |
| PEX12 | 0.733144 | 0.472448 | 0.587111 | 2.89367 |
| INSRR | 0.733166 | 0.472549 | -1.18264 | 0.888106 |
| LINC00565 | 0.733166 | 0.472607 | 1.263656 | 0.340552 |
| PCAT19 | 0.733166 | 0.47263 | 0.673412 | 1.670163 |

| | | | | |
|---|---|---|---|---|
| CCNK | 0.733199 | 0.472707 | -0.28345 | 6.098857 |
| WDR97 | 0.733635 | 0.473081 | 0.803871 | 1.415819 |
| RAP1A | 0.733635 | 0.4731 | 0.673326 | 1.172216 |
| NDUFA4 | 0.733741 | 0.473225 | -0.28619 | 5.80904 |
| CDNF | 0.733837 | 0.47339 | -0.8103 | 1.67435 |
| FAM21EP | 0.733837 | 0.473398 | 0.692584 | 1.693577 |
| BLOC1S5 | 0.734034 | 0.473581 | -0.92294 | 0.572783 |
| TRMT61B | 0.734095 | 0.473682 | 0.330447 | 4.09853 |
| MAPKAPK2 | 0.734095 | 0.473732 | 0.689226 | 2.033465 |
| LRRC69 | 0.734226 | 0.473873 | 0.669508 | 1.693597 |
| C19orf57 | 0.734273 | 0.473998 | -0.60037 | 2.296842 |
| CDRT4 | 0.734273 | 0.474015 | -0.54527 | 2.51396 |
| TMEM267 | 0.734599 | 0.474334 | 0.5466 | 2.997932 |
| XRN1 | 0.734599 | 0.4744 | -0.4086 | 3.633961 |
| LINC00910 | 0.734599 | 0.474409 | -0.55053 | 2.514069 |
| CHAC2 | 0.734599 | 0.47445 | -0.45138 | 3.363578 |
| KLF16 | 0.734628 | 0.474573 | 0.289643 | 4.865449 |
| FNDC8 | 0.734628 | 0.47458 | -0.68653 | 1.459998 |
| FOXP4-AS1 | 0.73491 | 0.474944 | 0.78769 | 1.719037 |
| SDE2 | 0.73491 | 0.474955 | 0.272693 | 5.960184 |
| IDH1 | 0.73491 | 0.475019 | 0.452496 | 2.997631 |
| RABGEF1 | 0.73491 | 0.475027 | -0.33774 | 3.809169 |
| TERF2IP | 0.73491 | 0.475042 | 0.253886 | 6.430103 |
| WDR64 | 0.735001 | 0.475157 | -1.28662 | 1.031062 |
| TWF1 | 0.735118 | 0.475289 | -0.8936 | 0.672326 |
| MYO15A | 0.735192 | 0.475393 | 0.707231 | 2.951909 |
| MCF2L2 | 0.735379 | 0.47557 | -0.68442 | 2.625041 |
| LOC101927 | 0.735419 | 0.475658 | -0.73497 | 1.433309 |
| GPT | 0.735419 | 0.475708 | -0.46819 | 3.114177 |
| SETD7 | 0.735748 | 0.476003 | 0.307803 | 7.191569 |
| RIMKLB | 0.735748 | 0.476033 | -0.41941 | 3.868985 |
| TMEM67 | 0.735834 | 0.476144 | -0.93883 | 0.986925 |
| SRSF8 | 0.735848 | 0.476209 | 0.51768 | 2.657856 |
| CCL19 | 0.736181 | 0.476481 | 1.061161 | 1.158556 |
| CEP63 | 0.736327 | 0.476632 | 0.801239 | 1.41686 |
| SOCS3 | 0.736458 | 0.476773 | 0.555298 | 6.844039 |
| MIR3162 | 0.736688 | 0.476978 | -1.36077 | 0.542432 |
| MTOR-AS1 | 0.736709 | 0.477119 | 0.720839 | 1.428419 |
| LOC286059 | 0.736709 | 0.477188 | 0.886734 | 1.11975 |
| MEX3B | 0.736709 | 0.477194 | 0.710732 | 1.770385 |
| DDX1 | 0.736709 | 0.477216 | -0.28348 | 7.240009 |
| ZNF320 | 0.736893 | 0.477413 | 0.365524 | 5.140645 |
| LINC01116 | 0.736893 | 0.477449 | -0.76805 | 1.387487 |
| NUMA1 | 0.736893 | 0.477543 | -1.03502 | 0.539578 |
| TBC1D32 | 0.736893 | 0.47756 | 0.914185 | 0.881345 |

| | | | | |
|---|---|---|---|---|
| CLDN15 | 0.737301 | 0.477966 | 0.457508 | 2.558111 |
| GBAP1 | 0.737301 | 0.477979 | 0.397408 | 3.957366 |
| CASZ1 | 0.737301 | 0.478113 | -0.28695 | 5.720136 |
| PPP1R2 | 0.737301 | 0.47817 | 1.024348 | 1.087904 |
| SNORD79 | 0.737301 | 0.478188 | 0.514646 | 3.685524 |
| MZT2B | 0.737301 | 0.478225 | -0.51644 | 3.283898 |
| SNORD34 | 0.737301 | 0.478244 | 0.482188 | 3.192158 |
| NCK2 | 0.737301 | 0.478283 | -0.95896 | 0.534548 |
| ATG13 | 0.737301 | 0.47833 | -0.72171 | 1.105969 |
| RPS27A | 0.73738 | 0.478437 | -0.86051 | 1.472296 |
| SLC37A1 | 0.737412 | 0.478514 | 0.326622 | 6.002243 |
| MOB1A | 0.737464 | 0.478756 | 0.901805 | 0.395515 |
| CD81-AS1 | 0.737464 | 0.478864 | 0.802853 | 0.762071 |
| TRIP6 | 0.737464 | 0.478907 | -0.27618 | 6.240549 |
| CUX2 | 0.737464 | 0.478924 | -0.99217 | 0.838644 |
| TPRG1-AS1 | 0.737464 | 0.478998 | 0.987906 | 0.338201 |
| LIMS2 | 0.737464 | 0.479059 | 1.006979 | 0.728517 |
| GRWD1 | 0.737464 | 0.479062 | 0.284572 | 5.357329 |
| LINC01878 | 0.737464 | 0.479154 | -0.80126 | 0.653985 |
| PDSS1 | 0.737464 | 0.479199 | 0.438886 | 4.008733 |
| ARHGAP9 | 0.737464 | 0.479217 | 0.835926 | 0.625162 |
| CAV1 | 0.737464 | 0.479266 | 0.798807 | 0.596444 |
| MCM5 | 0.737464 | 0.479268 | -0.30896 | 5.220451 |
| KRT19 | 0.737464 | 0.479321 | -0.38786 | 9.727611 |
| RNF113B | 0.737464 | 0.47936 | 0.930101 | 1.235381 |
| 9-Mar | 0.737464 | 0.47942 | 0.408432 | 3.334415 |
| TRAM1 | 0.737464 | 0.479447 | 0.719084 | 1.599273 |
| LOC100132 | 0.737477 | 0.479512 | -0.65481 | 2.104019 |
| EPPK1 | 0.73759 | 0.479651 | 0.433009 | 7.808946 |
| ZNF709 | 0.73759 | 0.479698 | 0.401258 | 3.420831 |
| TOP3A | 0.737592 | 0.479755 | 0.298521 | 4.733447 |
| PPP1R16A | 0.73782 | 0.480013 | 0.348222 | 4.367926 |
| LINC00921 | 0.73782 | 0.480016 | 0.482205 | 2.84171 |
| SRM | 0.738051 | 0.480222 | -0.33667 | 4.877203 |
| PRMT6 | 0.73824 | 0.480401 | -0.48914 | 3.422101 |
| LINC01465 | 0.738307 | 0.480501 | 0.561813 | 2.342033 |
| MPHOSPH( | 0.738825 | 0.480895 | -0.30703 | 4.918727 |
| EML4 | 0.73934 | 0.481361 | 0.541332 | 2.415185 |
| SMG1P5 | 0.73934 | 0.481372 | 0.451643 | 2.730912 |
| LCN10 | 0.73934 | 0.481446 | 1.199152 | 2.6665 |
| HNRNPA1L | 0.73934 | 0.481456 | 0.879594 | 0.416887 |
| RRP7BP | 0.73942 | 0.481564 | 0.320582 | 5.129635 |
| ANKRD62 | 0.739859 | 0.481948 | -0.6099 | 2.669723 |
| KCNV2 | 0.739859 | 0.481963 | -0.54355 | 2.468571 |
| ITGA6 | 0.740214 | 0.48225 | 0.847607 | 1.117433 |

| | | | | |
|---|---|---|---|---|
| 7-Mar | 0.740302 | 0.482374 | -0.78405 | 2.076496 |
| SMIM19 | 0.740302 | 0.482421 | -0.52936 | 1.705298 |
| MCM8-AS1 | 0.740381 | 0.482529 | 0.778509 | 1.199335 |
| HIST1H4H | 0.740422 | 0.482612 | -0.47325 | 4.521585 |
| RARA-AS1 | 0.740775 | 0.482898 | -0.69633 | 2.238094 |
| DHX30 | 0.741017 | 0.483112 | -0.91295 | 0.98602 |
| ANKRD33B | 0.741734 | 0.483645 | -0.44424 | 4.025723 |
| TTF1 | 0.741734 | 0.483693 | 0.415123 | 3.616192 |
| PTS | 0.741842 | 0.48384 | -0.334 | 4.249464 |
| PAN3-AS1 | 0.741842 | 0.483901 | 0.410958 | 3.30393 |
| NANP | 0.741842 | 0.483933 | 0.392021 | 3.694157 |
| LOC28378ξ | 0.742052 | 0.484126 | 0.372878 | 5.732439 |
| LINC00943 | 0.742202 | 0.484281 | 1.112378 | 0.808446 |
| PIGO | 0.742292 | 0.484396 | 0.46582 | 2.347569 |
| KIAA0100 | 0.742402 | 0.484524 | 0.250057 | 8.110568 |
| ATL2 | 0.742411 | 0.484587 | -0.65038 | 2.531559 |
| FAM131A | 0.742418 | 0.484697 | 0.585045 | 2.130437 |
| RUVBL2 | 0.742418 | 0.484704 | -0.25713 | 5.664063 |
| DIS3L2 | 0.742457 | 0.484855 | 0.466228 | 2.835607 |
| GAK | 0.742457 | 0.484898 | -0.51096 | 3.345463 |
| FBF1 | 0.742457 | 0.484909 | -0.37903 | 4.399844 |
| AKR7A2P1 | 0.742457 | 0.484956 | -0.7879 | 1.855523 |
| MOSPD2 | 0.742506 | 0.485059 | -0.57693 | 1.778469 |
| PICART1 | 0.742506 | 0.485101 | 0.883014 | 0.774077 |
| FAM208B | 0.742599 | 0.485219 | 0.253531 | 7.645191 |
| SERAC1 | 0.7426 | 0.485276 | -0.40901 | 3.861971 |
| C8G | 0.742786 | 0.485508 | 0.8981 | 1.98904 |
| HP09053 | 0.742786 | 0.485542 | -0.6954 | 1.526365 |
| TOPBP1 | 0.742786 | 0.485568 | 0.263463 | 6.640037 |
| APTR | 0.742808 | 0.485639 | 0.402451 | 3.335495 |
| LINC02614 | 0.742902 | 0.485756 | 0.917346 | 0.647529 |
| ZNF880 | 0.74293 | 0.485832 | 0.377949 | 3.682735 |
| PHC2 | 0.743323 | 0.486145 | 0.659754 | 2.034255 |
| KPNA1 | 0.743646 | 0.486428 | -0.51472 | 2.269217 |
| MYSM1 | 0.743646 | 0.48647 | -0.25674 | 7.619401 |
| TSPYL4 | 0.743698 | 0.486587 | -0.32696 | 5.553053 |
| POM121C | 0.743698 | 0.486618 | 0.261751 | 6.702081 |
| GPRC5C | 0.744307 | 0.487118 | -0.95508 | 1.108272 |
| BAP1 | 0.744307 | 0.487129 | -0.24878 | 6.709376 |
| DRG2 | 0.744726 | 0.48746 | -0.29102 | 4.638149 |
| IER2 | 0.74494 | 0.487657 | 0.311739 | 6.908933 |
| ING3 | 0.74522 | 0.487897 | -0.35783 | 3.40685 |
| TFDP1 | 0.745359 | 0.488064 | -0.93859 | 0.603419 |
| KCNS2 | 0.745359 | 0.488101 | 0.817051 | 1.333319 |
| CCDC18-AS | 0.745516 | 0.488261 | 0.317272 | 5.12381 |

| | | | | |
|---|---|---|---|---|
| NCBP1 | 0.745805 | 0.48858 | 0.246079 | 6.682482 |
| SLX4 | 0.745805 | 0.488644 | 0.303147 | 5.193739 |
| FAU | 0.745805 | 0.48865 | -0.26379 | 7.480124 |
| GTF2A1 | 0.745805 | 0.488678 | -0.99622 | 0.579692 |
| POLI | 0.746072 | 0.488988 | 0.309378 | 4.793403 |
| ETFDH | 0.746072 | 0.489032 | -0.72787 | 1.311264 |
| RRP7A | 0.746072 | 0.489085 | -0.28158 | 5.226506 |
| CYP2C9 | 0.746072 | 0.489099 | -0.51423 | 4.373133 |
| FAM133DP | 0.746072 | 0.489137 | -0.43719 | 3.363914 |
| GATAD2A | 0.746105 | 0.489216 | 1.239957 | 0.651938 |
| SERF2 | 0.746172 | 0.489397 | 0.680308 | 1.946477 |
| DNAJB13 | 0.746172 | 0.489408 | 0.976392 | 0.434308 |
| DCDC2B | 0.746172 | 0.48943 | 0.501521 | 2.98336 |
| TOB2 | 0.746437 | 0.489703 | 0.248214 | 6.981747 |
| TRIM13 | 0.746437 | 0.489718 | -0.63011 | 1.364413 |
| SCMH1 | 0.746836 | 0.490037 | 0.62357 | 2.462634 |
| ZRSR2 | 0.747095 | 0.490263 | 0.283906 | 5.940313 |
| ANXA2R | 0.747104 | 0.490326 | -0.66023 | 1.581825 |
| ASH1L | 0.747152 | 0.490458 | 0.24926 | 9.155272 |
| APLNR | 0.747152 | 0.490492 | 0.657316 | 2.442782 |
| MIR181A2I | 0.747152 | 0.490593 | 0.523747 | 3.115707 |
| ZNF740 | 0.747152 | 0.490625 | 0.256325 | 6.027182 |
| ABHD16B | 0.747152 | 0.490642 | -0.40874 | 2.71435 |
| LACTB | 0.747212 | 0.490739 | 0.651432 | 2.597199 |
| ZDHHC8 | 0.747245 | 0.490817 | -0.36641 | 3.424782 |
| UGDH-AS1 | 0.747348 | 0.490942 | -0.52405 | 2.769639 |
| CCZ1P-OR7 | 0.747568 | 0.491144 | 0.366445 | 4.494533 |
| SEC61B | 0.747613 | 0.491255 | -0.31512 | 4.828613 |
| GAMT | 0.747613 | 0.491287 | -0.81067 | 1.362176 |
| USH2A | 0.747631 | 0.4914 | -0.67055 | 2.694114 |
| ELL3 | 0.747631 | 0.491412 | -0.46766 | 4.02704 |
| KLB | 0.747702 | 0.491535 | -0.65784 | 2.195874 |
| SRSF4 | 0.747702 | 0.491573 | -0.26259 | 7.720747 |
| SLC22A14 | 0.747838 | 0.49172 | -1.01579 | 0.842815 |
| CACTIN | 0.747843 | 0.49178 | 0.631429 | 1.15936 |
| MRPS30 | 0.748115 | 0.492016 | 0.304011 | 4.893098 |
| CHAF1A | 0.748122 | 0.49212 | 0.250503 | 5.990683 |
| BORCS7 | 0.748122 | 0.492158 | -0.49713 | 2.520112 |
| THY1 | 0.748122 | 0.492192 | 0.960033 | 0.968393 |
| RWDD2B | 0.748203 | 0.492303 | -0.35248 | 3.905563 |
| 2-Sep | 0.748203 | 0.492359 | -0.48245 | 2.784631 |
| CNOT7 | 0.748407 | 0.49255 | 0.27794 | 5.566456 |
| OR52B6 | 0.748494 | 0.492736 | 0.757259 | 1.1032 |
| PCSK5 | 0.748494 | 0.492796 | -0.45616 | 4.097416 |
| SCARA5 | 0.748494 | 0.492917 | 0.852155 | 2.491165 |

| | | | | |
|---|---|---|---|---|
| CDC37 | 0.748494 | 0.492921 | 0.25643 | 7.559003 |
| LEMD2 | 0.748494 | 0.492949 | 0.378194 | 3.46996 |
| LOC728752 | 0.748494 | 0.49295 | 0.857832 | 1.038243 |
| SNORD6 | 0.748506 | 0.493053 | 0.769331 | 1.505108 |
| SLC18B1 | 0.748506 | 0.493072 | 0.332549 | 5.159385 |
| PHKA2-AS1 | 0.748571 | 0.493171 | 0.881742 | 1.108985 |
| SDHAF1 | 0.748692 | 0.493308 | 0.422059 | 2.841746 |
| LOC105378 | 0.748765 | 0.493487 | -0.75701 | 1.545484 |
| CYREN | 0.748765 | 0.493524 | -1.00226 | 1.276038 |
| RPL21P28 | 0.748765 | 0.493541 | -0.26891 | 5.753292 |
| LOC101927 | 0.748765 | 0.493598 | -1.20518 | 1.582879 |
| SNORA71B | 0.748765 | 0.493641 | 0.366542 | 4.993964 |
| RNY1 | 0.749084 | 0.493965 | 0.350735 | 9.131702 |
| DBI | 0.749084 | 0.493976 | 0.882391 | 0.595852 |
| POLR2D | 0.749084 | 0.494083 | 0.314372 | 4.939061 |
| PARPBP | 0.749084 | 0.494132 | 0.4234 | 3.462755 |
| SMCO4 | 0.749084 | 0.494137 | 0.507148 | 3.010101 |
| ACTB | 0.749569 | 0.494531 | -0.2909 | 12.26658 |
| CORO2A | 0.749569 | 0.494592 | -1.00319 | 0.967279 |
| ERFE | 0.749569 | 0.494629 | 0.743033 | 1.945519 |
| RGMB | 0.749651 | 0.494777 | 0.391454 | 6.0451 |
| RNF20 | 0.749651 | 0.494797 | -0.25945 | 7.168332 |
| GPM6B | 0.750102 | 0.495152 | -0.37886 | 3.601421 |
| NYAP1 | 0.750154 | 0.495244 | -0.84411 | 1.015669 |
| LINC02381 | 0.750335 | 0.495459 | -0.48971 | 3.437163 |
| FUT1 | 0.750335 | 0.495533 | -0.45468 | 3.671541 |
| TRIM9 | 0.750335 | 0.495534 | 0.80561 | 1.908567 |
| CLPX | 0.750386 | 0.495679 | -0.852 | 0.572703 |
| ZNF692 | 0.750386 | 0.495682 | 0.910622 | 0.87955 |
| RRS1 | 0.750604 | 0.495884 | 0.281195 | 4.94263 |
| GPR155 | 0.750604 | 0.496017 | 0.694911 | 1.47181 |
| NR1D1 | 0.750604 | 0.496043 | -0.4232 | 4.535073 |
| RPL13AP20 | 0.750604 | 0.496062 | -0.48578 | 2.842833 |
| PITPNA-AS | 0.750604 | 0.496113 | -0.64033 | 2.240587 |
| ALPK2 | 0.750609 | 0.496173 | 0.747039 | 3.114319 |
| ACOT8 | 0.750675 | 0.496359 | -0.34571 | 3.831093 |
| MKLN1-AS | 0.750675 | 0.496402 | 0.573069 | 2.597317 |
| ARGFXP2 | 0.750675 | 0.496451 | 0.441657 | 2.901229 |
| TNFRSF13E | 0.750675 | 0.496462 | -1.15404 | 0.997459 |
| STPG2 | 0.750675 | 0.496503 | -1.10285 | 0.855001 |
| CNOT8 | 0.750882 | 0.496697 | 0.931565 | 0.857257 |
| GINS4 | 0.75107 | 0.496878 | 0.416298 | 3.24024 |
| AK1 | 0.751109 | 0.496962 | 0.306296 | 4.821222 |
| USP32P1 | 0.751112 | 0.497021 | 0.557853 | 4.809793 |
| HSPA9 | 0.751498 | 0.497333 | -0.25694 | 8.392969 |

| | | | | |
|---|---|---|---|---|
| NUCB1-AS1 | 0.751542 | 0.49742 | -0.79237 | 0.687698 |
| GRIN3A | 0.75158 | 0.497573 | 0.624484 | 1.554724 |
| PRICKLE2-A | 0.75158 | 0.497597 | 0.965687 | 0.610938 |
| SNRPF | 0.75158 | 0.497617 | -0.30071 | 5.954288 |
| CXADR | 0.751599 | 0.497702 | 0.752966 | 1.943957 |
| AGPAT3 | 0.751599 | 0.497795 | -0.61039 | 1.395243 |
| RPS16 | 0.751599 | 0.497801 | -0.25876 | 7.734969 |
| NT5DC2 | 0.751625 | 0.497917 | -0.70565 | 0.999571 |
| PHC1 | 0.751625 | 0.497934 | -0.34514 | 5.428238 |
| SNORD3A | 0.751625 | 0.49799 | 0.319079 | 10.44014 |
| SRGAP2-AS | 0.751662 | 0.498072 | 0.764728 | 2.404064 |
| FAM95C | 0.751752 | 0.498189 | -0.48959 | 3.531129 |
| PMS2P9 | 0.751867 | 0.498356 | -0.26746 | 4.977578 |
| KLHDC7A | 0.751867 | 0.49838 | -0.60339 | 2.48622 |
| TUBE1 | 0.751923 | 0.498514 | 0.462103 | 4.313176 |
| LOC100506 | 0.751923 | 0.498569 | -0.83037 | 1.206179 |
| SIRT5 | 0.751923 | 0.498589 | 0.687718 | 2.116335 |
| LHFPL4 | 0.752142 | 0.498792 | -0.70845 | 1.610723 |
| CHRM3-AS | 0.752374 | 0.499046 | 0.878851 | 2.036376 |
| JMJD6 | 0.752374 | 0.49906 | 0.542815 | 3.257267 |
| STAT6 | 0.75238 | 0.499165 | 0.57186 | 2.902378 |
| GPR182 | 0.75238 | 0.499178 | -0.81831 | 0.846307 |
| CCNT2-AS1 | 0.752576 | 0.499366 | -0.71253 | 1.531827 |
| FCGR1A | 0.752646 | 0.499474 | 0.884742 | 0.725123 |
| FAM200A | 0.752646 | 0.499546 | 0.397034 | 4.285163 |
| IPO5 | 0.752646 | 0.499585 | 0.274692 | 7.694412 |
| CHN1 | 0.752716 | 0.499688 | 1.075384 | 0.949327 |
| TRMT1L | 0.752768 | 0.499818 | 0.587639 | 1.509534 |
| SELENOT | 0.752768 | 0.499869 | -0.23838 | 5.859032 |
| RBM42 | 0.752768 | 0.499978 | -0.24336 | 5.566877 |
| CARD10 | 0.752768 | 0.500037 | 0.355319 | 5.599457 |
| GMPPA | 0.752768 | 0.500041 | 0.652921 | 1.41701 |
| GPS1 | 0.752768 | 0.500114 | 0.59279 | 1.777797 |
| ZNF582 | 0.752768 | 0.500125 | -0.80682 | 1.397083 |
| PCAT2 | 0.752795 | 0.5002 | -1.19528 | 0.534078 |
| LMNTD2 | 0.752974 | 0.500376 | 0.462539 | 2.701572 |
| MED30 | 0.752984 | 0.50044 | 0.789081 | 0.757458 |
| DDIT4L | 0.753127 | 0.500602 | 1.014375 | 0.522705 |
| SLC39A6 | 0.753127 | 0.500671 | 0.34825 | 5.564579 |
| TMPO-AS1 | 0.753127 | 0.500707 | 0.724319 | 0.861154 |
| PLCG2 | 0.753158 | 0.500786 | 0.287324 | 5.517055 |
| NLN | 0.753232 | 0.50092 | 0.2731 | 5.565747 |
| SCN2B | 0.753232 | 0.500949 | 0.773214 | 1.379914 |
| DLG5-AS1 | 0.753277 | 0.501037 | -0.61317 | 1.371145 |
| MAP3K5 | 0.753359 | 0.501149 | -0.25816 | 6.671356 |

| | | | | |
|---|---|---|---|---|
| LOC100287 | 0.753524 | 0.501413 | 0.648458 | 1.681624 |
| PIGB | 0.753524 | 0.501475 | 0.360963 | 3.603297 |
| LRRC37A11 | 0.753524 | 0.501482 | -1.48557 | 0.981192 |
| LRRC59 | 0.753524 | 0.501488 | 0.275894 | 7.398884 |
| LOC101928 | 0.753641 | 0.501623 | 0.70817 | 1.410431 |
| ZNF536 | 0.753646 | 0.501733 | 0.811454 | 2.100693 |
| GTF2E2 | 0.753646 | 0.501742 | -0.26235 | 6.051471 |
| ABHD11-AS | 0.75385 | 0.501935 | 0.925287 | 2.011916 |
| ANKRD18A | 0.753909 | 0.502031 | 0.289885 | 5.550033 |
| MAP3K14 | 0.754086 | 0.502251 | 0.265229 | 5.456627 |
| ZNF438 | 0.754086 | 0.502264 | -0.63527 | 2.062687 |
| INTS6L | 0.754246 | 0.502428 | -0.31071 | 4.643398 |
| LINC00907 | 0.754338 | 0.502553 | 1.25944 | 0.481932 |
| DOCK10 | 0.754338 | 0.502605 | -0.67576 | 1.564846 |
| MRPL32 | 0.754723 | 0.503031 | 0.289428 | 4.890891 |
| AHCY | 0.754723 | 0.50307 | -0.72549 | 0.642123 |
| RSL24D1 | 0.754723 | 0.50312 | -0.31 | 5.855011 |
| NAA16 | 0.754723 | 0.503134 | 0.273271 | 5.117946 |
| ITGAD | 0.754723 | 0.503169 | 0.82771 | 1.727965 |
| TMEM17 | 0.754723 | 0.503206 | -0.75117 | 2.054284 |
| B3GNT4 | 0.754736 | 0.503272 | -0.61145 | 1.30382 |
| SDCBP2-AS | 0.754906 | 0.503443 | -0.57855 | 2.470367 |
| EP300 | 0.755045 | 0.503593 | -0.24206 | 8.87674 |
| MIR324 | 0.755333 | 0.503843 | 0.60869 | 1.554093 |
| B3GNT6 | 0.755433 | 0.503968 | -0.78215 | 2.205399 |
| MRPL4 | 0.755473 | 0.504052 | -0.66884 | 1.298896 |
| LOC101927 | 0.755493 | 0.504122 | -0.37499 | 3.634629 |
| 9-Sep | 0.75555 | 0.504218 | 0.296235 | 5.873976 |
| GNL2 | 0.756067 | 0.504621 | 0.261457 | 6.97317 |
| STRAP | 0.756249 | 0.504806 | -0.29553 | 6.646073 |
| GEN1 | 0.756249 | 0.504878 | -0.44445 | 2.690817 |
| MPHOSPH9 | 0.756249 | 0.504915 | -0.7678 | 1.281678 |
| STRC | 0.756569 | 0.505187 | -0.98803 | 0.468459 |
| FAM13B | 0.757 | 0.505532 | 0.699611 | 1.629832 |
| ANKDD1B | 0.757052 | 0.505624 | 0.684514 | 1.372003 |
| THAP2 | 0.7573 | 0.505929 | 0.488712 | 3.021591 |
| CSRP1 | 0.7573 | 0.505961 | 1.060349 | 0.656277 |
| LINC01239 | 0.7573 | 0.506041 | 1.01043 | 0.939607 |
| SNX16 | 0.7573 | 0.506121 | -0.97438 | 0.867264 |
| KATNB1 | 0.7573 | 0.506158 | -0.30834 | 4.454702 |
| ZNF500 | 0.7573 | 0.506204 | 0.808925 | 0.740036 |
| TXK | 0.7573 | 0.506263 | -0.5832 | 2.866605 |
| CDK16 | 0.7573 | 0.506293 | -0.51729 | 2.614326 |
| RASSF1 | 0.7573 | 0.506309 | 0.632617 | 2.232966 |
| NUP37 | 0.757374 | 0.506449 | -0.3552 | 3.805221 |

| | | | | |
|---|---|---|---|---|
| DDX51 | 0.757374 | 0.506474 | 0.293059 | 5.022374 |
| CEP19 | 0.757582 | 0.506691 | 0.513964 | 1.667768 |
| LRRC37B | 0.757582 | 0.506742 | 0.325607 | 4.37025 |
| RAB35 | 0.757582 | 0.506787 | -0.5997 | 1.556275 |
| C3orf62 | 0.757748 | 0.506955 | 0.345271 | 4.101004 |
| SNORA70 | 0.758645 | 0.507613 | -0.28726 | 7.862152 |
| PPM1A | 0.758835 | 0.507798 | -0.34601 | 4.396865 |
| XKR6 | 0.759147 | 0.508103 | 0.39146 | 2.668904 |
| ZNF815P | 0.759147 | 0.508169 | 0.605237 | 2.434503 |
| STYX | 0.759147 | 0.508181 | -0.79525 | 0.89624 |
| XK | 0.759357 | 0.508431 | 0.340366 | 4.258649 |
| GPR135 | 0.759357 | 0.508437 | 0.601007 | 2.333734 |
| KANK3 | 0.759372 | 0.508505 | -0.40662 | 2.55791 |
| MIR224 | 0.7598 | 0.508849 | -1.0243 | 1.009166 |
| NDST2 | 0.760093 | 0.509104 | -0.25525 | 5.44035 |
| GADD45B | 0.760107 | 0.509195 | -0.34536 | 5.73733 |
| NR2F2-AS1 | 0.760107 | 0.509267 | 0.753484 | 2.317083 |
| FDFT1 | 0.760107 | 0.509309 | -0.46517 | 3.667441 |
| MRPL12 | 0.760107 | 0.509344 | -0.29822 | 4.480104 |
| LOC72997( | 0.760166 | 0.509442 | 0.881086 | 1.430203 |
| ZC3H14 | 0.760433 | 0.509757 | -0.55244 | 2.006493 |
| SCARNA27 | 0.760433 | 0.509776 | 0.601537 | 2.271574 |
| TBC1D22A | 0.760433 | 0.509795 | 0.39169 | 2.857574 |
| LINC00643 | 0.760435 | 0.509854 | 1.26393 | 0.549918 |
| EPAS1 | 0.760569 | 0.510002 | 0.271072 | 7.835268 |
| PSTK | 0.760669 | 0.510215 | 0.477858 | 2.327408 |
| EEF1E1 | 0.760669 | 0.51022 | 0.663895 | 1.818907 |
| USP4 | 0.760669 | 0.51025 | -0.67115 | 1.56127 |
| SNORD38B | 0.760669 | 0.510361 | 0.40692 | 3.250988 |
| LCORL | 0.760669 | 0.510455 | -0.395 | 3.736639 |
| SNORD50B | 0.760669 | 0.510468 | 0.535507 | 1.575696 |
| KCTD18 | 0.760669 | 0.510475 | -0.27207 | 4.895596 |
| TNKS | 0.760721 | 0.510669 | 0.241916 | 7.182457 |
| FUBP1 | 0.760721 | 0.510679 | 0.5141 | 2.630076 |
| OSBP | 0.760721 | 0.510684 | 0.264117 | 7.582898 |
| ZNF407 | 0.760956 | 0.51098 | 0.33951 | 3.813986 |
| PMS2CL | 0.760956 | 0.511006 | 0.348549 | 4.305811 |
| CLSTN2 | 0.760956 | 0.511015 | -0.49869 | 2.53305 |
| LINC02067 | 0.761152 | 0.511205 | -0.2726 | 8.756798 |
| LYSMD1 | 0.761606 | 0.511568 | -0.51694 | 3.056654 |
| TMEM8A | 0.761773 | 0.511757 | 0.300209 | 5.989563 |
| ENKUR | 0.761773 | 0.511796 | 1.291946 | 0.631631 |
| RNF103 | 0.762062 | 0.512058 | 0.363 | 3.535131 |
| CENPP | 0.762062 | 0.512132 | 0.81355 | 1.059274 |
| FAM193B | 0.762062 | 0.512202 | -0.26682 | 4.948602 |

| | | | | |
|---|---|---|---|---|
| MEIS1 | 0.762062 | 0.512223 | 0.330783 | 6.707154 |
| TMEM62 | 0.762247 | 0.512405 | 0.329431 | 4.844289 |
| RNF207 | 0.76279 | 0.512828 | 0.306338 | 5.338548 |
| WIPF3 | 0.763028 | 0.513047 | -0.61973 | 3.810824 |
| BUD31 | 0.763379 | 0.513341 | 0.256672 | 5.472266 |
| CASC10 | 0.763549 | 0.513564 | -0.85327 | 0.917749 |
| IGF2BP2-AS | 0.763549 | 0.513571 | 0.538197 | 2.99693 |
| RBL1 | 0.763827 | 0.513817 | 0.492432 | 2.62841 |
| SLC25A40 | 0.764159 | 0.514158 | -0.27961 | 4.554436 |
| CEP170 | 0.764159 | 0.514181 | -1.24204 | 0.631538 |
| GOLGA6L10 | 0.764159 | 0.514215 | -0.56885 | 1.959234 |
| WBP1 | 0.7643 | 0.514384 | 0.471417 | 3.06609 |
| ZNF833P | 0.7643 | 0.514426 | 0.683086 | 1.306193 |
| SCTR | 0.764424 | 0.514588 | 0.784217 | 1.916847 |
| TUBA1B | 0.764424 | 0.514626 | 0.25494 | 7.744572 |
| ASCC2 | 0.764592 | 0.514797 | -0.71511 | 0.963802 |
| SPDYE3 | 0.764918 | 0.515128 | 0.392788 | 3.472671 |
| VPS4A | 0.764918 | 0.515186 | -0.24097 | 6.398653 |
| SLC35F1 | 0.764918 | 0.515191 | 0.976604 | 0.891162 |
| SUZ12P1 | 0.765172 | 0.515421 | 0.302442 | 4.505618 |
| PTPN18 | 0.765647 | 0.515799 | 0.550349 | 2.503331 |
| SPATS2 | 0.766173 | 0.516224 | 0.572651 | 2.468537 |
| KC6 | 0.766173 | 0.51627 | -1.02467 | 0.727627 |
| SEC11A | 0.766293 | 0.51641 | 0.8487 | 1.06392 |
| KDM4A-AS | 0.766367 | 0.516518 | 0.708531 | 1.315896 |
| DCAF4L1 | 0.766417 | 0.51661 | 0.870177 | 0.977703 |
| MIR6769A | 0.766439 | 0.516684 | -1.01662 | 0.734659 |
| BAZ2A | 0.766486 | 0.516773 | -0.74029 | 1.155229 |
| TMEM43 | 0.766633 | 0.516931 | -0.25106 | 7.211216 |
| PPIL1 | 0.766775 | 0.517106 | -0.30693 | 4.782185 |
| SCARNA21 | 0.766775 | 0.517144 | 0.267839 | 8.101517 |
| MRPL19 | 0.767271 | 0.517537 | 0.238229 | 6.526325 |
| CCNE2 | 0.767293 | 0.517665 | -0.34401 | 3.958892 |
| ZNF26 | 0.767293 | 0.517716 | -0.84662 | 0.665259 |
| POM121 | 0.767293 | 0.51783 | -0.27504 | 4.957522 |
| LOC730668 | 0.767293 | 0.51785 | 0.926975 | 0.558936 |
| SNW1 | 0.767293 | 0.517924 | -0.26248 | 6.656805 |
| TXNDC15 | 0.767293 | 0.517945 | 0.25444 | 5.708681 |
| RPL21 | 0.767293 | 0.517961 | -0.28274 | 6.423214 |
| GINS1 | 0.767702 | 0.518296 | 0.315807 | 4.306502 |
| PYCR2 | 0.767819 | 0.518433 | 0.868781 | 0.633573 |
| DNAJB9 | 0.767875 | 0.518557 | 0.268816 | 5.206459 |
| GMEB2 | 0.767875 | 0.518757 | -0.28321 | 5.129707 |
| 8-Sep | 0.767875 | 0.518802 | 0.801431 | 1.059936 |
| LOC100335 | 0.767875 | 0.518928 | 0.473891 | 3.158034 |

| | | | | |
|---|---|---|---|---|
| CDAN1 | 0.767875 | 0.518981 | 0.283528 | 4.925567 |
| ITPA | 0.767875 | 0.519029 | 0.985514 | 0.814947 |
| SMTN | 0.767875 | 0.51903 | 0.577434 | 1.569513 |
| ZFP3 | 0.767875 | 0.519038 | 0.35442 | 4.707574 |
| NUFIP1 | 0.767875 | 0.519063 | 0.291013 | 4.164135 |
| RXYLT1 | 0.767875 | 0.519155 | 0.801343 | 1.297096 |
| GLG1 | 0.767875 | 0.51918 | -0.72596 | 1.226029 |
| MIR4454 | 0.767875 | 0.519323 | 0.960571 | 0.353795 |
| RGP1 | 0.767875 | 0.519456 | 0.289042 | 4.260089 |
| FAH | 0.767875 | 0.519579 | -0.42435 | 3.013106 |
| ANGPTL1 | 0.767875 | 0.519593 | -0.28557 | 5.100069 |
| BCAS2 | 0.767875 | 0.519621 | -0.35565 | 5.405383 |
| EYA2 | 0.767875 | 0.519671 | -0.90047 | 0.892869 |
| STX18-AS1 | 0.767875 | 0.519716 | -0.6711 | 2.047756 |
| RPS10P7 | 0.767875 | 0.519771 | 0.521201 | 2.14965 |
| BCKDHB | 0.767875 | 0.519791 | -0.44899 | 3.353945 |
| TOMM6 | 0.767875 | 0.51986 | -0.25266 | 5.254116 |
| RBPMS-AS1 | 0.767875 | 0.519909 | 0.498167 | 2.616529 |
| SEC16A | 0.767875 | 0.51991 | 0.568262 | 2.508383 |
| MOGS | 0.767875 | 0.519931 | 0.908867 | 0.533978 |
| ZNF267 | 0.767875 | 0.519951 | 0.825737 | 0.704246 |
| CYP2R1 | 0.767875 | 0.519992 | -0.34706 | 3.229765 |
| ASAH2B | 0.768215 | 0.520281 | -0.89494 | 1.288335 |
| CD300A | 0.768301 | 0.520398 | 0.823036 | 1.13927 |
| TREX2 | 0.768476 | 0.520658 | 0.701388 | 1.430635 |
| PGGT1B | 0.768476 | 0.52074 | -0.2696 | 5.247716 |
| FAXC | 0.768476 | 0.520749 | -0.53527 | 3.057546 |
| ZNF354A | 0.768476 | 0.520762 | 0.358732 | 3.981772 |
| RELL2 | 0.768476 | 0.520809 | -0.94485 | 0.804036 |
| TMEM18 | 0.768701 | 0.521182 | -0.27089 | 4.499401 |
| RPN1 | 0.768701 | 0.521185 | 0.258558 | 7.831379 |
| AHI1 | 0.768701 | 0.521424 | 0.593785 | 1.911054 |
| ALKBH1 | 0.768701 | 0.521516 | 0.464064 | 3.471423 |
| ATF4 | 0.768701 | 0.52153 | -0.40799 | 3.108595 |
| SDK2 | 0.768701 | 0.521549 | -0.47907 | 4.208427 |
| FCGRT | 0.768701 | 0.52155 | 0.530548 | 2.736513 |
| ANKRD28 | 0.768701 | 0.52161 | -0.50916 | 2.599957 |
| LMBR1L | 0.768701 | 0.521753 | 0.686332 | 1.514204 |
| ASPSCR1 | 0.768701 | 0.521814 | 0.922116 | 0.809424 |
| RTN4RL1 | 0.768701 | 0.521832 | -0.71862 | 1.026896 |
| SLC44A5 | 0.768701 | 0.521908 | 1.001788 | 2.349243 |
| LOC100507 | 0.768701 | 0.521946 | 0.878606 | 0.68623 |
| EFCAB12 | 0.768701 | 0.522026 | 1.083461 | 0.563861 |
| PCMTD1 | 0.768701 | 0.522036 | 0.282094 | 5.077854 |
| BTD | 0.768701 | 0.522068 | 0.811886 | 1.535402 |

| | | | | |
|---|---|---|---|---|
| RBM26 | 0.768701 | 0.522148 | 1.047238 | 0.516438 |
| GTF2B | 0.768701 | 0.522224 | -0.25944 | 4.903997 |
| PRAF2 | 0.768701 | 0.522229 | 0.365349 | 3.728176 |
| NXT1 | 0.768701 | 0.522234 | -0.49 | 3.036649 |
| CENPBD1P | 0.768701 | 0.522268 | 0.338961 | 4.050877 |
| SLC20A2 | 0.768701 | 0.522313 | -0.65105 | 1.953291 |
| RBM11 | 0.768701 | 0.52234 | 0.813161 | 0.761771 |
| MIR4774 | 0.768701 | 0.522368 | -0.94041 | 0.331623 |
| GTF3C5 | 0.769682 | 0.523152 | 0.908223 | 0.448199 |
| MIR23A | 0.769682 | 0.523248 | -0.66896 | 2.017605 |
| PTOV1-AS1 | 0.769682 | 0.523291 | 0.319919 | 4.177791 |
| LOC100128 | 0.769682 | 0.523291 | -0.65334 | 1.544659 |
| CCDC14 | 0.769682 | 0.523331 | 0.606564 | 1.532427 |
| LOC101929 | 0.769682 | 0.523432 | -0.64913 | 2.826172 |
| MDFI | 0.769682 | 0.523462 | 0.603369 | 1.855479 |
| RNLS | 0.769682 | 0.523504 | 0.72098 | 1.139812 |
| ANGEL1 | 0.769876 | 0.523694 | 0.254425 | 4.766929 |
| WDR49 | 0.769935 | 0.523809 | 0.73424 | 2.102142 |
| LINC00514 | 0.769935 | 0.523869 | -0.91583 | 0.647115 |
| TIMMDC1 | 0.769935 | 0.524005 | -0.21876 | 5.855536 |
| SPDL1 | 0.769935 | 0.524027 | 0.282075 | 4.643668 |
| FMO5 | 0.769935 | 0.52406 | 0.958477 | 3.029277 |
| PDCL | 0.769935 | 0.524086 | 0.232791 | 5.345247 |
| LINC02145 | 0.769973 | 0.524171 | 1.039405 | 0.662342 |
| MRC2 | 0.770194 | 0.52438 | 0.250519 | 6.788323 |
| RIN3 | 0.770265 | 0.524535 | 0.325269 | 4.738709 |
| SNORD26 | 0.770265 | 0.524576 | 0.349578 | 8.293858 |
| MT1L | 0.770265 | 0.524605 | 0.857474 | 1.134025 |
| CNPY2 | 0.77076 | 0.525047 | 0.286992 | 4.484278 |
| ABCC6 | 0.77076 | 0.525106 | 0.974733 | 1.040542 |
| LOC646029 | 0.77076 | 0.525142 | 0.878213 | 0.731935 |
| CTBP1-AS | 0.77076 | 0.525177 | 0.406208 | 2.721601 |
| ANXA3 | 0.770788 | 0.525254 | -0.29073 | 6.821811 |
| CTBS | 0.770834 | 0.52538 | -0.35004 | 4.346922 |
| NDUFA9 | 0.770834 | 0.525403 | -0.25573 | 5.490837 |
| DCLRE1C | 0.770921 | 0.525521 | 0.678454 | 1.254323 |
| BTNL9 | 0.770954 | 0.52564 | 0.401116 | 3.512438 |
| BCL2L1 | 0.770954 | 0.525677 | 0.785385 | 0.888629 |
| MRPL41 | 0.770954 | 0.525759 | -0.35719 | 4.49059 |
| DIAPH2 | 0.770954 | 0.525807 | 0.415659 | 2.698883 |
| MIR200C | 0.770954 | 0.525838 | 0.609005 | 0.870156 |
| FIZ1 | 0.771003 | 0.52593 | 0.43401 | 2.899509 |
| CRY1 | 0.771162 | 0.526099 | 0.342147 | 5.194483 |
| GOLGA4 | 0.771162 | 0.526181 | -0.65311 | 1.573772 |
| ZMYND8 | 0.771162 | 0.526289 | -0.39402 | 2.423617 |

| | | | | |
|---|---|---|---|---|
| ZNF493 | 0.771162 | 0.52632 | 0.61558 | 1.559257 |
| AUP1 | 0.771162 | 0.526424 | 0.798073 | 0.402936 |
| TMEM60 | 0.771162 | 0.526448 | 0.386237 | 2.92203 |
| RIMS2 | 0.771162 | 0.526449 | -0.73568 | 1.302272 |
| ANKRD52 | 0.771658 | 0.526896 | 0.250839 | 6.918745 |
| SYNJ2 | 0.771658 | 0.526905 | 0.886047 | 0.625403 |
| KIDINS220 | 0.772193 | 0.527382 | -0.22403 | 8.256464 |
| SLC25A51 | 0.772193 | 0.527389 | -0.31434 | 3.725719 |
| LINC00852 | 0.772462 | 0.527666 | -0.60711 | 2.526475 |
| LOC10272 | 0.772462 | 0.527723 | -0.73688 | 0.56845 |
| ANKRD61 | 0.772462 | 0.527749 | 0.461794 | 2.944574 |
| LOC10050 | 0.772587 | 0.527893 | 0.613073 | 3.718638 |
| FBXL5 | 0.77295 | 0.528261 | -0.7381 | 0.608799 |
| ACACA | 0.77295 | 0.52835 | -0.79123 | 1.234455 |
| CREG1 | 0.77295 | 0.528366 | -0.25774 | 7.213657 |
| BLACAT1 | 0.77295 | 0.528377 | 0.407436 | 4.123672 |
| PPIL3 | 0.773042 | 0.528499 | 0.517836 | 2.443245 |
| FBXO5 | 0.773054 | 0.528566 | 0.650724 | 1.207823 |
| PCBD2 | 0.773284 | 0.528782 | -0.30154 | 4.5863 |
| CCNB2 | 0.773604 | 0.52906 | 0.371321 | 4.128345 |
| MYL12A | 0.773718 | 0.529197 | 0.602043 | 1.74932 |
| GATAD2B | 0.773745 | 0.529274 | -0.29597 | 4.649452 |
| HTT-AS | 0.773827 | 0.529404 | 1.246955 | 0.382281 |
| FUK | 0.773827 | 0.529448 | 0.316888 | 3.945078 |
| LOC10050 | 0.774005 | 0.529629 | 0.31249 | 5.29736 |
| FAM155B | 0.77407 | 0.529746 | 1.127283 | 0.55184 |
| GCLC | 0.77407 | 0.529791 | 0.7856 | 1.016446 |
| PHF7 | 0.774252 | 0.530025 | -0.47435 | 2.287702 |
| ZNF708 | 0.774252 | 0.530034 | -0.99511 | 0.872389 |
| DNAJC8 | 0.774284 | 0.530115 | -0.23039 | 6.366118 |
| MASP1 | 0.774393 | 0.530249 | 1.042252 | 0.639939 |
| ABCC5-AS1 | 0.774552 | 0.530416 | -0.52157 | 2.448413 |
| C2CD5 | 0.774973 | 0.530805 | 0.808013 | 0.975397 |
| RABIF | 0.774973 | 0.530864 | -0.44334 | 3.52984 |
| DNAJC11 | 0.774973 | 0.530946 | -0.22701 | 6.225243 |
| PDZD8 | 0.774973 | 0.530999 | 0.277102 | 7.876352 |
| IMPACT | 0.774973 | 0.531 | 0.331513 | 5.919902 |
| PSMC1 | 0.775077 | 0.531162 | -0.3276 | 5.299358 |
| ADAMTS5 | 0.775077 | 0.531201 | 0.517759 | 3.758834 |
| FER1L5 | 0.775077 | 0.531249 | 0.461042 | 2.823632 |
| ZNF486 | 0.77518 | 0.531378 | 0.478514 | 3.339039 |
| WASH5P | 0.775382 | 0.531623 | 0.492525 | 2.465823 |
| KDM4A | 0.775382 | 0.531635 | 0.247423 | 6.453444 |
| BOD1 | 0.775383 | 0.531695 | -0.75232 | 1.425841 |
| YWHAG | 0.775474 | 0.531852 | -0.26487 | 8.317676 |

| | | | | |
|---|---|---|---|---|
| SNORA21 | 0.775474 | 0.53189 | 0.271585 | 8.614084 |
| INTS4 | 0.775474 | 0.531942 | 0.270239 | 5.127465 |
| RAD51AP2 | 0.775474 | 0.531993 | -0.84894 | 1.051598 |
| LINC01873 | 0.775502 | 0.532071 | -0.68603 | 1.964244 |
| TIMM17A | 0.775558 | 0.532206 | 0.293151 | 4.923634 |
| CTDSPL2 | 0.775558 | 0.532274 | 0.221315 | 6.478329 |
| ZNF891 | 0.775558 | 0.532287 | -0.38148 | 3.037499 |
| LSM8 | 0.775626 | 0.532393 | 0.215322 | 6.016792 |
| FKBP9 | 0.776091 | 0.532822 | 0.765646 | 0.981135 |
| ARHGEF3-/ | 0.776091 | 0.532855 | 0.873878 | 0.840144 |
| MED13L | 0.776091 | 0.53289 | -0.22319 | 8.474204 |
| LOC100507 | 0.776264 | 0.533068 | 0.8747 | 0.690238 |
| PHF2 | 0.776395 | 0.533217 | 0.231735 | 6.492612 |
| SNORD97 | 0.776419 | 0.533293 | 0.337558 | 9.562559 |
| AGK | 0.776783 | 0.533602 | -0.27302 | 4.628297 |
| TRABD | 0.777032 | 0.533832 | 0.27255 | 5.744321 |
| CTDNEP1 | 0.777084 | 0.533944 | -0.52516 | 1.602776 |
| RHBDF1 | 0.777084 | 0.534138 | 0.226164 | 5.687122 |
| KIAA0586 | 0.777084 | 0.534141 | 0.804562 | 0.362841 |
| NANS | 0.777084 | 0.534144 | 0.282614 | 5.76383 |
| MIR924HG | 0.777084 | 0.534164 | 0.948618 | 0.644896 |
| INTS5 | 0.777247 | 0.53435 | -0.40737 | 4.147407 |
| GPR183 | 0.777247 | 0.534394 | 0.296366 | 4.901554 |
| SPATS2L | 0.777302 | 0.534491 | 0.722358 | 0.839908 |
| IPP | 0.77732 | 0.534616 | -0.34656 | 3.441834 |
| NUDCD3 | 0.77732 | 0.534649 | -0.23084 | 7.087015 |
| JPH3 | 0.77732 | 0.534695 | 0.840761 | 0.451999 |
| RNF145 | 0.77732 | 0.53478 | -0.46648 | 3.161878 |
| CAPN12 | 0.77732 | 0.534927 | 0.346402 | 4.037192 |
| NRXN3 | 0.77732 | 0.534977 | -1.07648 | 0.976055 |
| ACCS | 0.77732 | 0.535 | 0.732037 | 1.029985 |
| SV2C | 0.77732 | 0.535002 | 0.587317 | 1.776956 |
| ITFG2 | 0.77732 | 0.535056 | 0.346647 | 4.495757 |
| TARBP2 | 0.77732 | 0.535096 | -0.74339 | 0.745268 |
| MPV17L2 | 0.77739 | 0.535204 | 0.537106 | 2.041716 |
| EBAG9 | 0.777814 | 0.535555 | 0.821167 | 1.376804 |
| SART3 | 0.777901 | 0.535674 | 0.226871 | 6.890628 |
| TRMT12 | 0.777909 | 0.535738 | 0.370915 | 3.807889 |
| MBNL1 | 0.777983 | 0.535939 | 0.312878 | 3.458234 |
| RRP9 | 0.777983 | 0.536043 | 0.334191 | 3.886133 |
| AP3S2 | 0.777983 | 0.536063 | -0.72106 | 1.369008 |
| LMNB2 | 0.777983 | 0.536073 | 0.249826 | 7.228193 |
| GPR75 | 0.777983 | 0.536086 | 0.41812 | 2.559777 |
| SAMD1 | 0.778205 | 0.536299 | -0.25582 | 5.746969 |
| COPZ2 | 0.778382 | 0.536486 | 0.422646 | 3.20061 |

| | | | | |
|---|---|---|---|---|
| RSAD1 | 0.778382 | 0.536571 | -0.48379 | 1.646948 |
| YARS2 | 0.778382 | 0.536598 | -0.29122 | 3.881655 |
| CCDC144CI | 0.778438 | 0.536696 | 0.590526 | 1.56571 |
| LRPPRC | 0.778452 | 0.536787 | 0.25026 | 7.867411 |
| PTGES2 | 0.778452 | 0.536825 | -0.53861 | 1.873635 |
| C3orf36 | 0.778657 | 0.537025 | 0.989577 | 0.635008 |
| LRRC46 | 0.778687 | 0.537105 | 0.606965 | 2.198512 |
| DLG1 | 0.778733 | 0.537197 | -0.7272 | 0.484324 |
| ADA | 0.778751 | 0.537307 | -0.40299 | 3.834525 |
| CERS5 | 0.778751 | 0.537365 | 0.448433 | 3.30772 |
| EDC4 | 0.778751 | 0.537387 | 0.223711 | 6.060431 |
| SNORD113 | 0.778801 | 0.537481 | 1.18996 | 0.589232 |
| ZNF573 | 0.778898 | 0.537607 | 0.881376 | 1.200617 |
| SLC51A | 0.779014 | 0.537746 | -0.582 | 2.207877 |
| HAPLN1 | 0.779373 | 0.538099 | -0.60208 | 2.009206 |
| TMEM100 | 0.779373 | 0.538113 | 1.266088 | 0.408878 |
| ZCCHC14 | 0.779653 | 0.538365 | 0.220559 | 6.854519 |
| SLC35E2A | 0.779935 | 0.538661 | 0.315742 | 4.665659 |
| HINT3 | 0.779935 | 0.538679 | -0.23584 | 6.192847 |
| EFNA1 | 0.780171 | 0.538902 | -0.74737 | 1.18594 |
| HACD1 | 0.780331 | 0.539072 | -0.49787 | 2.231466 |
| ZNF132 | 0.780368 | 0.539168 | -0.47354 | 2.669569 |
| LINC02449 | 0.780368 | 0.539216 | -0.6859 | 0.785517 |
| MYO19 | 0.780392 | 0.539292 | -0.78185 | 0.993066 |
| CDC42EP1 | 0.780476 | 0.539516 | 0.32834 | 6.97684 |
| LINC00987 | 0.780476 | 0.539567 | 0.254241 | 4.785328 |
| RPP30 | 0.780476 | 0.539634 | 0.399259 | 3.192995 |
| ACOXL | 0.780476 | 0.539706 | 0.949295 | 0.997132 |
| GNB1L | 0.780476 | 0.539745 | 0.645713 | 1.438906 |
| LOC100506 | 0.780476 | 0.539754 | 0.362252 | 3.458436 |
| ZNF204P | 0.780476 | 0.539767 | 0.545341 | 2.430686 |
| SEMA6A | 0.780676 | 0.539965 | -0.90515 | 0.452825 |
| SLC3A2 | 0.780821 | 0.540124 | -0.47564 | 1.757733 |
| VSIG2 | 0.781161 | 0.540419 | -0.55548 | 4.790204 |
| KPNA2 | 0.781965 | 0.541064 | 0.361762 | 5.530137 |
| SLC2A1-AS | 0.781965 | 0.541094 | -0.65948 | 1.905449 |
| ZNF558 | 0.782114 | 0.541257 | 0.464688 | 2.638059 |
| SP2 | 0.782121 | 0.541321 | -0.26087 | 5.62501 |
| SFXN1 | 0.782136 | 0.541391 | -0.25375 | 5.456997 |
| EML5 | 0.782528 | 0.541723 | -0.31463 | 4.217236 |
| EGR4 | 0.782551 | 0.541798 | -1.02967 | 0.812669 |
| TM9SF4 | 0.782573 | 0.541873 | -0.22944 | 7.089765 |
| LINC01138 | 0.782848 | 0.542123 | 0.457042 | 3.532218 |
| TTL | 0.783018 | 0.5423 | 0.23042 | 6.003778 |
| MTG1 | 0.783062 | 0.54244 | 0.257451 | 5.36423 |

| | | | | |
|---|---|---|---|---|
| SNORD42B | 0.783062 | 0.542492 | 0.59236 | 1.801399 |
| GHRLOS | 0.783062 | 0.54251 | 0.78086 | 0.778278 |
| ING5 | 0.78323 | 0.54272 | 0.29111 | 5.145857 |
| LRRC41 | 0.78323 | 0.542746 | -0.23719 | 6.116004 |
| B4GALT7 | 0.783237 | 0.542833 | 0.312007 | 3.629137 |
| CENPB | 0.783237 | 0.54287 | 0.231582 | 6.807916 |
| MAFF | 0.783286 | 0.543021 | -0.79604 | 1.059357 |
| LINC00400 | 0.783286 | 0.543023 | -0.91108 | 0.501968 |
| RNU2-1 | 0.783445 | 0.543224 | -0.43491 | 3.478895 |
| WDR45 | 0.783445 | 0.543324 | 0.480826 | 1.890022 |
| HECTD2 | 0.783445 | 0.543346 | 0.509032 | 2.120131 |
| STARD7-AS | 0.783445 | 0.54339 | -0.36083 | 3.179038 |
| CEACAM6 | 0.783445 | 0.543442 | -0.51199 | 9.059887 |
| SPTLC1 | 0.783445 | 0.543581 | -0.49015 | 2.284479 |
| KMT2B | 0.783445 | 0.543608 | 0.220865 | 7.685733 |
| SF3A1 | 0.783445 | 0.543612 | 0.215928 | 7.690039 |
| RPL23P8 | 0.78356 | 0.54375 | -0.5389 | 1.559788 |
| TWF2 | 0.783714 | 0.543946 | -0.25474 | 5.193637 |
| TP73-AS1 | 0.783714 | 0.543977 | 0.434965 | 2.72265 |
| C11orf58 | 0.783742 | 0.544147 | -0.21755 | 7.595521 |
| 8-Mar | 0.783742 | 0.544205 | 0.552672 | 1.644928 |
| GPR174 | 0.783742 | 0.544217 | 0.86002 | 1.062625 |
| LSM6 | 0.783742 | 0.544235 | 0.340553 | 3.842969 |
| UBE2N | 0.783766 | 0.544327 | -0.28876 | 5.99277 |
| HMGN3-AS | 0.783766 | 0.544371 | 0.469508 | 3.220126 |
| GP5 | 0.783781 | 0.544441 | -1.15005 | 0.582607 |
| HCG4 | 0.783814 | 0.544525 | -0.70575 | 1.710255 |
| WRAP73 | 0.784029 | 0.544733 | 0.33678 | 3.693586 |
| PSMD6-AS | 0.784746 | 0.545291 | 0.295909 | 4.426405 |
| CDK13 | 0.785038 | 0.545612 | -0.99704 | 0.603093 |
| CLDN20 | 0.785038 | 0.545681 | 0.372144 | 2.833692 |
| FRG1DP | 0.785038 | 0.545776 | 0.786343 | 2.191194 |
| SETDB2 | 0.785038 | 0.545795 | 0.513933 | 2.172932 |
| PAMR1 | 0.785038 | 0.545804 | 1.160079 | 1.504942 |
| SNORD134 | 0.785038 | 0.545853 | -0.49034 | 2.015238 |
| IRX2 | 0.785082 | 0.545983 | -0.66686 | 1.999848 |
| NUP50-DT | 0.785082 | 0.546004 | -0.53337 | 1.969421 |
| RAB11B | 0.785102 | 0.54608 | -0.20969 | 6.048568 |
| LY9 | 0.785102 | 0.546137 | -0.91769 | 0.684564 |
| DGKE | 0.785156 | 0.546282 | 0.275562 | 5.113636 |
| LOC10272 | 0.785156 | 0.546313 | -0.61002 | 1.442315 |
| ZNRF3 | 0.785156 | 0.546494 | 0.78415 | 0.842582 |
| TIAF1 | 0.785156 | 0.546623 | -0.52282 | 1.844453 |
| TRAP1 | 0.785156 | 0.546708 | 0.467499 | 2.235591 |
| SNHG20 | 0.785156 | 0.54673 | 0.463602 | 2.278623 |

| | | | | |
|---|---|---|---|---|
| LOC100132 | 0.785156 | 0.546914 | 0.933272 | 0.549581 |
| FAHD2B | 0.785156 | 0.54694 | -0.51231 | 2.679162 |
| IL17RD | 0.785156 | 0.546961 | 0.305214 | 4.302079 |
| FAM160B1 | 0.785156 | 0.546977 | 0.250621 | 4.743767 |
| DPY19L3 | 0.785156 | 0.547015 | -0.81824 | 0.600465 |
| IL27RA | 0.785156 | 0.547036 | 0.499331 | 2.945129 |
| TULP4 | 0.785156 | 0.54706 | 0.242516 | 5.181214 |
| RPL15 | 0.785156 | 0.547066 | -0.57315 | 1.719052 |
| C6orf47 | 0.785156 | 0.547078 | -0.68767 | 0.380521 |
| SLC47A1 | 0.785156 | 0.547132 | 0.5772 | 1.512334 |
| GMDS-DT | 0.785295 | 0.547289 | 0.468223 | 2.953194 |
| PPP1R15A | 0.78559 | 0.547554 | 0.313581 | 8.218802 |
| NARF | 0.785714 | 0.547701 | -0.7516 | 0.847805 |
| CXCL12 | 0.786011 | 0.548032 | 0.524797 | 2.672068 |
| LINC00473 | 0.786011 | 0.548041 | -0.7926 | 1.266128 |
| RBM17 | 0.786011 | 0.548236 | -0.39727 | 3.174142 |
| LRRC57 | 0.786011 | 0.548312 | 0.251711 | 4.684041 |
| SETD3 | 0.786011 | 0.548331 | 0.248168 | 5.512235 |
| ST13P4 | 0.786011 | 0.548346 | -0.25038 | 4.58562 |
| AUTS2 | 0.786011 | 0.548408 | -0.91258 | 0.595375 |
| TNFRSF1A | 0.786011 | 0.548415 | -0.22533 | 6.696826 |
| PDCD5 | 0.786011 | 0.548447 | -0.2718 | 5.292366 |
| NDUFB2 | 0.786656 | 0.548966 | -0.37799 | 4.327264 |
| DNMBP | 0.786656 | 0.549017 | 0.250974 | 6.594422 |
| ATR | 0.787115 | 0.549397 | 0.225962 | 6.742015 |
| DLL1 | 0.787171 | 0.549537 | -0.47366 | 3.318177 |
| FAM243A | 0.787171 | 0.549556 | -1.2118 | 0.47079 |
| LCN12 | 0.78726 | 0.549678 | -0.76371 | 0.687491 |
| PLPBP | 0.787318 | 0.549779 | 0.238794 | 5.740379 |
| FOXP2 | 0.787324 | 0.549855 | 0.709382 | 2.431277 |
| SACS | 0.787324 | 0.549903 | 0.87898 | 0.9181 |
| SPINT2 | 0.787533 | 0.550191 | -0.29282 | 3.807659 |
| GNA11 | 0.787533 | 0.550215 | 0.211551 | 6.435607 |
| LARP7 | 0.787533 | 0.550229 | 0.578176 | 1.254839 |
| CHAD | 0.787564 | 0.550336 | -0.37504 | 3.785972 |
| SNRNP25 | 0.787564 | 0.550387 | -0.41221 | 3.456991 |
| RBM19 | 0.787564 | 0.550449 | -0.30431 | 3.748632 |
| FKBP2 | 0.787564 | 0.55049 | -0.58789 | 1.218922 |
| ATF1 | 0.787733 | 0.550669 | 0.277741 | 4.606821 |
| SHOC2 | 0.787934 | 0.550895 | -0.57265 | 1.040232 |
| NUDT3 | 0.787934 | 0.550943 | -0.40404 | 2.499887 |
| MINDY3 | 0.787934 | 0.550989 | -0.24522 | 5.188544 |
| EXOC2 | 0.788056 | 0.551135 | -0.68638 | 0.512662 |
| TMEM236 | 0.788293 | 0.55136 | 0.889471 | 0.762791 |
| C15orf40 | 0.788369 | 0.551497 | -0.48688 | 2.208773 |

| | | | | |
|---|---|---|---|---|
| MTBP | 0.788369 | 0.551613 | 0.371724 | 3.565019 |
| RNU4-2 | 0.788369 | 0.55164 | 0.311939 | 10.44172 |
| LONP2 | 0.788369 | 0.551654 | 0.328552 | 3.774398 |
| SLC31A1 | 0.788411 | 0.551743 | -0.27035 | 6.256907 |
| RHOXF1-AS | 0.788595 | 0.551932 | 0.547051 | 1.86233 |
| SMYD5 | 0.788627 | 0.552043 | 0.285006 | 4.33683 |
| ACSL1 | 0.788627 | 0.552102 | 0.378367 | 3.26761 |
| GOLGA8O | 0.788627 | 0.552135 | -0.55702 | 1.705432 |
| PRPF38A | 0.788712 | 0.552255 | 0.211332 | 6.356344 |
| MAP4K3-D | 0.78889 | 0.552477 | 0.378391 | 3.720138 |
| MLF2 | 0.78889 | 0.552542 | -0.48525 | 2.333376 |
| ERP29 | 0.78889 | 0.55264 | 0.629839 | 0.966329 |
| TDP1 | 0.78889 | 0.552675 | -0.56754 | 0.551409 |
| HCG25 | 0.78889 | 0.55268 | 0.601701 | 1.943952 |
| GET4 | 0.789224 | 0.552974 | 0.241029 | 5.751929 |
| LOC100499 | 0.789545 | 0.553259 | 0.753496 | 1.231669 |
| ZNF324 | 0.789603 | 0.55336 | 0.29139 | 4.077578 |
| USP51 | 0.789618 | 0.553431 | 0.76135 | 1.286135 |
| BRF1 | 0.789744 | 0.553601 | -0.46472 | 2.625116 |
| C3orf38 | 0.789744 | 0.553761 | 0.271205 | 4.312939 |
| SVIL | 0.789744 | 0.553769 | -0.30073 | 6.475463 |
| TESMIN | 0.789744 | 0.553773 | 0.768156 | 1.279423 |
| DBP | 0.789744 | 0.55382 | -0.34988 | 3.647786 |
| TSNARE1 | 0.789841 | 0.553948 | 0.325102 | 3.703267 |
| CLDN24 | 0.789987 | 0.554111 | 1.111309 | 0.386475 |
| R3HDM2 | 0.790047 | 0.554213 | 0.215887 | 7.284571 |
| STXBP4 | 0.790162 | 0.554354 | 0.338397 | 4.567109 |
| C6orf226 | 0.790825 | 0.554879 | 0.641979 | 1.340297 |
| ZFP28 | 0.790868 | 0.55497 | -0.35594 | 3.38967 |
| MIR6773 | 0.790902 | 0.555069 | -0.70498 | 1.06496 |
| ACSM3 | 0.790902 | 0.55515 | 0.784579 | 1.692137 |
| ACTRT3 | 0.790902 | 0.555175 | 0.741906 | 0.922753 |
| SATB2 | 0.791011 | 0.555311 | 0.673861 | 1.141867 |
| C2orf92 | 0.791195 | 0.555501 | 0.649518 | 1.828198 |
| HIST1H2AJ | 0.791448 | 0.555738 | 0.351207 | 5.115872 |
| LOC101928 | 0.791517 | 0.555848 | 0.515128 | 1.837979 |
| POF1B | 0.791655 | 0.556005 | 0.356085 | 7.13295 |
| MYO3B-AS | 0.791681 | 0.556083 | -1.07637 | 0.914216 |
| RAD52 | 0.792425 | 0.556666 | 0.56482 | 1.478754 |
| SNCG | 0.792444 | 0.556765 | -0.51317 | 3.141301 |
| HERC2P2 | 0.792444 | 0.556845 | 0.227336 | 6.915798 |
| LINC01572 | 0.792444 | 0.556861 | 0.365652 | 3.408161 |
| CHRNG | 0.792497 | 0.556959 | 0.524492 | 3.121051 |
| SERINC3 | 0.792743 | 0.557204 | -0.24878 | 6.285886 |
| RTN4IP1 | 0.792743 | 0.557252 | -0.31347 | 3.744998 |

| | | | | |
|---|---|---|---|---|
| FRYL | 0.792934 | 0.557447 | -0.21488 | 7.870855 |
| C12orf10 | 0.793461 | 0.557899 | -0.27598 | 3.849065 |
| PDCD6 | 0.793461 | 0.557938 | -0.34417 | 3.010193 |
| SLC7A11-A | 0.793574 | 0.558078 | 0.440306 | 3.272091 |
| MFSD14A | 0.793754 | 0.558377 | -0.23834 | 5.95815 |
| C1orf159 | 0.793754 | 0.558388 | -0.29983 | 4.134158 |
| EIF4E | 0.793754 | 0.558417 | -0.99231 | 0.478959 |
| CRLS1 | 0.793754 | 0.558446 | 0.511588 | 1.287999 |
| ARID1A | 0.79393 | 0.558777 | -0.2693 | 4.230683 |
| NFXL1 | 0.79393 | 0.55878 | -1.0374 | 0.371067 |
| TMEM14EI | 0.79393 | 0.558809 | 0.261918 | 4.860278 |
| GPS2 | 0.79393 | 0.558859 | -0.21989 | 6.516862 |
| EXOSC6 | 0.79393 | 0.558873 | -0.2704 | 4.267493 |
| PHF5A | 0.794354 | 0.559255 | 0.280112 | 4.304401 |
| STK11IP | 0.794354 | 0.559292 | 0.249334 | 4.608006 |
| GKN2 | 0.794392 | 0.55938 | 1.142692 | 3.334801 |
| ZNF117 | 0.794462 | 0.55949 | -0.31723 | 7.06655 |
| GSPT2 | 0.794743 | 0.559748 | 0.387702 | 3.214019 |
| PUS3 | 0.794746 | 0.559811 | -0.51338 | 1.68758 |
| PNPLA8 | 0.795272 | 0.560307 | -0.58933 | 1.841311 |
| TRAPPC10 | 0.795272 | 0.560333 | -0.21533 | 6.497049 |
| RNA45SN5 | 0.795272 | 0.560363 | -0.22457 | 15.16764 |
| CBX3 | 0.795462 | 0.560558 | 0.489542 | 1.596833 |
| CC2D1B | 0.795531 | 0.56073 | 0.212892 | 5.964896 |
| TMEM242 | 0.795531 | 0.560745 | 0.323381 | 3.888297 |
| LOC441455 | 0.795531 | 0.560788 | -0.85851 | 0.63876 |
| MRPL52 | 0.795611 | 0.560905 | 0.500479 | 2.042297 |
| YES1 | 0.795676 | 0.561012 | -0.2926 | 7.719603 |
| FKBP5 | 0.795726 | 0.561108 | 0.274823 | 6.422734 |
| MIR657 | 0.796009 | 0.561406 | -0.71717 | 0.712151 |
| DHRS9 | 0.796009 | 0.561428 | -0.79243 | 3.675717 |
| NLGN4Y | 0.796233 | 0.561675 | -1.0127 | 0.971166 |
| MAGOHB | 0.796233 | 0.561708 | 0.425261 | 3.184673 |
| TBC1D22A- | 0.796272 | 0.56189 | -0.25856 | 4.481327 |
| DPM2 | 0.796272 | 0.561905 | -0.26314 | 4.28954 |
| GLIPR2 | 0.796272 | 0.561949 | 0.529586 | 2.527331 |
| CLN5 | 0.796272 | 0.561992 | 0.24289 | 4.576875 |
| LOC103611 | 0.796272 | 0.562043 | 0.340347 | 3.867689 |
| SNORA69 | 0.796272 | 0.562099 | 0.491434 | 2.513573 |
| MAML1 | 0.796328 | 0.562256 | 0.20618 | 6.359533 |
| NOXRED1 | 0.796328 | 0.56226 | 0.592258 | 1.026342 |
| FAM133B | 0.796337 | 0.562395 | -0.79673 | 0.97557 |
| FAM66C | 0.796337 | 0.562424 | 0.71173 | 1.698191 |
| MTMR8 | 0.796337 | 0.562449 | -0.41524 | 3.212865 |
| CFAP410 | 0.796337 | 0.562531 | 0.752013 | 0.956516 |

| | | | | |
|---|---|---|---|---|
| SSTR2 | 0.796337 | 0.56257 | 0.734295 | 0.74159 |
| ZNF790 | 0.796362 | 0.56277 | -0.44631 | 2.791104 |
| LAGE3 | 0.796362 | 0.562817 | 0.541066 | 1.857072 |
| TMEM104 | 0.796362 | 0.562823 | -0.23559 | 4.573559 |
| LOC10023: | 0.796362 | 0.56283 | -0.67509 | 1.352599 |
| MPP4 | 0.796386 | 0.562908 | 0.950984 | 1.498729 |
| PPP1R12A | 0.797115 | 0.563494 | 0.442626 | 1.897733 |
| VARS | 0.797115 | 0.563545 | 0.475745 | 2.511139 |
| AQP11 | 0.797375 | 0.56379 | 0.475268 | 2.585517 |
| ECT2 | 0.797497 | 0.563937 | 0.918789 | 0.866325 |
| LOC73018: | 0.797505 | 0.564052 | 0.569409 | 1.924568 |
| SNORA3A | 0.797505 | 0.564064 | 0.28801 | 7.428643 |
| UCHL3 | 0.797745 | 0.564518 | -0.61567 | 1.204342 |
| ANKRD20A | 0.797745 | 0.564529 | 0.48631 | 2.610987 |
| LZTS1 | 0.797745 | 0.564537 | -0.29489 | 4.219413 |
| DANCR | 0.797745 | 0.564556 | -0.2767 | 4.859137 |
| GSE1 | 0.797745 | 0.564626 | -0.50883 | 1.711701 |
| STC2 | 0.797745 | 0.564658 | 0.4823 | 3.085163 |
| NPC1 | 0.797745 | 0.564659 | 0.260355 | 6.852844 |
| HTR3B | 0.797881 | 0.564816 | -1.17989 | 0.688692 |
| RPL30 | 0.797886 | 0.564905 | -0.23439 | 8.08854 |
| SH3BP5L | 0.797886 | 0.564941 | -0.22793 | 5.507543 |
| ITGB1BP1 | 0.798081 | 0.56514 | 0.770908 | 0.867835 |
| CCL22 | 0.798247 | 0.565319 | 0.480844 | 2.733539 |
| USP37 | 0.798368 | 0.565468 | 0.239338 | 6.411818 |
| KANK4 | 0.798368 | 0.565584 | 0.868305 | 0.845381 |
| LMF2 | 0.798368 | 0.565638 | -0.24109 | 5.146607 |
| SIRPA | 0.798368 | 0.565696 | -0.79217 | 0.691747 |
| ARNTL2 | 0.798368 | 0.565786 | 0.830545 | 1.547104 |
| C1GALT1 | 0.798368 | 0.56583 | 0.223934 | 7.006033 |
| RPS25 | 0.798368 | 0.5659 | -0.21721 | 7.900084 |
| SPDYE1 | 0.798368 | 0.565934 | -0.27222 | 4.788475 |
| HAND2-AS: | 0.798368 | 0.565952 | 0.962338 | 0.813488 |
| LINC01644 | 0.79841 | 0.566079 | 0.947578 | 0.299064 |
| FCHO2 | 0.79841 | 0.566103 | -0.70554 | 0.79685 |
| IAH1 | 0.798577 | 0.566282 | 0.282162 | 3.818952 |
| ZC3H6 | 0.798607 | 0.566385 | -0.23586 | 6.374478 |
| ZFAND3 | 0.798607 | 0.566425 | -0.21632 | 6.422793 |
| CPAMD8 | 0.79861 | 0.566575 | -0.49039 | 1.811507 |
| PIK3CA | 0.79861 | 0.566657 | 0.212838 | 6.162848 |
| LINC01473 | 0.79861 | 0.566695 | 0.475927 | 2.270356 |
| KIF9-AS1 | 0.79861 | 0.566721 | 0.627678 | 0.887322 |
| GOT2 | 0.79861 | 0.56674 | -0.81634 | 0.687881 |
| SLC7A6OS | 0.79861 | 0.566793 | 0.344013 | 3.118561 |
| ABRACL | 0.798857 | 0.567029 | 0.289427 | 4.464893 |

| | | | | |
|---|---|---|---|---|
| CTC-338M1 | 0.798873 | 0.567276 | 0.476774 | 2.114946 |
| GOLGA8B | 0.798873 | 0.567308 | 0.372444 | 3.051944 |
| MTM1 | 0.798873 | 0.567358 | -0.26348 | 5.199998 |
| ERLIN2 | 0.798873 | 0.567377 | 0.23539 | 6.187252 |
| EDRF1-AS1 | 0.798873 | 0.567414 | 0.60465 | 1.670125 |
| PRKD2 | 0.798873 | 0.567457 | -0.43136 | 1.824473 |
| MARK1 | 0.798873 | 0.567523 | -0.69456 | 1.785528 |
| SETD5 | 0.798873 | 0.567624 | -0.31433 | 3.117502 |
| SUN2 | 0.798873 | 0.567631 | -0.47869 | 2.485158 |
| ASH1L-AS1 | 0.798873 | 0.567649 | 0.339389 | 3.352199 |
| CSNK1E | 0.798955 | 0.567768 | -0.62938 | 1.826296 |
| TAF11 | 0.799029 | 0.568026 | -0.61258 | 1.49822 |
| ERCC6L2 | 0.799029 | 0.568028 | 0.208976 | 6.024109 |
| LMO7 | 0.799029 | 0.568125 | 0.35899 | 2.827823 |
| ADAM10 | 0.799029 | 0.568135 | 0.225218 | 7.699393 |
| PTPRCAP | 0.799029 | 0.56814 | 0.325344 | 3.410587 |
| MFSD9 | 0.799029 | 0.568187 | 0.227661 | 4.802447 |
| ZNF572 | 0.799748 | 0.568759 | 0.503219 | 2.13716 |
| DUSP28 | 0.799883 | 0.568915 | 0.480539 | 2.60023 |
| TMEM238L | 0.800016 | 0.569071 | 0.57105 | 2.975296 |
| WARS2-IT1 | 0.800696 | 0.569616 | 0.81502 | 0.915557 |
| GTF2H2B | 0.800896 | 0.569819 | 0.526681 | 1.398614 |
| STX16-NPE | 0.800918 | 0.569895 | 0.793388 | 0.807113 |
| MIB1 | 0.800976 | 0.569998 | 0.240955 | 7.711885 |
| SRGAP2C | 0.801005 | 0.570079 | 0.336775 | 3.543257 |
| SGK1 | 0.801114 | 0.570374 | 0.676105 | 0.655531 |
| MCEMP1 | 0.801114 | 0.570385 | -0.84948 | 1.315965 |
| LAMA5 | 0.801114 | 0.570547 | 0.243063 | 8.004778 |
| ANKHD1-EI | 0.801114 | 0.570579 | -0.78644 | 0.616679 |
| DEFB131B | 0.801114 | 0.570694 | -0.66941 | 1.328192 |
| MKLN1 | 0.801114 | 0.57073 | -0.6816 | 0.71673 |
| WDR83OS | 0.801114 | 0.570732 | -0.3623 | 3.240894 |
| ARAF | 0.801114 | 0.570836 | 0.748177 | 0.68163 |
| CTAGE8 | 0.801114 | 0.570914 | -0.51902 | 2.73557 |
| SNORD140 | 0.801114 | 0.570931 | 0.49956 | 1.561362 |
| ZNF319 | 0.801114 | 0.571005 | 0.323094 | 3.541129 |
| MTMR10 | 0.801114 | 0.57101 | -0.30775 | 4.301349 |
| INPP5F | 0.801114 | 0.57106 | 0.263858 | 4.419339 |
| TRHDE-AS1 | 0.801114 | 0.571122 | 1.064266 | 0.636746 |
| OGFR-AS1 | 0.801114 | 0.571144 | -0.71273 | 0.554543 |
| RMI1 | 0.801114 | 0.571259 | -0.28407 | 4.153483 |
| NTHL1 | 0.801114 | 0.571281 | 0.512328 | 2.716474 |
| PHF20L1 | 0.801114 | 0.571302 | 0.484268 | 2.715981 |
| FOXN3-AS2 | 0.801114 | 0.571317 | 0.430263 | 1.785629 |
| SRGAP2 | 0.801147 | 0.571434 | 0.315358 | 3.637978 |

| | | | | |
|---|---|---|---|---|
| TLE3 | 0.801147 | 0.571475 | -0.85873 | 0.651526 |
| TP53TG1 | 0.801147 | 0.571553 | -0.37237 | 2.782849 |
| RALGDS | 0.801147 | 0.571676 | -0.38256 | 2.863605 |
| FAM118A | 0.801147 | 0.571709 | -0.65518 | 1.225891 |
| RAB3A | 0.801147 | 0.571762 | 0.602098 | 1.757952 |
| TMEM102 | 0.801147 | 0.571774 | -0.33995 | 3.364194 |
| ANKRD46 | 0.801147 | 0.571829 | 0.544212 | 1.607818 |
| LOC10050( | 0.801291 | 0.571997 | -0.40057 | 3.06099 |
| QPRT | 0.801291 | 0.572138 | 0.550254 | 1.924187 |
| FAM174B | 0.801291 | 0.572207 | -0.35945 | 3.81885 |
| MRTO4 | 0.801291 | 0.572278 | -0.26716 | 5.118909 |
| GUSBP2 | 0.801291 | 0.572326 | 0.538311 | 1.927749 |
| SPDYE8P | 0.801291 | 0.572368 | 0.597672 | 2.428878 |
| MED14 | 0.801291 | 0.572394 | 0.225201 | 6.71992 |
| RBM4B | 0.801291 | 0.572478 | 0.44609 | 3.000192 |
| CPA3 | 0.801291 | 0.572515 | 0.351928 | 3.825044 |
| WFIKKN1 | 0.801291 | 0.572543 | 0.74642 | 1.026304 |
| DEPDC4 | 0.801642 | 0.572854 | 0.756963 | 0.906775 |
| TMEM167I | 0.801841 | 0.573082 | -0.23356 | 5.329562 |
| SPAG5-AS1 | 0.801841 | 0.573154 | -0.74714 | 1.01726 |
| SMARCAD1 | 0.801841 | 0.57318 | 0.480379 | 1.788948 |
| DIRAS1 | 0.802012 | 0.573397 | 0.546785 | 2.484162 |
| ZNF296 | 0.802012 | 0.573449 | -0.46513 | 2.960906 |
| SLCO3A1 | 0.802012 | 0.573486 | -0.32413 | 4.566681 |
| RASL11A | 0.802336 | 0.573778 | 0.62002 | 1.959241 |
| IFRD2 | 0.802557 | 0.573998 | 0.26726 | 4.69835 |
| LOC10272: | 0.802801 | 0.574245 | 0.864137 | 0.529524 |
| GRIPAP1 | 0.802801 | 0.574345 | 0.209492 | 6.782493 |
| KHSRP | 0.802801 | 0.574377 | 0.198057 | 7.632998 |
| SNRPD2 | 0.802801 | 0.574417 | 0.829393 | 0.32587 |
| KLHL17 | 0.803158 | 0.574734 | -0.31328 | 3.293922 |
| HTR3C | 0.803177 | 0.574817 | -1.12662 | 0.98047 |
| UTRN | 0.803177 | 0.57487 | 0.224721 | 9.354623 |
| NFX1 | 0.80383 | 0.575399 | 0.25181 | 4.814054 |
| SEL1L3 | 0.804909 | 0.576232 | 0.827084 | 0.555253 |
| CTSD | 0.804951 | 0.576323 | -0.21749 | 8.22163 |
| SF3B1 | 0.805045 | 0.576469 | 0.196336 | 9.281108 |
| ZMYM2 | 0.805045 | 0.576513 | -0.74623 | 0.660455 |
| PJA2 | 0.805474 | 0.576922 | -0.22504 | 8.05959 |
| YAE1 | 0.805474 | 0.576987 | 0.454114 | 2.263465 |
| LOC101928 | 0.805474 | 0.577005 | -0.46265 | 1.234677 |
| LYRM4-AS1 | 0.805771 | 0.577279 | 0.468693 | 2.252295 |
| BTBD2 | 0.805803 | 0.577363 | 0.217759 | 6.102049 |
| RHBDF2 | 0.805866 | 0.577489 | 0.798469 | 1.102681 |
| CLDN18 | 0.805866 | 0.577546 | 0.879155 | 3.169745 |

| | | | | |
|---|---|---|---|---|
| ZNF705E | 0.805866 | 0.577593 | -0.70115 | 1.507762 |
| PPIAP30 | 0.806242 | 0.577978 | 0.796186 | 0.532632 |
| BRWD1-AS | 0.806242 | 0.578069 | -0.71937 | 0.661535 |
| OGA | 0.806242 | 0.578081 | 0.541329 | 2.035929 |
| SAP25 | 0.806242 | 0.578202 | 0.43944 | 2.415314 |
| LOC440311 | 0.806242 | 0.578226 | 0.728232 | 0.455992 |
| SOS2 | 0.806242 | 0.578254 | -0.20709 | 6.891807 |
| HESX1 | 0.806242 | 0.578348 | 0.878465 | 0.659053 |
| STAB2 | 0.806242 | 0.578409 | 0.359168 | 2.54024 |
| CTD-2350J1 | 0.806242 | 0.578472 | -0.80881 | 0.57579 |
| PSTPIP2 | 0.806242 | 0.578524 | -0.26937 | 4.154447 |
| ARL5B | 0.806242 | 0.578538 | -0.25098 | 5.875952 |
| MYO10 | 0.80644 | 0.578741 | 0.241673 | 8.213369 |
| SLC25A5-A | 0.806577 | 0.578941 | -0.49748 | 2.041015 |
| PPP1R3E | 0.806577 | 0.578962 | -0.31137 | 4.188979 |
| ZNF609 | 0.806804 | 0.579207 | 0.192147 | 7.349587 |
| MMRN2 | 0.806804 | 0.579249 | 0.327242 | 4.250573 |
| RAD51-AS1 | 0.80688 | 0.579365 | -0.30022 | 4.130826 |
| NPY1R | 0.807049 | 0.579548 | 0.97789 | 0.424536 |
| ARL5A | 0.807077 | 0.579812 | 0.48865 | 1.701285 |
| HIST1H1B | 0.807077 | 0.579848 | -0.29911 | 5.930665 |
| LINC01238 | 0.807077 | 0.579914 | 0.466934 | 2.111127 |
| SS18L1 | 0.807077 | 0.579932 | -0.6255 | 0.545934 |
| ATXN2L | 0.807077 | 0.580014 | -0.36849 | 2.382476 |
| ZBTB20-AS | 0.807077 | 0.580084 | 0.550732 | 1.951582 |
| FAM122A | 0.807077 | 0.580105 | 0.225444 | 4.976899 |
| OR10A5 | 0.807077 | 0.580117 | -0.80513 | 0.882849 |
| NUTM2B | 0.807077 | 0.580121 | -0.41601 | 2.910561 |
| MTRNR2L1 | 0.807149 | 0.580259 | -0.53164 | 1.540947 |
| POLR2C | 0.807149 | 0.580296 | 0.220291 | 5.34487 |
| SOX11 | 0.807336 | 0.580501 | 0.87502 | 0.906991 |
| MRPL13 | 0.807336 | 0.580601 | -0.32969 | 4.338174 |
| PDE6D | 0.807336 | 0.580655 | 0.506819 | 2.186178 |
| MIA2 | 0.807336 | 0.580676 | 0.398358 | 3.138338 |
| METTL2A | 0.807594 | 0.580924 | 0.25363 | 4.274224 |
| SSB | 0.807967 | 0.581325 | 0.727867 | 0.693327 |
| FBXO6 | 0.807967 | 0.581333 | 0.433358 | 2.649747 |
| RAPGEF6 | 0.807967 | 0.581385 | -0.39936 | 2.515236 |
| CDK2AP1 | 0.807967 | 0.581446 | -0.56716 | 1.018235 |
| THAP11 | 0.807967 | 0.5815 | -0.27113 | 4.305238 |
| PPP1R3D | 0.807983 | 0.581599 | -0.37542 | 4.099977 |
| CCDC34 | 0.807983 | 0.581682 | -0.45772 | 2.992402 |
| CAMK2N1 | 0.807983 | 0.581696 | 0.228245 | 6.644989 |
| ERCC5 | 0.808512 | 0.582163 | -0.38376 | 2.890873 |
| ZNF443 | 0.808512 | 0.5822 | 0.327441 | 3.448448 |

| | | | | |
|---|---|---|---|---|
| LRRC3 | 0.808848 | 0.582504 | 0.448849 | 2.291768 |
| ZC3H8 | 0.808887 | 0.582593 | -0.24184 | 5.311433 |
| TTC32 | 0.80893 | 0.582745 | -0.44268 | 2.330163 |
| AK9 | 0.80893 | 0.582748 | 0.320273 | 3.837409 |
| CDKL1 | 0.809253 | 0.583042 | 0.334994 | 3.20093 |
| ARSK | 0.809623 | 0.583384 | 0.355652 | 3.074568 |
| RGPD2 | 0.809623 | 0.583484 | -0.75054 | 0.659093 |
| HSPA8 | 0.809623 | 0.583494 | 0.225114 | 8.185774 |
| MBNL2 | 0.809637 | 0.583566 | 0.794279 | 0.55782 |
| PARS2 | 0.810542 | 0.584294 | -0.53592 | 2.421967 |
| EIF3F | 0.810542 | 0.584361 | -0.19669 | 6.032065 |
| AMPD3 | 0.810542 | 0.584403 | -0.43154 | 2.198408 |
| SPOUT1 | 0.81115 | 0.58494 | 0.234363 | 5.230355 |
| UST-AS1 | 0.81115 | 0.584965 | 0.557737 | 1.56824 |
| CRIM1-DT | 0.811205 | 0.585122 | 0.639702 | 0.978755 |
| HSCB | 0.811205 | 0.585128 | 0.400877 | 3.263786 |
| C3orf35 | 0.81129 | 0.585311 | 0.589104 | 1.761037 |
| NDUFA5 | 0.81129 | 0.585392 | -0.75373 | 0.600738 |
| ATP1A1-AS | 0.81129 | 0.585458 | 0.494795 | 2.613702 |
| RNF6 | 0.81129 | 0.585563 | 0.556231 | 1.260604 |
| SHE | 0.81129 | 0.585608 | 0.359616 | 3.307053 |
| LOC10050€ | 0.81129 | 0.585645 | 0.366259 | 2.691913 |
| GLRX5 | 0.81129 | 0.585707 | -0.36919 | 4.121046 |
| MIR4728 | 0.81129 | 0.585827 | -0.83175 | 0.811154 |
| LOC28400¢ | 0.81129 | 0.585855 | -0.6606 | 1.082423 |
| GCNT4 | 0.81129 | 0.586008 | -0.425 | 3.266017 |
| PHKB | 0.81129 | 0.586062 | -0.59257 | 1.437217 |
| MIR933 | 0.81129 | 0.586126 | 0.863151 | 0.424053 |
| ADH5 | 0.81129 | 0.586162 | 0.194624 | 6.380599 |
| TLR5 | 0.81129 | 0.586177 | -0.45173 | 4.038008 |
| SFPQ | 0.81129 | 0.586347 | 0.198202 | 9.321019 |
| MIR4785 | 0.81129 | 0.586457 | -0.6513 | 1.208546 |
| SLC35E3 | 0.81129 | 0.586536 | 0.305112 | 4.012559 |
| ATP5F1D | 0.81129 | 0.586557 | 0.586453 | 1.281026 |
| ZNF606 | 0.81129 | 0.586572 | -0.35393 | 3.382041 |
| SNRPG | 0.81129 | 0.586586 | 0.540333 | 1.214801 |
| WNT6 | 0.81129 | 0.586589 | -0.76132 | 1.697599 |
| TSHZ2 | 0.81129 | 0.586595 | -0.28922 | 4.400108 |
| CNDP2 | 0.81129 | 0.586612 | -0.4041 | 3.216044 |
| SH3GL1P1 | 0.811536 | 0.586851 | 0.388498 | 3.038253 |
| GINM1 | 0.811551 | 0.586998 | 0.26655 | 5.193111 |
| THAP10 | 0.811551 | 0.587005 | -0.6762 | 0.772324 |
| PELO | 0.811551 | 0.587106 | 0.227852 | 5.066225 |
| THAP6 | 0.811551 | 0.587134 | -0.66549 | 1.772999 |
| GUCA1B | 0.811551 | 0.587171 | -0.5733 | 2.189776 |

| | | | | |
|---|---|---|---|---|
| KRR1 | 0.811821 | 0.587429 | -0.20657 | 5.765118 |
| SNORA80A | 0.811997 | 0.587643 | 0.354884 | 3.756243 |
| LOC10027( | 0.811997 | 0.58771 | 0.576086 | 1.438561 |
| AKR7A2 | 0.811997 | 0.587797 | -0.24584 | 4.760404 |
| GRAMD4 | 0.811997 | 0.587804 | -0.2072 | 6.528138 |
| LOC10099( | 0.811997 | 0.587865 | 0.474187 | 2.866651 |
| USP32P2 | 0.812097 | 0.588 | -0.46037 | 2.85244 |
| IL1A | 0.812257 | 0.588178 | -0.45084 | 4.358558 |
| RCL1 | 0.812518 | 0.588465 | 0.528191 | 1.853223 |
| OR2D3 | 0.812518 | 0.588491 | -0.85956 | 1.139192 |
| CMC4 | 0.812847 | 0.588841 | -0.45552 | 1.884584 |
| PTGES | 0.812847 | 0.588852 | 0.500032 | 3.629599 |
| PPTC7 | 0.812882 | 0.58894 | -0.21965 | 5.916445 |
| RBM15B | 0.812906 | 0.589095 | -0.20258 | 6.404295 |
| HIST3H2BB | 0.812906 | 0.589103 | 0.440576 | 2.772545 |
| C16orf46 | 0.812906 | 0.589143 | 0.726846 | 1.069071 |
| LOC10272: | 0.812911 | 0.589209 | -0.39968 | 2.784716 |
| STBD1 | 0.813093 | 0.589418 | 0.434415 | 1.707284 |
| PATL1 | 0.813093 | 0.589465 | 0.194637 | 6.789883 |
| SNORA7B | 0.81332 | 0.589691 | 0.276957 | 5.431746 |
| MASP2 | 0.813398 | 0.58981 | 0.395659 | 3.490105 |
| ZNF665 | 0.813573 | 0.590023 | 0.564477 | 1.91899 |
| LOC10012( | 0.813573 | 0.590076 | -0.46575 | 2.07021 |
| RASAL2 | 0.813573 | 0.590123 | -0.28158 | 4.216029 |
| TBC1D5 | 0.813573 | 0.590185 | -0.34246 | 3.277294 |
| LOC10260( | 0.813819 | 0.590425 | -0.51885 | 1.878983 |
| ANO2 | 0.813828 | 0.590499 | 0.816612 | 0.883185 |
| PRPF19 | 0.813828 | 0.59057 | -0.21026 | 6.24049 |
| SELENOW | 0.813828 | 0.590618 | 0.209873 | 6.109996 |
| DLG5 | 0.813844 | 0.590722 | -0.32128 | 4.664141 |
| METTL14 | 0.813844 | 0.590753 | 0.200634 | 5.415261 |
| SIN3B | 0.81391 | 0.590863 | 0.487564 | 2.342255 |
| CLDN16 | 0.81405 | 0.591027 | -0.82276 | 1.359393 |
| CFI | 0.814054 | 0.591092 | 0.36696 | 4.072411 |
| ACADM | 0.814157 | 0.591228 | 0.8108 | 0.491023 |
| PLEKHG6 | 0.814379 | 0.591452 | -0.64781 | 0.991989 |
| LEPROT | 0.814406 | 0.591547 | 0.346995 | 2.747528 |
| ZCCHC4 | 0.814406 | 0.591596 | 0.318208 | 3.953393 |
| RAPGEF3 | 0.814424 | 0.591794 | -0.72791 | 0.807011 |
| RAI14 | 0.814424 | 0.591795 | 0.484732 | 1.862548 |
| ANKRD55 | 0.814424 | 0.591795 | -0.74228 | 1.100183 |
| AURKAP1 | 0.814978 | 0.592259 | 0.381201 | 2.504383 |
| PLCXD3 | 0.815161 | 0.59249 | -0.67293 | 2.765224 |
| SLC25A20 | 0.815161 | 0.592517 | 0.295239 | 3.466565 |
| NAIP | 0.815361 | 0.592763 | 1.266275 | 0.655927 |

| | | | | |
|---|---|---|---|---|
| ZMYM6 | 0.815361 | 0.592831 | 0.201402 | 5.870693 |
| CCDC144B | 0.815361 | 0.592849 | 0.31295 | 6.506479 |
| HSPA5 | 0.815569 | 0.593106 | 0.262104 | 9.235881 |
| LOC101929 | 0.815569 | 0.593124 | 0.641189 | 1.497155 |
| LOC102723 | 0.815654 | 0.593248 | -0.66673 | 1.205516 |
| SGPP1 | 0.815865 | 0.593478 | 0.295509 | 3.88258 |
| LOC101929 | 0.815865 | 0.593526 | -0.67622 | 1.082918 |
| MCAT | 0.8159 | 0.593614 | -0.62232 | 0.856414 |
| PPP2CB | 0.815989 | 0.593876 | -0.21787 | 6.893066 |
| ST3GAL3 | 0.815989 | 0.59397 | 0.727107 | 1.214485 |
| MPP5 | 0.815989 | 0.593973 | -0.54215 | 1.908657 |
| RAB1A | 0.815989 | 0.59401 | -0.22288 | 4.78056 |
| PGAP2 | 0.815989 | 0.594022 | -0.67545 | 0.386125 |
| LOC100288 | 0.815989 | 0.594051 | -0.30388 | 4.044938 |
| SNORD1C | 0.816158 | 0.594249 | -0.27347 | 4.45257 |
| SIRT4 | 0.816158 | 0.594299 | 0.796738 | 0.740287 |
| LOC100129 | 0.816317 | 0.594477 | 0.582487 | 1.508767 |
| FBP2 | 0.816508 | 0.594678 | 0.852923 | 1.207926 |
| SLC7A8 | 0.816974 | 0.59508 | -0.32102 | 4.374232 |
| STX11 | 0.816984 | 0.59515 | -0.42128 | 3.585838 |
| SOX17 | 0.817104 | 0.595299 | -0.64984 | 1.143585 |
| SKAP2 | 0.817217 | 0.595444 | 0.325003 | 2.829314 |
| CALML6 | 0.817608 | 0.595791 | -0.68734 | 0.687096 |
| RHOT1 | 0.817674 | 0.595901 | -0.90483 | 0.424888 |
| FBXL8 | 0.817694 | 0.595978 | 0.346554 | 2.475143 |
| GTF2F2 | 0.817908 | 0.596209 | -0.23195 | 5.423187 |
| ANXA10 | 0.817908 | 0.596258 | 0.813054 | 4.004035 |
| UBE2I | 0.818114 | 0.596472 | -0.69109 | 1.071607 |
| GPIHBP1 | 0.81812 | 0.596538 | -0.64478 | 0.968775 |
| BMP7-AS1 | 0.818307 | 0.596737 | -0.62165 | 1.744699 |
| DHTKD1 | 0.818678 | 0.597081 | -0.211 | 5.737286 |
| RAMP2-AS | 0.818678 | 0.597132 | -1.10066 | 0.467965 |
| RARS2 | 0.818894 | 0.597382 | 0.184346 | 6.003964 |
| MPV17 | 0.818894 | 0.597436 | 0.262727 | 4.151182 |
| ZNF343 | 0.818894 | 0.597477 | 0.612116 | 1.446021 |
| ZNF862 | 0.818914 | 0.597554 | 0.237935 | 4.858538 |
| MTUS1 | 0.819055 | 0.597756 | 0.392591 | 3.524911 |
| HIRA | 0.819055 | 0.597782 | 0.203523 | 6.12971 |
| ERI2 | 0.819258 | 0.597992 | -0.29334 | 3.995689 |
| ANKRD17 | 0.81928 | 0.598182 | 0.247197 | 4.404523 |
| TMEM50B | 0.81928 | 0.598186 | 0.638285 | 1.200604 |
| SKA2 | 0.81928 | 0.598196 | 0.724695 | 0.410348 |
| FRMPD1 | 0.819536 | 0.598445 | -0.75499 | 1.317536 |
| SH3D21 | 0.819661 | 0.598598 | -0.52262 | 1.849103 |
| ARHGEF16 | 0.819676 | 0.598672 | -0.22289 | 5.71327 |

| | | | | |
|---|---|---|---|---|
| C15orf39 | 0.81973 | 0.598799 | -0.22587 | 5.350424 |
| ZNF512 | 0.81973 | 0.598837 | -0.69132 | 0.494519 |
| NSMCE2 | 0.819765 | 0.598924 | 0.282516 | 4.227592 |
| GMPR | 0.820121 | 0.599305 | 0.537452 | 3.42906 |
| C8orf76 | 0.820121 | 0.599463 | 0.548046 | 1.38115 |
| MAP4 | 0.820121 | 0.599517 | -0.30232 | 4.264022 |
| USP24 | 0.820121 | 0.599544 | 0.192719 | 8.216079 |
| SPICE1 | 0.820121 | 0.599573 | 0.192299 | 6.039161 |
| LINC01372 | 0.820121 | 0.599594 | 0.589439 | 0.649026 |
| SNORD19B | 0.820121 | 0.599622 | 0.615291 | 0.639479 |
| ZNF14 | 0.820394 | 0.599898 | 0.387737 | 3.444874 |
| ASF1A | 0.820394 | 0.599946 | 0.214164 | 4.804711 |
| ZNF490 | 0.820588 | 0.600151 | 0.22786 | 4.857815 |
| POLR2E | 0.82067 | 0.600274 | 0.542352 | 0.942353 |
| LPCAT3 | 0.820918 | 0.60055 | 0.211203 | 5.576708 |
| ATOH8 | 0.820918 | 0.60058 | 0.580197 | 2.306922 |
| SLC35A4 | 0.821007 | 0.600722 | -0.22421 | 6.009419 |
| CHADL | 0.821007 | 0.6008 | 0.548376 | 1.862534 |
| DNM1P46 | 0.821007 | 0.60088 | 0.535167 | 1.851032 |
| REST | 0.821007 | 0.600896 | -0.4738 | 1.618725 |
| TUBG2 | 0.821016 | 0.600964 | 0.266784 | 4.24899 |
| RTN4R | 0.821027 | 0.601035 | 0.546758 | 1.305074 |
| UBTD2 | 0.82104 | 0.601107 | 0.202199 | 5.105182 |
| LOC101928 | 0.821352 | 0.60142 | 0.570303 | 1.335763 |
| ZNF691 | 0.821352 | 0.601547 | 0.745931 | 1.20627 |
| ZFP82 | 0.821352 | 0.601561 | 0.484776 | 1.718473 |
| BPTF | 0.821352 | 0.601627 | 0.2598 | 4.109433 |
| TCF7L1 | 0.821352 | 0.601658 | 0.251894 | 4.391287 |
| BMPR1B | 0.821352 | 0.601711 | 0.770335 | 0.79561 |
| TAF6L | 0.821461 | 0.601854 | 0.317352 | 3.782645 |
| ZNHIT1 | 0.821518 | 0.602012 | -0.21163 | 4.948997 |
| MRGPRF-A | 0.821518 | 0.602164 | -0.65561 | 0.642151 |
| FBXO39 | 0.821518 | 0.602183 | 0.608805 | 0.962597 |
| ATP6V1B2 | 0.821518 | 0.6022 | -0.20456 | 6.482242 |
| NAP1L1 | 0.821518 | 0.602209 | 0.308622 | 3.403457 |
| MCOLN1 | 0.821556 | 0.602299 | -0.24852 | 3.868717 |
| ZNF33A | 0.821668 | 0.602444 | 0.382528 | 2.899093 |
| MTERF4 | 0.821783 | 0.602626 | -0.53922 | 1.725332 |
| COLEC10 | 0.821783 | 0.602712 | -0.84496 | 0.928875 |
| PANK4 | 0.821783 | 0.602787 | -0.59139 | 0.520452 |
| PP2D1 | 0.821783 | 0.602884 | -0.45139 | 2.359715 |
| ILF3-DT | 0.821783 | 0.603006 | 0.38434 | 3.366636 |
| STK17A | 0.821783 | 0.603013 | -0.19765 | 6.263986 |
| LOC729966 | 0.821783 | 0.603079 | 0.559095 | 1.432297 |
| ZNF107 | 0.821783 | 0.603084 | -0.83655 | 0.864968 |

| | | | | |
|---|---|---|---|---|
| WDR43 | 0.821783 | 0.603092 | -0.21354 | 7.238749 |
| TYW1B | 0.821862 | 0.603212 | 0.247384 | 4.297063 |
| CERK | 0.821957 | 0.603377 | -0.19206 | 6.218364 |
| TBC1D15 | 0.821957 | 0.603408 | 0.776029 | 0.795318 |
| KDELC2 | 0.822312 | 0.603793 | 0.213039 | 5.649868 |
| NSMAF | 0.822312 | 0.603843 | 0.55137 | 0.910241 |
| MRPL23 | 0.822312 | 0.603867 | -0.30904 | 3.633914 |
| CUEDC2 | 0.822312 | 0.603931 | 0.255422 | 4.3496 |
| LINC00927 | 0.822312 | 0.603981 | -1.23045 | 0.771061 |
| HIST3H2A | 0.822347 | 0.604138 | 0.482893 | 2.057309 |
| ADD1 | 0.822347 | 0.604208 | -0.37211 | 2.741035 |
| FAM184B | 0.822347 | 0.60422 | -0.29313 | 3.780261 |
| ZFYVE26 | 0.822347 | 0.604258 | -0.20269 | 6.402029 |
| NAP1L5 | 0.822521 | 0.604448 | 0.277981 | 3.777111 |
| ICAM5 | 0.822634 | 0.604594 | -0.5818 | 1.558079 |
| IRX1 | 0.82276 | 0.604749 | -0.89809 | 0.424907 |
| MED12L | 0.822887 | 0.605043 | 0.456011 | 2.201287 |
| PPM1G | 0.822887 | 0.605073 | -0.19767 | 7.287573 |
| ITIH3 | 0.822887 | 0.605078 | 0.787973 | 1.624626 |
| WDR81 | 0.822887 | 0.605167 | 0.373911 | 3.627727 |
| SESN1 | 0.822887 | 0.605236 | 0.332064 | 2.992397 |
| BTN3A1 | 0.822887 | 0.605239 | 0.6971 | 0.649276 |
| PSD2 | 0.822887 | 0.605292 | 0.645969 | 1.121602 |
| SPG11 | 0.822887 | 0.605344 | 0.485202 | 1.980964 |
| DDX6 | 0.823208 | 0.605643 | 0.654283 | 1.066238 |
| TMEM184B | 0.823217 | 0.605721 | 0.538765 | 1.782539 |
| FAM72D | 0.823217 | 0.605775 | 0.526501 | 1.466447 |
| ARRDC2 | 0.823259 | 0.605869 | -0.72866 | 0.429743 |
| LOC10012[illegible] | 0.8234 | 0.606035 | 0.691271 | 1.197769 |
| MIR320B2 | 0.823433 | 0.606233 | 0.614102 | 1.593685 |
| SNORD29 | 0.823433 | 0.606242 | -0.48348 | 1.398867 |
| GRTP1 | 0.823433 | 0.606248 | -0.34617 | 2.234673 |
| EGOT | 0.82374 | 0.606537 | -0.47387 | 2.417882 |
| PPM1D | 0.823777 | 0.606627 | 0.200747 | 5.035978 |
| VPS13A | 0.823837 | 0.606792 | -0.35949 | 3.545236 |
| TFDP2 | 0.823837 | 0.606797 | -0.649 | 0.724139 |
| EID2 | 0.823844 | 0.606864 | 0.272334 | 3.452402 |
| SNORA41 | 0.823953 | 0.60712 | 0.434329 | 2.516936 |
| CASP2 | 0.823953 | 0.607122 | -0.68781 | 1.1381 |
| ERGIC1 | 0.823953 | 0.607133 | 0.205807 | 7.44591 |
| UBXN4 | 0.824025 | 0.607249 | 0.176335 | 8.367645 |
| MAP3K7CL | 0.824217 | 0.607494 | -0.67214 | 1.385349 |
| LOC10106[illegible] | 0.824217 | 0.607516 | 0.664465 | 0.804368 |
| CEP350 | 0.824361 | 0.607786 | -0.18003 | 8.575793 |
| CHCHD1 | 0.824361 | 0.607787 | -0.26534 | 4.070244 |

| | | | | |
|---|---|---|---|---|
| NFATC3 | 0.824361 | 0.607905 | 0.684233 | 0.476816 |
| MIR491 | 0.824361 | 0.607928 | -0.83162 | 0.496972 |
| ACAD8 | 0.824361 | 0.607936 | 0.240704 | 4.23844 |
| ACSM6 | 0.82439 | 0.608025 | 0.582293 | 1.96546 |
| SNX29P1 | 0.82439 | 0.608083 | -0.48865 | 1.736682 |
| FNBP1L | 0.82448 | 0.608213 | 0.247483 | 4.471583 |
| EIPR1 | 0.824625 | 0.608383 | 0.227454 | 4.284736 |
| LEKR1 | 0.824646 | 0.608602 | 0.520375 | 1.441033 |
| MIPEP | 0.824646 | 0.608641 | -0.20656 | 4.79517 |
| TPBG | 0.824646 | 0.608644 | 0.57011 | 1.002856 |
| NPHP4 | 0.824646 | 0.608731 | 0.587017 | 1.400761 |
| MAN1A2 | 0.824646 | 0.60874 | -0.19444 | 7.953523 |
| TVP23B | 0.824646 | 0.608843 | 0.414417 | 1.839755 |
| SLC25A45 | 0.824646 | 0.608899 | 0.762828 | 0.428595 |
| LOC101927 | 0.824646 | 0.608901 | 0.432996 | 3.176577 |
| DDX43 | 0.824733 | 0.609115 | -0.47469 | 2.102642 |
| MIR3064 | 0.824733 | 0.609118 | -0.3192 | 3.328557 |
| SLC25A33 | 0.824733 | 0.609154 | 0.473119 | 1.952074 |
| ANKRA2 | 0.824811 | 0.609274 | -0.21822 | 5.236465 |
| ZNF702P | 0.824811 | 0.609337 | 0.632687 | 1.563109 |
| SAMD11 | 0.824919 | 0.60954 | 0.568728 | 2.636254 |
| LINC00173 | 0.824919 | 0.609542 | -0.57868 | 1.619185 |
| TSC2 | 0.825254 | 0.609853 | -0.74292 | 0.627442 |
| TTC24 | 0.825655 | 0.610212 | 0.871715 | 0.484012 |
| MRPL3 | 0.82571 | 0.610316 | 0.192964 | 6.258425 |
| LOC644285 | 0.826205 | 0.610814 | 0.225964 | 5.194365 |
| CBX4 | 0.826205 | 0.610899 | -0.1946 | 6.35544 |
| LOC645513 | 0.826205 | 0.610922 | 0.237195 | 3.985867 |
| MIR125B1 | 0.826205 | 0.610933 | 0.898708 | 0.582213 |
| PLEKHH1 | 0.826289 | 0.61106 | 0.231912 | 5.697492 |
| EIF2B5 | 0.826289 | 0.611122 | 0.18699 | 6.492187 |
| HOXA5 | 0.826541 | 0.611401 | 0.465195 | 3.017553 |
| LOC100506 | 0.826541 | 0.611496 | -0.30627 | 3.375874 |
| SND1-IT1 | 0.826541 | 0.611532 | 0.268649 | 3.992356 |
| MIR616 | 0.826541 | 0.611639 | -0.75035 | 0.927111 |
| CHIAP2 | 0.826541 | 0.611674 | -0.97243 | 1.023721 |
| ALOX15 | 0.826541 | 0.611686 | 0.654114 | 1.081325 |
| SLC25A29 | 0.826577 | 0.611775 | 0.534471 | 1.286509 |
| HPGDS | 0.826898 | 0.612112 | 0.612229 | 0.947753 |
| ALKBH5 | 0.826898 | 0.612168 | 0.182634 | 6.539786 |
| NEXMIF | 0.826898 | 0.612236 | 0.759775 | 2.144687 |
| OR4M2 | 0.826898 | 0.612265 | -1.04254 | 0.556127 |
| EBF2 | 0.826981 | 0.61239 | 0.728256 | 1.931001 |
| C2orf69 | 0.827011 | 0.612474 | -0.23951 | 4.712973 |
| GOLGA2P5 | 0.827017 | 0.612542 | 0.222031 | 4.124412 |

| | | | | |
|---|---|---|---|---|
| NARS2 | 0.827246 | 0.612775 | 0.58648 | 1.08493 |
| PART1 | 0.827309 | 0.612884 | -0.49624 | 2.375422 |
| C9orf64 | 0.827483 | 0.613076 | -0.56045 | 1.804678 |
| MIR3682 | 0.827494 | 0.613147 | 0.843607 | 0.575729 |
| LOC10192{ | 0.827619 | 0.613303 | 0.492364 | 2.015814 |
| OSBPL2 | 0.82763 | 0.613374 | -0.57416 | 1.177809 |
| TERF2 | 0.827667 | 0.613499 | 0.199069 | 5.417908 |
| PI4KAP2 | 0.827667 | 0.613528 | 0.219072 | 5.087143 |
| PRPF18 | 0.827694 | 0.613612 | 0.178498 | 5.790601 |
| LRCH4 | 0.827749 | 0.613749 | -0.2652 | 3.701781 |
| TULP2 | 0.827749 | 0.613778 | 0.701799 | 1.172272 |
| ADGRL1 | 0.827868 | 0.613929 | 0.625281 | 0.848853 |
| NCS1 | 0.82787 | 0.613994 | 0.64468 | 0.771252 |
| LINC00680 | 0.827973 | 0.614134 | 0.606839 | 0.905234 |
| MTMR11 | 0.828141 | 0.614321 | 0.38748 | 1.85563 |
| DIABLO | 0.828232 | 0.61452 | 0.487568 | 0.986345 |
| TIMM50 | 0.828232 | 0.614546 | 0.201567 | 4.713785 |
| LHX4 | 0.828232 | 0.614602 | -0.53829 | 1.668384 |
| STAG3L5P | 0.828232 | 0.614642 | 0.434404 | 2.670799 |
| NDRG2 | 0.828283 | 0.614758 | -0.57437 | 1.462521 |
| UBE2M | 0.828283 | 0.614805 | -0.18021 | 5.711299 |
| ANKRD16 | 0.828525 | 0.615089 | 0.682827 | 0.733746 |
| TAF1C | 0.828525 | 0.615111 | 0.647189 | 0.965362 |
| MCTS2P | 0.828559 | 0.6152 | -0.2444 | 3.702237 |
| MIR5699 | 0.828754 | 0.615408 | 0.540866 | 0.647702 |
| C16orf58 | 0.828773 | 0.615485 | 0.194709 | 5.82235 |
| TMC3-AS1 | 0.828818 | 0.615581 | 0.740066 | 1.055222 |
| STAM2 | 0.828853 | 0.615675 | -0.18026 | 6.470294 |
| FAM32A | 0.828853 | 0.615734 | 0.173542 | 6.104008 |
| LTO1 | 0.829213 | 0.616125 | 0.252289 | 4.63423 |
| SMIM4 | 0.829213 | 0.616127 | -0.30076 | 3.307742 |
| MC1R | 0.829391 | 0.616323 | 0.365053 | 2.950332 |
| SNORD144 | 0.829466 | 0.616442 | 0.527003 | 1.809156 |
| BEX1 | 0.829494 | 0.61659 | 0.978042 | 0.452713 |
| DYNC1H1 | 0.829494 | 0.616642 | -0.18094 | 10.13046 |
| YDJC | 0.829494 | 0.616653 | 0.337072 | 3.374606 |
| TSPAN31 | 0.829607 | 0.616923 | -0.22427 | 4.478173 |
| PUM3 | 0.829607 | 0.61701 | -0.19069 | 6.837555 |
| PRRC2C | 0.829607 | 0.617046 | 0.190058 | 10.71702 |
| OVCH1 | 0.829607 | 0.617059 | 0.774428 | 1.452426 |
| GPLD1 | 0.829607 | 0.617115 | -0.33492 | 3.229264 |
| PXMP4 | 0.829607 | 0.617116 | 0.656301 | 0.430113 |
| ZC3HAV1 | 0.829633 | 0.617228 | 0.186032 | 6.56895 |
| LOC72960: | 0.829633 | 0.617262 | -0.2378 | 4.003776 |
| PUS1 | 0.829691 | 0.617382 | -0.40346 | 2.348997 |

| | | | | |
|---|---|---|---|---|
| ARHGDIG | 0.829691 | 0.617432 | -0.81223 | 1.229192 |
| HEXA-AS1 | 0.829927 | 0.61767 | 0.618014 | 1.460497 |
| LOC10013( | 0.830029 | 0.617826 | 0.668034 | 1.088131 |
| SNORD124 | 0.830029 | 0.617873 | 0.795032 | 0.583377 |
| GPR171 | 0.830431 | 0.618302 | 0.560882 | 1.18463 |
| SMC3 | 0.830431 | 0.618328 | 0.199488 | 7.670798 |
| CMTM3 | 0.830431 | 0.618397 | 0.599144 | 1.343778 |
| PACRGL | 0.830431 | 0.618452 | -0.66194 | 0.753794 |
| MAB21L1 | 0.830431 | 0.618488 | -0.679 | 1.478992 |
| SNRNP35 | 0.830442 | 0.61856 | 0.538339 | 1.783361 |
| SNORA70G | 0.830457 | 0.618634 | 0.462135 | 2.562934 |
| RPL21P44 | 0.830459 | 0.61882 | -0.40479 | 2.685601 |
| AGAP1 | 0.830459 | 0.618827 | -0.49039 | 1.636871 |
| DOT1L | 0.830459 | 0.618838 | -0.19304 | 6.935441 |
| ELP4 | 0.830459 | 0.618947 | 0.669756 | 0.968551 |
| SHF | 0.830459 | 0.618981 | 0.482351 | 2.698326 |
| COX20 | 0.830459 | 0.619033 | 0.407619 | 2.053722 |
| DCST2 | 0.830459 | 0.619079 | 0.458384 | 2.094656 |
| CEACAM1( | 0.830497 | 0.61917 | 0.674425 | 0.632738 |
| ZNF569 | 0.830533 | 0.619263 | 0.412396 | 1.963265 |
| MAP2K4P1 | 0.830533 | 0.619324 | 0.478981 | 1.520297 |
| CYP2B6 | 0.83056 | 0.619502 | 0.959929 | 0.750691 |
| NQO2 | 0.83056 | 0.619515 | 0.685284 | 0.523709 |
| GTF2IP20 | 0.83056 | 0.619541 | -0.2633 | 3.677361 |
| XPA | 0.83056 | 0.61965 | 0.556347 | 1.16531 |
| SPTY2D1O! | 0.83056 | 0.61968 | -0.57758 | 1.801894 |
| HIST1H3D | 0.83056 | 0.619756 | 0.328245 | 4.311857 |
| TRADD | 0.83056 | 0.619837 | -0.24417 | 3.719647 |
| CPEB2-DT | 0.83056 | 0.61985 | 0.633044 | 1.038459 |
| NUGGC | 0.8307 | 0.620023 | 0.521557 | 3.205388 |
| FBXL7 | 0.8307 | 0.620081 | 0.541054 | 1.36491 |
| PI4KAP1 | 0.8312 | 0.62056 | 0.25384 | 4.994473 |
| ZBTB41 | 0.8312 | 0.620581 | 0.202076 | 6.247749 |
| PSMD4 | 0.831576 | 0.620994 | 0.175572 | 6.872747 |
| LPXN | 0.831576 | 0.621058 | 0.784188 | 0.38185 |
| HNRNPAB | 0.831576 | 0.621063 | 0.450082 | 1.064229 |
| SNTA1 | 0.831576 | 0.62123 | 0.277763 | 3.713774 |
| NBR1 | 0.831576 | 0.621294 | -0.60476 | 0.402908 |
| LINC02541 | 0.831576 | 0.621302 | 0.703853 | 1.290278 |
| NODAL | 0.831576 | 0.621306 | 0.547896 | 1.482976 |
| PCF11 | 0.831647 | 0.621435 | -0.17788 | 7.483761 |
| KIAA0895 | 0.831647 | 0.621485 | -0.6259 | 1.273031 |
| MTOR | 0.831778 | 0.621695 | -0.17708 | 7.317299 |
| LSM11 | 0.831778 | 0.621812 | 0.263522 | 4.042301 |
| C17orf100 | 0.831778 | 0.621822 | -0.43392 | 2.285774 |

| | | | | |
|---|---|---|---|---|
| ESRRB | 0.831778 | 0.621914 | -0.77715 | 2.150199 |
| LAMB3 | 0.831778 | 0.621942 | 0.529344 | 3.336179 |
| HNRNPA2E | 0.831778 | 0.621963 | -0.51961 | 1.778635 |
| PAXBP1-AS | 0.831835 | 0.622069 | 0.244537 | 3.620536 |
| LIPN | 0.831841 | 0.622149 | 0.652628 | 0.963913 |
| CTTN | 0.831841 | 0.622324 | 0.335674 | 3.008405 |
| FBXL16 | 0.831841 | 0.622333 | -0.29218 | 5.699092 |
| SDHAF2 | 0.831841 | 0.622379 | 0.24514 | 4.267359 |
| CHRNE | 0.831841 | 0.622515 | 0.495044 | 1.991324 |
| KCNH7 | 0.831841 | 0.622536 | 0.915159 | 0.718924 |
| ZIK1 | 0.831841 | 0.622563 | -0.5698 | 1.845733 |
| POU5F2 | 0.831841 | 0.622581 | 0.339323 | 2.61049 |
| PTP4A1 | 0.831892 | 0.622682 | 0.258408 | 8.249487 |
| OMA1 | 0.831946 | 0.622786 | -0.19843 | 5.264458 |
| ABCB11 | 0.831947 | 0.622851 | -1.07707 | 1.089266 |
| WDR34 | 0.832034 | 0.623003 | 0.208759 | 4.760169 |
| TXLNG | 0.832034 | 0.623043 | 0.381796 | 3.044353 |
| HOXD9 | 0.83211 | 0.623194 | 0.660497 | 1.151028 |
| SCD5 | 0.83211 | 0.623233 | -0.49813 | 3.00385 |
| MTRNR2L8 | 0.83211 | 0.62329 | -0.40698 | 5.874524 |
| FPGS | 0.832455 | 0.623612 | 0.470537 | 1.561659 |
| ZCRB1 | 0.832458 | 0.623778 | -0.1838 | 5.780049 |
| ATXN7 | 0.832458 | 0.62378 | 0.270106 | 2.894176 |
| GRIK1-AS2 | 0.832458 | 0.623821 | 0.686199 | 0.61499 |
| RHBDL3 | 0.832458 | 0.623897 | -0.67474 | 1.326138 |
| PPA1 | 0.832458 | 0.623986 | -0.20459 | 6.553007 |
| PPIG | 0.832458 | 0.623995 | 0.17338 | 8.334014 |
| SNORD43 | 0.832546 | 0.624214 | 0.521888 | 1.709842 |
| SUPT16H | 0.832546 | 0.624263 | 0.189402 | 7.757232 |
| CAMKK1 | 0.832546 | 0.624295 | 0.55695 | 1.2367 |
| DNAJC21 | 0.832546 | 0.624314 | -0.43002 | 1.676324 |
| MGC12916 | 0.833203 | 0.624871 | 0.361026 | 2.625244 |
| PREPL | 0.83348 | 0.625142 | -0.44081 | 2.231146 |
| RFX6 | 0.833597 | 0.625293 | -0.8981 | 0.500187 |
| KCNJ11 | 0.833766 | 0.625484 | -0.69588 | 0.673961 |
| KCTD4 | 0.833766 | 0.625547 | -0.44266 | 2.832319 |
| NCKAP1L | 0.833918 | 0.625754 | 0.80344 | 0.894608 |
| ADGRF3 | 0.833918 | 0.625788 | -0.44631 | 2.075104 |
| NEK7 | 0.834017 | 0.62604 | 0.189694 | 6.832844 |
| RILPL1 | 0.834017 | 0.626136 | 0.22605 | 4.351066 |
| LOC100288 | 0.834017 | 0.626269 | -0.49659 | 1.419363 |
| TSC22D1-A | 0.834017 | 0.626285 | 0.401488 | 2.564937 |
| LRIG3 | 0.834017 | 0.626324 | -0.40354 | 2.825584 |
| EEF1AKMT | 0.834017 | 0.62635 | 0.588961 | 0.801467 |
| FAM45A | 0.834017 | 0.626382 | -0.59686 | 0.799594 |

| | | | | |
|---|---|---|---|---|
| RNASEH2C | 0.834017 | 0.626385 | 0.240324 | 4.222084 |
| ZNF649 | 0.834017 | 0.626435 | 0.236741 | 3.831972 |
| KDR | 0.834107 | 0.626565 | 0.222194 | 4.906398 |
| MLLT11 | 0.834126 | 0.626643 | 0.383536 | 2.684098 |
| SLFN12L | 0.834245 | 0.626834 | 0.431498 | 2.724234 |
| PTEN | 0.834245 | 0.62686 | -0.33753 | 2.674238 |
| VSIR | 0.834491 | 0.627109 | 0.187179 | 5.78149 |
| VASN | 0.83472 | 0.627344 | -0.51499 | 0.612777 |
| SSH1 | 0.834952 | 0.627582 | 0.181211 | 6.116391 |
| PXN-AS1 | 0.834952 | 0.627646 | 0.64997 | 0.918593 |
| TMEM203 | 0.835023 | 0.627763 | -0.29572 | 3.635541 |
| MIR7849 | 0.835031 | 0.627881 | 0.699844 | 0.5629 |
| DUSP23 | 0.835031 | 0.627896 | 0.283395 | 3.664482 |
| TMEM182 | 0.835047 | 0.627972 | 0.364985 | 2.5381 |
| L3MBTL1 | 0.835261 | 0.628196 | -0.58848 | 0.825358 |
| LINC00649 | 0.835541 | 0.62847 | -0.26159 | 3.606132 |
| IQCG | 0.835714 | 0.628664 | 0.611273 | 0.602874 |
| SLC9C1 | 0.835807 | 0.628861 | 0.43587 | 2.464552 |
| CALCOCO2 | 0.835807 | 0.628862 | 0.498475 | 1.956737 |
| SHPRH | 0.835829 | 0.628942 | 0.43201 | 1.70438 |
| PLCE1-AS1 | 0.836076 | 0.629289 | 0.737565 | 0.943761 |
| ABCB8 | 0.836076 | 0.629296 | 0.551572 | 1.201658 |
| LINC01252 | 0.836076 | 0.629319 | 0.451676 | 1.641002 |
| SLC5A1 | 0.836462 | 0.6297 | 0.5738 | 1.202589 |
| SERTAD4-A | 0.836462 | 0.629762 | 0.707207 | 0.932418 |
| BLCAP | 0.836462 | 0.629801 | -0.21267 | 4.394906 |
| MAIP1 | 0.836482 | 0.629932 | -0.2367 | 3.944251 |
| ERCC2 | 0.836482 | 0.630047 | -0.28828 | 3.57618 |
| QRSL1 | 0.836482 | 0.630059 | 0.235176 | 4.701996 |
| RAD50 | 0.836482 | 0.630071 | -0.18262 | 7.800188 |
| STXBP5 | 0.836618 | 0.630237 | -0.65545 | 0.793459 |
| TRIM14 | 0.836677 | 0.630345 | -0.193 | 5.802022 |
| RAPGEF1 | 0.836817 | 0.630514 | 0.346803 | 3.100518 |
| TTLL7 | 0.836819 | 0.63059 | 0.364981 | 4.383144 |
| PABPN1 | 0.836819 | 0.630644 | -0.2432 | 5.250116 |
| TMED10P1 | 0.836835 | 0.630719 | 0.2996 | 3.261843 |
| TRAFD1 | 0.836849 | 0.630793 | 0.643352 | 0.3458 |
| SLMAP | 0.83691 | 0.630909 | -0.31654 | 2.6425 |
| NOP53-AS1 | 0.83691 | 0.631101 | 0.510154 | 1.217432 |
| PTPN4 | 0.83691 | 0.631145 | 0.191139 | 5.955741 |
| ZNF598 | 0.83691 | 0.631197 | 0.206147 | 6.221296 |
| FOXJ2 | 0.83691 | 0.631279 | 0.17186 | 6.024321 |
| DNAJB14 | 0.83691 | 0.631285 | 0.603415 | 0.72071 |
| SON | 0.83691 | 0.631291 | 0.198873 | 5.096939 |
| KCNJ9 | 0.83691 | 0.631395 | 0.55477 | 0.693396 |

| | | | | |
|---|---|---|---|---|
| TRIM27 | 0.83691 | 0.631485 | 0.333623 | 3.231232 |
| ACAD10 | 0.83691 | 0.631507 | 0.551177 | 1.077081 |
| PNN | 0.83691 | 0.631546 | -0.17821 | 9.367204 |
| INSYN2 | 0.83691 | 0.631605 | 0.243232 | 4.272933 |
| GLIDR | 0.837011 | 0.631745 | 0.61291 | 0.664456 |
| SLC27A5 | 0.837266 | 0.632001 | 0.406087 | 2.078108 |
| HIST1H4C | 0.837354 | 0.632131 | 0.214789 | 5.355455 |
| YAP1 | 0.837522 | 0.63233 | -0.62386 | 1.158816 |
| MTA3 | 0.837522 | 0.632386 | 0.476544 | 1.361825 |
| STOM | 0.837555 | 0.63248 | -0.6481 | 0.643211 |
| RANBP2 | 0.837555 | 0.632688 | 0.176792 | 9.158395 |
| SMC4 | 0.837555 | 0.63274 | -0.68966 | 1.275836 |
| MIPEPP3 | 0.837555 | 0.632745 | -0.50584 | 1.763798 |
| NOM1 | 0.837555 | 0.632768 | 0.195235 | 6.316758 |
| CHPF2 | 0.837555 | 0.632794 | 0.247984 | 3.977103 |
| BEAN1-AS1 | 0.837578 | 0.632961 | -0.60196 | 1.542761 |
| ARIH1 | 0.837578 | 0.633031 | -0.17268 | 7.384792 |
| BUD13 | 0.837578 | 0.633042 | 0.482483 | 2.219997 |
| AGPS | 0.837578 | 0.633176 | 0.187871 | 6.912532 |
| LINC01268 | 0.837578 | 0.633295 | 0.629939 | 1.16888 |
| NKX2-2 | 0.837578 | 0.633364 | 1.094229 | 1.10128 |
| MIR4256 | 0.837578 | 0.633384 | 0.739976 | 0.479586 |
| CTDSP2 | 0.837578 | 0.633427 | 0.17422 | 7.856775 |
| USP31 | 0.837578 | 0.63343 | 0.197903 | 6.779176 |
| KCNQ1 | 0.837578 | 0.63345 | -0.61555 | 1.940864 |
| GNPAT | 0.837637 | 0.63357 | 0.54478 | 1.189903 |
| ZNF721 | 0.837637 | 0.633642 | 0.183639 | 6.531314 |
| ERVFRD-1 | 0.837637 | 0.633719 | 0.300601 | 3.537069 |
| PLCG1-AS1 | 0.837637 | 0.633796 | -0.52667 | 1.953605 |
| ZMAT2 | 0.837637 | 0.633813 | -0.23139 | 7.536859 |
| SMURF2P1 | 0.837674 | 0.633968 | -0.18779 | 5.247927 |
| PRKAG2-AS | 0.837674 | 0.634039 | 0.668699 | 1.093285 |
| INTS3 | 0.837674 | 0.634046 | 0.270918 | 3.717057 |
| LACC1 | 0.837674 | 0.634096 | -0.6253 | 0.616031 |
| PPIA | 0.837688 | 0.634171 | 0.465003 | 2.345203 |
| LINC00863 | 0.837884 | 0.634429 | 0.427302 | 2.088728 |
| CCDC126 | 0.837884 | 0.634447 | 0.367906 | 3.485809 |
| VAV3 | 0.837907 | 0.634528 | 0.485583 | 3.068213 |
| PPHLN1 | 0.837912 | 0.634596 | 0.537153 | 1.434387 |
| SCARNA8 | 0.837933 | 0.634782 | 0.271861 | 5.402687 |
| INAFM2 | 0.837933 | 0.634834 | -0.25769 | 4.701141 |
| CALR | 0.837933 | 0.634888 | -0.17449 | 9.900903 |
| PRKCE | 0.837933 | 0.634889 | -0.18251 | 4.991145 |
| AASDHPPT | 0.837933 | 0.634931 | 0.19208 | 5.49028 |
| ATRN | 0.837938 | 0.635001 | 0.176947 | 5.657031 |

| | | | | |
|---|---|---|---|---|
| ANKRD39 | 0.837938 | 0.635063 | 0.27533 | 2.843526 |
| RAE1 | 0.838004 | 0.635176 | 0.498647 | 1.207301 |
| CTAGE6 | 0.838067 | 0.635392 | -0.52763 | 1.10142 |
| RTRAF | 0.838067 | 0.635441 | -0.17674 | 6.067521 |
| FAF2 | 0.838067 | 0.635475 | -0.18294 | 6.501189 |
| ADD3-AS1 | 0.838067 | 0.635604 | 0.525771 | 1.325037 |
| GPR55 | 0.838067 | 0.635613 | -0.49111 | 1.236661 |
| GPATCH8 | 0.838067 | 0.635684 | 0.431237 | 2.422787 |
| KDM2B | 0.838067 | 0.635716 | 0.533834 | 1.011578 |
| DYM | 0.838067 | 0.635735 | -0.1839 | 6.083443 |
| LOC10013: | 0.838088 | 0.635815 | 0.399439 | 2.117573 |
| SAXO2 | 0.838346 | 0.636075 | 0.752645 | 0.93788 |
| KLHL29 | 0.838428 | 0.636201 | 0.240028 | 4.614504 |
| MRS2P2 | 0.838467 | 0.636341 | 0.451213 | 2.743345 |
| PLBD1-AS1 | 0.838467 | 0.636358 | 0.562408 | 1.561843 |
| HM13 | 0.838527 | 0.636772 | -0.23442 | 4.694309 |
| IPO9-AS1 | 0.838527 | 0.636847 | 0.661457 | 0.446708 |
| COL20A1 | 0.838527 | 0.636917 | -0.75623 | 1.920799 |
| KIF16B | 0.838527 | 0.637011 | 0.411512 | 2.227507 |
| FAHD2CP | 0.838527 | 0.637018 | 0.634388 | 1.231175 |
| NDUFA8 | 0.838527 | 0.63715 | -0.2691 | 4.306816 |
| HIST1H4F | 0.838527 | 0.637179 | 0.271821 | 4.124342 |
| VPS37C | 0.838527 | 0.637193 | 0.198604 | 5.13603 |
| PITRM1-AS | 0.838527 | 0.637194 | 0.236486 | 4.293917 |
| WHAMM | 0.838527 | 0.637218 | -0.17064 | 5.62526 |
| LOC101927 | 0.838527 | 0.637234 | 0.435217 | 2.460083 |
| FBXW7 | 0.838527 | 0.637242 | -0.25729 | 3.715809 |
| ANKRD63 | 0.838527 | 0.637258 | 1.005176 | 0.598609 |
| KIAA0319L | 0.838527 | 0.637298 | -0.174 | 6.997464 |
| MACROD1 | 0.838622 | 0.637435 | -0.2741 | 3.598407 |
| SART1 | 0.838668 | 0.637533 | 0.189713 | 6.950025 |
| RASA4CP | 0.83868 | 0.637632 | -0.28885 | 3.295945 |
| REXO1L2P | 0.83868 | 0.637671 | -1.04456 | 0.460755 |
| MIR27B | 0.838808 | 0.637945 | 0.35118 | 2.700364 |
| ZFP30 | 0.838808 | 0.638044 | -0.22834 | 4.470745 |
| PCAT1 | 0.838808 | 0.638106 | -0.50229 | 1.696444 |
| HMBOX1 | 0.838808 | 0.638138 | 0.277425 | 3.273753 |
| RPN2 | 0.838808 | 0.638144 | 0.666611 | 0.500853 |
| LOC101926 | 0.838808 | 0.638151 | -0.25136 | 3.498414 |
| BBS4 | 0.839009 | 0.638382 | 0.490529 | 1.525539 |
| SLC6A10P | 0.839009 | 0.638432 | -0.6049 | 2.487138 |
| STIMATE | 0.83905 | 0.638616 | 0.208878 | 4.56234 |
| WNT10A | 0.83905 | 0.638661 | 0.442731 | 3.452141 |
| MGAT1 | 0.83905 | 0.638681 | -0.24372 | 3.629097 |
| ZBTB20-AS | 0.83905 | 0.638769 | 0.739652 | 0.419134 |

| | | | | |
|---|---|---|---|---|
| ZNF256 | 0.83905 | 0.638783 | -0.35178 | 2.874968 |
| ENTR1 | 0.83929 | 0.63903 | 0.592019 | 0.6199 |
| ERH | 0.839296 | 0.639104 | -0.2277 | 5.285344 |
| ZGRF1 | 0.839296 | 0.639162 | 0.221547 | 5.131226 |
| PARTICL | 0.839426 | 0.639368 | -0.51108 | 1.256842 |
| C12orf45 | 0.839426 | 0.639389 | 0.28106 | 3.576918 |
| HS3ST3B1 | 0.83947 | 0.639487 | -0.42585 | 2.255347 |
| LOC10105¢ | 0.839552 | 0.63969 | 0.676587 | 0.76073 |
| NSUN3 | 0.839552 | 0.639699 | 0.434127 | 2.523011 |
| DENND6B | 0.839552 | 0.639768 | 0.330027 | 3.064872 |
| SNORD14E | 0.839552 | 0.639805 | 0.509946 | 2.344907 |
| CCDC183 | 0.839576 | 0.639888 | 0.381254 | 3.070761 |
| C16orf71 | 0.839655 | 0.640058 | 0.603247 | 1.230865 |
| KDELR1 | 0.839655 | 0.640075 | 0.173858 | 7.166457 |
| VCL | 0.839862 | 0.640297 | 0.386151 | 2.332985 |
| CDHR2 | 0.839915 | 0.640402 | -0.49258 | 1.388187 |
| COPG1 | 0.840345 | 0.640794 | 0.182424 | 7.61805 |
| ZNF563 | 0.84074 | 0.64122 | -0.41566 | 2.587515 |
| LOC10192ξ | 0.84074 | 0.641385 | -0.64629 | 0.64053 |
| CARMIL3 | 0.84074 | 0.641387 | 0.333137 | 2.709745 |
| DNAJC9 | 0.84074 | 0.641391 | 0.181356 | 5.5319 |
| OCEL1 | 0.84074 | 0.641415 | 0.349107 | 2.097272 |
| TOMM7 | 0.84098 | 0.641662 | 0.226821 | 6.124991 |
| KCNA4 | 0.841033 | 0.641767 | -0.91142 | 0.451819 |
| BEST1 | 0.841051 | 0.641882 | -0.5464 | 1.558984 |
| MBOAT4 | 0.841051 | 0.641908 | 0.665832 | 1.033461 |
| CCDC168 | 0.841222 | 0.642104 | 0.52181 | 1.602858 |
| MYO18B | 0.841482 | 0.6424 | 0.639845 | 1.717295 |
| PPP1R15B | 0.841482 | 0.64243 | 0.173549 | 7.447373 |
| MEPCE | 0.841552 | 0.642705 | 0.282771 | 2.935688 |
| PPP4R3A | 0.841552 | 0.642755 | -0.19448 | 4.735574 |
| PGAM1P5 | 0.841552 | 0.642774 | -0.63595 | 1.069197 |
| TUBGCP3 | 0.841552 | 0.642802 | 0.500862 | 2.397848 |
| ZHX3 | 0.841552 | 0.642829 | 0.204005 | 6.672564 |
| FANCA | 0.841552 | 0.642919 | 0.496691 | 1.265309 |
| NUCB1 | 0.841552 | 0.642932 | 0.170862 | 7.235097 |
| F2RL3 | 0.842107 | 0.64342 | 0.350577 | 3.567454 |
| FOXD1 | 0.842125 | 0.643522 | -0.38569 | 1.996042 |
| SRSF2 | 0.842125 | 0.643621 | 0.21854 | 4.252042 |
| NANOS1 | 0.842125 | 0.643692 | 0.434499 | 1.683802 |
| MIR17HG | 0.842125 | 0.643751 | -0.38033 | 3.432425 |
| LOC101927 | 0.842125 | 0.643755 | -0.49479 | 2.580483 |
| NR1D2 | 0.842129 | 0.643883 | 0.693277 | 0.752907 |
| HGS | 0.842129 | 0.643997 | -0.16565 | 7.135589 |
| MED9 | 0.842129 | 0.644022 | 0.248211 | 3.77177 |

| | | | | |
|---|---|---|---|---|
| FAM120A | 0.842129 | 0.644031 | 0.233473 | 3.488014 |
| C1orf174 | 0.842129 | 0.64409 | 0.218505 | 4.128952 |
| SEC23IP | 0.842129 | 0.644166 | 0.330828 | 3.581862 |
| DAB2IP | 0.842129 | 0.644207 | -0.17594 | 5.431562 |
| FGD5-AS1 | 0.842223 | 0.644383 | 0.507429 | 1.398051 |
| AKAP11 | 0.842223 | 0.644408 | 0.171129 | 7.801445 |
| KCNJ10 | 0.842285 | 0.64457 | 0.463613 | 1.954325 |
| GALNT18 | 0.842285 | 0.644607 | -0.22798 | 4.090719 |
| PIGS | 0.842285 | 0.644648 | -0.17785 | 5.402059 |
| MIR5194 | 0.842304 | 0.644727 | 0.451188 | 1.980334 |
| LOC641367 | 0.842666 | 0.645068 | -0.41219 | 2.384328 |
| NDUFAF8 | 0.842778 | 0.645249 | 0.23951 | 4.63178 |
| UTP18 | 0.842778 | 0.645287 | 0.196817 | 5.505269 |
| JRK | 0.842778 | 0.645347 | 0.213474 | 5.135197 |
| LRIF1 | 0.842826 | 0.645447 | 0.423231 | 2.540297 |
| KDSR | 0.842841 | 0.645558 | -0.19308 | 6.256242 |
| NCAPD2 | 0.842841 | 0.645587 | 0.18733 | 6.591615 |
| LRFN1 | 0.842881 | 0.645682 | 0.483148 | 1.885919 |
| AFF1-AS1 | 0.842992 | 0.645832 | -0.27698 | 2.898073 |
| ARRDC3-AS | 0.843254 | 0.646096 | 0.342567 | 2.694891 |
| ZNF396 | 0.843364 | 0.646245 | 0.42304 | 2.130091 |
| SKI | 0.843745 | 0.646668 | 0.166555 | 7.427553 |
| LOC101927 | 0.843745 | 0.646777 | 0.389076 | 2.610107 |
| SUPT7L | 0.843745 | 0.646887 | -0.5564 | 1.362623 |
| ELOVL1 | 0.843745 | 0.646932 | -0.42606 | 1.260031 |
| NGLY1 | 0.843745 | 0.646944 | -0.47103 | 1.345817 |
| HIST1H2BN | 0.843745 | 0.646973 | 0.370438 | 4.166089 |
| DUS1L | 0.843745 | 0.646987 | -0.18559 | 6.018385 |
| FAM160B2 | 0.843808 | 0.647262 | -0.19158 | 6.125935 |
| RPLP1 | 0.843808 | 0.647324 | 0.684861 | 1.057292 |
| SNRNP40 | 0.843808 | 0.647324 | 0.191875 | 4.88762 |
| MIR590 | 0.843808 | 0.647399 | 0.417434 | 1.344002 |
| LINC01311 | 0.843808 | 0.647404 | -0.48698 | 1.66375 |
| NRARP | 0.843808 | 0.647421 | 0.239581 | 5.808721 |
| MIR4265 | 0.844012 | 0.64769 | -0.48286 | 0.711377 |
| RNU5E-1 | 0.844012 | 0.647706 | 0.340974 | 7.077751 |
| UBA2 | 0.844146 | 0.647882 | -0.16983 | 7.265842 |
| SKP1 | 0.844146 | 0.647937 | -0.31174 | 2.514022 |
| RABGGTB | 0.844231 | 0.648068 | -0.43578 | 2.053381 |
| TEF | 0.844346 | 0.64822 | 0.609752 | 0.401928 |
| RPAP1 | 0.844446 | 0.648361 | -0.19153 | 5.139903 |
| NUDT14 | 0.844864 | 0.648746 | -0.27568 | 3.146619 |
| ZNF236 | 0.844918 | 0.648975 | 0.509218 | 1.317705 |
| BICRAL | 0.844918 | 0.649004 | 0.169037 | 6.114499 |
| TATDN2 | 0.844918 | 0.649022 | 0.161565 | 6.519643 |

| | | | | |
|---|---|---|---|---|
| ZNF451-AS | 0.844918 | 0.649057 | -0.50962 | 1.905907 |
| WNT8B | 0.844918 | 0.64911 | 0.59857 | 1.023693 |
| DPP7 | 0.845148 | 0.649407 | 0.172447 | 5.40194 |
| EHD2 | 0.845148 | 0.649415 | -0.18216 | 6.721459 |
| ZMIZ2 | 0.845262 | 0.649567 | 0.422911 | 1.36924 |
| MIR4755 | 0.845487 | 0.649805 | -0.391 | 1.869104 |
| ZNF736 | 0.84567 | 0.650047 | -0.2184 | 4.914899 |
| RNU5A-1 | 0.84567 | 0.650097 | 0.259906 | 9.437802 |
| MIR181A2 | 0.84567 | 0.650178 | 0.679621 | 1.267939 |
| PRKXP1 | 0.84567 | 0.650256 | -0.21525 | 5.019303 |
| NOB1 | 0.84567 | 0.650268 | -0.60752 | 0.521914 |
| COMT | 0.8461 | 0.650709 | 0.337036 | 3.228025 |
| CEP162 | 0.8461 | 0.650748 | 0.611396 | 0.77712 |
| RPL32P3 | 0.8461 | 0.650792 | -0.23355 | 3.794943 |
| TNPO1 | 0.846136 | 0.650928 | 0.44123 | 0.652301 |
| RDH10-AS1 | 0.846136 | 0.650948 | 0.252613 | 4.323728 |
| RP1 | 0.846145 | 0.65102 | -0.63201 | 1.841242 |
| SP2-AS1 | 0.846478 | 0.65134 | 0.580357 | 1.272378 |
| ZNF688 | 0.846679 | 0.65156 | -0.47901 | 0.816887 |
| MRPL23-AS | 0.846747 | 0.651719 | 0.564469 | 0.420104 |
| TEX12 | 0.846747 | 0.651766 | 0.696077 | 0.782363 |
| TMEM238 | 0.846747 | 0.651806 | -0.25123 | 3.462115 |
| GALNT11 | 0.846821 | 0.651934 | -0.30987 | 2.716644 |
| CEBPD | 0.846821 | 0.652016 | 0.185265 | 6.730994 |
| ADAMTS9- | 0.846821 | 0.65208 | 0.857181 | 0.490332 |
| DCLRE1B | 0.846821 | 0.652222 | 0.230724 | 3.595453 |
| PTCH1 | 0.846821 | 0.652242 | 0.456057 | 2.107438 |
| CINP | 0.846821 | 0.65225 | -0.21658 | 3.811402 |
| ADAT3 | 0.846883 | 0.652362 | 0.422728 | 1.832816 |
| FBXO31 | 0.8469 | 0.652498 | -0.40287 | 1.859927 |
| SUB1 | 0.8469 | 0.652545 | -0.17879 | 6.763252 |
| ALKAL2 | 0.8469 | 0.652609 | -0.43007 | 1.892959 |
| C8orf82 | 0.8469 | 0.652633 | -0.23837 | 3.793573 |
| NAGS | 0.847254 | 0.652971 | 0.413601 | 1.763552 |
| NEDD4L | 0.847488 | 0.653259 | -0.291 | 3.603164 |
| IL17REL | 0.847488 | 0.653308 | 0.528387 | 1.418319 |
| STAG3L1 | 0.847488 | 0.65342 | 0.279512 | 3.322582 |
| FN3KRP | 0.847488 | 0.653446 | 0.540966 | 0.638125 |
| CSDC2 | 0.847488 | 0.653488 | 0.363896 | 2.556345 |
| LOC10042 | 0.847488 | 0.653539 | -0.5829 | 1.445834 |
| SECISBP2 | 0.847596 | 0.653686 | 0.561115 | 0.726162 |
| EIF2B2 | 0.847666 | 0.653935 | -0.22022 | 4.661851 |
| ACSF3 | 0.847666 | 0.654012 | -0.58106 | 0.94894 |
| LAMA1 | 0.847666 | 0.654022 | 0.37375 | 3.372115 |
| MAGEF1 | 0.847666 | 0.654061 | 0.178597 | 5.263874 |

| | | | | |
|---|---|---|---|---|
| MIRLET7A1 | 0.847666 | 0.654171 | 0.425759 | 2.643161 |
| ZNF543 | 0.847666 | 0.654193 | 0.194552 | 4.903758 |
| NPHP3 | 0.847666 | 0.654193 | 0.252329 | 3.861826 |
| LAMTOR4 | 0.847677 | 0.654266 | 0.274012 | 4.392637 |
| RPL6 | 0.847742 | 0.65438 | 0.480596 | 1.048993 |
| LOC10192 | 0.847756 | 0.654456 | 0.385475 | 1.992649 |
| RAB21 | 0.847973 | 0.654688 | -0.2024 | 8.114887 |
| WBP4 | 0.848299 | 0.655004 | 0.176659 | 5.16388 |
| COIL | 0.848548 | 0.655317 | 0.211364 | 5.562157 |
| UGT1A9 | 0.848548 | 0.655326 | 0.648674 | 1.248827 |
| PLA2G4A | 0.848788 | 0.655576 | -0.53309 | 1.318992 |
| SNORA28 | 0.848819 | 0.655714 | 0.337905 | 4.014647 |
| IDS | 0.848819 | 0.65578 | -0.4641 | 1.142604 |
| SRSF3 | 0.848819 | 0.655794 | -0.26515 | 3.472906 |
| SMIM10L1 | 0.848874 | 0.655901 | 0.240294 | 4.47593 |
| NUDT21 | 0.849051 | 0.656109 | -0.1636 | 7.005347 |
| LOC10013( | 0.849051 | 0.656197 | 0.262805 | 3.285766 |
| PTPN3 | 0.849051 | 0.656274 | -0.52001 | 1.846833 |
| RNF168 | 0.849051 | 0.656297 | -0.16793 | 7.108083 |
| GJA5 | 0.849125 | 0.656419 | 0.641843 | 0.561518 |
| ERCC6L | 0.849197 | 0.656539 | 0.322655 | 3.470762 |
| FBXL20 | 0.849234 | 0.656632 | 0.684458 | 0.411167 |
| ATRNL1 | 0.849433 | 0.65689 | -0.54381 | 1.201368 |
| CUTALP | 0.849433 | 0.656916 | 0.238327 | 4.591968 |
| LMO4 | 0.849713 | 0.657197 | -0.18857 | 6.619255 |
| TTLL9 | 0.849907 | 0.657411 | 0.76834 | 0.549867 |
| ZNF587B | 0.850013 | 0.657559 | 0.173106 | 6.038312 |
| LINC01389 | 0.850078 | 0.657674 | 0.518362 | 1.13961 |
| NXF1 | 0.850292 | 0.657904 | 0.186661 | 4.918842 |
| HOXA6 | 0.851336 | 0.658806 | 0.641528 | 0.771422 |
| PSAPL1 | 0.851336 | 0.658842 | -0.29213 | 3.303617 |
| C1GALT1C1 | 0.851361 | 0.658932 | -0.65043 | 1.749167 |
| NBPF9 | 0.851361 | 0.658991 | -0.50287 | 0.750489 |
| RPL23AP87 | 0.85164 | 0.659272 | -0.57152 | 1.121952 |
| SLC44A1 | 0.851934 | 0.659658 | -0.21452 | 4.839697 |
| NOMO1 | 0.851934 | 0.659674 | -0.24678 | 4.331145 |
| SLC9A1 | 0.851934 | 0.659744 | 0.367176 | 2.078921 |
| HIST1H1C | 0.851934 | 0.659759 | 0.18585 | 6.850604 |
| GNPTAB | 0.851974 | 0.659855 | -0.15966 | 7.019484 |
| BOD1L1 | 0.852016 | 0.659952 | 0.155523 | 8.50651 |
| EEF1DP3 | 0.85228 | 0.660222 | -0.46246 | 1.492967 |
| MIR3192 | 0.852347 | 0.660338 | 0.767292 | 0.397149 |
| MOCS3 | 0.852375 | 0.660484 | 0.208946 | 3.739233 |
| LITAF | 0.852375 | 0.66049 | 0.356994 | 1.514153 |
| NFIB | 0.852738 | 0.660836 | -0.53353 | 1.498526 |

| | | | | |
|---|---|---|---|---|
| AQP6 | 0.852967 | 0.661079 | 0.624328 | 0.593975 |
| AGAP7P | 0.853385 | 0.661468 | 0.191864 | 4.52522 |
| ARSI | 0.853445 | 0.661637 | -0.37166 | 2.440966 |
| HEXIM1 | 0.853445 | 0.661644 | 0.15709 | 6.514845 |
| CFLAR | 0.853629 | 0.661852 | 0.178994 | 6.630031 |
| ZNF638 | 0.853823 | 0.662109 | 0.422971 | 1.604759 |
| HRH1 | 0.853823 | 0.662133 | -0.49756 | 1.956184 |
| MRPL37 | 0.853881 | 0.662243 | -0.18379 | 5.421164 |
| WWP2 | 0.853949 | 0.66236 | 0.308953 | 3.242962 |
| SLC38A7 | 0.85402 | 0.662481 | 0.461359 | 1.786609 |
| LOC100133 | 0.854214 | 0.662696 | 0.208988 | 4.415506 |
| USF2 | 0.854247 | 0.662786 | -0.56262 | 0.892461 |
| MRPL14 | 0.85437 | 0.662947 | -0.29922 | 4.088999 |
| C6orf62 | 0.854428 | 0.663161 | -0.16006 | 7.090636 |
| SNAI3 | 0.854428 | 0.663224 | 0.411798 | 1.311907 |
| LMNB1 | 0.854428 | 0.663251 | -0.30178 | 2.690368 |
| HYMAI | 0.854428 | 0.663343 | -0.34568 | 3.2415 |
| ARFGAP2 | 0.854428 | 0.663363 | 0.493875 | 0.847758 |
| NUDT18 | 0.854428 | 0.663385 | 0.261455 | 3.569405 |
| TM4SF5 | 0.854428 | 0.663448 | 0.860399 | 1.250011 |
| PIH1D1 | 0.854804 | 0.663805 | 0.168098 | 5.208462 |
| IL6R | 0.854911 | 0.663954 | 0.54013 | 0.913741 |
| ARL6IP1 | 0.854952 | 0.66405 | 0.253957 | 3.442644 |
| MRPL30 | 0.855418 | 0.664478 | -0.21528 | 4.080555 |
| PDIA3 | 0.85542 | 0.664544 | 0.164224 | 8.14643 |
| R3HCC1L | 0.855423 | 0.664612 | -0.59029 | 1.040823 |
| APBA1 | 0.855495 | 0.664733 | -0.39906 | 2.200572 |
| SLC9A4 | 0.855531 | 0.664826 | -0.63387 | 4.665095 |
| VAC14 | 0.855531 | 0.664926 | 0.159549 | 5.410278 |
| LAMB2P1 | 0.855531 | 0.665011 | 0.545259 | 1.006441 |
| WDR70 | 0.855531 | 0.665022 | -0.16203 | 5.693881 |
| PHACTR2-A | 0.855929 | 0.665425 | 0.368179 | 2.751128 |
| SET | 0.855929 | 0.665462 | -0.2448 | 3.697445 |
| MSS51 | 0.856141 | 0.665692 | 0.298082 | 3.103952 |
| RFX1 | 0.856428 | 0.666004 | -0.20296 | 4.617908 |
| ZDHHC11 | 0.856428 | 0.666045 | -0.26512 | 3.23696 |
| VPS13B | 0.856444 | 0.666123 | -0.64429 | 1.607 |
| FAM114A1 | 0.856475 | 0.666212 | -0.25714 | 3.203931 |
| RHOH | 0.856515 | 0.666309 | 0.495959 | 1.488789 |
| MND1 | 0.856557 | 0.666414 | -0.86967 | 0.374197 |
| PWWP2A | 0.856557 | 0.666472 | -0.2138 | 4.051153 |
| PKN1 | 0.856656 | 0.666665 | 0.69947 | 0.411557 |
| LRRC27 | 0.856656 | 0.66668 | -0.76131 | 1.031705 |
| RNF13 | 0.85718 | 0.667175 | -0.42089 | 1.338452 |
| ACVR2B-AS | 0.85718 | 0.667218 | 0.518303 | 1.691854 |

| | | | | |
|---|---|---|---|---|
| NTN5 | 0.857309 | 0.667384 | -0.56742 | 0.921764 |
| AGRN | 0.857523 | 0.667627 | -0.53494 | 0.968484 |
| CCDC97 | 0.857523 | 0.667681 | -0.14474 | 5.885233 |
| GGN | 0.85756 | 0.667776 | 0.480177 | 1.018464 |
| COQ10B | 0.857608 | 0.667878 | 0.207956 | 4.950128 |
| ATP5IF1 | 0.8579 | 0.668171 | -0.23473 | 3.648142 |
| LOC101928 | 0.857911 | 0.668276 | -0.80216 | 0.69724 |
| SSR4P1 | 0.857911 | 0.66831 | 0.621704 | 0.422781 |
| IGHMBP2 | 0.85792 | 0.668382 | 0.184196 | 4.990552 |
| MMGT1 | 0.858214 | 0.668734 | 0.180992 | 5.569444 |
| RBCK1 | 0.858214 | 0.668743 | 0.408163 | 2.082768 |
| NDUFAF4P | 0.858437 | 0.668982 | 0.510331 | 1.416698 |
| SPATA2L | 0.85854 | 0.669128 | 0.297799 | 2.814096 |
| ARL11 | 0.858559 | 0.669207 | -0.28959 | 2.736786 |
| AR | 0.85867 | 0.669372 | -0.4695 | 1.432037 |
| RUVBL1-AS | 0.85867 | 0.669425 | -0.67807 | 0.669251 |
| ZNF141 | 0.858766 | 0.669604 | -0.20304 | 4.552782 |
| MCMDC2 | 0.858766 | 0.669631 | -0.38221 | 2.030081 |
| TBCB | 0.858913 | 0.669811 | 0.380947 | 1.581376 |
| SPRY3 | 0.859094 | 0.670082 | 0.710974 | 0.278222 |
| ACYP1 | 0.859094 | 0.670256 | 0.525685 | 1.168319 |
| ZNF544 | 0.859094 | 0.670262 | -0.19578 | 5.534829 |
| FLJ12825 | 0.859094 | 0.670544 | 0.410474 | 1.940663 |
| XPC | 0.859094 | 0.670572 | -0.6041 | 0.453497 |
| CCR2 | 0.859094 | 0.670619 | 0.473185 | 2.022531 |
| RBM25 | 0.859094 | 0.670635 | -0.16568 | 9.292083 |
| PALB2 | 0.859094 | 0.670709 | -0.17681 | 5.234053 |
| CCL24 | 0.859094 | 0.670801 | -0.56588 | 0.926386 |
| RSPH6A | 0.859094 | 0.671122 | 0.606735 | 0.681603 |
| MAML2 | 0.859094 | 0.671133 | 0.168267 | 7.053819 |
| SIK3 | 0.859094 | 0.67122 | -0.27676 | 2.288533 |
| LOC100506 | 0.859094 | 0.671251 | 0.514606 | 1.235499 |
| FASTKD5 | 0.859094 | 0.67136 | -0.20863 | 4.741714 |
| CA5BP1 | 0.859094 | 0.671418 | -0.25494 | 3.120909 |
| MFGE8 | 0.859094 | 0.671497 | 0.323324 | 1.801845 |
| ARCN1 | 0.859094 | 0.671503 | -0.34772 | 2.41442 |
| MINCR | 0.859094 | 0.671517 | 0.614288 | 0.528945 |
| GMFB | 0.859094 | 0.671648 | -0.1801 | 5.853903 |
| WAC | 0.859094 | 0.671671 | -0.35657 | 2.033929 |
| FAM131C | 0.859094 | 0.671726 | -0.58604 | 0.694316 |
| AFG3L2 | 0.859094 | 0.671736 | 0.212262 | 7.392134 |
| SNX13 | 0.859094 | 0.671738 | 0.156646 | 6.808614 |
| APOA1-AS | 0.859094 | 0.671795 | 0.405127 | 1.509079 |
| GPR22 | 0.859094 | 0.671854 | -0.24018 | 3.646846 |
| FANCC | 0.859094 | 0.671922 | -0.36099 | 3.403565 |

| | | | | |
|---|---|---|---|---|
| PRKAB2 | 0.859094 | 0.671984 | -0.56586 | 1.017972 |
| UTP20 | 0.859094 | 0.672011 | -0.15699 | 6.595574 |
| SLC30A7 | 0.859094 | 0.672015 | 0.327955 | 2.229409 |
| PHB2 | 0.859094 | 0.672079 | 0.513605 | 1.156411 |
| HINT1 | 0.859094 | 0.67209 | 0.506011 | 0.727896 |
| SLC22A3 | 0.859094 | 0.672168 | -0.30101 | 4.453401 |
| PRRC1 | 0.859094 | 0.672176 | 0.190716 | 4.723903 |
| SNORA2A | 0.859094 | 0.672224 | -0.36784 | 2.48038 |
| HIST2H2BA | 0.859094 | 0.672244 | -0.40007 | 2.456462 |
| DGKQ | 0.859094 | 0.672309 | -0.16785 | 4.864128 |
| IPO7 | 0.8592 | 0.672462 | 0.151192 | 8.001596 |
| OPHN1 | 0.8592 | 0.672664 | -0.1639 | 5.454851 |
| LOC101929 | 0.8592 | 0.672694 | 0.595263 | 0.593897 |
| TBCK | 0.8592 | 0.672716 | 0.352126 | 1.907674 |
| TRIB1 | 0.8592 | 0.672719 | 0.300171 | 3.218726 |
| HDDC2 | 0.859227 | 0.672806 | -0.18394 | 5.627963 |
| ING2 | 0.859294 | 0.672924 | -0.4697 | 1.51076 |
| NEFM | 0.859326 | 0.673014 | -0.72661 | 0.440505 |
| B3GALNT2 | 0.859446 | 0.673202 | -0.14842 | 5.691809 |
| RNF181 | 0.859446 | 0.673239 | -0.2169 | 4.208414 |
| TMED1 | 0.859533 | 0.673461 | -0.38693 | 1.453354 |
| TAS2R31 | 0.859533 | 0.6735 | 0.424174 | 2.722185 |
| NATD1 | 0.859533 | 0.673575 | -0.20498 | 4.553449 |
| GALNS | 0.859533 | 0.673699 | 0.233796 | 4.665522 |
| NUTF2 | 0.859533 | 0.673735 | 0.179301 | 5.043294 |
| PDLIM1 | 0.859533 | 0.67377 | -0.16511 | 7.273916 |
| SNORA47 | 0.859533 | 0.673782 | 0.353059 | 6.367452 |
| KIF1BP | 0.859533 | 0.673831 | 0.20086 | 5.34697 |
| YPEL2 | 0.85979 | 0.674105 | -0.1494 | 5.941912 |
| DLSTP1 | 0.85979 | 0.674164 | -0.28715 | 3.228125 |
| LOC728392 | 0.859888 | 0.674334 | 0.292533 | 2.960869 |
| ASB7 | 0.859888 | 0.674372 | 0.191227 | 4.295264 |
| LIMD1 | 0.860056 | 0.674569 | 0.185568 | 5.55399 |
| DYNC2LI1 | 0.860342 | 0.674859 | -0.56701 | 1.211592 |
| DBN1 | 0.860362 | 0.67494 | 0.411814 | 1.212818 |
| C1orf123 | 0.860381 | 0.675021 | -0.39788 | 1.874839 |
| RPL7L1 | 0.860404 | 0.675118 | -0.16102 | 5.791986 |
| RFT1 | 0.860404 | 0.67517 | -0.17516 | 4.809613 |
| HP1BP3 | 0.861072 | 0.675823 | -0.14861 | 7.967269 |
| COL18A1-A | 0.861072 | 0.675827 | 0.500262 | 1.422288 |
| DHRS13 | 0.861072 | 0.675953 | -0.25391 | 2.923322 |
| NTRK1 | 0.861072 | 0.675957 | 0.842769 | 0.706691 |
| CSPP1 | 0.861148 | 0.676138 | 0.335222 | 2.35043 |
| ZNF527 | 0.861148 | 0.676148 | 0.260884 | 3.726394 |
| NOL8 | 0.861318 | 0.676355 | 0.55795 | 1.124322 |

| | | | | |
|---|---|---|---|---|
| TAF2 | 0.861318 | 0.676482 | -0.15791 | 6.727764 |
| NPRL2 | 0.861318 | 0.676526 | 0.25113 | 3.547179 |
| GALNT3 | 0.861318 | 0.676544 | 0.180717 | 7.312852 |
| ELMOD3 | 0.861385 | 0.676662 | 0.262347 | 2.857764 |
| ZNF471 | 0.861703 | 0.677026 | -0.29145 | 3.864526 |
| LINC00899 | 0.861703 | 0.677051 | -0.4878 | 1.355584 |
| LRRC37A2 | 0.861703 | 0.677109 | 0.410401 | 2.693528 |
| ABCA11P | 0.862135 | 0.677514 | 0.316851 | 2.625097 |
| IL27 | 0.862249 | 0.677698 | -0.70565 | 0.649026 |
| PPT1 | 0.862249 | 0.677735 | 0.440914 | 1.84547 |
| DOCK4-AS1 | 0.862415 | 0.677957 | -0.29706 | 3.228754 |
| CDC73 | 0.862415 | 0.677997 | 0.144408 | 7.028912 |
| RAB6C | 0.86245 | 0.67809 | -0.3164 | 2.259693 |
| NOP58 | 0.862569 | 0.678286 | 0.172533 | 7.020311 |
| USP30-AS1 | 0.862569 | 0.678315 | 0.551282 | 0.701759 |
| WIPI1 | 0.862741 | 0.678525 | 0.160265 | 5.597381 |
| HS6ST2-AS | 0.862741 | 0.678582 | -0.68623 | 0.729194 |
| CYP2C19 | 0.862746 | 0.678677 | -0.30459 | 4.51849 |
| MIR23B | 0.862746 | 0.678717 | -0.45315 | 1.626057 |
| EHMT1 | 0.863353 | 0.679287 | -0.15555 | 5.7757 |
| PAN3 | 0.863353 | 0.679327 | 0.151408 | 7.149591 |
| HIF1AN | 0.863472 | 0.679486 | -0.15061 | 6.906169 |
| ADM5 | 0.863583 | 0.679639 | 0.503064 | 1.282502 |
| FGF7P3 | 0.863685 | 0.679792 | 0.336677 | 2.607718 |
| GKAP1 | 0.863685 | 0.679851 | 0.773897 | 0.572992 |
| FJX1 | 0.863693 | 0.679923 | -0.40862 | 1.994847 |
| ZNF138 | 0.86401 | 0.680304 | -0.42206 | 2.298299 |
| FBXL17 | 0.86401 | 0.680304 | -0.16408 | 5.215284 |
| ZNF322 | 0.864016 | 0.680375 | -0.41662 | 1.75509 |
| SNORA71A | 0.864132 | 0.680558 | -0.21638 | 5.308204 |
| LINC00597 | 0.864132 | 0.680618 | 0.309997 | 2.421514 |
| MFSD4A | 0.864132 | 0.680664 | -0.49564 | 5.6682 |
| WWC2-AS2 | 0.864213 | 0.680806 | -0.4697 | 0.898418 |
| TMEM201 | 0.864213 | 0.680876 | -0.22394 | 3.862079 |
| MRPL57 | 0.864213 | 0.680944 | 0.187415 | 4.504827 |
| ACOX1 | 0.864213 | 0.681087 | -0.46561 | 1.708766 |
| SLC12A4 | 0.864213 | 0.681159 | 0.535955 | 1.130189 |
| SP140 | 0.864213 | 0.68122 | 0.718039 | 0.535532 |
| TSPYL6 | 0.864213 | 0.681242 | 0.359447 | 2.71985 |
| KRBA2 | 0.864213 | 0.681254 | -0.39973 | 1.620795 |
| NT5C2 | 0.864241 | 0.681343 | -0.48038 | 0.697233 |
| PCDHB2 | 0.864264 | 0.681495 | 0.521596 | 1.773881 |
| MAP6D1 | 0.864264 | 0.681517 | 0.489889 | 0.896802 |
| EMG1 | 0.864264 | 0.681558 | -0.19598 | 4.885223 |
| ATG3 | 0.864403 | 0.681733 | 0.482104 | 1.012137 |

| | | | | |
|---|---|---|---|---|
| YOD1 | 0.86445 | 0.681836 | -0.50613 | 1.789125 |
| MIR5047 | 0.864667 | 0.682073 | 0.152758 | 5.254509 |
| CCNB1 | 0.864928 | 0.682346 | -0.2934 | 4.943855 |
| CCT6P3 | 0.865132 | 0.682628 | -0.19424 | 4.375876 |
| IK | 0.865132 | 0.682638 | -0.18623 | 8.150011 |
| RBSN | 0.865148 | 0.682717 | -0.4877 | 0.860179 |
| LRRC40 | 0.865246 | 0.68286 | 0.175662 | 4.64466 |
| ERVH48-1 | 0.865569 | 0.683229 | -0.33414 | 2.38923 |
| ALG1L9P | 0.865569 | 0.683366 | 0.479752 | 1.426599 |
| CCZ1B | 0.865569 | 0.683403 | -0.18948 | 5.254523 |
| NOL9 | 0.865569 | 0.6835 | -0.14917 | 6.072668 |
| DKC1 | 0.865569 | 0.683521 | 0.472283 | 1.779129 |
| TIGD2 | 0.865569 | 0.68353 | -0.27861 | 3.015933 |
| PRCC | 0.865569 | 0.683577 | 0.155089 | 5.964215 |
| INSIG2 | 0.865703 | 0.683749 | 0.16451 | 5.44305 |
| LINC00965 | 0.865997 | 0.684047 | 0.541752 | 0.494012 |
| DDN | 0.866261 | 0.684404 | -0.58487 | 0.577241 |
| EFR3A | 0.866261 | 0.684466 | -0.14909 | 7.238154 |
| FLJ13224 | 0.866261 | 0.684548 | 0.339642 | 1.879096 |
| OPLAH | 0.866261 | 0.684581 | -0.18907 | 5.054952 |
| PEA15 | 0.866261 | 0.684585 | -0.51459 | 0.694314 |
| ANXA2P1 | 0.866293 | 0.684676 | -0.39432 | 2.341701 |
| LZTR1 | 0.866572 | 0.684963 | 0.149056 | 5.842023 |
| CYP3A7-CY | 0.866574 | 0.685031 | -0.52288 | 1.002328 |
| SNORD36B | 0.866611 | 0.685126 | 0.346098 | 3.39846 |
| BMS1P20 | 0.866778 | 0.685422 | -0.15391 | 5.876286 |
| LOC10050§ | 0.866778 | 0.685426 | 0.314092 | 2.711712 |
| GPC2 | 0.866778 | 0.685472 | 0.488945 | 1.22291 |
| PTGFRN | 0.866778 | 0.685522 | -0.15384 | 7.652849 |
| MCRS1 | 0.86678 | 0.685637 | 0.520727 | 0.994392 |
| LINC00982 | 0.86678 | 0.685656 | -0.68854 | 4.210067 |
| SLC25A16 | 0.866919 | 0.685832 | 0.233068 | 4.147185 |
| APLN | 0.867023 | 0.68598 | 0.379127 | 2.166602 |
| ZNF137P | 0.867039 | 0.686084 | -0.31608 | 2.519146 |
| SH2D1B | 0.867039 | 0.686127 | 0.504011 | 1.624799 |
| NOP53 | 0.867039 | 0.686251 | 0.156954 | 7.614109 |
| MEG8 | 0.867039 | 0.686258 | 0.402101 | 1.725822 |
| STX8 | 0.867194 | 0.686446 | 0.552571 | 0.717141 |
| FHL5 | 0.867321 | 0.686657 | 0.654631 | 0.89382 |
| ZBTB44 | 0.867321 | 0.686704 | -0.17689 | 4.992958 |
| SNORA52 | 0.867321 | 0.686745 | 0.318548 | 4.1865 |
| ARF4 | 0.867349 | 0.68684 | 0.161621 | 7.395839 |
| ATP6V0B | 0.867349 | 0.686959 | 0.262646 | 2.945084 |
| RPA1 | 0.867349 | 0.686965 | -0.15944 | 6.289085 |
| WASH3P | 0.867361 | 0.687041 | 0.228824 | 4.202812 |

| | | | | |
|---|---|---|---|---|
| LINC01913 | 0.867539 | 0.687248 | 0.840562 | 1.428612 |
| PINK1-AS | 0.867919 | 0.687616 | -0.236 | 3.010375 |
| COL6A4P2 | 0.868094 | 0.68782 | 0.539964 | 1.846447 |
| TECPR1 | 0.868236 | 0.687999 | -0.159 | 5.865207 |
| TIAM2 | 0.868382 | 0.688322 | 0.269352 | 2.579906 |
| DLST | 0.868382 | 0.688349 | 0.305073 | 3.690961 |
| MGST3 | 0.868382 | 0.688355 | 0.286376 | 5.160177 |
| C16orf70 | 0.868382 | 0.688379 | -0.1877 | 4.548717 |
| MRPL17 | 0.868898 | 0.68893 | 0.184747 | 4.648829 |
| SF3B2 | 0.868898 | 0.688969 | 0.146076 | 8.487576 |
| ATXN2 | 0.868898 | 0.689016 | 0.519501 | 0.729146 |
| MIR4453H( | 0.868898 | 0.68906 | -0.27846 | 3.174544 |
| SMYD4 | 0.868898 | 0.689119 | 0.200451 | 4.602714 |
| RNF217-AS | 0.868932 | 0.689212 | 0.478128 | 2.216539 |
| NOA1 | 0.869005 | 0.689435 | -0.16629 | 4.841667 |
| CCDC28A | 0.869005 | 0.689482 | -0.19428 | 4.661442 |
| HIST1H2BI | 0.869005 | 0.689534 | -0.26464 | 4.107527 |
| GALNT7 | 0.869005 | 0.689581 | -0.16684 | 6.346064 |
| KCNS1 | 0.869005 | 0.689695 | -0.48949 | 1.65239 |
| LNCOC1 | 0.869005 | 0.689766 | -0.40655 | 1.282535 |
| CD47 | 0.869005 | 0.689797 | 0.461405 | 1.586427 |
| RPF1 | 0.869005 | 0.6898 | -0.19997 | 4.786858 |
| NBEAL1 | 0.869162 | 0.690011 | 0.146839 | 7.736362 |
| USB1 | 0.869162 | 0.690057 | -0.30281 | 2.967702 |
| CBX1 | 0.869293 | 0.690227 | -0.36075 | 1.770179 |
| C7orf43 | 0.869398 | 0.690424 | 0.502687 | 0.834585 |
| TUBGCP5 | 0.869398 | 0.690443 | -0.65641 | 0.507234 |
| AREL1 | 0.869512 | 0.69066 | 0.169692 | 6.310765 |
| MAGI1 | 0.869512 | 0.690667 | -0.39369 | 1.346253 |
| SNRPA | 0.869517 | 0.690737 | 0.149509 | 5.314253 |
| FAM169A | 0.869591 | 0.690862 | 0.701316 | 0.897602 |
| STX1B | 0.8697 | 0.691014 | -0.39289 | 2.040036 |
| PPP2R2D | 0.869733 | 0.691169 | 0.292415 | 3.64438 |
| DCC | 0.869733 | 0.691197 | 0.50218 | 1.804833 |
| NUS1 | 0.869733 | 0.69124 | -0.16341 | 6.017089 |
| CNTNAP3P | 0.869739 | 0.691311 | -0.19144 | 5.267289 |
| QRICH1 | 0.869973 | 0.69161 | 0.628072 | 0.419788 |
| NFYC-AS1 | 0.869973 | 0.691629 | 0.288836 | 3.340087 |
| APOPT1 | 0.870076 | 0.691777 | -0.26891 | 2.246173 |
| AKR7L | 0.870221 | 0.691959 | -0.42793 | 1.776927 |
| MIF-AS1 | 0.870429 | 0.692191 | 0.49663 | 1.743747 |
| EXOC3L4 | 0.870765 | 0.692586 | 0.517261 | 1.707982 |
| ZBTB7A | 0.870765 | 0.69259 | -0.3407 | 1.466348 |
| SNORA40 | 0.870902 | 0.692841 | 0.189965 | 6.129104 |
| TBPL1 | 0.870902 | 0.692906 | -0.41682 | 1.307969 |

| | | | | |
|---|---|---|---|---|
| ABR | 0.870902 | 0.692979 | 0.561321 | 1.373063 |
| MT1E | 0.870902 | 0.693038 | 0.328739 | 4.589029 |
| MORF4L1 | 0.870902 | 0.693094 | 0.394562 | 1.761927 |
| PIK3C3 | 0.870902 | 0.693098 | -0.55296 | 0.92748 |
| SIRT1 | 0.870902 | 0.693194 | 0.244395 | 3.412687 |
| MIR2116 | 0.870902 | 0.69323 | 0.62388 | 0.973342 |
| LARS2 | 0.870904 | 0.69338 | 0.163106 | 5.046885 |
| LRRN1 | 0.870904 | 0.693401 | 0.289288 | 2.224691 |
| SNORA75 | 0.870904 | 0.693431 | 0.228582 | 5.496586 |
| MIR938 | 0.871055 | 0.693631 | -0.56303 | 0.859075 |
| CSAD | 0.871055 | 0.693722 | 0.232805 | 3.136818 |
| DEPDC1 | 0.871055 | 0.69375 | 0.471863 | 1.890712 |
| COL6A6 | 0.871297 | 0.694017 | -0.42064 | 2.779749 |
| HMOX1 | 0.871297 | 0.694166 | -0.31868 | 4.669683 |
| SNORD121 | 0.871297 | 0.694171 | 0.436535 | 1.30447 |
| LINC00853 | 0.871297 | 0.694209 | -0.52525 | 1.022848 |
| CD2AP | 0.871373 | 0.694383 | 0.15687 | 8.397523 |
| OVOL3 | 0.871373 | 0.69451 | -0.47736 | 0.73426 |
| GLT8D1 | 0.871373 | 0.694511 | 0.511469 | 0.733938 |
| UBOX5-AS1 | 0.871373 | 0.694574 | 0.258585 | 2.251917 |
| MYL12B | 0.871373 | 0.694602 | -0.61336 | 1.619485 |
| GNAI1 | 0.871425 | 0.694709 | -0.29128 | 3.528217 |
| URB1-AS1 | 0.871448 | 0.694794 | 0.561877 | 0.288278 |
| EBLN2 | 0.871493 | 0.694957 | -0.25147 | 3.435766 |
| CAMKMT | 0.871493 | 0.69503 | -0.23804 | 3.54749 |
| GTF2IP1 | 0.871493 | 0.695036 | -0.14384 | 5.818473 |
| BRE-AS1 | 0.871493 | 0.695096 | -0.55989 | 0.66721 |
| NABP2 | 0.871661 | 0.695296 | 0.225396 | 4.087972 |
| PDAP1 | 0.872093 | 0.695707 | 0.170513 | 8.140452 |
| VAMP5 | 0.872598 | 0.696177 | 0.297521 | 3.29017 |
| PGBD5 | 0.872989 | 0.696559 | -0.41748 | 1.520777 |
| ABAT | 0.872989 | 0.696672 | 0.490987 | 0.781695 |
| FAM151B | 0.872989 | 0.696688 | -0.50958 | 1.897453 |
| DSTN | 0.873272 | 0.69698 | 0.347379 | 1.587165 |
| MIR2278 | 0.873372 | 0.697252 | -0.46605 | 1.186533 |
| LINC01410 | 0.873372 | 0.697322 | 0.254536 | 2.998014 |
| NPFF | 0.873372 | 0.697372 | 0.422409 | 1.15548 |
| CUL5 | 0.873372 | 0.697387 | 0.147554 | 6.729674 |
| RABGAP1L | 0.873372 | 0.697393 | -0.14536 | 6.6877 |
| ZNF575 | 0.873392 | 0.697475 | -0.34645 | 1.584023 |
| CSNK1G2 | 0.873838 | 0.697939 | 0.145331 | 6.113182 |
| MIR663A | 0.873838 | 0.697965 | 0.25309 | 2.668937 |
| MORN1 | 0.874606 | 0.698685 | 0.350423 | 1.880607 |
| NFASC | 0.874606 | 0.698753 | 0.45225 | 1.361478 |
| PRDX4 | 0.874606 | 0.698778 | 0.199402 | 5.178102 |

| | | | | |
|---|---|---|---|---|
| EVL | 0.874711 | 0.698929 | 0.192304 | 5.600767 |
| ID4 | 0.87476 | 0.699034 | 0.204431 | 5.635389 |
| UBE3C | 0.874801 | 0.699181 | 0.160406 | 7.476111 |
| MIR3139 | 0.874801 | 0.699201 | 0.488759 | 1.324362 |
| MBLAC1 | 0.874838 | 0.699312 | -0.3477 | 1.950634 |
| ART1 | 0.874838 | 0.699419 | -0.6445 | 0.500115 |
| CUL9 | 0.874838 | 0.699478 | -0.14158 | 6.751208 |
| ETFA | 0.874838 | 0.699497 | 0.512906 | 0.780161 |
| CCDC112 | 0.874931 | 0.699638 | 0.434573 | 0.765487 |
| LINC00485 | 0.87545 | 0.70012 | -0.80071 | 0.51153 |
| MGC7087( | 0.875754 | 0.700458 | 0.327368 | 2.596003 |
| ATXN1L | 0.875754 | 0.700496 | 0.137574 | 6.794439 |
| ACSM1 | 0.875903 | 0.700683 | 0.516818 | 1.522465 |
| ZNF611 | 0.875903 | 0.700749 | 0.598156 | 0.72626 |
| C15orf53 | 0.875918 | 0.700828 | -0.58399 | 0.738518 |
| GALT | 0.876123 | 0.70118 | -0.55339 | 0.53281 |
| BICRA | 0.876123 | 0.701218 | 0.155989 | 5.17879 |
| TMEM268 | 0.876123 | 0.701351 | 0.177917 | 4.694849 |
| FOXA1 | 0.876123 | 0.701401 | -0.2009 | 6.023107 |
| SETX | 0.876123 | 0.701486 | 0.143494 | 8.543668 |
| FRMPD2B | 0.876123 | 0.701549 | -0.54195 | 1.069482 |
| SNORD19 | 0.876123 | 0.701577 | -0.32506 | 1.081395 |
| MINOS1 | 0.876123 | 0.701593 | 0.406305 | 1.639509 |
| LINC01145 | 0.876123 | 0.701654 | -0.4841 | 0.817823 |
| ZNF384 | 0.876123 | 0.701746 | 0.462864 | 1.141152 |
| NIT2 | 0.876123 | 0.701786 | -0.18662 | 4.305816 |
| LINC01176 | 0.876123 | 0.701853 | -0.21291 | 3.291574 |
| LTBP4 | 0.876123 | 0.701927 | 0.37725 | 2.112364 |
| PATE4 | 0.876123 | 0.701935 | -0.56534 | 1.038376 |
| MUC4 | 0.876123 | 0.701994 | -0.36831 | 9.277895 |
| SLCO2A1 | 0.87614 | 0.702132 | 0.226192 | 4.536499 |
| PTGDR2 | 0.87614 | 0.70214 | -0.44543 | 1.751955 |
| TG | 0.876206 | 0.70232 | 0.334659 | 2.359214 |
| LINC01089 | 0.876206 | 0.702327 | -0.19929 | 4.297992 |
| CHCHD3 | 0.876476 | 0.702629 | -0.58908 | 0.880376 |
| ZBTB3 | 0.876476 | 0.702677 | 0.288034 | 3.013374 |
| KMT2E-AS1 | 0.87673 | 0.702948 | -0.14613 | 5.338665 |
| TWIST2 | 0.876734 | 0.703018 | 0.411876 | 2.332572 |
| CAND1 | 0.877192 | 0.703452 | -0.14374 | 7.464773 |
| NPAS4 | 0.87738 | 0.703669 | -0.60154 | 0.883816 |
| TMEM35A | 0.877611 | 0.703921 | 0.660381 | 1.303405 |
| RPRD1B | 0.877654 | 0.704023 | 0.133241 | 5.797452 |
| LGR4 | 0.877681 | 0.704111 | 0.186661 | 6.712116 |
| RBM15 | 0.877706 | 0.704199 | 0.305945 | 2.614275 |
| ACYP2 | 0.877786 | 0.704329 | 0.338774 | 1.993371 |

| | | | | |
|---|---|---|---|---|
| SEC31B | 0.877798 | 0.704406 | 0.160879 | 5.189133 |
| ZNF845 | 0.877937 | 0.704584 | -0.20969 | 4.297644 |
| ZDHHC1 | 0.877949 | 0.704808 | 0.281721 | 2.788487 |
| LRRC55 | 0.877949 | 0.704901 | 0.554571 | 0.548669 |
| DIMT1 | 0.877949 | 0.705069 | -0.21458 | 4.767018 |
| ALG10 | 0.877949 | 0.705088 | 0.290636 | 3.269866 |
| CASQ1 | 0.877949 | 0.705101 | -0.38976 | 1.564365 |
| SNORA33 | 0.877949 | 0.705126 | -0.21221 | 5.903855 |
| CILP | 0.877949 | 0.705147 | -0.29466 | 2.975954 |
| RPA4 | 0.877949 | 0.70526 | 0.345354 | 2.134162 |
| EIF2B3 | 0.877949 | 0.705291 | 0.366464 | 2.224718 |
| SAP18 | 0.877949 | 0.705303 | 0.13993 | 6.675828 |
| SMCR8 | 0.877949 | 0.705355 | 0.14904 | 6.184452 |
| ABHD6 | 0.877949 | 0.705396 | -0.21339 | 3.835697 |
| HIST1H3C | 0.877968 | 0.705534 | -0.19251 | 4.614298 |
| SNORA110 | 0.877968 | 0.705546 | 0.527107 | 0.59134 |
| MIR661 | 0.877968 | 0.705612 | -0.62087 | 0.789836 |
| PISD | 0.878107 | 0.705866 | 0.153488 | 5.398088 |
| RNF24 | 0.878107 | 0.705878 | 0.43759 | 1.155814 |
| FXYD7 | 0.878107 | 0.705925 | 0.5931 | 2.039431 |
| FAM13A-A | 0.878535 | 0.706336 | 0.216783 | 3.536869 |
| MIR3128 | 0.878847 | 0.706654 | 0.567781 | 1.359621 |
| WDR92 | 0.878976 | 0.706869 | 0.273459 | 2.149165 |
| UPF3A | 0.878976 | 0.706891 | -0.45656 | 1.400849 |
| TUT1 | 0.879201 | 0.707139 | 0.194102 | 4.949447 |
| LOC10192 | 0.879302 | 0.707299 | 0.209708 | 3.4134 |
| PDCD6IPP2 | 0.879302 | 0.707355 | -0.20724 | 3.555305 |
| DEF6 | 0.879394 | 0.707496 | -0.14458 | 5.991502 |
| RABEP2 | 0.87958 | 0.707713 | -0.1759 | 5.146438 |
| ATP5PD | 0.87969 | 0.70792 | 0.399495 | 1.151881 |
| TMEM115 | 0.87969 | 0.707935 | -0.15113 | 4.723118 |
| RNVU1-6 | 0.879697 | 0.708008 | 0.480092 | 0.907096 |
| VEGFC | 0.879797 | 0.708155 | 0.290193 | 2.321393 |
| MIR4296 | 0.879897 | 0.708303 | -0.34563 | 1.772417 |
| CHMP4B | 0.879915 | 0.708384 | -0.14392 | 7.836004 |
| POU6F1 | 0.880118 | 0.708763 | -0.42412 | 1.718766 |
| NPM3 | 0.880118 | 0.708774 | -0.26793 | 3.604136 |
| ZW10 | 0.880118 | 0.708868 | 0.154017 | 5.281825 |
| DAGLA | 0.880118 | 0.708888 | -0.21748 | 4.059577 |
| FBXL18 | 0.880118 | 0.708929 | 0.141105 | 5.43902 |
| ATF7 | 0.880118 | 0.708965 | 0.330076 | 1.52005 |
| DUT | 0.880118 | 0.709077 | 0.458259 | 0.820313 |
| SRRD | 0.880118 | 0.709084 | -0.21063 | 3.870048 |
| TCTEX1D4 | 0.88018 | 0.709234 | -0.22217 | 3.008281 |
| OR2A7 | 0.88018 | 0.709269 | -0.55642 | 0.391686 |

| | | | | |
|---|---|---|---|---|
| BROX | 0.880387 | 0.709809 | 0.34496 | 2.346389 |
| CDK8 | 0.880387 | 0.709856 | -0.17707 | 5.112724 |
| B4GAT1 | 0.880387 | 0.709929 | 0.242516 | 3.413269 |
| LOC64913: | 0.880387 | 0.70993 | 0.5449 | 0.545887 |
| ASTN2 | 0.880387 | 0.710023 | -0.41488 | 2.409853 |
| TLK2 | 0.880387 | 0.710122 | 0.542715 | 0.728626 |
| TFEB | 0.880387 | 0.710178 | -0.52692 | 0.945731 |
| KIAA0825 | 0.880387 | 0.710186 | 0.284659 | 3.08552 |
| MIR98 | 0.880387 | 0.710245 | 0.403469 | 1.358696 |
| FKBP11 | 0.880387 | 0.710249 | 0.321312 | 1.984508 |
| TJP3 | 0.880387 | 0.710318 | 0.310167 | 2.144049 |
| NFIA | 0.880387 | 0.710337 | 0.466584 | 0.528075 |
| SLC35D1 | 0.880387 | 0.710492 | -0.15403 | 5.65228 |
| RBBP6 | 0.880387 | 0.710498 | -0.41878 | 1.97049 |
| LINC00957 | 0.880387 | 0.710642 | -0.37557 | 1.745735 |
| PUS10 | 0.880387 | 0.710645 | -0.18546 | 4.025542 |
| THRIL | 0.880387 | 0.71076 | 0.202134 | 3.758164 |
| HGSNAT | 0.880387 | 0.710766 | 0.130593 | 6.995901 |
| KLF10 | 0.880387 | 0.710801 | 0.309742 | 1.837381 |
| RFTN2 | 0.880387 | 0.710843 | -0.26823 | 3.255874 |
| SLCO4A1-A | 0.880387 | 0.710845 | 0.233475 | 3.132937 |
| MIR3619 | 0.880458 | 0.710969 | 0.410965 | 1.568501 |
| ZC3H15 | 0.880557 | 0.711116 | 0.134687 | 7.361992 |
| SPRN | 0.880788 | 0.711414 | 0.230215 | 3.961961 |
| GOLGA1 | 0.880788 | 0.71147 | 0.132677 | 6.069206 |
| LOC100507 | 0.880788 | 0.711504 | 0.295418 | 3.341957 |
| GNAQ | 0.881023 | 0.71176 | -0.13084 | 7.343316 |
| SMAD2 | 0.881217 | 0.711985 | 0.287909 | 1.446166 |
| ZSWIM9 | 0.88132 | 0.712135 | 0.197658 | 4.774798 |
| MACC1-AS | 0.881323 | 0.712228 | 0.243707 | 3.013072 |
| KIAA0556 | 0.881323 | 0.712272 | -0.14481 | 6.521174 |
| SNORD1A | 0.881471 | 0.712472 | 0.525038 | 0.968444 |
| RAD21 | 0.881471 | 0.712526 | 0.151323 | 8.468996 |
| LINC00884 | 0.881678 | 0.71276 | 0.521583 | 0.528578 |
| ATP5F1A | 0.881686 | 0.712834 | -0.37585 | 1.227611 |
| C19orf67 | 0.882035 | 0.713183 | 0.512895 | 0.896587 |
| STX17-AS1 | 0.882052 | 0.713264 | 0.617629 | 0.556924 |
| PPM1L | 0.882134 | 0.713512 | -0.24301 | 2.892399 |
| PRSS48 | 0.882134 | 0.713525 | 0.197473 | 3.804602 |
| C22orf39 | 0.882134 | 0.713533 | 0.279718 | 3.573865 |
| TBC1D8 | 0.882139 | 0.713604 | 0.14241 | 7.034159 |
| MLLT1 | 0.882234 | 0.713748 | 0.127893 | 6.439196 |
| DDX21 | 0.882588 | 0.714101 | -0.26689 | 3.644483 |
| GCH1 | 0.882908 | 0.714437 | -0.47001 | 1.419096 |
| LINC01560 | 0.882908 | 0.714494 | 0.335673 | 2.094343 |

| | | | | |
|---|---|---|---|---|
| CCL13 | 0.883101 | 0.714718 | 0.654638 | 0.792475 |
| SSSCA1-AS | 0.88318 | 0.714849 | -0.47341 | 1.024223 |
| EEFSEC | 0.883247 | 0.714971 | -0.16527 | 4.114881 |
| PANK1 | 0.883305 | 0.715155 | 0.474443 | 0.419476 |
| ABCA7 | 0.883305 | 0.715208 | -0.1405 | 5.855293 |
| PIK3C2B | 0.883305 | 0.715283 | -0.15276 | 7.020159 |
| LOC10272 | 0.883305 | 0.715303 | -0.17076 | 3.88608 |
| MRPS9 | 0.883305 | 0.715355 | -0.15138 | 5.290057 |
| SNORA87 | 0.883912 | 0.715914 | -0.31156 | 2.240028 |
| PLLP | 0.884 | 0.716097 | -0.23082 | 3.831624 |
| HNRNPL | 0.884 | 0.716149 | -0.17607 | 4.072569 |
| GZF1 | 0.884 | 0.716295 | -0.42707 | 1.311012 |
| PDZRN3 | 0.884 | 0.716308 | -0.20848 | 4.398584 |
| DRICH1 | 0.884 | 0.716322 | 0.337127 | 2.000063 |
| LOC10050 | 0.884326 | 0.716675 | 0.203493 | 3.967176 |
| RBM39 | 0.884326 | 0.716721 | -0.48789 | 0.396474 |
| KLF11 | 0.884357 | 0.716813 | 0.515901 | 0.723909 |
| TMEM168 | 0.884441 | 0.716949 | 0.338318 | 1.63513 |
| NEMP1 | 0.884679 | 0.717209 | -0.4544 | 0.829502 |
| ZGLP1 | 0.884816 | 0.717387 | 0.414998 | 0.780738 |
| CYB561 | 0.885127 | 0.717707 | 0.427075 | 1.310651 |
| PCIF1 | 0.885175 | 0.717813 | 0.137568 | 5.258192 |
| RNF216 | 0.885196 | 0.717963 | 0.483111 | 0.650596 |
| AGAP11 | 0.885196 | 0.718089 | -0.23752 | 4.418465 |
| KLHL25 | 0.885196 | 0.718141 | 0.225927 | 4.31398 |
| SOSTDC1 | 0.885196 | 0.718156 | -0.38001 | 1.023593 |
| KLHL36 | 0.885196 | 0.718168 | 0.33488 | 1.078147 |
| FRAS1 | 0.885414 | 0.718412 | -0.2089 | 5.205955 |
| DRAM2 | 0.885742 | 0.71875 | 0.152639 | 4.784532 |
| EXOC6 | 0.885742 | 0.718872 | 0.568149 | 0.632984 |
| JAZF1 | 0.885742 | 0.718941 | 0.187205 | 3.976191 |
| PURB | 0.885742 | 0.718948 | -0.1339 | 7.548238 |
| NINJ2 | 0.885843 | 0.719128 | 0.395006 | 1.060645 |
| PCNX3 | 0.885843 | 0.719165 | 0.139839 | 6.818169 |
| HIST1H3G | 0.885891 | 0.719338 | -0.2032 | 4.339692 |
| KDM1A | 0.885891 | 0.719408 | -0.57747 | 0.401364 |
| CLDN8 | 0.885891 | 0.719495 | -0.51989 | 0.670199 |
| THEM6 | 0.885891 | 0.719503 | -0.16389 | 4.267755 |
| PTAR1 | 0.885891 | 0.719641 | 0.134925 | 7.356783 |
| LOC101927 | 0.885891 | 0.719641 | -0.36246 | 2.776483 |
| RPS18 | 0.885891 | 0.719726 | -0.20108 | 5.756641 |
| EIF4E2 | 0.885891 | 0.719744 | -0.17016 | 4.032724 |
| CHD7 | 0.885912 | 0.719829 | -0.1675 | 7.399838 |
| ZNF519 | 0.885954 | 0.71993 | 0.205733 | 4.303076 |
| PPP3CB | 0.886063 | 0.720132 | 0.499463 | 0.588185 |

| | | | | |
|---|---|---|---|---|
| DCAF13P3 | 0.886063 | 0.720205 | -0.27576 | 3.132947 |
| C9orf66 | 0.886063 | 0.720356 | -0.46542 | 1.178563 |
| CPT2 | 0.886063 | 0.720379 | -0.172 | 4.822355 |
| MED23 | 0.886063 | 0.720493 | 0.300561 | 3.001943 |
| KRT17P5 | 0.886063 | 0.720544 | -0.7822 | 0.673314 |
| CDRT15P1 | 0.886063 | 0.720592 | 0.427867 | 1.402799 |
| HIST1H2AH | 0.886063 | 0.720615 | -0.18866 | 4.306532 |
| NFKBIZ | 0.886063 | 0.720663 | -0.3099 | 2.361537 |
| MARS | 0.886063 | 0.720718 | 0.138761 | 6.66133 |
| TFB2M | 0.886063 | 0.720791 | 0.182234 | 4.508745 |
| TMCC3 | 0.886063 | 0.720876 | 0.457108 | 1.401268 |
| SOX18 | 0.886063 | 0.720936 | 0.359853 | 1.823052 |
| ENTPD1-AS | 0.886063 | 0.720975 | 0.327656 | 2.131521 |
| SNORD25 | 0.886063 | 0.721039 | 0.409314 | 1.486059 |
| HRAT5 | 0.886063 | 0.721204 | -0.56592 | 1.632301 |
| MAPK13 | 0.886063 | 0.721235 | -0.39346 | 1.132351 |
| SPATA24 | 0.886063 | 0.721235 | 0.39948 | 1.330218 |
| H2AFY | 0.886105 | 0.721379 | 0.520601 | 0.530257 |
| CCNA2 | 0.886105 | 0.721404 | -0.27639 | 4.22204 |
| SELENOO | 0.886131 | 0.721492 | 0.159554 | 4.669831 |
| CDK4 | 0.88655 | 0.721977 | 0.168171 | 4.917871 |
| ARL17A | 0.88655 | 0.722009 | -0.23395 | 4.079288 |
| CASP10 | 0.88655 | 0.722036 | -0.50757 | 0.756182 |
| ACOT7 | 0.886712 | 0.722236 | -0.45409 | 1.226553 |
| LINC00092 | 0.886721 | 0.722328 | 0.610092 | 0.589137 |
| MROH1 | 0.886721 | 0.722378 | -0.29596 | 1.746678 |
| RNF139-AS | 0.887263 | 0.722887 | 0.303746 | 2.535561 |
| ANGEL2 | 0.887661 | 0.723304 | 0.255504 | 2.709818 |
| LIG1 | 0.887661 | 0.723446 | -0.3089 | 2.671107 |
| GPX7 | 0.887661 | 0.723449 | 0.287918 | 2.38866 |
| PCNP | 0.887661 | 0.723483 | -0.14559 | 6.644238 |
| PIEZO1 | 0.88771 | 0.72359 | -0.1382 | 7.8625 |
| SLC18A2 | 0.887873 | 0.723791 | -0.192 | 4.678202 |
| REP15 | 0.888093 | 0.724107 | 0.411882 | 1.407871 |
| VPS18 | 0.888093 | 0.724141 | 0.193462 | 4.849405 |
| LOC728673 | 0.888093 | 0.724173 | 0.356734 | 1.693452 |
| ABRAXAS1 | 0.888433 | 0.724653 | 0.191069 | 3.5356 |
| SDCBP2 | 0.888433 | 0.724677 | -0.26704 | 2.525148 |
| NSRP1 | 0.888433 | 0.72478 | 0.300376 | 2.186542 |
| AGGF1 | 0.888433 | 0.724828 | -0.1254 | 6.587182 |
| FAM83D | 0.888433 | 0.724855 | -0.23264 | 6.082909 |
| SLC16A12 | 0.888433 | 0.724906 | 0.619407 | 0.814781 |
| NEK4 | 0.888433 | 0.724924 | -0.36519 | 1.230373 |
| ZBED4 | 0.888475 | 0.725026 | 0.137424 | 6.182023 |
| MIR4712 | 0.888498 | 0.725113 | -0.48448 | 1.544128 |

| | | | | |
|---|---|---|---|---|
| ZNF528 | 0.888645 | 0.725381 | -0.19093 | 4.436429 |
| URB1 | 0.888645 | 0.725403 | 0.143755 | 6.89326 |
| ZUP1 | 0.888645 | 0.725435 | 0.155172 | 4.514199 |
| EIF5B | 0.888753 | 0.725591 | -0.1261 | 9.139922 |
| PRPF39 | 0.888909 | 0.725911 | 0.12814 | 5.975844 |
| MGME1 | 0.888909 | 0.725938 | 0.548412 | 0.763908 |
| MAP7 | 0.888909 | 0.725988 | 0.531446 | 0.421025 |
| SSBP1 | 0.888909 | 0.726014 | 0.409545 | 1.851074 |
| PSME4 | 0.888909 | 0.726058 | 0.129016 | 7.674473 |
| AJM1 | 0.889147 | 0.72632 | 0.233253 | 2.82522 |
| CCBE1 | 0.889403 | 0.726628 | -0.29554 | 2.667709 |
| NEDD4 | 0.889403 | 0.726728 | 0.284169 | 2.291626 |
| LINC00032 | 0.889403 | 0.726732 | 0.511088 | 0.822807 |
| ZBTB25 | 0.889439 | 0.726829 | -0.29594 | 2.503101 |
| TAF10 | 0.889707 | 0.727116 | -0.13938 | 5.180051 |
| BCRP2 | 0.889845 | 0.727297 | 0.468963 | 0.952259 |
| PICSAR | 0.890089 | 0.727614 | 0.676466 | 1.001171 |
| TRIAP1 | 0.890089 | 0.727632 | -0.16883 | 4.139196 |
| CREBZF | 0.8901 | 0.727708 | 0.169407 | 4.222974 |
| WISP2 | 0.89014 | 0.727854 | -0.45716 | 1.273872 |
| METTL8 | 0.89014 | 0.727877 | -0.13831 | 6.236226 |
| LOC100128 | 0.890594 | 0.728322 | 0.350725 | 2.376831 |
| MAPK14 | 0.890594 | 0.728384 | 0.271638 | 2.075709 |
| EXTL3 | 0.890682 | 0.728523 | 0.14881 | 4.645063 |
| COL22A1 | 0.89071 | 0.728733 | 0.333161 | 2.651194 |
| NRDE2 | 0.89071 | 0.728739 | -0.13502 | 5.142073 |
| CRYBA1 | 0.89071 | 0.72875 | 0.394509 | 0.951974 |
| MIR1281 | 0.890923 | 0.728992 | 0.249838 | 2.659761 |
| FYTTD1 | 0.891084 | 0.729192 | 0.219769 | 3.135888 |
| LOC101927 | 0.891326 | 0.729485 | -0.4167 | 0.854435 |
| ANKHD1 | 0.891326 | 0.729526 | 0.331503 | 2.620869 |
| LINC01559 | 0.891479 | 0.72972 | -0.34022 | 4.693263 |
| SNORD10 | 0.891634 | 0.730041 | 0.153679 | 10.84439 |
| METTL2B | 0.891634 | 0.730051 | 0.154484 | 4.93119 |
| ZNF628 | 0.891634 | 0.730061 | -0.26377 | 3.11152 |
| PDXK | 0.891634 | 0.730118 | -0.13342 | 7.686506 |
| UBA5 | 0.891645 | 0.730195 | -0.17082 | 4.320855 |
| DCP1A | 0.89185 | 0.730455 | 0.480094 | 1.130106 |
| UQCRH | 0.89185 | 0.730499 | 0.459695 | 0.645864 |
| UBE2J1 | 0.891888 | 0.730598 | 0.13856 | 6.726213 |
| FSIP1 | 0.891976 | 0.730799 | 0.530399 | 0.488384 |
| EPHB4 | 0.891976 | 0.730884 | -0.15474 | 6.522138 |
| RNF5 | 0.891976 | 0.730941 | 0.341087 | 1.759054 |
| SORBS1 | 0.891976 | 0.730942 | 0.433553 | 1.202278 |
| LOC101927 | 0.89238 | 0.731389 | 0.591112 | 1.169278 |

| | | | | |
|---|---|---|---|---|
| RPTOR | 0.89238 | 0.731467 | 0.232311 | 1.997917 |
| LINC01637 | 0.89238 | 0.731477 | 0.442772 | 0.691069 |
| LINC02256 | 0.892423 | 0.73158 | -0.27645 | 2.611936 |
| YPEL4 | 0.892451 | 0.731689 | 0.323406 | 2.386702 |
| ZNF593 | 0.892451 | 0.73178 | 0.308731 | 3.067659 |
| BOC | 0.892451 | 0.731807 | 0.314559 | 3.098098 |
| TOB1-AS1 | 0.893763 | 0.732983 | -0.54299 | 0.420908 |
| MIR6504 | 0.893763 | 0.733019 | -0.52126 | 1.08628 |
| STX18 | 0.893797 | 0.733144 | -0.14798 | 5.19646 |
| EIF4G3 | 0.893797 | 0.733183 | -0.23085 | 3.32671 |
| SOX13 | 0.89389 | 0.733328 | 0.17502 | 5.491396 |
| SWAP70 | 0.894103 | 0.73357 | -0.31798 | 1.39114 |
| MRI1 | 0.894114 | 0.733648 | 0.272477 | 3.188286 |
| TTBK2 | 0.894116 | 0.733718 | 0.145413 | 5.331965 |
| SPRYD3 | 0.894195 | 0.733858 | 0.131827 | 6.560083 |
| LINC00672 | 0.894195 | 0.733919 | 0.22972 | 2.414931 |
| COPS8 | 0.894523 | 0.734256 | -0.42447 | 0.741451 |
| PSMG2 | 0.894569 | 0.734362 | -0.44777 | 0.626166 |
| LOC100506 | 0.894703 | 0.734616 | 0.259357 | 2.466039 |
| TMEM173 | 0.894703 | 0.734628 | 0.373594 | 1.27505 |
| DNASE1 | 0.894703 | 0.734711 | -0.16169 | 4.333684 |
| NUP98 | 0.894703 | 0.734745 | 0.393404 | 1.028367 |
| SAT2 | 0.894797 | 0.734983 | 0.151797 | 4.702324 |
| LOC399715 | 0.894797 | 0.735007 | -0.24728 | 3.259332 |
| MANEA | 0.894797 | 0.735026 | 0.143277 | 5.000087 |
| KRT42P | 0.894925 | 0.7352 | -0.46261 | 1.036394 |
| COMMD2 | 0.895126 | 0.73547 | 0.141377 | 5.170599 |
| CHST14 | 0.895126 | 0.735502 | 0.252166 | 2.719095 |
| NAPA-AS1 | 0.895252 | 0.735673 | -0.23521 | 3.041872 |
| UCK2 | 0.895715 | 0.736169 | -0.14 | 5.63361 |
| CHM | 0.895715 | 0.73619 | 0.132974 | 5.356119 |
| TSNAX | 0.895716 | 0.736259 | -0.15572 | 5.239406 |
| ZRANB2 | 0.895749 | 0.736355 | 0.336603 | 2.123688 |
| SAPCD2 | 0.895964 | 0.736644 | 0.178764 | 4.436379 |
| MOV10 | 0.895964 | 0.736724 | 0.28083 | 1.832151 |
| SNORA1 | 0.895964 | 0.736736 | -0.2306 | 4.978942 |
| RBBP9 | 0.89599 | 0.736826 | -0.15126 | 4.822816 |
| MPRIP | 0.896182 | 0.737052 | 0.352632 | 1.641888 |
| ZNF286B | 0.896225 | 0.737156 | -0.27028 | 2.438838 |
| DDOST | 0.896267 | 0.737276 | 0.125124 | 6.641088 |
| FBXO38 | 0.896267 | 0.737327 | 0.256493 | 2.746031 |
| NMD3 | 0.896511 | 0.737596 | 0.121319 | 6.51898 |
| TLE4 | 0.896525 | 0.737676 | -0.30437 | 1.321717 |
| SNORA44 | 0.897115 | 0.738229 | 0.18213 | 6.956548 |
| DBF4B | 0.897367 | 0.738506 | 0.321516 | 1.946881 |

| | | | | |
|---|---|---|---|---|
| VMP1 | 0.897397 | 0.738598 | -0.13895 | 8.319818 |
| LOC90246 | 0.89745 | 0.738842 | 0.463163 | 1.047612 |
| FPGT | 0.89745 | 0.738845 | -0.31729 | 2.179303 |
| LOC10192‹ | 0.89745 | 0.738847 | -0.36974 | 1.647824 |
| ADAM20P1 | 0.897452 | 0.738917 | -0.22971 | 3.019374 |
| ZFYVE28 | 0.897559 | 0.739073 | 0.421241 | 1.094286 |
| FARSA | 0.897695 | 0.739254 | -0.12553 | 5.438153 |
| CLDN12 | 0.897707 | 0.739332 | 0.426581 | 0.920946 |
| HEY2 | 0.897883 | 0.739595 | 0.290858 | 2.18442 |
| USP48 | 0.897883 | 0.739693 | 0.11613 | 6.7487 |
| CLCN5 | 0.897883 | 0.73972 | -0.44957 | 0.714631 |
| LOC101927 | 0.897883 | 0.739751 | -0.23495 | 3.795675 |
| COX17 | 0.897904 | 0.739891 | -0.17864 | 4.540651 |
| ST20-AS1 | 0.897904 | 0.73994 | -0.37597 | 1.327583 |
| AP1S1 | 0.897904 | 0.739974 | 0.155914 | 4.6556 |
| HSD17B1 | 0.898037 | 0.740151 | 0.221817 | 3.42529 |
| FAM13A | 0.898079 | 0.740255 | 0.257294 | 2.837398 |
| LMNA | 0.898304 | 0.740509 | -0.29885 | 2.86496 |
| SLC16A14 | 0.898315 | 0.740587 | 0.209986 | 3.476624 |
| BRIX1 | 0.898377 | 0.740734 | -0.17845 | 4.87449 |
| RUVBL1 | 0.898377 | 0.740774 | 0.130464 | 5.553244 |
| CGRRF1 | 0.898402 | 0.740915 | -0.327 | 2.304935 |
| ZNF528-AS | 0.898402 | 0.740932 | -0.37201 | 2.166992 |
| ASNSD1 | 0.898963 | 0.74148 | 0.134183 | 5.09422 |
| MALSU1 | 0.898963 | 0.741603 | 0.165968 | 3.895201 |
| UBE2G2 | 0.898963 | 0.741612 | 0.427742 | 0.835343 |
| INTU | 0.898963 | 0.741669 | 0.1375 | 5.001503 |
| FAM117B | 0.899069 | 0.741824 | -0.1299 | 5.423422 |
| TMEM192 | 0.899161 | 0.741972 | 0.173556 | 4.594363 |
| ZBTB46-AS | 0.899161 | 0.742037 | -0.35018 | 1.546695 |
| RAPGEF4 | 0.89945 | 0.742412 | 0.453578 | 0.77295 |
| ZNF491 | 0.89945 | 0.742456 | 0.381241 | 2.000161 |
| ADI1 | 0.89945 | 0.742491 | 0.215567 | 3.311097 |
| SERINC2 | 0.89945 | 0.74255 | -0.20654 | 3.530318 |
| AGBL5 | 0.899466 | 0.742632 | -0.38391 | 1.107515 |
| LINC02615 | 0.899601 | 0.742814 | 0.284468 | 3.498875 |
| LINC02586 | 0.899601 | 0.74288 | -0.58032 | 1.079585 |
| JPT2 | 0.899623 | 0.742976 | 0.140236 | 7.152978 |
| MUL1 | 0.899623 | 0.743035 | 0.133799 | 4.632717 |
| FBXW11 | 0.89976 | 0.743289 | 0.363282 | 1.15328 |
| IGFBP6 | 0.89976 | 0.743364 | -0.25838 | 4.692102 |
| SCP2 | 0.89976 | 0.743417 | 0.252693 | 2.837633 |
| ARHGAP24 | 0.89976 | 0.743441 | -0.28594 | 2.989413 |
| GADD45GI | 0.89976 | 0.743491 | -0.13951 | 5.149654 |
| SEMA5A | 0.899824 | 0.743614 | -0.14003 | 6.041761 |

| | | | | |
|---|---|---|---|---|
| ZNF488 | 0.899875 | 0.743724 | 0.306883 | 2.121591 |
| DNTTIP2 | 0.899911 | 0.743959 | -0.12629 | 6.677714 |
| OGDH | 0.899911 | 0.744025 | 0.472492 | 0.870416 |
| TMEM72 | 0.899911 | 0.744055 | 0.406044 | 1.261585 |
| CISD1 | 0.899911 | 0.744066 | 0.185941 | 3.989814 |
| MTMR4 | 0.899911 | 0.744097 | -0.12451 | 6.278253 |
| MED10 | 0.899955 | 0.744261 | 0.18498 | 4.11818 |
| DDTL | 0.899955 | 0.744315 | -0.2959 | 2.628457 |
| CDC123 | 0.899955 | 0.744339 | -0.13558 | 5.733776 |
| FGFR1 | 0.900163 | 0.74458 | 0.478287 | 0.48253 |
| FUBP3 | 0.900179 | 0.744729 | -0.12389 | 6.786064 |
| FLJ32255 | 0.900179 | 0.74473 | -0.28753 | 2.888881 |
| MRPL39 | 0.900183 | 0.744801 | 0.386604 | 0.957775 |
| PPM1J | 0.900207 | 0.74489 | -0.41994 | 1.093542 |
| B9D1 | 0.900253 | 0.745078 | -0.40555 | 1.032759 |
| MED28 | 0.900253 | 0.745097 | -0.11729 | 5.917164 |
| SMIM7 | 0.900253 | 0.745134 | 0.141674 | 4.967336 |
| DMPK | 0.90038 | 0.745458 | 0.272456 | 2.305679 |
| LINC02613 | 0.90038 | 0.745642 | 0.570518 | 0.454279 |
| LEMD1-AS1 | 0.90038 | 0.745693 | 0.416567 | 1.050094 |
| SCARNA18 | 0.90038 | 0.745716 | -0.26144 | 2.431426 |
| CHRM5 | 0.90038 | 0.745773 | -0.43357 | 0.864069 |
| KLC4 | 0.90038 | 0.745851 | 0.439561 | 0.495144 |
| LRRC2 | 0.90038 | 0.745873 | 0.354129 | 1.950844 |
| CLNS1A | 0.90038 | 0.745951 | 0.543107 | 0.379923 |
| UBL4A | 0.90038 | 0.745961 | 0.154296 | 4.306836 |
| GAL3ST4 | 0.90038 | 0.745986 | -0.21278 | 3.189917 |
| NICN1 | 0.90038 | 0.746125 | -0.16517 | 4.30548 |
| COPRS | 0.90038 | 0.746173 | 0.207495 | 3.77076 |
| ORAI3 | 0.90038 | 0.746179 | 0.18272 | 3.645136 |
| GTPBP2 | 0.90038 | 0.7462 | -0.18013 | 3.838582 |
| LOC100506 | 0.900391 | 0.746277 | 0.421496 | 1.08693 |
| C16orf86 | 0.900643 | 0.746607 | -0.3189 | 1.540943 |
| RNVU1-7 | 0.900643 | 0.746685 | -0.13347 | 7.135179 |
| LOC101927 | 0.900643 | 0.746706 | -0.23834 | 2.759653 |
| FAM53B-AS | 0.900643 | 0.746761 | -0.45236 | 1.285115 |
| GMCL1 | 0.900904 | 0.747083 | 0.133013 | 4.916475 |
| MED21 | 0.900904 | 0.747115 | -0.48835 | 0.752878 |
| NUTM2B-A | 0.900918 | 0.747251 | 0.138082 | 5.880534 |
| EIF3A | 0.900918 | 0.747264 | -0.1333 | 10.22049 |
| XIST | 0.900934 | 0.747345 | 0.717382 | 8.802706 |
| RPS24 | 0.901023 | 0.747488 | -0.41857 | 1.020996 |
| HNRNPUL1 | 0.90105 | 0.747579 | 0.158071 | 3.951796 |
| SNORD133 | 0.901158 | 0.747737 | 0.133116 | 8.901633 |
| MACROD2 | 0.901315 | 0.748001 | -0.2855 | 2.357295 |

| | | | | |
|---|---|---|---|---|
| BIRC6-AS2 | 0.901315 | 0.748058 | -0.31926 | 2.1748 |
| GGA1 | 0.901315 | 0.748073 | -0.29188 | 1.744392 |
| CCDC134 | 0.90147 | 0.74834 | 0.38579 | 1.733726 |
| RNVU1-15 | 0.90147 | 0.748376 | 0.341861 | 1.662575 |
| MIR4435-2 | 0.90147 | 0.748408 | 0.240896 | 3.286868 |
| NXPE1 | 0.901487 | 0.748606 | -0.45446 | 1.343968 |
| SLC25A24 | 0.901487 | 0.748624 | 0.234728 | 1.871253 |
| RPS11 | 0.901487 | 0.748705 | -0.12241 | 8.961044 |
| SLC33A1 | 0.901487 | 0.748733 | 0.197588 | 3.479933 |
| SLF1 | 0.901487 | 0.748766 | 0.152043 | 4.511468 |
| LOC10004 | 0.90168 | 0.748995 | 0.268775 | 3.039454 |
| RPL31 | 0.901705 | 0.749155 | 0.23768 | 3.427025 |
| SUZ12 | 0.901705 | 0.749205 | 0.122468 | 6.890916 |
| APCDD1L-I | 0.901705 | 0.749273 | -0.50032 | 0.439628 |
| DCAF13 | 0.901705 | 0.749323 | 0.13187 | 5.660345 |
| RPS15AP1( | 0.901705 | 0.74936 | 0.222106 | 3.659285 |
| LOC10028 | 0.901786 | 0.7495 | 0.283213 | 2.18715 |
| CCDC85B | 0.901786 | 0.749728 | 0.158727 | 4.577794 |
| BACE1-AS | 0.901786 | 0.749786 | -0.42337 | 1.140958 |
| FLJ45513 | 0.901786 | 0.749822 | 0.327409 | 1.411355 |
| TBC1D9 | 0.901786 | 0.749846 | -0.11851 | 5.985235 |
| KCNMB4 | 0.901786 | 0.749853 | 0.378051 | 1.67216 |
| ZDHHC7 | 0.901786 | 0.749907 | 0.372689 | 0.539096 |
| LINC01184 | 0.901807 | 0.75013 | -0.16487 | 5.303741 |
| THOP1 | 0.901807 | 0.750165 | 0.140372 | 4.892598 |
| GPATCH2L | 0.901807 | 0.750166 | -0.42215 | 1.128282 |
| HCN3 | 0.901807 | 0.7502 | -0.34283 | 0.708767 |
| PTPN2 | 0.901844 | 0.750354 | 0.293795 | 2.155952 |
| BHLHE22 | 0.901844 | 0.750369 | 0.371141 | 1.627238 |
| PBX2 | 0.901946 | 0.75054 | -0.17824 | 3.716709 |
| INTS6-AS1 | 0.901946 | 0.750654 | 0.291012 | 2.457298 |
| RRP1B | 0.901946 | 0.750659 | 0.133856 | 6.453214 |
| STARD4 | 0.901978 | 0.750755 | -0.66137 | 0.329674 |
| ATXN7L3B | 0.901982 | 0.750827 | -0.14245 | 6.623369 |
| PIN4P1 | 0.902035 | 0.750939 | 0.291665 | 2.047986 |
| CWC15 | 0.902101 | 0.751064 | -0.12064 | 5.64478 |
| WWP1 | 0.902101 | 0.751198 | 0.113 | 7.109843 |
| ATP5MD | 0.902101 | 0.751201 | -0.28792 | 2.073551 |
| RASAL2-AS | 0.902164 | 0.751322 | 0.363683 | 1.235693 |
| HIBCH | 0.902287 | 0.751493 | -0.43903 | 0.416078 |
| ESD | 0.902307 | 0.751627 | -0.14742 | 4.767561 |
| WDR11 | 0.902307 | 0.751647 | 0.120463 | 6.900477 |
| EXOG | 0.90233 | 0.751736 | 0.487695 | 1.088471 |
| C11orf71 | 0.902347 | 0.751852 | 0.330237 | 2.200687 |
| SNORA71C | 0.902347 | 0.751927 | -0.13954 | 5.363893 |

| | | | | |
|---|---|---|---|---|
| ESCO1 | 0.902347 | 0.751956 | 0.137067 | 6.683394 |
| DMTF1 | 0.902865 | 0.752457 | 0.217238 | 3.453378 |
| MAN1B1-D | 0.903013 | 0.752654 | -0.3442 | 0.82509 |
| NUDT5 | 0.903013 | 0.752751 | -0.15168 | 4.649693 |
| PSMD6 | 0.903013 | 0.752786 | 0.345503 | 1.820142 |
| TRIM38 | 0.903389 | 0.753168 | 0.120884 | 7.075163 |
| USP1 | 0.903464 | 0.753299 | -0.31141 | 1.152445 |
| MYH6 | 0.903526 | 0.753466 | 0.517886 | 0.897936 |
| PSAP | 0.903526 | 0.753491 | 0.431813 | 1.290593 |
| CSNK2A3 | 0.903526 | 0.753558 | 0.128285 | 5.27907 |
| PCK2 | 0.903539 | 0.753637 | -0.26881 | 1.362907 |
| RABL3 | 0.903609 | 0.753792 | -0.13612 | 5.029815 |
| ZNF767P | 0.903609 | 0.753945 | 0.136674 | 4.711966 |
| ODF3L1 | 0.903609 | 0.754108 | -0.51282 | 0.422975 |
| IRAIN | 0.903609 | 0.754122 | -0.31009 | 2.673588 |
| CCNB1IP1 | 0.903609 | 0.75416 | -0.33736 | 1.193949 |
| OCLM | 0.903609 | 0.754267 | -0.32134 | 3.148559 |
| RABL6 | 0.903609 | 0.754278 | 0.185519 | 3.404259 |
| PSMB7 | 0.903609 | 0.754289 | -0.14949 | 5.17643 |
| TIMM8B | 0.903609 | 0.754316 | -0.45295 | 0.677686 |
| ZNF518B | 0.903717 | 0.75454 | -0.14638 | 5.28493 |
| NECTIN2 | 0.903717 | 0.754549 | -0.12285 | 5.797175 |
| ATPAF2 | 0.903717 | 0.754612 | 0.21525 | 4.117127 |
| NAT9 | 0.903732 | 0.754694 | -0.45508 | 1.104284 |
| TRIM25 | 0.903748 | 0.754801 | 0.110469 | 6.985579 |
| NEIL1 | 0.903748 | 0.754845 | 0.395026 | 1.11134 |
| YIPF2 | 0.904387 | 0.755448 | 0.134459 | 5.247216 |
| PQLC2 | 0.904985 | 0.756016 | 0.344188 | 0.64447 |
| RN7SK | 0.905257 | 0.756363 | -0.11458 | 14.504 |
| EGR1 | 0.905257 | 0.756385 | -0.23071 | 8.29002 |
| IL11RA | 0.905257 | 0.756451 | 0.331669 | 0.681562 |
| MAP3K8 | 0.905304 | 0.756597 | -0.24969 | 2.603846 |
| ENAM | 0.905304 | 0.756628 | 0.436395 | 1.534558 |
| LTBP1 | 0.905328 | 0.756839 | 0.255044 | 2.303629 |
| C9orf78 | 0.905328 | 0.756864 | -0.1234 | 6.329116 |
| SNX18 | 0.905328 | 0.756993 | -0.41425 | 0.762531 |
| EME2 | 0.905328 | 0.757068 | 0.19986 | 3.095882 |
| POU5F1P3 | 0.905328 | 0.757086 | 0.424693 | 0.865038 |
| OXER1 | 0.905328 | 0.75721 | 0.256178 | 2.301239 |
| OSBPL11 | 0.905328 | 0.757211 | -0.13334 | 5.386545 |
| CCNL1 | 0.905328 | 0.757217 | 0.136501 | 5.649779 |
| CFAP53 | 0.905328 | 0.757351 | -0.32376 | 1.963233 |
| TTC28-AS1 | 0.905328 | 0.757359 | -0.19894 | 3.70393 |
| PCDH7 | 0.905328 | 0.757406 | 0.375775 | 1.175595 |
| MIRLET7F1 | 0.905558 | 0.757668 | 0.284331 | 2.591231 |

| | | | | |
|---|---|---|---|---|
| BRD9 | 0.905649 | 0.757817 | 0.226424 | 2.178968 |
| ZNF157 | 0.905649 | 0.757882 | 0.511508 | 0.430049 |
| EIF3L | 0.905661 | 0.757962 | -0.26368 | 1.91695 |
| TCOF1 | 0.905687 | 0.758052 | 0.326404 | 1.469073 |
| CAND1.11 | 0.905915 | 0.758428 | -0.37602 | 1.664459 |
| FZD7 | 0.905915 | 0.758456 | -0.16784 | 4.732952 |
| CSRP3 | 0.905915 | 0.758518 | -0.49007 | 0.733921 |
| NUP43 | 0.905915 | 0.758519 | -0.19762 | 3.139336 |
| RDH8 | 0.906007 | 0.758665 | 0.661383 | 0.552818 |
| COQ3 | 0.906386 | 0.759052 | -0.19577 | 2.939201 |
| HBS1L | 0.90639 | 0.759124 | -0.17184 | 3.494768 |
| C10orf95 | 0.906563 | 0.759338 | 0.403289 | 1.076882 |
| SNORA18 | 0.906636 | 0.759491 | 0.133416 | 7.258486 |
| SPRY4-AS1 | 0.906636 | 0.759645 | 0.468468 | 0.60918 |
| SLCO4C1 | 0.906636 | 0.759656 | 0.394693 | 0.924964 |
| TRIM59 | 0.906636 | 0.759692 | -0.14553 | 4.324757 |
| NAP1L2 | 0.906636 | 0.759744 | 0.318597 | 1.820468 |
| LOC642361 | 0.906736 | 0.759897 | 0.346502 | 0.896965 |
| LINC02141 | 0.906905 | 0.760214 | 0.57275 | 0.76264 |
| DNAH17 | 0.906905 | 0.760272 | 0.179041 | 3.23918 |
| PDK1 | 0.906905 | 0.760291 | 0.356454 | 1.26154 |
| HPS6 | 0.906905 | 0.760315 | 0.155956 | 4.155321 |
| PKD1L1 | 0.906948 | 0.760439 | -0.23441 | 2.741879 |
| STXBP3 | 0.906948 | 0.760645 | 0.109882 | 5.947455 |
| C9orf72 | 0.906948 | 0.760669 | 0.231039 | 2.534722 |
| AASS | 0.906948 | 0.760713 | 0.150142 | 6.433988 |
| UBE2E3 | 0.906948 | 0.760846 | 0.256419 | 2.092664 |
| PIGU | 0.906948 | 0.760884 | -0.14656 | 3.978812 |
| PIK3C2A | 0.906948 | 0.760897 | 0.107624 | 7.897449 |
| HIST1H2AM | 0.906948 | 0.760905 | -0.21119 | 4.534915 |
| PRKD3 | 0.907116 | 0.761123 | 0.109414 | 6.25326 |
| SNORD143 | 0.907116 | 0.761245 | 0.306478 | 1.969787 |
| IDH3B | 0.907116 | 0.761253 | -0.24006 | 2.63186 |
| LRMDA | 0.907132 | 0.761336 | -0.31972 | 2.318476 |
| FRK | 0.907202 | 0.761464 | 0.122316 | 6.064586 |
| KIAA0513 | 0.907288 | 0.761614 | -0.30906 | 1.535033 |
| DEPDC5 | 0.907288 | 0.761674 | -0.43144 | 0.321757 |
| LTN1 | 0.907383 | 0.761853 | -0.10585 | 6.925562 |
| TREM1 | 0.907383 | 0.761911 | 0.508544 | 1.041841 |
| MORF4L2-A | 0.907383 | 0.761976 | 0.374927 | 1.410932 |
| VTRNA1-3 | 0.907383 | 0.76203 | 0.2304 | 4.030693 |
| TLK1 | 0.907431 | 0.76214 | 0.188947 | 3.565543 |
| ZCCHC8 | 0.907582 | 0.762336 | -0.12591 | 5.571077 |
| XRCC4 | 0.907742 | 0.762539 | -0.36764 | 0.596551 |
| SDC4 | 0.908186 | 0.763035 | -0.11359 | 8.449247 |

| | | | | |
|---|---|---|---|---|
| DNAH10 | 0.908186 | 0.763051 | 0.401707 | 1.273212 |
| LOC10012‹ | 0.908267 | 0.763188 | -0.20461 | 2.727957 |
| PCP2 | 0.908336 | 0.763316 | 0.397333 | 1.229122 |
| XPNPEP3 | 0.908336 | 0.763384 | -0.12192 | 5.958418 |
| DBH | 0.908492 | 0.763585 | -0.52002 | 0.807593 |
| ABL1 | 0.908687 | 0.763818 | -0.18227 | 3.089372 |
| SLITRK6 | 0.908695 | 0.764044 | 0.290266 | 3.367104 |
| FAM180A | 0.908695 | 0.764052 | 0.304407 | 2.60161 |
| MRPL45P2 | 0.908695 | 0.764057 | -0.18369 | 3.867748 |
| FAM86FP | 0.908695 | 0.764102 | 0.313282 | 1.784516 |
| RPS20 | 0.908829 | 0.764284 | -0.26767 | 1.989884 |
| IARS | 0.908844 | 0.764419 | 0.461341 | 0.564707 |
| NMT1 | 0.908844 | 0.764436 | 0.106335 | 6.706322 |
| PYROXD1 | 0.908844 | 0.764503 | -0.1558 | 4.830637 |
| ACOX2 | 0.908846 | 0.764575 | 0.271617 | 2.150547 |
| KIF26A | 0.909101 | 0.764859 | 0.290944 | 3.05456 |
| TRIR | 0.90917 | 0.765065 | -0.10997 | 6.984176 |
| TMEM86B | 0.90917 | 0.765096 | -0.16019 | 3.932446 |
| PDGFB | 0.90917 | 0.765124 | 0.330796 | 1.909222 |
| TAOK1 | 0.909234 | 0.765248 | -0.19281 | 4.421651 |
| CBR4 | 0.909396 | 0.765453 | -0.12507 | 5.19664 |
| SGMS1-AS: | 0.909429 | 0.76555 | -0.31914 | 1.679253 |
| SLC4A11 | 0.909486 | 0.765722 | 0.274188 | 2.798391 |
| CPSF4 | 0.909486 | 0.76577 | -0.37557 | 0.727022 |
| SLC35A3 | 0.909486 | 0.765806 | -0.39631 | 0.447311 |
| SNTB2 | 0.909635 | 0.766061 | 0.127043 | 6.704745 |
| MYOSLID-A | 0.909635 | 0.76609 | 0.373652 | 0.555626 |
| IER3IP1 | 0.909635 | 0.76614 | 0.14763 | 5.790674 |
| HOXA4 | 0.909641 | 0.766214 | -0.23217 | 2.62109 |
| APOB | 0.909735 | 0.766384 | 0.322194 | 2.425972 |
| ABTB2 | 0.909735 | 0.766432 | 0.136466 | 4.736941 |
| SNORA23 | 0.909796 | 0.766586 | -0.12554 | 8.375977 |
| ZXDC | 0.909796 | 0.766668 | -0.13747 | 4.863108 |
| CYFIP1 | 0.909796 | 0.766739 | -0.28245 | 2.295885 |
| APPL2 | 0.909796 | 0.766823 | 0.461827 | 0.972585 |
| RALGPS1 | 0.909796 | 0.766865 | 0.138321 | 4.387841 |
| FANCG | 0.909796 | 0.7669 | 0.151105 | 4.000377 |
| SNORA66 | 0.909951 | 0.767099 | -0.18898 | 5.246805 |
| MICU1 | 0.910078 | 0.767275 | 0.399327 | 1.100952 |
| HDAC6 | 0.910243 | 0.767484 | 0.111546 | 6.130226 |
| PKNOX2 | 0.910307 | 0.767608 | 0.340331 | 2.03252 |
| MIR5192 | 0.910317 | 0.767685 | -0.35237 | 1.866828 |
| SEPSECS | 0.910549 | 0.767951 | -0.15325 | 4.638981 |
| POP5 | 0.910557 | 0.768027 | 0.424252 | 0.584935 |
| PEX7 | 0.910632 | 0.768265 | 0.27334 | 2.887904 |

| | | | | |
|---|---|---|---|---|
| MGC15885 | 0.910632 | 0.768327 | 0.395627 | 1.792851 |
| MED25 | 0.910632 | 0.768362 | 0.110277 | 5.522888 |
| PTGER3 | 0.910632 | 0.768368 | -0.35009 | 4.008905 |
| LMTK2 | 0.910962 | 0.768715 | -0.12879 | 7.751659 |
| ZNF148 | 0.911043 | 0.768853 | 0.107187 | 7.322457 |
| GGNBP2 | 0.91105 | 0.768959 | -0.22663 | 2.524879 |
| C19orf48 | 0.91105 | 0.768998 | -0.36112 | 1.623388 |
| IL1R1 | 0.911055 | 0.769071 | -0.30277 | 1.27602 |
| PXDNL | 0.911372 | 0.769409 | 0.359931 | 1.042699 |
| AVEN | 0.911392 | 0.769495 | -0.19243 | 4.168138 |
| TCFL5 | 0.911449 | 0.769613 | -0.22311 | 3.014339 |
| SLC36A4 | 0.911459 | 0.76969 | 0.302011 | 1.269114 |
| FAM216A | 0.911676 | 0.769949 | -0.19994 | 2.679134 |
| RSBN1L | 0.911676 | 0.770012 | 0.10862 | 5.757348 |
| MIR3941 | 0.912125 | 0.770461 | 0.352123 | 0.871206 |
| LINC02198 | 0.912234 | 0.770623 | 0.556158 | 1.118616 |
| MEX3C | 0.912241 | 0.770698 | 0.112188 | 5.782075 |
| HIST1H4L | 0.912241 | 0.770829 | -0.2412 | 3.836417 |
| CCND3 | 0.912241 | 0.770949 | 0.28463 | 2.215174 |
| ST7-OT4 | 0.912241 | 0.770961 | 0.234301 | 2.761751 |
| LARP1B | 0.912241 | 0.771003 | 0.13179 | 4.187367 |
| ATAD2 | 0.912241 | 0.771107 | 0.119691 | 6.727677 |
| APOBEC2 | 0.912241 | 0.771153 | 0.277205 | 2.208511 |
| LINC00598 | 0.912241 | 0.771225 | 0.370874 | 0.997885 |
| BCCIP | 0.912241 | 0.771292 | 0.165225 | 3.964199 |
| FLAD1 | 0.912241 | 0.771335 | 0.19932 | 2.553469 |
| CD99P1 | 0.912241 | 0.771394 | 0.437672 | 0.926233 |
| ZSCAN12 | 0.912268 | 0.771486 | 0.257122 | 2.451867 |
| HOXA-AS2 | 0.912286 | 0.771594 | 0.277675 | 1.408354 |
| SNIP1 | 0.912286 | 0.77164 | 0.123324 | 5.328551 |
| FRG1BP | 0.912365 | 0.771777 | 0.112851 | 5.14471 |
| TOP2B | 0.912577 | 0.772026 | -0.11197 | 8.336953 |
| ERGIC2 | 0.912766 | 0.772255 | -0.09957 | 5.892367 |
| LRRC58 | 0.913011 | 0.772588 | 0.116679 | 6.862474 |
| TLL1 | 0.913011 | 0.772688 | 0.362235 | 1.384259 |
| DIPK1B | 0.913011 | 0.77271 | -0.18259 | 2.698598 |
| SLC25A4 | 0.913011 | 0.772741 | -0.18778 | 4.926776 |
| ATAD2B | 0.913071 | 0.772914 | -0.36009 | 0.402443 |
| CH25H | 0.913071 | 0.773122 | -0.33217 | 2.023013 |
| KIAA1109 | 0.913071 | 0.773128 | 0.105684 | 8.698295 |
| MTREX | 0.913071 | 0.773137 | -0.10213 | 7.104553 |
| GPR89B | 0.913071 | 0.77321 | -0.19528 | 2.786516 |
| RRS1-AS1 | 0.913071 | 0.773217 | -0.34167 | 2.331149 |
| PLGLA | 0.913071 | 0.773278 | -0.39993 | 0.415704 |
| LOC10013( | 0.913159 | 0.77347 | 0.177102 | 2.782679 |

| | | | | |
|---|---|---|---|---|
| LCT | 0.913159 | 0.773492 | 0.438353 | 0.803828 |
| CHCHD6 | 0.913207 | 0.773602 | -0.24178 | 2.993434 |
| ZNF883 | 0.913265 | 0.773731 | -0.25549 | 2.349094 |
| ZNF672 | 0.913265 | 0.77379 | -0.12554 | 5.056839 |
| NOTCH1 | 0.913443 | 0.774018 | -0.12635 | 6.984488 |
| MAP1LC3B | 0.913443 | 0.774081 | -0.22677 | 2.736988 |
| GAPDHS | 0.9136 | 0.774295 | -0.17776 | 2.938283 |
| ICA1L | 0.9136 | 0.774353 | 0.444448 | 0.987999 |
| HERC1 | 0.91362 | 0.774442 | -0.1001 | 8.491034 |
| LINC01000 | 0.91362 | 0.774509 | -0.1096 | 8.156714 |
| HIST1H4J | 0.913835 | 0.774795 | -0.26093 | 1.85125 |
| DIP2A | 0.913835 | 0.774845 | 0.276419 | 1.265747 |
| EMC9 | 0.913835 | 0.774916 | 0.23588 | 2.510009 |
| EIF2A | 0.913835 | 0.775059 | -0.1015 | 6.731758 |
| AATK | 0.913835 | 0.775154 | 0.423128 | 0.750411 |
| HTR7P1 | 0.913835 | 0.775266 | -0.26022 | 2.30128 |
| HJURP | 0.913835 | 0.775284 | 0.400279 | 0.752559 |
| PRKACA | 0.913835 | 0.775293 | -0.20684 | 2.610773 |
| MDN1 | 0.913835 | 0.775318 | 0.108799 | 8.679126 |
| ZMYND19 | 0.913838 | 0.775428 | 0.131128 | 4.650328 |
| MIR637 | 0.913838 | 0.775563 | 0.401158 | 0.531775 |
| ATP2B4 | 0.913838 | 0.775602 | 0.299656 | 1.587595 |
| E2F4 | 0.913838 | 0.775635 | -0.10377 | 6.005472 |
| NHEJ1 | 0.913838 | 0.775669 | 0.155349 | 3.867264 |
| NADK2 | 0.914233 | 0.776117 | -0.33468 | 1.537775 |
| SNAP23 | 0.914233 | 0.776176 | 0.288293 | 0.664812 |
| IARS2 | 0.914233 | 0.776215 | -0.11672 | 7.155491 |
| HIST1H3H | 0.914233 | 0.776307 | 0.209032 | 4.054299 |
| HOXA2 | 0.914233 | 0.776452 | 0.280742 | 2.266659 |
| ANKDD1A | 0.914233 | 0.776697 | 0.136294 | 4.166294 |
| ZNF83 | 0.914233 | 0.77682 | -0.24817 | 2.65663 |
| RAB5C | 0.914233 | 0.776824 | 0.333594 | 0.601159 |
| COPS5 | 0.914233 | 0.77699 | 0.110915 | 5.411615 |
| ALDH1A1 | 0.914233 | 0.776993 | -0.21625 | 7.195074 |
| LINC01671 | 0.914233 | 0.777066 | 0.458511 | 0.881822 |
| TADA1 | 0.914233 | 0.777084 | 0.158869 | 4.115518 |
| ZNF639 | 0.914233 | 0.77709 | -0.49192 | 0.456589 |
| SNORA71E | 0.914233 | 0.777107 | -0.1552 | 4.724609 |
| EZH1 | 0.914233 | 0.777205 | -0.10652 | 6.731506 |
| LINC00939 | 0.914233 | 0.777223 | 0.457447 | 0.71585 |
| HSD17B12 | 0.914233 | 0.777229 | 0.101087 | 6.697366 |
| RUNX1T1 | 0.914233 | 0.777261 | 0.551963 | 0.720966 |
| 6-Mar | 0.914233 | 0.777328 | 0.368128 | 0.891518 |
| SLC4A2 | 0.914395 | 0.777535 | 0.254838 | 2.147072 |
| ZNF324B | 0.914994 | 0.778204 | -0.16278 | 3.095677 |

| | | | | |
|---|---|---|---|---|
| PLEKHA8 | 0.914994 | 0.778282 | 0.108753 | 5.978108 |
| RNASEH2B | 0.914994 | 0.778405 | 0.274577 | 2.304358 |
| GPR15 | 0.914994 | 0.778637 | -0.54467 | 0.707181 |
| GBP4 | 0.914994 | 0.778782 | 0.165519 | 5.407327 |
| L3HYPDH | 0.914994 | 0.778809 | -0.21928 | 3.003032 |
| LINC00593 | 0.914994 | 0.778825 | 0.593197 | 0.542115 |
| GPSM1 | 0.914994 | 0.778926 | -0.28846 | 2.281132 |
| EAF1 | 0.914994 | 0.778934 | 0.106609 | 6.104753 |
| KCNK15-AS | 0.914994 | 0.779048 | 0.393247 | 0.627966 |
| WDYHV1 | 0.914994 | 0.779101 | 0.421868 | 0.726675 |
| MRPL27 | 0.914994 | 0.779109 | -0.14092 | 3.995145 |
| KIAA0753 | 0.914994 | 0.779131 | -0.10811 | 5.987729 |
| DAGLB | 0.914994 | 0.779233 | -0.28323 | 1.621368 |
| TP53RK | 0.914994 | 0.779255 | 0.137935 | 4.496354 |
| FURIN | 0.914994 | 0.779311 | -0.36213 | 0.435874 |
| GLMN | 0.914994 | 0.779317 | -0.14953 | 3.753031 |
| BCAT2 | 0.914994 | 0.77935 | 0.334073 | 1.056381 |
| KCTD1 | 0.914994 | 0.779369 | -0.15964 | 4.077354 |
| ZDHHC8P1 | 0.915166 | 0.779757 | 0.361817 | 0.614153 |
| ZSCAN26 | 0.915166 | 0.7798 | 0.416211 | 0.369845 |
| YJEFN3 | 0.915166 | 0.779821 | 0.413386 | 0.845489 |
| SACM1L | 0.915166 | 0.779919 | 0.102999 | 6.43771 |
| LRP2BP | 0.915166 | 0.77993 | -0.13857 | 4.463553 |
| EP400 | 0.915166 | 0.779934 | 0.103597 | 8.17918 |
| ZNF91 | 0.915313 | 0.78013 | 0.303977 | 1.551604 |
| SNORA74A | 0.915842 | 0.78065 | -0.12561 | 6.426576 |
| INSM1 | 0.915899 | 0.780799 | 0.550418 | 0.811435 |
| FGD3 | 0.915899 | 0.780838 | -0.37353 | 1.203978 |
| LOC10013: | 0.916248 | 0.781206 | 0.201396 | 2.123332 |
| HIST1H4B | 0.916365 | 0.78154 | 0.120093 | 4.879754 |
| FAM41C | 0.916365 | 0.781545 | -0.4189 | 0.692588 |
| ANXA11 | 0.916365 | 0.781637 | -0.15999 | 4.028382 |
| CCAT2 | 0.916365 | 0.781657 | 0.423305 | 0.717639 |
| USP47 | 0.916365 | 0.781709 | 0.386147 | 0.533187 |
| LINC00641 | 0.916365 | 0.78178 | -0.10396 | 5.37374 |
| SNORD65 | 0.916365 | 0.781794 | 0.299961 | 2.038702 |
| PON3 | 0.916517 | 0.78205 | 0.421362 | 0.814085 |
| TMBIM6 | 0.916517 | 0.782089 | 0.259232 | 1.870724 |
| CASP3 | 0.916517 | 0.782133 | -0.29732 | 1.273577 |
| CXCL6 | 0.917322 | 0.782891 | -0.44022 | 3.477 |
| GPR21 | 0.917411 | 0.783037 | 0.201868 | 3.435238 |
| LOC10527 | 0.917507 | 0.783263 | 0.375276 | 0.4843 |
| ANKLE2 | 0.917507 | 0.783278 | -0.10467 | 7.384688 |
| IGF2R | 0.917507 | 0.783392 | 0.097679 | 8.814749 |
| ARID5B | 0.917507 | 0.783398 | 0.139176 | 4.457003 |

| | | | | |
|---|---|---|---|---|
| SNORD88A | 0.917741 | 0.783727 | -0.32653 | 1.641769 |
| TSEN54 | 0.917741 | 0.783738 | 0.129696 | 4.562419 |
| AGPAT5 | 0.917801 | 0.783859 | -0.10876 | 5.922983 |
| COA4 | 0.91808 | 0.784167 | -0.14958 | 4.298864 |
| LYL1 | 0.918143 | 0.784399 | 0.280758 | 1.721023 |
| KDM6B | 0.918143 | 0.784455 | 0.106702 | 7.294477 |
| CCM2L | 0.918143 | 0.784497 | -0.28346 | 1.718069 |
| EVI5L | 0.918143 | 0.784501 | -0.39726 | 0.668878 |
| SESN3 | 0.918377 | 0.78492 | 0.243133 | 2.029629 |
| SNORD92 | 0.918377 | 0.784933 | 0.169369 | 3.053967 |
| BRMS1L | 0.918377 | 0.784976 | -0.12043 | 4.583823 |
| GPR52 | 0.918377 | 0.784983 | -0.25722 | 2.834293 |
| PIP5K1P1 | 0.918377 | 0.785092 | 0.372604 | 0.806094 |
| GSTM2 | 0.918377 | 0.785215 | -0.28783 | 1.612573 |
| CISD3 | 0.918377 | 0.785232 | 0.345373 | 0.679541 |
| CCDC186 | 0.918377 | 0.785271 | -0.10155 | 7.986203 |
| HNRNPUL2 | 0.918377 | 0.785342 | 0.104191 | 6.249535 |
| FUT8 | 0.918377 | 0.785401 | -0.27542 | 1.228171 |
| PPFIBP1 | 0.918578 | 0.785659 | -0.3349 | 0.551816 |
| HCG17 | 0.918578 | 0.785713 | -0.26811 | 1.770596 |
| ACTG1P20 | 0.91874 | 0.785949 | -0.15623 | 3.711578 |
| RRNAD1 | 0.91874 | 0.785991 | -0.34023 | 1.15064 |
| MAPK8IP3 | 0.918967 | 0.786255 | 0.363539 | 0.827965 |
| TRAF3IP1 | 0.919721 | 0.786971 | -0.28435 | 1.59161 |
| FKBP1AP1 | 0.919833 | 0.787136 | 0.114516 | 4.294579 |
| TDRD7 | 0.919961 | 0.78736 | -0.2242 | 1.983954 |
| DPH1 | 0.919961 | 0.787389 | -0.15039 | 3.835722 |
| CPPED1 | 0.919961 | 0.787578 | 0.25825 | 2.226415 |
| DPH6 | 0.919961 | 0.787587 | 0.1846 | 2.856204 |
| USE1 | 0.919961 | 0.787638 | 0.139942 | 3.321052 |
| ERVW-1 | 0.919961 | 0.787667 | 0.344682 | 1.148212 |
| BTBD9 | 0.920075 | 0.787835 | 0.308932 | 1.054576 |
| C11orf65 | 0.920169 | 0.788003 | -0.37165 | 0.999197 |
| HIST1H2AG | 0.920169 | 0.788055 | -0.13231 | 4.659314 |
| SIAH1 | 0.920192 | 0.788145 | -0.18582 | 2.444463 |
| PSMD12 | 0.920266 | 0.788279 | -0.15261 | 3.75899 |
| ST7-AS2 | 0.92028 | 0.788372 | 0.252492 | 2.907299 |
| LINC00582 | 0.92028 | 0.78843 | -0.43883 | 0.519474 |
| SCARA3 | 0.920343 | 0.788554 | -0.16429 | 3.94322 |
| PXMP2 | 0.920677 | 0.789071 | -0.2143 | 3.818838 |
| CCZ1 | 0.920677 | 0.789131 | 0.126718 | 5.352649 |
| TNFSF12 | 0.920677 | 0.789153 | -0.34416 | 0.816636 |
| RNVU1-14 | 0.920677 | 0.789194 | 0.24555 | 3.153577 |
| C7orf31 | 0.920677 | 0.78926 | 0.198713 | 3.14894 |
| MIR4691 | 0.920677 | 0.789297 | -0.43921 | 0.485242 |

| | | | | |
|---|---|---|---|---|
| RPP25 | 0.920677 | 0.789461 | 0.163732 | 3.648695 |
| CEP164 | 0.920677 | 0.789476 | 0.218716 | 2.683294 |
| NYAP2 | 0.920677 | 0.789483 | -0.33689 | 1.368791 |
| NCOA7-AS1 | 0.920677 | 0.789543 | 0.463431 | 0.793483 |
| ARHGEF7 | 0.920805 | 0.789723 | -0.26688 | 1.971315 |
| FAM153C | 0.921016 | 0.789986 | -0.38504 | 1.319185 |
| BIN2 | 0.921016 | 0.790087 | 0.464137 | 0.604869 |
| DPP9-AS1 | 0.921016 | 0.790114 | -0.17508 | 2.883182 |
| TOM1L1 | 0.921222 | 0.790361 | 0.105538 | 5.772249 |
| SNORA62 | 0.921587 | 0.790745 | -0.14557 | 7.959758 |
| UBN2 | 0.921877 | 0.791088 | -0.09713 | 7.80136 |
| POLR1C | 0.921877 | 0.791134 | 0.12555 | 4.397324 |
| TFB1M | 0.922328 | 0.791591 | 0.162856 | 4.029767 |
| TSC22D3 | 0.922355 | 0.791746 | 0.152445 | 5.555969 |
| BBS7 | 0.922355 | 0.791765 | -0.23925 | 1.879525 |
| RAD51B | 0.922355 | 0.791904 | -0.20822 | 2.652035 |
| TMEM131 | 0.922355 | 0.791957 | 0.092478 | 7.658368 |
| DARS2 | 0.922355 | 0.791992 | 0.100647 | 5.45607 |
| TTC6 | 0.922355 | 0.792105 | 0.218892 | 4.39416 |
| MRPS17 | 0.922355 | 0.792106 | 0.123072 | 4.540382 |
| YY1AP1 | 0.922357 | 0.792178 | -0.22905 | 2.484657 |
| ZNF580 | 0.922661 | 0.7926 | 0.227254 | 1.99738 |
| AADAC | 0.922661 | 0.792645 | 0.310799 | 2.677962 |
| ZNF248 | 0.922661 | 0.79265 | -0.28332 | 1.883452 |
| CHP2 | 0.922728 | 0.792796 | 0.542342 | 0.80367 |
| MIR221 | 0.922728 | 0.792918 | -0.21953 | 2.359714 |
| RFWD3 | 0.922728 | 0.792994 | 0.109672 | 5.783924 |
| ZBED8 | 0.922728 | 0.793036 | 0.473521 | 1.11193 |
| GPR107 | 0.922728 | 0.793059 | 0.266908 | 2.110071 |
| TMEM69 | 0.922762 | 0.793159 | 0.149355 | 4.174226 |
| LDB1 | 0.922852 | 0.793348 | 0.173152 | 2.939937 |
| HDGFL3 | 0.922852 | 0.793377 | 0.129262 | 5.412853 |
| HDLBP | 0.922885 | 0.793486 | -0.33893 | 1.182276 |
| RNF44 | 0.922885 | 0.793583 | 0.092668 | 5.92383 |
| DCK | 0.922885 | 0.793675 | 0.13891 | 4.720056 |
| MTFMT | 0.922885 | 0.793701 | -0.15762 | 3.76219 |
| RBBP7 | 0.922885 | 0.793757 | -0.12638 | 4.559128 |
| EXOC3L2 | 0.923059 | 0.793977 | 0.250835 | 1.00758 |
| ADRA2C | 0.923476 | 0.794438 | -0.42224 | 1.441843 |
| ADCY10P1 | 0.923476 | 0.794477 | 0.161016 | 3.858788 |
| MED16 | 0.923629 | 0.794774 | -0.0941 | 5.575327 |
| FAM210B | 0.923629 | 0.794831 | -0.09887 | 6.078545 |
| FAM204A | 0.923629 | 0.79484 | 0.375448 | 1.378174 |
| ZNF276 | 0.923629 | 0.794889 | 0.232371 | 1.96455 |
| LINC01271 | 0.923637 | 0.794967 | -0.37785 | 0.961015 |

| | | | | |
|---|---|---|---|---|
| KIF24 | 0.923945 | 0.795346 | 0.130983 | 4.001255 |
| CCL17 | 0.923945 | 0.795373 | -0.42658 | 0.58962 |
| KPNA5 | 0.924016 | 0.795504 | 0.127374 | 4.287929 |
| HSD17B7 | 0.924118 | 0.795666 | -0.22458 | 2.441883 |
| ZNF784 | 0.924118 | 0.795733 | 0.188652 | 2.539993 |
| FAM200B | 0.924225 | 0.795896 | 0.13145 | 4.369379 |
| LOC101928 | 0.924461 | 0.796169 | 0.297696 | 1.351308 |
| CYP4F2 | 0.924557 | 0.796322 | -0.43951 | 0.36399 |
| TRIM41 | 0.924679 | 0.796498 | -0.17942 | 2.747612 |
| WDR59 | 0.924734 | 0.796616 | -0.0991 | 6.101113 |
| SETSIP | 0.924903 | 0.796832 | 0.168804 | 2.495655 |
| TM4SF4 | 0.925027 | 0.797009 | -0.46193 | 0.806802 |
| ANKRD49 | 0.925217 | 0.797244 | -0.12797 | 4.703479 |
| C1orf220 | 0.925452 | 0.797547 | 0.19043 | 2.573622 |
| OMP | 0.925452 | 0.797587 | 0.398854 | 0.687792 |
| TNPO2 | 0.925455 | 0.797725 | 0.201126 | 2.086871 |
| KPNB1 | 0.925455 | 0.79785 | -0.14331 | 3.192579 |
| MON2 | 0.925455 | 0.797875 | 0.278766 | 1.647931 |
| SLC26A4 | 0.925455 | 0.797905 | 0.380958 | 0.890909 |
| THOC7-AS1 | 0.925455 | 0.797942 | 0.222577 | 2.052859 |
| C5orf15 | 0.925518 | 0.798122 | 0.101373 | 5.203212 |
| GATA6-AS1 | 0.925518 | 0.798138 | -0.34327 | 2.947947 |
| SHISA9 | 0.925618 | 0.798338 | 0.284692 | 0.638996 |
| LOC100128 | 0.925618 | 0.798365 | -0.13785 | 3.731059 |
| CCDC40 | 0.925784 | 0.798578 | 0.392328 | 1.476892 |
| STARD10 | 0.925972 | 0.798812 | 0.154751 | 6.102757 |
| GLE1 | 0.926031 | 0.798933 | 0.115673 | 4.477895 |
| FOXP1 | 0.926209 | 0.799157 | -0.12012 | 4.382238 |
| PDCD1LG2 | 0.926424 | 0.79943 | 0.272136 | 1.783679 |
| GALE | 0.926424 | 0.799483 | 0.304006 | 1.704266 |
| IVNS1ABP | 0.926603 | 0.799709 | 0.09816 | 7.968894 |
| SSRP1 | 0.926725 | 0.799885 | 0.09876 | 7.580739 |
| PTGES3 | 0.926973 | 0.800256 | -0.15437 | 4.713521 |
| MRPS18B | 0.926973 | 0.800458 | 0.265954 | 2.37618 |
| ERICH5 | 0.926973 | 0.800543 | 0.331409 | 1.81446 |
| TMEM9B-A | 0.926973 | 0.800549 | 0.212853 | 2.127764 |
| MSMP | 0.926973 | 0.800557 | -0.18758 | 3.575893 |
| LOC407835 | 0.926973 | 0.8006 | -0.18616 | 2.14701 |
| GABPB2 | 0.926973 | 0.800622 | 0.113984 | 5.2513 |
| C1orf112 | 0.926973 | 0.800664 | 0.132163 | 4.02277 |
| CTSG | 0.927001 | 0.800759 | -0.36735 | 1.019739 |
| DYNC1LI2 | 0.927044 | 0.800866 | 0.135977 | 3.629241 |
| DNAJC24 | 0.927137 | 0.801061 | 0.14849 | 3.912114 |
| ZNF701 | 0.927137 | 0.801158 | 0.290911 | 1.541952 |
| LOC102031 | 0.927137 | 0.801178 | 0.234297 | 1.971145 |

| | | | | |
|---|---|---|---|---|
| HLCS | 0.927137 | 0.801229 | -0.29689 | 1.333745 |
| SNORA53 | 0.927157 | 0.801359 | -0.10875 | 7.432021 |
| KAT6A | 0.927157 | 0.801388 | 0.092018 | 7.699793 |
| STAM-AS1 | 0.927198 | 0.801532 | 0.348494 | 0.47175 |
| B9D2 | 0.927198 | 0.801565 | 0.270099 | 1.183496 |
| NELFE | 0.927272 | 0.801739 | 0.256013 | 2.083332 |
| PTPN21 | 0.927272 | 0.80177 | 0.102415 | 5.881617 |
| PFAS | 0.927388 | 0.801941 | 0.11027 | 5.092074 |
| L3MBTL2 | 0.927488 | 0.802172 | 0.107379 | 5.195163 |
| AGRP | 0.927488 | 0.802219 | -0.28716 | 1.01951 |
| PIK3CB | 0.927488 | 0.80224 | -0.09935 | 5.501669 |
| IL23A | 0.927505 | 0.802383 | 0.289517 | 2.627646 |
| IL2RB | 0.927505 | 0.802396 | 0.220527 | 3.367976 |
| CHD6 | 0.927597 | 0.802546 | 0.092601 | 7.763078 |
| PPAN | 0.927694 | 0.8027 | -0.33097 | 1.095813 |
| KDM3B | 0.92784 | 0.802973 | 0.089077 | 7.842346 |
| ARHGEF28 | 0.92784 | 0.803027 | -0.34223 | 1.117039 |
| PDCL2 | 0.92784 | 0.803281 | -0.46131 | 0.576877 |
| METTL22 | 0.92784 | 0.803321 | 0.124744 | 4.056471 |
| CDK2AP2 | 0.92784 | 0.803339 | -0.28802 | 1.167532 |
| HTATIP2 | 0.92784 | 0.803374 | 0.29807 | 1.18149 |
| CSNK2A1 | 0.92784 | 0.803411 | 0.324378 | 0.999236 |
| SOGA1 | 0.92784 | 0.803453 | 0.098758 | 5.964747 |
| CELF5 | 0.92784 | 0.803464 | 0.431049 | 0.496863 |
| LOC100507 | 0.927873 | 0.803657 | 0.274983 | 1.473054 |
| QTRT1 | 0.927873 | 0.803663 | 0.119329 | 4.691861 |
| RRAS2 | 0.927873 | 0.803725 | -0.29366 | 0.467053 |
| TRIM37 | 0.927873 | 0.803787 | 0.195528 | 3.180553 |
| GPR83 | 0.927873 | 0.803876 | 0.371018 | 1.180931 |
| IKZF5 | 0.927873 | 0.803936 | -0.22442 | 1.880744 |
| MIR4269 | 0.927873 | 0.803987 | 0.410645 | 0.552814 |
| FAM168B | 0.927965 | 0.804138 | -0.08561 | 7.361533 |
| MIR320E | 0.928203 | 0.804415 | -0.29037 | 1.191345 |
| MMS22L | 0.928348 | 0.804611 | 0.10356 | 4.736536 |
| MRPS23 | 0.928615 | 0.804932 | -0.1073 | 5.194413 |
| LTV1 | 0.928615 | 0.804984 | 0.103139 | 6.217694 |
| KIFAP3 | 0.928641 | 0.805134 | 0.3355 | 0.620101 |
| PTK2 | 0.928641 | 0.805148 | 0.333951 | 1.061056 |
| TXNRD2 | 0.928723 | 0.805313 | -0.18549 | 3.091166 |
| LINC01347 | 0.928723 | 0.80536 | -0.14711 | 3.066797 |
| MGC16025 | 0.928781 | 0.805481 | 0.216798 | 3.233838 |
| MORN2 | 0.928795 | 0.805565 | -0.24955 | 2.719822 |
| RNY3 | 0.928903 | 0.805729 | 0.136818 | 9.226864 |
| HNRNPA3P | 0.929085 | 0.805958 | 0.17481 | 2.301094 |
| CLCA1 | 0.929117 | 0.806106 | -0.35444 | 1.334388 |

| | | | | |
|---|---|---|---|---|
| EML6 | 0.929117 | 0.806127 | -0.12647 | 5.060335 |
| ST7 | 0.929275 | 0.806335 | 0.203583 | 2.234688 |
| TMEM86A | 0.929283 | 0.806479 | -0.19472 | 2.972559 |
| SGSH | 0.929283 | 0.806483 | -0.11771 | 4.708301 |
| TMEM185I | 0.929539 | 0.806777 | 0.08926 | 5.721884 |
| HIST1H2BC | 0.929571 | 0.806895 | 0.179651 | 3.957085 |
| POLE4 | 0.929571 | 0.806999 | 0.177684 | 3.393317 |
| HIST2H3D | 0.929571 | 0.807102 | -0.29932 | 0.980222 |
| NTM | 0.929571 | 0.807106 | -0.44005 | 0.549996 |
| LOC10050! | 0.929571 | 0.807158 | 0.157913 | 2.891345 |
| LZTS1-AS1 | 0.929573 | 0.807231 | -0.3889 | 0.597286 |
| CCDC184 | 0.929651 | 0.807369 | 0.385417 | 0.465377 |
| CROCC | 0.929749 | 0.807526 | -0.10886 | 6.118658 |
| TH2LCRR | 0.929889 | 0.807756 | -0.34653 | 1.229053 |
| CUX1 | 0.929889 | 0.807961 | -0.26099 | 1.491024 |
| ASPH | 0.929889 | 0.807973 | -0.13581 | 3.764162 |
| MINAR1 | 0.929889 | 0.808007 | 0.211526 | 2.045118 |
| KCNC1 | 0.929889 | 0.808084 | 0.333673 | 0.653138 |
| TMUB1 | 0.929889 | 0.808192 | -0.15684 | 3.41342 |
| ZC3H3 | 0.929889 | 0.8082 | -0.13586 | 4.428161 |
| FREM3 | 0.929889 | 0.808309 | -0.3852 | 0.862425 |
| FNBP4 | 0.929889 | 0.808329 | -0.08976 | 8.401641 |
| GOLGA3 | 0.929889 | 0.808531 | -0.08934 | 7.647838 |
| FGGY | 0.929889 | 0.808556 | 0.270142 | 1.960509 |
| LNCTAM34 | 0.929889 | 0.808673 | -0.25076 | 1.88858 |
| CNOT2 | 0.929889 | 0.808728 | -0.31025 | 0.938543 |
| GPN2 | 0.929889 | 0.808822 | 0.123718 | 3.750335 |
| PCNX1 | 0.929889 | 0.808857 | 0.161257 | 2.726485 |
| PSMA2 | 0.929889 | 0.808943 | 0.104792 | 5.805096 |
| DPCD | 0.929889 | 0.808961 | -0.13804 | 3.848963 |
| MAPK8IP2 | 0.929889 | 0.808987 | -0.25728 | 2.196726 |
| SORBS2 | 0.929889 | 0.808998 | 0.248028 | 2.035713 |
| PI4KB | 0.929889 | 0.809064 | 0.27662 | 1.269672 |
| SPIN1 | 0.92999 | 0.809223 | 0.104685 | 6.650714 |
| SH3BP5 | 0.93006 | 0.809424 | -0.24499 | 2.060777 |
| UBE4B | 0.93006 | 0.809541 | -0.20463 | 2.126276 |
| PCDH1 | 0.93006 | 0.809573 | -0.12282 | 4.695486 |
| LOC10013 | 0.93006 | 0.809659 | 0.336527 | 1.331406 |
| TRPC5 | 0.93006 | 0.809682 | 0.312761 | 1.189463 |
| SFT2D3 | 0.93006 | 0.809709 | 0.11754 | 4.117203 |
| ZNF292 | 0.930505 | 0.810239 | 0.090235 | 8.212971 |
| NDUFAB1 | 0.930505 | 0.810575 | 0.124365 | 5.098333 |
| SLC38A6 | 0.930505 | 0.810591 | 0.228397 | 2.000874 |
| SH2D3A | 0.930505 | 0.810599 | -0.09501 | 5.30508 |
| ABHD17C | 0.930505 | 0.810695 | -0.12053 | 6.303555 |

| | | | | |
|---|---|---|---|---|
| LOC10192S | 0.930505 | 0.810737 | 0.274901 | 1.751976 |
| SLC35F5 | 0.930505 | 0.810748 | 0.174897 | 2.738438 |
| HCG18 | 0.930505 | 0.810761 | -0.10469 | 4.626285 |
| DCTN6 | 0.930505 | 0.810815 | -0.14365 | 4.725986 |
| COG8 | 0.930505 | 0.810828 | -0.16843 | 3.775688 |
| PXN | 0.930505 | 0.810887 | 0.189366 | 2.300252 |
| DHODH | 0.930505 | 0.811088 | -0.17434 | 3.849356 |
| RAD54L2 | 0.930505 | 0.811303 | 0.087305 | 6.014871 |
| LINC01336 | 0.930505 | 0.811342 | 0.270617 | 2.057052 |
| IRAK2 | 0.930505 | 0.811391 | 0.166906 | 4.695095 |
| ICE2 | 0.930505 | 0.811404 | 0.127306 | 3.997908 |
| PEAK3 | 0.930505 | 0.811422 | 0.285129 | 1.287167 |
| KLF7 | 0.930505 | 0.811435 | -0.18954 | 2.588352 |
| APEH | 0.930505 | 0.811444 | -0.08841 | 5.900674 |
| ZNF397 | 0.930687 | 0.811674 | -0.11567 | 5.008658 |
| TLR9 | 0.930764 | 0.811875 | -0.305 | 0.569778 |
| ALG12 | 0.930764 | 0.811925 | -0.12903 | 4.158762 |
| DAB1 | 0.930764 | 0.811954 | 0.360025 | 1.118373 |
| SETD1B | 0.93078 | 0.81212 | 0.0848 | 7.569693 |
| PGP | 0.93078 | 0.812215 | 0.148692 | 3.892616 |
| NIPBL | 0.93078 | 0.812401 | -0.3172 | 0.610854 |
| MPZL1 | 0.93078 | 0.812447 | 0.211214 | 2.120142 |
| SPIN2A | 0.93078 | 0.812489 | 0.241218 | 1.654914 |
| CCNT1 | 0.93078 | 0.812508 | 0.281352 | 1.1957 |
| BIN3-IT1 | 0.93078 | 0.812535 | -0.20581 | 3.000526 |
| RBM22 | 0.93078 | 0.812703 | 0.089768 | 5.712501 |
| JUN | 0.93078 | 0.812715 | -0.11564 | 8.691417 |
| SMAD1 | 0.93078 | 0.81274 | -0.26691 | 0.568522 |
| CXorf40A | 0.93078 | 0.812849 | 0.313526 | 0.445746 |
| AP4S1 | 0.93078 | 0.812858 | 0.140071 | 3.018428 |
| GPR18 | 0.93078 | 0.81309 | -0.26242 | 1.702157 |
| BBOF1 | 0.93078 | 0.813117 | 0.147763 | 4.070396 |
| SCARNA16 | 0.93078 | 0.813134 | -0.15098 | 4.08288 |
| SNHG25 | 0.93078 | 0.813205 | 0.208429 | 2.687291 |
| WDR37 | 0.93078 | 0.813261 | 0.109011 | 5.018768 |
| EPS15 | 0.93078 | 0.813402 | 0.099179 | 5.251576 |
| FAM98B | 0.93078 | 0.813468 | 0.090705 | 5.519654 |
| FAM90A1 | 0.93078 | 0.813518 | 0.212377 | 2.082191 |
| SLC4A7 | 0.93078 | 0.813549 | 0.150155 | 3.051559 |
| FOXO3 | 0.93078 | 0.813595 | -0.23488 | 2.214717 |
| UBN1 | 0.93078 | 0.813599 | 0.19945 | 2.556407 |
| FAM3B | 0.930883 | 0.813771 | -0.27989 | 0.873404 |
| CCT2 | 0.930883 | 0.813831 | -0.298 | 1.141688 |
| BCL7B | 0.931394 | 0.814349 | -0.36786 | 0.553717 |
| ANKRD20A | 0.931457 | 0.814475 | -0.26658 | 2.654927 |

| | | | | |
|---|---|---|---|---|
| TONSL-AS1 | 0.931634 | 0.8147 | 0.230035 | 1.461598 |
| LINC01535 | 0.931659 | 0.814793 | 0.283313 | 0.841433 |
| GEMIN8P4 | 0.931703 | 0.814903 | -0.32946 | 0.885061 |
| ZNF860 | 0.931824 | 0.815155 | 0.139896 | 3.944288 |
| SEC22A | 0.931824 | 0.815188 | -0.16064 | 4.69904 |
| CCR4 | 0.931824 | 0.81526 | 0.25249 | 1.749864 |
| RBMX2 | 0.931824 | 0.815293 | 0.098209 | 4.939991 |
| SCYL2 | 0.931946 | 0.815533 | -0.16395 | 2.221151 |
| ACTG1P4 | 0.931946 | 0.815541 | -0.31822 | 1.110284 |
| COLCA1 | 0.932096 | 0.815744 | -0.34868 | 0.376197 |
| GRIK1 | 0.932262 | 0.815964 | -0.37444 | 0.402107 |
| TOB2P1 | 0.932262 | 0.816031 | 0.32434 | 0.663306 |
| C6orf163 | 0.932546 | 0.816351 | -0.20021 | 2.246298 |
| DDX59 | 0.932618 | 0.816657 | 0.094637 | 5.006649 |
| TMEM241 | 0.932618 | 0.816671 | -0.11836 | 4.090154 |
| DCAF10 | 0.932618 | 0.816691 | 0.168148 | 2.011726 |
| CEP170B | 0.932618 | 0.816703 | -0.30258 | 0.908436 |
| SUN1 | 0.932618 | 0.816769 | 0.165769 | 2.000521 |
| EFCAB14 | 0.932721 | 0.81693 | -0.09793 | 8.489211 |
| RBM27 | 0.932864 | 0.817127 | -0.08313 | 7.116352 |
| IFT43 | 0.933072 | 0.817431 | 0.268997 | 0.989809 |
| RPL36A | 0.933072 | 0.817451 | -0.13481 | 3.653395 |
| ANKRD50 | 0.933155 | 0.817599 | -0.16949 | 3.653399 |
| KCNA3 | 0.933155 | 0.817666 | 0.245359 | 2.420969 |
| ZNF876P | 0.933272 | 0.817859 | 0.192236 | 2.589093 |
| CFL1 | 0.933272 | 0.817911 | -0.09416 | 8.64622 |
| PLEKHG4 | 0.933682 | 0.818346 | -0.30068 | 1.524259 |
| FAM208A | 0.933682 | 0.818413 | -0.09759 | 5.450672 |
| JMJD8 | 0.933757 | 0.818611 | -0.11058 | 4.243985 |
| ZBED6CL | 0.933757 | 0.81862 | 0.169908 | 3.154986 |
| ALOX12P2 | 0.933807 | 0.818812 | 0.224387 | 2.911842 |
| C11orf87 | 0.933807 | 0.818881 | 0.466349 | 0.726643 |
| SYT9 | 0.933807 | 0.818963 | 0.409141 | 0.432237 |
| LINC02367 | 0.933807 | 0.819082 | 0.31095 | 0.415975 |
| DUSP3 | 0.933807 | 0.819084 | -0.0873 | 6.77172 |
| KLHL28 | 0.933807 | 0.819092 | 0.240085 | 1.657268 |
| SEC24B-AS | 0.933905 | 0.819249 | -0.33612 | 0.562073 |
| POM121L8 | 0.933992 | 0.819396 | -0.30336 | 1.184473 |
| PTPN14 | 0.934027 | 0.819498 | 0.091949 | 7.629798 |
| CATSPER2F | 0.934277 | 0.819788 | -0.19218 | 2.096087 |
| TMEM250 | 0.93439 | 0.819959 | 0.217861 | 1.363351 |
| XRCC5 | 0.934542 | 0.820164 | -0.08298 | 8.572823 |
| ZNF133 | 0.934783 | 0.820465 | 0.249036 | 2.020444 |
| ZER1 | 0.934783 | 0.820517 | -0.09018 | 5.873652 |
| ALYREF | 0.934876 | 0.82074 | -0.0958 | 5.004857 |

| | | | | |
|---|---|---|---|---|
| SGMS2 | 0.934876 | 0.82082 | -0.21635 | 1.999638 |
| ZNF668 | 0.934876 | 0.820877 | 0.248468 | 1.593745 |
| PACSIN2 | 0.934876 | 0.820887 | 0.132818 | 3.269692 |
| AGAP3 | 0.934876 | 0.821056 | 0.169442 | 2.677736 |
| CASKIN2 | 0.934876 | 0.821117 | -0.17965 | 2.321137 |
| HIST1H2AE | 0.934876 | 0.821134 | 0.17329 | 3.666598 |
| SLC35C1 | 0.934876 | 0.821188 | -0.14391 | 3.320782 |
| NSUN6 | 0.934876 | 0.821279 | 0.101591 | 4.599657 |
| LOC101928 | 0.934876 | 0.821311 | -0.37646 | 0.502291 |
| OSBPL3 | 0.93502 | 0.821767 | 0.203474 | 2.131527 |
| CDC5L | 0.93502 | 0.821784 | 0.077695 | 6.921728 |
| OVCA2 | 0.93502 | 0.821829 | 0.255257 | 1.269371 |
| DOLK | 0.93502 | 0.821872 | -0.13168 | 3.753292 |
| DNAJC17 | 0.93502 | 0.821985 | -0.11212 | 4.183974 |
| TRIM23 | 0.93502 | 0.822001 | -0.20978 | 2.129585 |
| MIR29C | 0.93502 | 0.822052 | 0.233069 | 2.260878 |
| SNORD15B | 0.93502 | 0.822071 | 0.096305 | 9.019397 |
| PKHD1L1 | 0.93502 | 0.82208 | 0.20681 | 3.337391 |
| AP1G1 | 0.935043 | 0.822187 | 0.217655 | 1.619913 |
| PSMD14 | 0.935043 | 0.822242 | -0.11754 | 6.143318 |
| SRGAP1 | 0.935097 | 0.822412 | -0.08679 | 6.699427 |
| NOCT | 0.935097 | 0.822433 | -0.11313 | 3.838809 |
| PCSK9 | 0.935118 | 0.822707 | 0.390491 | 0.863726 |
| ATP5F1E | 0.935118 | 0.822794 | -0.26094 | 0.829342 |
| ACSL4 | 0.935118 | 0.822886 | -0.40095 | 0.970201 |
| WDFY3-AS | 0.935118 | 0.82289 | -0.24046 | 1.434173 |
| KMT5B | 0.935118 | 0.822919 | 0.277812 | 1.711853 |
| LINC02102 | 0.935118 | 0.822938 | -0.23734 | 1.619171 |
| PEX13 | 0.935118 | 0.823014 | -0.08059 | 6.138845 |
| ALX1 | 0.935118 | 0.82302 | 0.468987 | 0.418665 |
| COL5A3 | 0.935462 | 0.823414 | 0.089173 | 5.683491 |
| MIR21 | 0.935462 | 0.823503 | 0.195379 | 3.07586 |
| RPS6KA2-A | 0.935462 | 0.823537 | 0.371324 | 1.030987 |
| SENP6 | 0.935507 | 0.823669 | 0.127972 | 3.481243 |
| CLEC2B | 0.935507 | 0.823826 | -0.13403 | 4.017356 |
| WHAMMP | 0.935507 | 0.823839 | -0.09329 | 5.16939 |
| B4GALT1 | 0.935507 | 0.823866 | 0.107917 | 8.034503 |
| RSL1D1 | 0.935507 | 0.823946 | 0.082616 | 7.582027 |
| ELMSAN1 | 0.935507 | 0.82411 | 0.190798 | 2.444497 |
| SELENOS | 0.935507 | 0.824171 | -0.13054 | 3.815962 |
| AGBL1 | 0.935507 | 0.824192 | -0.5112 | 0.677581 |
| CCDC137 | 0.935507 | 0.824219 | -0.09186 | 5.095434 |
| LOC100507 | 0.935519 | 0.824301 | -0.20955 | 1.605172 |
| CHRNB2 | 0.935554 | 0.824403 | 0.207824 | 2.090064 |
| TKFC | 0.935595 | 0.82451 | 0.112108 | 4.962472 |

| | | | | |
|---|---|---|---|---|
| MGMT | 0.935629 | 0.824611 | 0.123938 | 3.590084 |
| NDUFV3 | 0.935718 | 0.824841 | -0.12781 | 4.361895 |
| SLC13A4 | 0.935718 | 0.824906 | -0.13313 | 4.375439 |
| TMEM68 | 0.935718 | 0.825088 | -0.16527 | 2.651273 |
| RRP36 | 0.935718 | 0.825125 | 0.105161 | 4.902658 |
| ETAA1 | 0.935718 | 0.825147 | 0.082017 | 5.454558 |
| ACAT1 | 0.935718 | 0.825159 | -0.09328 | 5.505255 |
| METTL14-D | 0.935718 | 0.825238 | 0.337898 | 0.537033 |
| HS6ST3 | 0.935718 | 0.825289 | -0.35392 | 1.714477 |
| DECR2 | 0.935718 | 0.825332 | 0.238169 | 1.156483 |
| RNASEL | 0.935746 | 0.825427 | 0.11242 | 4.88964 |
| LBHD1 | 0.935907 | 0.82564 | 0.23484 | 1.830012 |
| F8 | 0.935918 | 0.825767 | -0.22877 | 2.116192 |
| CD177 | 0.935918 | 0.825793 | 0.249937 | 2.589423 |
| KLF15 | 0.93605 | 0.825981 | -0.26503 | 3.992308 |
| DNAJC30 | 0.936502 | 0.82657 | 0.139585 | 3.420055 |
| MOB3C | 0.936502 | 0.826659 | -0.22533 | 1.802328 |
| NISCH | 0.936502 | 0.826744 | 0.083197 | 5.970142 |
| KIAA1143 | 0.936502 | 0.826851 | 0.093895 | 5.428542 |
| TBL1XR1 | 0.936502 | 0.826869 | 0.083094 | 8.467455 |
| PRC1-AS1 | 0.936502 | 0.826871 | -0.3029 | 1.478968 |
| RNPEPL1 | 0.936502 | 0.826882 | 0.084317 | 6.004393 |
| RANBP6 | 0.936502 | 0.826951 | -0.15711 | 2.689338 |
| MIR624 | 0.93651 | 0.82703 | 0.353142 | 0.514165 |
| PPP1R26-A | 0.936544 | 0.827169 | 0.239396 | 2.197916 |
| ZNF420 | 0.936544 | 0.827283 | 0.136174 | 3.265796 |
| GSKIP | 0.936544 | 0.827379 | -0.24693 | 1.961026 |
| SLC2A12 | 0.936544 | 0.827484 | -0.16897 | 4.719778 |
| SERPINB9P | 0.936544 | 0.827486 | -0.2043 | 2.752656 |
| AMPD2 | 0.936544 | 0.827549 | 0.266239 | 1.484141 |
| ADHFE1 | 0.936544 | 0.827559 | -0.24188 | 3.15633 |
| TMEM214 | 0.936611 | 0.827689 | 0.294915 | 0.441432 |
| LIN7B | 0.93699 | 0.828165 | -0.34486 | 0.603372 |
| UBAP1L | 0.93699 | 0.828167 | 0.150328 | 2.279351 |
| CCDC91 | 0.937163 | 0.828493 | -0.07364 | 5.998077 |
| ELF2 | 0.937163 | 0.8285 | -0.10029 | 4.243865 |
| ASB16-AS1 | 0.937163 | 0.828568 | 0.219706 | 1.320245 |
| PDE7A | 0.937163 | 0.828605 | 0.293708 | 0.944164 |
| LOC339803 | 0.9372 | 0.828809 | 0.212459 | 1.660506 |
| TAB1 | 0.9372 | 0.828838 | 0.11257 | 4.091523 |
| IPO13 | 0.9372 | 0.828883 | -0.0805 | 5.576537 |
| SLC36A2 | 0.9372 | 0.829075 | -0.46725 | 0.56614 |
| PDXDC2P-N | 0.9372 | 0.829104 | 0.086212 | 7.4523 |
| ATP2B1-AS | 0.9372 | 0.829139 | -0.22237 | 2.204841 |
| IQCC | 0.9372 | 0.829162 | 0.317313 | 0.788125 |

| | | | | |
|---|---|---|---|---|
| PABPC4L | 0.9372 | 0.82921 | -0.22745 | 2.312005 |
| CFAP126 | 0.937334 | 0.8294 | 0.100222 | 4.832235 |
| DNAJC14 | 0.937342 | 0.829523 | 0.07945 | 5.69897 |
| DKK1 | 0.937342 | 0.82955 | -0.25255 | 3.958933 |
| CYYR1 | 0.937361 | 0.829641 | 0.172805 | 2.883523 |
| RABGAP1 | 0.937361 | 0.82971 | -0.08302 | 7.787468 |
| WDR5B | 0.937474 | 0.829881 | -0.10244 | 4.28308 |
| ZNF786 | 0.937651 | 0.8302 | 0.131835 | 3.6766 |
| FOSB | 0.937651 | 0.830386 | 0.247804 | 2.281012 |
| IL15RA | 0.937651 | 0.830394 | -0.38463 | 0.563762 |
| PAFAH2 | 0.937651 | 0.830412 | -0.08467 | 5.070701 |
| TIMELESS | 0.937651 | 0.830445 | 0.084044 | 5.775726 |
| LINC00667 | 0.937651 | 0.830467 | -0.13957 | 4.326158 |
| GMPS | 0.938181 | 0.831035 | 0.079504 | 6.498898 |
| HIST1H2BB | 0.938181 | 0.831079 | 0.099267 | 4.653101 |
| HCG4B | 0.938297 | 0.831277 | 0.231374 | 1.288557 |
| PCDHB3 | 0.938297 | 0.831325 | 0.239066 | 1.653575 |
| CRELD2 | 0.938388 | 0.831477 | -0.3661 | 0.726293 |
| RASGRP3 | 0.938532 | 0.831734 | 0.322468 | 1.462809 |
| LINC01087 | 0.938532 | 0.831857 | -0.49011 | 0.711009 |
| NECTIN3 | 0.938532 | 0.832055 | 0.261268 | 1.609838 |
| ADNP | 0.938532 | 0.832136 | 0.199816 | 2.714279 |
| LINC02401 | 0.938532 | 0.832197 | 0.45844 | 0.504813 |
| TSR3 | 0.938532 | 0.83221 | 0.099725 | 4.908625 |
| PVR | 0.938532 | 0.832301 | -0.21211 | 1.990218 |
| SNORD18A | 0.938532 | 0.832424 | 0.278447 | 0.776995 |
| MLANA | 0.938532 | 0.832439 | -0.14619 | 2.885369 |
| ITPKB | 0.938532 | 0.832535 | 0.088306 | 6.241658 |
| PSMD5 | 0.938532 | 0.832551 | 0.228929 | 0.660263 |
| ALG6 | 0.938532 | 0.832563 | -0.10782 | 3.914035 |
| FFAR2 | 0.938532 | 0.832653 | -0.31498 | 1.191615 |
| LOC10254€ | 0.938532 | 0.832682 | -0.24538 | 2.06975 |
| EXOSC2 | 0.938532 | 0.832777 | -0.22655 | 1.65066 |
| OTUD4 | 0.938532 | 0.83281 | 0.078402 | 7.087175 |
| HIST1H2BF | 0.938532 | 0.832849 | 0.115912 | 4.446291 |
| LRRC75B | 0.938532 | 0.832892 | 0.158901 | 2.016694 |
| LRRC8D | 0.938536 | 0.832967 | -0.25056 | 1.470291 |
| CLK1 | 0.938561 | 0.83306 | -0.08523 | 5.979643 |
| LOC10537¢ | 0.938574 | 0.8333 | 0.239026 | 0.638406 |
| HMG20B | 0.938574 | 0.833364 | 0.079924 | 6.196921 |
| PWP2 | 0.938574 | 0.833387 | 0.173097 | 3.044715 |
| DNAJC3 | 0.938574 | 0.8334 | 0.081604 | 7.321233 |
| DPAGT1 | 0.938574 | 0.833472 | 0.093911 | 4.639012 |
| FUT9 | 0.938574 | 0.833501 | 0.414678 | 4.762011 |
| CCDC173 | 0.938842 | 0.8339 | -0.24732 | 1.843305 |

| | | | | |
|---|---|---|---|---|
| ZMYM3 | 0.938842 | 0.833984 | 0.218605 | 1.161902 |
| COX7A2 | 0.938842 | 0.834016 | 0.29668 | 0.75584 |
| DLGAP1-AS | 0.938842 | 0.834025 | -0.40763 | 1.070005 |
| NUP50 | 0.938862 | 0.834115 | -0.15143 | 2.423966 |
| SNORA46 | 0.938909 | 0.834281 | -0.14093 | 4.350997 |
| OMG | 0.938909 | 0.834347 | 0.196048 | 3.326799 |
| KANTR | 0.938909 | 0.834404 | -0.11251 | 3.56123 |
| PTTG1IP | 0.938909 | 0.834554 | -0.3145 | 0.676966 |
| METTL18 | 0.938909 | 0.834572 | 0.154069 | 2.713758 |
| SNORD14B | 0.938909 | 0.834586 | -0.10553 | 5.421268 |
| CEP104 | 0.939094 | 0.834822 | 0.075341 | 6.085842 |
| DLC1 | 0.93938 | 0.835148 | -0.23239 | 1.806367 |
| LOC100505 | 0.939551 | 0.835372 | 0.210448 | 1.642689 |
| LINC02042 | 0.939636 | 0.835521 | 0.500313 | 0.626893 |
| SUGT1P4-S | 0.939636 | 0.83559 | 0.237256 | 1.885007 |
| MIR647 | 0.939804 | 0.835964 | -0.23558 | 1.531551 |
| SURF4 | 0.939804 | 0.835991 | -0.24933 | 0.468698 |
| AGAP9 | 0.939804 | 0.836016 | -0.11173 | 3.964679 |
| COG3 | 0.939804 | 0.83608 | 0.078478 | 6.601281 |
| FNDC9 | 0.939804 | 0.836097 | 0.27687 | 1.260031 |
| ARPC2 | 0.939858 | 0.836217 | -0.25814 | 0.412143 |
| ADSS | 0.939991 | 0.836407 | 0.084303 | 6.377181 |
| LOC100129 | 0.940295 | 0.836749 | 0.140509 | 3.31696 |
| CDC42 | 0.940298 | 0.836824 | 0.124115 | 3.638435 |
| CTAGE10P | 0.94056 | 0.837129 | 0.313117 | 0.676997 |
| TAF8 | 0.94071 | 0.837334 | 0.076395 | 5.55211 |
| ACBD6 | 0.940729 | 0.83746 | 0.096097 | 5.37957 |
| SMG5 | 0.940729 | 0.837494 | 0.077566 | 7.46858 |
| AKNA | 0.940775 | 0.837607 | 0.285995 | 0.846716 |
| TAF13 | 0.941081 | 0.83795 | -0.10411 | 4.54831 |
| FKBP15 | 0.941332 | 0.838246 | 0.07596 | 6.723666 |
| MIR601 | 0.941426 | 0.838401 | 0.390477 | 0.427693 |
| DVL3 | 0.941467 | 0.83857 | -0.07193 | 7.380776 |
| CTAGE9 | 0.941467 | 0.838591 | -0.2455 | 1.549227 |
| FAM135B | 0.941467 | 0.838653 | -0.2972 | 0.533879 |
| SOCS6 | 0.94148 | 0.838737 | -0.10149 | 6.528119 |
| TMEM50A | 0.941515 | 0.838839 | -0.07918 | 5.798655 |
| FBXO22 | 0.941529 | 0.838924 | 0.224199 | 2.251685 |
| ZNF644 | 0.941794 | 0.839293 | 0.075666 | 6.512163 |
| CA9 | 0.941794 | 0.839303 | -0.26005 | 4.92358 |
| SLC2A5 | 0.942022 | 0.839671 | 0.217026 | 1.685464 |
| AAK1 | 0.942022 | 0.839674 | -0.07672 | 7.895744 |
| GUSBP3 | 0.942022 | 0.839722 | -0.17004 | 1.982102 |
| MIR570HG | 0.942029 | 0.8398 | -0.12667 | 2.929359 |
| BRD4 | 0.942204 | 0.84015 | 0.07022 | 6.643443 |

| | | | | |
|---|---|---|---|---|
| LOC101928 | 0.942204 | 0.840193 | -0.1885 | 2.638948 |
| EIF1B-AS1 | 0.942204 | 0.840223 | 0.252583 | 1.843118 |
| NUDT16 | 0.942204 | 0.840243 | 0.202045 | 1.413301 |
| CIB1 | 0.942273 | 0.840376 | -0.3094 | 0.777348 |
| NRM | 0.942296 | 0.840468 | 0.291244 | 0.541001 |
| LOC389906 | 0.942388 | 0.840623 | 0.101563 | 4.097568 |
| OXA1L | 0.942433 | 0.840807 | 0.077528 | 5.972452 |
| RALY | 0.942433 | 0.840954 | -0.23198 | 1.545 |
| PTGER1 | 0.942433 | 0.840979 | -0.33418 | 0.41285 |
| ARHGAP31 | 0.942433 | 0.840996 | -0.2946 | 0.811431 |
| EFCAB7 | 0.942433 | 0.841022 | 0.174389 | 2.98006 |
| MTHFR | 0.942481 | 0.841166 | 0.075118 | 5.727478 |
| CD27 | 0.942481 | 0.841208 | 0.191504 | 1.189962 |
| SHISAL1 | 0.94262 | 0.841471 | -0.1557 | 2.623949 |
| ZNF699 | 0.94262 | 0.8415 | 0.12624 | 3.484272 |
| FAM192A | 0.94262 | 0.841548 | -0.0731 | 6.807271 |
| PNISR | 0.942671 | 0.841666 | 0.233828 | 1.082634 |
| C10orf90 | 0.942742 | 0.841808 | -0.37652 | 1.004278 |
| LINC00893 | 0.942742 | 0.841971 | 0.090403 | 4.648612 |
| ZNF517 | 0.942742 | 0.842061 | -0.18707 | 1.590024 |
| LOC100506 | 0.942742 | 0.842121 | -0.19746 | 1.675883 |
| ALKBH4 | 0.942742 | 0.842198 | -0.18288 | 2.488122 |
| SNORA50C | 0.942742 | 0.842215 | 0.097358 | 6.755456 |
| LNPEP | 0.942742 | 0.842288 | -0.2397 | 1.16996 |
| FOXC2 | 0.942742 | 0.84236 | 0.348153 | 0.677513 |
| AK3 | 0.942742 | 0.842392 | -0.20892 | 2.124969 |
| TANGO6 | 0.942742 | 0.842447 | -0.0809 | 4.91981 |
| RDH10 | 0.942922 | 0.84268 | 0.089879 | 6.119178 |
| GTPBP10 | 0.942967 | 0.842792 | 0.194567 | 2.154352 |
| DCTN5 | 0.943 | 0.842894 | -0.28485 | 0.908017 |
| LLPH | 0.943106 | 0.84306 | 0.104415 | 4.837229 |
| FAM186B | 0.943373 | 0.843371 | -0.17765 | 1.955076 |
| HNRNPA1P | 0.94368 | 0.843717 | 0.078982 | 7.96779 |
| LINC01494 | 0.943882 | 0.844006 | 0.404109 | 0.464711 |
| SNHG1 | 0.943882 | 0.844073 | 0.099286 | 5.055424 |
| RGMB-AS1 | 0.943882 | 0.844114 | -0.33841 | 1.911039 |
| LOC101929 | 0.943883 | 0.844254 | -0.26344 | 1.303418 |
| ATRX | 0.943883 | 0.844258 | 0.228919 | 0.944954 |
| CNIH4 | 0.94412 | 0.844542 | -0.19795 | 2.427227 |
| ZNF726 | 0.944256 | 0.844736 | -0.25461 | 1.524312 |
| DCAF5 | 0.944396 | 0.844969 | 0.124847 | 3.586538 |
| AATBC | 0.944396 | 0.845072 | 0.126895 | 3.775375 |
| TMEM220- | 0.944396 | 0.845077 | -0.23871 | 1.318109 |
| FBXO36 | 0.944437 | 0.845185 | -0.15798 | 2.625521 |
| GLRX3 | 0.944475 | 0.845356 | 0.219888 | 1.661793 |

| | | | | |
|---|---|---|---|---|
| SCAF11 | 0.944475 | 0.845363 | -0.0691 | 8.601546 |
| C14orf28 | 0.945138 | 0.846141 | -0.12334 | 3.351706 |
| VWA1 | 0.945138 | 0.846243 | -0.19855 | 1.834783 |
| POMT1 | 0.945138 | 0.846298 | 0.242833 | 0.735565 |
| SRPK2 | 0.945138 | 0.846301 | -0.14879 | 2.3876 |
| BMS1P2 | 0.945138 | 0.846317 | -0.07458 | 6.29796 |
| IFNGR2 | 0.945233 | 0.846474 | -0.11343 | 4.975695 |
| HBB | 0.945261 | 0.846572 | -0.14998 | 3.201896 |
| ZBTB16 | 0.945407 | 0.846793 | -0.19707 | 3.047245 |
| SH2B1 | 0.945407 | 0.846861 | 0.101447 | 4.561524 |
| WEE2-AS1 | 0.945407 | 0.846918 | -0.23396 | 0.757173 |
| PRR13 | 0.945411 | 0.846994 | -0.19132 | 2.08016 |
| CAMKV | 0.945507 | 0.847152 | -0.3452 | 0.812533 |
| PRKAR1A | 0.945851 | 0.847532 | -0.14099 | 1.954358 |
| NORAD | 0.945928 | 0.847691 | 0.072073 | 9.801367 |
| RIPK1 | 0.945928 | 0.847745 | -0.25148 | 1.251995 |
| DMRTA2 | 0.945935 | 0.847824 | 0.209707 | 1.541081 |
| FAM89A | 0.945935 | 0.847945 | -0.17644 | 2.591428 |
| SLC19A2 | 0.945935 | 0.848007 | -0.08768 | 4.963165 |
| LCMT2 | 0.945935 | 0.84804 | 0.129968 | 3.348267 |
| OR7E12P | 0.946085 | 0.848285 | -0.20845 | 2.201076 |
| SRP72 | 0.946085 | 0.848319 | -0.16523 | 2.341094 |
| LOC100507 | 0.946088 | 0.848473 | -0.10494 | 3.882995 |
| YIPF4 | 0.946088 | 0.848521 | 0.072593 | 6.121275 |
| GHET1 | 0.946088 | 0.848596 | 0.342989 | 0.353278 |
| TTC13 | 0.946088 | 0.84861 | 0.299345 | 0.519537 |
| PDCD10 | 0.946143 | 0.848805 | -0.14764 | 3.034732 |
| SMIM13 | 0.946143 | 0.848875 | -0.10553 | 4.001316 |
| MYH13 | 0.946143 | 0.848875 | 0.202325 | 2.470103 |
| HIVEP2 | 0.946158 | 0.848991 | -0.07141 | 7.784848 |
| CD55 | 0.946158 | 0.849033 | -0.213 | 4.361998 |
| MTHFD2L | 0.946167 | 0.849113 | -0.10907 | 3.39061 |
| CBWD2 | 0.946201 | 0.849216 | -0.079 | 5.213185 |
| RGS5 | 0.946232 | 0.849315 | -0.16615 | 1.993676 |
| ZNF350 | 0.946427 | 0.849702 | 0.110014 | 3.72986 |
| AIMP2 | 0.946427 | 0.849707 | -0.10988 | 3.376648 |
| PSCA | 0.946427 | 0.849742 | -0.1996 | 2.333893 |
| LRRC70 | 0.946427 | 0.84978 | -0.21099 | 1.772915 |
| TMEM87A | 0.946427 | 0.849973 | -0.11001 | 3.738503 |
| LOC101928 | 0.946427 | 0.850001 | -0.22357 | 1.365012 |
| EMC3-AS1 | 0.946427 | 0.850103 | 0.151145 | 3.498711 |
| LINC00894 | 0.946427 | 0.85025 | 0.080699 | 5.105164 |
| UBASH3B | 0.946427 | 0.85027 | 0.115007 | 4.250347 |
| ZNF599 | 0.946427 | 0.850283 | -0.18591 | 1.596703 |
| AGAP12P | 0.946427 | 0.850284 | -0.08493 | 4.561162 |

| | | | | |
|---|---|---|---|---|
| LINC02043 | 0.946553 | 0.850469 | 0.2098 | 0.536351 |
| SNORD31 | 0.946569 | 0.850664 | 0.169213 | 2.577005 |
| GDAP2 | 0.946569 | 0.850665 | 0.076854 | 6.001846 |
| ALDOA | 0.946569 | 0.850745 | -0.16264 | 2.167619 |
| HERC2 | 0.946569 | 0.850772 | -0.06804 | 6.38352 |
| PUS7 | 0.946667 | 0.851018 | -0.08808 | 5.863146 |
| SEC61A1 | 0.946667 | 0.851055 | 0.071017 | 7.804795 |
| ADSSL1 | 0.946667 | 0.851147 | 0.273318 | 0.469309 |
| FOXJ3 | 0.946667 | 0.851149 | 0.182849 | 2.294396 |
| TANK | 0.94679 | 0.85139 | -0.15745 | 2.450479 |
| SLC25A42 | 0.94679 | 0.851404 | -0.10778 | 3.478724 |
| LINC00115 | 0.946859 | 0.851538 | 0.11682 | 3.214887 |
| GCNT7 | 0.94695 | 0.851692 | 0.168207 | 2.370272 |
| FAM229A | 0.947 | 0.851879 | -0.24047 | 1.020366 |
| FLNB | 0.947 | 0.851881 | -0.15701 | 2.610989 |
| GNAS-AS1 | 0.947002 | 0.851956 | -0.238 | 1.160733 |
| H3F3C | 0.94702 | 0.852095 | 0.199025 | 1.802032 |
| SENP5 | 0.94702 | 0.85215 | -0.19213 | 2.441916 |
| TSSK4 | 0.94702 | 0.852219 | -0.26044 | 0.892711 |
| LOC643387 | 0.94702 | 0.85226 | -0.23771 | 0.619865 |
| DCTN4 | 0.947216 | 0.852509 | 0.211529 | 1.206091 |
| HDGF | 0.94745 | 0.852791 | -0.11351 | 3.795442 |
| SMIM20 | 0.947756 | 0.853139 | -0.11342 | 3.298948 |
| RAB8A | 0.947878 | 0.853322 | 0.068783 | 7.023688 |
| RCOR3 | 0.947986 | 0.853541 | 0.252485 | 0.771918 |
| CENPJ | 0.947986 | 0.853563 | 0.27911 | 0.356582 |
| TAPT1 | 0.947987 | 0.853637 | -0.07383 | 4.933796 |
| EWSR1 | 0.948041 | 0.853757 | -0.12419 | 3.973468 |
| KRAS | 0.948144 | 0.853922 | 0.231942 | 0.437871 |
| SCAND1 | 0.948205 | 0.85422 | 0.336947 | 0.342336 |
| CDKN2AIPI | 0.948205 | 0.854251 | -0.13707 | 3.334696 |
| NAGPA | 0.948205 | 0.854346 | 0.144491 | 2.803913 |
| COX7A1 | 0.948205 | 0.854367 | -0.15392 | 3.050797 |
| CDC42EP4 | 0.948205 | 0.854448 | -0.08497 | 6.126029 |
| LINC01004 | 0.948205 | 0.854451 | -0.06894 | 6.756974 |
| ANP32C | 0.948205 | 0.854483 | 0.234481 | 0.804428 |
| NELFB | 0.948404 | 0.854756 | -0.07285 | 5.554431 |
| ZCCHC9 | 0.948404 | 0.85486 | -0.28752 | 0.360056 |
| EIF4A2 | 0.948404 | 0.854952 | 0.065392 | 8.745498 |
| CBWD5 | 0.948404 | 0.855069 | 0.163456 | 2.461996 |
| UBE2Z | 0.948404 | 0.85508 | 0.064853 | 6.811219 |
| LOC100132 | 0.948404 | 0.85513 | 0.126677 | 3.053147 |
| SPEN | 0.948404 | 0.855168 | 0.065657 | 9.049651 |
| NINL | 0.948438 | 0.855317 | 0.106132 | 6.540575 |
| MIR581 | 0.948438 | 0.855344 | -0.30797 | 0.736323 |

| | | | | |
|---|---|---|---|---|
| TSIX | 0.948547 | 0.855635 | 0.233753 | 3.128246 |
| CCDC167 | 0.948547 | 0.855761 | -0.11008 | 2.872891 |
| ZNF662 | 0.948547 | 0.855764 | -0.22361 | 1.518868 |
| HIGD1A | 0.948547 | 0.855796 | 0.129696 | 2.477038 |
| HIST1H2AI | 0.948547 | 0.855805 | -0.11949 | 3.897833 |
| FBXW9 | 0.948547 | 0.855875 | 0.133479 | 3.045057 |
| LINC00996 | 0.948573 | 0.855971 | 0.278422 | 1.267119 |
| ANKRD27 | 0.948752 | 0.856205 | -0.06629 | 6.326139 |
| FBXL22 | 0.948792 | 0.856336 | 0.202227 | 1.579183 |
| AHSA1 | 0.948792 | 0.856388 | 0.075392 | 6.528706 |
| SNORA71D | 0.948792 | 0.856488 | 0.088433 | 6.064269 |
| TCF4-AS1 | 0.948792 | 0.85653 | -0.27001 | 1.054145 |
| GLOD5 | 0.948859 | 0.856732 | 0.28092 | 0.878834 |
| NT5DC1 | 0.948859 | 0.856735 | -0.09357 | 4.984649 |
| ZNF175 | 0.94895 | 0.856955 | 0.099856 | 3.538315 |
| CLEC19A | 0.94895 | 0.857084 | 0.366891 | 0.925281 |
| HM13-AS1 | 0.94895 | 0.857084 | 0.162557 | 1.961963 |
| ALLC | 0.94895 | 0.857107 | 0.374154 | 0.97092 |
| LOC646214 | 0.949683 | 0.857841 | -0.14594 | 3.52578 |
| PRRC2B | 0.949747 | 0.857971 | -0.06318 | 8.876621 |
| HS2ST1 | 0.949988 | 0.858261 | 0.082331 | 5.559788 |
| TIMM8A | 0.950073 | 0.858412 | 0.130064 | 2.629356 |
| PRKAA1 | 0.950073 | 0.858491 | 0.233986 | 1.342566 |
| NAE1 | 0.950073 | 0.858556 | 0.298989 | 0.57066 |
| LACTB2-AS | 0.950183 | 0.858927 | 0.156976 | 2.883188 |
| VWA5B1 | 0.950183 | 0.858936 | 0.238956 | 1.505049 |
| KLHL20 | 0.950183 | 0.858939 | -0.07481 | 5.23719 |
| C3orf52 | 0.950183 | 0.858945 | -0.22086 | 1.250264 |
| LINC00571 | 0.950204 | 0.859128 | 0.180562 | 2.034276 |
| HDAC1 | 0.950204 | 0.859143 | -0.06896 | 7.111811 |
| MIR7161 | 0.950204 | 0.8592 | -0.23559 | 1.275222 |
| RNPC3 | 0.950204 | 0.859253 | 0.081059 | 5.420323 |
| SNORD77 | 0.950219 | 0.85939 | 0.203423 | 1.457237 |
| ZNF831 | 0.950219 | 0.859412 | -0.12742 | 2.573252 |
| TCTN3 | 0.950575 | 0.859806 | 0.227325 | 1.432171 |
| SRRM5 | 0.950722 | 0.860012 | -0.11537 | 2.938487 |
| LINC00482 | 0.950727 | 0.860146 | -0.1457 | 2.936401 |
| RNF111 | 0.950727 | 0.860276 | -0.16067 | 2.062342 |
| VAPA | 0.950727 | 0.860343 | -0.18286 | 2.355792 |
| SRP68 | 0.950727 | 0.860368 | -0.19152 | 1.406311 |
| RBM33 | 0.950727 | 0.86042 | 0.066634 | 8.60966 |
| SNRPA1 | 0.950727 | 0.860451 | -0.10157 | 4.989935 |
| CHD1 | 0.950778 | 0.860569 | 0.066809 | 7.637617 |
| LOC145474 | 0.950928 | 0.860777 | -0.15622 | 2.281578 |
| F8A1 | 0.95093 | 0.8609 | -0.16645 | 2.554664 |

| | | | | |
|---|---|---|---|---|
| MIR429 | 0.95093 | 0.860924 | 0.196434 | 1.33408 |
| NDUFB4 | 0.951185 | 0.861227 | -0.17711 | 2.100232 |
| PXK | 0.951354 | 0.861514 | 0.249998 | 0.850831 |
| FYB2 | 0.951354 | 0.861526 | 0.252426 | 2.20419 |
| PALM3 | 0.951382 | 0.861624 | -0.21174 | 5.512603 |
| FNTA | 0.951506 | 0.861809 | -0.18422 | 1.934816 |
| SMC5 | 0.951546 | 0.86195 | -0.06268 | 8.111284 |
| DUSP9 | 0.951546 | 0.86199 | -0.33454 | 0.383427 |
| PPIEL | 0.951558 | 0.862073 | 0.1396 | 3.553677 |
| NPEPL1 | 0.951699 | 0.862422 | -0.18786 | 1.156786 |
| MIR27A | 0.951699 | 0.862594 | 0.228543 | 1.433094 |
| LMBR1 | 0.951699 | 0.862599 | 0.06721 | 6.221855 |
| TCF24 | 0.951699 | 0.862846 | 0.24347 | 0.436753 |
| SNAI3-AS1 | 0.951699 | 0.862937 | 0.159635 | 1.830161 |
| PSMD3 | 0.951699 | 0.863049 | -0.06586 | 6.359201 |
| CAPN3 | 0.951699 | 0.863111 | 0.143149 | 3.191851 |
| MYO9A | 0.951699 | 0.863119 | 0.067954 | 7.664318 |
| PFDN1 | 0.951699 | 0.863147 | 0.079131 | 5.354337 |
| TAF1L | 0.951699 | 0.863234 | -0.24557 | 0.576141 |
| C11orf49 | 0.951699 | 0.863251 | 0.188943 | 1.400447 |
| FAM27B | 0.951699 | 0.863283 | 0.182159 | 2.052421 |
| NAP1L4 | 0.951699 | 0.863412 | -0.10099 | 5.697622 |
| MRPL50 | 0.951699 | 0.863422 | 0.071961 | 5.214893 |
| BORCS6 | 0.951699 | 0.863428 | -0.10812 | 3.246114 |
| JAGN1 | 0.951699 | 0.863475 | 0.128682 | 3.922176 |
| LINCR-000 | 0.951699 | 0.863529 | 0.207129 | 1.079201 |
| POU5F1P5 | 0.951699 | 0.863563 | 0.149341 | 2.245176 |
| POLQ | 0.951699 | 0.863631 | 0.080723 | 5.730688 |
| CCSER2 | 0.951699 | 0.8637 | -0.20821 | 1.528897 |
| ATXN7L2 | 0.951699 | 0.863724 | -0.13397 | 2.646255 |
| NPIPB15 | 0.951865 | 0.863991 | -0.16826 | 3.386537 |
| SHPK | 0.951865 | 0.864072 | 0.077699 | 5.033296 |
| ZNF720 | 0.951865 | 0.864114 | 0.093321 | 4.488146 |
| CENPT | 0.951865 | 0.86417 | -0.06843 | 5.628912 |
| SPCS3 | 0.951865 | 0.864237 | -0.06559 | 6.983027 |
| VMA21 | 0.95193 | 0.864368 | -0.06713 | 5.866998 |
| FIG4 | 0.952156 | 0.864646 | 0.074651 | 5.049818 |
| ABCF3 | 0.952166 | 0.864763 | 0.058998 | 5.848584 |
| SMG1P7 | 0.952166 | 0.8648 | -0.09471 | 3.697017 |
| FTH1P3 | 0.952401 | 0.865104 | -0.09273 | 3.794866 |
| XXYLT1-AS | 0.952401 | 0.865159 | -0.17204 | 1.189893 |
| HAUS5 | 0.952405 | 0.865246 | -0.0767 | 4.713714 |
| LINC01128 | 0.952405 | 0.865325 | -0.13305 | 2.913529 |
| SERTAD4 | 0.952405 | 0.865437 | -0.12651 | 3.484588 |
| NDUFAF4 | 0.952405 | 0.865554 | 0.131766 | 3.063324 |

| | | | | |
|---|---|---|---|---|
| PSMA1 | 0.952405 | 0.865574 | 0.130477 | 2.991347 |
| USP22 | 0.952405 | 0.865598 | 0.063016 | 7.678589 |
| RBM26-AS1 | 0.95256 | 0.865812 | -0.18518 | 1.792407 |
| LOC10041 | 0.952765 | 0.866071 | -0.08176 | 5.232482 |
| C5orf34 | 0.952876 | 0.866304 | -0.14655 | 2.965538 |
| RTL10 | 0.952876 | 0.866337 | 0.073618 | 5.646727 |
| AAMDC | 0.952876 | 0.866589 | 0.279323 | 0.467279 |
| ELOVL5 | 0.952876 | 0.866634 | 0.129073 | 2.918974 |
| ASTN1 | 0.952876 | 0.866718 | 0.315503 | 0.596965 |
| KIN | 0.952876 | 0.866821 | -0.17761 | 2.138703 |
| ANKS6 | 0.952876 | 0.866826 | 0.072042 | 5.984056 |
| PSMC3 | 0.952876 | 0.866852 | -0.05927 | 6.431525 |
| PHF8 | 0.952876 | 0.866877 | 0.192209 | 1.659565 |
| RCOR2 | 0.952876 | 0.866898 | 0.176486 | 1.842357 |
| ATXN10 | 0.952888 | 0.866982 | -0.22613 | 1.109586 |
| NUP93 | 0.953013 | 0.867168 | 0.099967 | 3.044337 |
| STK38L | 0.953149 | 0.867445 | 0.06263 | 7.165967 |
| CDT1 | 0.953149 | 0.867462 | 0.097244 | 3.786156 |
| MOK | 0.953149 | 0.867509 | -0.14433 | 2.461339 |
| SLC39A1 | 0.953154 | 0.867833 | 0.252124 | 0.720223 |
| PDCD4-AS1 | 0.953154 | 0.867889 | -0.15989 | 1.837742 |
| HSP90B2P | 0.953154 | 0.867917 | 0.112806 | 3.055651 |
| CCDC60 | 0.953154 | 0.867953 | -0.18604 | 1.60885 |
| AKAP17A | 0.953154 | 0.867974 | 0.185214 | 1.32973 |
| ADAMTSL4 | 0.953154 | 0.868172 | -0.25266 | 0.592256 |
| UBE2Q2P1 | 0.953154 | 0.868322 | -0.07417 | 5.408114 |
| ZZZ3 | 0.953154 | 0.868455 | 0.060711 | 5.709374 |
| ADPRM | 0.953154 | 0.868485 | -0.10664 | 3.179683 |
| MAPK8 | 0.953154 | 0.868584 | -0.21301 | 1.19877 |
| NBPF20 | 0.953154 | 0.868597 | 0.117137 | 2.720277 |
| ZNF717 | 0.953154 | 0.868608 | 0.235854 | 1.194214 |
| LARS2-AS1 | 0.953154 | 0.868647 | 0.159985 | 1.093481 |
| TMEM225 | 0.953154 | 0.868689 | -0.38558 | 0.468225 |
| MOGAT1 | 0.953154 | 0.868709 | 0.224351 | 1.455521 |
| BAG5 | 0.953154 | 0.868719 | -0.2395 | 0.868313 |
| TCP1 | 0.953154 | 0.868804 | -0.21314 | 1.405748 |
| ADNP2 | 0.953154 | 0.868929 | 0.061882 | 6.051271 |
| ABCF2 | 0.953154 | 0.868966 | -0.13235 | 3.482958 |
| MRTFB | 0.953154 | 0.868967 | 0.312956 | 0.380302 |
| LOC441081 | 0.953237 | 0.869152 | -0.09519 | 3.823818 |
| ST7-AS1 | 0.953237 | 0.869189 | 0.202522 | 1.121432 |
| ELOF1 | 0.953237 | 0.86926 | 0.075574 | 4.132796 |
| PAQR8 | 0.953302 | 0.869519 | 0.074516 | 5.865614 |
| FAM129A | 0.953302 | 0.869553 | -0.07302 | 7.63324 |
| ARHGEF12 | 0.953302 | 0.869588 | -0.21745 | 1.657258 |

| | | | | |
|---|---|---|---|---|
| LINC02035 | 0.953302 | 0.86961 | -0.07956 | 4.831549 |
| AARS2 | 0.953678 | 0.870026 | -0.06574 | 5.615877 |
| MIR5690 | 0.95407 | 0.870517 | -0.10875 | 4.857578 |
| PNKD | 0.95407 | 0.870529 | 0.116366 | 2.677074 |
| ZBED6 | 0.954079 | 0.870729 | 0.063863 | 7.486502 |
| RMRP | 0.954079 | 0.87076 | 0.060111 | 12.30485 |
| PRR34-AS1 | 0.954079 | 0.870804 | 0.08552 | 4.366405 |
| CCS | 0.954079 | 0.870869 | 0.076359 | 4.41344 |
| UBR4 | 0.954079 | 0.8709 | 0.057582 | 9.459143 |
| GTF2F1 | 0.954084 | 0.870997 | 0.0591 | 6.824182 |
| BDP1 | 0.954084 | 0.871082 | -0.07525 | 5.261151 |
| AGAP1-IT1 | 0.954084 | 0.871146 | 0.106718 | 3.251189 |
| DUSP10 | 0.954084 | 0.871196 | -0.14007 | 3.568663 |
| RBM15-AS1 | 0.954233 | 0.871405 | 0.225552 | 0.429238 |
| LIPM | 0.954549 | 0.871766 | 0.270239 | 0.343879 |
| CHAF1B | 0.95467 | 0.871949 | 0.083916 | 3.964763 |
| TRMT6 | 0.954732 | 0.872146 | -0.17576 | 1.458168 |
| MFAP3 | 0.954732 | 0.872152 | -0.18096 | 2.018249 |
| PKD2L2 | 0.954783 | 0.872313 | -0.26571 | 0.740678 |
| SLED1 | 0.954783 | 0.872344 | 0.134214 | 2.635281 |
| ZNF425 | 0.955095 | 0.872702 | -0.1268 | 2.461593 |
| RUFY1 | 0.955194 | 0.872865 | -0.12743 | 2.202999 |
| ENTPD3-AS | 0.9552 | 0.872943 | 0.129857 | 1.997973 |
| FZD6 | 0.955254 | 0.873065 | 0.288649 | 0.362101 |
| LOC103344 | 0.955274 | 0.873165 | 0.084759 | 4.103607 |
| IQCD | 0.955274 | 0.873229 | -0.19281 | 1.530224 |
| NSD3 | 0.955431 | 0.873446 | 0.059484 | 6.506845 |
| DHX38 | 0.955489 | 0.873575 | -0.05963 | 7.369065 |
| SNORD14D | 0.955489 | 0.873683 | 0.098451 | 4.664765 |
| MEIS1-AS2 | 0.955489 | 0.873717 | 0.127815 | 2.844398 |
| RBM23 | 0.955565 | 0.873859 | 0.145699 | 1.85551 |
| TMEM128 | 0.955671 | 0.874029 | 0.218544 | 0.649522 |
| PRR14 | 0.955729 | 0.874155 | 0.062753 | 5.164364 |
| EPRS | 0.955884 | 0.874369 | 0.061365 | 8.221401 |
| ARV1 | 0.956048 | 0.874593 | 0.086728 | 4.412771 |
| PLEKHG4B | 0.956083 | 0.874713 | 0.149546 | 3.507962 |
| USP54 | 0.956083 | 0.874806 | -0.07088 | 7.493914 |
| MIR4449 | 0.956083 | 0.874843 | 0.09492 | 4.275517 |
| FAM149B1 | 0.956212 | 0.875034 | 0.065316 | 5.162602 |
| SPAG9 | 0.956264 | 0.875262 | -0.14598 | 2.850641 |
| ALKBH3 | 0.956264 | 0.875286 | 0.073295 | 4.427206 |
| CAB39 | 0.956264 | 0.8753 | 0.084437 | 3.935346 |
| USP3-AS1 | 0.956684 | 0.875762 | 0.113994 | 2.396593 |
| YRDC | 0.956684 | 0.875831 | 0.096848 | 3.708643 |
| ARHGAP11 | 0.95671 | 0.875927 | -0.22944 | 0.61648 |

| | | | | |
|---|---|---|---|---|
| FAM117A | 0.956831 | 0.876111 | -0.07782 | 3.643167 |
| TMEM191/ | 0.956886 | 0.876234 | -0.19478 | 0.858762 |
| TOP3B | 0.956887 | 0.876308 | -0.14631 | 1.597365 |
| RETREG2 | 0.956924 | 0.87642 | 0.055894 | 6.101606 |
| SNORA65 | 0.956924 | 0.876487 | -0.09054 | 7.350635 |
| KMO | 0.957091 | 0.876831 | 0.182142 | 1.908802 |
| MGAT4B | 0.957091 | 0.876834 | 0.098924 | 3.48565 |
| ACOT1 | 0.957091 | 0.87691 | -0.31085 | 0.599001 |
| FBXW2 | 0.957091 | 0.876941 | -0.05721 | 7.088021 |
| DNAJB12 | 0.957091 | 0.877005 | 0.123295 | 2.686102 |
| SNORD14A | 0.957145 | 0.87722 | 0.088207 | 5.28673 |
| TRMT61A | 0.957145 | 0.87733 | 0.075003 | 3.834516 |
| HIST1H2BN | 0.957145 | 0.877384 | 0.083183 | 4.1781 |
| RNF2 | 0.957145 | 0.877393 | -0.08819 | 4.918247 |
| LOC101927 | 0.957145 | 0.877474 | 0.279142 | 0.898818 |
| C6orf141 | 0.957145 | 0.877492 | -0.10696 | 3.002093 |
| RUBCN | 0.957251 | 0.87767 | -0.11374 | 2.415015 |
| RFNG | 0.957251 | 0.877735 | 0.075008 | 4.013962 |
| HIST1H2AK | 0.957371 | 0.87792 | -0.0998 | 2.811367 |
| HIST1H2AE | 0.957371 | 0.877996 | 0.074036 | 5.641257 |
| RNF32 | 0.957371 | 0.878065 | -0.14942 | 2.275779 |
| EIF5 | 0.957456 | 0.878215 | -0.12628 | 2.807996 |
| CDC37L1 | 0.957515 | 0.878379 | 0.063977 | 5.570639 |
| TOP1 | 0.957515 | 0.878415 | -0.05843 | 8.533271 |
| MED13 | 0.957529 | 0.878517 | -0.055 | 8.454445 |
| CHGB | 0.957529 | 0.878691 | -0.23362 | 1.815302 |
| BTG1 | 0.957529 | 0.878742 | 0.061987 | 7.262638 |
| STRADA | 0.957529 | 0.87882 | 0.131604 | 2.179742 |
| HECTD3 | 0.957529 | 0.878836 | 0.059116 | 6.276544 |
| ZFP64 | 0.957529 | 0.878866 | 0.112992 | 2.503499 |
| HNRNPA0 | 0.957695 | 0.879092 | 0.057733 | 6.677358 |
| GCN1 | 0.957695 | 0.879256 | -0.0629 | 8.218269 |
| KCP | 0.957695 | 0.87927 | -0.10543 | 2.902205 |
| LIN37 | 0.957695 | 0.879361 | 0.11827 | 3.01748 |
| TNFRSF21 | 0.957695 | 0.879477 | 0.071486 | 7.019301 |
| LINC00900 | 0.957695 | 0.879522 | 0.22131 | 0.478377 |
| BAG4 | 0.957695 | 0.879575 | -0.19781 | 1.058946 |
| PNRC1 | 0.957695 | 0.8797 | 0.058561 | 7.520655 |
| UROS | 0.957695 | 0.879756 | -0.07077 | 4.064301 |
| LINC01012 | 0.957695 | 0.879833 | 0.230051 | 0.787131 |
| RNF123 | 0.957695 | 0.879976 | 0.065368 | 5.496869 |
| CMTM4 | 0.957695 | 0.879987 | -0.06502 | 5.770579 |
| MXD4 | 0.957695 | 0.88002 | -0.06462 | 5.040515 |
| LSM12 | 0.957695 | 0.880108 | -0.07963 | 5.386146 |
| SLC37A4 | 0.957695 | 0.880113 | 0.245333 | 0.672009 |

| | | | | |
|---|---|---|---|---|
| SMIM18 | 0.957895 | 0.880458 | -0.18624 | 1.313086 |
| ZNF192P1 | 0.957895 | 0.880536 | 0.172031 | 2.070124 |
| GHR | 0.957895 | 0.880558 | -0.2035 | 1.869693 |
| TMEM144 | 0.957895 | 0.880604 | 0.071113 | 4.296908 |
| WNT3A | 0.957895 | 0.880662 | 0.183563 | 1.242316 |
| HGH1 | 0.957953 | 0.88088 | 0.079219 | 3.847049 |
| EP400P1 | 0.957953 | 0.880905 | 0.098374 | 3.200483 |
| RUNX3 | 0.957953 | 0.880934 | 0.161318 | 1.627916 |
| ELAC1 | 0.958028 | 0.881076 | 0.105703 | 2.773578 |
| HIGD2A | 0.958071 | 0.881189 | -0.08019 | 3.320052 |
| MAGOH2P | 0.958071 | 0.881261 | 0.171073 | 1.74138 |
| CIRBP-AS1 | 0.958185 | 0.881538 | 0.10979 | 3.055378 |
| LINC00536 | 0.958185 | 0.881566 | -0.2678 | 0.934668 |
| NXF3 | 0.958185 | 0.881586 | -0.3412 | 0.475866 |
| PIGL | 0.958258 | 0.881726 | 0.084098 | 3.766082 |
| GJC2 | 0.958319 | 0.881855 | 0.228704 | 0.917518 |
| PSMG3 | 0.95838 | 0.881984 | -0.14162 | 1.503214 |
| SERP1 | 0.958408 | 0.882102 | -0.05933 | 6.998355 |
| SNX9 | 0.958408 | 0.882201 | 0.058388 | 7.605032 |
| SEC13 | 0.958408 | 0.882229 | -0.21377 | 1.322544 |
| RPL23AP64 | 0.95846 | 0.882451 | -0.1391 | 3.124324 |
| SCARNA23 | 0.95846 | 0.882483 | 0.071741 | 4.727767 |
| PPM1N | 0.95846 | 0.882496 | -0.13792 | 1.8141 |
| B3GLCT | 0.958781 | 0.882865 | 0.076909 | 4.018946 |
| SNORD116 | 0.958852 | 0.883056 | 0.22248 | 0.410262 |
| RHEX | 0.958852 | 0.883076 | 0.179313 | 1.640406 |
| WDR55 | 0.958884 | 0.883179 | -0.06147 | 5.471213 |
| WBP2NL | 0.959038 | 0.883433 | 0.206722 | 1.203457 |
| CIAO2A | 0.959038 | 0.883467 | -0.08914 | 3.502696 |
| CDH8 | 0.959171 | 0.883662 | 0.226402 | 0.675841 |
| MEF2A | 0.959197 | 0.883759 | 0.158602 | 1.681453 |
| CD52 | 0.959215 | 0.883849 | -0.14796 | 2.02369 |
| KLHDC2 | 0.959237 | 0.883978 | -0.05622 | 5.588877 |
| MGC27345 | 0.959237 | 0.884015 | 0.055688 | 5.257405 |
| SNORA51 | 0.959633 | 0.884554 | 0.076682 | 5.220948 |
| UAP1 | 0.959633 | 0.884557 | -0.06219 | 6.539296 |
| CYB5R3 | 0.959633 | 0.8846 | 0.155681 | 1.782322 |
| RAD54L | 0.959681 | 0.884717 | 0.201081 | 0.863337 |
| CIAO1 | 0.959722 | 0.884865 | 0.052999 | 6.302613 |
| TRA2B | 0.959722 | 0.884901 | 0.094014 | 2.698823 |
| RRP8 | 0.95976 | 0.88501 | 0.065609 | 4.571666 |
| RAVER2 | 0.960255 | 0.885539 | -0.05645 | 5.332609 |
| COL18A1 | 0.96041 | 0.885755 | 0.176064 | 1.829501 |
| SENP2 | 0.960436 | 0.885853 | -0.05526 | 6.196428 |
| TBK1 | 0.960476 | 0.886016 | 0.05392 | 6.063672 |

| | | | | |
|---|---|---|---|---|
| TMEM183B | 0.960476 | 0.886074 | 0.113936 | 2.685368 |
| ABCC10 | 0.960476 | 0.886123 | -0.12541 | 2.595403 |
| NRBP2 | 0.960476 | 0.886182 | 0.056705 | 6.057293 |
| MCM3AP | 0.960515 | 0.886425 | 0.052105 | 7.452285 |
| GNG12-AS1 | 0.960515 | 0.886555 | 0.089917 | 3.008279 |
| SNHG15 | 0.960515 | 0.886556 | 0.088603 | 3.341054 |
| CCNL2 | 0.960515 | 0.886561 | -0.05464 | 7.415044 |
| MKKS | 0.960515 | 0.886756 | -0.1551 | 1.333329 |
| PLEKHG7 | 0.960515 | 0.886767 | -0.15042 | 2.474353 |
| HMGA1 | 0.960515 | 0.886802 | -0.19363 | 1.74429 |
| MIR6758 | 0.960515 | 0.88686 | -0.14103 | 1.40601 |
| TMPO | 0.960515 | 0.886877 | 0.062017 | 4.929315 |
| NCOR1 | 0.960519 | 0.887036 | 0.06588 | 4.435562 |
| UBALD1 | 0.960519 | 0.887065 | -0.06479 | 4.139841 |
| LOC101927 | 0.960519 | 0.887145 | -0.24427 | 0.484816 |
| PAWR | 0.960519 | 0.887174 | -0.05231 | 6.656659 |
| H2AFX | 0.960586 | 0.887309 | -0.06894 | 4.657271 |
| LUC7L2 | 0.960629 | 0.887477 | 0.079265 | 4.581025 |
| RAB3GAP2 | 0.960629 | 0.887494 | 0.05207 | 6.832907 |
| ZFR | 0.960814 | 0.887883 | -0.0531 | 7.582926 |
| LINC02421 | 0.960814 | 0.88792 | -0.19727 | 1.022888 |
| TP53TG5 | 0.960814 | 0.887948 | -0.14574 | 1.478235 |
| LOC284454 | 0.960814 | 0.887958 | -0.09753 | 6.411962 |
| PLEKHF2 | 0.961034 | 0.888235 | -0.06569 | 5.453482 |
| TRMT10C | 0.961231 | 0.88849 | 0.06443 | 4.780584 |
| GOLPH3 | 0.961422 | 0.88874 | 0.048696 | 6.826921 |
| LVRN | 0.96165 | 0.889024 | 0.244095 | 0.640236 |
| LINC01006 | 0.961774 | 0.889218 | 0.121761 | 2.760769 |
| DDX47 | 0.961774 | 0.889353 | 0.178918 | 1.22174 |
| MAU2 | 0.961774 | 0.889394 | 0.051843 | 6.495001 |
| SNORD53 | 0.961774 | 0.889432 | -0.12915 | 2.108725 |
| ZNF552 | 0.962037 | 0.889749 | -0.06047 | 5.228812 |
| UBE2O | 0.962343 | 0.890209 | 0.053755 | 6.230309 |
| ACOT4 | 0.962343 | 0.890236 | 0.172876 | 1.384167 |
| OR51E1 | 0.962343 | 0.890252 | 0.271158 | 1.144125 |
| THRAP3 | 0.962368 | 0.890461 | -0.05042 | 9.017126 |
| FOS | 0.962368 | 0.890463 | -0.1223 | 8.91667 |
| NDUFV2 | 0.962368 | 0.890495 | -0.06986 | 6.686095 |
| SNORD22 | 0.962435 | 0.890754 | 0.066733 | 8.124065 |
| LOC101927 | 0.962435 | 0.890791 | -0.29511 | 0.7503 |
| LINC00664 | 0.962435 | 0.890912 | -0.17352 | 1.080297 |
| CCAR2 | 0.962435 | 0.890956 | 0.17126 | 1.138798 |
| TNFRSF13C | 0.962435 | 0.890967 | 0.233902 | 0.413051 |
| RNF25 | 0.962435 | 0.891066 | -0.08334 | 3.443118 |
| SRFBP1 | 0.962435 | 0.89107 | -0.05808 | 4.901754 |

| | | | | |
|---|---|---|---|---|
| PIPOX | 0.962598 | 0.891396 | 0.167747 | 1.614535 |
| GFM2 | 0.962598 | 0.891433 | 0.269407 | 0.600163 |
| FAM167A | 0.962598 | 0.89147 | 0.129318 | 3.27741 |
| CEP83-DT | 0.962598 | 0.891514 | 0.226773 | 0.375645 |
| OPN3 | 0.962613 | 0.891602 | -0.09537 | 2.578769 |
| ECM2 | 0.962715 | 0.89177 | -0.21409 | 0.661316 |
| C12orf60 | 0.962871 | 0.891988 | -0.25512 | 0.419806 |
| SMAD3 | 0.962898 | 0.892158 | 0.081887 | 4.142017 |
| ACADSB | 0.962898 | 0.892159 | -0.05843 | 5.253144 |
| ZNF7 | 0.962934 | 0.892356 | 0.115632 | 2.301921 |
| SNORA11B | 0.962934 | 0.892399 | -0.11128 | 2.667411 |
| SPRR2G | 0.962934 | 0.892413 | -0.28611 | 1.135153 |
| HNRNPF | 0.963208 | 0.89274 | 0.101418 | 2.821552 |
| PRSS16 | 0.963418 | 0.893008 | 0.098496 | 3.102153 |
| QKI | 0.963466 | 0.893254 | -0.09507 | 2.982649 |
| TRUB2 | 0.963466 | 0.893277 | -0.07454 | 4.360303 |
| HUS1B | 0.963466 | 0.893324 | -0.18063 | 1.20562 |
| WBP1L | 0.963466 | 0.893429 | 0.113436 | 2.321478 |
| UCKL1 | 0.963466 | 0.893472 | -0.14594 | 1.387945 |
| ZNF337-AS | 0.963466 | 0.893493 | 0.086556 | 3.156192 |
| ZNF529-AS | 0.963672 | 0.894017 | -0.22321 | 0.91042 |
| OR10AD1 | 0.963672 | 0.894022 | 0.225309 | 0.678274 |
| NADK | 0.963672 | 0.894049 | 0.097293 | 2.656313 |
| ELP1 | 0.963672 | 0.894191 | 0.0496 | 6.389251 |
| SERTAD3 | 0.963672 | 0.894285 | 0.12476 | 1.580684 |
| CFAP70 | 0.963672 | 0.894301 | 0.123365 | 2.7167 |
| SNORA48 | 0.963672 | 0.894334 | 0.064585 | 7.74599 |
| RNF183 | 0.963672 | 0.894385 | -0.21356 | 1.168276 |
| KCNJ14 | 0.963672 | 0.89444 | 0.094931 | 2.381185 |
| CCDC89 | 0.963672 | 0.894481 | 0.187814 | 1.234841 |
| CEBPA-DT | 0.963672 | 0.894507 | 0.149073 | 1.385528 |
| TNFSF9 | 0.963672 | 0.89465 | -0.12987 | 2.844703 |
| NTPCR | 0.963672 | 0.894725 | -0.06375 | 4.369026 |
| NUDT11 | 0.963672 | 0.894784 | 0.169124 | 0.989806 |
| TBC1D10B | 0.963672 | 0.894786 | 0.048229 | 6.243472 |
| KIAA1958 | 0.963698 | 0.894903 | -0.26564 | 1.052228 |
| MFSD8 | 0.963698 | 0.894957 | 0.055354 | 5.166709 |
| MBD1 | 0.963893 | 0.895221 | -0.11776 | 1.906729 |
| SAC3D1 | 0.963893 | 0.895285 | 0.118169 | 2.590214 |
| SELENOH | 0.9639 | 0.895368 | -0.05583 | 5.274898 |
| PPP1R9B | 0.9639 | 0.895439 | 0.048108 | 7.188738 |
| MMP28 | 0.96402 | 0.895677 | 0.153747 | 1.089576 |
| SMARCA5-. | 0.96402 | 0.895762 | -0.15999 | 0.503054 |
| DBIL5P | 0.96402 | 0.89577 | 0.19236 | 1.256502 |
| TECPR2 | 0.96446 | 0.89626 | 0.10039 | 3.553001 |

| | | | | |
|---|---|---|---|---|
| CSMD1 | 0.96446 | 0.896384 | 0.149807 | 2.212341 |
| RGPD3 | 0.96446 | 0.896399 | -0.12874 | 2.39304 |
| FKSG29 | 0.964547 | 0.896554 | -0.13118 | 1.699575 |
| FBXO48 | 0.964614 | 0.89669 | 0.10884 | 2.151536 |
| CFAP58-DT | 0.964666 | 0.896812 | 0.21321 | 0.789826 |
| SYNJ2-IT1 | 0.964676 | 0.896894 | -0.10186 | 2.156132 |
| EXO5 | 0.964841 | 0.897159 | 0.106121 | 2.971582 |
| UVSSA | 0.964841 | 0.897195 | 0.209483 | 1.433487 |
| AKAP13 | 0.964845 | 0.897279 | 0.085304 | 3.079531 |
| RIOK2 | 0.964845 | 0.897386 | -0.09228 | 2.657391 |
| ANKRD12 | 0.964845 | 0.897419 | -0.13352 | 2.355243 |
| FOCAD | 0.964883 | 0.897528 | -0.05132 | 5.782225 |
| RAB30 | 0.965132 | 0.897953 | -0.17265 | 0.757492 |
| CAPN13 | 0.965132 | 0.897955 | -0.13517 | 3.81015 |
| LRRC56 | 0.965132 | 0.898054 | -0.16526 | 0.863257 |
| TMEM184( | 0.965132 | 0.898086 | 0.04895 | 5.46791 |
| BRPF3 | 0.965132 | 0.898127 | 0.049416 | 6.430429 |
| ATP8A1 | 0.965137 | 0.898207 | 0.231695 | 0.704467 |
| LOC100506 | 0.965137 | 0.89828 | -0.05935 | 5.696524 |
| PDCD11 | 0.965154 | 0.898369 | 0.054156 | 7.011174 |
| AATF | 0.965246 | 0.898533 | 0.101487 | 3.011538 |
| MAP4K4 | 0.965246 | 0.898601 | -0.07172 | 3.433538 |
| LIPT2 | 0.965258 | 0.898686 | -0.16781 | 1.117196 |
| BCL7C | 0.965276 | 0.898776 | -0.14055 | 1.706193 |
| WASH7P | 0.965293 | 0.898866 | -0.05995 | 4.502766 |
| GABRR2 | 0.965852 | 0.899487 | 0.183036 | 1.358773 |
| SUGP2 | 0.965852 | 0.899533 | -0.05221 | 4.975232 |
| ARL16 | 0.965885 | 0.899638 | 0.059256 | 4.336028 |
| RGS9BP | 0.965968 | 0.899852 | -0.17277 | 0.631597 |
| LOC286437 | 0.965968 | 0.899862 | 0.086321 | 3.507773 |
| PREP | 0.966066 | 0.900033 | -0.04712 | 5.98917 |
| MRPL15 | 0.966066 | 0.900101 | -0.05716 | 5.108195 |
| MIR6081 | 0.966095 | 0.90024 | -0.13002 | 1.652561 |
| TMEM94 | 0.966095 | 0.900276 | 0.050496 | 6.194111 |
| MAP3K14-/ | 0.966329 | 0.900672 | 0.172279 | 1.143616 |
| FYCO1 | 0.966329 | 0.901002 | -0.04578 | 7.497519 |
| SNPH | 0.966329 | 0.901035 | 0.089199 | 3.786114 |
| STAG3L2 | 0.966329 | 0.901047 | -0.05236 | 4.799705 |
| NAB1 | 0.966329 | 0.90112 | 0.050578 | 7.652598 |
| NFIX | 0.966329 | 0.901125 | -0.10415 | 1.933613 |
| OSBPL10-A | 0.966329 | 0.901167 | -0.1413 | 2.143211 |
| MSRB2 | 0.966329 | 0.901268 | -0.06868 | 3.858444 |
| EPG5 | 0.966329 | 0.901272 | -0.04758 | 6.849922 |
| ZC3H4 | 0.966329 | 0.901323 | 0.046898 | 7.245071 |
| SNORD18B | 0.966329 | 0.901437 | -0.22844 | 0.436241 |

| | | | | |
|---|---|---|---|---|
| PHF21A | 0.966329 | 0.901491 | 0.0997 | 2.488962 |
| MRPL34 | 0.966329 | 0.901539 | 0.076978 | 3.470934 |
| ZNF814 | 0.966329 | 0.901574 | 0.048669 | 5.616165 |
| SPTY2D1 | 0.966329 | 0.901635 | 0.046905 | 6.444871 |
| LINC01697 | 0.966329 | 0.901671 | 0.162351 | 1.25105 |
| ERV3-1 | 0.966515 | 0.901919 | -0.0732 | 6.480483 |
| ACTR1B | 0.966515 | 0.902175 | -0.04795 | 5.498797 |
| MDC1 | 0.966515 | 0.902233 | 0.097333 | 4.987613 |
| WBP11 | 0.966515 | 0.902238 | -0.04638 | 6.882945 |
| PPP2R5C | 0.966515 | 0.902324 | 0.101765 | 2.581012 |
| CAPN1 | 0.966515 | 0.902384 | -0.12921 | 1.737391 |
| SMAD1-AS | 0.966515 | 0.902435 | 0.122053 | 1.908916 |
| PSMD9 | 0.966515 | 0.902493 | 0.186512 | 0.638796 |
| NCAPG2 | 0.966515 | 0.902634 | 0.129936 | 1.899872 |
| LINC00261 | 0.966515 | 0.90267 | 0.178858 | 4.781639 |
| CYB5D1 | 0.966515 | 0.902703 | 0.048167 | 5.866702 |
| SNORD16 | 0.966515 | 0.902754 | -0.07308 | 5.502234 |
| LOC10042( | 0.966515 | 0.902947 | -0.28943 | 0.627822 |
| STAG2 | 0.966515 | 0.90295 | -0.09723 | 1.829863 |
| GTF3C2 | 0.966515 | 0.902966 | -0.12768 | 2.064072 |
| POMP | 0.966515 | 0.903023 | -0.04928 | 5.961582 |
| ERG | 0.966741 | 0.903332 | 0.142048 | 1.555937 |
| ACADL | 0.966741 | 0.903382 | 0.196165 | 1.54508 |
| LOC728095 | 0.966772 | 0.903485 | -0.21288 | 0.697772 |
| LMO3 | 0.966885 | 0.903813 | 0.174536 | 1.914777 |
| CACNG4 | 0.966885 | 0.903838 | -0.20235 | 0.80017 |
| DDX50 | 0.966885 | 0.903887 | 0.048841 | 5.692391 |
| AMIGO3 | 0.966885 | 0.903954 | -0.0773 | 3.811359 |
| FAM53B | 0.966885 | 0.903959 | 0.047361 | 6.200213 |
| LINC01948 | 0.966952 | 0.904095 | 0.193299 | 0.496812 |
| TMUB2 | 0.966959 | 0.904222 | -0.14435 | 1.76494 |
| SNORD44 | 0.966959 | 0.904297 | 0.118331 | 2.153658 |
| IFT80 | 0.966959 | 0.904374 | -0.12533 | 1.698019 |
| SNORD83A | 0.966959 | 0.904441 | -0.1029 | 2.533708 |
| PRPF4B | 0.966959 | 0.904495 | 0.042649 | 8.248525 |
| PASK | 0.966959 | 0.904561 | -0.0788 | 3.459752 |
| ZBTB1 | 0.966959 | 0.904654 | -0.05544 | 4.706298 |
| DEPTOR | 0.966959 | 0.904691 | -0.13767 | 2.336652 |
| WDFY1 | 0.966986 | 0.904873 | 0.04263 | 6.844045 |
| NPC2 | 0.966986 | 0.904882 | 0.051323 | 5.068801 |
| SECISBP2L | 0.966986 | 0.904944 | -0.17417 | 0.716529 |
| SOCS7 | 0.966986 | 0.905011 | -0.10568 | 3.721691 |
| TCEANC2 | 0.967108 | 0.905261 | 0.053159 | 4.817304 |
| MFAP3L | 0.967108 | 0.905349 | -0.3051 | 0.644506 |
| ERBIN | 0.967108 | 0.905423 | -0.10776 | 2.329134 |

| | | | | |
|---|---|---|---|---|
| MRPS6 | 0.967108 | 0.905447 | -0.0485 | 5.235876 |
| MIR1206 | 0.967108 | 0.905494 | -0.15666 | 1.399528 |
| ANAPC5 | 0.967582 | 0.906011 | -0.09804 | 2.375779 |
| IFNAR1 | 0.967631 | 0.906348 | -0.04338 | 6.59619 |
| CENPM | 0.967631 | 0.906372 | 0.178911 | 0.725869 |
| TMCO4 | 0.967631 | 0.906521 | -0.05609 | 5.113875 |
| XPO1 | 0.967631 | 0.906531 | 0.042801 | 8.046654 |
| DDX5 | 0.967631 | 0.906559 | -0.04209 | 10.12352 |
| LOC101927 | 0.967631 | 0.906631 | 0.246173 | 0.786601 |
| BMS1P4 | 0.967631 | 0.906699 | -0.05307 | 4.614645 |
| FARP2 | 0.967631 | 0.906855 | 0.069282 | 3.761672 |
| CARD8-AS1 | 0.967631 | 0.906861 | -0.14631 | 1.783383 |
| AIRN | 0.967631 | 0.906865 | 0.095143 | 2.78071 |
| KCNC4 | 0.967631 | 0.906922 | 0.184007 | 0.830511 |
| ITGB5 | 0.967631 | 0.906942 | -0.04801 | 7.163344 |
| GUSBP5 | 0.968085 | 0.907441 | 0.156091 | 0.776198 |
| TARDBP | 0.968105 | 0.907555 | -0.04022 | 7.981045 |
| MRPL16 | 0.968105 | 0.907608 | 0.054974 | 4.625999 |
| MON1B | 0.968211 | 0.907781 | -0.08722 | 2.692605 |
| ANGPTL7 | 0.968896 | 0.908497 | 0.078821 | 3.098391 |
| IGSF10 | 0.969024 | 0.908691 | 0.077237 | 3.528354 |
| GTF2H4 | 0.96907 | 0.908925 | -0.13126 | 0.658369 |
| ALDOB | 0.96907 | 0.909045 | -0.1936 | 2.583763 |
| SH3YL1 | 0.96907 | 0.909148 | 0.050464 | 5.038337 |
| FBXO33 | 0.96907 | 0.909177 | -0.0512 | 4.750181 |
| HINT2 | 0.96907 | 0.909299 | -0.07637 | 3.232986 |
| ZFYVE16 | 0.96907 | 0.909303 | -0.12134 | 2.033788 |
| LYPLAL1 | 0.96907 | 0.909328 | -0.14324 | 1.660435 |
| ASPDH | 0.96907 | 0.90934 | -0.1212 | 1.832302 |
| CFAP97 | 0.96907 | 0.909457 | -0.05007 | 5.043916 |
| ZNF264 | 0.96907 | 0.909473 | -0.04151 | 7.118419 |
| SYK | 0.969132 | 0.909615 | -0.23739 | 0.224955 |
| TAF5L | 0.969132 | 0.909733 | 0.065658 | 4.458873 |
| RASGRP2 | 0.969132 | 0.909752 | 0.146964 | 1.351186 |
| SMIM17 | 0.969165 | 0.909857 | -0.16742 | 0.613514 |
| PRPF31 | 0.969455 | 0.910376 | -0.116 | 1.982437 |
| TTLL3 | 0.969455 | 0.910483 | 0.084956 | 2.374912 |
| SULT1B1 | 0.969455 | 0.910521 | 0.099481 | 3.605084 |
| CHRNB1 | 0.969455 | 0.910544 | 0.086634 | 3.014516 |
| LINC00839 | 0.969455 | 0.910648 | -0.11862 | 1.187844 |
| VPS33A | 0.969455 | 0.910753 | 0.052067 | 4.716467 |
| TMEM39B | 0.969455 | 0.910785 | -0.07938 | 2.915789 |
| MIR570 | 0.969455 | 0.910857 | 0.080792 | 2.691278 |
| KBTBD7 | 0.969455 | 0.910867 | -0.07824 | 3.705308 |
| USP46 | 0.969455 | 0.910914 | -0.21393 | 0.596411 |

| | | | | |
|---|---|---|---|---|
| BAK1 | 0.969455 | 0.910942 | 0.05605 | 4.583013 |
| RIN1 | 0.969455 | 0.911016 | -0.06625 | 4.10535 |
| VDR | 0.969609 | 0.911234 | 0.156152 | 1.434317 |
| MIS18A | 0.969773 | 0.911462 | -0.05432 | 3.835196 |
| ARFGEF2 | 0.969791 | 0.911554 | 0.039536 | 8.126211 |
| ASCC3 | 0.969956 | 0.91183 | -0.04126 | 6.804311 |
| ULK2 | 0.969956 | 0.911856 | 0.088033 | 3.296041 |
| SMC2-AS1 | 0.970469 | 0.912413 | -0.20836 | 0.934802 |
| NSFP1 | 0.970583 | 0.912666 | 0.120884 | 1.052016 |
| C2CD4B | 0.970583 | 0.912668 | -0.18203 | 1.669656 |
| ZC3H12A | 0.970657 | 0.912837 | 0.065696 | 6.098526 |
| SEH1L | 0.970657 | 0.912911 | -0.07921 | 4.254614 |
| PAK3 | 0.970657 | 0.912959 | -0.19166 | 1.021394 |
| SKIDA1 | 0.970672 | 0.913047 | -0.10566 | 2.098126 |
| SLC16A10 | 0.970677 | 0.913172 | 0.113277 | 2.971147 |
| AKAP8L | 0.970677 | 0.913212 | -0.14436 | 0.877117 |
| HIC2 | 0.970677 | 0.913274 | 0.047768 | 4.968689 |
| KIRREL1 | 0.970964 | 0.913617 | -0.16639 | 1.040638 |
| MEA1 | 0.971382 | 0.914087 | -0.04253 | 5.709799 |
| OVGP1 | 0.971382 | 0.914181 | 0.096439 | 2.135737 |
| HIST1H1D | 0.971382 | 0.914342 | 0.043906 | 5.638053 |
| LOC10049 | 0.971382 | 0.914364 | -0.18861 | 0.533823 |
| ZNF217 | 0.971382 | 0.914381 | -0.03926 | 7.314167 |
| ORC5 | 0.971416 | 0.914541 | -0.09507 | 2.623507 |
| PRKRIP1 | 0.971416 | 0.914561 | 0.179834 | 0.984077 |
| PRR11 | 0.971453 | 0.914694 | -0.07946 | 4.342848 |
| AGO4 | 0.971453 | 0.914786 | 0.038374 | 6.727384 |
| ANP32A | 0.971453 | 0.914819 | -0.04502 | 6.966897 |
| DXO | 0.971463 | 0.914909 | -0.16031 | 0.318957 |
| SMIM26 | 0.971463 | 0.914976 | -0.06444 | 3.255981 |
| UBA6-AS1 | 0.971545 | 0.915179 | 0.073182 | 3.016859 |
| SLC7A14 | 0.971545 | 0.915201 | 0.178054 | 1.511004 |
| GGT8P | 0.971674 | 0.9154 | -0.12499 | 3.571953 |
| PMS2P5 | 0.971674 | 0.915506 | 0.102166 | 2.017845 |
| MEG3 | 0.971674 | 0.915615 | -0.07158 | 6.159696 |
| VWA5B2 | 0.971674 | 0.915689 | -0.12183 | 0.534543 |
| GULP1 | 0.971674 | 0.915693 | 0.201425 | 0.402226 |
| NPY | 0.97211 | 0.916278 | -0.23639 | 0.564327 |
| PCNX4 | 0.97211 | 0.916323 | 0.043394 | 5.228366 |
| HERPUD2 | 0.97211 | 0.916326 | 0.040736 | 5.914593 |
| SNORA16A | 0.972152 | 0.91644 | 0.063431 | 4.263632 |
| BTN2A1 | 0.972633 | 0.917026 | -0.16878 | 1.013576 |
| KIAA1841 | 0.972633 | 0.917065 | -0.10027 | 2.597258 |
| LOC10144 | 0.972633 | 0.917159 | -0.18488 | 0.910132 |
| TCAP | 0.972633 | 0.91719 | 0.146189 | 0.825766 |

| | | | | |
|---|---|---|---|---|
| LOC10272З | 0.972756 | 0.91738 | -0.12526 | 0.885192 |
| PM20D1 | 0.972812 | 0.917507 | 0.095966 | 3.893249 |
| PPIB | 0.972812 | 0.917631 | 0.041672 | 6.821562 |
| FAIM | 0.972812 | 0.917693 | -0.16753 | 0.838358 |
| NPM1 | 0.972812 | 0.917746 | 0.072342 | 2.831841 |
| FBXW4 | 0.972812 | 0.917803 | -0.04733 | 6.403559 |
| SPATA25 | 0.972815 | 0.91788 | 0.112293 | 1.400264 |
| CREB3L2 | 0.972819 | 0.917963 | -0.03977 | 7.806465 |
| HIST1H1E | 0.972819 | 0.918167 | 0.043126 | 6.959829 |
| TSTD3 | 0.972819 | 0.918167 | 0.065118 | 3.860084 |
| SGMS1 | 0.972819 | 0.918205 | -0.04085 | 5.549528 |
| SOD1 | 0.972819 | 0.918255 | 0.039923 | 7.075374 |
| MICU2 | 0.972944 | 0.918446 | -0.04396 | 5.838074 |
| CAPZA2 | 0.97303 | 0.918671 | 0.043141 | 6.92639 |
| SNHG19 | 0.97303 | 0.91872 | -0.08548 | 2.970985 |
| GPD2 | 0.97303 | 0.918751 | 0.12169 | 0.993234 |
| DOK4 | 0.973049 | 0.918842 | -0.04213 | 5.763135 |
| CALB1 | 0.9732 | 0.919059 | 0.127075 | 0.856133 |
| INAVA | 0.973219 | 0.919178 | 0.10242 | 1.623086 |
| KCNK1 | 0.973219 | 0.919225 | -0.05665 | 5.591907 |
| C8orf33 | 0.973398 | 0.919469 | 0.043678 | 5.178846 |
| RANBP1 | 0.973634 | 0.919802 | -0.21239 | 0.381997 |
| TOP1MT | 0.973634 | 0.9199 | 0.134337 | 1.60577 |
| DHX15 | 0.973634 | 0.919995 | 0.036241 | 7.855077 |
| PRPS1 | 0.973634 | 0.920027 | 0.109338 | 2.095846 |
| TBC1D20 | 0.973634 | 0.920062 | -0.0416 | 5.013544 |
| MIR1207 | 0.973808 | 0.920301 | -0.10911 | 1.948543 |
| DGKH | 0.9739 | 0.920472 | 0.064604 | 3.452959 |
| LINC01355 | 0.9739 | 0.920537 | 0.054908 | 4.562409 |
| GEMIN6 | 0.974011 | 0.920765 | 0.070716 | 3.239073 |
| TRIM11 | 0.974011 | 0.920849 | -0.03877 | 5.089899 |
| SCLT1 | 0.974011 | 0.920864 | 0.041031 | 5.136314 |
| NUP205 | 0.974055 | 0.92098 | 0.04358 | 7.103065 |
| CD58 | 0.974431 | 0.921606 | 0.063606 | 2.976554 |
| LYRM1 | 0.974431 | 0.92169 | 0.129241 | 1.247951 |
| EAF2 | 0.974431 | 0.921718 | -0.08934 | 2.439561 |
| ABCD4 | 0.974431 | 0.92173 | -0.13465 | 0.992031 |
| FCF1 | 0.974431 | 0.921837 | 0.040431 | 5.548264 |
| ARID3A | 0.974431 | 0.921857 | 0.046982 | 4.8562 |
| TCEA3 | 0.974431 | 0.921928 | -0.04756 | 6.781333 |
| PGBD4 | 0.974431 | 0.922033 | -0.10062 | 2.161856 |
| ZNF782 | 0.974431 | 0.922046 | 0.042505 | 5.044005 |
| ZFP1 | 0.974431 | 0.922078 | -0.04662 | 3.872643 |
| ZHX1-C8or | 0.974527 | 0.922324 | 0.13279 | 1.188096 |
| EBPL | 0.974527 | 0.922464 | -0.11356 | 1.646762 |

| | | | | |
|---|---|---|---|---|
| PSD4 | 0.974527 | 0.922507 | -0.04183 | 6.862981 |
| USP49 | 0.974527 | 0.922526 | -0.04858 | 4.433884 |
| LOC10192 | 0.974527 | 0.92254 | -0.14057 | 1.024548 |
| CREBBP | 0.97498 | 0.923043 | 0.160988 | 0.89541 |
| PIM3 | 0.975759 | 0.923856 | -0.05033 | 6.745134 |
| STIM1 | 0.976102 | 0.924254 | -0.12536 | 1.102727 |
| TMEM161 | 0.976148 | 0.924373 | 0.105186 | 2.274735 |
| GPR84 | 0.976177 | 0.924518 | -0.15179 | 1.104328 |
| FLJ37201 | 0.976177 | 0.924548 | 0.110633 | 1.574603 |
| HIST1H2B | 0.976229 | 0.924672 | 0.051464 | 4.455678 |
| TUBB6 | 0.976474 | 0.92502 | 0.156791 | 0.891391 |
| PSMB1 | 0.976474 | 0.925053 | -0.03766 | 6.141745 |
| TCF7 | 0.976491 | 0.925143 | -0.1169 | 1.127037 |
| ADAMTS1 | 0.976518 | 0.925264 | -0.0791 | 3.084173 |
| DUSP12 | 0.976518 | 0.925318 | -0.05346 | 3.682598 |
| SMARCC1 | 0.976664 | 0.925561 | 0.037392 | 8.49589 |
| NEIL3 | 0.976664 | 0.925605 | -0.09647 | 2.565622 |
| TBCD | 0.977039 | 0.926035 | 0.037121 | 5.970886 |
| KIF15 | 0.977075 | 0.926204 | 0.051662 | 4.646798 |
| GINS2 | 0.977075 | 0.926218 | -0.06616 | 2.433203 |
| SYCE1L | 0.977122 | 0.926337 | 0.125765 | 1.14025 |
| CLUHP3 | 0.977218 | 0.926503 | 0.046252 | 4.633458 |
| MIR26B | 0.977359 | 0.92678 | 0.123459 | 1.935356 |
| SSPO | 0.977359 | 0.926856 | 0.086114 | 2.323172 |
| STAU2 | 0.977359 | 0.926882 | 0.092752 | 1.89106 |
| SYNGAP1 | 0.977359 | 0.927054 | 0.05397 | 3.72097 |
| DKFZP434 | 0.977359 | 0.927117 | 0.138613 | 2.157574 |
| TCEA1 | 0.977359 | 0.927131 | 0.10239 | 1.704812 |
| SNHG5 | 0.977359 | 0.927257 | 0.04367 | 5.236656 |
| FAHD2A | 0.977359 | 0.927287 | 0.063927 | 3.640985 |
| WHAMMP | 0.977359 | 0.927314 | -0.0752 | 2.970921 |
| ZZEF1 | 0.977359 | 0.927468 | -0.03236 | 7.966682 |
| OXNAD1 | 0.977359 | 0.927665 | -0.0356 | 5.352034 |
| TNFAIP8 | 0.977359 | 0.927687 | -0.09 | 2.025313 |
| CBL | 0.977359 | 0.92775 | 0.035517 | 7.037812 |
| LINC01273 | 0.977359 | 0.927771 | 0.168873 | 1.123882 |
| MIR5687 | 0.977359 | 0.927873 | 0.137803 | 0.918456 |
| CRLF1 | 0.977359 | 0.927893 | 0.125605 | 1.741998 |
| NDUFAF3 | 0.977359 | 0.927902 | 0.10384 | 1.771817 |
| GCKR | 0.977559 | 0.928179 | -0.06622 | 3.135526 |
| COX4I2 | 0.977559 | 0.928278 | -0.17668 | 0.496716 |
| LOC10192 | 0.977559 | 0.928316 | 0.086702 | 2.126115 |
| VOPP1 | 0.977678 | 0.928503 | 0.086787 | 1.941248 |
| TAS2R5 | 0.977683 | 0.928583 | -0.11907 | 1.22693 |
| ADAL | 0.977699 | 0.928684 | 0.101511 | 1.72529 |

| | | | | |
|---|---|---|---|---|
| MPLKIP | 0.977699 | 0.928747 | 0.046884 | 4.437351 |
| TUBGCP2 | 0.977812 | 0.928929 | 0.123119 | 1.40127 |
| DNASE1L1 | 0.978088 | 0.929266 | 0.064232 | 3.177051 |
| SNHG4 | 0.978157 | 0.929406 | 0.078175 | 2.655772 |
| STK11 | 0.978165 | 0.929532 | -0.03182 | 6.652491 |
| NAXE | 0.978165 | 0.929563 | -0.039 | 4.698324 |
| NDUFS1 | 0.978286 | 0.929766 | 0.104454 | 1.170876 |
| ELOA2 | 0.978286 | 0.92983 | -0.17541 | 1.023622 |
| ASXL2 | 0.978286 | 0.929901 | 0.031882 | 7.549753 |
| MED31 | 0.978294 | 0.929984 | -0.09828 | 2.040686 |
| ZNHIT2 | 0.978379 | 0.930142 | 0.090213 | 2.532504 |
| SLC5A3 | 0.978379 | 0.930214 | 0.034516 | 6.998232 |
| CNST | 0.978412 | 0.93037 | -0.0389 | 4.805393 |
| VHL | 0.978412 | 0.9305 | -0.0854 | 1.612411 |
| DUSP2 | 0.978412 | 0.930554 | -0.09586 | 3.392789 |
| CENPC | 0.978412 | 0.930566 | -0.03398 | 6.329614 |
| SFTPA2 | 0.978412 | 0.930695 | 0.205142 | 0.667735 |
| LINC01303 | 0.978412 | 0.930735 | 0.071028 | 2.381947 |
| COX15 | 0.978412 | 0.930766 | 0.044665 | 4.167644 |
| HPF1 | 0.978632 | 0.9311 | 0.063823 | 3.342212 |
| NARS | 0.978632 | 0.931155 | -0.03841 | 8.169463 |
| FLT4 | 0.978632 | 0.931275 | 0.108583 | 1.281794 |
| LOC101927 | 0.978632 | 0.931338 | 0.119034 | 1.79597 |
| PTMAP11 | 0.978632 | 0.931349 | -0.04602 | 4.546872 |
| GLIS1 | 0.978632 | 0.931423 | -0.1322 | 1.232825 |
| AGBL3 | 0.978894 | 0.931747 | -0.10114 | 2.402841 |
| GPATCH3 | 0.978905 | 0.931851 | -0.03946 | 4.522715 |
| GNL1 | 0.978905 | 0.932014 | 0.094436 | 2.108531 |
| ZGPAT | 0.978905 | 0.932075 | 0.125675 | 0.790506 |
| MLKL | 0.978905 | 0.932161 | -0.05826 | 3.207443 |
| ASAP1-IT1 | 0.978905 | 0.932201 | 0.076955 | 2.299451 |
| USP40 | 0.978905 | 0.932206 | 0.034632 | 6.943684 |
| ASAP1 | 0.978945 | 0.932318 | -0.12454 | 1.357383 |
| PSMA3-AS1 | 0.979192 | 0.932628 | -0.09113 | 1.824643 |
| CBLC | 0.979331 | 0.932867 | 0.086025 | 1.542924 |
| ACOT11 | 0.979331 | 0.932909 | 0.071875 | 2.774208 |
| TRIP4 | 0.979549 | 0.933208 | -0.03125 | 5.571699 |
| ANAPC2 | 0.979549 | 0.933374 | 0.038324 | 5.001491 |
| ETS2 | 0.979549 | 0.933379 | 0.102695 | 0.992313 |
| DGUOK | 0.979549 | 0.933505 | 0.082622 | 2.262963 |
| LINC00506 | 0.979549 | 0.933532 | 0.151124 | 0.529228 |
| ZNF780A | 0.979549 | 0.933565 | 0.0484 | 3.817933 |
| LRRC61 | 0.97967 | 0.933755 | -0.13862 | 0.777804 |
| LDOC1 | 0.979778 | 0.933983 | 0.05835 | 3.580456 |
| SCAF8 | 0.979778 | 0.934105 | 0.057587 | 2.534188 |

| | | | | |
|---|---|---|---|---|
| REEP5 | 0.979778 | 0.934166 | 0.031561 | 6.860759 |
| KCNJ2-AS1 | 0.979778 | 0.93426 | -0.15421 | 0.839346 |
| LOC10013( | 0.979778 | 0.9343 | 0.062678 | 2.722195 |
| FAM136A | 0.979778 | 0.934306 | 0.03657 | 5.718094 |
| CUL4A | 0.979872 | 0.93447 | -0.1463 | 0.358689 |
| TIPARP-AS: | 0.979878 | 0.93467 | -0.064 | 1.706727 |
| SNORA36C | 0.979878 | 0.934796 | -0.12496 | 1.182697 |
| TSSK3 | 0.979878 | 0.934804 | 0.064535 | 3.077741 |
| RAB11FIP1 | 0.979878 | 0.935063 | 0.036362 | 5.949693 |
| HSP90AA1 | 0.979878 | 0.935077 | -0.15308 | 0.75509 |
| EIF4G2 | 0.979878 | 0.935149 | -0.05038 | 3.057635 |
| HCRTR1 | 0.979878 | 0.935207 | -0.1116 | 1.393285 |
| FAM86C2P | 0.979878 | 0.935261 | 0.068013 | 3.118152 |
| SNORA10 | 0.979878 | 0.935273 | 0.037246 | 6.229411 |
| VGLL4 | 0.979878 | 0.935367 | 0.047675 | 3.663709 |
| MIR1296 | 0.979878 | 0.93544 | -0.08975 | 1.860209 |
| C9orf84 | 0.979878 | 0.93547 | 0.107259 | 1.323121 |
| C1orf194 | 0.979878 | 0.935513 | -0.18248 | 0.657739 |
| SELENOK | 0.979878 | 0.935521 | 0.045046 | 4.769293 |
| LOC101927 | 0.97991 | 0.935627 | 0.07739 | 2.233791 |
| SNORA61 | 0.980014 | 0.9358 | 0.045695 | 7.09415 |
| GTPBP1 | 0.980157 | 0.936051 | 0.030476 | 6.701779 |
| DDB2 | 0.980157 | 0.936154 | -0.04367 | 4.167296 |
| CISD2 | 0.980157 | 0.936225 | -0.05018 | 3.641607 |
| ASAP2 | 0.980157 | 0.936236 | 0.105389 | 1.307023 |
| AFMID | 0.980324 | 0.93647 | -0.09397 | 1.793634 |
| ZNF335 | 0.980379 | 0.936597 | 0.030549 | 5.976381 |
| SMAD4 | 0.980414 | 0.936705 | -0.02963 | 6.702426 |
| ZNF17 | 0.980733 | 0.937085 | -0.03796 | 4.308168 |
| LOC101928 | 0.980845 | 0.937267 | -0.1009 | 1.077488 |
| RING1 | 0.980919 | 0.937412 | -0.0848 | 1.884715 |
| TSGA10 | 0.980948 | 0.937599 | -0.15951 | 0.392851 |
| CAHM | 0.980948 | 0.937655 | -0.108 | 0.519477 |
| CCSAP | 0.980948 | 0.937664 | 0.034953 | 4.93493 |
| BIRC6 | 0.981056 | 0.937842 | -0.02718 | 9.311539 |
| MANCR | 0.981097 | 0.937956 | 0.143896 | 0.627751 |
| OLMALINC | 0.981416 | 0.938363 | -0.06964 | 2.157475 |
| PCNT | 0.981416 | 0.93841 | 0.061031 | 3.286215 |
| ATP5F1C | 0.981433 | 0.938501 | -0.10865 | 1.186663 |
| JMY | 0.981469 | 0.938643 | 0.030385 | 6.634821 |
| ROS1 | 0.981469 | 0.9387 | 0.12059 | 1.127205 |
| C14orf119 | 0.981469 | 0.938761 | -0.06143 | 4.0808 |
| RHOA | 0.981841 | 0.939191 | -0.11567 | 0.986453 |
| ATP1A4 | 0.981858 | 0.939292 | -0.13967 | 1.320386 |
| WDR12 | 0.981858 | 0.939357 | -0.03664 | 4.920492 |

| | | | | |
|---|---|---|---|---|
| GEMIN4 | 0.981863 | 0.939437 | 0.041373 | 4.635635 |
| TAS2R30 | 0.98194 | 0.939586 | 0.177277 | 0.584403 |
| DENND5B-. | 0.982011 | 0.939732 | 0.13818 | 0.471084 |
| OGFRP1 | 0.982011 | 0.939803 | 0.103517 | 1.757466 |
| SNU13 | 0.982079 | 0.939943 | -0.1378 | 0.525894 |
| WAPL | 0.982144 | 0.94008 | 0.029206 | 7.940598 |
| NRF1 | 0.982173 | 0.940183 | -0.1543 | 0.358702 |
| CUL1 | 0.982187 | 0.940296 | -0.0292 | 6.65608 |
| WDR24 | 0.982187 | 0.940352 | 0.040574 | 4.756224 |
| RPS6KA1 | 0.982187 | 0.940426 | 0.088394 | 1.519157 |
| TAOK3 | 0.982187 | 0.940495 | -0.02737 | 7.21304 |
| WDR88 | 0.982291 | 0.940735 | 0.103986 | 1.566252 |
| HOXC4 | 0.982291 | 0.940745 | -0.14698 | 0.372382 |
| PFDN4 | 0.982671 | 0.941263 | 0.040569 | 4.120137 |
| MIR5695 | 0.982671 | 0.941327 | 0.129172 | 0.555671 |
| GNAI2 | 0.982671 | 0.941413 | -0.07011 | 2.011485 |
| B3GNT10 | 0.982671 | 0.941479 | 0.096236 | 1.364669 |
| RAPH1 | 0.982671 | 0.941509 | 0.028724 | 7.081374 |
| ZNF277 | 0.982671 | 0.941646 | 0.033644 | 4.956029 |
| ZNF449 | 0.982671 | 0.941683 | -0.03505 | 4.183905 |
| GMDS | 0.982671 | 0.941709 | 0.06692 | 2.48929 |
| BCL2L11 | 0.982671 | 0.941865 | -0.04734 | 3.358153 |
| C9orf3 | 0.982671 | 0.942002 | -0.06647 | 2.922065 |
| PLEKHJ1 | 0.982671 | 0.942084 | -0.04213 | 3.397765 |
| RPL13AP6 | 0.982671 | 0.942186 | 0.07584 | 1.649466 |
| CRYBB3 | 0.982671 | 0.94236 | -0.06662 | 1.638837 |
| IL1RL1 | 0.982671 | 0.942478 | 0.079618 | 1.364323 |
| WWC2-AS1 | 0.982671 | 0.942693 | 0.105616 | 1.444849 |
| MIR199A1 | 0.982671 | 0.942705 | 0.140057 | 0.361994 |
| LINC00707 | 0.982671 | 0.942727 | -0.12151 | 1.228919 |
| CAPN11 | 0.982671 | 0.942739 | -0.12341 | 0.629504 |
| GJB4 | 0.982671 | 0.94274 | 0.062008 | 2.881386 |
| KLF13 | 0.982671 | 0.942827 | 0.045153 | 4.189091 |
| HIST1H3B | 0.982671 | 0.942832 | -0.03574 | 5.38064 |
| GPAA1 | 0.982671 | 0.942895 | -0.02803 | 5.307389 |
| LCMT1 | 0.982671 | 0.942961 | 0.097195 | 1.423619 |
| MTDH | 0.982671 | 0.943048 | 0.028297 | 8.398439 |
| SPDYE6 | 0.982671 | 0.943088 | 0.039801 | 4.569772 |
| RNF19A | 0.982671 | 0.943124 | -0.04327 | 3.039444 |
| USP17L7 | 0.982671 | 0.943131 | 0.127073 | 0.705926 |
| GPX2 | 0.982765 | 0.943494 | -0.0612 | 2.68901 |
| PMPCB | 0.982765 | 0.943544 | -0.02819 | 5.913899 |
| METTL7A | 0.982765 | 0.943654 | -0.03778 | 6.705076 |
| HEATR5A | 0.982765 | 0.943755 | -0.02828 | 6.280032 |
| C4orf47 | 0.982765 | 0.943781 | -0.10259 | 1.826324 |

| | | | | |
|---|---|---|---|---|
| OXLD1 | 0.982765 | 0.943825 | 0.13015 | 0.510835 |
| PCDHB17P | 0.982765 | 0.943883 | 0.137824 | 0.658017 |
| AMMECR1 | 0.982765 | 0.943907 | 0.100795 | 0.65022 |
| CD44 | 0.982765 | 0.943966 | -0.04449 | 4.274359 |
| EPM2AIP1 | 0.982765 | 0.944086 | 0.032274 | 5.895476 |
| MIR4257 | 0.982765 | 0.944096 | 0.066564 | 2.160897 |
| FARS2 | 0.982765 | 0.944119 | -0.03568 | 4.183198 |
| UBTD1 | 0.982822 | 0.944341 | 0.05319 | 2.959225 |
| MAP3K13 | 0.982822 | 0.944346 | 0.033657 | 4.503473 |
| SNORD15A | 0.982822 | 0.944509 | 0.028458 | 8.01509 |
| ZNF808 | 0.982822 | 0.944601 | -0.02729 | 5.651417 |
| TRG-AS1 | 0.982822 | 0.944604 | -0.08968 | 2.281699 |
| KMT2D | 0.982822 | 0.944709 | -0.02522 | 9.156657 |
| NCL | 0.982822 | 0.944755 | 0.026839 | 10.32757 |
| ZBTB49 | 0.982822 | 0.94487 | 0.044835 | 3.272978 |
| ATP5MC3 | 0.982822 | 0.944942 | 0.077878 | 2.316613 |
| IRF2BPL | 0.982822 | 0.944965 | -0.0328 | 4.999356 |
| MRPL36 | 0.982822 | 0.945006 | -0.03342 | 3.448748 |
| PCSK4 | 0.982822 | 0.945073 | 0.056149 | 2.261581 |
| LOC10024( | 0.982988 | 0.94531 | 0.127113 | 0.998828 |
| CLPTM1 | 0.982988 | 0.945383 | 0.120769 | 0.614609 |
| LYST | 0.983047 | 0.945523 | 0.04462 | 3.403753 |
| LRTOMT | 0.983047 | 0.945623 | -0.09495 | 1.579753 |
| UTP3 | 0.983047 | 0.945736 | -0.0293 | 5.416769 |
| MCPH1 | 0.983047 | 0.945739 | 0.055516 | 2.367876 |
| SMARCC2 | 0.983098 | 0.945922 | 0.060278 | 3.175447 |
| WDR82 | 0.983098 | 0.945938 | -0.02478 | 7.292824 |
| NUP88 | 0.983144 | 0.946225 | 0.027767 | 5.805494 |
| HIST1H4D | 0.983144 | 0.946246 | 0.038248 | 4.045556 |
| TCF25 | 0.983144 | 0.946272 | 0.02514 | 7.585849 |
| KATNA1 | 0.983144 | 0.946365 | 0.066624 | 2.206767 |
| LIMK2 | 0.983144 | 0.946395 | -0.03861 | 3.841967 |
| CDH23 | 0.983144 | 0.946431 | -0.0659 | 1.773667 |
| SLC22A20P | 0.983458 | 0.946809 | 0.07356 | 1.647823 |
| EDF1 | 0.983553 | 0.947085 | 0.048436 | 2.894026 |
| SLC1A3 | 0.983553 | 0.947156 | 0.097005 | 1.124076 |
| DISC1 | 0.983553 | 0.947195 | 0.120184 | 0.56026 |
| BTBD18 | 0.983553 | 0.947241 | -0.04724 | 3.111762 |
| TMEM37 | 0.983553 | 0.947286 | -0.05429 | 2.968995 |
| ZNF513 | 0.983553 | 0.94735 | 0.125468 | 1.09983 |
| DRG1 | 0.983584 | 0.947455 | 0.029196 | 5.201224 |
| INTS4P2 | 0.983789 | 0.947794 | 0.058522 | 3.256564 |
| BIN3 | 0.983789 | 0.947802 | 0.025832 | 5.481444 |
| ZNF626 | 0.983876 | 0.94802 | 0.056733 | 3.026475 |
| RNVU1-19 | 0.983876 | 0.948036 | -0.09848 | 2.062415 |

| | | | | |
|---|---|---|---|---|
| FRS3 | 0.983912 | 0.948469 | 0.057453 | 2.635815 |
| MIR650 | 0.983912 | 0.948551 | 0.094365 | 1.617962 |
| LOC101927 | 0.983912 | 0.948552 | 0.094943 | 1.076209 |
| SHLD2P3 | 0.983912 | 0.948679 | -0.05684 | 2.957865 |
| LRP1 | 0.983912 | 0.948912 | 0.024848 | 9.78417 |
| LINC02478 | 0.983912 | 0.948925 | -0.16832 | 0.444996 |
| DBH-AS1 | 0.983912 | 0.949026 | -0.14018 | 0.928525 |
| OPN1SW | 0.983912 | 0.949192 | 0.142683 | 0.47886 |
| HIST1H3F | 0.983912 | 0.949245 | -0.03892 | 3.809654 |
| SPSB1 | 0.983912 | 0.949279 | 0.028673 | 5.275754 |
| IQSEC2 | 0.983912 | 0.949324 | -0.06172 | 1.829162 |
| TMED3 | 0.983912 | 0.949356 | 0.044783 | 2.937395 |
| IRF2BP1 | 0.983912 | 0.949468 | -0.03379 | 4.262305 |
| REG1A | 0.983912 | 0.949522 | 0.12491 | 6.257943 |
| CIRBP | 0.983912 | 0.949576 | -0.06889 | 1.385513 |
| SNORA2B | 0.983912 | 0.949577 | -0.06394 | 2.674022 |
| GGH | 0.983912 | 0.94959 | -0.04289 | 5.144541 |
| SNORD58A | 0.983912 | 0.949591 | 0.037928 | 6.270982 |
| PCOLCE-AS | 0.983912 | 0.949711 | -0.1313 | 0.65799 |
| C2orf27A | 0.983912 | 0.949734 | -0.12192 | 1.532385 |
| SPTBN4 | 0.983912 | 0.949808 | 0.082451 | 2.188474 |
| RNU5F-1 | 0.983912 | 0.94985 | -0.05297 | 4.753305 |
| TMEM38B | 0.983912 | 0.949852 | 0.049733 | 3.438544 |
| TNC | 0.983912 | 0.94998 | -0.03426 | 8.090384 |
| METTL21A | 0.983912 | 0.949986 | -0.09364 | 1.577682 |
| AQP7P1 | 0.983912 | 0.95002 | -0.03523 | 5.764983 |
| LONRF1 | 0.984071 | 0.950272 | -0.02826 | 5.541728 |
| SLC25A26 | 0.984071 | 0.950324 | 0.135963 | 0.636642 |
| CHD8 | 0.984128 | 0.950528 | 0.035799 | 4.187672 |
| CD3G | 0.984128 | 0.950657 | 0.07584 | 2.27615 |
| TMEM179B | 0.984128 | 0.950702 | -0.03209 | 4.128711 |
| ABCB10 | 0.984128 | 0.950725 | 0.025808 | 4.87175 |
| LINGO3 | 0.984128 | 0.950778 | 0.120354 | 1.067659 |
| CYHR1 | 0.984128 | 0.950857 | 0.033398 | 4.037693 |
| RRH | 0.984128 | 0.951107 | -0.15229 | 0.550826 |
| CNIH3 | 0.984128 | 0.951138 | -0.08647 | 1.212111 |
| BMS1P1 | 0.984128 | 0.951152 | -0.02527 | 5.700026 |
| C9orf41-AS | 0.984128 | 0.951203 | -0.03522 | 3.11271 |
| GTSE1-DT | 0.984128 | 0.951203 | 0.137411 | 0.419046 |
| ZNF24 | 0.984422 | 0.951563 | -0.02602 | 6.721796 |
| LMBRD1 | 0.984701 | 0.951946 | 0.024615 | 5.944622 |
| CCM2 | 0.984701 | 0.95204 | -0.12717 | 0.637956 |
| SFTPD | 0.984701 | 0.952126 | 0.095468 | 0.708479 |
| MIR22HG | 0.984701 | 0.952173 | -0.05418 | 3.171477 |
| SNX29 | 0.984701 | 0.952207 | -0.02562 | 6.662505 |

| | | | | |
|---|---|---|---|---|
| FGD4 | 0.984753 | 0.952366 | 0.057834 | 2.554332 |
| LEF1 | 0.984753 | 0.952408 | 0.111521 | 0.599515 |
| ZNF800 | 0.984866 | 0.95281 | 0.023451 | 6.242817 |
| CARS2 | 0.984866 | 0.952875 | -0.02444 | 5.360854 |
| C19orf24 | 0.984866 | 0.95302 | -0.03548 | 3.379022 |
| NKIRAS1 | 0.984866 | 0.953054 | -0.03861 | 3.823252 |
| NACC1 | 0.984866 | 0.953117 | -0.02551 | 6.265968 |
| DNAJC2 | 0.984866 | 0.953129 | 0.06638 | 1.711838 |
| ETV3 | 0.984866 | 0.953183 | -0.0274 | 4.765408 |
| NOC4L | 0.984866 | 0.953189 | 0.039971 | 3.659398 |
| SNX19 | 0.984866 | 0.953193 | -0.02959 | 5.179904 |
| DDB1 | 0.984943 | 0.953342 | -0.02174 | 7.932079 |
| ZC2HC1A | 0.985145 | 0.953613 | -0.0273 | 5.017054 |
| SLC38A2 | 0.985321 | 0.953859 | 0.054125 | 2.263234 |
| CD69 | 0.985532 | 0.954219 | 0.062563 | 2.559069 |
| TTC39C | 0.985532 | 0.954227 | -0.05498 | 2.83046 |
| MOXD1 | 0.985532 | 0.954289 | 0.037612 | 5.220861 |
| DPH3P1 | 0.985574 | 0.954416 | 0.080994 | 1.723044 |
| RAB6D | 0.985574 | 0.954479 | 0.081412 | 1.295612 |
| C12orf66 | 0.985626 | 0.954605 | -0.07472 | 1.412465 |
| UTP4 | 0.985761 | 0.95481 | 0.028381 | 4.182044 |
| FAAH2 | 0.985842 | 0.955088 | 0.03057 | 3.790609 |
| RNF139 | 0.985842 | 0.95509 | 0.02149 | 5.186731 |
| NDUFA11 | 0.985842 | 0.955114 | 0.069592 | 1.913131 |
| MRPL18 | 0.986099 | 0.955447 | 0.024445 | 5.263045 |
| AGO3 | 0.986099 | 0.955513 | 0.114647 | 0.764967 |
| DYNLRB1 | 0.986122 | 0.955611 | 0.100383 | 0.646257 |
| EXOSC1 | 0.986226 | 0.955843 | 0.043378 | 3.750603 |
| PPP1R37 | 0.986226 | 0.955862 | 0.024554 | 5.301488 |
| AVPI1 | 0.986357 | 0.956064 | -0.0555 | 4.396878 |
| CIC | 0.986368 | 0.95615 | 0.021169 | 5.627882 |
| ISOC1 | 0.986469 | 0.956473 | 0.034703 | 4.449361 |
| CNOT11 | 0.986469 | 0.95648 | -0.02013 | 6.479354 |
| ADRA1D | 0.986469 | 0.956569 | 0.072432 | 0.737585 |
| ATP6AP1L | 0.986469 | 0.956595 | 0.037182 | 4.049153 |
| C19orf54 | 0.986469 | 0.95684 | -0.02608 | 4.188067 |
| TMCC2 | 0.986469 | 0.956925 | 0.100887 | 0.789098 |
| C4orf36 | 0.986469 | 0.956927 | -0.1259 | 0.77379 |
| TMEM61 | 0.986469 | 0.956972 | -0.09185 | 0.703869 |
| GNB4 | 0.986469 | 0.956975 | 0.024946 | 6.083056 |
| MCF2L-AS1 | 0.986469 | 0.957046 | -0.07766 | 1.282418 |
| ATL3 | 0.986469 | 0.957075 | 0.090103 | 0.801518 |
| TGFA-IT1 | 0.986689 | 0.957523 | -0.10864 | 0.876416 |
| CYP2A6 | 0.986689 | 0.957577 | 0.091213 | 0.864992 |
| HIST1H2AL | 0.986689 | 0.957586 | 0.038511 | 3.950561 |

| | | | | |
|---|---|---|---|---|
| GPAT2 | 0.986689 | 0.957651 | 0.066133 | 2.444144 |
| MTMR9 | 0.986689 | 0.95769 | 0.02085 | 5.453141 |
| LOC101927 | 0.986689 | 0.957739 | 0.075286 | 1.455088 |
| KIAA2013 | 0.987162 | 0.958346 | -0.02101 | 5.241242 |
| SYF2 | 0.987162 | 0.958349 | 0.052748 | 1.891138 |
| STEAP3-AS | 0.987226 | 0.958486 | -0.05434 | 2.190785 |
| TNFRSF10A | 0.987285 | 0.958645 | 0.029745 | 4.828175 |
| SNORD142 | 0.987285 | 0.958765 | -0.06924 | 1.5147 |
| MED22 | 0.987285 | 0.958776 | 0.025248 | 4.326054 |
| LOC105747 | 0.987285 | 0.95899 | -0.04549 | 3.076779 |
| LOC101929 | 0.987285 | 0.959058 | 0.061185 | 0.747271 |
| SLC41A1 | 0.987285 | 0.959096 | -0.02427 | 5.767392 |
| MYORG | 0.987285 | 0.959115 | -0.02425 | 5.431875 |
| RNF113A | 0.987285 | 0.959161 | 0.026567 | 4.08995 |
| EGR3 | 0.987285 | 0.959221 | -0.08934 | 1.262681 |
| FNTB | 0.987378 | 0.959409 | 0.084162 | 0.801347 |
| ATP6V1FNI | 0.987378 | 0.959462 | -0.0438 | 3.446389 |
| FCGR1CP | 0.987444 | 0.959601 | -0.11136 | 0.680594 |
| MXRA8 | 0.987473 | 0.959725 | 0.102257 | 0.66511 |
| CCDC183-A | 0.987473 | 0.95978 | 0.047995 | 2.736852 |
| CH17-340N | 0.987706 | 0.960082 | 0.089585 | 1.02075 |
| TMEM42 | 0.987795 | 0.960385 | -0.04985 | 2.299662 |
| CLEC18C | 0.987795 | 0.960394 | -0.11763 | 0.758253 |
| ADAMTSL4 | 0.987795 | 0.960394 | -0.03311 | 4.351954 |
| CASP16P | 0.987994 | 0.960663 | 0.078134 | 1.526378 |
| ST3GAL4 | 0.988052 | 0.960878 | 0.093508 | 0.869415 |
| MMP24 | 0.988052 | 0.960934 | -0.02461 | 4.226467 |
| ESRRA | 0.988052 | 0.961015 | 0.044808 | 2.825841 |
| OPA1-AS1 | 0.988052 | 0.961074 | 0.054852 | 1.641522 |
| SNHG11 | 0.988052 | 0.961113 | -0.06164 | 2.060508 |
| MAFG-DT | 0.988052 | 0.961171 | -0.06118 | 2.051959 |
| SCO1 | 0.988091 | 0.961284 | -0.0205 | 5.361269 |
| SNORD116 | 0.988715 | 0.962005 | -0.06834 | 5.136087 |
| NR4A3 | 0.988715 | 0.962042 | -0.10318 | 1.779257 |
| SS18L2 | 0.988849 | 0.962248 | 0.041534 | 2.93557 |
| S100P | 0.988983 | 0.962453 | 0.029835 | 5.638624 |
| KCNJ2 | 0.989002 | 0.962547 | -0.02493 | 3.969044 |
| SETD2 | 0.989008 | 0.962671 | 0.017141 | 8.647283 |
| EIF2S3 | 0.989008 | 0.962734 | 0.018848 | 8.304553 |
| RBM44 | 0.989008 | 0.962779 | 0.033957 | 3.268019 |
| FOXK2 | 0.989374 | 0.963338 | -0.01697 | 6.402677 |
| VMAC | 0.989374 | 0.963351 | 0.029419 | 3.427967 |
| FGF14-IT1 | 0.989374 | 0.963361 | -0.09495 | 1.350879 |
| POLR2H | 0.989449 | 0.96351 | -0.07081 | 1.232987 |
| SNAPC1 | 0.989501 | 0.963678 | -0.02504 | 4.997792 |

| | | | | |
|---|---|---|---|---|
| LARGE-AS1 | 0.989501 | 0.963747 | 0.048955 | 2.016736 |
| RAB36 | 0.989501 | 0.963787 | 0.037567 | 3.135484 |
| TPI1P3 | 0.989617 | 0.964212 | 0.030742 | 3.177575 |
| EIF2AK3 | 0.989617 | 0.96422 | 0.034231 | 3.687714 |
| ZNF93 | 0.989617 | 0.96431 | 0.038348 | 3.01464 |
| RIBC2 | 0.989617 | 0.964324 | -0.05936 | 1.759754 |
| ANGPTL3 | 0.989617 | 0.964344 | 0.043251 | 2.816015 |
| LOC10272 | 0.989617 | 0.964353 | -0.09641 | 1.28898 |
| SULT1E1 | 0.989709 | 0.964517 | 0.041743 | 2.426388 |
| UBE2Q1-A | 0.989746 | 0.964679 | -0.07057 | 1.61683 |
| SLC7A5P1 | 0.989746 | 0.964768 | 0.07549 | 1.580537 |
| PIGH | 0.989746 | 0.96478 | 0.046113 | 2.50368 |
| UBE2L3 | 0.990108 | 0.965213 | 0.052459 | 2.381992 |
| ZNF737 | 0.990108 | 0.965284 | 0.024531 | 4.364447 |
| ZNF35 | 0.990166 | 0.965415 | 0.027377 | 3.868296 |
| CLDN4 | 0.990182 | 0.965523 | 0.024268 | 7.588501 |
| WDR13 | 0.990182 | 0.965582 | -0.028 | 2.759096 |
| ARGLU1 | 0.990228 | 0.965703 | 0.017808 | 8.275423 |
| TMEM132 | 0.990306 | 0.965878 | 0.089632 | 1.393536 |
| FAN1 | 0.990306 | 0.965948 | 0.056208 | 2.153574 |
| SNORD70 | 0.990306 | 0.966005 | 0.036009 | 2.767811 |
| CHD3 | 0.990483 | 0.966315 | 0.046822 | 2.842295 |
| SLC35A5 | 0.990483 | 0.966328 | -0.01816 | 4.615493 |
| KIAA1324 | 0.990594 | 0.966623 | -0.12699 | 0.450365 |
| SLC8A2 | 0.990594 | 0.966647 | -0.09521 | 1.183477 |
| HPRT1 | 0.990594 | 0.966878 | 0.01851 | 5.330841 |
| ZNF77 | 0.990594 | 0.966907 | -0.03555 | 2.868703 |
| MYO1D | 0.990594 | 0.96692 | 0.015623 | 6.421915 |
| MIER2 | 0.990594 | 0.967088 | -0.01973 | 5.276792 |
| HIST1H2BJ | 0.990594 | 0.967099 | -0.02721 | 4.771169 |
| VPS16 | 0.990594 | 0.967183 | 0.039591 | 2.310538 |
| TMEM39A | 0.990594 | 0.967262 | -0.08279 | 0.404213 |
| SPECC1L | 0.990594 | 0.967284 | -0.05023 | 1.907147 |
| CAPN10 | 0.990594 | 0.967307 | 0.064949 | 1.493462 |
| BCL9L | 0.990594 | 0.967343 | -0.0157 | 8.099044 |
| C19orf53 | 0.990708 | 0.967536 | 0.016413 | 5.731787 |
| DOCK6 | 0.990708 | 0.967605 | 0.01553 | 6.976322 |
| AKR7A3 | 0.990773 | 0.967744 | 0.043903 | 4.281722 |
| NDUFAF7 | 0.990887 | 0.967931 | 0.06716 | 1.092838 |
| CHCHD2 | 0.991118 | 0.968299 | -0.01602 | 6.791602 |
| EXOSC4 | 0.991118 | 0.968345 | -0.02941 | 3.280955 |
| ATP6AP2 | 0.991118 | 0.968439 | 0.014666 | 6.366711 |
| CHMP2A | 0.991118 | 0.968458 | 0.05782 | 0.462099 |
| ZNF674-AS | 0.991192 | 0.968658 | 0.034981 | 2.338673 |
| ODC1 | 0.991192 | 0.968692 | -0.07205 | 2.166195 |

| | | | | |
|---|---|---|---|---|
| ZC3H11A | 0.991192 | 0.968773 | 0.014727 | 8.574305 |
| RPAP2 | 0.991192 | 0.968833 | -0.01614 | 5.479738 |
| TMED8 | 0.991235 | 0.96895 | -0.02788 | 3.56575 |
| CCDC61 | 0.991308 | 0.969097 | -0.02453 | 3.75942 |
| CIDECP1 | 0.99181 | 0.969682 | 0.045917 | 1.746195 |
| PRPF40A | 0.99181 | 0.969824 | 0.013867 | 8.424832 |
| LRRC8B | 0.99181 | 0.969848 | -0.04254 | 2.264382 |
| STMN1 | 0.99181 | 0.969891 | 0.110518 | 0.329189 |
| RNF126 | 0.991811 | 0.970005 | -0.0175 | 5.399971 |
| ABCC12 | 0.991811 | 0.970043 | 0.074719 | 1.176148 |
| CHST3 | 0.992016 | 0.97032 | 0.017063 | 5.815207 |
| NET1 | 0.992016 | 0.970395 | 0.052477 | 2.040833 |
| LOC10272З | 0.992059 | 0.970512 | -0.09313 | 1.032737 |
| CDK9 | 0.992106 | 0.970706 | 0.01378 | 5.954409 |
| SH3D19 | 0.992106 | 0.970866 | 0.058661 | 1.483901 |
| RFX7 | 0.992106 | 0.970873 | -0.01634 | 6.656614 |
| MYO3A | 0.992106 | 0.970897 | -0.04108 | 2.202473 |
| CCDC7 | 0.992106 | 0.970936 | -0.02504 | 4.411859 |
| NOV | 0.992115 | 0.971029 | -0.02886 | 2.826648 |
| KIAA1656 | 0.992115 | 0.971107 | -0.03072 | 2.570962 |
| CCNE1 | 0.992115 | 0.971172 | 0.027527 | 2.995877 |
| LRRC47 | 0.992147 | 0.971297 | -0.01419 | 5.847612 |
| RNF166 | 0.992147 | 0.971355 | 0.038881 | 1.536606 |
| PTGER4P2- | 0.992276 | 0.971556 | 0.100519 | 0.588922 |
| GS1-124K5 | 0.992466 | 0.97186 | 0.023983 | 3.611692 |
| MST1 | 0.992466 | 0.97193 | -0.02111 | 3.780749 |
| ZBTB24 | 0.992466 | 0.972111 | -0.02181 | 3.988949 |
| POLR2J2 | 0.992466 | 0.972125 | -0.0907 | 0.49208 |
| SH3GL2 | 0.992466 | 0.972179 | 0.07913 | 1.372932 |
| POU5F1P4 | 0.992466 | 0.972206 | 0.062598 | 1.537646 |
| ELP6 | 0.992466 | 0.972319 | 0.026456 | 3.635179 |
| ACVR1 | 0.992466 | 0.972347 | -0.09863 | 0.55624 |
| C10orf88 | 0.992546 | 0.972501 | 0.030188 | 3.168717 |
| ACIN1 | 0.992559 | 0.972711 | 0.028175 | 3.493818 |
| ZFYVE21 | 0.992559 | 0.972758 | -0.10441 | 0.422642 |
| ATXN7L1 | 0.992559 | 0.972812 | -0.02873 | 2.911503 |
| ARR3 | 0.992559 | 0.972816 | -0.03588 | 2.331909 |
| CCDC18 | 0.992662 | 0.972993 | -0.07258 | 1.347658 |
| BLOC1S6 | 0.992809 | 0.973248 | 0.097582 | 0.516955 |
| MIR2355 | 0.992809 | 0.973289 | -0.1398 | 0.397149 |
| SNORA19 | 0.992812 | 0.973566 | 0.025181 | 3.536165 |
| EXOC3-AS1 | 0.992812 | 0.973609 | -0.06748 | 0.533252 |
| TARS | 0.992812 | 0.973629 | -0.06306 | 0.580692 |
| VPS26C | 0.992812 | 0.973659 | 0.012467 | 5.604307 |
| LINC00842 | 0.992812 | 0.97367 | -0.04955 | 1.876834 |

| | | | | |
|---|---|---|---|---|
| AKR1C2 | 0.993478 | 0.974399 | -0.06279 | 1.686865 |
| ACP4 | 0.99357 | 0.974565 | -0.05626 | 1.664829 |
| USP34 | 0.993649 | 0.974718 | 0.011353 | 9.052095 |
| LOC101593 | 0.9937 | 0.974844 | -0.10695 | 0.486167 |
| LOC441666 | 0.993738 | 0.974983 | -0.03571 | 2.440077 |
| SNORD127 | 0.993738 | 0.975032 | 0.040815 | 1.689818 |
| WDR60 | 0.993745 | 0.975116 | 0.012034 | 6.800329 |
| CAPRIN1 | 0.993782 | 0.975227 | 0.012518 | 6.419844 |
| COQ5 | 0.993923 | 0.975441 | -0.01523 | 4.438992 |
| SDF2 | 0.993926 | 0.97552 | 0.070993 | 0.818603 |
| LRRC37A | 0.994213 | 0.975877 | -0.04489 | 0.95282 |
| CA3 | 0.994527 | 0.976262 | -0.03219 | 1.984878 |
| SPRY1 | 0.994665 | 0.976555 | 0.048069 | 1.816742 |
| SMARCE1 | 0.994665 | 0.976574 | -0.01064 | 7.193404 |
| MAFG | 0.994665 | 0.97668 | -0.06496 | 0.785705 |
| GRTP1-AS1 | 0.994665 | 0.97678 | 0.050533 | 1.819028 |
| LOC155060 | 0.994665 | 0.976854 | -0.01952 | 3.877807 |
| RNF144A | 0.994665 | 0.976945 | -0.01498 | 4.832162 |
| JHY | 0.994665 | 0.976972 | 0.037402 | 2.801271 |
| RPS9 | 0.994665 | 0.977011 | -0.02018 | 3.518812 |
| TJAP1 | 0.994665 | 0.97715 | 0.081806 | 0.735843 |
| RASL11B | 0.994665 | 0.977216 | 0.068747 | 0.674462 |
| SLC38A5 | 0.994665 | 0.97723 | 0.026324 | 3.304291 |
| PAK2 | 0.994971 | 0.977628 | 0.010777 | 7.920225 |
| P4HTM | 0.994971 | 0.977683 | 0.090141 | 0.744638 |
| CTCF | 0.995015 | 0.977806 | -0.01239 | 4.891383 |
| SNHG12 | 0.995015 | 0.977878 | -0.01513 | 4.307779 |
| MIR548K | 0.995072 | 0.978079 | -0.11084 | 0.477502 |
| OPA3 | 0.995072 | 0.978086 | 0.010903 | 5.410042 |
| PIPSL | 0.995142 | 0.978241 | 0.030943 | 2.043284 |
| GLI4 | 0.995142 | 0.978334 | -0.01698 | 3.594691 |
| LOC105447 | 0.995142 | 0.978386 | -0.05354 | 0.731635 |
| ERN1 | 0.995142 | 0.978515 | -0.01181 | 5.827159 |
| HIP1 | 0.995142 | 0.978533 | -0.0571 | 0.887767 |
| C1orf195 | 0.995352 | 0.978816 | 0.021314 | 2.714986 |
| GLI3 | 0.995524 | 0.979142 | -0.01562 | 5.014241 |
| FAM156A | 0.995524 | 0.979211 | -0.07252 | 1.197481 |
| ITGA2B | 0.995524 | 0.979218 | 0.042793 | 1.141766 |
| DCAKD | 0.995524 | 0.9793 | -0.06463 | 0.898076 |
| C19orf18 | 0.995524 | 0.979453 | 0.082859 | 0.725578 |
| ABHD13 | 0.995524 | 0.979456 | -0.01135 | 5.719579 |
| CDC42SE2 | 0.995524 | 0.979608 | 0.03854 | 0.9276 |
| DSE | 0.995524 | 0.979701 | 0.033063 | 1.141062 |
| TLX2 | 0.995524 | 0.979707 | 0.026094 | 1.003885 |
| GSN | 0.995524 | 0.979743 | 0.025028 | 2.175061 |

| | | | | |
|---|---|---|---|---|
| TMEM167A | 0.995683 | 0.979976 | -0.0096 | 7.244218 |
| POP7 | 0.995794 | 0.980162 | 0.025633 | 3.385147 |
| HMGN2P46 | 0.995992 | 0.980432 | 0.077772 | 1.052713 |
| MST1P2 | 0.996001 | 0.98056 | -0.01806 | 3.229863 |
| HYDIN | 0.996001 | 0.980609 | -0.04207 | 1.652091 |
| CD300LB | 0.996001 | 0.981027 | -0.11842 | 0.570742 |
| LSP1 | 0.996001 | 0.981109 | 0.029854 | 2.55057 |
| CCDC86 | 0.996001 | 0.98116 | 0.016294 | 4.354585 |
| OGN | 0.996001 | 0.981237 | 0.074936 | 0.893086 |
| ADAT2 | 0.996001 | 0.981247 | 0.026432 | 3.322926 |
| MSL3 | 0.996001 | 0.981267 | -0.01679 | 3.467229 |
| UTP11 | 0.996001 | 0.981288 | 0.011084 | 5.541991 |
| GJA4 | 0.996001 | 0.981325 | -0.04482 | 1.826327 |
| PHLDA3 | 0.996001 | 0.981347 | -0.01462 | 2.787013 |
| SNRK-AS1 | 0.996001 | 0.981352 | 0.023666 | 3.011236 |
| MAP1LC3B | 0.996166 | 0.981659 | 0.011117 | 6.072453 |
| PCID2 | 0.996166 | 0.981666 | -0.06469 | 1.016724 |
| LINC02608 | 0.996348 | 0.981963 | -0.05647 | 0.741114 |
| SFXN5 | 0.996348 | 0.982002 | -0.01339 | 4.240074 |
| TAS2R43 | 0.996348 | 0.982097 | -0.06542 | 0.707602 |
| LINC00184 | 0.996348 | 0.982225 | 0.080209 | 0.72022 |
| FZD1 | 0.996348 | 0.982375 | 0.011195 | 5.568442 |
| SIM2 | 0.996348 | 0.982394 | 0.01646 | 4.864887 |
| NHS-AS1 | 0.996348 | 0.982444 | 0.070825 | 0.882063 |
| LOC101928 | 0.996348 | 0.98253 | 0.057618 | 0.890622 |
| LINC01270 | 0.996348 | 0.982587 | -0.05725 | 0.954333 |
| RNF170 | 0.996348 | 0.982645 | 0.047076 | 1.133479 |
| FAM122B | 0.996348 | 0.982681 | 0.016346 | 3.573985 |
| CCDC106 | 0.996371 | 0.982779 | -0.02004 | 2.779208 |
| B3GAT3 | 0.996444 | 0.982927 | -0.04362 | 1.229454 |
| SERPIND1 | 0.996599 | 0.983156 | 0.017434 | 3.149863 |
| YIPF3 | 0.996616 | 0.983258 | -0.00813 | 6.181 |
| PTMS | 0.996616 | 0.983324 | 0.008618 | 7.840735 |
| SHANK1 | 0.996656 | 0.98344 | 0.062246 | 1.116593 |
| UNKL | 0.99668 | 0.983574 | 0.017871 | 3.438318 |
| AHCYL2 | 0.99668 | 0.983634 | 0.036151 | 1.437803 |
| PAPOLA | 0.99668 | 0.983702 | 0.025835 | 2.647113 |
| SND1 | 0.99668 | 0.983768 | -0.00826 | 7.822346 |
| KLRB1 | 0.996744 | 0.983913 | -0.04489 | 1.176102 |
| SBDS | 0.996744 | 0.983983 | -0.00803 | 7.018391 |
| ANKRD13C | 0.996752 | 0.984109 | -0.00801 | 5.958019 |
| NFKBIB | 0.996752 | 0.984241 | -0.01309 | 3.656317 |
| TTC7A | 0.996752 | 0.984318 | -0.0462 | 1.218105 |
| SMCO1 | 0.996752 | 0.984332 | 0.047556 | 0.709427 |
| TRIL | 0.996752 | 0.984371 | 0.018249 | 3.324839 |

| | | | | |
|---|---|---|---|---|
| FIGNL1 | 0.996928 | 0.984671 | -0.01574 | 3.205061 |
| TSEN2 | 0.996928 | 0.984696 | -0.03022 | 2.253011 |
| ATXN1 | 0.997104 | 0.984946 | 0.057979 | 1.180755 |
| LYRM4 | 0.997141 | 0.985128 | 0.03706 | 1.438076 |
| UQCC2 | 0.997141 | 0.985135 | -0.00891 | 4.804436 |
| ANO9 | 0.997223 | 0.985306 | 0.010131 | 5.122514 |
| SEC14L1 | 0.997223 | 0.985455 | 0.02591 | 2.77347 |
| FBXO10 | 0.997223 | 0.985511 | -0.0118 | 3.960953 |
| PBLD | 0.997223 | 0.985569 | -0.01454 | 3.230914 |
| SNORD12B | 0.997223 | 0.985596 | 0.009915 | 6.709491 |
| EFHC2 | 0.997244 | 0.985692 | -0.0381 | 1.723283 |
| DDX46 | 0.997541 | 0.986062 | 0.033634 | 1.5423 |
| GRID1 | 0.997722 | 0.986317 | 0.03956 | 1.412992 |
| SMO | 0.997759 | 0.98643 | 0.011285 | 4.123755 |
| SNORD58C | 0.997849 | 0.986629 | -0.03442 | 1.332356 |
| ZNF90 | 0.997849 | 0.986671 | 0.025475 | 2.151003 |
| GRB10 | 0.997975 | 0.986879 | 0.045444 | 1.51529 |
| CALML5 | 0.997975 | 0.986948 | 0.024832 | 3.983969 |
| RAG1 | 0.997989 | 0.987037 | -0.04032 | 1.610381 |
| SNORA38 | 0.997993 | 0.987117 | -0.0361 | 2.455858 |
| RPS19 | 0.99812 | 0.98736 | -0.00642 | 8.387164 |
| UBTF | 0.99812 | 0.987415 | -0.04739 | 1.743564 |
| SCARNA20 | 0.99812 | 0.987471 | -0.01372 | 3.29845 |
| PHPT1 | 0.998173 | 0.9876 | 0.043489 | 1.873904 |
| MRPL35 | 0.998183 | 0.987698 | -0.01245 | 3.855912 |
| PYGO2 | 0.998183 | 0.987761 | 0.008557 | 5.145771 |
| SRRM2 | 0.998313 | 0.987966 | -0.00573 | 11.37023 |
| NME9 | 0.99861 | 0.988336 | 0.009072 | 4.963151 |
| CPSF6 | 0.998641 | 0.988472 | -0.04975 | 1.641668 |
| ACAP1 | 0.998641 | 0.988553 | 0.009636 | 4.299521 |
| CXorf40B | 0.998641 | 0.988595 | 0.010872 | 3.653597 |
| UCN2 | 0.999045 | 0.989071 | -0.03019 | 1.792143 |
| PVT1 | 0.999232 | 0.989333 | -0.00904 | 4.750821 |
| TMA16 | 0.999302 | 0.989478 | 0.006819 | 4.713356 |
| PTDSS2 | 0.999399 | 0.98965 | -0.01588 | 2.447532 |
| CDC40 | 0.999604 | 0.98993 | -0.00567 | 5.865487 |
| SOX10 | 0.999756 | 0.990156 | -0.02106 | 1.887101 |
| NIFK | 0.999828 | 0.990327 | -0.00705 | 5.385294 |
| NCOA6 | 0.999828 | 0.99038 | 0.005313 | 6.402664 |
| MAF1 | 0.999887 | 0.990537 | -0.00607 | 5.533281 |
| RMND1 | 0.999887 | 0.990591 | 0.018745 | 2.140699 |
| SNORA27 | 0.999889 | 0.990669 | 0.005502 | 6.173346 |
| BOLA1 | 1 | 0.99096 | 0.015809 | 2.307113 |
| SNCA-AS1 | 1 | 0.991061 | -0.06608 | 1.128119 |
| POMT2 | 1 | 0.991232 | 0.00855 | 4.159958 |

| | | | | |
|---|---|---|---|---|
| LINC00662 | 1 | 0.991469 | 0.020149 | 2.293768 |
| EIF1AX | 1 | 0.99159 | -0.0057 | 6.154061 |
| NUTM2G | 1 | 0.992067 | -0.00872 | 4.195989 |
| TPPP | 1 | 0.992276 | -0.00512 | 5.836225 |
| GGPS1 | 1 | 0.9926 | 0.013984 | 3.008028 |
| ZBTB6 | 1 | 0.993414 | 0.004553 | 4.828736 |
| PRKCZ | 1 | 0.99391 | -0.00964 | 3.338207 |
| KIFC1 | 1 | 0.994451 | 0.010701 | 2.993878 |
| OFD1 | 1 | 0.994962 | -0.00252 | 6.93446 |
| APOBEC3G | 1 | 0.995398 | -0.00642 | 3.520631 |
| BTBD19 | 1 | 0.995849 | 0.006672 | 4.061111 |
| DNAH11 | 1 | 0.996026 | -0.00631 | 4.749212 |
| SMG8 | 1 | 0.996112 | 0.003296 | 4.400643 |
| SMU1 | 1 | 0.996181 | 0.002252 | 6.720073 |
| LDLRAP1 | 1 | 0.996485 | -0.00256 | 5.150155 |
| RNF43 | 1 | 0.996556 | 0.008579 | 3.44121 |
| COL28A1 | 1 | 0.996627 | -0.00599 | 4.80198 |
| VTI1A | 1 | 0.996924 | 0.002035 | 6.412016 |
| DDX55 | 1 | 0.997246 | -0.00133 | 5.520352 |
| KDM5A | 1 | 0.99819 | -0.00097 | 8.199634 |
| ATP8B1 | 1 | 0.998689 | 0.001048 | 7.668373 |
| CDPF1 | 1 | 1 | -0.01406 | 1.869315 |
| CTU1 | 1 | 1 | -0.0077 | 2.365409 |
| ZNF37A | 1 | 1 | -0.03013 | 1.101613 |
| TFAP2A-AS | 1 | 1 | -0.02824 | 1.047452 |
| SNORD30 | 1 | 1 | -0.05005 | 0.899306 |
| SLC17A7 | 1 | 1 | 0.038892 | 0.496404 |
| NFRKB | 1 | 1 | -0.00288 | 1.762185 |
| LOC100506 | 1 | 1 | 0.026097 | 1.413461 |
| HAVCR1P1 | 1 | 1 | 0.007356 | 0.92569 |
| LIN7A | 1 | 1 | 0.040111 | 1.045488 |
| C18orf61 | 1 | 1 | -0.07106 | 0.586123 |
| CELP | 1 | 1 | 0.017201 | 0.810886 |
| LOC285847 | 1 | 1 | 0.053304 | 0.954093 |
| PPP4R2 | 1 | 1 | -0.04671 | 1.095839 |
| RNF4 | 1 | 1 | 0.025254 | 1.514926 |
| ZNF100 | 1 | 1 | -0.03585 | 0.59495 |
| P2RX5-TAX | 1 | 1 | -0.02592 | 1.495215 |
| OBSL1 | 1 | 1 | 0.017288 | 1.97333 |
| CTAGE15 | 1 | 1 | -0.01511 | 1.163729 |
| NDST3 | 1 | 1 | -0.03322 | 0.999821 |
| CHKB-DT | 1 | 1 | -0.02345 | 1.257182 |
| KCNG2 | 1 | 1 | -0.03045 | 0.513315 |
| NF2 | 1 | 1 | 0.078014 | 0.382524 |
| PLEKHD1 | 1 | 1 | 0.068741 | 0.483411 |

| | | | | |
|---|---|---|---|---|
| LRIG2 | 1 | 1 | -0.05566 | 0.45867 |
| APOLD1 | 1 | 1 | 0.049518 | 0.427973 |
| ATP5MC2 | 1 | 1 | -0.04946 | 0.671179 |
| SYCE2 | 1 | 1 | -0.04875 | 0.84694 |
| TNFAIP8L3 | 1 | 1 | 0.043074 | 0.783646 |
| CCDC13 | 1 | 1 | 0.042651 | 0.877924 |
| TP53I3 | 1 | 1 | 0.041879 | 1.134058 |
| CEP97 | 1 | 1 | 0.041718 | 0.399817 |
| OXT | 1 | 1 | 0.039948 | 0.719473 |
| CCNH | 1 | 1 | -0.03858 | 0.45082 |
| DDX11-AS1 | 1 | 1 | -0.03705 | 0.423086 |
| SLC25A21-/ | 1 | 1 | -0.03425 | 0.564425 |
| RAB40A | 1 | 1 | 0.03385 | 1.121613 |
| PID1 | 1 | 1 | -0.03137 | 0.787514 |
| LETMD1 | 1 | 1 | 0.030793 | 0.402947 |
| SAMD12-A | 1 | 1 | -0.03044 | 0.368778 |
| ABCA4 | 1 | 1 | -0.02973 | 0.95818 |
| LOC100131 | 1 | 1 | -0.02909 | 1.551641 |
| SNORD81 | 1 | 1 | -0.02732 | 1.688359 |
| CXXC5 | 1 | 1 | -0.02727 | 1.788788 |
| RGS17 | 1 | 1 | 0.025659 | 1.257065 |
| LOC644656 | 1 | 1 | 0.024528 | 1.251975 |
| SLC35G6 | 1 | 1 | 0.021059 | 0.946144 |
| ACSM5 | 1 | 1 | -0.02068 | 0.672714 |
| SIMC1 | 1 | 1 | -0.02031 | 1.564001 |
| CHST1 | 1 | 1 | 0.019432 | 1.734965 |
| UXS1 | 1 | 1 | 0.019245 | 1.285908 |
| ARHGEF10 | 1 | 1 | -0.01716 | 1.181607 |
| ATP6V0C | 1 | 1 | -0.01688 | 0.647128 |
| NPHS1 | 1 | 1 | -0.01676 | 0.944504 |
| LOC729867 | 1 | 1 | -0.01647 | 1.548252 |
| LOC101928 | 1 | 1 | -0.01569 | 0.450578 |
| DMXL1 | 1 | 1 | -0.01378 | 2.130052 |
| LINC01725 | 1 | 1 | -0.01351 | 0.942932 |
| FOXN3 | 1 | 1 | 0.013406 | 1.613409 |
| MAPK11 | 1 | 1 | 0.012833 | 1.254437 |
| STPG3-AS1 | 1 | 1 | 0.012639 | 0.837203 |
| RGPD4 | 1 | 1 | 0.012101 | 2.344136 |
| LURAP1 | 1 | 1 | 0.010069 | 1.950177 |
| TPH1 | 1 | 1 | 0.009925 | 1.484927 |
| PAIP2 | 1 | 1 | -0.00985 | 0.990624 |
| COL9A3 | 1 | 1 | -0.00971 | 1.982903 |
| PMS2P2 | 1 | 1 | -0.008 | 0.973494 |
| LOC101930 | 1 | 1 | -0.00772 | 1.34603 |
| RFX2 | 1 | 1 | -0.00768 | 0.378361 |

| | | | | |
|---|---|---|---|---|
| DNM1 | 1 | 1 | 0.007561 | 1.744963 |
| TXNDC5 | 1 | 1 | 0.00749 | 2.657957 |
| KLHDC3 | 1 | 1 | 0.007338 | 1.136098 |
| MCPH1-AS | 1 | 1 | 0.006032 | 2.586839 |
| RETREG3 | 1 | 1 | 0.005514 | 0.584392 |
| VPS26A | 1 | 1 | -0.00531 | 3.020299 |
| LOC10272З | 1 | 1 | 0.004727 | 1.623862 |
| DRD4 | 1 | 1 | -0.0046 | 0.671742 |
| TMEM132I | 1 | 1 | 0.00451 | 0.423371 |
| HYAL1 | 1 | 1 | 0.004443 | 1.466326 |
| GNG3 | 1 | 1 | 0.004401 | 1.264138 |
| GUSBP1 | 1 | 1 | -0.00435 | 4.272586 |
| UBE2MP1 | 1 | 1 | -0.00309 | 1.645655 |
| USP35 | 1 | 1 | 0.002811 | 4.823636 |
| MOCOS | 1 | 1 | -0.00252 | 4.336649 |
| UBE2QL1 | 1 | 1 | -0.00243 | 1.556981 |
| HSPA14 | 1 | 1 | -0.00236 | 4.554858 |
| XKR4 | 1 | 1 | 0.002116 | 0.7158 |
| CYP17A1 | 1 | 1 | 0.00171 | 0.654626 |
| ZFP2 | 1 | 1 | -0.00166 | 1.218708 |
| PPWD1 | 1 | 1 | 0.00166 | 1.277712 |
| GPKOW | 1 | 1 | 0.000963 | 4.771648 |
| PSME2 | 1 | 1 | -0.00066 | 5.348375 |
| ZFPL1 | 1 | 1 | -0.00064 | 4.338236 |
| TRAF7 | 1 | 1 | 0.000624 | 6.343914 |
| DNAJB2 | 1 | 1 | 0.000577 | 3.166466 |
| ZNF585B | 1 | 1 | 0.000379 | 4.716888 |
| LINC00665 | 1 | 1 | 3.45E-05 | 1.886169 |
