# Supplementary material for: Transcriptomic Profiling of Laser Capture Microdissected FFPE Esophageal Tissues Identifies Candidate Genes and Pathways Associated with Esophageal Adenocarcinoma
Source: Int J Mol Sci. 2026 Sep 14;27(18):8164. doi: 10.3390/ijms27188164 (PMC13607975; doi:10.3390/ijms27188164)
Supplement: Supplementary file 1 [file ijms-27-08164-s001.zip › Supplementary Table S4_BL vs AC-DE .pdf]

GeneSymb	FDR	pval	log2FC	log2CPM
TM4SF4	1.89E-14	1.44E-18	6.917552	6.096497
OTC	7.69E-10	1.17E-13	10.21249	3.806557
CLCA1	1.27E-06	2.91E-10	6.055191	5.896732
SLC28A2	1.37E-06	4.87E-10	4.035216	5.915484
REG4	1.37E-06	5.22E-10	5.479072	5.388436
ANPEP	1.78E-06	8.14E-10	5.863865	9.881295
GSTA1	5.72E-06	3.09E-09	6.12748	4.966648
ANOS1	5.72E-06	3.49E-09	-9.15028	3.36001
ENTPD5	3.80E-05	2.61E-08	3.089011	5.778764
SULT1B1	0.000121	9.95E-08	3.251914	5.9674
PBLD	0.000121	1.05E-07	2.764906	5.145286
TMPRSS15	0.000121	1.11E-07	6.707306	4.611503
INHBA	0.000205	2.03E-07	-4.00806	6.245719
SMIM32	0.000239	2.55E-07	5.978944	3.353186
FABP1	0.000741	8.78E-07	6.086009	5.998921
LGALS4	0.000741	9.03E-07	2.834821	8.933879
HAPLN4	0.000934	1.21E-06	4.896957	3.162662
CMBL	0.000972	1.33E-06	2.157746	6.520303
VWDE	0.001192	1.73E-06	-9.34351	3.545999
TM6SF2	0.00179	2.73E-06	4.162228	4.167495
TRHDE	0.001905	3.05E-06	5.067627	3.676265
HHIP	0.001995	3.35E-06	3.257071	5.200043
REP15	0.003107	5.45E-06	3.576501	4.076082
HSD17B2	0.00349	6.39E-06	3.171749	5.650393
CFTR	0.004756	9.06E-06	2.975735	7.336913
OLFM4	0.005157	1.03E-05	4.29334	9.473086
GALNT7	0.005157	1.06E-05	2.214931	7.775034
DPT	0.006048	1.29E-05	3.130413	4.557426
C3	0.007293	1.61E-05	-3.13463	8.646696
SLC3A1	0.007786	1.91E-05	3.791828	4.820386
HLA-DRB5	0.007786	1.92E-05	-7.37751	4.564837
ACE2	0.007786	1.92E-05	3.901778	4.558442
GPA33	0.007786	1.96E-05	3.410444	6.116402
PCK1	0.007786	2.02E-05	3.388248	6.462377
ZFP57	0.008359	2.23E-05	8.275092	1.992907
NBPF7	0.008726	2.42E-05	5.610201	2.295917
GPT	0.008726	2.46E-05	2.443081	4.521205
BMP5	0.008782	2.54E-05	6.282971	3.602438
COMP	0.013421	3.99E-05	-7.1832	3.438553
SLC10A5	0.017512	5.34E-05	3.580418	3.485091
SULT1E1	0.01764	5.66E-05	2.782071	4.336222
HAPLN1	0.01764	5.77E-05	3.401817	4.067499
VNN1	0.01764	5.78E-05	3.654557	5.443423
ATP4A	0.017935	6.01E-05	-11.1396	5.29857

SLC26A3	0.020458	7.02E-05	4.816572	4.909415
TRPA1	0.020461	7.26E-05	2.440632	6.140652
VILL	0.020461	7.33E-05	1.986197	6.743706
GALNT8	0.020673	7.56E-05	4.214491	2.822303
LINC02432	0.021084	7.87E-05	5.087959	2.119127
CSNK1A1P1	0.024725	9.42E-05	4.253253	3.135064
TTBK1	0.02482	9.65E-05	-7.91493	2.217323
ALDOB	0.026634	0.000106	4.901096	6.249879
ELFN2	0.027242	0.00011	-7.64718	1.978402
THBS1	0.028215	0.000116	-2.50864	10.32592
KRT20	0.02972	0.000127	5.972365	4.843199
ANO7	0.02972	0.000129	2.510744	5.061968
MS4A8	0.02972	0.000129	3.637977	4.34947
BST2	0.031243	0.000138	-2.03038	6.038012
MEIS1-AS3	0.031689	0.000144	-7.91173	2.204583
SOSTDC1	0.031689	0.000147	3.25276	2.998239
XIST	0.031689	0.000147	-11.2325	8.610522
SMPD3	0.033673	0.000159	2.2663	6.581875
SERPINA3	0.033673	0.000162	-4.50642	5.41103
EIF5A2	0.035804	0.000177	-3.8948	3.598963
POTEF	0.035804	0.000177	-9.46687	3.666676
TTLL6	0.037894	0.000191	3.45031	4.038005
GFPT2	0.041577	0.000212	-3.50876	4.695982
CHST5	0.042787	0.000222	3.995125	5.20437
MOGAT2	0.042787	0.000225	4.502356	2.558662
YBX2	0.043845	0.000234	-5.89969	2.721988
TXNDC5	0.044296	0.00024	2.534001	4.368502
VWA3A	0.044636	0.000245	-6.11578	3.383079
CAPN5	0.045743	0.000255	1.886409	7.133763
WNT11	0.045868	0.000259	-12.1466	6.296246
SNORD143	0.047916	0.000274	-7.33113	1.704759
CXCL5	0.052371	0.000303	-5.51358	5.022927
UGT1A5	0.05295	0.000314	5.175071	1.924227
TMEM229A	0.05295	0.000315	4.530959	2.586665
LINC00443	0.055917	0.000337	5.914645	1.565686
FER1L6	0.055917	0.000341	2.821002	7.475237
SI	0.056094	0.000346	5.169688	7.791887
HIGD1A	0.058174	0.000364	2.375365	4.126767
TMEM45B	0.058427	0.00037	1.778691	6.809398
MYOC	0.058788	0.000387	-7.17995	4.741979
LOC101409	0.058788	0.000396	-7.26345	1.644605
FAM83E	0.058788	0.000398	2.060939	6.043534
PROX1-AS1	0.058788	0.000401	-8.29776	2.567381
TFF3	0.058788	0.000402	2.982381	6.265233
CFI	0.058788	0.000402	1.935739	5.544295

C11orf96	0.058788	0.000403	-2.22491	5.223091
MST1L	0.061735	0.000433	-5.93612	3.195777
AFAP1-AS1	0.061735	0.00044	2.213123	6.209384
CLDN10-AS	0.061735	0.00044	5.806093	1.607837
LOC101928	0.061735	0.000442	4.040764	2.165515
SPACA4	0.061809	0.000448	3.237877	3.394796
IGFN1	0.062039	0.000454	-4.71236	6.465622
PLXNB1	0.06412	0.000474	3.110622	3.603306
MUC2	0.065336	0.000488	5.559032	10.97364
SST	0.065444	0.000494	6.25977	4.790158
PLAC8L1	0.066494	0.000507	5.447392	1.726897
JCHAIN	0.067949	0.000523	2.78816	10.04066
AQP2	0.069676	0.000546	-5.4815	4.313742
ERICH3	0.069676	0.000548	-8.16495	2.442134
FOXD1	0.069676	0.000559	2.755675	3.638459
SFRP4	0.069676	0.000559	-4.79602	6.255201
MIR3679	0.069676	0.000565	-7.25762	1.642116
SYNDIG1	0.069676	0.000568	-6.88943	1.334151
IGSF9B	0.070446	0.00058	-2.42564	4.989503
HMGCR	0.07091	0.000591	4.530469	2.296435
SMIM24	0.07091	0.000595	2.311718	6.77874
GAS2L2	0.071427	0.000609	-7.07636	1.489422
KCNK15	0.071427	0.00061	-7.13691	1.537486
PCSK5	0.071894	0.000619	2.531811	5.587259
C2orf40	0.076302	0.000668	-7.79731	2.103413
UGT2B17	0.076302	0.000669	6.228752	1.711917
BARX1	0.077404	0.000684	-6.56979	3.7003
ZNF728	0.077841	0.000694	-7.76647	2.077347
ZNF44	0.085586	0.00077	-6.59919	1.098097
HM13-AS1	0.087642	0.000795	-5.4524	1.645906
MIR548I2	0.089119	0.000815	3.044953	3.647976
MEP1A	0.089374	0.000828	4.936622	5.876683
CD70	0.089374	0.000831	-7.78213	2.101573
PHLDA2	0.089618	0.000847	-7.90455	2.192901
MUC3A	0.089618	0.000847	2.315295	10.28211
GALM	0.090404	0.000862	1.746036	5.6205
HGD	0.090404	0.000868	2.540027	5.157783
MIR590	0.092746	0.000898	-6.66479	1.147913
BTBD16	0.094108	0.000918	-5.51461	2.82065
NALCN	0.09445	0.000929	-3.5871	3.688837
LEFTY1	0.098491	0.000976	2.89306	5.987369
SNORA22	0.102441	0.001023	-2.06701	5.951503
SLC44A5	0.103037	0.001037	-8.00579	2.296119
PTGS2	0.103632	0.001051	-2.49173	4.905412
THBS2	0.105844	0.001086	-2.30477	7.843872

TREX2	0.105844	0.001096	-6.88575	1.331414
SSTR5-AS1	0.105844	0.001097	3.160982	3.241493
ICAM1	0.111949	0.001174	-2.33102	6.044454
NUP107	0.111949	0.001178	-1.89789	6.216661
LBH	0.12044	0.001283	-1.97556	6.651441
FAM177B	0.12044	0.001285	2.29042	5.12602
LSM12	0.122849	0.001323	-2.76316	4.967772
IFITM2	0.122849	0.00133	-1.72521	5.852099
ONECUT2	0.122884	0.001339	2.347269	7.258608
GGH	0.122939	0.001365	-4.60636	4.673816
SNORD116	0.122939	0.001366	-2.84338	7.382525
FOXRED1	0.122939	0.001373	-7.73615	2.060396
TIMP1	0.122939	0.001381	-2.00459	7.31334
USP17L2	0.122939	0.001387	-6.68963	1.17469
GOLGA8A	0.124244	0.001411	1.537084	5.589949
PCF11-AS1	0.125656	0.001438	-4.70012	2.088976
ALPG	0.125656	0.001446	-6.70771	2.684406
GPR3	0.125797	0.001461	-6.8332	1.286149
MIR1255B2	0.125797	0.001492	7.258879	1.138611
MET	0.125797	0.001494	8.038768	1.786669
SLC7A8	0.125797	0.001495	1.620121	5.290661
DEPP1	0.125797	0.001502	-2.07032	7.409267
RBBP6	0.125797	0.001512	-6.96458	1.399371
SLC9A3R1	0.125797	0.001515	1.4265	7.027117
TEX9	0.126241	0.00153	8.025783	1.775321
MLEC	0.130895	0.001596	3.525883	2.229038
CHGB	0.131101	0.001617	-6.89859	1.341353
FOXN3	0.131101	0.001624	2.352336	3.135985
ITLN1	0.131101	0.001629	3.826834	6.24347
LEKR1	0.131602	0.001651	-6.82153	1.274152
C4BPA	0.131602	0.001655	-7.52276	1.864189
FAM171A1	0.131758	0.001674	1.745284	6.454735
TNFAIP6	0.131758	0.001677	-5.67995	2.960389
SNORD116	0.13287	0.001701	-2.88452	8.807725
LOC28445	0.13425	0.001742	-1.65065	6.119922
LRRC66	0.13425	0.001748	2.36781	4.298075
ZNF470	0.13425	0.001763	-2.83637	3.634736
TPSB2	0.13425	0.001767	2.867159	3.817931
BTNL9	0.13425	0.00177	1.865	4.933149
B4GAT1	0.136243	0.001807	1.949305	4.827697
PDX1	0.136538	0.001821	1.903246	5.011086
HTR1B	0.137318	0.001848	-7.51332	1.865261
KCNK5	0.137318	0.001853	2.079801	6.618743
LINC01429	0.137804	0.001878	-7.15109	1.549724
SLC52A3	0.137804	0.00188	-2.28134	4.466633

ACY3	0.138873	0.001905	2.721545	3.119563
TCEAL2	0.140732	0.001942	-5.35459	2.663953
DNAJC9-AS	0.144597	0.002006	-4.76314	1.745862
STEAP1	0.14699	0.00205	-2.09639	5.195614
ZNF169	0.148011	0.002076	3.995925	1.974267
GATM	0.148032	0.002087	1.721961	5.978728
RBP2	0.148352	0.002117	-6.30614	0.876427
LINC00261	0.148352	0.002121	2.419551	6.532955
EVADR	0.148352	0.002127	4.551749	1.94125
PSD2	0.148352	0.002143	-6.51069	1.028069
C11orf86	0.148352	0.002159	6.207748	1.96858
CENPP	0.148352	0.002165	-6.49154	1.013008
LINC01615	0.148352	0.002171	-6.89205	1.336072
FRG1DP	0.14928	0.002196	-7.77136	2.077142
ARNT2	0.150417	0.002224	-2.53422	4.527785
MGC12916	0.151893	0.002258	-4.55066	2.371644
ATP2C2	0.153631	0.002317	4.544214	1.763528
SNORD116	0.153631	0.002322	-2.81888	7.8279
NMUR2	0.153631	0.002326	3.02511	3.275297
MADD	0.153631	0.002336	-7.26692	1.650926
ASB16-AS1	0.153631	0.002342	-6.56232	1.067594
TMC3-AS1	0.153792	0.002358	-6.46088	0.996768
C7	0.153792	0.002368	-3.28548	7.34587
ABCC6P2	0.153959	0.002405	4.270693	2.334378
LRRC3B	0.153959	0.002406	6.939761	0.898435
LMCD1-AS1	0.153959	0.002406	-5.58651	2.001842
ANXA10	0.153959	0.002417	2.737473	6.266629
GSTA2	0.154311	0.002435	5.453632	1.197124
NOTUM	0.154827	0.002455	-8.7252	6.98163
TTC38	0.156606	0.002495	1.517616	5.796833
DEGS2	0.157443	0.002525	2.589992	3.618038
MAP9	0.157443	0.002532	-1.9418	5.392164
S1PR3	0.158234	0.002561	-2.30831	5.30039
LOC101929	0.158234	0.002569	-6.51438	1.036742
ITGBL1	0.15898	0.002593	-2.63671	4.661655
IGF2BP3	0.162513	0.002663	-2.56947	4.882083
GAL	0.165412	0.002724	-6.92777	1.363944
KCND3-AS1	0.165412	0.002769	-7.1645	1.562538
TMEM253	0.165412	0.002776	2.589376	3.121794
LOC102724	0.165412	0.002782	3.288265	2.210172
CPA3	0.165412	0.002783	1.863322	5.221259
LRRC26	0.165412	0.002786	4.415334	2.016585
KIF26B	0.165412	0.002799	-2.61245	3.765997
FOXD4L5	0.165455	0.002832	-7.05788	1.478894
EEF1A2	0.165455	0.002837	-7.17154	1.57368

SAR1A	0.165455	0.002837	-6.66382	1.154096
FGF19	0.165604	0.002853	-9.86482	4.051972
FAM177A1	0.169013	0.00293	3.613669	3.675189
WNK4	0.169013	0.002937	2.034104	6.133289
ASB5	0.169296	0.002958	-7.63321	1.946352
FBXL18	0.169296	0.002972	-1.63256	5.281185
SNORD116	0.169296	0.002981	-2.75291	7.639669
FBXO39	0.169572	0.002999	-6.29955	0.863421
TST	0.170861	0.003042	4.489042	1.750172
ADAMTS4	0.170861	0.003047	-1.57887	6.047093
PDPR	0.172105	0.003086	-1.56822	6.545483
ETHE1	0.172105	0.003096	1.551895	5.16039
PRPH	0.173095	0.003127	-6.71078	1.190318
HEYL	0.17325	0.003151	-2.14743	5.128394
FAM216B	0.17325	0.003168	-7.16723	1.553661
TRIM17	0.17325	0.003171	-5.27507	2.498998
NKD1	0.17325	0.003182	-4.4452	8.872542
LRRIQ1	0.173435	0.003223	-6.96582	1.383441
HOXB13	0.173435	0.003255	3.16673	3.483487
ZNF32	0.173435	0.00326	-7.17407	1.577364
ACYP1	0.173435	0.003278	-6.50677	1.02922
HLA-F-AS1	0.173435	0.003281	-4.63393	1.603506
GUCY2C	0.173435	0.0033	2.138523	6.036085
LOC100268	0.173435	0.003307	-7.43056	1.792572
SNORD116	0.173435	0.00331	-2.55443	7.026403
MFSD4A-A	0.173435	0.003311	-6.89485	1.325166
LINC00323	0.173435	0.003318	-6.3687	0.927415
MYRFL	0.174161	0.003348	2.89338	3.924486
TLE4	0.174161	0.003365	-6.29079	0.862426
CCDC158	0.174161	0.00338	-4.13437	2.741922
IFT88	0.174161	0.003385	-6.64795	1.144251
KIAA1522	0.176869	0.003451	2.741927	3.141015
SNORD116	0.181951	0.003573	-2.59592	5.658928
LRP2	0.181951	0.003578	-3.21692	2.350845
UCA1	0.184418	0.003641	-4.76851	4.712298
LINC00271	0.184836	0.003663	-7.50051	1.85566
ABCD3	0.188478	0.003757	1.308176	6.711802
ASB4	0.188478	0.003764	-8.95964	3.183715
PAX9	0.189502	0.00381	2.226962	4.48606
SLC15A1	0.189502	0.003824	2.890947	3.314446
TNS4	0.189502	0.003828	-2.57131	6.233158
SALRNA2	0.191235	0.003893	-6.38068	0.928018
ALPK3	0.191235	0.003897	-1.60413	5.904173
BGN	0.191235	0.003911	-1.74739	7.492332
ATRIP	0.191235	0.003938	-6.21017	0.793496

PRB2	0.191235	0.003949	-5.7909	1.931995
PIGR	0.191235	0.003964	3.792363	13.07999
SDS	0.191235	0.003974	-3.47003	3.190409
CHRNB2	0.191235	0.003979	-5.34725	1.793951
FLOT1	0.196635	0.004107	2.540993	3.343489
NEPRO	0.197149	0.004132	-6.44474	0.964421
RFX6	0.197201	0.004174	4.057307	2.730277
CXCL13	0.197201	0.004174	-7.25189	1.631474
LOC10192(	0.197201	0.004193	-6.22063	0.819856
NVL	0.197201	0.004194	7.440258	1.297686
SNHG17	0.197697	0.004227	-7.50919	1.863141
LOC28493(	0.197697	0.004243	-7.81017	2.128845
LINC01266	0.197697	0.004249	-7.25605	1.627157
LINC01184	0.202247	0.004399	1.419018	6.17647
PRKAA2	0.202247	0.004434	-3.21487	4.559494
FAM204A	0.202247	0.004478	-6.68734	1.15699
MIR614	0.202247	0.004483	-3.78034	2.807005
TSFM	0.202247	0.0045	-4.61279	1.517133
PLCB1	0.202247	0.00451	7.457602	1.292813
SLC35A1	0.202247	0.004526	-4.30237	1.309475
SLITRK5	0.202247	0.004533	-7.16904	1.571304
SYN2	0.202247	0.004541	-7.39866	1.771459
SBK1	0.202247	0.004544	-3.76834	2.209148
RIMS3	0.202247	0.004565	-2.6966	3.914773
LINC01088	0.202247	0.004574	-6.97422	1.383918
CAPN14	0.202247	0.004581	4.160088	5.874715
PRSS12	0.202247	0.004582	1.623766	5.662114
AQP9	0.202247	0.004599	-4.43678	3.452258
SNORD110	0.202247	0.004609	-2.11599	4.409667
NBAS	0.202247	0.004609	3.430544	1.932952
TM4SF1	0.202864	0.004639	-1.29805	7.920671
LINC01600	0.205168	0.004707	-5.75158	1.895622
HLA-H	0.212269	0.004886	1.914987	5.296599
PLIN4	0.217249	0.005018	-2.72009	4.109692
EXOC3L4	0.217249	0.00504	2.333933	3.440652
C5orf22	0.217249	0.00505	-6.54791	1.054532
RAB27B	0.217643	0.005076	1.668669	7.174301
MIR1296	0.2181	0.005103	-7.016	1.443183
CCDC88C	0.218272	0.005124	1.369975	7.756984
PRKAR1B	0.219624	0.005173	-5.99393	0.654487
RASGRP2	0.22013	0.005201	-4.76545	1.091724
ARID5A	0.221513	0.005251	-1.66478	4.913768
PPP1R13B	0.222011	0.00528	1.24754	6.109311
IGFL4	0.222986	0.00532	-7.50893	1.865425
CAMKK1	0.223667	0.005353	-6.601	1.110375

GCSH	0.223852	0.005385	6.070669	1.018658
CDH22	0.223852	0.005397	-7.46244	1.8209
SERPINB6	0.223852	0.005409	1.43968	5.694867
NEUROG3	0.225515	0.005491	3.877127	1.907283
BIRC8	0.225515	0.005494	5.984794	1.035982
DSCC1	0.225515	0.0055	-3.01973	3.402654
RIPK2	0.225666	0.005521	-2.18428	4.569963
ETFBKMT	0.226208	0.005552	-6.93725	1.378542
GLP1R	0.227435	0.00561	3.562806	3.035101
BATF2	0.227435	0.005617	-6.56754	1.073876
GFM1	0.228194	0.005673	-4.7831	1.097471
ZNF713	0.228194	0.005683	-2.14119	3.959237
HFM1	0.228194	0.005687	-7.18171	1.568265
CACNA1C-/	0.228723	0.005718	-6.61718	1.113967
DIO3	0.230751	0.005804	-7.26419	1.656557
BLACAT1	0.230751	0.005826	-2.84924	3.945994
LOC101927	0.230751	0.005836	-6.21118	0.808745
FABP2	0.230751	0.005843	3.586744	3.775344
KIF3C	0.230751	0.005857	-2.0483	4.84642
CCL2	0.231194	0.005886	-2.78185	4.329492
LOC101927	0.231764	0.005918	2.903695	2.265626
UQCRQ	0.235271	0.006025	1.43485	5.694036
CACNG8	0.235654	0.006056	-3.08636	3.764645
DKK2	0.235654	0.006079	-7.18645	1.587971
CXCL2	0.235654	0.006089	-2.20255	4.990618
ATRNL1	0.235721	0.006113	2.970699	2.883201
AMN	0.235721	0.006148	2.468958	5.765218
LOC10013(	0.235721	0.006158	1.536749	4.858581
IL17REL	0.235721	0.006201	2.895323	3.558971
SLITRK3	0.235721	0.006211	-7.45183	1.797838
DLGAP3	0.235721	0.006221	-6.34792	0.909306
MME	0.235721	0.006224	-7.31902	1.697142
FAM200A	0.235721	0.006234	-2.61126	4.120883
TGM4	0.237017	0.006287	-7.02888	1.454412
SNORD116	0.237803	0.006353	-2.98835	5.559583
ALPP	0.237803	0.006358	-5.16858	1.968454
CNNM2	0.237803	0.006373	3.924267	1.451907
LNX1-AS2	0.237803	0.006382	4.504652	1.502366
COL23A1	0.237803	0.006398	2.310199	3.825989
ZNF826P	0.238043	0.006423	-5.25435	1.481486
CILP	0.238357	0.006449	2.061436	4.192469
GRM6	0.239437	0.006515	2.623901	3.122018
LINC01596	0.239437	0.006515	3.732217	1.560192
SOD2	0.242433	0.006619	-2.00821	8.060789
TRNP1	0.242433	0.006634	1.689805	6.27805

MIR4714	0.243342	0.006697	-6.61486	1.102429
FAM71D	0.243342	0.006705	-4.8907	1.814789
ABL2	0.243342	0.006714	-6.01254	0.662775
LINC01446	0.244074	0.006753	-7.80989	2.131211
MMP3	0.244186	0.006777	-4.17242	5.27833
LINC00032	0.244186	0.006793	2.995315	2.958346
PTGIS	0.245159	0.006839	-2.15979	5.599737
PAPPA	0.245949	0.00688	1.291995	7.438643
LRRC37A3	0.25232	0.00711	-6.26805	0.845582
SLC20A1	0.25232	0.007114	-1.3921	7.296294
SNORD116	0.25232	0.007116	-2.26955	7.034655
SLC24A5	0.254067	0.007184	-7.03445	1.437976
PLBD1	0.254389	0.007227	1.308975	6.671636
KRT15	0.254389	0.007235	3.361135	6.018337
KIF12	0.254389	0.007267	1.954809	4.680802
ANKRD36B	0.254389	0.007271	-1.39313	6.984498
LINC00278	0.254737	0.007375	2.984407	2.835066
TMC6	0.254737	0.007376	-6.28929	0.869714
HOXC13	0.254737	0.007395	-7.37785	1.739508
CD200R1L	0.254737	0.007411	6.970384	0.926942
RP1	0.254737	0.007413	-6.70693	1.195815
CYP2C19	0.254737	0.007449	2.025247	5.711296
LOC554206	0.254737	0.007454	-6.43571	0.961723
SNORD116	0.254737	0.00746	-2.46689	7.521011
KDM7A-DT	0.254737	0.007472	1.691401	4.630273
PLXNA1	0.254737	0.007475	-1.40512	7.263437
LOC729737	0.255118	0.007506	1.799074	4.051809
CKMT1B	0.25688	0.007577	1.873163	3.962661
CDS1	0.257207	0.007606	1.237016	5.986346
INPP1	0.257437	0.007641	-6.23456	0.830434
HLA-DRB1	0.257437	0.007652	2.157079	6.22925
TOX2	0.257687	0.007688	4.236243	1.540572
C5AR1	0.257687	0.007699	-1.89569	4.670904
MIR4712	0.257891	0.007725	-6.45156	0.986287
FCGBP	0.258638	0.007767	2.937843	11.8506
IQGAP3	0.26003	0.007872	-1.43446	6.589126
G0S2	0.26003	0.007887	-2.27703	4.717295
LRIG2	0.26003	0.007905	3.835944	2.978543
OSMR-AS1	0.26003	0.007948	-5.01879	1.528529
MDK	0.26003	0.00795	4.73335	1.415049
SLC30A1	0.26003	0.007991	-1.52152	5.112141
IQGAP2	0.26003	0.008001	1.759129	5.099316
MXD4	0.26003	0.008005	1.184288	5.819549
LINC01655	0.26003	0.008009	-6.89038	1.342855
LINC00598	0.26003	0.00803	-6.23973	0.823694

ANKRD36B	0.26003	0.008039	1.860839	6.261053
DEPDC4	0.26003	0.008047	-6.28004	0.847303
DNAH14	0.26125	0.008104	-2.28579	5.225195
EPB41	0.264755	0.008235	-4.71698	1.599384
SIX3	0.264755	0.008253	-6.42957	0.967542
MOV10	0.265392	0.008304	-3.75548	1.609017
PLCE1-AS2	0.265392	0.008315	2.06417	3.554662
TFIP11	0.265392	0.008334	-7.46146	1.823208
NDC80	0.265889	0.00837	-2.79897	5.427079
LOC101928	0.267683	0.008447	-6.74122	1.212664
SLCO4A1	0.267915	0.008474	-1.88314	5.170497
SPATA13-A	0.26901	0.008545	-2.7758	2.825442
PRR7	0.26901	0.00855	-6.7159	1.203052
GPR83	0.269217	0.008577	-6.45176	0.984071
NLGN1-AS1	0.269565	0.008609	-5.07193	1.888376
DCDC2C	0.269689	0.00866	-4.55197	1.526063
MYO7B	0.269689	0.008688	1.759866	7.793707
SH3GLB1	0.269689	0.008691	-6.31024	0.873988
LINC00494	0.269689	0.008714	-3.40565	2.548347
BBIP1	0.269689	0.008716	2.530301	2.916467
NFE2L3	0.269832	0.008741	-1.28461	6.292217
GIGYF2	0.269877	0.008763	-6.34218	0.891444
LINC00482	0.271205	0.008827	-3.77244	2.464988
JUNB	0.27174	0.008884	-1.34168	7.555583
SUCLG2-AS	0.27174	0.008889	-6.69701	1.174742
PGA4	0.27174	0.008906	-7.8851	5.057627
CST1	0.272156	0.008997	-6.24173	0.829698
MEDAG	0.272156	0.009019	-2.73896	5.582076
SULT1A1	0.272156	0.009021	-6.48035	1.010917
NALT1	0.272156	0.00903	-6.51994	1.021392
SPATA46	0.272156	0.009041	2.636816	2.552718
SNAI1	0.272156	0.009075	-5.01948	1.543174
MAGI1	0.272156	0.009087	-6.26959	0.858968
TNFRSF14-.	0.272156	0.009101	1.338972	5.613023
LGI2	0.272156	0.009106	-2.87581	3.974279
KIF3A	0.272186	0.00913	-6.44596	0.989255
CPS1-IT1	0.272186	0.009165	4.489897	1.574963
SLC22A31	0.272186	0.00917	-6.77922	1.255159
ESRP2	0.272218	0.009191	1.280256	6.492985
CCL28	0.273189	0.009245	3.161325	2.137622
MMP21	0.273792	0.009286	-6.5924	1.098404
OBSCN-AS1	0.27459	0.009334	-6.12783	0.752429
MIR151A	0.275207	0.009395	-6.29411	0.848454
S100A5	0.275207	0.009412	-6.20215	0.802022
MIR595	0.275207	0.009434	-6.73407	1.218188

SNORD116	0.275207	0.009439	-2.19079	8.314229
KCTD6	0.275323	0.009464	-6.46295	0.980569
ENO2	0.277962	0.009576	-1.55225	5.394745
RBM11	0.278389	0.009612	-6.11941	0.735626
KIAA1211	0.27928	0.009664	1.33834	6.610773
CCNB1IP1	0.279658	0.009698	-4.33047	0.764078
GAS1	0.282557	0.009821	-3.05507	3.168442
SNORD116	0.283289	0.009898	-2.37231	4.619211
BBOX1	0.283289	0.009933	2.548262	3.770083
SENP6	0.283289	0.009964	-2.62915	3.202272
CYP21A1P	0.283289	0.009975	-6.63596	1.115776
HTR2A	0.283289	0.00998	-6.45895	0.977227
UGT1A7	0.283289	0.00998	3.691694	2.79406
UBXN6	0.283289	0.010025	3.021668	2.319219
LOC10272З	0.283289	0.010039	2.358419	3.303634
PURPL	0.283289	0.010041	3.680576	3.719368
LINC01039	0.283289	0.010083	-6.48263	1.001203
ILDR2	0.283289	0.010084	-3.67783	3.357463
TXLNG	0.284399	0.010151	-3.25328	2.839295
INHBA-AS1	0.284399	0.010166	-6.16877	0.768843
KCNK6	0.28491	0.010206	1.188141	7.013851
LONRF2	0.285111	0.010273	-2.88312	6.258682
AQP7P3	0.285111	0.010273	-4.05105	1.820583
SEPHS2	0.285111	0.010279	1.204317	7.023019
GJA8	0.286449	0.010349	5.244734	1.044521
TP53TG5	0.286449	0.010407	-4.76902	1.096758
TMEM54	0.286449	0.010412	1.377644	5.505964
NAT2	0.286449	0.010426	3.223099	1.901004
RMND1	0.286449	0.010436	-4.34174	1.771222
RETREG1	0.286702	0.010467	2.088878	2.960928
PTGDR2	0.287614	0.010535	2.683057	3.313577
HSPB6	0.287614	0.010544	-2.06788	6.399506
LINC01363	0.289631	0.01064	6.157263	1.326099
KCNJ9	0.290092	0.010679	2.966483	2.784489
FRG1HP	0.290843	0.010729	-1.61668	5.142632
SIPA1L2	0.291252	0.010767	1.319997	7.723909
SOX11	0.292258	0.010837	-6.32119	0.867846
PCBP2-OT1	0.292258	0.010848	-3.29276	2.045854
CHMP5	0.292749	0.010889	-7.20033	1.599264
LRRC75A-A	0.294225	0.010966	-6.42849	0.978883
HNF4G	0.295902	0.011051	1.50468	6.251684
FNDC1	0.296141	0.011088	-2.43017	5.880022
PSAP	0.296141	0.011105	-6.61489	1.120443
TENM4	0.296141	0.01116	-2.40068	4.631533
PAIP2	0.296141	0.011188	-6.05387	0.679518

LPCAT4	0.296141	0.0112	1.228115	6.26174
FAM3D	0.296141	0.011221	2.808441	6.357072
NXF3	0.296141	0.011226	3.410198	2.481135
TAF6	0.296141	0.011241	-3.64929	1.830254
PCAT14	0.298977	0.011371	-6.92735	1.345814
SLC2A3	0.298979	0.011394	-1.71173	6.463694
LINC00707	0.301918	0.011529	-6.26683	0.835244
MIR4257	0.302488	0.011588	-3.7496	1.825933
RPL39L	0.302488	0.011597	-4.48485	1.79757
LOC399815	0.304052	0.011702	-6.49203	1.018389
CLIC5	0.304052	0.011703	-6.30614	0.861801
AXIN2	0.306612	0.011825	-2.64277	7.961351
DLG3-AS1	0.30729	0.011875	-4.76895	1.033903
CASQ2	0.30982	0.011996	-3.89434	3.222746
SYCP2	0.311676	0.012092	-2.4011	5.249971
KLF17	0.312592	0.012151	-6.41756	0.935201
KLHL11	0.314145	0.012235	-1.26078	5.785007
DUSP8	0.3146	0.012277	3.044564	2.78682
HDGFL3	0.315773	0.012347	-1.71957	5.236576
PLET1	0.316261	0.012439	-3.84704	1.820923
NDST1	0.316261	0.012453	2.462574	2.497358
USP31	0.316261	0.012466	-1.39914	6.691995
RAB3D	0.316261	0.012499	1.287772	6.839041
LAMC3	0.316261	0.012519	1.782074	3.908573
LINC00312	0.316261	0.012531	-2.63015	3.885851
SPTAN1	0.316261	0.012535	-6.02319	0.66251
LINC01416	0.31811	0.012639	-6.85029	1.287178
RSPO3	0.31811	0.012662	-3.30617	6.625533
DUSP5	0.31811	0.012681	-2.51378	5.295537
SNORD101	0.319506	0.012761	-3.4997	1.783202
TPSAB1	0.323086	0.012952	1.762546	4.736429
FAM95B1	0.323086	0.012953	-6.14169	0.758749
LOC101059	0.323851	0.013056	-6.06729	0.706594
SERPINE1	0.323851	0.013071	-1.69627	7.619032
CYBA	0.323851	0.013084	1.313357	6.676425
POLR2L	0.323851	0.013094	1.44722	5.115819
FGF13	0.323851	0.013109	-6.21136	0.785536
ADRA2A	0.323851	0.013153	1.995474	5.483876
ZFP92	0.323851	0.013156	-6.03681	0.676784
SOCS7	0.324696	0.013216	-3.02011	3.304066
YPEL5	0.326039	0.0133	-6.13032	0.735943
CAMK1G	0.326039	0.01337	-6.0909	0.715246
HS6ST3	0.326039	0.013375	-6.71492	1.178728
S100A14	0.326039	0.013377	1.512916	8.025001
POU5F1	0.326039	0.013394	4.091575	1.895997

FBXL12	0.327065	0.013478	2.976625	1.573018
SOD3	0.327065	0.013512	-1.94724	6.960705
PCLO	0.327065	0.013544	-6.64853	1.13784
SACS-AS1	0.327065	0.013548	-7.02503	1.43475
FEZF1-AS1	0.327065	0.013566	2.251515	3.34141
EPHA10	0.327065	0.013586	1.367645	5.365781
ECSIT	0.327431	0.013628	-3.93254	0.981636
SKIV2L	0.327431	0.013651	1.939201	3.227945
WIF1	0.328055	0.013713	-5.98195	3.848815
KRT4	0.328055	0.013741	5.530629	10.20693
SNORD114	0.328055	0.013752	-6.73056	1.216956
SQSTM1	0.329315	0.013836	-2.31926	2.935623
SH3RF2	0.329315	0.013855	1.322317	5.93039
GAS2L3	0.330716	0.013939	3.813548	2.175821
AGAP11	0.330817	0.01398	-3.02651	3.922163
NKAIN3	0.330817	0.01401	-6.03922	0.675139
KRTCAP2	0.330817	0.014019	-1.30384	5.243717
SERPINB9	0.331837	0.014108	-1.69398	5.928406
SNORD116	0.331837	0.014113	-2.88521	5.672486
UGT1A9	0.332349	0.01416	2.570996	3.16389
RSAD2	0.333455	0.014233	-2.36842	4.951071
MARK1	0.333584	0.014264	-4.79881	1.096452
LINC00664	0.334338	0.014333	-6.07701	0.700478
SLC2A1	0.334338	0.014347	-2.04929	7.213401
IRS2	0.334358	0.014373	-1.65582	6.903945
MIR1247	0.334378	0.0144	-6.91051	1.362166
RAB6D	0.335758	0.014487	2.317841	2.790572
LINC00333	0.335758	0.01451	5.504782	0.812924
ACVR1	0.335822	0.014539	2.765553	2.199149
DIRAS3	0.336642	0.0146	-3.74634	2.066025
SPATA2	0.337346	0.014656	-6.41208	0.959515
DGKQ	0.337864	0.014748	1.1611	5.57732
CCDC71L	0.337864	0.014765	-1.82078	5.480115
TGM2	0.337864	0.014779	-1.57579	6.864116
MT1E	0.337864	0.014782	1.593592	5.804033
GBP6	0.339085	0.014861	4.353171	6.332367
GPRC5A	0.340431	0.014946	-1.52098	8.540876
FAM167B	0.340431	0.015021	-6.10452	0.713768
YOD1	0.340431	0.015067	-6.69255	1.160164
LINC01606	0.340431	0.015099	-7.18597	1.573171
SMIM14	0.340431	0.015115	3.0473	2.115055
GPR84	0.340431	0.015145	-6.10638	0.715158
AKR1B10	0.340431	0.015156	2.4843	7.095831
CAPN9	0.340431	0.015165	3.814759	1.277629
HESX1	0.340431	0.015177	-6.02761	0.661127

FKBP10	0.340431	0.015179	-1.82013	6.853884
LCA5	0.341296	0.015244	3.921183	1.27331
DCBLD2	0.342743	0.015378	-2.23167	5.884247
MMP15	0.342743	0.015396	1.170666	6.083841
DCUN1D5	0.342743	0.015423	-1.28518	6.348986
PPFIA2-AS1	0.342743	0.015434	-6.53588	1.038732
NR1D2	0.342743	0.015439	-6.07523	0.70733
MUC16	0.34345	0.015581	-3.43819	6.516829
DCAF13	0.34345	0.015599	-1.3289	5.554723
RNF152	0.34345	0.015606	1.50021	4.153076
THEM5	0.34345	0.015611	-2.32225	3.388377
FAM92A	0.34345	0.015622	-3.51845	2.168419
SOCS3	0.34345	0.015639	-1.77409	6.822871
RNF138P1	0.34345	0.015671	1.152183	5.835299
DPP8	0.34345	0.01568	4.076774	0.895237
CA13	0.343901	0.015745	1.331476	5.162286
TRMT10C	0.343901	0.015787	-1.56838	4.594149
MIR421	0.343901	0.015802	-2.9715	2.061645
SLC25A20	0.343901	0.015806	1.382773	4.521338
ARHGAP44	0.343968	0.015835	1.618149	4.862155
MARVELD3	0.344676	0.015894	1.49972	3.937491
ZNF883	0.346537	0.016006	-3.78572	1.870618
NANOS1	0.3471	0.016096	-3.99427	1.509283
BCS1L	0.3471	0.016124	4.119969	1.083986
LOC157273	0.3471	0.016169	-6.7219	1.174721
LINC02495	0.3471	0.016194	-6.72181	1.20431
ZNF665	0.3471	0.016213	-3.6301	1.808971
MOGAT1	0.3471	0.016232	-6.7045	1.185777
LOC441081	0.3471	0.016259	1.284453	4.637088
ARL5A	0.3471	0.016278	-3.85841	1.568329
LOC101927	0.3471	0.016281	2.41271	3.031219
SORBS1	0.3471	0.016297	-6.51528	1.018901
PGA5	0.347492	0.016363	-7.5433	1.893956
CUL4B	0.347492	0.016368	2.142463	3.493433
AKR1C2	0.348435	0.016439	-4.37844	1.307261
KCNE3	0.34988	0.016546	1.678281	7.728322
COL6A1	0.34988	0.016561	1.06398	9.698983
RFLNB	0.351423	0.016679	1.283533	5.141526
PCDHB7	0.351423	0.016687	-3.41474	2.35055
PXDNL	0.351539	0.016742	-6.28321	0.837707
DDHD1	0.351539	0.016761	3.151426	1.696414
KIAA1324	0.351539	0.016773	3.33446	2.496569
CARD6	0.359164	0.017164	1.173668	5.904361
AGR3	0.359306	0.017198	1.793299	4.967982
GNAS-AS1	0.364899	0.017507	-6.13099	0.740757

PYGB	0.364899	0.017522	1.164554	8.749597
ZNF146	0.365232	0.017579	-4.35169	0.779975
LOC389602	0.365232	0.017616	1.682037	6.428019
LINC00630	0.365232	0.017621	-1.85378	4.023694
CYP2C9	0.366062	0.017689	1.815585	5.299424
ARHGDIG	0.370493	0.017954	-5.89954	0.585593
GRPR	0.370493	0.018025	-6.58492	1.063607
KCNH4	0.370493	0.018029	-5.77711	0.502559
ZNF93	0.370493	0.018039	-2.77626	2.697421
TWISTNB	0.370493	0.01805	-1.95893	5.044212
EPB41L4B	0.370493	0.018077	1.343453	5.150402
IFIT5	0.370493	0.018101	-2.00351	5.179946
SPATA13	0.371263	0.018167	-4.67836	1.618017
HCG4	0.371263	0.018195	-6.49798	1.034111
RNF217	0.373016	0.01831	-3.27932	1.939768
RECQL5	0.373463	0.0184	1.162347	5.308777
SULT4A1	0.373463	0.0184	-6.66087	1.152702
IFI44L	0.373463	0.018417	-2.14257	7.05018
DPY19L1	0.374153	0.01848	-1.35898	5.878314
ADAMTS12	0.374358	0.018538	-1.98702	4.675276
TSG1	0.374358	0.018548	-7.21627	1.579565
ADRA2B	0.374358	0.018575	-3.41521	1.66433
VASH1-AS1	0.375477	0.018659	-5.91701	0.596801
LOC10012[illegible]	0.376095	0.018719	-4.59125	0.950411
ADD3-AS1	0.378955	0.01889	-4.14729	1.21898
AASDHPPT	0.379431	0.018943	-1.2123	5.438476
LINC00434	0.379694	0.019007	-6.39688	0.938655
LINC00960	0.379694	0.019041	-6.15436	0.770415
EPHA2	0.379694	0.019043	-1.43252	7.453832
FLG	0.381291	0.019152	2.822571	6.122618
KCTD17	0.381702	0.019201	-3.94994	0.989797
LINC00943	0.382695	0.019281	-6.28897	0.852556
SLC7A5P2	0.383735	0.019373	-1.37633	6.51452
CLEC7A	0.383735	0.019391	-3.87341	2.147653
RGS7BP	0.383792	0.01943	-6.13896	0.739803
SUCLG1	0.383792	0.019465	1.130293	6.115837
ISG15	0.383792	0.019482	-2.0077	4.239726
DLX6	0.384012	0.019523	-6.64278	1.141949
MYH1	0.385265	0.019616	-6.62741	1.098278
CAPNS2	0.387107	0.019755	3.816684	1.487735
POU4F1	0.387107	0.019783	-6.29259	0.845537
CHPT1	0.387107	0.019854	1.08412	5.594942
STAT1	0.387107	0.019854	-1.15927	6.510948
MALL	0.387107	0.019857	1.925274	6.547525
NLRP1	0.387855	0.019925	1.7832	3.031822

PPP1R2	0.388147	0.019987	-6.60562	1.077083
DDX10	0.388147	0.020028	-1.28278	6.686346
ZBTB37	0.388147	0.020029	-2.33083	3.097902
RUNDC3B	0.388689	0.020125	5.603341	0.894163
CXCL8	0.388689	0.020158	-3.28003	5.309403
LOC100505	0.388689	0.020162	-7.09866	1.51127
GABRP	0.388689	0.020175	3.195939	2.57096
NID1	0.389184	0.02026	1.124296	8.694235
CDRT15P1	0.389184	0.02029	-4.19422	1.250733
RP9P	0.389184	0.02031	-1.67398	4.493005
CA8	0.389184	0.020321	1.709118	4.382969
FAM20C	0.389184	0.020349	-1.42904	6.192776
LOC101928	0.390477	0.020446	-6.04161	0.682594
C6orf141	0.391161	0.02052	1.594032	3.995607
LINC01770	0.391161	0.02056	-6.35242	0.918327
LYG1	0.391161	0.020572	-3.64753	1.896812
LEMD1-AS1	0.39141	0.020615	-6.32447	0.88655
ADAMTS8	0.393934	0.020778	-3.01776	3.294201
IQCH	0.394015	0.020812	-6.3829	0.931866
ZFP28	0.394108	0.020859	-2.84382	2.861502
APLF	0.394108	0.020877	-1.639	4.57758
CYP2S1	0.395607	0.020986	1.365498	6.95524
MYORG	0.3959	0.021073	1.17598	6.225652
VSIG2	0.3959	0.021134	1.613568	5.573239
GDNF	0.3959	0.021142	3.899345	1.163397
FIS1	0.3959	0.021172	1.112951	5.830343
ARHGEF26-	0.3959	0.021179	-6.14149	0.741068
HIST2H2AB	0.3959	0.021183	-1.67845	4.298634
TNFSF12	0.397653	0.021307	2.903307	2.502608
LARGE-AS1	0.398301	0.021403	1.662779	3.098626
MAP2	0.398301	0.021414	-2.32979	4.732492
RYR2	0.398301	0.021451	-1.51672	6.168094
LRP4-AS1	0.398301	0.021463	-5.4562	1.641446
HSPB7	0.399698	0.021593	-2.81996	4.365925
KLHL30	0.399698	0.021688	-3.48241	1.826974
DHRSX	0.399698	0.021701	-5.22432	1.370101
VWF	0.399698	0.02172	1.169654	8.667285
GNL3	0.399698	0.021727	2.910828	1.424362
SLK	0.399698	0.021781	-3.94462	1.051858
TMEM26	0.399698	0.021802	-6.04041	0.677731
IDO2	0.399698	0.021807	-6.30736	0.861025
AEBP1	0.399698	0.02184	-1.37246	8.824751
PLD1	0.399698	0.021848	4.382062	0.833673
EPB41L4A	0.399698	0.021874	1.055625	6.414331
GPRIN2	0.400674	0.021958	1.963653	2.837165

LOC442497	0.403769	0.022158	-3.81284	3.480585
ADAMTS20	0.406534	0.022355	-6.48584	1.000305
MGME1	0.406534	0.022403	-6.03088	0.675309
HHIPL2	0.406534	0.022441	-6.20356	0.787302
ARL13A	0.406534	0.022456	4.764911	1.727964
USP32P1	0.406534	0.022488	-2.27997	4.744088
MIR744	0.406534	0.022553	-6.74807	1.229577
YJEFN3	0.406534	0.022554	-6.06935	0.713591
VAX2	0.406534	0.022558	-6.82548	1.292497
C20orf203	0.408499	0.022724	-3.32997	1.465261
LINC00200	0.408499	0.022729	4.553352	0.856809
SNORA77	0.40877	0.022806	-3.08607	2.615156
FAM86B2	0.40877	0.02282	-6.09403	0.729844
CAMK2N1	0.40877	0.022861	1.009224	7.462376
SLC46A2	0.40877	0.022882	2.238347	2.327167
PLCE1-AS1	0.40877	0.0229	2.536229	2.808029
LOC101928	0.41004	0.023002	1.422395	3.932767
PSD	0.410684	0.023087	5.573716	0.662186
ANP32B	0.410684	0.023109	1.126194	9.15486
MIR5585	0.410684	0.023132	4.429438	1.014865
HHEX	0.410719	0.023166	2.206308	2.685424
LINC00243	0.410728	0.023197	-6.26213	0.821962
CNDP1	0.411323	0.023262	2.197363	2.81939
TTC21B	0.412443	0.023357	-1.11136	6.075573
KLF12	0.413881	0.02347	-1.52195	5.548861
MRM2	0.414069	0.023512	-1.36965	4.996103
TCEANC2	0.416593	0.023687	1.034853	5.568677
HIVEP2	0.417137	0.023755	-1.10297	7.624765
NIPAL2	0.417137	0.023782	1.15434	5.476721
OSGIN2	0.417803	0.023852	-3.698	0.869397
MIA2	0.41839	0.024047	1.664061	4.41706
FHAD1	0.41839	0.02405	-2.88598	3.862252
KCNN1	0.41839	0.024067	-6.3306	0.891926
ATAD3C	0.41839	0.024087	-2.9358	3.16703
MKX-AS1	0.41839	0.024094	4.827448	1.7145
BORCS8	0.41839	0.024098	-2.13157	3.257751
SALRNA3	0.41839	0.024108	-6.1495	0.755931
SNORD58C	0.419225	0.024189	-4.65902	1.003675
PLN	0.420362	0.024286	-1.80502	5.172673
SLC22A23	0.420777	0.024414	1.904365	3.267865
PGGHG	0.420777	0.02443	-1.19926	6.856463
MAPK8IP1I	0.420777	0.02446	4.10192	1.545561
PAX4	0.420777	0.024488	3.913568	2.041062
RTCA	0.420777	0.024535	-6.25133	0.847866
LINC01424	0.420777	0.024546	-5.97716	0.627834

ZBTB7C	0.420777	0.024588	1.529616	6.103883
STEAP3-AS	0.420777	0.024593	1.652188	3.234486
BPGM	0.420777	0.024647	-6.12756	0.732593
TXNL4B	0.420777	0.024668	2.692812	2.247534
NHLH1	0.420777	0.024683	3.1674	2.238284
GIF	0.420777	0.024695	-5.15891	5.260707
TRAF5	0.421239	0.024754	-3.78874	0.947006
LINC01291	0.421381	0.024801	-6.1354	0.764735
GLS	0.421381	0.024902	-1.17879	6.996988
CHRM1	0.421381	0.024916	3.168219	2.127247
SHLD2	0.421381	0.024922	-1.19395	6.111142
RNA28SN5	0.421381	0.024923	1.147235	16.52052
ERRFI1	0.421986	0.025037	-1.07802	7.859715
NUP205	0.421986	0.025039	-1.21963	6.971436
NRSN2	0.421986	0.025072	-1.90862	3.570007
ATP8B5P	0.421986	0.025116	-6.27802	0.860556
LINC01270	0.421986	0.02512	-5.98868	0.655976
TRIM58	0.423359	0.025276	-4.09893	1.621332
PALD1	0.423359	0.025332	-1.65783	5.176205
SCML2	0.423359	0.025335	-5.84362	0.555719
HMGCS2	0.423359	0.025343	2.778174	2.730686
MYOM3	0.423359	0.025363	-2.37867	3.758623
STAU1	0.423449	0.025435	-4.13628	1.501525
FGF18	0.423449	0.025439	-3.52338	2.592187
LINC01614	0.423449	0.025465	-4.52185	0.837925
RHPN1	0.425411	0.025628	-1.99089	4.394368
FIRRE	0.425411	0.025648	1.827997	4.381917
SNORD61	0.428184	0.025863	-2.11942	4.108083
LOC101928	0.428184	0.02588	4.058925	0.768336
RRP15	0.429112	0.025975	-1.21583	5.521263
PFKFB2	0.429112	0.026002	1.036724	7.135198
PTGIR	0.430343	0.026155	-2.96936	2.10067
MIR8058	0.430343	0.026168	5.431546	1.098489
NEK4	0.430343	0.026181	-4.35942	0.774834
EN2	0.430343	0.026222	-4.48495	2.688664
GPR149	0.430343	0.026253	5.478972	1.114796
DDAH1	0.430343	0.026304	1.786761	3.687228
NPC1	0.430343	0.026324	-1.24788	6.816537
SYNE4	0.430343	0.026363	2.898861	0.900437
TMEM187	0.430343	0.026392	-2.63715	2.163624
HIST2H2BA	0.430343	0.026416	-2.63096	1.958069
IL17RD	0.430343	0.026437	-2.13174	4.154454
STX19	0.431759	0.026557	1.853296	3.922201
CRISPLD1	0.432381	0.026628	-3.87864	2.349101
RPS2P32	0.435633	0.02689	-4.74224	1.690467

SHISAL1	0.435633	0.026928	1.673014	3.625707
PTPN14	0.435633	0.026936	-1.11701	7.555806
FAM122C	0.435633	0.026961	-3.29685	2.164607
ODF3B	0.43621	0.02703	-1.64674	4.327086
SPTBN5	0.437564	0.027148	-2.36165	5.914936
COX15	0.437564	0.027216	-1.61872	3.968428
MIR6739	0.437564	0.027259	3.340776	2.129386
RAD51-AS1	0.437564	0.027314	1.188536	4.786522
CMTM3	0.437564	0.027323	-5.04235	1.227992
UGT1A6	0.437564	0.027328	2.15459	3.68838
MIR4531	0.437564	0.027348	4.663749	1.489873
FAM186B	0.438012	0.027424	-4.00261	1.539191
RAD52	0.438012	0.027442	-3.60134	1.37532
ZNF718	0.438365	0.027512	1.990341	2.50325
SESN2	0.438365	0.027605	1.197518	4.96723
CAP2	0.438365	0.027606	-2.01169	4.336979
ZG16B	0.438365	0.027641	2.277183	3.248357
CBY3	0.438365	0.027661	-6.49889	1.036
RBFADN	0.438365	0.027682	-5.71826	0.469371
PRR5L	0.438365	0.027699	-3.49805	2.150533
CEACAM22	0.43894	0.027768	-6.15117	0.760438
MX2	0.43894	0.02783	-1.4535	5.673136
LINC01531	0.43894	0.027835	4.217198	2.015632
TBC1D15	0.439133	0.027881	-6.14843	0.746949
LINC02288	0.439727	0.027952	-6.57966	1.094946
WIPF3	0.439833	0.027992	-2.26801	3.17872
S100A7A	0.439994	0.028036	-6.85347	1.301472
ZFPM1	0.440938	0.028145	1.127194	5.474485
SNORA11B	0.440938	0.028184	1.987619	3.918564
HSPA1A	0.440938	0.028197	2.17109	3.912331
WDR35	0.441047	0.028238	-5.84117	0.546183
C1orf52	0.441441	0.028327	4.493375	1.440136
CHRM3	0.441441	0.028353	-2.79658	3.214451
C2orf72	0.441441	0.028364	1.466927	5.717042
MSH3	0.443679	0.028569	1.056925	5.963558
UBE4A	0.443679	0.028637	5.408181	1.059783
CD55	0.443679	0.028641	-2.64424	3.880387
SMCO4	0.443679	0.028643	1.467245	4.207723
ACP7	0.444302	0.028742	3.683971	1.973284
PDGFRB	0.444302	0.028751	-1.08239	7.700764
ITFG1	0.445158	0.028858	2.001861	2.798332
C2orf48	0.445158	0.028874	-2.71622	2.317584
ADAM21	0.446565	0.028999	-3.10047	1.505832
LINC01226	0.446636	0.029042	-5.03043	2.958038
DZIP3	0.446636	0.029072	-1.1423	5.669489

CLASP1	0.448618	0.029238	2.326649	1.996886
AGAP9	0.448618	0.029315	1.198984	4.724507
WRN	0.448618	0.029331	-1.11729	6.754137
TFF1	0.448618	0.029338	2.207257	8.748134
MARS	0.45044	0.029522	-1.14602	6.594763
SPDYE5	0.45044	0.029553	-1.33156	4.846371
RASSF8	0.45044	0.02956	-3.88324	1.70364
BMP3	0.450852	0.029634	-6.8755	1.296838
SNORD47	0.450852	0.029656	-1.56934	5.968932
TMEM240	0.451439	0.02975	-4.84875	1.450808
COX4I1	0.451439	0.029816	0.952702	8.034601
FEM1B	0.451439	0.02983	-0.97821	6.94705
PDE7B	0.451439	0.029842	1.32944	4.235754
RAB44	0.451439	0.029867	2.425997	2.177946
AHNAK2	0.452186	0.029952	-1.54842	8.85278
IFIH1	0.452186	0.029985	-1.3036	5.824238
SNORD116	0.45361	0.030114	-2.54968	4.506875
AFF1	0.453827	0.030191	1.560966	3.435115
OTULINL	0.453827	0.030216	1.139636	5.757844
SHISA3	0.453827	0.030244	-4.53101	1.913244
CST2	0.453827	0.030269	-4.68898	1.53139
EBF3	0.453827	0.030301	-2.30402	3.631303
SLC35A3	0.455214	0.030429	2.509745	1.730497
MIR4298	0.45755	0.030664	-6.55671	1.067747
NELFCD	0.45755	0.030716	-1.14522	6.226827
CD38	0.45755	0.03072	-5.87216	0.566037
MUC12	0.45755	0.030745	1.838317	6.484544
FIP1L1	0.45755	0.030759	3.074122	1.909317
IPO9	0.458601	0.030865	-1.01041	7.349655
KNOP1	0.459088	0.030947	-1.16655	5.917893
COX6B2	0.459088	0.030967	-4.23156	2.325663
ANKS4B	0.459849	0.031054	1.573453	5.700186
LOC284865	0.460475	0.031131	2.419988	2.303114
S100A4	0.460706	0.031182	-5.97939	0.632345
FKBP1A	0.461305	0.03127	-3.47922	1.5326
PPP3R2	0.461305	0.031338	-6.21298	0.801572
THOC3	0.461305	0.031367	1.921531	3.75222
XKR4	0.461305	0.031369	2.743693	2.418821
LOC101928	0.461305	0.031425	-6.89696	1.349523
FUK	0.461305	0.031433	1.154102	4.88101
ITCH	0.461454	0.031495	-6.22872	0.824654
POLD2	0.461454	0.031514	-4.06793	1.154837
MGC70870	0.463391	0.031682	-2.54935	2.431008
SNORA63	0.466082	0.031901	-1.24565	7.761987
CYP2W1	0.466847	0.031989	2.088135	3.343

C1QL1	0.467854	0.032106	3.168698	1.18989
SNORD117	0.467854	0.032129	-2.63637	3.404068
BPIFB1	0.470188	0.032325	-3.47788	3.591702
GNG7	0.470506	0.032413	1.501466	4.316106
ATP13A5	0.470506	0.032466	3.380086	1.911716
LINC01209	0.470506	0.032538	-6.89571	1.346732
ID4	0.470506	0.032538	-2.20525	5.429789
SNORD87	0.470506	0.032548	-1.52097	9.01711
GPR26	0.470506	0.032562	-4.7284	1.646973
TMEM44	0.47111	0.03264	3.508091	1.908496
VN1R5	0.471694	0.032717	-6.8648	1.326011
GNA11	0.473738	0.03292	0.927572	7.200829
HID1-AS1	0.473738	0.032942	3.212744	1.438133
PLEKHH1	0.473738	0.033073	1.106515	6.569057
C1orf216	0.473738	0.033137	-1.44759	4.181829
ADCY5	0.473738	0.03314	-3.10647	2.525458
RFX8	0.473738	0.033171	-3.56855	2.215857
PAMR1	0.473738	0.033207	-7.11978	1.534834
ANAPC1P1	0.473738	0.033208	1.68642	2.753568
QKI	0.473738	0.033234	-2.83921	2.599894
MIR1343	0.473738	0.033255	-5.78014	0.508875
DLX6-AS1	0.473738	0.033256	-6.60265	1.112334
DOCK7	0.474401	0.033338	2.924669	1.693245
HNF1A-AS1	0.47497	0.033414	1.349665	5.541874
ISM1	0.476938	0.033622	-2.67201	3.593968
IL20RB	0.476938	0.033631	1.964972	3.435822
LOC101928	0.476938	0.033672	-6.11771	0.728915
CD163	0.476938	0.033698	-7.38024	1.755185
SMG7-AS1	0.479237	0.03392	2.133728	2.885683
SUCLG2	0.479237	0.033934	1.09989	5.637311
DCDC1	0.479637	0.034004	-4.40629	2.395714
LNX1	0.479637	0.034035	2.514142	2.408067
IFITM1	0.480059	0.034102	-1.25792	6.148534
NUGGC	0.480924	0.034222	1.880225	4.669862
PROSER2-A	0.480924	0.034237	-2.81359	2.725516
LOC101930	0.481131	0.034341	2.053453	2.623534
FAM156A	0.481131	0.03436	-6.2744	0.862085
MTERF1	0.481131	0.034361	-3.14685	1.56006
LIMD2	0.481376	0.034415	-1.74318	3.86846
MIR2116	0.481438	0.034467	-6.3079	0.87349
NRK	0.481438	0.034505	-3.63479	2.641906
UBE2W	0.481438	0.034593	2.006364	2.897545
VTN	0.481438	0.034608	-2.65445	1.920215
LINC00622	0.481438	0.034776	-5.9656	0.646161
ZNF385D	0.481438	0.034807	-3.27539	2.668522

BAMBI	0.481438	0.03483	-3.11977	5.568981
CMTM4	0.481438	0.034918	0.941268	6.412538
CLMN	0.481438	0.034918	0.997414	8.070477
CHI3L1	0.481438	0.034963	-2.69641	5.339921
MIR4453H(	0.481438	0.034982	1.284711	3.892623
PMF1	0.481438	0.034991	-3.88329	1.006879
MPP4	0.481438	0.035012	-5.25938	1.445533
MRPS17	0.481438	0.035026	-1.90143	4.334628
F5	0.481438	0.035046	1.358962	7.525064
PPARGC1B	0.481438	0.035071	3.561016	1.151951
IDO1	0.481438	0.035087	-3.15976	2.483372
LINC00643	0.481438	0.035143	-5.99109	0.6521
LOC729966	0.481438	0.035149	2.238001	3.078334
PGC	0.481438	0.035154	-3.7901	6.570229
CHTF8	0.483702	0.035391	2.39364	2.626972
CRLF3	0.483702	0.035393	-3.32461	0.980369
LOC100131	0.484244	0.035469	-4.20798	0.681688
SERPINH1	0.484904	0.035555	-6.17952	0.788969
COL12A1	0.485489	0.035638	-1.02397	8.055578
GPRIN1	0.485489	0.035701	1.724173	5.237818
BMP8A	0.485489	0.035718	-1.24446	4.850313
FAM222A	0.485489	0.035772	1.729421	3.300321
INHBB	0.485489	0.035783	-3.15192	5.427006
CEL	0.485914	0.035851	-3.7098	2.233704
8-Mar	0.48653	0.035967	1.79202	3.008505
CAPN8	0.48653	0.035971	1.101857	8.310494
PPIH	0.486785	0.036063	-1.33189	4.521157
ZNF680	0.486785	0.036078	-1.31566	4.766504
SLC7A11	0.486785	0.036101	-1.1318	5.629877
LINC01689	0.490008	0.036383	4.890917	0.954647
SYNRG	0.490008	0.036414	4.350842	1.277588
ARHGAP28	0.49119	0.03654	1.885159	3.986988
AHCYL2	0.49199	0.036727	2.080765	2.74331
ZFPM2-AS1	0.49199	0.036734	-2.90362	2.215388
TTC22	0.49199	0.036755	1.641885	3.973692
BAG3	0.49199	0.036798	-1.31275	6.435367
TMEM229I	0.49199	0.036806	1.834343	3.477546
GPR180	0.49199	0.036841	-1.09762	5.327307
THAP3	0.49199	0.036862	-3.83045	0.961759
QSOX1	0.492435	0.03694	1.140833	6.734502
SNORD23	0.492435	0.036982	-2.73458	2.242324
PTX3	0.492435	0.037008	-3.8778	3.251535
MEPCE	0.492632	0.03706	1.28333	3.918601
KCNS2	0.493061	0.03713	2.063357	2.951596
KCNA5	0.49431	0.03729	-4.25281	1.681288

AKR7A3	0.49431	0.037324	1.495475	5.30021
MBD1	0.49431	0.037364	-3.94952	1.52106
GJD3	0.49431	0.037376	1.249978	4.242125
CBLL1	0.49431	0.037472	4.219922	1.483981
LOC40070€	0.49431	0.037481	-4.69895	2.657112
NEURL1	0.49431	0.037488	1.612276	3.075153
GADL1	0.494378	0.037618	-3.45517	3.21969
PHGR1	0.494378	0.037634	1.993857	4.784547
SLC25A28	0.494378	0.03764	-1.11084	5.026002
BPIFA2	0.494378	0.037644	4.18702	0.845592
LTBP2	0.494605	0.037707	-1.07251	7.474731
SLC37A1	0.494605	0.037736	1.111704	6.923373
NAPG	0.495652	0.037939	-1.15185	6.486625
ANKRD20A	0.495652	0.037979	-3.67345	2.166405
ARL6IP1	0.495652	0.038026	1.405872	4.496801
HMGCL	0.495652	0.038083	3.039565	1.259804
STC2	0.495652	0.038134	-2.56175	2.970283
DMRTA1	0.495652	0.038167	-6.19785	0.79232
LOC100652	0.495652	0.038173	-1.97919	4.068177
COX11	0.495652	0.038185	-1.52283	4.044301
MAP2K6	0.495652	0.038191	1.312214	4.571916
KRTAP10-3	0.495652	0.038194	-6.40156	0.964528
LTN1	0.495791	0.038242	-0.94464	6.786752
NCF1B	0.496061	0.038314	-2.60332	2.405511
ZNF45	0.496061	0.038398	-1.28582	5.093481
DUXAP10	0.496061	0.038413	-4.51903	0.840812
HELZ2	0.496061	0.038415	-1.18453	5.166913
SPNS3	0.496178	0.038507	-3.75473	0.836002
SPINK13	0.496178	0.038524	-6.4428	0.979466
KANSL1-AS	0.496178	0.038537	-6.00511	0.653196
LINC00467	0.496919	0.038636	-2.31864	3.201145
FBXO30	0.496919	0.038696	-1.09954	6.167509
ZNF696	0.496919	0.03872	-1.50032	4.023905
LOC100272	0.496919	0.038775	2.57705	2.134207
DBI	0.496919	0.038784	-5.92654	0.610753
USP37	0.497972	0.038968	-1.04429	6.414562
PIMREG	0.497972	0.038975	-6.5336	1.063238
MIR4697H(	0.497972	0.03898	-1.46674	5.567209
SLC4A8	0.498138	0.039045	3.465758	1.070316
RHBDF2	0.498138	0.039105	-6.52959	1.062749
FZD8	0.498138	0.03915	-1.85097	3.022103
PARD6B	0.498138	0.039183	-1.11781	6.079918
PAXIP1	0.498138	0.039264	-1.11954	5.619534
SYNM	0.498138	0.039278	-2.54522	5.265733
TRPM4	0.498138	0.039337	1.936658	3.403869

HIST1H2AK	0.498138	0.03935	-2.37572	2.466825
OAS3	0.498138	0.039353	-1.012	7.099231
AGPAT4-IT	0.498138	0.039395	-2.21713	2.517736
PPP1R15A	0.498138	0.039411	-1.09244	8.241497
ADAM17	0.500019	0.039598	-1.09883	6.375918
ENTPD3	0.50007	0.03964	4.500113	1.363228
OMP	0.500291	0.039717	2.419961	2.325084
PKIB	0.500291	0.039733	3.773868	0.749112
CXCL3	0.501354	0.039863	-2.21753	4.263102
FOXCUT	0.501354	0.039894	2.949924	1.910643
SLC26A4	0.502666	0.040062	-4.38752	0.733091
SATB1-AS1	0.502666	0.040075	-3.9239	1.030592
ARFGAP3	0.50288	0.040164	3.583004	1.316139
FGF7	0.50288	0.040169	-1.62692	6.4763
SCARNA6	0.503215	0.040259	-1.23998	7.244438
PDK4	0.503215	0.040315	-2.39281	10.80085
MIS12	0.503215	0.040442	2.576897	1.934944
RAD21-AS1	0.503215	0.040466	-5.71925	0.475216
HIP1R	0.503215	0.040468	1.02095	6.097182
UNC119B	0.503215	0.040509	0.98221	5.899022
MCTS2P	0.503215	0.040529	1.358812	4.489599
ZNF876P	0.503215	0.04054	-2.31567	2.389351
ZNF518B	0.503215	0.040577	-1.54169	4.99234
USP17L15	0.503215	0.040579	-5.78189	1.841585
BAZ1A	0.503734	0.040664	2.887063	1.499905
SNORD63	0.503734	0.040733	-1.86293	4.964256
TSPAN7	0.503734	0.040736	2.062179	3.127442
MCM6	0.504642	0.040848	-0.99403	5.995798
DUOXA2	0.505105	0.040924	2.495701	2.919463
GHSR	0.505216	0.040972	-6.00835	0.667168
CCDC13	0.507746	0.041216	2.50534	2.440276
NPHS1	0.507898	0.041267	-4.20056	0.661896
LOC646471	0.508109	0.041323	1.363895	4.10891
SNORD79	0.508251	0.041373	-1.91522	3.655173
ACSS1	0.509383	0.04158	3.211408	1.218227
C6orf222	0.509383	0.041584	-1.80673	4.719254
LIPN	0.509383	0.041618	-4.52762	0.897581
NOL7	0.509383	0.04162	-1.92826	2.663004
GAS1RR	0.509448	0.041664	-6.36463	0.918081
MKRN2OS	0.513401	0.042027	1.78806	3.001331
SGCE	0.516101	0.042287	-6.19762	0.802972
TTYH3	0.516374	0.042358	-0.99652	7.228944
CHD5	0.516374	0.042388	-3.58779	2.944957
AGFG2	0.518854	0.042711	1.076403	5.564534
PI15	0.518854	0.042805	-2.12664	5.394244

LOC10272:	0.518854	0.042815	3.609244	0.954427
TNFRSF12A	0.518854	0.042868	-1.51265	5.404865
LINC00310	0.518854	0.042883	-3.2392	0.905311
CCND2-AS1	0.518854	0.042893	4.222636	2.43291
LINC01637	0.518854	0.042906	2.228029	2.21124
LOC102724	0.518854	0.042908	-2.88792	1.9151
CPXM2	0.519069	0.043002	-1.88915	5.371483
ARHGEF17	0.519069	0.043005	-1.05677	6.479295
LSG1	0.519491	0.04313	-0.96617	6.332495
GGNBP2	0.519491	0.043161	1.811311	3.589936
ELMO3	0.519491	0.04319	1.160626	5.277392
NYAP2	0.519491	0.043199	-3.77827	0.922773
ITPA	0.520295	0.043344	-6.25234	0.815778
ARHGEF16	0.520295	0.043345	1.02123	6.319164
LOC101927	0.521622	0.043534	-2.89312	2.100367
NT5C1A	0.521622	0.043545	-7.11098	1.526915
FAM214A	0.521622	0.043575	1.769758	3.034328
PRKY	0.52218	0.043714	1.752287	5.834536
ITIH3	0.52218	0.043724	-3.76075	1.599668
PGAM2	0.52218	0.043741	-1.68944	3.457104
PIK3CA	0.525582	0.044066	-1.0095	6.165165
ANTXR1	0.52562	0.044109	-1.29046	7.98518
PROX1	0.526184	0.044196	-2.37699	4.830991
STARD3NL	0.526206	0.044303	-1.30146	4.5695
HSD17B14	0.526206	0.044322	-2.22373	3.371911
ZC3H12A	0.526206	0.044391	-1.15969	5.988511
RAB21	0.526206	0.044496	-1.12463	7.879811
DMAC1	0.526206	0.044545	1.312635	3.999995
TYSND1	0.526206	0.044549	-2.41859	2.249721
CHAF1B	0.526206	0.044598	-1.59294	3.794192
BMPR1A	0.526206	0.044605	-1.03978	5.873655
METTL4	0.526206	0.044628	3.154921	2.420584
2-Sep	0.526206	0.044632	1.58511	3.575658
EAF1	0.526206	0.044639	-0.99281	6.058122
SNORA87	0.526646	0.044717	1.955702	3.354417
RHBDF1	0.527165	0.044801	-1.17554	5.663968
PICSAR	0.527199	0.044844	-6.37404	0.945578
SH2D3C	0.52859	0.045022	2.011376	2.569744
CDKL2	0.52859	0.045074	-3.09308	1.686498
MAK16	0.52859	0.045083	-1.08499	5.490614
PM20D2	0.529791	0.045226	-1.00508	6.105736
FAR2P1	0.5312	0.045409	-4.07043	3.066875
MTERF4	0.5312	0.045443	1.897257	2.645865
NRN1	0.5312	0.045468	4.24663	0.814799
EPHA3	0.531621	0.045544	-2.21893	3.874694

PRKRA	0.531779	0.045598	-3.4318	0.871689
PADI2	0.53228	0.045682	1.523719	6.036582
PRKCI	0.534157	0.045916	-1.01908	7.602736
LCAL1	0.534157	0.045924	-4.19709	0.918786
LOC105375	0.534168	0.045966	2.10762	2.950182
GART	0.53573	0.046141	-3.54343	1.133111
BHLHE40	0.536313	0.046379	-1.07955	7.727667
KLHL15	0.536313	0.04644	-1.0785	4.916485
HUNK	0.536313	0.046457	-2.19822	4.394275
ZNF460	0.536313	0.046464	-0.88372	7.490008
RFNG	0.536313	0.046489	-1.29647	3.898394
RUNX1	0.536313	0.046521	-2.0601	3.911704
MIR5579	0.536313	0.046526	-5.94399	0.621414
CYP51A1	0.536313	0.046555	1.64995	3.780659
THBS3	0.536313	0.046606	-3.93604	1.532104
RPL13P5	0.536313	0.046649	-2.84436	1.491587
BTNL3	0.536313	0.046661	2.145163	4.831713
LINC01273	0.536313	0.046694	-3.27158	0.940265
HECTD2	0.536313	0.046723	-3.11547	1.988889
KIF11	0.537935	0.046906	-1.22005	5.95786
RNF5	0.537935	0.046946	2.058397	3.241378
TECTA	0.538029	0.046996	-2.83002	2.057209
FOXA3	0.538057	0.047087	1.358185	4.002072
FER1L6-AS:	0.538057	0.047118	3.316277	1.987665
LOC729732	0.538057	0.047132	1.006186	5.251443
SRP68	0.538057	0.047162	2.045353	2.604353
ASXL3	0.538632	0.047254	-2.05795	4.671332
MOB1B	0.53888	0.047333	-2.56245	2.177517
PPA2	0.53888	0.047357	-6.01849	0.669197
VIRMA	0.538905	0.047441	-1.59886	5.320466
AGMAT	0.538905	0.047442	1.229427	4.322574
FRMD8P1	0.539746	0.047635	-6.5675	1.087074
LINC01671	0.539746	0.047647	-6.13236	0.759158
LINC00840	0.539746	0.047682	-6.88521	1.338919
NPIPB11	0.539746	0.04769	-1.42982	4.335168
MRPS25	0.539746	0.047721	0.85731	6.872864
AOC4P	0.539881	0.047775	-3.65939	1.638996
METTL2B	0.543122	0.04821	-1.2042	4.86495
OXTR	0.543122	0.048299	-2.72686	4.061024
DUSP4	0.543122	0.048346	-1.41727	5.270952
CEP126	0.543122	0.048367	-1.52635	4.923526
GORASP2	0.543122	0.048383	-3.09332	1.616038
ZNF532	0.543122	0.048427	-1.00128	6.751724
DDX43	0.543122	0.048429	1.98909	3.13231
THSD4	0.543122	0.048484	1.23999	4.355733

LINC01022	0.543122	0.048495	3.337296	1.035782
KRTAP10-1	0.543122	0.04854	-7.46717	1.83532
PLSCR1	0.543122	0.048561	-1.09125	6.784177
MARCO	0.543122	0.048575	-3.35725	2.395075
MIR4637	0.543122	0.0486	-6.32396	0.90127
IER2	0.543125	0.048641	-1.02729	6.945789
SNORD116	0.544973	0.048848	-2.15811	4.595851
IL1RL1	0.545047	0.048896	1.704663	2.450065
UTP11	0.545205	0.048964	-1.04294	5.442346
CRNN	0.545205	0.048999	4.240553	7.810971
RPL22L1	0.545205	0.049035	-1.79513	5.42122
COL4A3	0.546027	0.04917	-1.20095	4.742483
MORC2	0.546027	0.049192	-2.1033	2.97988
TEX12	0.546282	0.049257	-6.10456	0.711468
SMC4	0.546865	0.049423	-4.30138	0.68566
GLTP	0.546865	0.049494	1.216594	6.112646
MIR548U	0.546865	0.049508	-2.97971	1.428015
LMTK3	0.546865	0.049517	-1.84131	3.877324
MIR5009	0.546865	0.04954	-7.09867	1.51831
AKR1C3	0.546865	0.04956	2.438473	2.506017
DEFB1	0.547355	0.049646	2.266408	2.10906
ATG3	0.547637	0.049773	-3.2656	0.936917
DUSP2	0.547637	0.049784	-2.25357	3.044972
CHMP6	0.547637	0.049797	1.009305	4.638811
MFSD3	0.547815	0.049854	-6.28294	0.873073
LOC403323	0.548186	0.049955	-3.45208	0.635101
PRKAA1	0.548186	0.049992	-3.55847	1.145223
MAPRE1	0.548186	0.050014	-0.87908	7.351369
CSGALNAC	0.549016	0.050159	-1.01923	6.827285
UQCRFS1	0.549016	0.050201	0.935745	5.754192
MYOCD	0.549016	0.050215	-6.34168	0.914687
LOC100507	0.549106	0.050265	2.671194	1.916104
WWC2	0.549785	0.050369	-1.02478	6.559477
SRSF10	0.549858	0.050437	-1.32427	4.913221
SHISA2	0.549858	0.050518	-3.81806	1.335483
NR4A3	0.549858	0.050525	-6.90814	1.351466
SNORD119	0.549858	0.050543	-1.45707	6.207393
GIPC3	0.550309	0.050627	1.564978	3.245681
NEK7	0.550587	0.050731	-1.1033	6.799028
LOC101927	0.550587	0.050842	-7.06653	1.491688
MGAT4B	0.550587	0.050926	1.15162	4.313322
TNNC2	0.550587	0.050942	-3.01655	1.613483
AMD1	0.550587	0.050965	3.457205	1.432439
MIR194-2H	0.550587	0.050985	1.163203	5.039866
CBX4	0.550587	0.051041	-0.90443	6.183076

SPOCK1	0.550587	0.051044	-1.89069	4.427277
IL23A	0.550587	0.051064	-3.32414	2.409282
TAL1	0.550587	0.051093	-6.00416	0.664763
SKINT1L	0.550587	0.051114	-3.88983	1.701836
NAMPT	0.550694	0.051166	-1.2849	8.219292
KBTBD11	0.551347	0.051269	-3.95247	2.194646
ASAP2	0.55242	0.05141	1.911896	2.542248
HPGDS	0.553288	0.051543	2.113605	2.493588
MAT2B	0.553288	0.051576	1.927905	3.386863
ADCY3	0.554322	0.05175	-1.03369	5.864298
CISD3	0.554322	0.051756	2.375955	2.255231
LOC101928	0.554755	0.051868	1.352526	3.65862
SBSPON	0.554755	0.051881	-2.6736	4.630338
ATP4B	0.556756	0.052111	-4.8498	4.193924
MGC32805	0.557283	0.052203	2.715397	1.916099
GOLM1	0.557471	0.052266	2.436702	2.582365
ABCC2	0.557471	0.052369	-3.04719	5.065971
LOC101928	0.557471	0.052374	-1.9702	3.246605
PPP1R1B	0.557471	0.05239	1.743088	5.046769
SNTB1	0.557527	0.052438	-1.10593	5.401576
C2orf27A	0.557719	0.052512	-3.61576	1.177563
CCND1	0.557719	0.052541	-1.19896	9.281006
CXCL10	0.557917	0.052637	-2.44854	2.752213
SMCHD1	0.557917	0.052645	-1.15974	9.028626
EPC2	0.558232	0.052784	-0.96623	6.259439
KLHL29	0.558232	0.052808	-1.73905	4.489222
HIST2H3D	0.558232	0.052844	-3.38311	0.619779
KANSL2	0.558232	0.052845	-1.12782	4.889752
SFRP1	0.559314	0.05299	-1.70059	5.316204
RAE1	0.559867	0.053085	-3.50863	1.097271
LOC100506	0.560068	0.053147	1.313883	4.139234
SNORD27	0.560486	0.053253	-1.26589	8.37151
SYT7	0.560486	0.053272	-5.96464	0.61881
TBCC	0.561301	0.053476	-1.21599	4.728724
KRTAP10-9	0.561301	0.053597	-5.35707	1.587403
EVPL	0.561301	0.053633	1.347042	8.074892
PROKR1	0.561301	0.053663	3.994189	1.489522
TGM3	0.561301	0.053671	3.868973	6.232396
C2CD4A	0.561301	0.053764	1.829948	3.246874
UTY	0.561301	0.053766	2.228257	2.554645
SAT1	0.561301	0.053814	-1.07166	6.447514
SEMA6A-A:	0.561301	0.053839	2.561244	2.041244
MRPL39	0.561301	0.05385	-4.07151	0.824125
NPSR1-AS1	0.561301	0.053925	-7.35993	1.74374
AATBC	0.561301	0.053927	-2.03746	3.560182

PTGES	0.561301	0.053927	-2.87167	3.464136
RNF181	0.561301	0.053995	-1.75015	3.858512
CLDN7	0.561301	0.054009	2.250406	1.97015
KRTAP10-1	0.561301	0.054034	-7.35196	1.736968
PRSS41	0.561518	0.054118	-6.87113	1.331203
SNORD102	0.561518	0.054162	-1.33742	5.64109
TRAF2	0.561518	0.054187	-1.05061	5.075838
ZNF483	0.561518	0.054236	-1.66826	4.358724
ZNRF3	0.561518	0.054269	-4.43406	0.842965
MIR3188	0.562205	0.054384	3.845341	1.907464
KRT19	0.562205	0.054486	1.13478	10.29991
UNC13B	0.562205	0.054514	0.868316	7.429709
ST3GAL3	0.562205	0.054542	1.770073	2.599289
SIK1	0.562205	0.054549	-1.64014	5.122217
CNPPD1	0.562638	0.054636	0.854907	6.220721
SLC40A1	0.562638	0.05468	0.971239	7.692422
RTTN	0.562638	0.054733	-1.09094	5.351739
POP4	0.562638	0.054797	-3.11015	0.999023
ZNF222	0.562638	0.054843	3.808879	1.792031
CHN2	0.562638	0.054894	1.449582	3.231678
CALHM6	0.562638	0.054925	-6.1144	0.74042
LINC02210	0.562638	0.05496	2.317117	2.223084
TRIM4	0.562638	0.054977	2.403385	1.920836
AMPD2	0.565043	0.05533	1.71477	2.665249
UGDH-AS1	0.565043	0.055339	1.592204	3.542483
ODF2L	0.565043	0.055341	3.643598	1.28639
MSX2	0.565421	0.055434	-1.96706	4.075713
SIRT5	0.565421	0.055496	-3.07356	2.071149
PCSK1N	0.565421	0.055508	-4.22133	1.978108
MAATS1	0.567141	0.05572	-2.18138	3.390425
CA5B	0.567897	0.055837	-1.37076	4.272683
TSACC	0.568493	0.055943	-4.30156	1.022597
ERV3-1	0.568493	0.055983	-1.38095	6.264461
KCND2	0.568923	0.05615	-3.15421	1.454061
HSPA6	0.568923	0.056183	-2.45966	3.645076
ROBO3	0.568923	0.056217	-1.9298	3.841923
LOC285889	0.568923	0.056268	2.360712	1.682823
S100P	0.568923	0.056277	1.256948	6.510505
DOP1B	0.568923	0.056285	0.922151	7.180575
CBX8	0.56919	0.056355	-1.54212	3.662964
GLS2	0.571046	0.056615	-6.10093	0.741154
SPEG	0.571046	0.056626	-1.52001	6.263916
SLC46A1	0.571273	0.056767	-3.91549	1.029304
PATE3	0.571273	0.056861	-5.92913	0.598256
B3GNT7	0.571273	0.056945	0.990406	5.696888

TEDC1	0.571273	0.056955	2.766891	1.057066
ABCC6P1	0.571273	0.056961	1.766315	3.368273
PRDM6	0.571273	0.056979	-2.69466	2.182817
ERCC5	0.571273	0.057003	1.425715	3.63656
VTRNA1-2	0.571273	0.057057	1.440716	8.786555
LGALS3	0.571273	0.057071	1.10679	4.943909
GAL3ST1	0.571273	0.057099	1.899388	2.317675
ITGA10	0.571273	0.057127	2.807654	1.210505
AMH	0.571308	0.057184	-1.96948	3.616551
PCDHB10	0.571308	0.057218	-2.08916	2.790896
BARX2	0.571383	0.057299	1.480991	4.749675
NFYC-AS1	0.571383	0.057328	-1.57746	3.278123
HRH1	0.571383	0.057356	-3.4961	1.372035
TMEM30B	0.571434	0.057476	1.103873	6.24262
SDC4	0.571434	0.057608	-0.90816	8.313768
MOXD1	0.571434	0.057609	-2.34826	4.91393
SLC16A9	0.571434	0.057633	1.522702	3.868442
DPP4	0.571434	0.057656	1.47713	7.060631
GRID2IP	0.571434	0.057662	-1.87716	3.901553
ANXA4	0.571434	0.057688	0.833339	8.029606
MUC5AC	0.571434	0.057718	2.077473	13.55864
ZNF282	0.571434	0.057753	2.650225	1.220608
CENPF	0.571952	0.057849	-1.10415	8.238462
TDGF1P3	0.572696	0.057968	-4.12857	1.192287
MKI67	0.574661	0.058211	-1.35561	4.467463
FADS1	0.574863	0.058275	-1.32462	5.385089
SMC3	0.574943	0.058389	-0.93142	7.68643
CDPF1	0.574943	0.058391	1.803901	3.02259
RNF216P1	0.574943	0.058415	-2.18214	2.275342
C3P1	0.579361	0.058937	2.577512	2.379198
INTU	0.579361	0.058952	-1.04997	4.961338
SLC12A6	0.580637	0.059126	1.696944	3.055396
CDHR1	0.581216	0.059253	2.716279	2.165808
KCNJ18	0.581216	0.059282	-7.25008	1.645203
FCN1	0.581216	0.059318	1.468342	3.396012
BNC2	0.581439	0.059385	-3.17048	1.893596
ICE1	0.581491	0.059434	-0.81912	7.009913
PI4KAP1	0.581911	0.059522	-1.25309	4.965565
F2RL2	0.582022	0.059615	-6.75785	1.238547
B4GALT1	0.582022	0.059622	-1.11332	7.955016
ESRRB	0.583234	0.059805	-5.17006	1.402003
ITGAX	0.583234	0.059835	-2.64268	2.131397
ACBD4	0.583505	0.059907	-3.65798	0.800039
HDAC4	0.583534	0.059972	-1.52547	7.74143
DNM3OS	0.583534	0.060028	-2.09466	4.273034

ATP10A	0.583534	0.060054	-1.46407	4.998521
CDKN2A	0.583534	0.060088	2.026745	3.533949
FSIP2-AS2	0.583699	0.06015	-6.66421	1.161472
AHR	0.585028	0.060354	-0.96344	8.404076
TONSL-AS1	0.585028	0.060439	1.615936	2.575008
FAM84A	0.585028	0.060442	1.37016	6.58005
MMP7	0.585028	0.06049	-2.3691	4.86338
SNORA14B	0.585028	0.060526	-1.19045	7.768247
NKILA	0.585028	0.060554	-2.75537	1.657066
TMEM108	0.585836	0.060682	3.784613	0.926013
CXCL16	0.58623	0.060811	-1.39297	4.770025
RBM48	0.58623	0.060832	-1.19297	4.68524
FABP3	0.58623	0.060857	-2.21401	3.304033
PLCG1-AS1	0.58706	0.061083	1.929596	2.904652
SCIN	0.58706	0.061101	2.075233	2.443427
PTENP1	0.58706	0.061126	1.159919	4.707375
SENP8	0.58706	0.061127	2.94237	1.422195
COTL1	0.58706	0.061167	0.834783	7.710527
CSTB	0.587402	0.061247	1.693279	8.27063
SPATA6L	0.587422	0.061294	1.104402	4.784406
ERMP1	0.588512	0.061453	0.844599	6.5123
ZSWIM5	0.588704	0.061561	1.09503	4.776274
ORM2	0.588704	0.061563	-3.26552	2.268098
RNMT	0.58965	0.061706	-1.64305	4.362638
ZNF692	0.589971	0.061785	-3.75819	0.919346
ADI1	0.590341	0.061975	1.204272	4.228481
BMP6	0.590341	0.061984	1.497699	3.533565
HIST1H2BN	0.590341	0.061989	-1.49025	4.022147
PLXNC1	0.590341	0.062042	-1.03615	5.166452
SNORD89	0.590341	0.06206	-1.34996	9.109841
TMPRSS2	0.590341	0.062225	2.226497	1.590625
FAM185A	0.590341	0.062231	2.540577	1.226111
ATP1B1	0.590341	0.062237	0.847311	9.476911
VN1R1	0.590341	0.062241	-2.24308	2.852013
USP38	0.590341	0.062274	3.871094	1.656218
PDLIM7	0.592629	0.06256	-2.01274	3.689326
DHCR24	0.592687	0.062651	0.97105	7.003005
RPS27	0.592687	0.062812	-1.03699	7.306769
ESYT2	0.592687	0.062819	-0.84154	8.249289
DZANK1	0.592687	0.062838	-1.74622	3.048799
USP12	0.592687	0.062853	-0.89577	6.687661
MIR635	0.592687	0.06288	2.749548	1.962554
ANKRD34B	0.592687	0.062883	-6.05927	0.703514
PRDM11	0.593419	0.063006	-1.63516	4.277613
ULBP2	0.593587	0.063145	-3.93681	1.02943

HERC2P7	0.593587	0.063184	1.114771	4.344637
DIS3L2	0.593587	0.063233	-2.14536	2.761839
LYNX1	0.593587	0.063249	-3.986	0.525392
MKKS	0.593587	0.06325	2.108714	2.58068
RNF150	0.594072	0.063347	-1.99355	3.857462
EPPK1	0.594913	0.063482	-0.94327	7.917239
JPH2	0.595086	0.063545	-1.95204	4.906662
MIR221	0.595268	0.06361	-2.63891	1.956235
FAM174B	0.596566	0.063861	1.093648	4.383714
TYMSOS	0.596566	0.063865	2.979716	1.405614
DUXAP9	0.596566	0.063937	-1.65284	5.322581
S100A3	0.596566	0.06398	-3.38055	1.003601
MRPS36	0.596566	0.064017	1.86396	2.606979
ST3GAL5-A	0.596566	0.064022	3.445328	1.177545
PPAN	0.597337	0.06415	-4.22836	0.690894
SPINK1	0.597362	0.064229	-1.67653	5.468162
HLA-DQB1-	0.597362	0.064244	-2.22624	2.426941
RPL23AP32	0.598042	0.064456	-3.41557	1.695425
FGF3	0.598042	0.064464	-4.43494	3.613892
GVINP1	0.598042	0.064478	1.011682	5.555486
GKN1	0.598042	0.064521	-4.97835	7.11913
FKBP9	0.598042	0.064545	-4.21211	0.956016
SNORD3D	0.598253	0.064656	-1.62025	6.194933
FAM83D	0.598253	0.064659	-1.76402	5.709894
NBR1	0.598367	0.064754	2.722384	1.69253
PGM5P2	0.598367	0.064762	-1.37779	4.564429
RGS16	0.598715	0.06495	-2.1088	3.603546
FLJ13224	0.598715	0.06497	1.651992	3.076214
PCSK6-AS1	0.598715	0.065039	-3.13462	1.992527
SNORD8	0.598715	0.065098	-1.41073	8.985506
BRI3BP	0.598715	0.065113	1.277661	4.270664
NBPF14	0.598715	0.065125	0.929894	5.074373
TENT5C	0.598715	0.065174	1.110172	7.619979
KLK1	0.598715	0.065181	1.70978	3.617551
ABCG5	0.598715	0.065252	2.78038	1.43682
APOB	0.598715	0.065259	2.233883	4.030875
MIR365B	0.598715	0.065345	-5.88807	0.587211
MIR7845	0.598715	0.065348	3.318732	1.352566
HIST1H4D	0.599211	0.065473	-1.69864	3.834596
IFNGR1	0.599211	0.065545	-0.86283	7.138492
LOC100287	0.599211	0.065563	-3.3525	1.180736
CYP51A1-A	0.599211	0.065593	3.309332	1.39122
KRT18	0.599211	0.065666	3.491816	1.466139
IMPA1	0.599211	0.065676	2.637867	0.853916
HSBP1L1	0.599445	0.065747	1.221058	3.887457

TTC39B	0.600159	0.065939	-3.9328	2.234106
TMEM132I	0.600159	0.065984	-3.05071	2.225932
LINC00526	0.600159	0.066087	2.988575	1.19631
MAPKAPK2	0.600159	0.066122	-2.71888	1.999452
YIPF6	0.600159	0.066217	3.9638	1.015454
SLC9A2	0.600159	0.066229	1.391527	5.665067
TSPEAR-AS	0.600159	0.066248	-2.78141	3.922658
TAS2R4	0.600159	0.066258	-2.86592	1.766097
FAM86C1	0.600159	0.066276	-4.21299	0.683082
LOC100505	0.600159	0.066285	1.109462	4.62518
LEPROT	0.600159	0.066329	-2.24044	2.618818
RABGGTB	0.600809	0.066467	1.732207	2.934699
FAM83C	0.600809	0.066492	2.260002	3.188009
SLF1	0.601045	0.066588	-1.28621	4.420227
NPTX2	0.601045	0.066655	-2.98752	2.790893
RAB11FIP1	0.601045	0.066656	-1.01654	5.867661
LOC105379	0.601121	0.06671	3.129384	1.395124
MAPK8IP2	0.601527	0.066877	-2.75031	1.767818
EFCC1	0.601527	0.066899	1.282857	3.770707
MICAL3	0.601527	0.066906	0.77601	7.066126
NR2F6	0.601527	0.066938	0.981529	5.694586
ANXA2P1	0.602659	0.06714	1.951771	3.416097
FXYD3	0.602659	0.067189	1.556153	3.484584
FBP2	0.602659	0.067202	-3.56593	1.231745
CMTM7	0.602771	0.06726	3.111671	1.03435
ALLC	0.603138	0.067471	-4.52109	0.847641
CDC42BPA	0.603138	0.067489	-1.2275	4.10214
HSPB8	0.603138	0.067505	-2.09098	4.918096
ECHS1	0.603138	0.067537	0.898972	6.145357
SCUBE2	0.603138	0.06754	2.776732	1.598113
ADAM15	0.603138	0.067586	3.079656	0.996052
MIR4441	0.603138	0.067647	-2.1772	2.832251
IGSF23	0.603138	0.067669	-5.74503	0.490123
ST20-AS1	0.604534	0.067882	1.87431	2.31086
PNMT	0.604534	0.067918	-6.13721	0.765227
FBXO45	0.605253	0.068045	-1.17144	5.367028
MOB1A	0.606954	0.068282	-3.85511	0.465355
SLC7A11-A	0.607168	0.068389	-1.50877	3.281765
RPL23AP64	0.607168	0.0685	-2.45874	2.757049
FSD1	0.607168	0.068543	3.155113	0.990173
TRIM16L	0.607168	0.068559	1.348043	3.848728
ITPK1-AS1	0.607168	0.06859	1.106498	4.885698
RFTN2	0.607168	0.068593	1.294203	3.978552
DNAL4	0.607168	0.068676	1.230693	3.739579
ELP4	0.607168	0.068677	-3.78734	0.935563

ARMC9	0.608045	0.068891	-1.19566	4.957541
CELF3	0.608045	0.069006	-7.08871	1.510335
GLIS1	0.608045	0.069023	2.054263	2.425668
FBXO17	0.608045	0.069042	-4.33784	0.773346
GPC1	0.608045	0.06909	-0.93503	5.892485
CEBPA-DT	0.608045	0.0691	1.808329	2.563973
GMDS	0.608045	0.0691	1.634244	3.5805
BUD31	0.608184	0.069172	-0.94544	5.513399
GSKIP	0.608184	0.069218	1.832675	2.9958
BNIP2	0.608184	0.069255	-0.88121	6.577107
HELB	0.60821	0.069304	-1.10785	5.211954
HOXB4	0.609597	0.069509	-1.21023	5.250993
STYK1	0.610286	0.069634	1.202087	4.380933
DMAC2L	0.61056	0.069753	2.246331	1.574412
B3GNT6	0.61056	0.069769	2.08495	3.129233
TOPBP1	0.61056	0.069837	-0.83856	6.709283
DIRC1	0.61056	0.069899	-6.43401	0.942981
GTF2F2	0.61056	0.069939	-1.03917	5.202518
FLNB	0.61056	0.069945	-2.56167	2.22553
WDR78	0.610898	0.07003	-3.29433	0.946889
HSPA12A	0.611493	0.070232	-1.8369	4.791353
CRYBG1	0.611493	0.070256	0.888826	7.782922
FSTL3	0.611493	0.070272	-1.54699	5.427751
ZNF280C	0.611493	0.070368	-1.1285	4.533843
MGAT4A	0.611493	0.07037	1.005482	5.551025
FAM30A	0.611493	0.070378	1.341313	5.249203
TAS2R3	0.613075	0.070606	-2.79769	1.592976
BCO1	0.614519	0.07082	1.597683	3.711856
GMPS	0.614967	0.070918	-0.94191	6.453741
PCP4	0.615454	0.071131	-3.35432	4.694788
TMEM184/	0.615454	0.071155	0.983943	6.537791
TPD52	0.615454	0.071158	2.739225	1.639519
EVPLL	0.615454	0.071162	2.428039	0.752572
UTP23	0.616249	0.071301	-0.98835	5.219069
MRPL13	0.616985	0.071433	-1.2371	4.021586
POLR3B	0.618896	0.07173	-3.98866	1.04942
NBL1	0.618896	0.071776	1.239994	3.499393
SLED1	0.618896	0.071796	-1.80766	2.469234
SRMS	0.619604	0.071925	-1.33279	4.372307
SIPA1L3	0.619963	0.072018	0.786454	8.044303
SNORD12C	0.619963	0.072081	-1.27911	5.416871
RRP1B	0.619963	0.07211	-0.92653	6.441329
FAM129B	0.619963	0.072156	1.578041	3.131337
FXYD7	0.620554	0.072291	-3.43264	1.939648
KSR2	0.620554	0.072319	1.935573	2.764738

ZNF286B	0.620848	0.072441	1.550869	3.311145
LOC38781(	0.620848	0.072498	-3.31202	0.60263
PMEPA1	0.620848	0.072537	-2.661	2.996565
GRAMD4	0.620848	0.072543	0.801336	7.01947
MARCKSL1	0.621751	0.072696	2.135754	1.936836
POLQ	0.622055	0.072778	-1.04446	5.657439
ZNF296	0.622598	0.072889	-2.24518	2.451246
TPRKB	0.623118	0.072998	-1.6698	4.0525
SNAPC1	0.623628	0.073105	-1.60528	4.755102
ZKSCAN3	0.624585	0.073265	-3.4435	0.972782
TOMM20	0.625061	0.073368	-0.86951	7.595793
CYP2C18	0.62597	0.073523	2.771503	1.211678
DBNL	0.626129	0.073589	3.867201	1.401648
LOC10013‍1	0.626238	0.073757	-2.62461	2.054438
NR5A2	0.626238	0.073836	3.122826	1.27603
VWA5B2	0.626238	0.073859	2.023289	1.64865
KRTAP10-8	0.626238	0.073874	-7.00061	1.444045
ZNF573	0.626238	0.073901	-3.61415	1.221972
H2AFY2	0.626238	0.073909	-1.99695	3.757204
MSX1	0.626238	0.073948	-3.87841	1.527728
CLDN23	0.626238	0.074015	1.219837	4.796974
LIPE-AS1	0.626238	0.074032	-2.97707	1.004131
PDE4D	0.62639	0.074136	0.852879	5.249124
CA2	0.62639	0.074145	3.031031	1.441017
MAT1A	0.626532	0.07421	-5.99337	0.669414
LINC02563	0.627453	0.0744	-6.49674	1.043915
ZNF569	0.627453	0.074436	-2.18341	1.892354
RASL10B	0.627453	0.074462	-2.75718	1.705865
KCNF1	0.627549	0.074522	3.070422	1.953731
LINC00239	0.627549	0.074648	-4.45574	1.176683
FAM120C	0.627549	0.074681	2.504153	1.639257
TICAM1	0.627549	0.074722	-1.08625	5.145624
SNORA5A	0.627549	0.074761	-1.08804	5.437841
LRBA	0.627549	0.074826	2.974521	1.299347
TAF8	0.627549	0.074844	-0.91168	5.516817
USP27X	0.627549	0.07487	-1.49421	3.18645
NRAS	0.627549	0.074907	-1.2631	7.25733
PIK3CG	0.627549	0.074952	3.230076	1.631059
ATOH8	0.627847	0.075038	1.292721	3.403794
SYNPO	0.627847	0.075142	-0.97533	6.094893
LINC00619	0.627847	0.075187	-6.25857	0.855599
SFXN3	0.627847	0.075222	-0.87658	6.129314
SUMO1P1	0.627847	0.07525	-6.2565	0.84367
BAIAP2L2	0.627847	0.075275	1.057113	6.643413
ME3	0.628152	0.075359	3.170349	1.094586

CTDSPL	0.628836	0.075531	-6.22729	0.819085
STK32C	0.628836	0.075537	-1.66774	3.552646
RBFOX3	0.629783	0.075752	-2.09399	2.523775
SALL4	0.629783	0.075804	-2.58151	2.61035
PSMD14	0.629783	0.075807	-1.16728	5.940776
FSIP1	0.629783	0.075843	2.175593	1.970609
DNAH5	0.630319	0.075955	-1.30425	5.750179
MYO3A	0.63135	0.076128	-3.1182	1.854336
RPL13	0.63149	0.076193	3.302301	2.038915
HIST1H3C	0.632098	0.076326	-1.21163	4.371112
NHS-AS1	0.632098	0.076363	1.850624	2.009162
S100B	0.632162	0.076431	2.162215	2.299085
SNORD114	0.632162	0.076467	2.855549	0.858614
ZNF442	0.632162	0.076515	-2.78487	2.062572
DNM1P35	0.634109	0.07681	-2.15616	2.230701
NDUFA4L2	0.634109	0.076847	1.480577	3.873358
TAAR1	0.634261	0.076914	-6.32619	0.895908
LINC01252	0.63513	0.077068	-2.46549	1.572486
RHEX	0.636682	0.077316	1.682479	2.786219
ZNF263	0.636682	0.077353	-0.87418	6.097724
DNAH6	0.636903	0.077428	-1.45861	5.553267
ABCC3	0.638112	0.077624	0.908485	8.120628
NHSL1	0.638444	0.077713	1.212895	4.095988
CPLX1	0.638593	0.07778	-2.15172	2.307424
METTL27	0.63894	0.077889	-3.36046	1.570223
EEF1AKMT	0.63894	0.078028	2.907796	1.869983
MIF-AS1	0.63894	0.078045	1.665565	3.014104
LOC202181	0.63894	0.078055	-1.51879	5.213224
TTC32	0.63894	0.078104	-2.52726	1.837862
UQCRHL	0.63894	0.078145	0.885723	5.246389
ADRB3	0.63894	0.078163	2.742322	1.055728
NAPA-AS1	0.639501	0.078301	-2.33504	2.630106
SCN3A	0.639501	0.078372	-6.10537	0.74591
ASCL2	0.639501	0.078378	-2.11433	4.612259
OSER1	0.641396	0.078659	-1.05884	5.62069
FAM135B	0.641664	0.078762	2.316154	1.780202
MTPAP	0.641664	0.078896	-1.0222	5.692271
TRIM22	0.641664	0.078945	-2.83892	1.699555
CCDC150	0.641664	0.078953	-1.95866	3.625533
LINC01126	0.641664	0.078956	-2.36777	2.163884
ATP6V1G1	0.641664	0.078985	-0.87647	6.147432
CCDC68	0.642108	0.079119	3.02329	2.03104
MIR3138	0.642108	0.079182	2.411653	1.350168
MAP3K7CL	0.642108	0.079187	-3.71854	0.796179
UBIAD1	0.642869	0.07935	-1.16513	4.124413

KIF13A	0.642869	0.079469	0.870645	5.403413
SPTY2D1	0.642869	0.079491	-0.95607	6.381493
MOCOS	0.642869	0.079515	-1.2927	4.169215
DSTNP2	0.642869	0.079526	-2.72647	2.123063
TSHZ2	0.642911	0.0796	-1.69724	4.026635
LINC01103	0.642911	0.079714	-6.20008	0.799894
MAGEA1	0.642911	0.079728	-6.25894	0.843996
MEI1	0.642911	0.079741	1.560783	4.070952
BBOX1-AS1	0.642911	0.079776	2.824999	1.448375
ACTR5	0.642961	0.079831	-1.16436	4.148397
ZNF337-AS	0.643606	0.07996	-1.98111	2.954089
BMS1P1	0.643697	0.080072	0.76524	6.266847
TMEM170I	0.643697	0.080079	-1.44005	3.921604
LRCH1	0.643697	0.080166	-1.03679	5.208978
PARP14	0.643697	0.080168	-0.8437	8.905151
ZFP3	0.64395	0.080455	-1.63632	4.64853
CHTF18	0.64395	0.080485	-1.07056	4.689327
OXER1	0.64395	0.080587	1.372911	3.310433
GTPBP4	0.64395	0.080595	-0.99181	6.738738
LOC101929	0.64395	0.080674	-2.0394	2.556758
THEM4	0.64395	0.080794	-1.12538	4.721879
FRG1	0.64395	0.080876	-3.37684	1.787673
WSB1	0.64395	0.080911	-0.90068	5.298967
LINC01981	0.64395	0.080936	-6.06882	0.703831
MIR31HG	0.64395	0.081002	1.550719	3.094876
CCNT2	0.64395	0.081014	-2.42839	2.248044
MBLAC2	0.64395	0.081053	1.249115	3.841307
COL4A4	0.64395	0.081115	-1.51884	5.713584
TCTEX1D4	0.64395	0.081146	-2.00574	2.637708
GHRLOS	0.64395	0.081206	-3.59123	0.793266
PINK1-AS	0.64395	0.081236	1.201241	3.698769
ALMS1	0.64395	0.081386	-0.8133	7.805047
DYNC2LI1	0.64395	0.081406	2.644097	2.64015
ZNF766	0.64395	0.081504	-0.92803	5.554571
TMEM192	0.64395	0.081511	-1.07978	4.570689
CFB	0.64395	0.081514	-1.37502	4.060561
SNORD67	0.64395	0.081521	-1.11228	6.324223
PSME2	0.64395	0.081539	0.825004	5.953126
LOC100506	0.64395	0.081579	1.803323	2.729401
PGLS	0.64395	0.08161	0.938969	4.791286
PAG1	0.64395	0.081615	0.864677	7.371323
LRRC40	0.64395	0.081632	-1.17588	4.588228
EMP3	0.64395	0.081639	1.324215	3.017645
TMPRSS11I	0.64395	0.081702	3.613591	1.927308
P3H3	0.64395	0.081718	-1.02755	5.269345

MEF2D	0.64395	0.081721	-1.67227	2.818812
LINC00515	0.645196	0.081934	-3.3471	1.269209
DIP2A-IT1	0.645196	0.082	1.85617	2.359144
CTRB1	0.645196	0.082026	3.645065	1.180565
UNC13A	0.645824	0.082246	-2.11953	3.495131
LOC154761	0.645824	0.082385	1.188319	3.7453
HIST1H2AC	0.645824	0.082387	-0.92741	5.395796
CFAP43	0.645824	0.082399	-1.88296	3.001507
THAP9-AS1	0.645824	0.08244	-2.45384	2.157767
FLJ31356	0.645824	0.082534	1.231101	3.70854
MIR6081	0.645824	0.08258	-3.16377	1.316802
C1orf198	0.645824	0.082625	-2.82602	1.11115
EPHB1	0.645824	0.082639	1.390941	4.115249
TTC41P	0.645824	0.0827	-2.24825	1.543284
NTN1	0.645824	0.082728	-1.24832	5.87511
ABCA8	0.645824	0.082803	2.534031	1.371036
LINC01195	0.645824	0.082826	-6.10391	0.743953
RRS1	0.645824	0.082836	-1.0136	4.980743
SCG2	0.645824	0.082845	-2.28506	2.772405
GPR155	0.645942	0.082909	-2.37864	1.482234
DRD1	0.646127	0.082982	-2.66102	2.182025
MTHFD2L	0.647479	0.083205	-1.32022	3.182875
EDAR	0.648939	0.083542	-1.14226	5.192234
TAGLN	0.648939	0.083694	-1.80748	2.768607
EDF1	0.648939	0.083699	1.138568	3.676477
ZSWIM4	0.648939	0.08373	-0.88443	5.653972
SLC16A1	0.648939	0.083811	2.354921	0.961826
LRRC19	0.648939	0.083849	1.937256	3.7249
HOXB9	0.648939	0.083874	-1.74311	4.569916
C6orf99	0.648939	0.083977	3.249505	1.251182
INPP5E	0.648939	0.083995	-1.20818	4.400671
STX11	0.648939	0.084085	-1.80023	3.117312
ZFP36	0.648939	0.084114	-0.86782	8.514095
PPP1R1A	0.648939	0.084207	-3.26006	2.825646
SLC25A21	0.648939	0.084233	2.211111	1.950291
METTL16	0.648939	0.084321	-0.81523	5.867293
KCNMB2	0.648939	0.08438	2.782319	2.284351
KCP	0.648939	0.084413	1.326811	3.723514
HLA-DRB3	0.648939	0.084437	2.552905	2.119067
TEP1	0.648939	0.084498	0.758144	7.934479
CLSTN3	0.648939	0.08452	-0.84129	5.542906
CSTA	0.648939	0.084541	3.196377	6.008036
ELOVL7	0.648939	0.084551	3.236751	1.409888
KLF10	0.648939	0.084631	-2.32881	1.71361
STX5	0.648939	0.084641	-4.0505	1.040015

MIR1537	0.648939	0.084646	3.34001	0.784797
CD47	0.648939	0.084716	-3.77702	1.432877
ARHGAP42	0.648939	0.084719	-1.14111	6.088263
EIF1AX	0.648939	0.084728	-1.028	6.041312
CILP2	0.649049	0.084845	-4.17647	0.958994
HDAC2-AS2	0.649049	0.084903	-2.44646	1.986258
SMG1P3	0.649049	0.084939	-0.85809	6.793602
LRRC70	0.649049	0.084978	-3.17599	1.368783
SPAG5-AS1	0.649049	0.08499	2.592308	2.274994
SRP54	0.649562	0.085126	-4.07224	0.87875
NEU1	0.649562	0.085156	-4.00541	1.091206
KIAA1147	0.650212	0.085291	-1.92402	4.519915
MIR4778	0.650665	0.0854	-6.1918	0.805546
IER5L	0.652032	0.085629	-1.41618	4.696636
ZSCAN12P1	0.652596	0.085801	-1.52214	3.312944
ZNF432	0.652596	0.085802	-1.1658	5.503401
GIMAP8	0.652816	0.085888	1.023054	5.133107
XDH	0.652816	0.085999	1.378931	5.666906
AK7	0.652816	0.086058	1.356624	2.980917
NIPAL1	0.652816	0.086061	1.095259	4.812442
LZTS2	0.652816	0.086124	-0.93943	5.871419
GCNT1	0.652816	0.08613	1.51198	3.374285
SUZ12	0.652876	0.086315	-0.8119	6.903726
LACTB2-AS	0.652876	0.086378	1.226458	3.782927
C2orf91	0.652876	0.086407	-2.80999	3.544369
STC1	0.652876	0.086437	-1.51573	5.396388
PTMS	0.652876	0.086452	0.873627	8.486891
TMEM144	0.652876	0.086472	0.883102	4.966115
DCAKD	0.652876	0.086486	2.543046	2.452924
FCHSD1	0.652951	0.086557	-0.94518	5.38685
SKIDA1	0.652951	0.086707	-2.96606	1.732812
GSAP	0.652951	0.08673	-0.86855	5.794292
PLD6	0.652951	0.086801	-1.84914	3.236641
H1FX-AS1	0.652951	0.086842	1.263649	3.745044
SPINK4	0.652951	0.086917	2.258961	4.878405
TNC	0.652951	0.086935	-1.10809	7.940394
ABCB1	0.652951	0.086945	-1.62325	4.601229
B4GALNT4	0.652951	0.086956	-1.83607	3.833443
ID3	0.652951	0.087029	-1.2535	4.290556
LMCD1	0.652951	0.087132	-1.3954	3.958267
RAVER2	0.652951	0.087164	0.763122	5.87608
YWHAG	0.652951	0.087168	-0.83717	8.120042
SLC4A4	0.652951	0.087193	2.449365	2.29051
SMAD1-AS	0.653372	0.087344	1.576122	2.971257
MIR145	0.653372	0.087349	-2.24081	3.654624

USP6	0.653425	0.087406	2.676711	1.388615
ITGA1	0.65466	0.087621	-1.04491	7.202492
ATP8B1	0.65519	0.087741	0.850166	8.289123
SLC22A20P	0.656977	0.088031	-2.94496	1.388518
TNFRSF9	0.657254	0.088118	-2.58346	2.058511
RAPGEF4-A	0.657694	0.08825	-3.44745	1.823156
HLA-DRB4	0.657694	0.08831	2.949728	2.375862
TEDC2	0.657694	0.088343	-2.73832	1.600973
RNFT1-DT	0.657694	0.088414	1.760312	2.434668
PFDN5	0.657694	0.088469	-1.51417	2.910478
NAT1	0.657694	0.088498	3.142131	0.82412
NMNAT1	0.657694	0.088528	2.907863	0.774233
GDF15	0.658269	0.088656	-1.31631	5.475959
GTPBP10	0.658307	0.088711	-2.6283	1.952815
LOC339666	0.658368	0.088769	1.849778	1.494019
FAM241A	0.659307	0.088946	0.925323	4.60605
RAB11FIP5	0.659842	0.089081	-0.89584	5.431071
MTOR-AS1	0.659842	0.089119	-2.32512	1.465883
TINAG	0.660064	0.089215	2.826202	1.785571
ZNF843	0.660064	0.089249	2.578149	1.4831
MIR648	0.660234	0.089353	1.508691	2.348571
ISCA2	0.660234	0.089416	2.380919	0.728912
CCPG1	0.660234	0.089465	2.767083	0.959594
TBCA	0.660234	0.089474	1.854183	3.118431
DRICH1	0.660673	0.08963	-2.64227	1.850509
CD1A	0.660673	0.089738	3.08453	1.523353
MIR3616	0.660673	0.08986	-5.12363	1.361822
TAS2R5	0.660673	0.089872	2.006063	2.428339
TESMIN	0.660673	0.089875	-2.56059	1.315993
IFI44	0.660673	0.089885	-1.127	5.987572
LOC101928	0.660673	0.08993	2.146238	1.672509
SZRD1	0.660673	0.089957	-4.69409	1.423853
ASTN2-AS1	0.660673	0.090055	1.396513	3.166185
INAFM2	0.660673	0.090072	-1.20625	4.427027
LY6D	0.660673	0.090147	2.511215	4.67737
GADD45G	0.660673	0.090207	-1.70486	3.161876
MIR23A	0.660673	0.090208	-2.25153	1.449057
PCSK6	0.660673	0.090286	2.538957	1.870854
SPACA6P-A	0.660673	0.090327	-2.78108	1.317987
C20orf27	0.660673	0.090355	3.001607	1.469432
CLDN4	0.660673	0.090396	-1.1824	7.443137
DDX58	0.660673	0.090512	-0.99946	6.440758
GRIK5	0.660673	0.090545	-4.04969	0.840905
PLAC8	0.660673	0.09055	1.61164	3.207077
POGK	0.660673	0.090591	-2.37323	1.862394

PIAS3	0.660791	0.090689	-0.87648	5.832062
HMGN5	0.660791	0.090733	0.895936	6.25914
SUPT20H	0.660791	0.090796	1.380617	3.437174
KCNMA1-A	0.660791	0.090818	-2.57833	1.548744
TMEM128	0.660791	0.090859	2.136235	2.013007
METTL1	0.662571	0.091156	2.94655	1.302436
LOC10050€	0.662571	0.091205	1.494838	3.839895
SNORA8	0.663692	0.091409	-0.85654	6.77498
SLC16A2	0.664748	0.091606	1.004756	5.066488
CHST3	0.664813	0.091665	-1.28106	5.663636
SIX4	0.665967	0.091875	-1.70195	3.989121
LMO4	0.666836	0.092046	0.799233	7.117828
BTD	0.667499	0.092229	-3.67349	1.517296
BCAS1	0.667499	0.092271	1.531569	4.343658
LOC25489€	0.667499	0.0923	-2.24195	2.676227
POU3F3	0.667499	0.092341	-3.33399	1.553999
ZNF519	0.667534	0.092396	-1.35559	4.228399
PCDHB8	0.668245	0.092546	-2.34315	2.810014
TOMM20L	0.669151	0.092887	2.6746	1.532498
BIRC3	0.669151	0.09289	-1.07033	6.688773
CLDN24	0.669151	0.092898	-5.72044	0.477179
MGAT2	0.669151	0.092952	0.831576	5.556922
NUP133	0.669151	0.092973	-0.79203	5.983628
HBEGF	0.669151	0.092998	-1.29781	5.228953
STIM1	0.669151	0.093062	-3.30712	0.805428
DNASE2	0.669151	0.093079	-0.99442	4.92171
ZNF594	0.669377	0.093162	-1.1397	4.934007
DGKD	0.669924	0.093289	2.030255	2.165128
SNORD135	0.670116	0.093415	-1.61802	4.034549
C17orf107	0.670116	0.093466	2.006727	2.741366
HSD17B11	0.670116	0.093525	0.720844	6.081411
MSRB1	0.670116	0.093559	-1.11817	4.322491
CCDC162P	0.670116	0.093571	1.417973	4.183663
FAM160B1	0.670942	0.093738	-1.47778	4.662858
ANKRD36C	0.671154	0.093818	-0.77685	7.982838
SV2C	0.67147	0.093968	-2.6327	1.734839
HSD11B2	0.67147	0.093968	1.236196	5.05738
FBXO5	0.67147	0.094016	-2.61214	1.216199
NCS1	0.672384	0.094222	-2.6778	0.782142
ABCC9	0.672384	0.094246	-2.2017	4.002502
LOC10012S	0.673289	0.094425	2.667512	0.840235
LINC00955	0.673794	0.094547	3.356195	1.13976
GRHL2	0.67407	0.094637	0.861176	6.734522
SMARCA4	0.675002	0.094843	-2.05031	2.455651
CASP16P	0.675002	0.094872	1.631571	2.592106

GRB2	0.675002	0.094957	-3.7493	0.877338
STARD5	0.675002	0.094974	1.236514	3.754326
KHDRBS3	0.675487	0.095101	-1.501	3.740107
OSTCP1	0.675487	0.095229	2.823678	1.303788
FER1L4	0.675487	0.095323	0.812574	7.099163
TATDN2	0.675487	0.09533	-0.74158	6.569381
FAM214B	0.675487	0.095336	2.276437	1.034974
FBXO48	0.675487	0.095392	-2.17167	1.945098
TMEM190	0.675487	0.095402	-2.22777	1.81183
RAB23	0.67633	0.095608	-2.88213	1.048025
POLR2J	0.67633	0.095654	-1.07862	4.632379
SYT11	0.67633	0.095676	-1.18051	5.216058
MMP28	0.677088	0.095835	2.010024	2.369785
BTN3A2	0.67712	0.095965	1.978302	1.255599
RAET1E	0.67712	0.095988	2.376657	3.662439
TLX2	0.67712	0.096001	1.885408	2.147546
PI4KAP2	0.67712	0.096046	-0.91331	5.121705
NKX2-2	0.677543	0.096157	-6.62744	1.138541
SSBP3-AS1	0.67813	0.096292	1.474324	3.328904
MSTO2P	0.678682	0.096452	-1.98832	2.804988
FNBP1L	0.678682	0.096484	0.905743	5.23639
STX18-IT1	0.678682	0.096585	-2.71344	1.729108
GJC2	0.678682	0.09662	1.9216	2.176093
ANKDD1A	0.678682	0.096629	-1.32365	4.065765
IFIT2	0.679478	0.096796	-1.76823	5.42894
CLCF1	0.679478	0.096846	-2.89386	1.678437
LILRB5	0.680919	0.097104	3.222685	1.003381
LINC00842	0.680944	0.097159	-2.75726	1.562057
PDLIM3	0.682329	0.097417	-1.93087	2.923878
NOV	0.682329	0.097474	-2.30353	2.530563
SNORD45B	0.682329	0.097513	-1.14424	5.478739
EIF4H	0.682604	0.097604	-2.87171	0.918528
PNO1	0.683434	0.097775	-0.89528	4.696552
ERCC4	0.683785	0.097877	-1.14649	5.003977
NAB2	0.683809	0.097933	-1.2498	5.134304
AFAP1	0.683906	0.097999	2.053607	0.904879
CH25H	0.684731	0.098204	-2.8559	1.547835
LEPROTL1	0.684731	0.098221	-1.06492	5.409176
FAM201A	0.684968	0.098354	-2.73259	2.219994
OCM	0.684968	0.09836	-6.5827	1.103646
STARD10	0.685232	0.098473	0.993043	6.884916
NAGA	0.685232	0.098502	-0.87632	5.109734
PHF20	0.68576	0.09863	-0.73582	6.682185
SLC5A4	0.685979	0.098714	-2.0443	2.417367
BTF3	0.686563	0.09885	2.043569	2.141248

THOC6	0.687102	0.09898	2.871184	1.022769
NR0B2	0.68759	0.099103	1.689347	2.119289
CRIP1	0.688453	0.099285	0.913226	5.284574
HIST1H2BF	0.688453	0.099332	-1.26016	4.347119
ATF7IP	0.689328	0.099544	-2.14466	3.208245
RNVU1-19	0.689328	0.099564	-2.87264	1.705232
MYH13	0.690039	0.099753	1.423172	3.481189
FGD2	0.690039	0.099772	0.929419	5.02127
CYP4V2	0.690323	0.099881	0.784078	6.070098
ACPP	0.690323	0.099918	2.417421	1.736733
LOC10023:	0.690401	0.100072	-3.93628	0.77378
XPO7	0.690401	0.100171	-0.77535	7.034536
CYTH1	0.690401	0.100179	1.475543	3.590304
LINC00698	0.690401	0.100194	2.451774	2.63821
TIMP2	0.690401	0.10024	-1.03104	8.518628
TOLLIP	0.690401	0.10027	0.788023	5.733214
LDB1	0.690401	0.100298	1.11799	3.777896
CCL8	0.69068	0.100461	-3.52766	0.729117
PTDSS1	0.69068	0.100469	1.758991	2.054529
MAP3K20-/	0.69068	0.100496	1.08155	4.611931
TOPORS	0.691295	0.100709	2.119959	1.855411
TMC7	0.691295	0.100722	-3.0605	0.945691
SLC25A2	0.691295	0.100744	-3.5365	1.24516
KLRK1	0.691563	0.100835	3.566497	1.304363
KRR1	0.691845	0.100959	-0.84498	5.602245
SPEM2	0.691845	0.101191	-5.85787	0.567684
CCDC9B	0.691845	0.10121	-1.13677	6.648977
ACOT4	0.691845	0.10122	1.777881	2.545513
CARD10	0.691845	0.101222	1.037399	6.498202
SHROOM2	0.691845	0.101243	-1.85721	4.78769
NUTM2A-A	0.691845	0.101246	-1.51356	3.865521
VANGL2	0.692012	0.101377	-1.7044	4.577941
TSPAN14	0.692012	0.101417	1.144633	3.550442
SNORD113	0.692012	0.101476	2.295624	1.861924
RAB31	0.692012	0.101481	-1.07116	6.918391
IQCG	0.692071	0.101566	-2.88953	0.615786
UBAP2	0.692071	0.10162	1.171913	3.780629
KCNS1	0.692071	0.101689	-2.89366	1.15782
ATCAY	0.692071	0.10181	-3.41117	1.013617
MIR5094	0.692071	0.101819	-5.92547	0.617634
ZNF597	0.692071	0.10192	-1.73714	4.492632
PRORSD1P	0.692071	0.102	-2.13349	2.215928
ATP10B	0.692071	0.10202	1.428934	7.119039
CACNA2D1	0.692071	0.102067	-0.97488	5.233656
TAMM41	0.692071	0.10213	1.313868	3.185161

FAM81A	0.692071	0.102145	1.25117	3.284527
PDCD6IPP2	0.692071	0.10223	-1.25944	3.310088
XPO4	0.692071	0.102235	-0.75419	6.815754
EEF1AKMT	0.692071	0.102276	-1.97786	2.59493
RTP5	0.692071	0.102281	3.027778	1.332228
RHOV	0.692093	0.102337	1.784183	4.154988
MOB3B	0.692304	0.102421	0.955381	6.331399
NUFIP2	0.692682	0.102605	-0.71768	8.916723
MITF	0.692682	0.102607	-1.85868	2.90318
ARSJ	0.692682	0.102635	0.82987	4.940432
PWWP2A	0.693328	0.102805	0.897399	4.579889
CHRM5	0.693328	0.102884	-3.23945	0.487625
GPRC5D	0.693328	0.102903	-1.63354	3.992305
SLC7A5	0.693328	0.10297	-1.30826	6.248496
NLN	0.693328	0.102995	-0.83064	5.645739
C9orf64	0.695047	0.103303	1.613784	2.539938
FGL2	0.696458	0.103566	-1.15697	7.732692
PPP1R32	0.696487	0.103662	-5.79294	0.526809
COL14A1	0.696487	0.103677	-1.168	8.306709
C1orf159	0.697251	0.103844	-1.6034	3.773271
MRPL19	0.697811	0.104032	-0.7361	6.611254
CHD8	0.697811	0.104033	-1.18293	4.077868
CCNG2	0.69816	0.104141	0.75679	6.32591
FRMD6	0.69816	0.104247	2.897293	1.539647
ST14	0.69816	0.104279	0.799365	8.217998
CYP4F2	0.69816	0.104315	2.793872	1.854285
PPIC	0.69816	0.104512	0.805836	5.797712
A2ML1	0.69816	0.104527	2.756294	5.493537
ADH1B	0.69816	0.10458	3.09267	0.895378
TIGD7	0.69816	0.104584	-1.51348	4.097452
LAMA3	0.69816	0.104588	-5.69055	0.457647
PHF21A	0.69816	0.104618	-2.13329	2.281369
MRPL42	0.698364	0.104747	2.647697	0.782734
ZNF800	0.698364	0.104766	-0.75681	6.222676
TSPEAR-AS	0.698364	0.104808	-5.85114	0.57454
SPDYE3	0.698779	0.104923	-1.3357	3.500321
SNORA49	0.699206	0.105041	-0.88011	8.019625
PLEK	0.699355	0.105116	-1.28367	4.715633
SAMD13	0.699748	0.105229	2.798282	1.49911
SCARNA21	0.700322	0.105369	-0.80248	8.184472
EVC	0.700394	0.105433	2.498239	1.719096
TNFRSF13E	0.700981	0.105575	2.112037	1.658754
DHX34	0.701119	0.105713	-0.86784	5.409005
MIR337	0.701119	0.105736	-5.68428	0.453875
SAMD5	0.701119	0.105756	1.019642	7.249932

TRMT10A	0.701151	0.105814	2.858008	0.862104
FZD5	0.701676	0.105947	0.818158	7.112481
GFI1	0.702266	0.106089	-5.89216	0.592945
RPL23AP53	0.702465	0.106173	-0.84356	5.7686
ZNF780A	0.703456	0.106376	0.920559	4.495036
FNIP1	0.703522	0.10644	2.555316	1.242562
ZNF697	0.703889	0.106568	-1.17876	4.858456
EML5	0.703889	0.106656	-1.23739	3.913388
OSBPL7	0.703889	0.106656	0.753745	6.134637
DBF4	0.704654	0.106826	-1.09478	4.767242
ANO6	0.704717	0.10692	-2.36459	2.362824
CHST1	0.704717	0.107068	-2.47111	1.487036
UPRT	0.704717	0.107101	3.662684	0.705258
SLC5A12	0.704717	0.107101	1.878097	2.087326
RUNX1-IT1	0.704717	0.107104	-1.94813	3.544446
HPRT1	0.7048	0.107188	-0.93692	5.253012
ATP6V0B	0.7048	0.107224	-1.80543	2.832836
MAGI1-AS1	0.70568	0.107412	-1.33928	4.336424
GOLGA8T	0.705907	0.1075	-1.94208	3.01343
NUDT16	0.706791	0.107728	1.554939	2.471792
ZMYND19	0.706791	0.107743	-1.36278	4.540944
ELL	0.70721	0.107921	-0.80161	5.675623
LSP1P4	0.70721	0.107983	-1.85365	2.798742
UNC13C	0.70721	0.108045	-3.27043	1.184875
SGF29	0.70721	0.108047	-1.20068	4.077494
LAMC2	0.70721	0.108111	-1.21068	6.119559
ARHGAP45	0.70721	0.108131	-1.67777	3.187255
SLC11A1	0.70721	0.108221	-1.25728	5.065948
DOK4	0.70721	0.108238	0.736667	6.300562
DQX1	0.708824	0.108598	0.994835	5.022228
ADGRL2	0.708824	0.108672	3.221059	0.834769
NR4A2	0.708824	0.108678	-1.2621	6.222739
PARP9	0.708824	0.108701	-1.05335	4.932151
EFHD2	0.709489	0.108935	0.838413	8.159117
ZNF571-AS	0.709489	0.109255	-2.47288	2.263034
ZNF117	0.709489	0.109271	-1.08428	6.785329
SPRY1	0.709489	0.109282	1.523243	2.815916
CCL11	0.709489	0.109358	1.426502	4.103802
FAM118B	0.709489	0.109393	0.803956	5.330334
IL6	0.709489	0.109453	-3.3325	2.777171
ATF1	0.709489	0.109492	-1.14223	4.609074
XACT	0.709489	0.109495	-6.38838	0.954742
INSM1	0.709489	0.109534	2.244881	2.40235
VPS35L	0.709489	0.109553	-2.6002	2.023388
ZNF536	0.709489	0.109597	1.606306	3.464057

PCGF2	0.709489	0.109724	-2.40998	2.349139
TNNC1	0.709489	0.109768	-2.72524	1.800843
KCNA2	0.709489	0.10977	2.201257	1.77126
PHLDA3	0.709489	0.109776	-1.50949	2.605403
CDR2	0.709489	0.109776	-0.80354	6.236033
ENPP6	0.709489	0.109776	2.292789	1.113387
SMIM4	0.709921	0.109912	-1.32237	3.010771
LIMA1	0.709921	0.109998	-1.84964	2.674648
NARS2	0.709921	0.110041	1.833724	2.436024
CCT6A	0.709921	0.110086	-3.51174	0.741225
FAM169B	0.709921	0.110134	2.591785	1.697043
SP2	0.709921	0.110168	-0.83459	5.438821
LOC33980[illegible]	0.710036	0.1103	-2.57723	1.490745
LOC33929[illegible]	0.710036	0.110301	3.167365	1.686794
ZNF426	0.710036	0.110365	2.342355	0.966155
LGI4	0.710036	0.110402	1.172974	4.299173
ITGAD	0.710824	0.11064	-2.32043	1.787541
ZC3H12C	0.710824	0.110657	-1.50452	5.065516
SOWAHB	0.710824	0.110696	-2.08818	2.948088
EIF1B-AS1	0.710824	0.110741	-2.30522	1.709949
SH3GL2	0.710913	0.110809	-4.30237	1.070822
PPIL1	0.711395	0.110939	-1.13598	4.49541
ELMOD3	0.711696	0.111043	-1.68904	2.767246
RNY5	0.711696	0.111125	-1.12212	8.422132
ATP5PB	0.711696	0.111168	0.702772	6.66394
SRXN1	0.711696	0.111203	-0.90388	5.296313
LGR4	0.712041	0.111346	0.891479	7.445035
C17orf67	0.712041	0.111365	-1.84776	3.295859
ISPD-AS1	0.712837	0.111544	-5.68993	0.457448
EGR3	0.71301	0.111688	-2.61832	1.012276
PLCL2	0.71301	0.11189	1.979957	2.460192
ITIH6	0.71301	0.111894	-4.60324	0.960563
PTPRC	0.71301	0.111998	1.02033	4.826834
RAI14	0.71301	0.112014	-2.11278	1.818323
GUCA1C	0.71301	0.11202	3.874056	0.880118
TSPAN1	0.71301	0.112025	0.915257	7.173721
NNMT	0.71301	0.112036	-0.97829	6.489038
OSBPL2	0.71301	0.11206	-3.7993	0.674251
YY1AP1	0.713263	0.112154	-2.57754	2.088017
PALMD	0.713341	0.112266	0.874038	5.457529
PLLP	0.713341	0.112275	1.03381	4.431364
MRPL43	0.71336	0.11235	2.321819	1.508782
CACNB2	0.71336	0.112387	-2.19335	2.9768
RPS14P3	0.71468	0.112673	-1.55835	3.397732
H1F0	0.71468	0.112761	-0.89294	7.823962

GRTP1-AS1	0.71468	0.112804	1.478952	2.785815
GOLGA6L2	0.71468	0.112813	-3.01613	1.436833
NGEF	0.715227	0.112954	1.203402	3.748358
SNORA54	0.716055	0.113149	-1.04453	6.899127
RFWD3	0.716055	0.113207	-0.7888	5.798077
UPK3A	0.716055	0.113265	-5.68676	0.455563
CD200	0.716055	0.113303	3.449698	0.80917
BIN2	0.717076	0.113519	-3.34989	0.552228
CDX1	0.717507	0.113642	2.02989	5.267125
REEP3	0.718063	0.113802	-0.80191	7.076782
DUSP12	0.718063	0.113839	-1.24057	3.519581
VMA21	0.719222	0.11408	-0.82156	5.777923
LOC100128	0.719222	0.114133	-3.49111	1.686662
HRCT1	0.719707	0.114292	2.129634	1.450892
ZNF791	0.719707	0.114319	-0.96635	5.751523
MAGEE1	0.7211	0.114596	-2.15597	1.818836
IRAK2	0.722227	0.11483	-1.61828	4.547221
LRTOMT	0.72235	0.114904	-2.7891	1.265205
ERGIC2	0.722612	0.115062	-0.86647	5.779108
TNFRSF25	0.722612	0.115094	-1.77722	3.247801
CYP4F62P	0.722612	0.11522	-6.28044	0.87394
PSMC3IP	0.722612	0.115221	1.653446	2.545529
C2CD2	0.722612	0.115221	1.360265	2.880396
GPX4	0.723106	0.115456	1.707354	1.733567
ZNF785	0.723106	0.115474	0.836096	5.022716
LOC102724	0.723106	0.115512	1.280373	3.472599
TIAM2	0.723106	0.115521	1.18332	3.493632
CCP110	0.723709	0.115708	-1.98027	2.376432
CWC22	0.723709	0.115747	-0.68192	6.64933
FDFT1	0.723709	0.115837	1.189632	4.234805
LOC643339	0.723709	0.115922	-2.92228	1.438132
CBLC	0.723709	0.116044	-2.40992	1.34585
DUSP10	0.723709	0.116067	-1.58683	3.302334
KIAA0754	0.723709	0.116146	-0.77253	7.468351
GUCY1A1	0.723709	0.116178	-2.1691	1.823652
CD300E	0.723709	0.116201	-1.68191	4.562186
IL18R1	0.723709	0.116276	2.350054	2.084552
SLURP1	0.723709	0.116299	2.859725	1.590611
LINC02421	0.723709	0.116316	-3.82205	0.692051
HMCN1	0.723709	0.116423	-1.35182	8.256458
LOC728554	0.723709	0.116568	0.946573	5.297603
MTURN	0.723709	0.116572	-0.83637	7.548704
MB21D2	0.723709	0.116596	-1.50603	3.554894
ABCA13	0.723709	0.116676	1.160012	4.269747
ITGA9-AS1	0.723709	0.116697	-3.49647	1.264798

MAX	0.723709	0.116776	2.221941	1.457183
CSF2RB	0.723709	0.116851	0.96028	5.091028
IL4R	0.723709	0.116857	0.788202	5.133142
BDKRB2	0.723709	0.116883	0.959315	5.129367
SEMA4B	0.723709	0.116886	1.680849	2.148325
GLP2R	0.724038	0.116994	1.952705	3.112726
ST6GALNA(	0.725191	0.11726	-1.98339	3.122891
GLCE	0.725191	0.117312	0.785639	5.969644
GRIN2C	0.725191	0.117395	3.653662	0.846818
CLIC4	0.725191	0.117426	-0.91028	8.118255
MIR1206	0.725191	0.117457	-2.931	1.068255
SNORA12	0.725336	0.117548	-0.90788	6.610407
POMP	0.725336	0.117591	-0.7984	5.892548
ERCC8	0.725353	0.117649	-2.91048	1.394794
LOC28559:	0.725363	0.117706	-2.87178	1.370836
DENND2D	0.726295	0.117921	1.593181	2.305111
LOC101927	0.726295	0.117968	-3.00439	1.441275
LATS2	0.726322	0.118027	-0.85088	6.305887
PTGER4	0.726427	0.1181	0.902144	5.200744
MGC15885	0.726898	0.118243	1.536475	2.935128
SRL	0.726898	0.118287	2.241048	1.818889
AKAP6	0.727391	0.11849	-1.3616	5.085999
SOX9	0.727391	0.118588	-0.68391	7.526462
MYCBP2-A!	0.727391	0.118644	1.677004	2.264171
LINC01168	0.727391	0.118649	2.629462	1.316819
CLUAP1	0.727391	0.118677	-1.65267	2.954988
HOXA2	0.727391	0.1187	-2.19392	2.138391
ARHGAP40	0.72815	0.118879	1.848812	2.938504
EIF1	0.728435	0.119081	-0.71772	7.543018
NUDT14	0.728435	0.11913	1.083542	3.745702
ENSA	0.728435	0.119165	1.297685	2.867312
GUCY2D	0.728435	0.119209	2.142299	1.555244
SELENOM	0.728435	0.119217	-1.01067	5.235386
HOXA11-A!	0.728435	0.119309	1.524425	3.392741
KRT1	0.728435	0.119315	2.769592	1.84826
RNVU1-3	0.7286	0.119397	2.958021	1.115041
NCOA4	0.728893	0.119501	2.229464	1.722628
DPYSL2	0.729007	0.119575	-1.03911	4.863097
NGRN	0.730097	0.119865	-2.34392	2.428043
ELK4	0.730097	0.119865	-0.70989	7.893527
DVL3	0.730097	0.119976	-0.72347	7.322484
DNAH2	0.730097	0.119976	-1.34387	4.535874
CALB1	0.730155	0.120042	-2.93846	0.693252
P4HB	0.730159	0.120117	0.698554	9.575701
SCARNA2	0.730159	0.120187	0.807136	8.407871

HOTTIP	0.730159	0.120269	1.215988	3.828698
TMEM221	0.730159	0.120303	-2.76151	1.320287
SNORD76	0.730159	0.120407	-1.04744	8.295608
PIP5K1C	0.730159	0.120432	1.277855	3.484457
TCHH	0.730159	0.120498	1.537408	3.164474
PTP4A1	0.730159	0.120538	-0.92075	8.289703
LAPTM4B	0.730159	0.120593	-0.8896	5.718379
PSMB4	0.730159	0.120624	-0.77717	5.597515
PIGB	0.730159	0.120695	1.063098	4.503693
FOXQ1	0.730159	0.120812	-0.91947	6.688678
MRFAP1L1	0.730159	0.120825	0.689066	6.817071
MZB1	0.730159	0.120891	1.286543	4.669376
TCP10L	0.730159	0.1209	-2.73398	0.814393
SMG1P1	0.730159	0.120941	-0.78616	6.901971
FICD	0.730159	0.120988	1.105614	3.296218
POLR2J3	0.730279	0.121064	1.127739	3.397475
MIR3671	0.731358	0.121336	2.253827	1.626093
C1orf61	0.731358	0.121411	2.8629	1.093048
OPLAH	0.731358	0.121517	0.886915	5.598431
DPY19L2P2	0.731358	0.121558	2.278821	1.210633
HCFC1-AS1	0.731358	0.121562	2.529985	1.126424
STEAP2	0.731358	0.121577	-2.16231	2.717076
MARK2	0.731451	0.121663	-2.75557	0.84411
ANKRD46	0.731451	0.121783	-1.98987	1.626305
TMEM47	0.731451	0.1218	-1.08663	5.597359
HIST1H2BN	0.731451	0.121831	-1.64702	4.108146
PBX2	0.731451	0.121902	1.055109	4.355627
CASC19	0.731451	0.121927	1.720707	2.250708
IZUMO1	0.731731	0.122047	-2.46795	1.004511
PKDCC	0.731731	0.122085	1.037888	6.399946
HDDC2	0.732245	0.122227	-0.88429	5.470661
ASAH1	0.732341	0.122351	1.79647	1.119593
CBR4	0.732341	0.122411	0.743706	5.70294
LOC729218	0.732341	0.122455	1.083553	3.640147
NAP1L1	0.732341	0.122466	-1.24485	3.404834
PTK2B	0.732347	0.122523	-2.06317	2.348401
SNORA71E	0.732381	0.122619	1.123026	5.419763
LINC01963	0.732381	0.12264	-1.39882	3.608234
MT1H	0.732577	0.122729	1.93419	3.885003
COL10A1	0.734264	0.123067	-1.04614	5.302922
MIR454	0.736151	0.123504	2.409739	2.352145
PDE3B	0.736151	0.12362	0.728541	6.143558
VSNL1	0.736151	0.12363	-1.47581	3.981021
BRCA2	0.736151	0.123641	-0.79564	6.07763
LOC72968[illegible]	0.736151	0.123664	1.844329	2.537331

RNVU1-1	0.736978	0.123893	-2.84979	1.080264
ERP29	0.736978	0.123916	1.809821	2.300871
UGCG	0.737553	0.124069	-0.84696	6.864718
AKNA	0.737943	0.124387	1.483432	1.844201
CINP	0.737943	0.124395	-1.05444	3.600125
MT1A	0.737943	0.12441	2.041776	1.843939
ALDH1B1	0.737943	0.124415	-0.79431	5.706215
AGO2	0.737943	0.12447	-2.47403	1.039783
HSPA13	0.737943	0.124536	-0.97495	5.177567
TNS1	0.737943	0.124599	-1.81329	3.149348
NUCKS1	0.737943	0.124636	-0.7148	9.267763
CBL	0.737943	0.124661	-0.69786	7.038918
XPOT	0.737943	0.124697	-0.82557	7.259946
SFRP2	0.738305	0.124821	-1.5052	6.457489
SHE	0.738305	0.12489	1.156922	4.250481
F3	0.738305	0.124927	1.348133	3.537938
MIR3162	0.738621	0.125048	3.178988	1.806636
RASSF1	0.738621	0.125141	-2.0414	2.25529
FUT1	0.738621	0.125151	-1.4401	3.260476
SUSD2	0.738621	0.125253	-1.22931	4.099931
LOC101928	0.738621	0.125366	-1.87839	2.790694
SLC17A7	0.738621	0.125374	1.959672	1.617745
RAPGEFL1	0.738621	0.12548	1.512646	3.778863
DCUN1D4	0.738621	0.125522	2.170137	1.172223
ARL17A	0.738621	0.125533	1.004035	4.668555
DNAJC2	0.738621	0.125543	-2.33217	1.501474
DOCK11	0.739688	0.125832	-0.91846	6.163496
LOC101929	0.739688	0.125837	-1.47833	3.120355
ECH1	0.740721	0.126069	0.701715	6.884796
MIR6769A	0.740948	0.126185	2.615808	1.759768
BCL2L15	0.740948	0.126221	1.041691	5.833528
RPGR	0.740948	0.126302	0.842853	6.177278
TBC1D22A	0.740948	0.126334	1.013438	3.733699
ANKRD19P	0.741141	0.126486	-1.43906	2.79389
AOX1	0.741141	0.126515	-1.21172	4.345403
ACVR1B	0.741141	0.126538	1.274895	2.968333
SOX9-AS1	0.741141	0.126612	-2.25754	1.845273
HIF1A-AS2	0.741141	0.126682	-3.11192	1.074528
TTN	0.741141	0.126706	-1.39129	3.812549
EHMT1	0.74118	0.126791	-0.78181	5.654902
SLC45A3	0.74118	0.126825	1.132904	5.022776
PRKAB1	0.741496	0.126991	0.684261	5.901785
FBXL16	0.741496	0.126995	-1.14495	5.425746
SDE2	0.741496	0.127049	-0.74541	6.060269
ZNF333	0.741743	0.127148	-1.40432	3.984995

USP32P2	0.742314	0.127304	1.567699	3.639881
LINC01484	0.742314	0.127581	2.718196	1.068341
GLA	0.742314	0.127605	-1.6592	3.537528
SEC1P	0.742314	0.127636	-3.37994	0.574497
SYTL4	0.742314	0.127673	2.19303	1.368127
FAH	0.742314	0.127688	1.062589	3.515848
DNALI1	0.742314	0.12772	-1.43501	4.461528
ZNF124	0.742314	0.127761	-1.33052	3.5448
SNORA71C	0.742314	0.127816	1.070276	6.039541
RLF	0.742314	0.127857	-0.72466	6.844333
PLEKHA1	0.742314	0.127923	-3.20983	0.992512
CYP8B1	0.742314	0.127925	2.352318	1.044208
FAM230B	0.742648	0.128039	3.238906	1.530854
TMEM199	0.742656	0.128207	-0.86663	4.583555
FBXO41	0.742656	0.128233	-0.87945	5.26893
RAB35	0.742656	0.128235	1.392387	2.129824
PCNX2	0.742656	0.128267	-0.82412	6.358967
SRBD1	0.743348	0.128443	-0.76631	5.992677
RGS5	0.743924	0.128605	-2.45571	1.658458
GRHL3	0.743924	0.128711	2.382199	2.170806
ZNF660	0.743924	0.128712	-1.73794	3.38604
SLC22A10	0.743959	0.128832	2.889439	2.182649
MST1	0.743959	0.128832	-1.14376	3.651641
SCNN1G	0.74413	0.128926	-2.13759	2.672371
B3GNT2	0.74413	0.128975	-0.75843	5.690474
NME5	0.744414	0.129081	2.45064	0.82015
CCDC38	0.74532	0.12931	-2.85175	0.900127
RAB6B	0.74532	0.129352	-1.12919	3.615414
WASHC5	0.745692	0.12951	-0.718	6.502413
LINC00173	0.745692	0.12953	-3.09843	1.070493
TMEM141	0.746089	0.129695	0.814291	4.894234
HMGN3-AS	0.746089	0.129736	-1.23611	3.292763
EPB41L5	0.746089	0.129785	-2.17258	2.015359
CSNK2A3	0.746089	0.129907	-0.83294	5.288757
CACNA2D4	0.746089	0.129923	-1.49733	3.566198
SNORD91B	0.746089	0.129957	-1.16705	6.094945
WDR1	0.746089	0.130091	0.666588	6.488007
AQP7P1	0.746089	0.130095	-0.91164	5.68014
WHAMMP	0.746089	0.130136	-1.63414	3.406942
HERC2P4	0.746089	0.130167	-1.83077	2.946306
CXCR1	0.746197	0.130248	-2.67227	1.94161
GATA6-AS1	0.746197	0.1303	1.231924	3.601078
ZNF292	0.746432	0.130406	-0.68909	8.244719
ENTPD3-AS	0.746432	0.130486	1.506445	3.021302
MNS1	0.746432	0.130584	-0.90674	4.872921

MSR1	0.746432	0.130606	-1.77642	3.973997
APOC1	0.746432	0.130647	-1.74689	2.422705
FAM133CP	0.746432	0.130682	-1.5776	3.610552
TERC	0.746526	0.130797	0.773356	5.957111
WDR12	0.746526	0.130813	-0.98962	4.809888
SLC18A2	0.746544	0.130891	1.003331	5.29183
CPS1	0.746544	0.130977	2.767936	0.876596
C17orf75	0.746544	0.131011	-1.09874	3.802704
LSAMP	0.746544	0.131043	-0.7343	6.70681
WIZ	0.746547	0.131101	-0.70726	5.761471
FAHD1	0.746683	0.131239	1.047589	3.917058
KRTAP12-3	0.746683	0.131309	-6.01548	0.690328
MIR641	0.746683	0.131348	2.923731	1.456437
KRTAP10-4	0.746683	0.131352	-6.01548	0.690328
LOC100422	0.746844	0.131479	-6.01452	0.681071
ADAMTS19	0.746844	0.131496	1.779395	1.952423
RAB6C	0.746844	0.131605	1.352681	2.9774
MTM1	0.746844	0.131608	0.877728	5.690859
SSSCA1-AS	0.746931	0.131681	1.852501	1.928089
ARHGEF9	0.747097	0.131815	2.483869	0.842932
DTX4	0.747097	0.131824	1.51405	2.774405
SCYL2	0.747108	0.131883	1.152331	2.901045
ARMH4	0.747115	0.131941	-1.68157	3.473118
IGSF10	0.747193	0.132012	1.352691	4.468747
SPDYE4	0.747449	0.132114	2.923491	1.828702
TRIM39	0.747458	0.132172	2.05263	1.463412
CRACR2A	0.74748	0.132233	1.185689	3.868731
PYCARD-AS	0.747649	0.132501	2.444284	0.97192
GLYCAM1	0.747649	0.13253	3.266307	0.849489
B3GALNT2	0.747649	0.132703	-0.77502	5.581346
PRPF40A	0.747649	0.132808	-0.67352	8.42128
CNDP2	0.747649	0.13281	1.294024	3.86698
SNX24	0.747649	0.132842	1.021903	4.117484
ELF3	0.747649	0.132911	1.38623	3.270455
CCDC122	0.747649	0.13295	-1.48456	4.12702
PAXBP1-AS	0.747649	0.132953	0.873642	4.365306
ZNF138	0.747649	0.133069	-2.28425	1.830056
OVOL1	0.747649	0.133186	1.671826	4.18167
FCGRT	0.747649	0.13323	1.010666	3.666342
TTC9C	0.747649	0.13324	-1.28429	3.988724
SUSD5	0.747649	0.133241	-2.32653	2.984541
NPFF	0.747649	0.133254	-2.25063	1.121867
LINC02362	0.747649	0.133286	2.920173	1.208732
KIAA1958	0.747649	0.133378	-5.99188	0.665074
B3GNTL1	0.747649	0.133596	-1.47499	3.426108

PALM3	0.747649	0.133624	-1.61466	5.185988
HIST2H2AC	0.747649	0.133705	-0.97931	4.947088
LINC01672	0.747649	0.133711	-5.99826	0.65985
ZNF284	0.747649	0.133717	-1.01068	4.223613
GDPD5	0.747649	0.133733	-1.45021	3.959799
PRKD3	0.747649	0.133761	-0.68115	6.295632
CEACAM6	0.747649	0.133804	1.405316	9.72309
CD300A	0.747649	0.13385	-2.18954	1.221863
GPR89B	0.747649	0.133859	1.013497	3.382563
LSP1	0.747649	0.134018	1.146316	3.329541
ALOX15	0.747649	0.134034	-3.45834	1.026379
FLJ12825	0.747649	0.134107	-2.11798	1.887455
LOC10012	0.747649	0.134112	0.699326	6.720068
MDC1	0.747649	0.134135	-1.52343	4.83438
ANGPTL8	0.747649	0.134176	1.526657	2.342534
KLF5	0.747649	0.134243	1.048569	6.428224
HLA-L	0.747649	0.134258	-1.0135	4.227858
GNPDA1	0.747989	0.134462	-0.89266	5.098534
IL2RA	0.747989	0.134491	2.442672	1.184747
CTRL	0.747989	0.13456	1.324941	2.361202
TNFSF9	0.747989	0.134688	-2.31893	2.500557
SURF4	0.747989	0.134822	1.682967	1.315452
GRM3	0.747989	0.134917	-3.22766	1.026756
ZNF420	0.747989	0.135068	-1.79065	3.10163
TMPRSS11	0.747989	0.13507	3.053374	4.735562
SLC30A4	0.747989	0.135083	0.815492	4.681426
CCNT1	0.747989	0.135099	-2.98723	1.038682
SOX14	0.747989	0.135109	-3.81557	0.421037
DNAAF1	0.747989	0.135124	1.988396	1.872594
LOC10192	0.747989	0.1352	2.102997	1.372642
LINC02586	0.747989	0.135354	-3.32259	0.613893
MARS2	0.747989	0.135371	-1.16657	4.184525
KRT13	0.747989	0.135407	3.284616	2.166122
SHCBP1	0.747989	0.135555	-1.01857	3.868999
SYNE3	0.747989	0.135596	0.852927	5.084427
SMDT1	0.747989	0.13569	-2.05302	2.081507
ANO9	0.747989	0.135719	0.821504	5.729293
SNORD116	0.747989	0.135784	-1.5529	4.159729
TAB1	0.747989	0.135803	0.815284	4.749861
MYEF2	0.747989	0.135846	-2.4349	2.023844
MYO19	0.747989	0.135858	1.962776	1.798246
FAM229A	0.747989	0.135903	1.863888	2.022249
SLC38A5	0.747989	0.135948	-1.44585	3.145679
NDUFA11	0.747989	0.135984	-2.94036	1.654186
CREB3L2	0.747989	0.136119	0.661548	8.306234

HIST2H2BE	0.747989	0.136189	-1.06792	4.839401
RETNLB	0.747989	0.136194	2.590411	1.637378
FMOD	0.747989	0.136235	-0.97499	6.181455
BOD1	0.747989	0.136259	1.60296	2.033058
ZIC2	0.747989	0.136355	1.392495	3.598992
CYP27B1	0.747989	0.136367	-1.82312	2.292669
NRSN1	0.747989	0.136373	2.737185	0.824775
USP11	0.747989	0.136386	-0.70501	6.099953
PGLYRP1	0.747989	0.136538	-2.78325	1.084973
CCDC173	0.747989	0.136673	-1.94874	1.531282
KLHL42	0.747989	0.136675	-0.70873	6.393416
ZADH2	0.747989	0.13684	1.143343	2.548477
GPT2	0.747989	0.13685	1.855297	1.77747
PARP12	0.747989	0.13691	-2.2162	2.067222
PIM2	0.747989	0.137026	0.905635	6.645952
GLMN	0.747989	0.137057	0.938811	4.339924
BCL9L	0.747989	0.137086	-0.69342	8.078446
CSRP2	0.747989	0.137142	-5.92177	0.615951
NUP160	0.747989	0.137205	-0.69672	6.873048
MCTP2	0.747989	0.137257	1.212975	3.189275
WDR92	0.747989	0.137552	1.416386	3.192422
JRK	0.747989	0.137561	-0.9206	5.16729
SPINT2	0.747989	0.137606	0.989551	4.34737
GLT8D1	0.747989	0.137612	-2.58614	0.724128
APOPT1	0.747989	0.137618	1.226142	2.921662
SRPX2	0.747989	0.137623	0.704425	5.537175
COPS5	0.747989	0.137637	-0.77323	5.425054
SNORD31	0.747989	0.137686	-1.79282	2.435984
DNAH11	0.747989	0.137822	-1.70474	4.511208
DNAJB11	0.747989	0.137871	-0.69265	7.243399
RBX1	0.747989	0.137897	-1.3533	3.871126
SPATA17	0.747989	0.137911	-2.14109	2.117593
FSTL1	0.747989	0.138002	-0.8256	8.413724
C17orf97	0.747989	0.138004	0.9771	4.040509
TM4SF5	0.747989	0.13801	1.997448	2.812432
TXNDC17	0.747989	0.138052	1.086776	3.913707
NKPD1	0.747989	0.138067	-1.933	2.581654
MSN	0.747989	0.138087	-0.8345	8.905338
HDLBP	0.747989	0.138139	2.027343	2.270345
RHBDD1	0.748022	0.138215	-2.51101	1.188606
LVRN	0.748022	0.13828	1.749594	1.748026
SLC33A1	0.748022	0.138316	1.006307	4.263168
RAB3B	0.748417	0.138446	1.138272	4.567292
BTNL8	0.749117	0.138668	2.539776	1.947173
TRIB1	0.749117	0.13876	-1.52293	3.160993

COPZ2	0.749117	0.138781	-1.38708	3.223642
ARRDC4	0.749117	0.138845	-0.70667	6.112553
CPA2	0.749117	0.138893	-5.87306	0.582215
RINT1	0.749117	0.138918	-0.87118	4.848448
MIR215	0.749387	0.139025	-5.89762	0.599185
TMEM238l	0.749494	0.13934	1.297836	4.080518
DUOX2	0.749494	0.13935	1.274311	7.257322
AMZ2P1	0.749494	0.13944	-1.05062	4.198674
RNF215	0.749494	0.139456	-1.22075	3.602259
WHAMMP:	0.749494	0.139461	-1.76607	2.719411
UQCRC1	0.749494	0.139469	0.66966	6.646818
GPC2	0.749494	0.139511	1.71533	2.468507
METTL7A	0.749494	0.139572	0.716598	7.234436
MRAP2	0.749494	0.1396	1.315789	4.151464
PRR15L	0.749494	0.139616	0.851049	6.676671
RETSAT	0.75005	0.13984	0.681537	6.774069
CCDC39	0.75005	0.139904	-1.38162	3.904733
MYL6	0.75005	0.139926	-1.21781	3.593438
ATP1A4	0.75005	0.139948	-2.84361	1.004606
ATP5F1A	0.750242	0.140088	1.560057	2.000473
KCNJ5	0.750242	0.140098	-1.9735	1.750814
SYNJ2BP	0.750493	0.140277	0.626833	7.216151
CTBP1-DT	0.750493	0.1403	0.868593	4.157291
SNORD94	0.750493	0.140317	-0.93683	6.61018
PGRMC2	0.750839	0.140499	0.670307	7.070263
LOC643201	0.750839	0.140529	1.449605	3.688534
SNORD60	0.750839	0.140553	-1.12842	7.323032
MIR324	0.750859	0.140687	-1.94176	1.591832
DPYD-AS1	0.750859	0.140947	2.21464	1.058068
ZNF496	0.750859	0.140947	-0.85952	4.919168
KRTAP12-2	0.750859	0.141072	-5.86683	0.586722
MICALL1	0.750859	0.141088	-0.68719	6.28873
TP53INP2	0.750859	0.141127	0.712762	6.695198
SNORD33	0.750859	0.141128	-0.97763	8.891987
ITPR1	0.750859	0.14116	2.183031	1.704298
UBE2Q1	0.750859	0.141263	-0.68049	6.121471
RNF133	0.750859	0.141279	-1.86302	1.986364
F11	0.750859	0.141285	-2.84987	1.119988
PPL	0.750859	0.141304	1.241331	9.168594
PLK3	0.750859	0.141352	-0.98492	5.478277
KIAA1755	0.750859	0.141358	-1.03918	4.456349
PLA2G4D	0.750881	0.14142	1.778763	2.246184
CD44	0.751222	0.141574	-1.28837	4.100367
SMARCA2	0.751222	0.141646	1.303304	2.414145
SNORA15	0.751222	0.141656	-2.88827	0.81948

IGSF21	0.751752	0.141813	1.748773	2.398586
DPY19L2P1	0.752093	0.141935	-2.0128	1.505499
SNORA32	0.753164	0.142194	-0.79178	5.640908
SEM1	0.754127	0.142433	-0.89477	7.033089
MTMR6	0.754133	0.142513	-0.76823	5.61758
NTSR1	0.754133	0.142578	-1.73484	3.59753
TSPAN12	0.754133	0.142607	0.835696	4.984776
EXOSC1	0.75569	0.143015	-1.45428	3.589265
ZNF600	0.75569	0.143017	-0.92338	5.371082
BSN	0.75612	0.143156	-1.25944	3.317467
NOA1	0.756217	0.143232	0.721401	5.309055
COMMD8	0.75626	0.143297	1.241878	3.880026
ATP1A2	0.756405	0.143384	-1.39854	5.11679
HPCAL1	0.756405	0.14344	1.186226	3.945588
ANKRD9	0.757379	0.143683	1.048253	4.43343
LOC10192¢	0.757481	0.143788	1.06867	3.392595
FRYL	0.757481	0.143861	0.615604	8.259711
AMBP	0.757481	0.143926	-2.53463	1.339112
CCNE2	0.757481	0.143933	0.842654	4.393666
HADHA	0.757655	0.144037	0.621477	7.820365
GSK3A	0.757655	0.144081	-0.69784	5.886434
FAM83B	0.757748	0.144157	0.778463	6.504669
C11orf16	0.758442	0.144348	-2.49837	0.800856
HOXB8	0.758442	0.144405	-2.39037	4.132097
DNAH7	0.758839	0.144538	-1.08841	4.894179
GRWD1	0.759052	0.144636	-0.84416	5.438498
KATNAL2	0.75934	0.144785	0.883725	4.051549
PITX1	0.75934	0.144807	1.060922	5.670376
PAFAH2	0.759393	0.144875	0.700201	5.569858
ZHX1-C8or	0.759997	0.145136	-2.97369	0.997177
SPHK2	0.759997	0.14516	2.051979	1.382028
LOC10050€	0.759997	0.145195	1.123947	3.339882
DGCR11	0.759997	0.145222	1.014754	3.613552
SNORA9	0.760041	0.145288	-1.09843	4.240175
PDAP1	0.760249	0.145386	-0.77886	8.187395
AGRP	0.760475	0.145572	1.817737	1.999944
SMC6	0.760475	0.145643	1.817389	1.015581
OSGEPL1-A	0.760475	0.145669	-2.23271	1.02112
MBOAT4	0.760475	0.145705	-3.02716	1.015414
BICC1	0.760475	0.145719	-0.9267	5.776526
SCD5	0.760715	0.145863	1.247352	3.576124
ARHGEF25	0.760715	0.145881	-2.63129	2.383941
MMP10	0.760799	0.145955	-2.16284	2.429447
KAZALD1	0.760874	0.146064	1.135032	3.812877
ANKRD10	0.760874	0.146085	-1.10753	4.459834

CD27	0.760983	0.146164	1.437732	2.15974
DNAJC21	0.761235	0.146283	1.231718	2.257433
GALNT15	0.761235	0.146359	-1.77859	3.842107
SLC6A19	0.761235	0.146387	1.457546	4.640098
GABARAPL	0.761272	0.146452	2.823183	1.128091
ANKRD33B	0.761687	0.14659	1.033521	4.493432
NINJ1	0.762002	0.146748	-0.88998	4.837788
MIR644A	0.762002	0.146829	2.503876	0.832524
SSBP1	0.762002	0.146881	1.395695	2.908963
AGAP1-IT1	0.762002	0.146883	1.083053	4.047456
INTS1	0.762278	0.146994	-0.66236	7.515286
ACTR3-AS1	0.762307	0.147058	1.692086	2.030863
LINC01402	0.76246	0.147214	2.431023	0.952951
DYM	0.76246	0.147289	-0.72122	5.962483
DHRS7	0.76246	0.147452	0.748071	5.5605
PGBD4	0.76246	0.147588	-1.70922	1.921255
SNORD91A	0.76246	0.147589	-2.72311	0.916809
ZNF91	0.76246	0.147659	-2.40582	1.441516
MIR4278	0.76246	0.147684	-5.71628	0.475804
CCDC181	0.76246	0.147685	-5.71628	0.475804
GCSHP3	0.76246	0.147689	-1.03212	4.16257
ZNF736	0.76246	0.147692	-1.04517	4.705968
RHOQ	0.76246	0.147727	-0.80492	6.345136
POU5F1B	0.762714	0.147834	-2.84636	1.219082
PPP2CB	0.762886	0.147991	-0.69777	6.760803
RPS6	0.762886	0.147997	0.633906	10.10296
MIR5003	0.762886	0.148049	2.488009	0.828777
KPNA1	0.762886	0.148151	1.252886	2.830002
SCLT1	0.762886	0.148158	0.658273	5.677252
MYLIP	0.763272	0.148291	-0.81772	5.252602
WDR24	0.763762	0.148444	-1.3235	4.620794
TPP2	0.763969	0.148543	-0.67975	7.206901
SCARNA3	0.764237	0.148653	-1.12951	3.778214
PCDH20	0.764456	0.148754	-1.89451	2.967341
MPV17	0.765959	0.149105	-0.88215	4.212051
PFDN2	0.766418	0.149285	-0.7914	5.402346
ARF6	0.766418	0.149311	0.654544	7.880204
CEP128	0.766647	0.149414	0.683805	6.324201
ACE	0.766675	0.14948	0.93798	4.176433
ACER2	0.766675	0.149537	1.019658	5.468389
MIGA2	0.767384	0.149783	0.726613	5.624287
ABCG8	0.767384	0.149796	2.350628	1.041763
LOC100287	0.767384	0.149861	-2.59586	1.667736
PRDM15	0.767384	0.149909	1.848363	1.336162
ZNF702P	0.767703	0.15003	1.638593	2.862777

LINC01594	0.767963	0.150139	-1.85783	2.965167
BBC3	0.768203	0.150245	1.662626	1.615126
SUMO2	0.768547	0.150431	1.918057	1.268857
VAT1L	0.768547	0.150535	1.437498	3.080376
ZNF251	0.768547	0.150563	-1.6692	2.182997
NEK6	0.768547	0.150568	1.550893	3.243547
ADCY4	0.768547	0.150739	1.966559	1.639924
STARD13-A	0.768547	0.150801	-1.72182	2.948173
ZNF304	0.768547	0.15082	-1.72822	2.548421
CIDECP1	0.768547	0.150863	-1.93847	1.556844
SNORD111	0.768547	0.150888	-1.18783	5.496488
HOXA10	0.768547	0.150898	2.063777	2.272741
LRRC8A	0.768851	0.151195	2.603211	1.158178
PGBD5	0.768851	0.151203	1.715243	2.38807
GGACT	0.768851	0.151265	2.090717	1.300878
SLCO2A1	0.768851	0.151325	0.956764	5.309146
GNAS	0.768851	0.151344	-1.79235	1.814602
MRPS18B	0.768851	0.151369	1.332384	3.371163
GZF1	0.768851	0.151461	1.415344	1.990746
FER1L5	0.768851	0.151493	-1.71841	2.810926
EHD3	0.768851	0.151503	0.988967	4.190297
SNORD1A	0.768851	0.151543	-2.60278	0.952494
GUSBP2	0.768947	0.151621	1.246347	2.968202
R3HCC1L	0.76901	0.151692	2.019643	1.982356
C2CD4D	0.769064	0.151761	-1.96214	1.988476
PHLDA1	0.769801	0.152012	-0.80117	7.454624
HIST1H1C	0.769801	0.152028	-0.73623	6.911278
LINC01004	0.769801	0.152112	0.600745	7.21655
FBL	0.769801	0.15216	0.680075	7.057565
TMPRSS9	0.769801	0.1522	-1.28126	3.773314
USH1C	0.769863	0.152271	0.936688	5.162793
SNORD84	0.770509	0.152591	-1.15469	4.542177
LINC00680	0.770509	0.152603	1.58223	2.102416
GNAQ	0.770509	0.152666	0.59219	7.766436
DECR2	0.770509	0.152748	1.344103	2.088382
TFCP2L1	0.770509	0.1528	0.761769	7.663557
CEP295	0.770509	0.152803	-0.71988	7.018171
GSPT2	0.770509	0.152851	-1.47964	3.202473
ULK1	0.770509	0.152869	-0.68625	5.97271
WISP2	0.770723	0.15297	-2.48402	0.863411
HNRNPL	0.77135	0.153182	0.829583	4.593399
OLMALINC	0.77135	0.153299	1.147374	2.886658
CRLS1	0.77135	0.15356	1.271172	2.291508
LPCAT1	0.77135	0.153561	-1.18469	4.487812
GOLGA4	0.77135	0.153626	-2.71176	1.009886

LINC02367	0.77135	0.15365	2.173516	1.803625
CAMTA1	0.77135	0.1537	-1.59854	3.722079
LINC00668	0.77135	0.153769	2.054449	3.061164
UNC5C	0.77135	0.15381	0.97176	4.870088
LOC105747	0.77135	0.15387	-1.2137	2.928856
MICU2	0.77135	0.154013	-0.79324	5.771773
LOC101928	0.77135	0.154039	-5.59722	0.406383
SNORD81	0.77135	0.154248	-2.51154	1.426645
MMP13	0.77135	0.154252	-3.19279	3.719566
LINC00266	0.77135	0.154263	-2.47026	0.669217
SNORD15B	0.77135	0.154282	-0.76755	9.03205
RC3H2	0.77135	0.154283	-1.06262	3.835785
CCDC80	0.77135	0.154436	-1.24923	5.446716
MID1IP1	0.77135	0.154458	-1.15275	3.961671
PDE4B	0.77135	0.154466	-2.08123	2.091514
ZNF469	0.77135	0.154479	-1.40549	5.441992
SMIM20	0.77135	0.154577	0.9498	3.908587
ANKFN1	0.77135	0.154579	-2.28898	2.725578
UBAP2L	0.77135	0.154607	-1.04449	4.546176
TTTY15	0.77135	0.154749	1.474356	4.313673
GAREM2	0.77135	0.154767	2.148917	1.166885
SLCO1B7	0.77135	0.154786	-3.51344	1.108575
NR2F2	0.77135	0.154804	-0.74262	5.653947
LCORL	0.77135	0.15482	0.960131	4.206749
ABCD4	0.77135	0.154858	1.628088	1.90679
LINC01481	0.771597	0.154966	-1.86671	1.560791
PUS10	0.771911	0.155141	0.995093	4.626482
NHLRC2	0.771911	0.155318	-0.66507	6.918902
REM2	0.771911	0.155336	2.082466	1.544795
PDGFRL	0.771911	0.15536	-1.65167	2.321888
COLEC12	0.771911	0.155375	-0.94994	5.080668
RUFY1	0.771911	0.155382	1.31501	2.98681
QSOX2	0.772232	0.155506	-0.93735	5.159845
KRI1	0.772461	0.155633	-0.68315	6.538975
HEATR1	0.772461	0.15567	-0.67949	7.161477
CATSPER4	0.772974	0.155935	-3.6573	0.819147
FGF2	0.772974	0.155963	-0.93028	5.553397
AMMECR1	0.772974	0.156029	1.57143	1.60998
SP9	0.772974	0.156075	3.023291	1.602181
CITED2	0.772974	0.156076	1.350326	3.352542
GAB2	0.772974	0.156194	-1.74434	2.694559
SBSN	0.772974	0.156327	2.66203	5.433744
MRPL16	0.772974	0.156342	0.779054	5.234688
NCKAP5	0.772974	0.156361	1.054765	3.909505
CLCN7	0.772974	0.156362	2.517678	0.929886

BUB1B	0.773032	0.156433	-0.96651	5.405381
CDO1	0.773032	0.156492	-2.52099	1.148206
CYTOR	0.774942	0.156938	-1.19922	4.051063
LOC101927	0.776411	0.157393	-1.89016	2.361469
ADARB2	0.776411	0.157455	1.607179	1.931547
SLIT1	0.776411	0.15752	1.348721	2.707684
DAB1	0.776411	0.157521	-2.23086	1.052224
POU2AF1	0.776411	0.157611	1.098297	5.984086
LINC02042	0.776411	0.157644	2.435743	2.294693
SCNN1B	0.776411	0.157649	2.086492	2.136707
CXorf21	0.776663	0.15776	1.620719	2.779627
CAVIN2	0.776941	0.157875	0.839628	5.945435
MEIS1	0.776956	0.158039	-1.10451	6.741224
RPS9	0.776956	0.158098	0.951482	4.177977
SDR9C7	0.776956	0.158195	3.083857	0.873241
SLC7A6OS	0.776956	0.158249	0.876917	3.900032
GLCCI1	0.776956	0.158348	0.73454	5.606464
PIWIL4	0.776956	0.158394	-1.12975	4.47995
WWC3	0.776956	0.158473	-0.78972	6.537217
DCUN1D3	0.776956	0.158654	-0.80403	5.483432
ANXA3	0.776956	0.158669	-0.83401	6.622624
STAB1	0.776956	0.158733	0.708059	7.810706
SESTD1	0.776956	0.158753	-0.66538	7.289953
BCL9	0.776956	0.15877	-0.66164	6.503679
RNY1	0.776956	0.15878	-0.81048	9.247183
LOC10013C	0.776956	0.158786	2.994303	0.960155
ZBTB39	0.776956	0.159005	-1.00688	4.921085
SNORD21	0.776956	0.159197	-0.92609	7.187107
MRI1	0.776956	0.159238	-1.84731	3.082176
NSUN3	0.776956	0.159317	0.961337	3.374019
MAST1	0.776956	0.159327	-1.98372	1.895183
RNF121	0.776956	0.15936	2.122602	0.756277
SMAP2	0.776956	0.159437	0.736096	5.913814
TRUB1	0.776956	0.159508	-0.99423	5.035072
RILP	0.776956	0.159557	1.587508	2.907379
UBXN2A	0.776956	0.159595	-1.02338	4.477415
TIMM10B	0.776956	0.159602	-0.63767	6.381063
RASSF3	0.776956	0.159608	-2.21287	1.938655
ELK2AP	0.776956	0.159702	-1.2491	5.203074
LINC01005	0.776956	0.159727	2.835536	1.298018
SIK2	0.776956	0.15974	-0.6465	6.758144
MYCBPAP	0.776956	0.159774	-1.77476	1.808229
LOC101928	0.776956	0.159782	1.157902	2.809647
KCNMA1	0.776956	0.159827	-2.15982	2.40162
LINC00578	0.776956	0.159869	-1.75551	3.708622

NOS2	0.776956	0.160014	1.335901	4.548497
IL1B	0.776956	0.160213	-1.64669	3.757105
RPS6KA5	0.776956	0.160305	-1.19881	3.56056
LOC28303ᶓ	0.776956	0.160333	2.799905	1.467632
CUBN	0.776956	0.160364	0.944838	4.430406
SLAIN1	0.776956	0.160372	1.607839	2.766459
MFSD14B	0.776956	0.160413	-0.64411	6.289234
PRPF3	0.776956	0.16042	-0.69476	7.182298
PSMB2	0.776956	0.160423	-2.34499	1.122045
PLEKHM3	0.776956	0.160425	-0.77488	5.520093
LYPLAL1-D	0.777351	0.160611	2.257247	1.130987
ZBTB34	0.777351	0.160653	-0.77554	5.503073
RHOXF1-AS	0.777351	0.160684	1.200071	2.880935
TMEM87A	0.777513	0.160777	0.948064	4.346583
NF1P2	0.777807	0.160897	3.326317	0.926497
PLEKHG2	0.777918	0.160979	-0.81423	6.321766
TMEM132(	0.777992	0.161054	1.448279	2.339197
FAM43A	0.778213	0.161176	-0.99408	4.749759
DONSON	0.778213	0.161295	-1.02022	4.229158
JKAMP	0.778213	0.16131	2.329697	1.668997
POM121C	0.778213	0.161337	-0.64673	6.823182
DLGAP4-AS	0.778406	0.161436	-1.3813	3.115112
ARFGAP2	0.778591	0.161577	-2.32567	0.84174
RNY4	0.778591	0.161647	-1.01527	10.95366
TUBA3D	0.778591	0.161652	-2.48594	1.051518
CRYZL2P	0.779016	0.161991	0.748225	7.448876
COL27A1	0.779016	0.162018	0.694821	7.748026
FTO-IT1	0.779016	0.162116	-2.21604	1.961517
C9orf152	0.779016	0.162122	0.810914	5.667674
GPATCH11	0.779016	0.162144	1.718605	1.446369
OSMR	0.779016	0.162162	-0.81784	6.235826
TMSB4Y	0.779016	0.162205	2.119201	2.149729
FRMD6-AS	0.779016	0.162227	1.800018	1.689005
STAMBPL1	0.779016	0.162275	-0.87678	5.338445
SLC39A6	0.779043	0.16234	-0.90308	5.653483
EFHB	0.779336	0.162461	-3.77865	0.700847
KIF1C	0.779994	0.162657	0.590097	8.715903
TSPYL1	0.780155	0.16275	0.677857	7.690334
LINC01003	0.780201	0.162931	-2.22447	1.011713
RNF175	0.780201	0.163005	-3.0803	1.343792
TBX1	0.780201	0.163009	-2.66693	2.373617
AKAP13	0.780201	0.163046	1.286649	3.971967
ZNF83	0.780201	0.163057	-2.07383	2.291021
MICU1	0.780391	0.163157	1.58658	2.230702
LOC101927	0.780532	0.163246	2.57341	1.809674

RAG1	0.781614	0.163582	1.276966	2.40607
LRP4	0.781614	0.163674	-1.40911	6.571803
TSPAN11	0.781614	0.163745	0.833919	4.942411
R3HDML	0.781614	0.163749	1.815309	1.506726
GREB1L	0.781614	0.16377	-1.88119	2.570275
LINC01089	0.782491	0.164036	0.780656	4.78223
MEX3A	0.782491	0.164073	-1.05697	4.691248
MIR28	0.782841	0.164257	-2.71489	1.396344
CFL1	0.782841	0.164265	0.653382	9.117711
TMEM245	0.782862	0.164329	-0.68809	7.236724
ASIC1	0.783231	0.164474	-2.63729	0.751967
NGB	0.783231	0.164643	-3.61972	1.473787
MACROD1	0.783231	0.164661	0.924137	4.120617
ZNF217	0.783231	0.164682	-0.61654	7.301169
NINJ2	0.783231	0.164842	1.537728	2.158991
FER1L6-AS1	0.783231	0.164848	1.392418	4.282288
GNAT2	0.783231	0.164919	1.123533	3.197938
GBP3	0.783231	0.164924	0.93243	6.809263
JMJD6	0.783231	0.164946	-1.86881	3.245824
MTA3	0.783231	0.165004	1.6779	2.585388
NBPF25P	0.783581	0.165199	-0.75626	5.117473
CNOT1	0.783581	0.165249	1.369826	2.575128
GAS5-AS1	0.783581	0.165279	-2.26017	1.837827
DUSP23	0.783581	0.165316	-1.37901	3.634569
DOCK9-DT	0.784242	0.165516	-2.25753	1.832187
U2SURP	0.784297	0.165633	-0.60402	8.333073
ST6GALNAC	0.784297	0.165647	-1.11044	3.542754
BRI3	0.784435	0.165736	-0.99124	4.651438
PAQR8	0.784677	0.165975	0.651063	6.419162
SCARNA28	0.784677	0.16601	-1.00468	5.848172
BCAS2	0.784677	0.166037	-0.98814	5.125473
DKK1	0.784677	0.16614	-2.66577	3.482694
CEMIP2	0.784677	0.166249	-1.1284	3.470192
CASTOR3	0.784677	0.166305	-1.09086	5.153046
PAK4	0.784677	0.166315	2.321571	0.997927
ABCA1	0.784677	0.166357	-0.73113	7.611224
TRAK1	0.784677	0.166396	0.590349	6.959155
ABCC6	0.784677	0.166492	1.770787	2.493636
NONO	0.784677	0.166496	1.87003	1.446838
LDLRAD2	0.784677	0.166505	1.190774	2.938468
APLNR	0.78491	0.166614	1.09216	3.444153
NUDT3	0.785157	0.166726	1.125043	3.047357
GBP1P1	0.785282	0.166813	-1.7059	2.590992
GATB	0.785778	0.166978	0.896855	4.354692
HMGA2	0.786029	0.167241	-1.39723	3.438058

LINC01801	0.786029	0.16733	-2.73838	1.270093
RDH14	0.786029	0.167332	1.282394	2.471266
SGMS1	0.786029	0.167491	0.613475	6.021451
LOC285847	0.786029	0.167533	1.9183	2.148784
ZNF165	0.786029	0.167577	-1.19842	4.55635
RNF144B	0.786029	0.167602	-1.24523	5.85082
ARL5B	0.786029	0.167707	-0.95701	5.647816
PI3	0.786029	0.167737	1.676961	7.020967
UTP4	0.786029	0.167782	-1.27431	4.04657
ZSCAN23	0.786029	0.167924	-1.53383	3.110048
HOXB2	0.786029	0.167952	-0.90437	4.873284
CCDC141	0.786029	0.167992	-1.45371	4.213022
RNF123	0.786029	0.167992	0.612803	6.029462
SNORD133	0.786029	0.168013	-0.71185	8.942406
QSER1	0.786029	0.168073	-0.76924	7.173877
HK3	0.786029	0.168077	1.179496	3.198116
SKI	0.786029	0.168162	-0.61326	7.51912
APMAP	0.786029	0.168169	-0.62216	6.484438
HECTD3	0.78607	0.168255	0.600552	6.796785
MAP1A	0.78607	0.168298	-0.79644	6.824794
SNORA75	0.786198	0.168514	-0.96775	5.515566
AGO3	0.786198	0.168596	1.89651	1.947911
TMX1	0.786198	0.168678	0.629934	6.017029
KRTAP10-1	0.786198	0.168727	-4.00073	1.897956
SMLR1	0.786198	0.168759	1.797472	1.937179
ASCL1	0.786198	0.168796	2.129288	0.878807
LRP1B	0.786198	0.168797	-1.73145	3.244426
RNF217-AS	0.786198	0.168949	-2.54106	2.124634
CHST7	0.786198	0.169068	1.075928	3.66469
MIR4449	0.786198	0.169099	0.90913	4.975978
STAG1	0.786198	0.169136	-0.59371	7.293958
TONSL	0.786198	0.169143	-0.87774	4.875324
C9orf85	0.786198	0.169194	1.096635	4.115403
ISCA1	0.786198	0.169201	-0.7738	5.16111
DENND3	0.786198	0.169224	-0.68757	6.443262
COQ4	0.787297	0.169579	1.056158	3.386162
TUBA3FP	0.787297	0.169581	1.77928	0.996362
ATMIN	0.787575	0.169724	1.315449	2.254218
EPM2A	0.787575	0.16977	-1.96622	2.41059
MIR17HG	0.787575	0.169825	-1.30238	3.09238
KMT2E-AS1	0.787575	0.169881	0.632288	5.773822
PRRG4	0.787972	0.1701	0.891621	5.777239
PRDM8	0.787972	0.170147	-2.7826	1.122675
CXCR4	0.787972	0.1702	1.056421	2.851025
HSD17B12	0.787972	0.170209	-0.63997	6.748812

PXN	0.787972	0.170292	-1.46853	2.224245
PLEKHA3	0.787972	0.170398	-0.76442	5.124624
TTN-AS1	0.787972	0.170432	-2.07682	2.06927
CNNM3	0.787972	0.170598	1.787553	2.094471
CSDC2	0.787972	0.170607	-1.43056	2.565385
TRIM66	0.787972	0.170609	0.601316	6.433668
PRPF38A	0.787972	0.170627	-0.61554	6.465746
SPRR2F	0.788312	0.170761	-4.01505	3.000389
ATXN7L3B	0.788665	0.170897	0.685713	7.087428
DAPK2	0.789382	0.171113	1.117387	3.921397
GBP1	0.789763	0.171289	-0.93401	5.960626
TMPRSS3	0.789763	0.171316	-2.73101	0.938769
TNN	0.789813	0.171387	-1.89351	2.0016
ACTG1P20	0.790002	0.171535	-1.08957	3.521094
GPR108	0.790002	0.171548	-0.69865	5.684236
AJUBA	0.790011	0.171673	-2.57698	1.210512
UNC13D	0.790011	0.171696	-0.81454	5.85625
ARL15	0.790011	0.171731	0.778458	4.222276
SPAG5	0.790222	0.171879	-0.76737	5.360974
MRGBP	0.790222	0.171897	-0.91273	4.664449
PDLIM1	0.790997	0.17222	0.617926	7.690137
LOC101928	0.790997	0.172241	1.815825	1.916882
PREP	0.790997	0.172247	0.647352	6.476633
FAM95C	0.791038	0.172316	1.174399	4.071331
TIGIT	0.79162	0.172558	-1.58874	2.731702
LETM1	0.79162	0.172626	0.599355	7.072561
SIGLEC15	0.79162	0.172647	1.908458	1.685368
PDCD6	0.79162	0.172684	1.038146	3.54971
PTPN3	0.791641	0.172751	-2.09109	1.384306
LINC01278	0.791641	0.172809	-0.81777	5.783674
LRGUK	0.791707	0.172884	-1.698	2.998834
LATS1	0.792071	0.173084	2.091195	1.193604
ALS2	0.792071	0.173084	-0.66237	6.093459
RIPOR2	0.792218	0.173177	1.361694	2.617713
MAPK14	0.794195	0.173779	-1.82705	1.976761
LINC01141	0.794195	0.173788	2.852515	1.708885
ACSM6	0.794195	0.17382	1.518682	3.193421
H3F3AP4	0.794195	0.173851	0.658693	6.331247
PLEKHJ1	0.794335	0.174016	0.967463	4.055703
ASPM	0.794335	0.174056	-0.92817	5.627935
IRF6	0.794335	0.174063	1.531542	1.475556
PRCC	0.794723	0.174362	-0.67849	6.031199
CFAP20	0.794723	0.17446	0.843942	4.072058
LGALS3BP	0.794723	0.174525	0.628298	8.264782
TCIM	0.794723	0.17454	-1.18698	7.539801

KRT17	0.794723	0.174569	-2.02387	7.918616
MIR663A	0.794723	0.174619	1.053212	3.50288
KDM6B	0.794723	0.174689	-0.62861	7.350298
LINC00970	0.794723	0.174747	1.907518	0.912596
HS3ST3B1	0.794723	0.174758	-2.07299	1.7976
RNF183	0.794723	0.174779	1.867428	2.206177
KDM1B	0.794723	0.174847	-0.6373	6.135372
ACCS	0.794723	0.174875	1.351033	2.144036
DPF2	0.794908	0.174977	-0.68314	5.64765
GRAMD2A	0.794925	0.175041	1.080669	3.276408
CDC26	0.795432	0.175255	-0.96055	4.260689
PTPRE	0.795432	0.175332	-1.77136	2.398116
IFT46	0.795432	0.175334	1.686828	1.849096
KCNE2	0.795789	0.175474	-2.83225	2.354053
POLR1A	0.796339	0.175656	-0.60284	7.055936
LINC01934	0.796427	0.175736	-2.21085	0.633298
JPT2	0.796446	0.175808	-0.70908	7.207484
LRFN3	0.796446	0.175862	-1.23601	3.67825
C2CD4D-AS	0.797178	0.176142	-1.51157	3.461199
MALRD1	0.797178	0.176145	1.847362	4.778443
LOC100134	0.797559	0.176292	-2.5428	1.183008
MFSD4A	0.797559	0.176401	-1.10631	5.287972
C2orf16	0.797559	0.176454	-1.65176	2.586869
HACD2	0.797559	0.176472	-0.86387	4.701896
JPX	0.797964	0.176623	-1.01173	4.197976
LOC729603	0.798731	0.176853	0.865456	4.50814
TPTE2P1	0.798774	0.176948	-2.50034	1.481216
SNORD1C	0.798774	0.17704	0.895671	4.95715
SNORD74	0.798774	0.177117	-2.38253	0.978223
MIR23B	0.798774	0.177154	-2.77671	1.173142
CAPN6	0.798774	0.177218	-1.11188	5.256153
C11orf95	0.798774	0.177247	-0.81051	5.136219
NCCRP1	0.798774	0.177289	2.441737	4.326785
FAM220A	0.798909	0.17746	-1.49766	3.566893
RNA45SN5	0.798909	0.177488	0.693893	15.59404
MCRIP2	0.798909	0.177594	1.013782	3.886746
CALML5	0.798909	0.177621	-3.23667	3.631087
EPHA1	0.798909	0.177685	0.80102	5.215496
GPALPP1	0.798909	0.177814	-1.24462	3.612978
CDRT4	0.798909	0.17786	1.185823	3.020286
LIX1L	0.798909	0.17787	-0.78351	5.545848
LOC100379	0.798909	0.177926	-1.55521	1.745578
TMTC2	0.798909	0.177928	0.698562	5.73783
ZNF302	0.799376	0.178135	1.175743	3.0665
LINC00957	0.799376	0.178179	-1.90808	1.38466

EFNB3	0.799376	0.178215	1.522087	1.958107
TRAPPC13	0.800082	0.178433	-1.62718	2.12207
LINC02078	0.800326	0.178548	-2.53149	1.609809
RPRD1B	0.800446	0.178636	-0.6152	5.872797
LOC105447	0.800646	0.17882	2.048155	1.930115
ZSCAN20	0.800646	0.178836	-1.04097	4.254446
GNG11	0.800646	0.178939	0.875999	4.349108
SNX18	0.800646	0.178982	2.154812	1.880609
RAI1	0.800646	0.178986	-0.64548	6.702756
DES	0.800957	0.179161	-1.45488	9.572463
GPR52	0.800957	0.179234	1.189089	3.505139
MIR100HG	0.800957	0.179239	-1.01529	5.973283
PECR	0.801296	0.179436	0.875599	4.194652
ASAP1-IT1	0.801296	0.179437	1.171437	3.111694
FDXR	0.801322	0.179524	1.251239	3.095162
GPS1	0.801322	0.179574	1.226877	2.818242
LY86	0.801322	0.179718	1.48782	2.759612
ZNF543	0.801322	0.179774	-0.78271	4.960942
GUSBP3	0.801322	0.17981	1.325537	2.752125
GNS	0.801322	0.17983	-0.73165	7.527804
LINC01239	0.801322	0.179943	-2.5838	1.056749
OVOL2	0.801322	0.179985	1.416753	2.486296
TRIM52-AS	0.801322	0.180025	2.564543	1.270263
RRAGA	0.801322	0.180053	0.674634	5.130184
MELTF-AS1	0.801839	0.180335	-1.61323	2.295121
ATP5F1D	0.801839	0.180391	-2.13882	1.292728
RRAS	0.801839	0.180466	-0.96092	4.412232
PALB2	0.801839	0.180632	-0.86069	5.08914
LOC100133	0.801839	0.180695	1.028231	2.98923
SP3	0.801839	0.180703	2.252363	1.471932
SASH1	0.801839	0.180756	0.608853	7.286261
SDHAF3	0.801839	0.180816	1.060961	4.46041
LGALS9C	0.801839	0.180852	1.717997	1.411463
PHLPP1	0.801839	0.180899	0.686736	5.413511
MED13	0.801839	0.181116	-0.59368	8.441984
MBOAT1	0.801839	0.181118	-1.53043	2.522832
STEAP3	0.801839	0.181127	1.483414	2.169074
LINC02487	0.801839	0.181182	2.222851	4.106271
UBE2V1	0.801839	0.181192	1.813808	1.322696
ZCCHC24	0.801839	0.181233	-0.77208	5.983549
BCAP31	0.801839	0.181405	-2.18418	0.941754
RNF128	0.801839	0.18172	0.87643	4.238998
ADGRF3	0.801839	0.181724	-2.04323	1.640783
COX7A2	0.801839	0.181743	1.539549	1.793196
IGFBP4	0.801839	0.181869	-0.80277	8.460693

TXNRD1	0.801839	0.181889	1.741416	1.418877
ELF2	0.801839	0.182129	-0.94857	4.118403
LINC01843	0.801839	0.182133	1.77502	1.626411
HCG26	0.801839	0.182174	0.932606	4.054352
TMBIM1	0.801839	0.182194	-0.7411	8.138801
KLHL26	0.801839	0.182212	-1.19047	3.447316
CLYBL	0.801839	0.182225	1.928998	0.859231
CYB5D2	0.801839	0.182226	1.469007	2.133878
DLSTP1	0.801839	0.182401	0.919883	3.733273
LINC01623	0.801839	0.182471	3.161572	0.943892
FIGN	0.801839	0.182514	-1.3841	3.596316
SCN1B	0.801839	0.182575	-1.77603	1.22401
LOC100507	0.801839	0.182691	0.839987	4.442264
ALDH9A1	0.801839	0.182733	0.613404	6.432817
LOC100128	0.801839	0.182806	-1.65182	2.342502
AP4B1-AS1	0.801839	0.182864	1.560295	2.903538
EPHA1-AS1	0.801839	0.182869	1.040711	3.744041
RFTN1	0.801839	0.182874	-0.78986	6.343956
SCML1	0.801839	0.182883	2.826512	0.649754
CSF1	0.801839	0.182934	1.919317	1.471789
IL1RAPL1	0.801839	0.182943	1.745439	1.927593
MCM5	0.801839	0.182969	-0.72811	5.040101
RPS10P7	0.801839	0.182999	1.034254	3.074012
PRR16	0.801839	0.183052	-2.695	1.118489
CDH6	0.801839	0.183135	0.921158	5.386054
ZSCAN25	0.801839	0.183155	-0.68333	5.361314
MIR3681H(	0.801839	0.183199	-3.2601	1.156943
ELOVL5	0.801839	0.183206	-1.39554	2.81886
TXLNGY	0.801839	0.18325	1.348669	5.641398
ORAI1	0.801839	0.183287	-1.61888	2.280165
PSMC1	0.801839	0.183347	-0.96988	5.0362
RBM4B	0.802803	0.183629	-1.62269	3.004126
SERPINF1	0.803769	0.183981	-0.76006	6.442564
LOC10027(	0.803769	0.184031	-2.04998	1.471864
MTRNR2L3	0.803769	0.184083	-2.38451	1.406723
ANAPC1	0.803769	0.184095	-0.62738	6.096516
GNAO1	0.804136	0.184419	-1.27847	3.58455
PURA	0.804136	0.184469	-0.81465	6.956614
LOXL1	0.804136	0.1845	-0.82231	4.660009
AVPI1	0.804136	0.184705	-1.21948	4.227139
PGM2L1	0.804136	0.184738	-0.81868	6.416784
HID1	0.804136	0.184762	0.836528	6.889455
DNAJC9	0.804136	0.184833	-0.69259	5.607177
ZNF451	0.804136	0.184876	-0.65448	6.131696
ABCA4	0.804136	0.184969	1.439611	1.811802

DENND2A	0.804136	0.18497	0.742474	4.945457
WDR13	0.804136	0.184971	0.947957	3.409622
SET	0.804136	0.184979	0.867563	4.193663
SF3A3	0.804136	0.185019	-0.60068	7.209867
BRF2	0.804136	0.185037	-1.05563	3.911575
APOD	0.804145	0.185127	-1.2859	5.643521
SNORD14C	0.804145	0.185162	-0.85812	8.242125
PAXIP1-AS2	0.80453	0.185327	-2.12044	2.921053
LONRF1	0.80453	0.185373	-1.08927	5.414324
SRD5A3	0.804716	0.185546	0.797871	4.833227
LOC100499	0.804716	0.185588	1.70457	1.42807
CHKA	0.804716	0.185643	1.996104	1.800657
MACF1	0.804716	0.185665	-0.59597	10.64048
FRS3	0.804716	0.185731	-1.50934	2.500877
TFAP2E	0.804716	0.185784	-1.63997	2.876414
IL23R	0.804851	0.18607	2.029319	1.6027
MIR4442	0.804851	0.186094	1.141556	2.248717
LINC01279	0.804851	0.186215	-1.15392	5.883709
RBBP8NL	0.804851	0.186289	1.461191	2.180408
OSM	0.804851	0.186327	-2.40685	2.08454
ATXN1	0.804851	0.186343	-2.40972	1.000106
CLHC1	0.804851	0.186346	-1.58152	3.414458
CLK4	0.804851	0.186352	0.672034	6.010706
SLC10A1	0.804851	0.1864	1.048043	2.984231
DDX18	0.804851	0.186429	-0.60837	7.118619
SNORD10	0.805297	0.186593	-0.74299	10.88865
MLLT1	0.805389	0.186789	-0.59903	6.515654
TYMS	0.805389	0.186795	-1.02532	4.235441
LINC00877	0.805389	0.186799	-2.18072	1.952543
MIR1183	0.805442	0.186872	-2.50966	1.38214
TNFRSF10A	0.805586	0.186983	-0.88681	4.783101
MIR662	0.805586	0.187028	-3.24868	0.486108
CBX2	0.805698	0.187143	-1.56075	2.505787
ABCB6	0.805698	0.187177	-0.73618	4.776033
C1R	0.805798	0.187262	-0.80668	8.68755
CYP2J2	0.805877	0.187342	0.995302	3.436092
CTSK	0.806649	0.187706	-0.90113	6.172894
INHA	0.806649	0.187745	-3.67855	0.647686
ERI1	0.806649	0.187827	-0.73218	5.17585
EXOC8	0.806649	0.187899	-0.62078	5.958413
SNORD114	0.806649	0.1879	-1.53535	3.207087
TRIAP1	0.806649	0.187951	-0.94524	3.980371
ASB1	0.806649	0.187952	-0.60514	5.84822
RPL23A	0.806719	0.18803	-0.65192	7.482937
CBLN3	0.806985	0.188193	1.359675	2.816884

SNHG5	0.806985	0.188253	0.699518	5.800896
LURAP1	0.806985	0.188276	-1.84084	1.759357
HS3ST3A1	0.807189	0.188385	-1.52834	2.393222
LINC01630	0.807374	0.18849	-3.95287	1.281655
SRF	0.807862	0.188702	0.718511	5.335323
MT1M	0.807862	0.188871	1.342766	3.427999
CHRNE	0.807862	0.188874	-1.86847	1.990154
RBM17	0.807862	0.188923	0.990541	3.659109
ATP23	0.807862	0.18894	3.040421	0.754687
OARD1	0.807862	0.189008	0.653973	5.191335
PROX2	0.807862	0.189035	-2.70996	1.216916
ZKSCAN2	0.808064	0.189241	-0.86172	4.681398
SNX9	0.808064	0.189319	-0.60876	7.638582
TRPM8	0.808064	0.189354	-2.21096	1.158614
TOX3	0.808064	0.189389	1.254378	3.541144
FOXK1	0.808064	0.189395	-0.64358	8.056657
C2CD3	0.808064	0.189452	-1.03988	4.32382
TCTEX1D2	0.809144	0.189767	1.546529	1.726759
SPTLC2	0.809184	0.189939	0.553858	7.353739
SNORA38	0.809184	0.189989	-1.92205	2.205924
PCGF6	0.809184	0.190029	-2.66591	0.997632
ZNF326	0.809184	0.190053	0.551814	6.787312
RPP25	0.809184	0.190084	0.818041	4.321232
PYCR2	0.810385	0.190455	-2.3153	0.756906
ATP13A4	0.810385	0.19056	1.170602	4.174298
VPS13A	0.810385	0.190653	1.121782	4.11863
PRSS8	0.810385	0.190657	0.680327	6.433875
LILRA6	0.810385	0.190725	-2.24301	1.461791
ZNF266	0.810385	0.190905	2.096799	0.980023
LOC72867	0.810385	0.190909	-1.94189	1.630296
SMIM25	0.810385	0.190948	-1.93314	1.589847
RRBP1	0.810385	0.190981	2.283171	1.23366
SNORD90	0.810385	0.191038	-1.24538	4.737458
LPGAT1	0.810385	0.191046	-0.64044	7.491748
PIGP	0.810459	0.191192	1.760644	1.232727
LOC101927	0.810459	0.191287	1.951638	1.344305
CT83	0.810459	0.191307	1.900847	3.107268
GPATCH2	0.810459	0.191342	-0.68875	5.150211
EBLN2	0.810459	0.191372	0.850559	3.926211
9-Sep	0.810569	0.19146	-0.66655	6.011389
SCARNA9	0.81133	0.191712	-0.6595	7.167518
LOC28400	0.81133	0.191797	1.730495	1.793966
LOC10192	0.81133	0.191895	1.805049	2.360893
RPS27A	0.81133	0.192085	1.368425	1.88957
ATXN1L	0.81133	0.192167	-0.60244	6.872933

BICDL2	0.81133	0.192331	0.70211	6.06182
ADNP2	0.81133	0.192477	-0.62803	6.081095
FGF11	0.81133	0.192589	2.028557	1.960392
EGR1	0.81133	0.192631	-0.77608	8.123585
GTPBP6	0.81133	0.192635	1.156405	3.080442
CABP7	0.81133	0.192653	1.971218	0.996628
APBA1	0.81133	0.192654	1.126261	2.750537
IL17RB	0.81133	0.192725	-0.95727	4.362834
FGD4	0.81133	0.19273	0.993649	3.262143
RNLS	0.81133	0.192731	1.428808	2.291486
ARL14EP	0.81133	0.192763	-0.90028	4.591814
ETV6	0.81133	0.192768	-0.57653	7.32556
GTPBP1	0.81133	0.192783	-0.58333	6.732705
UBXN7	0.81133	0.192815	-0.60688	6.787397
BAHCC1	0.811493	0.192959	-1.08131	5.530343
TMEM269	0.811493	0.192999	-2.28885	0.95701
BHLHE41	0.811493	0.193039	0.727173	6.400264
LOC221946	0.81276	0.19352	0.939648	4.271311
PRICKLE2-A	0.81276	0.193527	-1.56323	3.415916
DERA	0.81276	0.19353	1.478234	2.81397
PLCXD1	0.81276	0.193633	1.197938	3.329395
UBE2V2	0.81276	0.19365	-0.77981	5.368157
HIST1H4J	0.812953	0.193798	1.294951	2.555546
LINC00102	0.812953	0.193833	2.13395	1.16667
PSMC2	0.812953	0.193882	-0.67362	5.833525
LINC01814	0.812958	0.193975	0.91221	4.964349
LDLRAD4-A	0.812958	0.194008	-1.14949	3.67727
THSD7B	0.812958	0.194069	-1.60479	2.662479
LOC728752	0.813197	0.194238	1.382517	2.210071
GLI2	0.813197	0.194342	-0.9183	4.408779
LINC00989	0.813197	0.194399	2.311449	1.569483
CDCA5	0.813197	0.194544	-1.13321	4.223079
RASGRP3	0.813197	0.194586	-2.85509	1.289773
RASSF10	0.813197	0.194674	1.074873	4.017823
REG1A	0.813197	0.194679	-1.84471	6.025707
PANDAR	0.813197	0.194742	1.374339	2.368331
RPS6KA1	0.813197	0.194872	1.351549	2.40046
NDUFV2	0.813197	0.194876	-0.80787	6.598528
EIF2AK3	0.813197	0.194895	0.815294	4.296343
ABALON	0.813197	0.194999	-1.29906	2.746134
BTN3A1	0.813197	0.195079	1.508672	1.807969
SNX29P2	0.813197	0.195139	-1.07987	3.164129
FAM110D	0.813197	0.195142	2.050851	0.9431
ZNFX1	0.813197	0.195178	-0.57405	8.255054
GPKOW	0.813197	0.19518	-0.71511	4.752238

RAB11FIP2	0.813226	0.195311	-0.67769	6.438808
MRPL36	0.813226	0.195365	-0.99403	3.349633
WNT4	0.813226	0.195373	0.974529	3.882936
CC2D1A	0.813392	0.195474	0.57948	6.511547
RPTN	0.813402	0.195564	-2.16165	3.021248
TLR3	0.813402	0.195601	1.221379	3.005549
TRIM16	0.813824	0.195944	0.629968	5.995162
IFFO2	0.813824	0.195997	-0.7641	5.71933
RRM1	0.813824	0.196023	-0.59347	6.305879
TRIM59	0.813824	0.196045	-0.82807	4.203404
LIF	0.813824	0.196094	-1.84259	2.037184
SCFD2	0.813824	0.196119	0.628036	5.0632
LRRC58	0.813824	0.196156	-0.81196	6.867979
CRACR2B	0.813824	0.196199	1.110025	2.924313
KRT78	0.813931	0.196286	2.823157	2.21182
PCNP	0.814046	0.196376	-0.72538	6.542581
SOX12	0.81411	0.196454	-0.71873	5.145042
SLC45A4	0.814189	0.19658	-1.00191	4.374508
LNX1-AS1	0.814189	0.196597	2.054708	1.216563
EME2	0.814209	0.19681	0.979718	3.87745
VSTM2L	0.814209	0.19683	-1.95592	3.119544
GVQW3	0.814209	0.196844	0.900057	3.871145
SCMH1	0.814209	0.19685	-1.69441	2.523617
LINC00896	0.814459	0.196996	1.108361	2.904081
LIMK2	0.814459	0.197055	0.862052	4.446756
RAB12	0.814459	0.197107	-0.75246	6.035437
ALDH3A1	0.814459	0.197159	2.045054	1.020508
ARX	0.814733	0.197306	1.518279	3.515591
BLOC1S3	0.814733	0.197349	-0.94339	4.113921
IPO5	0.815125	0.197506	-0.65543	7.822187
TEN1-CDK3	0.815213	0.19759	1.826407	1.106456
CTD-2350J1	0.81523	0.197707	1.976602	1.36687
BMP7	0.81523	0.197728	-1.88035	4.529639
TMCC1-AS1	0.81523	0.19778	-1.88659	1.851385
PSD3	0.815267	0.197851	0.666079	6.347622
ADSS	0.815395	0.197961	-0.65324	6.419682
ELFN1	0.815395	0.198077	-2.41397	0.920895
INPP5F	0.815395	0.198095	-0.78204	4.508312
CTAGE15	0.815395	0.198214	-2.89538	0.941585
ARHGAP1	0.815395	0.198221	-0.57877	7.342542
THRIL	0.815395	0.198311	0.856289	4.471568
COPZ1	0.815395	0.198361	1.861238	1.837966
GMCL1	0.815395	0.198434	-0.68265	4.973058
XAB2	0.815395	0.198539	-0.63286	5.818632
SNORD82	0.815395	0.198558	-1.1115	3.889904

C4orf19	0.815395	0.198615	1.429011	2.229981
PNMA2	0.815395	0.198628	-1.04484	4.411375
ENDOV	0.815399	0.198713	1.814592	1.268184
WDR4	0.815399	0.198849	-2.78118	2.31945
DSG1	0.815399	0.198867	1.989768	3.836411
LINC02289	0.815399	0.198937	-2.58582	0.709413
RPL32P3	0.815399	0.19894	0.872626	4.308068
SOCS5	0.815712	0.19925	2.475378	1.217616
ARGFXP2	0.815712	0.199362	-1.41424	2.93142
BEAN1-AS1	0.815712	0.199364	-3.15255	1.005016
CCNC	0.815712	0.19961	1.080126	3.054974
NUTM2F	0.815712	0.199669	1.649834	1.635824
TTLL13P	0.815712	0.199743	-1.90879	1.957807
PPARGC1A	0.815712	0.199745	-0.85622	4.816162
SLC6A14	0.815712	0.199768	-1.19272	6.355587
ABCC13	0.815712	0.199852	2.263776	1.666603
TRAIP	0.815712	0.199887	-1.49815	2.790172
DIXDC1	0.815712	0.199909	-1.66276	1.601311
GZMB	0.815712	0.199951	-1.93246	2.720202
PZP	0.815712	0.200067	0.970572	4.373124
TGFBI	0.815712	0.200098	-0.75519	7.072705
CLCN3	0.815712	0.200106	1.416041	2.092979
CRYL1	0.815712	0.200107	0.705712	4.862242
PLXNA2	0.815712	0.200129	0.520334	7.616381
MTRNR2L8	0.815712	0.200206	-0.94081	5.587929
AREG	0.815712	0.200226	-1.20406	5.063395
LRCH3	0.815712	0.200264	-0.60164	6.329734
UBE2L6	0.815712	0.200322	2.126794	1.310339
RXRA	0.816314	0.200639	1.107789	2.715267
MATN3	0.816314	0.200674	-1.78969	2.289094
ALK	0.816314	0.200714	-1.60693	2.477535
ERVK13-1	0.816314	0.200739	-0.66524	6.83473
TMEM163	0.816314	0.200781	-1.10292	3.758473
LTV1	0.816347	0.200897	-0.77866	6.231605
NPTX1	0.816347	0.200966	-2.25346	1.862112
CDC20	0.816347	0.200986	-0.97411	4.532307
C2CD4B	0.816347	0.201038	1.222408	2.368581
GGA2	0.816574	0.201172	-0.58022	6.78358
SLC25A6	0.816574	0.201486	1.191872	6.429793
LOC10012[illegible]	0.816574	0.201536	-2.12879	1.045079
STPG2	0.816574	0.20156	1.855436	1.392456
ERICH1	0.816574	0.201573	-1.24496	3.381713
GABRE	0.816574	0.201719	-1.45616	4.842717
CLCA3P	0.816574	0.201758	1.376005	2.471575
FER	0.816574	0.201761	1.474026	2.756012

SMG5	0.816574	0.20179	-0.57082	7.528017
GXYLT2	0.816574	0.20183	-1.43298	3.181969
ETNK1	0.816574	0.201858	0.530868	7.650325
SMG1	0.816574	0.202009	-0.5627	9.341753
MAPK15	0.816574	0.202035	-1.0191	3.663179
MAML3	0.816574	0.202074	0.597729	6.092381
TRIM36	0.816574	0.202084	2.101764	0.872018
PRC1-AS1	0.816574	0.20209	-2.3645	1.11937
C9orf170	0.816927	0.202239	2.129971	1.408806
CLN5	0.817419	0.202438	-0.77439	4.653553
LCMT1-AS2	0.817419	0.202495	0.924873	3.822689
WDPCP	0.817419	0.202644	-1.25917	2.635985
SF3B4	0.817419	0.202653	-0.64481	6.015996
GAN	0.817419	0.202672	0.586567	5.759552
AMIGO2	0.817839	0.202839	-3.18282	0.861818
SNHG11	0.818038	0.202951	1.279044	2.868374
PTPRG	0.818151	0.203041	0.545894	7.344827
METRN	0.818248	0.203128	-1.69359	2.709809
NDN	0.81864	0.203298	-1.05022	3.46344
SNORD68	0.81864	0.203465	-1.08903	5.249268
CMIP	0.81864	0.203533	0.851409	4.168014
LINC02585	0.81864	0.203603	-2.40695	0.886845
RBMS2	0.81864	0.203701	-0.61508	7.146204
METTL8	0.81864	0.203704	-0.67737	6.159049
FGF7P3	0.81864	0.203793	-1.5132	2.57922
CDK6	0.81864	0.203805	-2.26309	1.912698
ZNF331	0.81864	0.204159	2.332423	0.917702
MDFI	0.81864	0.204239	-2.28007	1.84825
PMAIP1	0.81864	0.204291	-1.2234	4.327415
PPP1R14A	0.81864	0.204315	-2.80954	1.225105
FAT2	0.81864	0.204344	1.157244	5.983116
ZNF765	0.81864	0.204376	-0.66774	5.288041
DPP9-AS1	0.81864	0.204424	1.125116	3.556445
GIPC2	0.81864	0.20443	1.073581	2.461798
TNFAIP1	0.81864	0.204614	-0.59038	5.976641
NT5DC3	0.81864	0.204645	-0.667	6.585186
NME9	0.81864	0.204865	0.651509	5.487173
ADAMTSL1	0.81864	0.20493	-1.32408	3.546812
ABCA7	0.81864	0.20506	-0.70742	5.766161
HIST1H1A	0.81864	0.2051	1.445119	1.904729
CCT6P1	0.81864	0.205278	-0.89485	4.169164
OSBPL5	0.81864	0.205305	1.803261	1.152128
NME8	0.81864	0.205479	-2.28867	1.113288
SNAI3	0.81864	0.20548	1.436657	2.359692
NIPA2	0.81864	0.205621	2.133833	1.233708

KIF7	0.81864	0.205712	-0.95577	4.604947
CPSF6	0.81864	0.205775	1.515418	2.578737
KNTC1	0.81864	0.205794	-0.68938	6.519824
SPATA45	0.81864	0.205836	2.942755	0.82057
VGF	0.81864	0.205994	1.813155	1.297613
KCNA1	0.81864	0.206015	2.290599	1.627442
CFAP157	0.81864	0.206154	-1.43099	2.619131
MARCKS	0.81864	0.206196	0.577028	8.074832
FADS2	0.81864	0.206198	-1.68056	1.993714
ADD3	0.81864	0.206267	-1.65901	1.794696
FTO	0.81864	0.206272	-0.64567	6.422118
SERPINB3	0.81864	0.206282	2.70562	6.5467
LOC101927	0.81864	0.206296	-1.37407	2.986925
PTPRZ1	0.81864	0.206302	1.28925	2.352079
EML4	0.81864	0.206303	1.026015	3.336989
CYP4F22	0.81864	0.206363	2.048467	2.234458
IL11RA	0.81864	0.206393	1.685587	1.805488
ZNF398	0.81864	0.206393	-1.72061	2.034972
LEAP2	0.81864	0.206398	1.77211	1.352111
LOC101927	0.81864	0.206425	-2.41051	0.612662
KCNMB1	0.81864	0.206524	-1.32045	4.365292
PLEKHG6	0.81864	0.206559	1.876947	1.75512
THSD4-AS1	0.81864	0.206584	2.085116	1.32305
FHL5	0.81864	0.206597	-2.57129	0.916184
CIP2A	0.81864	0.206614	-0.80558	4.658288
C3orf52	0.81864	0.20677	-2.13861	0.969234
CGRRF1	0.81864	0.207084	1.337402	3.016489
RPL3	0.81864	0.207111	0.67514	5.337125
PWARSN	0.81864	0.20725	-1.21738	4.014195
TBX19	0.81864	0.207277	1.103065	2.996012
FTL	0.81864	0.20729	-0.55918	10.0106
ACOX3	0.81864	0.207394	1.85107	1.400012
PPP2R2D	0.81864	0.207415	-1.22642	3.641759
CAPN10	0.81864	0.207483	-1.81333	1.363917
PRKAR2B	0.81864	0.207495	0.732985	5.403726
IGDCC4	0.81864	0.207531	0.799474	4.490003
S100A11	0.81864	0.207613	-0.76132	7.790588
MCM9	0.81864	0.207663	0.608728	5.629406
LNCTAM34	0.81864	0.207744	1.388668	2.649984
FOXC1	0.81864	0.207863	-0.82446	4.926876
MPPED2	0.81864	0.207882	-2.75252	1.139667
REEP6	0.81864	0.2079	1.004393	3.657569
KAZN	0.81864	0.207955	-1.45108	2.190899
DDA1	0.81864	0.207966	-0.70991	5.518781
PDZD7	0.81864	0.207994	-2.28188	1.453374

MRPL27	0.81864	0.208013	0.721187	4.475233
SPOPL	0.81864	0.208127	-0.57693	6.092518
GCM2	0.81864	0.208181	-2.77084	0.592102
ESYT3	0.81864	0.208251	-0.97772	3.770676
CYYR1	0.81864	0.208304	1.198022	3.764038
CDT1	0.81864	0.208322	-0.91731	3.763079
RNF4	0.81864	0.208336	1.30232	2.344737
ANXA2	0.81864	0.208357	-1.21352	3.64448
DIPK2B	0.81864	0.20842	0.950227	4.598654
CAT	0.81864	0.208442	0.582637	6.600273
CRTAP	0.81864	0.208458	-0.58105	7.723169
POLR3A	0.81864	0.208516	-0.57135	6.118614
KANK1	0.81864	0.208547	0.930084	3.004949
PSMB1	0.81864	0.208591	-0.60561	6.129584
GPN1	0.819096	0.208786	-2.60697	0.93966
MFGE8	0.819096	0.208862	-1.82177	1.745456
BRWD3	0.819096	0.208894	-0.56706	7.405682
GABARAPL	0.819843	0.209229	-0.69335	6.092439
GLRX5	0.819843	0.209422	1.006568	4.629122
HLF	0.819843	0.209427	-1.22442	4.976884
TSC22D2	0.819843	0.209656	2.023245	2.175007
SLC25A45	0.819843	0.20967	1.620968	1.629037
SRGAP2D	0.819843	0.209678	-0.67072	6.598645
BAIAP2L1	0.819843	0.209723	-0.6821	7.819977
ATIC	0.819843	0.209878	-0.69578	5.953835
FASTKD3	0.819843	0.209944	-1.57894	2.326236
CKAP5	0.819843	0.210015	-1.1512	2.43092
LOC44120⁴	0.819843	0.210043	1.494015	2.029307
HP09053	0.819843	0.210095	-2.19077	1.004741
TOP1P1	0.819843	0.210165	-0.78404	4.408175
C1orf115	0.819843	0.210225	0.670968	5.905437
H3F3C	0.819843	0.210233	-2.2561	1.652575
ZBTB20-AS	0.819843	0.21025	-2.05994	1.650746
FLJ42102	0.819843	0.210279	2.744288	0.846943
SON	0.819843	0.210286	-0.65088	5.193128
SLC19A3	0.819843	0.210473	-1.60901	3.829504
CAMK2N2	0.819843	0.210498	-1.70148	1.739232
DACH1	0.819843	0.210536	1.875207	1.691224
SRSF9	0.819843	0.210545	0.595637	6.095969
FAT3	0.819843	0.210605	-1.60012	4.626678
PTPRVP	0.819843	0.210648	-2.37328	1.26594
IGFBP7-AS:	0.819843	0.210672	2.131172	1.795104
SNORA74B	0.819843	0.210719	-0.86866	5.479795
ZNF383	0.819843	0.210799	-1.20282	3.537657
RNU5E-1	0.819843	0.210835	-0.99497	7.121414

CIR1	0.819911	0.211001	-0.58566	6.404679
TSPAN5	0.819911	0.211011	0.823646	4.19049
LYPLA2	0.819911	0.21104	-0.68955	5.362132
USP46-AS1	0.820337	0.211339	-1.43884	2.18205
PARP6	0.820337	0.21136	-0.71334	5.847545
RNU6ATAC	0.820337	0.211386	-0.92221	5.620612
LINC02541	0.820337	0.211399	-2.82324	1.295765
ZNF394	0.820429	0.211486	-0.66073	5.788278
SLC6A17	0.820806	0.211672	-2.05871	1.176621
PRRT1	0.820806	0.211708	1.427282	1.545099
JDP2	0.82092	0.2118	1.618925	1.196887
HIST1H2BD	0.82094	0.211868	1.16935	2.920949
CDC6	0.821917	0.212196	-0.95109	5.02877
AKAP12	0.821917	0.212275	-1.29853	2.962911
DDX47	0.821917	0.212355	-2.62151	1.071745
CPLANE1	0.821917	0.21239	-0.69284	7.041501
NAV1	0.821917	0.212453	-0.93795	3.395951
SNHG1	0.821917	0.212497	-0.86806	5.044079
NEXN	0.821917	0.212606	-1.93116	2.159264
TMEM255E	0.821917	0.212621	-1.4008	3.14856
SPATA24	0.82217	0.212749	1.182492	2.251158
LINC01140	0.822382	0.212867	2.048929	0.827278
OSBPL10	0.822541	0.213263	-1.10721	2.720508
DEK	0.822541	0.213383	1.148527	2.431793
OTOP2	0.822541	0.213423	2.41305	1.440443
COX6B1	0.822541	0.213521	0.579274	6.829237
ZRANB2-AS	0.822541	0.213601	-2.24626	1.062249
GCOM1	0.822541	0.213633	-1.81192	2.687587
PRPF39	0.822541	0.213641	-0.58776	6.05675
CLDN1	0.822541	0.213654	-0.65959	5.918217
MAGED2	0.822541	0.213663	1.528071	1.04222
TREML3P	0.822541	0.213717	2.186578	1.021212
HIST1H1D	0.822541	0.213769	-0.60778	5.672228
CSGALNAC	0.822541	0.213836	-1.6241	2.591225
MAP3K21	0.822541	0.213962	-0.75271	5.692923
PLVAP	0.822541	0.213999	0.712785	6.381578
SLC25A37	0.822541	0.214056	-0.97365	3.649475
ITGA3	0.822541	0.214142	-0.61638	7.729279
NUAK1	0.822541	0.214165	0.717683	5.158599
PRRX2	0.822541	0.214198	-1.55203	2.620276
PPP3CB-AS	0.822541	0.214322	1.544564	2.713862
POLA1	0.822541	0.214395	-0.67132	5.857133
BAG2	0.822541	0.214449	-0.79479	4.462539
KRT7	0.822541	0.214451	-1.00109	7.978444
TNFRSF13C	0.822541	0.214473	1.894118	1.592031

XPO5	0.822541	0.214566	-0.61916	6.339273
AVEN	0.822541	0.214587	0.926367	4.735173
MCTS1	0.822541	0.214608	1.469333	1.81824
MINDY3	0.822541	0.214717	-0.68627	5.051635
VTRNA2-1	0.822541	0.214746	-0.78783	7.095108
JMJD1C	0.822541	0.214907	-0.84382	4.196274
TM9SF3	0.822541	0.215043	0.560509	9.697136
HPDL	0.822541	0.215144	1.11193	2.978042
CEP41	0.822541	0.215157	-2.82455	0.748171
PRRC2C	0.822541	0.21521	-0.5583	10.83479
PAGR1	0.822541	0.215242	0.572949	6.789954
SERPINA5	0.822541	0.215405	-2.3696	2.813993
DUXAP8	0.822541	0.215436	-1.46156	3.497037
ZCCHC14	0.822541	0.215532	-0.55993	6.986259
SLC22A15	0.822541	0.21554	0.714711	4.690318
C8orf59	0.822541	0.215543	2.322484	1.10146
ADO	0.822541	0.215655	-0.90138	5.124913
HTRA3	0.822541	0.215664	-1.15052	3.520404
F12	0.822541	0.2157	1.23828	2.411951
ATF4	0.822541	0.21572	0.871818	3.51873
PNPLA6	0.822541	0.215731	-2.14152	0.987958
ARMC6	0.822541	0.215732	-2.1493	1.127699
LOC100506	0.822541	0.215792	0.899982	3.826152
MMP9	0.822646	0.215963	-1.52894	3.695798
LINC02035	0.822646	0.21604	-0.83196	4.748059
MMRN1	0.822646	0.216099	1.561494	5.066093
ERCC6L2	0.822646	0.216183	-0.56304	6.150288
GADD45B	0.822646	0.216244	-1.08688	5.436156
RRN3P2	0.822646	0.216492	-0.75881	5.281952
MIR548AQ	0.822646	0.216522	1.887092	1.555764
CXCL6	0.822646	0.216583	-2.02189	2.974506
ALKAL1	0.822646	0.216715	-2.22802	1.414433
GALNT6	0.822646	0.216839	0.659866	6.924244
LLGL1	0.822646	0.216846	0.75522	4.386589
MYOZ1	0.822646	0.216862	1.753258	1.609314
QPCTL	0.822646	0.216907	1.877953	0.92223
CST7	0.822646	0.216982	-1.52929	3.804496
FRMPD4	0.822646	0.216995	2.216005	1.410844
PRRG1	0.822646	0.217013	-1.76757	1.470725
SH2B1	0.822646	0.217055	0.677458	5.142531
ANXA2R	0.822646	0.217283	1.389639	2.133728
CACYBP	0.822646	0.217381	-1.40601	2.592125
SERINC1	0.822646	0.217476	-0.61668	7.997691
COIL	0.822646	0.217596	-0.75482	5.63686
MAN2A1	0.822646	0.217617	0.517786	7.193588

LINC00623	0.822646	0.217636	-2.30951	1.22409
SNORA20	0.822646	0.217688	-0.75045	8.650366
IPO13	0.822646	0.217693	-0.66581	5.532322
INTS4P2	0.822646	0.217729	0.940647	3.950418
ANAPC15	0.822646	0.217763	2.248572	0.737876
MFSD9	0.822646	0.217819	-0.71053	4.895633
PRR15	0.822646	0.217857	0.680684	5.549677
IL13RA1	0.822646	0.217986	-0.56355	7.144247
CDCP2	0.822646	0.218079	2.081307	1.269168
STRIP2	0.822646	0.218119	-2.30835	3.674153
TERF2IP	0.822646	0.218181	-0.5515	6.58044
RTN4R	0.822646	0.218379	1.314322	2.350781
OBSCN	0.822646	0.218425	0.992412	4.006918
PURB	0.822646	0.218435	-0.56508	7.503075
LOC642943	0.822646	0.218475	-2.96539	1.180777
SLC7A4	0.822646	0.218554	-2.53306	1.531765
PRKAG2-AS	0.822646	0.218645	1.352136	2.189139
DPPA4	0.822646	0.218652	2.437192	0.802709
CEBPZ	0.822646	0.218715	-0.59533	6.934578
HS2ST1	0.822646	0.218753	-0.79462	5.548081
PRKXP1	0.822646	0.218767	-0.76329	4.881126
DYNLL1	0.822646	0.218826	1.80428	0.838487
TEF	0.822646	0.218876	1.619257	1.552946
LOC100506	0.822646	0.21892	-1.24902	2.726537
ZNF704	0.822646	0.219107	-0.86331	6.339934
CCDC9	0.822646	0.219138	-0.57612	6.595614
C1orf21	0.822646	0.219283	0.599866	6.979285
SOBP	0.822646	0.219298	-0.9162	4.867833
ATP5PD	0.822646	0.219417	-2.14272	1.120341
ZNF512	0.822646	0.21946	1.705609	1.122863
CHN1	0.822646	0.21947	-2.52447	1.09827
SOX4	0.822646	0.21955	0.624791	7.985624
CNIH4	0.822646	0.219568	-1.64712	2.144893
PER2	0.822646	0.219576	-0.58151	7.003358
PECAM1	0.822646	0.219651	0.621251	8.144939
POLR2I	0.822646	0.219703	-0.78842	4.052321
C2orf88	0.822646	0.219841	2.146637	0.978787
ETFRF1	0.822646	0.219858	0.754011	4.293014
PHGDH	0.822646	0.219889	-0.86207	4.454032
CHRNA3	0.822646	0.219915	1.831204	1.471789
APCDD1	0.822646	0.219924	-1.25606	6.484848
FAM161B	0.822646	0.220066	0.842016	3.848749
LINC00222	0.822646	0.220132	1.815911	1.39945
IDNK	0.822646	0.220137	-1.90068	1.86823
TNFAIP8L2	0.822646	0.220268	-1.55643	2.101577

MAP1LC3A	0.822646	0.220369	-1.29052	2.535542
PANK4	0.822646	0.220429	1.43525	1.068103
TMEM123	0.822646	0.220457	-0.60803	8.106855
LAMB1	0.822646	0.220476	0.553867	9.055141
RHEB	0.822646	0.220513	-1.01452	5.00387
ENPP1	0.822646	0.220588	-0.83291	4.549653
CCDC136	0.822646	0.220708	-1.08082	3.898752
RIC1	0.822646	0.220779	2.588699	0.916386
USP6NL	0.822646	0.220786	-1.72557	2.570631
HCG4B	0.822646	0.220802	1.43511	2.280815
KLF13	0.822646	0.220864	-0.89152	4.148143
NKG7	0.822646	0.220934	-1.44851	2.335646
FRY-AS1	0.822646	0.220966	1.225301	2.331554
TPX2	0.822646	0.221027	-0.76094	6.287456
TLN2	0.822646	0.221447	0.641645	7.633442
NPY6R	0.822646	0.22151	1.216676	3.382836
TMEM38A	0.822646	0.221574	1.319051	2.373095
DKFZp451E	0.822646	0.221659	2.199645	1.051864
NXF1	0.822646	0.22172	0.589623	5.495309
EFNA3	0.822646	0.221733	-1.3725	4.049079
GGT1	0.822646	0.221789	1.627266	1.286544
TTLL3	0.822646	0.221809	0.990282	3.086885
RBM19	0.822646	0.22195	0.698561	4.132645
ZDHHC7	0.822646	0.221952	1.510752	1.541896
PAX8-AS1	0.822646	0.22211	-0.61135	7.081115
PCDH12	0.822646	0.222134	-0.86172	4.226347
MACC1	0.822646	0.222176	-0.62226	8.24533
AKR7A2	0.822646	0.222186	0.62416	5.137621
SMAD4	0.822646	0.222188	0.519294	7.135992
EPHB4	0.822646	0.222285	0.630466	6.950298
NSUN5P2	0.822646	0.222286	1.236371	2.310396
SLC25A34	0.822646	0.222339	0.965047	2.920624
MCM8-AS1	0.822646	0.222453	-2.04989	1.294844
PMS2P1	0.822646	0.222501	0.677801	4.665622
GPR158	0.822646	0.22254	-2.49639	2.223657
CAPS2	0.822646	0.222601	-1.75935	1.827875
OR5C1	0.822646	0.22275	1.811853	1.379806
5-Mar	0.822646	0.222776	-0.72894	5.375573
GTF2IRD2	0.822646	0.222806	1.540946	2.10154
CHERP	0.822646	0.222851	-0.6035	6.276987
SUZ12P1	0.822646	0.222879	-0.76911	4.615549
KIAA1211L	0.822646	0.222932	0.598725	5.358143
DIS3	0.822646	0.222998	-1.6089	2.364073
SCP2	0.822646	0.223074	1.11634	3.705788
CD160	0.822646	0.223149	-2.51913	2.276287

CA1	0.822646	0.223171	2.248183	1.50036
PSMC3	0.822646	0.22322	-0.53444	6.434545
SCAP	0.822646	0.223238	0.605642	6.473752
PRSS33	0.822646	0.223305	-2.96546	2.789694
CPT1A	0.822646	0.223504	0.560047	6.584902
NDUFS8	0.822646	0.22362	-0.74479	4.361991
SP4	0.822646	0.223638	-1.00876	4.772306
LINC00665	0.822646	0.223738	-2.51824	1.638586
LINC00336	0.822646	0.223752	1.692088	1.957379
HTR6	0.822646	0.223768	-1.87301	1.545552
CHAD	0.822646	0.223831	0.802284	4.184279
SULT1C4	0.822646	0.223966	-1.38807	2.922899
FAM86C2P	0.822646	0.224127	-1.39776	2.999604
HMG20B	0.822646	0.224152	0.54607	6.704242
MAPK8	0.822646	0.224188	-1.84878	0.959175
GTF3A	0.822646	0.224196	-0.6919	5.493492
MEGF9	0.822646	0.224214	0.623143	5.870129
E2F3	0.822646	0.224229	1.421443	2.137356
SEC22B	0.822646	0.224301	0.527919	6.650582
MICAL2	0.822646	0.224412	-2.13434	0.811495
MEIS3P1	0.822646	0.224456	-0.89838	4.157856
LINC00235	0.822646	0.224496	1.76674	1.221993
RAPGEF6	0.822646	0.224506	1.109438	3.050305
OLFML2A	0.822646	0.224523	1.690117	2.353994
MIR21	0.822646	0.224579	-1.05209	3.072488
PFN1P2	0.822646	0.224597	-0.79814	4.595862
LAMB2	0.822646	0.224629	-0.55195	7.945993
KCNJ2	0.822646	0.224719	-0.97745	3.873519
WNT3	0.822646	0.224881	-1.73097	1.429047
LOC100506	0.822646	0.224886	-1.1406	3.307276
PSMB5	0.822646	0.224927	-2.42498	1.571929
TGIF1	0.822646	0.224993	-1.01347	3.679929
HEG1	0.822646	0.225015	-0.68572	7.233876
TMEM74B	0.822646	0.225133	-2.72224	1.399599
FAM27E3	0.822646	0.22524	0.861152	3.66111
SEC24D	0.822646	0.225249	-0.61076	6.860063
TSSK6	0.822646	0.225261	-1.89983	1.06935
H6PD	0.822646	0.225266	1.024431	2.600117
CARMIL3	0.822646	0.225287	-1.49676	2.687517
ZXDC	0.824285	0.225799	-0.82361	4.747362
CAVIN4	0.824357	0.22593	-2.14497	1.55809
PTPRJ	0.824357	0.225985	-0.62011	7.128139
SNHG22	0.824357	0.226082	-1.57354	2.482525
IMPDH2	0.824357	0.226105	0.629464	7.204461
LINC00907	0.824357	0.226133	2.053505	2.075488

BTAF1	0.824363	0.226197	-0.52723	7.970281
HNRNPM	0.824424	0.226277	2.17496	1.075797
MCAM	0.824729	0.226429	-0.67815	7.270156
LOC10537ε	0.824729	0.226527	-2.18633	0.983489
HPS3	0.824729	0.226591	-0.80591	3.870125
LINC02476	0.824729	0.226612	2.313223	1.297064
KCTD21	0.824743	0.226679	-1.03391	3.622635
CYP4Z1	0.825174	0.226918	-2.14899	0.647818
TBC1D9	0.825174	0.226933	-0.55691	5.948947
S100A9	0.825174	0.227086	1.7132	7.877919
ARL1	0.825174	0.227343	-1.34105	2.640204
MAP4K3-D	0.825174	0.227404	0.82285	4.497005
FAHD2CP	0.825174	0.227407	-1.93951	1.296881
SCYL3	0.825174	0.227452	-1.70366	1.711376
NELFE	0.825174	0.227477	-1.48958	2.04307
NNT-AS1	0.825174	0.227517	0.805091	4.748081
VOPP1	0.825174	0.227532	0.936511	2.619676
CEMP1	0.825174	0.227543	0.969724	2.877808
UHRF1	0.825174	0.227653	1.591434	1.235989
KAT2A	0.825174	0.227719	-0.58144	6.033372
PLPP6	0.825174	0.227739	0.693112	4.865476
MGC16025	0.825174	0.227741	-1.67143	3.13725
SPEN	0.82525	0.227824	-0.53066	9.116723
RBP5	0.825953	0.228081	-0.95364	4.137692
PFDN4	0.82598	0.228256	-1.15896	4.019056
RNU5D-1	0.82598	0.228263	-0.9983	4.373338
CRYM-AS1	0.82598	0.228278	-1.48425	2.405272
MIR4458H(	0.826067	0.228411	1.070673	3.019766
MRPL38	0.826067	0.228547	0.562944	5.305781
BRAP	0.826067	0.228589	-0.55866	5.79148
LINC02198	0.826067	0.228589	-3.60038	1.006643
MIR6124	0.826067	0.228631	1.523291	1.586892
SRRM2-AS1	0.826067	0.228744	-2.08745	0.956002
FBN3	0.826067	0.228796	1.697959	2.437941
SPSB1	0.826067	0.228853	-0.69064	5.277679
PDCL3P4	0.826067	0.228939	-0.97797	3.625147
SNORD114	0.826067	0.229071	-1.54925	2.514827
PHIP	0.826067	0.229074	-0.53286	8.71974
ERVW-1	0.826067	0.229079	-2.03343	1.118811
MICU3	0.826067	0.229158	-1.18055	3.449583
CLK2P1	0.826067	0.229239	-1.58296	2.136936
PGAP1	0.826067	0.229246	0.537925	6.263727
PIK3C2B	0.826081	0.229336	0.585194	7.430452
HIST1H4K	0.826081	0.229422	-1.51853	4.208443
PHF13	0.826081	0.229478	-0.78175	4.543552

SNORA18	0.826081	0.229503	-0.60655	7.33768
MIR200A	0.826081	0.229615	-1.74397	1.959011
GATA2	0.826081	0.229648	1.810122	0.652897
LBR	0.826081	0.22972	2.013312	1.066144
ZDHHC11	0.826081	0.229776	0.881389	3.731365
ATP6V1C1	0.826081	0.229827	-0.58998	6.342282
SPACA6	0.826081	0.22988	-1.02386	4.613569
CEP72	0.826187	0.229972	-1.07557	5.360276
ATXN7L1	0.826431	0.230103	1.030102	3.608451
LOC100505	0.826605	0.230221	-1.66035	2.670383
CLTB	0.826605	0.2303	1.231796	2.625242
SPINK7	0.826605	0.23039	2.130431	3.206891
TEX264	0.826605	0.230404	1.793373	0.923971
SORT1	0.826747	0.230506	1.094789	2.374532
LINC00920	0.826797	0.230585	-1.81427	1.466816
PSME4	0.826797	0.230646	-0.5188	7.77299
RBM23	0.826822	0.23081	-1.65535	1.75015
TLCD2	0.826822	0.230956	0.714909	6.809219
GTF3C3	0.826822	0.231123	-0.71723	4.61241
LOC645513	0.826822	0.231137	-0.8792	4.038361
TREML4	0.826822	0.231185	-2.72443	0.69189
MAB21L3	0.826822	0.231212	1.411028	1.879566
ELF4	0.826822	0.231241	-1.77364	1.449235
HAUS5	0.826822	0.231272	-0.76691	4.649401
PKD1P6-NI	0.826822	0.231302	-0.78476	4.586025
TCP11L1	0.826822	0.231316	-2.44528	0.538858
DLL4	0.826822	0.231349	0.799649	3.872182
CLEC3B	0.826822	0.231475	1.027592	2.652112
VCPIP1	0.826822	0.231511	-0.55156	7.118101
BCDIN3D	0.826822	0.231577	-1.47661	2.113658
ZNF71	0.826822	0.231598	-1.46304	3.243853
RAB40A	0.827167	0.231837	1.493658	2.063174
SHLD1	0.827167	0.231869	1.802292	1.090169
ACO2	0.827167	0.231938	0.583915	6.59936
PTDSS2	0.827167	0.231995	-1.39216	2.301603
RACGAP1	0.827167	0.23201	1.498591	1.446542
TNFRSF17	0.828157	0.232399	1.391418	2.823779
LOC200772	0.828157	0.232482	-2.4924	2.178406
VSIG10L	0.828157	0.232511	1.431423	4.700971
MORN3	0.828157	0.23254	-1.69049	1.78932
GTPBP8	0.828222	0.232686	2.025695	0.861848
ERMN	0.828222	0.232689	2.713679	1.094635
SNORD100	0.828222	0.232748	-1.04751	4.168878
PDLIM2	0.828244	0.232833	1.061775	3.069815
MGC2889	0.828244	0.232881	-2.92908	0.678611

BPTF	0.828291	0.232957	-0.74883	4.203766
PFKFB3	0.828402	0.233132	-1.24955	2.58391
GALK1	0.828402	0.233151	1.008504	3.402908
SDHA	0.828402	0.233178	-1.79022	1.239362
ARMC5	0.828424	0.233267	-2.05559	1.39439
ANP32C	0.828424	0.23331	1.502021	1.773033
CD7	0.828747	0.233464	-1.55167	2.909447
FOXO1	0.828962	0.233619	-0.64681	6.943536
ADCK2	0.828962	0.233871	0.824473	4.509005
ZNF514	0.828962	0.233896	-1.39509	2.832579
RAD18	0.828962	0.233974	-0.66431	5.405079
TAF9B	0.828962	0.234022	-0.76779	5.100947
RDH10-AS1	0.828962	0.23407	0.653863	4.95883
PACS1	0.828962	0.23415	-0.53496	6.641999
ZNF703	0.828962	0.234199	-0.78907	6.748887
DPY30	0.828962	0.234226	-0.87647	4.123315
YWHAQ	0.828962	0.23426	-0.54712	7.333368
HSF2BP	0.828962	0.234272	-1.80229	2.009904
SNORD2	0.828962	0.234283	-0.80248	4.861873
IARS	0.83059	0.234981	1.650752	1.70976
DLEU2	0.83059	0.235001	0.674417	4.559767
TRIM29	0.83059	0.23504	-1.09727	7.611599
ZC3H15	0.83059	0.235079	-0.52512	7.461731
CYP1B1	0.83059	0.235172	-1.23538	4.500887
SWSAP1	0.83059	0.23521	-1.86022	1.457194
KPNB1	0.83059	0.235287	-0.96611	3.049244
ANKRA2	0.83059	0.235294	0.556907	5.597425
HAND2	0.83059	0.23532	-1.88319	3.15948
DDX20	0.83059	0.235437	-0.73228	4.733978
RPE65	0.83059	0.235482	-2.4872	1.038361
ZNF813	0.83059	0.23551	-1.42267	3.145792
ST8SIA6	0.83059	0.235566	2.519015	0.85171
PSMD4	0.831186	0.235798	-0.52229	6.996708
GRID1	0.831333	0.235903	1.251409	2.230496
TSPY26P	0.831347	0.236027	-1.62901	1.539767
LINC00548	0.831347	0.236034	2.024307	1.025715
OSCP1	0.831377	0.236106	-1.4582	3.139215
OCLM	0.831575	0.236411	-1.66023	2.775246
SEPSECS	0.831575	0.236517	0.708254	5.106112
POLR3D	0.831575	0.236556	-0.64868	6.281415
NSD1	0.831575	0.236588	0.973405	3.44484
FBXO22	0.831575	0.23661	-1.99118	2.121015
LAMA5	0.831575	0.236659	-0.61682	8.133482
CERK	0.831575	0.236718	0.498286	6.562646
ZFP30	0.831575	0.236727	-0.82554	4.311894

TMEM139	0.831575	0.236733	1.742019	0.695101
RSRP1	0.831597	0.236802	0.511138	6.730257
LAMTOR5	0.83213	0.237017	0.729456	4.680884
RCBTB1	0.832702	0.237329	-0.63821	5.64332
UQCRH	0.832702	0.237368	-2.30515	0.65114
FEN1	0.832702	0.237453	-0.86636	4.616445
CEACAM8	0.832702	0.237455	-2.71942	2.435528
CAVIN3	0.832702	0.237528	-0.86428	3.847546
PYROXD2	0.832702	0.237615	0.782276	3.841854
RASGRF2	0.832702	0.237836	0.752774	5.137359
USP53	0.832702	0.237847	-0.55263	7.677184
RNF180	0.832702	0.237897	-1.44484	3.395116
RNF213	0.832702	0.237917	-0.57347	9.992921
VPS53	0.832702	0.238018	0.574003	6.439286
SIRPG-AS1	0.832702	0.238046	1.671844	1.51209
NAA35	0.832702	0.238056	-0.54901	6.216356
RAD54L2	0.832702	0.238081	-0.59658	6.074951
TMEM41B	0.832702	0.23858	0.574083	5.659329
ASNA1	0.832702	0.238665	-0.90913	4.290768
SUDS3	0.832702	0.238928	-0.54432	7.10795
GLTPD2	0.832702	0.238992	1.071999	2.635778
SEMA3A	0.832702	0.239051	-1.11796	5.301091
DGKH	0.832702	0.239185	-1.04912	3.391173
TNRC18	0.832702	0.23928	-0.52057	9.385681
RBKS	0.832702	0.239293	1.473391	0.845703
UBASH3B	0.832702	0.239317	-1.01385	4.208115
MSANTD4	0.832702	0.239441	-0.93504	5.586111
RFX1	0.832702	0.239524	-0.78059	4.488145
RRN3P3	0.832702	0.239567	-0.71201	4.493126
FRAT2	0.832702	0.239618	0.586274	5.65039
NUMBL	0.832702	0.239661	1.727854	1.107723
FSIP2	0.832702	0.239679	0.627058	6.377578
LINC00311	0.832702	0.239724	1.607458	1.806598
VPS4B	0.832702	0.239841	0.586495	7.084773
ZNF833P	0.832702	0.239875	-1.89171	1.380622
NRG1	0.832702	0.240138	1.54377	1.979947
BTBD6	0.832702	0.240167	0.557787	5.481008
RRP7A	0.832702	0.240233	-0.66011	5.077871
EMC7	0.832702	0.240269	0.523259	5.738535
CREBRF	0.832702	0.240279	-0.77449	6.028802
GRK3	0.832702	0.240303	-0.73893	5.456739
TBC1D31	0.832702	0.240358	-2.17889	1.260117
RBM26-AS1	0.832702	0.240508	-1.77979	1.524657
ETFB	0.832702	0.240537	1.767179	1.487659
TMEM43	0.832702	0.240655	-0.56862	7.10679

MPLKIP	0.832702	0.240661	-0.79877	4.422557
KBTBD2	0.832702	0.240691	-0.51795	6.413366
KRBA1	0.832702	0.240694	-2.56987	1.051128
MTMR10	0.832702	0.240701	-0.91208	4.079905
PGM3	0.832702	0.240911	-1.94817	2.004783
TTLL7	0.832702	0.240972	-1.15908	4.42743
LMOD1	0.832702	0.240995	-1.00861	8.373322
BRD4	0.832702	0.241209	-0.52872	6.713873
MCUB	0.832702	0.241252	-0.88012	3.299978
ZNF385C	0.832702	0.241269	-1.30298	2.156187
LOC101927	0.832702	0.241342	-1.35746	2.141694
DSP	0.832702	0.24137	0.748481	9.344973
FGF10	0.832702	0.241408	1.59727	1.757551
BACH1-IT2	0.832702	0.241445	-1.44339	2.771888
SFRP5	0.832702	0.241493	-2.50674	1.234592
ZMYM4	0.832702	0.241544	-0.53311	7.775035
ABHD2	0.832702	0.241578	0.975797	4.19747
TINAGL1	0.832702	0.24164	-1.40692	2.349739
MREG	0.832702	0.241692	1.080279	4.18582
DPM2	0.832702	0.241715	-0.81753	4.108733
STX17-AS1	0.832702	0.241724	-2.77293	0.580088
TPTE2P6	0.832702	0.241893	-2.03991	1.175493
LAMA1	0.832702	0.241965	-1.45145	3.34519
ZNF223	0.832702	0.241975	-1.0771	4.165111
C1orf112	0.832702	0.241978	-0.88621	4.026449
CD28	0.832702	0.242049	-2.73679	0.892393
KPNA4	0.832702	0.24212	-0.54896	7.812141
ZNF136	0.832702	0.242156	-1.05438	4.239812
THCAT155	0.832702	0.242244	2.087985	1.695834
ZNF367	0.832702	0.242345	-0.80967	4.356193
ZBTB20-AS	0.832702	0.242352	-1.75007	1.981654
FAS-AS1	0.832702	0.242449	1.72905	2.062738
C16orf91	0.832702	0.24245	-1.12084	2.74024
LINC01169	0.832702	0.242459	-2.7868	0.98458
DENND1A	0.832702	0.242471	-0.71551	4.271152
CDR2L	0.832702	0.242551	-0.62587	6.053291
RAP2C-AS1	0.832702	0.242554	1.028869	3.36008
MPC2	0.832702	0.242648	1.383895	1.501356
S100Z	0.832702	0.242677	2.065284	1.313599
CAND1	0.832702	0.242703	-0.50494	7.432055
CTD-2201I:	0.832702	0.242734	-1.68278	2.824815
NDUFB10	0.832702	0.242756	-0.55961	6.026658
RASA4B	0.832702	0.242817	0.958665	3.362909
RSF1	0.832702	0.242857	-0.53329	8.134072
IPP	0.832702	0.242979	-0.98808	3.188452

PCDH17	0.832702	0.24305	-0.87009	4.258348
MUC17	0.832702	0.243052	1.877457	2.594751
LOC10012¹	0.832702	0.243059	0.835634	3.992327
SDHAP2	0.832702	0.243081	0.503975	6.007719
ABHD12	0.832702	0.243113	0.604571	4.695693
CCDC85C	0.832702	0.243131	0.576168	5.53573
SHB	0.832702	0.243147	-0.54484	6.249836
LINC01473	0.832752	0.243225	-1.33062	2.342296
IL1A	0.832774	0.243294	-1.62541	3.898998
ZW10	0.833144	0.243466	-0.64209	5.362549
LOC34009(	0.83344	0.243616	1.573785	2.46147
ELOA	0.834282	0.244026	-0.56833	6.72588
SF3B5	0.834282	0.244084	0.823607	4.631329
CECR7	0.834282	0.244094	-1.78151	2.499055
IGF1	0.834282	0.244259	-1.9896	2.067517
NDUFV2-A!	0.834282	0.244353	-2.00345	2.191222
TMEM45A	0.834282	0.244423	1.115602	3.218345
ASH1L	0.834282	0.244424	-0.53254	9.311227
HERC5	0.834282	0.244475	-1.0374	4.732254
PTP4A2	0.834282	0.244572	0.702155	5.297203
LINGO3	0.834282	0.244582	1.268237	1.894045
BAIAP2-DT	0.834282	0.244686	-0.73324	4.323989
SHD	0.834282	0.244687	1.720701	1.44676
DNAJB14	0.834282	0.244743	1.273218	1.720332
NANS	0.834282	0.244752	0.593218	6.384353
PIEZO2	0.834318	0.244826	0.729196	6.007867
EXTL2	0.834766	0.245022	-2.38122	0.959659
NFX1	0.834831	0.245104	-0.66894	4.926865
RMRP	0.835027	0.245322	-0.51027	12.37385
KLLN	0.835027	0.245438	1.063557	3.386074
ZNF287	0.835027	0.245472	-1.14823	3.418897
PRDM7	0.835027	0.245474	1.729651	1.63117
RNF26	0.835027	0.245527	-0.62418	5.206135
LPP-AS1	0.835027	0.24555	-1.04772	3.15695
SCARNA5	0.835027	0.245648	-0.75183	8.625675
HSPE1	0.835027	0.245671	-1.00481	3.071231
SAP18	0.835581	0.245898	-0.56116	6.769587
DOT1L	0.835797	0.246025	-0.55891	6.861269
MAB21L4	0.835923	0.2462	2.267775	1.069405
TNFAIP8L3	0.835923	0.24623	-3.31279	0.598532
MEGF11	0.835923	0.246331	0.74953	3.852632
MAMDC4	0.835923	0.246366	0.567298	5.331758
TMEM161/	0.835923	0.246418	-1.90576	0.918355
TSC22D1	0.835923	0.246445	-0.67758	5.762661
MED15	0.83598	0.246525	0.851905	3.223526

PTGER3	0.836114	0.246687	-1.42181	3.654729
ZNF90	0.836114	0.246747	-1.72548	1.977605
SLC5A3	0.836114	0.246756	-0.52375	7.052014
METTL25	0.836912	0.247055	1.091809	3.128907
PKHD1L1	0.837065	0.247175	0.913033	4.075821
CYP2B6	0.837065	0.247264	1.611884	2.052153
SUPT6H	0.837065	0.247291	-0.49786	8.783822
OAF	0.837441	0.247466	0.513732	5.915168
PELI1	0.837591	0.247784	-0.53119	7.30058
LOC64898	0.837591	0.247824	1.54368	1.44636
MAP3K10	0.837591	0.248022	0.64192	4.930363
LARP1B	0.837591	0.248133	-0.72653	4.230034
BTBD19	0.837591	0.248134	-0.9161	3.997392
AADAC	0.837591	0.24829	1.240348	3.635641
CDC42BPG	0.837591	0.248419	0.701657	6.304256
GABARAP	0.837591	0.248457	0.505091	6.8526
FAM25A	0.837591	0.248535	2.104749	2.304063
HERPUD2	0.837591	0.248572	-0.55657	5.963134
ADGRF2	0.837591	0.248581	2.1435	1.170839
QPRT	0.837591	0.248594	1.098875	2.892006
6-Mar	0.837591	0.248617	-2.07374	0.84722
MAL2	0.837591	0.248637	-0.6472	7.740362
HCRTR1	0.837591	0.248638	-1.77783	1.18804
PDCD11	0.837591	0.248639	-0.5868	7.059165
HCFC1	0.837591	0.248664	-0.50057	7.6192
IDH1	0.837591	0.248738	0.756287	3.753372
IGFBP5	0.837591	0.248777	0.606496	10.80327
HOXD9	0.837591	0.248787	-2.41765	1.173985
RNF149	0.838354	0.249135	-0.63375	6.778938
KCNK3	0.838354	0.249148	0.939025	2.904544
FRMD6-AS	0.838354	0.2493	2.242897	1.162693
CCDC154	0.838354	0.249378	-1.56507	2.585437
DGKE	0.838354	0.249419	-0.82566	5.19176
SS18L1	0.838354	0.249443	1.576043	1.141837
TBCE	0.838354	0.249461	2.199277	0.773053
SCAF8	0.838598	0.249635	0.921038	3.206787
GS1-124K5	0.838598	0.249662	-1.30904	3.472048
EIF4EBP3	0.83865	0.249919	-1.9185	0.805933
ZNF853	0.83865	0.249967	-1.37802	3.205258
AGBL1	0.83865	0.249981	2.246407	1.756417
ZBTB5	0.83865	0.250012	-0.58331	5.499401
SLA	0.83865	0.250016	1.231867	2.392655
CTBS	0.83865	0.250061	0.719429	4.704552
ZHX2	0.839143	0.250284	-0.5421	5.885369
OAS1	0.839143	0.250336	1.215989	3.294957

ANKS1A	0.839346	0.25046	-0.53362	6.982962
KRT75	0.839376	0.250533	2.028796	0.80326
ATP1A1	0.839402	0.250605	0.752216	5.577068
BCL2L14	0.839591	0.250726	1.694935	1.07635
FIGNL1	0.839902	0.250882	-1.44396	3.033654
HOXB3	0.840767	0.251356	-0.73476	6.203587
NBPF15	0.840767	0.251393	-1.60196	3.071079
TMEM115	0.840767	0.251396	0.545117	5.111492
FOCAD-AS:	0.840767	0.251478	2.048251	1.33156
PRRC1	0.840767	0.251495	0.578537	5.28771
COL6A3	0.840767	0.251538	-0.74732	4.38585
LOC101927	0.840767	0.251589	2.03403	1.085567
ZNF148	0.841231	0.251792	-0.51569	7.413963
HLCS	0.841432	0.251965	1.303705	2.018251
FMO6P	0.841432	0.25206	1.469703	1.868711
FAM155A-I	0.841432	0.252263	1.179974	3.820632
MN1	0.841432	0.252438	-1.082	5.0508
LUC7L	0.841432	0.252471	-1.43061	2.0229
OMG	0.841432	0.252545	-1.18205	3.29335
TRIM9	0.841432	0.252566	-1.65733	2.023479
SMIM3	0.841432	0.252643	-0.85025	4.505886
ANKIB1	0.841432	0.25267	-0.54692	7.152625
ADAMTS7	0.841432	0.252671	0.671045	3.986318
SH3D21	0.841432	0.252679	1.259836	2.397066
RGMB	0.841432	0.252695	-0.6729	6.220991
MYH3	0.841432	0.25275	-1.14707	3.973545
ANKRD28	0.841432	0.25275	1.097259	3.073743
ITPRIPL2	0.841456	0.252821	0.720257	3.96267
FZD7	0.841551	0.25297	-0.85942	4.592222
NDFIP1	0.841551	0.253111	-0.6298	7.435451
NEXMIF	0.841551	0.253122	-2.23572	2.171298
SLC36A4	0.841551	0.253165	1.28522	2.182856
ARHGAP29	0.841551	0.253297	0.514278	5.985142
LINC00937	0.841551	0.253339	1.653584	1.350857
THNSL2	0.841551	0.253348	-1.8691	0.765856
ANKRD20A	0.841551	0.253363	-1.28769	4.062025
LAG3	0.841562	0.25346	-1.49566	2.251476
XRCC5	0.841562	0.253495	-0.50562	8.570443
CTAGE6	0.841623	0.253577	-2.1379	0.715277
SIGLEC8	0.841626	0.25373	1.61708	1.444503
FAM83A	0.841626	0.253755	1.717835	2.75751
IWS1	0.841626	0.25378	-0.51805	7.954493
SNAPC4	0.841626	0.253906	-0.55778	5.767744
WDR6	0.841626	0.253935	0.502298	7.106367
SLC8A2	0.841626	0.254071	-2.45006	0.945688

SMIM13	0.841626	0.254077	0.763424	4.517374
GCNT2	0.841626	0.254098	0.879706	4.734704
UTP25	0.841626	0.254156	-0.55217	6.169787
PROSER1	0.841869	0.254308	-0.55941	6.573671
MIB2	0.841869	0.254357	1.558417	1.931242
SREK1	0.842263	0.254541	-0.56888	6.001697
PSPH	0.842281	0.25471	-0.74916	4.515751
MAP1LC3C	0.842281	0.254722	-2.94915	0.696248
ELFN1-AS1	0.842281	0.25484	-2.37088	0.583494
CLDN12	0.842281	0.254858	1.387795	1.928974
PCDHB14	0.842281	0.254878	-1.0729	4.814645
ZNF425	0.842281	0.255124	-1.30328	2.280625
LINC00538	0.842281	0.255186	-3.438	1.3839
STON2	0.842281	0.255225	0.575172	5.527247
FANCA	0.842281	0.255242	-2.19137	1.250191
TLN1	0.842281	0.255286	-0.54512	9.700949
LOC100506	0.842281	0.25529	1.028484	3.078313
SNORD12	0.842281	0.255333	-0.7703	6.793993
PSMD6	0.842281	0.255381	-2.20156	1.718057
UBFD1	0.842348	0.255465	-0.55092	6.345807
CD48	0.842419	0.255551	-1.95059	1.393054
FABP5	0.842637	0.255691	1.190002	6.775841
DENND5B	0.842637	0.255793	1.107775	2.22047
RALGPS1	0.842637	0.255903	0.575833	4.930855
MTSS1L	0.842637	0.255916	-0.61387	6.616504
ZDHHC1	0.842637	0.255938	0.899191	3.553595
SLC35E4	0.842909	0.256085	-1.39759	3.270623
CHP1	0.842975	0.25622	0.548294	7.597145
SLC1A5	0.842975	0.25625	-0.85193	5.577317
HTRA2	0.842975	0.256298	1.49704	1.312346
MCM7	0.843054	0.256395	0.883549	3.222932
RRAGD	0.843054	0.25645	-0.81063	4.293701
PPP1R3F	0.843272	0.256582	1.233598	1.976494
LINC01133	0.843272	0.25673	0.798956	6.723694
CWC27	0.843272	0.25678	-1.14208	3.113483
PLEKHH2	0.843272	0.256845	-0.83282	7.180628
BLOC1S5	0.843272	0.256867	1.886854	1.18047
PSORS1C1	0.843272	0.257057	-2.16527	1.257947
PUS7	0.843272	0.257087	-0.66512	5.817502
SLC38A6	0.843272	0.257092	-1.53189	1.946111
CXADRP2	0.843272	0.257165	1.517485	2.112703
PI4K2A	0.843272	0.257283	-0.72087	5.297132
SLC35D1	0.843272	0.257299	0.541281	6.02799
SF3A2	0.843272	0.257317	-0.57693	6.012367
FAM13B	0.843272	0.257363	-1.79633	1.716316

SETBP1	0.843272	0.257417	-0.74048	6.274151
PPP2R5C	0.843387	0.257548	1.018627	3.316092
THRAP3	0.843387	0.257594	-0.49741	9.036207
SDC1	0.843387	0.257797	1.341707	1.46617
SNAI2	0.843387	0.257997	0.739697	5.166908
HACE1	0.843387	0.258073	-1.66673	1.589054
PPP1R36	0.843387	0.258113	1.182596	3.180635
AATK	0.843387	0.258368	-1.97172	0.766008
KCNJ12	0.843387	0.258424	-2.27734	1.983644
MMD	0.843387	0.258471	-0.83892	4.140865
RPS3A	0.843387	0.258506	0.613918	5.617741
DOCK10	0.843387	0.258518	1.205623	1.990259
ZNF256	0.843387	0.258553	-1.86001	2.47229
TPTEP1	0.843387	0.258595	1.182191	2.459797
PRR7-AS1	0.843387	0.258602	1.552461	1.069407
UQCC2	0.843387	0.258624	-0.74249	4.779163
SLC46A3	0.843387	0.258644	1.348402	2.351953
RNF169	0.843387	0.258713	-0.5167	7.339154
DNAJC30	0.843387	0.258724	-0.87832	3.433697
DDI1	0.843387	0.25877	2.067198	1.008503
RCCD1	0.843387	0.258927	-2.32919	0.923918
MARVELD1	0.843387	0.258959	-0.63681	5.763544
CMTR1	0.843387	0.258963	-0.54941	6.799672
IL12A-AS1	0.843387	0.258992	1.834436	0.819876
EPN3	0.843387	0.259011	0.776774	5.096998
RAB32	0.843387	0.259059	-1.25524	3.439534
CLOCK	0.843923	0.259399	-1.14887	2.763471
SHANK1	0.843923	0.259455	-1.62901	1.026698
CXCL9	0.843923	0.259534	-1.27406	3.597263
FBXL7	0.843923	0.259542	1.131522	2.316821
FYB2	0.843923	0.259704	-2.63661	2.036893
TTBK2	0.843923	0.25989	-0.93585	5.326651
SMAD2	0.843923	0.259929	0.989614	2.219797
TTC30A	0.843923	0.259932	-1.0349	3.719396
SNORD36C	0.843923	0.259955	-1.37776	3.217652
PML	0.843923	0.259957	-0.94668	4.712514
PRSS3	0.843923	0.259958	1.574786	1.296351
YBX3P1	0.843923	0.259995	-0.85497	3.155729
CLDN16	0.843949	0.260149	-2.8116	0.750602
SMTN	0.843949	0.260222	0.963077	2.45506
PCBD2	0.843949	0.260303	0.687845	4.963519
USP9Y	0.843949	0.260312	1.053439	6.434294
ABCB8	0.843949	0.26034	-2.05448	1.231604
SEL1L3	0.843949	0.260488	1.385997	1.635023
SYNJ2-IT1	0.843949	0.260539	-1.4187	1.969958

SLFN5	0.843949	0.260542	0.506541	6.72972
DLG5	0.843949	0.260582	-0.86515	4.449576
AFDN-DT	0.844129	0.260819	-1.40835	2.17887
CABLES1	0.844129	0.260958	1.137049	3.266196
NUP210	0.844129	0.261024	-0.68143	6.137392
CREBL2	0.844129	0.261102	-0.56396	6.351614
PKHD1	0.844129	0.261105	-1.68155	2.740208
ATP13A3	0.844129	0.261184	-0.55464	7.906748
RAD21	0.844129	0.261191	-0.57173	8.560596
PABPC5	0.844129	0.261258	-1.81336	1.378205
PSG2	0.844129	0.261283	-2.94994	0.603796
PRELID2	0.844129	0.261344	1.88198	1.235545
CCDC171	0.844129	0.261346	0.70429	4.86607
JMJD8	0.844559	0.261543	-0.81318	4.153869
PRPF31	0.844727	0.261659	-1.48965	1.774497
ZNF701	0.845194	0.26189	1.279321	2.475272
SLC26A10	0.845194	0.261933	-1.47911	2.331547
HIVEP1	0.84594	0.262229	-0.60476	6.942173
ANLN	0.846259	0.262392	-2.19053	1.105795
ANKDD1B	0.8466	0.2626	1.121983	2.371183
ELOB	0.8466	0.262627	1.704027	1.17518
SLC9A5	0.846766	0.262743	-1.0437	2.973292
PCDHB9	0.846845	0.262832	0.997157	3.642624
MIR95	0.846898	0.262913	2.089633	1.420233
FBXO31	0.847039	0.263044	1.007831	2.342238
FAM102A	0.847039	0.263099	0.598275	5.243787
EFNA1	0.847039	0.26315	1.551759	1.772094
CDKN1A	0.847234	0.263337	0.921516	2.794905
DBIL5P	0.847234	0.26334	-1.86814	1.17575
CYP27C1	0.847273	0.263417	-1.53455	2.265864
TMCO5A	0.847578	0.263643	2.382684	0.946122
KCTD9	0.847578	0.263666	-0.52616	6.720933
ZNF793	0.847578	0.263744	-1.22052	3.35334
CNOT7	0.847578	0.263978	-0.55587	5.723136
E2F7	0.847578	0.264024	-0.88301	4.020856
SNORD57	0.847578	0.264029	-0.98035	8.544723
SNHG15	0.847578	0.264052	-1.07554	3.285278
EIF2A	0.847578	0.264107	-0.50903	6.720301
KAT6B	0.847578	0.264192	-1.13022	3.975718
LOC10192‹	0.847578	0.26422	-1.23349	2.424555
MYO1G	0.847578	0.264222	-1.09223	4.322509
CTDSPL2	0.848576	0.264598	-0.58718	6.598611
GGT3P	0.848641	0.264683	1.419397	1.360478
CD69	0.849901	0.265267	1.032153	3.28121
PKD2	0.849901	0.265404	-0.62367	6.460036

ZC3H3	0.849901	0.265449	-0.77581	4.325862
SDR39U1	0.849901	0.265472	-1.81656	1.831668
FGF20	0.849901	0.265489	-2.59594	1.542686
ZNF623	0.849901	0.265614	-1.96557	1.265658
AKAP10	0.849901	0.265732	-0.52247	5.995476
LEMD2	0.849901	0.265793	-0.87694	3.590034
ZNF446	0.849901	0.265954	-1.06117	2.982795
CSNK1A1	0.849901	0.265962	-2.18681	1.781675
CHD3	0.849901	0.266048	1.01528	3.558539
FUT4	0.849901	0.266054	-0.5982	6.500949
ZGLP1	0.849901	0.266098	1.430121	1.806361
PLS1	0.849901	0.266099	0.924897	3.642563
PANK3	0.849901	0.26613	0.452321	7.467669
RAMP2-AS	0.849901	0.266145	1.934924	1.041973
MIR27B	0.849901	0.266241	-1.23899	2.735873
PRDX5	0.849901	0.266242	1.299424	1.392649
XCL1	0.849993	0.266463	-2.52288	0.967388
IFT57	0.849993	0.266496	-0.50579	6.247324
TBX2	0.849993	0.266502	0.583213	5.553018
PAH	0.849993	0.26653	-2.39039	1.840721
SLC15A4	0.850675	0.266865	-0.58747	4.97175
NDUFA10	0.850675	0.266922	0.490915	7.127494
CDC42EP5	0.850675	0.266938	1.274444	2.758098
DDX11-AS1	0.850703	0.267012	1.425442	1.227788
NORAD	0.850875	0.267231	-0.49372	9.882891
SEMG1	0.850875	0.267299	2.282051	1.679941
STAG2	0.850875	0.267322	1.038258	2.471992
HERC2P10	0.850875	0.267528	-2.17885	0.667406
SESN1	0.850875	0.267583	0.828543	3.746201
MMAA	0.850875	0.267626	-0.82295	4.497425
NAA15	0.850875	0.26765	-0.55465	7.13051
EIF2B3	0.850875	0.267733	-1.4656	2.230492
LRP11	0.850875	0.267769	-0.57851	6.072715
CFAP61	0.850875	0.267846	-2.90936	1.001932
VIPR1-AS1	0.850875	0.267949	2.086001	0.69683
SNORD80	0.850875	0.26796	-0.81221	4.333391
PSMD2	0.850875	0.267972	-0.62143	5.26653
ESRRA	0.850875	0.267974	0.848335	3.461663
GNB2	0.851219	0.268197	-0.55399	5.890489
SNORD134	0.851219	0.268212	-1.40003	1.651906
IGBP1	0.852137	0.268607	0.48855	6.219472
SH3GL1P2	0.852137	0.268631	-1.35066	3.029053
TMPRSS11	0.852595	0.268847	1.537914	4.830127
CWF19L2	0.852595	0.269026	-0.53731	6.277404
TMEM161	0.852595	0.269107	1.090997	3.057724

IVD	0.852595	0.26915	2.133912	0.756745
LOC440311	0.852595	0.269166	1.304834	1.467171
ZNF812P	0.852595	0.269348	1.671083	2.646015
RIDA	0.852595	0.269404	0.791032	3.805295
N4BP3	0.852595	0.269423	0.887688	3.122007
PIN4	0.852595	0.26945	1.741988	1.433114
GOLGA6L4	0.852595	0.269465	1.071329	2.126078
MXRA7	0.852595	0.26949	-1.05049	4.471904
LOC15506(	0.853015	0.269688	0.689958	4.408317
ZNF271P	0.853038	0.26976	1.778355	1.086898
C20orf194	0.854057	0.270158	-0.62532	6.497393
FBXO3	0.854057	0.270213	0.707501	4.766661
THUMPD3	0.854228	0.270347	-1.36151	2.23922
APOBEC3C	0.854228	0.270559	-0.66949	4.730483
PIH1D1	0.854228	0.270581	-0.56277	5.315776
BCL6B	0.854228	0.270643	-0.96174	3.388502
FTH1	0.854228	0.270646	-0.54599	10.19469
CRYBA1	0.854228	0.27067	-1.90186	0.954396
VGLL3	0.854228	0.270755	-0.78531	5.203353
LRPPRC	0.854228	0.270788	-0.54336	8.016689
CFAP44-AS	0.854674	0.271028	1.577975	1.523196
RPL19P12	0.854674	0.271261	0.816886	3.176615
TIMP4	0.854674	0.271264	-1.55077	2.671696
PTTG3P	0.854674	0.271318	-2.41324	0.945383
MIR4265	0.854674	0.27133	1.514255	1.354873
ZNF707	0.854674	0.27135	1.532983	1.529264
COL6A4P1	0.854674	0.271385	-1.71921	2.058782
E2F1	0.854717	0.27153	-1.03297	3.777978
BDH2	0.854717	0.271617	0.625349	5.39803
SNHG20	0.854717	0.271635	0.858309	3.090937
POP7	0.854717	0.271659	0.947631	4.061308
RTL10	0.854745	0.271786	-0.59434	5.706245
CNTD1	0.854745	0.271869	-1.21443	2.798273
RTL9	0.854745	0.271918	-2.60421	0.975677
PRR36	0.854745	0.271949	-1.76064	2.996708
LINC01599	0.854745	0.272036	1.652469	0.80718
CCNL1	0.854745	0.272059	-0.54155	5.744904
IL7R	0.854853	0.272228	1.8528	1.416119
LOC10050(	0.854853	0.272266	1.014983	2.083782
ARL3	0.854853	0.272348	-0.82831	4.888068
PLAUR	0.854853	0.272354	-1.89879	1.759225
LOC28576(	0.855048	0.272549	2.101341	1.274609
SYT10	0.855048	0.272676	2.441205	0.846733
MPV17L2	0.855048	0.272691	-1.61104	2.092375
CTSO	0.855048	0.272738	-1.43254	2.629135

LINC00869	0.855048	0.272742	-1.09882	3.297021
PTPRA	0.855137	0.272836	-1.73184	2.0182
PLEKHF1	0.855179	0.272978	-1.29386	2.544907
ATP8A1	0.855179	0.272979	1.578278	1.690623
UBL4A	0.855455	0.273133	-0.67183	4.380466
METTL6	0.85558	0.273238	1.773085	1.048482
SGIP1	0.855691	0.273338	-1.96992	1.521702
ITPR2	0.855969	0.273493	0.451665	8.186208
HSPA14	0.856072	0.273608	-0.7194	4.526872
NMNAT3	0.856072	0.273656	-1.66143	1.743989
GARS	0.856223	0.273769	1.230811	2.578384
PTRH2	0.85663	0.274025	-0.88753	3.214472
PPP1R11	0.85663	0.274137	0.951894	4.518708
MPRIP	0.85663	0.274146	-1.83399	1.604934
TSPAN31	0.85663	0.274167	-0.67162	4.360332
PTPN18	0.85663	0.274226	0.885789	3.359111
TMEM91	0.856826	0.274354	1.905936	1.710355
ASPH	0.857251	0.274691	0.73174	4.241037
BAZ1B	0.857251	0.27474	-0.49665	8.685938
GTSE1-DT	0.857251	0.274851	1.754388	1.48202
DLGAP1-AS	0.857251	0.274903	-0.88868	4.163792
TMEM92-A	0.857251	0.274928	-1.76591	2.039473
UGT1A10	0.857251	0.274951	1.266751	3.943341
ATP2B1-AS	0.857251	0.275003	0.954987	2.743064
SAMD4B	0.857251	0.275013	-0.77505	4.871375
LOC153684	0.857395	0.275124	0.766988	3.967646
HRAT92	0.857983	0.275378	-1.15107	2.98322
ACSL1	0.858202	0.275514	-0.92333	3.367585
LPIN2	0.858226	0.275624	-0.84111	8.614154
TMEM200C	0.858226	0.275653	1.914792	0.926323
KANTR	0.85834	0.275755	0.676901	4.035859
BLZF1	0.858492	0.275869	-0.59307	5.74962
GDPGP1	0.858649	0.275985	0.975272	2.744864
ZNF564	0.858661	0.276054	-0.6109	5.400172
LOC727896	0.859167	0.276282	-0.84583	5.657505
ZNF443	0.859414	0.276427	-1.06058	3.504792
CEP170P1	0.859668	0.276575	-1.06133	4.192139
COQ9	0.859749	0.276744	0.512718	5.370392
CNOT10	0.859749	0.276888	-1.56831	1.16527
ADGRD1	0.859749	0.276955	-0.79094	5.284156
NLRC5	0.859749	0.277007	-0.63707	7.046612
BIN3-IT1	0.859749	0.277009	-1.34524	2.746649
RSAD1	0.859749	0.277189	0.982766	2.06563
LDOC1	0.859749	0.277212	-0.85613	3.561348
AHCTF1	0.859749	0.2773	-0.53854	7.612171

FGF10-AS1	0.859749	0.277381	1.755793	1.396691
TRIL	0.859749	0.277457	0.915327	3.985484
ZNF276	0.859749	0.277478	-1.99896	1.841746
DLG5-AS1	0.859749	0.277526	-1.9504	0.938629
SKOR1	0.859749	0.277534	1.30393	1.637226
RHOBTB3	0.859749	0.277554	0.516527	6.794759
PROSER3	0.859749	0.277787	-0.67609	5.055609
CATIP-AS2	0.859749	0.277825	1.001977	2.209005
CYB5R1	0.859749	0.277935	-0.61181	4.991575
CCDC24	0.859749	0.277938	-0.88432	3.928195
ADAMTS15	0.859749	0.27794	0.798614	3.630072
KDM4C	0.859749	0.277959	0.721912	3.831915
NIFK	0.859749	0.278025	-0.91076	5.309301
CALM3	0.859749	0.278152	0.442792	8.348322
NDUFB7	0.859749	0.278437	0.497367	5.56285
LOC101927	0.859749	0.278438	-1.77433	1.395598
YTHDF1	0.859749	0.278512	-0.50114	6.283907
GBA2	0.859749	0.278526	0.479567	6.719406
LURAP1L	0.859749	0.278567	0.676619	4.406248
NEDD9	0.859749	0.278604	-0.92738	3.279464
PMS2P7	0.859749	0.278628	-1.13121	2.662513
PPM1H	0.859749	0.278668	0.577246	5.234198
FRRS1L	0.859749	0.278705	-2.23419	1.618899
SIGLEC9	0.859749	0.278741	-1.45057	1.654296
ZNF366	0.859749	0.278818	0.686915	4.109482
TPI1P3	0.859749	0.278829	0.73348	3.739431
LAPTM5	0.859946	0.278992	-0.564	6.795279
CNNM4	0.859946	0.279059	0.506674	6.419632
SPDL1	0.859946	0.279237	-0.59421	4.791195
UBC	0.859946	0.279244	-0.49916	9.861827
EBP	0.859946	0.279314	0.664869	4.832739
RAB7A	0.859946	0.279471	-0.49746	8.090278
EFHC1	0.859946	0.279479	-1.05845	3.177184
YRDC	0.859946	0.279522	-1.16826	3.629553
LINC00870	0.859946	0.27955	1.763963	1.588994
FZD9	0.859946	0.279569	2.066333	1.102193
EEF1AKMT	0.859946	0.279673	-2.18918	0.84743
SLC35C2	0.859946	0.279741	0.719702	3.944175
GNRH1	0.859946	0.279932	1.321156	2.188255
OXA1L	0.859946	0.27994	0.529135	6.466491
LINC00506	0.859946	0.280032	1.464826	1.438023
MCEMP1	0.859946	0.280042	-2.20125	0.729772
SNORD42B	0.859946	0.280059	-1.3741	1.914509
UQCR11	0.859946	0.280099	0.580686	5.700903
LBHD1	0.859946	0.280138	1.011836	2.616704

HEATR6	0.860029	0.280369	-0.58634	4.835865
MIR570	0.860029	0.280484	0.815111	3.31636
ZZEF1	0.860029	0.280541	0.43898	8.36343
LACTB2	0.860029	0.280597	0.622108	4.298095
NOD1	0.860029	0.280609	-0.53003	5.613672
SNORA58	0.860029	0.28066	0.619501	7.449201
LINC01281	0.860029	0.280745	-2.6522	1.330198
SLC16A11	0.860029	0.280753	1.317454	1.760979
SOCS1	0.860029	0.280812	0.894262	3.348307
VWA5A	0.860029	0.28082	1.244528	2.062739
MRPL12	0.860408	0.281047	0.681658	4.861263
RNPS1	0.860408	0.281075	-1.1009	3.384217
SEMA3C	0.860639	0.281216	-0.53911	6.724117
ADAP1	0.861578	0.281589	-1.56982	1.739746
NECAB1	0.861647	0.281677	-2.02456	2.545537
OLFM5P	0.861694	0.281758	2.078863	0.984109
CSNK2A2	0.861894	0.281964	-0.54725	5.35132
ZNF467	0.861894	0.281998	1.058212	2.767196
PINK1	0.861894	0.282021	-0.68384	4.217265
YARS2	0.862107	0.282156	-0.85223	3.683456
HAAO	0.862469	0.282354	0.815665	3.538021
LINC01138	0.862469	0.282498	-1.33335	3.576681
RPS17	0.862469	0.282582	1.868837	1.388638
FOXO6	0.862469	0.282635	-1.51126	2.387895
CTD-2270F	0.862469	0.282746	-1.8197	1.522438
NUDT18	0.862469	0.282793	-0.86501	3.643183
MAGI2-AS3	0.862469	0.282899	-0.87697	5.717402
TRPV6	0.862469	0.282911	1.51329	1.878637
TAS1R3	0.862469	0.282916	0.924955	2.924473
TMEM214	0.862469	0.2831	1.385408	1.356585
CERS5	0.862469	0.283151	-0.75964	3.488209
MCCC2	0.862469	0.283165	0.476251	5.962927
AKAP5	0.862469	0.283179	-0.85768	3.81722
LOC100129	0.862469	0.283195	0.80299	3.206822
NFYB	0.862773	0.283375	-0.57665	5.554799
LOC645752	0.862773	0.283495	1.162462	2.167532
HBA2	0.862773	0.283536	-1.42096	2.709269
ARMC1	0.862773	0.283558	-2.00216	1.355381
SEC16B	0.864609	0.284394	0.594515	6.424878
VPS37C	0.864609	0.284399	0.50243	5.676874
ENTPD1	0.864609	0.284448	1.768827	1.380075
PSTPIP1	0.864609	0.284568	0.603654	4.9193
GMPR	0.864609	0.284594	-1.00487	3.583634
FAM53B	0.864609	0.284622	0.470066	6.655391
EVI5L	0.864609	0.284739	1.941695	1.560524

EEF1DP3	0.864609	0.284756	1.248149	2.053718
SORL1	0.864609	0.284759	-0.79754	8.805898
FAM83H	0.864609	0.284876	-0.772	6.711167
GOLGA8IP	0.864609	0.284886	-2.14164	0.61948
TSEN54	0.86462	0.284959	-0.68046	4.621723
SDCCAG8	0.86462	0.285021	-1.28376	3.389093
C1QTNF7	0.864621	0.285088	-1.76141	2.080869
NPAS1	0.864747	0.285273	-1.34029	1.864695
CDA	0.864747	0.285371	-1.37615	3.765587
PGAM1P5	0.864747	0.285509	1.398341	1.58964
ZNF524	0.864747	0.285555	0.874603	3.602146
PLA2G2F	0.864747	0.285581	-2.46761	1.914559
CDNF	0.864747	0.285656	-2.12645	1.091326
ZNF431	0.864747	0.285685	0.55426	5.799682
YEATS2	0.864747	0.285716	-0.46	7.176778
SNORD71	0.864747	0.285754	-0.8941	5.483436
CTAGE9	0.864747	0.285851	-1.93381	1.24853
CRAMP1	0.864747	0.285898	-0.51728	7.08196
CAPN3	0.864747	0.286004	-1.28885	3.117377
FAM8A1	0.864747	0.28609	-0.5241	6.23303
RPL36AL	0.864747	0.286188	0.454511	7.517027
MTCP1	0.864747	0.286329	-1.70346	1.552476
HNRNPA1F	0.864747	0.286405	-2.11339	1.426947
COMMD3	0.864747	0.286417	-1.1675	2.937795
SCARNA16	0.864747	0.286432	0.657063	4.524207
ST18	0.864747	0.286449	-1.41591	3.905125
STAM	0.864747	0.286493	2.290988	0.67713
DNTTIP2	0.864747	0.286578	-0.5166	6.648849
SNORD38B	0.864747	0.286579	-0.89575	3.378084
PMS2CL	0.864855	0.286681	-0.76663	4.438384
KCNA3	0.864873	0.286881	1.265796	3.35334
HOXC9	0.864873	0.286926	-1.14378	3.711693
CSPG4	0.864873	0.286938	-0.57358	6.240984
SNRNP200	0.864873	0.286951	-0.47243	9.174318
CDH5	0.865145	0.287107	0.594876	5.76437
OGG1	0.865263	0.287212	1.411833	0.917132
DUSP28	0.865289	0.287289	-1.09091	2.715754
FCHSD2	0.865289	0.287352	-0.54701	6.555505
CHMP1B	0.865478	0.287481	-0.58794	6.708547
SAMD12	0.865523	0.287632	-1.13257	3.473273
CYTIP	0.865523	0.287708	0.595232	6.288168
SH3TC2	0.865523	0.287778	-0.7789	4.327709
SEH1L	0.865523	0.287802	-0.96468	4.127178
SNORD9	0.865523	0.287826	-0.89731	6.170909
LYRM4	0.865648	0.287933	-1.82487	1.296197

LY9	0.865925	0.288118	1.452639	1.109644
NMD3	0.865925	0.288268	-0.47595	6.629157
MOGS	0.865925	0.288282	1.417056	1.66669
APEH	0.865925	0.28829	0.483717	6.293023
RAB40B	0.866018	0.288438	-0.62502	5.134629
DTX2P1-UI	0.866018	0.288453	-0.53781	5.758128
SPECC1L	0.86671	0.288749	-1.68883	1.709522
METTL7B	0.866785	0.28884	0.772803	3.450094
MYL9	0.866811	0.288915	-1.23004	3.065547
DNM1P41	0.86724	0.289124	-1.51576	1.367968
ELMO1	0.867441	0.289257	0.841296	2.953848
NPHP4	0.867727	0.289443	-1.94327	1.455013
TMBIM4	0.867727	0.289519	-1.4257	2.496119
RASA4CP	0.867727	0.289551	0.744007	3.711265
TMEM151/	0.867885	0.289723	-1.86098	1.74451
ZNF30	0.867885	0.289736	-2.49781	1.028655
ZBED3-AS1	0.867984	0.289835	-1.73515	1.471955
TRAPPC10	0.868298	0.290059	-0.51397	6.426374
LOC14584!	0.868298	0.290073	2.01342	0.843986
RNU5F-1	0.86876	0.290401	-1.04338	4.615201
SMCO2	0.86876	0.2905	-1.45701	1.546737
ERI2	0.86876	0.290505	0.628161	4.349526
NOTCH1	0.86876	0.290557	-0.49718	6.967241
PDCD6IP	0.86876	0.290583	0.608342	4.311415
LOC10099(	0.86876	0.290706	1.503348	1.725405
CHMP2A	0.86876	0.29072	1.450187	1.293174
TDP1	0.86876	0.290756	1.572194	1.195173
PIGK	0.869486	0.291205	-0.55175	5.390259
KIAA1841	0.869486	0.291304	1.089318	3.285704
TTC28-AS1	0.869486	0.291326	-0.94418	3.543484
USP36	0.869486	0.291326	-0.46479	7.199286
RPUSD1	0.869486	0.291364	-0.75261	3.880164
PXDN	0.869486	0.291397	0.549821	7.064345
GATAD2B	0.869506	0.291533	-0.65484	4.494995
NUAK2	0.869506	0.291536	0.665715	4.924157
ZNF626	0.869544	0.291616	-1.10339	2.956078
SKP1	0.870029	0.291907	-1.14948	2.259371
CD63	0.870029	0.291952	0.846657	2.504961
LZTS1	0.870029	0.291977	-0.98265	3.982765
C8orf88	0.870313	0.292241	-1.4282	2.214656
KPRP	0.870313	0.292256	1.775984	2.159046
MED25	0.870313	0.292371	-0.5432	5.604433
SNORD32A	0.870313	0.292388	-0.72023	4.8177
STAM2	0.870313	0.292404	-0.48323	6.424258
DCLK1	0.870429	0.292554	-0.88196	3.94668

PPP1R2B	0.870429	0.292584	0.8216	3.298706
ZNF471	0.870429	0.292642	-1.3515	3.557023
RASEF	0.870504	0.292734	-0.56878	7.332851
PMS2P2	0.870557	0.292828	-1.38808	0.892889
TRADD	0.870557	0.292938	0.61554	4.094609
TINCR	0.870557	0.29295	1.48063	1.71002
C2CD5	0.871105	0.293204	1.265201	2.065845
MIR6883	0.871105	0.293394	1.618283	0.7876
PPP1R15B	0.871105	0.293447	-0.47115	7.581073
C19orf71	0.871105	0.29346	-0.8754	3.302293
LOC28304[illegible]	0.871105	0.29348	2.231442	1.001891
ZRANB1	0.871105	0.293533	-0.50227	6.79476
SEMA4C	0.871398	0.293698	-0.54981	5.403719
ADGRE4P	0.871525	0.29385	1.129817	2.71079
IHH	0.871525	0.293894	0.941266	3.695188
BRIX1	0.871525	0.294013	-0.64531	4.787847
CROT	0.871525	0.29404	-1.32538	1.81984
AP2S1	0.871525	0.294073	0.933273	2.391337
MRPS23	0.871831	0.294243	-0.60493	5.15358
NRAV	0.871989	0.294535	-0.80437	3.652644
LOC64428[illegible]	0.871989	0.294549	0.529739	5.759933
SF3B6	0.871989	0.294656	0.692015	5.68925
ATAD5	0.871989	0.294703	-0.50633	6.097431
FHDC1	0.871989	0.294706	0.595907	5.669391
DHX37	0.871989	0.294731	-0.60852	5.527867
PHC2	0.871989	0.294762	0.981317	2.971201
KLHL6	0.872208	0.294957	0.530485	6.072288
FAM27E2	0.872208	0.294968	1.245342	2.665484
OSBPL3	0.872434	0.295141	-1.29851	2.107676
PIGG	0.872434	0.295178	1.784706	0.735882
GDF11	0.872894	0.2954	0.643329	4.342308
MUC19	0.873146	0.295552	-1.42104	3.009122
WIPI1	0.87322	0.295644	-0.49154	5.72183
PLA2G3	0.873277	0.295782	-1.65165	3.076432
TNFRSF1B	0.873277	0.295801	0.547821	6.054902
PUM2	0.873277	0.295864	-1.50262	2.002961
OR2V2	0.873277	0.295929	2.295331	1.275388
MST1R	0.873445	0.296053	1.209133	0.972769
LOC100287[illegible]	0.873501	0.296185	-1.65144	2.00497
MTF1	0.873501	0.296205	-0.51269	6.100499
LRPAP1	0.873528	0.296437	0.640164	4.152364
DNAJC11	0.873528	0.296538	0.459634	6.533738
FCGR1A	0.873528	0.296552	-2.00164	0.856506
ARHGEF34[illegible]	0.873528	0.296602	-0.67288	7.492682
PSMA2	0.873528	0.296625	-0.59915	5.862558

EIF5A	0.873528	0.296647	0.855679	3.471134
C15orf39	0.873528	0.296741	-0.60807	5.24134
MRTFA-AS1	0.873528	0.296747	-0.91574	4.587009
SOX8	0.873687	0.296867	-1.72825	2.390056
RASAL2-AS	0.87384	0.297153	-1.94389	1.209812
PSTK	0.87384	0.297178	-1.54879	2.361697
RORA	0.87384	0.297179	1.164474	2.713021
RAB22A	0.87384	0.297297	-0.46172	7.422448
FN3KRP	0.87384	0.297304	-2.04538	0.700188
CEP63	0.87384	0.297353	1.137934	2.466617
TBC1D9B	0.87384	0.297385	1.422778	1.736847
PNLIPRP3	0.873953	0.297583	-3.4117	0.651787
MUC5B	0.873953	0.297645	1.083855	9.438216
TXNDC12	0.873953	0.297723	-0.63026	4.910535
ANKRD61	0.873953	0.297741	0.703956	3.687708
RPA1	0.873953	0.297791	-0.52797	6.243654
PCIF1	0.873953	0.29783	-0.53321	5.364
ACVR2B-AS	0.873953	0.298001	0.989059	2.577181
ERF	0.873953	0.298006	-1.83956	1.241033
MED12L	0.873953	0.298023	0.870668	3.001006
CEPT1	0.873958	0.298104	1.060432	2.528921
FANCD2	0.873958	0.298214	-1.71863	1.697426
PLEKHG1	0.873958	0.298253	-0.49294	6.873034
ADAMTS7P	0.873958	0.298291	1.686921	2.144983
FBXL19-AS1	0.874045	0.298472	-0.91256	3.688133
TNS3	0.874045	0.298496	-0.48564	8.069823
SNU13	0.874045	0.29856	1.363328	1.259754
6-Sep	0.874045	0.298588	-1.17488	2.769695
SRPRA	0.874207	0.298738	-1.43903	2.420454
CCND2	0.874207	0.298776	-0.67188	8.343387
CYP3A5	0.874911	0.299121	0.692131	7.449361
RAB1A	0.874911	0.29915	0.560362	5.134924
MYADM	0.875085	0.299276	1.064992	4.040407
IPW	0.875184	0.299377	-1.02344	3.484825
FXR1	0.875338	0.299496	1.339377	1.707385
TOP1P2	0.875466	0.29963	1.518852	0.911911
GALNT12	0.875466	0.2997	0.571614	5.870404
KIAA1549L	0.875466	0.29974	-1.18324	3.218459
SLC44A2	0.875602	0.299854	1.094623	2.469778
RP2	0.875773	0.299979	-0.58647	5.047785
SEC23A	0.876144	0.300256	-0.52673	6.964109
LINC00893	0.876144	0.300351	-0.63643	4.701742
FOXN3-AS1	0.876144	0.300436	1.150926	1.753223
GATA5	0.876144	0.300471	-2.04731	2.154454
GCFC2	0.876144	0.300504	-2.03622	0.978044

ABO	0.876144	0.300517	0.604013	5.690479
GRHL1	0.876144	0.300671	0.811859	5.249391
APPL2	0.876144	0.300707	1.438718	2.028186
C7orf43	0.876144	0.300707	1.300558	1.800336
KIAA0319L	0.876576	0.300934	0.433761	7.32158
ABHD5	0.876576	0.301101	-0.52439	5.622192
CPE	0.876576	0.301112	-0.60657	6.075103
IRAK1BP1	0.876576	0.301122	0.64985	3.980933
GJB2	0.876738	0.301297	1.031197	7.022115
LMBRD2	0.876738	0.301333	0.508681	5.253048
SLC35B4	0.876738	0.301379	-0.56022	5.220152
ARSD	0.87716	0.301702	0.454602	6.046652
RNU4ATAC	0.87716	0.301709	-0.71472	5.778059
MIR4523	0.87716	0.301724	-1.43	1.159868
MAP2K7	0.877298	0.301876	-1.58706	1.013578
ARG2	0.877298	0.301906	-1.01629	3.616123
ACP1	0.877344	0.301988	1.645813	1.268483
S100A8	0.877585	0.302138	1.729082	7.946065
PHKB	0.877663	0.302361	-1.69026	1.019375
CKMT1A	0.877663	0.302375	1.052012	2.693491
SNORD121	0.877663	0.302573	-1.73312	1.327023
ELK3	0.877663	0.30263	-0.55026	5.724921
ATAD2	0.877663	0.302662	-0.52092	6.822547
FRAS1	0.877663	0.302686	0.655667	5.624771
PWAR5	0.877663	0.30276	-0.79625	5.354049
ENTPD1-AS	0.877663	0.302804	1.016139	2.959285
FAM153A	0.877663	0.302932	-2.41587	1.300581
C16orf70	0.877663	0.303012	-0.95724	4.376094
STK32A	0.877663	0.303049	1.152529	2.069612
NEK3	0.877663	0.303075	1.586472	1.070902
YIPF3	0.877663	0.303126	0.452643	6.598972
KDM2A	0.877663	0.303331	-1.20286	2.54299
MIR5092	0.877663	0.303378	-2.16972	1.135939
ZNF485	0.877663	0.303401	-0.95945	3.378556
ONECUT3	0.877663	0.303504	-1.06277	4.017068
GCNT7	0.877663	0.303608	1.038821	3.147935
LINC00839	0.877663	0.303609	1.256835	1.918845
LINC01426	0.877663	0.303808	1.062226	1.865362
CNFN	0.877663	0.303849	1.627573	4.853372
WDR82	0.877663	0.303894	-0.46197	7.336327
B4GALT6	0.877663	0.30398	-0.73861	3.914402
HDHD3	0.877663	0.304024	1.075492	2.210897
LOC10099€	0.877663	0.304125	1.27584	1.871062
DMXL1	0.877663	0.304214	-1.30167	2.003396
NOP58	0.877663	0.304247	-0.47715	7.154452

CFAP57	0.877663	0.304266	-1.60107	2.671897
CITED4	0.877663	0.304319	0.738384	3.610907
PRICKLE2-A	0.877663	0.304459	-1.79549	1.55809
SERINC3	0.877663	0.304525	-0.52052	6.192158
FLJ20021	0.877663	0.304551	1.18468	2.030843
ABCC5	0.877663	0.304558	0.437327	7.00791
LINC01001	0.877663	0.30465	1.445339	1.433919
EXOSC6	0.877663	0.30468	-0.69975	4.122136
NCAM2	0.877663	0.304685	-1.37038	3.041891
M6PR	0.877663	0.304793	-1.19742	2.738328
LOC729609	0.877663	0.304819	1.336164	1.228678
MBTPS2	0.877663	0.304858	-0.54302	5.014776
MMP25-AS	0.877663	0.304906	-1.03618	3.447055
FLJ45513	0.877663	0.304911	1.426634	2.456382
KCNJ8	0.877663	0.304974	-0.91786	3.424899
EIF1B	0.878005	0.305298	-0.58934	4.678954
SAC3D1	0.878005	0.305317	0.950775	3.314052
C11orf49	0.878005	0.305318	-1.56092	1.347658
HNRNPUL2	0.878005	0.305361	-0.5044	6.348111
MYO10	0.878007	0.305461	0.492423	8.771699
KANK4	0.878007	0.305496	-1.76287	1.009957
TNKS	0.878037	0.305598	-0.46247	7.356187
ATG16L2	0.878037	0.305642	0.563435	5.442556
CMTM6	0.878037	0.305707	-0.46251	6.9814
BMS1P2	0.878126	0.305924	0.485314	6.695655
DCTN6	0.878126	0.305926	-0.71647	4.637508
ANAPC16	0.878126	0.305938	0.840797	2.931755
SNORD51	0.878255	0.30605	-2.17885	0.967848
PDCD5	0.878464	0.306257	-0.5737	5.178692
KLHL21	0.878464	0.306306	-0.62929	5.754578
LOC100996	0.878464	0.306336	0.713856	3.240735
STN1	0.878464	0.30641	-0.56485	5.682659
RIOK2	0.878464	0.306458	-1.26474	2.491692
C5orf34	0.878472	0.306528	-1.21396	2.773032
MTRNR2L2	0.878539	0.306716	0.691487	4.420677
ENPEP	0.878539	0.306738	-0.81296	3.717411
SNORD12B	0.878539	0.306752	-0.66062	6.708249
C9orf40	0.87874	0.306907	0.984686	2.717744
LOC101060	0.87874	0.307009	1.164321	1.773904
CCNT2-AS1	0.87874	0.307055	1.015829	1.847491
FNDC4	0.87874	0.30709	-0.88769	3.276592
ARL16	0.879784	0.307522	-0.63006	4.373196
MTCL1	0.880287	0.307767	-0.65616	7.23111
SLC25A5	0.880287	0.30795	0.52856	6.980687
CDRT1	0.880287	0.30795	1.50591	1.739849

EEPD1	0.880287	0.307966	-0.56687	5.902897
SMC5-AS1	0.880417	0.308137	-1.64994	1.305381
SH3BGRL2	0.880417	0.308146	0.469771	7.925963
NDUFB2-AS	0.880599	0.308384	1.312024	2.331775
ACSM1	0.880599	0.308582	1.166499	2.491301
SNORD144	0.880599	0.30865	-1.48391	1.8762
ADAMTS2	0.880599	0.308768	-0.67465	6.139877
MYO1E	0.880599	0.308775	-0.54064	8.506981
CNTNAP3P	0.880599	0.309068	-0.65161	5.169121
ZMAT2	0.880599	0.309091	-0.60785	7.430511
ATP8B2	0.880599	0.309097	-0.62362	6.028972
LOC100288	0.880599	0.309216	-0.79203	3.8584
FIGNL2	0.880599	0.309314	-1.5311	1.424579
SYCP2L	0.880599	0.309351	-1.32131	3.140453
LOC93622	0.880599	0.309392	0.640197	4.294433
RAP2B	0.880599	0.309446	-0.52019	7.155639
LRP2BP	0.880599	0.30951	-0.72179	4.37959
FAM184B	0.880599	0.309521	-0.89997	3.568722
MZT1	0.880599	0.309535	-0.62247	4.499688
UOX	0.880599	0.309723	2.009445	1.349081
C1GALT1C1	0.880599	0.309846	-2.17402	1.235694
ZNF320	0.880599	0.309873	0.579539	5.794602
AQR	0.880599	0.309889	-0.47205	6.70248
PCDHB4	0.880599	0.309921	-1.05112	3.043754
SMA4	0.880599	0.310014	0.513815	5.580224
C2orf70	0.880599	0.310093	1.263124	1.708421
GRIK1	0.880599	0.310101	1.614733	1.11163
CTBP1-AS	0.880599	0.310229	0.765045	3.469416
SH3BP1	0.880599	0.310263	0.662868	3.945741
TBP	0.880599	0.310271	1.35626	1.385724
CYP2R1	0.880599	0.310283	0.681958	3.578069
UNC5CL	0.880599	0.310354	0.623912	5.628055
UBA6	0.880599	0.310513	-0.4996	6.932709
PPIA	0.880599	0.310516	-1.37422	2.400014
SENP3	0.880599	0.310523	0.950716	1.971223
FJX1	0.880599	0.310539	-1.60093	1.629655
KAT6A	0.880599	0.310671	-0.44347	7.807616
ABR	0.880599	0.310673	1.280392	2.398117
FAM234B	0.880599	0.31069	-0.86679	3.129651
DNAJC15	0.880599	0.310693	-0.6189	6.147917
FTLP10	0.880914	0.310871	2.179569	1.083446
TRHDE-AS1	0.881507	0.311317	1.495503	1.858051
PPIEL	0.881507	0.311421	0.822135	4.212255
UBTF	0.881507	0.311468	1.185969	2.468834
MAD1L1	0.881507	0.31151	1.575971	1.34263

NFIA	0.881507	0.311565	1.152793	1.387851
NOP14-AS1	0.881507	0.311571	-0.77014	3.427523
NDUFA7	0.881507	0.311612	0.632378	4.143085
PDCL	0.881507	0.311684	-0.49395	5.501053
TMEM201	0.881507	0.311685	-0.7088	3.742633
FAM208B	0.881542	0.311813	-0.44569	7.829345
C15orf41	0.881542	0.311832	1.396834	0.794553
CAMK4	0.88161	0.311923	-1.15378	3.430261
KLHL28	0.881776	0.312075	1.022639	2.43826
LOC652276	0.881776	0.312116	-0.65322	5.52501
CA14	0.882127	0.312309	1.232362	1.72537
FRG1FP	0.882127	0.312495	1.584313	1.22623
SH3BP4	0.882127	0.312527	-0.50215	7.027676
LRRC71	0.882127	0.312549	1.32498	1.194113
PIK3C2A	0.882127	0.312688	-0.44198	8.010397
SEMA6A	0.882127	0.312749	1.569311	0.92412
SNAPC3	0.882127	0.312866	0.463788	5.895802
ZNF350	0.882127	0.312977	-1.04819	3.691293
ZNF831	0.882127	0.313061	0.873585	3.128756
SOX10	0.882127	0.313146	1.192689	2.666728
TSPYL5	0.882127	0.313179	-0.98346	3.725981
SLC6A8	0.882127	0.313326	1.369888	1.648494
NRTN	0.882127	0.313463	0.731491	3.906782
CCDC34	0.882127	0.313557	0.854155	3.366968
ITGB1BP1	0.882127	0.313684	-2.03118	0.981549
CD2BP2	0.882127	0.3137	1.548409	0.864451
FAM198B	0.882127	0.313793	1.661216	0.933604
SGK494	0.882127	0.31394	-0.52498	5.814248
ISX	0.882127	0.313949	2.014714	0.975319
DOCK6	0.882127	0.313977	0.419735	7.391796
DIRC2	0.882127	0.314159	-0.56539	5.488901
ZNF652	0.882127	0.314206	1.623855	0.859748
SEC14L1	0.882127	0.314326	-1.15448	2.668879
PON3	0.882127	0.314347	1.423664	1.848386
PMM1	0.882127	0.314588	0.629127	4.596436
AXL	0.882127	0.314595	1.681081	0.741388
ACTA2-AS1	0.882127	0.314692	-0.92761	4.117905
LINC00939	0.882127	0.314808	1.420171	1.730931
MAFK	0.882127	0.314941	-0.50102	6.135641
RRH	0.882127	0.314949	1.358717	1.270939
ARID5B	0.882127	0.314987	0.524044	4.977567
DICER1	0.882127	0.315078	-1.08425	2.500951
CHL1	0.882127	0.315089	2.11975	1.268726
CADM3-AS	0.882127	0.31514	1.420139	2.587245
LOC101928	0.882127	0.315148	1.001873	3.165219

LRP10	0.882127	0.315153	0.483052	7.597858
RFX3-AS1	0.882127	0.315242	1.543959	1.244037
TOP2A	0.882127	0.315248	-0.59608	7.590435
ARHGEF3-/	0.882127	0.315252	-1.74332	1.00467
MED1	0.882127	0.315343	-0.42518	7.585109
LINC00958	0.882127	0.315476	2.0733	1.012177
GABRR2	0.882127	0.315548	-1.61693	1.296086
ITPKC	0.882127	0.315655	-0.52922	6.435856
B4GALT5	0.882127	0.315655	-0.49794	7.05328
TP53RK	0.882127	0.315661	-0.76447	4.533776
IFITM10	0.882127	0.315725	0.719958	3.579218
TMX4	0.882127	0.315802	-0.50267	7.232061
LTBP4	0.882127	0.315892	0.939804	2.922421
TLK2	0.882127	0.315938	-2.08774	0.772663
SCNN1A	0.882127	0.315952	0.641234	6.389366
CORT	0.882127	0.31614	-2.47919	1.816045
PRKAG2	0.882127	0.316294	0.815548	3.090587
PODN	0.882127	0.316356	-1.78699	2.24802
LCA5L	0.882127	0.316624	1.004583	3.163849
SF3B1	0.882127	0.31664	-0.4279	9.442434
LOC646762	0.882127	0.316677	-0.54165	5.101081
STAR	0.882127	0.316825	1.342303	1.718452
DZIP1L	0.882127	0.316885	-0.85216	4.524994
STS	0.882127	0.31694	-1.07836	5.073698
NPIPB5	0.882127	0.317074	-0.58352	7.468946
MLIP	0.882127	0.317224	1.911033	0.94837
ANKRD40	0.882127	0.317254	-0.51024	6.527706
RGS12	0.882127	0.317285	0.716694	3.569322
SVEP1	0.882127	0.317339	-0.69279	6.913912
KRT6A	0.882127	0.317432	1.744813	9.52491
C6orf47	0.882127	0.31749	1.274707	0.824
ALPI	0.882127	0.317505	1.523855	1.886824
ROCK1P1	0.882127	0.317507	-0.78651	3.960062
SPRYD3	0.882127	0.317551	-0.49448	6.672294
KCNA7	0.882127	0.317584	-1.83339	1.356766
CEBPD	0.882127	0.317586	-0.50904	6.85551
CCNE1	0.882127	0.31776	0.885177	3.63515
DNAJC1	0.882127	0.317773	0.455505	6.264073
SLC48A1	0.882127	0.317822	-0.55383	4.701772
ALMS1P1	0.882127	0.317836	-1.64694	1.626369
BMS1	0.882127	0.317882	-0.45607	7.724027
GLUL	0.882127	0.317926	0.528787	5.338202
SNORA66	0.882127	0.317948	0.639615	5.657872
FAHD2A	0.882127	0.318017	0.635521	4.178629
TBX15	0.882127	0.318024	-1.20818	2.569247

DRAP1	0.882127	0.318096	-0.55706	5.587542
PP7080	0.882127	0.318143	0.697591	6.689628
CDK2AP2	0.882127	0.318158	1.223693	1.810271
METTL3	0.882127	0.318194	-0.52388	6.090363
RPS2	0.882127	0.318321	0.482976	8.72227
NANP	0.882127	0.318507	-0.74066	3.852263
POLR1D	0.882127	0.318526	0.621984	4.578868
TPRXL	0.882127	0.318582	-1.72941	2.831894
USP18	0.882127	0.318589	-0.9905	4.223883
ASB9P1	0.882127	0.318592	-1.60848	1.442135
CATSPERG	0.882127	0.318697	-1.10905	4.001728
BBS5	0.882127	0.318699	-0.92471	3.789603
CCDC57	0.882127	0.318711	-0.49325	6.016332
CCDC121	0.882127	0.31872	1.638294	0.754392
LINC00667	0.882127	0.318867	0.60733	4.75296
MINDY1	0.882127	0.318883	-1.10211	2.627684
FAM86HP	0.882127	0.319046	-0.99426	3.118624
LCMT2	0.882127	0.319066	-0.92392	3.348653
PMS2P3	0.882127	0.319194	-0.61715	5.252839
RPEL1	0.882127	0.319241	-1.56073	1.054559
LRRFIP1	0.882127	0.31926	-0.61268	4.249295
H2AFV	0.882127	0.319327	-0.47484	6.971978
MTG2	0.882127	0.319468	-0.6025	5.097914
ROMO1	0.882127	0.319498	-0.68435	3.949605
TRIM41	0.882127	0.319561	-1.16082	2.557835
JSRP1	0.882127	0.319626	-1.12623	2.642426
PSMD3	0.882127	0.319687	-0.47065	6.380096
SPDYE8P	0.882127	0.319688	-1.30093	2.549978
TMEM65	0.882127	0.319784	-0.52068	5.617254
ADAMTS5	0.882127	0.319805	0.567579	4.458272
DNAJC25	0.882127	0.319808	1.254243	2.109112
KIAA2013	0.882127	0.319823	0.457307	5.652365
TH2LCRR	0.882127	0.319951	1.158598	1.798154
ZNF815P	0.882127	0.320044	-1.03873	2.611116
SNHG19	0.882127	0.320075	-1.02244	2.862649
TMEM63B	0.882127	0.320081	0.428624	6.300248
AGAP7P	0.882127	0.320131	0.503359	5.065929
CCNF	0.882127	0.320174	-0.64158	4.489353
ZEB2	0.882601	0.320435	-0.65772	5.592825
ZBTB16	0.882601	0.320481	0.797916	3.531012
HTRA4	0.882723	0.320592	-1.73307	1.135405
GOLGA2P5	0.882903	0.320791	0.519522	4.678821
ACTB	0.882903	0.320799	-0.48793	12.1526
FTSJ3	0.882903	0.32086	-0.4459	6.697663
ZBTB20-AS	0.883176	0.321134	-1.08624	3.196393

MAPK8IP1	0.883176	0.321432	-1.06177	3.419748
COL21A1	0.883176	0.321454	-1.02217	4.071453
RPL12	0.883176	0.321528	0.45546	8.590711
TADA3	0.883176	0.32154	-1.91488	0.99187
GDE1	0.883176	0.32157	-0.45577	6.512996
AMBRA1	0.883176	0.321588	-0.93975	4.169551
ARHGAP9	0.883176	0.321607	1.09836	1.586848
NDUFAF2	0.883176	0.321624	-0.76122	4.062149
HOXD-AS2	0.883176	0.321632	1.943536	0.894219
LUCAT1	0.883263	0.322006	-1.95651	1.816626
CD40LG	0.883263	0.322007	-1.51241	2.426393
PLEKHM2	0.883263	0.322047	-0.46332	6.634975
RNASET2	0.883263	0.322354	0.475243	6.200651
TBC1D30	0.883263	0.322384	0.653045	4.774066
ZGPAT	0.883263	0.322471	1.323897	1.622179
MMS22L	0.883263	0.322523	-0.61851	4.794101
IQSEC3	0.883263	0.322585	-1.02151	2.812635
UBE2E1	0.883263	0.322615	1.539257	1.129381
ELMO1-AS:	0.883263	0.322769	1.49081	1.845357
ADAMTSL5	0.883263	0.32279	0.65398	3.742196
NPM1	0.883263	0.322793	-0.93275	2.806309
HIF1A	0.883263	0.322869	-1.2132	4.403172
ELOVL4	0.883263	0.323011	1.318672	2.105513
CASC4	0.883263	0.323051	0.789554	4.301401
CELSR1	0.883263	0.323063	-0.69125	6.664601
GTF2IP1	0.883263	0.323086	-0.50396	5.789824
ACAP3	0.883263	0.323227	0.467133	5.959742
FIBIN	0.883263	0.323307	0.715746	3.783927
RTL8B	0.883263	0.323324	-1.1071	2.722494
ANK3	0.883263	0.323394	0.554073	5.805503
TNFRSF1A	0.883263	0.323418	-0.53095	6.611035
HOMER3	0.883263	0.323449	1.41139	0.918456
SOX7	0.883263	0.323665	0.696021	3.830962
RAPGEF1	0.883263	0.323713	0.772574	3.821992
POLA2	0.883263	0.323776	-0.58528	4.678864
PLD3	0.883263	0.323782	-1.62785	0.766698
MINOS1	0.883263	0.323782	-1.73665	1.636576
TFDP2	0.883263	0.323796	1.392443	1.264204
CEP350	0.883263	0.323817	-0.4317	8.55033
HCG27	0.883263	0.323822	-1.35028	2.439029
LOC10013(	0.883263	0.323843	0.909722	2.83496
RPL21	0.883263	0.323886	-0.56523	6.301818
PARPBP	0.883376	0.323994	-0.78489	3.615549
LINC02615	0.883576	0.324331	0.860427	4.254867
CT62	0.883576	0.324339	1.18059	1.099606

ZNF33B	0.883576	0.324349	1.377597	0.913378
ILVBL	0.883576	0.32437	0.468112	5.698779
CCR1	0.883576	0.324404	-0.89664	3.836322
SNORA4	0.884003	0.324652	-0.92285	4.342217
TOP3B	0.884003	0.324696	1.208672	2.308418
SCAND1	0.884343	0.324918	1.444922	1.2615
NKAIN3-IT1	0.884343	0.325107	1.721939	1.463027
LCNL1	0.884343	0.325114	1.458672	1.283599
RN7SL1	0.884343	0.325132	0.414781	14.40247
KCTD4	0.884343	0.325226	-1.52169	2.431535
LOC100506	0.884343	0.325232	1.226583	2.032858
C22orf34	0.884343	0.325384	0.866332	3.584423
FAM110C	0.884343	0.325401	0.566321	5.631822
PGM1	0.884343	0.325596	0.712965	3.431966
DPY19L4	0.884343	0.325602	-0.50097	6.007023
CPEB2-DT	0.884343	0.325673	-1.61595	1.136267
CLCC1	0.884343	0.325716	1.676353	1.150382
CAPN13	0.884343	0.325722	0.858419	4.366183
LOC101928	0.884343	0.325772	1.676887	1.02203
TTC39A-AS	0.884343	0.325832	1.364574	1.417372
GRIN3B	0.884447	0.325938	-1.41988	1.959432
BAK1	0.884551	0.326043	0.534717	5.064511
PCNT	0.884581	0.326158	0.637465	3.818748
CNNM1	0.884581	0.326189	-0.97753	3.657904
ZDHHC17	0.885078	0.326445	-0.52717	5.980543
GATD1	0.885078	0.326507	0.419329	6.696487
OVGP1	0.885312	0.326661	-1.123	2.093596
RAB41	0.885486	0.326849	1.223382	1.67663
NLRP13	0.885486	0.326868	1.845362	1.268168
DOCK5	0.885486	0.326928	-0.44654	8.424291
LINC00504	0.885505	0.327002	-1.56474	0.742555
COPS6	0.885669	0.32716	-0.49231	6.302588
HIST1H3B	0.885669	0.327232	-0.66196	5.354685
SCUBE1	0.885669	0.327265	0.861904	5.62105
CTSC	0.885912	0.327452	0.465242	6.159414
MIR1281	0.885912	0.327681	0.881896	3.393982
PFDN1	0.885912	0.32771	-0.625	5.402681
DPEP2	0.885912	0.327719	-1.20678	2.645346
FADS3	0.885912	0.327728	-0.92332	4.278513
CD74	0.885912	0.32776	0.776362	3.469158
14-Sep	0.8866	0.328137	1.551506	1.756544
PDE1C	0.8866	0.328249	-2.34784	0.666797
TASP1	0.8866	0.328341	-0.63521	5.420192
SNORD29	0.8866	0.328351	1.021638	1.838248
CKB	0.8866	0.328353	-0.64824	5.135407

LOC101927	0.887149	0.328645	1.119289	2.219994
PQLC1	0.887149	0.328746	-1.98983	1.280488
MYO15B	0.887149	0.328805	0.452611	8.290342
MIR378H	0.887149	0.328827	-2.31034	1.072973
NUDT17	0.887202	0.329152	-1.08409	2.278976
TYRP1	0.887202	0.329224	1.109269	3.266817
CD80	0.887202	0.329321	-1.34321	2.379439
CRTC2	0.887202	0.329382	-0.46126	6.385489
FZD1	0.887202	0.3294	-0.57915	5.595709
BTG1	0.887202	0.329487	0.466488	7.722524
BACH1	0.887202	0.329584	0.666548	3.518233
KIF5B	0.887202	0.329747	-0.45094	9.298237
CEP250	0.887202	0.329753	-0.5077	7.845323
KCNK1	0.887202	0.329931	-0.66805	5.549915
BCAN	0.887202	0.330036	-0.99434	3.001629
LOC10013(	0.887202	0.33013	0.872568	3.480235
ERCC1	0.887202	0.330294	-1.45098	1.621911
ABHD13	0.887202	0.330297	-0.51948	5.748354
MAP1LC3B	0.887202	0.330318	-1.23822	2.506499
LRRK1	0.887202	0.330535	0.437071	6.633087
ZNF462	0.887202	0.330539	0.50552	6.776697
CRYZL1	0.887202	0.330619	-0.69922	5.060923
FLRT3	0.887202	0.330697	-1.57348	1.513659
TGS1	0.887202	0.330716	1.096419	2.035467
TENM3	0.887202	0.330785	-1.18033	4.187501
RANBP17	0.887202	0.33098	-0.75621	4.340636
JAK2	0.887202	0.331034	0.425596	7.133499
FOXJ1	0.887202	0.331043	-1.61636	3.205524
SLFNL1-AS:	0.887202	0.331091	-0.75026	4.165925
SMIM10	0.887202	0.331109	-1.09541	2.886983
TFB2M	0.887202	0.331139	-0.58497	4.619913
IQCH-AS1	0.887202	0.33121	1.448478	1.306012
ARID3C	0.887202	0.331225	1.779113	0.794147
ZSCAN22	0.887202	0.33126	0.615024	4.0571
C12orf75	0.887202	0.331288	-0.54013	5.977931
ZNF624	0.887202	0.331459	0.724638	3.876777
INO80D	0.887202	0.33155	-0.41819	8.210922
INSIG1	0.887202	0.331708	-1.89183	0.856986
PCDHB13	0.887202	0.331732	-1.18972	3.76132
TRIM31	0.887202	0.331782	0.799853	4.812121
CCDC40	0.887202	0.331853	-1.45522	1.516949
ARPC2	0.887202	0.331857	1.356746	1.066231
SENP5	0.887202	0.331877	-1.55163	2.187172
NPHP3	0.887202	0.331911	-0.63838	3.988622
PNPT1	0.887202	0.331922	-0.47883	6.273848

PIPOX	0.887202	0.331939	-1.64665	1.536942
TAF5	0.887202	0.332079	-0.76351	3.786155
DNAJC16	0.887202	0.33242	-2.3484	1.119262
PTCHD4	0.887202	0.332529	0.980621	3.097234
ACOX2	0.887202	0.332534	0.820151	2.86139
LRRN4	0.887202	0.332542	-1.23088	2.152376
PEBP1	0.887202	0.332671	0.410066	6.840073
APLN	0.887202	0.332778	0.860441	2.932023
UMAD1	0.887202	0.333081	-1.6616	1.171936
ZNF792	0.887202	0.333088	-0.77597	4.103921
RASL11B	0.887202	0.333214	1.361611	1.508549
C16orf46	0.887202	0.333276	-1.55859	1.219115
ALKAL2	0.887202	0.333349	0.963769	2.337515
LRRTM2	0.887202	0.333358	0.462044	5.84493
ZNF649	0.887202	0.333436	-0.85732	3.893424
PROCR	0.887202	0.3335	-0.6457	4.578508
GREB1	0.887202	0.333512	0.582959	5.045833
SNORA94	0.887202	0.333534	0.868974	2.306223
9-Mar	0.887202	0.333599	-0.70781	3.515542
CASC3	0.887202	0.3336	-0.42112	7.93372
ARFGEF2	0.887202	0.333633	-0.4142	8.21917
MEG3	0.887202	0.333696	0.466922	6.54551
SIGLEC14	0.887202	0.333811	1.039702	2.164381
URM1	0.887202	0.333828	-0.95879	3.188833
RPL7L1	0.887202	0.333894	-0.60129	5.726162
WRAP73	0.887202	0.334037	-0.76873	3.826388
WDR3	0.887202	0.334057	-0.44168	6.120564
LOC100505	0.887202	0.334075	-1.60386	1.236007
SLC9A9	0.887202	0.334201	0.727009	4.161565
CDC73	0.887202	0.334231	-0.41916	7.170703
BMP2	0.887202	0.33432	0.617293	5.735597
SH3BP2	0.887202	0.33439	1.010107	1.803479
SNORD30	0.887202	0.334431	1.285502	1.636876
SCRN2	0.887202	0.334505	-1.50397	1.388411
TMCC1	0.887202	0.334514	-0.79643	3.370738
OMA1	0.887202	0.334662	0.466792	5.592346
LMF1	0.887202	0.334666	-1.57756	2.495083
ATP2A2	0.887202	0.334884	-0.53226	7.467289
GPD1L	0.887202	0.334885	0.452143	5.743347
CCDC89	0.887202	0.334912	1.034213	1.986477
CHRNB1	0.887202	0.334956	-1.24155	2.927914
KLRA1P	0.887202	0.334966	-1.19829	3.238311
SAP30BP	0.887202	0.335051	1.816049	0.967144
ANKRD50	0.887202	0.335052	-0.73192	3.549748
NSMCE1-D	0.887202	0.335082	1.22635	2.271907

EPN2-IT1	0.887202	0.335235	1.174457	2.119034
SMIM26	0.887202	0.335252	-0.73766	3.214043
PLEKHA5	0.887202	0.335377	0.520496	5.355591
FRG1CP	0.887202	0.335598	-0.52382	5.084503
FBXO36	0.887202	0.335679	-1.25693	2.434412
SHH	0.887202	0.335768	-1.05811	4.245917
SNORD105	0.887202	0.33581	-0.88634	5.288886
HOXC11	0.887202	0.33585	-1.30003	2.630617
INPPL1	0.887202	0.335901	-0.44452	7.136064
SNORA71D	0.887202	0.335939	-0.60382	6.119331
EFL1P1	0.887202	0.335952	1.189468	1.105514
ERG	0.887202	0.336036	-1.63499	1.464745
ABHD8	0.887202	0.33618	-0.8364	3.206293
ANO8	0.887202	0.33625	-0.6236	4.905686
ZBTB41	0.887202	0.336335	-0.50888	6.38774
MGMT	0.887202	0.336387	-0.81765	3.61198
SSC4D	0.887202	0.336611	-1.55488	2.127637
ICAM5	0.887202	0.336634	1.283611	2.081602
HSPBAP1	0.887202	0.33666	-0.56404	4.392681
ZFAND3	0.887202	0.336677	-0.43708	6.374822
TUBB4B	0.887202	0.336688	-0.50929	7.156777
TET3	0.887202	0.337073	-0.45424	7.332997
CYP2B7P	0.887202	0.337089	1.578185	1.386152
LINC01268	0.887202	0.337214	1.106801	2.112667
RELB	0.887202	0.337237	-0.72372	4.691875
MMRN2	0.887202	0.337257	0.51419	4.848559
TNRC6C-AS	0.887202	0.337263	-0.87992	3.970933
ZBED6	0.887202	0.337299	-0.48128	7.569298
TBCD	0.887202	0.337304	0.412314	6.392368
ARHGAP31	0.887202	0.33748	1.460778	1.540119
LRFN1	0.887202	0.337533	-1.39427	1.940875
VDAC2	0.887202	0.3376	1.018166	1.999915
ATP5IF1	0.887202	0.337622	0.606376	4.02106
ITGAE	0.887202	0.337695	0.622238	3.902784
LOC100133	0.887202	0.337832	-0.62513	4.527206
EEF2	0.887202	0.337931	0.422772	11.16767
SMG1P2	0.887202	0.33794	-0.64539	4.152573
ANKRD36B	0.887202	0.338111	-0.75971	4.84813
NEFH	0.887202	0.338211	1.257867	1.875458
PGBD2	0.887202	0.33822	1.092803	2.356209
ZNF714	0.887202	0.338237	0.62332	4.521882
UGGT2	0.887202	0.338249	-0.44328	6.447573
SP100	0.887202	0.338293	-0.46946	6.57848
KIAA0556	0.887202	0.338338	-0.47575	6.503285
GABPB1-AS	0.887202	0.338488	0.485694	6.334541

ABCA5	0.887202	0.33849	0.757148	3.289024
MNX1-AS1	0.887202	0.338498	1.400137	0.981157
NFIX	0.887202	0.338508	0.827658	2.471876
CD177	0.887202	0.338541	-1.26824	2.569674
LITAF	0.887202	0.338635	-1.47124	1.52592
WBP1L	0.887202	0.338689	0.807373	2.954136
ARIH1	0.887202	0.338817	-0.42232	7.367792
ADAMTS3	0.887202	0.338819	0.983602	2.769248
GNL2	0.887202	0.33883	-0.44721	7.161469
SOGA3	0.887202	0.338883	0.789455	2.912034
GNPNAT1	0.887202	0.338918	0.535881	6.168737
ALG10B	0.887202	0.338971	-0.77002	3.945017
NAIF1	0.887202	0.339055	-0.85943	3.007313
FAM174A	0.887202	0.33909	0.681938	4.128831
MIR608	0.887202	0.339091	-2.3237	0.913661
PDIA3P1	0.887202	0.339222	0.464521	6.53286
MUTYH	0.887202	0.339228	1.666693	1.033122
FKBP1AP1	0.887202	0.339354	0.497359	4.787709
LOC100288	0.887202	0.339394	-1.13123	2.766803
PEAR1	0.887202	0.339435	-0.6659	4.379002
LOC284412	0.887202	0.339554	-0.82888	3.939683
EID1	0.887202	0.339582	-0.45121	7.339121
RSPH1	0.887202	0.339716	1.634587	0.906239
SNORD116	0.887202	0.339752	-2.24531	1.134179
ADGRL4	0.887202	0.339755	0.652915	4.53792
VAMP1	0.887202	0.339792	1.142398	2.367903
SGPP1	0.887202	0.339813	-0.70502	4.004641
TACR1	0.887202	0.339874	-1.17772	2.953919
FLJ32255	0.887202	0.339876	0.896129	3.362746
PRR14	0.887202	0.339943	-0.53556	5.22981
LINC02202	0.887202	0.340021	1.175046	1.002846
ARSB	0.887202	0.34005	-0.86844	4.429667
CDS2	0.887202	0.340199	-0.46662	7.366682
PHF5A	0.887202	0.340375	-0.63281	4.446137
OXCT1	0.887202	0.340381	0.516807	5.753821
C9orf16	0.887202	0.340409	-0.62466	3.992429
SACS	0.887202	0.34048	-1.81176	1.08224
UBE2Q2	0.887202	0.34049	-1.61956	1.075642
SCN2B	0.887202	0.340558	-1.64152	1.522399
ANXA1	0.887202	0.340602	0.775154	9.438598
C10orf143	0.887202	0.340612	-1.19849	2.220567
ZFP64	0.887202	0.340617	0.851671	3.165042
COL16A1	0.887202	0.340887	-0.47712	7.720765
ASXL1	0.887202	0.340945	-0.4293	7.508625
OTUD4	0.887202	0.34096	-0.41368	7.197091

RERE	0.887202	0.341028	1.144333	2.451267
MIR133A1I	0.887202	0.341049	-0.65513	3.871375
ZNF768	0.887202	0.341142	-0.49316	5.756654
PLEK2	0.887202	0.341236	0.682449	4.752575
GFRA3	0.887202	0.341258	1.460928	1.230665
FAM20B	0.887202	0.341441	-0.4588	6.882604
ASF1A	0.887202	0.341603	-0.52292	4.945506
NFKBIA	0.887202	0.34161	-0.4727	7.425597
HIST1H2BI	0.887202	0.341698	0.72312	4.52188
GNPAT	0.887202	0.341713	1.034593	2.082139
LOC10012ɛ	0.887202	0.341886	0.521236	4.816695
MIOS	0.887202	0.342085	-0.47012	5.85705
PHLPP2	0.887202	0.342095	-1.55515	1.205756
NSD3	0.887202	0.342166	-0.43025	6.606153
ACACB	0.887202	0.342197	0.490935	7.343815
RNU5B-1	0.887202	0.342255	-0.63635	9.152268
BBS4	0.887202	0.34237	-1.53822	1.58294
ZNF204P	0.887202	0.342402	-0.96841	2.601549
FAM157B	0.887202	0.342473	-1.80955	1.000026
FLVCR1	0.887202	0.342502	-0.52658	5.504105
ASB16	0.887202	0.342608	0.641425	4.053782
LINC00473	0.887202	0.342627	-1.64246	0.787032
SNORD113	0.887202	0.342644	-0.80218	4.918487
IMPAD1	0.887202	0.342683	-0.49197	7.283723
IRF2BPL	0.887202	0.342786	-0.53112	5.017946
SMIM7	0.887202	0.342841	-0.52369	5.075017
EPB41L3	0.887202	0.342869	1.176719	1.980441
RAPGEF5	0.887202	0.3429	0.428736	6.782728
PPP1R26	0.887202	0.3429	0.448045	5.739542
GTF2IRD2B	0.887202	0.342904	0.570178	5.03393
UFC1	0.887202	0.343223	-0.47881	5.823192
KLK13	0.887202	0.343313	1.636015	6.274774
SLCO4A1-A	0.887202	0.343321	-0.80792	3.206683
KCNIP2	0.887202	0.343322	-2.26876	1.20138
SNORD111	0.887202	0.343366	-0.97354	3.133164
CHST2	0.887202	0.343495	0.75778	4.582343
GAK	0.887202	0.343509	0.687047	3.606972
PARVG	0.887202	0.343544	-1.6235	1.294748
WDR18	0.887202	0.343558	-0.64754	4.148164
PLEKHA7	0.887202	0.343564	0.41372	7.447932
CASC2	0.887202	0.343601	-1.65797	2.137168
AQP1	0.887202	0.34372	-0.89391	3.454005
GFPT1	0.887202	0.343835	0.950232	2.611709
ZFHX4	0.887202	0.343909	-0.58633	5.833757
SNORD20	0.887202	0.343968	-0.86326	4.140351

TPR	0.887202	0.344049	-0.42755	9.28954
MIR4435-2	0.887202	0.344141	-0.974	3.320536
FUCA1	0.887202	0.344189	0.427231	5.649833
SNORA80B	0.887202	0.344222	0.667073	3.922094
MEIOC	0.887202	0.344231	-1.45421	1.924064
CYTH3	0.887202	0.344533	-0.50882	6.291069
GUCA1B	0.887202	0.344633	1.001909	2.577942
ZNF841	0.887202	0.344646	-0.52548	6.865045
ACYP2	0.887202	0.344647	-1.1677	2.043745
CYGB	0.887202	0.34468	0.524721	4.638331
OTOAP1	0.887202	0.344796	-1.64032	1.457227
PKM	0.887202	0.344927	-1.78037	1.162392
BTG2	0.887202	0.344969	0.426762	8.630699
USP42	0.887202	0.345001	-0.52891	5.898957
EXOC2	0.887202	0.345214	1.407411	1.03906
ZBTB45	0.887202	0.345253	1.366041	1.575354
PLEKHM1P	0.887202	0.345269	-0.46247	5.861889
SMG6	0.887202	0.345344	-0.44004	6.809156
ADAM23	0.887202	0.345388	-1.09154	4.667957
SERPINB8	0.887202	0.345542	-1.64824	0.65655
MED22	0.887202	0.345619	0.498221	4.776228
VSIG10	0.887202	0.345674	0.575086	5.628781
SLC22A13	0.887202	0.345688	1.194581	1.438364
UBE2Z	0.887202	0.345713	-0.42879	6.906305
COLCA1	0.887202	0.345745	1.36138	0.992857
AKAP7	0.887202	0.34579	-1.05919	3.064673
MPL	0.887202	0.345896	-0.89813	2.956535
GRAMD1C	0.887202	0.345987	-0.95235	2.673606
KCNK15-AS	0.887202	0.345989	1.483084	1.659554
NAT10	0.887202	0.346132	-1.00514	2.903007
TXNIP	0.887202	0.3463	-0.57662	7.375963
ACOT7	0.887202	0.346304	1.165816	1.743782
MAN1B1-D	0.887202	0.346324	-1.91953	0.571672
TNFAIP2	0.887202	0.346326	-0.50954	7.7668
FNBP1	0.887202	0.346359	-0.53474	7.750608
SDC3	0.887202	0.346364	-0.42191	7.035769
HELZ	0.887202	0.346474	-0.40815	8.181565
NRIP3	0.887202	0.346483	-1.08406	2.538507
SPIRE2	0.887202	0.346607	0.507195	6.027372
HYMAI	0.887202	0.346668	-1.04517	2.981655
RNF144A-A	0.887202	0.346873	-1.37335	1.249476
RIBC2	0.887202	0.34715	-1.50145	1.607532
GEMIN6	0.887202	0.34722	0.644705	3.780466
SNORA47	0.887202	0.347271	0.641224	7.049678
PI4KA	0.887202	0.347275	-0.41232	7.479621

SHPRH	0.887202	0.347375	-1.40783	1.752678
DHX57	0.887202	0.347405	-0.4755	5.579973
SPDYE6	0.887202	0.347415	-0.60282	4.600444
NUS1	0.887202	0.347443	-0.53496	5.964696
HGSNAT	0.887202	0.34749	-0.41342	7.131352
SPTSSA	0.887202	0.347522	-0.61234	5.096776
LINC02097	0.887202	0.347666	-1.45081	1.819991
SPINDOC	0.887202	0.347869	-0.65301	3.951761
IPPK	0.887202	0.347882	0.483844	5.171663
FAM117B	0.887202	0.347916	0.437731	5.770602
GRPEL1	0.887202	0.34794	0.605791	4.638257
HMGN4	0.887202	0.348043	-0.43707	5.730002
CCT6P3	0.887202	0.348116	-0.69373	4.271778
IQCD	0.887202	0.348119	1.096495	2.161434
LINC00314	0.887202	0.348138	-2.37484	1.138846
RNF216-IT1	0.887202	0.348142	-1.01501	2.435258
TTC9	0.887202	0.348219	0.855804	4.353434
ARHGEF38	0.887202	0.348246	0.591229	5.971514
SDC2	0.887202	0.348256	0.504842	5.911772
YWHAZ	0.887202	0.34827	0.538753	4.494646
NECTIN1	0.887202	0.348306	0.593374	5.958573
YAP1	0.887202	0.348321	-2.18108	0.731261
ERN2	0.887407	0.348554	1.125098	1.952428
HS3ST2	0.887407	0.348629	1.514069	1.173647
ACRBP	0.887407	0.348632	0.729482	3.066288
ANGEL1	0.887407	0.348731	0.443444	5.301498
HOXC-AS3	0.887407	0.34874	-1.78199	1.098301
TMEM104	0.887855	0.34905	0.455015	4.872545
ANK2	0.887855	0.349051	-0.64374	4.569201
URAHP	0.887939	0.349152	-1.34647	1.722892
GNAZ	0.888213	0.349428	-1.22843	3.664818
TRABD2A	0.888213	0.349445	0.834423	2.922388
LOC100131	0.888213	0.34959	-0.68068	3.752133
SNORD52	0.888213	0.349606	-1.18659	2.842171
VGLL4	0.888213	0.349648	0.53698	4.143304
ARSK	0.888213	0.34973	-0.78948	3.206183
CD300LB	0.888213	0.34974	1.476095	1.325311
PLA2G4E-A	0.888213	0.349801	1.750992	1.341104
RUVBL1	0.888258	0.349966	-0.47371	5.669124
GNAI1	0.888258	0.349981	-1.18768	3.259322
LY75	0.888258	0.350091	0.59877	4.239426
RPS4Y1	0.888258	0.350101	0.982903	6.840068
SLC25A24	0.888258	0.350236	0.909209	2.59238
KCTD18	0.888258	0.350249	-0.54266	4.788335
ASPSCR1	0.888258	0.350359	1.05553	1.805494

NDRG1	0.888258	0.350361	0.880182	4.288203
CLEC12A	0.888765	0.350629	1.431151	1.463158
MMP17	0.889106	0.350831	-1.57833	2.563923
CASTOR2	0.889119	0.350928	-0.57444	5.87513
KTN1-AS1	0.889119	0.350971	1.58262	1.177276
IGFBP1	0.889182	0.351097	-2.44415	1.88137
CHMP1B2F	0.889182	0.351144	-0.90992	4.571931
GPC6-AS2	0.889182	0.351213	-1.31692	2.110268
CLEC4E	0.889182	0.351268	0.888355	3.448468
LOC100132	0.889865	0.351605	0.934373	2.457988
CCSER1	0.889945	0.351705	-0.96772	3.551078
PAPOLG	0.890186	0.351868	-0.43428	6.022742
SYNGR3	0.890592	0.352097	-1.72079	0.673571
LOC257396	0.890592	0.352193	1.410533	1.848552
TBKBP1	0.890592	0.352305	-0.66968	4.353861
CRY1	0.890592	0.352338	-0.6227	5.356725
SCARNA10	0.890592	0.352368	-0.40765	7.781099
SH3KBP1	0.890804	0.352633	0.671581	3.092288
LINC00562	0.890804	0.35265	-1.59275	1.11519
GNA14	0.890804	0.352655	0.806624	3.657388
FBXO2	0.890925	0.352878	-1.03722	3.043034
TMPRSS11	0.890925	0.352917	1.783603	3.074054
SLC2A12	0.890925	0.352963	-0.60155	4.651947
PABPC1L	0.890925	0.353049	-0.59558	5.102687
MRPL3	0.890925	0.353197	-0.44636	6.413882
IL6R	0.890925	0.353266	-1.66354	0.990488
KLKB1	0.890925	0.353297	1.059732	1.91592
C9orf66	0.890925	0.353374	-1.92699	0.813283
BTBD18	0.890925	0.353509	-0.82738	3.053763
RTCB	0.890925	0.353791	0.402368	6.525604
GPRC5D-AS	0.890925	0.353896	0.721538	3.155951
LOC101929	0.890925	0.353958	1.09439	1.880965
AEN	0.890925	0.353983	-0.53276	5.089474
RANBP2	0.890925	0.353985	-0.40724	9.317649
SIMC1	0.890925	0.354022	-1.34712	1.452451
ZNF699	0.890925	0.354037	-0.88116	3.489887
RASA3	0.890925	0.354069	-0.42249	6.59482
LMAN2	0.890925	0.354123	0.417351	6.773361
LOC101927	0.890925	0.354171	1.109375	2.370416
TTC24	0.890925	0.354247	1.571111	1.691844
CRLF1	0.890925	0.354264	-1.53795	1.661668
SCARNA22	0.890925	0.354304	0.652503	3.956645
CACNA1S	0.890925	0.354356	-1.94219	1.253633
CNGB3	0.890925	0.354411	-1.67914	1.5196
CENPU	0.890925	0.354477	1.588241	1.115188

MESD	0.890925	0.354515	0.538125	4.398776
PCNX4	0.890925	0.354537	0.445398	5.66536
C1GALT1	0.891117	0.354681	-0.44977	7.173998
LOC101928	0.89127	0.354843	0.801768	2.858078
ZNF763	0.89127	0.354917	-1.08822	2.888725
COQ2	0.89127	0.354999	-0.62218	3.988793
WASH2P	0.89127	0.355013	-0.63839	4.783167
SLC2A4	0.89139	0.35522	-0.95549	3.817328
HAO1	0.89139	0.355259	1.763039	0.974116
MIRLET7BH	0.89139	0.355265	-1.24455	1.8905
SERP2	0.891422	0.355346	-1.71085	1.257989
ESCO2	0.891525	0.355502	-0.80483	3.681593
TRMT12	0.891525	0.355523	0.589498	4.464268
PUM3	0.891677	0.355651	-0.4346	6.805312
RPLP2	0.891898	0.355844	0.463148	9.451157
MRPS26	0.891898	0.355875	-0.58482	5.042107
NPIPB13	0.892121	0.356137	-0.46235	8.406278
ANKRD16	0.892121	0.356172	1.138067	1.679036
ABCC1	0.892121	0.35624	-0.51316	5.493452
ATG14	0.892121	0.356312	0.408642	6.194181
FAM122B	0.892121	0.356478	-0.72302	3.570232
ZSWIM8-AS	0.892121	0.356534	-1.07732	2.790389
TWIST1	0.892121	0.35654	-1.00303	4.6367
TAF7	0.892121	0.35656	-0.50279	7.257945
PDK1	0.892121	0.356696	-1.34044	1.30699
ARHGEF18	0.892121	0.356716	0.923103	2.153223
ZNF213-AS	0.892121	0.356772	-0.84833	3.453774
LOC101927	0.892121	0.35678	1.020154	2.40165
SZT2	0.892285	0.357004	-0.40158	7.986084
PTPN11	0.892285	0.357043	-0.43393	7.494762
DGKZ	0.892285	0.35705	-1.42386	1.548058
TTC12	0.892379	0.357156	-0.67289	4.507891
LOC642366	0.892446	0.35725	-1.44663	2.668054
TMEM238	0.893159	0.357805	0.599488	3.824897
ZC3H7A	0.893159	0.357839	-0.41736	6.948475
NCAPD3	0.893159	0.357923	-0.48222	6.091218
PKN2	0.893159	0.357945	0.381733	7.575992
POU6F1	0.893159	0.35799	-1.50084	1.388088
ZNF25	0.893159	0.358091	-0.57457	4.886726
ZNF225	0.893159	0.358157	-0.59782	4.548461
ATP1B3	0.893159	0.358205	-0.52398	6.559394
CLCA2	0.893159	0.358217	1.666735	5.332011
GAL3ST2	0.893159	0.358267	1.392536	2.26303
ANKRD13C	0.893159	0.358285	0.440752	6.367295
NAGK	0.893272	0.358525	0.416433	5.876914

STAG3L5P-	0.893272	0.358595	-1.33363	1.899386
NDUFAF8	0.893272	0.358616	-0.56637	4.778458
ZNF787	0.893272	0.358811	-0.59864	4.930781
MTBP	0.893272	0.358869	-0.9186	3.669661
C19orf54	0.893272	0.35902	0.559727	4.643569
RXYLT1	0.893272	0.359083	-1.68027	1.446914
WASF3	0.893272	0.359119	1.457394	1.046144
NDP	0.893272	0.359225	1.787944	0.998675
RHNO1	0.893272	0.359302	-2.10964	0.925753
OPA3	0.893272	0.359311	0.40848	5.813738
TACC3	0.893272	0.359329	-0.47359	5.524125
LOC100288	0.893272	0.359356	-0.8003	3.375695
SBF2-AS1	0.893272	0.359484	0.960259	2.976416
GRIN3A	0.893272	0.359572	0.978962	2.46225
FLJ42393	0.893272	0.359624	-0.63895	5.283314
SNX29	0.893272	0.359652	-0.48026	6.705076
SNHG6	0.893272	0.359687	-0.57718	5.652008
CHRM3-AS	0.893272	0.359722	1.018454	3.06167
EIF5B	0.893272	0.359774	-0.3962	9.153676
C3orf36	0.893272	0.359889	1.466194	1.83306
LRRC37A6F	0.893272	0.359989	-1.68056	2.148074
AGO4	0.893272	0.360078	-0.40262	6.822418
LINC00673	0.893272	0.360094	-0.82866	4.806132
BTN2A3P	0.893272	0.3601	0.619255	3.815191
GPR137B	0.893272	0.360167	-0.56772	5.251331
ADAMTS18	0.893272	0.360168	1.078891	2.689676
ULK4	0.89337	0.360276	0.434035	5.936583
FNDC3A	0.893406	0.360389	0.603711	3.567229
COL6A2	0.893406	0.360437	0.499862	5.578917
JAG1	0.893406	0.36056	-0.41926	7.719027
RNVU1-7	0.893406	0.36098	-0.41084	7.13606
MC1R	0.893406	0.360988	-0.83081	3.081353
LSM10	0.893406	0.361025	-0.7117	3.910356
CDC5L	0.893406	0.361088	-0.39171	7.039344
LINC01940	0.893406	0.361093	1.473129	0.805151
ZNF726	0.893406	0.361147	1.289854	2.22949
GCA	0.893406	0.361187	-0.57886	4.30588
VPS26B	0.893406	0.361301	0.398901	6.317259
SUGT1P3	0.893406	0.36139	-1.19273	2.601015
INTS8	0.893406	0.361405	-1.84222	0.649125
KDR	0.893406	0.361452	0.462342	5.434251
MCAT	0.893406	0.361454	1.201796	1.30328
PROB1	0.893406	0.361513	0.735771	3.279824
NEK5	0.893406	0.361534	-0.8678	3.221728
PCDH7	0.893406	0.36155	-2.01894	1.129304

MAP10	0.893406	0.361584	-0.92009	3.373501
TKT	0.893522	0.361699	-1.41533	1.394805
NFKBIZ	0.89374	0.361856	-0.97306	2.147691
SEMA5A	0.893753	0.361957	0.485011	6.40638
DIRAS1	0.893753	0.361997	0.795692	3.285013
PRKCH	0.893888	0.362152	-0.60299	5.38329
PKP1	0.893888	0.362188	1.824268	0.82324
ADAMTSL4	0.894059	0.362325	-0.65163	4.329506
PAXIP1-AS1	0.8942	0.362504	-0.97863	3.219244
HIRIP3	0.8942	0.362799	0.577509	5.160547
GOT1	0.8942	0.362862	0.457409	5.248966
NAA25	0.8942	0.362905	-0.45288	6.735379
ARPC1B	0.8942	0.362922	-0.51998	7.033755
GAS6-DT	0.8942	0.362929	1.10915	2.33851
BBS7	0.8942	0.363071	0.91332	2.387888
DHRS9	0.8942	0.363204	-1.76638	2.974362
ELOVL2	0.8942	0.363214	1.296089	1.265261
SNORD14E	0.8942	0.363222	0.844323	3.168657
SYF2	0.8942	0.363234	0.812986	2.489878
ATG2A	0.8942	0.363309	-0.41214	6.256864
SLC6A4	0.8942	0.363396	-1.29055	1.473153
LOC100507	0.8942	0.363586	-1.00106	3.174445
EPS8	0.8942	0.363771	-0.44177	7.564477
LRRC8D	0.8942	0.363772	1.037343	2.022042
PRPF18	0.8942	0.363959	-0.43845	5.942167
SCAMP2	0.8942	0.364069	0.431912	5.932981
HOXC10	0.8942	0.364174	-0.82283	5.772972
LOC100130	0.8942	0.364251	-1.42108	1.639061
SRPK2	0.8942	0.364312	-1.18418	2.220766
N4BP2L2-IT	0.8942	0.364357	-0.46806	6.205328
LGALS1	0.8942	0.364363	-0.56584	5.434726
BDNF	0.8942	0.364486	1.388884	0.833786
LOC100507	0.8942	0.364592	1.437183	1.020892
TPRG1L	0.8942	0.364757	0.458137	5.514867
RNF41	0.8942	0.364762	-1.40381	1.511267
CACNA1C-I	0.8942	0.364781	-1.276	1.859891
ARHGAP26	0.8942	0.364802	0.942029	2.76544
KCNQ1OT1	0.8942	0.364836	-0.5344	10.3969
APOBEC3D	0.8942	0.364865	0.678135	3.335909
CDH20	0.8942	0.364915	-1.9715	1.272708
MED23	0.8942	0.365007	-0.93767	3.062155
RNPEP	0.8942	0.365048	0.442769	6.755787
MAGOH	0.8942	0.365095	-0.56408	4.876113
SERINC5	0.8942	0.365208	0.393666	7.424813
DDX60L	0.8942	0.365274	-0.56486	5.153916

YY2	0.8942	0.365393	-0.98124	2.628153
LRRC14	0.8942	0.365449	-1.96178	1.243278
RNF168	0.8942	0.365634	-0.39725	7.0983
PTPN21	0.8942	0.365668	-0.45891	5.991558
EXOC5	0.8942	0.365723	-0.4366	6.606315
POC1B	0.8942	0.365724	1.49128	0.917413
WARS2-AS1	0.8942	0.365727	-0.75095	4.116245
ADRA2C	0.8942	0.365744	-1.79942	1.110923
THAP2	0.8942	0.365868	-1.06949	3.143589
SCARA3	0.8942	0.365913	-0.89007	3.798232
GPD2	0.8942	0.36606	1.274606	1.810677
IGFBP2	0.8942	0.366065	-1.10783	2.254351
PTPRD	0.8942	0.366374	-1.47292	1.536032
GALNT4	0.8942	0.366384	1.094025	3.12064
LOC101927	0.8942	0.366386	-1.08018	2.561839
DNAJC13	0.8942	0.36646	-0.38424	7.836032
RAMAC	0.8942	0.36649	-1.08498	2.296667
GDPD3	0.8942	0.366504	0.626437	4.345096
H1FX	0.8942	0.366567	0.40962	6.680421
SLC1A4	0.8942	0.366638	-0.87026	2.942141
TGM1	0.8942	0.366787	1.379105	6.119665
DIPK1A	0.8942	0.366797	0.511142	4.284604
MCF2L	0.8942	0.3668	0.835203	2.496215
KATNBL1P6	0.8942	0.366859	-1.50245	1.388617
FOXP2	0.8942	0.36689	-1.46275	2.560666
SOCS2-AS1	0.8942	0.367033	-1.36114	1.575664
RAB18	0.8942	0.367255	0.916127	2.607205
A2M	0.8942	0.367269	-0.59452	10.98859
OTUD3	0.8942	0.367323	-0.44113	6.371002
CREG1	0.8942	0.367342	-0.41839	7.146996
ARHGAP27	0.8942	0.367472	-0.44077	6.730888
PDE5A	0.8942	0.367508	0.849052	3.00637
PPP2R3A	0.8942	0.367523	-0.939	4.402993
ATP6V0D1	0.8942	0.367559	0.497978	5.260402
RAB30-AS1	0.8942	0.367604	-0.56423	4.595722
LOC101928	0.8942	0.367609	-1.67571	1.128641
POMT1	0.8942	0.367629	1.186309	1.5216
MYO15A	0.8942	0.367639	-1.18693	3.132458
MINK1	0.8942	0.367645	1.504081	0.849138
LOC100128	0.8942	0.36768	0.500368	5.876783
CDYL2	0.8942	0.367833	-0.6041	3.87385
C3orf38	0.8942	0.367891	-0.54335	4.470873
PAPSS1	0.8942	0.367894	-0.4291	6.017753
TMEM17	0.8942	0.367972	-1.33121	1.560337
BICRA	0.8942	0.367987	-0.46122	5.310929

PFKFB4	0.8942	0.368048	1.220955	1.510606
ZNF440	0.8942	0.368108	-0.55513	5.273422
LYPD2	0.894201	0.368177	1.800188	1.729256
NBPF12	0.894225	0.368254	-0.47349	6.220218
ICOS	0.894263	0.36837	-1.50486	1.938171
HJURP	0.894263	0.368407	-1.78368	0.783581
POTEE	0.894381	0.368537	-1.00214	3.356254
BRICD5	0.894381	0.368596	-1.19957	1.941017
TMEM205	0.894381	0.368659	1.229255	1.638133
GSPT1	0.894425	0.368746	1.763168	1.849208
HABP4	0.894443	0.368854	-0.70793	5.347135
RASA1	0.894443	0.36889	0.572708	3.765695
BSG	0.894996	0.369186	-1.43602	0.759309
ACTG2	0.895089	0.369335	-0.94181	4.368828
LOXL4	0.895089	0.369398	-1.01009	3.811419
C12orf73	0.895089	0.369444	-0.7784	3.012729
NBPF1	0.895089	0.369564	-0.54368	4.829169
JAKMIP3	0.895089	0.369652	-0.97111	3.749569
PRSS53	0.895089	0.369653	-0.94304	2.854424
COMMD2	0.895089	0.369754	-0.45645	5.302172
UBXN10	0.895089	0.369784	-0.5554	4.255109
DOCK3	0.895089	0.369941	-0.70043	4.509521
SERINC2	0.895089	0.369951	-0.98562	3.35333
SLC25A25	0.895089	0.369977	1.11528	1.89899
PROM2	0.895089	0.370043	0.537599	3.976177
FMNL2	0.895189	0.370152	-0.40801	7.430795
HPF1	0.895193	0.370251	-0.80395	3.334511
IRS1	0.895193	0.37029	-0.4978	6.650827
MECP2	0.895233	0.370375	-0.44642	6.509637
TOB1-AS1	0.895385	0.370507	1.306596	0.954879
BTBD8	0.895443	0.370599	0.800253	3.158365
ZNRF1	0.89602	0.371001	0.435922	5.204418
GON7	0.89602	0.371013	0.79467	2.756856
CHD1	0.89602	0.371042	-0.38252	7.749927
CHM	0.8961	0.37117	-0.46521	5.47264
LOC286297	0.8961	0.371352	-1.17444	2.511952
ARMCX1	0.8961	0.371406	-0.84383	3.638652
OCIAD1	0.8961	0.371486	1.334157	1.374422
SLC36A2	0.8961	0.371549	1.430164	1.180175
ARC	0.8961	0.371611	-1.77195	1.29195
ZNF777	0.8961	0.371672	-0.58652	4.982995
USP30-AS1	0.8961	0.371747	1.311645	1.717488
FOXP1	0.8961	0.371774	0.541034	4.781067
ATG2B	0.8961	0.371822	0.371542	7.247271
FAM238C	0.8961	0.371919	-2.0901	1.052182

SERPINB5	0.8961	0.371961	-0.82317	7.556905
RUBCN	0.8961	0.371967	0.702008	2.892675
GIMAP1	0.8961	0.372032	0.745168	2.954864
ARHGAP26	0.896112	0.372185	0.913128	2.888371
LOC642852	0.896112	0.372191	-0.45839	5.340206
KLF3	0.896112	0.372303	0.404953	8.082042
SLC6A10P	0.896112	0.372318	-1.82082	1.97963
ARF5	0.896112	0.372415	0.413078	5.550839
PSMB7	0.896112	0.372548	-0.54673	5.120804
ADGRF5	0.896112	0.372569	0.98721	1.741803
CEP68	0.896112	0.37263	-0.4375	6.345437
CCL13	0.896112	0.372651	1.277583	1.811558
RGPD3	0.896654	0.372945	-1.18588	2.232298
SBF2	0.896686	0.373101	-0.42997	7.174848
RAMP1	0.896686	0.373117	-1.7066	1.920959
MAP2K2	0.896686	0.373431	0.383658	6.750348
PROC	0.896686	0.373511	1.01224	2.186543
MIR3170	0.896686	0.37357	1.533184	1.221238
DYNC1LI2	0.896686	0.373625	0.540372	4.151386
DYRK1A	0.896686	0.373633	0.719715	4.184968
LINC00327	0.896686	0.373642	-1.5956	1.681839
BCL3	0.896686	0.373651	-0.52185	5.240396
KIAA0513	0.896686	0.373664	1.064648	2.081145
LANCL2	0.896686	0.37371	-0.45746	4.946565
RALBP1	0.896777	0.373858	-0.50584	8.146359
RPS18	0.896777	0.373954	0.687343	6.197359
MBNL2	0.896777	0.374	1.228356	1.594322
PTRHD1	0.896777	0.374039	0.928774	2.605166
KLRG1	0.896777	0.374163	-1.35241	1.741408
FBN1	0.896777	0.374205	-0.51409	8.890677
CTSH	0.896777	0.374247	-0.44702	6.1924
EIF2S3	0.896777	0.374379	-0.42654	8.375415
HEXA	0.896777	0.374382	0.394435	6.081574
IGSF6	0.896777	0.374475	0.616098	4.177966
NAGS	0.896777	0.374535	-1.53096	1.781245
SNORD55	0.896777	0.374771	-0.73548	7.497865
SUCNR1	0.896777	0.374798	-1.70054	1.476241
STAU2-AS1	0.896777	0.374824	0.910605	2.137972
RNF141	0.896777	0.374831	0.388855	6.155789
ARHGAP24	0.896777	0.374963	0.680513	3.367458
KLHL17	0.896777	0.374974	0.624783	3.633225
ABHD17B	0.896777	0.37505	1.003306	2.428444
TMEM177	0.896777	0.375056	1.175184	1.796552
RPL13AP20	0.896777	0.375145	0.750491	3.150245
LINC01762	0.896777	0.375244	1.841084	1.236326

SUN2	0.896777	0.375438	-1.25944	2.13059
EMC3	0.896777	0.375454	-0.5458	4.689314
TBC1D8	0.896777	0.375702	-0.39882	7.183299
ACTR3C	0.896777	0.37571	1.401497	1.114948
MAP3K8	0.896777	0.375742	-1.15903	2.376284
SPIN1	0.896777	0.375747	-0.48771	6.749879
STMP1	0.896777	0.375803	-0.49583	5.202712
HOXD10	0.896777	0.37584	-2.04286	1.864821
CCDC71	0.896777	0.375875	-0.6214	3.723895
CDR1	0.896777	0.375887	-0.76118	5.656527
CHPF2	0.896777	0.375995	0.570074	4.564958
COL5A3	0.896777	0.376087	0.444356	6.139884
SLCO1B3	0.896777	0.376088	-1.66045	4.17446
UQCRBP1	0.896777	0.376151	-0.94221	2.417844
MRPS9	0.896777	0.376209	-0.52022	5.2541
PKD1L3	0.896845	0.376306	0.864839	2.796341
SUCLA2	0.896915	0.376468	-0.46942	5.574526
EZR	0.896915	0.376555	1.198966	0.881711
TSPYL4	0.896915	0.376607	0.485902	5.822347
ZNF19	0.896915	0.376679	-1.19186	2.86585
ZNF33BP1	0.896915	0.376744	-1.28241	2.155557
CHMP2B	0.896915	0.376748	-1.50269	1.282056
ZNF106	0.896915	0.376814	-0.43318	6.958354
FAM78B	0.896997	0.376943	1.278183	1.322583
CHD6	0.896997	0.37707	-0.40184	7.888573
MED31	0.896997	0.377324	-1.45634	1.853908
ECPAS	0.896997	0.377324	-0.39561	8.214581
KIRREL1	0.896997	0.377385	-1.57421	0.862556
CEP152	0.896997	0.377411	-1.61978	1.18957
ETAA1	0.896997	0.377438	-0.43012	5.563125
LINC02584	0.896997	0.377575	-1.22384	3.214782
SNAI3-AS1	0.896997	0.377586	0.888628	2.513331
MIR101-1	0.896997	0.377595	-2.2118	1.446734
SLFN13	0.896997	0.3776	0.455204	6.324142
SPC25	0.897488	0.377875	1.341765	1.475508
LOC101928	0.898017	0.378167	-0.86093	3.167795
HSH2D	0.898493	0.378436	1.056669	2.376971
KL	0.898603	0.37855	1.024277	1.890002
SUGP1	0.898727	0.378746	-0.44959	5.296142
FAM104A	0.898727	0.37877	1.31364	0.874471
GLDC	0.898727	0.37883	1.262458	1.741017
TSR1	0.898727	0.378945	-0.44184	6.337359
CTAGE1	0.898727	0.379079	1.197333	0.897093
RFC3	0.898727	0.379106	-0.62573	3.679153
HIST3H2A	0.898727	0.379145	-1.10952	2.178817

CCDC28B	0.898727	0.379239	-1.40624	1.85481
PCK2	0.898727	0.379378	0.831261	1.809343
FAM206A	0.898727	0.37939	-0.62211	4.340726
CLDN15	0.898727	0.37942	0.702514	3.286617
AMBN	0.898727	0.379425	1.959843	0.870723
WBP11	0.898989	0.379603	-0.40657	6.931754
HIF3A	0.899393	0.379843	-1.87028	1.241392
MIR320E	0.899813	0.380089	1.119717	1.76673
COA5	0.899858	0.380217	0.457139	5.436832
ARNTL2-AS	0.899858	0.380307	1.071609	2.110233
TNR	0.899858	0.380313	1.018879	2.16019
CUL5	0.899969	0.380599	-0.41949	6.875875
CHAF1A	0.899969	0.380615	-0.41164	6.187202
FCER1G	0.899969	0.380735	-0.81995	4.223089
GUSBP11	0.899969	0.38088	-0.49026	5.741204
PTCH1	0.899969	0.380905	0.832031	2.893951
UTP6	0.899969	0.380972	-0.39367	6.640282
CMYA5	0.899969	0.381154	-0.63018	5.210666
HUS1B	0.899969	0.381181	1.184275	1.867481
LZTS1-AS1	0.899969	0.381386	1.528485	1.343779
CCT3	0.899969	0.381454	0.976725	2.282238
AGAP3	0.899969	0.381466	-0.85656	2.726752
ZNF687	0.899969	0.381467	-1.53771	1.033013
TMEM51-A	0.899969	0.381494	0.665343	3.371025
PRPH2	0.899969	0.38167	0.781551	3.020759
GPRC5C	0.899969	0.381676	1.143941	1.369077
DCP1A	0.899969	0.381744	1.186582	2.065992
DCC	0.899969	0.381907	1.013979	2.684493
COBL	0.899969	0.381963	0.530197	4.5829
GDI1	0.899969	0.381972	-0.3879	6.788694
PDK3	0.899969	0.381975	-0.50576	6.604621
KIAA1257	0.899969	0.382034	-0.97742	3.043743
VSIG1	0.899969	0.382192	1.476799	0.962266
LBX2-AS1	0.899969	0.38227	0.561078	3.929265
LMBRD1	0.899969	0.382383	-0.44145	6.018441
LRRC61	0.899969	0.382402	-1.86103	0.620261
TAF1D	0.899969	0.382492	-0.46813	7.130755
PAQR6	0.899969	0.382589	1.269302	1.084138
CAMK1	0.899969	0.382598	0.47792	4.381901
FBXW9	0.899969	0.382672	-0.99852	3.035871
RIPOR1	0.899969	0.382772	0.92638	2.540046
SNORD5	0.899969	0.382816	-1.11626	3.15462
GPR35	0.899969	0.382888	-1.64907	1.298535
VAPB	0.899969	0.382957	-0.92169	2.986668
CHP2	0.899969	0.383068	1.67193	2.007695

MEIS2	0.899969	0.383098	0.822954	2.452627
EIF4EBP1	0.899969	0.383125	-0.51223	4.79671
CAMK1D	0.899969	0.383128	0.619075	4.321044
LOC10013[illegible]	0.899969	0.383167	-1.86327	0.914901
RPIA	0.899969	0.383191	-0.50253	4.619888
CD1C	0.899969	0.383227	-1.34964	2.359198
ENPP4	0.899969	0.383229	-0.7609	4.74959
MIR650	0.899969	0.383241	0.981573	2.320491
SPEF2	0.900439	0.38351	0.761133	3.130008
TMEM35A	0.900601	0.383648	-1.97097	1.368269
INIP	0.900691	0.383815	-0.72349	3.994754
CDX2	0.900691	0.383823	0.553181	4.325199
LAMP3	0.901144	0.384085	-0.87848	3.962613
KDM4A-AS	0.901433	0.384477	0.910795	2.214253
TCF21	0.901433	0.384564	0.637072	4.83719
PTPRH	0.901433	0.384594	0.63848	3.88359
CSAD	0.901433	0.384596	-0.84413	3.20404
TMA16	0.901433	0.384672	-0.482	4.770825
NF1	0.901433	0.384725	-1.07377	2.631338
CDC34	0.901433	0.38473	0.42478	5.128366
NR1H2	0.901433	0.384776	-1.03775	2.504949
TCFL5	0.901433	0.384872	-1.11726	2.804682
DOLK	0.901433	0.384895	-0.72516	3.66978
HMG20A	0.901561	0.385118	1.770541	0.780591
LINC01515	0.901561	0.385124	-1.76546	1.471184
MCPH1-AS	0.901561	0.385156	-0.99889	2.526655
GTF2H5	0.901645	0.385261	0.538044	4.229634
IRGQ	0.901691	0.385505	-0.38284	6.823996
RBPMS2	0.901691	0.385531	-0.93443	3.929406
RNF6	0.901691	0.385566	-1.37969	1.366385
SNHG3	0.901691	0.385591	0.991411	2.34428
RRP9	0.901691	0.385624	-0.64181	4.051724
CTSB	0.902041	0.385842	1.329877	1.179315
PI4K2B	0.902083	0.385981	0.423715	5.849672
FOS	0.902083	0.385998	-0.65682	8.844356
STK17A	0.902264	0.386144	-0.37798	6.246467
GAPDHS	0.902386	0.386355	0.636053	3.358918
TTYH2	0.902386	0.386428	-1.17373	1.87409
BMP4	0.902386	0.386464	-1.76024	1.724436
LINC01790	0.902386	0.386471	-1.96566	0.878247
ADH5	0.902792	0.386782	0.362975	6.850087
RNF157	0.902792	0.386867	0.710776	4.135261
C16orf87	0.902792	0.386974	-0.79681	3.600523
CRCT1	0.902792	0.387009	1.661238	3.700451
11-Sep	0.902792	0.387016	-1.20585	2.690314

IQCB1	0.902792	0.387101	-0.86986	3.230468
SNORA46	0.902792	0.387203	-0.75583	4.252261
SETD7	0.902792	0.387389	-0.45828	7.395651
THAP10	0.902792	0.387418	1.268222	1.229075
WDR73	0.902792	0.387541	-1.1036	1.958332
JPH3	0.902792	0.387624	1.476056	1.622394
PDE3A	0.902792	0.387646	-1.2304	2.811921
LOC100287	0.902792	0.387663	1.499126	0.758905
DNAJA1	0.902792	0.38771	0.592286	4.756289
ELL3	0.902792	0.387856	0.688316	4.309017
RIPK1	0.902792	0.387883	0.984175	1.789052
XPO1	0.902792	0.387909	-0.37952	8.149561
SGMS2	0.902792	0.387925	-1.57815	1.746419
CABIN1	0.902792	0.387981	0.923869	2.069392
SNORA67	0.902792	0.388022	0.479639	5.866538
LINC01959	0.903311	0.388337	1.272212	1.146705
CDH23	0.903311	0.388449	-1.33194	1.638332
SLMAP	0.903311	0.388451	-0.82502	2.463928
DPYSL3	0.903384	0.388552	-1.10732	3.125294
NUP50	0.903577	0.388725	-1.10639	2.274792
TRIM38	0.903577	0.388785	-0.41046	7.205787
RCN3	0.903577	0.388924	-0.44521	5.559667
RSL24D1	0.903577	0.388988	-0.5351	5.727329
ZNF37BP	0.903577	0.389002	-0.44823	6.90621
TDG	0.903577	0.389056	-0.56356	5.48403
ZNF490	0.903577	0.389117	-0.53277	5.005589
DUBR	0.903782	0.389415	-0.91828	3.663443
UBE2E2	0.903782	0.38946	-0.8022	3.980385
CCDC69	0.903782	0.389669	0.45565	6.606798
PCDHB16	0.903782	0.38975	-0.69549	3.922204
NACAD	0.903782	0.389827	-1.02486	3.063011
CCDC78	0.903782	0.389828	-0.72954	3.293332
ENTR1	0.903782	0.389858	-1.57252	0.742223
UBE2Q2P1	0.903782	0.389863	-0.48793	5.423725
FAM91A1	0.903782	0.389969	1.190866	1.424332
MTMR3	0.903782	0.389974	1.071827	1.828779
SNORA33	0.903782	0.390014	-0.53888	5.825204
FZD2	0.903782	0.390179	-1.30977	1.980829
TAB3	0.903782	0.3902	-0.41279	6.141454
MANCR	0.903782	0.39023	-1.78894	0.588681
TSIX	0.903782	0.390259	-1.5237	3.026108
SERPINB9P	0.903782	0.390307	-1.36084	2.493488
WDR20	0.904005	0.390472	0.660747	2.906036
CD58	0.904517	0.390786	0.647228	3.511816
ZMPSTE24	0.904517	0.390831	-0.42343	7.02937

DUSP6	0.904544	0.390963	-0.70897	3.190786
AHCTF1P1	0.904544	0.391041	-0.42312	6.670215
S1PR4	0.904544	0.39105	-1.62478	1.376827
ZDHHC24	0.9046	0.391332	-0.82942	2.77911
WTAPP1	0.9046	0.391403	1.001001	2.549217
A1BG-AS1	0.9046	0.391553	-1.19534	1.466811
THOC2	0.9046	0.391715	-0.39142	8.383937
GALNT2	0.9046	0.391762	-0.8745	2.363905
SOX21-AS1	0.9046	0.391819	-1.4976	2.431768
UBTD2	0.9046	0.391855	0.385166	5.587186
SGSH	0.9046	0.39186	-0.54371	4.680507
URB1	0.9046	0.391893	-0.41675	7.036938
RAB3GAP2	0.9046	0.391896	-0.36701	6.948377
VAC14	0.9046	0.391903	-0.41245	5.559368
LINC00857	0.9046	0.392099	-0.73897	3.339597
SQLE	0.9046	0.392116	0.494434	6.021895
INTS10	0.9046	0.392204	-0.37901	7.101812
PHKG2	0.9046	0.392302	-1.02942	2.312636
CCDC124	0.9046	0.392352	1.329389	0.73234
LOC10019(	0.9046	0.39237	-0.42836	6.524122
FUT3	0.9046	0.3926	1.24176	1.426308
SIAH2	0.9046	0.392612	-0.44553	5.123157
PIK3CD	0.9046	0.392634	-0.52189	5.10706
WDR90	0.9046	0.392671	-0.46066	5.975694
CNTNAP2	0.9046	0.39269	-1.71414	1.630707
SPRN	0.9046	0.392798	0.560076	4.548619
GPR22	0.9046	0.39283	-0.65892	3.523228
ABLIM3	0.9046	0.392845	1.617656	0.85581
ZGRF1	0.9046	0.392867	-0.51038	5.279587
IRF2BP1	0.904632	0.393029	0.48343	4.685282
MMP14	0.904632	0.393072	-0.385	8.346533
TICAM2	0.904632	0.393088	-2.7701	0.840961
LINC01372	0.904792	0.393286	0.996082	1.501517
TVP23C	0.904792	0.393297	0.385646	5.903915
HOXC-AS1	0.904792	0.393364	1.072611	2.507323
PCDHB3	0.904794	0.393433	-1.41835	1.635624
DPP7	0.904995	0.393637	-0.46061	5.543763
SNORD58A	0.904995	0.393659	-0.57884	6.306703
EPAS1	0.90501	0.393803	0.412482	8.35813
BCL2	0.90501	0.393803	0.686679	4.489681
NDUFAF3	0.905082	0.393927	-1.61826	1.657043
FZR1	0.905082	0.393973	0.998668	2.142109
MRPL51	0.905375	0.394493	0.426148	6.214962
LOC105377	0.905375	0.394668	-1.25538	1.746611
TPM3	0.905375	0.394752	-0.44869	6.243037

LINC00853	0.905375	0.394814	0.957303	1.412718
PSMB10	0.905375	0.394825	0.503971	4.898035
SGMS1-AS1	0.905375	0.394939	-1.44812	1.395903
DUSP7	0.905375	0.394943	-0.66152	5.028154
TRUB2	0.905375	0.395082	0.511732	4.767717
C12orf76	0.905375	0.395138	0.962244	2.764044
POC5	0.905375	0.395156	-1.0175	2.414078
ACOX1	0.905375	0.395158	-1.69896	1.335844
CWC15	0.905375	0.395164	-0.43503	5.651286
AARS2	0.905375	0.395189	-0.47347	5.638652
ASMTL-AS1	0.905375	0.3952	0.837437	1.805148
EFCAB2	0.905375	0.395271	1.268021	0.678927
ATF6	0.905375	0.39553	-0.41133	7.269234
RAP1A	0.905375	0.395593	0.827589	2.002684
NPIPA1	0.905375	0.395767	-0.98934	2.15612
ZRANB2-AS	0.905375	0.395811	1.660473	0.69395
IL15RA	0.905375	0.396062	1.280868	1.102379
CACNB1	0.905375	0.396142	-0.91746	3.509621
PLPBP	0.905375	0.396202	-0.43361	5.918026
AQP11	0.905375	0.396442	0.66246	3.31561
SLFN12	0.905375	0.396449	-1.38183	1.099304
SLC38A10	0.905375	0.396514	0.356437	6.131311
ZNRF2P1	0.905375	0.396533	-0.64275	3.805791
CANT1	0.905375	0.396549	0.946021	1.598283
VAC14-AS1	0.905375	0.396558	0.978796	2.346639
RYBP	0.905375	0.39658	0.390193	6.16836
SPIN2A	0.905375	0.396582	0.829998	2.340599
IFI30	0.905375	0.396739	-0.42584	6.952823
CEP170B	0.905375	0.396741	1.028799	1.424308
TGFA-IT1	0.905375	0.396799	-1.585	0.733347
TNRC6A	0.905375	0.396811	-0.3919	8.226559
SLC16A14	0.905375	0.396886	-0.85513	3.522536
C11orf71	0.905375	0.39701	0.877779	2.962143
TNFRSF11E	0.905375	0.397181	-1.07152	2.817002
DNAJC5	0.905375	0.39722	-0.38438	7.180563
POLR2J2	0.905375	0.397299	1.188248	1.139306
EPS15L1	0.905375	0.397373	-0.77095	3.528341
CCDC192	0.905375	0.397401	-1.75739	1.290809
C10orf90	0.905375	0.397422	-1.85656	0.719644
C12orf10	0.905375	0.397497	0.522249	4.156326
CYP4Z2P	0.905375	0.397533	1.429682	1.695559
BRAF	0.905375	0.397556	-0.43432	6.061819
RBMX	0.905375	0.397623	-0.36755	6.15422
QRFP	0.905375	0.397648	1.188922	1.40457
PLA2G4E	0.905375	0.397654	-2.00576	1.715582

ANXA2P2	0.905375	0.397718	0.390292	7.537101
AP3S2	0.905375	0.397799	1.213187	1.784271
STRAP	0.905375	0.397862	-0.45495	6.544736
LOC100131	0.905375	0.39802	0.551043	3.97248
KLHDC10	0.905375	0.398046	-0.39466	6.294292
ZNF319	0.905375	0.398053	-0.78247	3.661224
MAPKAPK3	0.905375	0.398074	-1.6684	0.992198
ANKRD20A	0.905375	0.398094	0.684123	3.347174
SNIP1	0.905375	0.398123	-0.51618	5.432128
FAM92B	0.905375	0.398143	-1.72799	0.704528
USP51	0.905375	0.398273	-1.44131	1.454961
FAM83A-A	0.905375	0.398386	1.324375	1.724651
CTAGE8	0.905375	0.398397	-1.37502	2.326183
TTC5	0.905375	0.398405	-0.50569	4.309938
TUFM	0.905375	0.398448	0.388361	7.23613
FAM135A	0.905437	0.398544	-0.78424	3.165178
ALKBH1	0.905832	0.398863	-0.67707	3.676324
GALR2	0.905832	0.399019	1.406347	1.571846
SHANK3	0.905832	0.399036	0.430734	6.397323
SMU1	0.905832	0.39916	-0.4069	6.791847
ADA	0.905832	0.399175	-0.66606	3.628013
PGK1	0.905832	0.399184	-0.44295	7.723859
AKAP1	0.905832	0.399201	0.757396	2.895999
RN7SK	0.905866	0.399285	-0.36106	14.5358
LOC100506	0.905979	0.399453	1.324681	1.072929
LNPEP	0.905979	0.399505	-1.91313	0.917342
CNTD2	0.905979	0.399645	1.134422	1.178479
THRB-AS1	0.905979	0.399755	1.071028	1.761298
NME3	0.905979	0.399877	-0.83047	3.640721
CCSAP	0.905979	0.399913	-0.51552	4.994204
RBL1	0.905979	0.399937	-0.88215	2.795005
PAN3	0.905979	0.400034	-0.38291	7.306446
FALEC	0.905979	0.400354	-1.67588	1.236394
DHRS7B	0.905979	0.400409	-0.64634	3.859259
NUP43	0.905979	0.400412	0.605443	3.529191
SLC25A29	0.905979	0.400762	0.815634	2.054274
TSHZ1	0.905979	0.40077	0.720954	2.470926
ARID3A	0.905979	0.400821	-0.52324	4.915152
IDI2	0.905979	0.400822	-1.00843	2.616543
DHRS12	0.905979	0.400833	0.824606	2.233455
ING2	0.905979	0.400853	-1.47643	1.176666
SNORD104	0.905979	0.40089	-0.6097	6.797698
CARHSP1	0.905979	0.400997	-1.32565	1.402603
CTSG	0.905979	0.401063	1.210554	1.576112
DNM1	0.905979	0.401068	-1.31607	1.642692

C3orf35	0.905979	0.401112	0.866252	2.606344
AGAP4	0.905979	0.40114	-1.15085	2.391534
KRT10	0.905979	0.401163	0.593527	5.088571
ANKRD11	0.905979	0.401251	0.584616	3.638271
GATAD2A	0.905979	0.401279	-1.8487	0.886626
KDM4B	0.905979	0.401305	0.370669	6.619784
OSR1	0.905979	0.401341	-0.76089	3.807169
TTPA	0.905979	0.401406	-1.56956	2.393757
DPY19L1P2	0.905979	0.401407	0.892671	2.898171
SNX13	0.906052	0.401508	-0.36862	6.972252
RPL39	0.906556	0.401853	-0.45926	6.119383
IK	0.906556	0.401899	-0.46346	8.112721
PCAT19	0.906556	0.401939	0.768019	2.484998
SGCB	0.906557	0.402053	-0.43661	6.269041
GTF3C2	0.906557	0.402077	-1.48537	1.85145
ITGB8	0.906818	0.402346	-0.41183	7.193506
ARL6IP6	0.906818	0.402466	0.802127	2.879252
TEX26	0.906818	0.402511	1.710086	1.037213
CARNMT1	0.906818	0.402525	0.408915	5.508642
GZMA	0.906818	0.402539	-1.11982	3.071667
DNAH10	0.907007	0.402692	-1.6114	1.287241
LOC728392	0.907159	0.402828	-0.79014	3.073019
ALKBH4	0.907377	0.402994	0.758516	2.958435
SEMA4G	0.908077	0.403395	0.680861	3.783231
UBE2F	0.908077	0.403443	1.366295	1.286815
CTDSP2	0.90813	0.40359	-0.37526	8.023178
OST4	0.90813	0.403606	0.492394	5.101544
ITIH5	0.908335	0.403818	1.505292	0.972938
MYH8	0.908335	0.403835	1.205125	1.531378
FRRS1	0.908421	0.403943	0.757506	3.986035
SNORA19	0.908613	0.404125	-0.84904	3.499794
CCDC33	0.908613	0.404166	-1.58656	0.864816
CARMN	0.908825	0.404393	-1.61555	2.437108
ASPRV1	0.908825	0.404399	-1.11362	2.076265
LGALS7B	0.909578	0.404866	1.614281	0.747364
C16orf86	0.909578	0.404876	0.991529	2.040094
FARP2	0.909578	0.404943	0.498148	4.235515
FAM90A1	0.909612	0.405081	0.738734	2.718089
TLR2	0.909612	0.405096	-0.58388	4.837126
ST20	0.909971	0.405479	-1.26366	1.723302
SEC62	0.909971	0.405489	-0.40065	8.649886
LINC02381	0.909971	0.405541	-0.82124	3.153275
LOC101926	0.909971	0.405591	-1.3175	2.044252
LOC644656	0.909971	0.405603	0.9577	1.886157
RTL1	0.910201	0.405796	0.992616	2.012973

GRB10	0.910201	0.405863	-1.17429	1.468957
ASNSD1	0.910201	0.406009	-0.43869	5.223274
PVRIG	0.910201	0.4062	-0.6343	5.099105
CASD1	0.910201	0.4062	-0.48913	5.638053
COX8A	0.910201	0.406303	0.398534	5.337449
NSRP1	0.910201	0.406313	-1.19682	2.217792
NDUFA6-D	0.910201	0.406348	-1.11767	2.205757
SGTB	0.910201	0.406386	-0.7372	4.593411
ZNF239	0.910201	0.4064	0.967734	2.446106
IL20	0.910201	0.406469	1.72442	0.788271
NOS1AP	0.910202	0.406604	0.642487	3.392143
CSK	0.910202	0.406682	-1.08826	1.311524
LINC00950	0.910202	0.406696	-1.10576	2.777895
MELTF	0.910202	0.40677	-0.72731	3.542054
STAT6	0.910202	0.406893	0.708914	3.690342
CYP4F29P	0.910202	0.40696	1.384895	1.550053
IGFLR1	0.910202	0.407037	0.787425	2.67509
LINS1	0.910202	0.407151	-0.79379	4.03642
SNORD54	0.910202	0.407184	0.629401	4.001631
C8orf33	0.910202	0.407197	-0.45632	5.260919
ZNF277	0.910202	0.407232	0.419709	5.375511
SNORD17	0.910749	0.407591	-0.4572	9.449928
IL1R1	0.910749	0.407683	0.851209	1.705452
NECTIN3	0.910749	0.407802	0.830269	2.295924
LMNB1	0.910749	0.407851	-0.92965	2.484239
PKD1L1	0.910749	0.408081	0.946765	3.282952
SOX2	0.910749	0.408174	0.855355	4.588083
ENTPD7	0.910749	0.408204	-0.39727	6.544658
REXO1	0.910749	0.408295	-0.40654	6.18784
TBL3	0.910749	0.408597	-0.52737	4.463813
KCNJ15	0.910749	0.408599	1.406383	0.78164
PDE11A	0.910749	0.408673	0.894596	3.122734
TSTD2	0.910749	0.408727	-0.47281	4.962147
GIPC1	0.910749	0.408767	-1.41557	0.626939
CLEC14A	0.910749	0.408872	0.555988	3.941342
LINC01873	0.910749	0.408908	-1.56514	1.486425
RPL5	0.910749	0.408978	0.362286	9.00965
GOLGA8J	0.910749	0.409061	-1.73988	0.964464
NACA	0.910749	0.409087	0.458902	4.402206
PON2	0.910749	0.409172	1.495902	0.963791
PNPO	0.910749	0.409178	0.391176	6.222214
ZNF345	0.910749	0.409311	-1.46237	1.177841
MSRB2	0.910749	0.409421	0.478332	4.25361
SSRP1	0.910749	0.409436	-0.39788	7.709789
CDC14A	0.910749	0.409461	-0.96043	2.637068

ZNF493	0.910749	0.409502	1.043484	2.510185
ACTR1B	0.910749	0.409504	0.359461	5.854166
SMG9	0.910749	0.409523	-0.41119	5.505365
HNRNPA3F	0.910749	0.409583	0.678886	2.897152
SDHAF4	0.910749	0.409608	0.592183	3.515702
PRIM1	0.910749	0.409631	-0.60032	3.87395
PIEZO1	0.910749	0.409773	-0.40004	7.870732
TNRC6B	0.910749	0.409864	-0.54637	4.548986
LINC00601	0.910749	0.409901	1.604876	1.025302
TPBG	0.910749	0.410023	-1.50134	1.106591
TMEM168	0.910749	0.410066	-1.19817	1.686573
NPDC1	0.910749	0.41007	0.442906	5.869775
PRPF6	0.910749	0.410071	-0.40232	7.688324
SORBS3	0.910749	0.410239	0.513716	3.920898
PPM1N	0.910749	0.410254	0.847574	2.342683
KCNV2	0.910749	0.4103	-1.07147	2.122967
DMWD	0.910749	0.410323	-0.38691	6.238914
UACA	0.911133	0.4107	-0.70174	3.406749
MYO1H	0.911133	0.410725	-1.28218	1.971603
FOXN3-AS2	0.911133	0.410985	0.907824	2.589664
LOC646214	0.911133	0.411004	0.826041	4.053941
CLPTM1	0.911133	0.411046	1.11285	1.32184
TMC8	0.911133	0.411129	0.497368	4.856009
DENND4B	0.911133	0.411131	-0.39477	6.474902
LOC100289	0.911133	0.411162	-1.59814	0.848337
COX5B	0.911133	0.411228	0.348662	7.151871
RGPD4	0.911133	0.411237	-1.19567	2.250777
L3HYPDH	0.911133	0.411328	0.713584	3.43498
CD81	0.911133	0.411329	0.841762	0.926268
CREBZF	0.91132	0.411523	-0.72605	4.282493
RPL13AP17	0.91132	0.411553	1.524247	1.104768
ERVH48-1	0.911471	0.41169	0.769924	2.783377
MIR3140	0.91208	0.412035	-1.59683	1.41307
COPRS	0.91225	0.412181	-0.7391	3.847171
AP1S1	0.912447	0.41234	0.46206	5.155084
TRRAP	0.912504	0.41246	1.370988	1.300425
NHLRC1	0.912504	0.412508	0.951812	2.579925
RPL21P44	0.912504	0.412574	0.780289	3.051441
PACSIN2	0.912622	0.412723	0.667314	3.849385
SLC25A33	0.912622	0.412823	0.777238	2.713209
LINC01341	0.912622	0.412836	1.140322	1.038523
KHDC4	0.912734	0.41306	-0.366	7.148736
UTAT33	0.912734	0.413086	1.208679	1.159844
SMARCD1	0.912734	0.413126	1.310162	0.763382
CSRNP2	0.912734	0.413188	1.336399	0.648921

NDUFA9	0.912734	0.413235	0.391319	5.743755
CSPP1	0.912962	0.413414	-0.92868	2.443353
TP53TG1	0.912962	0.413502	0.757833	3.153974
VPS25	0.912962	0.413547	-0.45503	4.667484
RAMP3	0.913145	0.413699	0.69109	2.905012
LINC00571	0.913248	0.413815	-1.0164	2.060215
CCNB2	0.913518	0.414007	-0.62704	4.30214
EPCAM	0.913682	0.414336	0.460007	9.192238
CFAP45	0.913682	0.414477	-0.90139	3.069966
LOC38990€	0.913682	0.414546	-0.52149	4.1915
ENGASE	0.913682	0.414575	0.389009	6.690674
KDSR	0.913682	0.414675	-0.48711	6.201149
WDR55	0.913682	0.414813	0.417003	5.843141
LRRC37B	0.913682	0.414912	-0.69778	4.511058
CCDC114	0.913682	0.415013	1.091564	2.46241
RXFP4	0.913682	0.415072	0.941515	1.951335
EPS8L2	0.913682	0.415198	0.407178	7.877253
UTP3	0.913682	0.415274	-0.50815	5.448858
FENDRR	0.913682	0.415322	0.509019	6.247697
ARHGEF7	0.913682	0.415581	0.959392	2.486714
CABLES2	0.913682	0.415639	-0.64374	4.060392
MAST3	0.913682	0.415641	0.361701	5.766916
MORN2	0.913682	0.415696	-1.20566	2.477444
TERF2	0.913682	0.415712	0.374342	5.898102
POLN	0.913682	0.415771	0.83869	2.722561
ZNF503-AS	0.913682	0.415841	1.090566	1.24659
MYH7B	0.913682	0.415877	-0.5523	4.779945
UBOX5-AS1	0.913682	0.415931	-0.94115	2.321623
C9orf50	0.913682	0.415947	-1.11119	1.991196
MUCL3	0.913682	0.415964	0.856607	6.802594
SPTBN4	0.913682	0.415992	-1.06396	2.155763
TTLL4	0.913682	0.416062	-0.39941	5.82181
LINC00260	0.913682	0.416112	-0.85343	3.359407
NUP88	0.913682	0.416186	-0.42251	5.888586
SH2D6	0.913682	0.416259	1.16775	1.752778
MYL12A	0.913682	0.416383	0.720262	2.52177
GOLGA3	0.913682	0.416461	0.345572	7.973467
PRRT3	0.913682	0.4165	0.546064	3.686568
ZNF324B	0.913682	0.416564	-0.82543	2.982169
DHX30	0.913682	0.416611	1.054404	1.191365
ELP6	0.913682	0.416672	0.541464	4.114485
EPRS	0.913682	0.416677	-0.38689	8.33259
RTL6	0.913682	0.416684	-0.37215	6.105635
OTUD6B-A	0.913682	0.416781	-0.45042	4.99499
MRPL41	0.913682	0.416782	0.525376	4.75386

LOC101927	0.913682	0.416907	0.865653	2.221816
PHF3	0.913682	0.416918	0.546541	3.794062
USPL1	0.913682	0.417022	-0.45986	5.737811
SUSD4	0.913682	0.417121	1.40262	1.615484
MRPL14	0.913682	0.417128	0.657979	4.45064
MIDN	0.913682	0.417146	-0.37029	7.390854
RIOX1	0.913687	0.417277	-0.58526	3.942995
PPP2CA	0.913687	0.417288	0.403719	6.811329
MIR2052H	0.913826	0.417435	1.355973	0.907485
ZKSCAN4	0.913826	0.417491	-1.13763	1.690828
RPS6KA3	0.914017	0.417647	0.347578	7.732662
ZNF606	0.914054	0.417866	-0.76283	3.183058
TMEM185	0.914054	0.417866	-0.37145	5.854524
EBPL	0.914054	0.417873	-1.54035	1.465658
NCMAP	0.914085	0.418013	0.665765	3.37295
RASAL3	0.914085	0.418071	0.517139	4.318185
DVL2	0.914085	0.418154	0.444811	4.889736
GJA5	0.914085	0.418181	-1.40603	0.730324
CCDC30	0.914085	0.418326	-0.7265	3.853229
TES	0.914085	0.418327	-0.73477	4.33443
NPTXR	0.914085	0.418375	-0.84139	2.991223
COLGALT1	0.914275	0.418583	-0.36941	6.746684
DNAJC3-DT	0.914275	0.41865	-1.07265	2.884204
SLC30A5	0.914275	0.418851	-0.37296	5.963118
IQSEC2	0.914275	0.418862	0.747476	2.345921
EHBP1L1	0.914275	0.418956	0.342465	9.014182
MIS18A	0.914275	0.419006	-0.58476	3.83305
MASP2	0.914275	0.419085	-0.68542	3.669925
ZNF563	0.914275	0.419092	-1.34208	2.248766
TMEM140	0.914275	0.419217	-0.50245	4.746473
PPA1	0.914275	0.419348	-0.41119	6.516309
SOWAHD	0.914275	0.419351	1.182318	0.962112
SNORD116	0.914275	0.419358	-1.50417	1.568452
MIR570HG	0.914275	0.419368	-0.85671	2.833105
LOC101927	0.914389	0.41949	1.164645	1.224302
C8G	0.915133	0.419909	-1.77761	2.121184
HEXB	0.915133	0.420047	0.551713	3.648081
ARPC5L	0.915133	0.420201	-0.40917	5.839732
ACAD8	0.915133	0.420229	0.43321	4.75982
WDR88	0.915133	0.420312	-1.17665	1.54015
C11orf58	0.915133	0.420325	-0.35132	7.575814
WDR59	0.915133	0.420386	-0.40092	6.13302
VWA2	0.915133	0.420389	0.520628	6.123437
SH3GL1P1	0.915394	0.420579	-0.80382	3.18216
SNORA72	0.915465	0.420816	-0.55015	5.554888

ZNF653	0.915465	0.420842	-0.61423	3.885239
C1QTNF1	0.915465	0.420845	-1.30641	1.247121
SLFN12L	0.915465	0.420914	0.7815	3.472205
PFN2	0.915465	0.421189	-1.67332	0.533707
LINC01124	0.915465	0.421216	1.302194	1.093013
EMX2OS	0.915465	0.421229	-1.26131	2.995586
SLC35C1	0.915465	0.421236	0.581028	3.728102
INAFM1	0.915465	0.421288	-0.97175	2.64743
LOC10004	0.915465	0.421316	0.725778	3.718535
ZNF503	0.915465	0.421444	-0.59379	4.521374
KCNQ1-AS	0.915465	0.421486	1.216034	1.473095
AP3M2	0.915465	0.421598	1.279779	1.201899
SULT1C2	0.915465	0.42182	0.689163	3.860636
KDELR1	0.915465	0.421858	0.351713	7.624078
DPH7	0.915465	0.421878	-0.51339	4.180103
VAT1	0.915465	0.421926	-0.393	6.356023
CHST6	0.915465	0.422103	0.601324	3.523575
PPTC7	0.915465	0.422137	-0.42441	5.866656
CDYL	0.915465	0.422145	0.845501	2.050846
NELFB	0.915465	0.422148	0.368038	5.896445
EP400P1	0.915465	0.422184	0.533579	3.705271
PTCD3	0.915465	0.422216	-0.37347	7.33198
PTOV1-AS1	0.915488	0.422306	-0.5139	4.373268
AHDC1	0.915488	0.422367	0.394924	6.057692
CCDC15	0.91557	0.422502	-0.61909	3.899849
HEIH	0.91557	0.422675	0.617268	3.493329
CNPY3	0.91557	0.422706	0.359358	5.999502
NLRX1	0.91557	0.422827	0.924194	1.72023
ZNF732	0.91557	0.42295	1.190254	1.658198
DDX41	0.91557	0.422973	0.349043	5.90141
CCDC155	0.91557	0.423058	1.264947	1.309844
UBN2	0.91557	0.423132	-0.38538	7.834519
KLHL31	0.91557	0.423147	-0.8107	2.655952
LOC10013	0.91557	0.423199	-0.71755	3.45409
PPP2R3B	0.91557	0.423417	0.830053	2.089639
STK35	0.91557	0.42347	-0.37578	6.874503
BEND3	0.91557	0.423478	0.462669	4.586381
RIMKLB	0.91557	0.423481	-0.82895	3.60804
UBE2Q2P2	0.91557	0.423585	0.495339	5.063489
STON1	0.91557	0.423728	-2.21674	0.579787
IP6K1	0.91557	0.423743	-0.96541	2.54259
EVL	0.91557	0.423769	-0.49183	5.737325
RNF139	0.91557	0.423783	-0.42019	5.266395
KIAA1671	0.91557	0.4238	0.35654	8.179458
SPAG8	0.915855	0.424065	1.418788	0.697017

TEX22	0.915855	0.424072	1.001748	1.750776
ALPK2	0.915952	0.42426	-0.9524	3.331888
MRPL46	0.915952	0.424332	-0.72974	3.405265
ACOXL	0.915952	0.424338	0.963186	1.965318
ZIC5	0.915952	0.424396	0.87351	3.241683
MRPL30	0.916093	0.424531	0.486216	4.400639
ERN1	0.916258	0.424677	0.353556	6.192289
ZBTB4	0.91646	0.424841	0.810546	2.646669
WASHC1	0.916504	0.424943	-0.67207	3.14887
C11orf54	0.916504	0.425001	1.16343	1.691466
DLG3	0.916934	0.425349	0.762954	2.746047
LSM7	0.916934	0.425423	-0.49877	4.256043
KDELR2	0.916934	0.42545	-0.73381	3.01244
ZNF860	0.916934	0.425531	-0.64695	4.027705
TMEM62	0.916934	0.425642	0.405817	5.395518
AIMP2	0.916934	0.42569	-0.76841	3.303608
PRH1	0.916934	0.425692	-1.43666	0.878666
GCC1	0.916934	0.42576	-0.41654	5.722419
ANKRD20A	0.916934	0.425877	1.227399	2.016968
SHROOM3	0.916934	0.425899	0.37842	8.919768
CAVIN1	0.917312	0.426145	-0.44225	8.35076
UCHL1	0.91789	0.426483	-1.21655	1.612228
ASB7	0.917958	0.426667	-0.49314	4.436796
FRY	0.917958	0.426741	-0.46011	6.802337
SRRD	0.917958	0.426753	-0.58609	3.789272
LINC01311	0.917958	0.426917	0.854099	2.019599
RTKN2	0.917958	0.426984	-0.85114	4.31229
CREB3	0.917958	0.42706	-0.4495	5.392798
ZCWPW2	0.917958	0.427064	-1.24364	2.16431
POM121L8	0.917958	0.427082	1.019735	1.704561
SLC6A6	0.917958	0.42724	1.007777	1.756164
ZNF385B	0.917958	0.427261	1.579332	0.747098
GIMAP2	0.917958	0.427368	0.656107	3.859878
SNORD13	0.917958	0.427562	-0.54236	7.496078
APOBEC4	0.917958	0.427571	0.665709	2.923565
CNIH1	0.917958	0.427581	-0.46623	5.263947
YPEL2	0.917958	0.427616	0.342468	6.235337
COL5A2	0.917958	0.427764	0.346377	9.121431
CDKN1B	0.917958	0.427834	0.341038	6.616337
SNORA38B	0.917958	0.427858	0.661445	5.673554
MPHOSPH1	0.917958	0.427919	-0.36265	6.793999
RNF167	0.917958	0.427949	-0.4064	5.611691
KDM7A	0.917958	0.427984	0.366184	7.837997
LOC101928	0.918126	0.4282	-1.72964	1.587727
JCAD	0.918126	0.428267	-0.40321	6.602915

ZBTB47	0.918126	0.428333	-0.38319	6.070152
FREM1	0.918126	0.42839	-0.77536	3.254432
MIRLET7A1	0.918126	0.428476	-1.01922	2.755677
AKAP8L	0.918126	0.428634	0.99627	1.444835
MIR6870	0.918126	0.428736	1.507389	0.73698
SRGAP1	0.918126	0.428752	0.339876	7.018748
CCDC85B	0.918126	0.428765	-0.47207	4.713383
PPP1R18	0.918126	0.428818	-0.8382	3.3899
LINC00265	0.918126	0.428832	-0.54052	4.389071
MTRF1	0.918157	0.428965	-0.50317	4.846242
PSMA7	0.918157	0.429047	-0.42173	6.525299
POLR3K	0.918157	0.429081	-0.53475	3.827063
IARS2	0.918157	0.429126	-0.41178	7.169901
LINC00705	0.918369	0.429305	1.345719	0.738733
INKA1	0.918369	0.429395	0.996024	1.633567
MYL12B	0.918369	0.429457	-1.59977	1.2024
TRIM21	0.918369	0.429505	-0.43433	5.378373
KRT6C	0.919294	0.430052	1.445336	6.694022
COQ8B	0.919294	0.430085	-1.35889	1.013767
LOC100128	0.919294	0.430148	0.770832	2.898542
UGT1A1	0.919631	0.430396	0.844734	2.261864
PLEKHO1	0.919631	0.430543	0.714186	3.088317
LOC101927	0.919631	0.430641	1.092394	1.777631
AGAP6	0.919631	0.430652	-0.53476	4.774908
COX7C	0.919631	0.430686	0.345663	7.322344
CLEC12B	0.919631	0.430743	1.539781	0.824673
LOC100507	0.919631	0.430997	0.929812	2.222809
LOC100130	0.919631	0.431163	0.555205	3.991662
LOC101927	0.919631	0.431194	0.531884	4.038844
MRGPRF-A	0.919631	0.431285	0.949124	0.946974
DIP2B	0.919631	0.431522	0.366581	7.360209
TMEM119	0.919631	0.43153	-0.60734	4.251914
MYH15	0.919631	0.431595	-0.88242	2.772907
LIG4	0.919631	0.431636	-1.36023	1.044685
UNKL	0.919631	0.431655	-0.69118	3.442222
SNORD114	0.919631	0.431744	-1.16133	2.368441
MAU2	0.919631	0.431842	-0.38095	6.608496
RNF114	0.919631	0.431868	-0.36456	6.284577
HOXA13	0.919631	0.431886	0.412064	5.350343
CYP4F35P	0.919631	0.431892	-1.64108	1.426924
ASPHD2	0.919631	0.431905	0.47361	4.90069
MRS2	0.919631	0.431934	-1.25721	1.719454
TTC7B	0.919631	0.431972	-0.53627	4.63352
ERBB3	0.919631	0.431996	0.342936	8.819315
NIN	0.919631	0.432059	-0.48117	4.679218

RABAC1	0.919818	0.432218	0.413585	5.131631
ACSL5	0.919818	0.432337	0.740434	3.23682
SUN1	0.919818	0.432366	0.793207	2.643268
GLIS2	0.919818	0.432427	-0.74033	3.452918
COX19	0.9199	0.432535	-0.40452	5.86292
CKMT2-AS1	0.919922	0.432677	1.034555	1.595246
RBM45	0.919922	0.432686	0.567072	3.978479
HIST1H2AJ	0.919973	0.432891	-0.56492	5.304966
KDM2B	0.919973	0.432919	0.934076	1.837644
LOC654841	0.919973	0.433006	-1.55648	1.312442
SNORA44	0.919973	0.43304	-0.47968	7.096938
TARID	0.919973	0.43306	0.922795	3.203057
CAHM	0.920057	0.43317	1.147573	1.181008
AMER1	0.920234	0.433324	-0.45554	5.013407
COL18A1	0.920419	0.433544	0.734932	2.445309
TADA2B	0.920419	0.433577	0.354519	5.580928
LINC02298	0.920419	0.433621	1.073418	0.949043
COG1	0.920443	0.433753	-0.38444	5.586404
ADCY2	0.920443	0.433773	-1.02976	3.462606
RABIF	0.920647	0.433939	0.672376	3.823688
ZNF33A	0.920795	0.434079	-0.76408	3.058455
HEXD	0.920812	0.434157	-0.48837	4.432804
UQCRC2	0.921603	0.4346	0.345527	7.017775
ADAM11	0.921613	0.434705	-1.21519	2.195834
MAB21L1	0.921613	0.434746	0.954403	1.795212
ZRSR2	0.921803	0.43491	-0.37791	6.161049
CELA2B	0.921803	0.434976	1.088917	1.539943
LOC105378	0.92198	0.435129	1.045661	2.121281
FAM153C	0.922592	0.435489	-1.98407	0.991109
FAM114A1	0.9233	0.435893	0.547016	3.536396
GK3P	0.923988	0.436431	-0.8836	2.777231
FDPS	0.923988	0.436481	1.019198	0.84282
FBXO34	0.923988	0.436648	0.561459	3.334883
LINC02595	0.923988	0.436763	-0.93391	2.472581
LOC100996	0.923988	0.436794	-1.38861	1.901789
LOC101929	0.923988	0.436997	1.110256	0.667665
ARFGEF3	0.923988	0.437064	0.363324	7.940093
SCARNA7	0.923988	0.437176	-0.38362	8.686602
LRRC25	0.923988	0.437227	-1.10568	2.057027
ZNF528-AS	0.923988	0.437254	0.80597	2.570699
ENAM	0.923988	0.437264	1.0499	2.409505
FBXO6	0.923988	0.437295	0.624431	3.329434
LAMB4	0.923988	0.437311	1.497838	1.358883
USE1	0.923988	0.437332	0.477412	3.818003
GSDMC	0.923988	0.437563	1.154726	2.999615

RAB14	0.923988	0.437565	-0.38616	7.310007
OGFR-AS1	0.923988	0.437674	1.138318	0.920906
ZNF407	0.923988	0.437718	-0.54151	4.007608
ADGRL1	0.923988	0.437784	1.048775	1.760323
ZNF513	0.923988	0.437831	0.951011	1.761724
LOC10012€	0.923988	0.437873	0.908783	2.469366
TMED8	0.923988	0.437916	0.49517	3.98899
LOC10192€	0.923988	0.437958	1.346218	1.139777
SLC39A3	0.923988	0.438013	-0.67349	3.456004
UST	0.923988	0.438016	-0.54009	4.696446
RPS12	0.923988	0.43805	-0.42585	8.472169
SMARCE1	0.923988	0.438121	-0.34884	7.285401
RTN4RL2	0.923988	0.43819	0.668128	2.541462
LMNTD2	0.924228	0.438375	0.588942	3.386375
ARHGAP21	0.924301	0.438616	-0.36242	8.34614
UBA2	0.924301	0.438726	-0.35348	7.271099
ZBTB11	0.924301	0.438827	-0.37792	5.873938
MIR3682	0.924301	0.438924	-1.79482	0.751506
FKBP3	0.924301	0.439028	0.356213	5.54521
LOC10013(	0.924301	0.439057	-1.75266	1.387678
NPIPB15	0.924301	0.439156	-1.14765	3.191139
RPA3	0.924301	0.439189	-0.74291	4.526615
TOB2	0.924301	0.439278	0.317562	7.458153
LINC00641	0.924301	0.439333	-0.38651	5.404941
TCN1	0.924301	0.439387	-1.23136	3.365485
SLC35E2A	0.924301	0.439394	0.434597	5.223873
HEY2	0.924301	0.439472	-1.04768	2.239551
DHX9	0.924301	0.439489	-0.92354	2.317085
RHBDL3	0.924301	0.439494	1.001292	1.62901
BCRP3	0.924301	0.439578	-1.26647	1.668303
VPS16	0.924301	0.439643	-0.88476	2.297447
GCNA	0.924301	0.439706	-0.74663	3.64024
LOC44124:	0.924301	0.439747	0.583882	3.443028
FYN	0.924464	0.439895	-0.48426	5.132695
QRSL1	0.924858	0.440153	0.410639	5.212928
PLEC	0.925247	0.440491	-0.47022	5.660782
DCLK3	0.925247	0.440516	-1.54832	0.934316
SNORD93	0.925247	0.440559	-1.09233	2.884047
SNORA2A	0.925247	0.440744	-0.96613	2.243201
C6orf62	0.925247	0.44082	-0.36429	7.098575
GABPB2	0.925247	0.440823	-0.46431	5.370067
LENG8-AS1	0.925247	0.440838	-0.5	4.705075
PAK2	0.925247	0.440903	-0.35395	8.013323
UBAC1	0.925271	0.440985	-0.44135	5.249469
PDRG1	0.925376	0.441149	-0.54931	3.785725

C11orf42	0.925376	0.441176	0.889456	1.903861
SPTBN2	0.925414	0.441271	-0.67123	6.610968
PIPSL	0.925414	0.441425	0.862233	2.663906
KIF5A	0.925414	0.441484	-1.30151	2.133256
PSG10P	0.925414	0.441557	1.411236	1.29555
BATF	0.925414	0.441579	0.649391	2.897573
GALNT5	0.925414	0.441772	0.411479	7.026567
POT1	0.925414	0.441774	1.393111	0.792363
BTBD11	0.925414	0.441855	0.980084	2.543965
ZNF100	0.925414	0.442027	1.138624	1.283791
DISC1-IT1	0.925414	0.442143	-1.54949	1.315164
CUX1	0.925414	0.442179	0.812338	1.937765
SSTR2	0.925414	0.442216	1.045928	1.671664
SOX5	0.925414	0.442273	1.128474	1.252058
SNORA23	0.925414	0.44232	-0.39579	8.385488
FAM19A2	0.925414	0.442415	0.777918	2.379895
RAF1	0.925414	0.442418	-0.33898	7.011456
NXPE1	0.925414	0.442459	0.994293	1.778573
RALB	0.925414	0.442464	0.375587	7.186543
SOX15	0.925712	0.442677	1.08815	1.989022
SMAD1	0.925872	0.442837	0.998772	1.076956
CXCL14	0.925872	0.443021	0.613115	6.345058
CCDC6	0.925872	0.443105	-0.36999	7.671154
CDC37L1	0.925872	0.443155	-0.42216	5.670538
SLC25A40	0.925872	0.443242	-0.49718	4.456537
CTSE	0.925872	0.443344	1.006846	1.77135
INSYN2	0.925872	0.443353	0.445988	4.804363
LINC00938	0.925872	0.443376	0.396116	4.550042
TMEM38B	0.925872	0.443514	0.666182	3.975973
PSMC5	0.925872	0.443541	1.115134	1.570574
CASKIN2	0.925872	0.443601	0.682403	2.758318
BLOC1S4	0.925872	0.443696	0.592802	2.842851
C17orf100	0.925872	0.443719	-1.06559	2.00359
ALMS1-IT1	0.925872	0.443755	-0.65073	2.993103
ITGA5	0.925872	0.443942	-0.46613	7.638328
PCDHB12	0.925872	0.44403	0.739193	2.766952
RBM15	0.925872	0.444094	0.671536	3.269924
DCAF16	0.925872	0.444099	-0.37721	6.538918
PTPN7	0.925872	0.444383	1.16505	1.09792
ZBED6CL	0.925872	0.444409	0.637627	3.739155
GORAB-AS1	0.925872	0.444437	-0.86502	2.400621
DAZAP2	0.925872	0.444527	0.828241	2.38287
C1QTNF8	0.925872	0.444529	1.0742	2.242456
JMY	0.925872	0.444584	-0.38073	6.732222
PJA2	0.925872	0.444712	-0.39693	8.020196

HTT	0.925872	0.44485	0.310567	7.842897
MTMR9LP	0.925872	0.44488	0.627044	3.976069
LINC02245	0.925872	0.444902	-1.73043	1.099741
SNORA14A	0.925872	0.444939	-0.5384	3.911107
SERTAD3	0.925872	0.44502	-1.21685	1.549536
TFAP2C	0.925872	0.445036	0.708042	3.595185
ZNF750	0.925872	0.445286	0.548117	4.734763
TRMT44	0.925872	0.445327	0.418619	5.035722
MRPL20-AS	0.925872	0.445365	-0.64288	4.224313
TAF1C	0.925872	0.445374	1.071358	1.912803
AFF1-AS1	0.925872	0.445455	0.544656	3.218471
MRS2P2	0.925872	0.445485	-0.98571	2.865166
LOC100129	0.925872	0.445488	-1.38689	2.116614
POFUT2	0.925872	0.445643	-0.67612	2.969623
PIM3	0.925872	0.44565	-0.46648	6.769236
C12orf29	0.925872	0.445715	-0.82272	3.138172
USP24	0.925872	0.445717	-0.34784	8.40462
CENPE	0.926421	0.446074	1.496258	0.789165
TMED1	0.926421	0.446149	-1.0699	1.234067
BAZ2A	0.926421	0.446238	1.028406	1.43568
SATB2	0.926421	0.446264	0.933204	2.018936
CNOT8	0.926429	0.446346	0.984524	1.835389
GLIPR1	0.926429	0.446409	-0.49352	5.099003
TAF2	0.926439	0.446514	-0.34541	6.741175
AKR7A2P1	0.926439	0.446555	0.784679	2.011487
ARHGEF3	0.927043	0.446947	-0.6788	2.893351
AP5M1	0.927043	0.446987	0.661938	3.620739
PHKA2-AS1	0.927071	0.447071	-1.42594	1.326403
OTUD6B	0.927155	0.447241	-1.88221	0.639071
TTC17	0.927155	0.447253	-0.47192	5.966966
SETDB2	0.927387	0.447436	0.637984	2.880868
PPP2R5A	0.92769	0.447678	0.425038	4.551607
HIST2H2BC	0.92769	0.447723	-0.65776	2.775252
BICD2	0.927711	0.447805	0.615061	4.052696
WNT3A	0.927744	0.447926	-1.50192	1.206824
TSPYL2	0.927744	0.448013	-0.43778	7.109424
NOL12	0.927744	0.448043	0.424094	4.897632
ZBED3	0.927744	0.448103	0.579861	2.904165
PCSK7	0.927787	0.448217	-0.3634	7.717096
PLCG2	0.927787	0.448404	0.388525	6.041367
SNORA99	0.927787	0.448426	1.208847	0.662159
LINC02067	0.927787	0.448551	-0.3834	8.70064
PDCL3	0.927787	0.448554	-0.42849	5.044745
ATP5F1E	0.927787	0.448586	1.013604	1.365284
CIAO1	0.927787	0.448619	-0.344	6.425169

NDUFV3	0.927832	0.448712	0.519021	4.751843
SSC5D	0.927996	0.448992	0.476191	5.173043
COQ8A	0.927996	0.449027	-0.38467	6.310677
ATP9A	0.927996	0.449114	-0.35264	8.094442
SYNPR-AS1	0.927996	0.449132	1.156016	1.696434
LOC400867	0.927996	0.449211	-1.32752	1.249721
LINC01354	0.927996	0.449309	1.019878	0.944721
MUL1	0.927996	0.44943	-0.53589	4.732497
GNL1	0.927996	0.449439	-1.13422	2.075723
RNU11	0.927996	0.449478	0.409038	9.862208
KIAA0355	0.927996	0.449498	-0.47867	5.126136
MSL3P1	0.928141	0.449639	0.600026	3.403642
STUM	0.928156	0.449717	-0.94599	3.675092
MIR320D1	0.928196	0.449807	1.149961	1.843238
NOC3L	0.92824	0.449938	-0.37674	6.235612
RPL22	0.92824	0.449985	0.345984	8.568186
KCTD13	0.92824	0.450051	1.020355	1.674374
ADGRE5	0.92824	0.450218	-0.86446	2.31504
ZNF629	0.92824	0.450227	-0.37109	6.141931
CCDC144N	0.92824	0.450441	-1.42398	0.916978
HEXIM2	0.92824	0.450509	-1.47581	1.112777
TBC1D25	0.92824	0.45052	0.495953	4.397589
TLL2	0.92824	0.450572	0.660448	3.147438
EDEM3	0.92824	0.450591	0.329051	8.092135
ADIRF	0.92824	0.450607	-0.50812	4.915851
SRGAP3	0.928267	0.450691	1.306978	0.798536
SHANK2-AS	0.928421	0.450836	0.834823	2.083359
SLC16A7	0.928627	0.451007	1.32187	1.106794
SEC13	0.928661	0.451181	0.997217	1.865624
HOXA4	0.928661	0.451219	-0.9509	2.452237
TRAF3IP2	0.928661	0.451278	1.018888	1.590466
UTP18	0.928661	0.451307	-0.39202	5.683264
HMCN2	0.928669	0.451453	-0.57254	6.346851
TAF4	0.928669	0.451624	-0.47499	4.885746
TWSG1	0.928669	0.451639	-0.40647	6.223796
HCP5	0.928669	0.451734	-0.42504	5.668845
ZNF416	0.928669	0.45196	-0.66773	3.098354
SYNPO2	0.928669	0.451963	-0.62859	8.070731
IL5	0.928669	0.452069	1.331373	1.237636
HIST1H2BH	0.928669	0.452104	-0.44672	5.034257
PIGZ	0.928669	0.452123	-0.81289	3.317318
AGAP2	0.928669	0.452149	0.971549	1.613367
CTNNB1	0.928669	0.452171	-0.72403	2.721374
FOXJ2	0.928669	0.45223	-0.33809	6.203431
RTF1	0.928669	0.45223	-0.33005	7.315924

GSN	0.928817	0.452401	0.730837	2.734463
SNORD11B	0.928817	0.452444	-1.0226	2.571295
ZFYVE19	0.929172	0.452688	1.103589	1.233942
FBH1	0.929313	0.45285	-0.54199	4.22428
VASH1	0.929313	0.45294	0.388969	5.846981
ECE1	0.929313	0.452969	0.908774	1.875133
MRPL45P2	0.92942	0.45315	-0.62505	3.793186
NRDC	0.92942	0.453163	-1.1349	1.882388
MTMR2	0.929537	0.453316	-1.53489	0.612852
LOC101593	0.929537	0.453362	1.195271	1.157547
PRAF2	0.92962	0.453473	-0.60468	3.907072
MAGEH1	0.929668	0.453568	-1.05514	2.384212
INTS12	0.929795	0.45384	0.89325	2.239918
ST3GAL6	0.929795	0.45386	-1.97288	1.050771
CACNA1C-A	0.929795	0.4539	-0.97563	2.736412
CARD11	0.929795	0.454071	-0.4769	5.84678
WDR54	0.929795	0.454076	0.577027	3.698293
SHQ1	0.929795	0.454117	-0.45116	4.592295
SH3BGRL	0.929795	0.454125	-0.3953	6.50333
DNAJB7	0.93	0.454296	0.783525	1.652954
CELP	0.93051	0.454616	0.9601	1.42614
GOLPH3L	0.930696	0.454778	0.335186	6.459195
COG8	0.931127	0.455096	0.587789	4.174299
HIST1H4E	0.931127	0.455131	-0.39541	5.734907
TOX	0.931141	0.455209	0.510834	5.395156
TRPC6	0.931278	0.45538	-0.57442	4.383015
LINC01000	0.931278	0.455702	0.318009	8.461613
PRKRIP1	0.931278	0.455707	-1.7081	0.936549
FTSJ1	0.931278	0.455717	1.150759	1.058516
PGA3	0.931278	0.455838	-1.99748	2.441594
ROR1	0.931278	0.455925	0.606119	4.832107
TMF1	0.931278	0.45606	-0.33967	8.048274
ATP6AP2	0.931278	0.456078	-0.35088	6.464682
HIST1H1B	0.931278	0.456152	-0.52916	5.810279
NINL	0.931278	0.456188	-0.549	6.63223
KIAA1143	0.931278	0.456265	-0.41734	5.550576
IL15	0.931278	0.456476	1.007153	1.2709
APC	0.931278	0.456558	0.788096	2.136877
PHYH	0.931278	0.456564	1.103985	0.89103
KCNN4	0.931278	0.45679	-0.43017	6.034293
DMKN	0.931278	0.456852	-1.50076	0.967044
COL7A1	0.931278	0.456896	0.436661	8.178527
LOC100335	0.931278	0.457003	0.512481	3.812797
GNA12	0.931278	0.457038	-0.84756	2.508691
SYDE2	0.931278	0.457079	-0.6382	4.858599

SNCG	0.931278	0.457252	-0.94088	2.817243
MYC	0.931278	0.457477	-0.46812	6.626447
MYO5B	0.931278	0.457542	0.385951	8.172212
PIGL	0.931278	0.457566	0.492821	4.244692
JOSD1	0.931278	0.457618	-0.34577	6.79022
MDGA1	0.931278	0.45763	-0.59632	4.125796
PSMD8	0.931278	0.457638	0.3495	6.653476
SNORA40	0.931278	0.457671	-0.40774	6.296983
GACAT2	0.931278	0.457728	0.956784	1.492463
TM7SF3	0.931278	0.457764	-0.37935	7.103367
STK40	0.931278	0.457777	0.978064	1.738578
TRIM68	0.931278	0.457952	-1.26313	0.830619
KLHL35	0.931278	0.45799	-1.30492	1.872571
EFNA5	0.931278	0.458012	-0.78523	4.821927
SNORD70	0.931278	0.458029	-0.74442	2.771359
UCHL3	0.931278	0.458112	0.921233	1.506677
DEGS1	0.931278	0.458163	-0.4103	5.429373
CEP192	0.931278	0.458169	-0.39813	7.720894
CXorf40B	0.931278	0.45817	0.477599	4.090128
ABL1	0.931278	0.458184	0.515705	3.442591
PHB2	0.931278	0.45819	0.788093	1.910167
ABAT	0.931278	0.458257	-1.76863	0.853616
PXMP2	0.931592	0.458482	-0.64595	3.722487
MYLK4	0.931632	0.458573	0.857637	2.101418
LRRC49	0.932015	0.458938	1.415965	1.172892
EGFEM1P	0.932015	0.458993	0.974535	1.300458
PHLDB2	0.932015	0.459172	0.979068	0.867935
PRAM1	0.932015	0.459328	-0.94496	1.985346
FRA10AC1	0.932015	0.459474	-0.33482	6.090953
HIST1H4I	0.932015	0.459502	-0.80258	3.467227
MIR3165	0.932015	0.459616	1.514649	0.790418
MTMR9	0.932015	0.459842	-0.38356	5.546232
PTH2R	0.932015	0.459884	-1.95922	0.72706
EMILIN3	0.932015	0.459944	0.851963	2.469726
RNVU1-15	0.932015	0.45995	-1.00531	1.762615
TNXB	0.932015	0.45996	-0.74031	4.403705
GTF2A2	0.932015	0.459981	-0.49994	4.649881
SEC11A	0.932015	0.459989	0.905489	1.979054
PLXNA3	0.932015	0.45999	0.335221	6.653312
TMEM109	0.932015	0.46002	-0.38193	5.761294
KSR1	0.932015	0.460021	0.341428	5.701845
IRAIN	0.932015	0.46004	0.678277	3.041503
CCAR2	0.932024	0.460166	-1.36641	1.129809
TLR5	0.932024	0.460225	-0.87837	3.747154
HNRNPF	0.932024	0.460258	0.554894	3.330365

PDZRN4	0.932073	0.460353	-1.58088	1.627442
TIGD2	0.932562	0.460727	0.555338	3.348597
OMD	0.932562	0.460737	-0.78173	3.68547
RPS25	0.932791	0.461072	-0.342	7.886316
OR51I1	0.932791	0.461099	-2.36484	0.961251
CCNYL1	0.932791	0.461109	0.786872	1.798946
MINAR1	0.932791	0.461134	0.7212	2.672695
EPCAM-DT	0.932804	0.461257	0.88888	2.14419
GPR21	0.932804	0.461283	-0.79503	3.504469
CLIP1-AS1	0.933005	0.461496	-0.86094	2.41518
DMRTA2	0.933005	0.461525	0.876169	2.227252
GAD1	0.933042	0.461626	0.788727	2.69472
NUP188	0.933042	0.461829	-0.34516	6.816113
LOC102724	0.933042	0.46195	-1.5185	1.033811
TRMO	0.933042	0.461974	0.441873	4.304048
DTWD1	0.933042	0.462022	-1.01317	2.036249
ANKRD36	0.933042	0.462063	-0.36677	8.023257
ARMC2-AS	0.933042	0.462069	-1.03352	1.531299
ID2	0.933042	0.462112	-0.40333	5.368294
AFMID	0.9331	0.462256	0.644473	2.248536
BEND3P3	0.9331	0.462289	-0.43853	5.426946
DTX3L	0.9331	0.46246	-0.34616	7.61777
CYP2E1	0.9331	0.462464	-0.89477	3.184089
DOK6	0.9331	0.462538	-0.78704	3.866858
LOC340512	0.9331	0.462567	1.004185	1.849195
MIR497HG	0.933118	0.462775	-1.1626	1.09611
FNTA	0.933118	0.4628	-1.28303	1.744209
SPRED2	0.933118	0.462833	-0.76778	3.112758
LOC101926	0.933118	0.46288	1.264629	0.774896
AGAP12P	0.933118	0.462985	0.358323	4.894236
OR7E12P	0.933118	0.463028	0.723527	2.642531
KIAA0753	0.933118	0.463184	-0.365	6.024766
TRAPPC4	0.933118	0.463287	0.434026	4.005179
AP3S1	0.933118	0.463319	0.372551	5.870616
ASCL5	0.933118	0.463354	-1.43172	0.774322
EBF2	0.933118	0.463487	0.793436	2.805868
YBX1	0.933118	0.463509	-0.40334	5.781864
PRDM1	0.933118	0.463596	0.450105	5.408093
COL18A1-A	0.933118	0.463667	0.777049	2.177992
USP1	0.933118	0.463684	0.801303	1.563638
TMA7	0.933118	0.463973	0.325906	6.954103
TSSK4	0.933118	0.464006	0.983764	1.406437
ARHGEF37	0.933118	0.464102	0.394906	5.38115
ADCK5	0.933118	0.46412	0.492126	4.43021
C10orf99	0.933118	0.464138	1.393319	1.502199

RPL7A	0.933118	0.464199	0.343032	8.680917
INTS13	0.933118	0.464247	-0.38797	5.700939
C16orf72	0.933118	0.464377	-0.31623	6.953298
CD4	0.933118	0.464498	0.955852	2.241952
LOC643711	0.933118	0.464507	-2.46638	1.022347
MIR3941	0.933118	0.464512	-1.49474	0.909528
NAALADL2	0.933118	0.464514	0.418877	5.196262
SERINC4	0.933118	0.464567	0.820744	1.928718
XRCC1	0.933515	0.46519	-0.41569	5.10297
INSIG2	0.933515	0.465194	-0.39974	5.594756
KIAA1107	0.933515	0.465387	0.418819	4.313849
ZNF646	0.933515	0.465441	-0.38273	6.066133
DGKB	0.933515	0.465461	-1.74133	1.96275
HSP90B2P	0.933515	0.465555	0.508985	3.555245
HSD17B7P2	0.933515	0.465604	-0.75052	3.191046
SPR	0.933515	0.465726	0.457701	4.718961
CLEC16A	0.933515	0.465739	-0.39722	5.595073
USP50	0.933515	0.465838	-1.31954	2.15204
PGLYRP3	0.933515	0.465955	1.072998	1.307653
LOC641367	0.933515	0.465955	0.733794	2.731387
HTRA1	0.933515	0.466001	-0.46102	5.886308
BLVRB	0.933515	0.466012	0.470349	4.551613
TCF7	0.933515	0.466023	0.882623	1.668874
CTCF	0.933515	0.466026	0.353779	5.261388
TCF4	0.933515	0.466594	-0.73644	3.207662
EBAG9	0.933515	0.466595	-1.09026	1.625891
CENPI	0.933515	0.466597	-0.69257	3.439628
SH3YL1	0.933515	0.466685	-0.41795	5.138123
MRNIP	0.933515	0.4669	-1.04015	1.620349
ZNF253	0.933515	0.466924	-0.46021	5.027384
DCLRE1A	0.933515	0.466933	-1.31546	1.369518
CHMP4B	0.933515	0.467035	-0.33723	7.859999
ATOX1	0.933515	0.467079	0.587705	3.158363
FAM102B	0.933515	0.467092	0.319278	6.467283
ZNF776	0.933515	0.467171	-0.35014	6.104794
MYDGF	0.933515	0.467222	0.356731	5.660696
PITPNA-AS	0.933515	0.467336	-0.96754	1.875869
FOXH1	0.933515	0.4674	0.934648	1.306921
RBBP7	0.933515	0.467425	0.387661	4.8886
YY1	0.933515	0.467477	0.313259	7.003473
LOC440896	0.933515	0.467504	0.968839	1.489061
NLK	0.933515	0.467529	-0.36387	5.512335
HSPA7	0.933515	0.467564	0.453025	4.797592
FLT1	0.933515	0.467567	-0.42057	5.306224
PNKP	0.933515	0.467612	-0.37023	5.524993

LINC01116	0.933515	0.467776	-1.5973	0.930851
CNN3	0.933515	0.467814	-1.29844	2.184147
RNU5A-1	0.933515	0.467906	-0.44068	9.619114
PICART1	0.933515	0.468119	-1.43732	0.985634
LRMDA	0.933515	0.468149	0.72347	2.704921
CLEC18B	0.933515	0.468293	1.09842	1.632389
TARBP1	0.933515	0.468398	-0.4344	6.766729
FLJ37201	0.933515	0.468432	0.835844	2.198738
RNY3	0.933515	0.468499	-0.42992	9.357023
TMCO3	0.933515	0.468505	0.321427	7.388928
RNASEL	0.933515	0.468526	0.417697	5.354705
ARHGAP27	0.933515	0.468549	-1.42256	0.931439
ANKRD55	0.933515	0.468567	1.034751	1.370429
CGN	0.933515	0.468627	-0.42799	8.752899
CCDC32	0.933515	0.468732	0.972826	1.77024
CSNK1G2	0.933515	0.468746	-0.33408	6.285525
DGUOK	0.933515	0.468748	-1.14032	2.212895
DRG1	0.933515	0.46883	-0.42187	5.290314
NXT1	0.933515	0.468858	-0.79006	2.767955
MMP2	0.933515	0.468875	-0.58432	5.116019
LINC00589	0.933515	0.468892	-1.63722	1.021
CTLA4	0.933715	0.469088	1.083204	1.256821
NPAT	0.933715	0.469135	-0.3715	6.035683
TMEM160	0.933733	0.469357	-0.69631	2.900072
STXBP6	0.933733	0.469498	1.036215	1.521014
MRGPRX3	0.933733	0.469503	-1.54676	0.838963
TBX18	0.933733	0.469575	-1.05644	3.437336
SH3PXD2A	0.933733	0.469654	0.313852	8.784618
SLC39A8	0.933733	0.469682	0.878899	1.992435
PARP1	0.933733	0.46971	-0.31397	7.822779
SNORD140	0.933733	0.469713	-0.9707	1.72471
ZNF32-AS2	0.933777	0.469858	-0.72214	3.030985
AMDHD1	0.933777	0.470003	1.304609	1.097097
AHNAK	0.933777	0.470013	0.296999	12.62693
BTBD2	0.933777	0.47002	-0.34919	6.302905
SNORA62	0.933916	0.470161	0.424439	8.29335
RALY	0.934044	0.470297	0.957296	2.0839
PATE4	0.934236	0.470523	-1.5362	0.700017
FSTL4	0.934236	0.470536	1.261581	1.347952
CTNNA3	0.934246	0.470775	-1.46945	1.18627
RFXANK	0.934246	0.470881	1.05496	0.870529
DTHD1	0.934246	0.470893	0.750409	3.104121
LRRN1	0.934246	0.470927	0.666989	2.870972
CYP2G1P	0.934246	0.47102	-1.59711	1.199591
PSAPL1	0.934246	0.471128	-0.92213	3.094388

STAB2	0.934246	0.471185	-0.80803	2.677678
LINC02197	0.934246	0.471244	-1.8389	1.369819
TMEM225	0.934246	0.471276	1.354685	1.101999
ZNF578	0.934246	0.471313	-1.04032	2.125131
GDPD4	0.934246	0.471324	1.166401	0.884801
MIR27A	0.934297	0.471586	-1.2645	1.434099
NR1D1	0.934297	0.471612	-0.56325	4.340046
DENND6A	0.934297	0.471633	-0.31547	6.076276
CHML	0.934297	0.471662	-0.35495	5.86687
CPNE3	0.934297	0.471873	-0.34908	6.997887
LINC01061	0.934297	0.47197	0.460675	3.375186
TPRN	0.934297	0.471972	0.417175	4.863627
TBX4	0.934297	0.472064	-1.6938	1.283376
WFDC2	0.934297	0.472081	0.82446	3.200009
ZNF436-AS	0.934297	0.472156	0.592969	3.18306
XKRX	0.934297	0.472194	1.018432	1.860628
ZNF786	0.934297	0.472204	-0.51697	3.787337
MPZL1	0.934366	0.472339	-0.92365	2.173368
NEDD8	0.934366	0.472382	0.485597	3.660925
ADGRA3	0.934373	0.472577	-0.3707	6.016168
PIP5K1P1	0.934373	0.472595	1.112378	1.653355
HLA-DQB1	0.934373	0.472695	1.19635	1.187402
SWI5	0.934373	0.472715	0.55467	3.199612
DISP2	0.934373	0.472791	0.531884	3.451941
VAPA	0.934373	0.472911	-1.27685	2.145222
SLC5A5	0.934373	0.472937	-1.42612	4.076158
RABL3	0.934373	0.473007	0.40307	5.356334
NUDT11	0.934373	0.473083	0.971631	1.695986
PIK3AP1	0.934373	0.473098	-0.38039	5.472153
THAP4	0.934453	0.473269	0.416376	4.80154
COG3	0.934453	0.473281	-0.32745	6.74143
MCOLN1	0.934484	0.473367	0.403041	4.137602
FUT8-AS1	0.934568	0.473481	0.968898	1.161725
TAPBPL	0.934585	0.473642	0.390191	4.70826
GTF2H2B	0.934585	0.473749	-1.05204	1.561682
ZNF37A	0.934585	0.47385	0.855238	1.659559
ZNF720	0.934585	0.473935	-0.65099	4.538765
RAB10	0.934585	0.474024	0.372346	7.984298
THEMIS2	0.934585	0.474113	-1.18743	1.430371
PHF20L1	0.934585	0.474127	-0.99081	2.839025
LRRC59	0.934585	0.47416	-0.35923	7.618265
LOC38976!	0.934585	0.47424	-0.44955	4.50794
SPIN4	0.934585	0.474271	0.443208	4.328472
TTLL5	0.934585	0.474274	0.311923	6.52228
ZNF529-AS	0.934786	0.474571	0.942118	1.428746

HACD1	0.934786	0.474574	-1.03787	1.923775
CCNH	0.934786	0.47461	1.021024	1.066821
ADPGK-AS1	0.934786	0.474662	-1.07815	1.957444
TULP3	0.934786	0.474817	-0.4281	5.48923
ANKRD17	0.934786	0.474887	0.37412	4.900882
MLLT6	0.934786	0.474887	-0.5493	4.184185
ARHGAP6	0.934786	0.474958	-1.04565	2.436885
VWA1	0.934786	0.475079	-1.17801	1.663949
SLC25A5-A	0.934786	0.475088	0.732472	2.333506
SFXN1	0.934861	0.475262	-0.40661	5.399695
SAP30L-AS	0.934861	0.475268	0.395161	4.604331
REEP5	0.934922	0.475378	-0.33937	6.970372
KIF20A	0.934922	0.475509	-0.47385	4.244914
ACAD9	0.934922	0.475662	-0.97917	1.829237
PAK1IP1	0.934922	0.475827	-0.64122	4.706961
THUMPD1	0.934922	0.475879	-1.02128	2.415498
PPCS	0.934922	0.47612	0.728775	2.518271
CCDC22	0.934922	0.476205	0.391444	4.269092
DGKA	0.934922	0.476346	0.803595	2.217608
TJP3	0.934922	0.476509	0.598909	2.752281
LINC00662	0.934922	0.476539	0.889396	2.937146
ST3GAL4	0.934922	0.476579	-1.37705	0.83905
FAM198A	0.934922	0.476625	-0.71487	3.909058
ABCA11P	0.934922	0.476631	0.634028	3.273689
CBARP	0.934922	0.476757	-1.16597	1.767415
LOC101927	0.934922	0.476852	-1.34543	1.095552
JMJD7	0.934922	0.476921	0.5057	3.397483
MED26	0.934922	0.477096	-0.40985	4.864963
B4GALT4-A	0.934922	0.477155	0.98168	1.781481
BACE1-AS	0.934922	0.477183	-1.63078	0.86136
CASC16	0.934922	0.477259	1.343407	0.839876
C5orf66	0.934922	0.477399	0.462442	5.360586
METTL22	0.934922	0.477408	-0.61695	4.138056
ARHGAP23	0.934922	0.477566	-0.78327	3.349929
PRPS1	0.934922	0.477569	-0.95584	2.088686
LOC440173	0.934922	0.477901	-1.9471	0.82591
AHCY	0.934922	0.47795	1.127427	1.0089
MFSD5	0.934922	0.478149	1.221623	1.010408
SMARCAD1	0.934922	0.478289	-0.93169	1.955236
DDX28	0.934922	0.478305	0.542755	2.975927
CD72	0.934922	0.478327	-0.69323	3.41659
PGS1	0.934922	0.478341	-1.31856	1.643288
VAMP8	0.934922	0.478347	0.307114	5.894452
RALGAPA2	0.934922	0.478456	0.29501	8.460519
CRELD1	0.934922	0.478668	0.773822	2.669242

SOGA1	0.934922	0.478732	-0.37221	6.10347
VTRNA1-3	0.934922	0.479105	-0.75535	4.092139
SCAPER	0.934922	0.479134	0.442663	3.807184
MTMR4	0.934922	0.479236	0.321281	6.576445
TSHR	0.934922	0.479325	0.803284	2.819236
UBE2QL1	0.934922	0.479341	-1.07139	1.491923
ADORA2B	0.934922	0.479392	-0.73223	3.018458
PREX2	0.934922	0.479518	0.482674	4.511314
LHX4	0.934922	0.479554	0.753096	1.95137
YAF2	0.934922	0.479657	0.343275	5.204999
PPWD1	0.934922	0.479819	0.843659	1.854203
LOC28317	0.934922	0.479846	-1.12227	3.131663
ZNF530	0.934922	0.479853	-1.24803	1.472736
ZNF790	0.934922	0.479937	-0.7881	2.5596
STK39	0.934922	0.479961	0.292649	7.613919
TRIB2	0.934922	0.480031	-0.48244	4.768395
THNSL1	0.934922	0.480044	-1.07483	2.810125
DNAJC17	0.934922	0.48014	-0.46755	4.189454
FAM86FP	0.934922	0.480287	-0.88952	1.897309
PDXDC1	0.934922	0.480304	-0.82979	2.640216
UBE3C	0.934922	0.480325	-0.35832	7.643189
LINC01578	0.934922	0.480404	0.484726	3.506762
TMEM256	0.934922	0.480721	0.610013	3.583373
BTN2A1	0.934922	0.480766	-1.56919	0.851041
LOC10012	0.934922	0.480846	0.70074	2.132765
STPG3-AS1	0.934922	0.480857	-1.62949	0.749578
NAA38	0.934922	0.480935	-0.49824	3.812308
SNORA3A	0.934922	0.480972	-0.44154	7.628045
ZSCAN21	0.934922	0.480972	-0.93724	3.210668
PRDX6	0.934922	0.481007	-0.32668	7.110248
NIPBL-DT	0.934922	0.481094	-0.44288	4.966097
LOC10192	0.934922	0.481109	1.327589	0.757224
P2RY11	0.934922	0.481131	-1.16119	0.86172
FGD5-AS1	0.934922	0.481263	0.750914	2.138632
KPNA3	0.934922	0.481277	-0.35129	6.254847
PLEKHA6	0.934922	0.481383	0.324705	7.978491
LINC00612	0.934922	0.481387	-0.78341	1.960237
LINC01160	0.934922	0.481528	-1.22166	1.40082
HOXA9	0.934922	0.481769	-2.03974	0.570978
MATN1-AS	0.934922	0.481824	-1.15945	1.339858
MAB21L2	0.934922	0.48188	0.52226	3.692554
NEU3	0.934922	0.481927	0.374414	4.86507
ZC3H11A	0.934922	0.482048	-0.3176	8.689042
SHC2	0.934922	0.482097	-0.49007	4.339054
CENPM	0.934922	0.482129	-1.3712	0.747064

SNORA21	0.934922	0.482228	0.360193	9.121462
TRDMT1	0.934922	0.482263	-0.43667	4.307529
SBF1	0.934922	0.482302	0.289709	7.637976
SCARNA17	0.934922	0.482366	-0.37013	8.061947
ZC3HAV1L	0.934922	0.482381	-0.4966	4.472021
UBE2D2	0.934922	0.482427	0.65673	2.708213
SNHG28	0.934922	0.482488	0.830899	1.856297
LMTK2	0.934922	0.4826	-0.38001	7.770091
ISG20	0.934922	0.482621	1.254286	0.709906
AK1	0.934922	0.482669	0.335328	5.329082
MYO5C	0.934922	0.482685	0.397113	8.134339
ATF2	0.934922	0.482698	-1.3431	0.952415
XRN1	0.934922	0.482783	-0.55665	3.457428
ATL3	0.934922	0.482828	0.985904	1.475483
COX4I2	0.934922	0.482845	0.949703	1.0149
RTL8A	0.934922	0.482926	0.539199	2.835971
ANXA11	0.934922	0.483005	-0.56527	3.972764
UNC5B-AS1	0.934922	0.483057	-1.20794	1.505343
RPS26	0.934922	0.483253	-0.84368	4.5375
SNORD26	0.934922	0.483365	-0.45786	8.516835
KLK10	0.934922	0.483371	-1.30531	1.303073
HINFP	0.934922	0.483423	1.018651	0.88037
C9orf3	0.934922	0.48351	0.753717	3.454888
THY1	0.934922	0.483527	-1.41603	1.194008
SCARNA9L	0.934922	0.483544	-0.46788	4.681431
KIAA2026	0.934922	0.483611	0.299766	7.419517
SP5	0.934922	0.483665	-1.27836	2.233547
SLC39A14	0.934922	0.483739	0.514554	3.667118
SLC7A2	0.934922	0.483851	-1.29943	0.598078
AKAP8	0.934922	0.483909	-0.34762	7.019333
ALCAM	0.934922	0.483931	-0.99936	2.595096
MIR6773	0.934922	0.484058	0.973982	1.369685
AMIGO1	0.934922	0.484059	0.639399	3.325583
LINC01232	0.934922	0.484299	0.694208	2.686229
FOXO3	0.934922	0.484355	0.701072	2.629217
SNHG25	0.934922	0.484403	0.629868	3.283283
RAD50	0.934922	0.484411	0.297615	8.057779
INO80C	0.934922	0.484469	1.061923	1.199407
TAF12	0.934922	0.484511	0.678275	2.472044
NBPF20	0.934922	0.48455	0.55454	3.237514
LINC01590	0.934922	0.48461	-1.24022	0.577533
SLC9A3-AS	0.934922	0.484644	0.433564	4.303958
MYH6	0.934922	0.48469	-1.44988	0.983108
LGALS9B	0.934922	0.484694	0.870028	2.134064
MIR5695	0.934922	0.484877	0.992016	1.231038

CENPH	0.934922	0.485003	-0.64358	3.87273
PXK	0.934922	0.485166	-1.42968	0.861968
SNORA68	0.934922	0.485343	-0.40925	7.995134
GFOD1	0.934922	0.485384	0.933303	1.376502
TIA1	0.934922	0.485388	1.112294	1.682309
CCDC183	0.934922	0.485501	-0.69174	3.241209
CAPN7	0.934922	0.48565	-0.31498	6.695453
FLCN	0.934922	0.48568	-0.38732	5.288892
DLGAP1-AS	0.934922	0.485749	-1.14241	2.079941
FRMPD1	0.934922	0.485773	-1.32795	0.865112
ZNF585B	0.934922	0.485814	0.393271	5.111889
CHCHD2	0.934922	0.485851	-0.34107	6.879654
PITHD1	0.934922	0.48592	0.336205	5.790971
ACTR10	0.934922	0.485972	-0.37524	5.246786
LOC101928	0.934922	0.486135	0.848894	2.061823
COG7	0.934922	0.486239	-0.37178	5.509803
BCR	0.934922	0.48633	0.886356	1.397069
EIF4A3	0.934922	0.486361	-0.39455	6.040247
UBLCP1	0.934922	0.486467	-0.42076	4.856014
SCN4A	0.934922	0.486542	0.940421	1.846817
MAF	0.934922	0.486577	0.395053	5.361865
CLRN3	0.934922	0.486582	0.600776	4.693682
TTC3P1	0.934922	0.486683	-0.55693	3.931481
SGK2	0.934922	0.486806	0.662948	2.504468
PALLD	0.934922	0.486871	-0.43495	4.67264
PIK3C3	0.934922	0.486921	1.127481	1.362823
ANKRD13B	0.934922	0.486974	-0.56854	3.891703
ATP6AP1	0.934922	0.487028	-0.36119	5.85618
MIR601	0.934922	0.487043	1.225975	1.234728
CTC-338M1	0.934922	0.487077	-0.81309	2.298128
KAT2B	0.934922	0.487121	0.367207	5.93925
KIAA1614	0.934922	0.487175	0.528351	3.825713
GRTP1	0.934922	0.487196	-0.81161	2.052498
GUSBP1	0.934922	0.487259	0.381985	4.657483
ANGPTL1	0.934922	0.48729	0.396079	5.346115
ZNF709	0.934922	0.487327	-0.53139	3.640456
LHFPL2	0.934922	0.487347	-0.38135	6.787199
WAPL	0.934922	0.487482	-0.32883	8.056405
ENO4	0.934922	0.48753	-0.76555	2.690036
USP2-AS1	0.934922	0.487538	-1.15914	0.95511
EGLN1	0.934922	0.487557	-0.32906	6.723317
PSKH1	0.934922	0.487653	0.36342	4.951504
ZFP36L2	0.934922	0.487654	-0.33175	8.999171
IPMK	0.934922	0.487706	-0.51943	5.225808
LINC01572	0.934922	0.487761	0.53371	4.032293

ARMCX4	0.934922	0.487819	0.371009	5.14793
FAM199X	0.934922	0.487982	-0.31336	7.319863
LINC01006	0.934922	0.488008	0.587096	3.299803
MMACHC	0.934922	0.488127	-0.59047	3.158182
HACD3	0.934922	0.488307	0.306108	6.654801
LOC145783	0.934922	0.488332	-0.74051	3.207263
ZNF891	0.934922	0.488513	0.54069	3.303414
ILF3-DT	0.934922	0.48852	0.440796	3.957719
CCDC186	0.934922	0.488577	-0.32338	8.039252
TBCB	0.934922	0.488581	0.638189	2.233542
RUVBL2	0.934922	0.488713	-0.34694	5.62932
RNF145	0.934922	0.488859	0.466327	3.340373
LRP5L	0.934922	0.488897	-0.5418	3.554692
TIMM17A	0.934922	0.489003	-0.43903	5.124787
NTMT1	0.934922	0.489229	-1.49701	0.864578
GTF2H1	0.934922	0.489447	1.07204	1.036093
KDM5A	0.934922	0.489482	-0.30563	8.306911
PDZD2	0.934922	0.489602	0.368565	5.635609
ZSCAN5A	0.934922	0.489624	-0.75901	2.128935
TNFRSF10C	0.934922	0.489713	-0.63284	3.441331
VEZF1	0.934922	0.489736	-0.3174	6.929408
LINC01978	0.934922	0.489738	0.602627	2.976938
SNORD35B	0.934922	0.489759	-0.50753	5.006732
POLK	0.934922	0.489765	-0.32998	6.288832
TMPO	0.934922	0.489804	0.377984	5.337576
KRT16P1	0.934922	0.489812	1.316068	1.497581
PLXDC2	0.934922	0.489932	-1.09441	1.716657
RGS17	0.934922	0.489938	0.834947	1.845562
1-Sep	0.934922	0.489942	-0.5118	4.526458
ACAA2	0.934922	0.49004	-0.37215	6.33326
SPRY4-IT1	0.934922	0.490042	0.68592	2.537486
KHDC1	0.934922	0.490063	-0.85738	2.445048
ATXN10	0.934922	0.490084	0.878898	1.597327
SNORD14A	0.934922	0.49028	-0.52074	5.370609
PDGFA	0.934922	0.490382	-1.44527	1.075275
FLNA	0.934922	0.490435	-1.28457	1.15551
SRSF3	0.934922	0.490459	-0.69067	3.337757
COA7	0.934922	0.49046	-0.43885	4.560175
LOC145474	0.934922	0.490493	0.615598	2.694533
LOC101927	0.934922	0.490515	-1.08804	1.72612
RBM14	0.934922	0.490562	-0.40795	4.519927
EIF3G	0.934922	0.490606	-0.29819	6.925407
THOC1	0.934922	0.490683	-0.38033	6.206185
GOLGA2P7	0.934922	0.490692	-0.67824	3.171758
HIST4H4	0.934922	0.490955	0.538324	3.42711

NKAPP1	0.934922	0.490965	0.583439	3.229563
ZEB1-AS1	0.934922	0.49101	-0.75763	3.285484
KMT2E	0.934922	0.491042	-1.29678	1.774286
GLYCTK-AS	0.934922	0.491188	0.719781	2.705097
SLC9A4	0.934922	0.491212	-1.10724	4.208648
CYS1	0.934922	0.491245	-0.87132	2.674362
IDH3B	0.934922	0.491246	-0.90079	2.463231
REM1	0.934922	0.491401	0.953208	1.979751
PRMT6	0.934922	0.491406	0.525443	3.615976
ZNF77	0.934922	0.491452	0.475408	3.276329
VIL1	0.934922	0.491512	0.672116	7.332103
GPCPD1	0.934922	0.491579	-0.33569	6.766099
HSPA5	0.934922	0.491611	-0.40241	9.432528
LOC101926	0.934922	0.491619	-1.38009	0.846334
FAM171A2	0.934922	0.491657	1.057844	0.946282
MLPH	0.934922	0.491702	0.756442	2.270114
KLHL2	0.934922	0.49176	0.919971	0.868278
ABCG2	0.935119	0.491951	0.944122	2.303299
CIRBP	0.935119	0.492066	-1.18761	1.295631
KIF18A	0.935119	0.492141	-0.9198	3.52002
DNMBP-AS	0.935119	0.492149	-1.35097	0.828838
CNR1	0.935301	0.492316	1.12054	1.223493
ATG9B	0.935491	0.492616	1.227397	0.909406
GLOD4	0.935491	0.492624	0.348193	6.09723
SSR2	0.935491	0.492704	-0.33233	6.299827
MZT2A	0.935491	0.49279	-0.68385	3.578505
ATP5MGL	0.935491	0.492854	-1.60097	0.724401
KANK3	0.935491	0.492908	0.619672	2.845541
FAXC	0.935491	0.492915	-1.001	2.706715
MSS51	0.935497	0.493054	-0.66042	3.244841
SLC1A3	0.935497	0.49312	0.863099	1.752165
NPIPB4	0.935497	0.493187	-0.81826	2.801335
RNF113A	0.935497	0.493239	0.36703	4.478533
SLC27A1	0.935497	0.493275	-0.36118	5.694926
KRT6B	0.935509	0.493447	1.185467	6.821794
CACHD1	0.935509	0.493457	-1.1626	1.238473
SNORD88C	0.935509	0.493505	-0.78985	1.906941
LOC101927	0.935509	0.493566	-1.0587	0.672215
RPS3	0.935831	0.493808	-1.08345	1.847322
OSGIN1	0.935884	0.493923	-0.73295	2.575224
FAM120AC	0.935884	0.494009	-0.33695	5.853382
TALDO1	0.935884	0.494078	0.29893	6.734924
FAM76B	0.935884	0.494209	-0.38754	5.750584
MTMR7	0.935884	0.494216	-0.61278	4.13408
HIST1H3D	0.935884	0.494264	-0.59656	4.470872

MT2A	0.936278	0.494594	-0.45544	6.557591
CYP4F12	0.936278	0.494642	-1.51222	1.141193
UBR2	0.936278	0.494719	-0.29255	7.615234
ADAM20P1	0.936278	0.494822	-0.8549	2.865458
HNRNPA0	0.936278	0.494851	0.300278	7.053799
ELOA-AS1	0.936278	0.49511	-1.46651	1.548501
TVP23B	0.936278	0.495123	0.660963	2.516476
RNF112	0.936278	0.495384	0.776065	2.429226
PASK	0.936278	0.495414	-0.61834	3.436036
TSPAN33	0.936278	0.495522	0.576735	3.857241
IRAK3	0.936278	0.495581	-0.99454	2.069297
AP1G1	0.936278	0.495644	0.628349	2.195308
MUC13	0.936278	0.495657	-0.67553	9.373766
LOC100507	0.936278	0.495707	0.441515	4.238345
LINC01144	0.936278	0.49572	-0.97163	1.362208
ANKLE2	0.936278	0.495786	-0.31666	7.433566
LINC01312	0.936278	0.49582	1.103757	1.341825
CCDC170	0.936278	0.49586	-0.65214	3.172371
CBX7	0.936278	0.495876	-0.38488	6.19478
RAB30	0.936278	0.495899	0.918827	1.279928
SNX20	0.936869	0.496323	-1.1236	2.062686
PLEKHG7	0.936869	0.496399	0.832377	3.012952
MIR548AT	0.936869	0.496594	1.444167	0.754079
PRR12	0.936869	0.496646	-0.30908	7.089208
LINC00342	0.936869	0.496657	-0.34994	6.530361
ETFDH	0.936869	0.496709	-1.0741	0.951116
MLH3	0.936869	0.496712	-1.30707	2.179585
DDB1	0.937249	0.496985	-0.31857	8.028411
8-Sep	0.937441	0.49722	0.837942	1.923671
LOC101928	0.937441	0.497308	0.718595	2.567081
WDFY4	0.937441	0.497354	0.403713	5.629346
SLC7A5P1	0.937441	0.497452	0.764695	2.165067
EEF1A1	0.937441	0.497502	0.34078	11.04466
CMAHP	0.937441	0.497714	-0.44645	4.432646
NCAPG2	0.937441	0.4978	-0.93245	1.91089
DUSP11	0.937441	0.497893	-0.43001	4.825145
LOC101927	0.937441	0.497923	-1.65299	1.454658
ZNF396	0.937441	0.497939	-0.76449	2.307101
MIR3128	0.937441	0.497957	-1.71208	1.429389
HDAC1	0.937441	0.498007	0.300894	7.423904
SLC30A7	0.937441	0.498024	-0.7848	2.350607
SPOUT1	0.937441	0.498087	0.32391	5.703831
ZNF516	0.93755	0.498246	0.343104	6.538039
ACSS3	0.93755	0.498327	-0.82492	3.02376
PDXDC2P-N	0.93755	0.498359	0.305822	7.849051

IL18RAP	0.937628	0.498533	-0.75608	3.628136
BRD1	0.937628	0.498651	0.53984	3.330384
SNORD34	0.937628	0.498705	-0.59079	3.435127
NDUFS7	0.937628	0.498719	0.398881	4.295063
JAK3	0.937628	0.498818	-0.41902	5.403352
FITM2	0.937628	0.498839	-0.73147	2.559963
ZAP70	0.937628	0.498901	-0.95691	2.322554
ATP6V1B1	0.93772	0.499032	-0.69253	2.526124
TCEA1	0.93772	0.49918	-0.89017	1.72834
CUL1	0.93772	0.499213	-0.32895	6.741026
PCCA-AS1	0.93772	0.499236	0.65196	2.689078
PRDM4	0.93781	0.499369	0.326546	6.137985
MAP2K4P1	0.93781	0.499427	-1.13575	1.63582
INAVA	0.937839	0.499514	0.670721	2.163425
PNP	0.937866	0.499872	0.358778	6.137563
HSP90AB4I	0.937866	0.499949	0.500625	4.552639
POLG2	0.937866	0.499969	-0.40494	4.684516
GCLC	0.937866	0.499975	-1.20918	1.225198
MACC1-AS	0.937866	0.499979	0.478475	3.55122
ZSCAN29	0.937866	0.50001	-0.32061	6.412742
ALDH1A3	0.937866	0.500028	-0.82096	2.339607
ATM	0.937962	0.500181	-0.31512	8.15503
TYK2	0.937962	0.500223	-0.30708	6.9076
PEX12	0.938063	0.500501	-0.86388	3.103952
WASH8P	0.938063	0.500534	-0.4538	4.954816
GPR37	0.938063	0.50062	0.920972	1.443275
ARL14	0.938063	0.500627	0.417746	4.327893
OTULIN	0.938063	0.500653	-0.33276	6.499575
RNF11	0.938063	0.500991	0.311408	6.558625
SPAG16	0.938063	0.501162	0.84448	3.078461
EPS8L1	0.938063	0.50117	1.068054	1.054986
SAMD11	0.938063	0.501243	-1.02104	2.805188
PIBF1	0.938063	0.501388	-0.32004	6.042572
ZNF17	0.938063	0.5014	-0.54163	4.328427
HSPA1B	0.938063	0.501437	-0.8376	4.361522
PKIG	0.938063	0.501446	-1.29818	2.083983
TMEM61	0.938063	0.501563	1.09165	1.299495
ACTL10	0.938063	0.501575	-0.70377	3.388565
MIR320B2	0.938063	0.501632	-1.10275	1.775966
H2AFX	0.938063	0.501644	-0.42111	4.694135
SNORD103	0.938063	0.50165	-0.63652	3.868922
GRIN2B	0.938063	0.501682	-1.13708	2.845623
GEN1	0.938063	0.50171	0.569566	2.933348
RNF138	0.938063	0.501806	-1.10356	1.530575
MCF2L2	0.938063	0.50185	0.726248	2.796909

C19orf25	0.938068	0.501941	-0.51361	4.412038
HIST1H3F	0.938068	0.502095	-0.51147	3.83699
LINC01134	0.938068	0.502096	0.742168	2.409278
CYC1	0.938068	0.50239	0.354368	5.924264
BBS10	0.938068	0.502498	-0.55518	3.910403
ST8SIA4	0.938068	0.502541	-0.61191	3.900497
CLDND2	0.938068	0.502596	-1.05632	1.803703
RB1-DT	0.938068	0.502662	-1.5837	1.539229
TBC1D26	0.938068	0.502672	1.277013	0.764718
MEG9	0.938068	0.502864	-1.11749	0.978296
LOC101928	0.938068	0.502877	-0.86449	2.187605
NUDT19	0.938068	0.502909	0.375692	4.395093
SCARA5	0.938068	0.502944	0.524962	3.275296
LYPLA2P2	0.938068	0.503105	-1.23391	1.856914
NLGN2	0.938068	0.503449	-0.37756	5.44587
SCPEP1	0.938068	0.503512	-0.39779	5.282454
SLC12A7	0.938068	0.503548	-0.38167	6.420921
SV2A	0.938068	0.503566	-0.89479	2.573751
COA1	0.938068	0.503609	-0.36669	4.900216
DIMT1	0.938068	0.503722	-0.46263	4.712336
ZNF592	0.938068	0.503876	0.274947	6.913823
LINC01588	0.938068	0.503905	0.621435	2.582454
TBC1D5	0.938068	0.503926	0.48377	3.540581
CFAP97	0.938068	0.503975	-0.42109	5.092929
HERC2P9	0.938068	0.504037	-0.40149	5.136667
ULK2	0.938068	0.504061	-0.78326	3.312348
HERC2	0.938068	0.504166	0.286215	6.694065
ZEB2-AS1	0.938068	0.504296	-1.45494	1.68233
GBAP1	0.938068	0.504311	0.391727	4.528157
DDX24	0.938068	0.504355	-0.64055	4.196184
C1orf174	0.938068	0.504412	0.35285	4.60842
CALHM5	0.938068	0.504561	-0.82221	2.842447
TCIRG1	0.938068	0.504626	0.355316	4.708373
CCZ1B	0.938068	0.504707	0.324421	5.517255
BCL6	0.938068	0.504898	0.667331	2.736423
KIAA0087	0.938068	0.505052	-1.47154	0.859489
DAPK3	0.938068	0.505183	-0.31466	6.351145
SLC2A5	0.938068	0.505268	-1.0574	1.716474
PSMD7	0.938068	0.505381	-0.30003	7.065176
LRRC69	0.938068	0.505496	-0.8978	1.935126
LPIN1	0.938068	0.505591	0.7912	1.915023
FRAT1	0.938068	0.505612	0.521313	3.244032
SLC6A1	0.938068	0.505637	0.944723	1.322935
MAD2L1	0.938068	0.505815	0.51829	5.107651
SPIN3	0.938068	0.505837	0.361637	4.713149

RPN1	0.938068	0.505883	0.306201	8.305398
PIGS	0.938068	0.505914	-0.36163	5.401735
RPS11	0.938068	0.505932	-0.31517	9.006994
CCDC18-AS	0.938068	0.50601	0.337468	5.640035
SOCS6	0.938068	0.506017	-0.40885	6.551395
ILKAP	0.938068	0.506018	-0.38794	5.761198
KCNC4	0.938068	0.506047	-1.31802	0.851102
ALDOA	0.938068	0.506086	0.713777	2.616658
CIRBP-AS1	0.938068	0.506161	-0.62287	3.126104
TMEM9B-A	0.938068	0.50619	-0.72294	2.22917
ZNF658B	0.938068	0.506212	-0.92171	2.528587
ZNF598	0.938068	0.506252	-0.325	6.426561
ZNF134	0.938068	0.506314	-0.75313	3.582139
ZNF774	0.938068	0.506598	-0.73014	2.782886
VMAC	0.938068	0.50672	0.399163	3.834118
IRF3	0.938068	0.506804	-1.06465	2.178757
SCARNA12	0.938068	0.506846	-0.3051	6.797138
UBE2J1	0.938068	0.506984	0.291642	7.135911
ZNF614	0.938068	0.507124	-0.44062	4.127201
RELCH	0.938068	0.507189	0.305674	7.072509
TAS2R19	0.938068	0.507218	-1.2406	1.039358
CELF2-AS2	0.938068	0.50728	-0.57846	3.306343
DRAM1	0.938068	0.507283	-0.39709	5.459362
KANSL1	0.938068	0.507322	-0.84257	1.854265
LOC100130	0.938068	0.507352	-0.75169	2.745807
AMOT	0.938068	0.5074	-0.53861	3.348699
TSSC2	0.938068	0.507419	-0.88122	1.909569
CCDC28A	0.938068	0.507423	-0.38442	4.647211
DDX31	0.938068	0.507448	0.534928	3.942687
ZNF575	0.938068	0.507473	0.632669	1.909586
NGLY1	0.938068	0.507579	0.830925	1.709905
CCDC112	0.938068	0.507596	0.714102	1.429282
RNU4-1	0.938068	0.507667	-0.35762	8.870253
ZNF397	0.938068	0.50768	0.354296	5.324741
CBWD5	0.938068	0.50773	-0.72817	2.524872
NDUFS4	0.938068	0.508006	-0.38204	5.544426
EFNB2	0.938068	0.508035	-0.34188	7.529089
ZNF830	0.938068	0.50813	-0.44121	5.053609
CRYBG3	0.938068	0.508162	0.293889	7.810618
LOC101929	0.938068	0.508168	-0.85565	2.133229
TFAP2A-AS	0.938068	0.508183	-1.15905	0.994311
MIR646HG	0.938068	0.508221	-1.07607	1.440946
LOC100505	0.938068	0.508378	-0.82679	2.821936
TPRG1-AS2	0.938068	0.508415	1.211821	0.887716
DNAJC18	0.938068	0.508444	0.4927	4.26707

DNER	0.938068	0.508534	0.93741	1.132986
CCDC14	0.938068	0.508601	-0.90816	1.745318
TTC7A	0.938068	0.508623	0.902145	1.814193
CCNL2	0.938068	0.508636	-0.31131	7.496186
TFPI	0.938068	0.508651	0.367097	5.736712
SDF2L1	0.938068	0.5088	-0.54167	3.681525
FZD6	0.938068	0.508847	0.978556	1.086716
TESK1	0.938068	0.508859	0.338495	5.300737
SLC6A7	0.938137	0.509007	1.043055	2.079227
SETX	0.938137	0.50912	-0.29986	8.718584
PELO	0.938137	0.509131	-0.41083	5.248929
MPHOSPH8	0.938137	0.509345	-0.30642	8.116302
SNORD22	0.938137	0.509371	-0.38917	8.238527
MESTIT1	0.938137	0.509372	-0.92939	2.442281
TMEM200A	0.938137	0.509465	-1.30681	1.353085
FAM155A	0.938137	0.509537	0.633083	3.36476
LINC01798	0.938137	0.50954	0.966543	1.060729
KPNA2	0.938407	0.509758	-0.45853	5.757045
LXN	0.938427	0.509841	0.412078	4.148254
DNM1P46	0.938478	0.50994	0.710984	2.611529
LRIG3	0.93857	0.510128	0.580717	3.091705
SLX4	0.93857	0.510133	-0.37635	5.430637
EXOC6	0.938597	0.510299	0.808871	1.361325
GRK5	0.938597	0.51046	0.311227	6.528406
PDE6A	0.938597	0.510535	0.636764	2.266173
CCDC96	0.938597	0.51063	-0.85266	1.639658
TNNI3	0.938597	0.510685	-1.65297	1.046444
CIAO2A	0.938597	0.510845	0.436086	3.870631
TARBP2	0.938597	0.510848	0.964752	1.030726
HCFC2	0.938597	0.51086	0.337571	5.671098
TRPC5	0.938597	0.510879	-1.05733	1.270258
LONP2	0.938597	0.510926	-0.47169	3.976174
TRIM63	0.938597	0.510935	-1.28252	0.828461
PTPRB	0.938629	0.511023	-1.09339	1.19945
RBM12B-A	0.938932	0.51126	0.464499	3.602406
NFATC2IP	0.938992	0.511416	0.264658	6.931657
NEBL	0.938992	0.51156	-0.51174	4.967461
TP53I3	0.938992	0.511588	0.806096	1.695518
PEAK1	0.938992	0.511629	0.28961	8.383866
ANP32A	0.938992	0.51165	0.292899	7.29178
UBE2M	0.939718	0.512152	-0.35208	5.714625
MSI1	0.939718	0.512231	0.791134	1.71737
CCDC82	0.939718	0.512261	-0.30386	6.742296
STARD7	0.939888	0.512532	-0.28801	7.583943
PLCD3	0.939888	0.512684	0.394526	6.897483

FADD	0.939888	0.512687	-0.45169	5.12185
LINCR-000:	0.939888	0.512741	0.778111	1.704973
UBE2R2	0.939888	0.512765	-0.30273	6.88031
FBXL14	0.939888	0.513024	0.362737	4.53005
AQP7	0.939888	0.513088	-1.08332	2.729068
C5	0.939888	0.513101	0.919097	0.775826
NPIPB3	0.939888	0.513113	-0.37262	5.250884
SPCS1	0.939888	0.51325	0.32326	5.675915
MIR5699	0.939888	0.513255	-1.15738	0.807941
RAD23B	0.939888	0.513297	0.338657	4.921444
FAM216A	0.939888	0.513336	-0.81624	2.560371
ZNF391	0.939888	0.513414	-1.09937	1.877308
ZC3H12D	0.939888	0.513449	0.502138	3.780006
LOC10192S	0.939888	0.513513	-1.12218	1.151667
ATRN	0.939888	0.513615	0.287096	6.087902
IGFBP7	0.939888	0.513643	-0.50933	3.770256
NOS3	0.939951	0.513789	-0.58768	3.811722
PELI2	0.939951	0.513876	-0.34607	6.262539
RNF5P1	0.939951	0.514004	0.690635	2.040819
GIT2	0.939951	0.514021	-0.6427	3.186575
ANKRD1	0.939951	0.514094	1.160289	0.592983
TIAM1	0.939951	0.514174	0.511838	5.215258
RCC1L	0.939951	0.514217	-0.55907	3.941056
NUTF2	0.939951	0.514283	-0.39465	5.206768
PCED1A	0.939951	0.514322	0.484248	3.689342
SNORA55	0.940015	0.514437	0.430753	7.784054
TBK1	0.940015	0.514634	-0.30264	6.197436
GNG12-AS:	0.940015	0.514758	0.556122	3.515715
MICALCL	0.940015	0.51481	-0.5886	4.230968
DUS3L	0.940015	0.514816	0.64256	2.208486
CRYM	0.940015	0.514877	-0.82583	2.18272
GOLGA6L1	0.940015	0.514885	0.476615	4.014155
DOCK4	0.940015	0.51493	-0.32666	6.498326
CYP17A1	0.940242	0.51516	0.942076	1.259699
TIMMDC1	0.940242	0.515198	0.287373	6.087726
LOC10192S	0.940309	0.515406	0.710409	3.012667
RPTOR	0.940309	0.515444	-1.10295	2.02176
SHROOM1	0.940309	0.51545	0.711466	1.515384
LOC10192S	0.940488	0.515807	0.649382	2.202436
TET2	0.940488	0.51582	0.27016	6.848713
EHF	0.940488	0.51589	-1.03047	1.788469
MBL1P	0.940488	0.515934	0.93832	0.858511
LOC10041S	0.940488	0.515972	-0.36282	5.279274
ABRACL	0.940488	0.516019	-0.45222	4.652274
SETD1B	0.940488	0.5162	-0.28451	7.728283

NCLN	0.940488	0.516249	0.294267	6.791041
MVB12B	0.940488	0.516366	0.354153	4.580905
LOC440028	0.940488	0.516523	-0.99079	1.783672
ALG2	0.940488	0.516537	0.909336	1.010379
PPIG	0.940488	0.516605	-0.2904	8.533503
ALKBH3	0.940488	0.516657	-0.37847	4.551068
CS	0.940488	0.516706	0.30004	7.363752
MED14OS	0.940488	0.516987	0.984387	0.977506
LOC100996	0.940488	0.517111	0.893142	1.26898
ZNF232	0.940488	0.517115	0.339929	4.386273
LYPD3	0.940488	0.517117	0.887493	4.811834
FAM200B	0.940488	0.51732	0.360273	4.814114
RBM5-AS1	0.940488	0.517336	0.3505	5.049575
RHOB	0.940488	0.51747	-0.30286	8.029005
TMEM173	0.940488	0.517471	-0.96022	1.401951
FAM241B	0.940488	0.517573	-1.02131	2.342059
RHBDD2	0.940488	0.517702	0.895744	0.942822
NEDD4L	0.940488	0.51786	0.469113	3.877327
AACS	0.940488	0.517982	0.331445	5.722319
HNF4A-AS1	0.940488	0.518021	0.597793	3.849993
CPLANE2	0.940488	0.518021	0.753388	2.365145
LOC100133	0.940488	0.518048	-0.67593	2.232534
MRPS6	0.940488	0.518055	-0.33812	5.313985
UBA7	0.940488	0.518209	0.359084	6.198156
ADGRB3	0.940488	0.518219	-0.76258	2.995916
TMEM263	0.940488	0.518269	0.292546	6.338018
PPP1R14B	0.940488	0.518317	-0.42157	5.360955
CCR10	0.940488	0.518321	0.719925	2.172426
KY	0.940488	0.518498	-0.94515	1.479117
ANKRD44-I	0.940488	0.518549	-0.45175	4.192633
GOLGA5	0.940488	0.518552	-0.33013	6.311244
KPNA5	0.940488	0.518649	-0.42036	4.426493
UBR1	0.940488	0.518812	-0.30112	7.147677
RFFL	0.940488	0.518871	0.748608	1.345652
WEE2-AS1	0.940488	0.518901	0.99671	1.305521
ZNF552	0.940488	0.51901	-0.39897	5.282455
LOC101927	0.940488	0.519108	-1.48185	0.932433
R3HDM2	0.940488	0.519114	-0.28485	7.505554
SRSF12	0.940488	0.519131	-0.67591	3.07993
UBE2B	0.940488	0.519154	0.298633	5.628875
AGGF1	0.940488	0.519186	-0.29661	6.637035
SNORD56	0.940488	0.519226	-0.44723	6.38166
TNFAIP8	0.940488	0.519309	-0.90589	1.954826
ULBP3	0.940488	0.519315	0.618971	3.259209
LOC100507	0.940488	0.519341	0.521127	3.911265

TMEM242	0.940488	0.519347	0.430899	4.444234
NAF1	0.940817	0.5196	-0.73688	2.272153
MAPK8IP3	0.940914	0.519753	-1.43968	0.894749
ACOT11	0.940914	0.519798	-0.80523	2.793156
HMBOX1	0.940923	0.519874	0.412578	3.806341
MCPH1	0.941024	0.520002	-0.68678	2.402041
MIR3064	0.941098	0.520136	0.481742	3.595983
MMP16	0.941098	0.520186	-0.66767	3.243556
MIR29B2	0.941103	0.52035	-0.79492	1.747969
CBFA2T2	0.941103	0.52035	0.532724	2.499968
CH17-340M	0.941103	0.520404	0.843821	1.638653
CD248	0.941247	0.520556	-0.39083	5.401272
PNRC2	0.941459	0.520836	-0.28563	7.715661
TELO2	0.941459	0.520846	0.331859	5.465994
CISD1	0.941459	0.52093	-0.45594	4.139856
LOC101927	0.941459	0.521122	0.502944	3.217675
CSRP3	0.941459	0.521126	1.147686	1.15126
ZBTB7A	0.941459	0.521183	-0.94579	1.290808
CD276	0.941459	0.521228	-0.74505	2.556788
MCMDC2	0.941459	0.521334	-1.22601	1.75286
DYNLT3	0.941459	0.521342	-0.33881	5.710432
VSTM4	0.941459	0.52139	0.341371	4.896528
E2F2	0.941466	0.521504	-0.69048	3.408508
SLC44A3-A	0.941466	0.521577	0.474011	3.28272
CCR2	0.941466	0.521642	-0.84854	2.177098
GOLGA8O	0.941466	0.52177	0.731403	1.963921
LOC283788	0.941466	0.521807	0.328874	6.270527
GUCY1B2	0.941466	0.521825	-0.72274	2.691694
ABCA9	0.941492	0.52205	-0.56059	5.368391
C14orf28	0.941492	0.52207	0.488374	3.721944
ZNF70	0.941492	0.522215	-0.39397	4.804818
PRSS16	0.941492	0.52224	0.448192	3.567082
RHBDL2	0.941492	0.52229	0.899924	0.808718
PPP4R1L	0.941492	0.522376	0.314535	4.989483
KDELC1	0.941492	0.522473	0.550711	2.971545
ZNF544	0.941492	0.522666	0.342956	5.807615
ZFAND2A	0.941492	0.52276	-0.4034	4.558453
NAP1L4	0.941492	0.522848	-0.39634	5.734693
SYMPK	0.941492	0.522875	-0.28644	7.038638
LYSMD1	0.941492	0.52298	-0.63551	2.810835
HSPA2	0.941492	0.523262	0.396247	5.103455
DDX60	0.941492	0.523275	-0.34791	7.080176
IRF1	0.941492	0.523392	-0.35196	6.451655
SGTA	0.941492	0.523399	-0.29979	6.222016
SEMA6B	0.941492	0.523409	-0.54051	3.463

MCF2L-AS1	0.941492	0.523447	-1.15274	1.203834
C5AR2	0.941492	0.523461	1.152362	1.004484
TMX3	0.941492	0.523497	-0.34534	6.201425
KLF2	0.941492	0.523561	-0.41657	5.932982
ZDHHC21	0.941492	0.52369	0.290666	6.534094
SIRPA	0.941492	0.523712	0.88137	0.911027
SNORD88A	0.941492	0.523764	-0.96895	1.455901
USP13	0.941492	0.523809	-0.38501	5.382437
ATP5F1B	0.941492	0.523838	0.311035	8.151564
LINC01011	0.941492	0.523881	-0.91263	1.313905
GHITM	0.941492	0.523924	-0.34172	7.158582
LOC101929	0.941492	0.523976	-1.20563	1.491333
C3AR1	0.941492	0.524215	-0.85615	3.645699
SNORD132	0.941492	0.524241	0.946529	0.702986
EZH1	0.941492	0.524251	-0.29624	6.791979
GNA14-AS1	0.941492	0.524269	0.998171	1.032485
PIP4K2A	0.941492	0.524341	-0.30719	6.360254
GPR160	0.941492	0.524404	0.429838	5.808942
DCLRE1C	0.941492	0.524422	-1.16532	1.444003
LSM5	0.941566	0.524535	-1.13794	1.049982
SLC37A4	0.941694	0.524722	0.856612	1.31969
ZBTB38	0.941694	0.52486	-0.32837	7.941784
MST1P2	0.941694	0.524888	-0.57402	3.261878
ZNF818P	0.941694	0.524894	-0.83262	2.715388
DDOST	0.942013	0.525144	0.280379	7.040118
LRRC55	0.94202	0.525387	1.054576	1.349567
DLAT	0.94202	0.525447	-0.3158	5.989686
VAMP5	0.94202	0.525473	-0.53815	3.459073
LOC101927	0.94202	0.525506	0.705384	2.335067
ACOXL-AS1	0.94202	0.525506	0.945164	0.904985
RARRES1	0.942256	0.525841	-1.09087	2.491219
EFCAB13	0.942256	0.525978	-0.6133	4.522498
HDAC6	0.942256	0.525995	-0.29884	6.299969
HBS1L	0.942256	0.526009	-0.4966	3.458096
TOGARAM1	0.942256	0.526059	0.711417	2.621993
CYSRT1	0.942256	0.526069	0.75013	2.612374
SNX31	0.942279	0.52629	0.95488	1.442265
PHPT1	0.942279	0.526291	-1.04705	1.838454
CNTLN	0.942279	0.526297	-0.54195	4.903232
SEC14L5	0.942468	0.526531	0.90328	1.753251
HEATR3	0.942468	0.526562	0.364024	4.751949
ZNF354C	0.942468	0.526619	-0.51593	4.60198
MIB1	0.942541	0.526839	-0.33086	7.926416
TRG-AS1	0.942541	0.526847	-1.13072	2.131187
HMGXB3	0.942541	0.526875	-0.28878	6.483767

KCTD15	0.942602	0.527032	-1.11437	1.323343
CETN2	0.942602	0.527087	-0.50142	4.417403
TRAPPC12	0.942602	0.527124	0.300121	6.064049
AGAP1	0.942644	0.52722	0.724431	1.927325
NAB1	0.942736	0.52744	-0.30047	7.789304
EFNA2	0.942736	0.527473	0.787884	3.017605
PIGA	0.942736	0.527487	0.784111	1.521065
EREG	0.94288	0.527639	-0.49233	4.388829
RB1	0.942966	0.527807	-0.28883	7.0813
SNTN	0.942966	0.527831	-1.88986	1.153966
TRIR	0.943063	0.528119	-0.30849	7.037272
C9orf41-AS	0.943063	0.528138	0.452727	3.510579
NACC2	0.943063	0.528231	-0.3324	6.164054
COX10	0.943063	0.528248	0.362202	4.498534
MEGF6	0.943063	0.52829	-0.38605	6.674162
MIER2	0.943063	0.528317	-0.31406	5.377853
SAPCD2	0.943224	0.528497	-0.47212	4.567657
CHMP4A	0.943224	0.52855	0.2789	5.844897
DCHS2	0.943271	0.528767	-0.75342	4.323842
LUC7L2	0.943271	0.528807	-0.55111	4.653116
FAM180A	0.943271	0.528927	-0.89097	2.676527
PHF11	0.943271	0.529043	-0.79264	2.728506
FGD5	0.943271	0.529082	-0.49861	6.180349
DESI1	0.943271	0.529302	-0.27814	6.117652
SNRNP48	0.943271	0.529315	-0.31392	5.814908
OFD1	0.943271	0.529542	-0.2929	7.048618
GATAD1	0.943271	0.529554	-0.71634	3.175411
MRPS7	0.943271	0.529567	0.336841	5.056325
SNW1	0.943271	0.52962	-0.32429	6.621256
ARMT1	0.943271	0.529678	0.754843	1.892083
FAM222B	0.943271	0.529836	-0.79116	2.09135
ZNF608	0.943271	0.529873	0.329527	6.731926
METTL21EI	0.943271	0.529886	0.908148	0.939185
FOXK2	0.943271	0.530026	-0.28868	6.510654
ABHD17C	0.943271	0.530088	0.338729	6.605332
SMIM15	0.943271	0.530188	0.308621	5.691894
NOL8	0.943271	0.530327	0.816855	1.916263
SELENOT	0.943271	0.530467	-0.29804	5.843176
SFMBT1	0.943271	0.530528	0.283837	6.52846
TOMM34	0.943271	0.530571	-0.38744	4.698143
ANKHD1	0.943271	0.530607	0.596413	3.242008
FLJ22447	0.943271	0.530676	-1.29283	0.990235
ANAPC13	0.943271	0.530694	0.526044	2.493724
NODAL	0.943271	0.530943	-1.02371	1.659252
FREM2	0.943271	0.530945	-0.71844	4.290428

CBX5	0.943271	0.530985	0.55863	2.898385
ANO1	0.943271	0.531004	0.395267	5.614956
PRSS23	0.943271	0.531039	-0.91906	2.725653
B2M	0.943271	0.531362	0.319271	7.443037
BROX	0.943271	0.53138	-0.58095	2.53433
ZBED8	0.943271	0.531447	-1.39852	1.198487
BOLA1	0.943271	0.531521	-0.84237	2.295647
COG5	0.943271	0.531629	0.375383	4.229426
HOXA-AS2	0.943271	0.531666	-0.91833	1.511341
MYRF	0.943271	0.531684	0.648215	3.188237
CLN8	0.943271	0.531798	0.568587	4.025401
GUSBP9	0.943271	0.53183	0.53434	3.784239
SHC3	0.943271	0.531882	0.424358	4.991134
NAPSB	0.943271	0.531934	-0.67854	3.305714
IVL	0.943271	0.53195	1.188217	5.347873
SPATA1	0.943271	0.531955	0.561626	3.544048
NFAM1	0.943271	0.532004	-0.46136	3.741914
CFAP36	0.943271	0.532044	1.204824	0.717625
RND2	0.943271	0.532073	-1.29353	1.149532
TSPAN18	0.943271	0.532167	-0.40819	4.897188
FURIN	0.943271	0.532196	0.851092	0.844359
SHANK2	0.943271	0.532237	0.388392	5.307281
LCMT1	0.943271	0.532243	0.684998	1.975022
LOC22072	0.943271	0.532243	-0.33726	5.731043
ERBB2	0.943411	0.532394	0.865272	1.248584
CBWD6	0.943415	0.532469	-0.60858	3.41072
UBB	0.943415	0.532647	-1.48921	0.96459
PABPN1	0.943415	0.532694	0.353586	5.497183
VMP1	0.943415	0.532795	-0.26533	8.371175
GOLPH3	0.943415	0.532863	0.251998	7.177149
MFHAS1	0.943415	0.532867	-0.31244	6.187512
TRIM37	0.943415	0.532937	-0.74205	3.257662
FAM210B	0.943415	0.533027	0.313862	6.389198
LOX	0.943415	0.533124	-0.82248	2.830298
FNBP4	0.943415	0.533191	-0.28285	8.475757
NADK2	0.943415	0.533342	0.621132	1.856931
MAFIP	0.943415	0.533347	0.413118	4.596149
WDFY3	0.943415	0.533379	-0.27483	7.979841
PCSK4	0.943415	0.533405	0.642335	2.795035
DSG2	0.943415	0.533475	-0.38165	8.589485
TMEM52	0.943513	0.533603	-1.67323	1.294918
KIF9-AS1	0.943541	0.533712	0.763696	1.655555
LINC00475	0.943541	0.533762	0.669899	2.205209
HIST1H2BL	0.943657	0.533919	-0.51179	4.269823
RHOF	0.943657	0.533972	0.739908	2.863327

GZMK	0.943675	0.534058	-1.24268	1.741874
CTDNEP1	0.943675	0.534254	0.735376	1.868421
TAF1B	0.943675	0.534407	-0.37319	4.323902
LINCR-0002	0.943675	0.534421	1.14352	1.004653
GPR143	0.943675	0.534474	-1.16533	1.976862
NHSL2	0.943675	0.534538	-0.40406	4.132747
MBD2	0.943675	0.534583	0.280028	7.390932
ADORA2A-A	0.943675	0.534639	0.649311	2.560777
EXTL3	0.943675	0.534723	0.301189	5.067362
SMIM10L1	0.943675	0.534829	-0.40776	4.66193
PPP3CB	0.943675	0.534906	-1.52838	0.696504
RNASEH2A	0.943675	0.534938	-0.48262	3.811858
MLF2	0.943675	0.534974	0.590689	2.562564
LINC01410	0.943675	0.534994	-0.71611	3.106271
LINC01002	0.943675	0.535061	0.584731	2.34172
PPM1K	0.943753	0.535177	0.352636	5.694688
PA2G4P4	0.943785	0.535325	-0.61042	4.178724
LOC101928	0.943785	0.535339	0.629563	1.423101
BBS2	0.943841	0.535605	0.305182	5.703234
SIDT2	0.943841	0.535654	0.294405	6.161908
DENND1B	0.943841	0.535664	0.563277	2.804584
GLG1	0.943841	0.536006	0.802552	1.442218
RORB	0.943841	0.536183	0.716543	3.209183
NFE2L1	0.943841	0.536192	0.245721	8.948983
GPR135	0.943841	0.536305	-0.94406	2.536301
LEO1	0.943841	0.536395	0.500715	2.578902
ZSWIM9	0.943841	0.53648	0.35754	5.249999
SCARNA11	0.943841	0.536581	-0.54111	3.520331
AADACP1	0.943841	0.536623	0.622146	3.500611
NEGR1	0.943841	0.536639	0.389139	5.401332
PRKAB2	0.943841	0.536869	0.89392	1.357539
C8orf34	0.943841	0.536871	0.945763	1.419296
CHMP7	0.943841	0.536877	-1.11798	0.978864
AEBP2	0.943841	0.537019	-0.76589	2.049169
ZNF582	0.943841	0.537031	0.833117	1.557124
SLC16A3	0.943841	0.537086	-0.88847	2.200729
MEF2C-AS1	0.943841	0.537108	-0.97586	1.164288
GLDN	0.943841	0.537239	0.493695	3.540978
PPP4R3A	0.943841	0.537285	-0.36193	4.726669
CHST14	0.943841	0.537328	-0.72254	2.830974
GLOD5	0.943841	0.537645	0.819951	1.546629
ANKRD22	0.943841	0.537664	-0.40434	5.638587
FMO2	0.943841	0.537699	-1.19257	2.138452
ZNF264	0.943841	0.537723	-0.27851	7.217791
NDUFB1	0.943841	0.537726	0.470476	4.688351

OXSR1	0.943841	0.538061	-0.31231	7.320214
CC2D2B	0.943841	0.538183	-0.81483	2.97914
IDH3A	0.943841	0.538193	0.273924	6.156943
LINC02611	0.943841	0.538233	-1.42141	0.948426
BAG4	0.943841	0.538358	0.799391	1.513588
FLRT2	0.943841	0.538609	-0.58837	4.849001
SNORD112	0.943841	0.538614	-1.0483	1.16853
CNOT11	0.943841	0.538863	-0.27606	6.59038
ENO1	0.943841	0.538875	-0.32509	6.1676
PLXNA4	0.943841	0.538887	-0.5258	5.240943
CDCP1	0.943841	0.538918	-0.35883	6.542804
RNFT1	0.943841	0.53911	0.354644	4.075697
NSFP1	0.943841	0.539182	-1.03785	1.07374
MAP3K20	0.943841	0.539188	-0.32634	7.722876
GREM2	0.943841	0.53926	-0.7988	4.982731
STBD1	0.943841	0.539274	0.596307	2.353924
MIR7854	0.943841	0.539278	0.877267	0.846133
PLXNB2	0.943841	0.539297	0.263267	9.195379
LINC00113	0.943841	0.539397	1.036917	0.76738
ASPHD1	0.943841	0.539474	-0.44263	4.827725
XRCC2	0.943841	0.539547	-0.87877	3.406965
CYCSP52	0.943841	0.539616	-1.32981	1.022668
RTN4IP1	0.943841	0.53969	0.370352	3.970934
GSE1	0.943841	0.539903	-1.05562	1.429148
DKFZP434H	0.943841	0.540016	0.964197	0.697765
MRPL18	0.943841	0.540084	-0.34157	5.370159
NAALADL1	0.943841	0.540199	-0.97929	1.074465
SLC27A5	0.943841	0.540236	0.515388	2.69756
ZCCHC18	0.943841	0.540238	1.11457	1.036376
GPATCH1	0.943841	0.540375	-0.31644	5.390253
IRF8	0.943841	0.540444	0.332011	5.776543
MIR5192	0.943841	0.540464	-1.55354	1.561895
S1PR2	0.943841	0.540507	-0.50171	3.929811
RNA5S9	0.943841	0.540533	0.38817	8.315053
C15orf61	0.943841	0.540541	-1.04328	1.148509
CIZ1	0.943841	0.540547	-1.10663	1.571675
ARRDC1-AS	0.943841	0.540565	-0.75281	1.549634
PHLDB1	0.943841	0.540707	0.801527	1.511004
MYO18B	0.943841	0.540946	0.674962	2.478917
CARS2	0.943841	0.540947	-0.34028	5.450445
RND1	0.943841	0.540962	-0.78506	2.98272
ARHGAP27	0.943841	0.540985	0.308065	5.365357
PLEKHA4	0.943841	0.54104	-1.22879	1.174388
KREMEN1	0.943841	0.54106	0.368528	5.108092
FAM118A	0.943841	0.541287	-1.21931	0.892268

CYP27A1	0.943841	0.541338	-0.55441	3.971734
TET2-AS1	0.943841	0.54136	0.466282	3.304096
LDLRAP1	0.943841	0.541368	0.284403	5.490267
SNORD59A	0.943841	0.54139	-0.43512	6.559743
RGL4	0.943841	0.541498	0.29361	5.747027
DNASE1L1	0.943841	0.541539	-0.77816	3.181473
MFSD4B	0.943841	0.541582	-0.30529	5.630888
ARR3	0.943841	0.541589	-0.8563	2.291717
SLC25A16	0.943841	0.541715	-0.42115	4.333183
MAP6	0.943841	0.541739	-0.74259	2.618298
IPO8	0.943841	0.541765	0.342551	4.558115
NRARP	0.943841	0.541876	0.331016	6.279072
UBL3	0.943841	0.541884	-0.30257	6.787746
HOXB-AS1	0.943841	0.541917	0.821484	1.727853
SPATA2L	0.943841	0.541925	0.452743	3.359713
BEND7	0.943841	0.541989	-0.8878	2.204444
SLC45A1	0.943841	0.542042	-0.73082	2.588809
TP53INP1	0.943841	0.542082	-1.25123	1.075192
IGF2BP2-A!	0.943841	0.54212	-0.53789	3.283944
CASP2	0.943841	0.542155	-1.07947	0.802319
PDLIM5	0.943841	0.542187	0.61216	3.073942
RPL13AP5	0.943841	0.542368	0.310239	5.721225
TRPM5	0.943841	0.542404	-0.69242	3.38772
DIPK1B	0.943841	0.542448	0.51039	3.047455
MOCS3	0.943841	0.542564	-0.44941	3.900477
ASB12	0.943841	0.542639	1.056906	0.76557
MTRNR2L4	0.943841	0.542662	-1.06939	1.339358
PTGFRN	0.943841	0.542707	0.258383	7.906026
UBE2N	0.943841	0.542969	-0.36027	5.92714
KRT16P2	0.943841	0.542976	-1.79866	0.993489
USP7	0.943841	0.543137	-0.45751	3.926373
RAP2A	0.943841	0.543179	0.316401	6.077781
NCOA7	0.943841	0.543222	-0.611	3.167826
ELMOD2	0.943841	0.543276	0.347411	5.513682
SLC4A7	0.943841	0.543329	0.428321	3.525145
ACOT8	0.943841	0.543408	0.360786	4.031042
LINC01910	0.943841	0.543445	-1.45068	1.161196
SRGAP2C	0.943841	0.543457	0.457236	4.12076
ZNF358	0.943841	0.543461	-0.36999	5.073617
NDUFAB1	0.943841	0.543579	-0.40796	5.233138
FGD1	0.943841	0.543583	-0.42008	3.910294
LINC00597	0.943841	0.543623	0.465637	2.976019
HLA-DOA	0.943841	0.543706	0.989673	2.65521
MMP25	0.943841	0.543846	-0.48925	3.950452
TSPYL6	0.943841	0.543863	-0.72518	2.87523

SLC19A2	0.943841	0.543892	-0.34174	5.01586
EIF2AK4	0.943841	0.544027	0.262488	7.104328
TRIM62	0.943841	0.544053	-0.38789	4.276825
ACTC1	0.943841	0.54413	-0.94471	3.186972
PMS2P9	0.943841	0.544147	0.302252	5.191612
LOC10272	0.943922	0.544266	0.829601	1.427346
JUP	0.943939	0.544351	0.615663	2.698035
UGP2	0.943939	0.544664	0.320018	5.04217
HOXD3	0.943939	0.54472	0.862588	0.790325
DERL2	0.943939	0.544754	0.931837	1.540156
HSF1	0.943939	0.544759	-0.29202	5.946527
PTPRN2	0.943939	0.544771	-1.12447	1.81351
PP2D1	0.943939	0.544779	-0.80598	2.117838
DLST	0.943948	0.545076	-0.59162	3.853913
USP32	0.943948	0.545183	-0.29257	6.789139
SLC35A5	0.943948	0.545247	0.35249	4.979194
L3MBTL4	0.943948	0.545288	-0.64413	2.93982
GRASP	0.943948	0.545306	-0.99043	1.889293
C17orf82	0.943948	0.545323	0.887656	1.17762
SNORA57	0.943948	0.545338	0.335639	6.457477
DTD1	0.943948	0.545402	-0.37432	4.808955
SMYD3	0.943948	0.54544	0.670865	2.02982
EMC3-AS1	0.943948	0.545569	0.379842	3.96156
DANCR	0.943948	0.545576	0.329581	5.081999
TCHP	0.944359	0.545935	0.751823	1.104737
ITGA4	0.944359	0.545968	-0.37561	5.501805
LPXN	0.944359	0.546091	-1.3426	0.608342
HAND2-AS	0.944359	0.546173	-1.56241	1.035845
RNF216	0.944359	0.546174	-1.05444	0.808155
TTL	0.944472	0.546372	-0.28624	6.229293
LOC10192	0.944472	0.546512	-0.85588	1.472055
PDIA4	0.944472	0.546528	0.294572	8.677399
RN7SL2	0.944472	0.546577	0.24145	14.31419
LOC10192	0.944472	0.546693	-1.0526	1.787335
CHUK	0.944472	0.546695	-0.32899	5.763776
CRIM1	0.944472	0.546847	-0.28015	7.904157
TXNRD2	0.944472	0.546867	-0.67173	3.013859
DDX21	0.944472	0.546902	-0.59676	3.534019
PLAGL2	0.944472	0.54696	-0.28123	5.932466
B3GNT8	0.944472	0.54707	0.433594	3.17243
OXNAD1	0.944472	0.547103	0.304962	5.684413
CRTC3-AS1	0.944514	0.547316	0.885716	1.732079
ZNF888	0.944514	0.547318	-0.38672	5.478765
MAIP1	0.944514	0.547343	0.344649	4.189084
OAS2	0.944569	0.547447	-0.40354	4.931259

LOC105379	0.944792	0.547738	0.759316	1.234971
LOC407835	0.944792	0.547751	-0.77136	2.063023
SMIM6	0.944792	0.547844	0.662853	2.745111
GINS1	0.944792	0.547864	-0.4649	4.51023
IL2RB	0.944882	0.548043	0.458508	3.871322
UBE4B	0.944882	0.54806	-0.78849	2.019403
ARHGAP17	0.94505	0.548247	-1.22934	1.311734
RAB11B	0.94505	0.548302	-0.27565	6.062808
LOC100289	0.945058	0.548408	-0.51167	3.64678
CATSPER3	0.945058	0.548605	-0.70998	2.53326
TMEM164	0.945058	0.548632	0.441758	3.95799
FAM193B	0.945058	0.548643	0.30914	5.161955
PREPL	0.945058	0.548666	0.535938	2.460571
TSPAN8	0.945145	0.548811	0.356979	8.859475
ARHGEF35	0.945145	0.548916	0.470371	3.480686
RNF19A	0.945145	0.548933	-0.51054	3.075134
DLL1	0.945231	0.549055	0.5277	3.525263
SHKBP1	0.94538	0.549383	-0.29176	5.828676
ESR2	0.94538	0.549548	0.669786	1.818946
COX6C	0.94538	0.54962	-0.34064	6.457954
IGFBP6	0.94538	0.549682	-0.59443	4.58078
USP15	0.94538	0.549776	-0.40028	4.019342
ACVR2B	0.94538	0.549795	-0.33039	5.662064
PPP1R12B	0.94538	0.549802	-0.57778	3.920931
ELP5	0.94538	0.549948	0.563067	2.585376
PDE4DIP	0.94538	0.549957	0.316674	5.461326
GPN2	0.94538	0.549965	0.375019	4.192217
EIF1AX-AS1	0.94538	0.549994	-1.32113	1.030516
LNP1	0.94538	0.550026	0.420376	3.125011
LOC101927	0.94538	0.550079	0.812508	1.802743
MCIDAS	0.945707	0.550359	0.866713	1.155185
C6orf132	0.945707	0.550538	-0.31776	7.911753
SPESP1	0.945707	0.550616	-1.161	1.64347
OTUD1	0.945707	0.550632	-0.33328	5.238631
FCF1	0.945707	0.550827	-0.35056	5.659728
POLR2H	0.945707	0.55085	0.710992	1.705083
CEP112	0.945707	0.550873	0.644618	2.503945
SF3A1	0.945707	0.55088	-0.26201	7.918673
ZNF316	0.945707	0.550918	-0.28747	6.41393
SLC2A4RG	0.94571	0.551186	-0.33561	5.262771
FRMD4B	0.94571	0.551444	-0.31394	6.349377
GSN-AS1	0.94571	0.551592	0.304964	5.643151
GAS6	0.94571	0.551687	-0.33241	7.371155
AGT	0.94571	0.551696	0.388627	5.300499
TRIM15	0.94571	0.551724	0.914637	0.87027

TMEM223	0.94571	0.551895	0.435015	3.834194
TUBG2	0.94571	0.552035	-0.36868	4.467231
AGBL2	0.94571	0.552085	-0.6517	2.930051
DCTN4	0.94571	0.552122	-1.15512	1.233974
GNA15	0.94571	0.552158	0.422676	3.690227
TPSG1	0.94571	0.552377	-1.12363	1.611449
LOC101926	0.94571	0.552496	-0.61764	2.750994
EXOSC2	0.94571	0.552512	0.609474	2.014432
KANSL3	0.94571	0.552592	0.905404	1.003454
NOP53	0.94571	0.552608	0.264068	8.029124
AFG3L2	0.94571	0.552788	-0.36373	7.578421
SPRR1B	0.94571	0.552904	1.041726	5.744022
TNNI1	0.94571	0.552932	-0.54293	3.780954
MRPL20	0.94571	0.55301	0.356103	4.998543
EEFSEC	0.94571	0.553037	-0.36369	4.126809
SRGAP2-AS	0.94571	0.553203	-0.75784	2.718297
WDR27	0.94571	0.553207	-0.66071	3.105962
MYO9A	0.94571	0.553207	-0.29419	7.814268
PHF12	0.94571	0.553281	-0.31012	5.187084
PPM1A	0.94571	0.553324	-0.51649	4.259576
HLA-DMB	0.94571	0.553419	0.660129	2.434814
ZNF852	0.94571	0.553444	0.339755	4.445891
PAQR3	0.94571	0.553444	0.361389	4.438463
LOC101929	0.94571	0.5535	1.024396	1.068152
TSEN2	0.94571	0.553511	0.704362	2.77078
SETSIP	0.94571	0.553562	-0.62859	2.59958
PFKP	0.94571	0.553634	0.416715	3.184943
SLC22A3	0.94571	0.553662	-0.54791	4.31925
IPO9-AS1	0.94571	0.553745	-0.99076	0.683705
ISM1-AS1	0.94571	0.553785	-1.05198	1.775734
SNORA80A	0.94571	0.553846	-0.49425	3.97656
BET1	0.94571	0.553867	-0.82573	2.146547
GALNT1	0.94571	0.554005	0.283931	7.460592
PIGF	0.94571	0.554022	0.76368	2.214784
FDX2	0.94571	0.554066	0.419678	3.158203
LOC102723	0.94571	0.554122	0.824676	1.78689
MIR3125	0.94571	0.554251	1.122813	0.997712
ZBED2	0.94571	0.554268	-0.96041	2.122298
GNG3	0.94571	0.554272	-1.25607	1.210332
STX3	0.94571	0.554389	1.062069	0.766799
DLG2	0.94571	0.554443	0.738219	2.958877
AKR7L	0.94571	0.554447	0.611837	2.04572
TECPR1	0.94571	0.554467	0.270567	6.121596
NRF1	0.94571	0.554538	0.928442	0.841728
WHAMM	0.94571	0.554607	0.254117	5.865966

ZDHHC3	0.94571	0.554711	0.910372	1.108664
CHMP4C	0.94571	0.554744	0.345824	5.367937
SELP	0.94571	0.554811	-0.70539	3.031081
SECISBP2	0.94579	0.554931	0.963845	1.500473
PCYOX1	0.945905	0.55523	0.267676	7.144862
PTCH2	0.945905	0.555309	-0.37969	7.599732
CTSZ	0.945905	0.555386	-0.28952	7.3939
RIN1	0.945905	0.555405	0.380774	4.460617
SNORD37	0.945905	0.555584	-0.59562	3.149811
TMEM80	0.945905	0.555703	0.569288	1.578459
MRPL53	0.945905	0.555871	0.398399	4.165682
GRAP	0.945905	0.555984	-0.64325	2.240976
RIMS1	0.945905	0.555992	-1.16788	1.172658
GHET1	0.945905	0.556005	0.938689	1.060818
MRPL57	0.945905	0.55602	-0.33868	4.692417
NADSYN1	0.945905	0.556024	0.261012	6.398104
LOC105378	0.945905	0.556144	-0.61264	3.439808
GEM	0.945905	0.556147	0.654846	2.264415
COLGALT2	0.945905	0.556192	1.112013	0.992542
ADGB	0.945905	0.556222	-1.58186	0.99279
TRAM1L1	0.945905	0.556224	-1.00401	1.104051
GLI3	0.946174	0.556454	-0.39668	5.082021
TIMM29	0.946297	0.556796	-0.5948	2.95568
TOR3A	0.946297	0.556801	-0.34694	5.172791
FZD4	0.946297	0.556834	0.311858	5.746913
RFXAP	0.946297	0.556878	0.340453	4.204581
NUCB2	0.946297	0.557029	0.25842	7.096482
PLA2G16	0.946297	0.557202	0.892435	1.612869
TMEM220-	0.946297	0.557404	0.762782	1.714012
NOTCH4	0.946297	0.557439	-1.03863	1.468871
CRIP2	0.946297	0.557441	0.399841	4.223942
TMSB4X	0.946297	0.557474	-0.26887	9.203025
RDH10	0.946297	0.557576	0.273219	6.499906
SCAMP4	0.946297	0.557646	0.264672	5.516189
TENT5A	0.946297	0.557711	-0.32891	7.0096
KANK2	0.946297	0.557732	-0.45483	5.742519
ZBTB2	0.946297	0.557793	0.318775	4.950346
CEP162	0.946297	0.557867	-1.31019	0.923415
MIR1205	0.946297	0.557875	1.030643	1.424323
RARS2	0.946297	0.557876	-0.25865	6.217907
FUT10	0.946297	0.557897	-0.30323	5.299795
ZSWIM1	0.946472	0.558072	-0.33557	4.629116
FLJ42627	0.946488	0.558265	-0.30967	6.015795
SLC35F5	0.946488	0.558268	-0.72468	2.810796
HERC4	0.946488	0.558299	-0.52927	3.803245

BIRC6-AS2	0.946488	0.558428	-0.84805	1.991378
PKIA-AS1	0.946488	0.558526	0.817821	1.968455
SNORA71B	0.946488	0.558645	-0.40846	5.242128
LOC101927	0.946488	0.558674	-0.79108	2.63164
NIPA1	0.946488	0.558676	-0.97662	1.442907
PKNOX2	0.946488	0.558772	-0.89988	2.147487
CPNE8	0.946488	0.558803	0.323612	4.978654
DPH1	0.946571	0.558924	0.353746	4.135112
ZNF706	0.946772	0.559177	-0.84763	1.601914
HINT3	0.946772	0.559223	0.272048	6.407579
LOC100128	0.946772	0.559388	0.386983	4.052871
MAPKAPK5	0.946772	0.559529	0.333718	4.133334
GTF2A1	0.946772	0.559551	0.97371	0.764011
NFYC	0.946772	0.559551	-0.66735	2.864218
HRNR	0.946772	0.559624	-0.45011	3.731752
SMARCA5	0.946772	0.559627	-0.25756	8.086678
HOXB5	0.946772	0.55978	-0.47755	4.643124
TTLL12	0.946772	0.559895	0.324672	6.581413
ERBIN	0.946772	0.559907	-0.69175	2.290508
LOC102724	0.946772	0.56017	0.508867	3.399055
MXRA8	0.946772	0.560189	-1.59482	0.632956
C8orf44	0.946772	0.560193	-0.58767	3.206387
C19orf38	0.946772	0.560204	0.807236	1.491164
CBLB	0.946772	0.560278	-0.27177	6.887817
C9orf139	0.946772	0.560361	-1.03543	1.606756
SEC22C	0.946772	0.560387	0.278531	5.709294
NAP1L3	0.946772	0.560414	-0.74345	2.679586
RPL36A	0.946791	0.560555	-0.5939	3.607586
MFSD6	0.946791	0.56057	0.238878	7.942562
MTHFR	0.946918	0.560717	0.255971	6.091143
WASH5P	0.947063	0.560914	-0.68023	2.69555
TMEM14EP	0.947063	0.560947	-0.33146	5.08668
FAM45BP	0.947079	0.561029	0.583534	2.509103
NET1	0.947334	0.561305	-1.07324	1.997167
USP47	0.947334	0.561324	-0.98771	0.689778
PLCB4	0.947518	0.561513	0.805907	1.467906
LINC01225	0.947518	0.561632	-1.24938	2.30503
CHPF	0.947518	0.56165	-0.56808	3.464602
RAD51B	0.947635	0.561792	0.486907	2.983517
XKR6	0.947726	0.562146	-0.58815	2.87337
GRK6	0.947726	0.562165	0.867037	0.865695
MKNK2	0.947726	0.562187	0.254261	6.1217
DOK7	0.947726	0.562188	-0.9696	1.21434
SREK1IP1	0.947726	0.562217	-0.28133	5.873508
HIPK3	0.947726	0.562279	-0.5567	3.120699

TGFB3	0.948161	0.562678	0.29209	5.591633
ZNF587B	0.948161	0.562682	-0.28364	6.240398
IL36A	0.948291	0.562873	0.949024	3.802709
CD3G	0.948291	0.562934	-0.79249	2.274699
FMO4	0.948291	0.562975	0.524739	4.086781
MRPS10	0.94834	0.563125	-0.29963	5.253842
LOC65365З	0.94834	0.56322	-0.62834	3.324939
FAM155B	0.94834	0.563287	0.976329	1.498636
STAG3L3	0.94834	0.5633	0.332626	4.273128
ZNF684	0.94834	0.56338	-0.59071	3.479252
NUDCD3	0.94834	0.563438	-0.26657	7.092841
STX18-AS1	0.948423	0.563916	0.589675	2.181205
SEC31B	0.948423	0.563944	0.26437	5.598516
WDR60	0.948423	0.564006	-0.26079	6.933765
FOXC2	0.948423	0.564015	0.819272	1.375717
HRAT5	0.948423	0.564043	-0.96174	1.34791
ARHGEF5	0.948423	0.564121	0.296271	6.230883
FCRL3	0.948423	0.564174	-0.78946	2.182164
CCDC90B	0.948423	0.564257	0.791278	1.660018
RHOG	0.948423	0.564286	-0.35697	4.750364
LINC01554	0.948423	0.564302	0.93671	1.340594
DNMBP	0.948423	0.564348	0.254074	7.043542
LOC100505	0.948423	0.564401	0.46638	3.38603
PARGP1	0.948423	0.564427	-0.32442	5.229953
ESPNP	0.948473	0.564529	-1.04276	2.470414
TVP23A	0.948546	0.564696	0.516212	2.421556
ERG28	0.948546	0.564792	0.319654	4.400661
RNF111	0.948546	0.564841	0.494405	2.417206
NUP214	0.948546	0.56491	-0.26316	7.393364
VARS	0.948546	0.564978	-0.7707	2.700971
COL19A1	0.948546	0.565135	-1.21075	2.932519
PHF23	0.948546	0.565202	-0.93337	1.200087
UNC50	0.948546	0.565267	-0.30643	5.289567
H2AFY	0.948546	0.565272	0.848148	1.246327
PABPC1	0.948546	0.565309	0.271961	10.84716
PPP1R9B	0.948546	0.565414	-0.25639	7.339976
HYDIN2	0.948546	0.565542	-0.52563	4.270127
B3GALT2	0.948546	0.565645	-0.42752	3.806566
PDE12	0.948546	0.565709	-0.31425	5.526251
PHACTR2-A	0.948546	0.565869	0.511711	3.360735
PDZK1IP1	0.948546	0.566057	0.397768	5.134897
PPM1L	0.948546	0.566153	-0.67144	2.786049
ABT1	0.948546	0.566163	-0.44993	4.220426
PIKFYVE	0.948546	0.566326	-0.25385	7.138217
TBL2	0.948546	0.566348	0.294755	5.784138

NAV2	0.948546	0.566469	0.579397	2.428972
C1QTNF12	0.948546	0.566658	-0.93372	1.494459
DKFZP586I	0.948546	0.566739	0.29367	5.313026
SARS	0.948546	0.566866	0.238074	6.9785
LINC01105	0.948546	0.566887	0.850064	1.47723
UROS	0.948546	0.56696	0.31357	4.388632
TANGO6	0.948546	0.567068	-0.32447	4.985477
TSSK3	0.948546	0.567081	0.451871	3.516577
CDC42SE2	0.948546	0.567086	0.7578	1.464519
THAP8	0.948546	0.567151	-0.44782	3.492513
RRS1-AS1	0.948546	0.567385	-0.88605	2.122554
RAPH1	0.948546	0.567675	-0.25382	7.221083
RNF157-AS	0.948546	0.567792	-1.56271	1.01046
VPS36	0.948546	0.567825	-0.72673	2.049271
SNORA28	0.948546	0.56784	-0.54742	4.203504
SHLD2P3	0.948546	0.568001	-0.60173	2.957231
SLC25A4	0.948546	0.568112	-0.40557	4.907367
LCP1	0.948546	0.568207	-0.35048	6.930572
LYVE1	0.948546	0.568293	0.473418	4.002149
SLC2A10	0.948546	0.568454	-0.29131	5.706868
MIEN1	0.948546	0.568627	-0.33187	4.48517
P3H4	0.948546	0.568643	-0.42869	4.455693
YTHDF3	0.948546	0.568704	1.016688	0.587182
ITM2B	0.948546	0.568871	0.235494	8.598939
GJB3	0.948546	0.568883	0.73387	1.675863
LOC286437	0.948546	0.569041	0.373616	3.932849
LOC100505	0.948546	0.569085	-0.54725	3.180529
MTRNR2L1	0.948546	0.569096	0.605438	1.760283
ATP11B	0.948546	0.569115	0.228529	7.96867
LIG1	0.948546	0.569118	0.443656	2.931366
CCL21	0.948546	0.569139	0.714218	2.285389
ANGPTL2	0.948546	0.569179	0.299063	6.323819
DHX40	0.948546	0.569188	-1.1381	0.912881
RSPH3	0.948546	0.569281	0.289989	5.213771
CAPRIN1	0.948546	0.569332	-0.30696	6.534734
LOC101928	0.948546	0.56946	-1.09307	0.968365
SNORD14D	0.948546	0.569494	-0.3788	4.797885
NDUFS3	0.948546	0.569517	0.335292	4.770447
ZBTB11-AS	0.948546	0.569553	-0.81553	2.438484
LOC100506	0.948546	0.569588	-1.03302	1.480409
FAM83G	0.948546	0.569617	0.345894	5.173558
NRBP2	0.948546	0.569638	-0.27222	6.206845
C15orf65	0.948546	0.569693	0.836606	1.174311
THBD	0.948546	0.56974	-0.34629	5.179516
FAM228B	0.948546	0.56976	-0.77259	2.378501

FAM151B	0.948546	0.569819	-1.09378	1.579244
TBL1XR1	0.948546	0.569826	-0.25097	8.63667
PERP	0.948546	0.569844	-0.41941	8.947867
ETV5	0.948546	0.569914	-0.31966	5.783795
RRP7BP	0.948546	0.569927	-0.32078	5.386561
ST7L	0.948546	0.570082	0.825393	0.843391
ACACA	0.948546	0.570107	-1.38023	0.819514
HIST1H2AI	0.948546	0.57023	-0.48352	3.893993
C1QB	0.948546	0.570271	-0.37299	6.556144
RNF32	0.948546	0.570296	-0.68429	2.227244
FLT4	0.948546	0.570331	0.683627	1.830603
DYNLL2	0.948546	0.570373	0.250419	5.984472
MIOX	0.948546	0.570378	-1.25012	1.67095
SMA5	0.948546	0.570394	-0.69992	2.177648
LOC100131	0.948546	0.570402	-0.74537	3.074238
TSR3	0.948546	0.570429	-0.36283	5.054409
CPD	0.948703	0.570596	0.352392	5.039991
SMO	0.948843	0.570752	-0.46525	4.187104
NBEA	0.949161	0.571094	-0.39901	6.511618
LOC100289	0.949161	0.571224	-1.31912	0.588219
SNORA79	0.949161	0.57129	-0.81957	2.120817
LOC148696	0.949161	0.571313	0.250059	6.59759
PUSL1	0.949161	0.571423	-0.52585	3.295914
CALM1	0.949161	0.571432	0.242297	8.958947
FRMD3	0.949161	0.5715	-1.18116	1.759649
CELF2-AS1	0.949161	0.571624	0.447227	4.555243
LOC100126	0.949161	0.571653	-0.59525	3.388268
CAPN10-DT	0.949161	0.571675	-0.59321	3.785843
PPFIA1	0.949161	0.57177	-0.93451	1.521297
PHC1	0.949161	0.57183	-0.36435	5.335709
PIP4K2B	0.949161	0.571968	0.318046	5.679561
SENCR	0.949161	0.572139	0.683022	1.796491
ZCCHC3	0.949161	0.572204	-0.36614	5.121451
ITPKA	0.949161	0.572246	0.52832	3.539647
TET1	0.949161	0.572282	0.305519	5.219099
RHOBTB2	0.949161	0.572332	-1.06046	1.453592
TBC1D12	0.949161	0.572454	-0.26533	6.053188
GALNT17	0.949161	0.572462	-0.50458	3.960673
IFNK	0.949161	0.572487	0.542375	3.317459
PDE6D	0.949161	0.572566	-0.78294	2.391632
TCEA3	0.949161	0.572608	0.297873	7.10967
ST3GAL2	0.949204	0.573063	-0.34379	5.013257
A2MP1	0.949204	0.573128	-0.70073	2.023999
XPA	0.949204	0.573168	0.730454	1.912404
FBXL13	0.949204	0.573169	0.672889	1.820169

BAIAP2	0.949204	0.5732	-0.58559	3.409586
PHF8	0.949204	0.573523	0.659841	2.245285
NRDE2	0.949204	0.573584	0.262515	5.408456
RERE-AS1	0.949204	0.573614	-0.5705	2.510768
GORAB	0.949204	0.573615	0.842842	1.175843
NPB	0.949204	0.573661	0.942897	0.752192
ESD	0.949204	0.573678	-0.36997	4.781934
LOC101929	0.949204	0.573768	1.02972	0.618573
SNORD6	0.949204	0.573777	-0.86825	1.790876
AGO1	0.949204	0.573897	-1.16016	1.687917
MAGOH2P	0.949204	0.573901	-0.87384	1.79928
GJB7	0.949204	0.574015	-0.87666	2.259518
NLRP9	0.949204	0.57406	-0.82979	2.45981
PTGES2-AS	0.949204	0.57413	0.719039	2.653796
ZZZ3	0.949204	0.574208	-0.26754	5.863888
RABEP2	0.949204	0.574294	0.302337	5.413573
CABCOCO1	0.949204	0.574404	-0.98904	1.410224
CTSF	0.949204	0.574445	-0.49514	4.211169
AGK	0.949204	0.574463	-0.35814	4.581211
EIF3H	0.949204	0.574543	-0.29562	7.474804
ISG20L2	0.949204	0.57455	-0.28152	5.942746
ZC3H6	0.949204	0.574582	-0.3042	6.366506
PRKCA-AS1	0.949204	0.574608	0.349283	3.907658
LINC00654	0.949204	0.574708	-0.8127	3.054971
KIZ-AS1	0.949204	0.574769	-0.63742	3.130472
CENPBD1P	0.949204	0.574804	-0.39843	4.293027
LINC00674	0.949421	0.575123	-0.25532	7.557751
TIMP3	0.949421	0.575193	-0.28886	8.755499
IQCJ-SCHIP	0.949421	0.575228	-0.90356	1.975499
MECOM	0.949421	0.575399	0.32173	5.857811
LOC101928	0.949421	0.575402	-1.35992	1.068042
JHY	0.949421	0.575497	-0.81067	2.804301
TC2N	0.949421	0.5755	-0.97102	1.468105
COPG1	0.949421	0.575514	0.2469	8.031638
DDI2	0.949455	0.575668	-0.30182	5.522379
CALML6	0.949455	0.575789	0.799881	0.923034
TMEM241	0.949455	0.575812	0.345841	4.402702
SELENOO	0.949455	0.575824	-0.34035	4.848121
WWP2	0.949668	0.57612	0.365453	3.756449
TRIP13	0.949668	0.576159	-0.53286	3.165834
ZYG11B	0.949668	0.576233	-0.25537	6.285565
TFB1M	0.949668	0.576323	0.350255	4.482768
PIK3IP1-AS	0.949668	0.576353	0.716283	1.510549
LOC100130	0.949668	0.576446	0.862233	1.152101
PLBD1-AS1	0.949668	0.576559	0.572133	2.246555

AATF	0.949668	0.57662	-0.62134	3.090501
MMADHC	0.949668	0.5767	-0.29081	5.658742
CLIP3	0.949668	0.576722	-0.90485	1.194189
SIAH1	0.949668	0.576864	0.410484	2.754735
RPLP0P2	0.949668	0.576953	-1.11997	2.133233
UBE2E3	0.949668	0.576958	-0.69672	2.223065
SETD2	0.949668	0.576997	-0.25015	8.78648
SMTNL1	0.949668	0.577044	0.622263	1.392247
DVL1	0.949668	0.577191	0.265818	6.361129
PPP1R3G	0.949668	0.577215	0.839102	1.215163
GNAL	0.949668	0.577256	-1.15099	0.793026
MYO1D	0.949815	0.577478	-0.26048	6.553363
LOC101927	0.949815	0.577491	0.631981	2.217709
ADCY6	0.94984	0.577595	0.236356	7.157629
TRIO	0.94984	0.577817	-0.64356	2.644408
PLEKHF2	0.94984	0.57784	-0.31781	5.522111
LIMS1	0.94984	0.577858	-0.30346	4.850014
CASP4	0.94984	0.577868	-1.28843	0.966727
SUPT16H	0.950149	0.578239	-0.26396	7.972175
LOC101928	0.950149	0.578254	-0.91348	1.6542
OPA1-AS1	0.950149	0.578361	0.719438	2.185653
PEX13	0.950149	0.578456	-0.26793	6.216384
GPR141	0.950149	0.5785	-1.18449	0.727915
MIR6758	0.950149	0.578591	0.645201	1.826465
3-Sep	0.950149	0.578699	0.859409	1.600114
MIR5194	0.950149	0.578728	-0.94668	2.113133
FBXW4	0.950149	0.578731	-0.31335	6.494733
RNU2-1	0.950149	0.578823	-0.57563	3.292211
PPIL3	0.950149	0.578866	-0.65083	2.685455
LOC389247	0.950149	0.578925	0.694422	2.086969
SOS1	0.950257	0.579063	0.228376	7.590897
TMEM167A	0.950348	0.579247	0.227957	7.552177
KIF5C	0.950348	0.579386	0.858925	0.844401
PNN	0.950348	0.579389	0.230823	9.595553
LSM3	0.950348	0.579447	-0.32015	5.536262
ECHDC3	0.950348	0.579481	0.747109	1.643891
LOC101928	0.950514	0.579769	-1.09503	2.028773
IRF9	0.950514	0.579769	-0.30521	6.138715
SH2D1B	0.950514	0.579799	0.705474	2.351568
ANGPTL6	0.950545	0.579997	0.85043	0.9052
PGAM1	0.950545	0.580031	-0.67694	2.309744
NFRKB	0.950545	0.580089	0.599327	2.23845
UGT1A4	0.950545	0.580227	0.863609	1.017713
SAFB2	0.950545	0.580246	-0.25771	8.030067
SLC8B1	0.950545	0.580253	-0.30617	5.627656

PCDH1	0.950564	0.580368	0.322843	4.995523
CCL24	0.950564	0.580444	0.781965	1.205691
XXYLT1	0.950564	0.580482	-0.97802	0.762601
LOC101928	0.950625	0.580591	1.03419	0.922679
MAP3K14	0.950887	0.58085	-0.36932	5.668496
BMPR1B	0.950887	0.580896	-1.12014	1.033996
MIR429	0.950913	0.581084	0.642642	1.896851
SMYD2	0.950913	0.58115	-0.34028	4.695246
ALG10	0.950913	0.581344	-0.77737	3.371302
CD9	0.950913	0.581504	-0.27913	8.166778
LINC00524	0.950913	0.581558	0.975209	0.889102
YES1	0.950913	0.581651	-0.34425	7.653716
ZNF335	0.950913	0.581718	-0.26138	6.118075
PABPC3	0.950913	0.581752	0.487723	2.2618
LOC100129	0.950913	0.581808	-0.66736	4.194346
TMEM126B	0.950913	0.581888	-1.00026	1.181883
EXOC3-AS1	0.950913	0.58203	0.742111	1.00231
CDK4	0.950913	0.582088	0.324639	5.362476
PANX1	0.950913	0.582336	-0.35443	5.351745
UBA5	0.950913	0.58243	-0.36236	4.325479
C8orf37	0.950913	0.582492	-0.63204	2.496121
LRRFIP2	0.950913	0.582524	0.315123	4.468018
DSEL	0.950913	0.582574	0.378807	4.69707
FRG1JP	0.950913	0.582612	-0.62576	2.597446
USP49	0.950913	0.582614	-0.3274	4.514237
PGM2	0.950913	0.582703	0.29923	5.243446
ARHGAP10	0.950913	0.582782	-0.33018	6.864865
PTN	0.950913	0.582901	-0.67895	3.47504
UBXN4	0.950913	0.582922	-0.23242	8.587404
AASS	0.950913	0.582994	-0.34813	6.598992
KIF26A	0.950913	0.583071	-0.78917	3.159026
ANKS1B	0.950913	0.583113	-0.63208	2.929315
LINC01697	0.950913	0.583143	0.702414	1.82951
RNASEH2B	0.950913	0.583146	-0.64454	2.45258
ZNF527	0.950913	0.583191	0.356858	4.226968
STRBP	0.950913	0.583286	0.839104	1.095549
DOC2B	0.950913	0.583354	0.552943	3.102712
TULP2	0.950913	0.583397	-1.09102	1.373785
LRRC45	0.950913	0.583404	-0.37396	5.101835
SNORD116	0.950913	0.583422	-0.75822	5.052021
SLC5A1	0.950913	0.583513	0.657152	1.918235
MFSD8	0.950913	0.58362	0.248064	5.522346
SIX1	0.950913	0.583621	-0.80846	2.884746
TJP2	0.950913	0.583667	0.289779	5.259708
RPL15	0.95093	0.583749	0.666903	1.953086

NES	0.951486	0.584203	0.28152	6.667906
DCTN1-AS1	0.951486	0.584303	0.656694	2.012028
LOC10050€	0.951486	0.584308	0.87195	1.39003
C2orf42	0.951527	0.584406	-0.5024	3.915063
RECQL4	0.951614	0.584532	-0.33368	4.634386
ZNF137P	0.951972	0.585138	0.506375	2.801702
PRR34	0.951972	0.585445	-0.5974	3.588621
FOSB	0.951972	0.58556	-0.53358	2.448066
CLDN20	0.951972	0.585568	0.368924	3.375638
CD3E	0.951972	0.585569	-0.42242	4.203464
RSL1D1	0.951972	0.585659	-0.25242	7.754648
TUG1	0.951972	0.585748	-0.72903	2.912345
LRP3	0.951972	0.585836	-0.5476	3.665018
FAM72B	0.951972	0.586136	-0.8915	1.466202
TIMM8B	0.951972	0.586179	0.787633	1.02192
UBE2G2	0.951972	0.586209	-1.23155	0.949131
DPY19L2P4	0.951972	0.586243	-0.79078	2.151191
DIAPH2	0.951972	0.586248	0.487626	3.305706
LINC00858	0.951972	0.586248	0.611833	2.166926
SLC2A1-AS	0.951972	0.58635	-1.0805	1.51513
HDAC3	0.951972	0.586379	-0.32043	5.410545
ZFYVE26	0.951972	0.586424	0.247098	6.623469
MIR3936H(	0.951972	0.586438	0.425958	2.783297
EIF2B1	0.951972	0.586447	-0.27575	5.493582
NIPSNAP3/	0.951972	0.586449	-0.41631	4.133652
HIST1H3A	0.951972	0.586454	0.510647	2.636674
SOX17	0.951972	0.58682	0.707499	1.338283
MEOX2	0.951972	0.587003	0.572977	3.031159
NEK2	0.951972	0.587072	-0.97269	2.673343
GOSR2	0.951972	0.587326	-0.27339	5.855739
MTMR1	0.951972	0.587519	-0.50993	3.782224
NOP16	0.951972	0.587527	0.665475	1.924071
LOC72809!	0.951972	0.587652	0.759836	1.141998
MIR4256	0.951972	0.587729	-1.44943	0.665389
TRPM2-AS	0.951972	0.587888	0.507426	2.474708
CDK7	0.951972	0.587897	0.68147	2.05911
XYLT1	0.951972	0.587936	0.225996	6.62467
FOSL2	0.951972	0.588024	0.252729	9.151204
UBE2L3	0.951972	0.588047	-0.69712	2.41943
LINC00987	0.951972	0.588094	0.255526	5.232793
HTR7P1	0.951972	0.588128	-0.64393	2.20683
KDM4A	0.951972	0.588222	0.241035	6.898576
HNRNPA1	0.951972	0.588344	0.36739	3.968262
DHX29	0.951972	0.588355	-0.24883	6.592173
ATP2B4	0.951972	0.588392	-0.9162	1.691633

LOC73018З	0.951972	0.588487	0.541779	2.60543
SPRR2A	0.951972	0.588601	0.948996	6.004432
MAST2	0.951972	0.588637	0.221796	7.36336
ATP11A-AS	0.951972	0.58873	0.33433	4.190577
CCAT2	0.951972	0.588917	0.811403	1.428797
MIR4296	0.951972	0.588939	0.642467	2.105925
GKAP1	0.951972	0.588994	0.82783	1.399429
GMDS-DT	0.951972	0.589028	0.440297	3.574877
SRGAP2B	0.951972	0.589033	-0.47937	4.782293
MYSM1	0.951972	0.589045	-0.23731	7.617168
ASAP1-IT2	0.951972	0.589134	-0.56162	2.724739
MAN1A2	0.951972	0.589349	0.230545	8.16916
SRSF2	0.951972	0.589422	0.280438	4.692375
WDR41	0.951972	0.589439	0.234995	5.948343
ZNF708	0.951972	0.589452	0.887395	1.005325
SNORA69	0.951972	0.589508	0.457027	3.129474
MAT2A	0.951972	0.589603	-0.24425	7.911349
PTBP2	0.951972	0.589607	0.66005	2.975735
NELFA	0.951972	0.589665	0.296505	4.642096
ERGIC1	0.951972	0.589705	0.246582	7.870807
CCDC200	0.951972	0.589747	-0.45634	3.617216
TMEM33	0.951972	0.589751	-0.26244	6.691421
MIR4802	0.951972	0.589854	0.868988	0.982549
TM9SF4	0.951972	0.589876	-0.24502	7.102936
ZNF275	0.951972	0.589976	-0.2678	5.592283
DDX3X	0.951972	0.589978	-0.38169	4.696152
NUMA1	0.951972	0.590104	0.899729	0.660006
AKAP11	0.951972	0.590107	-0.25935	8.012275
RNF25	0.951972	0.590161	0.372785	3.780113
SRSF8	0.951972	0.590257	-0.53394	2.920838
LOC101927	0.951972	0.59036	-1.20187	1.326539
INTS6L	0.951972	0.590372	0.306295	4.830618
P2RY1	0.951972	0.590495	-0.69073	3.414621
GAR1	0.951972	0.59064	0.763488	1.518427
BOK	0.951972	0.5907	0.270243	5.014412
DHRS13	0.951972	0.590831	-0.49986	2.85598
EPHX4	0.951972	0.590888	-1.22638	1.45154
EMD	0.951972	0.59091	-0.33938	4.636123
DSCR9	0.951972	0.590934	-0.83079	1.646187
PDCD4-AS1	0.951972	0.591054	0.569357	2.226208
SNX27	0.951972	0.591112	-0.23689	7.382345
NDUFA8	0.951972	0.591148	-0.42544	4.240374
LOC103611	0.951972	0.591176	0.362609	4.400554
MGC27345	0.951972	0.591494	-0.27826	5.402238
LINC00900	0.951972	0.5915	0.704176	1.049693

RASSF4	0.951972	0.591628	0.300919	5.628784
LINC00863	0.951972	0.591756	-0.77024	2.25754
ZC3H13	0.951972	0.591855	-0.25492	9.131775
CDSN	0.951972	0.591903	-0.95333	2.988736
TRABD	0.951972	0.591911	-0.3307	5.974212
KRT3	0.951972	0.591959	0.845511	1.283858
ATP6V1A	0.951972	0.591965	0.250377	6.901384
DMD	0.951972	0.592048	-0.87673	2.881177
ADAM6	0.951972	0.59206	1.144545	1.57723
SREBF2	0.951972	0.59207	0.330129	4.584142
BRMS1L	0.951972	0.592115	-0.33487	4.620429
TMED6	0.951972	0.592128	0.587337	1.978703
PADI1	0.951972	0.592159	0.82936	4.756937
PAK3	0.951972	0.592179	-0.9588	0.951169
BCAR3	0.951972	0.592325	0.488047	2.241971
MVD	0.951972	0.592378	0.330003	4.522771
SMARCD3	0.951972	0.592422	0.539244	2.241719
ACTR8	0.951972	0.592535	0.28035	5.25855
CD27-AS1	0.951972	0.592592	-0.40627	3.70356
NRIP2	0.951972	0.59267	-0.33861	4.882681
CSNK2A1	0.951972	0.592799	-1.06458	1.097429
HGF	0.951972	0.592861	-0.90568	1.536392
B3GNT4	0.951972	0.592968	0.679592	1.521087
LINC00466	0.951972	0.592993	0.769851	1.83307
SRCAP	0.951972	0.593	-0.24708	9.037114
GJA4	0.951972	0.593028	0.64519	2.315296
HIST1H4H	0.951972	0.59307	-0.50578	4.308599
TACR2	0.951972	0.593185	-0.79572	3.496449
BLOC1S2	0.951972	0.593207	-0.71844	2.363585
HIST1H3E	0.951972	0.593288	0.582547	2.024829
ZFYVE28	0.951972	0.593315	-0.95281	1.235216
MIR10A	0.951972	0.593373	-1.12652	0.974432
APOBR	0.951972	0.593386	0.298283	5.345546
THSD7A	0.951972	0.593386	-0.34371	4.722068
NRIP1	0.95201	0.593483	0.245264	7.29132
PPP4R2	0.952119	0.593623	-1.00394	1.067423
MORF4L2-/	0.952349	0.593995	-0.82841	1.560241
SNORA24	0.952349	0.594039	0.330756	7.199782
GPR174	0.952349	0.594385	0.667036	1.857236
PPIF	0.952349	0.594397	0.273326	5.805159
CHAC2	0.952349	0.594421	-0.62414	3.147312
UBXN2B	0.952349	0.594451	-0.2583	5.97232
LCP2	0.952349	0.594462	-0.31633	5.915995
SNPH	0.952349	0.594516	0.290464	4.176103
ZNF214	0.952349	0.594619	-0.68146	2.674491

TEX41	0.952349	0.594689	-0.89411	1.320229
MAP6D1	0.952349	0.594768	-0.88546	1.094871
COL24A1	0.952349	0.594778	-0.51183	3.88243
PRNCR1	0.952349	0.594848	0.237986	5.880423
CDKN2AIPI	0.952349	0.594849	-0.54418	3.310729
SERTAD2	0.952349	0.594913	-0.291	6.422395
TXNDC9	0.952349	0.59503	-0.33554	4.955892
PCDHB18P	0.952349	0.595354	-0.68044	2.893427
CPSF3	0.952349	0.595381	-0.29897	5.752943
GNPTG	0.952349	0.59539	-0.32507	5.357643
FAM157A	0.952349	0.595391	-0.69794	2.17583
CACUL1	0.952349	0.595538	-0.23633	7.153776
DNAJA4	0.952349	0.59562	0.592867	2.12137
CLBA1	0.952349	0.595821	0.387345	3.248841
RAB1B	0.952349	0.595836	0.226137	7.068255
IL22RA1	0.952349	0.595839	0.407149	4.361124
ZNF365	0.952349	0.595895	0.728857	2.671592
RAD54L	0.952349	0.595962	-0.80698	0.971828
ZNF512B	0.952349	0.596042	-0.28195	5.255884
LAMB2P1	0.952349	0.596058	0.783193	1.767175
EIF5AL1	0.952349	0.596154	-0.51827	4.063965
DST	0.952349	0.596236	-0.30524	7.063706
MON1B	0.952349	0.596248	0.400496	3.039453
HSPA9	0.952349	0.59627	-0.23608	8.390151
MIR640	0.952349	0.596403	-0.9546	0.668321
TTF1	0.952349	0.596425	0.351202	4.170563
FAM84B	0.952349	0.596433	0.254808	6.753289
CYCS	0.952349	0.596484	0.238704	7.643264
HBP1	0.952349	0.596525	0.762433	0.979618
TEAD3	0.952424	0.596681	-0.2506	6.524915
STK17B	0.952424	0.596739	0.24075	7.013684
PNPLA3	0.952424	0.59679	-0.93785	2.601842
ZNF175	0.952903	0.597184	-0.63089	3.602688
RTN4	0.952903	0.597304	0.300643	4.131675
FGD6	0.952903	0.597437	-0.24556	7.155151
MIR181A2I	0.952903	0.597467	-0.43334	3.413562
RPS5	0.952903	0.597472	-0.2659	7.579547
FKBP5	0.952903	0.597525	-0.32177	6.655906
MTHFD1	0.953028	0.597723	0.228894	6.113795
MZF1-AS1	0.953028	0.597749	0.291618	4.874596
C11orf80	0.953269	0.597973	-0.95568	1.098072
EIF5	0.953597	0.598252	0.413861	3.139853
RPS6KB2	0.953648	0.59843	0.255376	5.740861
ATXN7L2	0.953648	0.598552	0.400519	2.977735
WWP1	0.953648	0.598611	-0.23119	7.299549

SUPT3H	0.953648	0.598916	-1.04639	1.017466
MFAP3	0.953648	0.598922	0.548746	2.387585
PNMA8B	0.953648	0.598942	0.569434	2.236876
MYH16	0.953648	0.599033	-0.80988	1.769578
ZNF574	0.953648	0.599107	-0.33122	4.599906
NDNF	0.953648	0.599155	0.425734	4.996807
TMEM88	0.953648	0.599158	-0.98423	1.313809
HLA-J	0.953648	0.59919	0.452926	2.912362
ST8SIA3	0.953648	0.599233	0.796753	2.577996
LOC10144ℇ	0.953648	0.599235	-0.90071	0.855291
BLID	0.953648	0.599301	-0.89215	2.077599
ADGRF4	0.953795	0.599504	0.746817	2.271177
TK2	0.953795	0.599712	0.537135	2.534297
CRYBG2	0.953795	0.59973	-0.59074	6.215664
SCART1	0.953795	0.599779	0.356288	4.738191
GSTM4	0.953795	0.599808	0.684413	1.828451
SNORD83A	0.953795	0.599829	0.413193	2.876667
ARAP1	0.953827	0.599939	-0.61545	2.77232
LOC10024(	0.953827	0.599995	0.760016	1.599139
PIK3CB	0.953857	0.600189	-0.29982	5.558706
PARVA	0.953857	0.600231	-0.27042	7.475211
MAP3K2	0.953857	0.600232	-0.23969	8.069238
PRPF8	0.953912	0.600339	0.25929	6.553384
PDSS1	0.954256	0.600661	0.356069	4.581627
CFAP73	0.954256	0.600768	-1.21326	1.042395
NCKAP1L	0.954256	0.600856	-1.16454	1.131444
SPDYE7P	0.954256	0.60101	0.351034	3.236154
C4orf47	0.954256	0.601042	-1.11005	1.71767
HIST1H4F	0.954256	0.601057	0.348686	4.628502
LRRC56	0.954256	0.601065	-1.22539	0.762951
ST7-OT3	0.954275	0.601187	-0.93735	1.197344
IFNE	0.954275	0.601222	-1.38885	0.921964
ABHD3	0.954433	0.60145	0.524532	2.483697
SLC35A4	0.954433	0.601467	-0.29079	6.007449
PRKX-AS1	0.954667	0.601688	-1.05646	0.634604
SEC63	0.954755	0.601816	-0.24406	7.997954
MGAM2	0.954818	0.602015	0.58586	3.891468
ADAR	0.954818	0.602059	-1.09211	1.22085
UHRF1BP1	0.954818	0.602131	-0.2441	5.792585
CYP20A1	0.954818	0.602147	0.217864	6.71751
SARAF	0.955003	0.602433	-0.74165	2.216401
BYSL	0.955003	0.60244	-0.35138	4.177505
ZFC3H1	0.955003	0.602482	-0.24202	8.474742
ZNF346	0.955068	0.602629	0.713755	1.456331
LINC00476	0.955068	0.602691	0.336428	4.471628

CDK9	0.955068	0.602811	-0.26206	6.084767
GEMIN4	0.955068	0.602946	-0.3666	4.745705
PRKD2	0.955068	0.602966	0.490812	2.044382
LINC01948	0.955068	0.603063	0.889833	1.148538
AFG1L	0.955068	0.60308	-0.39786	3.665244
EEF1E1	0.955068	0.603105	-0.7779	2.079732
APOBEC3F	0.955072	0.603313	-0.40549	3.244733
HUWE1	0.955072	0.603359	-0.22424	9.832143
MRPS11	0.955072	0.603477	0.733471	0.778779
FAM133B	0.955072	0.603664	-1.15227	0.609894
ATL2	0.955072	0.603665	-0.72019	2.197776
ZNF439	0.955072	0.603723	-0.52867	3.691277
DPY19L2	0.955072	0.603768	-0.5552	3.300079
TEFM	0.955072	0.603792	-0.48917	3.81729
MIR4755	0.955072	0.603833	0.542428	2.138234
METTL18	0.955072	0.603836	-0.47327	2.850218
LOC100129	0.955126	0.603981	0.31244	4.069345
MTAP	0.955126	0.604015	0.290426	5.73901
MISP	0.95583	0.604533	0.269143	8.045711
MTDH	0.955863	0.604703	-0.25024	8.540358
MFSD14A	0.955863	0.604818	-0.26751	5.953869
GHR	0.955863	0.604855	-0.89556	1.756758
ARL8B	0.955863	0.60494	-0.25471	6.681346
MICALL2	0.955863	0.604985	0.247683	6.947716
SLC10A6	0.955863	0.604991	0.732921	1.701833
MRPL37	0.955977	0.605193	-0.28674	5.440273
NAP1L2	0.955977	0.605209	0.597928	2.435189
OCEL1	0.956221	0.605437	0.425845	2.649045
PPFIBP2	0.956224	0.605512	0.36731	4.246232
FAM111A	0.956692	0.605938	-0.66976	2.960021
MTMR12	0.956692	0.605954	0.657498	1.41694
LOC100129	0.95679	0.606148	0.870006	1.181354
MKLN1-AS	0.95679	0.606162	-0.66531	2.848314
HTT-AS	0.956924	0.606363	0.732499	1.266941
SCCPDH	0.956924	0.606461	-0.25671	5.461623
ACTRT3	0.956924	0.606585	0.494938	1.606867
DNAH8	0.956924	0.60659	0.608279	2.13324
RBM20	0.956924	0.60666	-0.71177	3.69954
LOC642361	0.956924	0.606684	0.589916	1.474336
CDC42EP3	0.957415	0.607068	-0.90486	1.175797
STK4	0.957461	0.60718	-0.22298	7.756703
LOC101928	0.957461	0.607243	0.514862	3.761419
ALG1L9P	0.957942	0.607621	-1.1589	1.551535
CMC2	0.958093	0.607857	-0.49738	3.416178
ALG12	0.958093	0.607864	0.273423	4.432614

IVNS1ABP	0.95813	0.60796	-0.22622	8.154538
CAPZA1	0.958317	0.608165	0.223031	7.392403
SPTLC3	0.958317	0.608225	0.343224	4.148509
CASK	0.958366	0.608335	0.738574	0.670608
LOC64284€	0.958366	0.608442	0.274519	4.431081
AGPAT5	0.958366	0.608535	-0.28325	5.98403
LOC14366€	0.958366	0.608548	-0.96537	1.601427
TMEM59L	0.958372	0.608746	-0.83039	1.657979
PPBPP2	0.958372	0.608804	-1.60189	1.045996
SMAD5-AS	0.958372	0.608876	0.627156	1.449949
FANCF	0.958372	0.6089	0.312646	4.21574
LINC01389	0.958372	0.608917	0.641333	1.832246
FOXJ3	0.958643	0.609257	-0.81747	2.348957
MIIP	0.958643	0.609279	-0.38339	3.639842
ADCY10P1	0.958643	0.609308	-0.49037	3.987295
INTS6-AS1	0.958732	0.609495	-0.59183	2.629888
STPG4	0.958732	0.609514	0.768645	1.39362
TNKS2	0.958732	0.609593	-0.25049	7.43812
SLC12A4	0.958732	0.609836	-1.03678	1.305626
SLC51B	0.958732	0.609842	-0.80081	1.547757
LAMB3	0.958732	0.60994	-0.52235	3.616584
LINC00930	0.958732	0.609946	0.839656	1.080613
EP400	0.958732	0.609971	-0.23496	8.366261
GTF3C6	0.958732	0.610022	0.263412	5.857539
AGL	0.958914	0.610244	0.603777	1.546203
PAFAH1B1	0.958914	0.610325	-0.23456	7.759003
DANT2	0.958914	0.610471	-0.36078	5.049897
METTL21A	0.958914	0.61056	0.650671	2.039843
WASH3P	0.958914	0.61061	-0.35883	4.414326
UAP1	0.958914	0.610612	-0.27462	6.626618
RBPMS	0.958914	0.610681	-0.44458	4.478604
PJVK	0.958914	0.610723	-0.77027	2.444648
BCAT2	0.95894	0.610877	-0.72252	1.228679
LOC10013€	0.95894	0.610984	0.421056	2.893213
KRT5	0.95894	0.611048	1.004917	8.84647
RBMX2	0.95894	0.611157	-0.30978	5.094586
HIST1H1E	0.95894	0.611232	-0.27141	7.101108
ST5	0.95894	0.611433	0.485093	2.824957
DEUP1	0.95894	0.6115	-1.14205	1.824953
RNA18SN5	0.95894	0.611598	0.260814	11.87985
SESN3	0.95894	0.611659	0.518669	2.58011
LINC01260	0.95894	0.611674	-1.09161	1.472086
SIT1	0.95894	0.611678	-0.82157	1.140705
PTS	0.95894	0.611717	-0.36312	4.17556
SORBS2	0.95894	0.611939	0.483544	2.579109

WNK2	0.95894	0.611965	0.524956	2.405505
HLA-DQA1	0.95894	0.612049	-0.51776	5.25322
ZNF23	0.95894	0.61205	0.648899	0.909268
TTC3	0.95894	0.612058	0.2264	6.813255
GBP4	0.95894	0.612122	-0.39447	5.552981
TCTA	0.95894	0.612129	0.263743	4.798721
MPHOSPH9	0.959279	0.612426	-0.88324	0.941753
SNED1	0.959279	0.612678	-0.31715	6.125348
TPST1	0.959279	0.612719	0.272133	4.714982
SNORA60	0.959279	0.612874	-0.51767	4.527776
MIR200C	0.959279	0.612918	-0.75167	1.127275
CTHRC1	0.959279	0.61294	-0.52323	2.785883
HOXB7	0.959279	0.613101	-0.31973	5.565453
CASZ1	0.959279	0.613173	0.256241	5.905852
ITPR3	0.959279	0.613184	-0.25793	9.400421
KRT86	0.959279	0.613194	-0.84864	2.813504
PIP4P2	0.959279	0.613242	-0.57454	4.03069
RORC	0.959279	0.613246	0.535127	3.327099
CAPNS1	0.959279	0.613295	0.553245	1.104267
TSC22D1-A	0.959444	0.613474	-0.62052	2.765138
EPB41L1	0.959993	0.613898	0.383725	3.483362
SFXN4	0.960004	0.613978	0.736456	0.749047
CDK14	0.960144	0.614141	-0.75432	1.781071
CLNK	0.960312	0.614414	0.653923	2.090232
GTPBP2	0.960312	0.614429	0.317284	4.103522
PCID2	0.960312	0.614468	-1.09893	0.966278
RBM4	0.960312	0.614541	-0.31508	5.465769
DDX55	0.960397	0.614672	0.249268	5.848173
MED10	0.960397	0.61487	-0.4351	4.274162
HAS2	0.960397	0.614877	-0.60057	3.870393
SHPK	0.960397	0.61492	0.262984	5.403023
LOC100294	0.960397	0.614962	-0.31568	4.711932
COQ5	0.960517	0.615111	0.260337	4.766594
PPIL4	0.960716	0.615312	-0.231	6.905817
TCF7L2	0.960946	0.615582	-0.63377	2.597929
MRPL23-AS	0.960946	0.615606	0.73711	1.134937
LOC102546	0.961058	0.615839	-0.79561	1.949548
SRD5A1	0.961058	0.615937	0.379889	3.834939
ERICH4	0.961058	0.615993	0.693821	1.808309
DUSP14	0.961058	0.616033	-1.40406	1.591548
ZRANB2	0.961058	0.616098	-0.60527	2.306651
UST-AS1	0.961058	0.616235	-0.96285	1.75498
MIR29C	0.961058	0.616318	0.457972	2.779943
FGGY	0.961058	0.616339	-0.73077	2.077667
GOT2	0.961058	0.616375	0.819193	0.879249

USP48	0.961058	0.616631	0.205716	7.114047
STARD13	0.961058	0.616637	-0.5892	3.144154
CARM1	0.961058	0.616673	0.229591	6.069471
DDIT4	0.961058	0.616679	-0.30586	7.847519
ITPKB	0.961058	0.61678	-0.27084	6.406861
VWCE	0.961058	0.616785	-0.49132	2.849323
PDZRN3	0.961058	0.61685	-0.51613	4.336524
LINC00954	0.961338	0.617345	0.47914	2.719103
RHPN2	0.961338	0.617365	-0.30336	7.199576
ANKRD18A	0.961338	0.617387	-0.28173	5.806835
LINC00909	0.961338	0.617418	0.354333	3.896947
SH3BP5	0.961338	0.617458	0.604411	2.404429
SAMD10	0.961338	0.617469	0.423695	3.493702
LAP3	0.96142	0.61775	-0.25877	6.016028
POU5F1P4	0.96142	0.617756	-0.9437	1.550517
SNORD59B	0.96142	0.617806	-0.4816	3.678617
FOXG1	0.96142	0.617916	-1.14635	1.265211
ZNHIT1	0.96142	0.617977	0.239862	5.163037
HIST1H2AE	0.96142	0.618044	-0.49914	3.79667
STXBP4	0.96142	0.618317	0.296196	5.0724
LINC02145	0.96142	0.618334	0.825579	1.575543
LINC01235	0.96142	0.618552	0.562942	1.777258
ZBED4	0.96142	0.618601	-0.25524	6.375589
FAM3B	0.96142	0.618664	0.622037	1.199395
TLNRD1	0.96142	0.618693	-0.27715	5.641134
CYB5R4	0.96142	0.618756	0.270869	4.958452
PRPF4B	0.96142	0.61878	-0.22245	8.408234
TLR7	0.96142	0.618783	-0.72201	2.09272
HNRNPUL1	0.96142	0.618794	0.291983	4.37574
DDX19A	0.96142	0.618819	-0.23963	6.048286
PTCHD1	0.96142	0.61898	-0.83781	1.80086
BTBD3	0.96142	0.61912	0.677236	1.616036
FOXL2NB	0.96142	0.61921	0.926832	1.32238
MRTFB	0.96142	0.619244	0.737476	0.989232
TRMT5	0.96142	0.619409	-0.44106	4.221681
BAP1	0.96142	0.619479	0.213824	6.895126
RCN1	0.96142	0.619515	0.218619	7.178555
FAR1	0.96142	0.619646	-0.23158	7.716187
CCDC102A	0.96142	0.619817	-0.34057	3.860816
PRKCZ	0.96142	0.619855	-0.44766	3.406994
FAM219B	0.96142	0.619865	0.237387	5.991384
ANGEL2	0.96142	0.619932	-0.4693	2.898342
ATP6V0A2	0.96142	0.620014	0.232006	6.540363
PDE1A	0.96142	0.620087	-0.69107	2.956216
KRT16P3	0.96142	0.620096	-1.54075	1.040953

CTTNBP2N	0.96142	0.620102	-0.24564	6.854087
WAC-AS1	0.96142	0.620153	-0.23186	6.143155
MYH4	0.96142	0.620224	-1.05339	0.828017
S100A10	0.96142	0.620316	-0.32667	9.068117
ARID2	0.96142	0.620392	-0.22484	7.313792
ZNF441	0.96142	0.620557	-0.33333	4.804143
SULT1C2P1	0.96142	0.620567	0.509425	2.140411
LINC02614	0.96142	0.62069	-1.14757	0.912821
PAPLN	0.96142	0.620696	-0.30633	5.641135
IKZF5	0.96142	0.620728	0.52844	2.221844
L2HGDH	0.96142	0.620914	0.320746	4.284533
SYNPO2L	0.96142	0.620962	-0.82947	1.573191
CCNA2	0.96142	0.621104	0.412706	4.472654
MYL5	0.96142	0.621215	0.399382	2.508492
MTFMT	0.96142	0.621225	-0.39082	3.771329
ZNF579	0.96142	0.621342	-0.41814	3.74655
GOLGA8B	0.96142	0.621353	0.328907	3.576717
HIST1H2BG	0.96142	0.621389	-0.33342	4.583692
CPQ	0.96142	0.621391	0.271616	5.465089
ANXA9	0.96142	0.621394	-0.3626	4.300597
NFKBID	0.96142	0.621465	-0.30907	4.276105
CCDC61	0.96142	0.621479	0.304159	4.100663
SNORD28	0.961462	0.621663	-0.42006	4.115649
MID1	0.961462	0.621716	0.533372	2.842444
ADAM10	0.961462	0.621779	-0.23447	7.940236
CFAP58-DT	0.961462	0.621799	0.688052	1.375666
UPB1	0.961496	0.621912	-1.29536	1.010185
LINC01238	0.961496	0.622004	0.576398	2.777729
PIAS4	0.961496	0.622054	-0.25355	5.482833
MIR604	0.961496	0.622114	0.727036	0.974658
CCDC134	0.961546	0.622268	0.521594	2.335988
SFT2D2	0.961546	0.622293	-0.34476	4.870223
C16orf71	0.961622	0.622568	0.663282	1.970244
TRAF4	0.961622	0.622713	-0.26312	5.978367
HTR2B	0.961622	0.622805	-0.35442	3.99926
HAVCR2	0.961622	0.623048	0.344335	4.451008
NCOA6	0.961622	0.623176	-0.24997	6.535932
DNAJB2	0.961622	0.623193	-0.53122	3.209315
LINC00540	0.961622	0.623208	-0.83189	2.550458
KLF9	0.961622	0.623248	-0.25566	7.696325
TRAF3IP1	0.961622	0.623357	-0.80931	1.475842
DCST2	0.961622	0.623445	-0.80154	2.276562
CD8B	0.961622	0.623515	-0.99565	1.468743
SLAMF8	0.961622	0.623522	-0.65471	3.109869
SNORD15A	0.961622	0.623578	-0.26387	8.154646

TK1	0.961622	0.623638	0.245299	4.696686
KIAA0100	0.961622	0.623697	-0.21594	8.370718
PLA2R1	0.961622	0.623782	-0.7194	2.174615
MIPEP	0.961622	0.623844	-0.28492	4.811173
COL9A3	0.961622	0.623844	0.546149	2.437057
AIDA	0.961622	0.62388	0.253717	5.519111
OR13H1	0.961622	0.623899	0.772832	2.270766
HTR1D	0.961622	0.623967	0.514383	2.807715
RSC1A1	0.961622	0.623981	-0.356	4.259566
ZNF300P1	0.961622	0.624261	-0.71731	2.460394
LOC101927	0.961622	0.624296	0.889474	1.44376
KLC3	0.961622	0.624341	-0.75822	2.55524
C16orf74	0.961622	0.624349	0.49815	1.575747
NOTCH3	0.961622	0.62444	-0.24352	7.129379
SNORA16A	0.961622	0.624481	-0.41293	4.374476
ANKRD18B	0.961622	0.624503	-1.23394	2.927431
SPTLC1	0.961622	0.624541	-0.54793	2.088097
THEM6	0.961719	0.624702	-0.39724	4.273085
ZFR	0.961719	0.624791	-0.22633	7.696386
ZNF215	0.961719	0.624824	-0.37908	3.56236
SNORA25	0.961792	0.625017	0.237455	6.795701
SNRPF	0.961792	0.625139	0.283061	6.138615
GNB4	0.961792	0.625242	-0.31066	6.196709
UNC5A	0.961792	0.625308	0.573319	1.781081
TNFSF10	0.961792	0.625327	0.396786	2.669744
CPSF1	0.961792	0.625497	0.210359	6.948761
SRGN	0.961792	0.625511	0.601721	1.855681
MGAT1	0.961792	0.625532	0.322054	3.867209
ZMYM6	0.961792	0.625565	-0.23856	6.101865
ADAMTS9	0.961792	0.625771	-0.25812	6.834014
LIMS2	0.961792	0.625811	0.740958	1.609248
TRMT13	0.961792	0.625854	0.240382	4.97053
STX18	0.961792	0.62589	0.24307	5.448873
GAPDH	0.961792	0.62596	0.61044	2.104186
SNORD77	0.961792	0.625976	-0.66342	1.579763
RBM28	0.961792	0.626044	-0.41523	3.986971
GSTM2	0.961817	0.626253	-0.95438	1.450317
LOC101928	0.961817	0.626391	-0.45165	2.535003
LOC286059	0.961817	0.626443	-0.82716	1.444982
ARVCF	0.961817	0.626451	-0.38578	4.474131
PDZRN3-AS	0.961817	0.626539	-0.6408	2.25848
ZNF589	0.961817	0.626563	0.258162	5.691711
SLC7A14	0.961817	0.626627	-1.10069	1.515883
TCEAL4	0.961817	0.626891	-0.59282	2.225311
H3F3B	0.961817	0.62691	-0.21822	8.627898

DRG2	0.961817	0.62718	-0.28779	4.603671
MACROD2-	0.961817	0.627252	0.559522	2.448905
GRIK3	0.961817	0.627295	0.566194	2.480843
FOXE1	0.961817	0.627297	0.738333	1.414418
RABGAP1L	0.961817	0.627299	0.206953	6.927396
LOC28329S	0.961817	0.627483	-1.14853	1.710693
HOXB6	0.961817	0.627492	-0.39758	5.995195
MIR4534	0.961817	0.627517	-1.23216	0.774502
IGLON5	0.961817	0.627785	0.631109	1.528621
GRB7	0.961817	0.627831	0.530679	1.918862
NIT1	0.961817	0.627843	0.276085	4.419103
KRBA2	0.961817	0.627925	0.563004	1.892073
SEL1L	0.961817	0.627932	0.227572	7.627247
SACM1L	0.961817	0.627936	-0.2154	6.628975
HERC3	0.961817	0.628095	0.309294	3.791885
KIRREL3-AS	0.961817	0.628103	0.88051	0.700748
OTUD7B	0.961817	0.628121	-0.22506	7.193249
ZMAT1	0.961817	0.628195	-1.16697	1.202049
ZNF22	0.961817	0.62823	-0.29735	4.298641
TRAPPC2L	0.961817	0.628239	0.296033	3.906835
KLC1	0.961817	0.628357	0.282455	4.602317
CAMK2D	0.961817	0.628358	-0.63001	1.945203
KCNQ1	0.961817	0.628414	0.431174	2.043138
KCNJ14	0.961817	0.628506	-0.48988	2.492551
SLC17A5	0.961817	0.62857	0.253232	5.65493
SNORD19	0.961817	0.628627	0.540782	1.3691
KRT71	0.962081	0.628886	-0.9748	0.966145
KCTD5	0.962081	0.628994	-0.26452	5.297775
SLC16A8	0.962081	0.629201	-0.68125	2.985668
S100A6	0.962081	0.629236	-0.27429	10.02031
ZNF395	0.962081	0.62925	-0.2429	6.630236
MIR6801	0.962081	0.629347	-1.25222	1.508917
SNORD7	0.962081	0.629383	-0.90788	1.45204
FNDC3B	0.962081	0.629386	0.389229	3.358815
GINS2	0.962106	0.629513	-0.60458	2.444584
PCDHB2	0.962106	0.629549	0.575096	2.444693
LOXL2	0.962358	0.629787	-0.28931	6.055927
TBC1D10B	0.962591	0.630244	0.199249	6.575748
UTP14C	0.962591	0.630448	-0.21968	6.527191
MIR3654	0.962591	0.630526	-0.36079	4.853896
EPB41L2	0.962591	0.630573	0.390179	4.29028
OVCA2	0.962591	0.630621	0.49579	1.79848
IL17D	0.962591	0.6307	-0.75007	1.766082
KRT24	0.962591	0.630755	0.800214	3.622367
NBPF3	0.962591	0.63077	-0.7238	1.878999

SNORD99	0.962591	0.630782	0.312043	6.527854
RGP1	0.962591	0.630887	-0.30095	4.510543
OR2A7	0.962591	0.630916	0.797959	0.691133
ZBTB46	0.962591	0.630983	-0.43273	4.194694
ZNF438	0.962591	0.630992	-0.8834	1.719452
UBAP1L	0.962591	0.631027	-0.68523	2.361587
SCARNA8	0.962591	0.63104	-0.3402	5.629199
IQCC	0.9627	0.631185	0.614593	1.379526
MRTO4	0.962796	0.631395	-0.30907	5.089699
SMURF2	0.962796	0.631395	0.226481	6.217472
KIAA0586	0.962974	0.631627	-1.15982	0.614704
EFR3A	0.962974	0.631678	-0.22033	7.297728
DNAJC3	0.962974	0.631736	-0.23046	7.494663
GABPB1-IT	0.962974	0.631812	-0.35657	4.740481
SIM2	0.962974	0.631878	-0.41414	4.952249
ELOA2	0.963015	0.631978	0.690064	1.458813
GIMAP4	0.963091	0.632164	0.243388	5.650021
EFCAB14	0.963091	0.632198	-0.24585	8.565841
ASAH2B	0.963091	0.632249	-1.19941	0.840094
USP40	0.963229	0.632443	-0.23894	7.095887
BMI1	0.963229	0.632507	-0.53686	2.866913
HCAR1	0.963229	0.632559	-0.7564	1.929047
CHIC2	0.963264	0.632656	0.246351	4.904453
MRPS15	0.963352	0.632787	-0.28111	5.345447
CFAP44	0.96355	0.633043	0.249791	5.725278
RER1	0.96355	0.633064	0.197498	6.602808
OR2K2	0.963631	0.633326	0.966958	0.647674
SLC9C1	0.963631	0.633393	-0.65731	2.663201
DNAJB1	0.963631	0.633398	-0.36782	5.978492
LINC00431	0.963631	0.633496	-1.14249	0.925096
VTI1B	0.963631	0.63351	0.245333	5.419774
VSIG8	0.963631	0.633558	-0.58247	2.676023
PVT1	0.963811	0.63375	-0.32404	4.857168
APOL6	0.963838	0.633859	0.205028	7.63843
ATP5PO	0.963838	0.633914	0.22875	5.887974
ITGB1	0.963955	0.634065	0.535548	2.363794
C3orf80	0.964034	0.634201	-0.91585	0.932839
CEP290	0.964034	0.634362	-0.25263	6.823768
PXYLP1	0.964034	0.634419	-1.08251	1.424696
ORAI3	0.964034	0.634436	-0.47443	3.791982
COL17A1	0.964034	0.634609	0.281479	7.774194
CCDC191	0.964034	0.63466	-0.31141	5.511545
MACO1	0.964034	0.634697	0.389666	3.836441
APOLD1	0.964034	0.634777	0.62362	0.904729
C1orf35	0.964034	0.634842	0.80517	0.82313

DR1	0.964034	0.634994	0.222871	6.41645
FAM90A25	0.964034	0.63524	0.722195	1.43193
LINC00941	0.964034	0.635377	-0.55586	2.604306
DDIAS	0.964034	0.635451	-0.64531	3.452831
TMEM39B	0.964034	0.635557	0.338647	3.245259
RPAP1	0.964034	0.635693	-0.27546	5.166349
SHOC2	0.964034	0.635696	0.6174	1.227538
ERH	0.964034	0.635764	0.271908	5.499692
LINC01436	0.964034	0.635828	-0.71916	1.606015
ZNF337	0.964034	0.635879	-0.60117	2.473084
ABCF2	0.964034	0.635939	-0.50196	3.463982
A2M-AS1	0.964034	0.635959	-0.60905	2.713198
EIF4A2	0.964034	0.636047	-0.21101	8.921995
SELE	0.964034	0.636135	-0.59453	3.476437
TRIM56	0.964034	0.63617	0.196933	7.236207
GOLGA6L1(	0.964034	0.636177	0.526792	2.124703
MPZL2	0.964034	0.636239	0.299502	5.863488
FAM50A	0.964034	0.636479	0.194265	6.981396
HIPK1-AS1	0.964034	0.636496	0.677298	1.147524
FGF14-IT1	0.964034	0.636567	0.627611	1.788625
LRRC37A	0.964034	0.636576	0.546139	1.379699
RACK1	0.964034	0.636611	0.192731	8.963284
SKA2	0.964034	0.636688	0.670056	1.138861
PDCD7	0.964034	0.636699	0.219444	5.484765
FBXO11	0.964034	0.636706	-0.66031	2.388768
ZBTB49	0.964034	0.636773	-0.48388	3.354006
FKSG29	0.964034	0.636962	0.54576	2.094304
OR2D3	0.964034	0.637006	0.689769	1.242866
CYB561	0.964034	0.637112	-0.75312	1.505828
WDR37	0.964034	0.637149	0.226077	5.393554
BRAT1	0.964034	0.637196	-0.26723	5.459557
COX7A2L	0.964034	0.637217	0.226811	5.880366
ZNF836	0.964034	0.637239	-0.30755	5.071076
GALNS	0.964034	0.637276	-0.32966	4.882999
EID2	0.964224	0.63765	-0.3585	3.673409
FAM49A	0.964224	0.637693	-0.45174	4.6404
GMPPA	0.964224	0.637697	0.590943	2.146219
RIN3	0.964224	0.637729	0.256549	5.213666
ABCA6	0.964224	0.63777	-0.39854	6.139254
SAV1	0.964293	0.63798	-0.25209	5.570106
OGA	0.964293	0.638007	-0.49083	2.329678
RNF125	0.964293	0.63815	0.287256	4.829799
SART3	0.964293	0.638168	-0.23073	7.135635
HOXA5	0.964293	0.638493	0.500757	3.662992
PRR22	0.964293	0.638614	-0.64437	2.535063

IGFL1	0.964293	0.638701	0.894483	0.823964
NKIRAS1	0.964293	0.638817	-0.34199	3.906868
C1orf56	0.964293	0.638862	0.340074	3.115908
MOB3A	0.964293	0.638955	0.195368	6.005033
ANKRD20A	0.964293	0.639244	-0.62377	3.035982
LINC00922	0.964293	0.6393	-1.46536	1.301739
RTL8C	0.964293	0.639344	-0.22473	5.576203
DGCR9	0.964293	0.63937	0.752887	1.295379
SFXN5	0.964293	0.639381	0.242341	4.553519
FDX1	0.964293	0.639504	-0.27232	5.447037
PCBP1-AS1	0.964293	0.639561	0.276656	4.036014
CFAP70	0.964293	0.639569	0.445588	3.18773
SMG1P7	0.964293	0.639659	-0.38131	3.740511
SNORD16	0.964293	0.639671	-0.34716	5.560342
LOC100288	0.964293	0.639734	0.442771	3.211165
ZMYM5	0.964293	0.639735	-0.36468	3.628867
SRPRB	0.964293	0.639808	0.226163	5.762863
LAD1	0.964293	0.639833	0.268296	7.955658
FAM229B	0.964293	0.640016	-0.62414	2.898982
LRRC37A4P	0.964293	0.640021	-0.22001	6.471307
CFAP410	0.964293	0.640061	-1.02135	1.206374
RMND5A	0.964293	0.640122	-0.21199	7.187243
SSR4P1	0.964293	0.640127	0.749168	1.136372
BTBD9	0.964293	0.640145	0.542312	1.619756
MIR3175	0.964293	0.640164	0.451269	2.275882
TTC30B	0.964293	0.640167	-0.60363	2.829663
NAA30	0.964293	0.640322	0.214426	5.400647
APBB1IP	0.964293	0.64036	-0.33752	4.793129
NAP1L5	0.964293	0.640388	-0.35411	3.998354
COL9A2	0.964381	0.640543	-0.42729	5.60993
TUSC1	0.964381	0.640608	-0.53056	2.526493
HECW2	0.964381	0.640667	0.583585	1.752971
VRK1	0.964385	0.640743	0.273852	4.394546
ITGA7	0.964631	0.641049	-0.7601	1.418231
ZC3H14	0.964631	0.641053	-0.75489	1.749859
OR7E47P	0.96466	0.64118	-0.59471	1.921623
TMEM183B	0.96466	0.641324	-0.54931	2.790155
RABGAP1	0.96466	0.641327	-0.22611	7.88451
RPS19	0.96466	0.641523	-0.24028	8.519032
R3HDM4	0.96466	0.641573	-0.23692	6.050219
FMNL1	0.96466	0.641593	-0.26882	5.838235
PHAX	0.96466	0.641626	0.203448	5.605834
LOC100130	0.96466	0.641748	0.390286	3.312896
REXO2	0.96466	0.641801	-0.25551	5.657048
GALNT18	0.96466	0.641876	0.262814	4.30835

C5orf66-AS	0.96466	0.641924	-0.92806	1.187311
NCF1C	0.96466	0.641954	-0.43342	2.81493
RNF212B	0.964764	0.642287	0.610234	1.853326
CDH26	0.964764	0.642497	-0.77125	1.747112
PBRM1	0.964764	0.642509	-0.21797	7.932633
CRYBB3	0.964764	0.642564	-0.64743	1.652772
PHYKPL	0.964764	0.64258	0.453437	2.835717
FAIM2	0.964764	0.642764	0.384508	2.719651
NID2	0.964764	0.642802	-0.25406	5.904682
LMO3	0.964764	0.642884	-0.89865	1.969933
KIF24	0.964764	0.642913	-0.33937	4.164783
PCED1B-AS	0.964764	0.643144	-0.5204	3.801775
ZBTB6	0.964764	0.64316	-0.38091	4.909098
OGFOD2	0.964764	0.643246	-0.67911	2.06319
SPTB	0.964764	0.643381	-0.31775	4.04158
PSTPIP2	0.964764	0.643444	-0.44097	4.080445
XK	0.964764	0.643523	0.279494	4.755417
PLCXD3	0.964764	0.643543	-0.8988	2.375282
N4BP1	0.964764	0.643668	-0.21025	6.889545
TRA2B	0.964764	0.643878	-0.4772	2.807801
LTO1	0.964764	0.644156	-0.29455	4.874437
ADCY9	0.964764	0.64416	-0.27955	6.909435
FAM122A	0.964764	0.644163	0.240142	5.403584
APIP	0.964764	0.644171	0.286908	4.219993
MAP1B	0.964764	0.644212	-0.2755	8.905671
TMEM37	0.964764	0.644229	0.332758	3.302493
FXR2	0.964764	0.644357	0.220186	5.604186
HNRNPDL	0.964764	0.644505	-0.41685	3.090245
SORCS3	0.964764	0.644687	-0.95147	1.247425
CENPV	0.964764	0.64501	-0.37379	4.007389
TAF5L	0.964764	0.645122	-0.34475	4.58939
LOC101929	0.964764	0.64514	0.624833	2.158663
C11orf24	0.964764	0.645156	0.302396	4.249336
USF2	0.964764	0.645157	0.551069	1.08303
LINC00996	0.964764	0.64518	0.567291	1.830873
LOC285762	0.964764	0.645235	0.568036	2.209387
LINC01695	0.964764	0.645442	0.472393	2.597711
LRIG1	0.964764	0.645761	-0.23749	7.134833
HECA	0.964764	0.645786	-0.20712	6.944201
FAM13A-A	0.964764	0.645863	0.282441	3.980837
PILRB	0.964764	0.645872	-0.73129	2.15312
LINC00886	0.964764	0.645994	-0.55973	4.196483
OGFRP1	0.964764	0.646019	0.542348	2.263468
LINC02208	0.964764	0.646053	0.775465	0.888017
ALOX12P2	0.964764	0.646101	-0.56276	3.066626

IL10RA	0.964764	0.646158	0.498756	1.699115
REREP3	0.964764	0.646202	-0.58501	2.483104
LINC00092	0.964764	0.646261	0.75737	1.339719
CTAGE7P	0.964764	0.646354	0.419099	2.340485
OSBPL10-A	0.964764	0.646409	0.520299	2.524769
PFKL	0.964764	0.646444	-0.61517	3.044542
MBTPS1	0.964764	0.64648	-0.21056	7.70702
KLF11	0.964764	0.646528	-0.83145	0.925918
AGPAT3	0.964764	0.646558	-0.71345	1.153011
GLRX3	0.964764	0.646592	-0.78453	1.757791
HIST1H2BK	0.964764	0.646869	0.504299	2.751996
CCDC59	0.964764	0.646926	-0.93086	1.252208
PEX7	0.964764	0.646938	-0.41814	3.1002
SNORD3C	0.964764	0.646939	0.271174	9.038146
N4BP2L2	0.964764	0.647052	-0.21086	7.761689
MAPK11	0.964764	0.647077	0.596475	1.740174
ZNF644	0.964764	0.647083	-0.20744	6.69321
MRPS16	0.964764	0.647091	-0.22748	5.85092
SDHAP3	0.964764	0.647398	-0.36977	4.455461
GTF2F1	0.964764	0.647437	-0.2085	6.998848
LOC100505	0.964764	0.647534	-1.12382	2.065834
AHSA1	0.964764	0.647573	0.216155	6.876556
LOC100132	0.964764	0.647583	-0.43974	3.815701
ADCY1	0.964764	0.647625	-0.36558	4.031121
POLR3G	0.964764	0.647721	-0.57583	2.960547
LINC00861	0.964764	0.647879	-0.41285	3.95679
IER5	0.964764	0.64788	0.221661	6.27326
CELSR3	0.964764	0.647907	0.258686	6.18488
NCOA2	0.964764	0.647947	0.184988	7.397887
PLEKHO2	0.964764	0.647989	-0.81787	1.324834
GALNT10	0.964764	0.647997	0.210385	7.114663
TROAP	0.964764	0.648018	0.374312	3.511327
MXI1	0.964764	0.648056	0.513979	1.860336
TBC1D1	0.964764	0.648168	0.359806	2.959334
NCKAP5L	0.964764	0.64818	-0.25364	5.184552
HCN3	0.964764	0.648186	0.700374	1.060619
LINC01644	0.964764	0.648279	0.746196	1.10948
ORC5	0.964764	0.648326	-0.61996	2.604309
PRICKLE4	0.964764	0.64843	0.300556	4.345222
SNORD83B	0.964764	0.648497	-0.65946	3.605128
UCP3	0.964764	0.648561	-0.62912	1.412746
DACT3-AS1	0.964764	0.648676	0.54515	1.504899
FAM160A1	0.964764	0.648753	0.266252	5.219315
TMEM72	0.964764	0.648787	-1.0804	1.37269
SLC16A5	0.964764	0.648935	0.667816	0.817323

CRIPAK	0.964764	0.649057	0.27054	4.91598
TMEM208	0.964764	0.64907	-0.32635	4.446988
RASD2	0.964764	0.64907	0.483625	2.663854
TYRO3	0.964764	0.649086	-0.33312	3.766228
ZFHX2	0.964764	0.649097	0.2741	5.100143
KLHL4	0.964764	0.649098	0.761483	1.163763
DCLRE1B	0.964764	0.649204	0.279573	4.044046
LINC02102	0.964764	0.649207	-0.92338	1.475161
GRSF1	0.964764	0.649227	0.467724	1.745325
DELE1	0.964764	0.649279	-0.44418	3.835132
TPCN1	0.964764	0.649367	-0.77171	1.346803
HLA-E	0.964764	0.649419	0.308212	4.172745
LOC10666(	0.964764	0.649451	-0.71429	2.17406
LCT	0.964994	0.649679	-0.93198	0.961378
GPAT4	0.965053	0.649793	-0.20671	6.906723
IFT122	0.965132	0.649919	0.701703	1.00888
ZNF212	0.965134	0.649994	0.23938	4.939576
GID4	0.965146	0.650076	-0.29887	4.554128
EHD2	0.965171	0.650246	-0.28961	6.738714
FAM167A	0.965171	0.650316	-0.5551	3.386279
ABLIM2	0.965171	0.650356	0.528508	2.748024
ATE1-AS1	0.965171	0.650584	-0.83941	1.229356
MIR544B	0.965171	0.650631	-0.97095	1.023857
SECTM1	0.965171	0.65068	-0.41298	4.33535
RBPMS-AS1	0.965171	0.650964	0.407158	3.231376
ANKRD23	0.965171	0.651015	-0.43096	3.736451
LOC10028(	0.965171	0.651136	-0.67989	2.24719
SMPD2	0.965171	0.651138	0.33127	3.382192
BCL2L11	0.965171	0.651158	-0.55137	3.371841
S100A7	0.965171	0.651195	-1.38338	5.730573
SH2B3	0.965171	0.6513	-0.52428	2.839832
SPNS2	0.965171	0.65133	0.259229	5.777482
TDRD7	0.965171	0.651356	0.455443	2.295334
FAM162A	0.965171	0.651366	0.209291	5.060603
PRUNE2	0.965171	0.651375	0.798362	0.975969
MCEE	0.965171	0.651451	-0.33437	3.91425
TRAF3IP2-/	0.965171	0.651549	-0.45536	3.350599
GUSBP5	0.965171	0.651601	-0.82018	0.865062
RNASE8	0.965171	0.651638	-1.12235	0.598865
RORA-AS1	0.965198	0.651844	0.330876	3.661883
SYCE2	0.965198	0.651845	-0.84576	0.860081
SLC9C2	0.965198	0.651925	0.66524	1.392289
CBR3-AS1	0.965198	0.65195	0.534528	1.888841
LYST	0.965444	0.65219	0.368587	3.798106
NTPCR	0.965719	0.652495	-0.29633	4.457396

GGT8P	0.965719	0.652523	0.530146	3.982359
FBXO9	0.965764	0.652627	0.491604	2.479703
MITD1	0.966136	0.652957	-0.23437	5.48927
SYNGAP1	0.966136	0.653119	0.287303	4.08888
BICRAL	0.966136	0.653176	-0.21711	6.338152
AGBL5	0.966136	0.653295	-0.83203	0.969919
LINC02076	0.966136	0.653429	0.56597	1.138115
POLR2A	0.966136	0.653445	0.180757	8.699499
PSPC1	0.966136	0.6535	-0.25523	5.495778
TOMM22	0.966136	0.653516	0.225003	5.927503
CMAS	0.966136	0.653682	-0.28787	5.023749
MIR199A1	0.966136	0.653801	0.623848	0.838263
RGS3	0.966136	0.653898	0.364645	3.502469
DUSP3	0.966136	0.653906	-0.20946	6.869828
JARID2-AS1	0.966136	0.653907	-0.794	1.186655
RBCK1	0.966136	0.653912	-0.77915	2.243176
PAQR7	0.966136	0.653983	0.323893	4.08057
TBC1D20	0.966253	0.654266	-0.23244	5.130391
SLCO3A1	0.966253	0.654305	-0.35012	4.494515
RHOU	0.966253	0.654342	0.634073	0.809297
MROH6	0.966253	0.65444	-0.84305	0.71308
DTD2	0.966253	0.654483	-0.28924	4.533363
ZNF354A	0.966253	0.654504	0.278273	4.48909
MROH1	0.966371	0.654672	-0.67259	1.636168
TRIP4	0.966371	0.654731	-0.23802	5.690493
ZNF740	0.96642	0.655199	-0.21393	6.291727
ZBED5-AS1	0.96642	0.655236	-0.84418	0.946324
HIST1H3H	0.96642	0.655237	-0.43677	4.218622
VWA7	0.96642	0.655241	0.570566	2.337888
CDHR2	0.96642	0.655269	-0.74389	1.189093
CTTN	0.96642	0.65532	-0.47345	3.229065
EMC9	0.96642	0.655328	0.338538	2.974932
BCLAF3	0.96642	0.655407	-0.20627	6.425608
DRAM2	0.96642	0.655484	-0.26317	4.98129
NCAPH2	0.96642	0.655638	-0.75544	0.934265
PLEKHG4B	0.96642	0.655774	-0.63359	3.606425
SERF2	0.96642	0.655799	0.426166	2.627389
RFX7	0.96642	0.655808	-0.24089	6.784538
GPR107	0.96642	0.655868	-0.58876	2.265643
HIP1	0.96642	0.655908	0.53724	1.302507
PLTP	0.96642	0.655943	0.527926	2.64836
STX1B	0.966481	0.656181	-0.67568	1.886172
DMTF1	0.966481	0.656369	0.288843	3.895648
MBLAC1	0.966481	0.656436	0.502366	2.214195
TPMT	0.966481	0.656473	0.253147	5.886065

DENND4C	0.966481	0.656492	-0.46653	2.601633
POMGNT1	0.966481	0.65657	0.521901	2.105354
IL10	0.966481	0.656772	-0.95426	1.70348
RPL29	0.966481	0.656777	0.227161	7.40798
MUM1	0.966481	0.656842	-0.88723	0.943351
C11orf53	0.966481	0.656922	-0.7973	0.947075
MGA	0.966481	0.656966	-0.68395	1.668548
SCN9A	0.966481	0.657028	-0.531	3.499479
LRFN5	0.966481	0.657225	-0.64197	2.17594
SNORA51	0.966481	0.65742	0.245164	5.584094
ADM	0.966481	0.657423	-0.33541	5.559433
MDN1	0.966481	0.657443	-0.21048	8.875821
SNORA50A	0.966481	0.657652	-0.59342	3.267777
CDK5R1	0.966481	0.657683	-0.51179	2.839234
WWC2-AS2	0.966481	0.657688	0.563632	1.144973
FOXO3B	0.966481	0.657751	-0.2135	5.951182
RTN1	0.966481	0.657753	-0.61547	1.875278
HEPHL1	0.966481	0.657826	0.462289	4.403891
HIST1H2BC	0.966481	0.657849	-0.46799	4.097581
TESC-AS1	0.966481	0.658196	0.847771	0.766288
PRSS27	0.966481	0.658361	0.538598	3.202131
WDR5B	0.966481	0.658483	-0.27589	4.355251
ZNRF2	0.966481	0.658491	0.178177	6.251453
TMEM165	0.966481	0.658516	-0.72229	1.771005
SNORD46	0.966481	0.658536	-0.31372	7.199869
AKR1C1	0.966481	0.658561	0.285326	5.027212
LRRC1	0.966481	0.658587	0.201277	6.27922
FAHD2B	0.966481	0.658594	-0.547	2.474528
TKFC	0.966481	0.658624	0.246789	5.351307
NR2F1	0.966481	0.658728	0.245031	5.978837
HIST1H2BE	0.966481	0.658749	-0.36621	3.640428
GLI4	0.966481	0.658769	-0.3227	3.698103
GATA6	0.966481	0.658942	0.234371	6.635225
ARSI	0.966481	0.659013	-0.70288	2.255947
PADI3	0.966481	0.65907	-0.77679	1.71543
OBSL1	0.966481	0.659301	-0.61858	2.024441
LINC00471	0.966481	0.659322	0.548357	1.261667
MALSU1	0.966481	0.659398	-0.30945	4.082735
UBN1	0.966481	0.659451	-0.54871	2.697017
NDUFAF7	0.966481	0.659542	-1.0158	1.107863
SEMA3G	0.966481	0.659609	-0.4533	4.077008
FAM32A	0.966481	0.659911	-0.20812	6.331087
LOC441155	0.966481	0.659919	0.406576	2.412488
MED21	0.966481	0.659929	0.623562	0.965353
BCKDHB	0.966481	0.659963	-0.53893	3.170496

RAC1	0.966481	0.659991	-0.76036	0.866722
ST7-AS2	0.966481	0.660047	0.438091	3.433742
RPL10A	0.966481	0.660065	0.200012	7.970291
LINC01336	0.966481	0.660129	-0.86489	2.152206
ZNF705E	0.966481	0.660203	-0.97926	1.141166
YDJC	0.966481	0.660209	0.301369	3.882449
PODXL2	0.966481	0.660272	-0.32715	5.312423
ZNF174	0.966481	0.660319	-0.62361	2.462783
CENPB	0.966481	0.660325	0.192779	7.225796
ZNF318	0.966481	0.660331	-0.2231	6.886279
NTN5	0.966601	0.660633	0.636942	1.154936
MIR4454	0.966601	0.660668	0.687252	1.157813
TCP11L2	0.966601	0.660679	0.266043	4.722764
MIR762HG	0.966601	0.660765	-1.06741	1.001251
RNF222	0.966601	0.66081	0.733548	1.218115
RBM42	0.966601	0.660959	0.212297	5.754999
EIF2B5	0.966601	0.660963	-0.20788	6.728245
LOC643387	0.966601	0.661002	0.600767	0.956906
C10orf95	0.966895	0.661277	-0.82388	1.261632
LOC10050(	0.967256	0.661663	-0.45075	3.290102
MIR3605	0.967256	0.661671	0.766728	0.846017
CEP135	0.967269	0.661854	-0.23868	5.329815
RPS29	0.967269	0.661876	0.390069	3.313247
MIR5690	0.967269	0.661946	0.248827	5.126064
APP	0.967269	0.661974	0.49243	1.92195
SNORA73B	0.967274	0.662133	0.208397	9.782872
SNRK-AS1	0.967274	0.662194	-0.42349	3.107401
DIABLO	0.967274	0.662199	0.511194	1.580742
INSYN2B	0.967477	0.662412	-0.41347	3.482048
STT3B	0.967638	0.662733	-0.21469	7.998862
EVA1B	0.967638	0.663308	0.497549	1.589529
DNAJC24	0.967638	0.663392	-0.3023	4.093346
CCDC84	0.967638	0.663527	-0.84345	1.966122
NPR2	0.967638	0.663558	-0.25071	5.079911
SLC11A2	0.967638	0.663579	0.412701	2.68391
COL3A1	0.967638	0.663589	0.208077	11.61637
C19orf53	0.967638	0.663662	-0.20775	5.886553
CYSTM1	0.967638	0.663732	0.274835	7.151092
FAM238B	0.967638	0.663774	0.743884	1.187257
EXOC3L2	0.967638	0.663846	-0.91765	1.10313
DARS2	0.967638	0.664007	0.192141	5.809066
ADM2	0.967638	0.664032	0.291916	4.135288
HOOK1	0.967638	0.664251	-0.27852	6.056491
DENR	0.967638	0.664425	-0.2047	6.761271
TMPRSS11I	0.967638	0.664436	0.750935	2.994271

CNRIP1	0.967638	0.664506	-0.77864	2.024489
FOXP1-AS1	0.967638	0.664608	0.415272	3.336247
MOSPD2	0.967638	0.6647	-0.62187	1.54212
PITRM1-AS	0.967638	0.664705	-0.33464	4.511578
RNF126	0.967638	0.664752	-0.27419	5.514648
FEM1A	0.967638	0.664849	0.202162	5.774847
ADGRG7	0.967638	0.664861	0.678636	2.185481
MIPEPP3	0.967638	0.664863	-0.69042	1.549462
TAOK3	0.967638	0.664913	0.175675	7.492733
SGSM3	0.967638	0.664926	0.619116	0.761838
MEX3B	0.967638	0.665039	-0.6154	2.089066
MYCBP2	0.967638	0.665169	0.171944	8.718469
ZNF451-AS	0.967638	0.665173	0.477516	2.078398
GPR173	0.967638	0.665243	0.397261	2.9908
KLHL6-AS1	0.967638	0.665244	0.649763	2.112028
FAM53B-A:	0.967638	0.665306	0.549765	1.520761
MORN1	0.967638	0.665333	-0.4587	2.117186
RUFY2	0.967638	0.665358	-0.53738	3.561557
KHSRP	0.967638	0.665358	-0.19975	7.874777
MAP7	0.967638	0.665372	0.641084	1.081162
LOC100132	0.967638	0.665451	-0.48623	3.180132
LINC01347	0.967638	0.665591	0.339692	3.360823
MAML2	0.967638	0.6656	-0.20851	7.281666
UTP20	0.967638	0.665774	-0.2044	6.661309
SLC25A23	0.967638	0.665913	-0.20373	6.735878
GMEB2	0.967638	0.665942	-0.27973	5.104454
UCP2	0.967638	0.665953	0.282257	5.119242
LAPTM4A	0.967638	0.665958	-0.20097	7.359811
MRPL15	0.967638	0.666026	0.222265	5.395638
CDC42SE1	0.967638	0.666099	-0.62837	2.053241
OTOGL	0.967638	0.666128	-0.67643	2.452216
SNORD127	0.967638	0.666222	-0.81177	1.704878
P2RX1	0.967638	0.666242	0.394782	3.24281
SBDS	0.967638	0.666248	-0.19857	7.159696
DCBLD1	0.967638	0.666302	0.203175	5.495827
COL28A1	0.967638	0.666358	0.338926	5.148878
KHDRBS1	0.967953	0.666648	-0.50467	3.247732
TIPARP-AS:	0.967991	0.666748	0.444347	2.080882
SEPT5-GP1	0.968	0.667051	-0.88506	0.539562
FAM98B	0.968	0.667141	0.219776	5.875257
B3GAT3	0.968	0.667246	0.623143	1.700453
EXOSC4	0.968	0.667265	-0.44723	3.334676
DPCD	0.968	0.667279	-0.41188	3.866268
LINC00865	0.968	0.667401	0.39465	3.145907
MON2	0.968	0.667446	0.535838	2.203112

LOC10013(	0.968	0.667446	-0.44657	4.112461
CBX3	0.968	0.667474	0.388395	2.179139
SDF2	0.968	0.667492	0.547195	1.259541
TMEM69	0.968029	0.667586	-0.33527	4.347619
IFNGR2	0.968152	0.667858	-0.33631	5.023159
C5orf63	0.968152	0.668345	0.234636	4.853921
SLC26A2	0.968152	0.668429	0.243546	5.659169
TANK	0.968152	0.668481	0.33227	2.737159
SYT9	0.968152	0.668493	0.698509	1.078719
MOSMO	0.968152	0.66851	-0.2673	5.116827
PNPLA2	0.968152	0.668617	0.190724	6.559214
PDS5B	0.968152	0.668635	-0.21149	7.172929
TMEM41A	0.968152	0.668683	-0.25385	4.876303
C22orf39	0.968152	0.668758	-0.42657	3.784588
NDUFA4	0.968152	0.668887	-0.23183	5.792534
MIR200B	0.968152	0.668903	-0.57405	2.165033
ARRDC2	0.968152	0.669082	0.709119	0.633735
RPS20	0.968152	0.669116	0.430461	2.2687
DNTTIP1	0.968152	0.669133	-0.24341	5.40518
USH2A	0.968152	0.669153	0.481689	2.766304
CCDC50	0.968152	0.669246	0.255577	4.827124
DDX23	0.968152	0.669311	-0.20665	7.940246
EFS	0.968152	0.66942	0.613879	0.778344
SRSF4	0.968152	0.669459	0.183003	7.885969
LINC02256	0.968152	0.669538	0.397009	2.871283
C9orf163	0.968152	0.669577	0.581289	1.82724
LPL	0.968152	0.669694	0.333213	3.949868
FYTTD1	0.968152	0.6698	0.294037	3.583775
SRC	0.968152	0.669831	0.408866	2.020077
PAXBP1	0.968152	0.669891	-1.01805	0.811674
SNN	0.968152	0.669917	-0.28863	4.170123
ROM1	0.968152	0.670194	0.49839	1.950765
ANO2	0.968152	0.670471	-1.13792	1.119824
LINC00982	0.968152	0.67065	-0.56992	3.871342
FGFR2	0.968152	0.67067	-0.78385	2.57598
COX7A1	0.968152	0.67069	-0.45619	3.048564
CCNI	0.968152	0.6707	0.184016	8.010853
TMEM181	0.968152	0.670737	-0.20228	7.476364
RPL31	0.968152	0.670829	-0.60537	3.563717
LIN52	0.968152	0.67084	-0.4409	3.460364
FMN1	0.968152	0.670875	0.200026	5.27121
DHX58	0.968152	0.670903	0.253595	4.242783
DLEU7-AS1	0.968152	0.670995	-0.49189	3.02855
CYB5B	0.968152	0.671069	-0.24737	6.384214
ZFP41	0.968152	0.671108	0.238242	4.584178

LOC104054	0.968152	0.671194	-0.9584	1.07119
CTBP1	0.968152	0.671215	0.344032	2.895898
ABHD1	0.968152	0.671255	-0.49402	2.812109
CLMP	0.968152	0.671285	0.206362	6.20395
NAGPA-AS1	0.968152	0.671287	-1.11552	1.923779
NMT1	0.968152	0.671353	-0.18838	6.909012
FGR	0.968152	0.671425	0.451941	2.449841
ALX4	0.968152	0.671493	0.457777	1.964379
SMAD3	0.968152	0.671556	-0.33186	4.285082
FYCO1	0.968152	0.671619	-0.19322	7.625033
ICA1L	0.968152	0.671694	-0.85432	1.175067
CTR9	0.968152	0.67176	-0.19421	6.890673
AKT1S1	0.968152	0.671763	-0.50333	2.865787
SMARCC2	0.968152	0.671767	-0.41336	3.292909
GNG12	0.968152	0.671803	0.184852	7.525294
TSTD3	0.968177	0.671894	0.247594	4.223185
ZNF224	0.968344	0.672197	0.303461	3.219677
FBP1	0.968344	0.672217	-0.7197	0.707661
NAT14	0.968344	0.672232	-0.54718	2.278147
ZNF717	0.968371	0.672324	0.508624	1.728717
MAFG	0.96856	0.672872	-0.79503	0.797987
C10orf88	0.96856	0.672914	0.283159	3.52979
RNF170	0.96856	0.672929	-0.68471	1.204363
IFRD2	0.96856	0.673056	0.243352	5.151629
SNORD95	0.96856	0.673074	-0.41772	4.645665
MIR4728	0.96856	0.673114	-1.11368	0.475966
BBS1	0.96856	0.673282	-0.23354	5.30464
JAZF1	0.96856	0.67339	-0.31687	4.173623
HCG17	0.96856	0.673443	-0.72066	1.673042
TRIM27	0.96856	0.673456	-0.35108	3.47938
IER3	0.96856	0.673656	0.356032	2.411988
PPP3R1	0.96856	0.673669	-0.21621	7.056584
RDM1P5	0.96856	0.673975	0.650142	1.133879
MMGT1	0.96856	0.674042	0.21899	5.962991
LOC101929	0.96856	0.674136	-0.87738	0.899582
POP5	0.96856	0.674213	-0.91065	0.754154
TMEM97	0.96856	0.674344	-0.27657	4.653846
MIR1-1HG-	0.96856	0.674436	0.526348	2.416704
ZNF57	0.96856	0.674475	-0.3366	3.766737
KCNJ10	0.96856	0.674553	-0.57569	2.188534
ANKH	0.96856	0.674636	0.185665	6.738783
ARHGAP5-A	0.96856	0.674682	-0.62704	1.982543
LAGE3	0.96856	0.674686	-0.52606	2.13674
SNRPA	0.96856	0.674691	0.189584	5.686875
NBAT1	0.96856	0.674741	0.435684	3.662196

PRRT2	0.96856	0.674774	-0.52078	3.154916
POLR2D	0.96856	0.674861	-0.25783	5.211494
ATR	0.96856	0.674873	-0.19955	6.998186
RNF208	0.96856	0.674972	-0.5745	1.971612
ZNF668	0.96856	0.675008	0.435684	2.091607
PIGM	0.96856	0.675059	-0.27085	5.059724
GANC	0.96856	0.675082	0.206984	5.431945
COQ10B	0.96856	0.675095	0.221817	5.359981
ATP6V0C	0.96856	0.675166	0.470971	1.036876
CD2AP	0.96856	0.675176	-0.20618	8.611617
SLC36A1	0.96856	0.675187	-0.23096	5.611275
CASP3	0.96856	0.675216	-0.81596	1.165999
ABCA3	0.96856	0.67532	-0.37214	4.295751
EXT1	0.96856	0.675335	0.179072	7.059802
ALG1	0.968709	0.675512	-0.31825	4.010488
LOC72874[illegible]	0.969115	0.675894	-0.34231	4.259279
PMPCB	0.969115	0.675943	0.20605	6.204544
LINC00638	0.969125	0.676046	0.669784	0.823048
CFAP46	0.969125	0.676098	-0.49224	4.183108
CFAP58	0.969125	0.676172	0.563306	1.395174
CRIM1-DT	0.969229	0.676342	0.522114	1.644158
BMT2	0.969229	0.676392	-0.32727	4.61916
EXOSC8	0.969238	0.676534	-0.30513	4.487227
ZNF254	0.969238	0.676546	-0.88197	0.902431
ARL10	0.969267	0.676711	0.396966	2.834505
FAM210A	0.969267	0.676714	-1.01945	0.813349
SETMAR	0.969267	0.676827	-0.81265	1.580752
ITGA11	0.969267	0.676861	-0.25561	5.605002
SLC25A53	0.96945	0.677063	0.280396	3.26726
MAOB	0.969488	0.677164	0.28211	5.700543
USP22	0.969596	0.677313	-0.19622	7.857648
UBE2D4	0.969671	0.677458	-0.27142	4.399317
MIR196A1	0.969671	0.677574	0.616488	0.801952
ACOT13	0.969671	0.67767	0.558891	1.127173
C2orf92	0.969671	0.677743	-0.70413	2.108341
CEP70	0.969671	0.677767	0.61511	0.868594
JTB	0.969671	0.677809	-0.225	6.798651
KCTD7	0.970025	0.678241	0.280951	3.505899
NOMO1	0.970025	0.678273	0.271305	4.536344
AUNIP	0.970025	0.678362	-0.77242	1.251332
RGS18	0.970025	0.678492	-0.54908	2.055377
KIAA0895	0.970025	0.678499	0.552297	1.407304
KIDINS220	0.970025	0.67851	-0.19065	8.291533
TRIM7	0.970025	0.678778	-1.06163	0.911377
ME1	0.970025	0.678849	0.216534	5.793978

PPM1B	0.970025	0.678915	0.219479	5.111621
PAWR	0.970025	0.678915	0.181647	6.929957
PRPF40B	0.970025	0.678992	-0.85613	1.284058
VWA5B1	0.970025	0.679081	-0.60342	1.66078
RASSF2	0.970025	0.679127	0.347433	2.61027
BCAM	0.970025	0.679286	-0.42011	2.93367
RBBP9	0.970025	0.679373	-0.25358	4.874224
ZNF799	0.970025	0.67938	-0.51223	2.609656
GNAI2	0.970025	0.67938	0.505317	2.411522
CCDC88B	0.970025	0.679387	0.220659	5.764275
COPG2IT1	0.970102	0.679564	0.222475	4.696707
HMGN2P4(	0.970102	0.679589	0.483887	1.506925
IL1RL2	0.970157	0.679702	-0.58447	2.524987
MAFG-DT	0.970404	0.680071	-0.63841	2.065731
ARNTL2	0.970404	0.680093	-0.88937	1.839676
PGAM4	0.970404	0.680096	0.384815	2.925834
RAB38	0.970628	0.680399	0.470078	2.644305
ZNF488	0.970628	0.680401	0.37414	2.63517
MED14	0.970681	0.680527	-0.19794	6.975058
CALD1	0.970681	0.680586	-0.2702	9.069837
PRDX2	0.970869	0.680838	0.174479	5.744442
JUN	0.970869	0.680937	-0.23269	8.763321
LOC10192(	0.970869	0.680941	0.569091	1.101025
GAL3ST4	0.970889	0.681029	-0.46126	3.156478
ROCK1	0.97093	0.681198	-0.18644	8.979177
AFF4	0.97093	0.681208	-0.18366	8.552481
SDR16C5	0.97093	0.681279	-0.36892	4.519685
FLRT1	0.971144	0.681503	0.397154	3.87392
CACNG4	0.971174	0.681598	0.537281	1.13569
LOC10537(	0.971473	0.682177	0.44101	2.020045
DEXI	0.971473	0.682189	0.265326	4.647266
DECR1	0.971473	0.682225	0.182541	5.921302
ZNF674-AS	0.971473	0.682543	-0.43855	2.436407
LOC10050!	0.971473	0.682677	0.668311	0.733236
BRIP1	0.971473	0.682755	-0.30466	4.830315
ZCCHC4	0.971473	0.682805	0.237818	4.42434
PPIB	0.971473	0.682839	0.176189	7.136815
ARMC7	0.971473	0.682853	-0.6496	1.413011
FBXO8	0.971473	0.683077	-0.29429	4.211411
CLASP2	0.971473	0.683129	0.240314	4.82874
LARS2-AS1	0.971473	0.683132	-0.619	1.225114
H2AFZ	0.971473	0.683187	-0.22142	5.519566
NFKBIE	0.971473	0.683195	-0.39721	3.979907
LOC10192	0.971473	0.683318	0.412612	3.421584
DNAJB13	0.971473	0.683637	-0.7211	0.806494

SMIM5	0.971473	0.683776	0.255762	5.11453
LOC100507	0.971473	0.683789	0.551233	2.023674
MYOM2	0.971473	0.683906	-0.87319	1.124878
ASPG	0.971473	0.683977	0.490272	1.549716
PREB	0.971473	0.684038	0.193451	5.817745
SAMD15	0.971473	0.684152	0.351469	2.387088
KMO	0.971473	0.684154	-0.66735	1.996981
FKBP4	0.971473	0.684207	-0.23427	7.28233
MRPS35	0.971473	0.68434	0.667665	1.451666
ZBTB42	0.971473	0.684419	0.272912	3.535708
AP5B1	0.971473	0.684459	-0.2403	5.781791
NCOR1	0.971473	0.684498	0.219292	4.782578
SLC12A9	0.971473	0.684538	0.246147	3.757344
ARL6IP5	0.971473	0.684547	0.19199	6.578151
TEAD1	0.971473	0.684555	-0.18639	7.561389
ROR1-AS1	0.971473	0.684686	-0.57938	2.935921
DAGLA	0.971473	0.684814	0.261503	4.293653
DTX1	0.971473	0.684831	0.364675	3.10424
TBC1D16	0.971473	0.684919	-0.29524	4.126038
PGGT1B	0.971473	0.684994	-0.22578	5.242511
LOC105372	0.971473	0.68519	0.657155	0.981608
FBF1	0.971473	0.685235	0.240196	4.528894
ARCN1	0.971473	0.685326	-0.56175	2.294766
CSNK1G2-A	0.971473	0.685357	0.369668	2.340874
ANP32AP1	0.971473	0.685534	-0.61021	1.807095
ZBTB18	0.971473	0.685563	-0.42299	4.099966
ZNF404	0.971473	0.685702	-0.60999	2.119331
RGMB-AS1	0.971473	0.685745	-0.89152	1.728181
METTL14	0.971473	0.685757	0.176215	5.808955
SRP72	0.971473	0.685878	0.371231	2.641777
RBM41	0.971473	0.685958	-0.19846	6.196831
CAMSAP1	0.971473	0.685991	-0.19973	6.59244
ZNF429	0.971473	0.685991	-0.35796	3.826592
CEACAM16	0.971473	0.686051	0.600978	1.338241
CCDC12	0.971473	0.686062	-0.66592	1.685722
EIF2D	0.971473	0.686132	-0.30022	3.79485
TCF7L1	0.971473	0.686198	-0.2732	4.634293
PREX1	0.971473	0.686263	0.191094	6.905745
MED17	0.971473	0.686327	-0.21439	5.509902
APTR	0.971473	0.686369	0.257255	3.841993
CDKN2C	0.971473	0.686534	-0.50764	2.301167
LOC100129	0.971473	0.686593	-0.55336	1.797444
PRICKLE2	0.971473	0.686625	-0.25469	5.51105
LLGL2	0.971473	0.686694	0.322864	4.134638
LINC01128	0.971473	0.686839	-0.41353	2.939377

ARV1	0.971473	0.686948	0.234368	4.782122
ZBTB44	0.971473	0.687035	0.223453	5.217135
RASSF5	0.971473	0.68704	0.438166	2.245255
MFN1	0.971473	0.687049	-0.18913	7.075694
CCDC115	0.971473	0.687202	0.470163	1.659044
KLHDC1	0.971473	0.687286	-0.32007	3.539389
AP5Z1	0.971473	0.687307	-0.19955	6.075453
TSR2	0.971473	0.687323	0.190349	5.74673
PPP1R37	0.971473	0.687335	0.192875	5.622362
ELOVL1	0.971473	0.687418	-0.80844	1.09463
MIR7849	0.971473	0.687506	0.639688	1.278175
LOC101928	0.971473	0.687566	-0.82418	0.680072
RADIL	0.971473	0.687591	-0.74189	1.113496
DOCK9-AS1	0.971473	0.687613	0.592705	0.872223
CLIC3	0.971473	0.687879	0.452771	2.619811
TMEM132E	0.971473	0.687905	-0.84724	1.253329
COA3	0.971473	0.687914	0.249543	4.321691
DNPEP	0.971473	0.687961	-0.21398	5.397685
LNPK	0.971473	0.687989	0.46657	1.384563
RBM6	0.971473	0.687998	0.168512	7.328086
LOC653712	0.971473	0.688054	0.40868	2.127929
HGS	0.971473	0.68806	-0.18835	7.201241
CHST10	0.971473	0.688088	-0.52934	2.884458
RPA4	0.971473	0.688102	0.403815	2.677959
PTAR1	0.971912	0.688548	0.164529	7.713015
SPRY4-AS1	0.971912	0.688626	0.549973	1.193844
RCSD1	0.971912	0.688643	0.233106	5.614384
SNORA73A	0.971912	0.688735	0.199653	9.102811
ABHD4	0.971912	0.68879	0.225011	4.981904
LYL1	0.971912	0.689173	0.420882	2.232969
CLN6	0.971912	0.689324	-0.2492	4.806825
TMED2	0.971912	0.689326	-0.19168	8.491254
ODC1	0.971912	0.68942	-0.6329	2.158698
OGN	0.971912	0.689424	-0.94266	0.937775
GDAP2	0.971912	0.689448	-0.22757	6.174921
SMC1B	0.971912	0.689453	-0.98941	0.715695
SPATA12	0.971912	0.689634	0.328777	3.442421
TOP3A	0.971912	0.689687	-0.25498	5.003497
MRPS5	0.971912	0.689756	-0.18896	6.564142
SPATA25	0.971912	0.689764	0.447212	1.853495
JAK1	0.971912	0.689767	-0.17935	8.360544
B4GALT7	0.971912	0.689803	0.238116	4.097947
S100A12	0.971912	0.689821	-0.86488	2.295813
KDF1	0.971913	0.689965	-0.48334	2.954932
IGFBPL1	0.971913	0.689969	-0.64974	1.903273

FRZB	0.971981	0.690092	-0.3485	4.1516
ATP5MC2	0.972009	0.690212	-1.3239	0.612086
SAMD1	0.972009	0.690282	0.197563	5.921381
FBXO27	0.972009	0.69053	0.544262	1.295004
NRROS	0.972009	0.69057	-0.48607	2.582269
DDX5	0.972009	0.690591	-0.17275	10.25915
SUGP2	0.972009	0.690725	0.189205	5.250443
NSMCE2	0.972009	0.690748	-0.23862	4.493145
NIPAL3	0.972009	0.690814	0.170792	6.198133
AHCYL1	0.972009	0.690899	0.387147	2.735369
HRH2	0.972009	0.691038	0.541925	1.658778
MAPK1	0.972009	0.691099	-0.18287	6.775282
ZBTB24	0.972009	0.691103	-0.28765	4.091274
FNIP2	0.972009	0.691231	-0.18924	6.49305
NEIL3	0.972009	0.691238	0.382806	2.890678
MLKL	0.972009	0.691483	0.257093	3.509848
BICDL1	0.972009	0.691494	-0.25453	5.112032
TPPP	0.972009	0.691533	0.222046	6.159475
USP34	0.972009	0.691538	-0.17863	9.212744
CNTNAP3B	0.972009	0.691602	-0.43634	3.718801
TMUB1	0.972009	0.691603	-0.38526	3.426295
AARS	0.972009	0.691685	0.165481	7.53982
PDCD10	0.972009	0.691809	-0.46866	3.029434
FAM107B	0.972009	0.691815	0.401255	1.784952
TIAF1	0.972012	0.69192	0.441992	1.992317
ANKRD52	0.972012	0.692005	-0.19567	7.184327
NDUFS1	0.972012	0.69204	0.462449	1.619743
PITPNA	0.972038	0.692249	0.161215	6.843676
C10orf25	0.972038	0.692257	-0.68058	2.033867
PPP1R26-A	0.972038	0.692294	0.386932	2.681171
SNORA10	0.972038	0.692415	-0.21642	6.392962
TP53I13	0.972038	0.692469	-0.27731	4.459826
PEX14	0.972038	0.692546	-0.23655	4.863289
CCDC184	0.972038	0.69262	-0.76679	0.661428
OR7E14P	0.972038	0.692711	0.439324	2.166324
SNORD36B	0.972038	0.692726	-0.51238	3.606088
CEP89	0.972348	0.69321	-0.23121	5.634076
TTC13	0.972348	0.693355	0.559207	1.072534
CLUH	0.972348	0.693371	0.182887	6.868476
FAM129A	0.972348	0.693522	-0.22012	7.731153
DDX1	0.972348	0.69367	-0.20379	7.237263
VEGFA	0.972348	0.693771	0.491356	2.337386
MIR616	0.972348	0.693848	0.582673	1.051883
CCDC106	0.972348	0.693917	0.352755	3.138654
ARHGEF6	0.972348	0.693923	0.638563	1.014606

STK38L	0.972348	0.694052	-0.17268	7.352244
GALK2	0.972348	0.694101	0.524727	0.74275
FDPSP2	0.972348	0.694147	0.179623	5.767691
CCDC87	0.972348	0.694169	0.616024	1.135264
ATP13A1	0.972348	0.694191	-0.19569	6.622914
FAM98C	0.972348	0.694232	0.218442	4.106725
TRIP6	0.972348	0.694283	-0.19995	6.247109
VCP	0.972348	0.694286	0.161322	8.390043
GID8	0.972348	0.694382	0.16855	6.93732
THOP1	0.972348	0.694402	-0.29744	5.075405
CADPS2	0.972348	0.694428	-1.12254	0.723484
BIN3	0.972477	0.694595	-0.20965	5.642832
HRC	0.972601	0.694757	-0.49024	2.066097
PLS3	0.972691	0.694896	0.547811	1.170618
TMCC3	0.972908	0.695127	-0.80602	1.562863
SLC31A1	0.972908	0.695199	0.191828	6.411396
RBM33	0.973081	0.695474	-0.18206	8.797444
LCAT	0.973081	0.695534	0.321273	3.952754
WDR74	0.973081	0.695596	0.533659	0.734038
PARTICL	0.973081	0.69562	0.5066	1.423001
PLIN5	0.973125	0.695862	-0.49584	2.357934
MIR553	0.973125	0.695924	-0.69248	1.678739
ZXDB	0.973125	0.696106	0.195866	5.073418
LOC44043[illegible]	0.973125	0.696227	-0.22931	5.812513
ITGB7	0.973125	0.696313	0.492042	1.746622
UCK2	0.973125	0.696321	0.188226	5.8649
DCK	0.973125	0.696331	0.213971	5.098236
KLRB1	0.973125	0.696346	0.455792	1.552373
SMG1P5	0.973125	0.696385	0.260295	3.261289
AKIRIN1	0.973125	0.696459	0.377096	2.561904
HS3ST5	0.973125	0.696469	-0.85548	1.796218
ACTR1A	0.973125	0.696625	-0.19493	6.458225
ZNF721	0.973125	0.696673	-0.19021	6.771814
POLR2C	0.973125	0.696689	0.168125	5.740098
CASTOR1	0.973818	0.697259	-0.60224	1.999355
CD24	0.973905	0.697473	-0.5362	2.660758
TRMT1L	0.973905	0.697653	-0.61725	1.792033
DNAH3	0.973905	0.697701	-0.37751	3.853267
RFK	0.973905	0.697718	-0.27288	5.401266
ELAVL1	0.973905	0.697862	-0.19684	6.272376
BEND6	0.973905	0.697904	-0.75647	1.592012
SNORD49A	0.973905	0.698012	-0.40094	3.515349
DPYSL4	0.973905	0.698131	0.39721	2.473418
NICN1	0.973905	0.698179	0.216917	4.537249
TRABD2B	0.973905	0.698295	0.351511	3.56447

KATNA1	0.973905	0.69845	0.3543	2.605524
FMO5	0.973905	0.698544	-0.52221	3.468471
SAXO2	0.973905	0.698545	-0.9951	1.18401
RPL34-AS1	0.973905	0.698546	0.446623	2.140143
PLEKHA8P1	0.973905	0.698628	-0.37623	3.412964
HOOK3	0.973905	0.69872	-0.17138	8.247228
CNIH3	0.973905	0.698823	-0.68175	1.224562
EDEM1	0.973905	0.698828	0.163358	7.240102
DNHD1	0.973905	0.69887	0.197645	6.147371
UBE2MP1	0.973905	0.699085	-0.63766	1.686778
ZNF611	0.973905	0.699147	-1.1028	0.904648
FAM227B	0.973905	0.699157	-0.56897	1.776547
SPON1	0.973905	0.699178	0.19552	7.250389
WNT6	0.973905	0.699231	-0.89386	1.342681
APEX2	0.973905	0.699232	0.453695	1.521174
BCHE	0.973905	0.699353	-0.66201	3.029518
LARP4B	0.973905	0.699356	-0.18869	7.794498
SQOR	0.973905	0.699401	-1.13551	1.122638
KIF22	0.973948	0.699517	0.528011	1.095703
OR10AD1	0.973948	0.69958	-0.91516	0.779824
LYRM4-AS1	0.974137	0.69996	0.350056	2.824289
DLEU2L	0.974137	0.699979	-0.58152	2.63593
EXOC4	0.974137	0.699997	-0.20436	5.802117
CDKAL1	0.974137	0.700054	-0.19442	5.922053
JAM3	0.974137	0.700087	-0.86018	1.272686
ACADM	0.97422	0.7003	0.615841	1.226715
SLC25A17	0.97422	0.700338	-0.37912	2.979669
IGSF22	0.97422	0.700369	-0.42325	2.69641
MIR3916	0.974429	0.700594	-0.31563	4.184994
SSH2	0.97446	0.700777	-0.46473	3.049456
CCNK	0.97446	0.700902	0.17228	6.243136
PCOLCE	0.97446	0.70098	0.192504	4.757397
PIK3R1	0.97446	0.701022	0.191368	6.376737
MRPS31	0.97446	0.701037	0.194167	4.839191
PPP1R16A	0.97446	0.701143	-0.29573	4.647741
DNAH1	0.97446	0.701219	-0.21118	6.82702
SELENOS	0.97446	0.701234	0.254705	4.066462
LTB4R	0.97446	0.701284	0.297676	4.619686
GPC4	0.974476	0.70139	-0.26816	5.815566
PRKCA	0.974476	0.701528	0.16445	7.519152
ZNF814	0.974476	0.701593	-0.19217	5.791127
TUBA1B	0.974476	0.701638	-0.18752	8.013217
BTN1A1	0.974476	0.701667	0.533373	1.201249
LINC00926	0.974486	0.701749	0.292818	3.142828
FLJ44635	0.974501	0.701891	0.346901	2.785

THAP12	0.974501	0.702021	-1.01917	1.120329
NUP50-DT	0.974501	0.702039	-0.56032	1.775128
DSCAML1	0.974501	0.702066	-0.50579	2.709667
ITIH2	0.974501	0.702203	-0.69833	1.643961
EML6	0.974501	0.70231	-0.25657	5.12763
BUB3	0.974501	0.702393	-0.20982	5.31184
HSD17B3	0.974501	0.702401	-0.61061	1.786491
LOC101927	0.974501	0.702428	0.532807	1.123179
FAF2	0.974642	0.70263	-0.18871	6.554449
DDTL	0.974642	0.70275	0.341421	2.84889
COL4A2-AS	0.974642	0.702752	-0.77023	1.25848
TPCN2	0.974853	0.703003	0.173205	6.064766
SPRR2D	0.974853	0.703172	0.683946	4.700656
MTHFSD	0.974853	0.703241	0.611316	0.967036
C19orf18	0.974853	0.703268	-1.08985	0.758707
PLGRKT	0.974853	0.703338	0.240828	3.679285
DHX32	0.974853	0.703351	-0.19038	5.910601
MBNL1	0.974892	0.703503	-0.3179	3.712489
DCAF12	0.974892	0.703548	-0.19868	6.380048
FAM183A	0.974892	0.70366	0.588009	0.798423
FBXO7	0.974892	0.703747	0.375943	2.399049
CBX1	0.974892	0.703754	0.421942	1.998371
SNCAIP	0.974892	0.703911	0.520859	1.298782
OSTM1	0.974892	0.70394	-0.1926	5.572589
SPTA1	0.974892	0.704064	0.506735	1.890148
SLC35G6	0.974892	0.704111	-0.66342	1.026013
SUB1	0.974892	0.704122	0.174593	6.961516
RAB33B	0.974973	0.704254	-0.22634	4.817651
PEX3	0.975117	0.704435	-0.23296	4.576651
TIMM21	0.975117	0.704513	-0.26667	4.582733
RASIP1	0.975117	0.704583	0.264322	3.613848
KCNB2	0.975117	0.704655	-0.84506	1.467711
ATP6V1B2	0.975501	0.705007	-0.17973	6.525844
HOXA6	0.975733	0.705296	-1.05538	0.969099
TAGLN2	0.975733	0.705341	-0.46342	2.175112
SOCS2	0.975733	0.705425	-0.5993	2.198149
ZNF727	0.975733	0.705473	-0.76954	1.580905
OAT	0.975796	0.705599	0.334966	2.98418
SLC6A16	0.975796	0.70573	-0.40512	3.369617
LINC01559	0.975796	0.705741	0.309338	4.843277
FBXO33	0.97584	0.705847	0.211183	5.034545
ATP6V1D	0.976122	0.706126	-0.21658	5.369408
EDRF1	0.976212	0.706304	0.540814	1.413461
SCRG1	0.976212	0.706472	0.415273	2.289704
ZNF767P	0.976212	0.706478	0.187935	5.079281

LHCGR	0.976212	0.706489	0.519847	2.154385
ZDHHC20	0.976387	0.706895	-0.8159	0.941863
LOC10192€	0.976387	0.7069	0.292933	4.567183
CDK8	0.976387	0.706935	-0.2539	5.15279
ALOX12	0.976387	0.706971	0.230314	4.344543
RPS4X	0.976387	0.707029	-0.20405	9.75255
RPSAP9	0.976387	0.707176	0.484859	2.182338
ANKMY2	0.976387	0.707264	0.171691	5.818045
PCP4L1	0.976387	0.707335	0.475077	1.437362
GNB1L	0.976387	0.707343	-0.59446	1.755814
RFT1	0.976387	0.70744	-0.22292	4.861899
MLLT10	0.976387	0.707493	0.472717	1.514418
PKDREJ	0.976387	0.707549	-0.36659	2.626507
RRAS2	0.976387	0.707587	0.477585	0.749822
WDR36	0.976387	0.707682	-0.18073	6.204989
CTU1	0.976387	0.707811	-0.49165	2.430937
CYB5RL	0.976387	0.707817	0.259108	3.731653
KLHL36	0.976387	0.707881	-0.50829	1.300409
ACKR2	0.976392	0.707982	0.346791	2.160899
TUBGCP6	0.976392	0.708033	0.163282	7.160062
EEF1D	0.976457	0.708154	-0.93823	1.133685
LUM	0.97648	0.708296	-0.23605	8.306761
COL20A1	0.97648	0.708356	-0.82108	1.551055
DUSP1	0.97648	0.708394	-0.19857	8.129381
RUNDC1	0.976498	0.708482	-0.18203	6.268826
APC2	0.976584	0.708649	-0.28559	4.39728
LARP7	0.976584	0.708693	0.427497	1.878506
TULP4	0.976593	0.708774	-0.19302	5.447773
FBXL17	0.976926	0.70924	0.175446	5.424516
SRP19	0.976926	0.709244	-0.24106	4.900907
DNAH17	0.976926	0.709291	0.235989	3.645564
LINC01547	0.976926	0.709314	0.411678	2.101815
SH2D3A	0.976938	0.709397	0.179135	5.55823
IL13RA2	0.977029	0.709545	-0.84957	0.886015
ITGB5	0.977029	0.709619	-0.1859	7.290344
PLEKHG3	0.977029	0.709814	-0.20383	6.434495
AKIRIN2	0.977029	0.709937	-0.23527	5.168719
PRKAR1A	0.977029	0.71007	0.315087	2.243655
TMEM60	0.977029	0.710233	-0.37095	3.185962
LOC10013(	0.977029	0.710266	-0.27487	4.972031
LPAR2	0.977029	0.710301	0.225344	3.915903
C9orf78	0.977029	0.710306	0.162737	6.55129
ARHGEF38-	0.977029	0.710412	0.309197	3.071116
IFT80	0.977029	0.71042	-0.56918	1.703314
HEXIM1	0.977029	0.710431	-0.17944	6.747357

METAP1	0.977029	0.710525	0.170985	6.104384
LOC100131	0.977029	0.710534	0.491263	1.963179
LOC100506	0.977029	0.71058	-0.30503	3.735582
RNF223	0.977229	0.7108	-0.68502	1.943861
PARP8	0.977307	0.710932	-0.69353	1.204982
RBM47	0.977358	0.711043	0.209117	4.585972
SDHAP1	0.977395	0.711215	-0.17993	5.926983
PIK3R4	0.977395	0.711274	-0.20329	5.80729
CD99P1	0.977395	0.711381	-0.79748	1.126546
ABTB2	0.977395	0.711473	-0.23529	4.939826
HSD17B7	0.977395	0.711652	-0.62435	2.363178
NUTM2A	0.977395	0.711827	0.467818	1.655948
GABARAPL	0.977395	0.711924	-0.18987	5.381613
PPARD	0.977395	0.711945	-0.78949	1.747696
NACC1	0.977395	0.712032	-0.1998	6.396502
PLEKHG5	0.977395	0.7121	0.485357	0.930079
CALM2	0.977395	0.712172	0.157787	5.992914
ATXN2L	0.977395	0.712363	0.328696	2.557657
FANCG	0.977395	0.712477	-0.27592	4.196957
DYNC1LI1	0.977395	0.712491	0.158939	6.163035
CPPED1	0.977395	0.712579	-0.57713	2.386229
TMPO-AS1	0.977395	0.712641	-0.73911	1.164873
MIR4804	0.977395	0.71266	0.643876	0.740004
NUP37	0.977395	0.712684	-0.3146	3.72997
ME2	0.977395	0.712702	0.626669	0.98853
RNVU1-6	0.977395	0.712729	-0.74779	1.120534
RAB25	0.977395	0.712835	-0.67968	1.547278
COX20	0.977395	0.712841	-0.51409	2.294128
DIPK2A	0.977395	0.712841	0.385659	1.98294
LOC100506	0.977395	0.712902	-0.34248	3.494378
INF2	0.977395	0.713022	-0.32899	3.380684
SNORD36A	0.977395	0.71315	-0.27459	6.369595
LENG8	0.977395	0.713173	-0.19648	6.141817
ERO1A	0.977395	0.713217	0.211269	7.781555
C1orf123	0.977395	0.713233	0.423324	2.087143
TMEM100	0.977395	0.713305	-1.05182	0.784499
RAB27A	0.977575	0.713569	0.34047	2.281621
PRRT3-AS1	0.977575	0.713585	-0.52634	1.755401
ZNF607	0.977603	0.713698	-0.7589	1.165908
RING1	0.977603	0.713754	-0.58894	1.897923
MAPRE2	0.977669	0.713877	0.482587	1.388019
FFAR2	0.977725	0.714048	0.554385	1.488742
LOC100129	0.977725	0.714135	0.409292	1.85437
LINC01530	0.977725	0.714141	-0.65261	1.192683
C1QL3	0.977799	0.714323	-0.59802	2.337623

PTEN	0.977799	0.714366	0.330283	2.864458
OXCT2P1	0.977799	0.714419	-0.55376	2.285744
CTTNBP2	0.977955	0.714638	0.271444	4.018287
LOC101927	0.977955	0.714742	-0.51187	2.619044
FBXW11	0.977955	0.714757	0.414361	1.678108
BAZ2B	0.978075	0.715073	-0.41351	3.270364
MLLT11	0.978075	0.715234	0.337374	3.225259
LINC00852	0.978075	0.715363	0.382345	2.603158
LOC283856	0.978075	0.715433	0.679441	0.780767
GSTP1	0.978075	0.715559	0.188202	7.676447
LOC101929	0.978075	0.715768	-0.81943	1.132152
ZNF529	0.978075	0.715808	-0.47246	2.96149
METAP1D	0.978075	0.715946	-0.25687	3.861586
SLC13A4	0.978075	0.716062	0.223169	4.629617
LINC01145	0.978075	0.716109	0.5388	1.043806
GLIS3	0.978075	0.716139	0.237776	3.784638
RPS6KA2	0.978075	0.716182	0.263341	3.673919
MALAT1	0.978075	0.716203	-0.17267	16.77965
FAM27B	0.978075	0.716266	0.371645	2.507286
ARHGAP32	0.978075	0.716273	0.193507	4.91091
C12orf45	0.978075	0.716316	-0.31059	3.818155
PLEKHG4	0.978075	0.716328	-0.70094	1.433586
DHTKD1	0.978075	0.716386	0.172488	5.919657
IFT140	0.978075	0.71643	0.20832	6.375466
RPS28	0.978075	0.716451	-0.29031	5.117198
TMC2	0.978075	0.716461	-0.57562	1.901498
HLA-B	0.978075	0.716485	0.155172	8.859944
CCDC146	0.978097	0.716648	-0.21458	5.328406
ACAT1	0.978097	0.716665	-0.20477	5.603782
FBXL20	0.978097	0.716774	-0.86908	0.68446
RNASE7	0.978097	0.716799	0.492709	2.037628
PDIA3	0.978097	0.716968	0.155208	8.509322
UBE3A	0.978097	0.717165	0.51561	1.796815
NSMF	0.978097	0.717395	-1.17765	0.77507
EAF2	0.978097	0.717408	0.322633	2.745705
TNIK	0.978097	0.717507	0.437707	2.284083
DISC2	0.978097	0.717746	0.474366	1.352189
LOC100506	0.978097	0.717796	-0.53328	2.685044
USP35	0.978097	0.717931	-0.20651	4.974461
NAIP	0.978097	0.717951	-1.01218	1.029701
SPG7	0.978097	0.71798	-0.19002	5.841664
OR51E1	0.978097	0.718079	0.595538	1.704066
CAPN15	0.978097	0.718248	0.159962	6.764398
WDR81	0.978097	0.718271	0.27244	4.128419
HERC2P3	0.978097	0.718384	0.167898	5.576429

PDXK	0.978097	0.718414	-0.1785	7.775753
USP17L7	0.978097	0.718431	-0.80879	0.798342
FBXL22	0.978097	0.718433	0.408887	2.062038
NXPH3	0.978097	0.718506	-0.37422	3.405416
PHF14	0.978097	0.718575	0.158499	7.038506
ZDHHC8P1	0.978097	0.718694	-0.76074	0.80329
BAX	0.978097	0.718821	0.472212	0.733269
SMURF2P1	0.978097	0.719111	-0.18641	5.30732
LNCOC1	0.978097	0.719127	-0.87089	1.084696
KLC4	0.978097	0.71925	0.500635	1.06965
CUTC	0.978097	0.719397	-0.29325	4.165261
FAM168B	0.978097	0.719431	-0.15772	7.480029
ERICH5	0.978097	0.719465	0.330108	2.318721
TCF3	0.978097	0.719487	-0.35933	3.750763
TAF13	0.978097	0.719497	0.197644	4.792415
RPAP2	0.978097	0.719623	0.167662	5.76118
LIPH	0.978097	0.7197	0.153381	6.670292
SAYSD1	0.978097	0.719807	-0.24938	4.056157
NEIL1	0.978097	0.719926	0.432228	1.662488
SNORD43	0.978097	0.719989	-0.53795	1.988308
DNAJC12	0.978097	0.720216	-0.74703	0.95107
KIAA1551	0.978097	0.72024	-0.18323	8.147292
MAP3K1	0.978097	0.720483	-0.18308	7.013967
SGPP2	0.978097	0.720588	0.182823	5.284156
PCNX1	0.978097	0.720591	-0.34634	2.904745
LOC100422	0.978097	0.7206	0.506175	1.603489
EIF2B2	0.978097	0.720693	-0.23693	4.684953
FPGT	0.978097	0.720712	-0.52596	2.095886
SCAND2P	0.978097	0.72082	0.333585	2.296462
UBE2U	0.978097	0.720834	0.497491	2.1284
NECAP1	0.978097	0.720874	-0.69771	1.501903
HAS2-AS1	0.978097	0.720948	-0.60608	1.474385
WDR64	0.978097	0.720963	0.631177	0.873331
SMS	0.978097	0.720994	-0.41028	2.645368
LINC00628	0.978097	0.721003	-0.59581	1.939718
ZFPL1	0.978097	0.721068	-0.23789	4.480755
ITFG2	0.978097	0.721196	0.219977	4.977222
CHRNG	0.978097	0.721224	-0.32157	3.46689
MCM3AP	0.978097	0.721255	0.144164	7.76245
PRR13	0.978097	0.7213	-0.5704	2.039525
LINC00921	0.978097	0.721318	-0.32321	3.1707
A4GNT	0.978097	0.721371	-0.60895	3.384433
LINC01176	0.978097	0.721543	-0.33523	3.295505
NCBP1	0.978097	0.721543	-0.16331	6.960894
ORM1	0.978097	0.721667	0.598948	0.856626

PCMTD1	0.978097	0.721714	-0.19798	5.361643
TBCK	0.978097	0.721844	0.345861	2.412119
LOC101928	0.978097	0.721867	0.328466	3.359784
CNTN2	0.978097	0.721972	0.3362	2.029999
SEC61A1	0.978097	0.721997	0.15027	8.121901
L3MBTL1	0.978097	0.722013	-0.80179	0.626125
RHOT2	0.978097	0.722013	0.162704	6.472487
LARS2	0.978097	0.722026	-0.19569	5.274068
GNA13	0.978097	0.722058	-0.22306	5.041716
ALKBH3-AS	0.978097	0.722224	-0.68386	2.296235
LZTS3	0.978097	0.722287	-0.25601	6.321968
KLHDC7A	0.978097	0.722316	0.28624	2.520793
CAMLG	0.978097	0.722397	-0.21098	4.685212
SPPL3	0.978097	0.722434	-0.18224	5.903784
IREB2	0.978097	0.722461	-0.18868	6.812507
LINC01605	0.978097	0.722543	0.470666	1.311847
GKN2	0.978097	0.722598	-0.71456	3.773473
CFAP126	0.978097	0.722606	-0.2371	5.019944
ZDHHC18	0.978097	0.722641	-0.21319	5.100015
ALX3	0.978097	0.722698	0.339346	2.500021
SELENOW	0.978097	0.722768	0.165257	6.501096
RPL37	0.978097	0.722837	0.152934	8.210336
LOC100303	0.978146	0.722949	-0.43104	2.542836
PLCB2	0.97824	0.723164	0.492736	1.393963
CTBP2	0.97824	0.723222	-0.22463	5.06034
DGAT2L6	0.97824	0.723242	0.529148	1.35012
RELL1	0.978279	0.723345	0.17522	5.474219
AGMO	0.978386	0.72353	0.419425	1.659787
GLUD2	0.978386	0.723591	-0.44261	2.630568
RAD9A	0.978386	0.723648	-0.51143	2.340284
PRTG	0.978443	0.723842	0.273155	3.702681
NOC4L	0.978443	0.723849	-0.262	3.808791
FCER1A	0.978443	0.724014	-0.79884	0.948691
HSCB	0.978443	0.724048	0.240104	3.759771
CLEC11A	0.978443	0.724063	-0.2866	3.541756
GARS-DT	0.978511	0.724188	0.17477	6.012017
PTRH1	0.978586	0.724318	-0.46427	2.545562
CMC4	0.978668	0.724487	0.371137	2.032768
TRMT9B	0.978668	0.724575	-0.84877	1.330069
TMEM50A	0.978668	0.724656	0.155399	6.039367
NOL6	0.978668	0.724677	-0.27644	4.307864
SERTAD1	0.978719	0.724789	0.240175	3.252755
DSG3	0.978827	0.725044	0.418151	7.694672
TOMM7	0.978827	0.725053	-0.21932	6.37478
RUNX1T1	0.978827	0.725093	-1.05802	0.916773

MSRB3	0.979014	0.725306	-0.6252	1.191817
DDRGK1	0.979056	0.725588	0.163243	6.923488
COMTD1	0.979056	0.725597	0.234147	3.692602
NEXN-AS1	0.979056	0.725623	-0.49048	2.35196
TOR1A	0.979056	0.7257	0.177721	5.147173
TOM1L1	0.979056	0.72571	-0.19105	5.977213
LOC10192[illegible]	0.979078	0.725819	-0.76526	2.119732
WARS2-IT1	0.979078	0.725908	0.457838	1.615138
ZFPM2	0.979078	0.726009	-0.28469	4.976389
FASTKD5	0.979078	0.726025	-0.23793	4.769226
FGFBP1	0.979147	0.726284	-0.67473	2.949937
MIR4269	0.979147	0.726354	-0.99061	0.724282
JAGN1	0.979147	0.726366	0.275684	4.314928
TMOD4	0.979147	0.726375	-0.63062	1.707212
TPI1	0.979964	0.727065	-0.60863	2.276952
HOXC8	0.979964	0.72713	-0.33967	3.871427
SULT1A2	0.980036	0.727362	0.564337	0.974044
CLDN18	0.980036	0.727366	-0.54229	3.56941
IFI35	0.980036	0.727408	-0.22389	4.848603
SSH1	0.980149	0.727588	-0.17482	6.35526
RRP1	0.980149	0.727641	-0.23176	5.508389
PSMA3-AS[illegible]	0.980262	0.727913	0.338469	2.147321
CPED1	0.980262	0.727954	-0.28132	5.135202
LINC01589	0.980262	0.728171	0.534272	1.907304
EPC1	0.980262	0.728217	0.334771	1.994151
CBWD2	0.980262	0.728225	-0.19096	5.327377
RGPD2	0.980262	0.728231	0.530873	0.776783
MIR647	0.980262	0.728248	0.471565	1.847675
ESCO1	0.980342	0.728382	-0.19401	6.897319
MYO1F	0.980441	0.728568	-0.23516	5.854769
ZNF770	0.980441	0.728643	0.148855	6.711444
PABPC4L	0.980441	0.72868	-0.6664	2.235136
SCTR	0.980467	0.728837	-0.69902	2.243945
LIPE	0.980467	0.72895	-0.33726	3.656121
FAM171B	0.980467	0.729041	-0.42894	3.682877
NXPE3	0.980467	0.729241	-0.61877	1.437084
C15orf40	0.980467	0.729319	-0.46103	2.053444
LOC28507[illegible]	0.980467	0.72932	0.160216	5.547325
UNC93B1	0.980467	0.729534	-0.18781	6.3202
NLRP14	0.980467	0.72961	0.343197	1.315708
SSNA1	0.980467	0.729621	-0.2344	4.330681
SPRR2C	0.980467	0.729644	0.624905	1.809916
PLA2G4A	0.980467	0.729657	-0.73391	1.107125
SLC5A6	0.980467	0.72972	0.413219	1.672414
TMEM248	0.980467	0.72986	-0.16142	7.757931

AMIGO3	0.980467	0.729882	-0.29468	3.892868
TADA1	0.980467	0.729992	-0.35428	4.290308
LYRM1	0.980467	0.730001	-0.71273	1.339648
GPATCH2L	0.980467	0.730045	-0.82742	0.967114
APOBEC3G	0.980467	0.730066	-0.29823	3.62782
MED28	0.980467	0.730119	0.170794	6.147198
NRSN2-AS1	0.980703	0.730491	0.322807	3.156492
ERAP1	0.980703	0.730588	-0.2535	4.459823
UCN2	0.980703	0.730632	-0.65341	1.807555
RNF166	0.980703	0.730652	-0.6703	1.585535
SNORD142	0.980703	0.730782	-0.55306	1.555528
RHOA	0.980703	0.730839	-0.7002	0.996883
FOCAD	0.980703	0.730979	-0.17863	5.909739
DDX27	0.980703	0.731009	-0.16384	7.589837
CCL19	0.980703	0.731025	-0.70562	1.568537
BST1	0.980703	0.731102	0.366397	1.71719
SLU7	0.980703	0.731156	-0.15577	6.959985
NPM3	0.980703	0.731192	0.274012	3.804943
LINC01819	0.980713	0.73144	-0.59695	5.065851
ABCC5-AS1	0.980713	0.731476	-0.60145	2.221348
PTOV1-AS2	0.980713	0.731565	0.178178	4.675334
RGCC	0.980713	0.731566	0.218871	4.287969
IPO7	0.980713	0.731646	-0.15231	8.237485
GOLT1B	0.980713	0.731746	-0.22836	5.578128
RPL28	0.980713	0.731998	0.258168	3.142789
LINC00992	0.980713	0.732091	-0.93605	1.414998
PTGER2	0.980713	0.732102	0.302526	3.645666
FAM157C	0.980713	0.732129	0.231999	3.80999
WHAMMP:	0.980713	0.732228	0.163085	5.413438
LOC101927	0.980713	0.732245	0.42393	1.849792
KMT5C	0.980713	0.732375	-0.27226	4.124064
PHLDB3	0.980713	0.73241	0.187481	4.875544
ZMYND8	0.980713	0.732428	0.321988	2.588983
FAM126A	0.980713	0.732444	0.158419	5.942587
UBE2O	0.980713	0.732502	-0.1714	6.41617
CPOX	0.980713	0.732545	0.163414	5.110012
SPA17	0.980806	0.732755	0.373187	2.565329
KLF7	0.980806	0.732764	-0.52043	2.552419
LRRC47	0.980851	0.732882	-0.17772	6.000297
STX17	0.980851	0.732962	0.150219	6.949555
NARS	0.980851	0.733081	-0.19038	8.294709
SNORD24	0.980851	0.733096	-0.2944	3.677086
TRIM13	0.980871	0.733428	0.465464	1.480833
RPS6KA2-A	0.980871	0.733441	0.489745	1.605966
HSD17B1	0.980871	0.733489	-0.32051	3.641361

BCRP2	0.980871	0.733506	0.388996	1.507025
MYH9	0.980871	0.733591	0.133853	11.67027
ANKRD12	0.980871	0.733614	-0.54663	2.343084
LINC00115	0.980871	0.733635	0.216337	3.582474
CLPTM1L	0.980872	0.73378	0.169402	5.2075
DBN1	0.980872	0.733859	0.369819	1.758171
GPAA1	0.980872	0.73386	-0.1885	5.445569
SASH3	0.980914	0.733966	0.221467	4.30107
LHFPL3-AS1	0.980979	0.734174	-0.58055	3.826063
HEATR5A	0.980979	0.734365	0.147245	6.549751
ZCCHC7	0.980979	0.7344	0.56484	0.797892
MAP3K4	0.980979	0.734404	-0.50598	2.830744
MNX1	0.980979	0.734464	-0.61623	1.634582
FBXO10	0.980979	0.734737	-0.32975	4.063705
NAA20	0.980979	0.734779	-0.65781	1.366337
CCDC18	0.980979	0.734914	-0.61853	1.382975
FBXW2	0.980979	0.734917	0.14172	7.335015
INTS6	0.980979	0.735033	0.172856	4.678321
SNORA80E	0.980979	0.73506	-0.21786	8.732857
TMEM176B	0.980979	0.735118	0.424504	0.75546
ITSN1	0.980979	0.735147	-0.33681	4.4283
KCNMA1-A	0.980979	0.735152	-0.57987	2.63382
ZNF491	0.980979	0.735159	0.392959	2.561837
ASF1B	0.980979	0.735211	0.25827	3.606167
ADIPOR2	0.980996	0.735306	0.152727	6.759021
KIN	0.980996	0.735373	-0.50761	2.126612
CNTN3	0.981017	0.735464	-0.54628	3.133261
HS6ST1	0.98108	0.735614	0.177616	5.047629
ABCF3	0.98108	0.735768	-0.21454	6.021341
STRN3	0.98108	0.735792	0.226871	3.493361
NKAP	0.98108	0.735864	-0.18096	5.386194
SPRYD4	0.98108	0.735884	0.228945	3.829508
HPCA	0.98113	0.736107	-0.72088	1.296659
WWC2-AS1	0.98113	0.736209	-0.6559	1.532542
ZFP69B	0.98113	0.736229	-0.34216	3.413738
RDH8	0.98113	0.736273	-0.91359	0.793462
SPCS2	0.98113	0.736315	-0.18093	6.280347
MUC4	0.98113	0.736371	-0.37174	9.154269
TLR6	0.981189	0.736562	-0.26887	4.099442
EIF2S1	0.981189	0.736656	-0.17513	6.41907
SLC51A	0.981189	0.736736	0.355702	2.277386
CA11	0.981189	0.736843	-0.30078	4.058019
LINC00501	0.981189	0.736919	-0.81	1.400314
RDH12	0.981189	0.737059	0.453412	1.41655
UBE2G1	0.981189	0.737088	0.163645	5.850548

DLEU1	0.981189	0.737095	-0.32914	3.862477
YIPF4	0.981189	0.737136	-0.1622	6.314332
TMEM147-	0.981189	0.73727	-0.34682	2.84339
TMCO4	0.981189	0.737443	0.17181	5.386333
EFCAB12	0.981189	0.737449	-0.60988	0.988728
SPATA21	0.981189	0.737482	0.475607	0.950855
VPS26A	0.981189	0.7375	-0.30546	3.14109
C12orf49	0.981189	0.737537	0.15309	6.148766
NSMCE1	0.981273	0.737744	-0.20168	5.237963
EDRF1-AS1	0.981273	0.73775	-0.38024	2.026698
SNORA74A	0.981286	0.737899	0.169238	6.65746
DISP1	0.981286	0.737909	-0.24076	4.552048
BSDC1	0.981289	0.738015	0.198258	4.337332
ZNF229	0.981289	0.738224	-1.0233	0.907041
ASCC1	0.981289	0.738246	-0.40999	2.509755
CMTM8	0.981289	0.738346	0.296166	3.537363
C5orf67	0.981289	0.73848	0.503171	1.477861
SNAP23	0.981289	0.738546	-0.56294	0.862183
VRK3	0.981289	0.738564	-0.42074	1.610469
ZNF561-AS	0.981289	0.738589	0.243814	3.255486
CDCA7	0.981289	0.73862	-0.50114	2.029165
EPG5	0.981289	0.738841	-0.15816	6.985343
TUBBP5	0.981289	0.738865	0.381461	2.037466
GPR65	0.981289	0.738885	-0.43623	2.539397
GLE1	0.981289	0.739002	-0.22984	4.66391
FBXW5	0.981289	0.7391	-0.17408	6.117865
PSMD13	0.981289	0.739118	0.447758	1.123452
RALGAPA1	0.981289	0.739281	0.142587	6.589124
NDUFAF4P	0.981289	0.73947	-0.58709	1.677398
FAM131A	0.981289	0.739654	-0.40992	2.459893
TIMM50	0.981289	0.739658	0.158735	5.097101
NOL4L	0.981289	0.739722	0.49518	0.723883
CELSR2	0.981289	0.739768	0.193184	5.287454
ENC1	0.981289	0.739843	0.472011	0.956245
LOC10334	0.981289	0.73989	0.202596	4.455699
DEAF1	0.981289	0.739972	0.466586	0.732827
EIF4E2	0.981289	0.740008	-0.22554	4.086173
SNORD3A	0.981289	0.740084	0.170804	10.88602
HK2	0.981289	0.740134	-0.21012	7.330184
MAP1LC3B	0.981289	0.740161	-0.21168	6.210084
CRAT37	0.981289	0.740272	-0.7746	3.10898
NRXN3	0.981289	0.740405	0.555584	0.895222
INHBE	0.981289	0.740446	-0.72706	1.459583
MRPS28	0.981289	0.740501	-0.28001	3.984211
TDP2	0.981289	0.740564	0.151475	5.810697

FAM149B1	0.981289	0.74069	-0.18889	5.352203
TOP1MT	0.981289	0.740694	0.385263	2.031738
RAB28	0.981289	0.740717	-0.75164	1.334021
F11R	0.981289	0.740748	-0.15977	8.158173
HSD17B6	0.981289	0.740754	0.411721	2.722386
LOC101929	0.981428	0.741018	-0.65431	0.84729
TUT1	0.981428	0.74105	-0.20737	5.194418
ETS2	0.981428	0.741083	0.391062	1.40061
SLC31A2	0.981467	0.74124	-0.25852	4.154259
ANKRD27	0.981467	0.741295	-0.15123	6.459114
TMEM267	0.981467	0.741418	-0.31763	3.343101
SCGB2B3P	0.981467	0.741471	0.472145	1.346
LCE3D	0.981467	0.741492	0.598908	0.717322
RBM3	0.981467	0.741632	0.138954	7.594048
NSMAF	0.981467	0.741733	0.334336	1.467642
SNORD38A	0.981467	0.741796	-0.29901	5.442026
RPS16P5	0.981467	0.741854	-0.37191	4.224021
FGFR1OP	0.981467	0.741871	0.301047	3.155382
S100A2	0.981467	0.741935	0.387562	5.787679
KCND1	0.981546	0.74207	-0.38079	3.13151
ZNF622	0.981601	0.742234	0.141465	5.708701
ADNP	0.981601	0.742261	-0.46559	2.879193
GAS8-AS1	0.981617	0.742374	0.30757	2.193423
MAP3K11	0.981617	0.742422	0.142776	6.054722
MRPL32	0.9819	0.742732	-0.21147	5.17363
ZC3H4	0.9819	0.742949	-0.15928	7.434994
POLR2K	0.9819	0.74295	-0.19394	4.865772
C17orf51	0.9819	0.743006	-0.26724	4.724524
PFN1	0.9819	0.743514	0.144936	8.073678
CFAP100	0.9819	0.743515	-0.54378	2.687352
CNPY2	0.9819	0.743585	0.169453	4.914207
FOXP4-AS1	0.9819	0.743623	0.326173	2.380654
CCZ1P-OR7	0.9819	0.743636	-0.21336	4.812235
EMC2	0.9819	0.743687	-0.16304	6.028652
NOP53-AS1	0.9819	0.743756	0.36937	1.77485
LOC339059	0.9819	0.743794	0.314633	2.449739
FKBP11	0.9819	0.743828	-0.44522	2.205615
SMCR8	0.9819	0.743838	0.143079	6.53789
ARRDC5	0.9819	0.743898	0.285345	2.666137
AR	0.9819	0.743899	0.401279	1.601207
TWF2	0.9819	0.743953	0.15516	5.351245
NSUN7	0.9819	0.743984	0.228159	3.546945
UBAP1	0.982167	0.744261	-0.37153	2.440462
GPR27	0.982277	0.74442	0.562185	0.839788
MAP3K5	0.982277	0.744527	0.135331	6.812155

TAF10	0.982277	0.744569	0.150077	5.394573
GCNT4	0.982392	0.744731	-0.45328	3.119891
MNDA	0.982447	0.744966	0.290146	3.294163
UBR4	0.982447	0.744974	-0.14778	9.650934
RTRAF	0.982447	0.745074	0.141508	6.252931
BCYRN1	0.982447	0.745119	-0.42648	2.815644
TNKS1BP1	0.982447	0.745147	0.136819	8.93266
SND1-IT1	0.982538	0.745399	-0.24131	4.250593
EXOC3	0.982538	0.745487	0.143803	7.149527
KDELC2	0.982538	0.745495	-0.17649	5.906003
HEBP2	0.982538	0.745677	-0.16909	6.405197
MAPKAP1	0.982538	0.745699	-0.6956	1.078879
CYP46A1	0.982538	0.745785	0.474691	1.291912
USP45	0.982538	0.745805	-0.21244	5.703615
TTC14	0.982538	0.745815	-0.20542	5.124911
LYG2	0.982826	0.746178	0.523732	0.923367
AJM1	0.982826	0.746456	-0.428	3.015341
SEC14L2	0.982826	0.746475	-0.5448	1.982983
COX6A1	0.982826	0.746483	-0.19744	5.371913
SNX29P1	0.982826	0.746581	-0.52873	1.581093
LINC02363	0.982826	0.746588	0.374362	2.395026
CDC37	0.982826	0.746626	0.130566	7.959604
LINC00365	0.982826	0.746633	-0.49711	3.310484
ZNRF3-AS1	0.982831	0.7468	0.259834	3.012442
PPP1R3D	0.982831	0.746865	-0.32675	4.012956
ACTG1P17	0.982831	0.746911	0.524769	0.883553
MORC2-AS	0.982831	0.746938	-0.46352	2.712267
LCN12	0.982831	0.747011	0.41323	0.724097
RPS6KL1	0.983014	0.747226	0.205118	4.231278
KLHL7	0.983055	0.747437	0.484591	0.902211
EHD4-AS1	0.983055	0.747444	-0.45086	2.328658
PDGFB	0.983055	0.747481	-0.43067	2.142741
KIAA0825	0.983173	0.747788	-0.28682	3.340218
TMC3	0.983173	0.747789	0.383154	2.167389
IQSEC1	0.983173	0.747796	0.152621	5.575953
LINC02254	0.983494	0.748115	0.4696	1.255749
NDST2	0.983588	0.748317	0.145655	5.591021
FAM45A	0.983588	0.748401	0.418141	0.902475
CCDC137	0.983588	0.748429	-0.17671	5.205939
ATAD1	0.983588	0.748486	-0.16982	6.919527
UNC119	0.983591	0.748703	0.49245	0.746569
HIST1H4A	0.983591	0.748987	0.282963	2.66181
LOC101927	0.983591	0.749049	-0.54386	2.385255
OXR1	0.983591	0.749051	-0.46256	1.971375
RPGRIP1	0.983591	0.749153	0.447288	1.178764

B3GNT10	0.983591	0.749153	0.345977	1.763454
SRGAP2	0.983591	0.749258	0.24115	4.101073
LOC10050ͯ	0.983591	0.749283	-0.59631	1.774869
CTPS2	0.983591	0.749283	-0.42359	2.341785
TLR9	0.983591	0.749285	0.473375	0.853755
CFLAR	0.983591	0.749314	-0.15883	6.871022
DDX51	0.984006	0.749745	-0.23277	5.298107
ZBTB25	0.984006	0.74985	0.298133	2.70282
BHMT2	0.984006	0.749854	0.503776	1.097709
TNFSF8	0.984022	0.749942	-0.38529	3.701645
GLUD1	0.984022	0.75014	-0.15149	7.316928
GPM6B	0.984022	0.750157	-0.3343	3.512432
MLANA	0.984022	0.750188	0.265264	3.14768
CFLAR-AS1	0.984022	0.750259	-0.20443	4.668984
SMC5	0.984022	0.750317	-0.1388	8.249827
PLK1	0.984175	0.750551	-0.17814	5.177305
YJU2	0.984175	0.750584	-0.22029	4.771059
UBE2I	0.984177	0.750757	0.429193	1.159682
ABLIM1	0.984177	0.750789	0.204619	4.040026
COL15A1	0.984177	0.75081	0.144117	7.762502
LUC7L3	0.984305	0.75103	0.142512	6.071226
PCP2	0.984305	0.751108	-0.64846	1.435068
DUSP27	0.984305	0.751133	0.347118	6.863756
LOC28392	0.984568	0.751408	0.158425	6.042303
VPS9D1-AS	0.98487	0.751714	-0.24153	4.457769
TPP1	0.985093	0.752002	-0.15687	7.376639
FAN1	0.985093	0.752034	0.320569	2.535327
WBP2NL	0.985101	0.75213	0.353271	1.65623
SUFU	0.985101	0.752221	-0.22323	4.072878
ZNF628	0.985101	0.752296	0.227424	3.298437
HUS1	0.985101	0.75236	-0.57835	1.309689
SUOX	0.985101	0.752519	-0.83592	0.807449
ARFGAP1	0.985101	0.752581	0.259937	2.69219
B3GLCT	0.985101	0.752634	-0.25639	4.192834
RAB6A	0.985101	0.752751	0.343321	1.596982
MTREX	0.985101	0.752753	-0.14483	7.221488
TEKT4P2	0.985101	0.7529	-0.53048	1.853592
TMEM204	0.985101	0.752907	-0.52453	1.054734
CCT8	0.985101	0.75309	-0.42443	3.192139
ARAP2	0.985101	0.753092	-0.16899	6.701007
LHFPL6	0.985101	0.753159	-0.18994	6.016182
HERC1	0.985101	0.753294	0.123885	8.713367
SLC35F6	0.985101	0.753338	-0.16165	5.936959
GALNTL6	0.985101	0.753445	-1.01024	1.581204
LOC10537	0.985101	0.753522	0.253742	3.69431

TBC1D3P1-	0.985101	0.753538	-0.43361	2.177278
ZNF711	0.985101	0.753542	0.177766	4.751483
RIPK3	0.985113	0.753645	0.158363	4.873651
PMVK	0.985113	0.753718	-0.21055	4.557092
TRAF7	0.985113	0.753777	-0.17068	6.509001
SERPIND1	0.985185	0.753907	0.210613	3.464894
BCAT1	0.985205	0.754006	-0.79273	0.783425
RIF1	0.985205	0.754076	0.302306	1.887933
KCTD11	0.985205	0.754196	-0.22823	4.456374
NR3C1	0.985205	0.754222	0.288072	3.0757
MIR210HG	0.985301	0.754371	0.283636	2.906524
MXD3	0.985493	0.754779	0.307512	1.299892
PITPNM2-A	0.985493	0.754848	0.276812	2.665564
MPC1	0.985493	0.754861	0.250554	3.315542
GSTM5	0.985493	0.755023	0.333816	2.179899
PCOLCE2	0.985493	0.755146	-0.7077	0.86995
F2RL3	0.985493	0.755294	0.209033	4.03696
CCDC97	0.985493	0.755388	0.131885	6.087988
ALG6	0.985493	0.755434	-0.29163	3.977416
PLEKHH3	0.985493	0.755459	0.454511	0.867216
C19orf57	0.985493	0.755496	0.304922	2.33913
CCDC174	0.985493	0.755632	-0.14899	6.121854
NSUN6	0.985493	0.755646	0.163714	4.938143
KBTBD7	0.985493	0.755739	0.214611	3.986242
SH2B2	0.985493	0.755793	0.288093	1.998447
LINC01569	0.985493	0.755924	-0.40702	2.147545
FARSB	0.985493	0.755961	0.316204	2.084818
BRD3	0.985493	0.756025	-0.1526	6.759452
CENPT	0.985493	0.756204	-0.16608	5.757363
TIMM44	0.985493	0.756253	-0.16318	5.777574
TACO1	0.985493	0.756255	0.188418	3.897508
MPHOSPH6	0.985493	0.756261	-0.21211	4.904308
PPM1G	0.985493	0.756287	0.133965	7.464632
ANKRD62	0.985493	0.756298	0.340687	2.719786
LRRC9	0.985493	0.756321	-0.81569	0.7738
ZFAND6	0.985763	0.756603	0.334697	2.062549
CNTNAP3	0.985932	0.756808	-0.43927	3.321708
DNMT3A	0.986303	0.757222	-0.34864	3.122981
3-Mar	0.986303	0.757243	0.222407	3.937542
ZNF638	0.986385	0.757381	-0.53093	1.846914
LCN2	0.986413	0.757478	0.185631	7.606564
TOMM70	0.986469	0.757651	-0.14205	6.686583
DKFZP434A	0.986469	0.757699	-0.6591	1.469604
HGH1	0.986469	0.757746	0.174749	4.181492
KIF3B	0.986796	0.758073	-0.13928	7.430318

WDFY3-AS2	0.987029	0.758347	-0.66069	1.380939
C3orf67	0.987029	0.758402	0.307138	3.318237
KMT2D	0.987336	0.758869	-0.14025	9.313644
CIPC	0.987336	0.758911	-0.18863	5.127539
TMEM266	0.987336	0.758926	-0.41909	1.93576
GPR39	0.987336	0.759035	0.228439	3.243136
LIG3	0.987336	0.759179	-0.26075	3.268312
NDUFB2	0.987336	0.759399	0.20902	4.435459
AGPS	0.987336	0.759408	0.128434	7.274648
DDB2	0.987336	0.75947	-0.21518	4.296712
C19orf48	0.987336	0.759605	0.351697	1.801852
SNORA53	0.987336	0.759607	-0.16875	7.527353
ADGRF1	0.987336	0.759677	0.294877	3.943355
GLIPR2	0.987336	0.759699	0.257928	3.08339
NAXE	0.987336	0.75972	-0.18855	4.833956
ALDH1A1	0.987336	0.759897	0.197149	7.373411
TPH1	0.987336	0.759923	0.393797	1.878965
ST6GALNAC	0.987336	0.759986	0.317609	3.153445
P2RY13	0.987336	0.76002	0.334261	1.855085
MAPK6	0.987336	0.760072	0.143888	7.473429
IRF2	0.987336	0.760196	0.127632	6.124202
DCAF15	0.987336	0.760236	0.143834	4.887944
FIZ1	0.987336	0.760262	-0.31491	3.209593
MYO18A	0.987336	0.760323	0.412737	1.626603
UFL1	0.987336	0.760374	-0.15159	7.093602
SNORA11	0.987336	0.760498	-0.23091	7.975573
LRRC27	0.987336	0.760592	0.477115	1.118566
DDX3Y	0.987336	0.760595	0.277905	4.423286
WDR45B	0.987379	0.760854	0.138867	6.830929
NUFIP1	0.987379	0.760905	-0.18802	4.456169
CENPBD1	0.987379	0.760969	0.266374	2.652194
COL4A2	0.987379	0.76097	-0.16062	9.488317
KCNAB3	0.987379	0.761062	0.320487	2.200372
SIRPB1	0.987379	0.761139	0.457281	0.892847
SLC38A2	0.987379	0.761344	-0.51699	2.348153
VSIR	0.987379	0.761346	0.138923	6.14377
STAU2	0.987379	0.761347	-0.44	2.012515
GAS6-AS1	0.987379	0.761381	-0.31012	4.234433
NXPH4	0.987405	0.761476	-0.75568	1.100621
TPM4	0.987421	0.761811	0.132855	6.577201
CFH	0.987421	0.76184	-0.19765	7.742574
FUT8	0.987421	0.761995	-0.42734	1.217503
MCUR1	0.987421	0.762133	-0.17498	5.763873
ZNF517	0.987421	0.762374	-0.52843	1.586362
STX8	0.987421	0.762402	-0.58507	1.004338

PARM1	0.987421	0.762472	0.163767	6.444875
SPINK5	0.987421	0.762616	0.527374	0.836375
FANK1	0.987421	0.762617	0.2354	3.400275
KDELR3	0.987421	0.762637	0.20847	4.596218
ZNF561	0.987421	0.762674	0.145624	5.84659
RAB11B-AS	0.987421	0.762703	0.257884	2.346454
RWDD2B	0.987421	0.762772	-0.31997	3.82834
CYFIP2	0.987421	0.762801	0.451819	0.755728
METTL2A	0.987421	0.7629	-0.20498	4.542763
FAM72C	0.987421	0.762993	0.432634	1.229137
TMC5	0.987421	0.763089	-0.19655	4.9425
ZNF846	0.987421	0.763275	-0.22552	4.674808
SEMA3D	0.987421	0.763279	-0.49944	2.588761
PMP2	0.987421	0.763288	0.433024	0.94413
NDUFS2	0.987421	0.763426	-0.55285	1.839217
DCTN5	0.987421	0.763481	-0.77975	0.847436
RPS27L	0.987421	0.763535	0.136381	6.240603
EAPP	0.987421	0.763544	0.157129	5.5107
POM121L9	0.987421	0.763569	0.253878	3.643094
ARHGEF10	0.987421	0.763582	0.367041	1.52935
ASTN2	0.987421	0.763695	-0.38938	2.302646
EZR-AS1	0.987421	0.763709	0.257173	2.794389
TCEAL7	0.987421	0.763801	0.352137	1.986806
PROSER2	0.987421	0.763837	-0.42156	3.325912
CALR	0.987421	0.764063	0.12101	10.08386
SLC4A5	0.987421	0.764172	-0.26844	3.425812
CXCL12	0.987421	0.764174	0.284206	3.2459
RBM12B	0.987421	0.76434	-0.1463	6.650743
PTGDS	0.987421	0.764406	0.196971	5.597228
ZNF324	0.987421	0.764454	-0.29072	4.337708
RTKN	0.987421	0.764489	0.196091	3.5231
GK5	0.987421	0.764581	0.212011	3.688908
BMS1P4	0.987421	0.764635	-0.17263	4.747701
GUK1	0.987421	0.764654	-0.39327	2.726476
SRRM2	0.987421	0.764831	-0.14105	11.53968
SNORA48	0.987421	0.764914	-0.18295	7.93046
SH3D19	0.987421	0.764919	-0.53907	1.590475
LINC00639	0.987421	0.76492	0.453029	0.94837
USB1	0.987421	0.765056	-0.31201	2.933965
TMEM63C	0.987421	0.765112	0.330399	2.253638
TOB2P1	0.987421	0.765141	0.387392	1.167317
NREP	0.987421	0.765197	0.310483	2.216489
MFAP1	0.987421	0.765408	0.122828	6.742867
CEP164	0.987421	0.765409	-0.28268	2.91456
NOM1	0.987421	0.765502	-0.17117	6.570748

BEST1	0.987421	0.765545	-0.71095	1.310842
ADRA1D	0.987421	0.765581	-0.64081	0.833499
TACC2	0.987421	0.765583	-0.41449	2.084248
WDR11	0.987421	0.765628	-0.15013	7.127836
VAV3	0.987601	0.765842	0.215811	3.587855
GALNT3	0.987909	0.766157	-0.16708	7.561001
WASF2	0.987981	0.766288	0.158794	4.584636
NUDC	0.988275	0.766591	-0.13498	7.323663
CDKL1	0.988299	0.766685	-0.31722	3.467197
CCDC167	0.988442	0.766877	-0.30238	2.94375
CPT2	0.988442	0.766991	-0.19482	4.880863
SPRED1	0.988442	0.767022	-0.14168	6.537622
PHF7	0.988538	0.767172	0.315873	2.41577
SH3BP5L	0.988569	0.767294	-0.15743	5.555495
INPP5A	0.988569	0.767701	-0.15767	5.433438
NCAPD2	0.988569	0.767715	-0.15032	6.846567
CAMKMT	0.988569	0.767842	0.212063	3.740239
LOC101928	0.988569	0.768131	0.358284	1.393599
PAPOLA	0.988569	0.768136	-0.33779	2.764614
AIRN	0.988569	0.768151	0.257166	3.155217
AGPAT4	0.988569	0.768173	-0.15987	5.471685
TTC39C	0.988569	0.768204	0.223435	3.119967
SCARNA23	0.988569	0.768283	-0.18895	4.90975
PVR	0.988569	0.768508	0.300096	2.232144
CFAP54	0.988569	0.768535	0.235379	3.532607
LOC284581	0.988569	0.768639	0.242999	3.709954
RPL23AP87	0.988569	0.768687	-0.68429	0.911364
WDR45	0.988569	0.768716	0.27734	2.431065
RBP7	0.988569	0.768991	0.384937	1.453572
CYFIP1	0.988569	0.76901	0.294944	2.502603
DGLUCY	0.988569	0.769122	0.233398	2.860207
CDH15	0.988569	0.769132	0.409417	1.070825
SH3BP5-AS	0.988569	0.76915	0.161977	4.95304
CFDP1	0.988569	0.769203	0.120436	6.818303
TIRAP	0.988569	0.769369	-0.31322	3.793499
SCO1	0.988569	0.76947	-0.17691	5.505956
HLA-DRA	0.988569	0.769503	0.13969	6.270656
LOC102723	0.988569	0.769516	0.391664	1.193429
NTRK1	0.988569	0.769557	0.4505	1.371072
TMEM186	0.988569	0.769599	-0.22438	4.05231
PYGO2	0.988569	0.769658	0.147968	5.435249
FBXW7	0.988569	0.769685	-0.26054	3.715527
BMPR2	0.988569	0.769728	-0.14075	8.353433
DIO3OS	0.988569	0.76974	0.362147	2.203688
AP4S1	0.988569	0.769838	-0.31261	3.195546

UFM1	0.988569	0.769878	-0.25009	3.581364
EEF2K	0.988569	0.769887	-0.14875	7.221412
ABCD2	0.988569	0.7699	0.311213	2.4137
EXOSC7	0.988569	0.769981	0.269113	2.582124
TIGAR	0.988569	0.769984	-0.16759	5.752359
SPICE1	0.988656	0.770268	0.121977	6.40527
SNORA104	0.988656	0.770511	-0.39327	2.54005
RNF10	0.988656	0.770527	0.113291	7.471034
LOC100506	0.988656	0.770544	-0.3676	2.70015
RANGAP1	0.988656	0.770648	-0.26897	3.052503
NUP93	0.988656	0.77067	-0.24601	3.229769
CPTP	0.988656	0.770723	0.160182	4.295623
CC2D1B	0.988656	0.770911	-0.15907	6.231279
LINC00294	0.988656	0.770917	0.147374	4.927636
AREL1	0.988656	0.770976	-0.15853	6.552422
ZNF783	0.988656	0.771029	-0.22295	5.413175
P2RY14	0.988656	0.771115	0.480468	1.104651
SLF2	0.988656	0.77136	-0.1852	5.37922
B4GALT2	0.988656	0.771373	0.384912	1.045364
POF1B	0.988656	0.771392	-0.21866	7.421574
MGST3	0.988656	0.771437	0.207458	5.596724
COX7B	0.988656	0.771568	-0.18543	5.359318
HS3ST1	0.988656	0.771627	0.210371	3.622829
GET4	0.988656	0.771705	-0.15261	6.030877
COPS9	0.988656	0.771718	-0.622	1.45022
SNORA70G	0.988656	0.771831	0.280697	3.095003
SMIM19	0.988656	0.77189	-0.42276	1.560531
TSNARE1	0.988656	0.771932	0.174763	4.154277
LYRM9	0.988656	0.772135	0.205125	3.056795
RNF20	0.988656	0.772193	0.120493	7.308354
SKAP2	0.988656	0.772239	0.199695	3.285214
TMED9	0.988656	0.772316	0.130232	7.060696
BMS1P22	0.988656	0.7724	0.313792	2.367645
LOC102031	0.988656	0.772488	-0.3823	2.18083
LINC02516	0.988656	0.772492	0.344826	0.867729
FAM111A-I	0.988656	0.772541	-0.33331	3.13224
ATN1	0.988656	0.772699	-0.51683	1.56014
FUBP1	0.988656	0.772772	0.281203	3.194203
DCUN1D2	0.988656	0.772842	-0.1684	5.029908
LRRC2	0.988656	0.772873	-0.36657	2.219442
NEK9	0.988656	0.772909	-0.14689	7.427301
SLC38A1	0.988656	0.772966	-0.1764	4.529385
STAT5B	0.988656	0.772989	-0.13815	6.836244
MRPL54	0.988656	0.773158	0.231769	3.395395
RILPL1	0.988656	0.773229	0.166443	4.751806

SEPT7P2	0.988656	0.773258	-0.1825	5.050273
COX5A	0.988656	0.773293	0.153201	5.106139
C9orf72	0.988656	0.773297	0.28023	2.979931
PCNX3	0.988656	0.773378	-0.14469	7.056426
CD68	0.988656	0.773442	0.417773	0.727881
NRON	0.988664	0.773524	0.269744	2.653676
ABHD15	0.988665	0.773697	-0.20298	4.416096
ERLIN2	0.988665	0.773877	-0.14528	6.467418
KIF4A	0.988665	0.773961	-0.22587	5.219237
OGFOD1	0.988665	0.773985	-0.15538	5.815427
ROR2	0.988665	0.774008	-0.21388	4.922584
ACTN4	0.988665	0.774018	0.116968	9.758687
ARMH1	0.988665	0.774294	0.313644	1.850992
CCDC91	0.988665	0.774469	-0.13108	6.13512
TFAP4	0.988665	0.774536	-0.21626	4.81999
ZBTB1	0.988665	0.774593	0.145957	4.961681
TBRG1	0.988665	0.774657	0.231883	2.658589
DOCK4-AS1	0.988665	0.774702	-0.32052	3.188978
DDHD2	0.988665	0.774865	0.326624	1.491502
PSMC6	0.988665	0.775023	-0.17861	5.975871
MIR1914	0.988665	0.775238	-0.62031	1.098708
SMIM12	0.988665	0.775484	0.337065	1.687786
PRKRA-AS1	0.988665	0.775488	-0.36762	3.673168
CELF1	0.988665	0.775681	-0.13459	6.007044
PDZD8	0.988665	0.775681	0.133429	8.292263
IKZF2	0.988665	0.775755	0.427663	0.823458
RHOH	0.988665	0.775832	0.285729	2.009381
RPRD2	0.988665	0.775991	-0.7166	1.006413
POLR2E	0.988665	0.776049	-0.54122	1.232689
SCAF1	0.988665	0.776085	-0.1479	6.998709
ERCC6L	0.988665	0.776165	-0.30349	3.731857
ACKR3	0.988665	0.776216	0.225738	3.781067
FBXL8	0.988665	0.776244	-0.35685	2.734148
HES2	0.988665	0.776334	-0.35077	3.609091
P2RX5-TAX	0.988665	0.776367	-0.4977	1.56298
LINC00999	0.988665	0.776375	0.251148	3.093217
FOXF2	0.988665	0.776455	0.234251	4.370763
FAM86B3P	0.988665	0.776461	0.209121	2.799867
FBLN1	0.988665	0.776498	-0.18936	6.623046
MIA3	0.988665	0.776604	-0.1301	7.788319
GRK2	0.988665	0.776611	0.119171	7.380518
AVPR1A	0.988665	0.776792	0.24419	3.693178
NUTM2B	0.988665	0.776827	-0.35757	2.80749
GOLGA6L5	0.988665	0.776957	-0.29576	3.76934
PRH2	0.988665	0.776966	0.319852	1.05551

DCAF13P3	0.988665	0.776975	0.202466	3.305718
HIGD2A	0.988665	0.776989	0.175462	3.574155
AZGP1	0.988665	0.77703	-0.26792	4.452002
KLHL25	0.988665	0.777271	-0.25675	4.542085
RBM15B	0.988665	0.77741	0.119196	6.572337
PCDHB19P	0.988665	0.777489	-0.40459	3.159991
PHTF1	0.988665	0.777494	0.141658	5.39357
TUBG1	0.988665	0.777519	-0.18387	4.829029
RPL30	0.988665	0.777548	-0.15029	8.133362
ZNF880	0.988665	0.777612	0.175045	4.149934
RBP1	0.988665	0.777692	-0.50879	2.385133
GRN	0.988665	0.777703	0.112525	7.568665
MRPL4	0.988665	0.777718	-0.58697	1.067063
PRPF19	0.988665	0.777741	-0.14138	6.303089
SNORA70	0.988665	0.777777	-0.15973	7.869795
ABCA17P	0.988665	0.777779	-0.50033	2.443236
RAC2	0.988665	0.777806	-0.21111	4.519914
QPCT	0.988665	0.77782	-0.48924	2.235537
GPR87	0.988707	0.777928	0.372112	2.210107
HTATSF1P2	0.988852	0.778234	-0.33036	3.927968
CYP2A6	0.988852	0.778253	0.339093	1.253025
SLC3A2	0.988852	0.778269	0.237863	1.852354
MRPL48	0.98891	0.77839	-0.21677	4.852704
NKX2-8	0.988916	0.778531	-0.93602	0.999076
RNF113B	0.988916	0.778585	0.304908	1.923728
ZNF808	0.988916	0.778748	0.129689	5.91662
ZNF35	0.988916	0.778849	-0.24652	4.017084
GAS5	0.988916	0.779028	0.136147	5.736947
ZNF789	0.988916	0.779081	-0.20398	4.222059
FABP4	0.988916	0.779163	0.347487	1.356737
CAPN11	0.988916	0.779173	0.447691	0.987919
LOC65316(	0.988916	0.779199	0.194957	3.628409
TMEM14A	0.988916	0.779212	-0.2318	4.298128
SNHG7	0.988916	0.779298	0.259785	2.209776
NOXRED1	0.988916	0.779299	-0.54832	1.329751
SRP9	0.988918	0.77938	0.395274	1.177278
SAMM50	0.988918	0.779452	0.143552	5.181862
GTF2B	0.989137	0.779699	0.133071	5.042376
ADGRA2	0.989143	0.779805	-0.15177	6.420767
PATJ	0.989143	0.779928	-0.13911	8.484215
MIR661	0.989143	0.779995	0.350378	0.871091
PPP1R35	0.989143	0.780166	0.188973	3.726068
WDR33	0.989143	0.780244	-0.12852	7.096205
CSRP1	0.989143	0.780269	0.382819	1.393739
PPM1F	0.989143	0.780301	0.11605	5.825299

CDC42EP1	0.989143	0.780307	-0.20114	7.276175
LPP	0.989185	0.780416	-0.18921	4.584879
MAP3K14-/	0.989302	0.780584	-0.48609	1.31448
NCAM1	0.989422	0.780851	0.452087	0.907619
LLPH	0.989422	0.780923	0.161596	5.17719
FUT6	0.989422	0.780942	0.40489	0.874945
MIER1	0.989422	0.78098	-0.32325	3.761106
CNP	0.989558	0.781301	0.126198	6.085621
CRADD	0.989558	0.781312	0.251787	3.531714
SNORD44	0.989558	0.781313	0.283854	2.553601
LOC10049	0.989661	0.78147	0.3374	1.888769
MAZ	0.989757	0.781731	-0.63284	0.923218
MORF4L1	0.989757	0.781934	0.310479	2.279419
EMP1	0.989757	0.782013	0.197605	8.820731
TM9SF2	0.989757	0.782039	0.115445	8.317156
GFOD2	0.989757	0.782081	-0.17512	4.791828
ITGB4	0.989757	0.782091	-0.52875	1.830182
HIST1H2BJ	0.989757	0.782133	-0.26056	4.877166
ZNF384	0.989757	0.782249	-0.52531	1.401494
YLPM1	0.989757	0.78228	-0.15162	8.906998
C11orf65	0.989757	0.782313	0.375657	1.195881
UBD	0.989757	0.782375	0.483309	0.586349
SYNE1	0.98981	0.782493	0.310568	2.186552
LINC01137	0.989844	0.782716	-0.3776	2.53011
RRP8	0.989844	0.782846	0.126341	4.877652
SPTBN1	0.989844	0.782934	0.109145	8.576558
TBX6	0.989844	0.783012	-0.48516	1.990753
LMO7	0.989844	0.783022	0.239244	3.311822
TIGD1	0.989844	0.783138	0.154216	4.654602
IER3IP1	0.989844	0.783148	0.147322	6.140595
IRX3	0.989844	0.783154	0.346607	2.863065
PLCE1	0.989844	0.783279	-0.4727	1.463752
RPL23P8	0.989844	0.783355	0.265693	1.637538
BRD9	0.989844	0.783503	-0.32834	2.400378
MCCC1	0.989844	0.783762	-0.60207	1.042244
PLAC9	0.989844	0.783798	0.200208	3.926623
PATL1	0.989844	0.783798	-0.12975	7.056955
MSH5-SAP(	0.989844	0.783826	-0.42299	1.320943
C20orf202	0.989844	0.78396	0.399624	1.773933
CARD8-AS1	0.989844	0.784006	0.284635	2.047218
TPT1-AS1	0.989844	0.784083	0.176815	4.323539
GPATCH3	0.989844	0.784087	0.135183	4.782728
FAM117A	0.989844	0.784098	-0.1997	3.755004
LOC10050€	0.989844	0.784104	0.136886	5.72412
STK11IP	0.990019	0.784327	-0.16722	4.888937

MIR2278	0.990019	0.78442	0.322148	1.331696
PDSS2	0.990019	0.784534	-0.1892	5.048832
C16orf58	0.990019	0.784545	0.120145	6.191215
DYNLRB1	0.990042	0.784638	0.322419	1.035518
PPP1CB	0.990307	0.784995	-0.32879	1.938961
GOLGA2	0.990307	0.78507	-0.13148	8.145844
HACD4	0.990307	0.785241	-0.3157	3.17377
SEPSECS-A!	0.990307	0.785254	-0.42594	1.913448
LRG1	0.990307	0.785255	0.197305	3.916259
CHSY1	0.990307	0.785465	-0.13941	6.36921
BCL7C	0.990307	0.785466	-0.46906	1.732722
LTC4S	0.990307	0.785708	-0.42987	2.607637
KLHDC3	0.990307	0.785744	-0.50537	1.232528
SNX8	0.990307	0.785787	-0.14544	5.703547
CCDC127	0.990307	0.785863	-0.27838	4.126277
FERMT1	0.990307	0.785928	-0.13964	7.265946
SNORD114	0.990307	0.786032	-0.34097	3.05776
LINC00899	0.990307	0.786047	0.331812	1.50199
FEM1C	0.990307	0.786084	0.120547	6.791507
CHCHD1	0.990307	0.786091	0.158251	4.222818
WFIKKN1	0.990307	0.786144	0.317331	1.641636
LOC171391	0.990307	0.786207	0.365329	0.966819
TCAF2	0.990339	0.786363	-0.26978	2.967801
SNORD114	0.990339	0.786463	0.363916	0.987536
ALKBH7	0.990339	0.786483	-0.22332	3.613012
RPL27A	0.990339	0.786535	0.126566	8.937405
RNF148	0.990356	0.78668	-0.35028	2.58387
RCOR2	0.990356	0.786784	-0.50404	1.993215
HS1BP3	0.990356	0.786838	0.11911	5.367901
LOC10192!	0.990356	0.78685	-0.58137	1.044459
PHETA2	0.990429	0.787067	0.1883	3.286482
SIGLEC1	0.990429	0.787122	0.17709	5.202465
RPP30	0.990429	0.787177	-0.28384	3.499431
FAAH2	0.990429	0.787246	-0.18343	3.957457
GPX2	0.990429	0.787421	0.200833	2.959982
NAALADL2-	0.990429	0.787434	-0.70579	0.801286
CLSTN2	0.990429	0.787477	0.244847	2.610391
HIST1H2AL	0.990429	0.787534	-0.25301	4.096138
KATNB1	0.990429	0.787587	0.146164	4.583729
DAB2IP	0.990465	0.787692	0.127499	5.611538
MANSC1	0.990556	0.788	0.12446	5.736389
GTF2E1	0.990556	0.788023	-0.15668	4.896955
OLFML1	0.990556	0.788199	0.156352	4.282286
GTSE1	0.990556	0.788237	-0.243	3.67503
KYNU	0.990556	0.788384	-0.45938	1.219114

CRISPLD2	0.990556	0.78839	-0.14422	7.809637
TMEM132I	0.990556	0.788498	0.369993	0.806362
FAM228A	0.990556	0.78851	0.357793	1.189297
KIAA1328	0.990556	0.788599	-0.28088	4.885821
ZBTB20-AS	0.990556	0.788614	-0.57621	0.767189
ATF7	0.990556	0.788667	0.266596	1.979086
LOC100507	0.990556	0.788678	0.338771	1.88274
ANKRD30B	0.990556	0.788922	0.452771	1.059248
ADAM1A	0.990556	0.789007	0.144549	4.340682
RNF146	0.990556	0.789034	-0.3675	2.088947
POLR2G	0.990556	0.789051	-0.21819	4.140382
PRSS30P	0.990556	0.789143	-0.61793	1.744546
OCLNP1	0.990556	0.789227	-0.15752	5.804208
PLCL1	0.990556	0.789259	-0.32591	4.413006
SMARCC1	0.990556	0.789321	0.115961	8.788334
SUMO1P3	0.990556	0.789403	-0.17489	4.271729
CUTALP	0.990556	0.789425	0.147947	4.989582
ZFP1	0.990698	0.789614	-0.19427	4.002034
LOC100507	0.990881	0.789835	-0.74274	1.406231
SPRY2	0.991062	0.790205	0.129485	5.004545
PBOV1	0.991062	0.790344	0.2178	3.084977
DOP1A	0.991062	0.79038	0.323393	1.232567
RIMS2	0.991062	0.790498	-0.6318	1.033908
ZCRB1	0.991062	0.790511	-0.14259	5.85457
DACT3	0.991062	0.790711	0.31616	1.837002
HMGB1	0.991062	0.790807	0.134745	5.145419
CSTF3	0.991062	0.790928	0.132608	5.254733
LINC01082	0.991062	0.790967	-0.61233	1.520959
PPP2R2A	0.991062	0.790995	0.266896	1.834122
NYAP1	0.991062	0.791142	0.379898	1.009668
TREM1	0.991062	0.791157	-0.48388	1.322272
LOC101929	0.991062	0.791215	0.301125	2.222627
DPH3P1	0.991062	0.791267	-0.48289	1.83482
RPL35	0.991062	0.791304	-0.13318	8.225038
RNF13	0.991062	0.791315	0.265447	1.480812
MGAM	0.991062	0.791339	0.312014	1.609685
LRRC4B	0.991062	0.791442	-0.37967	2.500748
EIF3D	0.991062	0.791476	0.113137	7.859661
NCL	0.991062	0.791514	0.113988	10.61205
BLCAP	0.991062	0.791674	0.139248	4.565444
SLC6A11	0.991062	0.791698	0.293849	2.187289
SNORA16B	0.991062	0.791771	-0.73007	1.051634
EID2B	0.991062	0.791991	0.340547	1.866294
EPHB3	0.991062	0.792034	0.154304	5.916769
CNOT6	0.991062	0.792048	0.104396	6.601328

NKX6-1	0.991062	0.792117	-0.42781	3.3762
TRMT6	0.991062	0.792256	-0.48594	1.467301
ZNF132	0.991062	0.792292	0.246853	2.760353
ARID1A	0.991062	0.792426	-0.17695	4.246585
TMCO1-AS	0.991062	0.792487	0.307166	2.06044
FKBP15	0.991062	0.792593	-0.12698	6.930314
C5orf51	0.991062	0.792647	-0.17103	5.887933
CATSPERB	0.991062	0.792688	-0.52822	3.208732
NUPL2	0.991062	0.79285	0.139125	5.162365
SNHG18	0.991062	0.792983	0.205894	3.513234
WDR53	0.991062	0.793017	-0.37438	3.056081
ACKR4	0.991062	0.793153	-0.45222	1.752018
STK24	0.991062	0.79323	0.242488	2.777743
PLXDC1	0.991062	0.793264	-0.17624	5.512525
HIST1H3J	0.991062	0.793297	-0.39355	2.538452
GOLGA8S	0.991062	0.793478	-0.56467	1.298231
NPLOC4	0.991062	0.793485	-0.1268	6.311574
TEKT3	0.991062	0.793515	0.41401	1.284303
GOLGA8R	0.991062	0.793518	0.285751	2.1421
PDE10A	0.991062	0.793536	-0.57507	0.9932
LINC00997	0.991062	0.793555	0.148857	4.448009
SLC35F1	0.991062	0.793605	0.369309	1.605657
TMSB10	0.991118	0.793739	0.108645	9.349421
TYRO3P	0.991118	0.793801	-0.37819	2.723432
VAMP2	0.99116	0.79391	-0.12772	6.079628
TIPRL	0.991303	0.794269	-0.16599	4.595623
TMEM8B	0.991303	0.794288	0.234344	2.882772
LOC10012[illegible]	0.991303	0.794378	0.350956	0.838643
LINC01725	0.991303	0.794424	-0.56468	1.025831
LOC44030[illegible]	0.991303	0.794458	-0.13644	6.104459
SH3RF3-AS	0.991303	0.794478	-0.32969	2.89651
TMEM220	0.991347	0.794624	0.145501	4.839396
IMPG2	0.991347	0.794841	-0.34129	1.793682
EP300	0.991347	0.794851	-0.11931	8.927986
SULT2B1	0.991347	0.794851	0.44182	0.589982
RSPH6A	0.991347	0.794891	0.34452	1.255854
LOC44166[illegible]	0.991499	0.795246	0.238732	2.743648
PTGER1	0.991499	0.79529	0.401822	0.659047
MSANTD2	0.991499	0.795398	-0.17331	4.761461
TMBIM6	0.991499	0.79542	0.219013	2.287916
SPRR2G	0.991499	0.795477	-0.9486	1.010011
RENBP	0.991499	0.795788	0.20534	2.947951
NFIA-AS1	0.991499	0.795906	-0.30835	3.483999
LMF2	0.991499	0.795943	0.119047	5.292271
TRAK2	0.991499	0.795949	-0.13774	7.039005

SDCBP2-AS	0.991499	0.796152	-0.40066	2.265968
TMEM167E	0.991499	0.796158	-0.14636	5.372716
RPL21P28	0.991499	0.796163	0.115626	5.888226
TTTY10	0.991499	0.796258	0.344394	1.300989
FRMD4A	0.991499	0.79627	0.110186	6.371544
ZMYM3	0.991499	0.796402	-0.4281	1.360519
ZNF7	0.991499	0.796403	-0.32261	2.472192
STRN	0.991499	0.796412	-0.12843	7.700764
LRRK2	0.991499	0.796423	-0.25453	5.878383
LINC00511	0.991499	0.796672	0.277951	1.472596
MBD5	0.991499	0.796718	-0.14476	5.808267
CENPQ	0.991499	0.796772	-0.40635	2.785449
CMC1	0.991499	0.796827	-0.23321	3.464124
SNX19	0.991499	0.796841	-0.15382	5.332342
IQGAP1	0.991499	0.796862	0.10222	9.840022
TSSC4	0.991499	0.796961	-0.35929	2.207255
GFER	0.991499	0.797124	0.136241	4.424141
SNORA78	0.991499	0.79718	0.184833	4.176788
FAM133DP	0.991499	0.797282	0.200094	3.443331
FIG4	0.991499	0.797303	0.138813	5.365017
RPL24	0.991499	0.797309	0.112311	7.776577
PLEKHA8	0.991499	0.797375	-0.12351	6.203791
ADH1A	0.991499	0.797431	0.296729	2.23055
NIF3L1	0.99152	0.797732	0.317694	1.477508
ZC3H8	0.99152	0.797733	-0.15542	5.356326
CYHR1	0.99152	0.797758	0.148441	4.338707
COA4	0.99152	0.79802	0.158915	4.517563
KLHL23	0.99152	0.79831	0.40599	1.023127
ITIH4	0.99152	0.798319	-0.55724	0.838233
GGA1	0.99152	0.79847	-0.45267	1.702369
ACTA2	0.99152	0.798563	-0.38833	2.343775
CCR4	0.99152	0.798616	-0.37958	1.961458
GBP2	0.99152	0.798641	-0.14324	7.537076
HTATIP2	0.99152	0.798715	-0.567	1.37345
HYAL1	0.99152	0.798777	0.237222	1.786172
LOC101927	0.99152	0.798836	0.240957	2.755057
PLOD3	0.99152	0.798963	0.121226	5.851499
IAH1	0.99152	0.799089	-0.19689	4.101951
IL10RB-DT	0.99152	0.799232	-0.47722	1.704921
LINC00513	0.99152	0.799294	-0.17498	4.687558
EMG1	0.99152	0.799296	-0.17563	4.942168
DCN	0.99152	0.799329	0.1424	5.031315
PLEKHA2	0.99152	0.799329	-0.12155	6.798075
STAG3L1	0.99152	0.799568	0.157484	3.74054
BNIP3L	0.99152	0.799607	0.114525	7.023992

HEBP1	0.99152	0.799755	0.155466	4.612644
ROS1	0.99152	0.799849	0.329307	1.519529
NATD1	0.99152	0.799981	-0.1854	4.605203
PTPRS	0.99152	0.800011	-0.72758	0.759336
RGMA	0.99152	0.800038	0.423112	0.643093
KCTD3	0.99152	0.800206	0.098272	7.503681
REPIN1	0.99152	0.800233	-0.33307	2.417288
TLK1	0.99152	0.800236	-0.21314	3.803584
PPARG	0.99152	0.80063	-0.42491	2.66401
C9orf84	0.99152	0.80069	0.348311	1.744005
ARHGEF33	0.99152	0.800711	0.304059	1.800771
C10orf126	0.99152	0.800713	-0.93189	0.800377
RNASEH2B	0.99152	0.800735	0.267407	1.616266
RPL23AP7	0.99152	0.800775	-0.52203	0.787564
AKT1	0.99152	0.800865	0.247222	1.839006
QRICH2	0.99152	0.800888	0.222227	2.733558
BIRC2	0.99152	0.800902	0.223348	2.586619
TBPL1	0.99152	0.800912	-0.47875	1.217825
1-Mar	0.99152	0.800997	0.149413	4.477787
ORMDL1	0.99152	0.801028	0.309249	1.325791
PPFIBP1	0.99152	0.80105	0.399208	0.797794
VPS33A	0.99152	0.801257	-0.16048	4.903553
TANC2	0.99152	0.801304	0.097447	7.033468
ARF4	0.99152	0.801473	-0.12811	7.643819
LOC101928	0.99152	0.801554	0.313391	1.402762
RALGAPA1	0.99152	0.801566	-0.41189	2.197618
ZNF620	0.99152	0.801571	-0.857	2.382376
LOC728989	0.99152	0.801584	0.319979	1.062645
PRR34-AS1	0.99152	0.801614	-0.18835	4.566705
PPHLN1	0.99152	0.801631	0.29173	1.985539
SPATS2	0.99152	0.801669	-0.2949	2.832332
NCBP3	0.99152	0.801746	-0.11814	7.319374
SCAF11	0.99152	0.801775	-0.11153	8.745355
SAA2	0.99152	0.801836	0.34471	1.723011
NIT2	0.99152	0.801914	-0.18472	4.372899
ZNF782	0.99152	0.801979	0.125005	5.344503
XXYLT1-AS1	0.99152	0.80205	0.283767	1.445754
LOC101928	0.99152	0.802398	0.270481	2.638074
TRIM2	0.99152	0.802401	-0.36182	2.723635
LPCAT2	0.99152	0.802488	0.134646	5.313187
IDE	0.99152	0.802537	0.127773	5.581774
CACTIN	0.99152	0.802626	-0.42529	1.51224
F8A1	0.99152	0.802687	-0.36015	2.580113
LEF1	0.99152	0.802722	0.349813	1.005846
MUC6	0.99152	0.802726	0.216967	10.70077

SLC22A4	0.99152	0.802732	0.244944	2.505198
UNK	0.99152	0.802795	0.111744	6.0114
USP4	0.99152	0.803018	0.297784	1.562683
EEF1G	0.99152	0.803104	0.125571	9.251139
TF	0.99152	0.803151	0.264843	2.152362
LINC01012	0.99152	0.803308	-0.59304	0.971136
MLLT10P1	0.99152	0.803344	0.258142	1.993503
SCAMP1-A:	0.99152	0.803352	-0.23992	3.357451
CAD	0.99152	0.803424	0.277084	1.373666
CTSW	0.99152	0.803488	-0.34386	2.692909
OSBPL11	0.99152	0.803525	0.122471	5.587495
VPS4A	0.99152	0.803545	-0.11937	6.451391
RSPRY1	0.99152	0.803688	0.196421	2.925476
PCDHGB8P	0.99152	0.803704	0.299104	2.19163
PLAA	0.99152	0.803756	-0.16575	5.838256
TYW1B	0.99152	0.803784	-0.1566	4.574891
CNTNAP5	0.99152	0.803797	0.302147	1.385489
TCEA2	0.991566	0.803993	-0.34938	3.284841
CDC42EP2	0.991566	0.803998	-0.18517	4.112709
METTL24	0.991566	0.804185	0.329674	1.684203
MRPL35	0.991566	0.804244	0.168102	4.143007
UBE2Q1-A:	0.991566	0.804302	-0.49121	1.677513
RBM44	0.991566	0.804459	0.199504	3.574887
TMEM19	0.991566	0.804571	0.117636	5.65064
IFI16	0.991566	0.804622	0.233117	2.172906
SAP25	0.991566	0.804743	-0.28504	2.737034
SHLD2P1	0.991566	0.804778	-0.28708	3.006338
PARS2	0.991566	0.804832	0.248957	2.477574
SIRT2	0.991566	0.804851	-0.52313	1.61677
TRPV1	0.991566	0.804997	0.243873	2.292585
LINC00299	0.991566	0.805091	0.318419	1.397887
ZNF486	0.991566	0.805269	-0.29681	3.67269
SAMD4A	0.991566	0.805298	-0.49517	1.296239
ZNF235	0.991566	0.805312	-0.15435	4.965654
GTF2IP20	0.991566	0.805324	-0.19909	3.699911
NUP54	0.991566	0.805356	0.252029	1.518403
FHOD1	0.991566	0.8054	-0.14715	5.386297
TNFRSF10D	0.991566	0.805665	-0.139	5.392371
DNAJC14	0.991566	0.805684	-0.12193	5.915907
XRRA1	0.991566	0.805727	0.312126	1.365605
MIR3975	0.991566	0.805847	0.294155	1.925617
PSD4	0.991566	0.805873	0.111427	7.116849
MIR4426	0.991566	0.805917	-0.12277	7.01148
LOC644936	0.991566	0.805972	-0.56357	1.188471
C19orf44	0.991566	0.806063	0.311598	1.581861

CALCOCO2	0.991566	0.80617	0.260472	2.493172
PUS1	0.991566	0.806193	0.200437	2.462061
CD52	0.991566	0.806206	0.244688	2.260167
SWAP70	0.991566	0.806252	-0.51313	1.332567
LARP1	0.991576	0.806336	0.093001	8.882373
FARSA	0.991723	0.806545	-0.14909	5.542125
NCR3LG1	0.991723	0.806607	-0.13344	5.661155
SNCA-AS1	0.991905	0.80683	-0.9013	1.114568
SNHG12	0.991967	0.80729	0.131503	4.582651
CNOT3	0.991967	0.8074	-0.21504	4.088679
ZNF252P	0.991967	0.807423	-0.13701	5.852474
PXDC1	0.991967	0.807464	0.114371	5.515161
KIAA2012	0.991967	0.807574	0.314698	1.649237
NADK	0.991967	0.80767	-0.32841	2.810749
GULP1	0.991967	0.807761	0.296164	0.792584
ROCK2	0.991967	0.807845	-0.11717	8.734062
SNORA52	0.991967	0.808143	0.181011	4.630674
RPE	0.991967	0.808152	-0.63663	1.205983
TMED3	0.991967	0.808283	-0.23384	3.099225
ANKAR	0.991967	0.808283	-0.17156	4.71818
MIR142	0.991967	0.808418	0.306827	2.104636
GPX7	0.991967	0.808425	-0.35667	2.617463
NOVA2	0.991967	0.808514	0.352675	0.870694
NDRG2	0.991967	0.808571	-0.45046	1.301913
HIRA	0.991967	0.808622	0.101147	6.49455
EIF3E	0.991967	0.808648	-0.13748	7.898164
ARHGAP18	0.991967	0.808686	-0.12175	6.820539
BIRC6	0.991967	0.808689	-0.10698	9.478208
ST7-OT4	0.991967	0.808903	0.186439	3.171139
NPR1	0.991967	0.808943	0.155019	3.750244
B3GNT3	0.991967	0.808993	0.103462	7.028071
USP3-AS1	0.991967	0.809135	0.243697	2.769983
KATNBL1	0.991967	0.80922	-0.14787	4.96067
DGAT1	0.991967	0.809262	0.100371	6.133614
COPG2	0.991967	0.809368	-0.21017	3.86689
LINC01465	0.991967	0.809369	0.183161	2.877265
ARRDC3	0.991967	0.809393	-0.12531	7.532266
TMEM243	0.991967	0.809431	-0.17394	3.995504
TRMT61B	0.991967	0.809433	0.120093	4.524045
SIRT4	0.991967	0.809453	-0.62	1.075797
GOLT1A	0.991967	0.809476	-0.56092	2.203607
ABCD1	0.991967	0.809511	-0.19259	4.322655
SNX33	0.991967	0.809527	-0.12073	6.375052
HLA-DPB1	0.992099	0.809773	0.193942	4.730679
LOC72802[illegible]	0.992099	0.809786	-0.29263	2.770794

GLUD1P3	0.992166	0.809916	-0.24728	2.458052
TSPAN13	0.992216	0.810037	-0.16628	6.734403
SDHAF2	0.992216	0.810161	0.121254	4.65722
GDNF-AS1	0.992216	0.810184	0.263595	1.810825
CHADL	0.992256	0.810356	0.228987	2.401754
VPS39	0.992256	0.810368	0.364814	0.852142
TMEM8A	0.992276	0.81046	-0.13357	6.30563
MAML1	0.992332	0.810607	-0.11251	6.635052
DLC1	0.992332	0.810669	0.259788	2.028583
PHACTR4	0.992332	0.810732	-0.20323	3.478341
HOTAIRM1	0.99235	0.810883	0.251042	2.588543
PLCD4	0.99235	0.810898	0.14628	4.81916
ARHGEF28	0.992445	0.811192	-0.62349	1.054497
MIR26B	0.992445	0.811318	0.210351	2.301167
SNRNP40	0.992445	0.811427	0.118223	5.246757
VEGFC	0.992445	0.811545	0.189626	2.745402
DMTN	0.992445	0.811634	-0.68109	0.636682
LOC730668	0.992445	0.811706	0.288637	1.201868
LAMTOR1	0.992445	0.811744	0.106847	5.820614
ZNF317	0.992445	0.811841	-0.87824	0.83278
SRM	0.992445	0.811904	0.131946	4.979388
FLG2	0.992445	0.811943	0.271498	1.885946
PXN-AS1	0.992445	0.811959	-0.47787	1.245056
IBSP	0.992445	0.811961	0.340396	1.655782
TRIM47	0.992445	0.812112	-0.13227	4.975515
B3GNT5	0.992445	0.812343	-0.15963	6.464123
INTS4	0.992445	0.812403	0.10949	5.529432
HIST1H2AE	0.992445	0.812415	-0.154	5.836818
RARRES2	0.992445	0.812486	-0.143	5.410807
DIAPH2-AS	0.992445	0.812569	0.188317	3.376283
MIR181A1I	0.992445	0.812592	0.26029	2.064237
CEBPG	0.992445	0.812593	-0.30584	2.507281
KLHDC8B	0.992445	0.812616	0.225229	3.043367
ITGB3	0.992445	0.812643	-0.15632	5.590172
TEDDM1	0.992445	0.812746	-0.40706	2.019209
RIPK4	0.992445	0.812804	0.169204	5.374096
TIMM13	0.992445	0.81288	0.111841	5.916676
SLC25A51	0.992445	0.813053	-0.25822	3.700053
MLXIP	0.992445	0.813186	-0.1082	8.426332
C1orf226	0.992445	0.813214	0.134067	4.759951
RAB3IL1	0.992445	0.813222	-0.44219	0.769836
ACSL4	0.992445	0.813245	-0.69323	0.850772
LOC10013(	0.992446	0.813341	-0.56422	1.39382
RAET1L	0.992446	0.813513	-0.61524	1.606237
TOP1	0.992446	0.813642	-0.11249	8.682545

STARD7-AS	0.992446	0.813706	0.157599	3.290031
POLE	0.992446	0.813836	-0.11562	7.069483
BORCS7	0.992446	0.813838	-0.27383	2.39805
C1RL-AS1	0.992446	0.813845	-0.12222	6.488077
MSMO1	0.992446	0.81389	0.310346	0.697568
MIR4482	0.992446	0.813996	-0.55283	1.210085
TPTE2P5	0.992446	0.814153	0.314182	1.067145
ALDOC	0.992446	0.8142	-0.30256	3.138337
DKFZP434K	0.992446	0.814274	-0.45467	2.306151
SGK1	0.992446	0.8143	-0.52739	0.979446
SEC16A	0.992446	0.814305	0.192539	3.047983
CROCCP3	0.992455	0.814494	-0.2009	4.999097
KIF1B	0.992455	0.814622	-0.11095	7.795648
CYP2U1	0.992455	0.814708	0.140057	4.65084
RPF1	0.992455	0.814779	0.125638	4.956801
CASKIN1	0.992455	0.814811	0.250676	1.141968
ZNF681	0.992455	0.814835	-0.32683	2.967831
BOC	0.992455	0.814859	-0.37855	3.342847
PSCA	0.992455	0.814971	-0.53896	2.293604
DBR1	0.992455	0.815056	-0.14596	4.568245
ZNF784	0.992455	0.815069	-0.24762	2.766935
LGALSL	0.992457	0.815189	-0.13719	5.093286
REC114	0.992457	0.815458	-0.49999	0.732896
GPSM1	0.992457	0.815576	-0.30402	2.260656
PNKD	0.992457	0.815754	0.16614	3.023489
PAN3-AS1	0.992457	0.815846	0.147428	3.773969
PPIP5K2	0.992457	0.815917	0.275906	1.594974
ADAM19	0.992457	0.815922	0.110294	6.51654
DHX15	0.992457	0.815995	-0.10864	8.053744
SETD3	0.992457	0.816201	-0.11316	5.808027
FKBP2	0.992457	0.816232	-0.42782	1.067291
CDC42	0.992457	0.816264	0.16105	3.980962
EIF3M	0.992457	0.816302	0.135461	4.088341
ATP10D	0.992457	0.816314	-0.14143	6.389415
AMPD3	0.992457	0.816657	-0.35183	2.093383
CSMD1	0.992457	0.816909	0.249544	2.602301
OGDH	0.992457	0.81698	0.293312	1.390069
INTS5	0.992457	0.816986	0.170576	4.22601
TMEM42	0.992457	0.81708	-0.37014	2.381887
HCAR3	0.992457	0.817218	0.308753	1.364484
TMEM184C	0.992457	0.81724	-0.12254	5.669211
ARHGAP15	0.992457	0.817271	0.159077	3.909954
RASSF9	0.992457	0.817339	-0.23158	4.012627
SPRR2E	0.992457	0.817339	0.302643	4.826953
IKZF1	0.992457	0.817362	0.242866	1.68419

FBLIM1	0.992457	0.817475	0.218542	1.932972
CCDC168	0.992457	0.817495	0.270259	2.151901
CASC15	0.992457	0.817596	0.199607	4.141189
RCOR1	0.992457	0.817597	-0.13358	6.523363
KDM3B	0.992457	0.81779	0.094166	8.148231
RCAN2	0.992457	0.817818	0.253332	1.412124
TMEM236	0.992457	0.817929	0.318156	1.420971
GPR183	0.992457	0.818031	-0.15066	5.198286
RGS2	0.992457	0.818052	-0.14311	6.996437
CYREN	0.992457	0.818086	0.299792	1.134061
MIR181A2	0.992457	0.818153	0.274376	1.873807
DHODH	0.992457	0.818165	0.137312	4.045675
MAP2K1	0.992457	0.818209	0.098288	6.419828
PTBP3	0.992457	0.818231	0.295657	1.237216
BARHL1	0.992457	0.818427	0.258658	0.942319
ADAMTS6	0.992457	0.818427	-0.17653	4.841411
ARHGEF39	0.992457	0.818486	-0.18558	4.076066
ZNF865	0.992457	0.818488	-0.1516	5.060606
MEX3C	0.992457	0.818524	-0.12062	6.006773
LIPM	0.992457	0.81853	0.277568	0.7712
ESRG	0.992457	0.818591	0.258762	1.543335
ELN	0.992457	0.818713	-0.61406	0.903143
TARDBP	0.992457	0.818737	-0.10192	8.143572
ACBD6	0.992457	0.818786	0.114386	5.706104
THAP11	0.992457	0.818896	0.119862	4.443339
NEAT1	0.992457	0.818897	-0.12167	13.57367
CST3	0.992457	0.818928	0.097598	6.469095
COL4A1	0.992523	0.819198	-0.12255	9.214685
FAU	0.992523	0.819219	0.095376	7.60626
KIFC1	0.992523	0.819324	0.176236	3.285895
MAST4	0.992523	0.819331	0.258311	1.773509
SERP1	0.992523	0.819453	-0.11479	7.141241
ADD1	0.992523	0.819547	-0.27744	2.682498
CCDC7	0.992523	0.819651	-0.18712	4.554323
PARD6G	0.992523	0.819654	0.172471	3.952091
GLIS3-AS1	0.992523	0.819818	-0.66084	1.256506
ANKS6	0.992523	0.819942	-0.1326	6.193695
GPR137C	0.992523	0.819997	-0.455	1.956065
FAAH	0.992523	0.82004	-0.16466	4.96625
LRRC3	0.992523	0.820101	0.176475	2.78365
PIGO	0.992523	0.820163	-0.25815	2.688623
CASP8	0.992523	0.820177	0.214459	2.55119
CREB3L1	0.992523	0.820283	0.1387	9.137578
SHISA9	0.992523	0.820308	0.302648	1.092158
POLR1C	0.992523	0.820344	-0.17966	4.619309

TEC	0.992533	0.820428	0.112612	4.873523
KCNH7	0.99261	0.820622	-0.58823	1.115578
7-Mar	0.99261	0.820643	-0.39928	1.783888
LPP-AS2	0.993095	0.821366	0.146537	3.793855
TTC6	0.993095	0.821612	0.146758	4.78363
COMMD1	0.993095	0.82162	-0.16871	3.897663
PDZD11	0.993095	0.821638	0.140744	3.775084
HCG25	0.993095	0.821717	0.230267	2.510056
MIR924HG	0.993095	0.821817	-0.71419	1.006367
KIF19	0.993095	0.821906	0.258809	1.798463
OSGEPL1	0.993095	0.822007	-0.20254	3.940035
MCOLN2	0.993095	0.822031	0.169927	3.325442
PDXP	0.993095	0.822059	-0.20119	3.123307
ANAPC2	0.993095	0.822081	-0.14183	5.190401
TMEM18	0.993095	0.822251	0.123664	4.637857
LYPLAL1	0.993095	0.822286	0.200186	1.90104
GIMAP7	0.993095	0.822345	0.127861	5.127985
CTGF	0.993095	0.822399	0.11824	8.16648
MZT2B	0.993095	0.822529	-0.22045	3.149022
CACNA2D3	0.993095	0.822561	-0.42309	1.818791
GIHCG	0.993095	0.822585	0.262046	1.962773
RNF219	0.993095	0.822678	-0.1853	4.890474
LINC00963	0.993095	0.822851	-0.14909	4.469362
LINC01364	0.993095	0.822883	-0.42341	1.8463
LFNG	0.993095	0.822928	0.193768	2.725695
MFSD6L	0.993095	0.822969	0.212172	2.649491
MANBA	0.993095	0.823032	0.101363	6.23677
LMNB2	0.993095	0.823124	-0.12132	7.525463
HPR	0.993095	0.823137	-0.60828	0.934707
FAM66C	0.993095	0.82318	-0.39426	2.088276
SRFBP1	0.993095	0.823217	-0.1362	5.042909
CHKB-DT	0.993095	0.823312	0.260331	1.558955
ZNF283	0.993095	0.823384	0.252659	1.454693
MNT	0.993095	0.823639	0.09546	6.610271
PIP5K1A	0.993095	0.823655	-0.54257	0.72902
ING3	0.993095	0.823862	0.150383	3.508312
ZNF184	0.993095	0.823935	0.122876	4.923814
CRSP8P	0.993095	0.823947	0.201954	2.163655
CAPN12	0.993095	0.823971	-0.19147	4.353248
PIN4P1	0.993095	0.82435	-0.31601	2.291278
SDHAF1	0.993095	0.824466	0.14114	3.315126
TARS	0.993095	0.824474	-0.54276	0.672233
EI24	0.993095	0.824536	0.193746	1.866767
MIR3619	0.993095	0.824627	0.224284	2.053614
FBRS	0.993095	0.824633	-0.10364	6.95859

LDLRAD4	0.993095	0.824649	0.195225	2.118584
BCAR1	0.993095	0.824667	0.144304	3.104496
ORC4	0.993095	0.824775	-0.39207	1.958095
MCC	0.993095	0.824926	0.183105	2.504576
EIF4EBP2	0.993095	0.824972	0.086951	8.40622
RPS15AP1(	0.993095	0.82499	-0.22993	3.904393
EXO5	0.993095	0.82512	-0.27454	3.153927
KBTBD12	0.993095	0.825131	-0.31176	3.369813
KMT2B	0.993095	0.825136	-0.10943	7.974974
FOXN1	0.993095	0.825151	0.242171	2.576787
PRKX	0.993095	0.825205	-0.14718	5.781137
SMC2	0.993095	0.825221	-0.39466	1.973926
DPRXP4	0.993095	0.825273	0.254496	1.68753
HEPH	0.993095	0.825285	0.23	2.747971
MIRLET7F1	0.993095	0.825359	0.192039	3.024084
DND1	0.993193	0.825636	0.160683	3.529175
NOCT	0.993193	0.825682	-0.16374	3.946299
KCNIP4-IT1	0.993193	0.825729	0.331136	1.15711
PRMT9	0.993193	0.825743	-0.4867	0.822777
TMEM86A	0.993264	0.825931	0.190237	3.1799
UBE2C	0.993264	0.825991	-0.34456	2.470908
ZNF24	0.993264	0.82603	0.10434	6.968752
ISOC1	0.993311	0.826144	0.160148	4.750869
WASH7P	0.99334	0.826243	0.112083	4.750388
KCMF1	0.993368	0.826343	-0.10756	6.597726
SEC24A	0.99342	0.826603	0.097758	6.008905
WDR70	0.99342	0.826632	0.089856	5.874419
IFRD1	0.99342	0.826651	-0.32316	2.266616
LOC10012¢	0.99342	0.826732	0.244822	2.345538
CPNE2	0.99342	0.826765	0.112854	4.414965
TBX21	0.993479	0.82689	0.281933	1.300973
DEF6	0.993873	0.827344	0.095379	6.18182
SNORD45A	0.993873	0.827369	-0.14919	9.394839
ASXL2	0.993915	0.827479	-0.0999	7.7469
ZSCAN12	0.993933	0.827601	-0.34111	2.68431
SS18L2	0.993933	0.827731	0.18082	3.24509
LINC01989	0.993933	0.827759	-0.69794	0.98492
NAA16	0.993933	0.827798	0.099673	5.512351
SNORA81	0.993933	0.828064	-0.25374	3.390836
UPF3A	0.993933	0.828072	0.306421	1.54869
EXOSC3	0.993933	0.828075	-0.45678	0.997341
MSI2	0.993933	0.82812	0.117817	4.839692
LOC28333!	0.993933	0.828213	-0.16923	4.367374
PA2G4	0.993933	0.828252	-0.10683	7.249101
PRXL2A	0.994027	0.828448	0.23929	1.944345

PCAT1	0.994027	0.828608	0.254707	1.785734
SPTY2D1O(	0.994027	0.82862	-0.40136	1.63692
DCDC2B	0.994027	0.828641	-0.20709	3.360591
FOXD2	0.994027	0.828709	0.176111	3.227338
TOR4A	0.994031	0.828881	0.109941	6.118985
ENKD1	0.994031	0.829159	-0.19321	3.854125
ANKRD39	0.994031	0.829326	-0.19538	3.122673
ERFE	0.994031	0.829375	-0.38328	2.343336
RHBDD3	0.994031	0.82949	-0.18145	3.640826
AGR2	0.994031	0.829763	-0.22448	10.45459
FOXE3	0.994031	0.829773	0.288662	0.592262
DMPK	0.994031	0.829817	0.213678	2.74787
OTUD5	0.994031	0.829942	-0.22363	2.727432
RP9	0.994031	0.829967	-0.28663	3.771343
PNPLA8	0.994031	0.829975	-0.41591	1.660465
SUGT1P4-S	0.994031	0.830011	-0.41572	2.101986
ZC2HC1A	0.994031	0.830033	-0.14654	5.172043
ABCC10	0.994031	0.830167	-0.28614	2.665281
CDC123	0.994031	0.830185	-0.12265	5.838735
FA2H	0.994031	0.830229	-0.16563	5.044304
TBC1D32	0.994031	0.830354	-0.46824	1.308885
PPAT	0.994031	0.830533	-0.17152	4.940131
PFAS	0.994031	0.830662	-0.12193	5.322226
TOP2B	0.994031	0.830776	-0.11009	8.461774
RPL4	0.994031	0.830793	-0.11963	9.642343
RNA5-8SN5	0.994031	0.8308	0.111277	12.22539
RAB36	0.994031	0.830827	-0.23962	3.296248
DLX3	0.994031	0.830923	-0.61	1.297821
RIOK1	0.994031	0.830949	-0.21287	4.185356
CLK1	0.994031	0.830971	-0.10501	6.115428
PPP1CC	0.994031	0.830984	0.132384	4.697998
BORCS6	0.994031	0.831081	-0.24989	3.331531
IGHMBP2	0.994031	0.83119	0.102026	5.346536
PRXL2C	0.994031	0.831202	-0.21335	4.257668
LOC101928	0.994031	0.831212	-0.33101	2.548898
GPATCH8	0.994031	0.83129	0.203463	2.921615
FNDC9	0.994031	0.831332	0.23719	1.683806
AKR1B1	0.994031	0.83138	0.106666	4.76522
ETV3	0.994031	0.831568	-0.14719	4.922643
FAM136A	0.994031	0.831613	0.099298	5.998088
DCAF5	0.994031	0.83164	-0.22652	3.787914
CAPZB	0.994031	0.831654	0.109589	4.47051
SBK3	0.994031	0.83174	0.266056	1.016521
NCOA5	0.994031	0.831789	0.087969	6.223095
C1orf220	0.994031	0.831941	0.148011	2.949272

ATP1A1-AS	0.994031	0.832071	-0.24806	2.970486
ZNF572	0.994031	0.832123	0.21	2.66553
NDUFB4	0.994031	0.832152	-0.40608	2.119672
LOC339874	0.994031	0.832221	0.164844	3.703632
MAP4K4	0.994031	0.832264	0.148368	3.683558
BFAR	0.994031	0.832284	-0.19301	3.635504
C7orf31	0.994031	0.832389	0.15223	3.525843
THSD4-AS2	0.994031	0.832426	0.154592	3.857837
GALNT16	0.994056	0.832522	0.257414	1.003191
ATXN7	0.994146	0.832741	-0.22147	3.162977
AP4M1	0.994146	0.83276	-0.17737	4.187059
PRADC1	0.994146	0.832842	-0.30733	2.427069
LINC00649	0.994146	0.8331	0.138643	3.755979
FAM21EP	0.994146	0.83316	-0.37824	2.071152
ARHGDIB	0.994146	0.83317	0.100437	6.259292
EBI3	0.994146	0.833318	0.287567	1.317875
LOC389831	0.994146	0.833325	-0.28194	3.458211
EMILIN2	0.994146	0.833442	-0.11587	5.637813
LOC100133	0.994146	0.833508	-0.5316	1.493444
TESK2	0.994146	0.833981	0.134548	4.245119
RMI1	0.994146	0.833989	0.123984	4.284615
PPP2R5B	0.994146	0.833997	-0.14298	4.783455
KLF3-AS1	0.994146	0.834031	0.258325	1.377255
GSS	0.994146	0.834034	0.104599	5.167976
LRRC46	0.994146	0.834053	-0.26284	2.588661
VPS37D	0.994146	0.834168	0.231935	1.452412
LOC101928	0.994146	0.83417	-0.5157	0.706672
GRIP2	0.994146	0.83417	0.162952	3.435536
KNDC1	0.994146	0.834188	-0.41719	2.425787
RNU4-2	0.994146	0.834307	0.09936	10.84831
SOX21	0.994146	0.834311	0.216909	2.271423
ALDH2	0.994146	0.83434	0.223844	1.541433
TIMM10	0.994146	0.834416	-0.18281	3.644124
FBN2	0.994256	0.834813	0.127851	5.312955
KIF15	0.994256	0.834849	-0.15732	4.833558
MYCL	0.994256	0.834857	0.275425	0.90443
SNORA5C	0.994256	0.835161	-0.22297	3.736601
LOC389834	0.994256	0.835457	-0.15637	5.841626
MBOAT2	0.994256	0.835541	0.117322	6.654201
NCOA7-AS1	0.994256	0.835542	-0.54279	1.060336
BCL11A	0.994256	0.835593	0.215403	2.446527
FUCA2	0.994256	0.835683	-0.11441	5.991768
ZNF80	0.994256	0.835713	-0.51462	1.533815
POLR2J4	0.994256	0.835967	-0.12189	5.334277
CORO6	0.994256	0.83603	0.197205	2.869324

RAB3A	0.994256	0.836059	-0.38748	2.108589
SLC35D2	0.994256	0.836223	0.169554	2.543748
DLGAP1-AS	0.994256	0.836262	0.294062	1.22243
NPEPL1	0.994256	0.836277	-0.39611	1.207054
C1QTNF6	0.994256	0.8364	-0.32277	2.233405
MANEA-DT	0.994256	0.836468	0.194036	2.78054
CLIC2	0.994256	0.836487	-0.21402	3.674035
ANKRD34A	0.994256	0.836595	0.202747	2.426746
SCARNA27	0.994256	0.836609	0.178405	2.820493
TMEM178I	0.994256	0.83668	0.186801	3.114268
LOC392232	0.994256	0.83676	-0.37205	1.455701
PPP6R1	0.994256	0.836765	-0.09953	7.9604
ARGLU1	0.994256	0.83684	0.086269	8.546047
CSKMT	0.994256	0.836868	0.150388	2.743517
HAUS7	0.994256	0.836875	-0.31796	1.981424
CUX2	0.994256	0.836966	0.292966	0.742496
RIMBP2	0.994256	0.837064	0.171433	3.953759
F13A1	0.994256	0.837187	0.119601	6.629752
TENT5B	0.994256	0.83722	0.168653	3.148267
RAPGEF2	0.994256	0.83728	-0.10117	7.314212
SLC2A13	0.994256	0.837418	-0.14008	5.788136
ERP44	0.994256	0.837507	0.081192	6.993584
MRPS21	0.994256	0.837539	-0.29264	2.337167
CLMAT3	0.994256	0.837697	0.209834	2.424473
TTC33	0.994256	0.837904	0.101811	5.325611
LINC00887	0.994256	0.837915	0.216763	1.698908
FAAP20	0.994256	0.837961	-0.19965	3.51324
KMT5A	0.994256	0.838005	0.081017	6.119856
OR2AE1	0.994256	0.838162	-0.70895	0.856357
AMY2B	0.994256	0.838172	-0.16649	4.405312
PSMA1	0.994256	0.838381	-0.22761	3.198116
RAB20	0.994256	0.838445	0.115147	5.073873
SLC41A2	0.994256	0.838496	0.08841	6.140031
LINC00910	0.994256	0.838496	-0.22725	2.38463
SNORA1	0.994256	0.838783	0.132039	5.139313
RPS23	0.994256	0.838815	0.086325	8.212581
WNT10A	0.994256	0.838821	-0.28991	3.786613
C3orf62	0.994256	0.838932	-0.21276	4.40355
RAET1K	0.994256	0.839155	-0.4677	0.911242
LOC101928	0.994256	0.839158	0.267951	0.905367
VWA8	0.994256	0.839168	0.099664	5.545719
TPM1	0.994256	0.839202	-0.16733	4.214001
FBLN5	0.994256	0.839332	-0.12867	6.114508
LOC100289	0.994256	0.839385	-0.24583	3.266548
RAB9B	0.994256	0.839517	0.167991	3.343955

E2F4	0.994256	0.83956	-0.10437	6.134819
NTNG2	0.994256	0.83959	-0.29246	2.699593
CNTNAP1	0.994256	0.839623	0.116107	5.532409
NFKBIB	0.994256	0.839719	0.123277	3.918215
YIPF2	0.994256	0.83974	0.092584	5.573138
GPR75	0.994256	0.839787	0.148839	3.028905
TMEM258	0.994256	0.839856	0.157083	3.274356
RNF207	0.994256	0.839881	0.093135	5.742584
PGM5	0.994256	0.839884	-0.19113	5.731846
SARM1	0.994256	0.840003	-0.17946	3.912335
CAPN2	0.994256	0.84012	0.126598	4.200124
TPGS1	0.994256	0.840391	-0.20006	3.140501
ISL1	0.994256	0.840423	-0.30146	3.193906
OSGEP	0.994256	0.840574	0.094729	4.970892
SELENOK	0.994256	0.840595	-0.14567	4.953225
MYO6	0.994256	0.840715	-0.34722	2.26252
HOXB-AS3	0.994256	0.840832	-0.35209	2.952239
PWP2	0.994256	0.840857	0.137515	3.405413
ZNF879	0.994256	0.840891	-0.37123	1.804761
LOC10192	0.994256	0.840899	0.244678	2.063574
ALOX12B	0.994256	0.840901	0.207495	2.615518
NFASC	0.994256	0.840902	-0.43884	1.640054
HOXA11	0.994256	0.84094	0.150103	3.154492
RPL7	0.994256	0.840968	0.090095	8.159279
NOL9	0.994256	0.841114	0.085507	6.252846
ANGPTL3	0.994256	0.841294	-0.2107	2.997386
CUL9	0.994256	0.841382	-0.10102	6.866854
RGSL1	0.994256	0.841388	0.294096	1.276654
MCU	0.994256	0.841549	0.120011	4.63297
RETREG2	0.994256	0.841776	-0.10517	6.311287
LOC100506	0.994256	0.841804	0.099621	5.938103
ELOF1	0.994256	0.841949	-0.13424	4.341076
KCNAB2	0.994256	0.842025	0.199383	2.077799
ZFP2	0.994256	0.842081	0.245107	1.529693
SLC35B3	0.994256	0.842099	-0.66182	0.978775
CLPP	0.994256	0.842112	-0.15885	3.925725
FEZ1	0.994256	0.842195	-0.23538	3.365353
AQP3	0.994256	0.842204	0.170323	7.454883
WEE1	0.994256	0.842241	-0.21213	2.903209
TSG101	0.994256	0.84226	-0.11614	5.57193
AGBL4	0.994256	0.842309	0.205135	2.188561
CRIPT	0.994256	0.842381	-0.11316	5.943834
MBTD1	0.994256	0.842689	0.084665	6.258262
TMED10	0.994256	0.842775	0.078877	8.418338
CCS	0.994256	0.842997	0.110427	4.721975

RUFY3	0.994256	0.843197	0.155994	3.101071
TCTN3	0.994256	0.843216	-0.36913	1.652071
TAOK1	0.994256	0.843246	0.122912	4.597965
CFAP74	0.994256	0.843306	0.236898	1.240376
C1orf74	0.994256	0.843338	0.146276	3.748674
STARD4-AS	0.994256	0.843415	-0.14117	4.8474
MRC1	0.994256	0.843543	0.106544	7.166287
PYGO1	0.994256	0.843674	-0.22543	3.89935
SNORD35A	0.994256	0.843742	-0.14421	5.9493
TOE1	0.994256	0.843797	-0.16086	3.989824
LOC10192S	0.994256	0.843896	-0.12304	5.4732
LINC01482	0.994256	0.843968	0.256148	1.344438
OR7E2P	0.994256	0.843974	-0.29063	2.475449
PET117	0.994256	0.843975	-0.60395	0.621939
SHC4	0.994256	0.843998	0.237697	1.546764
MAN1A1	0.994256	0.844012	0.08812	7.49917
TCF4-AS1	0.994256	0.844108	-0.46256	1.063897
SPG11	0.994256	0.84412	0.166472	2.475746
MAGEF1	0.994256	0.844179	-0.10417	5.533086
RPL18A	0.994256	0.84423	0.095439	7.561647
MRPL28	0.994256	0.844306	0.11684	4.258977
SPPL2A	0.994256	0.844574	0.084165	5.714058
RANBP9	0.994256	0.844646	-0.10504	6.937778
ARFGEF1	0.994256	0.844679	0.073359	8.336629
CAB39	0.994256	0.844733	0.109734	4.238634
LOC64602S	0.994256	0.844776	-0.47956	1.130528
TMEM265	0.994256	0.844857	-0.15463	4.168366
VIM	0.994256	0.844892	-0.11523	8.822448
IGIP	0.994256	0.844896	-0.1444	5.125071
RBM27	0.994256	0.844962	0.075849	7.326225
LOC101927	0.994256	0.845023	0.138397	3.652786
ARRDC3-AS	0.994256	0.845035	-0.29711	2.971118
LHX4-AS1	0.994256	0.845036	-0.1998	3.438534
SNORD98	0.994256	0.845141	-0.42576	1.167303
ZNF141	0.994256	0.845175	-0.14201	4.619452
PRR14L	0.994256	0.845237	0.076097	8.008507
IMPA2	0.994256	0.845362	0.095451	6.191311
CCAT1	0.994256	0.845383	-0.15081	4.467048
PTPRQ	0.994256	0.845547	-0.52538	0.921201
ALG11	0.994256	0.84556	0.120434	4.451703
TMED7	0.994256	0.845643	-0.13666	6.593776
CLEC4D	0.994256	0.846037	-0.42812	1.282973
TMEM268	0.994256	0.84609	-0.12757	4.954043
ATAD3B	0.994256	0.846166	0.118067	4.010601
ZNF341-AS	0.994256	0.846239	0.277133	0.990951

RAD51C	0.994256	0.846277	-0.50808	0.904129
LOC72861:	0.994256	0.846295	-0.16431	4.34212
VTRNA1-1	0.994256	0.846345	0.106416	8.407111
SLC20A2	0.994256	0.846381	-0.40926	1.725516
MRPL17	0.994256	0.846413	-0.11978	4.915841
TMED10P1	0.994256	0.846438	-0.22753	3.534508
CGAS	0.994256	0.846463	0.115149	3.946757
DHRS3	0.994256	0.846493	-0.12605	6.241938
LCN10	0.994256	0.846519	0.198364	3.426805
TWIST2	0.994256	0.846584	0.186569	2.795305
TRIM52	0.994256	0.846584	0.092918	5.234369
HLA-DMA	0.994256	0.846729	-0.40545	0.629104
SLC7A1	0.994256	0.846737	0.086058	7.356793
TREML2	0.994256	0.84677	0.170555	2.048102
SAMMSON	0.994256	0.84682	0.230655	1.335158
ERCC6	0.994256	0.84686	-0.11824	5.335437
HAVCR1P1	0.99428	0.847007	0.187189	1.226439
ASH1L-AS1	0.99428	0.84707	-0.18854	3.667339
RPLP1	0.99428	0.847345	-0.52242	1.399739
VPS26C	0.99428	0.847357	-0.10525	5.792717
IL10RB	0.99428	0.847372	-0.11831	5.102638
SERTAD4	0.99428	0.847413	0.150926	3.71014
SNORA7B	0.99428	0.847462	-0.1302	5.739468
FAM149A	0.99428	0.847498	0.252271	0.641278
HCG18	0.99428	0.847724	0.090864	4.838795
ZC3H7B	0.99428	0.847727	-0.09421	7.373203
PRDX4	0.99428	0.847792	0.099757	5.53292
SNX30	0.99428	0.84789	0.082133	6.218967
LOC101927	0.99428	0.848028	0.137601	3.95493
CHDH	0.99428	0.848213	0.093779	5.100389
TIMELESS	0.99428	0.848372	-0.10143	6.001436
HMOX1	0.99428	0.848493	-0.21686	4.634876
TMEM203	0.99428	0.848526	0.132032	3.756451
HDAC9	0.99428	0.848535	0.27866	1.253891
JPT1	0.99428	0.848582	-0.23875	2.617223
VENTX	0.99428	0.848782	-0.42972	1.208088
DLEU7	0.99428	0.848807	-0.16656	4.093407
CCR8	0.99428	0.848934	0.264762	1.492063
MAF1	0.99428	0.849292	-0.10164	5.715437
HSBP1	0.99428	0.849361	-0.12106	5.958317
CENPC	0.99428	0.849409	-0.09362	6.498832
ZNRD1ASP	0.99428	0.849502	-0.17613	3.694722
PHF2	0.99428	0.849557	-0.09647	6.791988
ZNF14	0.99428	0.849661	-0.23794	3.766852
TUBE1	0.99428	0.849714	-0.15224	4.691027

PPP1R16B	0.99428	0.849768	0.22116	1.365027
ETFA	0.99428	0.849786	0.210575	1.276892
ZFHX3	0.99428	0.849847	-0.10215	6.069331
ATXN2	0.99428	0.849848	-0.45844	1.027312
IMP3	0.99428	0.849888	-0.14828	4.493463
SERAC1	0.99428	0.849892	-0.16309	3.804603
APBA3	0.99428	0.850139	-0.14589	4.43881
ENDOD1	0.99428	0.850317	-0.11127	6.784435
LINC01477	0.99428	0.850331	-0.8349	1.110799
CCDC85A	0.99428	0.850372	-0.42751	1.899241
GPR171	0.99428	0.850482	0.237059	1.718273
ANXA5	0.99428	0.850749	-0.09388	8.219153
EMILIN1	0.99428	0.850805	-0.10759	7.578662
CSTF2T	0.99428	0.850833	-0.10289	6.197124
NAGPA	0.99428	0.850888	-0.1966	3.02943
SLC8A1-AS	0.99428	0.850938	-0.22399	2.986138
PIGH	0.99428	0.851047	0.162022	2.803982
TMOD3	0.99428	0.851089	0.078434	7.966112
NWD2	0.99428	0.851135	-0.42817	1.26689
TM4SF20	0.99428	0.851166	-0.32323	6.956382
GAMT	0.99428	0.851178	0.234793	1.308965
TUBGCP2	0.99428	0.851295	0.216063	1.774118
PER1	0.99428	0.851356	-0.10188	7.452652
NUBPL	0.99428	0.851452	0.287645	0.819407
FLJ16779	0.99428	0.851469	0.201889	1.613997
LOC101928	0.99428	0.85152	0.269187	1.272396
CERS4	0.99428	0.851793	-0.31761	2.698575
MSL3	0.99428	0.851793	0.132319	3.736126
MIR98	0.99428	0.851802	-0.39694	1.624093
YARS	0.99428	0.851863	0.07784	6.665684
NEFL	0.99428	0.851977	-0.57439	1.16449
PMM2	0.99428	0.852119	-0.09622	6.890457
ZNF662	0.99428	0.852395	-0.42084	1.520042
ERICH2	0.99428	0.852429	-0.49519	1.103831
LSM11	0.99428	0.852435	0.12414	4.43996
N6AMT1	0.99428	0.852504	-0.41093	0.75397
LHFPL3	0.99428	0.852529	0.18183	2.837631
PTGES2	0.99428	0.852623	0.19038	1.896683
FBRSL1	0.99428	0.852714	-0.10351	6.439799
PANK2	0.99428	0.852735	-0.23983	2.301583
TM2D1	0.99428	0.852792	0.089481	5.432658
STX7	0.99428	0.852917	-0.08938	7.158314
OPHN1	0.99428	0.853023	0.075966	5.627147
QTRT1	0.99428	0.853175	-0.1068	4.932036
TMEM184I	0.99428	0.853281	0.194814	2.310516

TLR1	0.99428	0.853332	0.125627	3.613977
FPR3	0.99428	0.853463	-0.19585	4.611727
AK9	0.99428	0.853525	0.117641	4.255028
LINC01342	0.99428	0.853577	0.136376	3.591657
MAP3K13	0.99428	0.853669	0.10227	4.777646
SNORD113	0.99428	0.853714	0.27405	1.312337
MSC	0.99428	0.853808	-0.24133	3.229869
ZUP1	0.99428	0.853837	-0.11819	4.767823
WBP2	0.99428	0.853863	-0.08782	6.59613
GPR82	0.99428	0.853876	0.099969	4.272331
ADSSL1	0.99428	0.853876	0.174806	0.847993
ZNF835	0.99428	0.853879	0.287654	0.918925
HOXA3	0.99428	0.853934	0.215708	1.419268
APPL1	0.99428	0.854	-0.09675	7.173989
PIAS1	0.99428	0.854038	0.076456	6.540239
ST7	0.99428	0.85404	0.194323	2.634761
ABCB10	0.99428	0.854387	0.081717	5.140459
FLOT2	0.99428	0.854426	-0.09562	6.477015
GBE1	0.99428	0.854434	-0.10517	5.539321
ARL2BP	0.99428	0.854468	-0.12511	5.161888
CCER2	0.99428	0.854555	0.194724	1.089389
AQP5	0.99428	0.854648	-0.2247	4.829754
HIST1H2BC	0.99428	0.85481	0.089475	6.627159
ZNF362	0.99428	0.855082	0.086559	5.476672
ARHGEF12	0.99428	0.855245	-0.5323	1.620582
THOC7-AS1	0.99428	0.855257	0.14073	2.435657
FRG1BP	0.99428	0.85528	-0.12794	5.377223
NBEAL1	0.99428	0.855352	0.070746	8.062019
EPB41L4A-,	0.99428	0.855377	0.106009	4.051044
IRX2	0.99428	0.855408	-0.46918	1.772621
SMG8	0.99428	0.855463	-0.13147	4.577484
HINT1	0.99428	0.855562	0.190085	1.204696
MID1IP1-A	0.99428	0.855564	0.17452	2.849939
SLC4A1AP	0.99428	0.85563	-0.09197	6.324859
EXOSC5	0.99428	0.855672	0.131466	2.779213
SYBU	0.99428	0.85571	0.177809	1.933039
HCAR2	0.99428	0.855813	-0.41676	1.411692
PRKCB	0.99428	0.855872	-0.12944	4.465615
TDRD3	0.99428	0.856001	-0.47524	1.120401
JRKL	0.99428	0.856021	-0.40722	1.640505
TUSC2	0.99428	0.856043	0.100723	4.183763
SDCBP2	0.99428	0.856061	-0.25586	2.535326
WAC	0.99428	0.856096	0.147647	2.147404
PPM1J	0.99428	0.856137	0.200699	1.210708
SNORA71A	0.99428	0.856283	0.104354	5.461992

NDUFAF6	0.99428	0.85631	0.113099	4.178367
SLC16A10	0.99428	0.856388	-0.21151	3.171317
HIST1H2BB	0.99428	0.856429	-0.14295	4.870835
SHROOM4	0.99428	0.856432	-0.18578	3.569953
HIST1H4C	0.99428	0.856445	-0.11559	5.631906
SLC10A7	0.99428	0.856609	-0.30426	2.373313
ANKRD63	0.99428	0.856643	-0.7633	0.94379
FEZ2	0.99428	0.856693	0.177507	1.216814
RHOD	0.99428	0.856695	0.184474	1.296653
C2orf68	0.99428	0.856724	-0.09937	6.172666
PAIP2B	0.99428	0.856785	0.137989	4.25078
GP1BA	0.99428	0.856813	-0.22636	3.241231
PTGES3	0.99428	0.856912	0.11241	4.899042
LIPC-AS1	0.99428	0.857059	-0.5148	1.127934
SLC35F2	0.99428	0.857097	-0.11755	5.163344
YTHDC2	0.99428	0.857111	0.070545	6.654774
OVCH1	0.9945	0.857377	0.185495	2.051601
ACHE	0.994519	0.857469	-0.50163	1.171959
DGCR6L	0.994628	0.857639	0.108827	3.585782
SLC25A44	0.994637	0.857788	-0.24238	3.029584
LOC100132	0.994637	0.857852	0.147399	2.063079
COMT	0.994637	0.857934	-0.21574	3.533616
MIR3176	0.994637	0.858036	0.163216	1.723792
LINC01132	0.994637	0.858113	-0.34291	1.273265
FHL1	0.994637	0.858156	0.172905	2.850113
FTH1P3	0.994637	0.858298	-0.14555	3.918671
MS4A4A	0.994637	0.858301	0.246572	1.010263
SNORA110	0.994637	0.858406	-0.43691	0.90017
TMEM154	0.994637	0.858454	0.119643	4.419436
EPS15	0.994637	0.858481	-0.12714	5.470898
GJA1	0.994826	0.858823	-0.13312	6.924342
CP	0.994826	0.858839	0.199494	1.827999
NTHL1	0.994826	0.858871	-0.28046	3.074007
CYBC1	0.994957	0.85906	0.098746	3.634838
PSORS1C2	0.995222	0.859365	-0.37107	2.126507
EFNB1	0.995251	0.859536	-0.13485	6.434191
RBM22	0.995251	0.859573	-0.0947	5.937502
LOC100134	0.995251	0.859617	-0.21428	3.489331
ASCC3	0.995426	0.859906	0.067094	7.034876
LOC729970	0.995426	0.85992	-0.38731	1.876957
ERI3-IT1	0.995495	0.860056	0.113043	3.425222
BDKRB1	0.995581	0.860342	0.218362	0.73727
POLE4	0.995581	0.860451	-0.17269	3.641231
TRANK1	0.995581	0.860597	0.075116	7.468921
ISL2	0.995581	0.860653	0.20456	1.325378

LOC10099€	0.995581	0.860796	-0.62288	1.929086
METRNL	0.995581	0.860801	0.128192	3.672809
DNAJB9	0.995581	0.860849	0.083145	5.584471
NUDT15	0.995581	0.860898	-0.12176	4.596845
BBOF1	0.995581	0.860992	-0.15161	4.311699
DCAF10	0.995581	0.861063	-0.24557	2.233364
RIOK3	0.995581	0.861063	-0.10579	7.71198
DCAF7	0.995581	0.861079	0.064768	6.955748
MED16	0.995581	0.861117	-0.09308	5.717764
ACOT2	0.996183	0.861801	0.160381	1.946891
VPS35	0.996183	0.861814	0.067778	7.190233
SLC35E1	0.996183	0.861865	-0.08332	6.747548
C1QBP	0.996263	0.862152	-0.10954	6.071627
GRHPR	0.996263	0.862159	-0.10799	5.209812
BRK1	0.996263	0.86245	0.073501	5.988172
GPC6	0.996263	0.862541	0.090088	5.917743
FAM72D	0.996263	0.862628	0.170843	1.979925
COX10-AS1	0.996263	0.86272	-0.15784	3.799265
SNORD25	0.996263	0.862772	-0.30089	1.787091
CTSS	0.996263	0.862779	0.173392	1.608531
C8orf82	0.996263	0.862847	-0.14515	3.840887
PEAK3	0.996263	0.862888	0.179278	1.711065
DLEU1-AS1	0.996263	0.862926	0.10422	3.199197
POTEG	0.996263	0.863032	0.232241	1.22995
EXOC6B	0.996263	0.86312	-0.08589	6.569942
EBF4	0.996263	0.863143	-0.19987	3.209818
BOD1L1	0.996263	0.863205	-0.0799	8.773618
ACBD7	0.996263	0.863284	0.167315	1.632929
LOC103021	0.996263	0.863312	0.122182	2.928734
DYNC1H1	0.996263	0.86346	-0.07977	10.22699
SLC35E2B	0.996263	0.863661	-0.40348	1.233871
CNST	0.996263	0.863691	-0.10288	4.969028
DPH2	0.996263	0.863719	0.156742	2.793991
OPN1SW	0.996263	0.86372	0.220645	0.86163
PTPN2	0.996263	0.863974	0.170546	2.576369
TMEM87B	0.996263	0.864014	-0.08889	7.58955
STX12	0.996263	0.864021	-0.08701	6.442996
SLC25A38	0.996263	0.864273	0.093687	4.845576
DUT	0.996263	0.864303	0.158221	1.291165
TAS2R30	0.996263	0.864397	-0.53789	0.76893
MKNK1-AS	0.996263	0.864397	0.108997	3.408593
PCGF1	0.996263	0.86446	-0.13908	3.967326
MTMR11	0.996263	0.864523	0.142271	2.295924
KCNMB2-A	0.996263	0.864557	-0.51245	1.522707
CNPY4	0.996263	0.864577	0.104823	4.088753

HIST1H4B	0.996263	0.864612	-0.10526	5.119981
SFPQ	0.996263	0.864664	-0.08074	9.609968
ITPRIP	0.996263	0.864725	-0.22814	3.031707
ATF5	0.996263	0.864744	0.152735	2.410842
SNORA92	0.996514	0.865121	0.156623	1.86376
C1orf116	0.996514	0.865127	-0.34799	1.521684
MIR155HG	0.996514	0.865206	-0.29559	2.205422
NDUFA6	0.996514	0.865271	-0.19812	4.643704
SLC25A35	0.996514	0.86541	0.188213	1.750788
FAM41C	0.996514	0.865443	0.247558	0.846941
TMEM86B	0.996514	0.865523	-0.1421	4.021064
MIR30E	0.996514	0.865622	-0.44055	0.980017
LOC389641	0.996514	0.865686	0.157916	2.539605
ABCA10	0.996514	0.865721	-0.16741	5.640815
LAMP2	0.996604	0.865876	-0.10271	6.551391
CERCAM	0.996607	0.865954	-0.39911	0.952173
FOXL1	0.996638	0.866171	-0.18521	3.324807
ZNF616	0.996638	0.866214	0.089188	4.51407
SNORD96A	0.996638	0.866296	-0.15793	4.502341
EVI2B	0.996638	0.866446	-0.09564	6.168345
TCAP	0.996638	0.866465	0.133287	1.155424
IFT43	0.996638	0.866636	-0.39003	1.219617
TXN2	0.996638	0.866676	0.072402	5.380807
LCN6	0.996638	0.866834	0.219268	0.874095
C5orf15	0.996638	0.866871	-0.10567	5.428629
HOXC-AS2	0.996638	0.866906	-0.20564	3.066334
ZMIZ1	0.996638	0.866911	-0.07776	7.795918
MAMSTR	0.996638	0.866954	0.239005	0.705866
MDH2	0.996638	0.866969	0.164874	0.703042
SELENOH	0.996747	0.867168	0.077487	5.505689
RANBP6	0.996747	0.867216	-0.18365	2.770572
COL22A1	0.996846	0.867378	-0.24662	2.935727
PLPP3	0.996906	0.867525	-0.10687	5.946833
SH3PXD2B	0.996906	0.867582	0.074007	7.326879
RNF115	0.996936	0.867684	0.069721	6.041521
MIR1249	0.997114	0.86816	0.213121	0.724903
EMB	0.997114	0.868222	-0.09101	6.426653
MRPL1	0.997114	0.868243	-0.21203	3.63959
ACAP1	0.997114	0.868249	0.083248	4.561347
CPAMD8	0.997114	0.86829	0.177282	1.874808
PRPSAP1	0.997114	0.868407	0.071236	5.319972
VPS13B	0.997114	0.868411	0.18088	1.588575
LOC28314(	0.997114	0.868479	0.101839	4.838538
TMEM183/	0.997114	0.868689	0.061484	6.151169
LINC02043	0.997114	0.868769	-0.41267	0.747294

MERTK	0.997114	0.868812	0.077845	6.159238
MRPL50	0.997114	0.868832	-0.09681	5.430201
SEMA3F	0.997114	0.868914	-0.14261	5.297429
MAGOHB	0.997114	0.869037	0.113462	3.647589
UBE2E4P	0.997114	0.869127	0.151287	2.840892
CEP19	0.997114	0.869172	0.131305	2.156903
ZSCAN16	0.997114	0.869251	0.093833	4.38763
ZNF710	0.997114	0.869304	0.07277	5.623387
SEC61B	0.997114	0.869344	0.077657	4.913625
HP1BP3	0.997114	0.869359	-0.07769	8.084521
MCM3AP-A	0.997152	0.869518	-0.55012	0.714199
ZNF202	0.997152	0.869544	0.218159	0.569009
ANKRD37	0.997152	0.869668	-0.32963	1.920497
ESPN	0.997152	0.869696	0.093928	4.69915
LMBR1L	0.997209	0.869845	-0.32783	1.914395
ZNF749	0.997209	0.870132	-0.12728	4.59366
FH	0.997209	0.870173	-0.11159	5.406598
YWHAH	0.997209	0.870382	0.062301	7.372522
HKDC1	0.997209	0.870447	0.101382	6.967783
TAS2R31	0.997209	0.870544	0.126314	3.18753
MIR548AR	0.997209	0.870564	-0.37197	2.263176
SLAIN2	0.997209	0.870614	0.060529	6.845997
HBB	0.997209	0.870633	0.138217	3.398914
MGC16275	0.997209	0.870653	-0.34273	1.113341
NHEJ1	0.997209	0.870704	-0.129	4.113804
SLC30A9	0.997209	0.870741	0.064704	7.091887
NPAS2	0.997209	0.870754	0.062716	6.477978
LINC01355	0.997209	0.87081	0.084472	4.851618
EIF3A	0.997287	0.870954	0.067164	10.40563
FAM89A	0.997309	0.87105	-0.26356	2.632991
FAM110B	0.997467	0.871408	-0.19703	3.125392
SAT2	0.997467	0.871443	0.075087	5.028551
ELP1	0.997467	0.871471	0.063331	6.661519
ATF3	0.997467	0.871525	-0.33882	1.618927
C19orf24	0.997467	0.871567	0.093986	3.627252
TMTC3	0.997471	0.8717	-0.08734	5.901989
FSCN1	0.997471	0.871723	0.08362	5.214977
SDR42E1	0.99756	0.871877	-0.1479	3.813436
CCDC8	0.997568	0.872028	-0.24904	3.786729
ERVFRD-1	0.997568	0.872035	-0.1839	3.830201
SORCS2	0.997725	0.872249	0.087633	4.146483
CHD7	0.99784	0.872481	-0.09937	7.499289
TEX10	0.99784	0.872502	-0.39195	1.101968
HLA-DRB6	0.997868	0.872602	-0.34289	1.149131
DCAF4L1	0.997881	0.872689	-0.34151	1.416289

TNRC18P1	0.997953	0.87292	0.137097	2.397929
TOR1AIP2	0.997953	0.87296	-0.08221	6.497526
TRIM14	0.997953	0.872991	-0.09091	5.883862
FAXDC2	0.997953	0.873093	-0.11828	5.268843
TEX45	0.997953	0.873181	0.145616	1.24191
LOR	0.997953	0.873209	0.199963	0.972483
RCAN1	0.998072	0.873406	-0.13749	3.91004
MRPS22	0.998072	0.873526	-0.0908	5.655219
HIST1H4L	0.998072	0.873565	-0.21137	3.857466
TSL	0.998072	0.873659	-0.42003	0.911105
CIC	0.998072	0.873758	-0.09185	5.829324
ADGRB1	0.998072	0.87388	-0.27483	2.304678
ASPN	0.998072	0.873883	-0.28419	2.374288
ASB8	0.998072	0.873921	-0.10317	4.450345
SLC25A43	0.998132	0.87405	-0.11306	4.677113
FOXA1	0.998226	0.874209	-0.11969	6.095315
SSH3	0.998412	0.874448	0.068017	6.146828
RGS1	0.998434	0.87462	-0.10788	6.992827
STIP1	0.998434	0.874668	-0.38916	1.274713
PLA2G12A	0.998434	0.874882	0.067671	5.84282
EGLN3	0.998434	0.874939	0.132687	2.447538
PIGV	0.998434	0.875022	-0.31144	1.154858
COL1A1	0.998434	0.875054	-0.08626	11.41645
LRRC8B	0.998434	0.875153	0.143588	2.522986
NECTIN4	0.998434	0.875168	0.092215	6.329395
DNAH9	0.998434	0.87528	0.181453	1.413142
TMEM81	0.998434	0.875346	0.143518	1.41211
ICMT	0.998434	0.875383	-0.07595	6.279088
PRIM2	0.998434	0.875415	0.096758	3.469486
PGRMC1	0.998434	0.875586	-0.29178	1.023532
SLC38A7	0.998434	0.875642	-0.25314	2.130092
SLC25A15	0.998434	0.875657	-0.1001	4.941524
SHF	0.998434	0.875829	-0.26061	3.025485
FAT1	0.998434	0.875859	-0.08331	10.53928
TRIOBP	0.998434	0.875945	0.093505	4.486151
NPRL2	0.998434	0.876038	-0.13754	3.843497
CDAN1	0.998434	0.87605	-0.09417	5.245698
PLA2G15	0.998434	0.876143	-0.14033	3.689908
ADAL	0.998434	0.876253	0.156683	2.070326
FN3K	0.998434	0.87644	0.083053	5.065312
IGF2	0.998434	0.876486	-0.29518	1.650437
NGFR	0.998434	0.876489	0.120709	3.167203
LRIF1	0.998434	0.876514	0.128861	3.007164
LRRC37A8P	0.998434	0.876586	0.166729	2.309702
POMT2	0.998434	0.876598	0.076598	4.420049

CXorf38	0.998437	0.876736	0.065479	5.591118
ARHGAP31	0.998437	0.87678	0.07358	5.890262
TUBGCP3	0.998437	0.876829	-0.25978	2.753332
TMEM250	0.998531	0.877002	-0.37281	1.568716
PSMG3	0.998531	0.877112	-0.28211	1.586959
WAS	0.998531	0.877206	-0.15773	3.445417
PIGU	0.998531	0.877256	-0.1346	4.086191
NKTR	0.998531	0.877292	-0.07844	9.268299
SEC14L1P1	0.998587	0.877504	0.111713	2.968369
TMEM161I	0.998587	0.877536	0.108432	3.107751
ZNF445	0.998587	0.87763	0.059764	7.028281
TNFSF4	0.998587	0.877678	0.169842	1.641802
ZNF197	0.998587	0.877721	-0.09619	5.607998
PTPRCAP	0.998658	0.877903	-0.1367	3.728389
TSPAN15	0.998658	0.87803	0.067965	6.643429
CD1D	0.998658	0.878102	0.121418	2.663428
RARG	0.998658	0.878139	0.164743	1.566312
ADAP2	0.998658	0.878164	-0.11622	4.338194
PCDHB17P	0.998678	0.878259	-0.4413	0.845412
STXBP5-AS	0.998976	0.87867	-0.40432	0.899257
SPATA5	0.998976	0.878673	-0.10402	4.903129
PPDPF	0.999057	0.878966	0.063245	7.70636
GABBR2	0.999057	0.87899	-0.49347	0.692972
AURKAP1	0.999057	0.879143	-0.20691	2.82671
LOC91450	0.999057	0.879197	-0.31324	1.693537
C15orf62	0.999057	0.879217	-0.12381	3.955687
ZNF449	0.999057	0.879252	-0.10663	4.348083
OSTF1	0.999057	0.879277	0.080021	5.338959
POU5F1P5	0.9991	0.879466	0.102771	2.578329
TRIM25	0.9991	0.879484	0.056182	7.284693
NRM	0.9991	0.879567	-0.3911	0.796847
CHCHD3	0.9991	0.879619	0.189385	0.938593
ADAMTS1C	0.999183	0.87982	0.109199	4.070191
C1orf195	0.999183	0.879845	0.107405	2.993856
ABI3BP	0.999323	0.880091	-0.11594	6.562806
COQ3	0.999323	0.88012	0.103189	3.103535
PPM1D	0.999406	0.880521	-0.08525	5.324586
PPP4R1	0.999406	0.880639	-0.24173	2.327798
GPR153	0.999406	0.880729	0.079711	4.916786
CDK16	0.999406	0.880842	-0.17012	2.519012
PCSK9	0.999406	0.88085	-0.46559	1.113701
KCNK12	0.999406	0.880886	-0.43211	0.968299
CNOT2	0.999406	0.880995	0.190705	1.125917
LOC101928	0.999406	0.881	-0.19422	2.244808
LYZ	0.999406	0.881089	0.103271	11.47792

STAM-AS1	0.999406	0.881098	-0.41773	0.728486
ATP5MD	0.999406	0.881117	-0.24963	2.087815
FAM169A	0.999406	0.881291	-0.5188	1.254166
MXRA5	0.999406	0.881365	-0.09291	9.16449
SNORA65	0.999406	0.881367	-0.12261	7.477121
ZNF192P1	0.999406	0.881635	0.109938	2.428717
WDR83OS	0.999406	0.88164	-0.2057	3.206665
POR	0.999406	0.881646	-0.08321	6.01162
HIC2	0.999406	0.881656	-0.09651	5.18088
SUPT7L	0.999406	0.881701	0.15943	1.398794
SAP30	0.999406	0.881717	-0.09573	4.879252
LPAR6	0.999455	0.881868	0.156732	1.614368
NIPBL	0.999455	0.881982	0.188121	0.78894
WBP1	0.999455	0.881989	0.092143	3.538438
RWDD2A	0.999614	0.882306	0.113635	3.09057
ELL2	0.999614	0.882356	0.061308	7.376136
BATF3	0.999614	0.882428	-0.21693	2.764024
PATL2	0.999614	0.882434	-0.19209	3.30302
EPHX1	0.999793	0.882668	0.176322	1.302445
MEF2A	0.999942	0.882878	-0.28508	1.88014
MRPS31P5	0.999942	0.882952	0.121	2.49952
TFRC	1	0.883113	-0.55482	0.866761
GPR157	1	0.883333	-0.31797	1.853896
SUSD6	1	0.883428	0.058722	7.164945
LINC02412	1	0.883453	0.196414	1.008933
LOC101927	1	0.883554	0.175249	1.467653
WDFY1	1	0.883864	-0.07025	7.058537
ADHFE1	1	0.884002	-0.20835	3.189059
FAM13A	1	0.884011	-0.19731	3.111777
NDEL1	1	0.88407	0.12448	2.404469
CYBB	1	0.884148	0.065869	6.096128
NMT2	1	0.884203	0.116375	3.075695
TAF3	1	0.884259	-0.08114	5.929891
PDP2	1	0.884304	-0.09875	4.77309
CLDN3	1	0.884352	0.084947	4.609988
ADAMTS9-.	1	0.884389	0.113918	3.470901
PAXX	1	0.884508	0.083212	3.92824
EPN1	1	0.884565	-0.21026	2.884591
HMGN2	1	0.884566	-0.07267	6.549142
SLC16A12	1	0.88465	-0.47689	1.148017
RSBN1L	1	0.885063	0.058182	6.058855
SPINK9	1	0.88518	-0.4815	1.335634
DLGAP4	1	0.885205	0.072811	5.020733
DIP2A	1	0.885261	0.107674	1.639657
ABHD14A	1	0.885399	0.105819	1.342827

NPEPPS	1	0.885428	-0.07891	7.285311
IKBIP	1	0.885611	-0.18619	3.444197
GDI2	1	0.885633	0.168266	1.204744
LOC10099€	1	0.88569	-0.20785	3.226386
ZNF580	1	0.885699	-0.21456	2.25687
CCDC157	1	0.885962	0.183904	0.85041
MAP1S	1	0.886099	-0.31775	2.025883
TACSTD2	1	0.886151	-0.14149	7.610253
MSH6	1	0.88626	0.137743	1.382565
LRRC8C	1	0.88634	-0.10349	5.113558
PSMD12	1	0.886534	0.092334	3.943384
RPS7	1	0.886603	0.0601	7.34266
PTPA	1	0.886668	-0.39999	0.722461
SLC18B1	1	0.886684	-0.10727	5.494036
SPIRE1	1	0.886691	-0.3774	0.77083
RDH11	1	0.88672	0.13149	1.327406
UHRF1BP1	1	0.886725	0.060628	6.630727
MT1X	1	0.886889	0.079155	4.90426
DPY19L1P1	1	0.886955	-0.27514	3.387491
RAB8A	1	0.887026	0.055687	7.297014
MIR1207	1	0.88714	0.107306	2.173936
CA5BP1	1	0.887337	-0.14345	3.166703
LRRC57	1	0.887439	0.069151	5.051241
CSNK1G1	1	0.887444	0.057408	6.13097
NFIB	1	0.887569	0.135591	1.530867
GPR162	1	0.887594	0.127796	1.785127
RNF103	1	0.887602	0.075595	3.952451
ARHGEF19	1	0.887684	-0.14862	3.691996
TMEM191/	1	0.887779	0.193024	1.098299
FAM193A	1	0.887881	0.132493	1.706321
SYNGR2	1	0.887888	-0.07798	7.343568
TOMM6	1	0.887971	0.054703	5.371796
SDHB	1	0.887982	-0.08861	5.56816
ZNF671	1	0.887996	-0.1624	4.011294
LAMTOR4	1	0.888156	-0.12759	4.699276
RANBP10	1	0.88822	0.063664	5.703653
NBEAL2	1	0.888224	-0.09929	8.727
PM20D1	1	0.888408	-0.17666	4.093505
TMEM179I	1	0.888522	0.065389	4.366501
CEBPB-AS1	1	0.888538	-0.28855	2.414049
WDR91	1	0.888753	-0.08378	5.308761
SND1	1	0.888774	-0.07893	8.009749
HIST1H3I	1	0.888852	-0.15303	4.670041
CREBBP	1	0.889011	0.165276	1.270687
EHD4	1	0.889095	0.061541	6.060429

SETD5	1	0.889116	-0.16811	3.123415
RUSC2	1	0.889131	-0.42964	0.776734
DDX12P	1	0.889147	0.064344	4.902207
AVL9	1	0.889163	-0.0746	7.233686
PI4KB	1	0.889175	-0.32459	1.519254
LINC00346	1	0.889177	0.093813	3.77265
TRIM11	1	0.889376	0.054728	5.319226
ZDHHC23	1	0.88941	-0.10204	5.330141
MAN2A2	1	0.889525	-0.07482	6.854479
GPI	1	0.889608	0.08694	3.823528
LOC102724	1	0.889622	0.154927	1.442796
ZNF688	1	0.889894	-0.23005	0.793364
BRD7P3	1	0.889948	0.119402	2.393392
KCTD20	1	0.890075	0.132556	2.559448
GGPS1	1	0.890177	0.085947	3.282842
RAB5C	1	0.890196	-0.36103	0.861628
PDZK1P1	1	0.890224	0.079117	4.346629
INPP5B	1	0.890294	-0.16002	2.733658
HLX	1	0.890413	-0.21525	2.322392
UQCC3	1	0.890458	-0.15737	3.745985
CYP2A13	1	0.890731	0.139416	1.204454
TAF11	1	0.891135	0.124177	1.47975
POMGNT2	1	0.89117	-0.16477	3.93583
SPCS3	1	0.891223	0.052936	7.190666
LOC100287	1	0.891285	-0.27056	1.013914
GPIHBP1	1	0.891293	-0.36203	0.830398
AFDN	1	0.891311	-0.09408	4.786359
GIGYF1	1	0.891509	-0.07027	7.918414
UBALD2	1	0.891512	0.055216	6.060041
INA	1	0.891666	-0.49376	0.759587
DNAJB12	1	0.891759	-0.15544	2.907805
PRSS22	1	0.891786	-0.10737	5.672572
SEC22A	1	0.891958	0.08042	4.878233
ZFP69	1	0.892015	-0.12524	4.306615
DAG1	1	0.892442	0.089126	1.867721
GCN1	1	0.892448	-0.07841	8.378522
KIF16B	1	0.892508	-0.20804	2.563744
ADM5	1	0.89253	0.125368	1.753014
GALE	1	0.892771	0.107419	2.094581
LIMD1	1	0.892805	-0.0828	5.836709
DDX59	1	0.892874	0.059595	5.30272
TXLNA	1	0.892924	-0.06773	7.555247
TRAM2	1	0.893002	0.050535	6.936202
FAM78A	1	0.893014	0.075427	4.099938
CLNS1A	1	0.893221	0.152859	0.866408

ELF1	1	0.893293	-0.35102	1.911257
CCBE1	1	0.89331	-0.28011	2.65063
ARMH3	1	0.893339	-0.0721	6.11282
BMPER	1	0.89336	-0.30369	2.348261
LCOR	1	0.893506	-0.07038	6.406922
EMP2	1	0.893512	-0.07609	7.081942
LMBR1	1	0.893618	0.052192	6.49905
FAM186A	1	0.893624	0.113056	2.875007
NYNRIN	1	0.893856	0.058882	5.655757
DFFA	1	0.893919	-0.18159	3.195112
SOX13	1	0.894045	0.065195	5.831837
ASPDH	1	0.894071	-0.3059	1.918682
FUT9	1	0.894083	0.099358	5.22009
RPS21	1	0.894157	-0.08506	7.630136
SLC25A3	1	0.89424	-0.2009	2.336787
OSBPL1A	1	0.894433	0.069184	4.655334
RPL19	1	0.894452	-0.07277	9.59327
LOC646903	1	0.894622	0.11612	1.925314
CKAP4	1	0.894711	-0.07073	7.07782
MOB3C	1	0.89475	0.109234	1.972816
MBP	1	0.89493	-0.08366	5.544452
CEP57L1	1	0.894954	0.098011	1.236864
L3MBTL4-A	1	0.894978	-0.33603	1.1385
SNORA36C	1	0.895004	0.12055	1.389651
STRADA	1	0.895037	-0.21627	2.398213
TSPAN32	1	0.895081	0.111318	2.091992
PCDHB5	1	0.895148	-0.23181	2.358041
FAM87B	1	0.895359	-0.25879	2.508946
RTF2	1	0.895379	0.148022	1.072615
HIST3H2BB	1	0.895524	-0.22886	3.101716
JADE2	1	0.895554	-0.25602	1.924815
GRIN2D	1	0.895608	-0.08702	5.662112
STK11	1	0.895619	0.05166	6.884488
ING1	1	0.895657	0.093355	3.623769
DBH	1	0.895749	-0.42464	0.728674
CYB5R3	1	0.895905	-0.18855	2.014323
PCDHB15	1	0.896082	0.080344	3.359018
SMIM18	1	0.896585	-0.35664	1.36004
VASP	1	0.896701	-0.06615	7.643082
SMNDC1	1	0.896737	-0.08535	5.409
HPS6	1	0.896774	-0.1057	4.4153
LOC100132	1	0.896861	0.147947	1.762
ICAM3	1	0.897001	0.072062	4.037615
ZNF329	1	0.897167	-0.16621	3.075413
MYCT1	1	0.897249	0.093584	3.108082

HKR1	1	0.897304	0.05388	6.672403
RNF31	1	0.897464	0.07136	3.329022
MIR3142H(	1	0.897637	0.097722	3.211877
LTBR	1	0.897704	-0.14713	3.761947
SHANK2-AS	1	0.89771	0.07567	3.047763
TG	1	0.897843	-0.22017	2.645636
MLYCD	1	0.897936	-0.10144	4.742281
SPSB3	1	0.898093	-0.08686	5.587019
CA3-AS1	1	0.898136	0.154402	1.336023
NACA4P	1	0.89826	-0.09995	4.217431
RPS18P9	1	0.898319	-0.174	2.650633
CCDC47	1	0.898356	-0.06283	7.840408
UVSSA	1	0.898362	-0.32373	1.659275
ORMDL3	1	0.898498	0.048616	6.341607
LOC102723	1	0.898554	-0.41906	1.121832
SNORD50B	1	0.898565	0.129133	2.075813
CCL22	1	0.898671	-0.19396	3.078439
JARID2	1	0.898696	-0.0988	4.227931
PPP1R12A	1	0.898733	0.082895	2.345518
CCDC60	1	0.898817	0.133272	1.812485
LOC101241	1	0.898975	0.108543	2.81515
MANF	1	0.899063	-0.09205	5.486854
ZDHHC8	1	0.899204	-0.13582	3.413897
ANAPC5	1	0.89925	0.087683	2.587178
POMZP3	1	0.899267	-0.42935	1.771686
TRAM1	1	0.899285	-0.16833	2.054505
NEMP2	1	0.899335	-0.14729	4.563103
WDR97	1	0.899341	-0.23235	1.887706
FBXO42	1	0.899357	0.05864	5.337058
KCNB1	1	0.899383	-0.32617	2.68016
RHOJ	1	0.899559	0.07203	4.326938
LINC01504	1	0.899596	0.141106	1.541527
ATRX	1	0.899675	-0.31396	1.173755
ZNF518A	1	0.8998	-0.5075	0.748327
LINC01356	1	0.899955	0.094434	2.362717
DAAM2	1	0.899971	-0.22275	2.089602
GAS7	1	0.899976	-0.24327	1.982444
SECISBP2L	1	0.900009	0.10727	0.905342
ZBTB7B	1	0.900009	0.129583	0.928924
A1BG	1	0.900142	0.113264	1.530123
NPC2	1	0.900265	-0.08111	5.284296
EPN2-AS1	1	0.900278	0.096656	3.004521
SNORA13	1	0.900316	-0.11164	6.1322
GPRIN3	1	0.900401	-0.08137	6.42713
LOC101929	1	0.900411	-0.30984	1.815039

UGT8	1	0.900483	0.103343	2.107834
SCX	1	0.900514	-0.19198	2.85018
TSC22D3	1	0.900555	0.061754	5.877242
SPDYE1	1	0.90071	-0.09713	4.837619
TXLNB	1	0.900842	0.096439	3.512961
FBXO4	1	0.900881	-0.30822	1.81118
ZCCHC2	1	0.901011	-0.07906	4.820029
RAI1-AS1	1	0.901014	-0.20749	2.530062
CSRNP1	1	0.901116	-0.07719	5.870613
RNF122	1	0.901159	0.091354	3.320709
RASL11A	1	0.901197	0.093212	2.467992
SEC23IP	1	0.901311	0.076553	3.988786
KCNA6	1	0.901334	-0.37344	1.174843
LIN7C	1	0.901356	-0.06649	6.669359
ANKRD13A	1	0.901402	0.051157	6.651236
EMC4	1	0.901565	0.111518	2.003304
PIK3CD-AS1	1	0.901622	-0.2907	0.689438
ARAF	1	0.901829	-0.31858	1.095379
JPH1	1	0.901862	-0.25133	2.517373
GLB1L2	1	0.901892	0.071981	4.728386
NDUFA13	1	0.901908	0.048591	6.015222
KCNE4	1	0.901928	-0.13004	3.854254
SCARNA20	1	0.902004	-0.17538	3.458836
SPAG1	1	0.902053	-0.34341	1.097649
WBP4	1	0.902114	0.057147	5.497617
FAM208A	1	0.902256	0.049156	5.64879
CHIAP2	1	0.902519	0.121524	0.84238
SELENOP	1	0.902544	-0.36736	1.684835
KLF16	1	0.902627	0.050236	5.246433
ZNF350-AS	1	0.902868	-0.41622	1.083208
STX16-NPE	1	0.902884	-0.40103	1.203463
TXK	1	0.902968	0.10312	2.828217
HECTD4	1	0.902981	0.043841	8.416442
ABRAXAS2	1	0.903143	-0.09653	5.362838
HCG11	1	0.903217	-0.08757	5.330764
KCNMB4	1	0.903222	-0.25239	1.985708
PDF	1	0.903338	0.11737	1.011021
LOC101927	1	0.903406	-0.35118	0.747883
LURAP1L-A	1	0.903441	-0.26571	2.047202
SP2-AS1	1	0.90347	0.084871	1.765897
HMGN1	1	0.903502	-0.07345	6.784521
ATP5MC3	1	0.903517	-0.24262	2.486588
CCDC130	1	0.903637	0.050047	5.295372
ARHGAP35	1	0.903715	-0.05977	7.96945
LOC100128	1	0.90376	0.076669	3.430245

CD36	1	0.90393	-0.18449	2.793884
C12orf66	1	0.90395	0.07968	1.646956
USP12-AS1	1	0.904032	0.079555	2.968327
HYDIN	1	0.904219	-0.3542	1.745632
DIP2C	1	0.904343	-0.06655	6.724673
RBM25	1	0.904358	0.045039	9.450057
NUTM2G	1	0.904463	-0.09763	4.384049
RALA	1	0.904488	0.045295	6.916082
HINT2	1	0.904551	0.073862	3.452465
TTLL11	1	0.904587	0.08461	2.298149
NT5DC1	1	0.904743	0.063286	5.187538
FOXF1	1	0.904769	-0.08866	5.496628
BRINP1	1	0.904929	-0.41045	1.106741
FBXO46	1	0.905083	0.054909	5.345346
STXBP3	1	0.9053	-0.06346	6.200239
DEPDC1	1	0.905595	0.100269	2.350885
COX17	1	0.905627	0.055226	4.699585
ASAP1	1	0.905789	0.114027	1.577886
RPL23	1	0.905829	-0.0675	8.867944
TRMT61A	1	0.905885	-0.11474	4.056302
DENND6B	1	0.905978	-0.12542	3.395942
GNPTAB	1	0.905983	-0.06019	7.134945
EID3	1	0.906214	-0.12671	4.472504
LOC101929	1	0.906313	0.095352	2.133288
LOC100506	1	0.906377	0.088548	1.689909
RPS8	1	0.906438	0.047757	9.058921
ZNF845	1	0.906684	0.064732	4.440946
ZNF780B	1	0.906777	-0.07097	6.471913
C3orf70	1	0.90699	0.065387	4.761311
WDR49	1	0.90702	-0.33826	2.517059
ARAP3	1	0.907193	0.054737	5.363596
SLC24A1	1	0.907239	-0.22193	2.158066
SNORD18A	1	0.907303	0.120947	1.159495
BHLHE22	1	0.907322	-0.2595	1.926407
IMP4	1	0.907347	0.046781	5.154555
LIPT2	1	0.907396	-0.37199	1.181728
MPP5	1	0.907517	-0.24868	1.794461
LRFN4	1	0.907551	0.054443	4.627919
TAPT1	1	0.907597	0.047809	5.138691
TMEM260	1	0.907615	-0.07166	5.458175
LRRC18	1	0.90766	0.094151	2.177681
PARVB	1	0.9078	0.067185	2.499068
DKC1	1	0.907973	-0.24571	2.119139
MAGT1	1	0.907997	-0.06854	6.764796
ADAMTS1	1	0.908009	-0.0714	7.536482

POLB	1	0.908047	-0.08488	4.433762
ZMIZ2	1	0.908097	0.110627	1.812626
LDHA	1	0.908107	0.149709	1.081737
C22orf46	1	0.908196	-0.07317	6.834903
SPAG9	1	0.908236	0.083792	3.03917
PWWP2B	1	0.908436	-0.19768	2.558956
NALCN-AS1	1	0.908632	0.139941	0.749102
GOLGB1	1	0.90869	0.084413	1.408614
YPEL3	1	0.908744	-0.18461	2.71328
CEP44	1	0.908789	-0.08459	4.378132
PATZ1	1	0.908889	0.083992	2.959997
LOC101927	1	0.908955	0.07909	3.696419
GNG5	1	0.908991	0.061639	5.592087
BMP7-AS1	1	0.909026	0.091094	1.72868
DAD1	1	0.909091	0.046222	5.664263
CCNB1	1	0.909221	0.062951	5.03484
TMEM68	1	0.909387	0.100227	2.839301
ST13P4	1	0.909397	0.045523	4.705257
BHMT	1	0.909432	-0.33104	1.089533
CUEDC2	1	0.909601	0.054048	4.718376
MMP24	1	0.909624	-0.10032	4.402206
MEAK7	1	0.909711	0.051891	5.671935
EPM2AIP1	1	0.909806	0.048984	6.15071
MIR34AHG	1	0.909808	0.048086	6.175593
SLC27A4	1	0.909915	-0.08776	4.986918
ATPAF2	1	0.909992	-0.08492	4.411216
B3GNT9	1	0.910071	0.061696	4.010795
LOC101928	1	0.910093	0.11073	1.796268
CDCA4	1	0.910127	-0.33836	0.582981
LOC101928	1	0.910178	0.076135	1.405298
NOTCH2	1	0.910378	-0.06686	7.749927
CHCHD6	1	0.910449	0.075609	3.138121
USF3	1	0.910477	0.04163	7.008685
GGN	1	0.910519	-0.24828	1.370854
HNRNPA2B	1	0.910557	-0.30884	1.65596
RPS13	1	0.910636	0.043652	7.477395
ARF3	1	0.910722	0.042518	7.490785
LRP5	1	0.910767	0.064109	3.107962
FAM126B	1	0.910844	-0.05827	6.859576
PGP	1	0.911128	-0.11857	4.14859
EGFR-AS1	1	0.91115	-0.14421	3.209516
LOC100506	1	0.911211	-0.17158	4.134633
TNK2	1	0.911249	-0.11372	3.944524
GSDMB	1	0.911249	-0.30233	1.651584
PRR11	1	0.911692	0.068906	4.549744

SNORD66	1	0.911696	0.07273	2.003783
RASAL2	1	0.911732	-0.10809	4.261463
DPP9	1	0.911783	-0.062	6.877092
KLHL20	1	0.911822	0.042559	5.444989
SYNGR1	1	0.912011	-0.16754	3.170865
CLEC2B	1	0.912321	-0.10072	4.122646
SIRT7	1	0.912346	-0.07831	5.618359
SNORA26	1	0.912587	-0.07659	8.640221
MTMR8	1	0.912705	-0.15097	3.17505
SFTPA2	1	0.912809	0.047342	1.026442
ARL4D	1	0.912841	0.080175	2.698027
ZNF354B	1	0.912875	-0.08357	4.643815
MUS81	1	0.912945	0.045822	5.171072
RRNAD1	1	0.913051	-0.37461	1.136518
LOC10028ٗ	1	0.913148	0.089822	2.574009
EIF3L	1	0.913165	0.067153	2.048
VPS9D1	1	0.9132	-0.09589	4.00785
LINC00894	1	0.913258	0.042955	5.389113
CEACAM18	1	0.913329	-0.40243	1.905162
GTDC1	1	0.913451	-0.21183	2.245725
HDGF	1	0.913748	0.055233	3.984434
TMEM63A	1	0.913753	-0.06614	8.311252
RABGEF1	1	0.913939	0.052161	3.88172
TRIM44	1	0.913946	-0.06297	8.562268
ACADSB	1	0.913983	-0.07016	5.420399
UCKL1-AS1	1	0.914127	-0.0968	3.977402
LRP1	1	0.914175	0.038661	10.03284
PHKA2	1	0.914256	-0.07306	5.857021
LOC101928	1	0.914386	0.130818	1.558172
TFAP2A	1	0.914412	-0.17952	2.438081
CHSY3	1	0.914464	-0.18216	2.77878
SNRNP25	1	0.914622	-0.16503	3.414323
PYM1	1	0.914696	0.078914	1.027046
CDK17	1	0.914729	-0.22703	2.154283
TMEM75	1	0.914895	0.050587	4.564516
LPCAT3	1	0.915043	0.0426	5.91344
TMTC1	1	0.915157	-0.28008	0.993022
SART1	1	0.91517	0.039206	7.285986
HIBADH	1	0.915173	0.044693	5.759375
FOXN2	1	0.915231	-0.07327	5.835723
NMRAL2P	1	0.915252	-0.24969	2.447678
FRMPD3	1	0.915269	-0.22815	1.873074
ACAP2	1	0.915473	-0.05834	7.451762
BMS1P20	1	0.91561	-0.06541	5.989778
OR52B6	1	0.915754	-0.2439	1.541204

KLHL24	1	0.915956	0.038372	7.460558
RAPGEF3	1	0.915998	-0.36162	0.647244
LRRN4CL	1	0.916042	-0.13956	3.506359
ANKRD44	1	0.916064	0.055518	5.247809
SIPA1L1	1	0.916078	0.175667	0.778351
SAAL1	1	0.91623	0.055315	3.974247
DSTN	1	0.916278	-0.18457	1.899003
TEPSIN	1	0.916284	-0.07206	4.557809
CFAP53	1	0.916339	-0.21443	1.976385
PLUT	1	0.916496	0.060545	2.254796
WDR43	1	0.916582	-0.0639	7.327943
SERTAD4-A	1	0.9166	0.053485	1.438985
LOC100505	1	0.916621	-0.09868	4.257386
LOC100506	1	0.916705	-0.13615	2.918528
RNU12	1	0.91694	0.050095	8.075607
GPR18	1	0.91695	0.093657	1.844968
OCLN	1	0.917084	-0.24468	1.234005
RNF186	1	0.917101	-0.39865	2.56945
RAMP2	1	0.917312	0.08638	2.346678
SNORD11	1	0.91757	0.060047	4.6382
SRRM5	1	0.917625	-0.16894	3.046702
CCDC86	1	0.917657	-0.08279	4.558472
EIPR1	1	0.917718	0.041985	4.638164
PTPN4	1	0.91782	0.039548	6.291775
TAOK2	1	0.91787	-0.06918	4.910701
CEACAM1	1	0.917887	-0.41819	1.012239
CCNB3	1	0.918149	0.094401	1.195071
VWA3B	1	0.91821	0.046114	2.211297
ADPRM	1	0.9185	0.063995	3.370229
LRCH4	1	0.918607	-0.08869	3.757642
SIK3	1	0.918624	0.068071	2.405301
MROH2B	1	0.918716	-0.25875	2.671087
MON1A	1	0.918761	-0.3159	1.237881
ZER1	1	0.918801	0.038277	6.066538
TRMT11	1	0.918844	0.038927	5.626365
EIF3J-DT	1	0.918884	-0.30195	1.250063
PPIAP30	1	0.918945	-0.36317	0.943094
HIST1H2AH	1	0.919007	0.047496	4.455189
ZNF558	1	0.919012	0.065184	3.093593
GPAT2	1	0.919184	0.063139	2.728809
C6orf163	1	0.919387	0.065435	2.411336
YME1L1	1	0.919476	-0.18842	2.299877
XKR8	1	0.919493	-0.0947	3.661448
FAM83F	1	0.919578	-0.09811	4.813636
STAG3L5P	1	0.91972	0.068396	3.11475

WDR34	1	0.919786	0.041094	5.09616
NKX6-3	1	0.919872	-0.57163	0.820556
CFAP300	1	0.919884	-0.33283	1.28982
RAPGEF4	1	0.919966	0.061691	1.208006
POM121L1	1	0.920309	0.088223	1.221337
LAX1	1	0.920355	-0.25687	0.781699
FUNDC2	1	0.920478	-0.06613	5.228884
ZNF8	1	0.920525	0.044429	5.276063
SDK1	1	0.920549	0.046878	5.498545
C3orf67-AS	1	0.920564	-0.39451	0.764497
MSL1	1	0.920603	-0.05195	7.246181
PCBP1	1	0.920631	-0.05273	7.477356
RNPC3	1	0.920739	0.046429	5.700518
LINC00426	1	0.920741	0.066998	2.165367
RNF44	1	0.920994	0.036252	6.203527
NSD2	1	0.92101	0.046735	5.327713
SLC43A3	1	0.921135	-0.23266	1.739351
KPNA7	1	0.921154	0.052422	2.725516
L3MBTL2	1	0.921184	-0.06273	5.448435
ZNF133	1	0.921227	-0.23799	2.287475
FAF1	1	0.921394	0.034775	6.65311
STK10	1	0.921566	0.036749	6.690729
NRBP1	1	0.92165	-0.0539	7.344854
FAR2	1	0.921702	0.090537	1.631257
CACNA1C-A	1	0.921775	0.070962	2.869604
TPM3P9	1	0.921808	-0.07616	5.294426
SFT2D3	1	0.92186	-0.07976	4.372531
LIPC	1	0.921991	0.093716	1.430819
ZNF672	1	0.922001	0.041708	5.237984
OSBP	1	0.922093	-0.05871	7.914203
ITPRID2	1	0.922222	-0.07695	5.285213
YKT6	1	0.922259	-0.059	6.755066
DHX38	1	0.922361	-0.05272	7.542538
GOLGA7B	1	0.922575	-0.18488	3.11423
ZBTB3	1	0.922581	-0.11387	3.332783
GSTCD	1	0.922652	0.045877	1.089814
CDC42EP4	1	0.922754	-0.05745	6.284906
NPAS4	1	0.922761	0.10132	0.900542
SNORD14B	1	0.922779	-0.07562	5.558661
PPID	1	0.922787	-0.05843	6.063185
HEATR5B	1	0.9228	0.033195	6.826484
PDCD2L	1	0.922878	0.055338	3.49012
RPL35A	1	0.923041	0.092173	0.957652
RAVER1	1	0.923056	0.036128	6.158278
MCM3	1	0.923082	-0.24601	1.54086

MPP7	1	0.92336	-0.08572	5.354544
ZSWIM6	1	0.92337	-0.052	6.546184
ISCU	1	0.923451	-0.20939	2.268391
SPAG7	1	0.923454	0.035796	5.472311
EXD3	1	0.92353	-0.08125	4.318405
DOCK2	1	0.92358	0.038717	6.35425
SNORA3B	1	0.923644	0.037282	8.735033
ZNF775	1	0.923712	0.067113	2.692757
MED12	1	0.923783	0.032154	7.31165
KBTBD6	1	0.923789	-0.09688	4.097481
CCDC144CI	1	0.923896	0.06902	2.063366
SCD	1	0.923931	-0.10378	7.580441
PLP2	1	0.924108	-0.08306	5.922007
CCZ1	1	0.924111	-0.07491	5.609389
ICE2	1	0.924183	0.049055	4.302056
AP2B1	1	0.924229	0.100132	1.064285
TMEM120I	1	0.924266	-0.06322	5.368062
CKS2	1	0.924273	-0.10418	4.294574
MMP12	1	0.92429	0.0497	4.099453
CDK2AP1	1	0.924319	0.02052	1.013697
FAM151A	1	0.924321	-0.1151	2.943819
CLUHP3	1	0.924463	0.041862	4.897615
FAM66B	1	0.924498	-0.19779	1.346496
TRIM31-AS	1	0.924644	0.050799	2.87438
NUDT5	1	0.924678	-0.07419	4.767257
PRMT2	1	0.924697	-0.05779	6.307249
ZNF609	1	0.924731	0.032107	7.676859
ZNF781	1	0.925114	0.077657	2.461495
CCDC153	1	0.925224	0.037867	2.408325
ADCY7	1	0.925245	0.038145	5.782619
TECPR2	1	0.925337	0.052385	3.842654
NDUFAF4	1	0.925422	0.053633	3.374501
BRPF3	1	0.925451	0.03304	6.693236
EXOC3L1	1	0.925506	-0.25474	1.919817
LINC01091	1	0.925509	-0.27562	1.449332
TAB2	1	0.925597	-0.17279	1.919929
LOC100287	1	0.925733	-0.19431	1.617638
IL6ST	1	0.925742	-0.26469	1.756489
SENP2	1	0.925759	-0.05204	6.371509
NRG4	1	0.92613	0.053902	3.939566
UAP1L1	1	0.926144	-0.064	5.034191
VPS18	1	0.926149	0.042549	5.178695
PRKACA	1	0.926173	-0.14565	2.67946
CCND3	1	0.926682	-0.13508	2.523876
KLHL14	1	0.926817	-0.22581	1.376834

MIR3139	1	0.92687	0.073574	1.783204
TNPO1	1	0.926951	-0.33269	0.955806
NAV2-AS5	1	0.926984	-0.19298	2.947024
ZCCHC8	1	0.927345	-0.06045	5.705924
TMUB2	1	0.92742	0.077907	1.958738
NOTCH2NL	1	0.927453	-0.07263	4.560286
XPNPEP2	1	0.927478	-0.22728	2.0962
RNF144A	1	0.927555	0.037884	5.060361
THRA	1	0.927621	0.042627	4.401989
FTX	1	0.927633	0.039451	5.419906
DUS1L	1	0.927681	0.033043	6.162754
SH3TC1	1	0.927762	0.034715	5.636017
KCTD12	1	0.927834	0.036229	7.570585
BNIP3	1	0.927843	-0.06895	5.09011
ABHD16B	1	0.927977	-0.15421	2.687052
FAM98A	1	0.928031	-0.31814	1.024324
DBH-AS1	1	0.928166	0.007084	1.07858
KRTAP5-AS	1	0.928229	-0.26255	1.953199
MSMP	1	0.928402	0.049693	3.727343
ADGRL3	1	0.928474	0.045377	5.827661
ACP4	1	0.928568	-0.27608	1.777428
IL1RN	1	0.928573	-0.21669	3.833555
PROCA1	1	0.928621	0.121735	0.796218
LINC-PINT	1	0.928661	0.043764	4.939737
PNRC1	1	0.928686	-0.05143	7.744714
TMEM125	1	0.928735	0.033214	4.189955
LOC15077€	1	0.928843	-0.06126	5.363909
SLC47A1	1	0.928927	-0.20888	1.902904
ADAT3	1	0.929087	0.041438	2.252778
F8	1	0.929128	0.067477	2.262079
CTSD	1	0.929289	0.031065	8.339842
UXS1	1	0.929294	-0.24931	1.461539
MIR600HG	1	0.929335	0.035078	4.774704
MRPL40	1	0.929359	0.039991	4.254743
SPAG17	1	0.929366	-0.17648	3.16339
GSTA4	1	0.929723	-0.0942	4.149829
GRPEL2	1	0.929788	0.042678	5.092999
ZNF500	1	0.92985	-0.24079	1.197708
LOC10272З	1	0.929866	0.041704	1.05665
FAM83H-A	1	0.929916	-0.08605	4.46572
PARD6G-A!	1	0.929948	0.06878	1.902954
TRIP11	1	0.929951	0.030387	7.995545
WNT8B	1	0.93003	-0.23195	1.419453
STIMATE	1	0.930069	-0.0644	4.86297
LINC00607	1	0.930129	-0.22524	1.071573

SOS2	1	0.930237	-0.04801	6.987933
RARA-AS1	1	0.930286	0.066483	2.140819
F2RL1	1	0.930306	0.033435	6.168162
RABL6	1	0.930431	0.052082	3.729838
CYB5D1	1	0.930558	0.031959	6.127871
LINC01106	1	0.930811	0.036992	4.669919
FUBP3	1	0.930916	0.030599	6.956421
TNFRSF21	1	0.930979	0.030665	7.284302
MIATNB	1	0.931055	0.044818	3.729292
CAMK2G	1	0.931093	-0.33344	0.818519
CROCCP2	1	0.931144	-0.06896	5.549495
HMGA1	1	0.931219	0.045083	1.901952
SFN	1	0.931534	-0.10834	6.713704
HES1	1	0.931573	0.032543	6.53671
LINC00696	1	0.931718	0.064926	0.973884
MRPL44	1	0.931759	0.037818	4.654793
C10orf82	1	0.931808	0.01283	0.710337
FRMPD2B	1	0.931874	-0.37133	0.96203
KLHDC2	1	0.931898	-0.04954	5.765272
MRC2	1	0.931934	-0.05104	7.107897
SLC39A1	1	0.932075	-0.31315	0.969251
DLG4	1	0.9321	0.064147	1.448847
USP54	1	0.932107	-0.05979	7.664829
ACBD3	1	0.932122	0.028541	8.277034
MIR548V	1	0.932166	-0.16472	2.715109
CCDC142	1	0.932194	-0.07259	4.710955
SNAP47	1	0.932337	-0.07479	4.539541
SNORA64	1	0.93235	-0.05715	7.480115
NUP98	1	0.932481	-0.23926	1.35244
CERNA1	1	0.932627	-0.2494	1.425901
CALML3	1	0.932675	-0.34539	5.454299
ZC3HAV1	1	0.932874	-0.04688	6.862974
CRKL	1	0.932987	-0.04516	7.09584
CA9	1	0.933044	0.041169	5.029089
PCF11	1	0.933113	-0.04699	7.599976
MFSD13A	1	0.933259	0.04917	3.409008
GCHFR	1	0.933369	-0.14048	2.797446
PACRG-AS3	1	0.933389	-0.2681	0.894414
ATP6AP1L	1	0.933458	-0.08062	4.269014
LOC10013(	1	0.933533	0.040478	3.648156
TTF2	1	0.933658	0.032768	5.688846
NAGLU	1	0.933717	0.028465	4.763614
GLO1	1	0.933785	-0.05299	6.007694
RBM15-AS1	1	0.933794	-0.30799	0.670542
GPSM2	1	0.934041	-0.05309	5.648198

WTIP	1	0.934143	0.042598	3.649243
TRAF1	1	0.934219	-0.36281	0.69729
RBM26	1	0.934247	-0.35963	1.00644
CDKL5	1	0.934256	-0.35359	1.17657
LOC399715	1	0.934448	-0.12178	3.312198
GEMIN8P4	1	0.934608	0.011762	0.980011
NDUFS6	1	0.934612	0.029216	5.418132
PPP1R14C	1	0.934615	0.042925	3.515743
SCARB2	1	0.934645	-0.06415	4.695398
FLJ37035	1	0.934673	0.034642	1.653288
DDX6	1	0.934813	-0.23354	1.473497
VRK2	1	0.934868	-0.22044	1.714947
HECTD1	1	0.93499	-0.04631	9.090098
UTRN	1	0.934996	-0.05173	9.664043
LAMP1	1	0.935048	-0.04979	7.949589
IMPACT	1	0.935107	0.034039	6.31113
GPLD1	1	0.935143	0.049337	3.314241
BUD13	1	0.935358	-0.18215	2.595069
MORC3	1	0.93539	0.030034	6.677592
NTRK3	1	0.935407	0.017869	1.952037
RNF24	1	0.935411	0.020508	1.542726
LOC101928	1	0.93544	0.041414	1.298258
WSCD1	1	0.93554	-0.13386	2.78942
MAFB	1	0.935541	0.030585	5.855898
STX6	1	0.935659	-0.20371	1.440256
SSU72	1	0.935794	0.026702	6.387945
RNF220	1	0.936015	-0.04653	6.073341
TTC39C-AS	1	0.936033	-0.23106	1.868127
SCARNA1	1	0.936101	-0.06354	5.164237
LRP6	1	0.936102	0.025842	7.844822
COL1A2	1	0.936131	-0.04929	11.05275
HIF1AN	1	0.936258	0.026542	7.067199
AK3	1	0.93628	0.026815	2.262658
ZNF81	1	0.936321	-0.05425	5.298176
C1QA	1	0.936359	-0.05618	5.822167
BRSK1	1	0.936404	0.032662	3.838082
AOAH-IT1	1	0.936471	-0.19334	2.29623
INPP4A	1	0.93648	0.058389	2.625461
SYCE1L	1	0.936541	0.052394	1.449574
TTC28	1	0.936573	0.031152	6.619952
RBM43	1	0.936594	-0.08035	4.584053
OR10A4	1	0.936646	0.085131	0.687352
GPS2	1	0.9368	-0.04655	6.611352
FANCC	1	0.936934	0.042219	3.467229
LOC100506	1	0.936957	0.013603	1.036706

CLEC19A	1	0.937008	0.038094	1.315555
FNDC8	1	0.937081	0.051985	1.390407
ALS2CL	1	0.937124	-0.19701	2.529591
CUEDC1	1	0.937189	0.085676	1.458529
LINC01303	1	0.93731	0.054261	2.653224
DCHS1	1	0.9374	-0.0543	6.321501
LACTB	1	0.937538	0.050191	3.104416
SH3PXD2A-	1	0.937578	-0.15069	3.608381
UBA1	1	0.93762	0.036079	3.39616
ECHDC2	1	0.937626	-0.22141	1.553774
SELENOI	1	0.93764	-0.04944	6.909481
SLC9A7P1	1	0.937737	0.046958	1.995356
MIR4676	1	0.937756	-0.29749	1.914119
GRIPAP1	1	0.937877	-0.04427	7.089845
SCN7A	1	0.937908	0.050149	1.347781
TMEM50B	1	0.938038	-0.22494	1.61466
C8orf76	1	0.938277	-0.18056	1.763143
MED9	1	0.938291	0.033789	4.125255
LDHAL6B	1	0.93833	-0.17677	2.418541
YPEL4	1	0.938334	-0.20243	2.689374
RNASEH2C	1	0.938423	-0.06983	4.538401
HERC2P2	1	0.938507	-0.0491	7.229789
MACROD2	1	0.938537	-0.15194	2.400898
TBX5-AS1	1	0.938573	0.052059	2.592172
BRF1	1	0.938759	-0.15363	2.566875
DNAH12	1	0.93877	-0.18452	2.540616
SPATS2L	1	0.938953	-0.21427	1.269819
MBD6	1	0.939062	0.025047	7.177043
MYLK-AS2	1	0.939087	0.050556	2.111055
LOC100507	1	0.939259	0.014427	1.228842
KBTBD8	1	0.939365	0.045284	3.590872
CD93	1	0.939388	-0.05907	7.136339
SNORA6	1	0.939532	-0.05171	8.100603
COL6A6	1	0.939594	0.02677	2.803593
GGT7	1	0.939596	-0.07209	4.196814
PDCD1LG2	1	0.939604	0.023553	2.136189
ZNF506	1	0.939699	0.041251	2.445776
DSG2-AS1	1	0.939834	-0.19586	1.987454
SVBP	1	0.939945	-0.14615	2.772478
FLJ46906	1	0.940025	0.044935	2.473676
ABRAXAS1	1	0.940074	0.040545	3.866583
TJAP1	1	0.940155	-0.3311	0.926295
FAM81B	1	0.940156	0.042118	1.430178
SNORD50A	1	0.940181	0.031436	4.000448
NUCB1	1	0.940306	0.023853	7.548183

FARP1	1	0.940321	-0.14977	2.421643
TRIM8	1	0.940379	-0.04409	7.355488
PKD1P1	1	0.940404	-0.04339	7.366492
TMEM158	1	0.940409	0.03164	3.309501
DICER1-AS1	1	0.940511	-0.2091	1.343455
GTF2H4	1	0.940604	-0.17788	0.804201
ZNF547	1	0.940656	-0.18096	2.373749
WNT9A	1	0.940744	0.065183	0.873747
TXNDC15	1	0.940854	-0.04807	6.03016
ZNF695	1	0.941159	0.035984	2.711662
KCTD1	1	0.94117	-0.08226	4.192501
HHIPL1	1	0.941188	0.051098	2.144554
ZDHHC9	1	0.941245	0.03583	1.091869
NOC2L	1	0.941405	-0.05182	6.211767
UBA6-AS1	1	0.94167	0.036522	3.297044
PACRGL	1	0.941755	0.050276	0.741396
LMAN1	1	0.94183	0.025848	8.23837
ZNF599	1	0.941843	0.064169	1.775299
PYROXD1	1	0.941919	-0.05827	4.945705
RRP36	1	0.942119	0.023622	5.18833
CROCC	1	0.942243	-0.05462	6.272788
MPEG1	1	0.942311	0.029379	5.97708
PNMA1	1	0.942488	-0.05533	5.329282
POM121	1	0.94249	-0.05666	5.013395
GATA2-AS1	1	0.942574	0.019993	3.044621
ZNF75D	1	0.942584	-0.09889	4.363827
ESPL1	1	0.942632	-0.11246	4.276091
ACAD10	1	0.942751	-0.22479	1.452949
RPL37A	1	0.943132	-0.04531	8.324577
KIF14	1	0.943242	-0.22379	1.485229
RAB19	1	0.943351	-0.13745	1.924792
SERPINE2	1	0.943389	0.015435	1.335294
RPS6KA6	1	0.943396	0.038494	2.336804
TACC1	1	0.943446	0.045977	2.717088
KRT14	1	0.943582	-0.30189	7.848657
HSPA8	1	0.943786	-0.04666	8.500193
MCRS1	1	0.94383	0.045891	1.449315
PLPP5	1	0.943849	-0.05394	4.934672
MRPS30	1	0.943901	-0.05924	5.237109
CNOT9	1	0.943998	0.060929	0.843401
UBE2D3	1	0.944122	0.023538	5.293186
TRAPPC8	1	0.944153	-0.04246	7.128029
C2orf69	1	0.944165	0.02873	4.822346
GUF1	1	0.944408	0.022677	5.9889
ZNF691	1	0.944464	0.038591	1.740571

TGFBR2	1	0.944557	-0.16028	1.87686
COL6A4P2	1	0.944591	-0.19757	2.218109
HNRNPA1F	1	0.94465	0.022484	8.239672
PRKAR2A	1	0.94471	0.021775	6.16705
LRRCC1	1	0.944853	-0.04641	5.258585
ZNF428	1	0.945294	-0.08227	3.089235
POLI	1	0.945519	0.028806	5.171678
RP1L1	1	0.945596	0.026725	2.419799
ACSM5	1	0.945672	-0.34327	0.822875
TAF6L	1	0.945776	-0.08063	4.133221
ZNF618	1	0.945839	0.023355	6.503893
UBTD1	1	0.9459	0.042374	3.22849
CYP7B1	1	0.945912	0.033623	3.617404
TMEM94	1	0.945947	0.021653	6.45447
CNKSR3	1	0.946163	-0.05339	5.971199
TTC37	1	0.946168	-0.03673	7.323261
HBA1	1	0.946238	-0.20844	1.873207
PLPP2	1	0.946255	-0.2226	2.200163
CCSER2	1	0.946458	-0.23619	1.590629
KMT5B	1	0.946553	0.026704	2.077975
RNF139-AS	1	0.94656	-0.11455	2.852515
BMP2K	1	0.946582	-0.05697	4.752909
LOC101929	1	0.946654	0.006594	2.089253
SNORD53	1	0.946657	-0.18307	2.217534
HDHD2	1	0.946813	-0.05107	5.290152
STARD9	1	0.946829	0.025049	6.992068
HSD3BP4	1	0.946864	-0.08296	4.213562
LOC102723	1	0.946919	-0.1913	2.740608
C19orf67	1	0.94699	-0.28883	1.223663
TCF25	1	0.947036	-0.04019	7.807748
TXN	1	0.947115	-0.31859	0.673588
C11orf91	1	0.947255	-0.00708	1.756897
FANCE	1	0.947356	-0.13074	3.247847
CD180	1	0.947624	0.023092	2.456023
SLC35E3	1	0.947674	-0.06995	4.352305
TGFB1	1	0.947709	-0.05231	6.253018
N4BP2	1	0.948409	0.021438	6.37471
LSAMP-AS1	1	0.948467	-0.3218	1.356635
SLCO1B1	1	0.9485	-0.36196	1.488558
ITGA9	1	0.948526	-0.0566	5.586181
BIK	1	0.94856	-0.07734	4.279736
USO1	1	0.948602	-0.09464	3.080857
CXCR6	1	0.948603	-0.11061	3.855621
ZNF593	1	0.948616	0.029738	3.438456
EMX1	1	0.948655	-0.00501	1.330638

MAK	1	0.948798	-0.16159	2.697034
TRAM2-AS1	1	0.949146	0.022369	1.18247
ALG1L2	1	0.949174	-0.01071	1.128245
POU5F1P3	1	0.949218	-0.22436	1.201007
SNX32	1	0.949262	0.040593	2.128645
EBLN3P	1	0.949336	-0.03994	7.363657
PRO1804	1	0.949381	-0.15385	1.945756
ADRB2	1	0.949405	0.037593	2.817689
RARS	1	0.949408	0.019547	6.173906
SOD1	1	0.949527	-0.03939	7.303985
HIST1H3G	1	0.949547	-0.07195	4.423306
NTN4	1	0.949643	0.023079	6.028585
ZDHHC2	1	0.949697	-0.06566	5.274777
DHDH	1	0.949962	0.048469	1.403711
LOC105375	1	0.950068	0.026	2.444965
DBT	1	0.950084	0.023529	5.932555
LNX2	1	0.950111	0.020295	6.006255
MYPOP	1	0.950111	-0.08174	3.888547
LINC00324	1	0.950273	-0.16939	2.076289
IGBP1P1	1	0.950335	0.024895	1.144591
SH3RF1	1	0.950371	-0.0425	6.920015
RNPEPL1	1	0.950406	0.018236	6.276584
DGKI	1	0.950452	-0.11446	3.054159
SDK2	1	0.950481	0.020366	4.191216
BANP	1	0.950486	0.023572	0.826676
KIF13B	1	0.950877	0.018169	8.809675
EIF4G2	1	0.951056	-0.07874	3.225908
TDRP	1	0.951066	0.033767	0.889762
SNTA1	1	0.951269	-0.06342	4.041172
LOC100507	1	0.951402	-0.23388	1.608684
RNASE6	1	0.951454	-0.09255	3.15562
SMYD5	1	0.951491	-0.05892	4.673431
COL18A1-A	1	0.951498	-0.11922	2.953678
ZNF208	1	0.951783	0.027319	4.27566
LINC01535	1	0.951802	-0.15973	1.148069
NDUFA1	1	0.951862	0.021264	5.595431
SRSF6	1	0.951899	0.030007	4.107506
DPAGT1	1	0.952002	-0.04805	4.888942
SNAPC5	1	0.952283	-0.06267	3.997464
GALNT11	1	0.952308	0.022415	2.798076
RALGDS	1	0.952346	0.015886	2.902886
SNORD113	1	0.952368	-0.31285	1.523654
BMP8B	1	0.952533	0.025563	3.378626
SNORA41	1	0.952645	0.026187	2.941721
UVRAG	1	0.952696	-0.0388	6.212653

CAND1.11	1	0.952703	-0.24894	1.610282
HSPA4	1	0.952812	0.018158	7.599388
ZFP82	1	0.952892	-0.22553	2.083882
LOC729867	1	0.95293	-0.11938	1.736934
SNRPA1	1	0.953011	0.015304	5.173125
TPRG1	1	0.9531	0.00844	3.541776
SPATA33	1	0.953108	0.040607	1.961383
KPNA6	1	0.953626	0.017527	7.687268
GTF2E2	1	0.953713	-0.04184	6.119166
FBXO38	1	0.953722	0.028023	3.105422
MEA1	1	0.953834	-0.03749	5.900044
LOC285819	1	0.953991	-0.21102	1.213512
EEA1	1	0.954235	-0.03425	7.670004
LRRC37A2	1	0.954441	0.024103	3.116304
PDGFRA	1	0.954455	0.018249	7.961897
HK1	1	0.954501	-0.14839	1.96545
GCKR	1	0.954504	0.032964	3.338751
YAE1	1	0.954593	-0.11196	2.64819
GNAI3	1	0.95474	-0.04264	7.006621
ZNF248	1	0.954773	-0.01019	1.969901
SLITRK6	1	0.954778	0.01166	3.738323
SLC9A1	1	0.954938	0.015722	2.457529
RRN3P1	1	0.954959	-0.05273	4.554936
LOC101928	1	0.955121	-0.15597	2.066656
MED13L	1	0.955126	0.015766	8.585441
BCL2L1	1	0.955135	-0.19505	1.357278
PDE7A	1	0.955135	-0.00353	1.296171
LONRF3	1	0.955161	-0.32554	1.021774
GJB5	1	0.955217	0.02198	2.062647
ZC3H12B	1	0.955404	0.026344	4.132185
KCNC3	1	0.955487	-0.06247	4.531038
CELF2	1	0.955542	-0.09747	3.382998
ZXDA	1	0.955984	0.027068	4.359802
SLX4IP	1	0.956003	-0.06842	4.201807
CDC42BPB	1	0.956003	-0.03227	8.631643
SBNO2	1	0.956063	0.018283	2.241889
HARBI1	1	0.956226	0.022507	2.98899
PRRC2B	1	0.95626	0.014953	9.072622
SF3B3	1	0.956268	-0.03116	7.58386
ZNF510	1	0.956305	-0.3063	1.099364
DUSP16	1	0.956377	-0.03386	7.10468
SIRT1	1	0.956448	0.012303	3.758508
SYNJ2	1	0.956629	0.00202	1.1618
MYBPC3	1	0.956751	-0.08227	2.863239
SFXN2	1	0.956773	0.012598	4.547586

TMEM232	1	0.956866	-0.18122	1.960412
APOOL	1	0.956977	0.019566	5.729439
ZNF10	1	0.957012	-0.04831	4.84411
SIN3B	1	0.957019	0.018195	2.777635
NMUR1	1	0.957143	-0.27306	0.964627
ODAM	1	0.957148	-0.15254	2.596383
PRAG1	1	0.957192	0.014378	5.922215
MUT	1	0.957246	-0.0335	6.145467
CDC23	1	0.957488	-0.05871	4.983178
KLK11	1	0.95772	0.025697	1.151696
LOC10192¹	1	0.957744	-0.16782	1.315172
TRIM23	1	0.957774	-0.16145	2.210192
RUNX3	1	0.957805	-0.15014	1.870309
MOK	1	0.957807	0.021818	2.633278
GPBP1L1	1	0.957867	0.014377	7.867922
ELMSAN1	1	0.957968	-0.13262	2.708788
MT1L	1	0.958061	-0.18403	1.61427
PICALM	1	0.958074	0.031548	1.631715
ITPKB-IT1	1	0.958147	0.014299	2.751449
PLA2G5	1	0.958216	-0.178	1.851196
TBC1D8B	1	0.958248	-0.04702	5.04251
CTNNA1	1	0.958251	-0.15265	1.937812
SRRM4	1	0.958365	-0.3043	1.386316
DAGLB	1	0.958556	0.025201	1.73473
KLF15	1	0.958558	-0.09756	4.051592
NBR2	1	0.958609	-0.04915	4.862678
PRKD1	1	0.958743	-0.06617	4.07035
CCDC126	1	0.958786	-0.06862	3.849205
NOP10	1	0.958879	-0.0446	5.055125
INTS11	1	0.9589	-0.30202	1.444507
ZNF554	1	0.958914	0.020204	3.788713
SNORD97	1	0.959074	0.014207	9.951726
PSMD6-AS	1	0.959146	-0.0531	4.770251
RPL13AP6	1	0.959172	0.015572	1.919083
HOXD4	1	0.959253	-0.21188	1.286203
RBM8A	1	0.959318	-0.03406	6.999864
EFCAB7	1	0.959434	0.013275	3.302758
TRAP1	1	0.95956	-0.08959	2.638373
SMYD4	1	0.959608	-0.04759	4.902734
RAD51D	1	0.959723	0.020113	2.864778
DEFB131B	1	0.959792	-0.18082	1.207134
MINDY4	1	0.959918	0.036997	0.695886
HSP90B1	1	0.960005	-0.03381	10.3641
MIR548L	1	0.960076	-0.24234	1.070195
CDC25B	1	0.960092	-0.20251	1.090465

ZBTB32	1	0.960684	0.01041	2.652498
CPSF2	1	0.960717	0.013666	6.926516
SOWAHC	1	0.960758	-0.06229	6.121717
MTLN	1	0.960925	0.013896	2.174459
LRRC41	1	0.960951	-0.03711	6.204151
DNASE1	1	0.961	-0.04711	4.45976
C2orf76	1	0.961006	-6.76E-05	2.389007
TEX261	1	0.961122	-0.03111	6.384316
NR4A1	1	0.961251	-0.07309	4.083091
PART1	1	0.961267	-0.14195	2.301123
CC2D2A	1	0.961346	-0.03331	6.030696
LRRC75B	1	0.961427	-0.14218	2.265183
SMARCD2	1	0.961511	-0.03234	6.737414
LSM14B	1	0.961522	-0.03817	5.741261
RTL5	1	0.96153	0.019636	4.591023
MGAT5	1	0.961764	-0.03105	8.037447
GUSBP4	1	0.961822	0.015583	5.368821
MINOS1P1	1	0.961892	0.015515	3.986852
HYKK	1	0.962015	0.042362	0.685691
GPX3	1	0.962084	-0.03932	6.641168
NR2C1	1	0.962311	0.020615	3.882302
TBC1D22A-	1	0.962343	0.00528	4.574069
NEDD4	1	0.962462	-0.13902	2.593207
LINC01679	1	0.96253	0.045446	1.327821
KLHL41	1	0.96259	-0.10982	2.363463
ING5	1	0.96261	0.009082	5.516056
NR2F2-AS1	1	0.962691	-0.11416	2.815533
SNORD45C	1	0.962825	-0.047	7.031898
AGAP5	1	0.963132	0.00073	2.964972
LIN7A	1	0.963365	-0.15535	1.260117
IKZF4	1	0.963539	-0.05954	4.37266
LINC00565	1	0.96374	-0.2235	0.89378
DBP	1	0.963894	-0.06834	3.674984
RFX3	1	0.96398	0.011413	2.402798
LSM8	1	0.963981	0.012952	6.348535
SAMHD1	1	0.964091	0.011732	7.449288
AIFM1	1	0.964225	-0.29583	0.649293
WIPF2	1	0.96445	-0.02795	6.981315
SOX18	1	0.964454	0.021053	2.209578
TMEM131	1	0.964665	-0.02697	7.914974
NUDCD2	1	0.964666	0.020113	3.032411
NABP2	1	0.964846	-0.05244	4.396912
PPM1M	1	0.96502	0.038077	1.304004
SETD1A	1	0.965288	-0.03145	6.748071
SLC44A1	1	0.965563	-0.04439	4.931114

ZNF343	1	0.965629	-0.19237	1.862091
FAM86DP	1	0.965753	-0.04127	4.420867
ATP6V0E2-	1	0.965823	-0.18149	0.620939
NUTM2B-A	1	0.966041	-0.03473	6.157009
EIF3I	1	0.96613	-0.03591	6.570744
PFN4	1	0.96615	-0.1847	1.415182
SCARNA4	1	0.966346	0.006413	9.007156
MRPL23	1	0.966481	0.006464	3.704447
PTMAP11	1	0.966525	-0.03979	4.729165
MTUS1	1	0.966594	0.013209	3.933101
BRWD1-AS	1	0.966764	-0.20854	1.197429
TMEM102	1	0.966782	-0.06771	3.391848
PTPN9	1	0.966838	0.009842	6.110878
ERO1B	1	0.966873	-0.04202	5.192736
SNORA30	1	0.966877	-0.13995	2.299188
PABPC4-AS	1	0.966934	-0.0901	2.725813
FLJ37453	1	0.967014	-0.0935	3.393586
YIF1A	1	0.967068	-0.26495	1.33155
C6orf226	1	0.967274	-0.15275	1.764271
IL2RG	1	0.967416	-0.04245	5.30422
STOX2	1	0.967459	0.010718	3.15068
ADAM20	1	0.967484	-0.12931	1.979851
SH3BGRL3	1	0.967502	0.009316	7.263882
TBC1D13	1	0.967626	-0.18515	1.478604
CAPN1	1	0.967645	-0.17152	1.862772
SEMA4D	1	0.968131	0.011166	5.875598
SNORD92	1	0.968163	0.002367	3.362038
DTX3	1	0.968192	-0.19425	1.144107
FAM192A	1	0.968251	0.009379	6.997823
SIX5	1	0.968379	0.011767	4.560224
CASQ1	1	0.968489	-0.12214	1.576127
NFATC3	1	0.968593	-0.17858	0.898039
CCDC93	1	0.968684	-0.0269	7.378007
UBR3	1	0.968772	0.009207	7.475475
KIF1BP	1	0.968813	0.010772	5.671072
ATP5F1C	1	0.969001	-0.26818	1.289786
GYS2	1	0.969004	-0.24181	1.538739
LOC101926	1	0.96909	-0.00459	3.600425
SLC41A1	1	0.969288	-0.02868	5.970257
HIST1H2AN	1	0.969389	-0.07229	4.620945
NUDT21	1	0.969428	-0.0276	7.133223
LINC00884	1	0.969454	0.041646	0.9591
KIAA1217	1	0.969464	0.008366	6.987583
LDB2	1	0.969507	-0.14323	2.132048
CHD2	1	0.96951	-0.02501	9.042554

SCARNA18	1	0.969703	0.023622	2.539594
TTC23	1	0.969823	-0.00064	2.45518
EIF3F	1	0.96986	0.008377	6.158695
CADM4	1	0.969889	0.005633	4.101899
EGFR	1	0.96997	0.007549	7.309318
WNK1	1	0.970073	0.008322	3.579684
POLRMT	1	0.970119	-0.03423	6.062966
LZTR1	1	0.970217	0.007165	6.142616
TFF2	1	0.970263	-0.0905	7.388849
ACIN1	1	0.97028	-0.05796	3.711622
FBXL4	1	0.97033	-0.07769	3.372078
VHL	1	0.970361	-0.17242	1.744618
BRWD1	1	0.970433	-0.02595	7.193221
RAB39B	1	0.970435	-0.16195	1.92781
IFT172	1	0.970457	-0.03036	6.272158
S1PR1	1	0.970484	-0.04831	5.161272
DPYD	1	0.970575	0.0077	5.694462
SLC4A2	1	0.970764	-0.00147	2.489169
EFR3B	1	0.970911	-0.07521	3.192577
FPGS	1	0.970947	-0.13066	1.947768
GAU1	1	0.97116	-0.14004	1.983442
ADPRHL2	1	0.971319	0.004298	4.297986
NAV2-AS4	1	0.97146	-0.13072	2.585428
MEIS1-AS2	1	0.971629	-0.09208	3.100462
PAPPA2	1	0.971717	-0.23638	0.817127
TMEM35B	1	0.971798	0.007977	3.69596
THAP6	1	0.971876	-0.1777	1.613877
LHFPL4	1	0.972079	-0.17384	1.443533
SLC16A13	1	0.972198	-0.09892	2.358333
HNMT	1	0.972344	0.007017	4.73742
AVIL	1	0.972363	-0.04493	4.293767
PCDHB11	1	0.972824	-0.12155	2.662712
ARHGEF4	1	0.972881	-0.10626	2.820575
CCDC152	1	0.972955	-0.10566	2.254765
DCP1B	1	0.973096	0.006412	4.343028
C16orf54	1	0.97315	-0.09833	2.983465
P3H2	1	0.973264	-0.18308	1.4897
MIR5687	1	0.9734	-0.20813	1.156688
LGALS2	1	0.973451	-0.06776	3.400662
VTI1A	1	0.9737	-0.02441	6.62866
ZNF135	1	0.973714	-0.17277	2.002621
AHI1	1	0.973774	-0.08578	2.362668
CAPZA2	1	0.973857	-0.02789	7.151007
HM13	1	0.973889	0.006473	4.801902
ZHX3	1	0.973907	-0.03029	6.985316

GTF3C5	1	0.973996	-0.24702	0.901606
CCDC183-A	1	0.974076	0.010649	2.990559
OPN3	1	0.974122	0.015069	2.76466
YBX3	1	0.974147	-0.04992	3.510007
DIRC3	1	0.974305	-0.10355	2.486535
CXorf40A	1	0.974439	-0.16756	0.760833
MFN2	1	0.974466	0.007114	2.898139
PRKCE	1	0.974658	0.004746	5.124746
SLC4A11	1	0.974691	-0.10718	3.122145
AGBL3	1	0.974733	-0.08199	2.553331
MLH1	1	0.974811	-0.19482	1.058721
XYLB	1	0.974825	0.003376	5.514276
ABHD6	1	0.974944	-0.05078	3.932233
MRPL34	1	0.974998	-0.05445	3.710291
S100A1	1	0.975132	-0.1364	2.209127
VPS29	1	0.975342	-0.16591	2.368934
ALYREF	1	0.975349	-0.0315	5.164325
ZNF252P-A	1	0.97546	-0.00707	3.233006
SNHG4	1	0.975532	-0.0927	2.889582
MSH4	1	0.975958	-0.13186	2.983897
KIAA1109	1	0.976214	0.004602	8.976319
RPL27	1	0.976323	0.003944	8.192411
RPPH1	1	0.976345	-0.02303	12.67537
ACP6	1	0.976434	-0.03816	5.238921
RBL2	1	0.976529	0.005189	7.565539
GADD45GI	1	0.976659	0.001204	5.30479
TFAP2B	1	0.976903	-0.22126	1.289452
ZNF528	1	0.97718	-0.00015	4.568243
PDK2	1	0.977298	-0.15059	2.054846
SYPL2	1	0.977501	-0.14737	1.88
LRRC4	1	0.977555	-0.03958	4.869067
SGPL1	1	0.977572	-0.03016	7.561466
MTOR	1	0.977826	-0.02008	7.441339
POLR3GL	1	0.978035	0.004084	6.262465
ANKRD49	1	0.978365	-0.03966	4.851186
TMEM176A	1	0.978581	-0.04409	5.160597
ACAA1	1	0.978781	-0.17611	0.866209
RNF43	1	0.978856	-0.09452	3.655571
TMEM254	1	0.978858	-0.0994	2.267177
GOLGA1	1	0.97922	-0.02209	6.347964
RIMKLA	1	0.979257	0.006758	3.860593
RAB8B	1	0.979565	-0.02637	6.272763
FAM160B2	1	0.979749	0.000596	6.253199
BICD1	1	0.979757	-0.15717	1.29452
TNPO2	1	0.979886	-0.10072	2.368273

PDIA2	1	0.979951	-0.08936	3.290362
FGD3	1	0.980025	-0.17051	1.21498
SYS1	1	0.980226	-0.05424	3.156572
IFNAR1	1	0.980239	0.003633	6.797207
CDC40	1	0.980423	-0.02507	6.077627
DCPS	1	0.980817	0.005928	3.883207
KIAA0895L	1	0.980916	-0.027	5.651
KMT2C	1	0.981007	-0.01837	9.686144
SNORA2B	1	0.981044	-0.07693	2.841047
GABRA3	1	0.98108	-0.31989	0.972569
ELAC1	1	0.981117	-0.0489	3.025199
LINC02532	1	0.981376	-0.1524	2.094068
DNAJC8	1	0.981431	-0.01996	6.461085
MFSD1	1	0.981535	-0.21955	1.282943
OGFR	1	0.98158	-0.02259	6.179393
RIC8B	1	0.981589	-0.02962	4.88989
SVIL-AS1	1	0.981698	-0.12308	1.887344
AIM2	1	0.981848	-0.15335	1.898978
BTBD10	1	0.981948	-0.07022	3.141243
BDP1	1	0.982007	-0.0337	5.43475
RPS6KA2-IT	1	0.982222	-0.13042	2.487985
ZNF654	1	0.982276	0.003744	5.680183
NQO2	1	0.982484	-0.17191	0.954923
NEK8	1	0.982599	-0.06932	3.488718
AP3D1	1	0.982679	-0.13539	2.583978
PUS3	1	0.982717	-0.19088	1.603804
UBQLN2	1	0.982735	-0.02358	5.607861
RNF187	1	0.982917	-0.02344	5.928188
SNORD41	1	0.983062	-0.03693	6.208054
LOC101927	1	0.983183	-0.10571	2.595129
FBXL3	1	0.983593	0.00132	6.60587
DNAJB6	1	0.983731	-0.02512	5.630731
ZNF69	1	0.983986	-0.06941	3.596391
SF3B2	1	0.984011	-0.01799	8.777862
MANEA	1	0.984042	-0.02577	5.279148
CCDC144B	1	0.984335	-0.02949	6.868805
SVIL	1	0.984345	-0.00311	6.540761
CYR61	1	0.984823	-0.00288	6.812515
FRK	1	0.984952	-0.00139	6.342393
AMFR	1	0.985283	-0.00087	6.542057
FZD10	1	0.98549	-0.12864	2.23862
SPON2	1	0.985783	-0.06007	3.295064
AHSA2P	1	0.985915	-0.00157	7.065579
ESAM	1	0.986006	-0.03077	3.779505
TYW1	1	0.986126	-0.00387	6.349609

ZFYVE16	1	0.98627	-0.1238	2.158757
B4GALNT3	1	0.986511	-0.03491	7.045028
XPNPEP3	1	0.98659	-0.02121	6.116549
WFDC21P	1	0.986609	-0.15369	3.101387
SYP	1	0.986699	-0.06683	2.582339
SSTR1	1	0.986705	-0.06408	4.790591
EDC4	1	0.98712	-0.01737	6.385055
C1RL	1	0.987247	-0.04405	3.526545
LINC01979	1	0.987258	-0.06719	2.907044
EMBP1	1	0.987933	-0.039	3.568047
LOC100507	1	0.987971	-0.15247	1.176248
POU5F2	1	0.988161	-0.06613	2.966569
CISD2	1	0.988273	-0.05338	3.82134
TRIM28	1	0.988301	-0.00296	7.405117
STAG3L2	1	0.988315	-0.02629	4.988582
SSB	1	0.988335	-0.22558	1.103155
PKN1	1	0.988346	-0.14214	0.855311
NCF1	1	0.988348	-0.08831	2.315068
ATP6V0E1	1	0.98836	-0.02199	5.910577
HMGN3	1	0.988566	-0.23372	0.877497
MAPK13	1	0.988734	-0.21152	1.12397
LINC01359	1	0.989074	-0.07605	3.234618
TFEB	1	0.98913	-0.12909	0.935235
INTS3	1	0.989145	-0.04064	4.052868
SNORA61	1	0.989228	-0.01067	7.335095
MAPK1IP1I	1	0.989251	-0.01436	7.394804
PIP5K1B	1	0.989392	-0.17322	1.083002
WASL	1	0.989435	-0.01502	7.588974
BOP1	1	0.989446	-0.00664	5.953218
MIR5047	1	0.989456	-0.01911	5.545266
GCNT3	1	0.989735	-0.04426	7.270607
KLHL3	1	0.990053	-0.08413	2.262012
TMEM72-A	1	0.990072	-0.13683	0.886822
ACTG1P4	1	0.990145	-0.20736	1.133483
RCL1	1	0.990157	-0.10811	2.264389
FARS2	1	0.990492	-0.02264	4.379874
BEST4	1	0.990691	-0.11773	1.716585
DNAJA2	1	0.990858	-0.0027	7.082837
SLPI	1	0.990862	-0.01971	6.64033
ST7-AS1	1	0.990879	-0.13966	1.405242
GINM1	1	0.990934	-0.01583	5.535318
BLK	1	0.991188	-0.09057	2.585917
ARL11	1	0.991284	-0.0886	2.785365
DDX50	1	0.991432	-0.0053	5.941301
VPS37B	1	0.991872	-0.01823	6.896911

LINC00672	1	0.992007	-0.08448	2.717199
CNTRL	1	0.992387	-0.01534	7.192666
HHIP-AS1	1	0.992553	-0.26266	0.993828
LOC10028ɛ	1	0.992565	-0.08328	1.391931
DPH6	1	0.992582	-0.04047	3.160622
CCDC159	1	0.992608	-0.02588	4.904271
IL1R2	1	0.992784	-0.1685	1.760144
RPS16	1	0.992788	-0.00642	7.819578
MTA2	1	0.992799	-0.00654	7.253475
CD81-AS1	1	0.992807	-0.13759	1.25791
PDS5A	1	0.992816	-0.01429	7.641534
LINC00174	1	0.992833	-0.02296	4.913699
MIS18BP1	1	0.992864	-0.01406	6.661067
ZNF737	1	0.992915	-0.02462	4.598209
HNRNPA3	1	0.992972	-0.0128	8.874954
CCDC149	1	0.993035	-0.14956	1.303292
LAMC1	1	0.993061	-0.0143	8.985594
FASN	1	0.993543	-0.00826	7.723397
FOXD2-AS1	1	0.993547	-0.09744	2.652626
LRRC7	1	0.993705	-0.30024	0.915496
BCCIP	1	0.993732	-0.02177	4.259717
AAK1	1	0.9938	-0.00525	8.073822
SNORD65	1	0.993879	-0.08872	2.369178
KLRC4	1	0.993937	-0.16108	1.343662
GMFB	1	0.994376	-0.00655	5.975214
PPP1R3E	1	0.994477	-0.02458	4.246666
IGF2R	1	0.994623	-0.00507	9.083842
KCTD19	1	0.994821	-0.14684	1.312336
TCOF1	1	0.994923	-0.09117	1.80455
KLHL9	1	0.995197	-0.01666	5.83842
HSPB1	1	0.995275	-0.01434	7.632989
SNORA50C	1	0.9954	-0.01296	7.025625
ZDHHC5	1	0.995636	-0.00678	7.474721
MIR6504	1	0.995745	-0.21511	1.041121
SNAP29	1	0.995853	-0.00719	5.861249
SNRPG	1	0.996037	-0.11682	1.627999
LINC01560	1	0.996282	-0.05807	2.443278
KRT16	1	0.996627	-0.1733	7.187602
NECTIN2	1	0.996635	-0.0138	5.947918
PTPN12	1	0.996811	-0.08139	1.5693
SNRNP35	1	0.996961	-0.09428	2.196576
LIN37	1	0.997115	-0.0317	3.287868
ZNF862	1	0.99737	-0.01752	5.191055
ATP6V1FNI	1	0.997586	-0.04881	3.630534
CEP104	1	0.997686	-0.00622	6.346367

NISCH	1	0.997754	-0.01154	6.232644
UCKL1	1	0.997777	-0.14734	1.505412
PARP4	1	0.997916	-0.00702	8.817061
VAMP3	1	0.997963	-0.01112	6.466067
SNTB2	1	0.998023	-0.01218	6.976814
SLC25A25-/	1	0.99825	-0.0116	5.970344
VDR	1	0.998305	-0.13159	1.683979
NMI	1	0.998325	-0.01435	5.175123
CD5	1	0.998427	-0.02662	4.535323
C14orf119	1	0.998521	-0.04052	4.25071
PID1	1	0.998752	-0.11583	0.992671
ZNF236	1	0.998985	-0.16585	1.686444
ATP6V1E2	1	0.99899	-0.07499	2.558201
LOC28371(	1	0.9991	-0.09786	0.616218
SLC1A1	1	0.99914	-0.02593	6.53339
TRPV2	1	0.999283	-0.02633	3.982198
LINC00663	1	0.999332	-0.12617	2.004929
MSL2	1	0.999383	-0.02142	4.120978
GJB4	1	0.999539	-0.08263	3.101983
ANKRD20A	1	0.999631	-0.07228	2.230229
KRTAP10-7	1	0.99969	-0.28402	1.573657
SH3RF3	1	0.999968	-0.01414	4.981899
PTPRR	1	1	-0.25758	1.223027
OXT	1	1	-0.233	0.89141
XAGE2	1	1	-0.23213	0.965875
PRICKLE2-/	1	1	-0.22841	1.089882
CRIP3	1	1	-0.21759	0.626084
CASC21	1	1	-0.19723	1.027682
LOC90246	1	1	-0.19666	1.407394
CEND1	1	1	-0.19124	1.056602
ABI2	1	1	-0.19077	1.116788
LOC57453ɛ	1	1	-0.19066	1.164267
PGLYRP4	1	1	-0.18496	0.900051
RASSF1-AS	1	1	-0.1829	0.822979
ARL6IP4	1	1	-0.16811	0.711066
MIR5190	1	1	-0.16335	1.036674
SLC7A7	1	1	-0.15973	1.107029
SPOCK2	1	1	-0.15961	0.801987
SEMA5A-A:	1	1	-0.15855	1.626107
CCT2	1	1	-0.15161	1.192923
TMEM79	1	1	-0.14817	1.092911
P4HTM	1	1	-0.14763	0.958805
TMEM145	1	1	-0.14294	0.597576
ASB14	1	1	-0.14167	0.75658
SIGLEC5	1	1	-0.13939	0.929076

ECT2	1	1	-0.13786	1.345009
CREG2	1	1	-0.13226	1.797483
STON1-GTF	1	1	-0.13185	1.018456
GDF6	1	1	-0.13109	1.512894
LOC101928	1	1	-0.12856	2.059693
AKAP17A	1	1	-0.12111	1.599792
LINC02060	1	1	-0.12055	1.165222
LOXHD1	1	1	-0.11866	1.038836
GLIDR	1	1	-0.11665	1.08715
S100G	1	1	-0.11371	1.475974
MIR1304	1	1	-0.10863	1.004958
NAPSA	1	1	-0.1067	1.699039
RHPN1-AS1	1	1	-0.10664	1.117335
GPR55	1	1	-0.10557	1.218311
CSNK1E	1	1	-0.1037	1.706316
SRGAP3-AS	1	1	-0.10354	0.966824
LOC102606	1	1	-0.10061	1.823809
ABHD11-AS	1	1	-0.09904	2.550151
CASC10	1	1	-0.09853	0.754126
FGF9	1	1	-0.09603	1.807609
MIR559	1	1	-0.09514	1.285971
ZBTB46-AS	1	1	-0.09477	1.591562
OXCT2	1	1	-0.09462	1.588033
MIR7161	1	1	-0.09382	1.387116
JMJD1C-AS	1	1	-0.09203	1.822819
LINC01197	1	1	-0.09034	0.871443
LOC101928	1	1	-0.08885	1.771531
CHRNA5	1	1	-0.08765	1.851131
ECHDC1	1	1	-0.08744	0.834573
CDH17	1	1	-0.08497	1.5727
RPL6	1	1	-0.08388	1.43569
DSE	1	1	-0.08309	1.364493
DDX46	1	1	-0.08228	1.765026
RMST	1	1	-0.08059	1.592638
TMEM182	1	1	-0.07828	2.898993
GM2A	1	1	-0.07692	1.968702
ITGA2B	1	1	-0.07646	1.368588
TLL1	1	1	-0.07606	1.738017
AXIN1	1	1	-0.07507	1.148732
OVCH2	1	1	-0.07464	2.561245
IDS	1	1	-0.07447	1.157783
EZH2	1	1	-0.07248	1.593847
MTRNR2L9	1	1	-0.06882	1.398049
CBR3	1	1	-0.06777	2.675904
RBPJ	1	1	-0.0676	1.580013

APOA1-AS	1	1	-0.06682	1.891846
ANXA6	1	1	-0.06581	2.663231
C1QTNF2	1	1	-0.0649	1.563642
S100A13	1	1	-0.06458	2.179979
ADRB1	1	1	-0.06358	1.692735
GCH1	1	1	-0.06309	1.409742
PMS2P5	1	1	-0.06308	2.26762
UPK1B	1	1	-0.06249	2.067113
ADAMTS16	1	1	-0.06225	1.563269
LTBP1	1	1	-0.06194	2.61571
PSMD5	1	1	-0.06064	0.946121
MRPL52	1	1	-0.06011	2.460177
NT5M	1	1	-0.05996	1.74259
TP53I11	1	1	-0.05964	1.733101
CXXC5	1	1	-0.05943	1.980659
CORO1C	1	1	-0.05892	1.604021
CES3	1	1	-0.05855	0.764057
CATSPER2F	1	1	-0.05835	2.215377
LOC102723	1	1	-0.05761	1.8455
ZIK1	1	1	-0.05666	1.78588
FAM162B	1	1	-0.05633	1.857322
DNAJC27-A	1	1	-0.056	1.395287
EXOG	1	1	-0.05526	1.514617
SULT1C3	1	1	-0.05433	4.174009
LINC02538	1	1	-0.05419	1.584806
LINC01521	1	1	-0.05413	2.644208
FAM50B	1	1	-0.05293	2.83437
NR2E3	1	1	-0.05265	1.949252
RCOR3	1	1	-0.05206	1.103996
LINC01730	1	1	-0.05138	2.035287
MIRLET7I	1	1	-0.04972	0.655478
TMEM150C	1	1	-0.04897	2.494044
BHLHA15	1	1	-0.04875	1.840147
SUMO4	1	1	-0.0479	2.828339
SP140	1	1	0.047573	1.026025
ZCWPW1	1	1	-0.04658	0.780592
NCAN	1	1	-0.04616	0.943816
ATP11A	1	1	-0.04609	2.164362
FRS2	1	1	0.045572	1.277726
EGOT	1	1	-0.04502	2.396566
CYP4X1	1	1	-0.04436	3.7057
LOC100270	1	1	-0.04358	1.777417
SPTSSB	1	1	-0.04328	3.520671
LINC02544	1	1	-0.04306	2.72577
TNS2	1	1	-0.04285	2.808564

HOXC5	1	1	-0.04251	1.389012
PPT1	1	1	-0.042	2.248278
HASPIN	1	1	-0.04193	3.159642
APOBEC2	1	1	-0.04185	2.548668
C11orf1	1	1	-0.04138	3.352746
LOC101927	1	1	-0.04126	2.38307
RAB17	1	1	-0.04091	1.045123
NAAA	1	1	-0.04067	2.451334
RARRES3	1	1	-0.03916	3.893962
AQP6	1	1	-0.03914	1.057069
IL27RA	1	1	-0.03795	3.364261
RTP4	1	1	-0.03743	2.571454
PQLC2	1	1	-0.03704	0.985038
PPP1R3C	1	1	-0.0367	4.167048
ANKRD24	1	1	-0.03632	3.266188
CXADR	1	1	-0.0362	2.462106
ZNF322	1	1	-0.03579	1.754535
FBXW4P1	1	1	-0.03543	3.661806
ITGA6	1	1	-0.0354	1.645757
SF1	1	1	-0.035	1.911918
EFHC2	1	1	-0.03468	1.924789
ANKRD31	1	1	-0.0344	1.970956
REST	1	1	-0.03392	1.618784
ZNF675	1	1	-0.03391	3.764197
ADAMTS17	1	1	-0.03382	3.72446
SNORD124	1	1	-0.03302	1.090906
MIR22HG	1	1	-0.03277	3.343192
NCBP2	1	1	-0.03231	2.604147
RNVU1-14	1	1	-0.03216	3.478409
GRIK1-AS2	1	1	0.031976	1.110947
AKR1C4	1	1	-0.03191	2.073797
PSORS1C3	1	1	-0.03184	2.317888
MAMDC2-/	1	1	-0.03136	2.747698
VCL	1	1	-0.03132	2.709346
ADAT2	1	1	-0.03131	3.551172
RGS14	1	1	-0.03093	3.672999
MEG8	1	1	-0.03089	2.109504
PLAGL1	1	1	-0.0307	2.936606
CCM2L	1	1	-0.03053	1.809143
FBXL15	1	1	-0.03006	3.658173
NDST3	1	1	-0.02982	1.212611
USP3	1	1	0.02981	1.343566
LOC101928	1	1	-0.02979	3.397721
HLTF	1	1	-0.02944	2.441438
ACSM3	1	1	-0.02904	2.217154

EFCAB5	1	1	-0.02887	0.930353
GPR4	1	1	-0.02715	3.079196
B3GALT6	1	1	-0.02697	4.133067
ZNHIT2	1	1	-0.02673	2.800046
TCP1	1	1	0.026684	1.550224
TIFA	1	1	-0.02662	3.883329
CCM2	1	1	-0.02641	0.831281
GINS4	1	1	-0.02598	3.640092
TUBB1	1	1	-0.02541	2.756562
ANAPC7	1	1	-0.02526	3.682594
ARHGAP39	1	1	0.025073	1.558325
PLCXD2-AS	1	1	-0.02423	0.745658
CAST	1	1	-0.02375	3.359768
FUNDC1	1	1	-0.02318	3.764079
LMNA	1	1	-0.02315	2.928689
UBALD1	1	1	-0.02248	4.32907
EIF4G3	1	1	-0.02219	3.429456
OR10A5	1	1	0.022114	0.776163
B9D2	1	1	0.022113	1.537246
FGFBP3	1	1	-0.02197	2.93094
LCLAT1	1	1	0.021864	1.7968
CASC9	1	1	-0.02178	0.891772
EWSR1	1	1	-0.02133	4.131278
PNISR	1	1	0.021305	1.429072
MAP4	1	1	-0.02128	4.32856
PLEKHS1	1	1	-0.02126	2.900688
NTN3	1	1	0.021235	1.878002
RPRD1A	1	1	-0.02097	2.89688
LINC01913	1	1	-0.02059	1.97035
ANGPTL7	1	1	-0.02058	3.347267
SMCR5	1	1	-0.02057	3.411324
SERPINB1	1	1	-0.02031	2.802209
LOC10192[illegible]	1	1	-0.02024	4.53927
ANP32A-IT	1	1	-0.01962	4.329464
ORMDL2	1	1	-0.01924	3.831091
ZSCAN2	1	1	-0.01889	3.830776
KCNH8	1	1	-0.01874	4.932753
XRN2	1	1	-0.01862	3.257543
SLC24A3	1	1	-0.01834	5.350022
SVIP	1	1	-0.01832	5.250858
PRSS48	1	1	-0.01828	4.116956
NLGN1	1	1	-0.01738	3.082403
FZD10-DT	1	1	-0.0172	1.989027
BLMH	1	1	-0.01708	5.003541
BCL10	1	1	-0.01637	5.257606

WDFY2	1	1	-0.01635	5.207091
ZSCAN9	1	1	-0.01595	1.583535
ZNF230	1	1	-0.01593	3.620425
KIFC2	1	1	-0.01575	5.747727
RPS26P11	1	1	-0.0154	3.430506
WFDC3	1	1	-0.01527	0.795198
TSHZ3	1	1	-0.01519	4.721055
TDRD9	1	1	-0.01476	2.04306
INE1	1	1	-0.0147	4.609402
WNT5A	1	1	-0.01449	2.796998
PIRT	1	1	0.01417	0.676487
CD2	1	1	-0.01416	4.128071
RPS24	1	1	-0.01408	1.074019
ITK	1	1	-0.01376	4.69447
HNRNPAB	1	1	-0.01282	1.470935
SNORA27	1	1	-0.01275	6.393182
PISD	1	1	-0.01256	5.693412
MTG1	1	1	-0.01248	5.709508
LINC02384	1	1	0.011633	1.500764
PIAS2	1	1	-0.01146	5.228154
WNT7B	1	1	-0.01144	2.720874
LOC93429	1	1	-0.01108	0.604824
KALRN	1	1	-0.01098	3.210814
ZBTB20	1	1	-0.01027	4.419586
P3H1	1	1	-0.00978	0.961037
ACADL	1	1	-0.00977	1.862043
DEPTOR	1	1	-0.00958	2.506228
HCLS1	1	1	-0.00922	2.831034
PLXND1	1	1	-0.00921	7.185438
POFUT1	1	1	-0.00908	6.701259
PRKG1	1	1	-0.00889	4.125303
C6orf106	1	1	-0.00885	3.539521
HEXA-AS1	1	1	-0.00883	1.931541
ALKBH5	1	1	-0.00864	6.848754
TIMM8A	1	1	0.00862	2.920665
ZNF581	1	1	-0.00855	2.73489
KLB	1	1	-0.00835	2.086165
FAM120A	1	1	-0.0079	3.819955
KLHL18	1	1	-0.00786	5.052297
FLAD1	1	1	-0.00733	2.877988
KIAA1656	1	1	-0.00691	2.78819
PLEKHD1	1	1	-0.00679	0.754563
OIP5-AS1	1	1	-0.00621	5.764732
TSNAX	1	1	-0.00576	5.382997
SNORD113	1	1	-0.00558	2.405868

ABHD18	1	1	-0.00539	5.206595
TP73-AS1	1	1	-0.00477	3.142464
DBF4B	1	1	-0.00442	2.315797
KIF2A	1	1	-0.00404	1.358375
B9D1	1	1	0.004025	1.09945
LINC01909	1	1	0.003999	1.572782
DHX8	1	1	-0.00399	3.412563
SLC25A42	1	1	-0.00394	3.647787
SOAT2	1	1	0.003688	0.979564
UBE2S	1	1	-0.00332	4.388219
OGFRL1	1	1	0.003151	5.492922
MIR378J	1	1	0.002544	1.64209
RUVBL1-AS	1	1	-0.00247	0.651676
LSM6	1	1	-0.00243	4.222049
SNORA37	1	1	-0.00225	4.844467
NPTN-IT1	1	1	0.002142	4.846662
ERCC2	1	1	0.002109	3.650923
RNF2	1	1	-0.00161	5.096933
PTK2	1	1	0.001218	1.432972
TARSL2	1	1	-0.00119	5.719284
LOC10272[illegible]	1	1	-0.0011	4.02679
SSPO	1	1	-0.00109	2.586666
HIST1H2AG	1	1	-0.00097	4.81355
HOMEZ	1	1	0.000899	4.888693
HRASLS2	1	1	0.000781	2.386352
CA3	1	1	-0.00053	2.204695
